# The Shared and Specific Genes and a Comparative Genomics Analysis within Three *Hanseniaspora* Strains

**DOI:** 10.1155/2019/7910865

**Published:** 2019-06-02

**Authors:** Kai Chen, Zhonghuan Tian, Fatang Jiang, Yunjiang Cheng, Chao-an Long

**Affiliations:** ^1^School of Bioengineering and Food, Hubei University of Technology, Wuhan 430068, China; ^2^Key Laboratory of Horticultural Plant Biology of the Ministry of Education, National Centre of Citrus Breeding, Huazhong Agricultural University, Wuhan 430070, China

## Abstract

*Kloeckera apiculata* plays an important role in the inhibition of citrus postharvest blue and green mould diseases. This study was based on the previous genome sequencing of *K. apiculata* strain 34-9. After homologous comparison, scaffold 27 was defined as the mitochondrial (mt) sequence of *K. apiculata* 34-9. The comparison showed a high level of sequence identity between scaffold 27 and the known mtDNA of *Hanseniaspora uvarum*. The genome sequence of *H. vineae* T02/19AF showed several short and discontinuous fragments homologous to the mtDNA of *H. uvarum*. The shared and specific genes of *K. apiculata*, *H. uvarum*, and *H. vineae* were analysed by family using the TreeFam methodology. GO analysis was used to classify the shared and specific genes. Most of the gene families were classified into the functional categories of cellular component and metabolic processes. The whole-genome phylogram and genome synteny analysis showed that *K. apiculata* was more closely related to *H. uvarum* than to *H. vineae*. The genomic comparisons clearly displayed the locations of the homologous regions in each genome. This analysis could contribute to discovering the genomic similarities and differences within the genus *Hanseniaspora*. In addition, some regions were not collinearity-matched in the genome of *K. apiculata* compared with that of *H. uvarum* or *H. vineae*, and these sequences might have resulted from evolutionary variations.

## 1. Introduction

*K. apiculata* is the anamorphic state of *H. uvarum*. It usually corresponds to *H. uvarum* in the related field [1]. *K. apiculata* was reported as having a role in spontaneous wine fermentation [2–5]. Strain 34-9 was identified as *K. apiculata* by morphology, physiology, biochemistry, and molecular biology. It is used as a biocontrol agent for citrus fungal diseases such as *Penicillium italicum* and for strawberries during the pre- and postharvest periods to control *Botrytis cinerea* [6]. *K. apiculata* also produces 2-phenylethanol, which inhibits *Penicillium* mould on citrus fruits [7]. The antagonist *H. uvarum* P-2 was capable of inhibiting the grey mould decay of grape berries with no obvious effect on the postharvest quality of the grapes [8]. In the *Kloeckera* genus, *Kloeckera apis* could significantly reduce the conidial germination and mycelial growth of the causal agent of pineapple fusariosis, *Fusarium guttiforme* [9]. Thus, research on *K. apiculata* is very important to the horticultural industry.

Comparative genomics analysis is very helpful for studying evolution and variation. Analysis of shared and specific genes in different species could contribute to understanding their related features across many species. Shared single-copy genes are known to be valuable phylogenetic markers for plant families. A set of 959 single-copy genes was identified as shared among the genomes of *Arabidopsis thaliana*, *Populus trichocarpa*, *Vitis vinifera*, and *Oryza sativa* [10]. In fungi, a cross-species comparison of glomerular transcriptional networks was performed to define molecular similarities and differences. Shared and specific-species transcriptional networks were found [11]. Genetic and genomic data are needed for such studies. Accordingly, *K. apiculata* strain 34-9 was isolated from citrus roots in China and sequenced, yielding a genome approximately 8.1 Mb in size that was ultimately assembled into 41 scaffolds [12]. In a previous study, the genome of *H. uvarum* DSM 2768 (a winemaking yeast, Osnabrück) was 9.5 Mb in size with 335 scaffolds; its mtDNA sequence was also reported [13]. *H. vineae* T02/19AF, a winemaking yeast strain native to Uruguay, was found to have a genome 11.34 Mb in size with 87 scaffolds [14].

This work is based on our previous study, in which we performed the genome sequencing of *K. apiculata* 34-9 [12], and is aimed at analysing the shared and specific genes of *K. apiculata*, *H. uvarum*, and *H. vineae* via performing comparative genomics analysis. The evolutionary relationships and characteristics of the shared and specific genes were examined.

## 2. Materials and Methods

### 2.1. Mitochondrial Sequence

To identify mitochondrial sequences, the known and annotated mitochondrial sequence (GenBank accession no. DQ058142) of *H. uvarum* MUCL 31704 [13] was used as a BLAST query to determine the mitochondrial sequence of *K. apiculata* strain 34-9 and *H. vineae* T02/19AF (Accession number JFAV03000000) [14]. Scaffold 27 of *K. apiculata* was aligned with the mitochondrial sequence of *H. uvarum* using the software Mauve 2.3.1 by running “Align with progressiveMauve.” Because several short and discontinuous sequences matched between the mtDNA of *H. uvarum* and the genome of *H. vineae*, and these sequences were distributed in different scaffolds, the mtDNA of *H. uvarum* was aligned with the whole genome (87 scaffolds) of *H. vineae* using Mauve 2.3.1.

### 2.2. Analysis of Shared and Specific Genes

Analysing shared and specific genes among species in the same genus could contribute to excavating their evolutionary relationship. Thus, to better understand their genomic characteristics and evolutionary relationships, the shared and specific genes of *K. apiculata* 34-9 (accession number JPPO02000000) (Supplementary file 1), *H. uvarum* DSM 2768 (accession number APLS01000000), and *H. vineae* T02/19AF (accession number JFAV03000000) were analysed using the TreeFam methodology [15, 16]. It is noteworthy that the available data from *H. vineae* T02/19AF included only the assembled genome but no protein sequence. We predicted 4746 proteins in *H. vineae* T02/19AF (Supplementary file 2) by applying Augustus, which was used in its genome publications [14, 17]. Based on the TreeFam methodology, all the protein sequences of *K. apiculata* (3786), *H. uvarum* (3043), and *H. vineae* (4746) were used in an all-against-all BLASTP analysis with an *e*-value of 1*e*-7. The identities of the proteins in the three yeasts were calculated. Then, the tool Solar was used to conjoin the fragmental alignment for each gene pair. Finally, we used the Hierarchical Clustering Algorithms of Hcluster-sg to calculate the mean distances so that the gene family clusters could be extracted [16]. The distribution of the shared and specific genes in the three *Hanseniaspora* strains is indicated in a Venn diagram. All shared and specific gene families were classified by GO analysis using InterProScan (https://www.ebi.ac.uk/interpro/interproscan.html) according to the biological process [18].

### 2.3. Phylogenetic Analysis

To learn species relationship intuitively and directly, phylogenetic relationships were reconstructed based on the whole genomes using CVTree with the maximum likelihood approach to calculate branch lengths (*k* = 3) [19, 20]. In addition, collinearity maps were generated using SyMAP [21, 22]. The three whole genomes (*K. apiculata*, GenBank accession no. JPPO02000000, 41 scaffolds; *H. uvarum*, GenBank accession no. APLS00000000, 335 scaffolds; *H. vineae*, GenBank accession no. JFAV03000000, 87 scaffolds) were aligned with each other to construct collinearity relationships.

## 3. Results and Discussion

**Figure 1:**
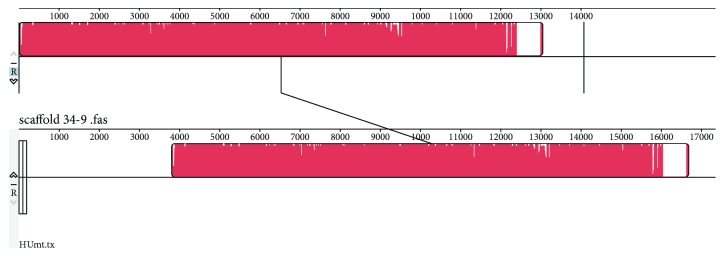
Sequence alignment between scaffold 27 and the mitochondrial sequence of *H. uvarum*.

The mitochondrial sequence of *H. uvarum* was used as a BLAST query against the genome sequence (41 scaffolds) of *K. apiculata* 34-9. The results showed that it strongly matched scaffold 27; thus, scaffold 27 represents the mitochondrial sequence of *K. apiculata* 34-9. Additionally, scaffold 27 (accession no. JPPO02000001) was uploaded to GenBank as the mitochondrial sequence. The alignment of scaffold 27 with the known mitochondrial sequence by the software Mauve 2.3.1 also showed high identity. The longest continuous fragment matched between the two sequences was 12,774 bp with 99% identity, and the remaining short fragments in scaffold 27 also had high identities, above 97%, with the known mitochondrial sequence (Figure 1). This comparative analysis could help to annotate the mitochondrial DNA of *K. apiculata* strain 34-9 [23]. According to BLAST, several short and discontinuous sequences were matched between the mtDNA of *H. uvarum* and the genome of *H. vineae*. No integrated mitochondrial sequence was found in the genome sequence (JFAV03000000, 87 scaffolds) of *H. vineae*. Scaffolds 79, 73, and 68 had some obvious matching with the mtDNA of *H. uvarum* with relatively high alignment scores (Figure S1A). Mauve alignment indicated that there were 3 locally collinear blocks (LCBs) with a minimum weight of 728 between the mitochondrial sequence of *H. uvarum* and the genome sequence of *H. vineae* (Figure S1B). The locations of these 3 LCBs are shown in Figure S1A. This finding suggested that the mitochondrial sequence in *H. vineae* T02/19AF (accession number JFAV03000000) could be reassembled based on these fragments following the known mitochondrial sequence of *H. uvarum*. However, there was a definite difference between these two yeasts. To some extent, the distance of the relationship among yeasts could be revealed by comparing their mitochondrial sequences.

**Figure 2:**
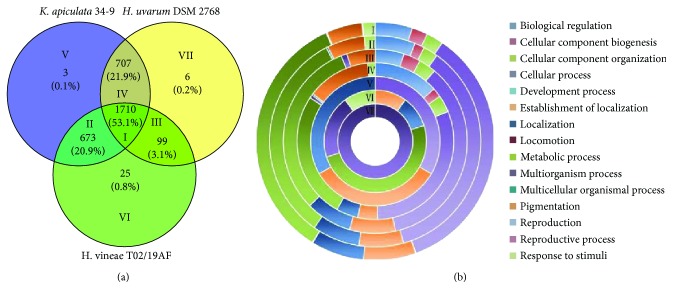
Profiles of the shared and specific gene families of *K. apiculata*, *H. uvarum*, and *H. vineae*. (a) Venn diagram for the shared and specific gene families. (b) GO categories of all gene families according to the biological process. All proteins of *K. apiculata*, *H. uvarum*, and *H. vineae* were annotated according to GO categories using InterProScan (https://www.ebi.ac.uk/interpro/interproscan.html) under default parameters. These GO IDs were assigned to the genes in the seven areas in (a). Then, the gene family files with GO IDs were submitted to the WEGO website (http://wego.genomics.org.cn/cgi-bin/wego/index.pl) for further GO categorization. The circles I to VII correspond to the areas in (a).

Among the analyses of shared and specific genes, a total of 3223 (Supplementary file 3) gene families were obtained. They were divided into single-copy orthologue families, multiple-copy orthologue families, unique families, and other orthologue families (Table 1) [24]. The numbers of shared genes (single-copy and multiple-copy genes) in *K. apiculata* 34-9, *H. uvarum* DSM 2768, and *H. vineae* T02/19AF were calculated to be 2108, 2056, and 2242, respectively (Supplementary file 4). Correspondingly, their specific genes (unique families plus genes not distributed in any families) were 275, 129, and 1695 (Supplementary file 5). Based on the analysis of shared and specific genes, the three *Hanseniaspora* strains shared 1710 gene families (Figure 2(a)). The numbers of unique gene families for the three yeasts were 3, 6, and 25, respectively. *K. apiculata* shared more gene families (707) with *H. uvarum* than *H. vineae* (673). Notably, *H. uvarum* shared only 99 gene families with *H. vineae*. Most of the gene families (circle I) were classified into the functional categories of the cellular component (756 families) and metabolic process (744 families). In addition, most of the specific gene families (circles V to VII) of the three *Hanseniaspora* strains could not be annotated with GO categories (Figure 2(b)) [25].

**Figure 3:**
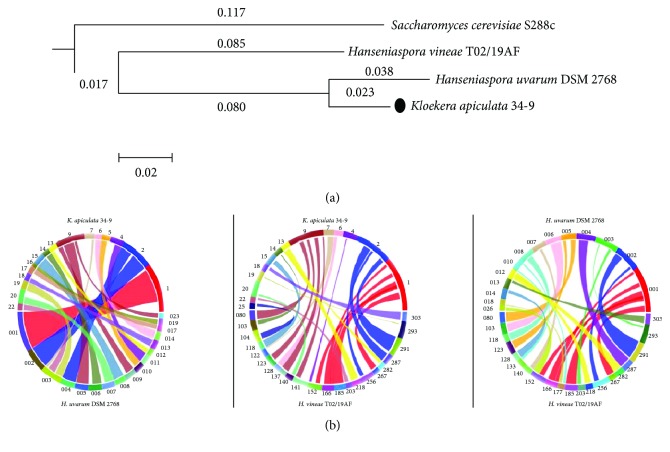
Genome comparisons between *K. apiculata*, *H. uvarum*, and *H. vineae*: (a) phylogenetic relationship; (b) genome synteny maps.

Whole-genome phylogram analysis was used to display the phylogenetic relationships among *K. apiculata*, *H. uvarum*, and *H. vineae* with *Saccharomyces cerevisiae* as an outgroup (Figure 3(a)). The results showed that *K. apiculata* was more closely related to *H. uvarum* than to *H. vineae*, which was as expected, as *K. apiculata* was the anamorphic state of *H. uvarum*. The collinearity degree between *K. apiculata* and *H. uvarum* was higher than that between *K. apiculata* and *H. vineae* or that between *H. uvarum* and *H. vineae* in the genome synteny analysis (Figure 3(b); Supplementary file 6). However, some regions of the genome of *K. apiculata* could not be matched in the other two genome datasets, which could reflect a degree of species specificity. Because the genome data were sequenced and assembled, there were gaps and misreads among the scaffold sequences. Although *K. apiculata* 34-9 is the anamorphic state of *H. uvarum*, they are the same species with genome differences. *H. vineae* is another species, and the three strains come from different locations, where different environments have affected the strains for long periods, which may have led to genetic variations during evolution; thus, differences are frequent throughout their whole genomes.

The complete genome data of *K. apiculata* 34-9 and its mitochondrial sequence will facilitate the further study of its biocontrol effects, flocculating ability [26], and wine fermentation, among other properties. We clarified the genomic features of *K. apiculata* 34-9 and the evolutionary situation of *Hanseniaspora* spp. through analysis of shared and specific family genes and comparative genomic analysis in this study.

## 4. Conclusion

In this work, scaffold 27 of *K. apiculata* 34-9 was found to represent its mitochondrial sequence, with high identity to the mitochondrial sequence of *H. uvarum* MUCL 31704. There were 3 LCBs with a minimum weight of 728 in the alignment between the mitochondrial sequence of *H. uvarum* and the genome sequence of *H. vineae*. No integrated mitochondrial sequence was found in the genome sequence (JFAV03000000, 87 scaffolds) of *H. vineae*. The shared and specific genes of *K. apiculata* 34-9, *H. uvarum* DSM 2768, and *H. vineae* T02/19AF were identified. In the GO analysis, most of the gene families were classified into the functional categories of the cellular component and metabolic process, although most of the specific gene families in the three *Hanseniaspora* strains could not be annotated with GO categories. Genome synteny maps revealed that some genetic variations may have arisen during the evolution of *K. apiculata*.

## Figures and Tables

**Table 1:** Statistics of orthologue families between *K. apiculata*, *H. uvarum*, and *H. vineae*.

Species	Single-copy orthologue family (gene)	Multiple-copy orthologue family (gene)	Unique family (gene)	Other orthologue family (gene)
*K. apiculata* 34-9	1400 (1400)	310 (708)	3 (8)	1510 (1403)
*H. uvarum* DSM 2768	1400 (1400)	310 (656)	6 (13)	1507 (858)
*H. vineae* T02/19AF	1400 (1400)	310 (842)	25 (64)	1488 (809)

## Data Availability

The genome sequences of *Kloeckera apiculata* 34-9 have been deposited into NCBI under accession number JPPO02000000. Genome sequences of *Hanseniaspora uvarum* DSM 2768 and *Hanseniaspora vineae* T02/19AF are under accession numbers APLS01000000 and JFAV00000000, respectively. Other relevant data supporting the findings are available in the paper and the supplementary information files.

## Additional Points

*Nucleotide Sequence Accession Number*. This whole genome sequencing project has been deposited at DDBJ/EMBL/GenBank under the accession number JPPO00000000. The version described in this paper is version JPPO02000000. This yeast is available from the China Center for Type Culture Collection with the accession number CCTCC No. AB 2015432 and is also available from the host institution.

## Supplementary Materials

Supplementary 1 Figure S1: BLAST and Mauve between the mitochondrial sequence of *H. uvarum* and the genome sequence of *H. vineae*.

Supplementary 2 File 1: All protein sequences of *K. apiculata* 34-9.

Supplementary 3 File 2: All protein sequences of *H. vineae*.

Supplementary 4 File 3: The result of all gene family analyses of *K. apiculata* 34-9, *H. uvarum* DSM 2768, and *H. vineae* T02/19AF.

Supplementary 5 File 4: Results of the shared gene families.

Supplementary 6 File 5: Lists of the specific genes of *K. apiculata* 34-9, *H. uvarum* DSM 2768, and *H. vineae* T02/19AF.

Supplementary 7 File 6: Blocks of the genome synteny analysis between *K. apiculata*, *H. uvarum*, and *H. vineae*.
