# Supplementary figures and images for: The Shared and Specific Genes and a Comparative Genomics Analysis within Three Hanseniaspora Strains
Source: Int J Genomics. 2019 Jun 2;2019:7910865. doi: 10.1155/2019/7910865 (PMC6589277; doi:10.1155/2019/7910865)

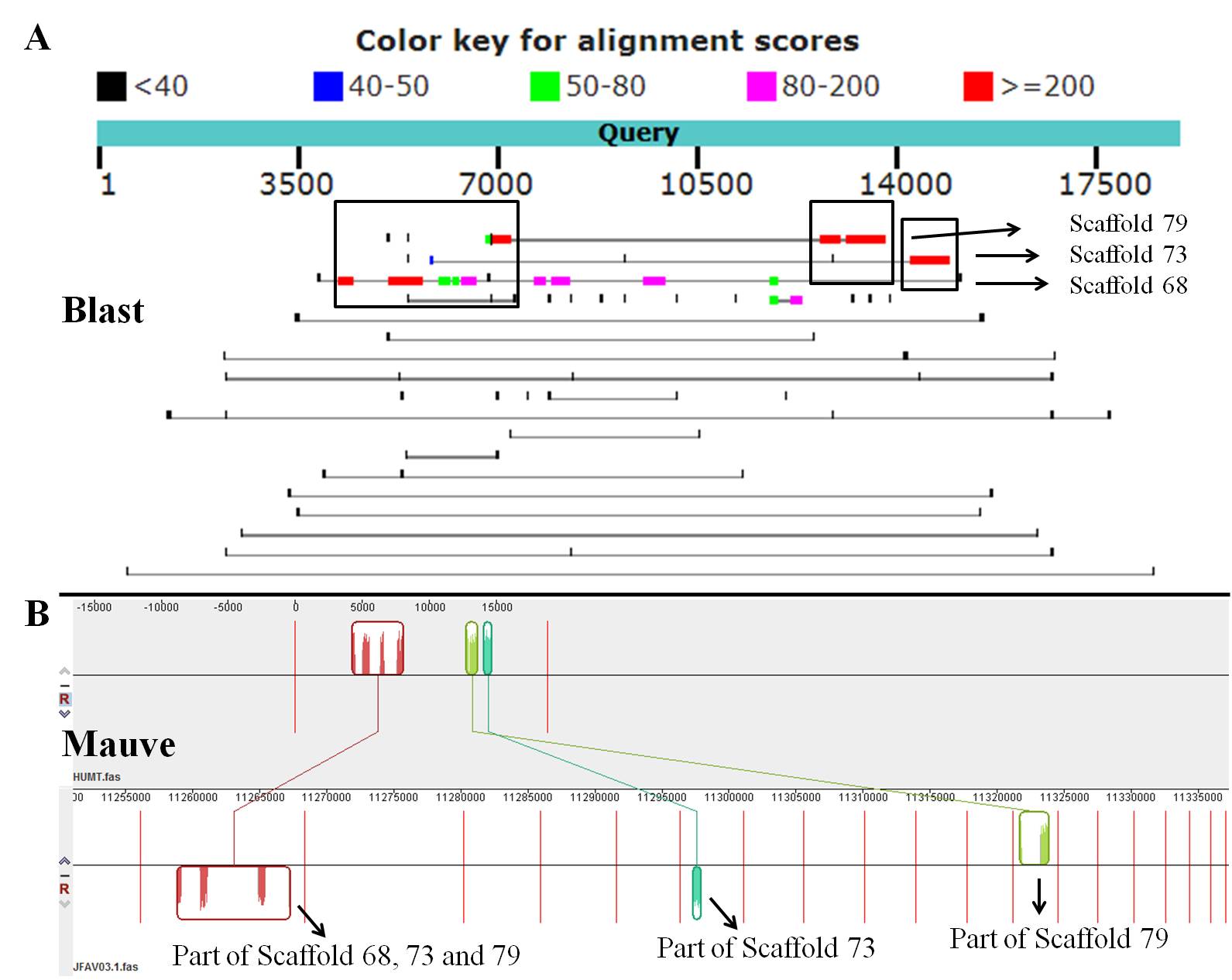


Fig. S1

Supplement: Supplementary 1 — Figure S1: BLAST and Mauve between the mitochondrial sequence of H. uvarum and the genome sequence of H. vineae. [file 7910865.f1.docx]
