# Supplementary material for: The Shared and Specific Genes and a Comparative Genomics Analysis within Three Hanseniaspora Strains
Source: Int J Genomics. 2019 Jun 2;2019:7910865. doi: 10.1155/2019/7910865 (PMC6589277; doi:10.1155/2019/7910865)
Supplement: Supplementary 2 — File 1: All protein sequences of K. apiculata 34-9. [file 7910865.f2.doc]

**Supplementary Table 1.** All protein sequences of *K. apiculata* 34-9.

>34-9_0001

MGRDYVFKGEKEDPLENYVEFKAKFKFAMRSLSDLTPEKQEETVKESLPQILRSRALKQY

QFWADRSWEMKEIWTSLDAFFGYTDDTGTAMNYALSTAICNDKWTDSTNVSIAYENWIKI

EDRIKGWSAEKLVFEVMKGQVIGRESRKFVSEMEECTISEFFAQIYKFMRMRNPHKPVNT

TRHLEESRQEVYTVTVSDKRERNRDHNWKSKRRISVRGYEELWEALCKKRKIPNSEANKM

LAKSRFDAKTCLYCGNGTHSGFTCRVPKKASGAITPVYVIDWSGKNWVKALRVSNEDENV

NIDTLLDTGAAISLIHPTLVDRLQLTVRKTSPGISISGIEKGSISLRDIVTFTVLNNKVE

YDVTAYVFDKTPQPLLLGFSDLVRLKFVAEPAKHQVLFNQELVQIMNNAVVCNDEEIISN

QSESSVKVSSTPEIYNPRPIGENRNITTSWPMFVGVTTMEKEAFLKALGIPEGIKINDPF

FLLDEIDDGLTTMNHGNGSKKFQAKEKQLLEEFESIFQSTFKDTNAKAEGSNSMKPKMRI

KLKEEFKDWSKSITRFEKDPIRIEIIEKTVKELLEADIIERSVSNHSYPAFLVPKGKDKY

RMVTDYRLLNDHTVSEETDLPTVQEMIQQLGKKKFFSSMDLLNGYFQMEVEESDRHLTAF

TVRSGHYQYKRVPFGLKNAPFEFTKFMRYVLHGIEDVLFYMDDVLITSDSLEAHEAKLRE

VFTRLREQGVTLKKEKCHLFRTGVKFLGYMVTRKGVQMTSERTEAITNMVCPKNVSEARS

FVGMANYFRRFIQHFATIVNPIHVYINEKVEWGKDQQTAFELLKEHLTSDPVVAPYVPGA

EMRITCDASKVAIGSVLEIAELVQEKKVWHVVEYFSKSLKKAEKNYYVGDLEFLAIVAAL

KKFRYYLIGKKFTIRTDHAPLSAYNHFTDLSSRLQRQLDVTLEFDFRIEILKGKDNTVAD

ALSRMPTQSRDGAEELSKTPVLAFTASADFTNYLTTEINVQEFRKYYAEDEFCSAMILQL

RNAENAVNSNFEFDEPDVEDQRLFEKYTKEYAGSSILRADVQVSNKILYYRGRAVVPKAM

FIRVMNVFHDNPFYGGHSGASTVMLKISDIFYWKKMNTQIHAYVKSCLVCQTTMRLNHSK

GRLMPLEVPAGRFYELSMDFLSGIHKADEEGHDRILVIIDRFSKYVILIPTKKSLTAEEL

VYVLFREVFSKFGVPQSIVSDNDVLFDSKIYTEAAKRFGITLKKTTPYHPQADGQAERAI

QLLSPFKIAFGYNPRRVTPNTLVKDNDVMWDIAVEAKFIENMVKENLEDARIRMQMNDNN

VDLILKKGDFVLVNRAVIYAKGPYMKLMPLYLGPFKVEGVVNKNAYYIQFPGTRRRSNQV

NVKDLKLYNRRNVYYARPPVTPLEKALRIGQISQIVGWDDKSFYVKMDTVDPEITVEYPT

NNFIVHGRCGVPRVVSDKLLSLDNEDEGDRY

>34-9_0002

MDEIESHSANPSQEIKNDRGRKKDLRNAFFDIVN

>34-9_0004

VLSSTSSTLSISSAYYSNSSVVISSTVYTGTSTSSISTSIASITTPLTTSTSNTIVSSTS

GGSSVVVSFSSSAVTTTSSLSSTVISISSTQSISSITSSSVPSDVSTSASFFSTISTTNS

LSSSKTSEISTVISSTALESTTASTTASNSLVSSKTDGSTASVSIETATETRNTKTSNTE

VTSANSASTSKSVHQIYTTKVPATTTRKSTNSLGSVVTATVLTTSLQTVTCTETKCIKSS

LPVTTGPVPTSTITSSNAQNAASSQETGKSNASLSNVSNETPTTASEGNNNTNAKTTRIS

SHSATILSPSSAISTTAQLTVPTSSEFIGAASVNAKGSIFSVILGLVMVLVL

>34-9_0005

MNTIINVSNIFFKNKSGTTTEKTTHEVRNTDQEDIVNEKIHDGYAFNGTGQGKNELKKEE

VNFQTLRNPPASKSQIHLVKIFYTIVYEVLIMFFYIILSPFYFFDTYTTKINSSVVANGD

LNNTGTSFGLIDKDLGTEKQERLFFPKKLIPQSVLTKRKVLILDLDETLIHSTNVSEELS

NKFEKLNAEDLRVIDNNVATVNVETSDTKRLRNTNKVDSFQIEVSFRLPKNTTIPSIFAN

AHNSKQTNVTSIYKVFQRPYLIQFLKTCNIWYDIIIYTASLREYSEPIINQLEQLSGVQF

INKLYRKDCKVSPSGYIKPLNLITDMYAAHDILSTHASSSDLSSARTAGLTQRKMKNNKY

ISKESMIIIDNMPVSFREDMDNGIQIKSWYGDPKDIELLKLLPLLEGLKNVADVRLVIGL

RTLNPELLN

>34-9_0006

MSDDEALKTKLIFEACKRLFLSNSFDEFDEYISKVKNLFDIPDILGRHRDEIIKCLEYYN

RFLLKNRSKIENFPKKYYESCKYIIKLDFIKGYYNLLVITKCNLDSKNLNSNIKYIEKFY

YKFTYFLDNSSYDNSKSNLNRIVAIKNSYSNFKKENTKIPTTMHPIKRYRDLAQEDMTEG

EYRLKEDMKKYKSMISDPKKQIPMFLYHFPDDVNMKILSSLTSKDFLELRCANSLVNKFI

CGMYLHRMDDFYKNIMIDNDDSFKKFVRMIGYFKVRASPIEKLTFDFDSLSNDKALTIEP

KAIQKIINLIKVKELCIINHGWTILNILPFNIVSKHGYKLEKLHVKIGKIINDNYRFLEN

SLVLFTGAFLQKLAVEYINMVYIKEVPSIGIKSTTNAIQSYDGSILRRQEISALESLEII

SFLDTAMFPLLNKYTKNKIFMELFIKFDEVFNNLKEITLRNFSIFNMNSLFTYITDNPLR

ITKLELLRIPALPRIPKLFLLLKDLNNLKTFSLHSSSNLRYTFTSINNFILIKNIYDTNE

ISDEFLERFVIIPTWPKLEELTLIETSINKPILKSLLNENIRFLCLNDNLNLKYMPAERV

SELNFNIDIPKYFITDFGEFYEVVPKLEVFSLNNLDFDMHSTFLTHNTNIFYKSLFKMNL

KTLDVSMNQSFCLRLFDFLRFVGLQAKIDDLKVRNLVYDDLFNQDQKLKEKFEQKIKILN

LVTGIITNKIRQFKNDNDYVCWYRSCVLNKYQGI

>34-9_0007

MQNNSAEYLNGGYAANNNPITHGHSVYTEPGYVAKDFVATDISFNNIAFCASQPDNAS

>34-9_0008

MINRDIRGEILGFMKDEQLRKHLPRMFSLIKNESWGLEDDQIPS

>34-9_0009

MNRFKKSVIKIVKKYIIISIMCQQLIDDNIARLSDFT

>34-9_0010

MNDPDLAEDLTPLLKHVFTAKNLATLLKIVKRDYAN

>34-9_0011

MSQQTHSMENIGEIKERFDIIYAFYLRIRTTPFFLSKMYKNLNDDERIFFTRMHQCFNKI

YYDKNGALNLIKKNISRILLDEEFNTGIYFNDSISYEIVQSSYTNAIFSKRYDSQKNH

>34-9_0012

MSTVPADSSECISDAFDQQKIGATFSNDKGSNDFNLYDEDKINIYSVLPVYDKPWYRIRH

FQKLLFIILIVALTASNTGFESSLLNSLYTEKDFNNAIGNVSGSVLGALTSGYSMGCFIS

FFFSAKVNDKFGRKKCLLFFNVVIIIGVAVQSIAGAWKTNGFPKNYKKKDVLGTLIAGRM

IIGIGSGVIQSSAPALIAEIAYPEPTARTIQINYYNSNWYLGAVLAAWLAFGVKSVAHHW

AWRIPTICQCGLPILQLILIPLFVPESPRWYVAQGRLTEAREILNDLHAGNMENGQELVD

YEMTEIQLAIEQENIAAQTSFQQKQNLHTMTDQNQTPIELKNTNLFKPLKLSDKITLNHR

AILAPLTRLRADANHVLRNETDYASSEEWKEFIAKPYNKTGDKVRSLVEEYYYQRSQRPG

TLIISEGTFPYAAAGGESFAPGIYTNEQIESLKKVIDAVHANGSYYFVQFWNLGRTADPE

VLKNEGHKFVAPSENYISKDEGSDIHAAGGYLLNQFFDKKINQRTDKYGNQSFENRARLY

FEILDDVIDKFGADRVGTKLSAYTDVNGLSQFANLEDTTDFYKYIAVELEKRRLNGKGPV

YLTLQEPRIDESFVDVLDTDIGKVSNKFFVETFKGVIIKSGNFSNSFQSAQEAVNETDKI

LLAYGRHFIANPDLIDRLENGWELNNYDRNTFYSGGVNGYIDYPYYETN

>34-9_0013

MDSFDNLYFSLALFKLKHDTKMLKNVTIIGFEFKEKRFRDLHWKYVGAKNKLTDCTLEFN

SNIPIHHDLEKHDVYIQSVNDSERLYGYEKFVNDPFAVRTKLFEKKKLRNFKALSAKDSD

YGKYLIDIDPMLSDQDILIEIEQSELYV

>34-9_0014

MTVKIAIVIYSMYGHIATLAETEKKGILSSVPDSEVTIFQVPETLPQEVLTKMGAPPKKD

YPIITTEILAQHDAFLFGFNTRFGNVSAQMKTFLDRTGGLWASGGLAGKLVGFFESSGTG

TNAVTIINNLSFFVHHGCIFVPLGYAAAFAELTDLSVAKGGTAWGAGTIAGPNGELQPNE

V

>34-9_0015

MNIVLGAKYIGAGIAAVGLIGAGIGIAIVFAALINAVSRNPSMTKLLFPYAILGFSLSES

TDIAVLYFILALFAAFIGLSMSMFMRLELIHSGSVIINNHHLFNSMVSAHAIMMVFLAIM

PAIIGAFGNYFLPVALGDADMAFPRLNNVRFFDAEGSICYSFKNNIPQLTVAVSNQYSHD

IEYFKLLGGDIYFSKSGHGNFLWSTQSKQDIIDFLNYNKNFSSRTTKFNQLSLVTLYYQL

KDIKAYNPQSDFHKL

>34-9_0016

MFVVGLDTDLRAYFTSATMIIAVPLGRLRFGVPVLFALAFLVLFLAGGVTGVMLSNASVD

LAFHDTYYVVAHFHYVLSLGAVFGIFTVLGLDYNKNLARIHFVLLVIGTNLTFMPMHYLG

INGMPRRISDYPDSYNA

>34-9_0017

MVHFKDIINISYPVYIAIARLYRLRLIISGSIVFRSKDELNDYSGLAINAQLVYTIGLST

GIIIGCTILGIKLHGMNFFTLFLPNGAPTGILIVLFFIELLSYCSRAVSLGLRLGANTLS

GHLLVDIVATLIYAFGTLSMILAVVGTALFGLLVAIQLLEIAIALIQVYVFSILTVNYLK

DVVDLH

>34-9_0018

MLYLNDVPTPYSLYFQDSASPAMEGIIELHDTIIFIVTSMDVIHDFAVPSLGLKIDANPG

RLNMLSTIINRNGVFYGMCSELCGVGSLLGLSLVIQICTGLFLAMHYSSDTSLAFNLVEH

IMRNVNNGGFSVSNALIKRFFAFHFLLPFIVAAVVIMHMMALHLHGSSNPLGTSGNYDRV

GMHGYFIFKDLILIFLFLIVFSYFVFFAPELLNHLDNNIMANPMSLPSSIVPE

>34-9_0019

MDVRIHWSTKFFLYLETLRNDPTKGSIYVVISELIFETTYSKDYLTVSIVPKKLMVPDTV

RKLQKDGYHITGEINTDLMKFDYVNNNVKLITYETPKLPNMQFYSHTPIYSKLFDEKRKT

GINLDGNLKILIDNVKYGSGKQCYEINKVIHSLNISDYHLKLFNSKYNDLISIKPKDSLK

ENFVLKPFFKLLNLYVIPYIFIILQVLFKEILLNILKPFYSLRYYSVLFLQLDLRIKQFT

YFPMQYLSIGERSTLNTFSKNEKLYASYMDYIRYYNTLWLIVNDYSFSLTVSALLRIHKH

SIINFLMTFLNQIFIKKVFYLTNALSQNPFGIKLNDELTKFLRDLFYWIIEFVDNTYFKF

LTNEITLSYIIDALYVLTYCFGVTFFIAFLIDYLTILTLHFKIFYKISYKLYRIQLKLLI

SLVYLFCGKKYNTLRDRIDNESYSLEVLLVGVLIFMILIFLMPTTIAFYLIYTFLQYIAK

SLEIILMTVIMCFNHFPLFIIMLKLKDQKRIPNEIQLNTGYETIIQSPVIKLSSESLKWK

EIFTNFWNIITQFQNEIISVDLITKIILGKDILIDKYALYEPLYSTVPENTITFKDLAHK

LLYDNPRKYL

>34-9_0020

MSSLQKALKPAKEIKNTLPKLEKLRCTIFDKFYNPDNLRVGVEVWEKPLLGPSLRNYYGS

RTNLNFSEFMTSFRKNLEGTDFKLQDQREIDRLQYVEERKRIGKGAPKKKNEKVEKKNKK

KK

>34-9_0021

MSLPATFDLTPEDAQLLLAAKAHLGHPNVQVSYVHSTRPDGVNVIDAGKTWEKIVLAARI

IAAIPNPEDVVAISSKIYGQRAVLKFAAHTGATAIAGRFTPGNFTNYITKSFKEPRLIIV

TDPRSDAQAIKESSYVNIPVIALTDLDSPTEYVDVAIPCNNRGKKSIGLIWYLLAREVLR

LRGALTERTEAWSIMPDLYFYRDPEAENEEVAEEAETEEATEEVAAEADAEAAPEASEWT

ATAEESY

>34-9_0022

MKIPDFNHKLDREISYRIRIDSARYLLCFIFLVIVLYIYIPKLSISYSYHTPCRTNTNTA

QESKRKIISLNNICLICAVLAGVSYTSIVSYIQTLDLVLITKRLGRNCMSLYVPLLFLTM

RPSPLPNVLYLNLIPIHKWLGRLLVLQALIHSILYTCLYYKTEVLWKLAKLANLYGIFSM

FGFILIAGLSISKIRRSDYRIFYISHYLLTWMTVIMLQYHARPRATYSTTANVCILVFQI

LYRVYLTREITFDDIEDVSPTFKRIKIPMHLLAKKPISPGCHIRINNKHENFFKNFFFNF

CVPLQHPYTIESLPTDEHCTLIIKNGRFPIIQGNKYYVTGCFEPRLPFFNNNIFQEKTKK

YPTAFNKLFGNKDKKVIIVTGGSAITFGLPILQTLNNENYKCKMYWITRDLHDLNLIKKN

YQNLEIFVTKTTSEDENIIVDRNSIIRDINLEHGLDELQTTPLIQSTVDPNSPNAPIQAM

LSNPHSISLDEYQETTKASDILKNQADADEALNSSIVYRNYGDNNDEIDFTKASKEDLKK

KKSQMFTGENFRQPSTLVDLHNDRSLFDTYDYGDRTYGSINRVKKHGGYRNSLDKITIPK

GLKINIGRPTLGFKELLWIYNEDKECNGNNLNDSSCLENIYLKTLGVGDGNFQLPKSSSS

EDKDVLVLAAGPTGLVGNTKNWAILHNFQFYEEAFFV

>34-9_0023

MSEEQKDEFFKPVGEIVEETNQTSNNIQQDPENPNVLYYTEPKEDESPVTTIESLCMQCQ

NPHGKTNLLMTTIPHFRKIIIMSFECDKCGFKNNEIQTGNDLQVKGVDFIMDINKLQKDD

SFVKDLADFMNRQIVKSAYGVVSFQFIPLNKDDEIVQFEIPKGKAQLSTTEGLMMDTILD

LNALQHLRKYQNIEAYEKIDELIHCVYAMISKNNNGKLEEIVDGQEQKIHENIDFNQEKV

SKYISSINEIKVNVKDPSGNSFLEYKPFEENSTINYSKKDYFRSDEDNFHLGLITEDQLE

EKKVLNQVVDKYLPTFDDKKNNSAADEQMVDEFVNDIETFVASCPSCLAEDCITNMKPLN

IPHFKQVLIMSTKCDNCGYKSNEVKTGGGISQYGKKTILTITSETIEEDLKRDLLKSETC

KLFIPQIHLDIQEGTLGGRFTTLEGILKQVHDELYERVFQESYDSMEESTKQNWLSFFKN

LTNVMNCKLPEGCDKLEVMLIDPLANSYLQNIYAPDEDPMIEYQSFKRSRRENEDLGILE

LLKQEGLSEGEEDERDIDDE

>34-9_0024

MGLAGERKKQRIGWDPRNLNWLATGSNPTETEKDASQKTFQESSFGLKLLMKHGYDKKTK

SKTSEEIMESIINSKEFGHNIKIKVKDDKFGIGYTIGSSQINKKINPRTGRVEAVEGNDN

FGLDMFQQILFNMNNKESSKDKKKRKKIEAQNAQLDAVRTERIKGKYGMDFVKGEVLKSE

LAAILKKEKEIKEKKEKKEKKEKKDKKEKKSKKRKRDDDSDDIENSKKKSKKDKKDKKSK

KDKKHKVKVIDHDMKIEDVKPVDFKGTRYAARSKFIRSKRSAISDPSALKEIFMIDDIQS

SVNLKKT

>34-9_0025

MYSSKLIFAALFTALFNVLVNASDLPAIEIKGNKFFFSNNGSQFYIRGVAYQADSANATD

DSTITDPLADYETCSRDIPYLQELNTNVVRVYALNATEDHSQCMQALNDAGIYLIADLSI

PSVSINRNSPSWDVTLYDRYKKVVDEFQNYTNVLGFFAGNEVSNEWNNTDASAFVKAAIR

DTKAYIKDKGYRSIPVGYSSNDDSVTRLDMADYFACGDSDERADFYGINMYEWCGKSSFQ

LSGYKQRTEDFKNYTIPLFMSEYGCNEVTPRVFTEIAALFSDEMTDVWSGGIVYMYYQES

NNYGLVSISNGKVSTMADYSYYSSEINAVSPTSTNSASYTPSATSMTCPSVQAKTWLADD

VLPPTPDKALCECAYDSLSCVVSTDVSDDDYADLYEYICSEIDCSGISANGTTGKYGDLS

ACDAEVKLSFLLDIYYNSIGESYACDFSGSAVLKDASTQKGCSAVVSQLGSIGTNVISAT

ATYTGDFSYGTATGTADADSSASSTTVSSKAKSSGTASNTASSSSSSASSSKSKNMAPAA

APSSSKVLISLLATIGVFAGMSFAMI

>34-9_0026

MDNHSKSSHSDSESFFENIRLEYPAWFNDLNKDLDYLSNDDYYYSGSNQNGNSPVSFEDI

YTIFEELQRKFGFQVDNMKNMYIHLLTQLDSRASRMSAKMALVTLHADYIGGDHANYRKW

YFSCQLDLDEEVGFNNLKLHGKSHQRNKSKAKKNKQTLKTYWEALDNQQKARDEITSTEF

KNITFFPRKRSSNPDFSEKDTITLDKSVTDTILQRSSENVKTSIKKSEGNDVKNTESFKK

QEYKRKTFWKKKGKSLKYCSYLWKKKMNSLSFVDMIRQVALYLLCWGEANNVRFTPECLC

FIFKCCWDYDQYTLSMDKLSLNEVEKLLYQDKPEFYYLDSIITPLYTFLQQQVYEKDKNG

NLQRKPKDHKEVIGYDDVNQLFWYPEGIEMIVLENETRLIDIPFNQRFLYLEKCQWKKVF

YKTFYEKRSWFHCLTNFNRFWIVHLTTFWYFTSLNAPTFYTANYHQLLNNPPTAQTTLAV

MSLAGTLACLIQITATIFEFTYVPRKWPGAQYLGKRLIGLIILLLVNFCPSMFVLVKFKF

NQFIESYFFLTLSLKDPLRVLSILNMSRCYGDSMLPKVLVCDLQPKITIVLLLLTDLILF

FLDTYLWYIICNCFFSIALSFSLGTSILTPWKNIYVRLPSRIYSKIIATNQMSEIYKTKW

LVSQVWNALIISFFREHLLTIEHTKKMLYTGKEDCLKAPTFFIAEDDSTFKSTEYFAKDS

EAQRRISFFAQSLSTPIIEPLPVECMQTFTVMIPHYSEKIMMSLPELIKEKDIDTKLTLL

EYLQQLYKEEWKCFVKDTKVLANELGYLNNNISSTLKLENQAKKTDAQFQDSRSSISNNS

EENKNFDLAFETVGFNTSNPIFTTRTRIWASLRTQTLYRTISGFQNYIRALKILYRVENP

LMIEVFGEDKDGLELELESMANKKFKMVVAMQRYSNFTTEELNDTDFLLKAYPHMTISYL

EEDFVENQDTHEIKRYYYSCAVDGFSKVDKKTNKRIPNFKIKLSGNPILGDGKSDNQNHS

IIFYRGEYIQVVDANQDNYIEECLKIRSVLNEFEEVDIDSIHPYKVDNDYMKDESILNSE

NNTPVAIIGAREYIFSENIGILGDIAAGKEQTFGTLFARTLAEIGGKLHYGHPDFLNAIF

MTTRGGISKAQKGLHLNEDIYAGITATCRGGRIKHSDYFQCGKGRDLGFGSILNFTTKIG

AGMGEQLLSREYYYLGTQMPIDRFLSFFYAHPGFHLNNLFISLSVRGVWKAFARFVHHLL

SFSPLFEVFVCQIYSNSLISDITFGSAKYLATGRGFAITRISFAELYFKFTTSSIYTGSK

IFLMLTFAVSTMWQPALLWFTITLMSLTVAPFFFNPHQFMINEFFYDYIVVLKYFINGNS

HFDKYSWANCTKLQRVRYTGLDKKRLGDMSEKQNIDINIIGKRNLILSEIVIPFIACIFT

TSAYLFINSQTGVKNAVPTTSALRLVIVTALPLILNCVTVSMFAVISMVLGPIAHVFSNT

HAVTGISALTHFCGCIYYLIDFELIFVLEGLKFSKSILLLLAVINIQQLIFRIITVFFLT

KELKSSQPSFSWWSAIWIKQGLGWNILTAPFREYICKCMENSYFAADFFLTHALLYCLAP

ICFIPFVDSFHSMILFWLKPHELSLRQVYYEKKNKKKRNRQTLNGVAMFVLIFTAISVFL

AIPVVLRHINLVETMVEPAIDKLPYVGKLIQPRHQNNNDTGPRAPSSVVKSTPDYEGYAT

VDFW

>34-9_0027

MKFTTTASVLSALALIKQATAFSAKGVSYSPYTDSGDCKTASEVASDLSQLTEFEIIRLY

GTDCNQVENVLAAKSSSQTIFQGLYFMNQITADVGTISAAIESTGASWSDFNTVSVGNEL

VNDGEATVDQVAAYVAEARTALSSAGFTGNVVAVDTFIAVINNPGLCELSDYMAVNAHAY

FDYNTAAADAGPWVLEQIQRVWYACNGEKSVLITETGWPYAGDTYGAAIASTEAQSAALS

SIQDTCGDDVFLFSAFDNKWASPGAYDIEQYFGIIH

>34-9_0028

MSENNINLQAFKLIDAESYLKNERNMAVAENDDDKVNVESEKIDIKSIQKFVTLFNQQTN

EELTESIIKYFFKQIFGDFNKFSNCNFNCVIISGLLIRANYLNDILILEYLQGLLKAKQL

HKIKVLFHILQDKVDDEDVIMSDIKENILQILSRRNIELPLYPEDFEWRIIFDLVDAINK

HVHAYSFTKDDLNTLETEFEEQVVKNPKEASSFIDDHYKHEEKAKVNKYIYQVIKSSGTS

DEVVHKLLSNKENDSFTVISTILQICSKEKLYSRIYENILIKICEIGSIWETSLIKNFEV

FYSVEIEDEEIYPDTFNIKHLSTLFAKLLANDSLDFKTLKIIKINSRETTERKRVFLKYL

FTELVSEMSLEKVKKILLLNKLLKPHYKDTLFPSIENPKDTIYSINFFTAIGLGELTVKM

RKQMDTLKK

>34-9_0029

MSSILIVIDHDNLDLDALIKNNTILSADGTYKEVTTIDVFLNYLISDPFELDYKLNKIYT

IIREKCEYQVSINVFFKHENLKLDKILAYPYTKTFLSENTSLSIPHSPYKEYVNKTKINY

QQEISSSTKFYNAKCFDISAVGGTFDHIHDGHKILLSISIFLTKVKLIIGLTAEKLLVNK

KFKDQLESFEVRKNNVLKFVNYVDKSLVFDILPINDVCGPTGYEPNIQALIVSKETEAGG

QTINKVRKEKALRELDVFVVDILGGNEKLSSTTLRQLQYEKLNKL

>34-9_0030

MSNQSSKVIDLGSPIEKLFPPLLSYEVLYEGGFTWHIDNWKTLEDRVKATNNKKIESESF

CIENSPDDGDFDWKLLLYPLGNNNPKCVAVYLNPTPKNKTSEEWSCCVQFSIFFSRPGQD

DVHVGKVSYYRFSEVDTDWGFSDMIEVEKLKTRYKELKDTVPTGLLIDENNLNVTCYIRV

IKDETGVTWHNFKNYDSKKVTNYIGFRNQGATCYLNSLLQTYFILNKFREMVYNIDTSYK

TEATENVDLALQRLFYNLQTSDTAVNTVELTKSFGWDSSDSFIQNDIQELNRLLLDKLED

SMDLKSLFVGEMKIYIKCLNVDYESSRLEDFWDIQLNVKNMSNIQESFDYYVQTEILEGD

NAYSTDDYGLQQAKKGVIFTKLPNILFIQLNRFEYNYELDKLAKINDRYEFFESLDLSKY

MEKSEGDEIYELHGVLVHSGGIDTGHYYAILKPKDEWLCFDDDRVWRVKSDEVFENNFGY

QQKSESVLRMMGKPSLQKYLMRRQTNAYMLVYIKKDKTNEILKESGDDIVPQHVKSKLIK

EKKAELEQQIILNELRTTFKLKGFDLFANPDTKLYHPDFNNEAHIQLLKVSREIDVAELK

EMEAFKNKNIWLMGYSKSQALRIYKRIETGKLKYYINELLNCYIFTEDIFNLNNKDHYGS

FLLFVKLFGEKEEDDQILPLQVNPFTKICDIYDQVIKKNPKAEPICYEEIGPNDVEMLKM

DSLLIENELSNGDIISFGNDVLNIYKNIRFRIKLFFTVYDGELKHKDYLDLKRMDKYKKM

NKTVSVWSNANDTYEVLAKNLGEKLGIEPKNLRICADYENKQIFLKSRTPLKQYLIKNYT

FDTVPNFMFQILNVSLPSFENMRKIKVKLFTNNNYINYKMIEIPLLKSETVKHLRSILDK

KFEEMSDFKEDKSEGTMTLWTNNSHFRFTGILTDDVFIDEIGNTEIVCARKLQIDESLKS

IIVLQCSKTVNNTHGVSFVFQLKQRENFIDALDRLNSLFGLSKKEFSKIKLSSFSHKTST

TINISNMSNIDLQKIEPYDIYNSSDVLFFDHPDRLKAVYANRQMSIK

>34-9_0031

MPKENLLFQLDNKEFQYHKLNYSLRYFNPRFHTTKLIKNANKHSKNSQKNNKKLKGVSLD

ELFDELTALKKEILNKKLYRLCLKFNSKIDNMPNDKNSAEDKIILKQLVLNRFMKSLKKK

FKKYTAEYGQDLTEMDENAIPNPFKNYEDWITDKDMIKELESNLNVKKLDDVSKASKEIN

KVISKLYQDKSLKLVMSEFEDGMDVFLNINRGVKKVERKELQTKNDQEVYDDGHNEYNES

KYAANDDEDYAEYDNMIANSDEEEDDDDKNYTPTRSTLPELEHGFIDTYDAESEEELDRE

MKQEKPEKKKKNRRGQRERRLIWEKKYGRTANHVQKKIQEETANKLERQKAYEERVVKRF

EREQEYRDKQHAKIQKAKEYASKEFHPSWEAKRIQEEKTKNVKFQGKKVTF

>34-9_0032

MSVETIISSDTFVSHNSLNKTLETASNYLQTISVWKTIVTLIVIGLVYDQVSYQQKKGSI

AGPKYKVWPIIGPFLESLDPKFEEYKAKWDSGDLSCVSIFHKFVVIASSRDLSRKIFQSS

KYVKPCVVDVAVKILRPSNWVFLDGKDHTDYRRSLNGLFTQQALSQYLPSQEVVMDKYID

HFVEFSKKNDYKPQVFFHEMREIMCALSLKAFCGEYITEDQIRKVADDYYLVTAALELVN

FPIIIPYTKTWYGKRVADMTMKIFEGCAQMAKDHIAAGGESVCVLDAWCKLIHDSKNSDS

DDAKLYHREFTNREMSEAIFTFLFASQDASSSLACWLFQIVADRPDVLAKVREEQLAVRN

NDPTKRLSLDMIDEMHYTNMVVKETLRYRPPVIMVPYVVKQAFPVTPTYTAPKGSMLIPT

LYPALHDPEVYEQPDEFIPERWVEGSKASEGKKNWLVFGTGPHVCLGQKYVMMTFAGLLG

KFALNTNWTHEVTPLSETIKVFATIFPKDDLLLSFEKRDPLTGESIAK

>34-9_0033

MNDDLFDEMILLSPEQVNYNLGYKEGEKISERRNIQEGKEYGMMIGYQSFLIIGQVYSIV

ECLILDETLKINEGMKKSCIEIINLIDDEIKESDNDADIVQKNIDVMSKIKNKLKLIMIM

FSKYDKSSSLTYEKIEKVYNKVGNGLIPSSKLNEVEEDKSNLVNGMDTDW

>34-9_0034

MSLENDLKLEIKQNKEYINNLLKENQHLNDLINAFKKRQSKEESIIPSLRAKMKKYYDLY

NIQVEKLNSLEKENKEYKEDVWRMRDELKVLEEKHELEIEEKLKHLRVETDILKNKINSN

NEGNEKHLKIENEQLMEKIETLNNKILELENEICITKESHKTNDTKSHNGRRVSSISSYN

NTFLKDSSHISNDFNDTNLLFKQADSYIKKPIIPNTVIDKEIDLIDDLENVLKSYEKKFE

EEDAKKKRIDDTFKLFEKIKQRMVSDEQKVKVQEKKEVREDLETPLKLNKLEEQVETITE

TLNKLIVKKESKPKKTVHPSLIKRYPRLDLDDVSVNESQIDVLGQRRSSSIDQTQVKHNI

FNDNVLRNVSNATSRTLNSVRTGLDEINSKNDQIPQDADMDTLNKICAEYEGREVNIDEL

VDNH

>34-9_0035

MNFSSNLEHTASKMEDQSSKNYNLATRKSNINSMELESRKVFAKYWSTDGVFYYCMEINN

ISVCRRSIDSFVNGTKLLNAANLTRGRRDGLLKKVTNKFVVRNGIAPLRGVWIPLHVAQD

FAGLEDIEDICYPLLEENLEDAFKSNPEFVNAINKKMKNFKDSTESIEGNKPLLSSKTNS

ISNADDVVQSLKENISTESLMKFVKADHNQLLKPKYLKNPSPINMNMSKTNGSSSVVLST

KLSVNNLYPTPKIADLNSVKFHDEKSSIKSQNDENNFHNETSNSTRVMTPISKIIKNPSY

VTASGNTYSSDITGNTNTRTVINTPHGYVPCNVDSLNRKPYEVNPHVYGHLAHANSVRLN

NHQPHYMMYQPPGNMFGMSVYNNTFQGEDHFNKMYSSSLQQPQSHQNMNYHVNYMNNNYD

DQYHYHQPYIMHNLMNANYQKQMNLQNNNTLNDQPQSEV

>34-9_0036

MNNTNSQYNSTESSIRNMEMNKSNSSKNIESESSSSSRGSMFLNKVVLPPLAQQLTNQHI

IQPHASTYRYPQNSSTTESPLWSIVNKQRPVETNNVFTNYPGSMDDSLAAARLVVNFSNT

QQPHGFHNSSSNLPSMGTFAPTGYNRQDAIPRPDTAANEGKLNNVMVQERENNGVKDKLK

SEDIPVKKISKPKKKEKCMVCSEMFSNLKTHSALHMDVEKRPFKCLFCSRGFARNNDLQR

HQRKHLLEKNLMDVNSGLTKKKNTKNVCATDLGFKCPFYENKEDLQPFGSGHENIDEEDY

VPKRCHSTGIFTRCDTFKNHLKALHFRYPKGTVRKNRANVSGFCKHCSMQFENCKIWADE

HVLKGKCNNNRPFKNYMLVKTIPISGVDKNQNVLLVQQREDSPAVSVERVPTSASVLDLN

NL

>34-9_0037

MDTVKSEPTLHISKRNSFLPKVKYYYRSTCSIILLGTCAFYGIFASIYYNIKGTRQLGQY

ATAKAYALVMKKFLGIEVKVEGRKYLETSSPCIFIANHQSALDILVLGETWPKKCVVTAK

KALKYVPFLGWFMSLSGALFLDRSNRSKSLGTLNNGIKEIIDIKGSIWMFVEGTRSHTTK

LEMLPFKKGAFWLAKDSNLPIVPVIISNTSTINNNKLKIFNQGTIHVKVLEPISLKDVQS

KEDMNAFVDKVETLMVNELKDNVGYATPTSFETGLPKEYAS

>34-9_0038

MEILDKCFFYDLNDILKFKKYNVFDLDLKQTFKNQETVFDTENCNFNVSFPNKSTLNHFT

HITLENGLNLEYQDFILSKLHGVAKAHNGNKSDLSKEHNIQHLILIFDISSSWNAKINKN

QHKTSENFIEIEGITAVLRIPLLKILDSLEHQDNPIFLKVLQFFRLFLSSPTTIADFFKH

FLKLDITDFNLLAIIIDNLTFLSIYDEPQIIELTRKSKLNNLSKSLEKTQLEYAQLMEFM

DYLNELHRTLGTLIVTTSFRI

>34-9_0039

MLQSTFYNLKDLTLEDVGFAASNKLGGAGSSAFSSTDQKKMSIKKIKELLDSNNEVKLKN

AMKAIIYKESTGLLTNEKAMGLYTYVLKNVNCQDLKTKKLCFDFLITYMPLDESSAAYLA

MNSVQQLLHSKDSQARELALIVLSKFKSKAAVPLILQFSKDMLRDFDPIVRRTFALTFRS

CWEQNGINDQDLSDILKGIIRDSNYEVVSSCVSLLKDLIIKDEKFLAILHGYYKRFVNNM

DLFEDYCFDDLMYIFKLYNSKFVLPTSEDFVCFNKSLDKILIENSSSTKTFGALKVLITH

RSTENKDIIIDAFLNLLAMNKNYNEAESLILEYILEISSSDTYGLKNLFANHHKHFGLSS

FRDNEKIQYLKLQIIVSNLTESSVEEVFSLLQTYAKIVNFSPKIRATALYGIIECCNIDS

KLNTKAMAWMVTYMKSSSCKKYPEVNNAAFSLLRVITSNNSVQDNLPAVYLLTKALLTKS

NEASKVFGDDLFPETKAGIIWLVADYSLQNIKLGSQLLKKILHTFAYEDPRVRLQILTLA

AKLYTNYKSSLEGDEPENNVYKQMFGDLISLSKFDPDYEIVDRARYLNGLLNNCGKEICQ

LILQAPKTVLNYMEISESIPLYVTKSLQAIDFADAQDMSKEREDEVKVYDFEQKSVSISS

EDYQKHSLSNDVSSSFAKKDFNNNTRLIRTDDFVSVKNRKDYKLKTMEDFFAEDNVYKAP

QKKVVRKIVYQEETDSEEEDDDDEETGSEEEEEDDDDEDEETGSEEETSEEGAESS

>34-9_0040

MGIILEPDFNNARFHLFKTLANILDVIDLNEFIEQQGYLMEKIWLMLVFLIMSDNSETKD

LQDDTITKKQSHQEDMNSVLENESKQIIKKVYKKIGFNSIVKLFKKHINDETMADSIAMC

ISLCDSNDHDVLSFIKMAMENNKLGNVRFIGCKSIFYLSANTSTQSELDITFFLSLISKM

LLDPDLKNKSVAILTTNLIISKDKKQVFDVYEQTVLNNITNQVQHARGKLREYHLRAMSS

LLPMMDQENLKIYGEIILKFCMESLGAVFDEKSKTYKKKNEKEQGLKNQNEDFKITVLLV

IQNLYKLEDSSDETFFFNSNMKMFLLHIFFENFWTRHTALSSAKLINTVQFTTNTMFQKV

NTELIFEHFVECLKDNLEVLREMVAWGIVKMLNSEDQMNLVIKEETERVIIDALLFSFQD

QKSDNQCYVRCFDVLAVKLGTNFKPYLLPIISTCLQLMSHKNSSMRKNAANLASRLVKTC

KALDEQGLIYKLGVVFYENLGEPVGEVLQYVIKCIHEVLLSIEDIKKLEPPVTQLLPALT

PILRNPYYGVSYNLLNIITLISEKCSDMIPPREWNRICNELLELFKTPIKDLRIKANDTF

GVIAAGVGPQDIIGTLLNNLKMSERQIRLCSSIALAIVAKSCGTYTVLPVLLKEYSTPDT

NIKNGILKALAFMFEYVGEESKDYVYLVVPMIQHALTDRNVVLRQTGATVVRSLAVSCKN

CDCEDAFVHFLSLLIPNIFETSPHAIEKIRPAIDGLRVVIGTEILMQYMWTGLTHPSSAV

RNAYYAVYNEIAKEENERLVPLYPLNDIPELKHIL

>34-9_0041

AGYLARDKGYFYAFLLPLLIFAIIPFILIALKPRIKDPEPVGKSVIQDFFKIMKVCLEKG

FIQRFRKGTFFEYALPSNLAATQEGRNSWIRSKPQSGIYDERTVTDIKLTLKSSRMFLYW

VIYYINDLGLASLINNLSAAMQSNGVPNDVINNFNPLAIIVFTPILDYGIYPFLTKIRLN

PNVTIRIFIGFMLAAGAMAGGAILQRMVYNKSPCEIESQDEALLKLSLMPFFSKLTEDRK

IRYIKEYKIKGALKSFSEGKKPENISDRQWKHVMKLRFQELTRDAYKEYNAEKKKKQKEK

KKEQKKNKVLEPKVGDIEFKEKEERMSNNIKLVIDCSFDDLMLPKEVKSMSTQITRIYNC

NKLSTNPFEEILVSSFNKRLRTRFENEITSYKKWSNEGNLKFVEEDILENSTSIVPENYV

YLSADASDELVDFEENKTYIIGGIVDKGRYKKLCYEKANKLGIKTAKLPIGKYIKMHGNK

VLTSLHVVQLMNEYLKNGKDWEVAFDSIMPKRKVIE

>34-9_0042

MSAKTYKRFNDSDDSMAVFQDCLTTFNESPVNPNKCRKLLSKLYRLIINSTSNEPIFKPQ

EATSLFFSISKLFQHQDNDSLRQMVYLVIKELSSMSDDILMATASIMKDIQSQDSIIKPN

AIRSLVRVLDSSTALTAERLLKSSIISSNNDISSAALVSAYHLLEISEPTSKRLINETVE

SIDSKKVNSKLPMKLPASDTNSENFERLQYHTAHNTHMSQYHSLALASSLKKNDEMGLLK

LIKQCDPTTVDNPFARMEILRLCHRLVLKNSSKIDIILPYLDWSLNGSSTNNFIVLEGCK

IIFNLKLKDLYHSAIDRLSYLINDRAISSKFAAVRLVSDYCHLLTPELANSIAADIEVLV

NSENKSVSSYAISTLLKIGNSDNIESLVNGLKGSGMSDDFKIILIDSLAGLINRFPKVIV

DFLLDGLKSHDGSNLLKNKIVNSLSILFENDAINNNLDESFKINILESFCDFIEDCEYTD

VLSRVIYILGEYGSKISIDSSVFVRHIYNRINLENSIIRAAAVTALAKFPTITEPLLKNI

AKNDLDDEVRDRAIITVSSLDTPNQEAIPTVNPNDLIHSDRYLRILELRMNKYTEKNDFT

NFDIPAEYEIDIKEIEATDNTNTKNNNGDSNVSGELSAEDNEYSQDGTDDGNTIGTNPTD

SIIESKIRHKYTELSESILNEVTKSKKLTDSTSEFIISCQKFIFTLNEDDKKEILAFKIE

NTMDFPVQNINILDSEDVIENIEPQERVELLVRAENLVLESGKYWNLRFEVDDFEEIFEF

NALL

>34-9_0043

MSTEEQKIEQPIEQPVEETVAATEPKAEGIVEKAKETVNEAVESAEEAIESVEKAVKSKT

EEGAATEETSEPAKKVNKLLAPLKKCFAKVNRRKMSIFDDKIAFNWLFSIYDPNSRNEAL

LLLGKNRQKFPDLGKVIWNSPGMVTILLQEIIKVYPYLTGSKDHVLTSEISTRICNVLVL

FQCIAFDEETRTDLINSEILSYLFPFLQCPQNTVLQEYATFKQLDVHDKILKSRPFEYLR

LTALGVFGSFVRVDSSNVVEYLISTDLVPQCLIIMETCSELSRIVALFIFMRIVLNKKGL

NYVVSSENRILSVLSVLKGMIYSLQLPKELQPNASNTNDQSNNGNNPPGRTFKNVLRCYL

SLCDVKELRTIIKDKIPPEIFYVMRSKDNLTEPQKNTRLNIALENGFNIDTCQLLLHIKN

EDPTVFKLQVQLLQLLGMPVAP

>34-9_0044

MSSLPPNVEAGRKSDSYDTEDLSGSEPAFKRHRAEYNYDEEPVVPELESKVLPEQINYEF

SEVVPSIMDYDSSDDDINSELLNESSDSSDGNTPENPKEEDDDVEDPMNDGRLHNEEEDA

ILIDDLDAYPSKESINDDNDKKIGEQEVSNDIATDKKDDDDADVQLLKNEASKLHVTNES

DDDDSDYDYKEDSFKWELDIKNPEMEIPVLRKRPISLKIDESTGKCIFPLITKEKSLHCR

SCLKYYDNETFLNAYIGNDQPPAGFYYLLRLLGFRMTNEKLLMKIMLSILNPENMGLPKK

LTEIDELSLENMQELLYFDKSDCDHLLKDLTIALNNVLVGRIRLPNFNRLDQVIEKIKNA

NKILVLTGAGISTSLGIPDFRSKNGLYEKVSKLNLDDPQDVFTLDIFLENPSIFYSIAHE

ILPPDGIYSPLHAFIKLLQDKNKLLRNYTQNIDNIESFVGIDDDKVIQCHGSFATATCVT

CNYCIKGTDIYSYIRSNTIPLCPYCLEEK

>34-9_0045

MLKSNINNGGYNSTVGYACPAKVTNPYISKDLNVLNPCFISLVQKYVAIIFLISIWYELI

NLKGPIKLSSSLFKVTKKHAFNLLIVLGSIGSVAYQLKINKVFYNDSNTYLKNNAIWNLI

YLLLGSLPMQYLQYFKTPAVSTSNIFYNTIQAFFYGFRIYQSSSLSASDYGVLPSEYSSI

DVSLFFLTLKNLTYELFIYVPNSEITEYWEESKSSGGEYPPSNIVQLVTFTWMNPFVVEV

YKNGKVVDPYNMPIPPCHLDAREATDSLKASWAEQLWKRDLNDSSAVKTKHPTILFSAVW

KTFGWTVSVAFVYELVADVCSSIQPIFLQHFLRSFSVSESSYPLLNSFFLALTMYLSTIF

LTIFHNQFFIVIFEAGLSIRSSLMSLAYQKALKLSQASRDKKTTGDILNLISVDVLNIQR

FFENAQSIVGVPVSFVIVMTTLYKLLGEAMLGGIVAMIVMIPINAKLSSITSSLYDKNMK

YKDMRSKLMTEILNSIKSIKLYAWEKPMLAKLTHIRNDLELQNFIKLSIYNNIIYFAWTM

VPLFVSISSFVSYAKLTGKPLTAEIIFPALSLFDMLGEFIYAIPDIITEIIETKVSLNRL

QEFLFFEEINDTYVIREGKNKDKSIPAVEIKDASFIWANNQNNEDDEKKIESETSNDEEN

VASGVRYALKNINFKAQTGGLTCIVGRVASGKTTLLKSILGYMSAIPSDETGLKTPQIIH

RFNSVAYCPQQPWIMNASIKENIVFGYKFDENIYQATLKACQLERDLEIWADGDGTVVGE

RGISLSGGQRARIALARAVYSRADVYLLDDVLSAVDAEVGKNIITQVLSRETGLLKDKTV

ILSTNSIKILKDSSMIYALQDGSIVETSSYKQAVSNADENSVLKKLIDEFGHQLNESSES

ISEGEDVKDFVSEEVLKNANDKTAFEDVLLESGNDLSQKTNSADISEIKSMISRRVSIAS

FKPNRVLIEQENDNRKTKQKAETKKEGKVSMDVYLTYARACGIFGVCIFFFFTFSANIFV

FVEKWWLKVWADAGELEGGPTHTLFYYVGIYGLISVSVCIFAVVRSISSTQFSAIRASRV

LHNDMANAVLRAPMEFFETTPLGRITNRFASDISLVDQNLQFILSFFIRNCLNYVTTVII

LSYSMPPFFVLNAVLIVIYYYYQKYYVVMTRDLKRLMSISFSPIMSVLSESLVGESVINA

FDQYDRFNYFNYENIQFNVNCVFNFRSTNRWLTVRLEIVGATIVLFTAVYCCISTGSNKP

ISAGMVGLLMSYSMQLSGSLMWLVRMYTQVETNVVSVERIKEYCDLTPEAPMEIEYNDKQ

LPENWPEKGEVIFEDYSTTYRKELDPSLRGVSFKINPGEKIGICGRSGSGKSTLTLGIFR

ILEPLLGKIIIDGIDITQLGLNYRKKLSIIPQEGVAFEGSVRYNLDPFDEYTNDNLVEAL

KLSHLYPHIEKMCKEDIDLKPENQSADAPDVAKTEVTIDQLLETKITDNGSNLSAGTKQL

LCLARALLNIL

>34-9_0046

MSHRRKAPSGPIPAKLKAALAAKKGISKESTKPENQDPIIHPMTQANLTAYEGNAKEIDV

LNQAVKIMNTDPKQTKKILKLVDTNLKKNGVHFNSLLLKALVNQKSDNVIEAKANLQKAM

SKVPGSTKNVLNLNIITAMSCQLISSYYKECEDFENNYIWLKRAYLKSGGHQNMGNVMML

NQLCLLAAQLRKYGELLEFREHHLAARPGFRANWTGFALALDLNNNTNAAVEKLTQFEQA

AEGKITEKEAFEDGECIFYKNELMLKLAKNDDVKLKEVLQHLEDKKALINDKLGFLERKA

EVFLKLGDLNSASKIYRKLIHRNPDNFKYFKMLEITLGIKNDNGLKYRMYSNLLKKYPLS

EPCRFMPLTFIQDDAELKTYLKFYLELYWAKGVFASFQLLKPLLRNLKLSTKFQKLAEEI

CLERLTSLEENIKNDSQSKDKLESIIRYFLSNLYLFQRNYDKALNEIQRCQYLDAEGKTL

EFELFQAKILKHMKEFKTACELMEIVRNKDTKDRFLNNKSCKYHLRADNVDKAVELISMF

TKNDTSYCDTGITDLHSVQNTWFLSEKAEAYSRLFQKELVKLKEDIDEVEKIKIAKSALQ

YKNLAVKRYEAVFRIYGIYKNDQHDFNSYSLRRGTVQPLMQLIKWADKIHTLPVYRRILK

GMSELVKSIPEFLDIVNTFKAPEKIFLINHSPKEIKQVAVCAPEVDDDPFGHKYDKINQL

NTLAEVLIKPYFDKYNSEVSKKNREYTLSFVINKWLQNDDAVQNKLLQDYEEILNKSSQN

GSLTFQDISFNKELLSAFKQYLKNESIDFQSTAGIFNNLLINDLVAFI

>34-9_0047

MSYPVIPLYACALFFYMGAEVGTASWIYTYLLEYKKGDSTHMSWIVSSYWAGLTFGRFYF

GMMINNWFVNEYQALKFFIKMTTLCTVCFLLLGGIYSHSALYFTSFGAALFSCGMFIGPI

FPLLSIIGIDILDEHVQVKGLSSAISLGSIGAAFMPFLDGLLMNHLGLSSLPFLVLISTL

ICLVAIHLYPVLIKGKDHYFNPKPRSSSINL

>34-9_0048

MENNASTNGPLKKKFKIEKTVDKQGFRTTTNKATNQEPEQIVYTIDDQYRKVEPYYFTYK

TYCKERWRNTNIHELLTTEFRARKPEDYARAIDEGKVLVNENPIDRTYIIKNGDLLQHTT

HTHEPKVNNKPIKIVYQDEEMIPSQTEFKRISYDKESNTSIVLCKPLTGRTHQIRLHLQY

LGYPIANDPLYSSPKLWGPKIAKNAEYENSLDDICKNLEEVGKTTVSTNWWCENNNINKD

EGERFTGKHCEVCGGDIHEDPRKDELELWLHAYKYESLKNEDPTKNWSYSTDLPDWCTNV

HAKYMEMALEEADKCEATHKAFNVGCVITHDNEVLSLGFSRELEGNTHAEQNALQKLKDK

NIEIPKGADLYTTMEPCSERLSGNLPCVDRIIEHKDKIETVFIGCAEPQTFIQYNTSIKK

LIDSGINYVILPGFKERAEKIAFKGHLK

>34-9_0049

MSTAKPDLKDVNPRGIPKALFIDLNDTKITSNLELTVANVQQNLQKYEYMLKSKDQQLKS

ITDITKDIKTNSKVIDTLIAQNADDSDDEDGEFQDIQYELEDGLFAFASIPKKSTTNTVS

LWLGSGILMEFTYEEAQQILSEKSAVYTNKINEIMEDIEFLREQITTLQVNMSKVYNYSI

LQKRKLEQQQSVKA

>34-9_0050

MIIISSEKNNTYTNEGSTDIADMAAEEEMNHIVDDEYSANEDEQPLDTIKVLPRSSYLST

RKNQIAASSTDTASLSNNSIKSYNSQQKYDMNNGGKKLSILYGAALMEDDVSMTSSDISR

DLEIIQMKETIKNLQNTVTRLEDFFTVLSKRLDDMEKS

>34-9_0051

MLNRSLLNREVFNISKPSLLTTKLYKSLDFKPKVIDTTYQEKLLKKAKERGFNTIEELKE

SLKEELEKKKKDFNQVDPLNTLKKYQEAQSKEIEEDMNKPTTKSLGAIPENKEKKPFKTL

SSYVDKEKFMELSPQEIEFLWRARFSTTKMAENHLHAVLQKNIWTKMFDNAKENPVFVLP

LPRDITPEQNTDLKTGAELFYVQWSFPDKFNTHCIITSLVEYQLHKEFAKPYLTISFFGD

MLERKNILLMVGQLDQDSSINMNDCTLLLLNLQRFYGVIEGGLVNERLAMLRAFNKGDTS

FDVNKLIIMSRQKVTFLGPVGTYSHQAALQQFPDSSKYELIPSSSIPSCFEAVLSNNEIE

YAVIPLENSTNGQVVFTYDLIRDVLNKNNNDESSKSINDYNNRKYPPLNIIGEQYVKIEH

CLVVSDKDDLKNFDSINFVNLYSHPQVWGQVSEYLNKPDFKGKFKNIIDCKSTSEAMLRC

KEDKQKGIHSLAIGSGAGAKVNECEIVEEGINNMKGNTTRFLTFVKRKDNEKQLSTLLLN

SESLNEKSEEKIVMLTFTTLKEGPGSLVEVLKIFQNFNINMTSISSRPIGQQSGKWQYIF

YVEYDYIEGLNWRDEILKEIDDRCLTWALWGIFPRDPGYYV

>34-9_0052

MGFIVPGRNNTKLLSNKSKVKVNESNILKALHYNQLSFRQEKKLHVQTVKDHKKIDIYDI

PSDVLKRIFLELDISDVNKLPVFLRSHFNYIYSHDSYWRYSYINMRLSKESNAFILEMYD

YDYYLEVYLNSVLSGGFNNVNSINNIKNLMPDIQLNDRTTQILTSFVFKYAELYKKEANY

QEFLWAYMPMFIHISNKSNIFKEHPWCCYMLEQNCLLMYKNNTAMNLGEVLDLCLEMSDT

MYSMKLANSYTVKEPRTTNKSYFRGCFIRFIILNMVKNKVFHVHEAQSFANFIKNLIIFV

ATDDIQYNSVFDISKNYEEEYDVKDVEIRETFRNKDIWICILRAAIKNINRNEYEKQAYI

EIYNLLIKQMNYQDENGSSKIPDVMGLL

>34-9_0053

MFGNIWNTKDELPELGEIQLSKEENINKKNELKKLIKSLLLNFLLLITNLSVNTDLDIAE

DSKENHLENIRIILVNIHHYLNEYRPHQVKENFIMLLEEQISFKKLEIEHITKVVKEVKE

ELSTTITALVASFIGLGALYYNQNSLIYPSWVNNARVYVRKPEFPLSNYFQEDYITTTDN

ESIQVYSFIHNEEKRKTLLMFRPNAGNIGDSIPLIKILFESHNLNVVVWSYRGYGKSSGK

PTEKGIKVDSESIYDFLVKKELIYESTNELIDKPLILHGTSIGGAVAINFAYKHKDVIDS

LILENTFLSLHKVVPYIFPWLKYFTFLVTDKWQSESIIGKLDPQMNLLILNSMKDEIVPS

SHSKELYELSNSKYKKIHRFNRGHHNDLIIQDGYFEILHEFLAQREIII

>34-9_0054

MRVPNGNNNKIYDSYGTPGITHSVRINTDISSIDKIHDDSRRDRLIVTTGKYNLSVYKVV

DTDIQDSTTIKHASFIKKQEKKALNEIILMKDLLHNDVFNSNSSISLNTSFNTGNMSNVD

LYNSYLSVSIPKDTKNHSNTANMSVSPQGSFKLLNHNNSINTGLTSSGSSPSIHLHNPHL

TLQASRNNSTNSIKTKTISTNSDVKTGYLAHNNIIAVTNTSTTVSLYDINRMEKNSIFGV

IQEHTRTINSIDFHPLQSHSLLAGDMNGLVKLYDLRKIRSSSTLPLKNVSDLTITTKQND

AIRDIKWNPNSNYSFATVHDSGSILKYDIRYPSQPEKKISAHDGPGLCIHWMYEGTDYLA

SGGRDGKLCYWYMGDSRPSLGMPEVSINVGSSIDKLKFRPKSKHDKHDTFQTQSGYLMNN

HEVAISCPKDQSKNNIIIYKPSQMYMPLKVIGTQRSTTGFVWFDENNLFSIDKGNCINGF

DLNKEPEMKNNLGGKGMKWRDIQGDGLVFASPIGVKERYEEVETFLDEAEEANIDDQDEY

NNEVSSQLISTTPNNATITSSFGNNSFKNSFSKSPVMSSSNISFVQRQRNNLLLKEKKRM

STGSPSRENLFLANSENGKCNNSHNTFLMNLKKNGNKKMANSVDKTGSSDESPFHQLMKS

HSNWTSITSHNSIDSNNTNGRLSTNSHVSKTNSFNNLSMMGASKKQENVIYSLSLPLIYD

TLVNLEEGEKKTEWSVKMNENILKLRVDPVRRFKYLSRNLAYANVNYDRGEEQIKDKKSD

YIDYEKAERERIIQNLGFHDDWLATTEDKCIASTLDNTKEVQKTSVVKKKVDDNVTKPNI

RIYKEDYEKLKRMCSHNSKIYEELKDLSKAKLWNMIGKGINYQLDLLIKMSVRVCMIS

>34-9_0055

MSDSKRISLSSYPVNRFSEENNADTYVDPPRPSRIQGMMNMGEEENSDEFISDYEDEKDK

LASNDLKNLYPKRPKDNSFLQQRLKSYNPIFTPGLTISIFLSICAIFVLFGGILISISKK

TNEIKIYYQECSTMASTNDYTTIPKTYVESYFHQLKTADQDILKAEWKYTPDSNAVDTGN

SFSENGTCHIRFTTPYVIRKPVFMSYLIKDYYPNHRRYALSFNEDQLEGKETSINDVHTS

TGIKCAEIYKDSETGKQIYPCGLIANAMFNDSFSMTLVNENNQNQNYVMDNSAITWSTNP

NRFKKTKLSYKDIAPPPFWKEKYPDGYNETNVPDISTWQEFQNWMDNPALPIFSKLLGKN

TTQNLLPSTYQVDITLNWPVREFSGHKAFYLTHGSSIGGRNNFLGEVYLVGGLVCLAAVV

VVIGGKLLSGRRSADKSRLSWNQ

>34-9_0056

MLKSLLNKRFNSSSSFKYQVPTYNRPSDIIITSGKGVYLYDSINNKKYLDFTAGIAVTAL

GHNDAQIAKIISDQVQPGTGLIHSSNLYYNKPSLDLGIKIVDKTVQSGGMHDAKQVFFCN

SGTEANEAAIKFCIKYQETKLNKENIKYRFLALKNSFHGRSLGALAATYNFNYRKPYLSS

LMNVDFIDIADLSTLKDSVAKAKSNGEVLAGCIVEPIQGEGGINPIPSSLLIELKKTLTS

HDIPLVYDEIQSGVSRSGKLWTHSHYPQEAHPDAITFAKGMGNGVPIGGIIVNDKFTNVI

KAGDHGTTFGGNPLVCAVANHVIERVTEEDFLKNVEKNGEYLQQQLQKKFVDQYSDICVT

VKGKGLMVGLEFKEPPKELVKQCQENGLLVITAGKSTLRFVPALNIKKEEIDEGLVILEN

AFKKIYQK

>34-9_0057

MIVNNLFFKVLLLVSSFILSISSNENILQKRDYVTTSIATHLTTLPGGNVITIQAATVIT

VTEATTPTSTTSDLTTTSTTSAPTTAATTSSTSSATSTTSTLSSTSVTTAVAAAAVDTSS

TSTTSASSLATTTTAAADTKNTADATKGVGVVTTGDFLGTAKSTSSKATGSASDETDIYT

GPRANPSTSLTPLPTAPVTTLQIESYETITMKHTTYTTTRAKTSMYVTLTVQSMVEVIQT

TFAQRFKTMYSSTFSGSSGSIGLGTLSGEIGKVKTSYLYTEDGTNNAMMNSNNWGLLGMF

LSLLYMFI

>34-9_0058

MSTNNLLKILSDDQDDDLTQPPEDITLQDRQDAINSIHPFGIKIWKPALYKKDRSIDQSV

FKDMYNGIDSKNTLPVTSRDIKHGEDELTIGIKLCNVFYCLTIGFSLSLVLFFLGLLTFL

LVLPINGFTEAGNTFRIFFNMISFIIYPFGKVLYLDHGVLSNLHSEEVTKRTVAIGTNYN

SMPVKAKYTINDSLRMKITKTWSNLVYKAFVNIVLRIIIYIYVAVLWLGVVSIPMSHMLL

VIFSVLKKFPLSYNVASLKKINVSMNNENRSLLSNDIKLQDILICTYKWCGWKYYKLTID

GTNVIVMNLLLVVVITILDFFVFKQFNDLTLFILCLISTFPLSFYIGGAIASISISTSMA

LGSVINALFSTIIEIFLYAVALNSNKGLLVEGGIIGSLLAGTLCLPGLSMLGGSFYKKTQ

LYNPKSAGVSSAMLIFAILTLLIPSWFYIIYGGVIMICEDDSGCRFENRPLQEDDFFKNK

IKPLSGVIAVCLALVYIMGLWFTLKTHASMIWTIPNTSKQNEVTTRSSVSGSTRRNVSEG

VITKTDSIRRDFNDSQSPARIIHQMTSVSSLEENIKGHDAPNWNKFKSFTILLTSTILYS

LIAEILIHSLDFIIKAFPILKPKFLGVTIFALIPNLTEFLNAYSFARNGNVALSMEIGAA

YVLQVVLLQVPALVIYSIIKMDFGKEHIGSIKEWAFTLIFPKFDVMATLSSVFLFTYLYS

EGKSNYLKGALLLLFYTILIIGLFLQSGLDFD

>34-9_0059

MSTEEQPETKTLNNVAELDNSKYIAKLDNYKAKHPLKDEWSFWYVMPKKDETEQWSDLTK

DIYTFGTLEEFWGLQAALPSVENLSTEYMFFKKDIKPEWEDPKNHHGGKWVISLVPEELG

PDVITKFWGRIMFSIIGNNFINNENVNGFYISFKKHFFKLQVWTNTTDKDVVFPIGQQMK

ELLVNSEFGMHEESQMRKTNSFNPTRFTNKNPLYSQLHNATQEKDKSYKKPHAKSSIPVD

QIKIVFNHHNRDKESNEIVINLIDKA

>34-9_0060

MSSNPLQYTWSFFFNLPPSLTDPLESWEDQLVKAADINSLEQFWGLQNSLPNVKDIKTEY

MFFKQNIQPKWEDPQNEKGGRFVIHLNDINIENFWNRLLFSILGNDKFLVDNDLSDLING

FYISYKRKLFKAQVWIKDCTNTEKIQQLGEFLLQLLNKSNGSIEDEVEGKTVEVVNEISS

DELEERPYKIYFFSQDRKNPDCVNLSFKL

>34-9_0061

MDNNKYQLPLSNFNSNDSYLTILKDNNKDDEHSSDDDLIDNPYVISVNQTFIDTPQKTSN

EMFMNHNKINSLARITSVNSGSYIPSEDIRSMTPLSNQTSSGNTSRQVSNANLKKMMLMN

AHNNKNSNISLTSLNNKYKVQDSNFGDRSSFADLMSLNNTNVPNKTKFQLHLVTSREEDD

DEDDFYFSNDIKKLSTQLQQSRLLSPNQDDNNSETNSNFSIVSSLEHRSPTNNTMNRTVP

EPFNKVGAKKGRKYKVKKHYKYIFILSGLPASGKSTLSQNMIQFMTQKYLKGEYSNEILK

DNFSTAHKNSAIKMDKNDLKMGIDMYSKGGDSVRKRASSINSNNDILEVDRHDKNCINTK

LKIEIFNAGKIRRSDSYAKTKQMYMLSSNSNEDLFSPKNKSKKDVFAKLTLNSLFRNLTN

TDNDLDMAIFDATNSTRERRKFVVEEIYRQEQALLKNFLTMKNLLDEEEDDDDEETIIEQ

IEIIPVFLQIMCTNRDFITYNIYNKSFNDDYYDKEFDFAIKDFSKRVIYYSQEYCPITHQ

EIDELQRASMKGNNLFFINLENSKNSSYETNITNDDLHKDLILDGLIDFAKSYNQLPESI

SYVNKVESFYTNENIQNSENVQILKHFLNDDYFQRLNKNLRKTCKEFAIDVPSVK

>34-9_0062

MSDISNTSSNENGKKLTAKHGFKKPAINQSNNILYRHHFLKKLNISPSEYSRNQVNKNPY

ILHNKRKTILHGLVDEPSLVLSTNPSPSLSYQRSINTITKLHRYILTSSQSSQNPLYQSA

STTILNANMQISSIAFHPTTKRFIAATGTGELTYWNVKDTQMENITQGHDSIIKKIIMSK

NGSWMVSGDDEGILKIWQAGNLRLTKTIENTRDGLEKELLTLGNVRDMCFNWDDSKIVVC

GDDKLVKIYDFQNGTMEKVLKGHNWDVNSCDWHESMGLLISGSKDNMIKLWDPRSSKCVK

TILSYRSSVNKVRFQKYTQNSNKNFKFYALGKDSVLKSFDMRNMKMPIKSYKDNNFHTFE

VDTNNGNRLACGLKTGSLQFYDTSDDLNTFYHEIPSAHNGTINTLAWSPRGNLLLTNATD

RSIGIWGRGEIDNEQLYYKPNTSKGK

>34-9_0063

MTQFTLNTDLLLDALLQNLGLVVSYFFFRTSLAKAAIIQQIYLFLRKDIFLINEWLNRCV

YNYFNPKNKWKSLEEIIQSDKKVLCVLTGGSHGLGKQLYTKLSRKYCKKYSNFEMLIIDK

EPMDHDAETAENIFFIQHDFINRIENKLDKFKSKDYDYKIVINNCGTRFEFHDFLETWKD

SSRFLELLNINSLSACDLLSIFEPDYIVTISSILSMISPKNGFLYSSSKNIIANMHESYV

QIENKKGMLVLPGQLKGTDLFKKFEPLFKSSSKKFFSPLVSTDDLSDEIIEGLEGLLNKV

IVRPYYGFFIKILTFMPYWLQKPLRVLSSVDEVEN

>34-9_0064

MKVIYPGYGTPKFEAPVLLNGTLHTKVETYIEEVSKYKLSNFKYCEVCDFMKPERTHHCK

TCKTCVLKMDHHCPWFGICIGIKNHKRFILFLVASLCFTAVIWIDTFKHVYFGIKSMNEG

DERTVQFKLCYLLLIATVFTLTLVVFTGYSIYLVFKNTTTIESMNIQDKKDKRDLLMEEG

HENIDEGHIYDLGYYQNWKQVMGQKWYQWFMFSDHDNEKLLSSTHRGFLFPVNDDILHRQ

QDLLNRGLI

>34-9_0065

MTNQNIHNMYVKAIPWEHFEELRGCCNEEFVCLSRNYILKNEIFVKRLNKMTIKAGSQNM

EMYVKIVCLGLSEEEVWVSPSLYYELSLNSLIDDVELETKAIEQSRKDIITTGLKLQRCL

NQNHIKLENRNVIDQKLIELLKGEYANSYFRNGMFIPIIAFGSIVWFRVVSDIKLPFKLA

HTDECTIDNKIFEHPFALGYAQYPNWHFYYSPKVKKFEYGINWPAYEQISSLFLKEKSKK

GLLLYSDTNGESVGLFGKEHMIRTFAECNGFSIIQIDIIDICIDNEDMKSRYVKLRAMTD

IYTEIFDQRLEPKYSNKIIFLFKNVHLLFPKDHEYDPIDLRIYSLFQSLLVKFDLVFTSN

SLEMTSMDCIRSLCVYESYFDLLNEEQRYDILKQSDFLAYIQTKSNSIDPLFLESIAKIT

NTFTPYMLMKLIANCKIQSLHTETSIKAQIEKMKKIKANKAGAPTVPKVLWDDIGGLQNV

KQEIIKTLNPTDSFEKKVLKRSGLLLFGVPGSGKTLLAKAIATNFSMNFFSVKGPELLDM

YIGESESNIRKIFKKAFDNKPCVLFFDELDSIASKNNSSNMDRVVAQTLNEIDRVNQSGD

LNVFVVGATNRPDLIDDSFTRPGRFDKMIYLGVIEDMNEKINVLKTLTRKFTMDFAWENV

HTFINEKVSNNLTGADYQNLVNQVYNHVVEERIKELEKKHADIDKTEWNDKFTDADWFGN

ESCVLTININHFEYVLKSFRSSISASEIKKYEALRDKYCD

>34-9_0066

MVYNDIPFDGIKLRENDKRRVAYFYDADVGNYAYGAGHAMKPHRIRMTHSLIMNYGLYKK

MEIYRAKPATKQEMCQFHTDEYIDFLSRVTLDNVHQFAKETVKFNINDDCPVFDGLYEYC

AISGGGSMEGAARLNRGKCDVAINYAGGLHHAKKSEASGFCYLNDIVLGIIELLRFHPRV

LYIDIDVHHGDGVEEAFYTTDRVMTCSFHKYGEFFPGTGELRDVGVGKGKNYSVNVPLRD

GIDDASYKNVFEPVIGKIMEWYQPSAVVLQCGGDSLSGDRLGCFNLSMQGHANCVNYVKS

FGIPLMIVGGGGYTMRNVARTWCYETGILNNVLLDENLPYNDYFEYYGPDYKLDVRQSNM

FNANSPKYLDNILINIFENLEHTKYAPSVQLNIVPNDKENLGDVEEDTAEALDTKGGSQY

ARDNIVETYNEY

>34-9_0067

MAVKGLGQIDAKYDGSKLKVGIIHARWNDKIIAALVNGCISKLKSFGVLEENIIVETCPG

SFELPYATKRFYEHLHPEVDVVIPIGVLIKGSTMHFEYISESVTHAIMNLQFQLNKPVIF

GLLTCLTEEQALARAGIDEAKSMHNHGEDWGAAAVEMALKYE

>34-9_0068

MDSAADLPMEPANDMSLDSQKDTTVQTTKPSIVKRLYVGNLNTIIENETLEILFKDIVKR

FSRYGKVLKDKEDLSKSSVYVTKNFGYVTMKFEDISKYTQLLKSYNNVNYKGNKLIVQEA

KSSYKEKLNEEIKQNLLFDNNKYKKLAMKKDYEHYKKMKNIKLTFKDRLQVLPGRHRVTK

RNYTKSNKDEVTGKLVKYSNKRRQTFRIYLNGALKTFVLSNKKKLWGVQKGRTIRDLTFE

FDTNTLSWRNGLGHTIEHLNYNKLGRNERLELDSRKDTEDLGSMDIEEEEIDNDKLADIF

SRIDFDKQVEVGEDGLGDKSEYGIDYELKHGFYDSDEEEDEEPKKEKVKESSEESDAQTE

SSSESESESSSDEEFMPKFGVPKTSNTETISNTETLRGLFQSKNTSEQENKTSSLKLIVD

DDDIDHEKDNEIKHMAEKAKEYISKETTTQLQSFDNNTVVKRDVGLFFPHFNSFFLQPQT

SLNKIKTVQIETFEDWDEKFWENRGKWTSEFKNRKRGELKKIRKRQTNSTVLL

>34-9_0069

MTTSGVSNSYGEYRNLMEWPSTLDIPLLSSEDELVNIDFKQKESLLDNPEDFTQLLIEEN

CNKSYWLTIAINYVKAGKIKQGIKLIELALNEFQGPESAHLRTFLAWAFLDLSKSSFQNA

DNSDKLDCLKKAEYHLQNSIQFEPIWIGNMLATIDVFYQKGEYDKALETLQLFTKAITSN

NSQQQNSNKNVFFSLMKAKLLYQKKNYLGSLKIFQELIIMNPVLIPDPRIGCGLCYWQLG

DFEMALNAWERSLELNPDNFDVKILLLLSKYHATINKSLNDEQFVGNYTSSINDLKKIYA

LNKFILKEQKDRSIEMPEFTHVEEINPSVFILLQMYYFFAEEYETCIEIYEKKFENKLEL

QVSKEIASISLFWAGRSFFAMDNLKQALKFFQKSLSLDEKNGLSLFGLGQIEYRDNFLDE

AILTFQNFNEKIDKHSIEGNYVLGCLNAAKLINVLNLNDRSAETVIGQYLQTDAIRSKSI

EYLETYIKLVNINKKSQMVSLKAYIILSQLYEQKNMFEKCVFYYEKIIEQLKFLKNDVNW

DISNNLACYQYGIGKYEEAKANFDIALEKFEQDSEIVDDKIKYTIEYNIIRTEEHITKEE

DKSSILKKYNDLIEKIPENMNLYVQLRKCLLDKNSTELFKNISDENPYNLEVRSIHSWMI

KADASLGIEDEIIHNKFTLEKVSDKDSYALITLGNIYINICRKLKRDGQNPSEYKLKNSY

LKALKLFRNALNLDPYNVYAAQGIAIVYAGLGKHTESLQILRRCRDSINDKSVFINLAQE

LTAINDYIKAIDNYNIALSKTKDLIGKADVLNMLGRVWLLRYYKDGSIDFIYRAVESNQK

AIHFVEEEVGVDTKDHVYEKFMMDLKFNQISLYMILISKIVKIPQGQRSITKMENVLVNV

TDIIQFIEFVLNSKDLIQFFHPKDLSHKYDKLKGELIGNLTDAIEEQKNFDLALENRLKE

AKEYVENEKLQETKEKEEQELKEQELKRIELEKYAKLQEEAQKIIDERQELIDDENDVVV

DENKDRDAEFVVDKPKKNSKKRKANVVVEDDEDLDELEAGNESYASLPRSKRGKKSALSS

EYIEESDHNEVELEEAPDALLEDAEPTPEVEDEQ

>34-9_0070

MAKQEHNNKQAKASKLKNTRTIRTIQYNENNDAKKKSDYNGLLSKDLSLIKTEDYINLKQ

AEIKAFIDTIIKNKKYGSNKRAYQSLPRHLRRRTGSHNIRRVPKRLRKRTLKEMMKSDQK

VMDGNTSLMKHNKSLTNKQYYKQKMAVKLLKLLTRSKFMRRSIPSKIGKFKIGSRFNLRQ

RIKIMENSIKEMKQQMGKEDSTDVLNNRLGHIDLVGNNKAVEDTLRLNKSIKYYKRQQGK

DQCKWLPSHVYNIKRCHMIKYHGFKVALKPTLKCYRLINRNNGISNKADKTLIMDTSHLG

KILITLEKGEDLFELLNSKFDKKTLKFSNKGVYVDGVYYGSVNYLKWIEKKKKILLMLDS

AVYKKVIKKLLSLYTNEVKEGVLNVQDLTFAVGSIKIIGTQSLNTLLKCIRTYQENEEDE

SLLNFTEFYQLASQFKDYSNFINDNFIMGLNIKDPRLTRTPSLPQLKSEFSESDMLKMFN

KLQNLNKYSIPKESFDLFDSHKRYESYKDKLTIKDLHKVLNTGNTSSCTSKIPLLLLKDN

DHIKNSFTIMLPYHWTMPLYYKLNRMPHVFLVGLNQINQIAYDHGAQSVQRNVLTDVTSW

QYNFFEYFNALGDREHWTRKGKGRRVNFDKIKAEFRDGEKIDEMGDYFKSDWKFLYEYIV

RDKNLDIIKFIKEYEWKYSNWENDFKTRFTLFDKDEDKIVLKCNKIKLVKNGNIKKNARV

YRINKAHVDGSNPCITDLIGFVSLSNYDLSEGKSTGFIYYNLGHNIENIDEYYKDKFLIK

NTGEDVFRPAKLMN

>34-9_0071

MTLPAFDESRRESHASNVSESSTKPNKVSSTEPYQYDDFTIYENSHMNFNVFSSSKFKTK

IIVGFIIVVTTILLSISQLSNRHKRPQPYDVYSAANINLSGVNLLRYFEDNIHSTYDEVS

YYDDFRGYIDDLSVKSGISQTEILTRDVSVLQYVHSHNDYWRKLPFYDAILHGVNSIEAD

IWFLEEEKDNLLAVGHNKDYLKPKQRNLNTLYLDPIKKILDQNNNGKDGNKLNGIFFNSP

EVPTILYIDFKNDKTQNLQAYDELIKELKPLKKYLTTQLDFDKNEYKPLIIELTGNFPEL

SNYEDFIFLDSSMIKVFKKEVQFKSPVVSESLDNLIGFCTADDSMSLVKIKSLDSDDMKI

CLKTLINQLHDQNYKVRIWGVPQFPVYNRDILWKQQLNDFQVDFLNVDDLDSVTTF

>34-9_0072

MGKTNKMNVSQFLQYINKTVFKSLEEDLPSEYPILAKYKQIRFMRKLMSSTVDVKGCMNT

PLVINYKYKIIPSIKDLKLQVLISLMLSPQTKDEMTFDAFENMFIDSLKFDKKGVRKGIS

IEYLKQKKTDEIDELIKKVGFHKRKAQNIKDLSELTEIPEKYEEVLDIKGVGPKIGLLYM

QNGVGENMGIGVDTHVNRFASKFEWIKNDKGRKPLKNAEHTRELLEASIEKKFWAEINLI

LVGFGQNICTSTQRPKCDVCLVSKCKDRRVEIDETLKNGIVKDIEDLDISYLSKENADKN

YRKILAWKVLVQERYGIEMRFGDEDLLGDWDQKFDQLSKELYGYGLLEEKDANEPKFKKV

KIEVKQEII

>34-9_0073

MSGSITPEVGAPVASEHLDSPISTPSNENSIAEIKNDELDQALDKEHRYDDAELKVLQDS

LNSKGFSQFTGVVFLCLLISFGGFIFGFDTGTIGGFFNMGNFRKRFGSYHKGRKEFYFTN

ARTGLMVSIFNIGCAIGGLALGGLSDRFGRKMGLTIVTCIYMVGILIQICCQPHTHVWVQ

YFIGRLIAGLGFGAIAVICPTFLAETAPANLRSICVTFYQLMITLGIFMGYCCNYGTKQY

SDNSTAQWRVAVGLCFAWALLMIAGITFVPESARYLIQKDRLEEGKKSIAHLNKASEDDA

IVIAEFDAILAAVEAERLAGEASIKELFSTKTKVLQRLIMGDSFQTSIVIGVVNFFSTLV

SLFFVNRFGRRQLLLAGAVLMVCCYVVFASVGVKKLYIGGYSADAKTSKGAGNCMIVFTC

FYIFCFANTWAPLAYVICAESYPLRVKNKCMAIAVGSNWLWGFLISFFTPFITSAIHFSY

GYVFMGCMIFAFFYVFFTIPETKGLTLEEVNEMWLDGVLPWKSASWVPASKRDESYDSEA

LKHDEKKGFKKFF

>34-9_0074

MSDNSEVESFNAKHVEASGGTKDPYTGVPDTNSVQSLARTFTHMSLASSSNDNEIYGVAV

DGPEGVESYNAKIDPNSEEFDAHAWMRNLNRLRSADPDYYKNISLGMAYKNLSAFGDSSD

VVYQPTVLNVFQKSIEDIYRKVRKTRPSDKFTILKPMDGILKPGTLNVVLGKPGSGCSTL

LKTLSSSTFGFEVTKDSVISYDGITPKEIENNYRGDVVYQGEVDIHFPHLTVFETLNNVA

LLTTPKNRIKGLSREEFSKHMAEATMAMYGLSHTKNTKVGNELVRGVSGGERKRVSICEI

SLVNGKIVCYDNSTRGLDSASTLSFIKSLKTQSKASDTTSVVAIYQCSQDAYDLFDNVIV

LDEGYQLYNGPGSKAKDYFIKMGYVCPERQTTADFLTAVTSPTERIKNTEMMEKGIKIPE

TSLEMYEYWKASEECQELKSEIDYYLSHIDSSLKDQFHQAHTASQAKRARPSSSYLLTFQ

LQVKYLLQRNFTRIKNDIGLSMFQVLGNSFMALIIASMFYKVMYYTNTSTFFYRGGTLFY

AVLFNSFSSLLEIMTLYEARPIIEKQKNLAMYHPSAEAVSSILSEMPAKIITAIAFNMFY

YWMTNLKRDAGAFFFYLLMNFVCLLAMSHIFRFIGSATKSFPGAMVPASVILLGISMYAG

FAIPKTSMLGWSKWIYWINPIQYGFESLMINEFHGVEYPCSSYIPSGTGYSDFDSAYKTC

SVVGAVPGSDIVSGDLFLKLSYGYEHSHKWRGFGVVLAYAIFFFGVYLTFTEYNESAKQK

GEIIVFPQAVVRKIKKMSKSTHDLESASASDESYTDKKLVSDDYDESQDSYNDVGLSESE

ATLHWRDLCYDVQIKGETRRILNNVDGWVAKNSITALMGSSGAGKTTLMDCLASRVTMGV

ITGDILVNGRLRDEGFPRSIGYCQQQDLHLATATVRESLKFSAYLRQPASVSKEEKDAYV

ESVIKILDMQRYADAVVGVMGEGLNVEQRKRLTIGVELAAKPKLLMFLDEPTSGLDSQTA

WSICQLMRKLANHGQAILCTIHQPSAMLIDQFDRLLFLQKGGKTAYFGDLGEGCKTMIDY

FESKGAPKCPPNANPAEWMLDIVQARDYHEAWKSSDEYKQVHATLDEMERELPNIVISNA

DDTSAFAASFPVQLFYVYKRVVQQYWRTPIYLWAKFFVTGASELFIGFTFFKANHTLQGL

KNQMLAIFMFVVIFNPFLQQYLPIFTQQRDLYEARERPSRTFSWYAFLIGQMLAEILPNI

LCAILGFFCFYYPIGFASNASYSGQLVEKSGLFFFYSLIFFTWIGSTALMVAAPFEDPQA

GGHLANLMFTMALSFNGVFVGPGKLPGFWKFMYRVSPLTYFVDGALSIGLSGNKVECSQY

EYTDVQLPAGMTCGEYLSPYIEKVGTGYILDKSATSECKMCQISTTNAFLTTVSSKYSRR

WRNLGIFIAYIAFNYVMAMFLYWWARVPKKANRVSDEKDSSKDKKDEKK

>34-9_0075

MLITYGDSHYYHFPKGGVEYNEVRYKKCESTQNYEGVPNMKYCFMERVKGRITKDLGKYY

YYNDSVITDKLHQWVNKKQGKFPKSIDYYYEMTVEKVCDEWPDGNLRPRKWFDLQECLNE

LVSNGRYAHFKAALRLSIIVYNKLGKVLLSTGECDIELNKVKIEHEPYVWRSSKLPEGNW

ISFEQ

>34-9_0076

YKGINVDLNYYLKVKVNLLNKAPITKYKKIWVNLYDDVTNLKPKPIKLDIGIENCLHIEF

EFGKNFYTLNDLILGRIYFLLNRLKIKKMELCLITRETTNNSTKSKVCETVPISYEIMDG

SPVKGETIPIRLFLSGYNLLPSIDLGSFSVKSYLSLVIYDEDGRRYFKQAEIDMYRTRE

>34-9_0077

MALFNLKDIINIVHVINTCSSLIIGIVSIFIIKKTNDKQISLMIISISSLLITILNLFTE

LNTSSLLRLDNISSSYYKRNFKNNSLICLIILNFLNFSSSIVFIDKLISKDNKIIEESSQ

SIKHLVVIDLAFIILQQLTFFSILYSFNSDNQRYDMQDPESLIMKPKRIPTKNKRVLANK

SSEQTLTCDYDFMDAVNSESEEHGKQKIHNVNTNARWKRVFSTVLKKKSGHSNISVKDEE

MNISFANKNLETYNNSSNINVNSQNDLHHTLSVMLDIDNSKDFLSKTNINESFERRIAAE

HNAMTKMNTSLLPPRLSSKKSMTLLKGKIVNNNPHRGYNSGSRINSENSTSSNEQDLSQI

PNKFHNNLQLLIENDNIHLDQTLFGKEDVAGHLDRQYTIDGVVNDINIAENCENKSSARN

IFTDEHSNEKKDVIDILDDFLTINKHKKNQLSLDTKSIAMVDDDMMNSAFSDDFNYSPTK

SMNSLMIPKHKKTTSLSSLSPKRQQSTNTYKHKKNSQSMFINGNRFAKKSISHKLSLSNI

SFDIDDNGKIDFTTPYKNINNEEFNSIKTVSTTNKSNIILNNELIEDQEEYRKISDKSFD

YPKVLVSEYDKEKWKNISDYT

>34-9_0078

MDSSTIELDSLLNIVRTKDNGITSQKELNHIQNKALNEVTSNLRNRDYQTYLGYLIQFLS

MESETEWNLNNQFHIDVIEFVETILKVMDTSMYSKLREDLRINDIIDEKEFDLFTIPTLS

IFMKTSTKDNELDNLNILKYYCSLKENYNNDSDYSKNVYLKKVSLLENYMNSLKANNKDE

ILKLVPFIEEKFISIAIDENSSSEKEEEYYDFIGKKCYLSSLALSVITKDYYKKFDLKLL

FLDYDLVTLIKDYKIIEFNLVIQFYTQFFITTSKRKSLYKCVKETGMNKKIADLLDILTN

ADDFSLIESYSKSYIFQIISKVSILDNKLLFDLFQKNQIDLSSKSEYKYLLDFYSFLDPG

FILKYYKKDIKLLFKVDYFVERVYSYVSVMRNICSNEELWSFVIEPIISICRRTLPLLEI

LVLLDKFSQYEHCLLTLLRQETWMFQYLYGELLDEELVVLKENILNNLIIKHGEFVRETN

PEMHKKLKEYVFGNDREAKVSVATMTK

>34-9_0079

MISLGQGFFSYSPPQFLLDATSNALNSDPLNNQYAPTRGDLRLIESIKNYYNKIYHNTLE

NKHITITTGANEAILSILIGLLNPHDEVIVLEPFFDQYISNIQIPQGKIKYVTIHPPTDL

SQGKHSGNDWKINYEELESAINEKTKILILNTPHNPIGKIFTEEELVKIGDICYKHNIKI

ISDEVYEQLYFDDKIGFPRIATLDPKYAEITLTVGSFGKAFCATGHRVGYIISKNAELLT

YPAAAHTRICFSTPHPPQIACAESLTLASENGYFEDMRVNYNDKFQKLMKVFDYLNISYT

IPQGTYFIVADFSKVNVPFETYDFPLELQNKPKDFKLCYWLAVELGIVTIPTTEFFTEEY

VQKNSSVEYLLRFAVCKTDDYIDKACERLQELKPYMK

>34-9_0080

MIRLNTFIFKLKPRCYGTDMFKKFASGSVQKQPAPRKLPLKDQLEFEGLIKKANAEIIFE

EYNEKLNTSEPLQNTKELEQIITERQEQIKNFKGLEEEKSSTAKTYSTNTIPEFEGNVNP

KTGEVNGPKQDPLKYGDWAYNGRVTDF

>34-9_0081

KPEDAKPSIFGQTATPGSKPSIFGQSNETNDKTNNSILSADSAKTENKTLLSSPKKTAPR

GLSGASPTKTNISESPLISKLEKPKEPKKPELTEKFLETLKTLSIKEELTEKLVVKDSKK

DTSLIKDFKPVNFDINRSLFANNIVEEAVQSILSKDHNQEFLQYELNNFLFDHAGKKVMC

KNFIHSNGNTVYYNENNEVLLFEKGNIKKININKNLRLENFENWQISYNGEDLLLFYYKG

AEQSKFHILDIKIDNISTLKTKFTNGIKKLSWHPLANNSVVTFMTNDNELYQLNVWTGQL

LHISKQILGFYDEQGTKSLRSVFDTLQSENDFDFSNITNFELSDDGWKLYLIDSIENNVY

AIYPFVPLSNKMFTKKDNLEEMREECYWLQTEYQIDHHVDEIVQKDIEEFLFDEKWINSE

QNKNGDEMMSCEVAEDWYLNMGVQGPMTFSNFPVNLYEHSLVDMKCISNTHNLIKQILVL

LYSNGQTVVVFNTNDPVIFRNNWKTLVLDLPELQHFEFFENRLKYEIKEFLPSHINEGCL

AVTKNNEVVLLNFLDIFCRFESVYFHDHKEDIFENPCETSLLLTLNKNLSEVLFLNDKDV

LYFDESKNKLSTLTYPSFKYVQGREYSTKSNKNEFLSNLLSDVIHEDASKDEVKPLSNVS

TSCLNTYLSLLSQFQEEINKLRSIDTVSNLPLLNLSEDKPEELLSSMEKVGYSMSKALSL

SYSLQTWMRICLAEFKNTLDDNLHNRLVDNNTIEELVNNIKNISDDYEGTLKEWEKRLAR

LQSIKEKVNNVEKLSLENERIDASVLKKYKTTIDSKVLDTLKMMEKMRDFEKDIKFLFMK

LLDNKIE

>34-9_0082

MLSWNNSGQKYFFYDLISNHLKKNEKLCWWLILATLILTVVTFDKKNYIGVLSASYLSWL

IFIYKLNWEKKNSSLDLSWMKYYDLFEENDDLIHTAKKIFSILATLLVLGKLLKNKSEKS

CSQIKYVTYFLIIQSSIINIPMFSIFIITEYLLDASKYSEKTKIVIEMMLEHLTFFFFGN

TNSIATIDLINAYNGVSKNYKIEIVGFLMLCSTFAPSIYFSLHQSKRDYNLTLKYSLVLN

SIWSAFFLLSCFIGRYHLFVWSVFSPKLCYYLAWNVFMHLIVKVALPLIIF

>34-9_0083

MLKVILSSTLSSIVQKRSEECKGSSQECEKPASSSYTKAIILGVLIPVAVIAIIFGGIFI

HLKKKSKREQVEMQEDPDFDGNLDFYEYDTPQIANTFNQVNNDNFLLSKENDLDYEKEAE

YEANNNRYNSSSRRQAEHSNPFD

>34-9_0084

MNYTCNACWIQFTSTKDQRGHMQTEWHRYNLKRKVAELPAIDEKTFNEKVSKINEQRKEV

DNFGINTSEKDTKLSPKDLKKLQKQELLKKKQDLLELAKKQIELKLKRIQDDGSLTLSPE

VEKSLSEKIQQLSSDIEKIQFKNKPKEPLSEEEIYKKKLANQKEFKETDCLFCPLGSNKK

ITTYEENVEHMNLTHGFSFPEEDYLSDPKGLFKYLSEKIGLGNMCLSCNFEGKSTESCQQ

HMIMKQHCKIPYFTEDETFEISEFYDFTEYNKTRQFNKKYGSESVIDEDDDEWDDVEDDD

TNEEPENQLVVTRRIDKNGNYIYEDDEGIDYTDAIFNSGEKLYLPNGLVLGNKKDLQVYA

KHTKKERTVTEGQGTVLAAESRHFITLVDTKELAEKKRTWQKQTSDAKRDEKRYAKNANN

QMFYRDQLLQ

>34-9_0085

MHTYKKLLVSDEFQEVPIPPISEEELKLLKAEYEASKNNTNASSLKPYGNAYQYKMADQT

IIVTGGSQGLGLELAKYHHNNDENAVIIILSRSEAKLINACKKICNNTEFSKLEEFDTNK

TRLYYHPIDLSNAGKVENLLPLLAKLPTIQKTYLTAGGASVKLFRDYSISELQQGINMNY

SSSLFLSHVLSKNELCKHLIYFSSEVSFFPFIGYSSYTPSKTSLKSLVNILRQEFPDCRL

TNVFPGNFASEGYAEENLTKPKITKDIEGSSKPISVEACREIIINDINKGYDDITTDFIG

WVLMACDMGLNKNWNYSKFWFLQLILGVFANLIIIPIYMVILKYDINKYLRKAQVSDKTK

KQ

>34-9_0086

MFFGRGPFNLKATELKNPKQIIIAKSLASEILDHNMIQTTTAKAKFINSHFYKFVDKVLK

YEESSGNQELQNKYKTEAKDLLSGRLNDSTKRDELFSKALLMANKWKNRVGGKHIKKIEF

LKLSQLEPAHHDAHPVTFIELRDLPLFVEDTDKPTYGNLTLWLALESLFNEQQYSKHSKG

QLFATIRDYLKNSTTEEKEHFFDVELKKARKVIQTKKFNEQQSLIGNEDVVEDSLSKEES

KAPEFKYVESEDKELYSNIKKEYQEYDRAIIQREKKHMNHSIEHLGEVNMEARVHGVKVM

KNCEY

>34-9_0087

MNDSGLNTTTSMKIDTNQTSILPSNEILDKKRKTFQLSRNNTPSPIEMKKMKFGKLVNTE

EDITLQKFVDLNTLPIIKFADAYELYLKNLNLELPECDKVFPWFHEYENNDREVNIDDLL

DFKQNHTLILKSFYLLSDQSLSESYIENSGVLKGSINLKILFHQLDNDNSFEFLKKTLTD

EINFYNRDMFNLGLPNNFIQGDIITDCLFNICKINNILPILKTRDINTSTLKFHSPIIKH

IDPSSYRKFDIQSCKMFLLSSRVLVYCNHNDGIDCDICRGANLLVKLVYHNTISKLRIFG

DLTDIQTFKNTYLNVSIIDEFLNGNDHDFIGIPLLSTNALNKPEHQLVSKFDSYIINNWE

KDFLYRERLEMSRISSNTPFNQNLWIGNSIDFEISHMGLVLSKPVDEVNTFHRDNTICFL

DKLDPLEVNDIHVFNYPQCFWDIFVHCKPFFSNTEEMFERKAIIESKLEENQKIYFNFPA

SGTLGLGNLTLHLIRNIFQCIDYIHQMTTVLNRKALIFAQDGYSESSFLILAYTIYSKQL

ILKDAILYVHKELKRPFFVFPNDLEVLSYLQILLLSKAKERTLYEQKKTAKIEERFEITN

EDFTNMFFNTSIVPNYKSDLDCMMNLKGPLPSQILDYLYLGSLKHAESFNLMKDMNINCI

ISVGEKLSILNGKRYDFVTVDENTNRKIVIYKCQGITIYHIDQLLDNGHDPLLGSLFDIL

QFIEDFRKNNSHPKILVHCMVGVSRSATVVIAEVMKELKVSLVRAYLYVRVRRLNIIIQP

TLLFMYELLKFEEKLQEMCDEPIKGIPKGCKRSIDWASLCCSIDELNEYYLNPSN

>34-9_0088

MDSKHTQFSHVDLEEILDLSYLEPPNGLLYQCITTPTRFLLYRDNNFVDAHYFEDESIYS

QEIKTHVYSSQNSIVFIKNGTFETWKVKEDKLILSNTHKFDDINHLEYVFQDIIDADNRN

IKSACLIGYLNDTLRIWQVCFDNENDESYIVFQQSLWIEGYIDDLCMFPVDSTSLDFSAC

AIKTEDTIKIITISMPYISNAQRYLEDLDIIKKVSDMKLQYKVDGGDPLTFNKLDNFGNN

KLKNKKILILNYREVIMGSYYLNLNLLYSGELPRHIDDLKITNSFSKYRKTYSLSNDLQT

SFIKMIDNKDELKNTECCLVQHANSMISIMFYNSNLINLKDPSFVNLTYVKDIKSLKFFK

DLIFGKFNLLLYVGNKVRSGTIDDINNVIKKGNKLNSSLSLKYNEDDKKKNMKVKTYANI

NELALLIKILPFECSNLYVFEKKLKVESDYKKMKILNDKDTKHSDEYLFYVNNNFNNNSI

LTNILSGNKYDLQHKGFKFIESLQGFLYLEMIATDHKVLMNLKLLVYADNKWISKELVSK

FHKKFPIRFHDLHKTSVKFMPNIYDSNEVTGLLTYVDKEDIIECYYFSIYEKKEVEVSYK

FDIDNGTSNHFEKEEFNIETHKEKGMIQSKIQAKCHIQEETSPKSNVIILEPKFFNADID

IFWIVSDLNLQVVVINKVDNQELKFWSYQKHQDVNKKHWLDSSNWCVSSDGSKCIFSSNN

ELKVFDILEEYFIDFNIDNKLLVDKLETNSIDSQIIKMIDLDFKTSLIFQFFVDNEELKV

RVQSLDMIVLQSTLTPISHNSTINKDNDKIFSIVIVNDENEFNRFKLLVGYKNLVNKVGR

IREYDLTLLL

>34-9_0089

MSLHYLHTDNFGGMKVHENYEADLYTTNDFEFFDEVEEQNDFNSINPLIVNPVEEQEPKF

KVNRILNEPLNSKKFTDYVNHYQLLDGENKEELQNFYDLKDLDLSFMYNESFNKIINIAN

GNQSVIEENEFELDKELVDIIGLDNMVLNSISKKYRVDHTLLSRKQKYELKKYRNRKASK

KFRYKKKNKKNELLKHLTNLNSICDNLETRVDDLLDENKKLKNVFKSKISNSK

>34-9_0090

MASQSYLYNKNVFNENRINMHMDDENISSDKDESESMNVLVIDNKVKHKYEDNDINFYAN

DIDPNDSNNIPADDSDIESSRVMMSHNSQLNTENISSMSNKNELKSVIYNSRDSNLENSK

NPSSLNDSMVQTVAKSPRKNGLEYLGNNNVLPMLDINAVKLEKQIDDKDVPQFQMYMNRK

NFELEKENKIIQSRMQYKTATDDLGMDESSFENNMNPDNNSSVNGFSDMNKMDFLEPVHE

NVFQVVSDVSSNSSDPDASSIDNKARLKGMSAFGDKRSVDDSVISAGPVQEANMVFERIV

NVDNMSPSFGNNENSKPDTTFDREMEEKDDVLGPFELNKINDFGKNRVQAQSVHITGKSG

YSNATTAVDSANNNQANIFDEKNKQNLKHAHNWKHKKDLSTTTPEDIGMEYYEPKGAWGW

RRTTKNSNENKNGSKLFSSDDINVAYNKDTKQFVNDTLQNIPNNTTSYLNNNSDDLLNTE

NIVNGNYAAFRNSRILTKTNNLTHSLSNVDEDAYVSDITHPLNIVGIPAANSYDEETHNL

EDESENSYDNNESSLSNHPQDISKISSKSFDKLTSLLDVNPFELNLSNTDMIDVLENLKI

VQDLNPYILKLDLSINEASYLQNVDSDISKLVHLKELLMYHMHLKDINFLKNGTFLHLEH

LNLSHNDLETLILPQNLHPLISLKSLNLSHNMLKGHVRLDLSKFFPSLEYLNLNGNSDLE

SIEFIVPDEDVMIIKKLSLIGLKSLKKIEFFCKNTKKKTDFVIDEIDITQVNVSLLKSLN

STSTMNVKIKHMKISFSTIDGENNFASMTFPNIDTIFVKNSEWSTDIFGCLNQSLSRLQI

SNVLFDMGSFDNSSIRNNLTNLESITITDTNLKPPMNNFLHFIKTNLNAENLKEFNIKEN

FNIEQAYFSEPKKIKLFRIGTGMIFPRLCKMDQYDLIEIWKDRVIKNPNN

>34-9_0091

MAIKNKISKNSRAAKRAGVSTRNELKELQNVGRAEKTDFSRVHIKQAILEKNSNLLQSKI

DRKRESKIKNNITKRKEKIIAQYKLSQNSKLENLKIDNKALLTNIEKSLNINNQLHNKIL

KSKQRARYVQSARKAGWEQTNALAKQEIDQVNNIYIKEDPTKLLKATEPEEDNDEVETFA

QQEERLRNERNKPKNLFELLDVEIEE

>34-9_0092

MQALNDQPSDDEIVNIENASYLPDDIKYKFPTKKSYASLHSIQSPSTTYLHMEEEHRKLT

EAQLTQNLTNDTENRAKLIPNFNDGNILSSESPTNKQDDTEHEFMNEPSSIDLSQQNFMI

TDFKIGKGHDRKFSSNEMKGFTTIRNSLLRPKNTDIDNQRPKISSHSKPSIKRSSYLNHG

SHTLDRSLDSPRSIEEYYRSTPRQTRQKSRSINNVNNNKFSANESIRSNHTTSFGSPESL

KKTRENFEIRTLKNDIKQLHMENQLLKEKLSQASTHNSIFTESSNADNANKDIIEKLQNQ

LLEAEEMLSKNDAENASLVDRIYECEDYIESLEKKVDDYSTYTEDIIAVLNHEVASIKNR

DQFQTLNERYKIKEYNEEKIRENFINIYNFITTLLTLFVDGNREKQFIQDELLHDMHDSD

APSLNDNKALLESNQEMRDMIFNLKNSIENLSLKNSENEKSLTNTTNITENKSSMLSDNV

PKEKLDQLDARVSHIYNELIKTNNENLKNLKLELNEKSKEVSKLMDIKEKLHKTLHENDQ

YIAIQNEKIMKLKDHAEFFSIKHYKVVTGLYDSCEYFLKELFLEIQTGDHDSLKKPIRKL

EKVKYLIQRDFESNINTIHGNLFALIKFIEESVQVLISKFNDDILIKNKAINYKIQKGLE

KKWTNESNFSHEDRGTSRSINAKKL

>34-9_0093

MTSVGTGYDLSNSVFSPDGRNFQVEYAGKAVENGSTTVGLKFKDGVVFAVEKTITNKLLV

PGRNRKIQTIDRHVGCCYSGLIPDGKHLVNRGREEASSFKKTYGKNIPLYALSERLSQYV

QAYTLYNSVRPFGIAAIFGGVLKSENGGDNKPQLYMLEPNGSCFGYKGIASGKGRQTARS

EIEKLISADDFDIETFSAKDAVKEAARIIYLAHEDNKDKDFELELSWCSLSETDGLHKFV

PKEIYDEAVQYAKEETGLGEDEDSEDNDEDMDE

>34-9_0094

MFNISRFVPRTPFFSKHIITTKRYAAVSILGYDLYKQWYLSNHVANEANVLSRNTLIART

WKALSISEKEGWVLKAKMMTENMIEQKKQVYESKQFSIFSRMFCFYNIPGIIQEIKASNP

INSYDSLFEGKLKILEFLLELVSSEGNRVEKVKIYVQLLNSLMNPDFADLESPEMQKLQS

LFPESSTSDIYSIWVELLSDVQDISFKNFVKFTTEHENSKILILFIKYFQRLTDKVPNAP

NFIKDLGKITPYINSSSSRFELFFSDQFSVIDNPYEFMINFKSILKETHIRYNELTDEEL

KTLDEKSLQLERYPVVQQGLPIYKKNLPYPLYVKESFYKRYFDAILHSGEWVSSPNTTKA

FKNYNFEESEVIEAEEIESINAKGDDESKEKYLKKPLKAFIDLNKEWHDMSNNKKTQEYW

QKMIQRFDDGVKKYTSPHEAEMRKLMGAVKVFDNPLRFKGYNPKLLYAELNFSQFNKYVE

ISSRRNKSFSFSKNRTSSVSAQKKTIIKSLDSSDPTNDDLNNGSVFYNFYERLFETQYDC

FELEDVFKAWDNLSNEEKCRYYILCSVLRFQRGCNICFKIPVVFGKKLGTSDFTYVLNKH

MDASYLKEIYPYNETKDLSLQPELWPQVYAKRARALKRFADESLNIYKYLENVNEKTKTK

FRKVIIHKRSVAKSMATLFESRVPDNSGKTLTYEDFFLSRKHEILKEWGFDNIQEYISSF

ENSIDLFHDSIKLIHSQIDNYEAHLGELIIKNGFSSKLTGDIQSTIKFKDFNSPPKIYFK

LFSFANRRKKTLLQACIDENIILGKPLTEKDILGDKELPDYVYQTSSSQKLYDYLCSIAL

LSSIKATNNCIVSTPHEKNPAKFHIIEKMGPTFFFKNVLKKFPYETVSSTLLRAEHNAYM

HAYRDGSLQKAADAGKKSSSPSKLDCSFLVEFRERMFEEQVSIYKKMKGNDPSSNIKFTE

VVVPKEYVLALLDKLEYKYNQPKQNKNDKCHIKKEVDQMTIA

>34-9_0095

MSTRKRLNIPNTIEFLKNRLYLGAFDFDPVNTDEATFFTMDDELFYNNFHLDFGPFHVGH

LYRFALRLHEELTVSTNAEKAVVFYSSTSTKKRANAACLLCCYMILIQSWTPHQVLQPLA

QVDPPFMPFRDAGYSVADFEISIQDVVYGCWKAKEHELLDLHKIDLDRYEYYEKVDNGDL

NILTPNFIAFASPRDDDPRGLYALGEDSNLKINKTFRKVIDIFKKTNTKLVVRLNSHLYN

KAHFEKNGIQHLDMIFEDGTCPDESFVLNFIGCVETILKENEDLGDEKPKIAVHCKAGLG

RTGCLIGAYLIYKYDFTANEVIGFLRLIRPGMLVGPQQHWLYINQHAFRDLRYNKKISLE

PSDIINGMYPLVDLEEYKIQKKKKLSKERGNQAYYGDNYGNLTENSDYSLVGNNEDVPKS

PETNNRNSNNLTHGVPLRSPGQPRKNITGVNTVEKYDAKQAQRAREQEQKILQGSPQRSP

KPRNLSNSNNAKENRNITGEEMKMIYNAKQQQSQRRNISSNNVSGASMATNTLSNNNSSN

GYPSSVSNYRNVSSGSVRKVSGNTYRNK

>34-9_0096

MPPSFENNKRDSDLENTPKEEDILMDDFVDANSDINEEPVEESNIEEDAQIAELIAPYIA

NLSMKNPMLYDQLIHTIDQNRILQNVNPDEESEFEKIQQMKRDMREDLEADWNAVDPVPF

LGEDLENNDELDTINERRSSRHSMHSDDSSRSKKKTSLNSVLNKFGLGIVDEDDESGKSD

NQSQLNEKHTLPDNASHYTSAMKSYASAASKPESISGKITSVLKKTIGLEEGYDPTAIYD

NESTEDINDFVLDDMDAQELPETRLSGEQIMSSKIDNTFNKDAQSTTPRTPGLMNKFMFK

YKNQKTETGDNQTTGSYRMPRFQSNRPREAIKYIPKKIMPEKGIRLKRGAGGSAAITVHL

ATLLNKQRFIIRLCKALMLYGAPTHRLEDYMKTTSSVLEILGQFLYLPGCMIISFDSVST

ASDGGELHLIKCNQGVNLWKLHKVYKIYKLVIHDQIALEDASTEIDAILVEPNLYNKYWN

VFFYAFASSMVTPFAFGGDWINIAISFCIGMGVGVLQNLIAPKSSLYSNVFEISASIVVS

FCARALGSIPNSNICFGALVQGSLALILPGYIILCGSLELQNKNLLAGSVRMIYAIIYSL

FLSFGITLGAALFAWIYNNATNETTCVKNVPDLYKLLWVPVFSILLALINQAHWTQLFVM

TVISCLGYLTTYYSGKHFSNSTEFCSALAAFVIGILGNLYSRVYSGLAVSAMLPAIFVQV

PSGIASKSSLLTGVSVANQIVNGTKTQVEETGTNSLSFGLQMIQVAIGVTVGLFSSTIFV

YPFGKQKTGLFTL

>34-9_0097

MSSLRDQLSQIATQTQGIALDRKRRQKIHSASLIYIPKVAAAQDFEFIYDQIYDHYLELC

SIDSRFDAFKNSLFAESSITLDRNTLNDDENKDLNTNINMFLLMVSSKWHLNSTLYAVEW

LVRRFQIQIHNVEFFLFCSIHHYQTAVFSKILSISKLPNNYSVTLSPFQKPDSMPPNSSS

LLKLFHDFEIRSNYFKFMSRLIKHKLGSQSVYTFISVLAINLVATYPNILSDLIPSVLEI

SAKLLACKDSSFKEAQTAAHTMLLVLSKTDLNETIIFAAVETILTNIRDNSNTQKTGFIT

ICSLLQSLNGEVKQLSPRIFKLLNEKYGKTLVYNIGIQELSVNKFVVSYIRSAIRYDYQD

GLSAVIEVLKSYSFLSKYEKKLIISDLIKLSEIVQDKSILAPIFTYLVKADELLVIESLI

ALNISPEIFEIRLTTSLFKESEEMDSNNAEKVLKEISDSKIVGSMTTIEPYKEWESKHSQ

HVFNENISVLSIDTARFNKLLSFFVEALGRKYSTKQFLEFFFTNFESRITFLIRVLASSA

PSALKLVIVENLQRITGSISKTMNLFSLVPTLICALNDKSKSVREAIIRYLRSQVLKRCT

NDTFFMIDSIYGKDVEISLFEPKQCDKWLKNFLDNYNVDIDSFYDFCINKKSESIDMIFW

ADQANKSKLYSCKNTFINIITSSKFYNSNYSELLSSNLKSFIDNYDDIKAQCIEEKLNFS

SFETTMCSIVCKGEKNESNINFITSSLNSQYKSLSEVVILRLYKIFITLKFNTKINILNR

IIDATAKTPELLNYDSLEVLQTLNIDCDVYSAILNSSNIIMEESEEAQQQHLLKKRRRRS

SASVKSVLQKDEVTQLAEYHLKKITIIIESIDGSRNLKNVNEQFLAAMFNHLNDLETLDN

DGGLPILYVQEILCNCLLKSINILKEQQQNQKSKTKLHTLRPDILVAAIRSSPSPQVQNK

LLMVISALASLSPEIILHSIMPIFTFMGTHSIRQDDEFTNNVVENTIKAVIPALLQNSGN

IDSELEVLLSSFVAAFNHVPKHRRVKLFTTLISTLGTANSMHSFLFLIGEQFYNANTSFK

IGETKEIITFTKSFINNFAAEEQLSAIHDFLNLINVLPLIINNTAESGELFNKAKKVMLQ

RSLFSSNILRMNETELINFRKSLLVFLSGIIAESDDTYYTAGSLKLRIMSSILEGKSEIK

QLVSTTTKTILQMINDIDSHITSFKNSKSTRVNKKLLKTKRQSGSSTSSSKSGSEDNEYT

KQEILTDIKGLQFEILSDVLALLTIQDYTDAVIPLMCNEQDDQIRYHMTLKISKRFSYEP

VGETVDIMKQVVDVLFANLMDSSSSNEIIYVSLNALSYAINKYKETLADELPKALEISVN

KLNDEFNENVVISSLSVINICIQLLGIKSIQYYSKTVPKALALSNDLQNDKLHLKNKLVK

EELQLSILLLFATVLKKIPMFASSNLQDILLSIMYAFNVKEEIRLNIINLLLENCDLKEV

LNKLHKIWQSGIFTVRNEKPNGVQSMALSIYLSTLEKTINLMDKKTCKIQSAVFFKLLTH

LFEFRSYSSFDNNTINKIESMVHVIAHAFILKINDQLFRPLFAYLVKWAFLGENVNNKEM

TRNERLVQFFKLFNKLQEQLRSILTSYFTYILDDTNQILMDMTEKYDVTNVNLRRLVIVC

FGLSFKYDKDEYWRSSSRFEMVCPTIISQLKNIEPPLGKFLAKTIGALAQCNSMADEHNK

MMQKEIIKHMKPSCRSNEKLWAIRSIKLIYAKVGESWLVLLPQLVPIVAELLEDDDETIE

SEVRTGLVKVFENVLGEPFDKYLD

>34-9_0098

MPIKANYLKRKYVQIEFDALPSYYCVIIIRNQKLVDNSKEKYQGRNEEPISYNKVSRLKL

NDFMISIGTVFGNLSGITDGYIYHNKSADLKKGNKYSLYCIEEGDGSFRIDFLDIPESPF

IENIKDTVNNKQETSNMNTKGFLESQLAFQNLDHYPLQKKNKEVQQKQKWTESNQQELSK

TLQHRNTDVFDEDYQVPVMVFKGSKVPLIRDRNNSFIGVVEVL

>34-9_0099

MNNPIISLKNKTLDDSTLDSKTYHPQVLTDPKLPPITLDKLPAKLQIISYKDNYKAIGCV

GSGSFGTVNLCKKVSSTTVDFPNSMIDSSIATLHDNFYNSQNNLVAVKIMNSRLNELSSY

SRVRELKFIFNVKAHKNLLRVFECFIDDQNYKLHIALEVMDQNLYELIQLRLKKNSGLFS

TSTLLSILSQVLYGLYHIHENGFFHRDLKPENILVSTTFKYFDKQYYDNMEHRYNYVVKI

ADYGLSRYVNSDNNHFTAYVSTRWYRSPEILLRDRSYCKPIDIWAFGCVAFECATFTPLF

SGSDEIDQVWKILCTLGTPYKTKDNFIQNYQSIGGNWQEAELLAYRLNLNIPYIQGVPIE

DKIWSLVSQSSIYQKPKNHTTLKKLVEVLKGCLTWSPSTRTTAEGLSQLSYFANSEVDSY

HKKRTNIEKLKIVELMKATSSHDSTKVSPIEMSSKSKFPSRGLKSLNFSSMNSSGLDFQE

MFEDFKPSYSNLYESSMAHPKCNANINLSIEQSSEYLYGAHNISTEPDFENSLFNIYDTE

LTTDHSAVSFQHKSTETRNVESPLKSVNYNTVIPNNSNYVLQKTSININKNSCLITNGGI

TKSTKKKVSSNGENKITKALKDGSDMQLINSAFLNKTKYIVNEKK

>34-9_0100

MPKITPNIHSRKYPFQVSPGPAVAALSDISINTNQQPNQIVKGIIAAEILETNQAIPLPL

KKVHTMEEPTIHRSLPNLKIEVNIEDYIVNSEDNIVSCICDQNNPGDEDDEEKENESEDE

KEEQPEEEDDDDDEDSSVNDVFWLQCDHCNRWVHGKCYGLKDDKDIDTMNFHCHICSPQD

HPIAIKKFKSNVAKQLRNLAKVNANNKQNLKTKQKETVRKPSVKKSTVGNAEDDKKKETK

STGTTTSSKPKGGKEDSVKSEKESENKEPEDEPVDDTIKRVRLKNIRDLYGNIYYPLDKN

QYIENYINEYLQAVCKKETDERFMLQNYPENFVNVKIDVKTPNAKSGDKFTGIPSLYVIS

KESIEEGDLIHEIIGKVGDQKNYIQDPKNQYRLLGVCKPKVLFHPKWPIYIDMRHAGNEV

RYLRKSCHPNCEVVAALKPNETQPSFYLRALKDISSNEELTLAWQWDINHPIWKLVPGDH

QLKYEKSAMKKRFETEYPDYKFDPSLPFSMDDVSEPESYFLVYCVDTILTMAECGCPNHK

KCQLRNVKKYYNNVLKLKKSMSVAQMRYKTYDLLNKQKSKEPKQTSIINDMVTKEENRYV

NRFNNYKSFVDRKRKLEEMSLELKDPKKTLYVDSTMGSGKEISTKEEGKKAVGHHLPSPL

SPQQYKLEMIAQCKLQDALEDKVESIKKKQKISLENSIPKFINWIQNSKIANSGLLLKSA

KVQALQNIILTSGKKAIDKTEIISNVIKGRDIITINEEQNKKLKQINELKHKEASLSLVN

KSSSPGEDKHNSTFQHANVGSLIESKSDTKLTKSSSLEIETNKTSENIQVQPIKKKMSFA

DYRKKSKPN

>34-9_0101

MEHTNSNSKYDIKELYVQFLIQDADLTMPIAAIESLIQILKTHNPTTSAEMINIINGSIK

ELTEGIPNSISLKAGCDIFYRFVIKHLTGNNDKSISSTSFESFKNHLFDNGQLFIDRSKT

SRFKIAEYGTQFIKDGDLILVHGHSRSVIQLLLHAKNQNDKKRFKVLISEGRPTNGGILT

LKKLREENIYCNLIPDTAINSILHKVDKVFVGAEGVTESGGCINLIGTSTIGILAKEKNI

PFYVVSESHKFVRMFPLMPNDLPMLQDPLNFEINKDNNKVDFDSQNELLLESRKDLLNIK

NKEDLLLLSDKHLTAPLLDFTKYEYITAIITDLGIKTPSAISEELMNMWYD

>34-9_0102

MRAKKVKAETANEDINEMYVETHEEEFEDTDPIIETIPMFLNPSNNENIQLWQFTNKNKK

HVASPSNNIDNNLYNVRYKKATGSWEVDFNLANANANLPFYNIPRLASKYNEEETNTSFK

QQIYEEPGNTDPQNMTFFQNMTGVEIDNQSSFTQLVGVLTDKEDGSKALELVKMTKNVQF

RPSFKYIDEVKTKSSAPIANTQQTQQQGATMVTLSAANKNDQNGNAAKLTGSLLAYKLND

EEPSIQYLYDREKPYIKDYINRDQTIKGNSGKPMSHRSYMDSLLYK

>34-9_0103

MSDDMFLLNIDTSGGASSTGVSSKTNTTNMKGNWKQRRQQVKQMRKSQPKTSEVNTEKNG

LVSMNAELFNNRNTRVDKKKNKNDKKGQNKPNPGYKGKGDSKDQTFVSSLFSSNKEITTK

VDEEPVVEEVKEEPKEEIKEEASETAEIEKEDITKNSELFDNQETSAFIDLGIKSPKLVR

ALNTKLKIETPTRIQSLGIPRIIKDDQKNIFIHSRTGSGKTLTYLLPIYETILRNSQNLG

RKSGCTGIIITPTRELAVQLEKIINTSLNIGPSVQAVVLIGGEKKQSEKKRLRKGFNILI

GTPGRLLDHFKTTESVKEYIKSSSLKYLVLDEGDRLMELGFQKDLKEVFSIIKEEFCGGN

DKSVKKILCSATTGKDGKLGEFINLKDYQMITANSSNEQELTIANSKISETLVQDCVIVP

PKLRLATLAGVINNTTIEHSKEVDSEGNVILRTMVFLSCSDSVDFHFNIFSGLSGNKYIN

RIEESIAETKTGSSVFPSLNDQNLKEFSYVFYKLHGSLHQNIRSKTLQHFGSNKAAVGKN

VGSNKCYKKHLVMFCTDVASRGLDLPHITKVIEYDPPFTVEDHIHRVGRTARLSATQKVK

GEATIFLLPGEEEGYLEELNKLHDGKIQTKGYESILKKAFTNNEIKRNDYDSKNDRKGKG

SKTSSWDSNVTTWHMNVERIILEKEDLKKSATNGFISHIRAYTTHIAREKKFFNVKFIHL

GHLCKSFGLREKPKQLAYSGNTNKKVQRKRKFGDISDNESGEEFQMDSEDEDFKRDTKLK

NMTNKNKMFELSRMALKNMASEFQY

>34-9_0104

MAVEDAKEYAIKHIIDHYPYIAANETEAERVINQVIHKTSDYELQVFFESFFGKDNEVKE

FANKFKELLNKASKLASDSLDYTKRSKSTTDNSGTSKKTVTRRSHTDKSKNGSTTSEILL

KKIEQLKQKQEKNKKTDTSNKEKGDVRVVKDTQEKNFKKTFDQDHVSGLPENLQKFENVP

MTEESIEAMSAYFSNMISEHQIRNQPLKKQTEYTVCGCQSIKHGLYNLSPNCLRCGKIIC

NLEAKQTGGEYCVFCKNPLLSEDQIKNYIELLELDKLNITKKKMSEKLQKLINDKRKYYT

DLNTNKKSKNNGSLTFNVQGRYQDKKLVNKQLDMILSQSSQRDQSIKHFMSQDKDILSLQ

SKIESLDMQIVNKSTKYQEDQQLKDAKSRLEKLLNFQNTSEERTKIIDVAGEFDFGVSGD

VKDAFEGTAEERALKMKLKQRNMKLLKEQKLQQSGKGKNEVVFEVDANGNFNIKKVERDL

TSSNKANEDVAIDEEDESLLEEINILREKIYNLKLKDFELSSKKVFDPLAPENKLKDVKY

TKNVYNIITPEEQVKNPKKSKKSNAIPKSIAKDEKFIFDTNKTSNIGLGLDSLEENIALF

I

>34-9_0105

MSSINSLFKVAFQTVSIFGKAVGQAYRQSQAQSAARKAAGISGNGVSGSFAQEFGGINVS

ESMDILNLKGPDAKDLKKINDRYNTLLKINDPEKGGSFYIQSKVYRAAERLKYDLKMEDP

EGYKKLLEELNLELNDNVAKEEAKDIKVEKK

>34-9_0106

MSLTYEQAKAEVEEIVSNNKYVFFTASWCPDCVYLKEIFNKYGILDKFYIWELSRYEKGS

DDFKLYTKVWFEEAFGQQNIPLLFVNGVYLGTEHQIRSWEKHGKIEENLKKLELI

>34-9_0107

MPYNPDMKNLSTIAPTDNNSPVNIPNNDIADLRYAMEQQFLKQLQLKNGSNQKEDNASKD

KQLDDKTSNVITNTKISEESQSISIDDSQNTDVLKKTVNPNNADSDIICSNCSTKKTPLW

RRDENGYILCNACGLFFKLHGKPRPISLKTDVIRSRNRKTKSEKLKIKNSEDKPVPKKRK

SNKKSNSPGGPHTTNPLLVSSKSNGLISAAAYLGSNPSSAGSGLIKIVPKSENDYLLQNQ

QFNGSYKISANQGIVNKVSKPNGIAMYADSSNEQQPSQSNSSNFLKPMLSNNSNASDDKG

SLGQRMVSINSMHNAGATPRLGPIWANSQQTVVSPSGFKSPAMLAIDKLASVSRQVASPL

LSTPQDKKYTLPPLNSKDSQNLSTELVSPEPLNKLPNLKNVLINDNKEGKGVASISSILS

THKMIDEDAKDKQIRILTTDNKELELKLDICKNKIMELERKIYDLENLKT

>34-9_0108

MYITNVTTGNSTKPRVQENSNNSIISRYCRNGSQFSHLPLNKKHNPNYLLYEIPEQYNSK

NSNNSTNSSKSDSTRYILKSINNNSNEGLFKRNYQISFNQDRHQSKDEYVPTISMIVSKR

NGSKASMSPGTQVQNSFSRGSTTSSEDNSTLVNSTNSILKTGYSSTKSLLDLVSENENKI

QNVVESKDSTTNLHKQLRINQKLTILKQNIDKKSNILDGIQNPETYFYSDVKFMNDKINH

QYKNMKLLKSQDFQNIQSEVKIMTKKKLHVSEKYLELVVKNKVLDKDDKKIFIEHSEDYI

PTDYLNKLAEEAWLKAEESMDFTNKQ

>34-9_0109

MKTDSSSSSIQHKDEKKHISNQATNLSENLRTVVSRRGDRRIQIVTSTNQQLALEELGYK

QELIRTYTWYEMFGVCFSIMGLMPSIGSTSYQAFSSGASSATWGWLAGSVGILTIGIALA

EIGSSFPTASAIYLATWNWSPPKYRNLISYAVGFTDTVSLSASVCSITSGCASQIYSCVQ

LYKPDFVVTNGNTYGLYAALIIFVAIFGSMSSSINAKIQNVSSITNIFVLILVFIALPAA

THRNNIPFNDAKFIFTNVERADTNWPSGWFWLINAFQPAVWTIGAFDAAIHMQEESIPKE

RIPGRPVILDPAASPASFGIISSIIACGILGWLMMIVLCACMGPSIIDVVNTAYDQPITQ

IFMYSMGQKWTVAIMSLMAWSSLLMAQSCLLSTSRQFWAMARDYGIPTKVGSEFFAKVWH

NQPINAMIGSSALSLILGLMMFGGDACSAALFSLSICGMYMAIIVPIILRLTYARKDFNP

GTFYLGPFWSPLISWCAVVFMAWMIFIVCVPLTYGNQLDKNSMNYTVVIGPGFTLLAMIH

YVLYQHKFYKGPHSNLSDEEFIELMGEQGIDNIISNHEKYESEKK

>34-9_0110

MVNSKTLLVAAGLSLSAQAAVMPAQIQLQQAFNLNDDEVIQSFETKESNDDLYYQDSWKF

HLPYFLKPTVNTETLQSKITLESLNKSANALYALAGESVEEFGHPTRVIGSKGHLATLDW

IKARLSKLSNYYDISEQEFDAIYGRVKNATVEFEDGSFLEGVIGMSLTPGVDSFYGKVVE

IPNLGCNEEDFLSVGKVKKNSIALIKRGECPFGDKSKNAKTAGFKGAIIYNNEKIPKAVS

GTLGDDVNSTVATVGIPLAQGEQLKNAIIETKNKLYVNFAVDAYVKKIPTKNVIAETKRG

DAENIVALGSHTDGVEAGPGINDDGSGTISLLTVAEQLAGYKVNNKVRFMFVAAEEEGLL

GSAYYANSLSQEENKKIRLMMDYDMMASPNYQFQVYNASNAVNPKGSEEIKNLYIDYYVS

HGHNYTLIPFDGRSDYVGFLDVGIPAGGIAAGAEALNTDNGKVLDKCYHELCDDVSNLNF

DAFLVNTQLIAHSVATYAKDLSDFPLREFEEEVEENNFQYQASHLLF

>34-9_0111

MSLPESFQNNFLPEEIQFLIENELITVIPYMDGKKSTKKPTAQNKRRLKLLQLKKREREE

RKTAVYSDDEDEDFVENKEKLARINKNVNSTYLDSPEQEEMEDEEASEFDNFDWSFITTD

HNVLRQIKSLIPIEIPLWMAMILKKQRFCRLVPPTWFSERKLKEFLMFEKKNPYKFSSLP

WNWMIITKLFLNKFQEDLDSESNIDTIRGLLQDIREIRMAKVQKGIEILNESHLQLDNLS

LLEINEFRPFIITTMNKMKNLHKSSLTEEDLREAEIEYEKSKNNPIQSNHYDNDNIPIND

YDMDDYNI

>34-9_0112

MSNSNNETIKIQVALVPPNCFPIYTQTNPMMNPYSMMLMNPYMNPHMNPQMFAFQQQQMG

GMPNFPFMNTANYDISSSSHMNPFGPNNPNINNNMSGASIDNINGTHATSNSISGSTNTN

NKSFNAQVEFHPSVLTFNVFIDPSKTILQLADAIYAKTSRLYPNLKRPLDILKLKDFEKN

DLDIEDTVDDCFLKGNVSRLTVLCCTKFPLDFNTRNNNYRVSYVQGDSDYSNIMNSDFNN

PEYDQSEEEGNFKESRVNSMTQLQLSRKRNVSLVYQNKQKVPSIIIQKKNRRQSNSLKDT

NSNNKDKSPVDRDSLLPPPVERSPQMRISSNIGETRKRIFSTNLGQAMDAVSRSEEVDPD

KRKTIDMVMIQNEANDEHIDTPSKDVSEVDVSPYKNATPNRMNSGLRMISANNTVVKSQP

MPNMLMNSVQRHSTIPKKTGEIDQVKSSKQHGKLDFGGLTDPILKSPGSNSILPPKGDRI

PMKQGLRDDINMLTSSSEDEDLDDEVHKQPLVTESHIVNNISSGKNDDLKDNVGQLTPLN

VKTNSKKIAEDSPLRNEDADNSNNTSLLKMAQLPNEKAENVQLEDKMEVDDDNEDNSVIM

NALDQDSKPLVIEGGRTKRRAALAAAGKLDNRSVRVTNNEGKEVIMPYNKVNSYMKNQKA

AKEVTPVPEVVKKNNNLFDSLSNGGVSEPINSPMSVKTKNVEEKDVDMESSSSSSSSDSS

SDSSSSDEDNENVEEDNNARKVKVDVDTTSQRVPSAKQLNSVGKITEPVLTKNPAENVQE

TENSKLSTLQRMRMHKNQKSLGSLSDLIARGIPSVMDRAAEKRLLIEKQRKDEEEKRELL

KKQKEEENSSSSDSSSSDSDSDSDSSSDSDFDSKKKSNFIKKPKKKSESAFASLIKDSKK

L

>34-9_0113

MYIKNITIQGFKTFKNQVVIEDLSPNENIIIGTNGSGKTNLFAAIQFVTSEKTFNLSTEE

RLAYIYQGAGTVMSCFVEIELDTGVKIRRTLGLKKDEFTVDGRVKTKPDYKNILISLGLN

NLGSTNDYHIVPQGRITGLTNASNEERLNILKDVTGANNFELKLEKAVKDMDETKIKRQR

VDSELKELETRLSDLSEEKRHLLELQQLEKKKKLLQYVIHENYFNNINKEITDLNEKYEE

LLDQTDVSAAEVEKNEALIEEQETSLKQLKDEYQDHEDNKTISDELISIQNELSKLENKK

NIIEKKINGLEKELNTLKATDEELKASFKENFDKRSKLEPEFEEFIKKETDLQEELKKLE

GSQKYLLVKQGRYAKFKDITERNIWINEQIKSKNDIISELNDSIDASAIELANNKTKKLY

EEMSNIQLNSMNECTNQIAELEKKLLTKQDIYYELVDKRTELWRSEQNLKGTVDKLEEEI

KSLNHKNYQTLDRSTALGLENLPIVVKELGFDNQKVFGLVGNLVHYSNKYKQSLESVAGV

SLFNVVVDTDTTASEIMAHMQKNKLGRLSFIPLNRMKNSNSIGYPPESVGAYVPLIKKLK

YESYLEPCMKLLFGKSLVVKNLETGSILAKQYGFSAVTPDGDRVDSTGAITGGFSNISQK

SVRLDTLKAIVSMKEELTKSSKELQVIESEIKSLDDNSIQSDIEIQQIIADVRLQKQLLK

KLQETHDQKNKEVFETEALVDELKEKENKINNEVKLVQIEKATLEENLRKPFGEVLSQEE

TEQLASLNDKISAIAVELNSLKTNYTEIGLELNVLHQEREFIGGQVNSNDINVKAIEEKL

INLQTKKQTVSKRLKTVKGKESNLKEEINSSSEELAKLSTQIASIEESLNELKSSLNEGQ

EELNKLQLASEKSMSKISQLNARKNALANKIAELGFISDDGLNEVKKLTENQILQNLEQL

TIQLQSFSNVNKRAGESFMRFLTIKENLEADALELKESEKSINTLIKTLQKQKKSAIDEV

FGKIQKAFSIIFKEICPKGSGELVINKSNNSEYEGVSVLVSFNSEDDEQLYVEQLSGGQK

TVCSIALILSIQMIEPSSFYLFDEIDAALDKEFRTSIARVINELTRGKIIIDENKKENID

VKQNSQFICTTFRRELLNHADKFLRVRYTNKHSLVETVSRNDAIDFVKGEKKQRIGDV

>34-9_0114

MPVTLDIARKEVAELLKNNRFVQLNASWCPDCVYTQSVFRKYDVIDKVKFFEIGKYSRGT

PDFENYYLAFQEAANKKNLPLIFVDGKFVATEHTFHDWENSKTLEQQFKNLGLL

>34-9_0115

MNNSTLINKVAPKNGEFYLCLGIEGSANKLGIGIIKQAVSDPLDLHVISNIRDTYNAPPG

EGFMPRDTNRHHKNWISRLLIKAIDHAKETFDSDFNINKIDCIAFTKGPGMGAPLNSCAI

FARTLSLRLNIPLIPVNHCVGHIEMGRAITKSQNPVVLYVSGGNTQIIAYSNNRYRIFGE

TLDIAIGNCLDRFARILKISNDPSPGYNIELLARNGKNLIELPYTVKGMDLSMSGILQYL

EQLTKAFLKNGQSFTKQKKRKLLEYLNDTDVIDNTEITIEDLCYSFQEHLFSMLIEITER

AMAHVGASEVLIVGGVGCNLRLQQMMELMCKDRNATLYATDERFCIDNGVMIAQAGLLQY

RCGYKIDSLKDTIVTQSFRTDEVFVNWRD

>34-9_0116

MYRSTSKQVIKRLQSTKPSGQVIGIDLGTTNSAVAVMEGKVAKIIENAEGARTTPSVVAF

SKDGERLVGIPAKRQAVVNPQNTLFATKRLIGRRFEDQEVQRDIKEVPYKIVKHSNGDAW

VEANGEKYSPAQVGGFVLNKMKETAEAYLSTKVKDAVVTVPAYFNDSQRQATKNAGQIVG

LNVLRVVNEPTAAALAYGLEKTDESGIVAVFDLGGGTFDISILEIANGVFEVKSTNGDTH

LGGEDFDIALMRSLFEKFKAQNGGFDLSKEKMAVQRIREAAEKAKIELSSTLSTEVNLPF

IAADATGPKHLNIKYTRSDFEKLVADLIKKTIEPCKKAIKDAGISTSEIKEVILVGGMSR

MPKVVETVKQIFGKEPSKAVNPDEAVAMGAAIQGGVLAGEVTDVLLLDVTPLSLGIETMG

GVFTRLIPRNTTLPCNKSQVFSTAANGQTSVDIRVFQGERELVKDNKLIGNFTLNGIPPA

AKGVPKIEVTFDIDADGIINVSAKDQTSNKDASITVAGSSGLTDAEIEQMINDAEKFKES

DAQRKKIIETANKAETDITKYESSIAEHREKLNADSLKEFEQLVADTRELIAKSLSDESA

TADVEVLETKLKELETKSLQLFEVLFKGEEKK

>34-9_0117

MVRRNNFKIAVIGDEGVGKTTFLHILSTNPITTPVPKRYPNIIIKTDDFPDSANCPLTTT

LIDCSNNMMTVSLLKEIDCILLIYNDLQSYNRVYEHWMLELRKMGMDKPLIVLKNEFVDS

IIEENQELVDPKKNDIEEKENCEMELIMLTTEFKEISSTLHGPLISKSAANFNLVHAVVY

HCIRAVVYALPPLYNYKTNELKKSCQIAMKRIFTLADDDMDGILNFYEMKTLQEKCFPVS

KNMSSFDELEYERLCIEIKMYKQEEIKKLDNNNHYLDMSMKKQSQKNGIDLYDFYTLIKI

YIERGKFETVWGLLRGFKYNDILQISDELLYPPLDSFIDDGSFIELSFTGYRFLIEVFRK

YEIDNDGGLNNTELNKTFDICPNGSIPELWILTNFPNSCTTDRYGNVNLSGWLAQWAMTT

FLDYKTTTKYLVYLGYQHDPRDCLKITKPKKYKKRNGLLYRASVNDRKVFNCFVIGKAKS

GKSTLLRSFANKDDSDTSNVFSYYSPTIRPNMVTNSLELDDGKQYYLILQEFGQTEEEVL

SNEKKWEQCDVVCLTYDSSDPESFSYILQAIQKYKHLLKIPCIVVALKADLDTQDQRCIT

QPNDFCDSLNLDNPLRISAFWPSMTQLFEKIVNVSLQPLKATPGFDSDLKKYSINDEFRD

NLGNTIGAIGVLSVLSIGLYKMFAKKT

>34-9_0118

MDLDNSDTPPIKRKRARISYSCIVCREKRTKCDKNSICSACKNRGTTCQYDTVTQPKPKR

PNKDALILRLSKQLEFYKDLACKYTPKEKLSLFNDDIYSIDYALGTRTLKKANIRQDFIT

DGSNTDNDEEHYILTLKRNDDNFLYDIFTDMYIFKTDDYVNTLFFKKTNTNTTLNSKKDD

ISIINNLLSNQTFKSEFQKLQSLKFTQALHIENSTKENKEFFNPYSGLFNTFRFDNVLEN

TIENVIETEPSDLLKNIIILLNRILPSESLIHHYKKLYFETLYPEYPYLDIEFFETTLLE

VLHFDNEKVQLNLGKDSIHNKICMIALLLLILYLAIFNENDNKDLDNSIIGDYIGIAMKL

LLSTDYLSNPDEHKFSLLCQVWVCLTISPESKYSEDITKKAPTASLVGVITSLAYDLKIG

NDTDLDERYHRISNDLRILRRKLSAAYTLITLSEDIFKYGLKGNKKFQNLTHNIDIKSLG

SKNELENEAYKWCILRNHQYKLIYDIMAMFLKKHPEDGNDEFIENVDIIVLNKKLKLLFE

FIDTTFPFDQMQKLSSTDDTFNFPTSSKKYSINKYLVKNRFIMHNWIFSRSVILRILHVV

LVSCEYWAFESFNKVANKWFMYYINESLHQSMDLLMLFRKIHDLEYIEYVGSAHSLFQEY

IVLYTYSKAFILLLQFIGKCFVFKVYLQMMVMNEKDDSTKLKVEMHLVEEMKDVSSKVIF

KALQEYSYLYRFKRYKCFKVCLFLDFFKQIFTDDTLFNTMFETDKKYNPDAPLDPEVCNS

TLMRHYLKFYDYLDDFKSMTEYFRSEEIQSYLQKKPFKQKLNNNSLNFNINGSSMWSGLQ

EIEKQPYMNYPRIHEYPGHFYAQQNISPTNTPSKLGEPIYPNAMNDITHNVKFSNRNINN

ISKLVPNGRYVSNFDPNYYKPYQRNGVLGQNNMAPNGTLQFNNPKPTQVNAMNNPQINHM

HNLNNNLGNPQMGPSTFMNHLHVNGRSSNPNPMINRADNTPQNGGQSSSATVGSENPTSI

SATDNDGSNDDFSFEDYLRQLDLQKFDVFGSAFLL

>34-9_0119

MSDSPSTSGVKRRIKVSYTCRECRSKRTKCDKKSPCLACKNRKSACEYDSERQIKPRRPN

KDSLILRLSNQLDYYKKMCKQFVPKSEFRDFSSDLVDIDYALGSRTLKKQNTFMTNEEDT

INDLIIHSTNNNDSGEMLSIKYLLNNDDHVKRLFFKTPDKNKQTTNKISNITPSSIYYNK

LTPYQKYQFKDFSEKLLSDKAYSNESAQSEYYAKAFQFSQYDNRFDDLNIVEDYVIDKPT

PLLIMIQEDIQDNLPSYTDLHFLINIYINNFYSEAPLFDITEIQTFIKENITKGKNDKII

IKPLETDIRNKVCYYSMIFIICGVSSISHKLKTYSKNENVLDFKYQMDNSKSIILSMQLI

KCSKVYLDPNIIKLTAFMNIWLNFRYLPNPNFHSNVNDDIPTRLLTHSLLVLSDNINLYT

LNHDNPNIGLFENDKEKNMKNYFILLFSIVVSIDDIYNQKCVTFKIDDNQRERLLQYSYK

YNPLTNSESLTQTVNNNYNLYLVKLIMLYHIQDCYALITKITGSINLTQFEKRLNFMLEF

YNRELSLNYMKTPNTKSIIFDKFTPNIKLDQTVAINKILFEISLIKTTSELKIYSLLLNK

LEENKNIKYIDYFMKMINKIIDNLIMLNDFTNGSYQKYLGDLESIMMAKNCKRLFNQIIF

CTLQLLSKFILNNHEFSEIITVLFKILYKCINLYTLKYRFIHYDAFKVCLFVENVIINYV

NGEYSMFKGITNEVGQVLSNHKNEIEVAFRQKRLTDILNKDNLLELDLNYMNKLKSNINF

SHLFTDDFLTK

>34-9_0120

MADIDINSYDFSDYKDISLDTLTIDTSSIDEELLSKYNYKAVEDTFEFFNDMDSFIIING

LPVAPVSKLAALNKVVNKIFSSCGEITSLNIPTDETKTKGFAFVQYKEPTMAKKAIKLLN

GKNLDNSHQFKVNSLKEIEEFGIEEFNSTFVKPKVNKPISTDLLDNWLLDGREQFLMSID

EDSQLVWNQRSDVPKLICDNNQWVEAAPPKFTSNGSFLISLHKDYAKSWGGSKMDFITDF

HHKNVIKYDISNTSKYLITFSSSPINETYGMFDQTNYGHSIAIWDIQTGALLKTFPLPSL

SNEIQKPLTWPFIKWSNDDSYCCRAGPDAIAIYKSSENFKLNDLVKIPNLQDFEFSPNKI

VINKNKNIATDLLIYWTPDIQNQNCKVTLMEYPKKNILRSVNMMQALNMSFYWQNNSEYL

AIKIDKFSNKAKTKLCSNIEIINFAESNFPITKLELKERILTISWEPNSDRFVTAAIDEN

KYSLDDTLPPKVVQFFNTLNNAGKSWVLAYQTGRSHVNKLSWSPLGRILTVNTCCIDKKQ

KNSELIFYDLDYPGQNNIITKLLNIQKSEKLILSKDKKSRNRSADEDVNLSGTQVDRSGS

IFNNGNNLKLRELKNYTNFSFTDVAWDTTGRFFTAFCSAAKYSTGHGYAIFGLNGLPIRR

EEQMGFKWFAWRPRPKSTLTSQELKKIKKNLNEYAKKFKDLDSRTDDLNYARLQQEKQDL

LEEWTAYRNEVAKQVGKEYIFDIDPYVDELEKTEYNGDVVISTKDIVLEDEEFDALVN

>34-9_0121

MKASFNILLAFLFLQTANAVGQKNFNSKIGCQIHNDMELVSGLAGSVYYYPWLSYNSASK

SGSNNFDLYTTNAYLSGGYIGDGSIIAEGHSVYTKPGVIANDVTATELYFRYDNPCAANG

NCNGEVWFDLDYFSNSDGTPVSVPFKQFAFHMNGYIVPNITGQYILNLKYVDDLAIVNIG

SKGFTSPNCCSNYSPTGDVSGNNTIQTLWNPTGPSGINQAVLQLVAGVAYPIEFFYVNRG

SAGALQFEYTDPNGNVHQTFDDANKANSLKISGLAILLLQFI

>34-9_0122

MSDSRYTPTDYTNSNFFQPIQLSDKITLKHRGVMAPLTRIRNVAGNTYNLSTEYTETPEW

KEYIAKPENKTGEKVRGLVEEYYYQRSQKEGTLIIIEGTIISDRFGGSPYVPGIFNDAQV

KNLSKIVDAIHENKSYAVQQIVGLGKKADAEYLHSKGFKYLAPSVVYVAENDRYETKLKS

EKSGNPLQECSLEDIQNIKDDFVSSAAKAFEAGCDAIEIHGANGYLLNQFLDKKTNKRTD

KYGTSNNEDRARLFLEVYDEIVAAHGNDRVGIRLSPFGLMGDMTAATDPEDTVQFYKFLY

QELEKRRLAGKGPLYISLIEPRTDEIDLSQENEKVSNEFVFDNFKGVVIRAGNIILDNEY

TKKIVDQNDKTLIAFGRYFISNPDLIERLEKGLEINEYDRDTFYTGGIKGYVDYDYHK

>34-9_0123

MFRRNKKEAKEESEDLSNFIRRSTPIEAFPEDIKANYVPTNDEYTEEQVEKFKELREYID

TTKEFPINEKDVKNFVNHKAHDIKVQPLAEIEKFWLSDKCLYRYLKACKWVLKDATERLV

LTITWRREFGIIKLKEESSESAEKERLLGFDDISEENETGKELLMGFDNDMRPILYLKPG

RQNTQPSKRQVQHLVFMLERAIDLMDENIQDSLTLIIDFKNKHDDVDKVTSKLPPLSVGK

EVLHILQTHYPERLGRALLFNMHWIAKSFLNLISPFIDPMTRSKIVYDVKDVEQYIKKDQ

LEKDYGGLILFKYEHQKYWPALQKLTEEKRADKYASIEV

>34-9_0124

MAPESVTKIRDDLKNKKTLGYGLMSFTWRANPVPIEEAITTMRELLKVVPADTHKILFNL

GEFYGEPYLNLKYAKKFIDSLDAEDKKKIFVSVKGGMDNATLAPAGTRQGIVKSVEGCLD

VLGFIDIFECARIDLDHWEESFDTLIEYVEAGKIGALSLSELTKDQIEMVFNKKNYKDYI

VCCELELSLFSNKIIQDGTTEYLNNHGVSVVAYSPLGRGLLTKQIKSVKDIPEGDFRSHL

KRFQDEAMAHNIKLINFLEENFTSKRDITLPQLALAWVRKNNTIYNNINFIPIPSGTTPA

RVQENFAIKEISDEEFAKINEFLKSFTTAGDRYETA

>34-9_0125

MSTEILPRQTIWNKIYNNKYINGLITQSDKDIRILYLCVFIRQFQYGLLNQILIFYFKEL

HYNSEKIGVFLSLTLFGDVILSWILTWYADALKRSNIIKLGIILMFINGLTFIVYDKNFT

VLLIVSTLGIVGDGSDVGPFKSIEESCMAHLTKKKYRPIIYSWHYLFSSIGFSIGCWIAG

TIVDYLMDNKIYTSYLNCYKFVFGVYCLLSVIKFIAVQFFSENVNMNFHYNDDEIEDQET

SNTVLDSEERPLLAENQKDVVSRSLLHQPSNAGNVEMVPPAKIVNEVQQTVSQRRRDSSN

IVPITQESSKLSSATVSILIKLLVIFMLDSFSYGLITSWIPYYYEKTFLLTYTHLGLLFS

VGKIITASTVFPSSMITYALGPVKATFVVQSISALFLIMIPLVDFNLKASISALMGYYAT

ASMDVTPRQILLTDLVSANEITKVMGIVNVGKTLARCFGQLLSGYVVQSGLLRYAFIISG

SVLLSADFILILSFIGIDKKVLQHLRKK

>34-9_0126

MFSVYNLLNYIQRFKSTLTSDVILQNGMNKSISPAQTTLTENQKRKINGEKLNTNDTLNE

QVLNEKLILLEAKLTHISAKSLGIVTLLPDMIKEIYLEFVKHYHLFLPVVDIDKDPEAIY

NLSPCLFWVIILIGMRRLPRYHQSINDFNSHINVVKKVKKNSSNGGLSIDSNSPMKHLII

WVKNIMAEIAMQPIIKYYFTDDELNNSDPEFEPILNVSSVYSVQAFLLYSFWPSSSSSIS

CDTSWNTIGSAMMQSIRLGLNSAEHSVEYKTNNLQFITDQIKTWTASNVLSQYIASTFGF

PSYVSFDNAILSNCELSIYGETENDKIIINKPLKQMLHIIKFQNDCVKSLQDRDNQYDIL

LKLEQQMNILNTDLIQNGEVNDITRFFFLVTKINLFSNYFLKNDKTSTISDDEDCNDEKK

FTDKFLNEYKSEHVDITTKVGLTKLNNTCIDLIQHCNEMNKKQPDIIKYLPGVFVLNIWQ

AACIICKLSFSSLQTNIELKKSKKTYDLAVKLAQGCSLMKFDLPYRSSRIMKSIWQIYEN

LYTEWVKEYREEEFNLNLNITNRLSASVFFDCLYVLKQHSGIKRKQEMDKMKNAEIDEST

FKETGDRAKAFEEEKKGKKAKNSVQSPSVAVNATKIIQTIPLDPEPMTIPASTQSEGTSS

FNTTPDTQASIKSPKSAGAKKEILDLKSMLNKTIPVQSKANNNSNTNQGISSRGASPFLL

MLNNNLSNKMEKSLGTHAKSSSVREEAPAVPFPIQNSPLAIPNTQLNKSGRFFGGLEQPT

PLSAKSPKFMNFMNVENNVLSPGPARVNTPNMLFGTNNNMITEIFGNEPAKSSVHDESES

IASSSNYISNANNEFLWDDIDMMMNEFAFNPTV

>34-9_0127

MAVDKFGNTIDDDYVEQQDEELFEQEGDNYDDEVPSGDSEKPKKTVSFQVEDEEDAKREF

EEGGGLPAQPENPDFSSLTPLSPEIINRQATINIGTIGHVAHGKSTVVRAISGVQPVRFK

DELERNITIKLGYANAKIYKCPECPEPDCYRSFKSDKEIHPKCQTEGCEGRYEVVRHVSF

VDCPGHDILMSTMLSGAAVMDAALLLIAGNEPCPQPQTSEHLAAIEIMKLKHVIILQNKV

DLMREESALDHQKSILKFIKGTIADGAPIVPISAQLKYNIDAVNECIVKNIPVPPRDFNV

TPRLIVIRSFDVNKPGAEIRDLKGGVAGGSILNGVFKLGDEIEVRPGIVTKDKQGKIQCK

PIFSKIVSLFAENNDLKFAVPGGLIGVGTKVDPTLCRADRLVGQVVGAKGHLPSIYTDIE

INYFLLRRLLGVKTDGSKQTKVRKLEPGEVLMVNIGSTATGARVVAVKADMARLQLTSPA

CTEVNEKIALSRRIEKHWRLIGWATIKKGSTLEPLSA

>34-9_0128

MADMLKSKDNTNNDSITINKRISVSDLIQESLSLKSFISLTNTVNEEKAIKTSKKTLHEV

TTKDFNRTPKGLMFAKDIKDIFCIILICLDLKRRLVDNTSTHKKKRNFFFNLASSSNKEV

SNEAYFCFSLDDCIEEMQKLAVNIEMETTTVSISYSIKPDMSLQLIKIFFNAKLLHTPTN

RTNTELNNKSLLQPTPKGVSILTKYIKDMGIKKHIPGIVFSNLNSTYLFNFERSLVTDRI

IYNHYFIKLIFTKMFSDNPTIWTPDTKPEKIASLKTLLGNDNNVNEFDFTDKQTQNIMDL

ISHQHNQTLPNTINPFGDKNIVVPDEMNDTALLNNENRISPLYHKFFTNPESDSHVQYYS

HLNGIRMYKNKEFIKSIKTEKNTIKSKKHVISYSFSTKAIVQWLMDCTDLIYFKEAVIMA

SLFYKCGLIKPICYSPSVAKEDNFTVSKKNLYTFTKKGCDWLNWNENFMESLYSDWNQER

KKRLSQLEESVVTTDFNKKEIHSDSYDLNKENHIISLDSVLNDPGKRFLFKSHLDKEMCS

ENFSAYCDIKKLLKKMSNLENFVDVKEKNKRLANKNAKSIQLYNSLKARLMKESNESLSM

AYQIFATYIADRASKLINIDYYLRNSISTVILNSNSSLTATSSLNPFKEVLEPKEPATSD

ESAKMKNLVIEDAVDPVTKNNTTEWSIKTTNLDRKTRPIELNILNDSSKKIINKDSEYYF

SNQILRDNQVLDSPTERLVTHSLYTLSKLNPLFEDVNRKLYKIMETDSLPKFLNSNKDIL

DNIIQ

>34-9_0129

MSLPTESPTSDLKHQPKKSPYSLFKVVKTNKDLQKCSKTGHSMIDLTSKPTNLNDPQYHG

MVTEIQDHYEPLIKHIMGKGSFGEVIKGINLQTNRLVAIKKLKIMKSDDVFPIVAQREIM

ILKRMSHHNVITLNDVIFDNFEKLAIEPKDQTLKKNASANSTTAVSSNKNPFMTEAEVNS

LGNPANIAPDRFFYMVLPYMVSDLAGIIHNPYFELTIPKIKNVLKQMLSGLDYIHKMKYF

HRDIKTANILIDQNGVLKIADFGLSRLYYGCPPNNHLPGGSGAGMKFTALVVTRWYRAPE

IVLCDRFYTTAIDIWGVGCIFGEFFIKKPILQGKTDIEQGHLIFQMFGKPSAQNWPTLKY

LKQFDAHYSNKNYLGNYKGIFGTFLDNKGLDLFEKMMTLDPYQRITASAALEHEWFAAEP

LPVETLSFEKVMESHESDINNFDKLKEEMLSKAANSEKFDSLNREESKSASFNQQIPSGS

RHSRDGLEKPALKKGRSEGDLNKTLQLLDDVNPMSAEKNVHKYNRNRQQQSYNNRNIIGN

RFEKKSRFEGTNDAKVNSRFDNRHKSRFEPENKNYVNPAAKPDSAVADPVQSGSKRPGNE

VTTFINDYKKKKSEMAASQNSSIETRKQPVEQSSDRKPKKKESGSIKISIEDMYE

>34-9_0130

MDTGVFDEQTIHKLRLSLLKKVNIERRVPDCYKENLELIKWKLNKCCNSSGGKRNESFIV

QVFNPVDSGIVKRTIDEALTSDIKNNNIVINLSGVVDTTVDTLLTQFENQIYRNMDINTL

KRKKKAENKRDDDEEEEDLVIIDKLFKDKMDRYFTFLETGETINNARLDHNIEFASGENK

TKAFDTFLSFLANDGQLILNKSTSSSIGRNQLPTVFFIVDSLELFSEDIERQTLLYNLFD

FMDGNNDKYAFSTCFVGVTTLPYIENMLEKRVHSRFSNTFIPFKPMNDFATFKKDFYTSL

LFEFDYETENYDSFILSGMESQWNSLVYDWENQNLSDASDDEMTLNRFLQIVFYSNKNTQ

QLQMGLLNGLNYNNFNIKKWKDIKVEFPKAVINYFMNTNRNTIGELVKSLTDLEILLLIL

MTKKNKLLLAKEDERFTYKNSNKTTSLVQVIRLYDDLYSSKNAKDIPIRKFSKTVVKSGW

ERLIKLGIINHRTKMKSHVDLVRVFKTANSSRYTTLSNPTMERYACLLNLDEIRKIITPR

HRLFDLTSI

>34-9_0131

MSQNNNVNGSIRRKLAIVGDGACGKTCLLMVFAKGKFPTIYVPTVFDNYVADVEVDGRRV

ELALWDTAGQEDFDRLRPLSYPDSNVVIICFSVDLPDSLENVLEKWISEVLHFCQGVPII

LVGCKVDLRNDPAIIQHLQSIGSEGPVSQQQGQDVADQIGATDYIECSAKTGFGVREVFE

AATRASLMGKQNKSKLNKKSGKNGKKDKKKCTIL

>34-9_0132

MSTYAKLFRMPPITPRWNTIKDNVKRQVFAETELEQRTLKSISRNTLLPTKVRMQAQLQL

SAMPKLSKMSQIRNRCVMTGTGRSVVGDGVKLNRIQFRTRAISGELPEYKIGGW

>34-9_0133

MSEYFSTNSGAIQFKQSQLTHWNAHLKKLHSPEEIIKWAILTFDNIYQISALGISGLSII

HLLSEKFTDEKIDCVFIDTLHHFPQTLDLVDKIQKKYYNKNVISDFHVIKPLGLESEKEF

SDKYGDNLWISDDDKYDYFSKVEPLERTYKQFNIDLVITGRRQTQGNDRTDLDILEYDAL

NKVFKLNPFYNYTFKQVHDIVIEGDVPTNELLQYGYKSIGDYHSTLPTVGDDERGGRWSD

KKGKTECGIHQPERFSKFK

>34-9_0134

MTDQLTNIQTKTKDLIETFNELNLTVYDYANTEDTQKSILNNLNKIFSTLKELNQDSFAL

SKTDKNVNIPLDVIQYIENTRNPDVYTREFVESIKLANDYQREKQLAFKSMSKKLGEGIL

DVFCGDDDEDQLDEAEKLKIKESVQSIWKRGGIQ

>34-9_0135

MDSTLLNITIECENAIFTMAMHPSKNQMLIGFSTGHVCCIEYNINKDNKIKMVFNKIGKE

FEYELFNENDDAIESSETSKFVNLLWKTKRHEKSCRNVIYIDDKTAVSVGSDNHMKLFKC

GNGKILDKYHFEDLDKLSVKPNCLYTFNEYILMGTEEKGIVKVMKYDVSDNYKLEMFNTL

TGLHNGDSINKIEALNKNNYKKVIKKTGKNKIQDEFISEALSVHKFISVGQTCLQIWDCR

WKDISKVQSSEDQEDELLSLCFLNDDENDTTLFCGQGQGVMTTWKYGLNEYTDQIGRIKI

SKNEGIESLIPTMLNNNRIWCGVTDGFVYLIDGKVKRIVKKVHHGKYEDVSLLDIDYEYR

LVSGSMNTIKLWEIDQGEEDEQELMSEDSSENDFSDDSDSSFDAENDMLSGEEEDEESNS

DDEAIASFKKAMEEKPRSIEVQEIKMSNKDINKISKKQQKKKQQELKRKQESLKHGIRKF

EVVGQGKAWFCTVISVFGCVILTVLGHLYNSGHEEFVGHSVNDPTIEEGVLIAKTIFTTA

LVYLGLVIFCGSQIYLHNRKHQVRI

>34-9_0136

MSKLNSTQFPDHLQYLLSESKFLHLATSNPDTNVPSISMMHYLYIAEEQTFLAEDSKLIV

FLTPRHTTKFKNISLNKHVSVLLHDWMLSGKENPNEENENFSNLLKQLNQQELKRNISAS

ITGEAFVLKDEKEIQYYQDVLQKEDPESMNYKCDSYAIIKIKITSVKTSDEKNNINVIE

>34-9_0137

MSDVDFLNKGLELVTGAIDLDNNKKYKEAYESYYNGLDYLMLAIKYEKNEKSKELIRVKF

MEYLNRAEKLKKFLEEEEKQPAKETNAKKTESANTDEDKLKSSISQAILTEKPNVQWSDI

AGLETAKESLKEAVILPVKFPHLFKGKRKPTTGILLYGPPGTGKSYLAKAVATEAQSTFF

SISSSDLVSKWMGESEKLVKNLFHLAREKKPSIIFIDEIDALTSARSDNESEASRRIKTE

LLVQMNGVGNDTQGVLVLGATNIPWQLDSAIRRRFEKRIYIALPELGARCKMFELNVGDT

PCTLKRKDYRVLGKMTEGYSGSDINTVVKDALMEPIRKIQTATHFKKVEVDGNTKYTPCS

PGDKHAIEMSWLDIENGEDLLEPDLTLKDFIKSVKRSRPTVNQHDLDKQIEFTNDFGQEG

N

>34-9_0138

MEINQNKLKAILAKMNVDEDKSNINAKKMKKSKQLVGENKANGKARHRVQKLNKVNKTKQ

QNSSIFSISKVLKFFFSNDANKDNNADETLYDIQSRFQNQDKVKTIKKKLVNNSNKSTKS

TFVDDSVFSSSKRPLVDDSIFNSKGKNYYKTSISRKPQKEEEVDPITLKQLTNKLLEYEK

TISLLEKAVSDMSNEVTLMQQQHKYNNGFVDMSLIKEKRKINNLQSSDPIMQFEENSNNN

INDSLKKKKHGSIIAFDKLSPIKRQNTFLSPERDSNILDGSPKGTDKKRGNAITSSPTKA

YKADKLSNSNITRLLRNEHNKDDDSYDWRQIEEDSISSSDIE

>34-9_0139

MEHIFLPVDIPNHVIRPIIYRIITKQHNLLLTTSGLQTLAYIIGNKAIGGEWRTTQLNQT

KKIIDSIAREWIGRNYGKKFEDRANIMSIYKDLYESKKDTVSFNNENIEISFNDIEPGNS

TSVQDVVMKDEEDQFNVSMDVEQEQTMLKNYHNFIKFSSYDELPSLKYEHQHKTFNLKNS

LFADMEHNHKNVINPLAKPQTLINRIELIKIKHLKNSTNGDVKFNPIKDLLGRNEEVFTL

CGILRKNFKNEWILEDPSGTVKLKLDNMEYDHNDYYLVPGEILILKGIYFTIGEIFHVRY

AYFPKNEPRPVTISKRLNKMDYLGINKEQNYVLNEDFFLKLEDINVKLKKNWIAFLGGDM

FLDDQRVLNGIDKVLETFQADTLAIPRFIVFQGSFVSKSLKVDIMSNISNVYKQYSENFN

TLFGIINKYQNLLDSKTQFIFIPGENDPWFGTGDIFPKSGIPDFFIKNQNYPSVLKNSHF

KSNPVKLIYLNQEIMIVNNKYYNQFITSGIDLEIDSVHASEDENNIIDQLENKKKEKYKV

AKTLISQQHLFFHKNVINWDFDHFLQLNPLPTSIVLIDTSASSYHTSFNGVQVVNTGKFI

SNSETSLCGWWEYAPCLRKYFWKEMKI

>34-9_0140

MAPLAVKFDLFVNRYSYNSLHILYYIRKYTPEIFGDNNLYEIVDKGLAYLKKCFNYDGGF

GLKPQRESHGAHVWTSFASFAIWDKIDEYFSKQELEELKWWLSERQNLDGGLNGRPCKLS

DCCYSWWCLASLKLINYSKHFKDSKHDCLQSIDLHKLKEFIINCQDEVNGGISDRPGNVV

DIFHTCFGLTGMSLIEEILKEYQFKQIDPRFCMLKKIIDTV

>34-9_0141

MTQSPVHKERIVDMCSLNYSSNNLITISQDKTWSLHDLNNLSTILYKQDGYTSGLSAMSV

SEYDHLLVTATLDEIQMHDLRSGSLLGRSKNMDKEPCHAMKLLGNNHTLVTGGQGKLKIF

DLRKININGINDCLIDEKLVHQGNLVTSIDFKDDVLVSTGFKNASVEINTLKNGFIQNIN

ENRESMSIVGDKLLTSKILNNGQILSAGFNSKINFIY

>34-9_0142

MNLHNILNQDCSSILKTKKSHVSNDTNSLDSVKVYELPCYFSEEQLELYKKVIQLHYSDI

LQLINNDSKDMSDYIKNSMDLLAINLESVSIHPYVLIMSKLPNSNRQLVFKEFVKTQLLS

NSGKLYALKMLIENIKETKSKKDVVIYFRGDFNINNLYDFEDTNYKHLSYIPGDILQEIQ

LRRNTVSDLQIGEVKQDLNEEDSELENFGKSNINEPISTVNTKDHSGDESNDSESGEDAN

KKKSNAAKNLSTKSNEEDSIQSSIGGRSKSSLSSTTQPTRICDILEAILTASLTPKNSNK

IHVQRYDGKKGLKRDIKSTNNGDNSSKDYDNRSGASNGKKNNSNQDELTVHLVSNMEHEC

PVEFADLVITLDSCSPPKSFIPLQNVDTIALFTPYSIDHFLKLKEKNSLMSVKDVVMDVI

CNKDRAGSLDDSWISEYVNGLNGVLPLKNNNLFSKKDFFNSKSYTKGNKQLTEQILENSN

DLYGDFLENYVNGLDSGDSIFNFLRYLKNIDHTINSSELSDSDISNIRKLIKNRIHLRDD

DNLLNEDNFKISKNLIVNFDQRILYQLNKGFNYFSTNKLNRELSTEELFDKLQNEKELKK

YAERFNDSIDKEIEFFDSEYNSLVKKIVVEGIQYTKESNEKIVQGLEKQKELREEISKLK

EETSKKDNTGISNSEAEINSLKKKVNDKIKESEYLKSEINKAEKSIVDSDIELLSIQQKL

ETIESEIKAKLSGINSKKRQISELCSSEVAEKTKSIIEETSNLFTIVAELPSKRSKRQRR

R

>34-9_0143

MSVEEKVDVDLYDRQIRLWGMSTQKLILKSNVLVVNLNGLGTEIMKNLLLSGIGNITVLD

DHDIDVDDVNTYYNQFFIYKSNLEKLGGRVKRLDIAEANMRDMNPRCNLTTLTTDTNKKL

LDKGFLNKFDLVIITEILDNDFLCQINDLTRELNLPIYIAQSYGLSSMVFVDYITRFSLS

EKLLDIKNDASQIEKEKSIIAKGDFPVKLTKNTELASKELVKNEEDGNMKLILKTLNKFI

PFRDLIDGLNSEDCLSTQFNNRQLRNLKQNTTIPFILNSLNPKIFSADEYFTKCKGIELP

AVVAVVGAALVQDFISSLNKTKLINNLLLFDAKNDEMPVIEI

>34-9_0144

MDFDLNEDINGIRFAWNCFPQTKQECERNVVPLSCLYTPLKEIENMPVANYNPVVCAAQK

CKAILNPYSVIDISSKTWSCKLCNHRNNLPTQYHAISQESIPLELSSTTIEYVHVGKQVS

VPPIFLFVVDLTTDAENLEALKESIITSLSLLPPNLLVGLITYDRNVMLYDLSCNGIEKI

NVFRGDREYEMAQISEILSGVKELNASTAANNAEALKRFFLPLEQVEFKLTEILDNLNVA

DWPINGGCRPLRATSSALNISSLLMEKCYKNASGRIILFSSGPGTMNPGQIVSPELKDPL

RSHHDIDSDNAKHYKKAVKFYNSIATRCSNNSHTVDIFGGSYDQVGVSEMKQLTDSTGGV

LLLTDAFSTAIFKQSFLRLFSKDGEGYLTMGFNGRLAVKTSPDLKIQGLIGHASADEVNK

TGTVGASNVSLNSIGIGGTSSWKMSTISPYHNYAIYFEMSNTTPSGMDQGMINQQQQYPL

AYTQFITTYEHSSGTLRTRVTTVANEMKAAVLMARLAVHKAEKEDEQDVVRWIDRALIKL

CQSYSDYTKGDPLSFKLSSNFSLYPQFLFYLRRSQFLSVFNNSPDETAFYRHVFTREDCT

NSLIMIQPTLTSYSMETEPEPVLLDSISVKPNTILLLDTFFYILIYHGEQVAQWRKAGYQ

NDPTYQDFKNLLEEPKLEAAELLVDRFPLPRFIDTEYGGSQSRFLMSKLNPSVAYTDQDY

SAGNIVLTDDVSLQTFMKHLQSVVVNDEQ

>34-9_0145

MVVVSIRYLAIPKLLSVFNAEQMMLQYVDVLFNTPFISYKTLKECILFTKYKNDGSNLNQ

MPLYVLLFAKIPEYLHFYVFALVDILTLMYFIKLAVPTKVGASLKTKFWLYLFNPLTFLN

LVMQTQFVFTQFFIVAALYYCQNYKLNTNNVYKAATAIAMSAYLDVYNIGLSLICLNFFL

ETKLKQAYIIAFIATMFVLYAISYQINPYFIENVIFSCLLFKEQYPNIGLWWYFFIEMFQ

EYRNFFKFVFNGYCYIFTIPIYLRFKNYPLQAAVILFTWITMFKPYPSIGELGLILTVFV

SLFDMNIVDNKLIMWLLVIHSLVLLPVFYHLWITVGSGNSNFFYAMTLVYVVSIALILLG

MIKSVLYKEYVEVNDLEKEAKDGDKPQKKLNLVCV

>34-9_0146

MAGKKAVKKETKKAVKKTTVKLPNIPAKKDSGLENGTKSSKNNKKVPALKHKLLTENDPS

TADHESKDESPSQQDFLLPTNYLVSLYGKPIEVHLKFNDAVYKGILLSIDEYFNLRLENT

VEFIKEKETGKIGDCFIRCNTVLMISHK

>34-9_0147

MAIENSIVVPAYHEKLNIKPLTERLFKALGEEGSAKTELIFVDDNSKDGSVEEVEALQKR

GYNVKIIVRTTERGLSSAVLKGFYESKGEYLVCMDADLQHPPEYVPELLARLRLHPFVIG

TRYAAGVEMDKDWPLYRRVISSGARLMAKPLTLASDPMSGFFGVQKKELAKASEGSINAK

GFKIALDLLVKLPFDDALIGEQPFSFGVRTEGESKLSSKVMILYIAQLKELYIYKFGAEN

IISLIAIWAILLLIIFYRLFF

>34-9_0148

MSDPNLNESYNSLLQNSAFFPKTNDGKKLNEAKLIEMFFVKTSMLISNSKSLYFLNHNEE

DGGENINPDKVIIDNISTFHTKYLENEQNISVWRDIDVEKLYESHRSKKSKFPPPLVIET

YLDLINLEPNQNLYIIKKDSNQDSQSSLPNITGSAIDSDTLARSVLVCKGGKKNEIVLER

WLLELDFEESTPDALHFNTFDNEDGKDLSFEEFVNNKFLIFLRYLVSLLRMLPSQELHEK

AHSEEESGKFPAVRVSTRILDGSKPILSKGRIGLSKPLGTSTQNHLEQKSISPLETDLGL

LRVNVSYRQNVNFFVLNQQEITQILSSRQPSSQTSGNINMQRLSPNMNNHNSVDSVHSLL

SNDPIRSNGSRKSLNSYNNRGSIQYQSNFKVGSIGSVVSNSLTRNPSNSSVIATLRAHRS

STSSTNNVIPQTSQNHASSESLISQHNLEPGIHQSIGSLHSEIKPSLQHESLLKRQINSN

NSITSSVHSSIINERKFSRGSLKSETTRHNSEADEFLSLIGDEANDRKGSDGLMEKDDDF

AVTQSVTESIYRFKGMKIEDSKTFENSISRSGTGSRYADVLANYRNMSSSSMVSSRKNSD

TRDSLIDEKESGFNQYGLSPSKGFDAENLKAVLDKSRRASVESSKRMMISTSRNSIYSHT

ENDKIDFSDRRKDTGSLSSVSSPKDITAFKSMDNSRRRRSSASSSYSIPKFSHSLNNGNR

YSGSPGSNLVHSLSSNIASRKASMDNVSLAIADTSAVYADFEKPKSNLIKNNNTSLQQIP

FNDSNNGNTKQDNTEITDKNNDHDDGRKFQHSSYNDEDDLLFFMGDSN

>34-9_0149

MSNNHLRHQNGESDKEENIALIGENIDSRTKPQDLSVNDLTTFFLQDEVTPVGINDANSQ

NEMQRSITASEISVLDTASQTFKSSNAKRLKKHKCTYPDCNKLFSRPSVLKEHIQTKHLN

IRKYNCTECEQGYTKKLHLQRHYISAHSIENKPFSCSFCDKKLLTKQHLDTHERTHTKPF

RCKFNDQCEAGFTTQKLLDGHIERAHLSKHEERLTCQFCKQKLQSPSKLQQHIFKNHNDS

DTISMTSEEDKKLIQFQMSLKIKRYQCSTTDCSKTFNTWSHYQQHMKHDHPKLECLICKK

KCVGEKGLQMHMMVHDDSMINKIWKCLICQEKFAKKNELSEHCKISHDLDVGHNTEETYQ

NINEDTFVMRKKIKTTDVDLWKSNIKILNKIDNGESMAEVLLNSLGKKYQCTVPNCYRKF

KIKENYDKHLEKHNEYIKKMSLLEPNLEE

>34-9_0150

MSDREEAYNDNNDEQYADFDKEMEQFSDNENIEAQADINDNNTEAKQETEEIIIASGAAQ

FDQEIMEQHNSTGFNKLQLLKEKAIKKEDRTTTPYMTKYERARILGARSLQISMNAPTFV

TLDGETDTLKIATKELEENKIPLVIRRYLPDGSFEDWYATELIVDF

>34-9_0151

MITRSESTNFSNLSSDLIHNFKQLFKFIDVDNDNSITKQDIQKLIRIINLNDKVNVDEMF

PVNSDSITYPEFLSIMGKLFQGFPNNQQLREALTSFADEVNQKDLNKIDVDHLFEYLNKT

DFYITKKEEFVKQWKHFILSEKGTTYFLGENFLKTFE

>34-9_0152

MAKKNGNSKLSESDLLTIKGYMKNAKEDFVKKNFDDCIEWCDYILEIDQESYLALLLKGK

CLNMLKEFKKALSVYSINIKLDSNNMLGYKGEWEVFNDYLKVNGSNEKILENFEKEICSF

DVLFEFLYSFYTKCQEVNDSLTYNNIITFIIDFKKMIPQCESVLLSKCIPDSNPTSFFNT

IGCRIYSKKDSLLKSLKFELKAEKSKKIKFKALYQMKDEAMYKESLCDMYKTSPIEKIYV

ELIGCILEEEDHDIQDRFQYEEELVSYRLEKCSNLPLDEKTHYFETEVLEYIDNLVLIGP

DQISQKIFNLHLNWLDFGSLEDDLNHMNKLIMKYITRFPDDKLTLLLFTLIMNPENFLVI

QSAELDTIKENLNLNENLSDVDLCYGAMLDIMKDLKNTSLLANRLVVNHLTLIKKYEEAL

PIVQKGIEMCKTTYINYGYNMPNTKLTFSLDLALIYTYVEAPKYHTLAVKLYDQYITSDP

NNLRAKYGKAMILMERKEYEKAAMMITEYLDHNPNTLNALERLSFCELKLGNYQLSLRYV

DEIMKISSNEENPLDREFECQVRYQQSLMYLERFKHEEVEEDLDLAYESLIKSIKVNEYH

WQSYQQLGIIYSEYILDHARAFKCFWKAFFLYNGDMISAHYIVKNLCDKQEWKIAASICK

DLIDSGYVKRELQFENWPYRVLGIHSLNIGQVDDAVNYLQSSMRIDNEDKQTLVTLGQAY

LENGKVEAALKVFSRTIELYPDYEYGVFFYAIVLSRLGKFEDAEVIFNGICFDDESVDFK

DCYLLEYITHLANHAEYLSQQGFLSKSCLKAQKIVECVHICIHDLKIPHFNSLWTNLNRA

LKIYLGIGSKHDDVPIETLLDIFESAGILENEIEILKYNPVTIDSLNEEEPLSEDIDFNE

TVGIICKCMILAAKCSFIVNDYGKAIRAIKASLWFNLATAELTTSVMTGNAFYRDLAIDS

FKKSIKFQSNVSASWNGLGMATMDINYRVSQHCFVKALSISSTEPLTFNNLAILALKYKD

TAFANELFEKSQALFPINYFSWFGLALSYNIQGMDDIASRYFKHSYVLSKGKSDFIAIYY

AMSVLKENFASKKTNNDILQLQEFIFTTRSLEMYLKKKPHDSLALQSLLVLLERVKEIEK

AMPIIEDLIFKFESKISESMDNGLVISVCRLKSQYSRLLLTNGNYAKALEEAESSKEISE

FAENVTETLKMSNAVVIGLCKFFQGDVDNSLSILAEYSYESVLELFYKISFQAKEDIQFE

SWKNTKLLKLFTAIGLLNKDESIMRDTLKHLCKSNKFEEEEILELVLMIHQNMNKKDNTQ

ILHRSLFMNPNNIKAWEKEDPKIAFKLMNKKPLQLSTPEMKSEMLIKTGNINMIQRAMFL

TPWLKEPVKSFIEVYKEVK

>34-9_0153

MGLLDTIDKIENNKNDSSTTTQNPNDIGTRNVNINNDDFYYSKDMENISVRSIRPQSFLY

TTLIKKFLGEKAEAITQILVSKQRVTLKALYSYLNSTEVPMDLPSIKRTLVSMIQLNLIK

YYQDEIPKNFNPKLNNNKKFKQKIHYSLNDEGFNCLLFSGEMITTCTMYHSYMGKAAMEL

IDQIMTNCLQFGYLSINQIIMTVKNIDHPDIVEFGDEDYIKQCIEILIKDGWLVRTTPLD

QCPPKDLWGLVYNREYYNIGQVDKTLSELKRKHQSVIKSKEKFNVLTSFSQGDIVKVSLD

RLWKYKRVMHIAKLGESIHGIHIAKILNFCGDIVGNKQPTLRDPLKQCGLFDDANEKTAF

YEESEYKDENTKGCSFDALDIQRFIKQRKIPINLSNVLINSIVEKNNAASSKRVKRENAN

LNDIQEENEEELDLDLDYSEANANGKRSIKDEDEDFDINTRASIPEINACLQLLSDDMQT

IQFLKKTASGRYYFPFTELNKIIKQNVYDGLIESSLGVSSLRLLRCIRTNRLVTEKTLCT

LVLLKDKDVRHIVSKLIKINAVEIQELPKASDRSAARTVFLYRIDEAHQYQYMEDNLCWN

LANLINKIELLKEKNKVLLAKAQREDVVGKEEELLLSSELTQLKMINEREMSSYVRLNRL

LSLWEVFQF

>34-9_0154

MLSQTIKRSYSAATNVSSLSLKLVNTTGAGKKQGLAHLLSRYNFLDTTEKSGLRLARESE

VIGGTFKSTPLRDGSIELKATFLKENLPYFLQALNNTVSKSAFKKYQFDEEVIPAVLNDA

ADAASCSQLKAKDLLYQLTFKNSVLGQPVAYDQVDSISYEDVKEFASKLYSSSNAVLTAE

NVEEAALKQFIAENEFLSTIPAKISSSNATKSYVGESAALRFAAPTGNSTAAIAIPVAEK

DFATYETLKNFVTSPLFKASQDVSKAEFETYADKKVGVFSIYATGPAPAKSIKAIVESLK

KGVSISGAAKFTETFSGVKSTATTYKLDKFNYVAVGKTTELPTINEL

>34-9_0155

MTDERYTPVKLSDSNLFKPLKLNDNITLQHRAAMAPLTRHRADEKYVLKQDLSHWEEDDW

KKFAAEPANKVDSVNKKGLVTEYYHQRSERPGTLIISEATFISAKAGGYDTVPGIWNDEQ

VASLTKVIDAIHANKSYMFVQLWNLGRQADPIVLKNEGFEYLSASAVYPINDNAIDSTKK

AKECGNPLKACSIQDIEDFKRDYLVAARNAFKAGADGVEIHSANGYLLNQFIDLKINKRT

DQYGPQSFENRSRFLFEVLDSLVEEFGGDKVALRLSPFGTFGDMSGDDSYPETIQMYTYI

YQELEKRRAAGKGPVYLSLVEPRVTNPFLTEGEGVLATVNNEFAYDYFKGVILRAGDLVL

GNKYTKEIINKNDRTLAGFGRYWISNPDLVDRLEKELPLNKYDRNTFYAETYKVYEYDDE

TSDWQFTNCQGPLMLYERYLNINPQTGEIHGYQLIENEVDDIYETDQLTGEDGYRFGLMV

FNRSEQVNFSLGISNNIDFINKQKALKQTSDKETETFFQVKVDLKEDLIILKSHLGQVYG

FWIEKEDERLAVFNLLRQFVVLQ

>34-9_0156

MSEEQATVETVAPVVLATAIPEDVVEGQQEIRLFNKWSFQEVEVKDPSLVAYVQIQNPVY

VGHTAGRYANKRFRKAQCPIIERLTNSLMMNGRNNGKKLKAVRIVKHTLEIINVLTDLNP

LQVVVDAVVNSGAKEDTTRVGGGGAARRQAVDVSPLRRVNTAISLLTVGAREASFRNIKT

IAETLAEELINAAKGSSTSFAIKKKDELERVAKSNR

>34-9_0157

MVTTKLNEKVIKKNLTSLSMDDEGYEPSQTLTDLQDISMSNYVAKQMIDLYKTEFKTLDD

EDIRRKSLYDEQLESYGNIKGQFTALLTSQSKVTSLMRLYTPLSEKGESIIIELPKQYDD

KLDPECDAIAILEKHKRFKRHVKMVKDVQDVPESGFDVWELCFKGNLTKNDMELEVLSMI

LEENKMNYLFNKKIHVDEHFLSDKIIAIYFDDVLYNSSLVNEDSHNVYYRYKVLVEKSTT

KFNDFVHSIYVENFKENSIKEIQDIEHKFGLFDLGDILPGSVLPFDLNLDYVPNSLSFDK

GCYIGQELTTRMYSTDKIVKRGVPIDVLKSEKNLQPQVGWDLYTDYEFKTTDEEEPKVND

VFGSSKQGIKKRNKPVGKLIINNDEKNVRLVSLKIKYINEIFANKNKEIKFYLTPSKNEM

KSKGLKKDDYKVDVEIKTPYWIDEYTEE

>34-9_0158

MVLPLIRRTALQAVSKKTFSTSATALAASGKVRAVIGAIVDVQFEQGSLPAILNALEIPT

DNGKLILEVAQHLGENTVRTIAMDGTEGLVRGTSVVDTGAPISVPVGRETLGRILNVVGE

PIDERGPIKTKKRNPIHADPPTFAEQSTAAEVLETGIKVVDLLAPYARGGKIGLFGGAGV

GKTVFIQELINNIAKAHGGFSVFTGVGERTREGNDLYREMKETGVINLEGDSKVALVFGQ

MNEPPGARARVALTDEEGQDVLLFIDNIFRFTQAGSEVSALLGRIPSAVGYQPTLATDMG

LLQERITTTKKGSVTSVQAVYVPADDLTDPAPATTFAHLDATTVLSRSISELGIYPAVDP

LDSNSRLLDASIVGQEHYDVANQVQQTLQAYKSLQDIIAILGMDELSEADKLTVERARKI

QRFLSQPFAVAEVFTGIPGALVRLKDTISSFKAVLDGKYDHLPENAFYMVGGIEDVVAKA

EKLLAESK

>34-9_0159

MTYSVGILGCGVMGQSFLSAVQKFRVLGTSEDKEKLPTKFYTTNHNEESVKNVKKVLEEN

IKKITEDEEVLKNFGFEDHECYTGKDNIKLIEKSDVVLFSIKPYQMEQVLNQIKEAGFDL

KNKVFISLVAGWTTESMREILNNKEMIITRLMTNTPAKFGEGAVVVSHTGSEELLQKWKE

KLNFMIEQLGVMVELPESKMDAATALVGSGPAFVLNFLEAMVDAGIKMGIPYKESYILAL

KTVEGTAKMGQLTNYTHPSVLKHQVCTPGGTTIAGLVEMDNRGVKSGVINGITEAARVAS

ELGKKK

>34-9_0160

MPPKKTSVMEVATKNETLTAENSHETFSIDPDLINILQNNIIDNLKNDDNSIINQHKNNT

EIDISCLKMEEFISLVLKERKVSFKELTVDSLQEPEEEAFEDDNEDVDVEDDNIIETTKE

NGYEKLTIEQFETLKKESFNAIGSAINESSLLLEVISLLLSGPKPKQGSMTMSPYLKEHL

PMGSVNSDKVLMKQILDNDTVKNYLIKMAWKLKTIKNIEEYNEYNFNKLNEQMLIENKYW

SIIADSKLSQKGTLIKNYNNNTLGVKYGVNSNQFASLNIVNQKVEQFEVQVIPNIASHRI

LYTNKEEGLSDDAIIFKYLSVEVSDGENVSKSTIPESILSIVSKAIKPNELSAQLERLNE

LQFQELIFKTLKLECKNANILNKSFNLKEENTIEVLTNGKQKITIQFKNLLKSELLELNQ

SKLDDASLPNNILEFIKKNLLFLNKLRCFNNVTNPPIYYSKKRITNPQSENYANIIHNLL

LKINHEHIVKLVTSTISEYESLNLVASKNELIKQNQKWKSEELDNANLKKLKMETTNSNL

N

>34-9_0161

MALFTSDFIIKCRAATYIFLSILCFKNISAITTNNSLVTFSIAMRLPQVVINENSIIVGI

LSIFLFNIGLLDVLSLIHYGKIKAHFYKDFVLLRLTSAFVITFISYFNKVNVKWHNNFVF

LYGFIELWFNFIIYNCLKEELNDVNIKTKRKYEDKVLNEQIDNDEED

>34-9_0162

MTTPRLNFDDIDIEKIPSVYPTMEELMSPNDINGFINNPKIYKIGMEYGMIKIVAPEAHR

QAIVNNLEDKENDPNFKIKIREQVLNELELSNRSYKLFDKQLEAFAKISGVEHKSVEKIK

VDDKDSEFHLYDLYVKIMTTYNSGDVDEFGRYLPKLAKIGKRTLKETNIDENQSTQKKRR

SSRIKNTVYYNVNELQKQEGNENIKNSTAESNQEDDWKVPDPKTCRFLQIPMNKGNVQPS

THSLKFWKSLGPNYIKIYFFYQQYLEKYILALHKYEGTKPWACSENFKTNGISILHHKFE

ETVKEEEEEAESLSDNLLEEVCRICQKDKKLYQCEYCLESYHKNCIPFVSGKNGKLCDNC

FLGNFEYGFQELEKKVSIDEFKKMCKNDNIKTFNEDDDLLSIEKQFWSIVYGNERIKSYY

GADIHNDTKNYISGFENQPSDKIKNSFLNLMNLPNDKHTLLPLLTSITGISLPWCYFGSK

FSVFGIHLEDHFTYSVNYQFSGNSKIWYCIKISDSDKFHEYIKERYPDFIRRQKDILHQL

TATISPYDIEIHKKIGIKFFKACQNPYEYIITFPKCWHFGFNLGFNHNEAVNFILPNWIP

YAIEADNVYRRDGRKNVFDIYKLIQKNAILNKDYSSIGKLFEHVKKVLESLKLSVDILLK

EKLIDEYAPTKLDLSTYKDSYLLNEISCDKCKKICSFAMVLIFNDYALYDNWANLSLNQI

LIFSKKINGTAEGHDYENLDNYISNHSDNEYKGLHLYCVDCYIHNHQSFKTYLTTEIVYL

EDDMNRNDAFKRDNLYGFPISLR

>34-9_0163

MAEVESKLATHEPMNVEEDISIENQHQTPEIKLQDELVDNLFSLCKQLVTSSGSSDIYKV

LLQVAKLRLSLTTENLTIFLNILCPNEFPNKQSLLKYITKKKATVDNEEYFRNQLPIEFY

YLSSSGNEILIKQEILLLVGLLIQLYLLDSNLIEELNSFNRNEIIQNLFDKNQSVSIFDC

DLLTAKIWFYIQLADEKLNNKSDYTIIEHLLDHLKFAVLKHQPETQVSIICGILRAYLVR

GDILSASEFVSKVEYPENFNISTSLEARYLFYMAKIFAIQLDYETANEYIVSAIRKAPLN

DEGSKGFLQIAYKLKCVVDLLLGNIPELSFFKNSIIEGEVIKPYFLLTKSVKLGDINNFT

NIINKYKNTFHRDDLHMLSTRLRSNVLRAGMKNISKTYSKIHLRDICHKLKLDSEQTTEY

IVAKCIKENVIDAKIDYETSVVETFKESVLYETREPQDIFDQRIYFANQIKNDCLKSLNY

PKKDKKEPDVEEDEEDIFAMNPETLSSLLNDFYEDEFDEDF

>34-9_0164

MSFIISKLPIGDSLDLSNFPFKKVIIGITCANFLFERYVAYRQFKLLTSNDRKIPEALRG

KVDEKKFIESDAYGLAKSKFEMVTAFTGMVTGLITLTKYSSIWNYSVSIGNAALSFVLRR

PFELSLIGNNIAFFILSNLISTLLTLPSSYYQHFVLEEKFGFNKMTRSLFFFDTTKLYFL

ISALGSVALYVLGTVLKKCTENFVFYIWVFILCLQISLFLLNDLVIQPLFYTFSPLNNKE

LEDKINALAKECGFPLSEIYMIDGSKRSSHSNAYFSGLPFMKKKIVLFDTLIKDSSIDEV

VAVLGHEIGHWKMNHISQMLVISQVHLSIFFKLFDIVFKNPKMFSDFNFIIDPSSNLPTA

SLPGSKYAVVNDFPFLVGLLVFSQLIQPTDLLMNFAMNLFSRHNEYEADNYAARLGKGED

LKKSLVQLQVTNLSTVSVDKLYSMYHYSHPVVSERLEAVDKYIAKQAKKSD

>34-9_0165

MYAIRSYYVKFSLFPNSQNLYLRNLHEVVLSFLAYSIIYEYLSTPILDIFLPLFFVGKKR

AKQLEQWKDLKTSKDNKKKQSFLNYKVHIVTMVQCLISIALVLPTLNLDFGLNIIYFQDD

FITMISSVTAGYFLWDLYVCVKWFELFQFEFLIHAICSLFVFISAIHPNYQNYVSKFLLF

ELSSPFVNVNWLFSTLMKEFEVQIPMILNAINGILLMLVFFLVRICWGWSCILIMAYQVY

SKKWYADPNFPKTVIIITFSINMALNTLNCVWLNKMVKIAKKMAGVGASKKKD

>34-9_0166

MSYPLVCLGNPLLDVQANVEPEYLAKYDLKSNDAILIEDAKSDVRMKIFDELVEKKDTIF

VAGGAAQNTARGAAFELGPNKVAYFGCVGEDKFSQQLLQENEKAGVKSLYLINKEIGTGK

CAALINGHDRSLVTDLGAANHFKPEHLDENWEYVKNAKAFYIGGFHLTVSPAAIVKLGKH

AQENNKPFVINFSAPFIPQFFKDALVQVLPYATHIIANETEAAAFSESFYSGENITDLKE

IAKKIIADYNQKATVIFTHGLEPTVLVNSDLSTKEISVHALDSSKIVDTNGAGDAFAGGF

VAGIILDEDIEKCIDRGQALAKISIQQVGPSFPVK

>34-9_0167

MLICVNLIGGQGFAEFEFIVSSIKLVAVLSFLIFGLIMDLGGVKGQPYIGGKYWREPGAF

RGENGINKFKGLCVCFVFAGFSYGGFESSVLLSSVVERPVAALKKARKMLVYRIVIIYLL

LILFIGFLVPYNSPKLLGGGSSESDASPLIIAASKVNAYPHILNAVIIMSVLSVANNSLY

TSSRVLHSIFSQFYPNPKLTYIDRRGRPLACLLIAGLFGLLCFIAAAPFRVTFFNWLLAI

SGLSLLFTYAGMNLAHIRFRRAMKVQGRSLNELAFISQTGVWGSYWGFILIVLIFIAQFW

VAIVPIGEGKADANAFFQNYLCAVVWIICYIGYKLYSKEWKVLIPAAKIDIISDRIIHDH

EAILEEEREANLRLKNSSFAFGFATNICYALDFLCVLPLELVTSALLIRYWTTSVNADIF

VSIFYVIIFMINLVGGNGYAEFEFVISSIKLTAIMSFLIYGLIKDLGGTPNQKYIGGKYW

REPGAFRGDDGINRFKGLCVCFVFAAFAYGGFESSVLVSSVVERPAQALKKGRKMLLYRI

FIIYLGLLLFIGLLVPYTNPKLLGGSGSESDASPLIIAVSNVNVYPHILNAVILLSVLSV

ANNALYTSSRTLHSLFSQFWPHPNLTYVDKRGRPLTCMAVAAVFGLLCLFAAAPFRVTFF

NWLLSISGLAALFTYGGMNIAHIRFRQAMKAQGRSLNELAFISPTGVWGSYWGVFMIGLI

FIAQFWVALVPIGSAKPDANNFFQNYLCAIVWIICYIGYKIYKKEWQILIPVDQIDIESN

RIIYDHEIIKLEEQEKKLKLKNSSVFKKFLSFWC

>34-9_0168

MSSIDSLENKVTSPLTSNDNQNSSDLSYLKKDTRSYELTADNEDTNEGLQRQMQTFMLLV

SMCYAEFEFMITAVKFTAVLSFFFFALIKDLGGVKGEPYIGGKYWRDPGAFRGENAANRF

KGLCACFVFAIMSYGGFESSVLLSSVVQQPVRALKKGRKMLCYRIAILYLGLIVFISLVV

PYNSPKLLGGGNAASDASPLIIATSNIVAYPHILNAVILMSVLSVANNSLYCASRVWLSF

FTQYYPHPTLTYVDRRGRPMTCLIIAFAFGLLCFLAAASFRVVFFNWLIAVAGLAFLFTY

SGMNLAHIRFRQAMKHQGRSLNELAYVSQTGVWGSYWGLLVMGLLFIAQFWIAIVPIGEG

KANANSFFQNYLCVVVWIVCYFGYKLYNRDWKIFIPLEEMDIMTNRTIYDGEAIKEEENE

RALRIRNSSIFKRILNFWC

>34-9_0169

MFKFLVDPGFALATNCVYALNWLCVLPLELVTSSLLIRYWTTKVNSDIFVVCFYFLLISI

NLFGGQGYAEFEFLISSIKLTAIISFSIFALIKDLGGVKGTPYIGGKNWRDPGAFRGENG

INKFKGLCACFAFAGLSYGGFEASVLLSSVVERPVAALKKAKKMLLYRIFAIYLSLVVFI

GLLVPYTSPKLLGGGSESDASPLIIAAANVKVYPHILNAVILLSVLSVANNALYSASRVL

HSLFTQFWPNPKLTYVDRKGRPLACLLIADIFGLLCFFAAADFRVTFFDWLLSIASLALL

FTYAGMNLAHIRFRKAMKYNNRSLNELAFISQTGVWGSYWGVLVIALIFIAQFWVALVPI

GTNKADANGFFQNYLCVVVWIIIYVGYKLYNKSWRIFIPIEEIDIVRDRIIFDHSAIVEE

EEEVKLRIKNSGLVTRIKHFWC

>34-9_0170

MNEALSNDDLNFVSTDMTNKVTVSSKETYLEDYDLESTTSNTSNQRPIRPRHLFLVSIGT

GVGTGMLVSTGSALRQAGPANLIIAYILVSSFIFTTYNSISELAVVYKDLSGSYNDMFKF

LIDPGFAFATVFVYALNYLCIMPLELVTSSLLIRYWTTKVNSDIFVVCFYVLINAVNFIG

SIGFAEFEFFVSALKLIAVISFMIFGIIKDVGGVPGQEYIGGKYWRSPGAFSSHSKIERF

KGLCACFVFACFSYGGLEVSVMLSSVVQNPVYALKKARKMLLYRIFLIYFGIIIFIGLLV

PYTNEKLLGGGSSASDASPLIIAVANIKVYPHILNAVILLSVLSVANCAYYSAARILHSF

FVQFYPHPTLIKVDRKGRAVTCMFITSIFGLLSFFAAAPFRVDFFNWLLALSGLAVLFLY

FGFNLAHIRFRAAMKVQNRSLNELAFISPTGVWGSYWGIFMVVIIFIAQFWVALTPIGTH

TPNAQSFFQNYLCAILWILCYIGHKLYNRNCQFLIPLDKLNIDKDRIIYNHEELMQQEKE

HLKMTQNGRFWKKLFNFFSLYNINKMSEEQKPEVKPATTDADHVSIKVTDGDSDIHFKIK

KGATLKKLMDAWAKRQGKDVSALVFTFEGITLTPEHTPEDLAMENNDIIEVYRHQSGGYV

C

>34-9_0171

MQQNNFNNQQYSPHNNPYQNMLYGQEFNNNNQTANINQFYSNRQNSIPNGNNMLSNFYNN

NQHLNGTAIIDEDDFNYQNNNQFNQFRNNSIGYNQTRLSQGSVGYNNNFDNSNMLDNYNY

TNNFSQQNNQFIRPASFSQHSTNSSNSNNNHNFNYSRMSSFSNSEAANPYNDQPPMYNRA

SFGSNFSNHPANVGNMRNNSASFISEYNNNALSSASTITEPLIQSSMLNFNMNKNSQIQN

TAVLTQGNNILNSRKTSTSSTSNHSVTTPSGNNALLNTPRRQSKSSTSEKKNSISKDSSV

KSEDGLLNSNIVVKPQQLIKTTSKEKSSTDSKKPVVGVSKTIESTETTSGSSNFTNTSFQ

IPIISTKKQEQLLAKIKGNGLYINETTNKQEVSKDLQTLYDQAGQNYFAQEAVFQFVNSL

KAKLKKDYLNNINGNNKFNKFMKYLFSCNEQFVSEQFNSSSNILKDTTPFTSSQGKNLCL

VALKNGKLELLSVSKHFTNSQTLKPKNLVIIDGDRGIDLAMVVEPHMSFVVSLLINFLKK

KIHFDSLITDPKKHHPNKDFIDALLERHTHVLGTKPLNKIIDTKLYDLQELTQLIIPSKQ

VVRFASPKDCTGSLLQKLQEELKALNFALSKLDSYNNDALEGQENKNNKLNIKILNSEYQ

FDKKKLTFYYICKERNDYRELIKELFRFYKTRIWLCAIPNNLGIIEKGEWFSGSEKNLIK

GTFEVSKKKNIVNFEEIVLDQFQIAVYIELLAQLF

>34-9_0172

MTLKKPLKQTNEPTTKKANLPEDVANLQKRAFSLSTRPVSRLDPGIYGYDSSVNNSKTML

PEESDDSNSEYESESEEENTVIDEVEVERRMMESLRRSKFLGSQFDLAMKLASTGLSSST

TKAAKRRSLLAIQSQYGGSVIKKNKKHSAKRKSARKSLIKKKDLLEECDDDNKAILDDSK

AVNKSTDNVDENTVLVYELEDENDEVNKLSDVVIPRVLKNGENEKRLSLLSTFSSTNLSK

HLKEENNNKEQFLFEDKNYEEDGEVEIVENKDGEIVKSRSSQMRLNFADRFTGSDEEDEE

EPKEVKLPDLPPVDQSKSARNSLLLEQKLKENERKISEKQEKEVEAKKQDDKIVIPKKNT

LTEEQTIQRKKSGIFRIFTPNTEITDDVKIIKYGDKHDKELLKKIYYQFKDWEKSKLGLK

HIETDGSSYIKGGISSDNVLSLRSTAFELTIEGLNIKLRKLSGSKKSFNRFSKELEKWLK

>34-9_0173

MSTIQEQEQFHHSREATDTSKIGPWKMAETIGFGSSGKVRLAIHEKTGQQAAVKIVSKSL

FMAHQNTTSTVAGGNEDKDFLPYGIEREIIIMKLLNHPNVLRLYDVWETETDLFMVLEYV

EKGELFKLLVERGPLTEREAVRFFRQIIIGISYCHALGIAHRDLKPENLLLDHKSNIKIA

DFGMAALETKDKLLETSCGSPHYASPEIVSGLPYQGFESDVWSCGVILYALLTGVLPFDE

ENGDIRQILLKVQAGRFYMPENISREAQDLLAKILTVDPKKRIGTRDILKHPLLKKYPSI

KDSMSIRNLPREDTYLNPLADGADIDPSILNNLVVLWNGKDRQEIIKSLKEPKANLEKTF

YGLLYNFKVGIQQQHKILMAEEEDEEIVPNKRSKKRTSPQKQKRYSSSTSSFNALPKSQS

KRLSQISVSSASRKSSGSVSRPTSQYLTNQKHLNTSSSVSSSNSNDVTSSSLGSTPLKRN

GTLNGGYVVDDGFSPSRDPPPPPTYTETPKDLEPQQIEEAVANKRISKSISKRLSRNISR

NSLISFTQDNNLINHSPEKSNVEYTSLGQTSRQGKRSSLTTKLISTYAKLTTLQESDWGY

IETETRRTSQSFATLIDEVFEYEMLEKERKKIIKEKLIKERELKKREEELRREIEEKEKA

LREEQHKKELAEKREREKLESENLKRERCLKIMVHEKKLEQRLSNTKERLVYLKSLLNEE

SLKILEEINLEEYMNDDVANSDFVDQTEDIGDELNDDDNSSAFTIDDYEKDEPDTKE

>34-9_0174

MNKVFNQTFANTHSKIEFPKGNLNVVYPSSSEPISPGTQLKAKDVQDIPLVTVSSDKVTS

QESLYTLIMSDIDASAEGCHYLVTNLQPTVKNDKLVVTNKSKNKNIVLEYVPPGPRIEQN

HAYVWLLFQGVPDPSKAKAVGECRRGYGTDEKYHGAEWFQDISGLGELIAFNYHYVKNDL

A

>34-9_0175

MINTLNLPKSIGAALDQHSILSDIFPKSDISLPVLSQSLLSVQYGQHQASLGNTLPVNDT

QELPQIQAILTENFFNNSSEDLQINDIDTEFTLILTDPDAPSRADKKWSEYLHYIGTGLK

INATHPGNTDKFKIIDASHLKTLVDYQGPAPPKGTGVHRYVWLLFKGSLSKEDVAGLSDN

RVNWNYGEPATGVERFVKEKKLGPLVGINFFFAENKD

>34-9_0176

MGKKFLFTSESVGEGHPDKICDQVSDAVLDACLAQDPLSKVACETAAKTGMIMVFGEITT

KAVLDYQKIVRDTVKQIGYDDSSKGFDYKTCNVLVAIEQQSPDIAQGVHETKDSLEELGA

GDQGIMFGYATDETPELLPLTILLAHKLNMAMSDARRDGSLPWLRPDTKTQVTIEYEEVE

GRWVPLRIDTVVVSAQHAEEISTEDLRSEIKEKIVKKVCPPELINEDTKYYIQPSGRFVI

GGPQGDAGLTGRKIIVDAYGGASAVGGGAFSGKDYSKVDRSAAYAARWVAKSLIKAGLCK

RVEVQFSYAIGIAEPLSVHVNTYGTATKSDEEILQIIKNNFDLRPGVLVRELDLARPIYL

PTASNGHFTNQSYPWEIVKELKF

>34-9_0177

MSNIKRLTRESSDLNANESLSLLSYYHNLSLVETLITTNYEDKSLISDLMDTIENIKPEV

SEIEDENKVKIFIKYLIVMYKNNIDLTKQYLSGKASSNVDEDKNNAVQSIWLNIDLIETF

EKSFTSEYIESNEELLELVKKLDGQNKVKTLKMLLKKVLTFVPDFDKQDDELLQQQLDTY

EDKEAADSEEEAPEFESDKEISAINHNASDLNLPVLPDTHATEPETALDLEDIALTRKTN

IEQQESSDESDYNTSDISEIVTSQETIDKMVKLAKIGIQCLQYEDFNVAKDNFANLIEIL

NQELDKIEKN

>34-9_0178

MDDHNDNKETMIMFLQNFIDDRDTPNLFNILDDLIAHNSSNKLTLDFSISIDDDGNSTLH

WLSNLCLIDVILDILRIHKSDNFNDENLFMKYSQNLKIIIDFKVLNNYQETCLCQCIKSR

NSYITKSFNKMLDIYQDLIDFKNEQNQTILHILSENYLQEASEYYLETILAFSKSKFDIE

YYNQWINTKDYKTGNTILHTSSINSNFDLIKILLKYGADPYVTNNEYTRSLDYGIGELSF

NNKNPSLKLLEDELFNINQIYEQDNKNYKEDIVNLESNLEAKRLENNKLEKIIYQNDLVK

NLDNLKTYNFLNDGLMKQKEILKSQFDSFPEFETFIKNFMSQKDNKDFMIPLISQDLLRY

VDDGIGDNLNQKLVNELDELSVEQLDTMIETYKSQNNSLANLLTNMHNQRSLELNKYREL

IAKSLDMSLDDPDEIDRLIKNMSASIDSS

>34-9_0179

MSEIAAKQRKIKMSTVDSYNPSESTFIGAPSSSLANDLKNTNINLSSSIGSNTNTHNSST

ILESENANYSIESNPEDEFGMPTNDVQEPSIKKILQILTFDIKENEQSKTITIGRSSSKN

DYQLSPKSTLVSRQHLAVTYFPNSNTLRIKCYGKNGLVVNFPKMLNYKLIRQLKDKIYEL

VPIEESSSSDDTDKEVFKNKGITSFVLNEKEICFIPYMTGLQLGFGTISLELKIKHSVVE

QEISNNESTADNIKASKNEDQSVLLSSPLSSVVEYHEEEEISNNEVIQNLNDRLALNDTN

EDKNNAGKKASHKRTHTETESVHTNVVKKQKTSKSTSSLLQDLMEKIEVEKIMVKEVVHQ

LTNQIAFANIQQIPLKQLYESNSICSTKLSLPEFKIFLEKFLLKLEPSIQIILRKGKDAA

GKLLDPEFFYDVEKDHNKERVLIVNNLKGGRSGLRSCRKVHKQYFWKKPTK

>34-9_0180

MLSNTILGKDPKGTPSFGKKHNKTHTLCNRCGTRAFHIQKKTCASCGYPAAKMRSYNWGA

KAQRRRTTGTGRMRYLKNVSRRFKNGFQSGVAKPQTA

>34-9_0181

MDFDSSEEEDEYLATYIDRHREKLKETQPVVDTANIQDNLFIAKGEIAVLKEKYEKSEKL

NVSNTTSYLKEKEQLISKYEDELNQLKSEISALNHEKTFLNHKLKVSSRKFEYENDSNSM

IIDSENNVMSKSSPAVLNKIKDLSNSSILEKRKKRDEDIHNEILNDEVLPKRTLLKTSIF

DKVPEDNDFIEDEPVNVKQKLESSTISVQNNTTKNLSQEIKPKKHFLNFKTTVKFNDNYN

LLTLLIGYKLPSFEKTNIELLDINSKDDQIGTKFINMAREFVMKLSLSEFIIKLIENLEG

YVKNFADRKIDIHYNNSIPLIISLMYQIVNFRPSVFTNVYLYYRIIKVYELIKEYEFVFK

SKSILDAKLQKIIPKTDVVYFDFNDSTVKAKRFNIGIQSLEKNNENLFLKNHTSLLSTSY

NDVQDSKSRQNNQQADEDDLLIDDGIGYIAKRNKWIDRQLSQKTDPELIKYRDINEIKDP

VDYLYEMPSDHVEMVDYLIVIFSFDLFESLISNLDFIFGKKELFDNEDIDIVSEIYDCIK

ALFDITFTTSYQPLILPILTSLNVFNDFLNINLKLNYKNYVIKFIDMISVQSEWLLKINL

KNYLHKLPKKQSICSLKRFIGGKAISQRNTLDNYYSLLLNETCQLKTFENDDYIIVHEVC

EDISCVILDNIFDFINDWFKCFIYSKGDKKMIDNVDQNGTLTTLLKNVTVILTTNFMACQ

GEKTKSDLVLKKRNTIKCIRILQMFHDRLNTLKLLLEKKPIRKNSEVEENEVSKNSIDSS

NINSLNLEHLDKTNFDTIKVVTDKMEHLIINDENWKKDLIVYVSRIIYQDQKNVLIKHEL

LKDFLTDLLDGLLTFEESESIYDALIL

>34-9_0182

MVEDSKIRDKQNQEKFEKGTYRRQPLPATLVPKQPTVLTSRDKDTQRLIVVLSQASLETY

KISSSNSSNHKGGEDRYVLLNCDDHQGFLKKAGRDISEARPDITHQCLLTLLDSPINKAG

KLQVYIQTSKGVLIEVSPSIRIPRTFKRFSGVMVQLLHKLSIKSINSHEKLLTVIKNPIT

EHLPTKCKKVTLSFDSEVIKVKDYVTEKLDSDESICVFVGAMARGNDDFADEFVDEKVGL

SNYPLSASVACSKFCHGCEDAWGIL

>34-9_0183

MSETLVICSSIIYFILDIWLGTYACLFTVAAEVTVDVATNATEAVISVSNKTMHAVAKDL

DIGLNGLSDGVDDLIKIYDKVEDKIKQIFTSSTSKDQTNISNKVKHLNLTISSLNNFYIN

PSINSKLKKLSGEVPDFSTLKNKTKHWIETPFEKIASELKNPNYYELTEPLNSSLSLKPY

KLILPNNNTTASLQINYNSTSMKWCEKELVPEIKKFMKKSKKDLITFKKVIIIIAIILFI

ASLVPEFYKEFFFWRKMSSMRSNLEDKLAFEKNSEEYKVSNGLFTNDSSQFDFKNTKQDY

KEFEEIKANHTGDDFNLGSKVQDAYLMTFQDWQINLLGRIPFLKDLIIFNSNNLADKNNN

FDMKKLKINWTLKYMLSKRTSLLLLICIAGYFICLIQYLIIHRIDVFINTKNHKKLKIFK

FLLKDNKIKQVVDAIGSSLDTWQDTTNNYIKGVETRGNNEVVVKNIDYLADKVNKTALMI

YTDISNTIDTAFNQSVFDKPLKNLMNCVIGNKLLSISKGAVWVKKEVHVDLPKISLSEAY

SIFNANNSTGYQSQIMTEITSIEADMINEINHMIQYYYKDVIKSDDSNNKGNVRSFSVGN

VEKTTYKKQAIIEDNNDFLKPPPSMNAAISEKKRSSSLTNMMRILSVNTPNNESPKSNNS

SDKTSPIGFHKAIDLENHSIAPELVPIVTLLSAQAHRRYHVGVFLLYNDLNNDGLPGSRK

WQEQYAVLIGNQLALWNSSDLDETNMKLNQNTKTQQELAHDLFNVASKPKYLNFIDAKFK

ILDGKDNTIVLSTTMKNRYFMKFSDKKSFNLWRSALRLCNFEYSSLQEAYTGAFLSSKGA

KLSDIKVILADSKFNYEDWVSVRFGAGMPWKRCYAVVTPAKQSSKKKKKNNGSSYGEVVF

YENEKKISNKKYAMATVTSANSIFAIYPSSPLLIDNSTMLKIEGEIEFININNDSKSKKS

SKLETTSKQGDIFIMPEKHNGVPGYDTIIRFLVPMLNSFHLYGRPDRLISSRDATNSLLF

GLPTLPEIHYLQVADLLPLAERSDSRNWGDKIWKDHIKELLAGKIRKGYSGCGSASGYSG

AVISGSITTNELFGGSPRMQPIARYLSGSESPSKPTNAQERLTSNPIQNNFGQEAKKVDI

NNVSGTSKMKINTKNINAPEIKQEYKTPVNEVPNMNIPSFSNKGSHEKEVKKPISSPYHE

YKKSSSVEQKKSSPPPKPKAVKPVDIGFISGANANVFASSVPYTTKQPDVEKLKSSNKNK

WDSVSSNTSIIDSYANSNTHNTNFAMPTSGLGIVSSSHTTPKKTELKQEQNSAQDDDYVV

NTTDDPLADFYKLSRTLSNFNSLPKLASNQRESTENVTSKKAKAVEQEPKFLQPEMRDAS

TGQMFYDAPSGVPKQANLKKSPQLHNMATFTKEEPKPKKAEHHLYQNPTISNANKSIKVP

QENVNPYYKQQQGDLYSKKNVNSVPQMRIKSPTGINQYPPNGGYSPVQQNFQHNNGGYSP

VQQNFQHSNGGYSPVHQNFQHNNGVYPPVHPNFHPNNNGTPPAQQHFQQNGYQHGNYSQM

NNNRNRQASPTVPQQQYVMPSGIPVKKKVPVSQQYKNANNGGYSQFMPN

>34-9_0184

MFRNTLFAYNACRQWSMRSFANPRIHKTNGMWRMYGSMRINSHARLTYSTKTTESNKNKE

GKNSEGKSTDKGNFREEAKRLFKLLRPEYKWISFAVFCLILQSTSSMFVPKLTGDLLDLS

NTYILKNNSSEEGEKALNYTESGRMKFELWGLSETQFFTVVGSVLVVGCLSNALRLIILR

TTGERIIARLRTRLLNRIFEQDSKFWDVNKTGDILSRLNSDCVVVSRSLTNNISDGARSL

FQGGIGIFMMLTISTELAGSIILFLTPAVVISRFLGKRAKTISKKVQEKIGEILKINEYQ

LNNQNLIRNYSAERVELVRYSDSIRNFYKASLKDVMNSALAQSTNQILGTTSLVACLLIG

TNMVQQGHLTAGELSSFAMYAIYSASAFAGLFNFYNEINKGIGSYSRVIEIYDEKPSISP

YKGKKDIYKAIENYNHNNVNNQTPIVEFQDVKFSYPTRKEVAIFNHLNFKINKGENICIV

GSSGKGKSSLLNLLFRTYDIENGTIKVYNEDIKNFSLRKYRRHVGVVPQEPPLISGSILD

NITYALTDSSSKKRKFTKEELAAAIEKANCTDFINSFPEGLMTRVSNKSGLSVGQKQRLA

LARQFLNNPDILFLDESTSALDGVSENIVVSNIQKRSSLEGKTTISIAHRKSTIKFASRI

IVLGDDGKVAETGSYEELVAKKDSKLNDLLAHNMNNL

>34-9_0185

MLKIGAGIYKDWKDRLTYKSYKSDWTDALSYRVLPSTIEIFFNNLLPAIAFSQDLSNRTS

YQYGINEILLSQGISGLLFGLAGYPLSIVGVSAPSCILCYTIYNIFVDKQTNIPKTKYDD

GFEFFPFMFWVYLWSAIFTFLSSIFNFLSFFQYVTVFPCDIFGLFINVVYVVKGCQLLGE

SFHKNDTLDHVADGFGNITIALCMTFFGLLAKNITSTRLFNHKIRIIIKDYCIFISVIFW

SGVIHFGNAFKFVHFEKLNISKSFEPTSKARNSSWLAVNHSISAKYVFLALPFGIIVWIL

LFFDHNISSLMAQSHEYKLKKKSMYNWDFCLLSVHTFLCGILGLPAGHCLIPQSFIHTET

LIIYKDAKKEKKTDEVNADDSANIYISGVVEQRFTNSMQGLLMIVCMCRPFLVCLNQIPQ

CVLSGLFFILGIGGIQGNAIIARILFLLSDYKTGSLEQVKASKLFLFAMISVFFAVIEVL

ISQLDYVSIAFPIILIISIFVTFFFKYIFTNEELEILDNKVVSRESIKNLLPENLNHY

>34-9_0186

MLLKYSLITLLIYIKSVSGSIGDWYPEQIQCKYHCEKDNCDYTTSLMDEDVDVSSYLYKP

TFSQVNPSFIDKLLFWDCISLCDYQCQQLTTDMITSRMSNTDEKIVQFHGKWPFVRIFYI

QEFWSTLFSLGNFIPHFLAYKKLNKFKLQKRHFLINNYKLVSIMGSLAWFFSTIFHFRDT

ILTEKLDYFFAGGTVLSGFYACTMRFLLGGDLRKHQKYGSLGFSICLLIFTSHVFRLYID

WSYTYNMRFNILFGVLQYIMLITIAVRNYYTYKNNRYVSLFKVSFLPVLLVIGTSLSMSF

ELFDIFIPWLQLDSHAIWHGLTILPSFYLYEFFIEDYKALMGSIKN

>34-9_0187

MNYNNLESEPFVDNELEDIQKDINTKIDFLQEKVIKDLIRVRLFQNKKALIDQYSINDQN

NSNSNQVDSNEDNDDIEIKLLKDKNNELENLIESKKNEIDQLRKTQESIETDLQNTINDL

KLNCEQYNEYIENLKNSVLANKTNEKKQLMNDHKAENETINNKNELISKILQLKIEIIRN

SIVFQFKNKYSVTLTKDTNNTNKITDWKSKSYNLSISDRNKLEKEFNSMDLKSWIVNVRE

LLIK

>34-9_0188

MTNTPANEYVQSIIKGFEIDSDLLKKLTSKYIESMDNGLSHSSGGKSHLPMIPTFITKLP

NGTEKTDILACDLGGTSFRCLYIKLKGNSKFTTEMKKWEIPSDYLAEEGADLPENAFFDY

LANKIVSFVQYHNSNDENKDLLSTKKNFNLGFTFSYPVEQTALNAGNLIRWTKGFEIKSV

VGKDIVELLQKSIDNKLNSVDPEKYSFTISCVALCNDTTGTLLSGAYTSTYDSQDIVDPI

MSIICGTGFNIAYMEDLASCTKLTEEQVSTIKTKDVTEMCINTESGSFDNELSVLPVTQY

DKIVDEESTNPGYHLFEKRISGMFLGEILRLVIVDLIQKEFLLEPKPSKKQYTHKFSLKT

EVLSHIEIDDSKDLKQTELYLIESLGLRTTTEQRHYLQQITRAISRRSAYLVSVAISAII

IKSGRLDPKSSKYKYHQELEVCCDGSVVQYYPGYKSMIRHGLSLGSLGHEGERRVHISMA

LDGSGLGAGLAALTASK

>34-9_0189

MPFNGKSANTNSMMRLFKKFFENTNENNAIAQSNWSVWWNRDPKPSSIIFKASESRLVKE

LIEPDIVKKGITDYSINTYTMKDGLQTWAFKNNSRDNKALQSKLIKTPLVLIHGYLTSSM

HYHKNVADLMTKYKEVYLIDLPGHGATDFHQKIVYPDHEPAHKLELEFERKYEHKENGET

SKNKELFFYNKKQLDSANYQRLLKGIRKDFFNDSIERWIRTHKMNKINLVGHSYGGYFSF

HFWRDRPDLVNKLVMLSPLGVEKNIWSINNLILQHDKYKVEHDPTAWTYLPNRKVPEFFL

NNQYKIFMAMGPVGVRLLQNFFKHRFNKESPLFQKYSFENCYLQSNDNLELNTILFNSFF

TNYLTSYDPILDYGEDIEKRKDDIKILYGDHDWMDKRCAKEFTHSSIVPNSGHNLNIDNP

KDTLNEIVNFLK

>34-9_0190

MLNSRLTPVVHELAESLKSSKIFGLFSNNLFKSSSLPSPNSYEALRKSHTEYIKNGLSKH

FYDERLQVDVKTYEMNSGMYVTYFKNLYPNTKHKLPLVIVGDPILSSVSILNPPTILTLL

KKYSRIYIVDLPGVGLNNEKKLVESYLPFRNTLPLIDPNFRIRKSRLNNSIEYLTDFEDH

HYLDKIDKIREFYFDKAFDEFITEHQFQKVDMLTISVSSYFVINFFENCNKSVVDKLYIV

GNNPLKNSIFSIDRANPRDVSVSQQRIYNFKDTMVNHNPAKNTCMIAKMRSEVLNTIRDY

IKFGDVSKNAILYDFFRLNAHMLEKTYEKHFDPFVDVTYNLFAKNFYPFENYLFVYEHYN

KFININSYEVVDLENQGFMSSLMNVQPKEYVDFKNKISSNDGTFLDGNDANVIIGLKRR

>34-9_0191

MFGFAKKLVKNLEQSVNETINTINTRNNTAISDQYFQSIPEHLLIRYYDSTNTLKVITNV

DQLYGLRVVQTDEYQLQVTSFFDYIIGINDQPIPVVFNEYGYMVVDYNAIYKILNDLVLG

EKEVKLNIWSGKGGFYREEYLTLEPNSEIQEINISTEENNNPIFLSLGFKVQWQPLIAST

YVYHILSIQDNNINKNRLVANSDYIIGCQQGLLCTGGEKLLYNILQSRQNQEIELQVYNS

EHDVLRNVVFQLDDKAKLGCNVGYGYLHRIPIPREVQEKMVSEYNDQEEVKPENILSIPL

QQEHKNGIQISENELKTEFKPLSFEQQISNANNRKKHHADAKKLDNSLLSQLQEPEETVY

KSEASRGSIAPPPIINKN

>34-9_0192

MLFSSKLAGITALASLTLAQEQSAIAPEDSHVVKLSQDSFDSFQKENELFLAEFFAPWCG

HCKTLAPHYVEAAKTLHEEYQIPIVQVDCTDNQELCMEQGIKGYPTLKVFKKNGEIVSDY

QGQRSKDAIVQFMVKQSLPAVSEFTDLESLNEFLAKQKDNVIISQYFADKDSQQLNETFY

NVAENLRDEYVFVNYLGNDEVKSYNISVLTPSQDPIAFEATVAEEATLKESSESLSDFIA

IESKPYFGEIDGSSYELYMNADIPLAYFFYQSEEQREEYLPLFNKLGKQYRGKLNFAGLD

ASKYGKHAENLNLEEQFPLFAIHELESNLKFGPEQIPSEEFTEETVVELKESSIEEFVEK

YLNKELSPIIKSEEIPEVQESNVMKIVGKSHDEIRFDENKDVLVKYYAPWCGHCKKMAPL

YETLADIYALDEEAKEKVVIGKIDGTLNDVFGIEITGYPTLALYPAGDLSKEPIIYKGPR

TLESFLQFYCIINGPLYNEDEDNNDTSISHFYPRPSIGLRLWGPLVPASDCNWGLWLLTG

TQLTVGYYFLRKFRISIKEYFMNKPITKKIKDIPSLNRFSNESKGTIQYTKTNHAGGNLI

NSKTGQMGMGNFGGSQSFHIQRQYTKWEILMNRYKKSKAYKYVENFKRFGFLLLGTALTS

LSILEAMRLLLLDYDPWAEQAKISRDQKFYNDASKFYREGVDPSKTRIRVKDQGTGKLVD

VTNDDSLKRNIALARAGLLQNDWIFEWFGPLDLKPLTFNEFLTHIEHYEINKAALIESRT

KDSVVHALREEMKNCKAMELGLFDKNKKNRDLMMSIVESKANIGNFNANSPLGDVANQLS

SLNENNAINNDNAKNIKYNLGEHVNNLNKSNVVILPPNARHEDQMSIIDTWEANEPWTAL

GLSLDVTVKVVPYSRNEEFHKSGLGSNIKHIFPYNEKDSEFIFISEDSILSYVDASSGEV

LLRQVLDNKYTHYKLLNDSRSLGLYESKHNNLAKVLDLNTGIYSENVEAFHDLPSKELDF

KRNDVAFHFNQEDLSITLDTKVQKSLKYSFDEKHFLVGKNMDLEIKYIDVNEKGTLLKVL

IFDKYNNIYYKFDVNLTTRRLLNKWNLDNVSSNISHYTILEPIFVDIDFPEDFTIAPSDT

TNVQQLLHNYIERVKFSVNAVRNYVFVEKKIKWATFILDVFSENKSEEAVQQRNRSFGFN

KYLVAINEDNMVFGIDMNTGLTLWRFKIANKDAVIKGVVSDSNNVIEFIDEYGMVYKYTI

NDFTADGFDFEVELLPLKYFKGSKITGTRQLSTQSLLIQFSNDQDFVYFVNDIFEEKFFS

DVVIKTLESKIVSQDIKNLKTIWAYDVDIDRYNLIKVTGKQLNDKVANVAITDVEKTLFK

YINHNMIAVVLQDKQSDLLIVDILDSETGSIIQTVDIEDSKVDFESTIDVLFTENTLLVS

YKSLDPVPEQKISVIELFENLETKEINFNVVSYIYPEVIVQMALTNTKYGVSVKNILFKL

ENGQVTMVPKTLLSTIRKNEKEMSDKEKMFIQPYYQVIPVNDHYVLTNTRQLLKPKNASD

VELFSIDTNVESTTFVLDIEKYDIFINKIPTSNEFDILQENYQKYPILITVLIVLIALNF

IQPMIKKRQLKLNLAHYNTMQLSFIITSFLAFFLALAQAGELTELEIKTVKSVADCKQTA

QKGDLISVHYTGSLLSDGTVFDSSINRGQPIQFTLGVGQVIKGWDEGLIGACVGEKRDLY

IPSALGYGSRGAGNVIPPNADLLFETELVAVK

>34-9_0193

MLKQQYFISLITILLVFIKLSKSSCINSVKEFESSKQFKKETRAQISKPINEICKNSYSV

ESAAQLNRLSNECHIIQGSLTFSSYQDPIVDLGNIEVIEGNLVMENSANIVRIDCPKLNT

IKGEFKIQVLTSLTSIHLRELHTVQKIYWRVVPILSTVEFIKGVSQIEEVTISDTSLVGF

EGFSREKVKQLKIFNINNNRFMETINCDIQSVSQQLSIQSNAKEVKVSMNSLVWANNMTV

RDTSMVSSPNLQYVNNSLEFISNHFSAIDLPKLKAIGGTLRLDNNKVLNKINMNNVSDVF

GGIMITSNNNLEKIDFLKSLQQIGGGIYFNGNIEEIELPNLKLVKGSVQINSTSDQLDCS

SWTAPPSGISIIRGGKIECSSSKKQTKISLNHKGEILDTATTDLDPVDSTKVVNKNYGIT

AKTSDTLGYIPLEANNRSEKEIHSNTPDEALENSEQPEQTSEVEMRKRNSTEIEKEQPKK

PRKKRKVYSCQECRKMKTRCDFQPLLGKCHRCVVLSIPCSLAEERKEEIQQVLEAMNKNK

VKTVFDVYEKDKIQLSQAMQNGYADVTSENNIDHNNYNTNERATGDYLKLQNDVNYLMMK

MNTFENKLDMLLTHLTNGNLNKFNIPAEQNFKNENAFNDNDDVEKMNNTNSHGASIENPP

FRLLDQIEKELFGISDDYNTNRKLTEREILAKSQRAWVVARQKFMEFFYNHEELCYKLAH

AFLKKAHFWIIPGGIKTIDREYVEKHPFIVSVFAIIAMGFDENENFSTEQEILYPIVERL

LSNTLTMFDQLHDHDIEAILYISMYTISRKSKKFRQVKFDGLILSKFATDSLLNLIDFHK

IKENVDNGLFDGNDLFHLRILNSLTTLKMGYCVGYCNFSIQDKQLREFNDLTVRFPQSTF

GDEIKLGEIKLGDIVNNIFLNFDAFYRESLSLYQADNEKNVIKVQAMRSWLETYRELSSK

DGSNLLIFTYDFYYIVVFRNILSEFATKSYETFLENKDYFLSCLSTVKKYCFAILTAFLQ

LPTSLIKGCPIITLQQTVYSCLSLCDFLLCFNTKEKAKILNLCTKIYWHLNSIGEKLNEA

TQNVGIIIKTLIDSSLNQSSKQNKDGSVMNMKDLFIQMQHGSKHNSIKRKKTKNSNRNKE

NHSSISSVTSKVKGNTSSTNSVYSPSGNMSSVSSVNNDNTPTGNLSRAMNPMERNTNSNP

VYSNGNMQMPQFNFGNPLINQNVLLPDVEQFQSFEDFFQDFFQNRLKPSSQNMFQGNP

>34-9_0194

MEVNKYVYNAISDEELMSLASKNQNNYKSFENLSSKSNDYLIVNCIGRGNFGDVYQAIYL

PNVNYEYFPVEFIRKLQNFEYDEIILKFLNFINDEYFVAVKIVDLDESDEDIDILCQEIQ

FLTTLQSDDPKRQNIVKYFKTFVDHCKMWIVMEYCGGGSIYEILKHLPIKLDKHKVIEHL

REDQISYVTKEVINSLLVLHDKGSIHRDIKLANILLKDSGQIKLSDFGVSGTLNNTLKRR

ETFVGTPYWMAPEVIKNGYEDPKSGIKIEGYDEKADYWSLGITIMELVNKKPPLIDNDPM

LIVSKIPDLDPPTLKDNKAWKCSKHLINFVECCLEKNPVTRMNGHSLLKQKFIKKYESSN

DATLKHEICFVKKLKLCKSLNRLKRGLNGMDIWFRKPRYYKPTKFIEQLEEYESDKENDH

NWSFTVTSKTPNLTKKLSKTSINSSSLRKNSAENYTPLSMKESAKRKMYNDLVKPTIKVD

DLNSNLLGQNTEDESNDESMDLTSNTSEVCTKNGNVHVYNDILLPVFSDIIKRSVHPETQ

NDVVKLLKNFHDTEKNQPGFSNVFVEEISYKLNELKLEI

>34-9_0195

MRAEIIFKTIHKHLLSILKMKFSTVAFSVAAASVAFAANSSNASNSSNSSNGTTTPTGAG

AANSATFGVAAVAALAAAALF

>34-9_0196

MDMMGDIVERDPLESFADIEIKENDVSPSFSDATNNSTGFPVLSDKKTLQSWRVRFQEKK

VKDKKLAKQERKAEPVQEEKKNFDNEDMSEAERIHLENINLMMSMSSEQIENERQEIMDN

MSPKVLEKLIKRFSSTGSRSKNKDEDTSLPLFTDIDTNKYWVGGTNTMTDLPKLDDNKVN

DILNIKNTDKSDKRKIRFEEPEVIEIEEEVNIEYPEVENEDDFVTDDYQLLQKMKDTDSK

ELLKDVHFVTKKQMEEYEPLSIDDAEFEKKLKEKYFPDLPTEVDKMRWMEPIDDDSNITN

NKVIDTVADLRFDFKGNLCAPQKEVDSTKDGLHHHADNPDLKGYTLEELQNYALSTYPGQ

KCIALQTLGRIMYKVKAEGYYQLIPEIDQEQFEKLNGDITPIIEQIYGMLWAWIKHLKIV

EIIESNLGNKNMSIKNYATEAMWMLKQGEYKQKKIQEKRRAMAEEI

>34-9_0197

MSALDLNNTKQEKSQSPLSSNASILSIPIENCIKKLSIDAHTNCTDTSLFLESKQKFIDN

LGIDIGGTLAKYTYYNTQTEMVEFGFKETDDIEGLVLIIKDIIKSRKEQNPSESFWIKNI

MATGGGAHKFYDILNTNFSDLNIHKKDEMKSLIFGLNFFINLKQDEIFTYSDTQGKSYLN

KNSEKKDDEEIMLVNIGSGVSMLKLNMSNLQYERVGGSSLGGGTLWGLLTLITGISDYDE

MLRLAGNGDNSNVDMLVGDIYGTAYDAIGLKSTAIASSMAKIYKKPETDFSYGDISKSLL

YTVSNNIGQISYLQAKIHNVSKIFFAGSYIRGHLMTMHTLDYAINFWSKNEKSAFFIEHE

SYLGAMGALISE

>34-9_0198

MSVYPKTLKKTVTDIYNKAVSTGSVIKFESSSKFLKTSNSGEANLKYHLTFCDIDGQKPQ

KPEREDVVKEKFNPFLKQEPELTVLDNIMDNEYKLVLNKYPIVDEHLLLVTQKEIPQSSL

LTPNDLNAAYKLLKTLQQEFDEDEEDDESDDEDVKKSRYAVFYNCGPNSGFSIDHKHLQC

IKLPNKLKTYQDFLVQEYPEPYIPNTKREPLEYEKVAYANFNIPLPKDLSKDDEYLVMCY

VSLLQRSLSFFQQWDVEDPKERLPTSYSFIMTTDWMTIIPRSKSVAEVEFEEKKYTLGTN

ALGYLHMLLFKEEQKELYEGMLAKKDSTPVEDFLLNCGFPNNFSAAPAESDY

>34-9_0199

MAQSFTSIVKAVSYINSTPSLKKVVLPLSEGFKYLSGYRELGLKMNDLIHEENDITETAL

RRLPKDQQYDRIYRIVQAHQLELTHHILPKEKQLKAENDDNYLLPYLLEAEAEAIEREQL

DNLEVK

>34-9_0200

MSASTDSSNNPSFNKRHRHKRSFALSGDNNFDFLPSINNRKNSNSSLPEFINTGNKNGLS

NNSPVIDFDNAEKYIQQSNTTQSKKESILRHKRSESAPTEMLSFNTIEDDFTSMNGNWLD

LSNTTSEDQIKKYWDNNYSTVQQDLVNEPCNTGTSKNNDLLIEVEEEDENNSGSNNSSPL

KNRMSYGVPEKTSTNSMNNSKTVRHISNNSYRSITPSSATSNLSSSSFRLTLSTPTTAIA

SSSPLAMTSSTTNNTNTNRSIGRTKHTNNFHQRYHNFGIPYSPSASSGTANGTGSSQGSS

RVSSANSGDSGLSTDKRDIYNSLKAQEGISGLSPLASEVISKYATMGKKKSNGKMGVAKK

SSSGNLNTPFNFRSQEYDISKDLEDLKILDLANDEDEQLKLYLAEENYKKQASISTITQI

NFNNGFDSGTMLDNNISGHDRHTMLDDERKRFSKHSRQYTDTESNITLTVPGNKETKCPD

DYEKNLQAFEEESVPTVQKKQASSGSQEEVKSFISEVDNNKKKGSSQNLRSRISSSISFS

NLSLKANDVDDNKKIKKSLSIQSLSKFLRPHNDEDNTEKVEGNNKKEKRRRDTSKRQSLM

AWIFKSK

>34-9_0201

MSNEIVNSRLSILYFDKSFLYTEDHTVIEQDDDITFGNNLIIKNAKPTDVYFQERYNVKV

NNTNSNVKFTYSNANKGLEYKSNNIFKPLFLENGFNQYVFNGNNTRFDNKQDFVYNIRQF

LTKYDIKLSAFEDIFHNLIHKPDFYDSIELTIVNDKLVFQLTYPQHSFEEEKIIDLNEFS

DTGMFFTVKENTLDTNIQGMIYKLNENKFQKTNFYISLYENDFTNMDLFKLDFKQEGIHP

EISIENQVSKDIKVYNPFANDCSRYLLFNIPKQSFVNKFDNEIKFDDEHWKLVDISEEKI

DLEIPDYKIDSYDNAYYLFKKENFKQSHMNDISKFKFNLHNRYIDSAVPNQKGYSSFDFE

YFAFDHCSDPSREFIMTPFNQKSHLGGYYAQNFANPLSTNQGTYTNLLKHGIETIEIPYP

NTNNFYKIQLATWIVVIVSMFYILKKTLFYKRATQPKPTESSNDEKKANS

>34-9_0202

MGKTQKKNSKGRLDKYYYLAKEKGYRARSSFKIIQINEKYGHFLERSKVVIDLCAAPGSW

CQVASNLCPINSLIIGVDIVPIKNLPNCITFQSDITTEDCRSKLRGYMKTWKADTVLHDG

APNVGLGWVQDAFTQSHLTLQALKLACENLVVGGTFVTKIFRSKDYNKLMWVFQQLFDKV

EATKPPASRNVSAEIFVVCRGFKAPKKMDPRLLDPKEVFEELPDKAANQEMKVYNPEKKV

RKRDGYEEGDHLLYHTTDIMDFVKTEDPIEMLAGLNKFTTNEDDKDWQILKRMDFTTKEF

MSCIEDLKVLGRKDFKMILKWRKYSRDTLELDDKEEDKEEVDGVSALTEEEKITKELQDL

QEKKRLEKKREKRKKNEIKQKEIVRMQMNMLTPKDIGIEAGMDGRDHLFNLKTAEKTGQL

DDLIKGKKRMIFSKEDVITGTEIVIDDDAVLSDRDDMRDLDDLEAQMDLMYDNYKERKSE

RDSRFKAKQSRQEADDWEGFEKDNQDDEITDVEMDDDEEQEIDSDSDEAITRLISRIKGE

KGDQSLSRDARALFNDPIFDGIEADLPSNKPELDEEPKLKPTKKNKKDVAEKKTEKFEVD

SDVDMSDKDNSDFDIVPTKESSDESSDYDSDAEANEDARKKHEKDIELATVQAMTLAHEL

ALGQKTKHDLIDETYNRYSSRDRDNLPEWFVQDEREHTKINKPITKEAAMAIKERLKALN

ARPIKKVAEAKARKRMRAVTKIERLKKKAGIINDNSEKSERDKADEISKLMRKLKKKANV

KPKVTLVVAAGSNKKLAGRPKGVRGKYKMVDGVLKNESRALRRISKKNKGKK

>34-9_0203

MENNNYSENYIHTDEFPQQYIRNKENGVEGYPKLQKLKELKEQQIKDYSKKAYGCRMNVD

KMVPYLQKWVGEYSLNFDVVMIGCLSENQFLYPVLRSLPIDKLISKPGFLFIWASSNKIN

ELSRYLNQEIVAKKFRRSEELVFVPVNKDSCFYPGLSNDTDESLLDKMQWHCWMCITGTV

RRATDGRLIHCNVDTDLMLENDTPLSTAASTDNSNYESQKLSKKRDSGNNCVPSQIYQIA

ENFSTATRRLHIIPSRTGTEIPVKLRKGWVIMSPDVMLNNFKPKEYVKETQDMLNLPEDP

EIEKLRPKSPVQKGKRVMTYS

>34-9_0204

MVYHGKIKRKVVTEQEDIILLNVCINLLPCLESLGNSSYKGVFWKNVCKNFNKQTQSNRA

VRQIRNRFKSMYEYYLKLKKSNFFESHELKSENLTEEDVFFQALLECFSQIYYDERGILN

HRVQKTEKIEYKTKTMSSTLDELLSKSKDNTQNNYTTDSIVNNMNSNVPNEASFDLLDVF

YDGVTYGYKIDGDGMFNRKMQLTSVSNILTNTKYKNNK

>34-9_0205

MSFAHNRNTTNLKRKQESYKANLVPHRNNNKVADYYKKRLLNKISFWTNKDAKNNVNLAL

AAPNSNINISTSSSGNEYEDGDVEDNDIINDYEADFDQIKPKNNGHLDGQNDYFSNTPGQ

NNSPLTIESYIFKNNNNRTDNSVLNEDSELNLSQKVGNFNTLKLNKSFKDLLVRVSPFKN

STSNVYEDREDINEETDNNKKFVDFKNADFCQDLKAKEKVLTYVDEAIDDVWGRIYDSST

FAEEEIMTLYEQKMPTYISFDHDQMNKNQRRNHSSFSSTASANSKYLFENMENNTELNIS

FSDGNSRRRSTMDSMNQNRTSFFHDAFAQKWKRLEVNLKDTKTKMESLMTSKNVEDMKLF

WDLWDSIKTECIQLLEVDEDEESENLSESQYKSVIENLEHSRVYYN

>34-9_0206

MSDQQSKQEQIIPSQSILDSRWDAVIANGITKTTLGLVGGIAASVLFKRRPAFVFLGTGI

GFGMAFAEAGIRSINA

>34-9_0207

MGNKSFGKAKQVTGNNKGFKKTNDRFKSLKPTPLVNNSKFKKKQNKLKLEEINQDFKNNN

DKLEDVHQLMKTYDHQKKNNITNDNIVAKTSLPSAENIQKMSEIDLKVKKQSKKLKEKAE

KELNEQLDFISGFKL

>34-9_0208

MVSTHNKDKPWDTPDIDKWKIEDFTEADNASGLPFAEESSFMTLFPKYREQYLRSIWNDV

TKSLDSHHIACVLDLVEGSMTVKTTRKTFDPSIILKARDLIKLLARSVPFPQAVKILDAY

TACDIIKISSFTSNKDRFVKRRQRLVGPNGNTLKALELLTNCYILVQGNTVACMGPHKGL

KVLRRIIEDCMNNVHPIYHIKELMIKRELAKKPELAEEDWSRFLPMFKKRNVQRKKPSKI

KEKKQNDEKKVYTPFPPTQQPRKIDLEIESGEYFLSKREKDNKKSFERKQKQEERSIERE

EERAKHYVAPEEEEYVSAIDKEKKEKKELKEKKKQEKKEKKEKKEKKEKKRKSETNDDDV

ADEDKKKKKKAKKE

>34-9_0209

MSFISNLLKSGEKKKNTEDNFKDDSNVFLFDSDDESINSDVVSQTEPKKKRKTSLLKRIR

NAFDKSSIIESDEEVGTQPTQALSKNTENEQHAPTQPTQLPIIEKKAMEPQSLTQPTQLK

GIFDEESDIKAPSNTQPTQLKGIFDEESDIEAPSNTQPTQLKGSLENEGNMSDSHPKTQE

TQLKKIVEDDDKTKPDTIQTQPTQLNIQQTKGELSETQPTQLKAIFDDEDDEFFENMNAP

PIKDKKSTGLKAIFEESDDSLFDELPEVPGFEKSKVANLAKEDEPVQLEIEPNSDTDEYN

SDVSDFEIELESDKEAEADLEKEEIEVKKEAPLPNKTFSQEYIMLSDDEDDDEFDTIKSR

HNESAETKATSMIIQAHIARQQSIKNNENLKKNKKKGMDVLDLFAQLRKKAVKQSQKTRA

NLEIVEKLSVEDDGGSEKEEKSDQEDLSDMDNHLNVDSDYDGDEEIDDLSSNDEVIEQQE

EEIFYGKKAVAKDTVPFEKSPGSKEFSDPIEENTNMEKEFDFEKEKRRMERAKLRREALK

KESNSNVENPFLAEEAEESDSEEEDALRNFGIKDKNKEDDEKVDDVLHTSDEEMFDDDSD

IENNEEDLRQLERVQQARDEEELEKQIQKTLKGERRGIDREEGFLDDDNDFEQDDEYIAY

KKRQDAMEAKISKRMNKSKKAKINVLEGVNARISLPENKIGVLDKFTVTSTLSFLARDST

LEENGIIEEKSESDDKDDNIMRKFQTKMAFSTSPFDEPKNLEDDDDQADSLMFIKNVKKI

VKKPAIESEEMKFRLGVKTYTKNHTRNPHTKTITKGADRYSFNKNGKREVPFLGKKRKPE

SKDSLFNHDVKKHKV

>34-9_0210

MEDSFFDEDNQRNEDHDFVTNHSPNNESIKSFKAFKTLKLPSKKTRDITDFSTTTCVNET

SDDFNDDAYAQLTQNENQDTVLNVNGLDLAIRPKRNRKSYNNLSRTSVHSDSYSIFNGRN

RLSIAIFSDLDCVSIKDDATVIGVEDTSEDFEEVSSNLSEKDDEAQIYDPFADESLRPLR

RYNSHESILSKKNVLIIEPEKLEFNQRPNVEKLYLGSFTKKEEKFKRSFDYLRNGQLSYL

TSQPVTAMCNKKAITTTTNKNADSNGFCNPFMLPLVTKTKRDPLVFKTKDSLKQYLSEDN

VTPIHSLKNIKTSKVNETTIDFDNKTIKRKSSIIWDLPKKCETTVHSSFNSNNSKKENHS

YKNPFLAVAKRQASLKITRKGNAVVDNGTEVCSTKLEVDLLKDALLDL

>34-9_0211

MAKEYTIACVGKPSVGKSSTINSLTKNESLFKIGNYPFTTLTAQEGVSYIPFKCKCNKNA

NCKPTYGKCDAGKRYIPIKVLDIPGLIPEAHLNLGLGNKFLDSLRTADLLIHCIDISGTT

NAKGESTRGYDPINDVEFLINEIKLWIYNNLEKKWGSIVRRHINTNASIVETLTNQLAGY

SANMSMVQKTIDLLMHKQEDEAESKYLPPLDKWDEELILKCIECFMEIKFPMVLALNKID

HPDANKNISKLVLKYQGKYEIVLTTAITEIFLQKLHTQGFIKYESGTEFLDTFEDDPENL

KELPPAIIEKIEKIRDLILYRYGSTGIQDLLKTSAKVLDLIPVYTVKNLNNLDDADSNSY

KDAFLIKNGSKVGDVKKHLFGIEVSIGSILTTGGKRVSEEDIITKGVNDVLCFKLAPK

>34-9_0212

MLLSTKLFVFLVAAAFNFHVVAAKVSFAKWETASEISPFYGCKVAVTETAKYCETDDQYI

YGCQCDDVVAFGAMAYCAMENNVNLDHFLKYFNTECNALLVKKYDFTKQDIEDSYKNTSK

YIKTIADFPNFNYTTDILRTPVKTPQLYYSLAYRTYYDYYQNCSWGIIFGAIFLGFWLFY

FVVFGMYQVLFKYIRPTALINAEFKFKKYNYIMFSKLYNWLDFCKIPIDPNEFNLTKCIG

IGATLIIFFVGSCWNYHSFEGNFFWAKRSSQISRYVGDRTGYLCMFPLNLSIFLAARNNF

LLWCTGWNLADMIFFHKFFGLLAVVLASAHGIAYWVNAVKTDYYSESYKDDYWIAGVYAI

VIGAAIVILSNHRFVRKHFYEIFLVIHIVLAVVFVIGIWRHLNPLDLVQYIIPFIGIWSL

ERLIRLINMFVVFGGYRKNTATLYIDESNDDKNDIFIKLDINNYNKSLFKIKEGNFGFVY

LGLPFCFWQSHPFTFIKIQDSENFSIVMKVKKGITMKLYKKFLSLGVKTMDLRVCIEGPY

GHSERDFTNKHDQLCVITTGTGIAGPLGYLSSHRASKSFDGQLKPNVLHWGVKSFNIVKT

LENELKTVLSDKNLNIEINIYCKNFVVNSYVEPKKSGTDSLTSSTSELLANFKDKFNVVS

GYMNSQNCVDSIFEENNSSLIMTCGLMSVSYQVKKQYMENVIKNKSTNHISFIDESQSW

>34-9_0213

MLLIRKILFFIFAATQLQEVLAKIEFDKWDKASEMTPFYACKVATTKMAMYCKKDKIKSY

NCQCKDVVAFGAMSYCAIENNVNLDHYLYYYNQRCNALDIGKFHFTEDDVRESYSNVTKY

IKKASEVPKYNVSNDVLRTPIAYKQKTYDLAYEAYYDNRQNHSWGIIFGLIFLGFWLLYF

VLFGVYQLFFKVLRIKFLINAEFKFKKVVFVVFSKIYDILDRFHIPIDPNEFNLTKSIGI

GATLIILLVGACWNYHSMEGNYLFPKRTSQIARYIADRTGYISVFPLNLSILLASRNNFL

LWCTGWNLADMIFFHKFFGLLTVILASAHGIAYWIFCVQTDTYAAHYKKAYWRAGIYTII

AGATMVFLSNHRIVRKHFYEIFLVIHIVLAVVFIVGLWHHLNPLDLVQYVIPYIGVWSLE

RLTRLINMFIIFGGYRKNTAILYEDATSEDKNDMFIKLKISNYNKSLFNIKEGNFGFVYL

GLPFCFWQSHPFTFVKLDNSDEFSIVMKVKKGISMKLYKKFKKLGKNTMDLRVCIEGPYG

NSERNYVNKHDQLSIVTTGTGIAGPMSYLSSHRASKTHDGSLKPNVLHWGVRSPIIVKAF

EDEIKTMVNKEGLNIKINIYCRKLEISESIEPKETSNSSSQSSLDETLTKFNSKIEYHAC

YMNAKDCVNESFENHNSSVIMTCGLMAVSYQVKKQYMTNIINDKNKQHVGFVDESQSW

>34-9_0214

MVMNFLLVLTVGFMIGLSIFVLHKALTALHTEKLVITINPFETTKTIFDNAYNDVKWVFE

LVNTTSKTFSDILKPLNSLRNMDEEIITTELKQVHQKYNPMTIKGTMNILSVVKGKVFNK

NETAEVQTEIPEKKSNTRPLSQATGVSFDSMDGELTRRKIILRRDSSDKKADKNTIQRIS

SIDVAESLPKNFFQDKNSTKIAEDSTVSSNTTKKLDDNTTNI

>34-9_0215

MLRASAIIKRTKFTNTSTSAIIYKQFHKNKKLPPLPTLEDPKWNANTAVSSILYESPPVK

PIDQNKKSHVLNCLVTNEPGVLSRISGTLAARGFNIDSLVVCNTEVKELSRMTIVLKGLD

GVVEQARRQIEDLVPVYAVLDYTQSDNIQREMLLARVSLLGAEYFEDLLQHHNKGTGIDI

NESDLIKPQEQKYHPKNLPFSELTRLKNQHLANIKMLTENFGGKIVDISENNCIVEVSAK

PKRITNFLQLLKPYGLLEVARSGVMALPRTPLKTSEEEDDEDISKKINDIVDVSQLPPG

>34-9_0216

MLRKDLLVNFNTIAKRTATKRAGGGKTNNKDSAGRALGAKKVELQFVQANEVLMKQTGTK

FFGGENTYSGRDHTINSSAPGYVRFYIDPFHPKKRFVGVALSKDSVLPRGHFLPKERRFN

NIKLNPREKIEMKLINNLLESAPRFFTHPREQRKIFRIQQNMKDRIAHYDAKQTRFNNLI

KEQKVLEGLWGEDFVKTLSAEENQFVVEFLYLMQNKFEYSHNIDAAFEYTKNEIKYDNKL

QEKRSASPTDVNIKYQYIIPRYYDYSVKYNEALAKKIQETVDFNKLDLDIIKKIPSQEKE

AKKAQLIEEVSNIKPFLYTKNDANFKKITSMLSIKNALIKQVFDNTELNQLKNKLFPEVL

PSSWEGVLSTDKKDRNAIAKKMYDPDTKTFEDVLVSKFAFKDNAKDLLL

>34-9_0217

MAVQLGKTSQVHQSHYIFQQDEQVYFQLFLNNKNTINILKNGVIHQSISLASYLKEVGAL

SVCFFTGDVILVSESNEIYMINYISITQQWILLKRPLFSSSITTEITAIEHLNKKEVLIC

DILNIAVYKISYTSDEVPSLIRTWVNKNPVSPVFNITKTNSKTGQSNEYMCRKFNLLTYN

DSLVYVWKRTVIDVDNDIYSYQLSLINTDKLNSSTVTWNIWSKINNSEILYTLNDDSILR

IWQNRAEALQISLAEIGHQYMFLIKNLKDRNVIVTFGFDSENKVHYSIHEITFDYNSKKL

LLNTVKKNLNFESGVLLPETWSKSDYLEFLVSEKNHMIFFNNFDKQSTAVVDYEPERFLF

TLRSVAAGITSNQKIISSTVNTNNFYSQTVTNSNEVTMWQVENNIMHSKVCTGNFTNSIS

KIVQESSGEPLYLFSDKVVVGDKFISFEEGACLDIGVLNHDNLIVLFKDRIDVYFTDTLK

KKYSFDITDSLFSFIHENSVVVVRSEKSKTFIVKYILKTDDFQSFDIKTIEKSFSFDLDT

NVEVGKYKNGIIALKNIEHDILFYDVGEMFMLKKLTMENDSEPNDWINHCLLTALIFIDE

IINEHYPKTTFFGLNYIYKAKSNDIKIKDSILANFEKEFTTPENIDFSTGFKGIVNSAEQ

IKLDFDDLCKSLFQETKNPHDVGLYYLAMNKLPEYKLIWKYSTKKEADKINNFLLNYNSK

KCLNNGYKLLSLHRYSEACWFFLLGNDYKSCFYTILKKMHDFNLAVAVLKVFDPTGERLK

ECLQTYCLEYFKDLNKFWEYLYSGLIINNYNMEFVELDQFIVSLKDLECLLMIVKKYGTE

DLEQDVLKRITRLATLSNNDKLLIHYSIEYKSQTKIPFILKESIDTKPKAGLFKSFNEKP

KLQETLQVTNTEPKSMLDDWM

>34-9_0218

MSVEEIFENQFAYFDISVNKDFVGRIVFKLYPNKAPKACSNFFELCKRDTNGYTNTLIHR

IIKNFMIQAGDITYGNLDNINEELLGTGGESIYENSSFFEDENFMDAEEFKTKRDDYKQR

KMKLVMANHGESNTNKSQFFILTADDSSHLVGKHTVFGEVVKGLEVIRLLENVEVSEETG

FPKNLCYISQSGEFVEGMDVPFAKGCNSDILGDIYTEFPFDEFSIGEDDFAQALKVIETI

KGSGGALFKQKKYSDATFKYLKSLRYLNEYIPDIDVDKEMHLAYKAMKVTLYLNIALCYI

NNKNYELGLKFCDYILDNGVGLKPETIAKACYRKSLCLIPKFRYEDALKQLNLGLKHTPE

DQNIIKKIEYVESLVEKEKEKQKKTMSRFFE

>34-9_0219

MTANSKKNHSYDQISAIIKECNVLSTVMSKQLQTTYKGRNIFHNQLSKNQRKFSGNHQNI

KNKINLNDFKSLSNDTQGENEPLIYGFIELKTILMNIKDLNEIDSLTILQPFLLVISTSS

TSGYITNLALIAVNKFLFQLNILDSASTDYILAVRQCMVSLTHCRFEGTDQMFDDAVLLK

VLELIQKIFVSDFGHILTDSQVYDVLQTVLSLACNKKRTEILRRAAESTLSDIVTKLMHK

VNVIDPTLSDTTSNNNIIVNDTVFVDKEKKPLIDDLIGSSQKYTNLNTQENENANTSQES

LSKEITPEDQNSIEEQNISEDEVEEQPYGLPVVKDFISILVSLLSPEQSLKHNYSTKNLA

FQLLTIVIEISGRNFPKHPNLMLLLSDSICKNLAYIFKNILYTKVSLLNSALQLFNVLCS

QLGDKIELQLEFMFTLLLDFIIEDSEDSENKYANSNDTNKKHVPTQTTSLMKEMIIDSIS

LLWSIPVSSNTHQGNMNLFTSLFIRYDCRLDRSDLAVFLMNKMCKLSLPEMANISTEAVP

PTCLNGILSFIEYLYDSIADNGGYTNDLNDLECEDLQNKMKKNQFIKATESFNNKPKNGI

PKFIEYGFIEDNDDEKIMSFLFEQSNSLNKKTLGEYLGTLENVDKLKIFIEYFNFTGLKV

DEALRVMLTKFRLPGESQQIERVVDAFSHKYVKDQDYESKENVTTSSEEIPEDDYDSMRP

DTDTILVLSYSIIMLNTDLHNPQIKKHMSFIDYSLNLKGCYNGENFPVWYLNKIYESIKF

NEIVMPEEHHGNELWFDDKWSNIITSLNTFNGFQKEYSNKELLRYNRELFTEMAEKILPV

LFEIFHIASDDYISSMILSALDKCATLLENFGLKDLYNSMIYQLAVSTRLVDLELSDNLL

KIKDIIQVKYQYQSVIKYEEDFDDVPFCEIINEEEGKSLTVSKKAILLGKNLKAQLSLIL

LFRMIKLNTHEDFVDIEIWKCILRVLFVLYESKMIDPDIFRAFQKYNKLPRLPKPHAYNT

INKKSHEEGKKNKSGFFTAVASYLRNDDLEPTDEEIEIAEIAFNTIEAAEVEETIRENQN

NYKDMVFLNLIFETLNKISNSVENESLRLFLLEILVALSINLETKDNKINRIYELLDIKH

LTNNSKSFTLRVLIYKLEILKIYPSNIKNLIDELLTSNKIYDDDFYGVKSIGTEALKKIL

FIVNEDDILIMKHENFWRLIRKFSTFSKNCGMIYDFLSKMHDLKKFNHIGSNNYMLLLGL

LDEISSKGSVGVKMEHEEDKAIVEISKKTIKLTEDLIQYIDFEANDDTLESSEQLFALIQ

AITHQCMNPYKPIREYAQSVLNNIINSKDTQLEYKKHLELNNFANSAISPLLDSLDDIDE

KVNILVILKTFYLDQYLENKHLDHTNYPTILHIFNKHNSGNDAVEKLLQDLITEKNEMSA

>34-9_0220

MSTLSYEARSEAHSAPVVKKLLNIMVEKKTNLCASLDVNTTAELLDLVEKLGPHICLLKT

HIDIISDFSMEHTIAPLKALQKKHNFLIFEDRKFADIGNTVKLQYSSGVYRIVEWSDITN

AHGVTGEGIVKGLKEATIGIDEPRGLLMLAELSSKGSLAYGEYTKKTVEIAKSDKEFVVG

FIAQKDMGGRDEGFDWLIMTPGVGLDDKGDALGQQYRTVNEVVSTGSDIIIVGRGLYGKG

RDPVTEALRYKEAGWNAYLKRIVEQKQMKDFMRLYSNLVEKCFSDCVEDFTSSKLNNREL

TCISKCAEKFLKHSERVGKRFQEQNALMNQNLR

>34-9_0221

MANLKKVIDLIANNTHEANKLINIPAQGRIISKKQFKKVMFYNITDGANTIQITDTNGLL

KYKNLKVDEVIKLNNYIIPSREDNKVKNEIYINENSILEVTGPIDPEDYVFQSKQKKTLS

YLRNFPQYKFQTAYLSNLLKFRSFVEMKILNHMVEEGFVKVAPPLITENDCENAGETFSV

ISTVKKHFFKPTKTQKENKVGGQSNLTVSTQLHLEVLNRSIQKVFTMTPCFRAEKSDTNR

HLSEFWMLEAELSYVDKVEEVCDVTEKMIRSVISDLLKDNRGLSFISTYEPFEDTYDHIE

QGDEAQVTRPQRLLKRWMQLDQSEWVRLDYSEAIELIQKQIQEKDGFFINESITWGDEIC

TEHEKWLATEHFKSPVFVLNYPKKCKPFYMKKQKDKEMRDTVANFDLLFPEIGEIVGGSL

REDSYKLLTGEMQERNMNMAHMKWYTDLRLNGSVPHGGFGLGFERLIVHLYGLKNIKDAI

PFYRSYNNTLKV

>34-9_0222

MKFHSALKESISVPTSNELFSEVPGDINFTSIDNLLYNQLVQAINTILQGLEFVNKSSTD

VDFQLSNNEKASIRNGIDKCLKILYEDCYNYYKHSAKEIFLQNANKVIQGFIQHTKLDME

PQASNDLNKNEKEIYAKIKQSELIKTWDLLNLFLANNISKDLLLKDEDILEGFDGTKLDF

GMFVLDENQYIDWNRSQELWLKSSKTLITIENYLKIIDEPSCIIKDIDTVDNPEGDDDDL

LISEGKIQLVCKVSLSEFDKPMKSRCGHTFDDNSIRSAFNYSSARVSRQCLEDACSVKLT

YPTDFKLDETMIFRVACSRINNKK

>34-9_0223

MSSKIVSDIVKKTPEYIISVQRLGKSHSPEFNIVVKSTETKQRNKTMPIEVLGYFRPKPI

YDAETKTFIKKVELDFDRCKYWVSIGSELTKPVFDLFDKAGFGAFGKNAEDRFYLKKADP

KNGQIVREAEESIKKINLDKWRKEFVA

>34-9_0224

MSEKYSQLLKGLKKSEILDVINALQTKVTLDDDYKTPNAHAKKEILVESALSLISDNESN

EDWNTIVKEIPYLNELEVSEKSESEEEEQKEEKSESEEEEETGCACGNNPEECECEDCNC

EGCIKNDSCEDSCCFSYEPTLKERIQWKIYDFRWFLLLQNETLKSFCTSAAGLFKISTSL

ELIILFSYVYENINTLVYCPKITSHISNPHVLLVLKFLEILAGYIGIFVFVPFVISYYFN

FLPEPIVEEDDDDELYDSESDEEYDDYVAPSDEDEEVGEVEEIEEEDASQEGEDLNDALV

ENGDEYDYEDLDEEYLGDSDIEDDYDYEDYPNLTLVDEPEKDQYDPFTYSLAKIAVSYIC

LRYGVGLCEFNNLVVLSKLCEVVIGFGIVGALIGLYN

>34-9_0225

MQEDNSSVSGENVHIDDYDDYDAMNDQNEIDNYSIIGQNISKLKRVLGWFGFSNSDNFYE

IGDWSIDDHVELYNLDGDGNVSNNEGEDVVRIMKREKMKKLRRILMKLIGLIAVGFLFFY

IVANLVIVNKEYSEIKEAHESVSTAEAATTFADVEDNIFKKPFPTSTSYTSLTKTASSSE

SETSSTSKSTSSETSSKTTSKTTPSSTSKTTSKGKATAKTTASTTTHTKATTKTTASATT

QTKA

>34-9_0226

MNTPSDDILMSSENKVTKKTRNRKTRNRTPLSCSVCRSRKVKCDKNKPHCNSCVNTGYKH

LCQYLSPEWSKDGYNHIFQEKELTDLKDKIKYLEDMLATYQHKEQSTKLQIKNKELEYTS

PSSIKFKHDHGKKYNNDEINVSKRFESLHINPKTNSFTYLGSTHYLSILKGDPYLKLLWE

HVFKIREKLMEYQVYQRNVSLKKQMKKSFNNPNVPDQLEKKCPVNHKQMMQESDVKKCPV

NDQEIMQNANIKKCPVSHSSAPDNLPKGKCPVAHMGATSAEPNQNFRQGRCPVSHENLNQ

RRSVSPLSREKSSETSQGSQNDFLQDLLQNLPEPYTIKDNLDLFFKETYPEIPILEKKLF

YTEILHILGIADFLNSSKDDYNLNLDNLFKCSQIGILLIILRLGCVNNSQDMNIDLKLGL

DDDFVKEHLEFHLPTDNNFNISPESAINNKPIEYNKKKSKFNIDPKFIHAIYENFIKNNG

NKEPSTTDGYYGNGIVFTNLSVIHFLIFYKWYLGICPENDTVDTLTNVNSTNKKSELLLA

EIVQLSFDSGIHRDPDNFTNLTTNILNKSNEYVNREDKDLKIVAKTISQSIQIFKNQWRK

TWFYLVSMDINQCLNNGTPRLLRKLGSFSDVKLPYNNALRDEGENPKFENDSNFKNSGET

DTIKSNSENPETKSNTNSIEYLLLNQQVDYCFGNNLGEIIIGKNTKLFYQIDLVMIAISE

LVLNSQPGFKAKKWQMDFVVDVLEKILENKNNEDIIKKCINLLLDNNLLDPLEAYAIKEY

GFENITPAMCKTDSQKQRFSEDTGYNLPSLKEIFSTVDNKRKNDSKSNSPKHNYNGHDLT

DHGKKLNIPQENTTKCLFFKKHLQIQMMLYNVNYILFTSYEPRIQNKNVDEDLMDLAVYY

CQQSLKYSTEIFKTSLIFFHNLNANKIFKTQMKIVLIPYCLDACYRSMQFMICLILRQKV

GSLLKDLNNVFSKKISGIQNNTSSDSESDSDSFTKSFRKKKRSEFNKMIFENNTLSLQEP

YIAELLMSRMDIFHNLTRILAVKYKYSLKLSRSTGFFLTLLRPSSSSNKSLLKQNTGNNH

FNSRLSVCPVSNNNSFSSLTQSFKNIPSLIIQANSDQLSRCPVYQDIGFNNKLPSLTSLT

MPMNGSIGSIREPSISSFNYQPITSKINGNISSVRAQEDSSINFKDASNSRSNSLLSIKE

APLIVKKNLLESNILPLPDNKTTPRNSITQQRIENETISTSSTDYIPDFEEFLKQVGLSP

SVNPIGNNGANGMLPFFENTPDGIDSLDMNSLISPNNIMQASLNFLIPSDTPTEISNDDV

LVKREDSNQSVWDINEDLIN

>34-9_0227

MGNCLSCFKSTTHESTDRDFENQRLINPNEGDNNYGANKEIDEENNNIHQNNMSLPINED

EREAVSLLLQYLEDKDRYDFYTGGPLEALTTLSYSENINLRRSAALAFAEITEKFVRAVP

KEVIEPILALLTDEKNSLDEQIKIATCAALGNLAVNDNNKKIIVSMGCLNPLVEQMLSDD

VELQCNAVGCITNLATIDENKFKIAKSGALIPLIQLAKSNHTRVQRNSTGALLNMTHSVE

TRRELINVGAIPVLIGLLSSTDADVQYYCTTALSNIAVDEENREKMQATEPDLVPSLIKL

MDSKASRVKCQAILALRNLASDREYQLSIVRFGGLSHLYNLIQSGSIPVVLASVACMRNI

SIHPQNEALIIKTGFLEPLIYLLNFKESEEIQCHAISALRNLAAASPKTRLMFFKLGCVA

ECCKIANSSPESVQSEISATFAILALDEVTKLKLLKETNILEVLIPMTFISNPEVSGNAA

ATLANLCSRLNVKPISEDAEYEVDNEFDYYEKVLSAWENPQDGIVGFLRRFLTSEFITFE

HIALWTILQLADSEDERFITLIKENVDVINSVKKLLKQNYYITVNQNEEGRNKEDDDENK

YDAALELYGLSKQILEVLDIQS

>34-9_0228

MVGIKENFKVENVVVVGSGISGFTTASVLLDKYPQIKKLIIVGREIINDPAYTSLQHEFT

SWAGGANHMSFAADDDYKQQERDLITYKKFMYLGKSFKNSGIRIMKNKLIMFKTSNTPWY

IKDHACEGICTISDAELEYRGLDPNIYKGFEYLTCTITPYIYLGFLRNKLEKTEKVAIKK

CPKTLFSFADVQDFVGFKPDLIVNCTGLGARSILSTYSCEKAEVEENIISYKGQICVINK

DLPFQLTVEDLPSDEYVCDYVKKIEGAYQFSHVFPRSDGYAIVGGISCPGVYDNTKDKDV

STKLIENITKFVPELVENGPIEIIFDYVALRPGRKGGVRLEYKKYDDYNVIHNYGIGGAG

FQASVGLALEVSELVNINIMNNKSNAVDIQTSNLNEETPADDYSKYVDYIIKLFLKDNTT

VEGTIVRYGSESISLKVKEKKAKSKTYERGLITDLKVINIPKVVNKKAKKASPAKAKSAS

SNNNKNESTFGTLDIDTIKKTDFNFESNNEKYDYNRAELKNLNDDKLKTNDEEKNDLDLN

PKQSNSKGQKAIKNSKQKPKSEESPKPDSNQLLSLLQKSSTKEVSEPVQDIKKEKSKKKK

SKQKKESKVAESDKETSQEAIDINSLPITKAINLSHLCGRINLESSRQKKLKKKLKKKKT

TGKKDDDVEEEDVLEPVEKNDAELKDASEPAKQERLTEKKRKEVEAKKKNKTTLKARDMS

SDDSSNTSSSASDIYTTDNSYESRVNSLSLSSDESESSNNLSLISNEDTDSSSDDYSLSN

SKLDMSLNIDSDSSSDAEITQSIKEDNELVNVPASTNEEKSVEPAKKNPKKNKNRNKKQQ

AKNKKNKDAQSKKSPIEEEDVVRLLLEKAMSAGLISGDVLKKQDFLADKKPEAPKKKANN

RKKQQNNKKKTPAQPTDTTEVNVNDKLEKVISETLISTTTVSTTKPKNKGKQKKSKKSTK

ESSLEPTPSNPDQ

>34-9_0229

MPFSHHSHSKTFISHGSGPLDEVITNVLKKKFHTFCFTEHMPRLSSKYLYPEEIADKQDT

SFLKNQFEAYLKTVLDVKANEKNLGTNFIIGTECECIDTKHIKYAKEMFDSIYKGKLQFL

LGSVHHVNSLDIDYDIETYDKSILASSNSFLQFLKTYFGLQYEMLTTLNPKIIGHFDLPS

LFLNQTDLVINKTTGKIIDDYMESKKDYAIRIKDYSIDDFVFDIYKEELLPLVEKNFKYI

IENNLALEINTSGLRKGLKYPYPSIGLAKHFKNLGGKFVLSDDSHGLNQLATNYKKCKDE

YINGELKLDTIYYLAEVEEQSLEDGLPKVDFKSMTIEEFNNQQFWNL

>34-9_0230

MSLEGSFAKLTVDDSAAASTPLKALVFKSKTNAGAKPIVVVALQETKTPSNLVAAVTESK

EPRLAKNEYFDQHFKIDDATKFNLSHLENAESEFYLVLDSSISTIAEETIFSTNNETIKF

TAKSLTSYITSLAKADLKTVDFSKVPEVSPAAKKAPAKKAPAKKETNPDAKLIGVTVKKA

DDFSAWYQQILTKGEMLDFYDVSGCYILRPASYAIWEQIQKWFDAKIKKLGVQNTYFPMF

VSSKVLEREKDHIEGFAPEVAWVTKAGQSDLEEPIAIRPTSETVMYPYFAKWIQSHRDLP

LKVNQWNNVVRWEFKHPQPFIRTREFLWQEGHTAFANAEDAQEEVLQILDFYAGVYKDLL

AVPVIKGKKTEKEKFAGGVWTTTCEGYIPQTGRGIQGATSHYLGQNFSKMFNFSVESPLG

KDHPKLFAYQNSWGLSTRSIGVMVMIHSDDKGLVIPPRVSQYQAVIVPVGLTNKTTEEEK

NLILDTAASIDKRLKVEDIRSIADFRENYTPGWKFANWELKGVPLRIEVGPRDLKANQVT

IVRRNDGKKTTVALDQLEAEISKLLEEMHWDLYNAAKEKYDSHRVIVEEWKDFVPALNGK

NVVLSPWCGVEECEEDIKKASAKKNDGEEFAEDDKAPSMGAKSLCIPFEQPELKEDAKCV

KCGAKAVNYTMFGDLTKLQ

>34-9_0231

MNYSTIPSNTEKMLQFYKSKYEKYNKRKYYKYYVFGSIITLGFVGYNTSETVNATLRYGY

MSIRRIYNVTSALTKCIYYYTMTISKREGMSIEDYSHLLKKTHKKAADVALDSIQQNGEW

TNTMIPLQQECTKSSLEDIDLMFKKDLGKSINEVFVEFDPEPIGVASLAQVHKAKIYNPE

TGEIQEVAVKCQHPTLSTLVPLDVMLTSYIFQVLDWWFPEYSLVWLSEELRESIFVELDF

NNERKNAEKTANHFAKKNFDFLTIPKVFDAHKRFIIMEFLTGSRLDAFDYEGKNISRSKV

SNELGKMVNEMVFDNSVGVHTDIHGGNIAIKYNPKKKDNFEVILYDHGLYRYPTIETRLV

YSKFWLSLLNRDIEGMKKYLIILGHINEDEFPLLAACLTGRSINTIVNEDLLNDTNDLTL

RENERNEMRERFLSANSQGGEIFLQLMSILSKIPSVVLLLLKANDLNRHLQESVLSHDDS

SKKEYVLDTLRSYLMIGSYASKNVFENDTQSSLLKRSIIALKWSFRLFAFDCYIKLHNIK

SWFYNKVSIFI

>34-9_0232

MPDNTDSFVKKLIDSGKEQIDTATKKEYTSSFDKWIKSPSSKIYDYATGKFYRSPSGDAN

TYVKNFLDSKEPKENSGSFFVSGIKSSISGTGNILKSSFNYVSSWANSIVRRSSVSVSET

NGKIIGASSGTVADKINPAVSYVTDLSKDAYGKAGSAFSASKDYLKGINSDGSLSDGVNA

KVNSTFSFISGFSKDLYSKSGNYLSFFKDYLKSIDTDNGLNFKVKSSYSYLKDLSKDVFD

KTGSVLTSGKESIVKKSTNAISDGKKFTESSEKSLKIGSGAAAGSTAAFAVKDAVVDHVS

NPIVESIPKADVVNNAIAVSGQELLGSDNLVVFDQQSPTVKSSSGLLKGFNYTKYLLAST

GSSINQNKLLYSSIILAGLLINETGVRYAKAKANAQYNVLSYQDMNKNIVLVLGSMLDPI

TKHEVEDLLNRGFIVYVSSVDAKNSPFPEDFLTSKGNDVKLASSLDETNFPKVAHRKNSD

ILSDISSEDDDRSSVESSYKKISDSDWILSSTQCFSNQNASQDFTRLNYITNKPDDIKSL

AKLIKEKNWNLAAIVFSYDNVSNNIRSSSSDFKDIILNNMMEMISTLIKASQIWPKTKIV

LFNNSLSKINEITTTKKNKKNQEPSLQVLCNNLVKTVYDTLTQSKHEKVYLINIGILKDA

EGHILNYKNLSKKLSKHGKNIGSNLFTEFLNPVQNIIMDITTSGIFYDMSSKNVISCGSL

TALNDNLKICLNESIILRLILFSTQFITLSSNYKNLLCKSVTVKCSTVKDFITKNKN

>34-9_0233

MQNNKESFARNSPIGHLLNASKMAFLPNTDHKSDEQTVKRKRNRKINSCKFCYRRHMKCN

KEKPFCDVCIERDADTCEYYDENEKPSPTSPISKKRKNPKKSEVLKLVLGDRIALEDSGY

SHITDLNILPNPLTANPTFTIKDERTLFFGPTCFRSSLYKVESDSGALHNVFKVWNVFKK

EKDAMRKTLKFSLAQESKLLETGTNENLQFSNIINVIPKTKEEIKYYISLYFKSPLHDSI

TLLSEDSVMRYIDEVFLVNAATNTISEITYTNKRNYYKAGIVLYILGLTYFDTNYPDIVV

SFFMFLQGHSTGKLMFIERVQFLTLKCLFSVLNGFTGGDFSHIINLIDTMCTSLVEFQLN

KFNAVKIYENNIALQHVKTALSGDYTLLSNLFFTGLFLDVICAYQNGRPLFIGFDHYPNE

WLLLEDVNQPTDMSKKQIHDKHHMIIMKKFLYYSRALISEIYKPTGLPKINKHVNNLIDF

IHNDLNLNEMCYGDTIPSDEYMLQEKIFQSFIASFGTDLLLSVLSMRKDYKYDEQILEEL

SDTAANMLCNQILFYSLIVGQIDIYLTKCIHERQLLFEEKNKYLMLENNSKTLGCDKVLF

YMSFSPRYCNTMRSFVEFFKVLLSIPLSDPLIEGTFEYMDELTKEEELYLKTIDSFVYSD

SKEQNSDNLRQYVRDINLKQEIIKKFNHTSSRNEYHLPKIYKKFHPDMIISINKDLSKFK

FTSLQLYGWFVNLHNEQNALDRKLKDQTEQFKLQVAHLQSKQKFARKSFSLFLSQIINEK

TKRGKSLKSSLLKKSLIKPKPILKVPVDDGSTVNNQHSNSAFNKTSSSANASPLVTPVGG

SSRSNSFCFMAKTPGLNSPISDFSQIQGGILNNTSASPFLGWDMNFIDFSSKMNWFLDPL

LPQPSYNEAEINFMIQSPKPEESNNKEQKRVPDKK

>34-9_0234

MGVLTNSKSSTSVASLNKQTHPGLSKNHSTLSLFGHKNQSHANLQSNSATSLAKSHNKSS

LSPVASSAQISGKQNGELILFDIRIDKTNDTSYTTPIRDSSNFLSSFSQTDENYYQDIII

IKGTGQDEVNSEGVSLSGKIVLSVSQPIPIKKLNLRLVGEVKMRIPVGENGLERYSTFDK

RIYEKLWDYTYFKQFLKFEEKRSDSNLSIPESSSKQRPHLAHKRTKSGANLKRLASFSSL

SFSTTSLSALSQTGSSGQDVLLPGNYELPFSLITPPNIPESLDLKECFVNYSFSCSIERP

SKKTDLILKKKLKIIRTLTPDSVELFETALISNTWPNKIEYVLSTPAKSNIIGSLIPLRL

ELFPLSKKLRLGSIKVQLVEQISVFSPVIGTKTFERVCAKKKVSDPLRSCRYYDEKFVSE

YPDNIIPSENEVVEDRGEFLDKWDVMIYLETPSSLSKVAHDCVIGNFVKIRHKVKFVVGL

INPDDHVSELRAALPLQLFVSPFAPVTAKSYYIDPLFNRRSNPNFQEDKDMEDDIYLFDE

SKNVLVDSVLKAYKVASSEQLNNLGILPKGGNPNIDFINGIRKSDTHLHTNGENMEDPMR

IRNSHVLLPKLKKDTAERDIFEYSQKHSSVVIADMMSPPDYNKRVYDTLLTGKKDEGSYE

QNVSLPAENETNLLKGKSNSMSNLDMRRLQYGVSTISINNESNNLDDVLEENARETKERN

KSLERIPSYYDAMISLNKLHNSSVEVRDLPPIYNPEWDAEYEEHNNITKLPKSRGNSTDN

LSQNNSYSDLASLGRNQVRNHRVARFQLGVSMTPINTGGEGSSIPSQIPFDAKLKVVIEF

LSLNMFKSAFTTYNYYLKTAPFATNFLTTGFLFGAGDVIAQYILPSEHTGNTEEIYDYKR

TARAVFYGSVIFSPIGSRWMYFLQKRVHWPGKSMSNSGLNTISRVLVDQIFFSPVSLVFY

FSTMSILEHGFNTDIIKRKLQNSAWSTLLTNWSVWPFVQVVNFNYIKVEHRLLVINVIAI

FWNTYLSFKNSNG

>34-9_0235

MSNTADEEILIPKPITDWIIKILTNLYNDKNTSNNTGIDMQKMIFFDLLHLLQVCKNPDN

KWNLKFKMKTKKALNNDSGLVELMICVYGPLVMSTDTGDVKLFVDIFIPRHETYKISRAP

IVLIDFEQTLSNNLNNDTLKYWLKDEVKFQEDYVATDQWMALDFKKRNLVNLAEKVIQFS

QTSLSKFEEEFSKKPKTLQKNLLNGKHKTDKVPEITKEIANINILDDVNLENELKQKMKE

NKEHLLLALQNKVDNIHNTAIPSINKHNEKKQKQLQEFIQYYEDALESYKLRFQFARSVE

EQRVSHEDLDLIPLLKQSKPLSKLGLTEIVDIANINFDSQYENWYIERSSKLDAYKQLYG

DLKEDFVKASLHDTKDKTSADLIDFNTSSGSNKDYENLKSQLKIMENLAADEFDIRFDLL

NYDKKI

>34-9_0236

MAQEEFIITPNTAGGASNSAADWPLLLKNYDKLIVRTNHYTPIPQGSAPLNRDLKSYVSS

GVINLDKPSNPSSHEVVAWVKRILRCEKTGHSGTLDPKVTGCLIVCIDRATRLVKSQQGA

GKEYVCIVRLHEALENGEKDLNKALENLTGALFQRPPLISAVKRQLRVRNIYDSKLIEFD

DKRNLGVFWASCEAGTYMRTLCVHLGMLLGVGGHMQELRRVRSGALSEADNLVTLHDVMD

AQWVYDNTRDESYLRKIIQPLETLLVGYKRIVVKDSAVNAICYGAKLMIPGLLRYEEGIE

QYDEVVLITTKGEAIAVAIAQMSTVDLASCDHGIVAKVKRCIMERDLYPRRWGMGDVASA

KKQMKKDGKLDKFGRFNEKTPEDWKKSYKPLEDEAPEDETKKSEEKTEEKAEELKKEIES

RDAEKSEEKKEKKEKKDKKEKKEKKEKKEKKDKKRKAEDDADAESDKKSKKAKKSKD

>34-9_0237

MVKETKLYDLLGCSPSASEQELKKGYRKAALKYHPDKPTGDADKFKEIGEAYEILSDKDK

REVYDQYGLEAARGGANSMPGGGNPFGGAGGNPFGGFGGGGHGGHSFSDEDAFNIFSQFF

GGGSGGNPFGGAGGAGGHPFGGAGGNPFGGSSGMPGGFSFDDGQSRQRSAPEEETYQINL

PVSLEDLFVGKKKSFNIGRKNIRGEKEMKQFDIQLRPGWKAGTKLTFKNEGDINPATGRR

KNIQFVIQEKANDKFQRVDDDLIYYLPVTLKESLLGFEKQIETIDGRRILIKKVNPIQRE

EENRYPGQGMSMKGDINKRGTLIVKYKVDCPLHLTPEQREAISRVF

>34-9_0238

MSVLLVSGGYDHQICFWEALTGVCSRTIAHPDSQINKLRISHSKEILAAAGNPNIRLYNI

NRGEVPQQKQNKSSGASNTNEVLTTLEGHKSNVTSIQFQQDDKWLVSSGEDGTIKVWDIR

APNVQRNYKTNCPINEVVIHPNQGELISCDADGRIKIWDLSYNKCSATIVPEDADNSLQS

LSVANDGSVLIAGNKKGHCYVWDMPNHTDATLLSPLAKFRAHTTFITKLLISQDSKHLAT

CSADGSARVWDICDLNNIILKQTLDGHKRWVWDCSFSADSAYLVTASTDHFVRLWDLSSN

EIVRQYSGHSKGVTCVALNDI

>34-9_0239

MAPINKPVTTRVNGIVMETNPEILLKKRRRAEETRLEKQQQAAKKIQKQAKQKANKQAKG

KFIRAETIVATTLATEREKERIKRINKVEHLKSQNKTDHLASTVDYFLKVTENPDYDEES

DEADNEELLQEKIKYSGEESLLFVLRIKGPVSVKIPHKPFTVLQLLRLEELNTGVFLKLN

DKTFPLLKIIAPYVVCGHPSLKSIRSLIQKRSTVIHKKDNIEGEDEEEEKEVILNDNNIV

EDKLGEYGIVCIDDIIHEISTLSEYFSPCNFFLQPFKLSREVSGFGAISKLRKLESKKKM

QGLLSGLSNSGTAPVIQVDIDAFIEKLN

>34-9_0240

MSLFDVCKKSLASSLHHNSHCFYFKSKQINVLENPTDFYTHLKDKIKTSQNRIFIASLYL

GKKEQELINCLSDAMKKNTNLKLYFIVDGLRGTRESPNPCSASLLASLVQKYGNRVDVRC

YRAPQYFRWYNKLLPSRINEGVGLQHMKIYGFDDEVILSGANLSNDYFTDRQDRYYIFKN

NKFFTAYYFKIHQLVSKLSYKVVYRDTAAKFELLWPVSNISCDPKHNRLKFVQESKESIK

SFMESPVDNLSLEDNHDEYPSYVYPLTQLSPFFKNSHEDASTEKKTLMKLFEDLKSSKTG

FNWKFTTGYFNIERGLRDLLIETSANENVSGKIITASAKANGFYLSKGVSGLLPDAYHHL

QYKFLKLAKSKNSNISIDEWAKGQVNTPNGWSYHAKGIWILPKNNDAMPILTTIGSSNYT

TRAYTLDIESDCVILTKDKDLRQQIQGELDNLYKNVHPVKFEDFAEKPEKKVKLHVKLAT

KVMEKKL

>34-9_0241

MNLLKPISSAPPSTGALQLINSINRFSYILRLLAFLIQLRFLRQRRSINAFAIDSVILCL

SNELMSFLLYGVQYKILKIPNEANKLRYPLLFKGNNTFPRISSFTLLINLVIVVTLLLTA

KQYHRLIKTENIFQGVSKTMCIYITTLISTAFIVKIISTHENNLKYNFNNCDYYELVDLM

KNYLLDPVVLFPQVSLSFMSQSCTGVSELFIYLISASNLISIFSSWLFFKDEKLYMGWYL

IDINTSSLFSSIVQLLCCFLIILQMVFAYKNHKANVPLKIDAIEEKKLRNE

>34-9_0242

MNLDQEREGDNFVEKYIHLVKSTYQTYLDKTTPHIKYRWTVFGVVFFLFFVRIVSVQGFY

VVCYGYCIFLLNQFLLFLSPKFDMTLQQEQSNDALEAGEEEEYNDPGLYKKSVTEKEFRP

FIRKLPEFKFWNKAVTGVVICLLLTFFSVFDLPVFWPILVIYFILLTFLTMKAQIQHMVK

YKYIPFDIGKKRYRNKAKLSTNNTTMSV

>34-9_0243

MRLISESGFEDKGKKLVLKAEEKEDLFALYNIINVDDELLFRKQVSHKGEKKGEGARKVQ

VQTVKIRVVSSEFEPWNESLRYKGVTVEPDETWNNPDLSGGMFFAFEIDYKYSFTLFKYE

YTPFCAKTIKDACNIDLKTDIGAVVLQEGVAHVCAVTPYQTSLKSKITTSLAKKKRGIDI

MKNDSKLDKFYHQIIENMTRHFDFNKLKLILLCSPGFFAQNLYNNLIAYASEKKLVDIIK

NKSKIVVAHCSTGYIQSISEVMKNPSYKQLLSETKTFNEAQIFDDFMDHLNKEDFKAWYG

LKEIQKANDIAAIDTLMISDSLLRSDYLDERQLYSALTSAVERSGGKVVVFSSMNQTGEE

LNRLTGVACILKYPVYDLDETENEDESDDSDEESD

>34-9_0244

MFANRFFSTLTKTKVPGFVFDIDGVLVQGSRAIPQARTALRLLNKNKIPFVFLTNSGGLL

EEEKTAQVLTKLGMLGEIPNVKTILSHTPYKAFKDEYKTVFTVGPIGSSRVLKNYGFKNV

YEAADLIGKYPGIAPFNMIDKRSEKYVSKNGEIPASGLKFDAVMVLSDPRDWSADLQIVS

DILTTDKGRLGTTENKELSKSIPIYWSQRDFQWKTEYSLPRFALGAFREILYKVIAIIKS

PTLVTLPKDLIKTEEEVLKTKAIKEFEIKKFIDERVMGKPEKIAYEFAEKELNVEFNNIY

KNDDKFELGDVYMVGDNPLSDIIGGKNYGWKTCLVKTGVYNDEYDLQVEPTNIVDNVEDA

VVTGLKQSGFEIEKS

>34-9_0245

MSASIEQNSTFTQNSSVTNNNPVENSFITPESYVGFETITSQIENKLVRRGFQFNIMCVG

YSGLGKSSFINTLFSSYLKEGAKADDGSLEEHIAPKTTEIKTKSFVLKEGKVKLNVNLID

TPGFGDQINNKKQWEPIIKHIKEQYSLYLRKELTANRERSIKDSRVHCVLYFLPPNLNKG

LSELDIITLKKLSEISNVIPVISKADTLTKSELQQYKKVIQKEFELLHLNLYPHDLSDIF

SDEDDEDESQKELAKLVPFAIISSNDVLDGSKIRKNDKTEDTIKVEDPNVCEFVFLREFL

IRSNLQDLIETTGIVHYENFRAKQLIALKESSNKK

>34-9_0246

MFSATRPRLSQLSAIRINNSQSLIINHGWLLNITAESNNIWRKYNEFRSGLVDLFKNLGI

WLAVPKQRVSYSKKRQRQLDLQKRTKLIPHLEGCPICGNFKRKHTLCMNCFNKAKDFLFG

FIKKEKPASKQDYLEPEAKSLLYSPAKQVKNDYFYNKKQLEIDEKIEHKRNIKETLPFPF

SKK

>34-9_0247

MSDTSVLKKQKLNDKGSSPNDSLKIDLSSFRANTNTFLSTTKHPLGILPDGNYLMSIRGS

NEKKIKHAEDVQKNLGHLSMWSYEQIIEFVKNYVDDPQTLKSLQHSSRLMYGLLFEDDIW

KNCYTKEYGKLEEEQKSKEIKNTIVPFKELGASDTWRGTWRKTLLNVPEEEEEAMIKSQE

LIFSDFIFRPYQNRHINYKKTFKKILELEKTNFENCNNGNLLFSIDRFVEDEFYKENNFA

TKFHNKPFILQNTLACKPKRINKIEDLLETFTPESKFRQEVVEWSLKEYFKYYHANKDES

PLYLFDCNKDLLSKLKEYFVKPNYSELDFFNLFEETRPDHLWIIAGPGNTGSTFHKDPNS

TSAWNQLLSGLKLWIMLPPDVSPPGVIADSEEENVTAPLSLSEWVNAGFFNDCLKLCEKN

TSEKQYCLIGCTYPGETIYVPSNWWHSVINIEASVAMTGNFVPKENLHRVLNFFKNRKLQ

ISGFHLKNLIVSMADFHSKNLNAYKATTENMDAIIGRFVNFFESGKSVSLLKDIDDEDCG

VLEDEFNSILNEIPIYEYFVLLIDSDPKLGLHLQDSMKKLQELEKQQNSGQQAKVRESEM

WAKLIQHIEVAQKGANSGFAFNFADESSDDD

>34-9_0248

MSINNKENIRRGSSSISSIAFTNSPATFSMGNHVLINKKISTYISSLSVDDETPSSNNKV

IVILKDNSKLQDKGCLYEEYRSLEEIYIKKGYTVIIPNIQADNVNVEDIRNFLKYIRDNF

PKEFISLLSADECCNNVLMAMGLEKSLIDCLYFINPIKLQLGAFNNINSNIPVMMHFNNE

KVPMSIRYVIESKLKELNFKYQLIVDNLYELLGNENIAFERVCLEQLYWFEQWANIV

>34-9_0249

MKRNSIFKNILVLLSVLSLHSFFVSAINNDINYDNKDDLPIIRFKQPNKRDIIQSGDVTR

NRYNPSKRSVADSHYEYSGNNALDVSSSNLFKRDILGISTSSSSSSATNKSGGSKHIKTT

SLLTCMDNSQFSSTFFDVAYYPKNKSVVFNIEATTTISSKIMIEFQVITYGLIVITKTLN

LCSLNEKSLCPMNAGRIDVSSTYTITSDLVNEIPGVAYTVPNIDAMVKVLAYTISDNDEV

DYNSPVACVIAPLSNGKTVEQTYASWIIAAISGVGILTSAIFSIRGTSAINTGNTVMSTT

AAHIASNSISLFTYFQNLSILAMMAVVNLPPIAAAWCQNFQWSMGLIRVQFMQDWISWYI

QSTGGMVTNILKNKSVLSIYVQKKLKKRGISLSKSFSDSIFADKSLYTNDERNITAVASK

TLILRGMNRVGYQAGIEITNLFLTSIVFLLFVLFVFLVVLTLFKSAIEIMIKGRALKAEK

FNKFLEFRLQWGTIIKGTLFRMIVIAFPQLILMCIWEFTQNNSTAVLIDAIIIFILTLGL

LVYATIKVVLKGREGIQFYQNPAYFLFGDLKFLNKFGFLYIQFKASCYWWLVPFLCYIFL

KSLFVACLQSNGKVQSIVVWAIESFYFFSLCYFKPYLDKRTNAFNISIHLVNWLNSLFFV

FFSTVFGQPLVVSSVMAVVLFVMNAVFALYLLIFIFVTCFLAIARKNPDLRYKPLKDDRN

AFFHLHNNKFDGVQEKNAQDYMELSDLGKQAMMTNETDKERMLRHASMFNDNEGYENSVS

NVEEEETQLHNGLNTNNQQFHTGNDFGQYNTSEMQLENPFGNKYSKTSSSESFKNSYGQN

KNGSYKQNPFAE

>34-9_0250

MSVLFECSLSSSGSVSISDGNRLLEFVQAINSISYILPNNIGDKCVGNIHMDKNGISFIF

NNVYDSIKVDIFVSDTVFNSFKYNSTKKVDNTGNDFTSIYINYSTLLYSLQNISDFHSGS

TINNNASISDSAELFIHYSEQIYNASRNNADITALSDSEEGVDNNEAVDLENHVGNNIDN

TLNNFNLILQDEFIKELISIKTFIRPANKSLNIGSADPNNIQFDCIIKSKTLLTTLNNLK

PFQSHMKNVYLWFKQIVLKPKFKHREFFTGGKSSDQSLRIPNIIFFNKNDEIGNVKISIL

NDINGKNFVKPKKRKQNKDNGYVANTDNIELLKFNTDGETENEAMILLDFKKLLRIIPIL

KISDKVLIQGDSNGTLFIQALVGYDDPADLNKERISIEVTIPTKDIEVEENISIDQIKQL

IAEYDNAKKPPKRIKPLESDIQAQVTDKVSTKSTMSKSTQSQYKKEKQTKLYF

>34-9_0251

MSDVEQVEIEEVVAQQPVADFEEALKVVLRNALIHDGLSRGLKESVKTLAKNEAELVILV

ESVTEDSIKNLIKGLAKGGEEPIPIVYVSDAKQLGEWAGLSKVDREGNARKVVGASVVVI

KNFGVDTPSVDVVLSKAA

>34-9_0252

MGKLEDQILDAEIDIKTLNSNCVSKSTTSLYDTFKSVLGKINKSTADGTEVQLTLLLVQK

ELQISHIEDIDRKIIELNHDRKESLQIIASIEKEEINIKKTKESLKAKSERLSNLQAIAE

EKENVKETSIKFVSQPLPKNKRRPLKSETRIKRPSRNITPVLQNYYKPLSIINEYQLPGL

SLKSIDFDIPFGSMFFLSKESPHSIGMCDINGAVDIMNTKSKEHDSDGNENSEDLATQIG

NEDITHFSGSQATINCFKYHDNTLFAGSNDAKVYIYSKYNNVEEEAANNFAPIATLDSHT

NEVTCIDYEGDVLVSGAQDRTVRQWDLNKECCVNVIDLNMIVQGPISNSSRIFSGSMTPS

ATSWMSTSTGFRSRSDSIISRSGSVFDFNAIMNGPSTNADHDIDTIYSPYISSGLESSIG

NDAEFASSKNSYYSQPPFYVSKIQVFDAALATGTSDGIVRLWDIRSKNVMRQLNSSASSQ

NNFSSICDMKFDSHQIIASNYNGEVIIWDLRMGKILNLLKVGDEICSGSKINFEMNRNYI

VANNSFDGTVHLYDRIKQTFVKWDEKNEDTFSSNVTSIRFKSGYLLEGYNDGIVKSWRI

>34-9_0253

MSSKMKKVFALTKVLEEQIKFEIGYSSSQKIIQNPFRILDNRSFDKENSKYLILYKNFVD

QSLNKQYEMFVKTDLSALLKYDYSIDNEKIEEKLENLITGNKFSSREYEMPDGGDKTSSF

NISKGSKNISEEYVKLHPDDFLDVTVEHYDTFYTKLIKTDVVIRSKIDIQKCLYIETMID

FEEKSLYIERIMPFMDKNAVNDNNLIVVPKPLLKNETKTQTALIKGPPFAYMDPTIQENL

YDLFKNWGIMENKQFIIDIFKSAELKEQDLYLDWLFRLKDHLSTSSK

>34-9_0254

MNFNNGSPTIVVLKEGTDKSQGKAQIISNINACLSIQDILKPTLGPLGSDILLSDQTNKT

IITNDGATILKSLDIVHPACQILVDISKSQDMEIGDGTTTVTLLACELMKESKQFLEEGI

NSNLIMKSYNMALKKAIELIKSDLSVKIDTSNENEEALRDLLEKCAKTAMSSKLINSNSK

FFVNMCVDAILTLDRQHLDLNNVGIKKVSGGSMEDSLFVKGVAFAKTFSYAGFEQQPKYF

TNPKILSLNLELELKAERDNAEVRIEKVEEYQKIVDAEWKLIHDKLELIKNTGANIILSK

LPIGDLATQYFADNGIFCSGRVDPEDLNRVIKAVGGSIQTSVSDLKPEHLGTCGIFEEKQ

IGNERYNLFTECSNTKTCTLVLRGGANQVIEEVARSLNDALMIVKRSLQLNSIVIGGGAI

EMAISKRLRDYAKTIKGKQQLMINAFAKALEIIPRQLCENAGLDSVELLNKLRMSHSVNQ

GKDNAFVGINFQIENIANNFESFIWEPMSNKINSLQSATEACNLILSIDETITNKQQGDE

GGNIQGIRGQGMGM

>34-9_0255

MQRNNRQQEALSNTLFNLKFTQKQLTKQANKSLKESGTNEKKLGKLLGEGNSDDNEELIK

ILASNVIRKKNEYLNLIKLTNKLDIIISKLNTSMIMQQVNGQFMQITHNLDIALKNMNLE

NITMVMNKFDEQVKEIDLNTETLTSVTDSNSVDPLKFEDKDKVNELINKVKDNNNLKYPK

VSAVEDSLVSEKEDVGVDADVEDKLAQRLKALRG

>34-9_0256

MSRPQVTVQSLEGASTSSAVQLPAVFSAPIRTDIVQSVFTRVNKNKRQAYAVASNAGEQT

SAISWGTGRAIARIPRVGGSGTHRSGQAAFGNMCRGGRMFAPTKVWRKWNVKVNHNEKRF

ATVSAIAATSVSSLVLARGHKIEKIPEVPLVVSSELENVTKTKDAVAVLKAVGAHADVVK

VIKSKKMRAGKGKYRGRRFTQRKGPLVVYAQDNGLVKAFRNIPGVETANVESLGLLQLAP

GAHLGRFVIWTESAFAKLDSIYGSESVASSKSGYSLPSNIVSTTDIQRVINSAEIQSVVR

EAGQPTQKRSHVLKKNPLKNKQVLLRLNPYAKVYSAEKVASKKQDKSGKAASAAFLATLN

QD

>34-9_0257

MRVLNFKIQATIKAQFKSKTFLKCFHTSSITCSEITIPLKDKRNIGIIAHIDAGKTTTTE

RLLYYTNNKKSIGNVDAGDTTTDFLKEERARGITIQSACISIFTKNKLINVIDTPGHIDF

SYEVVKSLKVLDGAVLILDSSMGVEAQTLTVFKQAHTLPKVCYINKMDSMGTNFNKCLKD

IMLKLESKPLVLTYPIFSLDTVGLSKDTDDDKYNSYILTENDVNNLESNDKFVGVLDVIN

GCATIYKPADPNYLKVYTYEQLNPNLKKKLELARNSLVETLTSFDSSLLDYILESEIEDY

VRDPKLTAKVLNESIRKLTIQKEVIPVVCGSSFQNIGVHNLIDSILEYLPSPIDSVKVPE

VEYQGKKVQLTYNPKNDNLDVLKTDKISMAQVFKIVKYPILGNLVYFRVYSGSFTNNSKV

FNSSKYTQLLQNSSDSKNSIKLKNVASGYPIKQLYLMQGNLPIKTKKLNVGDIGCVAEKA

FVDQTQISLETDFEDLKTSSIGALSTGDTIISGTNGISGILKSLPTKDSKSYENLSTLKI

TPVSSISHPYKLSIQVLKPNKFHSLITALLEIRRIDPSIDFQYDELLNQIVLEGLGELHL

EIVVKRLLDSTGGEDPGYGFGNDLAVGDISVSFMEKLKTNSQNSSWAKFDSELENPTDIV

LEVFDKCKFDYRFGSFVSNESEEILEEIENYKANGYVVFNTVNDNDNNYVVVPQNLITNW

NEFNQMSSEKFITCLRASCLGICTKGGFNHNLPLRSFVFVLSEENFEIATDFNDASKMIN

SMRTVFSEHLKKTYSKSDFELQEPFVDASLSTEEKYISSVSHDLTTKRKAIIESIGNESE

TTDFNSEKAKLDTTKAIENFNSLLYVPKSQVGDPEVVIDLLKDINSQDRSQVTKTICFKA

PIRKMTGYMPALRKLTQGKCTLDVSFKGYNTINPSDVSNINF

>34-9_0258

MSSLQSSSLGTPLNWEDSIPFIEKVKTNGITQLLNILEKADQIKDKPYLWGDELEYLLID

AKNNKISVDNDDILTILNTEFEKECKDNDLVYHPEYGRYMIEATPYFPYNTTSDLKSYLE

PELNMVKRKKFLDDKLLKKRGLCLLEMPNYPRLGSKNFLYDYDYNGNDFVNEQNKNIFSQ

SLFLPDEITNRHPRFPTLTANIRKRRNRKVNLQIPMYKDEFTPKFDDTVHDRKWFDMDVE

FVKDDAEAIEKHFNAKSENPLKTYKLEEQHIYIDAMGFGMGACCLQTTYQAPDMDSARYL

YDSLANFTSILLALSAGSPFWKGYVSDWDTRWEVVSASVDSRLSYEENNHTHDNSKGYNV

SCDDKGTLVDIPLKKVAKSRYSKIDLFLGSSRTSKDLSEVNDVEVVINEKVFDRVNKALN

GDENLAKHFAHLFIRDPIVIFEENVDEVDGEMDHFENINSTNWQSLRFKVPHKISSGSEP

EPGFRVEFRPLEIQLSDFENAAFAFILNLIVQFILNPDNNINFYIPMSKVWANFDIASER

NSLLKNKFEWITKLKAFNDSTHIASETTPMTADQIMHNPESGIISCIVNAQLKTLNFIKK

SETWEDLKSYDDESKTRAYYYIKLLSDRSKGILPTDASWQREYVMSHPSYKKDSRVTEEI

NFDLINLVKNIHCYSPKTKDEENWLYKLFGEELGQYLVNHKL

>34-9_0259

MPTILTKDKLINADTVGNNFIYVNDKVKYKKQITVFPLEEVTEFQQRSKVKRKEPLPEPG

IYLNISDDQYPLYSNLKDKYNFFKNNFNKKLKYTLFKPLNTKSEYQKIDITNDSSYYQLI

TNFEQLLQRTLQCTIQPKLMKKGSSGTYMIFDSIDTNPSFIFKPMNEEPYSIMHQPKWLK

FLQYKLLPWTFGRECLINNGNYGYITDLQVSRLDYMLFGEDTIVPWCDVCVLDSASFFAK

NSVRTNIRVFFKDIVTDIEQNYEQSINWVYYCFVFIKKFFLKIFKLIVFIFTGTFSGERI

AVQRKLGSLQVFERDFVTADVFFKNNPLPEQDLEDNETFNIWTPELLNELHIKLQKLFVL

DFLIRNTDRNLDNWMINYSPDNESGNRIKIKAIDNSLAMPFHHPNSIRTYPFEWLNHLPL

YILKKKLDPEFIQALIMKLSNDSWWESFRLIFWECHSRSGKVFVANSTDANFANYYDNPN

LNLFDNNKFKLNIDYKVDLQWSLLKGQAFLLYKLLKAGISGIEVDIIDLIQMERCYVYED

SCYYYRSNHKESDSTLKKEIWQSLGLDINEMFDKFERKDIIAPEYDFNHNWDLFLKGKIS

DEQWLNFLRTSKYQKKGDGLDVLNLQANIDNLEDIEDVSDLDASESSELINDTPNKFVIA

SSINYDTSLISEYFYDAVSSINNQTDDNASENDPMINASTHYESFPIDVPIYKGNDKEIL

AESLHSVLSKPYESNKTSIISGYGACINPNEESSKPPEYEIFYIDRLEVCKEPKALFKTW

>34-9_0260

MSVANPTIDTKEDVASINVGTNNGSNQNISSAGILERPEINKFLNESDIATNTLLTKFKT

SIIICEEFSKIIKRKYSLESNYNDEFIKSSKNYFTGQDGNSSFSATLNACIKDVLKYDNK

LIQVKQSYIKALVKMYDELSALLLTMTKLRKQTKEKIKKLEKDVSDSIHQAEKAKNKYDS

LCQDYTKLKKGDPTKTKLTIRGSKTLPEQEEDLRKKIDMADLDYKQKVDLSTTLRNKFLS

IERPQIVKNLKDMILEMDVALSIQIQKYAIWNENYFLNSGIVICPLNDMEKSMKTTTNSI

NVEYDLYNYLNKYVLNKNKSSMVNKNLIPVQYKKSPYIGGSKNTPNTSAGSAISNPVSGS

FVYNSGVGGIPDTNSITATPSNNNFSIAPMKNTNPFVKKHQQSSQMQSPNLNSFHQGNAT

SNSEAETINNPRGGSVNSYKTLDPVGSPSIPQQSATPAPPSSIIESIVHTEADSKNAANM

NGENGELAASFKTFGISLSQLILLEQDSLLPKFVKQCIHVIDKYGLDIEGIYRKSSNVLE

LQKYIKLINQDPSNYALLLPPRSSENGHGVEEHVILVSNLLKHFFKNLPVKFISSSFLSE

FQTYIAIPSNSESNIVMKRNYMHGIVYKLKDEQYWVLRALLFHFKRILDNSNVNRMSLKA

MCIIWGPILISSPSGDDLNSNQNEFGDSNLSLDNSNEYMKDVQFQIKVMEGLFDVAEQAF

EPE

>34-9_0261

MLDLQYMILLSKTGKVHCIRWFGEYTTKEKQVLLKDIISLVLTRKPKESNIIEYDMTGHA

SKNTSKNGHRIMYKRYASLYFVAGIEDSDSSNELLILEAIHRFVESLDKYFGNVCELDII

YNFEKCYYIMLETFSSDGNLLESNKRKILQDVQLMDQLESGEGLNGLLG

>34-9_0262

MLYLIGLGLAYPSDITVRGLQKVQQSKKVYLEHYTSILMNASLEELCEFYGKEEVILADR

EMVEGGCEEMIEASLTEDVSFLVVGDVFGATTHTDLILRCKQRNVPFEVIHNASVMNAAG

ACGLQLYNFGQTISMVFFTDSWKPDSWYDKIMENRKIGLHTLVLLDIKVKEQSIENMARG

RLIYEPPRYMSIQQCCEQLLEIEDIRGEKAYTKDTPCVAISRLGSDKQAFKSGTIEQLAS

YDSGEPLHTLVILGRQTHDLELDYILEFADDRENLTEMLRKDQEYFKPLPWVPPTDDEMS

D

>34-9_0263

MAKDNKSFMNSQSSIDNSVVINYVNFNQSGSCISVGTNKGVTIFNSEPFGKYYTSMNSPS

QHDLENEENSNTDDAKCNFGIVEMVYNTALLACVGLGEQVNLSPRKLRLVNTRKRKTIVE

FSFHSAILSIKMNKSRLVILLKSQIYIYDITTMKLLHLIETSDLFEGIIDISQVIDDKEF

PQILCYSSPQKVISSEMNSHLTTNNLSLLNKVGTGYHSNNTKLKEISGEDSNDFEIQGPN

NPTANSHGDIVIFNLLTLQPTHVINAHKAAISTFKLNEKGTLLATSSVKGTIVRVFDISR

GVKKYQFRRGTYNTKIYCLQFDKTDQFLLCCSSNLTVHIFKMNEETSQEYMESDTDNLNK

KNSNEQDVDAEPIVDNSRLTMGRIIRKSSQNAMKKIGNALNLKMTGLEPNRHFASLKIPS

SKSNNAANMSSSFNDDDNDSIRTGTSMHSNAEGGGNNFSINNSKDLEIKATFADFIDINI

DEYPEMTNKAVNLPDMEHPGSINPENESAKYDTKLLRVLPIYVVTSQGHFYKYFLDPVNG

GDCILIQDYQMVF

>34-9_0264

MLRLSSSIKYNKLSISKFHISTIRLTNKWKDLDLEKKQNFIRQFVTLYKEKNPCSKSNVM

YKELSDGMEEHGDTPYVFGILYNELVDVASGKSVSNQKGDGPMGDADFLKLVSK

>34-9_0265

MIFNSLSIKRLSSTSTSLPFKKHAKLNFDLYSPQHSTYGKLPYHCQEPIIFLHGIYGYSK

SFNSDYQQLSNLLHTPIYSVDMRCHGETENCLPFTYDALAGDLDNFVITHNIKKPSLIGF

SLGAKLAMLAILKSPHLYTSGVIVDNVPLKQPRIKPNLTAFGNALRDSVFKSGVKRNDPS

WISKSFNVMKDVCSDMPANFYLLHNIQPKPSYLKKYSTEESENSLFCKVPIRELSSHVVE

NVPDWPEEDLAGVKTDVPILVVKASTSGFVNEDGVAALEKHFSDFTIVEVAGTHLVMKER

PQEYISAVGRWFYQQNCKKAAALTKAKKTNDQKITQQPILSYKKIEIA

>34-9_0266

MNSTINLFSFDLDFNPTSNLEDVKLLLDIQNNSIPIQLTEDIALEIDINPYDIQLVLINN

ETRIEKDFKFSNINITEALHEDGDFHWLLALFIKYLATINNNKINQILASANIKKVSSFT

LQKSIDNDKLRFSFKYILTENSFVKIPSPLLTYLAHLNVKKRTNKNEIDIKSIFNKNLES

YTLEANKTNEGIDAKSKQKLYPLVLYSYQEKTVNWIVQKENEPFEELTFNVIKANPLKYL

KKLQLGYIYLNNETLLYNTITNYIMSRKTAIDYIYSEHLKYKKTLENGNLKGYSGIIAED

MSLGKTVEIISAIILNKLTDDQIKYYNNWRRFYVDNKEDAATQTVKKLIKSNLVVCTDMI

LKQWVSSFSSFSDTLNYKVFHYTGFKGFQKEFPGTTLNECAEILSGYEIVITSYSVLKNE

VPYVEYADNRMNAVRRTRTKNEKVFDYSSPLCSVTFFRAIFDESQSLSQASLSSLSSISR

IHTWAISATPLKYNDLDKNISELANLLRCLQIHPFDFANVKVTNFQSIQDWVFNKETKRS

VAEDFINTIIEMDIARRHTRDYVQKQLNIPKQHKFLLPVYLTPIEREVYNQVYDKYYVEM

HDSRYYTDDVNVLNNYLTDLTTMCMAALGIHDYRGRNGIFHDSFYLANQQHTPEDSMSMI

LSNLLEANKRALALELRRYIDLYVQKSRYLIEKESQYEQSEPILLKALKIIDENIVGKKE

ADYNNDTIPLSLIRHSVVFFLGDCYYNLGLQAVESNNVQLKQEYEKKENTFYEEAAKMRK

SILQEQIEEVENSLSYLKTDLFQKPPLIASYSNFFTEEMEEMYVKNILYMGQVELTHTKL

NLFLKSVYDDLEDGFDEEVNKVNKFYNKSLYYISKAVDEQIAVDGTEVKKSLNEIKFEAF

KSKTAFVEKNFKVYFKKANPLLLGITKSIKTMNKQITLLNELYFDIIALLSIEIIPVESE

EKDVDPDKPTEYETSLEAQQDISFKIELIELILSNRRFITLSVGTNKFVVPEHFKAFEKD

GYFITNLKDTWYENYTDFLNYTAIDKNLTWKSVEFLIDSTKVSKDSELHTEWRNRLKELN

KVFNKKINYYKSLQQISDKVIDLDSIADPGKVLRINNSFERFVADSEAIQRKLKSKDVYL

SNLATLQKGEDNKCLICLEYIETGSILECGHRFCRDCIQHWLKTSSTCALCKMPCSQLEV

HDFYIDLNPNDVGKQNAKSKQKNEQAENLFTETLNGYKVYDDVKYLSKLKVPDSKYGSKV

NVLLRLLKFIELKDEDSGYENKSQIIIYTRFPKIIPFLERILSESGFKSIVALDNKMKGS

QIDEFKKDKTIRVLIMTTNDNAGLTLVNANVLILFDPILQTSVESQAINRISRIGQNKET

YVFNFVTMNTVEENIIRYKQHLLKQSDKIQTSVDTAASSVNNMSNKTSFKRSRNGSTSNL

EAHSLHVEFLKNCLFNDFKQV

>34-9_0267

MFKEIKNIFKKDKQASKSPTPEKKKIQESRKTSNEINRDVASSNVPETEDQAHQTSEHPI

SSEHKKNRDIKFKPEYMDKNSPDESNMNSALQNPESRSRLQSVASRNTEVTHGSSVVSLD

SAWVEDDYEDEDDFDEESEVSRNIEDEIDLDEVDTVSQIQTSHNGEHEKPLRSVHYPTQP

QSFTVNRTLYPEQNELDEYLLLEKIGEGAFSQVYKSVKFNMNLIYSNEQDFDEEEYEGEY

VAIKIINKQMLDSTNSRNDKPKITSREQVLKEIMIHKLVSQNVDYKTNRSWSDKNAELNE

DILKRDVDINSEDGSTHIVRFVDFIETDSCYYMIQELVTGGEIFGEVVKYTYFSEDLSRF

VVRQLAFAIKHLHKLGVVHRDIKLENLLFETIPFFERNIQKYDPQLILRSSDDPKNKLDE

GIFIPGVGAGNIGIVKLADFGLSKKVVNMNGADLSTPCGTVGYTAPEVVRDERYSFQVDL

WGIGCVLYTVLCGFPPFYDEKIDVLTEKISKGEYNFLRPWWDEISDGAKNCVSRLLEIDP

LKRYTIEELLADPWFSTYDCQEFYSIETKNRLKKLQTKVFDRRLKRVLKQKELSKTSDAK

THKKSRSEVDDDFVMTIPIKSSIDTSNTLLYSPAAVAMRDAFDITNAVQRNKEESSNLAE

NNTRNMAKQAVAKNAANNGLTDISEDQESEDFSDYANAVNNNSLDTKFFQLKLNSSTIVK

RRHNGANTGSKVKTKSPLANISN

>34-9_0268

MSDSIQVLNELFGKLAVATVDNRAEVSTEISSFLNGNIIEHDIPVQFFKQLEEALNNKKT

AANALSAVASVASENNLSPSVEPYIVNLTPIITGLAGSKDKETQDLAKVALCALAKAIDP

MSVKVFLPFLTASLSSTSKWQEKVADLAAISILVDTAKTQVALRMPELIPVLSEAMWDTK

KDVKVAASETITKSTETVENKDIEKFIPQLIACIATPTLVTETVHLLGATTFVAEVTPAT

LSIMVPLLARGLAERDTSIKRKAAVIIDNMCKLVEDPQVVAPFLGELLPGLKTNSITIAD

PEAREVTLRGLKTLRRVGNVGEGDVFPEISNAGDIATTKGVWAELTKSINVEVDAKFEPV

VTYIAAIAGELIDERIIDQQTWFTHILPYSTVFLKEKNAKEIVDDFRKRAVDNIPQGPNF

DDEEEEGEDLCNCEFSLAYGAKILLNKTQLRLKRGRRYGLCGPNGAGKSTLMRAIANEQV

DGFPTKEECMTVYVEHDIDGTHAETSVVDFVLAGDVGTKEEIIAKLLEFGFSDEMIAQPI

TALSGGWKMKLALARAVLKNADILLLDEPTNHLDTVNVAWLVNYLNTCGITSIIVSHDSG

FLDNVCQYIIHYEGLKLKKYKGNLSEFVVKCPTAQSYYELGASDLEFQFPEPGFLEGVKT

KQKAIVKVSDMTFQYPGTTKPQISNISFQCSLSSRIAVIGPNGAGKSTLINVLTGELLPT

EGEVYTHENCRIAYIKQHAFAHIESHLDKTPSEYIQWRFQTGEDRETMDRANRQINEDDE

AGMKKIFEIENTPRRIQEIHARRKFKNSYEYECSFLLGENIGMKSERWVPMSSVDNAWIP

RSELVDSHSKLVAEVDMKEALASGQFRALTRKEIESHCAMLGLEAELVSHSRIRGLSGGQ

KVKLVLAAGTWQRPHLIVLDEPTNYLDRDSLGALSKALKAFEGGVIIITHSAEFTKDLTE

EVWAVKDGKMVPSGHNWVSGQGAGPRLTKKEDEGDKFDAMGNKIASAEKKAKLSAADIRK

KKKERMAKRKAAKARGEEYVSSDEGL

>34-9_0269

MKLSIFLKALTCLQMIATSVNAGLITSKEKTVDSLDPLKNIDSEKPPEGVEWQDWHMDHE

HQLKEYEPEQFFALHDIKKKGYFDSNDILTMYGLQREEIIGDGDGMGNHDASEKQVDQEL

KDRLVKFVFKLFDLDDNDRITKEEFLIISKEGKKFPDLGVGIGHHADFELEYEIHHWNKY

HKDQDPDVEIIHKEDVEHDLLHHEHEIEHEESVQRGSSRQTVITDDELELQINVNNIPSR

FRSKLY

>34-9_0270

MVKAIVFSDFDGTITLEDSNDTLSDNFGISKEQRLELFKELINSEGSFRDSFQIMMDNIN

LPIDECVDYLVERINLDPGFKTCLEYCNKNDICVVIISSGMRPIIQALLRHLLGKDEADK

LTIVCNETEVLDEKTGKWTIKYKNPDSIHGHDKSISIDELKQATELSGYKVDQVNEKPIY

FYCGDGVSDVSAAAKCDLLFAREGKDLITFCEKGDIPYHKFQSWKDILNGIKQVLEEGKD

VKDLMQNHKHA

>34-9_0271

MSTTKKITVLPGDHVGQEVVVEAIKVLRAIQESSTKDIKFEFDNQLIGGAAIDATGKALT

DEALKSCESADAVLLGAVGGPKWGPDSPVRPEQGLLQIRKALKLYANLRPCNFISEALLD

LSPLKKEIAKGTDFTVVRELVGGIYFGDRIEDNGSGVAQDTEIYSVPEVERITRMGCFLA

LQHNPPLPVWSLDKANVLASSRLWRKTVERVIKEEFPTIEYHHQLIDSAAMILVKSPTTL

NGIILCNNMFGDIISDEASVIPGSLGLLPSASLSSLPDTNSAFGLYEPCHGSAPDLEKNK

VNPLATILSAAMMCKLSLDLYDEGVAIEKACKDAVESGIMTGDLGGSSSTTEVGDAVASA

VKKYL

>34-9_0272

MLGKIIRNRTVALNSAKLNIKGVCTSHISRAYQSTTNDVDFKNHIDHRFDDSRLITHTKL

QKPTEAGAEESLEGSPSQTIATALSHAYRIDPTRGTRPIYLDVQATTPLDPRVLDKMMNY

YTGLYGNPHSNTHSYGWETNTEIENAREQVADLIGANAKEIIFTSGATESNNLAIKGIAK

FYGSSSSSSGVKKNHIITTNTEHKCVLEASKSLKDEGFEVTYLKVDNNGLVDLKELEKHI

SEKTCLVSIMGVNNEIGVVQNLKAIGELCRSKKVYFHSDCAQAYGKIPIDVEDMKIDLMS

ISSHKIYGPKGIGALYVRRRPRVRLQPIISGGGQERNLRSGTLAPPLVVGFGEAARLMAV

ESEADIEHVTRLSKKLTDALLKIEETSLNGDPDHRYPGCVNVSFAFVEGESLLMALRDVA

LSSGSACTSASLEPSYVLSALGKDEALAHSSIRFGIGRFTTEEEIDYVIKAITERVEFLR

EMSPLWELHKDGVDLNTIEWSGH

>34-9_0273

MLVQLTKIINLLWLISFVVSVKALPIAENTDLIKRGFGNSYVPVQQCYPNVNQNGMKGVP

ITADVLFVQYNYDIDSYLVTINFKVTDPDITIENLNELKWLNVEPYTESMIFSRNLQLFP

DLNLQDFTISAFFKGYESYQNSNALSFNNQLGLQFDFCQYNVNGGSSSVGACSCWNYGST

SFDYYFGCNTDNYCNSQNAWPDYIWPKECQPSNCDVPSLIHENTYQQSYCSVNGYTSVIA

TKTHECVTKCKTSTTSTLHSTTVITAVQPVVCTYTIFTTDEITHVETLVTKSNGTPTTNY

ITSVETSVGSTVTTSSDTICKATATTITTSSTTGTASTTVDTLVCTYTIFTTDEITHVET

FVTESDGTPTTGYVTSVPISLFKIFFILVSESNF

>34-9_0274

MTEINFLYSLIAGGIAGTSTDVFFYPIDTIKTRLQSKEGFFKSGGYKNVYSGISACIIAS

APSASLFFVTYDYFKHTLKLNHSTRLSDHQVHMLSASLGEITACSVRVPAEVIKQRTQAT

LGSTPLITLKAIIKESQGSISLLKNNLYKGFGMTVTREIPFTCIQFPLYEYMKLKWSNHS

KIQKKDLEPWKGAICGSLAGATAAAITTPLDVIKTRLMLSKLSYKVTIGDIIKELYREQR

YGMSFFKGIGPRTLWIGVGGAVFLGMYESVVAVLKKDDHKSLN

>34-9_0275

MDLNLKFDLESVKIEYLNGSENGSVNDSGLPKDGIKYRLINIPDEMLSDLQAQELKLKGD

NENLYFTTNSKVFTVKENFHSNTVMLMTHSQDTRNPPNFVAYTMQQSELELLQVKLKLTT

DHIPLYTIEDYNGTCGNSDEKKQHTFIKKRDLIDSLPLSYKDFDEQWNGSLLIELPDMSV

KKISPMVESEVLELILLSIIALKMDYNNVQFDKILEKLKEINFDANEQIDLLVKAVFYKY

TFNNSELNMKEIAKFYGLKTLKKVCPASRKKYIDLNDFYVYWKDSFPDYFNCEIDVAILI

GHFVKAVDSNKILEIEKERLPKDIVKRVKYLMNIQPAWEQLHIQPFFDELNVKNIKTDNF

IMKYAKKKRERSGKVIITSR

>34-9_0276

MVINKEHRRNKSSLGSTNTFIVNSVVKQFENESDSDHSDSDVVNNNDFTANANDLSSVYI

DNTKNDDYEDVSEIPMQNQNIIEKTNTNSKPSFNIDKISLPTNIFNFLQPNSQTLKESKE

FHDIFSESCIIKLNDEIILQKFIAIVYNIEKKADSFCCFLIDKFGCTFHKNIDISKNSKY

FEAIQNLPIEHQNLNVSKSVAAAVLQKYAFILKHHPEFVENQELLNPITAANTLINSELI

ETNEVVTPLQVCLKIQQFSLLNDRQLFSNFIDVVYNTNLGLEKMEQNNHLVFFLGEQLEQ

LFNPLTEYSPEEMEQTYKPTSDTVKVEEAPIIQTIIKEFISVQTYLTIDLVRFLTKFIIP

IRIAITKQQIPGLTNAKFNEVFPPTIDEVTRVNCIFLDALKISSEYGLKECMKSMNQTIS

YFYKAYTRHEAATKNCFKKVELFFKQYPGIQKYVDLDKYTVLKIDTVINAPQEKLFKIKL

ILDRLVKENTKNSSGNIDEDVKAQYDHIVDVINQFGTSEDLQHNNTEKNYQQRILTPSGK

LLTEIATNWPVELQYKWLKRKVVGTFDCINQYTGLKNCVVIFSDYIVCLEVLEDNQTLNN

PLISDVLMNSLINEKPLPSSIPSLKVTNYSAVSKTLITAYGGKGQLRIDILNDDQALYMQ

LVNPTDSVEYICELLTKACILCKSSAFHLFKESYEIEKKNEHTKFNCYVTAHDLLNYEKE

KIKSPFTIFLNIKPTLELLKDNGIFYGVFVNNLNSTDVEITILSLSKTFKTEIVVSKISE

YNHVIMKKMTDSIFIDYYYSLNSPLLDKILSINANIVDQIEKPLIKLKESVVLSSGDLKR

FSTLMNPQSPLLDFASPQSYRNVQLPTKNVETEDINKESIEEKKTISQIPDTLKPESNLE

KKEAVTMVPVTNLQTKKLTTSKEEKTVINQKVAPKTAETNKSKDKQNRKKNRLSGFFSNI

FGGSSKSKMESKKSSGPKTSNSNHKILEPNYKHKPVATKSNVLNKPNELNIKSLSSVNKK

EYASSPSKKSISSPVKIQRESYKEDDLPKLNSNLDTKLFNNSKGSVLTSATVTTKKEGLT

IDTKSPFVNDEELYSDTADSSGLDTEIGQAYMSPISNSHESKFRMITPEKLEVFEKKPSI

FDVNQFHTPNNNDKLEDIDDMIIEQDGNTKRVRISQSPSFRELFLKLETDIKPLNIRSSP

NYWSSTSLNASGLTNEKGTKPGSKAYLGSIYETVIEEDEEDEIKDDSSFDEENNDQLKKS

LVINTKVDHVTEIPKHSPLEAHFPTSATSSKSKGKWAQNEKYGNKFKVVRYENAANPSPQ

KSDYLKSPSKTPLMKNNNDDITDDIKTMTLDGVNLDFVSPVKTNNEALETTKPGNRIVSD

LKIDFSNIGLGNDNLSQQDSLKPSSPLMAVFNQSPYETNVQNDRSKNPVIKPKTKKFDIR

SESFGYLSDFI

>34-9_0277

MDLTNTYRYREYYENDLPLTDLAPKLVNTISTSAKNVEAADDDNEKTHTHLNLKQILSKE

IINQKHLTTLFMKTGDVSPLINLNQNLAMNVDLTKLPGVLNEQNPALLYPLAGEVKIEAK

DKTLLRDPKIRLKELQDDLHTQEKSSKAEDNKGVNVDNLDSSVFQKAKIDASKATFLMRT

EYSTRSKANANDSNKSATTDLRNKLFLKQQKTQKKLQRLNGSEFVERIQKSFQSLETLED

VSLKNFRHPVKKNLKPKRIWKLLPDANTMDENFISLKLNGSAAITTKEEAEKVKLNTAIF

RSVQVEEDEWMSLYSLKDKKLNNELLNEQEISKDDLKNIPAEHTPKVYMFNRIRDFNIKE

DTTNENHDPEVFIQFNKEENVASYKIIKKQLVLNKRRHNDLLKDIIKENNFDKLEVSYNV

PTEEEQMEHDNIRAVYDPIEFPKM

>34-9_0278

MSFSIVKEIAVCKGKLYKLTHESSTVKGNMNLNLYVPSKTSSTKIPTIYYLSGLTCSPDN

CTEKGFVQFQADKYGFAVVYPDTSPRNVTWPKDVNIDTSNWALGEGAGFYLNSDKFENFK

MFDYICKELPVSLNESEFGKTIDFIEKKSIMGHSMGGMGALNFYLKGKLNEISPYVSCSA

FAPVANPTDCPWGVDAFEFYFKKGIKAGEAIDPTLIIGSEAFKGKSLDILITTGDSDPFF

KNQLRTENFIKAVEENNVQNVTINIEKGFDHSYFFVSTFVPQHCEFHAQKLGLI

>34-9_0279

MARILSDDSNLLHSWSFFKNFKENILSSILIEKLNLLILGTSDTNKLILVDTSDNYKIIA

DYRITNVEDTTDSFASVLCMESYADKYLFVGCSDSLIRIYKVTNTSLQLLNTIYSTMDVG

DILSLKYLEQYDTLVIGTQRCCLLYVTDILESKKINNTLKEINSGESAIADENNHISRLP

SKRYDKFFDSFGPTGNKSTVSTRSLSNATQDDVTFQSMNIIQVPQENIINFAHNGFIYCM

DTYTDTTDFNSPKQYLITSAGDGLTKRWIMDIDTKVLHLKNVIDINDDITGYGFSDDEEE

KENDAVISQFIKYPLLFQGISEMRINVISLLTDRLIETIDLNEVNDSNEVVANGESLLHA

IKASSINDSILFIAANDNVYQYNLEGFGSSKGDENAELLDHKVVKRRGSLVPDVKIKHFI

RFIRSQIKSQILNICVDGNKLAVFESDGTVYLFDLSDVLIPESESPALNEAELVAISKEN

NVLTDNDLYHKSEILIKDLSKLISFKTTAENKQQIHACASYLQSLLNRLHANVEVFPNSI

NGTPIIKATFAANSKSIQNTRNKKNVLIYAHYDVVPSSESDNEFQLFVKDGFLQGRGVSD

NKGPIISMIHGISELAEAKNLKNNVVFIFEGCEESGSVGFEETLKSNPKLTQSVDYIIMA

NSYWIGNKYPCLNYGMRGVLNLEISVEGIRDLHSGTDGGIFSETTKDLMFLISNLIDYRK

NTLNVPGFVEDLNNQELCKNEINFFNDIIATGEGGVSLETLINKWTKPSFSITSMNGSSE

KSIVPKEANITLSIRTVPTQKSVSILKNLIIEKIKKDFDSLNSSNKLKFEVLNEAEPWLG

DFNDRIYKIASEELKSIWKNKVLLIREGGSIPTLKILERMHPTAKIMILPNGQASDNAHL

QGEKFRLENFFNLKTSVKNIVNRL

>34-9_0280

MAYNYSTPPDQVIQQDDIEIEDLQKDLIKKRLDCQNLSKHTVKADCLNSIDEYFKLEFNK

ESLKLIVVAGNNEDTTATPVENEYLSNMDHNQLFYSDLSSEESDLDTHLDFSEKYKRCIH

STPTPQVSYKSDSRKCSISSDLSVDTLISKQNYIENFLSSLQSYKTTNNLKSFQSFWQNC

GYNDNFIKPASNIKKRRKLRNDNSESLKNLPRLQSPILFSKKD

>34-9_0281

GITGKYGVRYGSSLRRQVKKLEVQQHSRYDCSFCGKKSVKRGATGIWNCGSCKKTVAGGA

YTVSTAAAATVRSTIRRLRDMVEA

>34-9_0282

KDLENKKITHYKNSDSSVSLLDLYCSDNENENLSLSINNDTFGGLNSPRLVRDDFFYNPT

TKPMSSESFNWRHFKYSSNSGADVDENQNNESIGLSSLPDIDTEKSTQFGNNTDSSVIKT

SKSNSSLNTAFFRRWKSPSKTSTNQLNLGEFPVLYAKSKNEKNANKPLIKKLTEKLIMNK

KESEIEEDQLNANLPFKSYKNEVGKKFRFFSEPTTSDADNEFNTNIEK

>34-9_0283

MVNLIAEKDYFQVEPTLNSGIDTNYAFVKPFDNYQSIQNIITKIEQEPNTRLFILARHGQ

GYHNAAVERYGEEIWDLKWAHKNGDGVLEDWNDANLTEKGINQVKRTGKVVFGEKMKGIW

ADSYYCSPLRRCLQTFRYEWTEKFNIEKKDQEDLKKTIDVIIKENIRETIGVHTCDWRQN

KSKSYDECIKPYEDNTSDFQIKFDYEEGFKEEDPLYSDTYRETDDDIDARIEKALKEIWE

LEDKKEAENLEYRGKVISLTCHEGVIKSALRVLKHHPIPRLETSGVVMVIIKNTEI

>34-9_0284

MVDYKCRKYLKQVESHIEVYNNKGTQYKLKLAETSDELDSMPYETAKERTSKYLELIEEN

LGEYYTEFNGLINNIDEETLKNDTEGEYKWQEHKYFEMISSKGLFYVEVLNNENDFLGFF

SFNITNDSLIKNEDSHVNKKVKLSFFDKPKGRIEKKNIFYLMEIQLKKKASNNGLGKLIF

DNFIFELSKDQDFDIEFVCFKKNHIGNKFYKKMGIPMNHDYKKTIVSKEMDEIINIYRMG

NIIE

>34-9_0285

MNFLTPSNIKSLINFEKIASNLTSNNYAIYAKYLSYLTIPVSVIFGIANLFHVNLVIIFS

IISLVQGVVLLFLEVPFLLKICPLSENFIAYLKVINDDNKKKLILYAVLAIVQWCSLIFK

ATSLIVVAVCLTCSFISYGVAYTKDQHISSEDSVIKSPFDDDFAV

>34-9_0286

MSDQYENKFDDLLSKLNSVAQNTNEYNNLDSKYILRQRIIIINNYDGFDLFIKNKIIQQL

MMLGNKSNNHNYDLLLEDIYVSYTTKPVNDDIEVDVLNQNNIKYDPKNDYNIINDYKCTH

FESDLKIYNQEEDLWYSCKNCYLDTKGRHTKAKKKIITKVFTHIKCDKCDYINALNQINE

FEIDLNSHQIEDNDTRANDSEDNKKSCMSCEKPFSEYQCHFCLIFENDEENYMYHCPYCK

ECKLGEGLGIDYNHCKECDCCMPLEMFHNEEVSHKCIRNNLSSDCTICGVRLNAYQSYER

LQVDENTDDKESTNSTNTASNNHLDKNNEKGPMKDVMKSFQNNGVRKRPIIDIDVGRVQF

LVPCNHAIHETCLKEYLKNGQYKCPICQVSIVDMEINFKLLDEEIQQCPLPEPYNLWRCV

YKCNDCTTRGITSYHFLGIKCRCCFSFNTVLLQVLKHAEEMANANEQTSNHTSIDTKRSI

VSKEMVNESFMLRKRSNSELNDNDMDDFLNKFLSKDEDLSISHVLQGLKQYYNFKKNTGK

KTRTEVENIGLADSVDVNSMMHRLKFNVLDRFLSKKPNTDTESDIAESSNDDHDISEGDS

RQKTLFNDIGDSFKQFISFLEDSNAHDI

>34-9_0287

MTSDNNDHLHDHNNHYSNDNMHDEFLNGNDNHNEELMNKTGDINDIDQYIQNIPVSADQS

DKHIIGNVIRPSMNIFGNLHQTPVQTLNTTGTTTNSNNMNAYYSQILSNYNGNGHENGFN

VPNNVNTHSYDAYNNNDHDNIQGNNILGVFDNTNHLEKEHTENDLLKHRETGRKSNADSY

NHLTGIPQLDAHVIANMSWFQNPPSHLYKNLDVQQLPTSREKEEAEAIIANMNLDLSTEQ

ALGVQQRRIIDDDVLKKQIKTKKTTTTKKTKARKAMIHDLSLLTKQSINEISPLLSDMNN

YNKYLQTSQFKNDTKTQRRVQKRKSLLKQGPKRPSSAYFFYCQEERKNLQKQFPTLMVPE

LQKKLGEQWRSLTDAEKEPYKEAHRLAWEKYKVEKDEYVKTLPPKKPSGPFVDFINSQKD

RLTEKNHGKKLQMQELTRLCVEEWKNLDENTKLRYNLRHKEKIDEWIKAYNDIDVKEMKE

LMKMKQDMLEAKNLAIEKNRIYQEELAADLERQERIKKLEQRALDDFNRMENMGP

>34-9_0288

MFNFGRKPKSLQNNVPDIKVSSGAGIQRQAQCSTLPNQQQSNAQGPRNKVELLPDHGPLN

WLQLYEKQNVQIKVVLMMVITILLSVFENDDFKTLFIKDNELKNTEQVDFLIYLHETVFA

KNTSNYKLLHNIQLKNDFRKEKVSIFEKQQDEDQDVYNPIFEQILKVIDMPTLIRLFSFK

PSLPTTQLGFLKTSSPKGLKLRPEYVLNNYNLKNVNLFWICLDKRIYYITPYLLYHPGGD

AILLKLLTSSRNTVERIDKMKEFQRYHRWVNETDLLSSYIIGYL

>34-9_0289

MVATNSIKLLAPDIHRNLAELVSKRLSMPLTPCKLKRDSNNEITFSIGESVRDQDIFIIT

QIGSGNVNNKVLELLIMINACKTASARRITAIIPNFPYARQDRKDKSRAPITAKLVADML

TTSGCDHVITMDLHASQIQGFFDIPVDNLYAEPSVIRYIQNNYMKNQQDIIMISPDAGGA

KRAASLADRLDLNFALIHKERAKANEVSRMVLVGDVTNKICIIVDDMADTCGTLAKAAEV

LLEHGASKVLAIVTHGLLSGNAINNINNSKLDKVVCTNTVPFEEKMEICSKLDTIDVSGV

LAEAIRRLHNGESISYLFKNYPL

>34-9_0290

MEGKKVIKCCLFDMDGLLINTEDIYTLSTTKLLAEYGKPAMSWDFKMQVMGLPGPEVFKK

FIDYYDLKGKITYEEYAQKQHFYQEKAWNECDYLPGVQSFLKYLMEEKKVPCAICTSSDD

KKFQLKTIRNLKQDGMIDQFVHVIKGDDPRLANGLGKPHPRIYELGLAGINEKLGLDIKP

EETLVFEDALNGCKAGKAFGGTVCWIPHTEIFGKEEIPEFKTFFKDENEGFVLQSMDKFP

KHKFDFQNI

>34-9_0291

MSVDPLYITPHETALAVIATSMKKSRLTLQTLIINSVMGGVLFSSGGMLYSIAVGNNPEL

SVNNPGLLSILGGLFFGIGLFYVIINGCDLFNSNILFFTVGLLRNAVNVYDLTISWVVSW

LGNLAGSLFMSYVICHLSGSMKSENLKQGTIDIAEFKLSFSFVEVFLKGVAGNFFVCLAV

YLQLMSKPMHVKFLTIILPVFTFVTSGFTHTVAEMFLIPTGMFNGANISVKLFIWKNLIP

ASVGNIVGGMLFSLTIPYYLHLYVVEQDRKLLQLPEYDAFDEQPEINVDSRVVKAKPRRM

IHKIKKINRKGNKKSKYNSSSETSSPSSKSASYINPPGVFPIEGFIPLTKEKTIMNGETE

DEEKNMNDDDYEDDNSEDMSSVEIRTEDENTNEDGDVEYDLVEPYTPDQTALNLPETYNS

YSNQDLADMDVVSTMDSLDLENQQIKANKSQIKDMLKEKEKNESEEYEKTGRYNPEKNKL

GTKLSKIFTSVNEDVAKSKKMHTLEKLKKTLSNSSNKLISKKNDSTESKLNALPSKNNKG

SSQPPVDKDPAQSRRSSLASSRHSLSSKAKFRRELKNQNMTNKALMMADPVAGSVDLDES

FVQRPSRLRRKYSYHRHQSPAALMRNYGYDNDNSEEQSIGD

>34-9_0292

MSDRSLNIALHRYHHILNQSFDLPDKKLVDIFFILLSNHIQQGKQSDLDLNLLYIYLNKV

FFKLANNDQNQKEKLFLTNNEIQVILQNIIQNREAINDDFLIFQILESILIFYKELRYIN

DDIFYFQLISLIIVKIVFKDFEKDVMPLSSGVTFLSSILIISKKEVLSKRDLHKIAPGII

TKLIKVLSNNWKHDTNYLKVALTLLTQTILKCKEEFSKNENILIYQSLDSMINKNWTVNY

KSTNTKVDSIILQDFIIPLKDFELVSELLIIEYLYRYESYSLELQKQEVFDQYSITNVLC

EKIKMIDVLYFDLKTFKIFKNGLMKLSQAMNGEERNYFNKQFNLFVEYGYTKPQKQSFLN

QKEVQLPSHISNNTLQNKELSVKFMVNEKVNSFAQIDNQLLDMVLNGEIKSKWLELAEVW

QVFFNQKDILEIMNEYSMSSDDELNDKRIYLYYYMFFQMANVTDTTEKQNHTNNDDIIKN

LDSINIDDFLTFSDDDEFLTKDLKVQNSPAEVTIQDEDEIMLDQCLQLIDVSPSQIFTIS

DAYFICGYISSSYTCVKDLQDLETELIPLLLTNIDNPVSQYSLQILSNRIYPNKQLSLSF

QNMIKRNTDVILSHVSSIISSSSMSNQQMWIVFTKNVFQVCGIEVVSKADDVFNLMFELM

AMKSLNPSDDGHEMTSILELFSVVFDLIIDQYKNLINNESSAKVSRTTLKDVVDLFKNHS

YMYDSDEEDDIEEINAVVPAENQEVSLADEPAYTGEIPEHIYKLVIELLGYTERIYPLLS

ESSVKSYTTLLNNIKKTCLILNTSAKNYLPQLAQVIYPFVERIVKSGILGTKRVGSFVAD

PVPFGRISFFGFFHR

>34-9_0293

MLVKEAPKAVVSNNNFLPTNIIDKTSSPPQQKQTKRNISHACINCQKKKCKCIPIENLEL

PDKLLCETCKQSKKLYCEFRDQKKRGPSSIDTGEIDKSVRNFKNESVIRKDDINIIKGVL

NKHKNNKTMTKGKINKETIHLPLPPTSNLFPASRNITPSTYQAAQMTYFQNFNSAGLQTP

NKFNNPLLSSIPNITKPPTHKHPDTEWIHESRNVSTFLLNPSVESDTLETQDTKHIQREK

STEKLFEFLLYGYNNNPQKNNPHYEVKRKISDLLNSLKKKTVDDIYDDVVTLENLLSEYL

TK

>34-9_0294

MTSLEKRPSPMQRQASIIDENATANDLVIHQMYKYLNSGDFKESVEMKSFYQLFKRYILS

KDIKSEVSWDEIKSPTHKEVVDYNELTDKDLDASKILSKLAVLKLNGGLGTSMGCVGPKS

VIEVRDGNSFLDLSVKQIDFLNTKYKSNVPLLLMNSFNTNNDTQQIIKKYSNSNCKIRSF

NQSKFPRVYKDSLLPVASDMDAELDSWYPPGHGDLFESLYNSGELDELINNGTEVLFVSN

GDNLGATVDLNILNYMLETGSEYIMELTDKTRADVKGGTLITKNGKVELLEVAQVPKEHI

DDFKNIRKFKYFNTNNLWINLKAMKQLIVEQKLKMEIIPNVKSVVRNGHEIPVLQLETAV

GAAIRFFSNAHGVVVPRSRFLPVKTCSDLLMVKSNLFNLEDGCSLTLTPERFFGGNPLIK

LGSHFKKLKSFQERIPHIPKIVELDHLTISGNVFLGKNVQLKGTVIIVCSEGERIDIPNG

SVLENVVVTGNLQILEH

>34-9_0295

MLLNKFINKRYYSSLNSDKLVLGIDLGTTNSLASHIPKSLANAKTHAPTILTTTKTPSIV

GFAKKNNLEGISTDLTNDKHLFNQLKENLSVFIGDQAQNQYRLNPENTFKAMKRIIGTFN

ENDEIFESYKNQYSNNVSLNNSVYFTLFKDSKLKVNAEIIAGFILSEIKAKALQELNMEN

AKVDAVITVPAYFNNLQRKATLNAADLADINCLRIINEPTAAALSFGVLKEQNKDGVYAV

YDLGGGTFDISILELDGAVFEVRGTSGDLKLGGEDLDYLIRDYVLDKFLKLNDNINKNDC

KIELSHKTTSEINIPFAFKNSHGDYIHFKMEIKESEIDKLAEPLVKKTIKIFKKCLKDAK

IDKQSLQQILLVGGMTRMPYIRKQIIKNFISDNNSEIDFDNKLNYKINPDDSVCLGASIQ

AGLLTGEIKDVLLLDVNPLTLGMETYGGLMSPMLQKNCNVPIEYKEQFTTGIDNQQIVKI

NIYQGESKLCRDNVKLGEFVLSGIPQLPKGVPKIEVSYKLDSNGILNVTARELLTNLECN

LELITTAPIENNYKKQVNSDVDVNDLSWVFENLGLWQLSFKELDLQFRQYTGTVHFSENI

ANEFNDLKAKFAAWETMKEDSSKDHEMVEFFAKKGGMNRLKARINKLQTDFMSELKGNKV

>34-9_0296

MSGKKQRFIVSNTPIDDLESYHKYLSKEIGSKDHGKTSEDSNELRGILNDMKKVGFNNLK

NEAKTSLCLAILADVIYSSTKSNSFTATDIKTFFLTLLREVRNNALENNLADYVLCKYAI

NKIIACDAVLLILHTKECSQILKEIYHLNDDISDDIKARELKDAAVKLITSLIIEDSEKE

NNEFLDSHCKQFMNEIIYENEICKETIQVNYQIFQSSIQSFYNEYILTDDGTFDLKNVKN

NVLEQIDSFTHKGFIKLNKLGNLFKKLYIINPALILYCNGLFQTLMNSPNKYLRILGIRM

VKTLITENLYFIYNHDFIFKIYLTRMSDESHLVRLEILNGDFAKMCESIYQNDKEIFNYI

EKILEKGLSDINADIRLETVTQIYKGSIDLQYNANVYDLIIGLCRDKNLAIRGRSCTYLS

KLALSSKVKNIDGLEAKIVNTLVDLYYINDQDLNDVIDTFLSDYIAYDNKNLADLYFMLK

EEKSKHALLALLPRYKIFNKILLKFIQLVIKDEASDDSLEFTKLLRWLKINYNRNDIELF

TSLINHDVAVKIRPFLTNRKSFTNAEDKKNFYLDLKNEGITSNEYIKKFKVLMIRCTNTF

INLEDVYEKPNENEKFFDVLLSNKQVLQNLIDLERLIDSFKNNKLSTAIKRILMLKEVLP

NMELSQDILNYFNQFVESDNKLDEYEMYGLSEILPKEDFSAFEFTQSKVTALDSITLTEC

NLMSKSPREHEEMIDSLLDLALTTDINESITIEEFEDERDEIEQIELADQFSNKSEIDPK

NNNLLKIILLMRTRSDNLPFLKSLLETQGCCIKKSECPEQPQFWRWIIMNELINLYLSKC

LDSALYFEDFLGESQIHLQWYAIDIHRYVRQKFFDFLITNFKELPMSVSFFIFYIDMSLD

GSNIFNTYVTFLENIIADQTFNKDRYFERLISRFLHNLSYLPTVSVLNPERAEDKEQDIE

EQKHEVLEDLIAKISLFLRFVMTKENCTLILGYCNTIFNHKDKLNEDNKDIYLISDICIT

MINQVIKMKGWVDLKVTDEVINDKIKLPLDLYVKKENTKDEIIKSIKELLSTEDKEFISK

LSNKNKNLMKIDSHLQNTIVNSTLSKHNDISNALPSKKKINDLEKSTADVNEGVRKSKRV

KRDVNYDENISTT

>34-9_0297

MINDLYKNEFENLRKLDYKQPLLNITGFLYTLDDLEVNKNDYNTNLISNNTSNNWFGYPI

ENKNYDYTFDIKEQSNNYLPQNLLNRIFSDGDSSIWKDNSDTYIYQSNVSSTTLRGLFYS

RNIQDEDKVHMPIPQFMKHKNESKDANGEEQILPDRGSFFINDTTLFEQSMNINSTDSNL

INMDISTTVKLEINDFFNYPGLKPGSLQFHSAELSKSSARFTSLISKLLIVYDSQTGRLF

GITNSGKFHGMLMIPQLFSNNENEFNRYKEDTISFVNHTYFEGNKRSNNTFTIDDILILN

ERSSNPEFLLYAQTKPVSERPADKNKILNTVDLNNLYGKDHNLLIEDMLIYSPNFGLSLE

TASSNKILAKKNFKGVLGSLFPIYLQESTMKYVTLPFILYSIVFLMQISKLDSPSELNKI

SIDFLKFITFTDCFVGGVFIVLGIVVTTYSDLTPNVSLHYADYLTKPIVNSFVKVKDIIV

NGNDNLVYDLSKNIRTTQFSWFAINVVLFMGMGIAIEAKVLVLCIQSQFLERSISWLSLF

RRFTPMSVTTRTSQEEHAATSEPNTNPTGENNEPTSEPNEENNFLTNNTEGSEDFTFLYS

KIQGRIYTFMVLFAFAIVFIYSMNIFLRHLICNLVVLVYTSLFWSQISRFILIDLDPMTT

NLQSRFVIVTGLTRLIPIGYFYLNENNFFQHQTDINWFIVYCINISVQVVLLLIQLKYGG

RSIISDKLERVIEFILSNGSKSIKHRYNYSKSVTKEQLAHLGEKNEFICPICMDNDDIIH

LKVEDQTSSTEEADTEETQLLEEINVGNDHVESYMITPCNHVYHHDCLSYWMNNKLICPV

CRNNLPPL

>34-9_0298

MYKYNPVRDSSSSITTILPVNNSNTNTTSLLKKSTENKNTYEHQGTVRNKDPTSDGNLII

TINDKDKLKISFIDIRLNSLYKDMLIIEQPELLMNDFTNDNYMINISGNIIISCIKPLIL

LKPISLELKGTFKLDFIEINNDINSKKKDDDDGRDESVVVVKENRPFFLTKWDNVIDYPD

GEVVDICKNQYESDLIKLKESYRKKKKNKNKKNKLKTQSMYNIFGMRFEENGNSSDEEDN

ASLPLTPVNTRDNVFNSKKPSLKTIYQSAPDLHQIIPTGGISQGFRPTIKTNNSFRFLDP

GNMDNVQIDIGETPDDTTILDSEAHAENKENKIYMNPGNYEFPFRLKVNQRDMPETIEGL

QAGSLTYMLTMKMYCENYGVVEHVKYLRVLKTLKANNINIHTGFKFKHDLKAKQKKVVRV

EKDKQDKSKEIMEGLKVNADELSKAPITATSTNADTALSTSLTTDENTEVVDEFVTQLQM

HIETPSKAYPLGGSIPLKFQIAPLIKNFKIDSIKISLNQKSILWDSNNEKYTIEKTIKDW

YIDEFQGISGIEKVYLKDENAKAETKDKDSLAKKKKINKLKILSKNNDKEKKSNEGAKGY

EKSFEYRLCDSDIEFLYLLPVPNNLKEITQNSLISENFYNGDKLNSHLLLNESLEDEQEF

EKKYGYKDYKVLKLPRISNTHNLKVQIYVLKKNKKIPIEFSIPLMLFCSPNVQIQMRKVL

LDKFKRIHFRKGEVELFFKDLPKGIDYTNDDSTTISEGGNTHEDTRNLMYSFIPRYFPQL

QQVQPPPIYADSSKDEQVFTSMNKKEGIENSKVVEDINKNPPSYENFRFDGSNYANRSDH

QKIMKSERLTRDYYYGIYRDDGSNTANRNKSRKFKEGVLQYEEVSPQYKNV

>34-9_0299

MSRRAPSSNVSSERACMLCGLVQSLNEFKSTSCPNCSSIFDNYKPNEPLDLKIYDCISPS

FEGLICIMKPEKSWCSKWLRFNKYVPGMYAVKINGRLPEEILADLPHFVPRDGSVQWESL

KTILTIEIYTSLHLRLTRKNKKEEDMSSSSVINIDKEQALKATADLAEMPLPLNDDKLTH

RIIFPAVPKFINEIQEKSGLPYYAIIPITTLSIKLFVTLPISIWNRKLLVKQNELRAFTK

SISPVMKMKLSAHNSASGHNLKPEQIRMLSMKEQRKMQKKVFKENGIQVWKNILQPVSQL

PIWMAFTYGIRELPAYVAQFTKPKENVPADKIDPSIASTTNMGPSDGEVVKFGYSPLDDL

FPIFDVQGLNITPILSSSLLGSLTLLNIQHYDKINQSRQWYDIGKEHNGKLLRNGIMTVS

RFISLGLVFTSFQQPDLVVLYWIVSQLGQYGVNKLVNRYPFEKKLV

>34-9_0300

MAQGKLALNKNKKDTSKKYRVLKQNRQAKCFQKKVYTPKNNTNDKKTIFNINKKYSNNNL

ILTEKLISGKVGHLEILKGTRKEIQKEKNKAAVANGSKK

>34-9_0301

MTILAVTGPIAFSDFRIANIVSELSSKINVSTDKIAIDSSFIHYIELINNNTTLSEQEKN

HLFELLKYDPFPTNEVLYNIINDDLASLPQENSQSFTLRIIPRQGTISPWSSKATNIVQV

CGLEDVVKRVERGILLNITVTSTEKVSITASDLTSCYDRMTQDLFINDKPDLKTYFHQTG

SPRPLQTIEFHKLPTYKEKYDLLNSYNKVRGLALDDGEMKYLIESFVDRSPTDVELFSFA

QVNSEHCRHKIFNASWEIDGIQKPMTLFQMIKNTFKKTSDFVVSAYSDNAAVMDLTHTKD

EFYMSPQYSSKLWNQQLEQVHLLMKVETHNHPTAISPFPGASTGSGGEIRDEGATGRGSK

TKCGLSGFAVSNLNIPGFIQPWEKKFLSEEDKLGKPSHVASALDIMIEAPLGSAAFNNEF

GRPNINGYFRTLTLETFIEDKDNKTSGVKRDIRGFHKPIMLAGGMGSIRPSFSVKDTPIR

PNDLLIVLGGQGMLIGLGGGAASSGNTEDGNADLDFASVQRGNPEMERRCQEVINACVSL

GPNKTTDCKCDKPQCSISCSSQKGNPILSIHDVGAGGLSNALPELVHDNNLGAIFDLRKI

LTLEPNMSPLEIWCNESQERYVLGIDPKDLPLFEEICLRERAPFAVVGHATAEQKLVLND

PLLNSTPIDLEMDILFGKPPKMSKSAVTSDLILKTADLSTIKFADALEKVLSLPAVASKS

FLITIGDRTVTGLIDRDQFVGPYQTPVADCGITATSLYPATSSINDISHAQGEALSMGER

PTLALISAAGSAKMSIAESLLNILSADIKSLKNCKISANWMSPASHQGEGAKLYEAVQAI

GIDLCPDLDLAIPVGKDSMSMKMKWKDSNTSESKEVTAPLSLNITCFSAVNDTTRTWTPE

LNTKNTTKGSLVYVDLANGYKTLGGSALLQVYNQLGNECPTVRENLLLKNFINASIELHQ

QDIVESYHDISDGGLITTLLEMAFTSRVGIDLELPIKEEKDLISYLFNEELGAVFQVSDY

AKFVEILNKFNIKSDLIHQIGTINSASNNLTIKNNENKIIHESSIVSHLKTWYETSYQIQ

KLRDNPVSATEEFNNLDSFNATNGIQYKIPYKVGPQEAALLDASRKPKVAILREQGVNGH

YEMAWSFQQAGFDTYDVTMTDLLSKKVVLKDFVGLAACGGFSYGDVLGAGNGWATSVLYN

DYLREEFNDFFNVRKNTFAIGACNGCQFLTRLGDLINGNKDSNKWPIFTRNRSEQYEARV

CVIKVAENIDDKVIFFKDMEGAEIPIAVAHGEGQATFINKSVDSFEAADLCPIRYVDYNG

EVSQVHPYNPNGSPNGIAGCVSENGRVLSMMPHPERVVRLEANSYYPKEGTEEWKGYGPW

IQLFKNCRKWVADNY

>34-9_0302

MGLTEALNAAVFPSNSTLTNLLINNDKQNPLSFNQVLNNVYDIQPHLSTIEKYWASWYMY

MNNDVLATGIMFFVLHEFMYFFRCLPWFIIDNLKWCQKYKIQPTKKPSYKEQMHCLKSVL

LSHFLVEAIPIWTFHPMCEKLGITMEVPFPSWKKMAMEISLFFFLEDTWHYWAHRLFHFG

IFYKYIHKQHHRYAAPFGLAAEYAHPLETLSLGFGTVGFPIIYCLFSKNLHLFTLCLWIT

LRLFQAVDSHSGYDFPWSLNKFLPFWAGAEHHDLHHHYFIGNYASSFRWWDYYLDTEAGP

EAKMEREERMKRKSE

>34-9_0303

MNTRDLNTPVSFNSHIDRLNSTATQSDYEENTAKPALANSSSVSCELVLGADLSYQFTRI

TNQLNQQQLNSSVIEPVNVTNDVITPVDGLSIGSQDDASDLGSISKETFNMYKALNDNSD

IIDSANSVVPIKFPSGTAGDNSESFSGVSLSSKAGFLKKSSSKIESAFGKASTTLAASAN

NLAKQSPMAKIFTGISFESFKFKELEDRVMNYNLVDVDLGDKGNSALSAEKSTVDKSFKT

APQSTLSNNKSSTQHNLWKMNEGQIYIESQSLAKLNPDLEIGLENLPKQRLSNVFKHSNK

AVLCKKNIGSNNLVNLMVVGQQGMGKSTFVNTLFQSEILKTGSDFKNEYFDVGSKVAKKR

IFTEYADPLNKDTPSTTTIQKTYVMLEENKVNLKLCIIDTPGFGDYSDNSFSWVPICEYI

DSQYTNYMFNEEQPYRDLLLDSRVHCCLYFINPTNKGLTALDVIAMQEISKRVNLVPVIA

KCDGLTETEFFHFKKDIKEIIKVQKIKICDLLCSENQVDYSEEVHENEKCLANMEEIYDY

PFGVVGGTYFEDKQTFGRMYRHGFVDVMDENVSDFVKLRKFLVEDKTLDLIEKTNMYYEE

WRDKLCMLRYKKVNEVIKMKELEAQNDKEEYEEGAVDNLLIPNIDDDKFNKMKGMVLEGD

GINSNKLRVYEVYEVINKKYLDRHMIDWDPIFIHKQWELKKAFSSAVYEQDKKFKEWKRN

LFNKQSKYNEEIDALVTKVKSYQRECSKIEETINKMRMGKQNNVKAT

>34-9_0304

MRRKVNYSQGDNLPLYATPQQSLQQQKLIDKKNKQQEEDEEMKKMRLKQELFLQKKQQQT

REQLLSKMNAAGSSQRASTGTSSNNSRNTYSSSNTQKQSTNYRSSYQASQKHQPVPVSNQ

TLYKSQTTNKNDKYTIPGGLENSRQTSINSQGSNHYQPQQHQFSQTYHQPESNTQPNYYI

AQDLSFTNMANSGVLGSTMNDQVTNSGYTRPPPPLNVSTAGYGYSQDGNFVSPMGSSPSS

SSTLSNNIMMKNLSLNGRDPNSSMLSSGSNNYNELITEQLTYSNLHKGNSAGSHEANIYT

QHGEDPKEVASKLFYKYDVTKSGRLTTNELQQVLQNDDHSKFQKSSIDSIINLFGVSRFN

TLNLNEFVYMYQKLNKWRNVYIRHDTNGSHTLTTLEFTESVKSMGYKIPIEVVENLFEQF

ADLIGGSKCLKFDRFVESVIWLIRLTKVFRQFDVENNGVGVIEYKDFIDVVLYLGKFLPK

>34-9_0305

MTTNLNFKKGKRRIKSTVKKFNPQVDIINSQLADDILKLNGMHDSLHLQKKVRDEVSSNE

TLHVSENSERNVNMKEDDLPFASLKTLEILKQNDLLMSQLSKEFNFFSRPSIVDQINLLK

FPTSFTCKQNNCNMSFITELNLKRHVVLEHLNCGIKENINSINLICNKCVQEKMCMFLFT

DLHEYLTHYRRHESKEFCGFINHIFVDLNREVDGSNFMMLGNNDILSEKYSFVVKRNEIF

EINQMLARLRTNQGLDTWQIEQM

>34-9_0306

MLIPREERTKIHAALFNCIHVYENRFTLRLNVKADERDYDKEVAKRTIQNGKKAGVLVAK

KDFEIQHDEIATKNLYVIKAMQSLTSKGFVKTQFSWQYYYYTLTAEGVEYLREYLHLGEN

VVPSTHNANANANPAQNKKY

>34-9_0307

MESYNTGEDGDSNENLHLKTFKNTPSNTIILPDKTKPSKRKSLQALISIEPEPIDRSNTN

NLIDENDGIIQPEPNFIPMINSLLMNSSDFRDNSSSYRNNDSDMDNQTTFITEFDSIDNN

STINRLFFNKNYNNKVMSVLLKLRNKVNRINVQRIPKAGGRVSYLLLFLLFLLGGLNFIY

FKDYRTKLMKKNRLFATVIANGLLNGFSDIVAQLITCFSSRANNIRLSGDLLDARITNSN

QDNSDALDLDDLEYQNSLIDNDSIYLLDSIEHTNDVQLWRLLCFTFWGSFMANFQVPWYI

ILNKLFTEDPSIVQTFERILSDQLFYSPISLYCFFAFANYIMDAGDVYTFRNKIKKIYLS

TLAINYLLWPVMQFINFSFIPNDDQPLFSSYVSVVWNIVLSLRNASTK

>34-9_0308

MADQKRFVQDNTNHTLRSFASSRSGNSYYSETDDPYHYDDNENGNIIDIKHHRKRRESNL

SAIPNSNENQDSSFNNHMAFNYFNDGDIINASSYTSTQSNMDIIFQNESDFVVHSDSDSQ

LNVRLGNIDVRHRRRNTSNTFQRPPTTYGSIHSYPLSTTSSNDSNYPSSTASSLFDQAES

VLGPSSGEYVFGNEEDEDDNLLPHGHYNTNQGRHSNGSMNDLGNFRRRSSTNSHYELKDL

SKLGRDDLFRLFKFNSKHKQTQKTSLQRLYISEEDLVLGIQGLSTTLLKTVMFYILSVLS

LGILPVLCHWFPKLRIVFQYNKSELFCSNKVLIETELGDLDIIPVSKEWYGNKKINTVFA

YPEEKYLGYSLKVLSYFNYRFFRLYYDPLEDMFKTNKDWYDKENWTDYDLVQEGVFTEEG

TIDDRIKLFGHNSCQLEVKSIMELIFQEALHPFFVFQIFSMILWAYDDYIYYALCILIIS

VLSVAQTVFETRQSSLKLQSLAHTESSARVFRNEFWMSIPSTEIIPGDIIDISDPNLKNI

PCDCLLLSGQVLVNESMLSGESIPINKTSCNSSFLLKSFFDKSLSKESKYKLYNGTEIIK

TKATESNPQVTALCLTTGFNTVKGSLIRSIVFPSTSNKDNGKDMTKQAYKYIFYMTLLAL

LGFMISAVNFKRLHLPTKLIIVRALDIVTIVVPPGLPATLSVAVTFSISRLKNKCKVFCI

KPTKINSAGGVSCWVFDKTGTLTGEGLVVKGIIENSESIVNIEKDNYEGAVISSINDLSM

KMLQYCLACCHGNTLVRDPNNGNTEIIGDPLDVEMFNFTNSKMSETVHDDGFLINNKYNV

FKVFEFDSHLRRMGVLMEHVSKKNTFFSLVKGSPESILNLCNPHTIPHNYDSLVKHYAHQ

GYRLIALAGNTINLSSGHDAEYTREYCEQNLMFLGFIIFENKIKPQSRPVIMELKNAGIP

SIMCTGDNILTAISVSKEASILRNEEYCFVPIFNEDQEKLQWECVDNPDIKLDSDSLQPL

DQDVVEKGFSLAVTGDAFNEIFGDHVKLSTDNEEEIEETHDAHFIKYSNHYKSVVLMKGK

VYARMSPDDKEDLVNNLQDLDFNVGFCGDGANDVGALRSADCGVSLSEAEASVAAPFTSQ

IFDISCVLNIMKEGRCSIVTSFSCFQYMSLYSAIQFITVTVLYSQGSNLGDLQYLFIDLL

LIIPLAITMSWSAPNYGPLVSKRPSMNLVSSKILVPIVINVFLILLGQMIPWYISKFMKW

YVKPVVGGQEDINSTDNTVLFLVSSFQYIFMSVVLTQGPPYRESIFDNHAYISNVFACIV

ATLALMFGKYNNMFGCWMQLTELSFAFKTFILSWCILEFFGHEWLPKMFNKRFKKKVSSK

RYKRILQREKYMSV

>34-9_0309

MVVQGNKKRSFEDTNPLTKADNTQELTKSKLVREEENAFPRGGASVLTPLELKQVANEAA

SDVLFSNNTEEETTNAPKKRKTVNAIEDNEEGDDEDMRKKFQHLTYKTIPKNSNLLGRLT

KVTDFGLTFEFEDGLLGYCSIENISSNILPEDVDMDENSDVSDASDEEYEDEKEDKTEEV

KLQNLFPVGNYYTCKVIENHVLQNSTKKGKKKLTVTINPEDINKEVDDTDILQKGATIQG

SIKTIEDHGCVIDLGLEDTKISAFLPKKEFNEEEQDLIKKGNVILFTVLKTSDRVVTLTR

QASKELRTCDTISNIESVLPGAYVKFLVDSVDAKQGVFGKIFGSIPAILSLAQLNNSSVD

QFEIGENYPVRVISKIDYKNFAFMHVSQANTIVNLSASKDNVASAFPIGFKFEEPLKIIL

RDSSYIYVKLNDKINGCIHLSKIEGDITAKDKQIKESRIVSYHQFNEMFVLTNLKSQIEV

EYLTIRDVPVGTILTGEVREVDAKKGVSLKLMNGFIGKVLMNDLSDIKLNYPERKFKIGT

KVKGRVLSINLKNGQVMMTLRKQLVNGFQDKDNTKIITGFEGLNEGEFSLATVDSFNNYG

VTVKFFNNTRGFIPKNEVSDAFVKDASKFLKVNQIVKVKILQVDESKNRIIASCKLNSKE

EVEEVNNILDSLVLGRSVEECIVVEKTKESIIVELLKNRLIRGVVYVGHLSDSRIEMNRN

LIKKITIGDQIQALVIDKDAKTKIINFSLKQSLITSASEEKLPVSFEQVAEAGPETAMFG

YIKSISQRGIFVGFNGKFVGLVLPSYTGLSREEDMSSKFYAEQSVKVYLLKTDPEQQRFL

LSFIPPKSESKKSPKNIIEVINPVDESVKTLQDLTIGNKIKVQIKSNKRKQINVSVSDNI

HGRIDLTQLGKSIEEINSIKQSIEIGQVIEAYIVGQHDVKAAKYLPITNNLDKNALIEFS

LTNESLKSFEDFKVDEEIIGYVNNTTENLRWITLSPSVKGKVTKKYLKNSASEIAQLSNY

KIIEIDNEHKILNLEPVLNTLDLTSLNSGDKLEANVIAIEDSFMLFRISGFEQEAIAHIT

DILNNFESNKISKEKYSIGMKVSVFVKNVDIEKKRLAVTLLKNNKSQVELASLTRGSKLS

VLVKKITNAGVFVYVSNTLDGFIPVSLISDSYLKEWKRQFKPLQVIKTVVIRAEKADNIT

LSSRESDLTTENVDDLGKRRGKFGKEFADVEVGKVYDGVVRGVTDFGVFINLEGFDKVSG

LAHKMEISDDSEALKDRDLNDLFGEGDVVRCFVIKKNEGKKQLSLSLKGSKVKAIADEED

EVAESDDEDVMDVEYNNESDSDEEEVEKTTIPELSEDGLSLTTGFDWTGKILDQVVSEDE

SDSDDEAVDFTKKTKASAKKVVDKTIALTTETPQSNADFERLILSDPNNSINWLNFTAFQ

LTVGEVTKAREILERALNTINYREEQEKLNIWVARLNLEFTFGNADSLNEVFIKACSYMD

DYVMHVKLIMIYTSAGKLDEADKLYTKAIKKFGAERLPIWEKYGEFLLSSEKKEEAHLLL

VKALKQLPKREHVTIVKKFCQLEYKYGDIEQGRTLMEGLLVDAAKRIDLWNVYIDLEIKY

NSKQKSNIEDLFERCVANPKINKKQAKFFFKKWVEYEESHKDVKMIDYVAAKASEYIRSS

Q

>34-9_0310

MSKPIVTKDTPHSIEICLEVRKMVYVLWCSIIFYNLYEAGIFYVALARAVLLFSHLKDVF

LYTSIIATKIQAYNESLLLGLKRNLIIQLVCMSPSHNDLLKEELRNNFCLNHPFKKVTYV

FISVIFHCIIISKVFFEHKLLILLCFLVFFEYRYNFNQFISISLKCQASSDDEQQLNKKV

YFNPGTPSFIKPLPTKPAFHLVNSFKLSKNTDDSSPYNNTLSTVSKKLTYSTKTNVKDLK

KYGPKPGNDINKSIFFDMTSKEGLGLWGEYLKKLNSNSKKTIKNISPMVEDFKIVDTKFD

IDIELEKMKESGQLKKYLDLQYRRVFITELRVWVTKNRLARVAG

>34-9_0311

MASSKCFRYLDRILRGVEIHNPTILEASLQPMASKFKEENKTVYIFEYNSVFAAQTSAFN

KIFEPKVHYLSINNTIVEKHINERFSLQNITRGKPWSMDNVINHRIFEFEKEWPFNKLKN

PVVICGSLEKTPARNFAFLRGWIFGYFFNTTNIINNQMKSLHLTDEAKVNEITAKEQKIF

EQNYSVTHNKFDDKVLTLYKHEMEENHQFLEILDLTYNESLNRKKVTQATNKQHKSQSLL

FPTRILSALALSTIGVEFYSLARNINTPTQLNHKQFELMYNTNKLVIVHADTALRVLSKK

YKDMSTDTEFSAFDFRSKLSFRMQIIYDIEPLTFISPKNPNNYTLFKRIKSIGKDATTED

FRKGNVVGAYSYESILSYLKKAYPEMRAIERVEKTDVTFKNYELDGPAVIMNLKPSEYLT

KNFDKIDFIKLRRVLSSIVYNPKRSILYNLNFLSTENVIKEIKERVDLEYYENTTAIDMD

KEKFFELYELFDEISSKWNLAYDDLEIVESDMDFE

>34-9_0312

MSDFVEQDKIGMDAAFEQAFISYKKENGIPIGACLINNLTHEVIGVGHNERIQKNSPTLH

GETSCLENCGRLKASVYKNCTLYTTLSPCIMCTGTVLLYGIKRIVVGEDKTFEGEIELLE

KRGGCEVIVTNDQRCIDIMDEFIKEKPEVWNEDIGEED

>34-9_0313

MSLPRIKLNLSNSIQGETNPLIESSIILRVSNNLNTGLLSKTDILELLNYDDCINIEYLG

LNKLILSVNMNIIGSESIEYYACELLNLPTVVEVYKSIDNGKNMLKNNNINQVIYIHKKI

NKYEDIFNIKVDHLNSELINEDSNKDPIYTDYHEKDYKEIVQLYDNLKKVDFKNNKKYDL

ILKNINQKNLINHSNILKNGLTPPLHNVVKRRQRRQLSLNQIKKLEYTLNKLVQNDLLSE

SVEYEFISESEIDNDKSEEHQNNVTVANDDHLKNQLKNVIQQQKDEESEESDLELELEKV

LLEQDEADEPLEEQAVVEQDEQDDEQDEEQEEDNQQNDFDEDDELTLLIDELKSLQNVKQ

TNLKSFHNCKNQFMKERILERIKKIDKEIEVIENKIKNMKQEDEGDGEDEEEEEDEDIDG

LFSDEEPENEANEGEKEQSEQVEEPKANTMEKDSDYYESDDDMLELFGGSENEDSFIDST

RVFSEEEINRALLQWQLLKQKLSPEQLANNPQFQFYTNFLRQAAAQKKKLLEQQQQQQQK

NNEVAVSPRSNPTSEQPPSDNEKLRTYNTRTGLPPNQDDILLTQTKYFQLLMQKKDIPVD

LKAFIENDANKCSIENYKPLVQHIGMRMFQLKQNQAGQTVSQNVNQQPKQGSQPLQQPPI

QSQQQVVSQPSPRVSNPMSAQGFGQQSPQGFSQQTPQGPNQQYPQTINHQQQQKMNQQPT

PNNATSAPNSAIDPRPVPENKMMETKIPIWKPIFDSKVSNLQVNDVMEPHLRVETFKLPD

TQEKALDKLKRINATDKHEDINIDDIDELEKFLIEKYRPSINSLQANKQNGTSYEDLDTF

KVYPSDLPGGIKLYKILELYQFSITLFKEYDLENCFTILMILDHYKKDYPGDEVDEVIAE

VEIRIKELQLHQVQKNLRGEIIQYQSFNKQLLLSKYNFKNNSLNNNMYGQYKNLDYSDIK

FVENSIAKIELENKERLLSSHKTELTDMMNVFQGSESSSAIDNLITKENNLKAQLKNHQQ

NNTFLLKEDVKRIERNAKKRLQALKENDEEAYVKLLDQTKDTRITRLLKQTNSFLQSLTN

AVKDQQDYTKDKIKQNTDIKVDYTNVVEDVNVQDDDEDNTDYYSVAHRVKEPILAQPTIL

IGGQLKDYQLKGLEWMISLYNNKLNGILADEMGLGKTVQTLSLLTYLYEHKGIKGPFLVL

VPLSTMTNWVNEFKRWAPVLNVIAYKGTPNERKNMQKRLKNLEFDVCLTTFEYIIREKAV

LSKIKWVHMIIDEGHRMKNAESKLSLTINKFYHTDYRLILTGTPLQNNLPELWALMNFVL

PKIFNSGKSFDDWFNTPFANTGSQDKLELTEEETLLIIRRLHKVLRPFLLRRLKKDVEKE

LPNKIEKVIKCKKTSLQQILYEQMLKYKKIYTSTDSTSKSLQAKGFNNQIMQLKKICNHP

FVFPQVEDSLMIEKGTVNNLIYRTSGKFALLKSILPKLKATNHRVLIFFQMTTVMDIMQD

FLNWLNIKHFRLDGGTKTDERTVMLNEFNAPDSEYFCFLLSTRAGGLGLNLQTADTVIIF

DSDWNPHQDLQAQDRAHRIGQKNEVRILRLITEDSVEEAILEKARSKLDLDGKVIQAGKF

DNKSTAEEQEFLLRDLMKKEEERQLKLQEKEKLKSQLKSGDLEENYEDDDFDNDEINELL

ARGKDDLETFKKMDIEARKEDLDQGIFTRMIQRKELPDIYNMDIALEIEKERKAEELKAL

DSISGGRLAKRKTVSYLDDDTKWLKQLEVSDNDDILDDEEEIDDELDGLELDEDGEIVEP

ADDQPVKRKQSVALEDVDVVEPAGVSVFDKGLCLINEYTAFRDNLPNKLQFEKSSIVQEM

FKTADETEKLIKEDEAILDVYPWPTDEEKRQILEENKETKPPVQSKYLLKGDSELCVKPN

NPCDHRHLLLIENKDLKKLLTKAKSLNVSLTALLYTFWNISFHQPEKEKTKTAFFIPADL

RGRINIGNDKYNLDEELIIPKNTVGCAISFYNNQAPIITDKECVLGSKEFVELANFYNTA

INDWVRTEKLSDLSFFKNVIGTTDQRKLFSGYTNLYNENKPKLLPSNYAMSNLGVNFVHK

KEYSINVEHSVFGQAMRVDDFVTSSIISSPKGMCYHILYRDNSEKFVSKVKDRFIKNLYD

FIA

>34-9_0314

MLTLSDVQKFYDYKEQHKQVSGKVYIGCELVADLKVNINILQDICKEIMLIHKQLCAQVN

ENHQVYLLDVNKLNFDDFVFVSDINTDINDYSVVKYLLEGPPNNGKINTPLWRLIYFEKM

NYLVFNFGHCFYDGISGVNVLKLILDRLNNKPIVNIDYDEFKDHVSDQSLLEYGIVDLYP

RPTYGLYDKLQIFKNKLFEGIVEPSMFRLINSFPRLLEFNYFKSFNRLYDPHVSPAKGKY

IDSYHCINISFMSTKKLIKISKLLNTTLNTLLLTLLSISSPKVNKHYANNSKFNIPINMR

PYLFKDDASELGSSLIALAISSTQITTPLINIDNILDNERINITGLQAIVDELDPLIKKS

INESITMENYSIASFYKNDLDKLLTQSPYIDFELSNLGLFKHPLIKDVIFNQGLEPMNYM

NISAIGGINGTNINFMWHHSINDFSKQRFVMFEKLITILCKQDIDNI

>34-9_0315

MSDLSNSEDEILSNTSSIEDEYQTPDEISSEEEFENENETDKRRRLAKQYLENIREEANE

IVNEQLMNKRKLIDTELDDSKQKKVKISNNIDEYNNFDAADLEREILENRLYKDNLSQEG

KMYRYLAADFKDNIKIIKKQFNRTADYLTSMDLFQENAITTLLDLENNNMLNSGNKPPFL

VTVSKDFIMTKYDISNFNTRIKKVKQIKIGDIKFKPKNLLTEHDNNTLEHYKNINSVVIS

PDGKNIVTGGMDRKLIIWSSETLNPIKGIPTKDRKGEIESMGFRNNSDQLFVACKDYKIR

VYNVKQFQQMDTLFGHQGPITDVSVLNMERCISVGSYDKSLMLWKLQDESRLVFKLPQKV

KSQEQLKQDFIKYKKDNKVKEDIKESIVMKDHKLPNFFPSPTTLDSCIMIDDTHFISSSD

DGNIQLWSTQKKKPLFQVPQAHGFMNTNDKIRYTAEQDHLQIKLQENIQHNKHFKISAMA

NIPYSDLLITGSDSGVLKIWKIDIVSNKITLIKNVHDVYGMVMSIKVLNINDQKFRLIVC

CSKEEKKGRWEKIGNKARNGIYDIVLEREGK

>34-9_0316

MTHKRVFIDDINILDIKFNKLLIVHSQFKAYSILINASNIKYIHYNCPLPLLISQNSIKN

IGISNNNTPWISTLDGFYSLVNSYNQNKSYWVNSLNYENEIYRLNGGFIIQQQDDGEEDP

DYLKARIYENHYILPVNLISDFDELILGYTINQNAVKLPNINTFNTSSTPITLVELQKNI

NNDDSLDDKNFNNLLNCEVDSKSTLQLPNYYRQVDSLVQNSIQSDIVNSTILENSLVNED

DSVQLLKNLNNTWDSSVLRLFDIKCQQNDIKCCYSLVLSLKQLKCLDKAKEISKMYGIYE

LSDKINDLIDSKTAV

>34-9_0317

MENYNNYEDIIHSIQLNNHDLNDINDVSNLLSEQSLYLNYNIDSLYEESIEYKYTNNNAN

LIDTYLSYIQNDDHNNIDLNILIISTQNLLNYIGENTSLNAKISTLINQLMDNVIMRIHK

ILNLLIYDSISISNVIDFIKNDIDDDQITILDDRLKDLKRQVNENLTKLDLDITDYPFLK

SII

>34-9_0318

MNDPRFELYDKENDLDIENNEEINNALKEIEKHKSEIAKLEGLISKWKKEIMRKRKLQGL

DNDSGNTMFKKLLESEKPKPSKKPMPASSMNYMLRKVHSFYNDRLFNGNSNNRLEYQTEE

VMDECVYTNTYISLRYIPLSVVEEILQTYTVLGFDKLFKLIRPPRYELSSEVRVDNFCIV

GIIHEILPIKFKQKEFFERSKIDIDKHTVRNSKAEAYSRNFSIVLTDLKHTLLLTIHGNK

LIKEYYNSLKAGDIIMVETPNVYRYSNKFGFSIDESGGQNQIMELGKCANYSKCGMDLKD

GRKCSSFYDNRNHKCCEYHEEIDMDVKLSRRMELNSGYLGRRIGPQEKIRNAETIGQKDL

KFKNQRSFNRFHNSIEYEDTVKERQFEATKEAANEE

>34-9_0319

MSGFNFFGDQKDMNSEENKNKANTPIEFSSSEAEDKKEDVNPLDMFADSALADANNDDVP

LIGEEEEVSYTEGSNASEDDEDYDDVEDGEEFISEDEDNNNEGEEEEEKEGDDNDEYSNS

DGDSSVSNGGIVEKALEVAAKSEEEPITHFDQILSDVSKIKNDNKEQELSEKNTFLLNTV

MKLSQQASHFKNRVSENILENHQLVDDFSQKLAKKDAALKNLQVDIEELEQDVKESEALK

NSLKKKYDELKKSKNIEIQSLHEDLIRAESSNKQLHSEMEIKVEHTNKDQITIGKLEEDN

TKLRKDIEILGYKMNTTEETLKSEIEFKNLHISKLQNQIQSFHQIGVKNNVSVDDYNQLV

ESFKESTDEIDRLKDIIDDREHDLKMLLTKNITESTDDFNSIDILKKELMLEKLEKDTLT

KQIEEIVKGLENTLNESKDYKQVIDTLETQLLNSNALLESIENDRNDIFNENIKDKATVE

KLEKILAERDQLITDLSSQVQYVLLQLTLRTDSDSLSILSKDELNKIKKLTKMNRNKSGS

NGNMNIQDVISDNLVLFKDIDNLQKQNQNLVVSLRELGYKLESLQSIKESQELNNDLNSK

TTELQQQIDSLENKKLISEQELENLQSLIETNKDYKSKAQTDEIVKNYKVQIEHLEKQLE

LALDKVLKSSEESSKSLIDNMTKITELNVEIGDLKTLNKTSEYQLKQALQDKESLSSLLK

KEETLRDELHKHNTDLLIRNKEWETSLMETQESLKNAEAENINLNKTLKSVTVDNQSIQN

KNVKLSNQVSEMNRKMDNIERLRKDMEYLLRHSDEDYWENLALLEKKIKNNEDKEVVNSE

NNYDSVKHILNAFEKHMAYHSNQASELRKLLQEKNDIIEKLNVDSRVSDLVAELDIKKNE

YEELQKVLDSYKQEYSEEKTRLELSNNENTQQLNHLQQEIVKLNGDIQNRDTSISELNDK

IVTLKNEVEESKKSLESLNVEFKQKVRNLEIDYDELEHTCKEYEERNKSLSDEKDEAVKS

LEELAKKDEAAKSLEEVAKKDEEITALKEQIDALKGELSTKETPETNEDEVNSLKKEIED

LKKQIEAKNETEKVDEDAVNDKQVEEVLRKEVESLSTKKIELEEKVKSLEAENREQRASV

LKVKLLEQKLKMLSNNAPAVEKSASSAAPLAKEPATTPAFSFNIPKESDVSSTNTFAMNS

IPQKQAPAFLGHDESKDSSSTANNSTKRALENENDEGEKRVKKEESKESSIGKELSSEKE

EIKNVNEFKFGFGKPEEEASKKNDFRFDFDQKDDASKKNDFKFGFGNPETDKKNDFKFGF

GQKDEASKNSVFGFPAGNAFNGSANSNPFGNMNNNQNAFSFGSKPSRFESFTKKDENKKE

ESEEKNIQPPVATKDQRMSHSGAALFRKSAGILTIDESKYPARIHWKSTDGTQVHTIILD

QVSKLKATPANSTKMMIKLIGKLTKEEMDEGIIDPVSVFQFNNRMVMENIKNTLQIIISR

YKDNVVYEQKMLKEKEHENENNMNLINTESLEDSLSVSNLLSNLQLQQNYLKQDLALSKK

FQNSVIKNKLPAKEFWITRITELRSFALSNSQKFGPYNVLSTIKPVASSDNKVNVSVTKD

KLKAILATYPVVKLAFDENVPLNFHEQEFWSRFFQSNLFRKLRGELIMANTRGDIIIDKY

LENEITLNSKEDEHNMNKIKVSKVLDLKGNMDDDPLKFPNKQDFTMRYGADPQGQTEGVL

DILKGMNRLSEKLMNSLQNEYGRKDENKKRKQDELDNDEGFDDLVEPPKIKYTKIKIQES

NLEDNLENADLNSVNLNELGNRMTAQLENIKNELLNEDRKVSLFDFEKHKAACYEANNKI

LNILDKNQSFINKEDSDNLSSFMNVVYDVNYKKLGIPQEVYDSCSLLNETCNEFLKHFYN

HLNTLDPRRSMIIRRLNKHLVQCDVQLNEINKSFKDYDSDVEYMLKPVHQAVKMSLKKFD

DCLEK

>34-9_0320

MSPLSSGVLKLYVEKWHLFIKRRHPLKRRKVKNSVICNHLNVYVSQDVLKHFVKTLTGKT

ITLEVESAETIDAVKAKIQDKEGIPPDQQRLIFAGKQLEDGRTLSDYNIQKESTLHLVLR

LRGGVIEPSLKILASKFNCDKSICRKCYARLPPRATNCRKRKCGHTNQLRPKKKLK

>34-9_0321

MQFGRKILKNLERSLKPPYQINIRYQLIVFVLIIAVFSLVTLSLITGLYFTKNSKSLRAD

KLYVAAQLKSSQIDQTLNYYYYQCSYLTTKAEIESSLIGYKAGNNSAANWYESTETLDKF

LSSSNTFTNCVLYDSNFVEVLSVTNNGSGNYIPKSILTSLLPLSTDIPLPSSVVNIGMLT

NPIVNTTTNDYSDYISEHPNTLLSLTLPVITSESILLRNSETIGYLTIVVVANQLLNIVN

NSDVSIGADSTTSYTTETSALDSFTMQVLGATYENSSVDGYNYIFPPKGTLPKFTEVKFS

IDNGSFIDQSFSNTKTTGYDTDTKLLNQRVSLGYSKCTFNLVNWVAVITQPESVFLGPNN

RLVRIIIITVFSVTGFVIILTYCLAGVAVKPIVRLQRGTELIMKGRVNQPPYSAHSNAKN

EHTTSKSSMRNSHYFEDTFKNRYTNTSTPISLEHINRNSLQRSIVDTYPGFLDNPDNASK

KHNYFSYHKATKLFPSLFKSNHNNSSSVSIQIEEPVATKSNHYRNVSNGIISHSERQDEK

VVVLDNHHDVILEKAAIDDPIKDDNTKHLQLNYEDNLSPILTSYRVPTDTRVMKDELTVL

TETFNNMTHELDLYYSVLEDRVKERTKELEVAKVQAERANEAKTVFIANISHELRTPLNG

ILGMCSVALSSDDLTDELKDNLKLIYKSGELLLHLLTELLTFSKNVLKKTTLERRSFMLL

NDILHPIHSIFSKIAKDNEVQLQLILKPNQAREILWYGDSHRILQIVMNLVSNALKFTPT

HGVVKVVVEVLGATKISKDVKDMLFNKEEAFSPFNDTRDTSLYSTQELSSDEEKMIEVEH

GFKIIEGTQLDQSNMASSENNNPFNSPLDDIQKSKTLNSLSNNTNDEEFDTNTTITTTNS

SLSDLQIDRRYEKQNNTEDDLLLDSNLENKFMIKITVDDSGSGISPALQDKIFEPFVQGD

QTLSRQFGGTGLGLSIVKQLAKMMNGDVFLKSEIGKGSEFSVYLEVDLLKQLSPVEDNND

YNDEYNENSKKFKHKLNKWSNKKNTKERPVLTKKEKSYETSQSLRGSQTDRSEKPVLMQS

STGTARSSHIVPTLNNMKLEAIASPEANPAPLEEKTTTSPETPKTVTQNARILIAEDNKV

NQEVIKRMLMLLKYKNLTLAADGLEALEFLLNSKDETTESENEGAFGYDLIFMDVQMPKM

DGLEATKKIRENGFKGPIVALTAFADEDNIQNCLDVGMNGFVSKPVKKKELKKTIEKYLN

Q

>34-9_0322

MSFDYQFNNDNQSIKSFKTYTSYSNQSIIFKNTNKVKTKLNTQNVYFNHMNYKDRDKAED

DDIKTDIKNLLINSDEDSKLNINDIDTNKKPVDKKPNVPIMVENNDVADAKDILVFLTNG

KLLYSTNIDLYNKENMESDISLEISVLSLLLETLNYNILNIQHKKKSILFYYNHITPNVK

IVYRLNARNTTNVLILKQLDEYIMSSILNYQSIQKQLSKFEGLDLRFNLDYKKLDKINQF

VLKS

>34-9_0323

MLPYSLNVKLQFANSKDIDIVLQSSILTDDLLNIETTKNADIVGDINKFYIQYSVSDTIL

DLNFHSLDVKALRLNVNNIIENIKATEAMNNEQDIQSDVNMDETNHSMPAATTGSKQNQL

VIKLQSIYRLIMKQESILQVRCSNLTEQPTATVDIDALWSIYKVNTYLVENYIDFLTTAL

SEGQTIQDLNIGKEIVEMYRIERRLWVYGCITFLDVLKNFMNNNLDPTIWSQFIINVFIN

LSEMLVELPESYKHPWYERLGDLSRMAIALYPRGFIDWKLSSEYWYKKAFDYNVGHGKLY

YHISTVEQNTLQAYVHLGKSVFCKDMFIPSQHYMQLVIDNIYQRAYSEFQLAESNVPLLD

HYYRQYVEYKNGYNLLHFPDGTNMNPEQLERGLMTHFFTNHHYQHYIFISHIVEYLKHVE

VISMPTFNTNKDLQQIVLNYFEKNFGEWKKTTSSLFPKENSNFFNEKYIFLQDSEKVKFY

FSTAALFAQSHILQIVGFGNPINPFAKLFGLSEMLHLKKSKKENKKQLQESNPTANPVEE

DYLNISYEEASKHIPEITNKCSTIVLKKYLNGPLLPALPHLLVYGYFFMAIDKKMGNERE

ETYEYNDEKIEFEKLNDDDLAWWNKHMIDLFPWEEIVDFLNVLILFSLKNCPEKYASHIQ

SIKNELFNEEDKNEAIYVKNIPEIKGLIGTLWFDCLNIKVDCDNYEDTDYGIEFDKEVEN

GESFWNRLSEILKVFFKLALKNSFGLKLCETSHIGLGSHLFQFEFSKNERNFKNNISLII

DYYNDNIVKLAGSKGFIVDFKDFTGFGDRITGCIYTSGSLERLNIQGGDDLDFENRYFEN

FDNLEFDLFQKYIKFKDLNLAGSYFILDATSWLRHSGRIFKIMANNLIQLGICLTTFQEL

RFLRKSKDITVSDAATRAVIILRHLYDQKKILPLRFTGNLATTIEEHLEYETKMTWKTHV

DEFVIDAIFKNDRRLFDMNLEGAETGEPNDQDMDLVAEGNEETNAPKKEIFLVSDDAPMR

EKASRYQITSISSRFMFILCNELGNNLSTN

>34-9_0324

MTQSSFEEPVKQINDSNISIKHFQVTKAYKRLLKYSNKYIKVFKTNNICTESISEKDERI

KLIFDDCLFSKLETVLKLYPPINKEVVQQRFGNTAIRLFHDHLNNNNVIDEILYNLLCQL

YEINQTGSLNNTFLKESVVEIKHYLMNAFGNRDRMDYGTGHELSFLAFLMCLDILKIIDY

NIHSLTFLFDRYYKFVENIILTYNLEPAGSHGVWGLDDNFHFSYILGSAQMMVNPVCKPK

DIIAYAKNDVNQNNFLLWNVNLIKKVKGLKNIKEHSPVLNDILMNIKTWDKINIGLYKMW

IDEVLLKFPVVQHFYFGKYFFSWKLTKKIKNDIIQKRLFNETGSEEETKSSLLKYRMNQI

TVDNVINKAEKDDQSSNDRRMFPMNPPKRKTIVSSEKRINKPTRLLKSKSMITSRTESKL

SLHDIVNKTKHSSYLLEKSKSKKRDSTTDNIKKILQNNQQRNKLLSLRNKNDEIAMDANN

LPSKGFDVIYENKYDLQSIVSRINTDPLMLHSTLNKSLKTTFNKILLLNTSLKFFFDYKP

NDIVSLHNIKIVYNSINIKNSNLGLLPKVLVIKCDEFKEKCLYLVDMKKFNNSKEDKFHK

TLTSKLTESKDWVIQISQTNTFDVLDMEYCIKWRIISKPKRS

>34-9_0325

MSDDPKSLIKQAFEKSKPVSGFFWKLISSPEDQYEEAIDLYIQAAKIYRLGNGKVSKDYI

KSADCYKEAAVVALERLENYNEAAMNYVEAYKLCKMGDEYSNACEYLQEAINIFTKHMGQ

FRRAANYQFELGEIFELQLHQLKPAREAYELAGDWYLQDNAIQLSNKCYLKYADLLSMSY

ENNDDLFKSIKMYKNLIDTNIKRNLNLWIIKDYYLKVGLLQLLLTDSVASERTLVEAKNT

LRDDQDCKEFKLLESLIECYKEQDIDLMSQTLMKYDKFDKLDNWKTTILLKIKESLIGTE

EDDLL

>34-9_0326

MAQHPEFNSNGFQAPKLEDYLGKIKEFEIYTKIKIQIPHNFNERMTWQPFEDEIVLDHVD

KYITDNNGFFKDIKNLSDLKDKIIDTANFFSSKDFSNEWINVSTILWDTHLKRYLELITL

IKNEDIDNIPEHLHYLISRSSTIISLQDLLKKRRSAMIKKRWLNVLDPYLKKGKWSKEED

EFLLEKFRQYGPMWSKIASEVPRRTVDQCSKRFSEALDPEKDLKNYVNWTESEDIFLINQ

MNNIGTKWRTIAMDLSKALQSNRSALNCRNRWRTLINSYIKNRNNSAVHMLLADLTVNGE

FKDIVKLREKLVKQNIEKEKKKEYKEQKKILKTKNKKKKKKDLDNDHDGSVDDDKEDENI

DNEDDFEDDEDTQQETPLDYKNFKMMDNTEPPFNTTPGFLIKDDKHKFNYSAKNFMNAET

NNKLNGGDTMSNVDEPHTAYQWNRITPSIASNNMDSQYEQQYSQANSIAALSPGQYHMSM

SPPQTGISLMQTPKPHDIISSLRTTQNNNIMNSPPSLLSNLVSANVNTANNMNDVFADKN

YDKTESNTNTSNNTINKKVNKTFLQKSQARPSSVPLNNNTTEWKFQLKQKNLTLSSGNIT

NEKLVGILIEQAKLNNLKISIHQHIHNHYVPILDNSEQESNTNQTTPESTIQKFSNNSIS

SSNSSNNFGNKVVKDMNEIGGYRTKHFKQLDSKKLPKLGSSYGVKRKGSVTGSSPRRSGS

MDMSGKSVQLNNKRRKSYQFGTKTTSEESEAYDFFDSLMDTNNKNNESENDDDLYMLGFN

PS

>34-9_0327

MFKYISNALIKKQTSTGLMAKRFNAKIINAKKPITINRELPDPIAMKGRNKPKKILLSAL

YIAGLWIGAELIFNNERCDNASVNNTLLELRRNKKYRELLGNNIRLDGTFVPWVYGTLNQ

VKGDVDIQFFVVGNKDKKAKVFFKAQKTPDELHFNIVHWYFHLVDEPDVKYDLFNGDLNK

RPL

>34-9_0328

MTSICVSKYKKFYWQTIKKLNPRVVSSISFNYRLSMKTLPSDISKDRSIEAVDIVIDPLI

QLVLQANFLKQKTSNPKTFLNLPFLNESINIKKSDYLKRNTYGAQNLKVVEVLKKTCWKN

LQYQEILDTFLDCGKLWEKEVDSFTAFKIDFKDQNQINKYDISDEFDFLEYLNYFKEYYR

EKCLLTVQIQEIKEVMEAFKSNLNDFAHFNEVMLKEKTLLDMLIKSVVKVVSLIDEEILE

EDKLYCLSNLLKLKDCKRVNFYNVENNTIKHGLKLNTLLDLVYLSIRNNKQMWFFLMKCI

NFKKDVISEINSCFIKTNYILDSLATFYSKNYTMVENDKFNKYFGAQIMKIKQQIIPILK

SYYHQMNIYDELVYEYETAGINKLHMNIINNFGTLVKHTANIHIKNISKCQCIEDDSNKT

TLIKIMKKFLQDIKQAHDKNSQEKETKKYELQQIKEELSFENSGMVYEAIATCDALKQLR

NYKLKRKKLRKKLSKTINIDLSYDGLQNLINKTKAL

>34-9_0329

MSNNDGRGRAPKISLDLSNSVNHPNNSIDHNMLFPSVTNNAIYSRRSLSRVTSRGTNNQL

NRSRSRSRRSNTNNTIDTDNSDYDTISNVSNRTSESRSIHESTLKSDLKYSILNKNPYTE

RIKIIHSDVDSDYDSDDSDHSYDSYDDNFSDLILNHEEVPELSKKKNKKTEHALQYSSDE

EKILDANDDMFKNQLKYNFTDLVMPNTDIPFKDVLEQCKEWINKYKAEYNNQLEPELLES

IDHKTFCNNTCEKLTLVLGDEHSEQPSIFHKTVEADELPKTYILQTDGSLETIAYTITTL

LKPKDQLYIVCHFEQSSLVKLTQSLISTAQKVLSVFDHVNTSIDLKDVLVNITILKHHYP

KHFLSGLIHAVRPVLLIVNMTIIKGWLNGFVCGVPLLVFKRESRTSFFRM

>34-9_0330

MSVKSPFKLNVLMFQLNANNSVKLTNVRNKINNILESNKFNFKAANTNKLIVFPELAYHK

YLFDSNKDVEQYLQNNPDENNIIDYFKTLSKKYNSYVISGFIERDSVVKDKFYNSSICVY

KDKIIKIHRKKHLYYTDEKWSTETTQDFSTFKMDLVNDSDSKEINCSMGICMDINPYKFI

APFDKYEYSSYCAKENVDLVVCPTAWTNPCSVLQNETETDKKKRYLELLEKMKPNLTNPD

LVDDREILHVNSLMNEKTYSTTEFLYRPDIQTINYWYSRFKPMHALNKRYLLFCDKTGIE

RNSVLYNGSSTCIELVDGKAMVKGCLTTSDESVLSCELEF

>34-9_0331

MPKYIQKHQINPDLKKVIIIGSGGLAIGQAGEFDYSGSQAIKALKEENIEVVLVNPNIAT

NQTSETLADKIYYLPVTLEYVSYIIQKEQPQGILLTFGGQTALNCGIALEKSGVLAKFNV

KVLGTPIITLETSEDREMFADALKEINIPIAESVACSTVDEAVAAVQQIGFPVIVRSAFA

LGGLGSGFAYNLDQLNGLVTRALSLSPQVLVEKSMRGWKEVEYEVVRDSPGNCITVCNME

NFDPLGVHTGDSIVVAPSQTLSDEEFHMLRSAAIKIINHLGVVGECNVQYALAPDSLDYC

VIEVNARLSRSSALASKATGYPLAYTAAKIALGYTLPQLPNPITKTTVANFEPSLDYTVI

KVPKWDLRKFQHVQTDIGSAMKSVGEVMSIGRSFEEAFQKALREVDPSLLGFQGAAEFED

KKVLDDVLANPTDKRWMAVGQALIHENYTVDQVHELSRIDKWFLHKCQNIAKMQNYLKTF

KSAKDIDSKTLLKAKKIGFSDKQIGLFTNTPELEVRKLRLEYGFHPWVKRIDTLAAEVPA

STNYLYMTYNASYHDVEFNENPYVVLGSGVYRIGSSVEFDWCAVNTVKSLRNRGEKVTMI

NYNPETVSTDFDEVDRLYFEELSLERCLDIIDLEQPQGTIISMGGQLPQNIALPLFNQGV

KIMGTSPKDIDNAENRHIFSTILDSINVDQPLWKELSTLQEASDFAEGNYPVLIRPSYVL

SGAAMAVVDDAEELHEKLEVAADVSPEHPVVMSKFHLNSRELDIDSVACDGKLIAYAVSE

HLEDAGVHSGDASLVLPPQNTTKDEISKLKTIAEKVAKAWSITGPFNMQILILPNGDIKV

IECNIRASRSLPFVSKVLGCNFVDLAVSAFFNDQSKINEFKAKHGDDLMLGKKYDYVAVK

SPQFSFNRLAGADPFLSVEMASTGEVCSLGKNLNDAYWGSIQSTMNFKVPISTDLEKKLG

VLFGGNLPQLEEVYKTFESIGKYRLFAADKETCQRLGESAELLEFPEDTSDKKTLRKLYE

DNNVTCVVNLSHARAKSTEDLDYLMRRNSIDFGLCLFNEPNTSLIFAQCLKEKYDEKLAL

LSQGIDFTTIPEEVKAWDEFVDLK

>34-9_0332

MGLCASKEQPYKKKGAEKPPTNKTKQKVEAPKNPIVDVNDTDNHNTRNPNLHENESNEGS

VYNKSNENSGSKITEAVNSGNNSDHVNFQKNPDVSSSNDGASIPKKYNINNPNVKMSTSK

LMKEDLFQQGPNKSVLSMSGTRDLTKIQSPLANLQDTSNKEDTYKVLLLGVGESGKSTVL

KQFKILRQGGYTDKELHEMRTVIYKNLMDIASDIIKARDKYKIPISESDIKDLASIKDVD

EFRNTLTLSIDNRKDYIKKEGPLEDIEVDDIIALPSIFFAYLAILWKTSATQALLKSKHR

NSFHLLGNAQYFIDSLERISNPNYVPTGDDVLKARLKTTGIQETVVEMKDEHITLNICDV

GGQRVERKKWIHCFSNVSILIFCVSLSDYDSPLEEDPKINGLTESIQLFDSIVNSKWFVN

TQVMLFLNKIDIFLKKLPKSPLSTYFPGYQGDPKDAKASVKYILSLLKEKNTSNLTIYPH

VTQATDSSNIELIFATLAETVLENRIKNLGAI

>34-9_0333

MESIYFLFITILARIIASNKTSEAIASKELFNPAYERLSFFSKISALTNCITYDQLQENK

TFLEGGCPLHLHFCSDISYNHFADQVIIKKIILAKNKFELGTGFIAIDHHHQAIHMAFRG

SSTRWDWLHDFSIAPTEYTPFSFKKYVKQVELGNIPRCDDCKLHSGFNKFLSSLQESFLT

TLENTSNSYPDYNLVISGHSLGAAVAVLCGLEVKIRGYNPTVVTYAQPKIFNSKMSQWVN

EMFDIFTLDASNRCHNTVDLKSGYYRLVHINDYVTDLPPLYSHSGLQININKIMLPHEID

DLSYAGLNYASKNDIQASEKIVGPESLSIDETSLATDLLHLYEHRNYFIQINECDYF

>34-9_0334

MMFPIVYNQPPLYYAQRPYDFYSPYVQRPSYSEQRFYNEPLTYKFNEVDDTCDEMLIGKQ

ASAHLYNEFMEEYLQEIILQRKNSFRFNQHYGHPLFDSFFNGLDPYQSASELLTKDDIKK

ANEAFSRNIFKNYKFSISKNSKELSIMNANNKIVKIFELPNSYDNISIESLTTLETGLAV

IRLNLKKSSVIENPVKKVQKKTSRSFENKNVLALKEAYENQLKEQEKLKVKQQEEEIKQN

KREKELALKAAEEMANKKRELKAQKEEEQKKLKAKQLEQAKKDYEIKKMKLKEQKERMER

QAEKLKQKEKALQELYDKELQKLELEAQLASEPTVYSPIPNEENESDEDVHMNEESQCSD

DSDSLESKINKTTEIPLKKKTSITVEDIEDESDKEYRISLMKFPNTNAVLEGV

>34-9_0335

MISSHSVLSKLINGGIYDNASIWAIPLSIIQLLSVFFLMGAISEDFLALNVNLLCNSSNL

LVSVLMAFCNSSPDLISNFVAWYSSAKLEKGSDSDVSNTADTLAISEVMGACGTILFMAI

GFILLAFNKLYKKILIQRFEYENINGFHNGEIQSENDLTDNLRTVTNASLENRINQFVNE

VSNQQYSNELTILLRPLLNKLSNDMIFFSLGFSLILMCCKIKSISLFVCIALLVLYFMFM

LNSIRHHKKHVNDTTNYESSLADQIEMENTDAFDIDNQEEADMIFKNQMEENNSFFENDN

FNFFVSMFDSEEFKETNLSNEDIILEEQSNVWDTNEDPRDVLKLSNKAKQSSDRLSNIKN

HTNSFTYSDNPSEPNQIVENAIIYDKTTENGIMKKFKDSIVFHYIERKRETFQKNNILKK

IIFLMTEPILALAVAVCPRYNKAIPKKEDSYLVLTIQCYTCMFFTGFVFSTFIKRNYLWF

DFALAFVLGSILMAVQVRMRIKYKKISEFSLNRPLTGAASNDHRLATSERISNTTRYEEF

INESTLLLEFRLECCIFSLVGIINSIFWIVILSNNLIQIIEFYQKQFNISETILGMTLFA

WGNSVPDILSNVAVLKLYANDIPQGLDQIPTLLYKWQVQSLVKYCHISIVTCISSSTINS

MIGIGFNALIAILKKRPFEITWSLKDISKKTQIHLLITSSSVLIYAMIVALILKIFVKNK

QLLEKYIIDGYFNNREVVIEESKDGSTMENGKNNLFNSLEYTLYLPGCV

>34-9_0336

YYNFSVAAVEYSEFYVVANLDQSAGEASLSFDLYSSCSSEFTSVSHSVLEDKSTSTKLWQ

EIELSEIPSNTRGLLDAKLVVPLVDGTVSTTQVVNFVVPLYTSKRDTLVSEYLVYTFTLK

ATTAGDNFSTNVQTSRIISAYEAASEFGITTSNSSTTTTSSSSTQTSLSSVSGSTASTVS

STSSATSTTGTSSEGSSATSTTGTSSEGSSATSSAGNNGNGGSRGTKSENSGGAQTTSSA

KSGITATASGATQKTATVSDEQTTLLTVTNCGTKTCTVLATSKINSGSSVTTGVPPSSTT

GGSPKSNTGVPGSNGETNHASSTEASSNTPTATGNNNGNSPSSTSAGKPNTLATTSKASA

TAAITTQSAYDNGAISNKYGSGSFFRNLVLMTLSVMFFI

>34-9_0337

LQKLNTVNGISYSIKPISTSTVPNIKFLLNNLLFINQTENDILYKPKNTFKKVYFTKENK

HKNFKTLENLLFITNTSNFSLNKENLVLKILHSTDYPTAQKNIICHILSNDNTINSNTHS

IPLQIFDSKSNTCFLKLPMEFKIIEMMFLKDFVAYLNIVNYQKSIADYHKNKDYNIYKIK

IKNKFLLRYPIWLIKEAYIFILKQVCDDTGLEKFFIIFFFKFIAGNNVCYNLPDGNVYRR

HVENSNKSKRHNHNMSRFEYISSLDSQFSKIISKLSVYLLLIEYCRQQKIQIISLFCNKQ

EEIVLIVRNAPQIIQGIKSSIEQVFYIDDYIYGIDNIIGVYQFF

>34-9_0338

MSSNETSLKSNKPLFAKTLQRLNKPSLSLKSDKAPMSLPKSLTHKKPKNLTLKLNNFTND

EKESTKESGNAGSATIDLQNLDIFSNNKANNLKKTSNNSLPGTPTTVLSYISNGADSSNM

DVGKFDGTDENIALEDLAQLGKIGAGNSGTVMKVLHVPSQIVYAKKIIPLPSTLVRDGKG

IGGGIAAISDSQNRQIKLQIKRELEIMDKIGRQDENSNIVTFKNAFYNTIEIEELITPLT

NSDDISNDMDNLKIQEQQQEIEVFNKNASTANEIVIIMEYMNLGSLDKILQVYKEHCRRT

GQKINPKTSWFNRNFVISRIAHDVLNGLDFLYDNFKIIHRDIKPSNILLNSKGLVKICDF

GVSKNTVNSIVDTFVGTSTYMSPERIQGGIYTTKGDVWSLGLSIIELLTGKFPLDNRDVS

YKDEFNNINNKNSLMNMDGDNSLRPDGILDLLQRIVNEPSPHLPTSYSFDENLIDFVSKC

CIKNPDERASIKELLKHKYILRYNFNENAKKQETYVKDFRKYCKELKKLIHDEKLIKRQK

QIGISKKL

>34-9_0339

MAESNIINLFSQNSPKDSKEIKYEDCIPCQVMATAFALSLGAYMQTQHLYPKETKKGKKL

NPVEWEKMYPKWYRTTMKATGLGLIVFGCVRGSEHWLWNKEEKKNE

>34-9_0340

MSEELSDFQKRRLENIKRNNDLLKNLNFKKLPIPEAPKATPKTKTTATKRKKKSTAYDNI

PVRRRTTRSTNNIKIPDDFRGIDELDIDSALQNEPKNEEDTLVLEKMKLEEERRKNFRIS

GDIKLGDYIDSNIMNFDINKISAGDFFKQQKSKKTDDSFDGLELYPHFPPNEIKITDSRI

SSLFFHPLSEYNQKLIIGGDKEGMLGLWKIKETAQDEYETEIPDINKLKLFKSNIGNIGI

LPSSINEVFFSSYDGSFRIMDLNSMNCREAFYMNPSSGYEGISDIKFYDDNTVYFTTLTG

EFFQHDLREDFDKVKPLRMANKKIGSMDIDPKNKFQIATGSLDRTLKIWDIRKVIKSEDE

DDLNAGECIKTYDSRLSISAVSYSAEDHTLVCNGYDNTVRLFDDKELRSSSESLTPTNTI

THNCKTGKWTSILKARFKPNKDGY

>34-9_0341

MSKQVNLTDDFEKAANCLSNAFLNSPSNDYLMKKFMNIPLSENCSKKRIKSILEYFCAFY

HDNGAEIAEVEDFNTVSVYTTPKKHVEQWMTNDEVFNQIFFHDLHDLYDKYIPKGMGYYY

LFIIGRDLLDVTKGKGNVRAIFESYRKRADEDNACIVLEAISEEAKQVYEYFGFKTYSKF

NYGKNEVNGKGELDPNGEGFDAYLMIYHKSPLPGFIE

>34-9_0342

MLQLLNIYKTEQIIDMNDKPSIRDLFQDNKSWEEALNDEDFINYQYPLKLPSVIYSNDIK

HAEDNCIIFVLTEAGSVKKAIKSLLSLIEDTSKMKSYPVYFITGFFLDNNSLLKIFNTFD

PLEIDITIIKITDIIVDEKMCGESIDYLFQGFSDIKCNEYRENRNVRMNNDFENLYPLGL

FKDNKEVKWKQIRNANKNSNFKRVYEEYGEASIDFIKLFSYDLYQFDIFKNYKKFMIIRP

GIVVNADTNIDLFDSYYEDNDYTARFLNFRYSQRYRNASIQEFLKIIFQNLNLTIDSIFT

NDNDSIWKQMFNYALKNTKLYNRAKTRIFNVQNTMKNDEMDLFNGILIDTHDSIFISKFE

YFRSKEYELFFKNMFLAHSIYNDAMIPNDPITLFLSLMKRNDQDDNPFYRKDFEIISDVD

IKYDISGDGIFNEFIHNNAGNVESYININNINFEFTLDDLVSEETLLPSKKREVATTSLN

NTVEQEDSLLFSFKREDLKDIDLDNKYLIDQRFFSIFTFKNQVKIFKKHEFIEMIKSQIF

QKHQKEFTKEQVGKLVDLYIYDNIKKLYRIFNKEE

>34-9_0343

MSSNKPLRALISLYDYHSSNDSELSFDKNTIIYQLKGNNDDWGDGIIYINRIEVKRGWFP

LSYCNEILILNDNYFYKDQIINPSNIVKNIDTNAHNHIRTIKNVIRYKFNNTHTNDIIYT

VEKVFKTLYFKSLNKYVIFGNNVKNNENRYSKYLPNDPNIFILNKDTNIQSFDHLLSSVN

EHLTNAKIAIIGENNFSILSNNIGVLKNEKSFKTYINNNIIDWTNFKFYKGSYISFTKNL

NLVSKNSILIQNCIRMYIKDKNLNIDKVVRKKLKQLFKLLNQRFYVISVNTYLYFNSEKY

NINSSYNKNWYGGINEKYQNNWGNSVDLENFSNLKRQSGQYTIANEMNRQSSQYTINNED

SKQKIPYSVNHNPNSSMNPSNTTHGLHIDLLIQSIFKEFNNYDIIINEIFNYLVDFQNKK

QKFHLNNGSIVSVNDTFIPQLVPHFIKDTFEQDDTGGSNIQNNSLLNLINPNSINLTSVS

DSSTKRFVTKKFAQKFQKDLLNILEKTNDAVLILERPANYERDLSFAFAANDVLKFNKYF

LKQFDDIDFELYRYLSNIEFNPEEDGLAEYKELIQWKKLLINDMRLIFLEYTFTKQKYHD

MVGEGIMNTQCLWLQDPYVFCSMKGDYFYNDTKSSKSPILDSLIKALLEQDVETNDTEFV

DPETQLKTTMKYFLNNFNQLLNLINQIANITDNFLNSALRFINNPKVATLVSIITKLKEK

FESIDNEEFPEDEDLNTQENAKSLFTFRKSNNGDYGEVFVNPLHQNATISSVNNTSMESI

KKLTPDTTEQGLFDENGNDWLLKYEYDDMLIYEDNKEIKAGTKIALIEHLTSHITTDPWF

NEILFQNFKTIFDSTFEFFNYILSRYNLIPQEGLNYKEYEIWVKKKLNVVKISSYLALFV

FLKDYWFPQYLEKDLSKFTYLLDVFKTDNMPDVDIFIELFTAKILKPLKVNAKLKDNEMI

VKKYAPKVVPSHLYFKNIEQKQCPLYLDLLSFSARTFAENITLKEFDVFKNISSLECLDR

ILHSNKNWQGGSPNIKSFINMSNLITNFITYEIVKELDINKRIRILERLIDIIVELFELS

NFSAMTSIISALSSSSIFRLKKTWSGISKDHRENYYKFSKIMDSKKNFTTYRELFNKVKL

VKPALPFFGVYLSDLIFTQMGNPDLIRVESINEPLINFKKRSKLQLIIKEIKQLQKVNYD

NTLKMDNQCMWFIETYCYKEPYHSLSKKGPHYVLVIDQLYAQSLIIEPKLNSNQINKQHL

SVMDSHDKKDAFILSDQKEPHLNHKNSGLSSKTTQKAAVKKLKQFEK

>34-9_0344

MSAPPKTFSVSPPDRGSFPLDHWGECTKQMEKNCKLLAKSYLKCRMDNQLMEKDDWENLG

LPKDE

>34-9_0345

MSEVEKTTTEPTLIGPDGQPISKKGLKKLQKEKEKLEKKLAKQQELEAQKKLKELELENA

VDDVAFKYGKLPLLQSQDSSKVDRFQLKDFLKLTSAEQETFATENKTVEIRARLHNLRQQ

SANLLFLTFRQQENLIQGLLKKNDNVEDLNLKISKNMIKWASSLNSESILIVKGSLKKVD

QPINSCTCKDYELQIQEIKVLSEVLPNKLPILIEDCSRSEKDAIESGLPVTNLDTRLDNR

VIDLRTVTNQSIFKIRAAIIKLFREFLSDKNEFVEIQSPKLLGAPSEGGSNVFEVSYFNK

KAYLAQSPQFYKQQLIAADFEKVFEIGPVFRAENSNTHRHMTEFVGVDMEMTIENNYSEV

LDKLSDMFIFVFTQLKERYGDLIDIVRKQYPMEDFLIPEKMVKITFKEGIELLRTVGNKP

ELGDYDDLSTENEKLLGRIIKEKYNTDFYILDKFPLSLRPFYTMPCPEDPNYSNSYDFFM

RGEEILSGAQRIHDPALLKERMEHHGLRLDDPGLEDYYNAFEYGCPPHAGGGIGLDRVLM

FYLDLKNIRRASLFPRDPKRLRP

>34-9_0346

MYKINNIVTQQEQEAASPEPTNQPKHNINIDLNNINIQQTNNLNYNTNHHSRKLSLINDN

DNMYINHNPSINYFLSIQTLQNDTQFNEFLNSLNITYSDSYYNLTKLIPLITSIQSCNLN

YIRFEQMIYKNNQINVEKLNIEDSKAKKYRKQVYSKYILNFYENLKSLINNSLNNLYLLK

DKDLAFNNNILNLLIIQLVYLKFRLKGFYESQNYKIHHLKDGYGEFDLNEKFYSNQLSID

KKLGGGTYSKVYMVKSVYPNYMHFINNETLNQVNNQLFQYRIKNNHKKIINNDGVLIEID

INGNEYVLLDHDDDEDVDFFNQENYDIDNNYNLNRFSNISFNNFLNNCQINNIDLTSQDI

FIPILLYNNIFNSDHYELGDEFLFYLGQHFYQTFLINNPDISPGYNNLLDFKNFVETSFN

SIFIATSYFIQYDHSTSCETCKQYSHDLKDLLNTIQLELSMNDSYTDIISSPRVQIKLSK

YSCLLVTTILENITSSSIFTNSFIFKNQIIFDSCIYFWISDMNIWKFMFGYFDGNLTNMP

LLPNLNHTKDNNIALYDTAKMSRENGIINAYLLLMCNIDNLNVFPFFNNDDYSNDTDNQI

FIRLIYGRFFSVNDDTVPYESQYLNLLTNPEKIYQPDFPYSDDSSSNTFALKIIRYIDKY

RIAARQELLLLYLIKEKDPQRIKNCLIIDSSLDMKGHIMILAPYYPCSVFDFSSSNALAY

FPLSHTKLITKQLFEAINFLHSLGFIHTDLKPENMVFETQDLLYSYRISNTLRNTLSNRR

LKASDGVRKLLKTPKIVLIDFGTSLKIDSYHPDIICTRHYRAPEVILGYEYDLKSDVWSL

GCILYEFIIGEVLFHMHNNEAHLIMIQMFMNMTLRYNINKSKFKNLKVYSKNYSNQLNKD

TKAIKYLFNAHDNYDFNWDLFINPNIESSNSLIEQNRSNELLIDENRCNYELKAKEIIET

TKRLDITITSKLKYFHEVNTFEWLELINFEKILNDNWKDILVKNQEKILTNEMFIFDYDY

HLSTHLCLPTDISLDELFDNQIDLNFMNFDCNLKFYNNQFFYLKQIMIDHFNTFDNVESL

DKFIINKIMTSKSNHESYKECLSQFDGFKDELFKALNLKNSKCLDYKLFKSWYLFIDLLR

CCFVINEHERLSISELLEHPFFKDNKNNFDEFGNEIKSHISNSGMFGYDNDSSSDEKTDS

PSNENSDSEIEEIYELEDNVMIWDVATMVMYFDKLVMYWYLKEL

>34-9_0347

MTSYQIKTEYARHLELIESIPVDLKFLSNLHFEVKETLLLNFKKTNNIRNNIDEIFNDCE

IIQLLLQQILIDPVNFLINVEDLEKLMMKSLLKMKNKSNELNELLENLELSMNSLNSQLF

NTNDENAANDTNQGLILIMDTVTNEFEAFMKISEIIAQLHQQVKQIKSMRK

>34-9_0348

MGIPKFYRYIAQRWPAIQQEVESHETVPEYDNLYLDMNSILHNCTRTPSVNVILTDEMVC

QKVFAYIDHLFNCVKPQSTIYLAIDGVAPRAKINQQRSRRFRAAQEFEEARDEAIENGTY

IQPPPEDENTFYKSSISPGTVFMAKMSEQLKYFIHDKVSNDNSWKNINVIFSGHEVPGEG

EHKIMEYIRSLKSSPNYNINTRHCIYGLDADLIMLGLVTHEPYVSILREEVFFGKQNDKI

VTLDNQKFYLLHISIVREYLKLEFDTLIKDMQFEYDFERVLDDFVLAMMAIGNDFLPHLP

DLEINKGAFDYILQGFKSIFQDLTGYMSENAVINFEKFGVWVNFLANIEKALFEKQDVDV

TWFNQKMIELSKESENKRNAIGRSVLIKDQKKIIGYLRKWVYKNAQTLLGPELFKNPPIL

NLNKNNIVLSESDPETFELLKTFAHKLNYNLIHSASTNEYSIKMFIEEKYQTETETQEDM

EERVREARQIFRNYDHSVVFEDQETLNEKKLMIEERFDTWKAGYYKKKMHLDYIQKPHDV

VELAENYAEGLQWVMYYYYRGCASWNWYYKYHYAPRISDLASGFQKKFEFELGTPLLPFV

QLMAILPPLFKINVPSAFRHLMVDENSPIIDAYPTDFEIDLNGKRQAWEAVILLDAIDIN

RLKDAMEPHYNKLTEEEKQRNTHGHDIIFIYDSQVDNVYKSPLNGLFQSLPNNHCVEQPY

ILQQLPLDKIKFGLHDGVMVGKQSFSSFPSLHYLPFSFDTEHNETRVFNMGSKDLSVVLH

FDQNDISTVNDETIIHSYLGKNVYTRYPNVRLSKVHAISTAEGRFVLDKHGKVNQISHRN

VNNFNRDASKLESNLKSRFAFIIGKVRFIFEVLPVEGVIRAEDGSYKLSFTTPNEPEIYP

FQLIVDEVGNDDIRFVEKDPVAIEDEFPLGSEHIFLGDFAYGAQCSISGYSDDKKTLKLK

VDTSSVIDNSIPFVKAVADTDNKALKYYPQYQITKKLNLSPIFFSRILGRLYVVFKNSRN

NLGFNLVDMQDNFRALGYAEKEVNWSFSDMVVTLFKEFKARYPLLFNGINDLMKSVSKNA

RVPNIVIEDLYNNNAKEFEAEISSFKKWRDEIKDNSVYVSVFSKSLSRNGMAEVEKIAIM

NDKAQSKVPTYKKLASVPTSAIMDPEESYGLLRTQRFDLGDRVSYIQHHGKIPYGSRGTV

IGYLHEGTVTNIQVLFDQEVVSGNTLGDRIQTTRAILLSKSVLLNISSRSLIFKDPTADV

ADKQAEQINKVSAEEKKKKAKELLKVIKANDAKKPPAQLIDKTGGLKKKLEDQKKAEAAK

KSSQTTSKKPKSDHKPKDKNAPKSENKPKAKNAPKSENKPKAKKAPKSENKPKAKNAPKP

DAKTENTEQKPSVSVA

>34-9_0349

MSDNQSKTISFKEYLATKSKNSPKTPIQLTKNETKDLRRSKPYPAKRQPRQSTQKVEKIQ

QKRPVIKTEKVVKKERGAISSFRAYTPLDKNISIPRNRFNIKPGFFWDGVDRSTGFEKKF

LKVDQELKLDKHKQKNDDGINMEI

>34-9_0350

MDTFFKELDVMSNGELWQVIEYAQNRLKTNCTKKNRHLLSTQPEINTQDEFLETKNEISD

INITDSGSFDDYNDKNFVVVETKFLKVEDVQRQVNKNEFTNTNDLKRKLEEIDNLINGPN

KIKKESKSELTTGMNLKTEFFNEPQNIKIAPFQQFDPNEKVDEKDVDYYPNVVLVDELIQ

KEMINLPTDLSKEDQRRKNLATFLQLHSHFPACKKLSLNYNPIRLMPWRIQDFKINENSE

YGREKEIKKTKKYWENKKKWGKYNSKGNNDYNDKYFENFYLFFDNMKNVENPLRTVDRIS

YPSTLEINQDKHYLSIYEYNECKKMLLLALDQSIPFQERGYLFREDLLNQLVDSGKMDID

FDTLVISHMKDIITYEQA

>34-9_0351

MSETSSLKRKKQQQQQHIVGVHYSLGNKIGEGSFGIIFEGMNLLYNNTNMTKYVAIKFEP

KKSDTPQLKDEYRSYKILKNQAHVPKVYYYGQEGSNNILIIDLLGPSLEDLFEWCGRQFS

IKTCTLLAIQMIDTLKTIHSNNLIYRDIKPDNFLIAEYQLTSYISNSSSSSIKGYNSSSH

NNAGKIVCNNINNPDKDPNYVYIVDFGMAKQFRDPISKQHIPYREKKSLSGTARYMSINT

HLGKEQSRRDDLESLAHVFFYFLRGHLPWQGLKAANNKLKYEKIGKTKQASTADFLCMNG

KIPWQFGEILTYARNLNFEEEPDYEHLKKVLYSVFDELNIENDGRYDWMDLNGGRGWDIR

LNKRNNLNGYGGGNTNNNNIANNGGKFRQQQNTARQKPMDNLKQIQKDSQKLENQQQQAL

RQKKSLQNVNETMSERRRSEELKAYYFNSSNDNSVSMNPNSDGYQSFENGNKMRYSRLSA

DQQYGSIPNNNRYKGQLLPNKKQNGLMYEEEQDLEAGLLQDYEEEGYEDGTLGRKLKKVF

CCCSIM

>34-9_0352

MTVSFDPDTLLKKLTLDEKIQLLTAKDYWHTVPIPRLNIPSIKVTDGPCGSRGSKSFNST

KTAAFPCGTALGSLFDKKRLFEIGQVMATEAEHKSAKVILGPTTNIQRGPLGGRGFESIS

EDPLLSGLSAAEIVKGIQHSGRVMATMKHYVCNDMEIDRFGYDVLVTERALREIYLEPFR

LAVKHADPMFFMTGYNKVQGVHCSTSDKNIKQILHGEWESRALVMSDWYGSSHPMESIKA

GLDFDFPGPEKFRSVQTIKHLLFSKAETRDGTIFNENDIDVRVLKILNMIKYFVDVYGTT

DFTFDEDTKNNTKETSQFLRQIAAEAIVLLKNEDSILPLKKSDAEDLCIIGPNAKAKFHT

GGGSASLNAYYIVSPYEGILNVTEKESLPYSKGCDAHKTLSNFIENCETEDGEKGLSVKF

YQKENHRETGEKPFKVEVVDRSFNKMSDMRDPHIDLEKKLFYCDWEGLYTCQDGDGIYEF

GCQVAGTALFYLDDKLIIDNKHDQEGGDFAYKSGTIEKKAFRIEFGSISTSKLNVVSEIT

FGALQVGINKVIDSKLEIEYAVFLAKKHKNCIVVIGLNGEYETEGDDRPHMNLPGLTDTL

VTEILKANPNAIVVNQSGTPVTVPWIDQCKVFLQSYYGGNELGNAIADVIFGNVNPGAKL

SLSWPKKNEDNPAFLNFTSHMGRVLYGEDIYVGYKFYEHMKREVEFPFGYGLSYTDFEIS

DLKTSLKDNDLLVEVTVKNTGKVEGSEVVQVYTSRVSESQAPRVPKELKEFEKVTLAPGK

QTVVKLNLSVKDSFSYFNEIRDKWHLEAGKYQILVGNSSVNLPLSNELEIEDDLYWSGL

>34-9_0353

MSTNQSSSNLSFDGVEKNKQNNESDDVNKHADYHLGHNSDDPSIGIHTVLPTYTQKWYTM

KHFQRLMWVIFIISLTSCNTGYDGSLLNSLYTEKDFNNAIGNVSGSILGALTNGYVFGCL

ISFAFSAKMNDILGRKKCLIYCNVIMIIGVLLQSVAGAWKHDGYPEHYTKRDVLGMMIAG

RIVIGIGSGVIQLSAPSLIAEVAYPEPTARSIQINYYNSNWYLGAIVAAWISFGVKNVAH

HWSWRIPTIIQCLFAVAQLILVPWFVPESPRWYVSQGRFEEARDVLNDLHAGYLENGQEL

IDYEMTEIQLAIEQERAAAQTSYRDLFKTKANLKRMWIICWVAIFMQLSGNGLVSYYLGK

VLNSIGYTSTSEQLIINAGLMIYNLGVCIIQSFWVVPLIKKRVILMKASVGGMLISFIIW

TILSAKAQQNDFKDKSMGKAVLAFIFIYYFFYNLGLNGLPFTYCTEILPYTVRAKGLSVF

VTVQFIVQIYNGFVNPIAMDAIHWKYYIVYVCILAVEFCVCFTFIETSGRTLEEVADVFG

DGIEDLGAVSGIAALTDGKKNRNNVEDTEFIE

>34-9_0354

MLSSSKNSIKKHLLQHAIKPSLIITKGSVLHTTKKSYSTPPSNGTFGTRSTVIQLLNNIG

SKREVEQYLRYFTSVQQQQFAIIKVGGAIISDTLPELASCLAFLYHVGLYPIVLHGTGPQ

VNARLEDKKIEPEYIDGIRITDPQTMSVVRQCFLEQNLKLVTALENLGVRARPIMNGVFQ

ADYLNKEKYNLVGDIKKVFKSPIEASIKAGCLPILTSLAETESGQMLNVNADVAAGELAR

VFEPLKIVYLNEKGGIVNGNTKEIVSVINLDEEYEHLLKQEWVKYGTKLKIKEIKELLDY

LPRSSSVAIINVHDLQKELFTDSGAGTLVRRGYKLVERNSLKDFDSVSLDSLRSCLDKDI

KSKNSINDEEISVAKYLSELETRTFKILSNEPNDVVAIVLDDKQEIVTKLDRFMCTQQGW

LNKVTDNVFTQLKKKYSSLQWVVDEKDPNIAFHYAKSEGSYLKDGKVLFWYGVQDLDTVT

ALITEFCKTLDKSPFSYESKKSTVFGNASGAKRNYSTIVRTPSIEDGKFCQDVKKVALIG

ARGYTGKNLIELVNNHPFLELAHVSSRELCGQKLQNYTKSEIIYENLSIADIKQLETDKK

VDIWVMALPNKVCIPFVEAIESIDNCKTSKIIDLSADFRFVDESSWKYGLPELTSRSLIS

QAKKISNPGCYATGTQLSIAPLNNNINGLPVVFGVSGYSGAGTKPSPKNDVKNLENNLIP

YALTDHIHEREVSKRLGRDIAFIPHVGQWFQGISLTVSIPLKSGENYTNESICDVYKTFY

KDEKLVKVLNVGQECTVKNIAGYHGVVIGGFKVNEAGNRVVVTAFIDNLLKGAATQCLQN

INLALGYYEYEGIPKNKIIPTI

>34-9_0355

MSEKQYGIGFDLMAKMGYKTGQGLGKNNQGIKKCIDVIKDSKPTTTGIGKETAILRQQNA

MNTEIIVISDDDEDNTPEVKDFEEKLLGMGVKRDIVETLKKQGLNKLKQFYESRLRKRNQ

T

>34-9_0356

MSTIYVKIVSSENLYRRDMFRSPDPFAVLTIDGAQTKSTSAAKKTLNPYWNELFKFANVN

ENSTLVIQIFDQKKFKKADQGFLGVVNIRIGDVLGHLDDNIDAEAGKEFTITRDLKKSND

GNAVSGRLILILSKGQAASNPAAVADPQSTRPPAAANTPSHARNPSNISSTSNSLPSAPN

NSTTDRVSSTSGSRRRSTASASTRNIQQDANVDSNKQYSSFEDQYGRLPPGWERRTDNFG

RTYYVDHNTRTTSWKRPAMDNSAALPQQPSTNLATESSTSIDERRRQFDHRTLPGPESNS

ATNLQQAAPAPAAAQTATSTSTPSGLGELPPGWEQRYTPEGRSYFVDHNTRTTTWVDPRR

QQYIRTYGPTSSVVQQQPVSQLGPLPSGWEMRLTNTARIYFVDHNTKTTTWDDPRLPSSL

DQNVPQYKRDFRRKVVYFRSQPALRILPGQCSVKVKRSNIFEDAYQEIMRQSPEDLKKRL

MIEFDGEEGVDYGGVSREFFFLLSHEMFNPVYCLFEYSSHDNYTIQINPNSGVNPEHLNY

FKFIGRVVGLGVYHRRFLDAFFIGAFYKLLLRKKVGLQDMESVDNEVYNSLKWMMENEIN

GILDLTFAVDDEVFGEIVQVELKPGGKDIEVTDDNKKEYVELFTQWKIHDRVSAQLKAFM

DGFNELIPEDLITVFDERELELLIGGISEIDVEDWKKHTDYRGYQEGDQTVQWFWKIVSE

WDNESKARLLQFTTGTSRIPVNGFKDLQGSDGPRRFTIEKAGEADQLPKSHTCFNRVDLP

NYDDYESMKRKLTWAVEETIGFGQE

>34-9_0357

MSEEKLETKPKDVLEDKPKQSDKTKRSLSEETSEFEEPEDKPNIKKQKIEEESAEKPAEK

PKFVFGGGSTFSQGFKFNKDMIPANAKKAKEAEANEDKDKNTKETEPKKETPVFGTFGSS

TTFGQGFGLLKEIQKKKETTNEEKNDEEEDKKEEETKKAEQKPVLTKLETVENGEENEDI

VFNSNTKMYSLTSLKEGWKERGVGPIHLNHNKETDSYRLVIRQRLTFQVLLNLKIVKGIK

IFKGFPGSTNSEKFVRMVVTLDKQVVQYCMKLNSEDLANKLFDKLESLVKKK

>34-9_0358

MDDTSKLNTSKLGTQKYWDDFYDREIDNFSQDKNDLGERWFDDSDAEFKMIELLMAEASD

PDTQIKFSNSNTSTSNFIDLGTGNGHFLFELMLDEDFDENFVDNNRLLGCDYSAKSIELA

KNIQKEKFLDNESIQKQLNFEVIDLFDEKSQFFNTVKNDKNEKFEVVTDKGTFDAIALSG

MTKFSESLEKEISLVDYYPHIIDNLLAQNGLFLITSCNFTQEELIRIIESTGNLKYYKNV

NYPFIEFGGIKGSTICTVAFTTK

>34-9_0359

MYNPELFYELNTPVAETEFQVIKKAYELNSSDKRPAFNYAWVLIRSNNKDAQVQGVNLLT

QIYKTIEAEKKKEALFFLTLGNFKLKKYELSLKYIDSLINAEDDLHNDTSSLRIVRDKIK

EEISRTAGTAFVGLTIGVGLLAAGLTVANKWYRTGGRK

>34-9_0360

MFVVKRDGRKVPVQFDKITSRIARLCYGLDAKHIDPVKITQRIISGVYEGVTTIELDNLA

AETCAYMTTVHPDYAVLAARIAISNLHKQTKKLFSQVIEDLYNYVNERNGVLSPMISDDV

YKTVMKHKDTLDATIIFDRDFNYNYFGFKTLERSYLLKINGKIVERPQHLIMRVAVGIHG

ENIEKVIETYNLMSQRYFTHASPTLFNAGTPRPQMSSCFLISMKDDSIDGIYDTLKECAQ

ISKTAGGIGLHIHNIRSTGSYIAGTNGTSNGLIPMVRVFNNTARYVDQGGNKRPGAFALY

LEPWHSDIFDFIDIRKNHGKEEIRARDLFPALWIPDLFMKRVKANGDWTLFSPSEAPGLS

DAYGDDFEKLYDQYELEGRGRKTIKAQKLWYAILEAQTETGTPFIVYKDACNKKSNQKNL

GTIKSSNLCCEIVEYSDDKETAVCNLASVALPAFVKVSEDQKTQTYDFKKLHEVCKVIAK

NLNAVIDRNYYPVETARRSNMRHRPIAVGVQGLADTFMMLRVPFDSEKAKELNIQIFETI

YHAALEASTELAEIEGTYASYEGSPASQGVLQYDFWNVAPTTLWDWTELKAKIAKHGLRN

SLLVAPMPTASTSQILGYNECFEPFTSNMYSRRTLSGEYQIVNPYLLKDLVDMGIWDEAM

KSNIIANNGSIQQLPNIPQEMKDLYKTVWELSQKTIIDMAADRQAFIDQSHSMNIHIMAP

TMGKLTSMHFYGWSKGLKTGMYYLRTQAASAAIQFTIDAKVAEQAASSVSLLETLDRPSY

IPHEFKQIENWEPTKEGKIVRWRASEDREADELKAAEESKKRFVRRIGNSTPVVEKPVEK

ETSPNDTLIEPSTSNENVKDDEEVDIFNSKVIACAIDNPESCTMCSG

>34-9_0361

MSLKHKYIKLDDNNLELETFSLPSPREPSDQVSEIPEDFHQRFESYFQPYVNSQEELSQQ

PQIEGKIINNENEISQDSEFRYKNEYYTDKQDYRNFNIFQKLWHGPENVVDEPPRYQDNT

IFFKIDQFPRNYFQYRFSKTLRYFILFLVTFLQILLITVFFVLPYSKISDESIIPLECNS

RVGYPSGVLNNRCGVNLEKCGFEYDAEEHVLINPIFKNNEDYKDGVTLRCPALCDQGGLI

YAPLEVGDQRIRYRNYIIGDHVYRGDSYPCAAAYHHFVSSHKKKNQYRRRGILNYAKNVI

KTSINSVVKHSLISKFTGGCITIRSAGAQLDYPAFESSENINFNSFYPDSFVLENADFNS

SFKNGCLDPRFYVLGINLIIFGVVFYLYESSVAFWWIVVEGYWFQSMIMDPALLVDPRDW

STFNELISLTMQKFLPLCFIVYVLYDASIKRTLNDEYESGIWKLIMLTTFWVGLLNNVTF

DRLPVDRLTVSDLKEQKGGFLATIFILSIILMSATTQTIKVWKANLFQKYFKFYIMLISG

LILLGFLPGFGLRIHHYILALLLIPGCSTRGKSAYVLQGVLLGLLLNGIARWDFASIIET

QRALLRGSAGDLVKPPIFDIPAAPKIKNLNRFDEITWKLDSSDSSFSTFNAVSLLVNDME

VYVGTNTTISIHTLMDENALLSDLINGALLYENNIKMYLRLARCDLLDSSKRGDYTNAGT

LIYPYGNWTTPAPGVT

>34-9_0362

MATINREDLGQDLMAHAIHEINENFEKLDIHSDNGILGLDFLNYKTEVSNENNSLNFDLQ

KEYDSILGFLNQKVENIEVNKAKHTAYIKKIEDLQKIMQKSINGSAIAKGTNFDQLIKFN

DDLTNINQSIDYYHKFVDFENICNEVELLISELLEEVDFLEMPDTEFIDRNYSQKILYVH

HQINELKKFEQFAKKLSVYENTDKEIRNIISKFFMSVEKYSIIKEKFNKFIKNLITDLID

FLRSERFMIILIFFKILEKDTDADMEIMDTMNFLKTKQESLIIVKKIDSKKYQTENEDTD

VVFDNLICQEIIKNRINTRLELNRMMDFFIETLNAEVTDMFSNLESTYHVNTAEIDYEVL

DNLEWIMNELLVVFVHLHKFCWVKFPIREIYFKAYYEHLNKLIMNILAKEPDFNNLVKIL

QLDQIFLKFFNKDLQIKALKWESPSIIGEKEKNLLIDDYLNLLNKKMDEWVYNIQKEETA

LFVMRDKEPSFDENKKLWILDLKNLFQMFIQQMDLCCDIGYIQIFLGLDRLEYLIAVTND

QMKGADLTVMIQDKYTQLVSNKHKDTIDSEIDLVLDGYADVAKVCTNAIIDLITDDVKDN

LALVLSSKWYVNDNDTLILQTINIVQDYLQDIQQFMNEYVFVTFLENLLEEIVNKYIESL

NYKPMIVKKKIIIGFKKDLEMLFKLFQNYKPKKDDQTDEEEEDYTYDEMLESKFKIMEYF

IDLICCPLSLEDNVEDFKSIWKNLITNYYDCPMNLIYRIVKLRIKEEKEVSTKSSTNNSI

KMNQLFEACKQIKEVNLRHVKETSLDMNTVPTFVAKLNIN

>34-9_0363

MSTTTPLLQKGPNKKIALPTRVEPKVFFANERTFLSWLNFTIMLGGLSVGLLNFGDKVSK

ISSIFFTLVSVGCMIYALVIYHWRANAIRKRGSGPYDDRMGPTVLCFFLLLAIVVNFILR

FKHDQ

>34-9_0364

MLKLNNTAYASALKNPVQYSKFGISQAIQRRLNHTLNFDMKLPNGHNYDQPTGLFINNEF

VKPLSKSKPSIPVYNPYNESLICDIYEGGADDVEQAIDAAEKAFKTWKLTTPEERSKLLY

KLADLIEQNIDLLASIETLDNGKALSSAKGDVGLMANYFRYGASMCDKLHGKIFQNNAKD

GFGYTVREPVGVCGLISSFNFPLMFVSWKLVPALVTGNTCVFKPSEHSCLSTLYFANLIK

EAGFPPGVVNFITGLGPVGATLTSSPKIKKISFTGSVGTAKKIMSSVGIKKVTLELGGKS

PSIVTNKAKEDLDKVIDNICLGIFFNSGEVCCAGSRVYVQEDIYDELVEKLVKKAESLKV

GDGFSDPYFGPLTNKMQLDKVKNYVDIATKEGAKLLTGGKQIGDKGYLFQPTIFGDCTND

MRVVKEEIFGPVLSLIKYKDVKDAVAMANDTEYGLAAGVFTSDLNEAIQISNELNAGTVW

VNTYNNFHPSLPFGGHSSSGYGSEMSEEAFHCYTNVKAVKMNIKSSDAITIRTRKVITNP

LLARKQFVIDVIHPNRANVSKDELREKLAQSYKADKDAVSVFGFRTQYGGGKSTGFGLVY

NSVADAKKFEPTYRLVRYGLAEKVEKPSRQQRKQKKNRGLKVFGTGKSLAKKVARRNAD

>34-9_0365

MQNNSGIPMKSYNHSYVRKSQTNFDSNNYMNSSNHSMANPLIQNLELNRNSNYHSSSHAI

NINHNSHSNNDNFKKGNENNIFKEQTEMRFGNQLYTNDSWGSNSSLESLNLILEKQRKQQ

LNNPLHQTHIFLPVKIKLEYDPITKRTMLNHYEIIKEMGQGQFGKVKLVKNMHTNELNAM

KIVNKHSTSMAKKFSLEPNHNTLNADFETDNINSNQIDEFGNAVNPSLIDHGVSDDKIRR

EVTIMKLLKNKNVIKLIEVLNDPESKKIYLILEYCAHGEIRWCSGDQLEINAKGPSLLSF

EKSKDYFRQLVLGVEYLQMKNVLHRDLKPANLLLTANDVVKISDFGCSLLLNTCTEEDLI

KTVGTPVFYAPEICISNAVSFYKTKSIRQVISFSLDIWALGITLYCFLFGKLPFNSSHEL

KLFEIICHGKLEFPMNLPNLEVREILQAQNILLKLLDKNPLTRITLEEIKNHPFTLDGLS

DSQRVAFLNDKIQTSDYSDVEYDDFMNDDNYYTHKDYQNNEYELDINGSHRKHHLKQSNA

DTKFVSLPLNSSFASLDSFYIDSNARYLARQDSKNHVGSDLRDNNGNLAMSIDINSSTIP

KTLRQFTAGKRLSLESTGMIQRAFDSRFNKRYDKKSLDLNKANPEYSLPKSHYLPQNSSI

NSSNLNDKSDTKHWKYYNEQSDTDSDDNAFDDHFEFTGYYKNDNYNNDLDNQNFSDAESL

PFEFKEDSDNEDAAYDQMKSNIAHLENSDTADNLGLKLKDPNSVVYMVDALNTNGTPNIN

EHLNKVNESHFGHGKKTGNLILMDSSEDEDANEDESFFYSNDNKREYPLVNSSHNSTSKS

PLSDMTQEKSSDPNLVQNSNQSSSLSSVVDLQGIQQVDIPEDLLIQMNPSGVDIKKNYSE

QARMVNHSSIDNDTTRKIHSVTDLKKIRSNNGLLNFRVPMKNMLMKTPAASNNQLFAPRF

NPLQPASNTDITNSFTTEFTKTTANSVLHTNSTSNVQTYSNKENHQHRTTEHGSNMKSVD

ILQSFIDDRKIG

>34-9_0366

MDKLDNQIYEKKTVKEGSKNNFCLVCMESTNICLYKKNFYNFEFNKKTYADWFYVCEWHL

KHADFLEACESQEIKDLRQELKNTNEKIKTIKKKIDEEKGQIKTPEWLNNAMSSTGSYIT

GKLPFSGSKTEEDGNKQNDTEMIIEKKTNKQEIEDLLDNITGKEQQIKKLEESTKFYKLS

DFTYMNRKDSLASSLKAKFIKKQPVIQNKKVAVTNTLSFPSAPTHEL

>34-9_0367

MSDTINKNITINDQSLTISNKIGKGSYATVYKAFMSSNEEPVAIKIIKNSNIISNNLKAN

NNILNNIEVEINILKRLKNPNIVKLISYSIPKTLQSNDEEVSNNKNIIIVMEFCDIGDLS

NIIRFSNTNSMDHKKQISSNFNYHMLEKITNRFPMNTYNGLNCVLTYSFIKQIANALHFI

QSKNLIHRDIKPQNLLLTSQADNLVPVLKIADFGFARFLPQQSMAETLCGSPQWMAPEIL

SYKKYDAKIDLWSVGCVIYEMCFGKPAFQAKNYIDLLNKIKTVGLKFPAAGSSKSDTSLS

SNSSSSSDDDFDDSDEIDEYDILDSNMEELIKRLLTYNPVKRMSFDEFFNHHLVKSFDSI

EDTFNVEKLLALKESNSARAELKDHNFKPITYNEVFDSSSDEEYEDSANKNITTSQQYSE

ADGYLMIPNEAIITTNEFPINGPRRKSKVLVKRTSSNKNDHSGIAAKHALKSNIESPAQL

LRRESSISNTSADIYEEKKRRSSSFAGMTKAITKAFRSASIISNASSQSSSRVSPDDHQQ

EKLSTKNIRTDLVLRYDQYLDLTAELPNRLKAKTNKTENIFSTQTLSTQNSDISKVRQLS

KTHVFMKYLKQEESGLSGLSNDDFELLIQLKIWFLGYLKKQMDSYKSKDYETLKYFKDKF

DKVLDSVEVQYEEFVNMQSDNGKNDLPDKDTWSNRQIQDSLWKLSKKFARLGYKLETKKV

FDKAALAYATSLWFMDFIITDFDFLDTSDNKFERMCVLTGDRLSSVYKY

>34-9_0368

MSESNNNKRPLDLDDLNDDDLYNDNNNNKKVKIEEVETKQESESEYTDASESDDDIIFNF

GNNSKTLSSSTANKSSDSTSAKSVDIENKTSSINNSLETSLDSTGEKDKAALTTDGDDYE

EGEDEEIQPNFLIEDESIPFFITSKDNKPIDIKKDDDFEGVDLKEIEPSFMKEKPWRSSR

AKIENYFNYGFNEATWMEYLNKHTKYNVKYSGSKLLYQLLLLQNQNRLDSYNNINKNNYQ

SVNSDNVKSNPQQQMNIPEVAKLKNLLKYQQKQQLREPPLPIIQPIINAQGNIINPNAMN

NSSNSSNMPPGMDMNMPSGNMFPFPPFNMMNMGNNNINNKHKPRK

>34-9_0369

MDTSLDMDSMNLLQEMEDISNALKKQQLEQLSNSETITDINQLINNENNISSDDDISNMP

VSNFKNNSIQKERLTSISNAIQNNKISRYQSTDNESEIFVSAKEDFVTASEQSETDLDRS

DEDMFNKQRKKSSSTELKKEHMKMRINSFNSDIFNKRLFSMNSDILSHVTISDQKGNNSR

QVSQEVSNTDKEEHTLMLDEQTQDVTKMLQRIDTGLENNHIVMDSDNSKDSTSYNNTLDS

DGDFNSSFSLKIANKSNDAFNASDIESVASVKYSKFENDIKNNSDLRMSSNDEKRSISSS

SNKFKTNRIISPSQIVQLSPNPPSFSSFENSFAVFDYSIHKDTRRSEYKDVMNRIVSSEI

EYPKTSVNNSQLDQSRNVLRNLRRRVTSDILKAEVSDSENTIPQSNNNFTSNRIASSHSL

LSEMNKLWDSAISDTAKEEPTNDDDKLKEDSNNENKISHIWKNENSSLTNLNVDAYKQSE

MVKRTLKNSDDHVIQRTSTGLGIYHNLELIKESSDKQKDAFMGSASSDFAKEETGKKVII

GNVQDLLLQNGISLHDIKFDQESIKPQKDEIQFEKHDVLPQTPKESSNDFKKMKVSSPVK

VVNREIVEKKPLVAIEQESEIDKNNEFDGKKRVLKLKNNELSNLGYLYISLNTLSLSNFI

NKSYPNMTFADKKAELQILFDNGKKIIESEWIPLTNINSGSLGENIDISSQEYMTEIPIK

DTLDGYDDIVSSLQFTFKMRYDQPENKVVEIQDRIPIIQNNKSNTDEPRTNSFTKKIFNR

KGKTGSSNSNNSSIFTKAGRRVEYQTVIKKVEQKVQDPWSTNFSPDGETFGLFTAQLSFS

DICLMTNVLNSKKGDVLATSSFNNANLLKNIGSLDFKILHLSHVNNVVPKSISAISEIIN

RKEKILSIKFDRHGIMYQQGLDVPESSGVRRRKFVLENGLLNVYNVDDSEELKISINLEN

VNDILLDCESCPAPGFNFEMVFDDKSRVVLSCDTINQRKMWLNKIKEVLDVLRVVRELDI

EIK

>34-9_0370

MPIINRRILRGLTYTVLSLILIIAIFLFSNVHEDYNSQAVYNKLFDGQTHEPQHVNYGVR

RLANFYDPLADKTLNQVHINSNLYEPIKFDEWFIDTSKPEYTKMNATMVSLVRNEELSNM

LTSIEEFEASFNHKYHYDWVFLNDKEFTPRFKKQVTEKCSGNVKFGLVPEEHWSYPDFID

QDKAAQTRIDMANVIYGASESYRFMCRYQSGFFWRHPIMLEYDWYWRVEPSTRYPCNIPE

DLFKYMNDNEKIYGFTQSLHEYEATIPSLYDSVLTFTNNHPQYVPHKNFASFISEDDGET

YNGCHFWSNFEIANMKLWRSKQYNEFFDYLDKQGGFFYERHGDAPVHSVFASIFLPMDQI

EYLNIGYYHVPFWNVPYAVGKNSDSDEYLLEKTKEKYLDLKCKVDPREDFTINGFSCGEL

YFRQKKLRTLHTVPDDEALDWMKITGATPKEE

>34-9_0371

MTQQVIDTSTLQTRFNKLLITDNDKIILTPFNCIFIYNKNKENWVSYTSIKEIILYEKSI

KIIFKDLSTTDIMGDNNKGLLSDIFTSLCLKYYQNKPLQYSAEIIFTYLSASNTSNILIN

CLPENIITKELDRQKFKKHNSNWIVSDINSHYQICDSYPQKMILPNNSSLKNSIIHAKNF

RDQHRFPIISYLNASSNLILRSSQPMTGIMNKSNSQDEFLIKEYLRNADISDKNKFIIVD

CRPLKNVYAQKYILGGGTENMKNYQFTDLKSKEYANSKAYFLGFPNVHILSDQNEAMLSC

HIIGNSKQKDENMKPLDFDWYLNIQHVLKELDYLLKEYLLNSSHLLIHCTHGWDRTSLMT

SLLMICTDPYYRTIEGFFVLIQLEWLNYGFRFAERFDVNEYFIDDMEDMMTEEDIESQEN

KTNNGFFMNKIDSFMDKTKNNNISDIHTTLKKKLGTLDITAISNKFTTNDDIGLDPIIKK

KLSEHSCFKTKKKMIDHSDFEQSFNCDEENEIKKIFKNPDSKGGVSPVFIQFLDIIYQLI

KQHESKFQYNGKFLLRLLKEITSGKYYEFLLNNDKERTQYLHSLKLDKEKKVKVSWYNMI

DTKESEKNSDYETPTDQSDHWIFPNESKVTFWKALWRS

>34-9_0372

MNNACYSSSSYVKIADIHRYKIKYKLYDDSLEDYLNGGSSYVKGIPDKITVTITNTVPLS

MRATGLLLGPYSLYVDLKKKKYDHKEKYFEKSDLPIFEPNLLPSQDIIFDLYLNDLHENE

IEWELDIISQALFTRNCKIFYKVDIKYHFNDKIYVPKTRTELELLKQNLSVTKLDSQDIW

EYFNTSRKLKAKEDNGQKIHLVVLTHGMMSNVTADMLFLAEQIKKSDDNCHITGFTDNVC

KTERGVRYQGIKNGEFIIKEIEKLGVDNINKISFIGHSLGGLIQLFAVSYLFKKYPETMY

KLKLENFIALASPLLGVLNENPKIFAKLLHIGIIGKSGQDLGLIPMIEYKDEPFLSVLLC

AHTRNVLRRFAKRTVYANSVNDGVVPLYTSSLLFIEFDDIRQKMKQKQNSSTDNLEVVTE

DYKTEENEKKQNKFFWKPLIKLSTLFAPNMSQDSSQDGDEITTNKNKEETENLFELPKMS

LFDTAQNVLLQPIPKMNYIIDPSTRDSTIIHDKVYTAEMIEKIIASNEYKKMIDINISKD

EVSKSFEQELEIAKLWHHKITWRKVVCALEPDAHNNINVRRNFHNAYGWEVIDHMISEHF

VSPSLETPEEYYVEETNDVSDYYWLLNFDEESLIDGGVTGMIPTISDLVDRWYNKFRLTV

SGEENTEETGDDELSRRMSEFSLEEQEILFNYIR

>34-9_0373

MELIDENQDFTKSHKRYDKLIAKCENIIETIFKSITSKKLIYLCFPPETVMDFEQELVLM

FENIRQNLKGLNKDIVLTGLTESAGLEERLNSLDEVIAIAIHKSEVLKNIVDQEGWESER

VQRIMEEEIFDPVLLNMSDFIRNDQYNVLVHSLIPELQKRNDFLKDKVQALEKEALKNID

ILSDKSIKINQSIIDNINKYESSSPEYINSVSQLKKAAVSFIENELDFDFPQAQ

>34-9_0374

MSFSNSNVGSLKQPFSQDEINNYKSTFTSLDKEQLNILTGDSLKHLFIKSGITNDLLAIV

WSLADYDNKGFLTLDQFSICCRLIGYIQAQGGVLSKPLGEGDYQVFVKLPDFSGKFKPNK

NVSRQPSFIINRQASNASLQSHNNTQIPLPTPQEISQFGQLFDSYSKGQSIISGDDAREI

LQKSRLPTQALVEIWNIADQNDVGYLTKEQFVIAMFLVQLIMNNRVSLPLPDVLQPSIWN

FVKQNLSQSQIPQRISSPVQRNSTNNSFNAVPIDQWALNVETSKNFDAIFNSLDKAHRGK

IGSDVLVPYFMESGLSQDTLAVIWDLSDINNNAEFTNVEFSIAMFLIQKTNSGIELPDIV

PQSLLNSSLSLHGKTDGTGSSFTNDSNQFNANSLSIPNRNSKPNFENAQNTSSRSPLNDL

LTLDNTGFSSPNTTGNNNGVFAAAPVSNNNANVTPVKKFKPSSTFGQSIIAEEPEPIQEP

PAIGKSTTGSSPSPLRYNYTSNLPAVPNFASISSPVNNQRSSSVSGVDNSNSMAISNATT

DIANLSTQTRSLSNQTKMAQDRKQRSEQELAKILSTKAAIEQRLNALRQAYTTETALAEQ

AEKQVQELTAEVTALEQESQVLETNYATVKEQHDLHNNNLNLLLQKNNELKQKISDLNNN

NEILSKDLDFKKQEMVKHEGLVSVTGKQLELSEINSEKLQNEISLIDQHFSTFLSKRSEL

DNYEKSLQEQHNLMQGRHAELEESFQSLDARQQDLEQKTLEISKQEEIYNENVKRLQEMF

ADFQIQQQRLEEERQILEENEMKLENDRNDYLDRVHAFATQDLDDQVATPISPVNEHTGS

AIKLDEVTKNIEPSEGGATQLSTDLEKLTEKESIRKVVNSMDDVPIQQSNTSSLINNGSE

TNNRLSALNEIKEDEPVDKGIPGEFEPAEKSINTNASIIDEEDITDEQNIKDIPEVPPKD

VNEPSIEKLIDQENASFNNSPIIQSKTIDENVEGNEVVGLPISGPISTEEFPPLREIEYE

ESDSSDEESEEVPAGSLDEPVVIDDVQKHEVDDFDGLEAAQEEDDEEEENSDADVTASNT

VPTNSNNPEWDALFQGLEKTKPEVPEKDVSNKMAVSPKDLAIEELIDMGFSEEDSITALE

KFNWHLESAAQYLITKN

>34-9_0375

MLKFVNQSKVIVIKNNTSFQKNLFSTSSVILNKDTKVFKPSKEDIPVWEEQISEIHTKIE

KLDSNKEDNLFFNEHDVLPLRGDTFIPNKEQIQDAVAVPKDVLVKKNLDFDLEQVINMTM

RHGDKKKATETIMKALKLLQMKVYKDKIPRGNNSLEMYSHIVPEVPFEFKEEDNLNNVSD

GEKIVNDMLVNDTMQYKQKFDNDIKFKETEYSLPSQVSMLKYCLTQLAPLCKIRNSIISG

RKVTTPVVLNRRQRNYRAWKWIYDASWNRSAKDQSIKLYEELLKVYDGTSSLYAKKDQIH

LECVQNRAQIKNVIRKQ

>34-9_0376

MSIYNNAVDHEVLKLSYGFNDHNSILYDFIVSYFYRSQNLDNLAIVDIKNYSLLFTDLIP

NRLFSDIVQFIATKYNTSLSTQIMNTIIDLPVFKKLKKDNNSITLQAKDEKHIELLRSMY

ESYYHIDKKTKKITHMCFPKATFDRLLQLLLNPLDFTTSLCDYFKILSKLFIPLPMVNNI

LTIPFGDYNICNNSKSTNIKILFQKFASEKIITLNEKNEHIVQPKLEVFIKPDSEIIRNK

EVKNFVQTVRNIMAYNFGFDKVNELDDQDIPETQEELLKSNGEDKISNEINHEELKLHQS

ESKNDYDKNTSEESDYSDIAFADDSNDHSSTAESLDSSDDDSDIVNLPGNNQSG

>34-9_0377

MLRSTVLKRAYANVPLTGAPKITKLNNITVITKPSVSKVKDVSLVFGNAGSTNENYYNNG

VSQLLAESFTSNNAENAFSNGFKLNSSVGKEFQTFSASGSDVSKALSLLQESALTQETLL

NNFKSVKDSVAAKIAKYETTEYAKIVEEHLHATAFQNTPLSVPVSGTSETVSSLVDSDVS

EFINKALTQNTLTIVQQGSELNHEKFVELVSKLSLNKVSNTITPVVKSDFIGSEVRLRDD

TLPKAWWSIAVEGESVTSSNYYVAKVAANVFGAFNLVDPASRKQGSKLVNDVLEYEIIDS

FNHFSKSYKNTGLWGFAAETTKIQQIDEFTHFALKQWNRLTVSVTDLEVARAKQALKLQI

ASEESIESLAGLIAGGAEVVSLEEQFEAIDKITASDIKNWASEKVWDRDVAVSGTGQIED

LFDYMKIRNDMSMMRW

>34-9_0378

MATSTKNAKKANPFKLPLILYNTIAGVLWSIHLFNTIQFILKNDQSVTSFYKNTEGFLTI

TQSLAVIEIFNSLFKIVRSPLVTTAAQVSSRLLIVIGSFQLSTKPISSTIGYEYITLSLA

WSITEVVRYFFYAFNLIDIQPKILLFLRYNMFPILYPMGVTSELIILYKTVQINDSQLLK

FIYIGSMLAYIPGFPVLFSHMWVQRKKAMKEMFSTKKVD

>34-9_0379

MLSTKYNCAVKSLFKNVKIQPIVRFNSIKNHNIKNTHIEYAAPKPVENIKEYKDFLPQPI

ATIKSEKNINIKEYNIKDANDSKLLPLKLASHLYATLNIYTRPFTVQKGDTIILPSQLKN

LNVGDEIDFHDVTVIGSTNYKIVDHPINPDIFEIKGRIVEKSKSAEFYEDRTTKRNRRVK

HIVKTVNMTMIEITHLRLK

>34-9_0380

MALLKKKQSRDQYHSRKSSLNSSVSSASSSHSTVPKSFVEDKTSYSPSSPLLANTHNTSM

ASSKSQPVTRNSSRMSSAPNATNNGSNSTIPNTPLYSASGNPPSANSAVKRFSLSLRSFS

SNSSLNNNNTTLSKEDLSKPNDLSALSSPLNGASLPDSKLIFDWDVTDPKSWTWQRVVLW

LQVNEFSPKWISFFRRSQISGNDFLKLMAYENFAKIEKYLPATLNSSYKRFQHVLKRTME

HNVIYQHTSNSRSVSSLDSIYSRKSNKHLRNGSDQLSLNSSTNLPVNKSTEKLLGVIEND

EEHESFEQNSLNTHNDISPTIDESNISSTSTIQITTTENSKKLENSIKVPKLNIPVPPND

SKLRKKSDNQKHESLSALYRRSFISIMGNSNSGNTESVTNKEDVSVSPQNSKNLTVSNKP

TSTDSKRNSPASSPASSGYSFFKKIHKHNPSDLSSATTPIAMKNIIPELIIDEKFLPKEN

PLTLDETDLIKPDVYIYCTKDRQGFIPLNVKNVKDSATLKAAIATNNNINHKNYSIYLVD

YEFEIAEHALNDEIMKKIIDAGFPEKYNKFYVRNHLKIQLNRNRSSSSLLSQQSRKMSLK

STVSKTSLGSTSDIDEVDNGFKYPKTPSHILENAQDYISASHTIRPNRSRKSTVSKNTSS

HVNEPYSNTQQTSSNHSSFKVIRPDTSNKIDFNKKRETPFVKLNPTREAPPPPLSPAVSA

RSGSDNELKKRRRPPPPNMVYNSDNDSDINSVKTKISNQSLSFLTATPNVYNNLDGDSLV

HQPPKLTFSRRSSLVSLSKRSSQTGFNKPDSQQSNTQHGHHTRLSRSNSSIQSSIFTSPP

KLLKRDSSRRIVSSALAGGDTFDENDIVFKNVPEFSDSSNDDDTFIKKGYENSSDDGIAR

RIDDALIGKRKDKVEKINEASEDSEPQKEVDCLLKEFELSLKSEKTNQNEITFDGKIGES

TTEDSDDSGIAWVHSKTKKKSLLEGEISSEGDHIVKKAEKDALSMSNTSDSDDGIVWSHP

NGTVKKKNRVSSTKARPKLNINTENVFTDSFNDLDLQNIIHEEDNDEFKTANEEQDVRKD

KKRLSLLSKDDKVGFSFDSESMLGSLIHESPSTQEPLHPRNTSLIARKMTLRPSADIVYK

NLEVFFPGRDLDKPIFEGDTSIVSPKSSTSSRFNRSGDRHNSIIEASTIQHSSEKDDYTF

SSSGGSINKNMSNTPNTSINSHSSNKPMRKKTLRILANEALAITKKKESENNKLPNTNIA

ALNQNSTTTESVLPLLKRQNTTKLWGKKVIEITEKEKVVEINKNKNSKGQYREFAWIRGE

VIGRGSFGCVYLGMNLTTGEMIAVKQVESSTESQIEALRSEMDTLKNLDHLNIVQYLGFE

AKNNVYSLFLEYVAGGSVGSLIRMFGKFDDELIRFLNQQVLQGLSYLHNQGILHRDMKAD

NLLLDLDGVCKISDFGISKKSTDIYSNSEMTMTGTIFWMAPEMVDTKQGYSAKVDVWSLG

CIVLEMFAGKRPWSNLEVVAAMFKIGIEFLNLCFRVDPEKRPTVDYLLNNKIFASNPNFT

FMDHALAKFTNDNHKTMSLPAEKN

>34-9_0381

MFVSAAKLKSDQHNLSSERGNPSINDDFHVTIDQIFKTPSHRRKHHKVKPHRDIHIQESY

YWEIDQMTIPTLNNNKIKNLHIKPGKSTHFIYYFIASCYIPIITAVIGPISHMFSIACLV

NKWRISRSSKEEIRDTPLVLILNCVSLFIGGVSNVVLLLHFSGKIRYTIAQKVNITGWTF

SGLILLVDIILFRCIEFDSKLHEKGIGFWFAVYTCILYFLCGGLLSIHLVGYRRGKYKAT

FNLSHDERAVMLYTFGFSLWLMWGAWLYSKCLFTDVSYGVSLYALLCGALTVGFGDIYST

KTSGRIISMVYFLSSIIIIGLIISLTSQIIKNTFQSIIHLHLCERARIKAVKKIEDYNEQ

FDPDDKSTGNYESVSFFDSGDMISKESFAFYAYKKGNNYTDVIQFTKMRKLHIKYRKFAK

VSGFLTALSLFLIFWLFGSMIFKFSEGWTYFDSIYFAFLDCLLTIGYGDFILTTGSSRAF

FVLFALAAIPLMTSLINAIGDSLSSLGWSFVKYAVIAAQSIYDISYYILGVFYELIGKKI

RKTKADDDNDVVFSKRPLPFNLSSSKTNQLKEELQDLLQVSFDINALKEETQREKYEYHW

LEVCSKLSKNYELLSHISPHYKLSFQEWIDYFKLSFYANKKILHTKHFWLSHESPIRFDT

NEPRFVYLQLVHLMSEHVQFKKNEKMQGHIDNYINPHMLGRHGNSPYDSESENSEADVHN

SQQSWEE

>34-9_0382

MTSHNEDISSVYKIKDIDTNQWQIDDKQGANAKLRIDNTKSVDTTRQIDHTKSVNTKRKT

DSMENFNTKKIKYNNNIDMKELVLPQSNNSSQFCYRHNPIHHQNKSAYHDCIKTKSNEID

IIHHKITEELSSNIDDNQKKTIEDFVKIFIKSKDSEIHSKHFNESNENINHNNIDSFDKN

QILSIVLNSLCSPQLSHIAETCQDLIKLDFLTSLPREISIQILQYLDCQSLCMASQVSKQ

WQKLADDDTVWYFMCLQHIDKKCEKCGWGLPLLQMKNRRYISQNNYSNFSNQNSNNSMNS

RRDLLRDINVIPILADFQNQSNTNQDDENEENINDMKSGINNEDNSSVNNRNNNNRLPPP

LKDLKNSAVQMRPWKTVYKERFNIEKNWRRGIFKSFNFRAHLDSILSVKIQFGLLFTGSY

DNTLAIWKIPSLSTDYYNSNKKNTNEYYKQLMQPQLLRRLTGHQDAIKTITFNSTTLITG

SLDKTIKIWNFQTGQCISTYRGHTDAVLSIDSCGSYIASGSADKTVRVWHIDSRTCYTLK

GHTDWVNCVKLDYESRTCFSCSDDFGIKMWDINENKLIKNFKGHLGQVQKIVLLHLQDDV

NLITDDNCIDQNNNRIDSNNVESGLKKGVKFPTHVVSCSLDSTIKIWDVKTGKCIRTQFG

HVGGVWDIECDNFRIISCSRDKHTKVWDLQNGKLLLNFNESDNNTEDLNSNIINGNNGNC

GAIKNCLDLGDSEFITGDDSGFIKIYNFNV

>34-9_0383

MELVNHLNDRLLIAIPKKGRLYEKCSKILKDSDIYFKKNPRLDIALGTNLPIAIIFLRAA

DIPTFVNNGKCILGITGVDEVQECEQNDQISLAMDLNFGNCKLQVQIPENSESIKTVEDL

VGKTIVTSFVNLTKQYFMKLENCTDVTQLKTKVLYVGGSVEASCALGVADGIVDLVESGE

TMRACGLKAIETLLSTSAHLIENEEKIKELSEDLQKLTKVIKSRIQGVLTAQKYVYCNYN

APQVNLPELLKITPGRRAPTISKIDDEGWVAVSAMIERKRKGDIMDDLVNKGASDIMVFE

ISNCRV

>34-9_0384

MAFFNRFSRNYKDIDQSTKHSLDDFINRSDNKGKCCECLSPSVTFVSITFKCVLCSRCGH

AHIDNMPNSVVKSTVYKESWNKQDLKTLKNSGGNRENYKIYNGKNQPFPYDADIDKVEVD

KYLRDKYIFGNYMTKVDERYLYNNICSDDDVFEDNSTSKRGSVQNSSFNSKIDNKPDLPS

KPRPNTKNFNPSSAVFTGEDQFTSQNNSSSYMAEPQFTSQPKPAVFDGSFDYQPYNNNAY

GYIQPDVLEKVREQQVYQQQVMTQPSNQHQEIMNMYSQPGVYQSGVEIGPNNPQYQDILN

QQNLIQQQQYQQQLNVVVVNVVVVNVEQQMMLQQQMFQQNQMNNNNGFY

>34-9_0385

MSTIRHLVNIKDLTDLEFQTLVFKAQEFKKSFKAGNSEDFASNSKKLLGRTGALVFSKRS

TRTRISNEGALTYFGCQPMFLGKDDIQLGVNETFYDSIKVISSMTSCILARVNKHSEIQE

LVKHSSVPVINSLCDLYHPLQAICDLLTIIEKYGVDNLKNLTLCWIGDANNVINDLMFAC

LKFGISVKIATPKGIEMHEGLVKEAAELAKKNGCVFECNHDAKYSATGSDILVTDTFISM

GEEWQKVAKLKQFEGFQINDEIAAVAKPDYTFMHCLPRHTEEVTDSVFYDEKHNYMLPLL

LLKYL

>34-9_0386

MERDDNLDVELTVKTPDRGQQDSIDNNITPPNIYNDNSFITDNEVNASYRKLNNKSNFSA

ARKPQLNTLRQSNLISSSNIDSSIKRSDTMNSDFHRKTTFNTLNGALKSPRDPRYEATKD

KLVHSSSIPIKEGHSRKKSSDSNDDMQSMYSVSSSITASFSKNFLSGFYKNKIKRRRKGQ

IVLLSEKYWMKDENCKECFYCARTFHAFRRKHHCRLCGQIFCSSCAFLVPGEKFGYEHKL

RFCKICVEHLENDLDSSEDDDYTSFANETIEHDEDDYDNTEVKDNLGIINNASSSMLAND

MEQDFISKNTADMASIFGEDDAKLLTESFNPPKLTIAARKTGESLSIETSHMRPRSQSRH

FKTPVKDFKTGSLSYTQNTLGSNKDKLTTPTHMSKYNSRATYGSNMAHVALMNQAKHNSM

ANISFNNSNGNDYIFPNNNDGNKLGDKTTGYVTQLLSPLGNSQMNKSRNSEIKNHSSKHY

LSQSQIFPENEVYWQNKNGIMNAAHSANIYNNNFVSNNKSNLSFQSSDQNIPLNKRLITV

KSEASLSKMPTLLTDNSQDVKNQQHTNQLKNTYSNVYNDYDNGKNNYVNDIANKRHFSPP

VSNAHLHKDNKITQDLGTTQRIYKTLSKTFTPTSSFINRSISFNGTANNLAKYKKSELDL

TYKDHNISDIESINGDEEDEASMSIFAALNSKVDGYTPEQQSNKINFDFTNLEDNIMKIN

ENNIGKKQSQNFSFDQNVNSEGLNNPVFRNRQDTDAYKRAEQSLQRMRSRRRSKPKFSFS

TVSSSTGGYLSSNIFLQNNNTKLQSTPQLMPSNNVDMNKATTASLQPTKINASGTTSHSL

IESSSKSESLTVSPTLEEYISNTSETLLFHLRLIVQQTLLDQGIQEELIKKWRFLIEDLV

ICYDRIKIHTKLSDSLDYRQYIKIKRICGGRIEDSKFVNGLIFSKNLPLRNMPKNILKPK

ILLLMFPISYEKAQDKILSVDVLLTQELQYLKSLVSRILALEPDLVFVSDNVSGQALGML

EEAGIVCMFNMKPQVIEKISKFCEADVVNSVDKLVVNVKLGICEEFSVKTYRYGNTIKTY

TMLTGCNSANGGTVLLRGLDDEGLRKIKNAVEFVTYVILSLRFESCFLKDSFINININDY

KNSFSGSEIQHMETAESQFLENFNKRLMSTSPCVTFPIPFLLQRTQYLQAKIQRISNLKE

DINFYSDEEFESYFNKKPFLLDIKKKGNLSNLDLRYYTQLILDKKLESLQEKFNNKFRQW

SIFSSLSDNLLSIGAHQSISLLHSMVSYNTGTPCIGPILISIEYYWDNDVTIGQFVENVV

NTVDYSCTSGCGGLFIDHYRTYVHGSAKVDVIVERLPSKVPSLKNIILMWSYCKKCKIST

PVLKMDSDTWNFSFGKYMELLFWGNTMHKANIQGCKHDVFKDQIKYFGYNDIVIRLEWSS

IEVHSLVTPKQHISWQPQKDILLKLDLLNTIIEQVHLLYGSIVTRLKRVKLDSSIESLNI

EGRAKSIELQQSSINEKEIFLKYANEVYMNTPGTDHLSLNAVVSRLYGKSLTWMKTVTDF

EKKYLPSERDIARITSNQLKKLFMDDVDTQNDSKMDTLKDVEENGSDNAVEMTPDPHTSL

EPLFSAQNNLTPDKSYSHLYKVKSNLSETDSISRSHTPDKLSVHESTDNNILSKHLRTMS

QSNGNKNKQFFLKNINDGKQDISDVQKNRDNANSENKEELADGKVGKLANYFNNMYFDNL

SREFEHQREQEKADFQKKYQYSNKPTKIQDSKPKVEVFQNVEDAVAENITYSSNLNDRLG

STSMNREDSVKTSNNVYSINSDNSNADLETSLEAWENGEEVRAEQLARHLDENNPRISII

KETDDSENNDEVNNKKQQEKGSLLQLLKNFWADRSSALWEPIHLPLLANEHIFDNNLVII

REDEPSSIISFCLNSKEYFKKMEQTYLDYSTNIGLDTSDIENEVMKFENKEVVTEKGEDE

DENKDENVQSNPEYIKKLMENPALIEAMMNKPTPIHLRYQVEDENVVMACKIFFADQFDA

LRRAYNIINPLEFIQSLSRCVKWDSSGGKSGSVFLKTSDDRFVLKELSHSEIDAFVQFAP

NYFKYMSESIYQKLPTAIAKIVGFFQIQLRNNDTGKTVKFDCIITENLFYGKNNVRIFDL

KGSMRNRHVQQTGKENEVLLDENMVEYIYESPIFVREYDKKLLRASVWNDTLFLSKMNVM

DYSLVIGIDNNNHKLIVGIIDCIRTFTWDKKLESWVKEKGLVGGGTKEPTVVTPKQYKNR

FREAMERYILMVPDPWSKSEHSK

>34-9_0387

MNFLGSQEILSNLSQPASYLYEKMSEQGSNALYNQQQIVSLTGQTMENSRLSLQELIDKK

LLQVLKQNNEVVFKKVDITEATKKLSMNKDEQLVYSHIEQSAREGIWVKTIKAKTNLHQH

IVLKCLKNLESQRYIKCIKSVKFPSRKIYMLYNLQPSIEVTGGPWYTDGELDDEFISHLL

TIVWRFAAEKTFPEGFNNYAKVNMKTGENEKVYTKNVVNYVTKQEILEFIQKINISKVEL

SIPDIESLCEVLYYDNRLTKVGVDAYKVTLQSVLDLMNDNLVQDKKEDKNEELQFLELFE

DSYSLYKSKSFVEPTLNDRETVYFDEWTL

>34-9_0388

MSWTGFKKAVNRAGNSVLVKDVDKTVDKEYDVELRRYKVLERAAGNLTKDSKGFLDALRA

VTASQVTIAEIISNLYDDSKSYNSGYNVGNYYLQCVRDFDSETVKQLDGPFRETVLDPIT

KFSNYFQEIGEAIKKRDHKKQDYDAAKSKVRRLIDKPAKDAGKLPKAEKDLQYAKDVYDQ

LNDQLKQELPQLVSLRVPYFDPSFESLVKIQLRFCTEGYTRLAQIQQYLDEQSRDDYANG

VLDSKMEQILQQMSSLDICALGYK

>34-9_0389

MKNFILLLTKFSTYFKMIIVCFINNLVDKHIIDVLNESRTLSKVNMSRSNNTSQAESFHS

EKRDFSFPLIDMDTEDLRSRSLPHILGDPLVSDIKVLPSGPKFPAILEEPELEENGVVTN

YKKKKFILKRFDFKTIIIDKKRLSAKMKILVYTILAFSITLLLRVSYIQTSLNNCEDKFN

SLKNNKSLRNLSKRDIIGYFLFAKSSSLNNFEEMCSVGHMHKDRLNVIVVPAILTMIDYT

ICSDYPTSSKFLKNLTLLEVYHNLNSLDNNLKLKMFIWLIANFAFVMNLKYNQFVLRTIN

GLESYDIFRLFQVTLNNNLVYLRLITFYSMYRIIPIGTLLPLALLMKMMMALTQIINLRF

VQQKDPYNVFKLSMTALVWLVSCVAFGQAKSYESLLNPKSNLVYIFKRLCVLMACKYYAI

LVFYYKLTLIRLDAYLHENRLNLLRKPLFLFKPVLLSVLNAEVNLNFIINKTRNLLKNGC

FISHDEYIIAKCLYIETFQNIRQPSRDELRQDEVIDDFATVVYLTNKRLVKWTNNGYTQI

NNDSFQKLSWDFFVNKFYGRSLEIEELIEFGKEENVEEAGFMNELNPKDTFYVENKFKGK

LIASGDKNKYCFIHPTSKDNDSVNLFLLDVASKKFKNVLLEEYLIADIIDCYYFQEGPLG

IDSANSLLVVTRSVEIIEVDLYSYRITKKKLNLLNKIDNWKRLKTDRMAERSIIYDTNRK

YIGQYVRFRGWKVLPFPVELNTMNTMNNVMMKKGSSMNIINNAGTSAPMMKKFKMFPMIQ

QTSDPTSSMSQHVADANSNDIILKVKTLEHLGLIYEIIENEMKYKNKWVIRILDCITGKK

IKQFEIPNKHVDINSLRIAHEKDLKFCGFCGFLSCKKLHLHYLVRNPNNKEITLNIIVLI

NGRVKNMKKQRICFRTERDSRDVRCHGINEMDEVYKKIKIPEREVEMIYNKYEDGYVYFS

KKNKNKFYKINLDLFKVSSIPYSFENHGFENERIVGFDNFKYNIPKAITDEEIRKNDLRE

LENDREDLLFEDEMKKKRMVKDEFKRLRYLYDTKENRMYYY

>34-9_0390

MEHLAFTLGGLTAAGGAMGYVKSGSKPSLIGGLAIGGLYIANGLFIKNDTFDDNYKTAVT

GLVGTSAVLSALGLSRSIKSGFKKPVPLVLFSIGSIGLGYYAFKYNKFYL

>34-9_0391

MSINSTKNLDLTKGKTVPAIVIGSGLTGLTVTDTLLNKYNLKNVVLVEKFGSIGGNSIKA

SSGISVSNSEQQHALNIDDNADYFYKDLIKSYKLADDNPLLQKLSIDCNKAMDYLNKDIG

LSLDKISQLGGHSKRRTHKSSNGMPPGFEMISSLKKRIIDNPNVDIQLKTKLVDFNIDFS

NGIKTCIFMDLETGKTEKLYTDNLILATGGFSRNKNLIKNFLEADISKPQYKASSSHDII

PKELILKKLDSLPTSNGEFATGDLLEILLAKPNLNFDFKDLAEVQIHPSGFIDPKDPDNP

SKILAGEQLRGNGGILINPLTGARFVNELDTRDKVTFAIFEQNVLNDRVFLILSEKGAEY

NMDTIGFYQSRGLIKKIKINELYQDNKLKEKIVSELRSYSILKNDRFGRDLKMNLFEEDL

NEFSEIYVAEITPILHYTMGGISINEKGLILDTNSQVINNGLYCAGEQSGGVHGKNRLGG

CSLLESVVYGLTVGNEIGVKYKA

>34-9_0392

MIMDLFWIIIIDKQVTGTSVKEIPSLLSNNQNISLETAEYLQKISRKFIDKTTYPNEIIT

EVDDPFVFKCIIDNYDKTQRVFVVGSYVQNFNVAQSPNILNKYLLKMKEKSEVYRMYNDI

ITESQKKKFIEYINKEI

>34-9_0393

MVSNIHLVVLVHGLWGCDRDMEFMNKTLLDHHANNIELSHLSELEDCEKDKQYNLIYTSN

QNSKFKTYDGIEICGKRLQLEIYDVINTIYQKNDNVQINKMSIIAYSLGGLISRYCLGLL

YNDNFFSSEWIGHLSENPIQFVNFITFVSPHVGVISPYSRVAKLVGHSGVQLYLKDKDKI

IYQMSLPNSSFVKGLKKFKHLKLYSNCINDLKTNFFTTGISHVDPFKVFYENNNDIKWVK

FVDGYKNVVIDYESSHLTIESKDTNDFEHEEKFSVINQEIAKEIDVDVELPAASKNNKFA

QAIISYYEKITSIIFLIIFVAFRTPYEIILSYIRRQKFIQKYKTDFKLINHIKTNMQIKP

DFKMQSESSDASESENEATPLMITATKSNNEVIAESFNINDTSALSLAKRTFKSLIDIDN

KPSYYLRELIDLGYEELFHLKQTFFDIEIVNPSDFELQHRIISNLNKYNLKWERYPCLIR

ESKFTHAAIIVRYDKPKDNFTEGTSVIKHLIDHLSL

>34-9_0394

MYANEKRQIITKIRNKEILIVKNGTYLFFPHEAHLWDLEGDSVIMFKFFIEKKKISNKEN

DEEHQRLLEVIKQENIEGNRWVFDNTFFGSGSFGVVCGCLDMHNQKKLTVCKIIKDTIMP

VDKVNEKDFILAYKKKFAKPPLLYSRVKNEKNIMQNLNHPNVVKFIGHYATKTTYGDELD

NGDVFWKSDYLIFQEQAFGGDLFSYLLDSKTLKLRSLPEAESLLIIYQIMQALKYLHSQN

LAHRDLKLDNVLLETPEKLSRIMLTDFGIAKQTPLRMKTCIGTPEYCAPEVGDFHKNTMR

DIIRNKLNNKENEEAVHFGYNKKCDIWSLGIIFYYLLTGKAFFEKKYDQGVMSGFDDYTL

DVLQNWIYGKVLKCKKKLSISHECIQFLNHLLTINEQNRHDILDCFKDRYIQGHKKSLDN

IYRGILKSSRYNESVIPSGNKAKKRKIT

>34-9_0395

MNQNYEFKTPATPPAEDGMDTPQSIAELDDDDILDLNITSYNKLPPITPLVEHENVERPE

EDVIMIEDSDIEEINYEPAIAFTPTAIYPHRERLIHDEEIEIVKRRLSDEQQMKFLNYCE

ETSAEIQKEYIKWKNKETEMGDIKENEHLIITLTKFKRLIDFVWHTIDWNSKDTAVLLQY

FESDDFEVLEYIQIFKEVQDLGQVSIFLKLADDLLSTVIKYKILETETESSIFQWIFKVF

YILDDIFMALAYDLKTQTNFREIKNRNN

>34-9_0396

MTENIKTLEHSSDKIADIKVVMQRECEWYSTVDLNSALENLDSISDREMIMNHLLEINKQ

SQETDKFLEKLYLSNNKLNSLPMEISFLHNLKVLDLKNNNFHEIPFHILPSSITILEISD

NKLDKAINIPQNKINELVNLKYLNINNNNFTSINQLISLTKLPSLKIVDVNGIPDIDLLP

TISKDFLVNLRSYVTNTNSNSTNMISNNSHSKYNEYFKRLSVLPEDVDEDQTFDSSNSIL

TSKNHNNNLNTQNTLNNVSMKTKTSLFKNPAANLHTSALSQEIGRSRTPTLTDHPSATVQ

PMFGTVVRSRTSSVSDISGSNSANIKKNIQAMGNLNNEGNTLNIKKNANKNRSRKSSTTN

VSTTSAHGYGNLTPNNQNHRVSSFSFEHKTNLMDDQRKVSKENGKFEFGSLSKDTIDQVR

KKSQIIKYDKLLICCRRLLFTFTESQQTLRRVAQFGKDKTIAMNVIQLLYSTSHAIDNLV

EVLEKCEEEETSSNGDVNVNSKKILVKTCQDIFPFFKQIFKLLKTNFNSFFQNIELCFLR

MFYMNLMNSYTELYNAFMIIKHKSTNTTPLVNEKSRKSSLVKSDSLESKSNGVNSPNLFE

NSVIINEMPNNSVLNSNQSTETSSVTSSTKSVSGLNQTQIPTITPILISPPAISSAEPSI

LSPNLSQGNIPTASASSHTTAARSVQTTSEVHKRQNSEDYKLIQSLKHLHMMISNGVVKE

IEEMIIGNEVFEGLLQSCKNCKKIDVLLNNEIQSQEKNSKIWDLMSKQIKSVLIFLSSVK

QRLTELTDIISSENNKNLLSENLSSLAKLTKEITILLDKSSYKPVSSNNNNL

>34-9_0397

MNNLNNAGLFKICGVRELARESLFDKNLHYYTSIHKNKKHLIMFDSLKKLILDFMMCCHT

KMKLSTPTLLLSFKILEKYIFMRNKTHEYLQELIEDRLQFQLIGIVSLWIASKYIDTKYK

HFTIDTLLELISAPQYPKSYWTRFQNEKNNKTGSSMSTPIATPKLVTPLGKTTNSKEYNY

DSMRLDNSRQHNIQYNTEKEYTENIQKITNKRNSLKRHVKTIELDILNKLDWSIKSTSAI

NSKNFLFFYDTNDINQLKFGSQMLSELVSFHCELFKNRISIHDISNAALQVMKLAILNYK

MDRFVHLDKNSIAYKIVKLFGNCHKQDQLPFSFKLKYFPKNTIQYPVFLKSLLRYVDKMY

EINEANAVQKLSKVLPTTPITPDLIKNGSKRSRQDAEMDSETLVTNQNIGLDKDYSQGHL

EKKKLMFELRRESNSE

>34-9_0398

MESDKKRQKISTQPDLSKPFKEIKLRKPLKFQYSPEFVDSLKAENIKETFKSKHDELLLV

EKLVAGGYGSGTQRFKMTFDEFYRRLNNGEQLYLSTQYLMDDPDSNMAEFEDDSDGNESI

GGFGDNPELLNGLGSDFEDDYVVDAEDDEDDEGENPEEMIERVEDIFQRPMQKLIHKLPL

KFEFTDKLILQQINLWMGSSAKVVKYTDKDFEDLDQIGKKVPNGYTSTGLHHDHADNIYI

PVEGHKRFTLFPPSLAKSLYTVGDVEKVFENGIIDYKMNEKAPNWVTVNANGSQLPDLEP

FNQGKMNHKVDPPSFSKINPLFLHLDEIVDDNIRAKLHEKGSQMFPEFFKDEVLSQRIII

DLSPGDVLYLPAGWFHEVSSRGKYHIACNYWFVPPDNFDDEKMYKDDLIYDINAMNQKAI

DFYKMTTEEEE

>34-9_0399

MPSIDEMDALKTPVSDHVTLATSLKKTSPSQKKTYNKLKIKQTPVHYTDDDYFPSTRVTA

VRVPPEMDESLSKDTVLLNILNILYENESGNELENGLTVKQICDMLLVKDPNMISLSTKF

ANLISAKINAYAKKVENRVRGDYKVKIKYWIIRKWAKGNSPRRMVYIYKGLLPDDYTEAP

PKMPDSLPTSMEIGNKQEVGAVFDKSTVKEDKIKEIPGKNINYSTIINGTGLTRLNSING

VMNNSSKSKTALPKNNNKFSVNFNRFQQFGYKGMSGTLEEKNGSNTKFESLEDINKSPLI

DKSLDLDDDCITNNLTSSANEAKQQT

>34-9_0400

MLRFNRVNSLLRNPSRFTSLRFKSTLTAEQISLLNEDRVIDKTDVIIIGGGPAGLTTSIR

LKQKNPSLRVVLLEKASEFGGHTVSGVIMEPRAINELFPNDINKSGIALPENLLEKVNDE

SLKFLLPNGWKLPLPEPPAMKNKDKNFVGSLSQVVQYLSEKAEELGVELYNGIACSELIY

DDKKEKVIGVATQDMGIMKDGSPGDNFERGMEFHSRQVVLAEGCHGSLSKKAMKQFNLRE

GKDDQSYGLGIKEIWEVPKEQHKKGFISHTMGYPMDLQTYGGGFQYHYGENLVAVGLVVG

LDYKNPYISPYKEFQKMKHHPFYSEVLKNGKCLSYAARALNEGGFQCIPKLNFPGGCLIG

ASAGLMNVPKIKGTHTAIKSGILAADAIVEEFKSKNFATVEEIGEDEVLETELEVLNLKN

YQESFNKSWIYEELYSVRNIRPSFNTKLGALFGMCFSGLDSYILKGKLPFTFNYHKGGDV

LIILTSVSRTGTHHAENEPSHLKIKDLKDYKNKSFEKYQGIEEKFCPAGVYEYIKENDDV

RLQVNSQNCIHCKTCDIKSPIQAIDWQVPEGGDGPKYTNT

>34-9_0401

MVSTPISTTSTISSRIVSNQLMNTKVSEDDSLFHICDRLIKRLGQYKPLKPYIESAEMLA

EYSSEKQEDLFNNTYKSLDNCHTLNTDNINNNSRPSSTLYRKSSQVDSRRASVSSFQSNG

ITNSTINNSSIHSPSVASTHSSDILSVSNNQNIYLNNIESNLQQIPTFTIGSLPPSRTKD

PVTTLWKLFQMGAPLCIIFNAIKPQYKINVVSSDELKICKKSIYDFISSCKIHLEFHDEE

LFTISDVFNNGTFTLVKVIEVVLTLIGYSIKIFPSIPTTDPSLIKNQPIPENLKKDDRYK

IFREFLETERKYVYDLETLQDFNAQLLTRNYITINESTTLFPNITEIIEFQRRFLISLEI

NNNLLTLDELRLGSIFVNSYNYFKLYELWSIGQTAAIDYISKLPIYSNVSNPHPNASKLI

IQNKMELQSFLLKPIQRLCKYPLLIRELYQASSSNVMFYKELELGLEVSKKIATNMNEHQ

RKLENEIVMKELVVGRVSNWRGYDISKFGDLISHDKVVITNNDVDFVDAKIFEISLFEKI

IIIFNEVNAQGNIVNKDENSSGNMEGNSSGLKITLKKNKSTINHKALLEKENELQQFKIN

TAHKLDLKGRIMVSTITSLQEHYSLPRSIDIKWESSNEHGNFNLKFKNEDIKDVWANYIS

QFMKTNQPPSRMSRPSQRSVSMSSQLSESTSSVSLSTPISTPAIKNNLRLSHGKRQQNRN

SKLRNISETSQLFENEHRNISESFRLSIPAKQILVRIQRGLDFHSILMANDIKYEQFYTL

VLKKLHTDEYIRLKYQDEDMDYVMLEGEDDWEIVKDWIEETNQKILTVFAF

>34-9_0402

MSFAQKLLKIDAFTKTKDDVRIKTRSGGLITLMLILTSLILIVNEWKMFNTIQVIPSLVV

DRDRNKRMNMFVDIDFLNMPCDSLYLDVMDESGEIQLDLTGTDNCEKIPLSDTSCKVKGN

IYLNRLQGHFHFAPGKPFQNPVTGQHSHDIQNYNNQDFDHKINKITFEYDQDLMKNNDNK

LIQSFFEESINQVISEPLTGREVLEKNAFNQYSYFLKIVPKRFEDLTKVEPNRHKKELLL

DGTSSDSRNNKLEKIQYTATTHSKSIHGGKDEDHPNTIHQKGGIPGIYFIFDISSLKIIN

KLQYRMNWYGFILNLLSSLGGIFALYTILDKVAYKTVNYYRSKKDQ

>34-9_0403

MKQEFKSFSTSIADVLPPINSKSPSDSATNNHTKLINSLLLSDDNTNYDQPENFLEIEIV

SARTYNQDNSFVTIYKDTSENIKGTTPNKQPVYTDYEIICRTNISLFKKKISKVRRRYSD

FVYFKKCILQELALNIANNNNNSSKINIPEVPSKMILNNRFNYDLINIRLKELEKWLQII

VGHPLLRSNSKVLKRFIQEESFVG

>34-9_0404

MSNQEDEQPADNDSYNELGMNEILIEGEDDVFRNVQIGLEGEYRDLLIDYWQNLVDNIQS

LNENLLILQNPEFVNYFNNVSTNIIESDNESEEENDESLQTSIKLPMTGKNAVNYEVYQQ

LTQILKNEDDSFQQMKKVVINKKSEDYDPFRNSKLPLARIKKLMKMDEKLKMISAEAPIL

FSKICEIFIQEVTLRAWTIVDFHKRRTVQKQDILEALKKSDMYDFLIDLINNEA

>34-9_0405

MDGHYKLAITIELNTARIKAEEDKDYDLLFKILDKSFQLINTELISLEDQLSLPYYSKEL

PELELIDFTINLFKKLIVDTNTNFKVTEDIQNEKIFKYLPVINDLLNSSLATSFDNLLPI

LKTLLTCLILKVPNFTKYLYQLIIEHDNTDLFNDNTKNLMNLISILVNQCQELFRCDSYT

KVPPILQFNDIKKKETTLLCDEFNVDDDILLKEKYLVYKRLLVTFLLNVSINLSEGDLTL

GNHINLGTLLLTKKETSFLKNTMVHFDKIAGNIIIYFVTVLIREHFLIYAVFDSILENLF

QVIIQRQQFHKPIYLRLLEFQIDKKWQLPNTRVIDYRVEKRNMERVWKLFIKNSIRANFI

NAKNGVYNADQWKFINDLKRYYDGKEEHFKRNKKDWRLDQPFFPKEKDLLFLKKNKKHNV

LYRLQNNLLKRKNKDLSITSDSKKLKSKSGISQLYSQCEPENQFDMTQIDTDTLLKICLN

TFTNDLTFQPILNKLISVSKDYLEINNGVVSKENRMYNPYEEKEDVENSDNEETENEVEV

KETDNVYNSKGEIVSEKEFEFDDKEKQEQLKVLVQNLFKSQMKHDSLVKETKENNAEIKL

LNLDNCITKKPNPNVTLIIRLCSQLDDDIIRKQLQEYIMQHTLDMDNLMVTINYILEWMN

NEWLILTKKKLKEKKEVHFDKYYYWLNLTLEDLILKVDNSNKKEFIQLFSGLPLLNCNVF

EMHFSKLLLDPYKQQLGYMTLKYILMFRPSMITDVLTFLDKQVNHNTDLDLEVKVQLEQL

>34-9_0406

MTIFGSMIDLDEHLKPKIMKTVNVFKNSLTTTNKCHKDIVNNLKVLMTYDIASEPPQYNH

INNCELKNDMNQTLINTLQLSKKAKEYSVKKILYNACNHIFNPPCTRFENKHFSIELKLI

KDFNNPETTNHSLISKSSVDTVFSKRVHANSISSYTKRKSSVVTSVIEELNNMKELQQTD

DLAISLKLKKSNSQQKSENLFTDRYFNTNIFNSNQSSRASLNLQDTRKSQSPTDFNIMDF

AYSQTLNEEYDTALNKDPRVVLFPTVTCKNPSSNTHTLDSVRRLSQLAADDNANFVEDEN

DRDDESNQSSSGLLRVQSRYSDTSNNEVREDDVSDRIAKTILKSKVSMLKGSIQLNNKSD

TQALLLSNLKIKLCGYKLIYGHLVLIEEIKEKSTRFDNLSAATESVNGLRVTKPFITKEL

DLLQNTNGSLVVRNNKEICFEFLIDSTEFPSTLNCQYGKIEYRLEVYSNLVNIGNVILND

LIVIHKTLEPEVCNLIDQNDSVYKLNNALNVNNKRSTNNGQTYNLRYTDFEKSGFDISNL

IEEDDYLKKHWLNKYIKEHCKDPKEIKYLKDELTQPKIFYDVFLSSKTIILNEPFQLFFS

IKKALNCNNDWFITECSLTLEQHYCFPVTKKIINKDYENAKIEKKKYKKIFFHNVQHLKP

KFSEKRESIYVEDAVIHSDLSFYEEIFNEHKEYIRKNSADSIMPHYDELNTLKPEVNDEI

QNMISVSHKLRIGISMESDRSEVEEKGVKLKKQFSMSIPVYVITSSMHSTMILPEYIK

>34-9_0407

MSDLIEQEDVRISNYQPLVSPAYLQNEIKASEICFETAKRGRKEAADIIHGRDDRVLVVV

GPCSIHDLDAAQEYALRLKKLSDELNGELCIVMRAYLEKPRTTVGWKGLINDPDVNNTFD

INKGLKMSRQLFVNLTSLGLPIGSEMLDTISPQYLADLVSFGAIGARTTESQLHRELASG

LSFPIGFKNGTDGSLGVAIDACKSASSSHHFMGVTKHGIAAITTTKGNKDCFVILRGGKK

GTNYDAKSVADAKAELPEGSNGIMIDCSHGNSNKDFRNQPKVNDSICEQIAAGEKSIVGV

MIESNINEGNQPVSSEGLSALKYGVSITDACISWESTVTVLNKLADAVKARRALAN

>34-9_0408

MSTEKLVYVIDVGSGNLKSLINTCKYLNYEVTLITEPSQFPKANGKKTKVIFPGVGNYGH

FVKKLYESGLEEPIREYIANGGMIMGVCVGLQTFFKKSEESPEVKGLNYIGGDEIYLKKF

NDSTKPVPEIGWNTLYGNNFFYNLDPYKRYYYVHSFAAILPKHLEDADEIDGWKIVKTRY

GDEVFIAAMWKDNVVASQFHPEKSGAAGLQYIDAFLNDKTSPFDTSVYSEQEKQEKSNDY

INFGLARRIIACLDVRSNDQGDLVVTKGDQYDVREKSDDGGDVRNLGKPVSLAQQYYEQG

ADEVTFLNITSFRNCPLKDLPMLDVLSKAAETCFVPLTVGGGIKDVVDVDGTIVRADEVA

SLYFRSGADKVSIGTDAVYAAEDYYKNGNKGNGQSPIETISKKFGAQAVVISVDPKRVYV

NSPDDVKHNTIKTEVKGPNNEEYCWYQCTIKGGRESRDIGVYEFVQACEALGAGEILLNC

IDKDGSNSGYDFELINMCKSAVSIPVIASSGAGVPQHFEDAFKYTSCDACLGAGMFHRNE

YTVKEVKEHLVHSGFKARMDY

>34-9_0409

MFLQDIKISSIVAGGMHSLALTYDNDIYSWGTNDMGVLGRDTVTNAKVSLKEADAQAESD

DDDDGDLNDLESTPAKVENLPKDNGNIVQIGATDNLSIVLYENGDVYAWGTFRNNEGILG

FYKKEIKIQNTPFKIPLNFLKNKKAKIVQIATGKDHVLLLDSFGIVYAWGTYQQGQLARK

IMDQHALANLFPQAVVFKSRSKELIKSIFSGENHSFAISHSGTLYSWGLNQFGQCGTDKQ

LQDGGLIYAPKKVLLPADVKDASEIKLVAAGEHHSLVLLNNGKLLAFGRRDMFEVGLTPE

QIPEENAYLDPNSKKIRSVPVPTYIPSEKKFNNVAAGSHHSLATTDNGLLYSWGFGETYA

LGLGPAGEDVEIPTKVMNTAVKEMKMTFVSAGGQFSLAAGVDLTEEEIEKREDEEE

>34-9_0410

MGRISNRKKIVKHDPLVESNVVSKKQQYRHGNKQENSDDEDVEKETFINSKQTKKILDMA

KKQQEEVYFEETGKHMNEDKNLRIVEEDEDEEEELSEFEEDYLEYNDDIEDFANDFAEVD

PEEARILEEYLEKSTKAKVAPGLTLADKILNSIKAKEMEQAVPATNNDNLHDFSAAPSRD

EGVALPPKIIKAYSIIQQILTTWTHGKLPKLFKILPSLRNWQDILYVLQPETWTPHVIYE

ATKLFVSNLNPKEAAKFLNIVLLPRFMTEVAEREKHDVSYHTYRAMRKSLYKPAAFFKGF

LFPLLEQESCNSREALLVGSVLAKVSVPVLHSSAALSWLVSQERDMSNANMVFIRVLLEK

RYALPYQVIDECVFYFMRFRNVLDGQEVDEKTEILPLVWHKAFLSFATRYKNDITQDQRD

FLLEVLRQRGHKDMAKEIRKELLAGVSREFESTISYNQGDIELA

>34-9_0411

MTSLKTFSDPFIELPLKVDFDLESDNKNELENLLINKTYNELKKIKEQSIKKTLIFKLLS

KHNLKSDVLKNITRDPELLFGYGSIVEFQNKYSLLEYKLFHKINSDNDKKSKLILEDTIE

DNDGWEIDEDLDLDLNDAPIAEEPEEVAVFDNHVEDVDPEKSELYALKKRLFTKNIADEN

KNLTAEQENEIEERNHEDIIKELESLTHSLKNSSVAFQTQLNNTDKDILEKTEANLLSNG

ERLQLLGKKLGTFSKSKLGIFFYLVSILVMILGLFITYLIIKIFPEM

>34-9_0412

MKPFITGHEDLVHDVKYDYYGRQLATCSSDLTIKIFVLNPSANEWEISETFKAHDSNIVS

LDWCAPEFGRLIISASYDGFVKIWEESSSGKQTSNLLASNKQAPAKKKGNWQLIAKLSDS

KAPVFNCKFAPSFLGLKIGIIGHDSHLRIYSCSDASHASTWELKESYNMIPEGFTQPVAH

LQSDYCLDWCKSKFLDETIVCSCLDLLSFMQRDPSNGNALKKIVDLYDHGGLIREVSWAP

SMGRSYELVATACQDGFVRIFKITGINSLGNTEYEEEEKKENINEMQKHYTHSHSPNFNV

ELVSKNDDHNAEVWSVAWNLTGTILSSSGDDGKIRLWKQSHMGDYKCVSVVTPDE

>34-9_0413

MSDLEHETPALGIKTKYPPCCLQLNANATTVLVGTYDLIKETGKRYGTIERYDLIQSEDQ

PPNLELKEEVLNISDDVEVRMPAILDLKIIRQFEEDDVISFVTCHSTGCLNFWIYNKFDN

KIFHKNNIQVTDDDSILLTSLHIKNTTQDTSTLKILCTATDGSAALVTINNGNGEESIEF

IQDNHGLECWTAEFGDGNCFDQCFFTGGDDATIKLFDTRVGLDSAIWSNSRVHDAGIVSL

KTNASSNSGKNGKTYSSGNENSILSGSYDDTLQVNDLRMIGSQIYPGLVKPILKESKNLG

GGVWRLIDTEESDELLVCCMYNGAKIVKVDYDTEPVFTEKNYVKEGHDSMCYGGDYRNKT

AATCSFYDNSLQVWKK

>34-9_0414

MAYLVQSGYDAKVPNAESIYDFSNDWFKPLNNSGDVIVKVEAEDENSIPEDNEINLSILE

HSLANYDSYDTCFLPSNFKLRLYAYFFLISLIQNVVVFIYIVAGYNLGRKIYHIVEAEQE

FNTITARSSKLDSNGKFQFHENFSWDIKAPIVAVLPKIALGSSALCAIIKVASNKARGST

FFTKDLIKNVKKQISLKLLSFVIFVYGMLLAVVALFSITYFYKTLCLFWNAIVHDSSYLT

GQKSFLIFIYRMFLEPEILLWFVPLFFVILCYTIYTIHSTTGIDGVDNRLPSEMWNTFFV

NTLCRDFKVWFNFWGKPFMYQMTMVIVYSTIHSIYYNDEISLLKVRSLQDLNYSLYYMQL

YLLLDGRRPLSILYKLTFLGPIATKVVKLIFSVLKTKWNKWKLITLENKLGEEDIVILND

E

>34-9_0415

MSDLITKFNSLNIKSTITLNDEQKALSSQWEELYKTNTVINHIPELNLALRDSVYLIPNA

TEVSELDVKVAEQALPVIKSLVTSSKDLAAVIGAHRHIIRFIRFVLLKLNATDDVISDLN

LNVALTKEIIEKKKKAAPAEDKAEAPAAAKADKKKDDKPKGKPDEETLKKLREEAKAKKA

AKKEQKAKEQPKEQTAAKPSVSAIDFRVGFIEKCIPHENADSLYVSTIQCGDEEGPRTVC

SGLVKHFPLEAMQKRYVVTVCNLKPVNMRGIKSCAMVLCANNADDGKVEFVNPPAGSQPG

DKVFFEGYGEEEPLAVLNPKKKIWEAFQPNFSSNENKEIIFKDEDGVKKLVNKKGEVFTV

ESLVNASVPGNGRIGKHRKHPGGRGMAGGQHHHRINLDKYHPGYFGKVGMRYFHKQQNHF

WKPVLNLDKLWTLIPEEKRDDYLKNSSSTSAPVIDTLAAGYGKVLGKGKLPQVPVIVKAR

FVSKLAEEKIRAAGGVVELIA

>34-9_0416

MSTTNKWLPQDHVSKALKEVFIENAAREMTPTRQMLYVFHIAEALKYQRRTGWVDRGIED

CESISDHLFRITFMSQFLKTPGLDISKCFSIALAHDVAEAIVGDITPTDVKIDKTEKSYR

EKITIEYITDLIKPYNEKAALKLKEDWNAYEYQTCLEAKYVKDLDKYEMLVQAMEYEKRH

PGVDLEEFWSALEVIKTDEVKGWAKELIQEREDNKAQ

>34-9_0417

MSLEVEDYSVYYPLLVASGLSFIGYKITSAIIPKVKQSFIKIGLFGKDLSKVGKPVIPET

IGVIPSIILVLLLFINIPLLFIKNQETSILFPHSKFSEFLSSILCLESILILGLLDDLFD

LKWRHKFFLPMFACIPVLVVYYVDYGITTIMVPPFLQNENLIKLIPFIGNTYQNGTIDLK

IFYYFYMGCIAIFCPNSINIFAGVNGLEVGQILVLSSIFLINDALYLFTSDLQTTKHAHI

FSSTLIIQLFGCSLALYKYNKFPAQVFVGDTFCYFAGMVFGVCGILGHFSKTVLLFFIPQ

IFNFAYSMPQLFGLVPCPRHRMPKFNEKDGLLYTSRAILDGKTPYDRENKVKKVKPLVIV

VLKLLSKVNLIDLEYDKKTGELLTCSNMTLINLVLVWSGPLREDKLCQRLLLLQFAIGIC

AIVLRHLGGALIYGHDNLFKVY

>34-9_0418

MVELDVYFNKIKALKDNLPENFDSTDHYLIEKSQLKSSGNSQQTLDFNSTLKRFIKLLEI

TPLFKRFLVEKVAQEKTSKKINANHSEHFLFTKVYEYIEKKYPNLLIEDGTTKNSDSKGT

AHRNKLSEKEEDKLLINDEDNEIEDEKTETLEKEPVLQFSESPIYVNGELRPYQIQGLNW

MIELFNNNLSGILADEMGLGKTLQTVSFLGYLRYFKQKKGPFLIITPKSTLNNWKREFNM

WTPEVNCFVLQGDKQERSDIVNQKLLTKDFDVVVASYETVIIEKMQFKKFDWEYVVIDEA

HRIKNEQSLLSQVIREIKSKNRMLITGTPLQNNLHELWALLNFLLPDIFTSAETFDEWFG

KPKADEEKAESEEKDSDLVVKQLHSILTPFLLRRVKQDVEHSLKPKVELTVYTGLSAMQK

KWYRQILEKDIDSINGNNKGSESKTQLLNIMMQLRKCCNHPYLFDGAEPGPPFTLDQHLI

DNSAKLKVLDQLLAKFKRDGSRVLIFSQMSRVLDILEDYCYFKEYEYCRIDGSTDHEDRI

KAIDDYNSPDSKKFVFLLTTRAGGLGINLTTADVVVLFDSDWNPQADLQAMDRAHRIGQK

KQVKVFRFITEKTVEEKILERAKQKLRLDQLVIQQGRMKEAEDKKKTNSKDELLSMIQHG

AKELFEQNKNSDGSDKDDEFDLEKLLRESEQRTKELNEKYESLTLDDIQNVKTEAGSTYE

WDGQSFKKKATSYNLIDASELIKQQQLQSRRDRQTNYNVDQYYSNALNGRSTANIKPQKK

LKMEKPEYLPLHHFYPERTRKLSEKKRLYETKINEIVPTMDDCKLTYELTSEDWDDGDEE

FKKKSLNLLKESIKKAKKLTERESSELDELRAQGFTNWTKLEFRKLITALQRHGRQNMDA

VASELKQTKTLEEVEKYFDVFWANIHLVDNYEKIVSNIELEEQKIKKLYKQQEALRIKVS

AYHDPLKDLVIKYPSSASLSKRTFSEEQDKFLVVMLLKYGVFDGKAYDKIKQDINKSPMF

FTDFVFKNKHSVDIQRRCGTLLQYIEKENLNIQKDKAFKSKLKEDEELYGAIEPSKKKVK

KTIIKE

>34-9_0419

MSGFYDLTCKDGKGEDFSFENFKGKVVLIFNSASKCGFTKQLDGLEELNKKYKDQGLVVV

GFPCNQFAGQEPGTNEDIVTFCKLTYGVSFPMMDKIDVNGDNESPVYKYLKSQKSGLLGL

KRIKWNFEKFIINKEGKVVNRYASTTSPESLEKEIETLLK

>34-9_0420

MSNKVQQVATKSLMAKYLNKDTLKYMLTTHFWGPVSNFGIPIAAIYDLNKDEALISGSMT

SALVVYSAVFMKYALAVTPKNYLLFGCHVINTVAQTGQGIRYINYHYLGGKEKADEAEPA

K

>34-9_0421

MNNDTSFQYPKYTRKLFVQDDETKQVESENKTTNDVNIKSESPNNTPPLNQNSIANQLYQ

QNPPQIPANQMSNQYYSYQNIHYTPPNNMGYINNPNNVPNQMYMYQQPMSYMQQMPQQVY

QPYPMVPTQIQPPNYVELMKAMHQPQSQYMNQQVLMNGGSYNQNMLNVQKSQQVDISAKL

TKRGASLDDYEDAEESSDIPYKNKKQEHHENNKTKKAKVRDSQTKHKPVISYEDTYFTPK

PKSTGKVAKVSAPKVKSNSFADKYKKLIGKKENVEALESKSSDSDSAPEEIKIEKKVTLL

PVVEKLNESVTTLPVIDKVETVLEPADKEESSSDDSENDVDEEQKTQFIQGTGISLESEE

DIKKWQEQRRINWMLKVSNKKNLHNEILKNPENFKDSEDSEIQTFLKFQNDHNITADAQE

TQKNTFKEFGKIKETVRKMFQQIDKDNNNSSVTNIASMKLIKMEEEKDNLKILPFIKALG

DMNKLDYTLTDNEKEKLFGPKK

>34-9_0422

MIYELKQILLPFENVYQNVIVDSSEGSKGLPTLVNTLILSPNHRTFAQDLIKVLNDSVEV

DSDLIISHLINIAQLFNPQQNVNAAFLRQTLNDFINQKSNLDPIFNGTFKTISDVNTSSV

SGIELSIFQLYQLNVMHMWVTDDENISTKSLKDTQLIIMNGFGLNREARKDPSVLEIGNN

RQLVEEFNQVKSFLASTANQATDLGLNLAKENLRFEDVVVFYRNENFQTLLKARDDTLYI

LAPILESDLNWRSMKSVNGTGDEFLSTITNESNVAALNELSDEEFARQLQMQEDERYAQQ

ISGNRNRSPRDSNKPADSKQYIKKKQKQVKKNMKESKLKKKKKACIVM

>34-9_0423

MFNSINSFLKQKDSDIFPVGVSYVPALIPKDTIVYHGGNGIPDGFEWVAMDVEFSMNFAA

RSSAVRRGLAQVEYQKHLKLKPKDEEKFGPRKEIDFCSISEDHQPFDIKKTRPGKKNEPP

TLMSFRLKHDLDKLIYLDGASASKATKTGEMDTQVLLYNQIKKHFPGFKDSPEKFMVERD

YAEKICKWGKPLGIQGYIRLELGFEFVLCDFHEHLEMVSNVTITPTNKFFELPEPVEISK

AAGWPINCTDGNLINADLTAEQKQILDLENLRYKQLLSLDTMDGWEWLKAGHKHNQGEKK

IGLDYRYLVSAINKTELDTDNYKWRIIKDDKGSEKLEEDIFAELSQYYSNIKQFKPTEGT

NWQLKTEGIVQKFTPFLKSIEQTLNNVEMDVASKALSINQILNPLIRRYSDNYPMKYERD

EIINTGVWEYTYPTHELKTDADNLIFSSLVTVTKEIFVHLIDLHDLALEVSNKFLQNGKI

NKTDNYDLLFQKRQESTTELIKTLNWINFGYECSLKCGIGEICFTPSWGPSPLGWISIIE

RDNKYSQKQGKLPIGFVKDEVNGFTRLDNEQVCLGLDYLIDDRIEFDF

>34-9_0424

MSYEKELEVVNKIQNDLPSNQLDKVRHIRNIGVSAHIDSGKTTFTERVLFYTGRINAIHE

VRGKDGHGATMDSMDLEREKGITIQSAATYCDWNHTVTKKDYHFNLIDTPGHIDFTIEVE

RALRVLDGAVLVVCAASGVQSQTITVDRQMKRYNVPRVTFINKMDRLGANPYKVINDLET

KLKLPCAAIQVAIGAEDSFKGAVDVITKKAVYYDGENGENIRIEDEIPADLVELVEERRA

LLIEKLADVDDEMANIYLEEAEPTIEQINGAIRRATIARKFSPVLVGSALANKGVQKVLD

AVVDYLPNPAEVLNKALDITEGNEKEKEIILDCNTNSQVVALAFKLEEGRYGQLTYMRVY

QGKLVKGATIIHTKTGKRIKISRLARMHSNEMEEVNEIGAGEICALFGIDCASGDTFTDN

STDMKISMNSMFVPEAVVTKSIKPVNIDQLTNFSKALQKFNREDPTFRIRYDEESKETII

SGMGELHLEIYVERLKREFNVSCVVGKPQVSYRETLTAPIDFDYTHKRQSGGAGQYGRVI

GAFELLEPVDGSDYVPMKVDFETNVVGGKISEKHLVACGRGFEDSCNRGPLIGAKVIGVK

MTIDDGASHAVDSSELAFSLAAQGAFKQAFTQGQPQILEPYMKLEVTSPVEFQSSILGLL

NKNLAIIKETENKEDEVTIFADCSLEKLFGFSTSLRACTQGKGSFSMEFSHYMPVLPSDQ

VTIIERFNPDMAKNKKGVADFSYEVNNDEEDERKVNSNPKLSYKEQKQLNIEKLKEQKRE

RRAKKKEAALAKFKNSKENRDEHLKRIAEIKEKNIQRKKRNEKLNVKTRRGQPVMSFKID

DLLSKIQSQQE

>34-9_0425

MIDQQSYSNMNEPKNNKQALPPIQSLLCSYPKDDALEIACCDKQTQKKKKSRCNLPKHVT

NILNEWLSDHLDNPYPTASEKKELIEKTNLSSVQLSNWFINVRRRKLFSQYYDLKDKPIS

RSSSITKQRNTEDKDKQLELEYMHTKKRMSLQNILNVCSPEDESCTTENTNDCKKNNYTS

RNKSTSSPRIIMEADEIKLEEKFNFQPFTKRKKLMDRLYDLKKIYYVNDGDCTGEQP

>34-9_0426

MTTIAEFEEVHYNHTAKRLDAPSSYLIRKAKLENRKVQYEKKNMMRSMADALFSENKEPE

ENVDEAGVEMLTNAMTSKTIYVGNISEYTKEEQIYELFSEVGSVQTVIMGLDRLKFTPCG

FAFVIFDDIESTLNAVKFTKNAILDGKRITVDLDSGFENGREFGRGFAGGQRTRADYNRM

KQFVAESKRYGARSNVYIPNKNKKSNEDTEVNQEQQ

>34-9_0427

MGNSPSRPNEYNNNNNVGQSAHTNEDDLSGKGNGLDDPDDSRRHQTNIKTKNITKTDKAG

MNKQFPLKKPEVIDEVFDFESDTDSSKNALNSRSTSRRNSSTKSFSMEKTKKTESYSTTN

SKSTTYSSSYTSSNVNIGDGSFISEEVNFEKVKSLDEIKNDTADYKLNFTTLQETKNPAI

KTKITEKKTLTSKNKDAHDSLLKPPVLHVPTPLASLATSSSVSSGSTNLNELPNNKPSIN

RESNNLISSKHVNGFKGSKHTNKVLNVDDCIERLRHLADKNIYQKKFPFEKWEIELICMK

AREVFLSQPSLLKLKSPTKVVGDVHGQFNDLLRILKLSGDPSETGYLFLGDYVDRGKQSL

ETITLLLLYKIKYSQTFFMLRGNHESSQVTKIYGFYDECKRRCGTSKVWKYYVDCFNSLP

FAGIISDKIFCVHGGISPYLQDMSQIEKILRPTEVPQDGLLTDLLWSDPEKNIVDWQPNS

DRGVSCYFSKKNVSTFCKRFQFDLIIRGHMVVEDGYEFFGKKRLVTIFSAPNYCGEFNNW

GAVMDVDKDLICSFELLKPRKNEKKLK

>34-9_0428

MSNKNLKISDFEIGKTLGKGKLGRVYCCQHKSSGYIVAIKVMNIKELKDFKLTNQLKREI

SIQGKLQHKSIIKLYNWFYDKKRIYLIMEYCYNGELFKTLQKTKNGILPEFKVRKYISDL

LMSFKYLQDNHILHRDLKPENILLDEYGDIKLTDFGWATQIDKSSNSRDTGKRTTLCGTL

DYLSPEIIKNQEYDHKVDNWALGVLTFELLFGYAPFEHSTKEKTYKMICDGAINWPTDNY

KQNNVSSLAKDFIGRLLVVDVNKRMSIKEAIAHSWFKTASSPTA

>34-9_0429

MTFDPTEETINVPYEKHEALESLLKKYTDSKLDCQNIIPGIVAGYANNEKILDLAAAGVL

DHGAKDRDAPENKMQTESTFLLWSTTKLLTLTGLFQLVEEGKISLEDPIEKHIPGFKNKK

VATEVNESGVVTKSEEMNIVPQIKHLTTHTCGFSYPFFNENYQKFMDVANIGGDLLKADK

EQFLNLPLVFQPGSKWQYGFNIDVIGLIIEEITGSTLNDYLKKNIFEKCNMSSFSFVRDE

SHVKSWVRLHTRDASSTKTFGNITQTPEFTPLNPKTHCGGHGCFGTVKDYLKFLQCFINK

GVQPMTGQRILKEETISNAFFSDQLKNIGPNCEITSLPNSQPLITNPIDVYPATKKTWSH

ALLINEEPIDLGDGISRSKNSGMWCGLANLYYVIDLEKKVVLYWGEELFPFADMNSFVGF

TEFEKFVYTQVTN

>34-9_0430

MLKKVLQESKKDAYRENEDHIDDNEINEYASDVKEEDLDAVKSEDNVEATPDREWRRSKR

RRLASPRSEQISTFSTDGYNAEEEPQLTPNTRQARKVTSSRNQAGSIDSMLDQDSETKKH

PLNEDDEEHKEDIKKTETFEEEETASAEQKPKRTYNRRTTQNGTNSRPIEGSRRNVASKS

KSKNNISELEINEAEDLQQSHMEKLLKLAQESSKPRQTPEGITVEEMYERVTAISAFLER

SKEELMFLQKNQNVLLSFVENPEFIEEYKKNVDIDLLLKQMNSLNGDLGKFGKV

>34-9_0431

MNLEKVSEEYLNQIFNVPPKKLVKLNIKVLVLDDITTPILSTNCSQTKLLENDIYLVENL

KLLSNFSQLDNRDTMRQLRCIVYISNTDESINNLCKELKNPKYGEYQIFLNNTVTKIQLE

KLATNDHFEVVTSVINLYQDYIPLNQEFFYPSDQIKYKSLKSYLKTMSPGDPDSSMSLMF

KNSAYLEGLAESLASTVISLESDVDVFYEKVSTKLAKPLALGVNRILREAHKNSVLKNQD

KKDTCLLIVDRFSFDPVTPLLQPWTYQSMIKEYLGLDRYVVDMSRRDVDILKDDEENKTK

DPVIYKQQQQLLLNPKEDTFFAKTMYNNYGDLNDTFQTYLADYKFKMKNKKTINDLQDIK

TFILQYPEFNKLSKNVTKHMAIISELDSDLKKLNIWSVSEVEQNVVSQIHSDADYNEFLP

DIVKIFDDEKIDFHYKIKLLLVFGLSNYKYLPKLLQAIEMKLPSKAIEFAKTISKQHQLQ

SSKLNSTDKQKLQSQTEVSTDFLGGLAKQFNANKNVDTGVPHSRNKDNIYMMHHGALEIL

IYKSDFLKNFQSVDFEDRALDTALVKDLSEKYKNIIFYVVGGITYEECRVVREFNSDFTY

NSKGKKVLLGGTTFLSTQEYMNDLESLNEIKKRKASFSFVELYI

>34-9_0432

MKFTTAIASVAAAASFASAAAIDQEQLTNGTYIDIPQESILSFLDLTDSPEVSVYPIKEG

SKTGLIFVNSTIVDQAYSETTALTRKREAVAEADPWAWIRMWPGGVMFTKRDAEAEAEAE

ADPWAWIRMWPGGVMFTKRDAEAEADAEPWAWIRMWPGGVMFT

>34-9_0433

MLRRSLQLNVTRRSLRFQSNTVKPLASIDLKTFSDLYNPKLTTSFDPLDTFTKRHIGPNP

SDVASMLKQLKQTSLETFIKQVVPKDVLVQRPLDFKILNDLNRGNVSQGLSESEMLDNLK

NIAKLNKFEYRNFIGKGYYNTNLPLVIQRNLLESPEWYTSYTPYQAEISQGRLESLLNFQ

TLVTDLTGLEIANASLLDEGTAAGEALLVAYHLMKGKKKTFILDKNLHLQTKNVIKSRGD

PFNVQFVEIDFLKLEKDNVDLKTVLTDDVLTNSFGCMIQYPGSNGNVLNPSTLSSISDLL

HQKKNLLITATDLLALTLLPPPSTFDSDICLGSSQRFGFPTGFGGPHAAFFSCKKTLSRK

IPGRIVGVSKGRLGERALRLALQTREQHIKRDKATSNICTAQCLGANVAAMYAVYHGPKG

LQDIAKRVYAMTAVLAEHIKNYGSCFEIITGDNFFDTLTIKLRKDNKVFASIDEVIELAK

EKHSINLYKVDNDTLSLSLDETVTLEEVVALSKIFGVEVPINVKELSQLPYPTIKNERPL

DFLKQPVFNENQSETSMLRYLHKLQSKDVSLANSMIPLGSCTMKLNPTTAMVPITWPEFT

NIHPFQPRDQVKGYEKLIKETSQYIATVTGLPGVSLQPHSGSAGEYTGLRVIRQFLNDSD

KTNARKICLIPVSAHGTNPASAAMCDFKVVPVKCLDDGSLDLVDLKLKAEKHAHELACTM

ITYPSTYGIFEGGIKEAIDIVHKNGGLVYMDGANMNAQVGLTSPGDLGADVVHLNLHKTF

AICHAGGGPGVGPIAVTDALTPYLPGHEITGLVHLEETGNEIKSVSSTAYGNAPLLAISY

AYMKMLGEKGLPYASIMAILNANYMKSRLLEHYPILFLKDDTTHCAHEFIIDLREFEKKG

IKAIDVSKRLQDYGFHSPTLAFPVPGTLMVEPTESESLKEIDRFCDAMISIRKEIDWFLA

GDERGQVLPNAPHTLHDLLLNSSKYSDEYKKIAAFPMKDQQLNAEFWKQWPTVTEVK

>34-9_0434

MDILHCNDEYNLVAKCNYNLVNNTENIFFQKLKKNYSNNSIQSIDFEDYSINYNNISYTL

PITNIKFLPKSFYRTDDTNRLLMSSSTTLDVLHFNEDTKSLNNILNLTFSKTNNTNKFLL

PPITSFDFNLNNCTDVIQSCIDTTCSVYDLSKQKIKSQLIAHDDLVLDVKYLNNNNVKKN

AELFMTSGKDGSLRLFDLRVLDHSSILYEDSQKNPLTNIEINPLDSNKILCFSTNDQITF

IDLRYEKNPIKKYNLHSKISSCLWMDNGLDFLVGDQNNMITHWNINSLKTSHFPDNFFSD

NKNNGIYKMKYFKEDNLIYYMDKDNKIEVVPFTS

>34-9_0435

MSSIIHKTRVPQHLIPFKVGTVLSSNPLTKTIIVSVEKQHTFQQIGKTIIKHISMAAHDE

NLKAKVGDIVRVGNFKKTIDPQNYKKKYTLMEILDTKAERNAELLSRQREKIAKFEEEKL

EELNKDRLKRNKEIETNNYRDVALFKDVYLLNSNNQVSNEVLEEIAQRHEVTYNNDTQKS

LLVKLLDFERYNTLNNKLENAERHFFIMKAIKNIHTNKLPKLVEYDTFIVKSFDEQKQTL

HELTKSMTLDELKIQLE

>34-9_0436

MAFSLNMNLVKEYMFRPTALASGLPIFLTILSIINFESPLETEFISILKSLSTWNFKVGN

YKFIKLLIVFLLACNYERQKGTIRFGVILNLISVFSGLPYILIGKLFGLNMEIQGLNYWN

VILWLGLDIKNLQMFKKIDTLQKTIIIKVIGLVSLLLLGFDEFNWLIALTTTTVGFVYYF

MPVIFDLKLNLKSDLFLKIETFKYWTHSANPYENTNLYSLLKIIHIDYLMEREIIEKQHI

GGELENQSSLEHNLNFDEEELLKTEQVPEFKSKLDELQHLEEFEVDTFALPPLNFNETMK

PRSRKGTLTKPASFTNSPKRSRKNTIKTELENARAMSNSPVKGRSRSNTIGKTE

>34-9_0437

MNRNLIFYTVTIYLNFIVWGIIQERLFDSNILIKFNNVIILLTNIISLFFIKPSNADISQ

LYKLIKQKQNIKYFIILVITQTMTQPLNYYITSNYGIDYLFLQLSKSCKMIPVIIIHKFI

YKRNIDNTKIIISLLITLSIVIFNYKPNSSVNLSGFLLLLPLTMEGFTNTSQDQLFKKNK

LNSKVLLIFNNIINIIIHGGYLIMFDNKQFINFAFKYNWSLNIIIEVVIYMILQIIGTLL

IFKVLYEFDSLILLRITVLRKVSSLLISILFLNSNKKFNQYEKVGVAGVITTLLLEFYLK

NKSNAFNVAALQLGSSKEGTDATIEKIVSYKDEIISKNIKLCVLPEALLGGYPKGNNFGA

WLGYRALQGKKDYAEYHKAAVFVCKENPQIIKLEKLAREADCTLVVGCIEKVPENSSCYC

TMIFIDAKLGYVAKHRKTMPTGSERLIWAQGDGSTLSTHTFKNENQDVKIGGAICWENLC

PLLRFHMYTKNLSIWCAPTVDAREIWRSLMKTIAYEGRLFLISSCQYVPSATEMGYGMEH

PDPVLAAEGKRILPQWDGGHEDEMPCINGGSVIVNPYGDIIAGPILGKEGLIFAEVDLDL

ILQSRYDFDPTGHYHRSDIFQLTVNEK

>34-9_0438

MSEEIVVINPKTEQPLVTKTTHTNGLIETNYTKFFENFEFTCIHEKHTNPVFNKCLSAAF

ENLGLTDQKQFTRTINATKQNVVACNLSCKAKDNIGDVCNLKINIRKDKNTGKWHVFLNN

LTNFICNCGSTPESINANLNSKDLDEKQKSIINNLSSNINNGVDYYKTLKKFKLNQPGTK

TFSELREIIVEELKSQLGIKKNFKIQIRRTGGSKESDYAFLDCFLNDKKDSLCKFKASFK

RDKSNNEWYLDDFSSRNKFLCTCKHVSDKNDEFGYIVNSKVLKGNAEEEEQLEEKRKQIE

NDNTNILDKKKKLKVKSVVKKPRVTKPKKKLEKEVIVQSNSPLDNNTEEGIVAETNETKK

EPETVENILNAAIEAVESVVHNIDQKHSVNENSNIFEQENLKEIEQDKVDEIDESSNTQE

IVEDENTIERDLKRKNDDEVEEQNKKKQKK

>34-9_0439

MILKVSLLYPIISLLACISFGHGNTHSSANDDDDYKHLNTIYSLPDLLANNKLANNKDKG

LPNYEFQNTELLDGRIVLNYNELGGGMLSKSGKHFKQSDSFAIEYTIRNLANLADKPKDN

SLFDSTDNFAFFLADDLNKIDDLIINEGNINKLVFNGILLYLDAKDKYGGELKFIMNDGA

SKYSTMNDLYQNSIGSCLINYYEDSSIPITIRLNYDASQHLLSVQVDNKICLQTRSVNLN

SFDNGKIYKIATTSSGLTKSNSKFEVLKLNFFDSLIQDSYLPNIKSMPQPKILNKIVNKD

TGDVVYQEIDKLSDSDINLLKLYKKLNTIEGKVIANDNVDLFKKVERLEDLSIKQFKQIE

KLLSVIEVLASSSTEGSNILDNSEFKDIVGVNEKLEKLLAEQQEWKSSHQFNSKSNSNSD

SKVHVDEIIFKLLLWLSPLGVIMLVMVYFTFSIKREIGKQKLL

>34-9_0440

MFFKLPALRAVQIVLISFLAVTLTTKIYLNNGPNIKNKNIDKNFKILKDDHNIDIDPLDR

SPTLTPSLDKLGIFDPHENGGSSPFMISSDLKTCNNIAKDMINKYPGVNYVDASITIGLC

LGMINFFNSGVGGGGYATLYDFEKQQSKFYDFRETAPAKWDNDLYNLTKYGGLSIATPGE

LKGFATLFENHGSGKITFKQILEPIVNLGRNGFVVEEVLGATLEKYVEILEALNKENPNF

NLSDWEFIYSDFENGKALKQGEVMYRKKFADTLEYIGEHGVDKAFYSKDSPLVKRMVNFI

NNNDGILSIEDFEKYEVHEGKTLEYNLGTNFYGKKVITSSGSSSGFSLISGLKIMSTCGL

SGKKVGSDMEPKATFQLVEAMKWMGSARTRLGDYFYNSSLSINEYPKRIKDIFLNEEWTN

KACEVLLNPKMKTLPNVLDYDPKFIHSEAHGTSHYSIMDNKGNAIALTTTINLLCGSLLH

DPETGMIFNNEMDDFSSPYSEPNSFGLLSSEYNVPQPFKRPLSSMAPTIVIDELGRVEIV

IGASGGSRITTSIFQALIRLLFYEMPLLETISYPRLHHQLMPDTLEIESYKLVGNQTIEI

LKNSNGYETIESVGKAVLNGIHRTNRNVMHVVGDYWRKRCSGDIVLYRTLSTGIQDWVLK

AEAMFHLSIAFIRSFAPEDEDINKSNFPISLLHVLNDNKYPPEPSTNKNMLQIPVISLQR

HYNNYDETDTTQKQKLKPWSLIENINELFKSDDFTILENDSNQIDPINNNKILKLSRQQK

ALIKSIFSEKSTIETISKKMSKQSKKIMKEFTTRHSSFQAGWNTNPVPSQKVKANGFKNS

PDDYLTWTTCYIDDFYIWTWMSSLSNEESPRQRNIFGRSLILEFEFDAFKKWVIFEEVSI

VKEQSVPKISPRSELRSISGIENKLPLEDTNQSLFVDNSKERKVSRDNVPTLQKVEHLED

SSDHDTSNASSNYNNKTFLNGYMTNGDITNVSAVSKQESENFVLPAVDESAFNIDLPDID

PSQITIDGSDDVTYKNRYVVNSNDSITDAIRGLGKELNNVEKQIKPNNLDPFSQSNGSTI

KSLQFGNSSSNISNRDTHNEYVQPVYSDPTTYVPERSPERHRQLSSDVEKQYFNEPTKQS

HSKQNSLTPVNVNNYTQQQEGQVKQSSTVQQGYQYQGENSRPRVPAEQNMYNKVEDHRPI

AQPQSPVKQHQSQQYQTQQSPVKQQYQQVPIQQSPVKQQYQQVPIQQVPSQQPAIQQHNA

YLLSLALRYNCMLNNKEKYKEYYKQLYINENTKNPFTDTPVKYGVITYTNDEVDILGDKK

VTPEGELLGNRKLLLKKITKFNNNKYICEADEVSKLLNITKELLKDPMNFNLQTEYYNEK

EWFYVKDCFMRFGASIIVKGIRILDDYYESRLQKNGITVGYRVHRIPDDTYAKSLNNFNI

SNKEINVEPEGYWPKADESLGLIDDSYLGSLPIQNKPLKYINKVQLTALKNNNLLNEKLY

EVWNQNVITNQNDEIKNMNIGNIWRLDNMIKQNLDGLGDQENEKIDTLYYNPDGFENKQE

DIKQMKDNILNLTTQEILELQNKHFVLKTSFEANKVRKSRKHIITKKYEYNQINCFKNKK

EKTSEISKEFYQKIMNTVYESEDDPYNQRNESKPDTLDPVENTDFWTFEEEEYDELKRLQ

TVDIEIRVPDKNVYEWFKTVNNALKK

>34-9_0441

MNKIITETKANLSSNNLQKLIFWSHGGPTFAIKDDPFGCSGAFNKVEALGSKIKNEYNPD

YIVIISAHWQPTDRNSPDAFKTVYLSDTKASNDKPQENELIYDFYGFPDEMYSWKFKNTF

NKQLINRITKLSDDTLIKIKPKVRGLDHGLWVPGKVAQLDKPGNNTDELIPVVQLSMLPD

VPALSRGSVDIRETLNTHYNLGQLVNKIRADNGAVILSGMSVHNLRDLQSIFLGKKLPYA

KQFDDYLRDLIIKDKQEKINKDYDTNSLYNGLMKLGEDKKLTSLLLQAHSPGIDHFLPFV

IGCGALTNDEYFKEQYCEETGSLGWGIYTSEKVPQK

>34-9_0442

MFALKNNKHLIRNVSTFKRSTRYLSTNFKQTANSRVKRNYLLLSTALLVSGYGVYNSNNN

KIVHNEPSKKNLPTISLEEFVKHSTPEDCWVIINGIVYDLTNFISQHPGGPEIIKANCGH

DVSKLFNPLHAKDMIDKYIDKKDIKGVIDPNCDFPDKLVTKYDFPGETRESISLKNKLKN

NLPDIDDIKTIFDFEYICSKILPKKTWFYYSSGAEDEFSLRENHNVYHRVQFRPKILVDV

SEVSTECEMLGKKRDVPFFVCATALAALGNESGEIGIAEGCAKGEFTVPQMISTLSSKSL

GEITAARKNNQDQWFQLYVNTDRSRALDLIKEAEKTGCSAIFVTVDAPSLGNREKDVKLP

GKSSKEDGVSKGLSTFIDPSLDWNDVKYLKTKTNLPMFIKGIQRKEDALTAAKDGFNILL

SNHGGRQLDHTVPPLQTLSEVVPLLKENGFKPGKDLEVFIDGGVRRGNDVLKAIALGATG

VGLGRPILYSNSAYGAEGVNKCKEIMRAEIAMNMRLLGVTKLDQLDESFVDIEGMKMKNV

RLNQDFLGEKVYEPREVIKWDKYDELTSEN

>34-9_0443

MFGSKFIRSFKALQVTAPATRNYAKVFQRTKPHVNIGTIGHVDHGKTTLTAAITKHLSKK

GGADFLDYSSIDKAPEERARGITISTAHVEYETDARHYSHVDCPGHADYIKNMITGAATM

DGAVIVVAATDGHKPQTREHLLLARQVGIQKLVVFINKVDQIDDPEMLELAEMDMRDILS

HYGFDGENTPVIFGSALAALEDKTPEIGAERIEKLMEAIDSYIPTPVRDLSKPFLLPIEG

THSISGRGTVITGRVERGEIKKGDAVEIIGGKEVVSATITGVEMFRKELEKAIAGDNAGL

LIRGIRRDQVSRGMLAVKPGSVKTHTKFLTSLYVLTKDEGGRHTGFNANFKPQIFIRTAD

VSSTLTFPEGADPNVTVMPGDNQEMIVTLEKPTGIEIGQRFNLREGGKTIGTGLITRIIE

>34-9_0444

MNKQEPKNTLKKSSRTVKKGIKVRPVFKIPPPVFQGIKNHFHCLYCKSSYKGTPKIIANH

VSGHSHKQNVKVYYTNKFLALYDRSKLKKQGQFKLANDINKFENTLLNNRKLGFQFINQP

GNDEEIGSDMFSKLKFLAHRNYQNGIPDFILKNRAIEKNRKRLMVKNKLNERKKNKNVIT

NKDRQQFYEAKKLLKQLNKQNSHKKYLIKRSRRKLLKRLKSHTDENLNLNYNFGGNRRVK

YNHDVKDNSLQVLNHIYKKSPNFNRVFLNASENDLDIMKIREEYISAFNENLKQRKLRSL

KNKIAKVRYRIAVKRKYGKRKYNPKSKSNQPKSVSTERLFLDKFNKDFSEENLLKHLKLK

LKHSSNITEKRRMKFKARSNKYKLFKEDIKAEEFVKKIQKEDNKYTNSLLLPPRTLNFNN

NTLTKHQVNFDDIITFPKLSIKSKQKMGPIPSWLSDHSNNKLLNRRLMYPKVSDLNDKIP

ENLLNHLHKSKRLKQLIKQSLYKEQHIKSMFKYQAILWRNFKRRNSIYKLNNGLVNNKKV

VKLIQAKLLGKKWFNKVYGDREGKKLKLMKLSRYKAIKDKKLKYKKTTTSKGGSKNLVLK

NGKKIKVDKTSPKQGLELDTKIKVIKPIRKVVAPVKKPTSGAKSHVHKQNLSVSDRINNN

LSTSSQKKVFKSYSRFEGSGSLPKPSQIVSRFENHQPTQAVLNRSIRATMETAKPLQASR

FEPNNYVRPSLNSSVEPNKSARPISNSSVGSSKSVTPIPHSRFENNRFSASPPTTSPAAA

KNPVVYQKPVIKTSRFEGSNQKFQPKRFIDKQSTASRYAGKKPSRFQG

>34-9_0445

MFTSVFKTSSKQLFHTSAKRQNIITDLYISQLKNVKVATTFTGPAPVKEFKPDAIIAALQ

NASTKDTIIANADTLREYNQLEPTFSTTVQDTNDWYFKNELDPAKQGDWLNLQGGIDVLK

ETPGLGEKWVEENKAKYEKWE

>34-9_0446

MGLIKLKLKAGDKKITKESSKLFETPKEKLSLGTLNTANMPMKTNVEAVNTDFETYLTNI

ESIGSKRDSESLEISDQIFQKYHEDEDDQMKKKIKKQDDDHKKKPNRNKKKVIKSEKANI

ATSINHNDDYDAMSETELLKLKIEKQVTIEITSNGSSITKIPKINNKWIDFGIPPQIYKN

FQANLKFQKPTTVQALAYPLITKTTGFNLIAISPTGTGKTLAYVLPIIKKMFSHSEKGMI

SLIIAPTRELTKQVHNQFSHYLGDIDMISLVMTNPGEDISSEISSIKKIMNENNKNKKLV

VISTPGRLIDLLTVNNGNLLDYSMIGDIVMDEFDRLLDLGFAPQIKNILNAVKGNQLCNK

MMFSATMPVFLEAFANKMLTESTKTLVKFTIGGVNKCRENILQQVEVVTDKIEKYNALVD

IINKYKKEPLVVFVKTQIICDQIGSDIDSKFYKDNNIYLLHAGRSVEERSQVVDEYKQNC

IEFKKEGSNSAIFKRPILFCTQLLSRGLDIPKINIIVIYDSINSESQYVHTVGRTGRHEN

QNGICITILNENEVESAQVLYRQIIKYPKEKSFKQMPDKTKKIIETMNAEFWDGVKNGKF

KNMDQGFSGKGLNNFDGSAALNSDEEDENSIEDLSLNISNITFDNKKKKHTQSIYVDDLP

QMIKRELLKASQLERIQNKTGSIISIVGTYKKNKGVEINVSNSDKLNLLETMTVINDFFN

SLKNKFIKQQDQGFSI

>34-9_0447

MFNSVLKIRPGIQLVTISNRALVHNATIQCSKRYLSTKFLDKYKLPKQSPGGVDGTINDK

LKTPDLNFFEGGFHWGYEKVVALAIIPTSLLPFYSYMYLGNLSISPVWDSLLSILLLMHA

SYGYKACIIDYIPERKYGYWHKFCKKLLQFGGVLSLYGIYIMETENNGMTDLVRRLWRAK

SSNDEDEAKNYI

>34-9_0448

MNKRYYKLIFQITALIVCGTIVLIIVKKNDTSKQILETTIKPKANKQNLGYISDLHTISC

LILPALGEFCMNSYSEDSIIKKDLNQNRYKYKNSYKKYLQFGFSKYLIIKRLKTTDDAVL

EHILTKPPKDEDYKKLQLLNETLYLTYDHLDLSKVITSGAYKDIDVLFGESSVDSRRGYT

VLEDVVFGESRKQPMYLSVQKMAKDSFEENKKDIGTPNVISLTKKEQSKFKILQLADLHY

AVGKGKCRDPYPEDNFKETCEADVLTNTFVENILDIEKPDFVVFSGDQIMGNLCTEDSES

CLLKLVNPLVQRKINYTMIWGNHDDEGSLTREQLSKFVETLPYSQYQHTIINKNEINGYG

NNILKMQNDNDEPILKLFMLDSHKYSPKPKIYPGYDWIKEEQWDYLSKMNDANDDSINIA

FYHIPLPEYKDPSMEIVNGEYREGVTAPKYDYGTVNEYFTKNNIKLGLCGHDHVNDYCGK

IKDSKGTDRYVCFGGATGMGGYGGYGGYERRVRIINIDFKTNTIETYKRVMGNEKVKIDE

LVINK

>34-9_0449

MSNIDTQSSILTTKSSNLLNYKNGNVYNYWKYYKLYDESIIKNNFSMVSDSKSINNNMKY

VKSSRYIIYSNNKLINIFDTKSNSFAVSNINIDDFDVSKHEITHLKITETITEGISDSDI

FVTVVFEKSQSENNINSPYIIKIYNLSDILQNVNVHNTSNNTLKYHTMITVNDMSGKNKF

PVSCFEISNNLNVALLGLGNGAVYLIRGDFKRDRGYKQRLIYKNLSHQMITNLRLVNNDK

YVVMSTIDLLLILSTDGVINDKKHLEKNFYKNISNIEGANFNLLDVDEKQNLMYVLNVDI

VDVYDLANDFNKIDSFTVNISNKSVNNLYLKCINESELIIVTQTNLDDETLINETSNNRN

TVKATIIDVNNHITTLTTVLNNKVNEIIPTINGLSVILKSGTIHNFAKKDINKTVDLILN

GLNISDVDYKLAIKLLSNSPVENNEAVIKKYGDFLYNENRLDEAMEQYMKCIEYFMNYSN

IENLTSMKPGESSETFPSITDIVIKYAMDNNKENFINSKQNFESLVKFLKTLIMKSFTAK

SDYTTLLMILLIKMASWDELLDLVNDISRNGNYSSQPGKARGINNYKDYLYTIQQDEDYW

YDDKKIFDVDTITDLLIDSAEMYNGEYSGEIKDILFKLTVKFNKNPEKIVEILLNYLNKE

DFALKWIRRLDSIGFLNVIFGSSGVGRILLDLQEKTPKFDILDLYVKLFTGEYKQLFLDG

TESKQIYEPPKPSIVFSYFFQKDDLFKEFLEQVINSETIKNEQEFQKCVNALYATYQNLD

MKEEASALLSKHKNYVSKLLQKDESLATDDDEAVINNYITLNDINGLIKVTETDEKLQNI

QKYTFMRLLRHVTSKPSILNDFTEERLKNLINYILENEILDMINLINVLSETNVASFGII

KNVFIEWLSQQEVTLEQQEEKITKIVEVNNNKNPTCLVCELPLEKGFLKFACGHNVHRAC

LADDTDIMCPDCGGKLLQYEEEIKTFNSFSKEKDNEIENKMKNSTNSDTNEVFDVLLELL

GNGALEIE

>34-9_0450

MDNFNIMGSDNMTPIIEKETQFANPYATTLWKSSDENKKTVNKLKTDSRDNLKKTLSTRR

NISLKSLHLSNKVNKETVNSASKKKNIASYILEIDESEFFGLPSVSSEYSDSLQPINNFT

NDTVFVRKKRICNSAVHNKVESPISLTYLKTPLAQSDSSFKFKAVKDKTDFLSVLEIFTI

YEYSYLENLRIVKNCFHHQFSNNMEVKLKLTTTNDIYAGEVLIFGNIETMIDLNVNFLND

VKDCVSSYFNINRYDSQFLQFN

>34-9_0451

MSAEAPVTSKYADLPVTVEKNIPVTYDLGLLSVFDSNPINGDNFNSSNAKREQHIKDLTR

DNTQLLINQLLQQPIQTTTDSAKSTISLIQLPQPITEIPREKALPKPKAPTKWELFAAKK

GIQKKRKEGKLVYDEHKGEWVNKWGYNKKSDVLAEDWLVEIDDKDTKNPEGLIDPRSLKR

AERKKLIKRNELQHKKNLQNNK

>34-9_0452

MSNLNKTETPNNGSVNELINDVDLNDNDFDLFDVSDAESDKEEAPAKKVKLDKKFSAKPK

ERGIFKDGFESEQVVNYSASNDLLPQTDENKNVDEEEKVQIKHQVRHQVALPPNYDYIPI

TEHKRVNQAREYPFTLDPFQDTAISCIDRNESVLVAAHTSAGKTVVAEYAIAQTLRDKQR

IIYTSPIKALSNQKYREFLKDFGDVGLMTGDITINPDASCIVMTTEILRSMLYRGSEMMR

EVKWVVFDEVHYMRDKERGVIWEETIILLPDNCRYVFLSATIPNAKEFAEWICLIHHTPC

HIVYTDYRPTPLQHYLFPVGGNGIHLVVDEKSNFREENFQKAMKGIGSSEDSNDNQKSKS

NKGGAAKGADSKGDIFKIVKMIWQKRYNPVIVFSFSKRDCESLAMKMTSIDFNDEDEKNS

IEQIFNNAIAILPEKDRQLPQITQLLPLLKRGIGIHHSGLLPILKEVIEILFQEGFLKCL

FATETFSIGLNMPAKTVIFTGVRKWDGNSIRWVSGGEYIQMSGRAGRRGLDDRGIVIMMI

DEKMEPQVAKGMVKGQSDRLDSAFHLGYNMILNLLRVEGVSPEYMLEHSFFQFQNSESLP

ILYSKLNDAELDLKTNYVIENESDVAHLYDLNQQLTQLQQQQRLVITHPSNSAKFLNTGR

LIRVNIKGKDFGWGVVENYQKRPISRYNNKNYTDHEAFVVNVLLNSVYIDSPTTLVKPLN

PDFPDEVRPSKPGEQSIQGCILPIDLYSIQDISKVKIKMPEHLKNKEQIRIVSKSLTEVQ

KRLNNSIPLMNPVTGMKIQDDEFVNLTKKIQFLNDQIENNELQKSLLKEELYSKYVQKMQ

LKDDIKKIKDKINETVSIIQLDDLKKRKRVLRRLGFCNEDDIVQLKGRVACSISSGDELL

ITELLFDGVFNDLDPAQCAALLSCMAFQERTKTEPKLSAALSEPLAKLKSIANSVAKVMK

ESGLEIVEKEYVEQFRPNLMEVVYEWCRGAEFSHLCHLTEVYEGSLIRGFKRLEELLRQM

IDTCSIIGNDQLKEKFNEAMKLIHRDIVAAGSLYLES

>34-9_0453

MTSLSPDQVLFSKVYFLYLLLKSVPDIQVSDTQYRPISNHRTIVEIIDTKLLRKEYPVSF

KNKFFKQIKLKIYEKDIKKIESIYEEVDTIKLIDLLKNVFYDFNILDYFVFLNNLGSDKM

TENDALKGIYLRFNNYLHSVKINFAACSIIKTEKSSFVKNVHTLLKDSKNLINLTIQKDN

HFCQQVVQYVLCLFPLEFLYCKLNNEITKLEIVKNIQSNYLKNEKNIQLKNKENECLGNV

YVEGLSKATDHSNLYNKDFSYNKKVNDDDVTTTYYDQTNQSSINYHFDTTANSVSTSKSF

IGSNISDTILVNDDFYKLKATSYNLFNDIINTFEFEQKLEFIKNVIKTLGEFKNFENTFV

DAIFKQNLDMFFNSLFKLFHSIYEDFLKRLLVQKTVKHLALIHKSYTQFKIFLIKASFNF

DERWDYLFETLNIKQFEDFFEQKFKSYFENQKFAIVNLITDTETIQNALESLDPRYDFIK

INFIRIFKAKFTRNYKNMIKKFKTRAKVKEFEVFKIFQNFVFTNITRFNLKSTFDENKNF

NSYFVKFYKNFVLFLSDKFYKAIKEIFSSKEIDEEFRSIKILDVLKLGRVLGKELQDLFF

TTNGIVYTNVMVHFTRNISRDLLELLSDIRNDFVKNMININAGINSEQNYQKSFRLLKDT

CSIIIFINEYRKIFESSNQKGSELTNNKIQYYELESNLNLEDIKNYKIEIKEKILYSNET

LFMHTYESEEDLEVQLFYKEKQLYKIPITDVSNEGRVQIELKKRKILLNLKFRMVVVEDF

SIGFYVDMILKNGSLLCKYINKEIVKLEFDNFGNEALLQKLKNTGVLSTVKGLESDYFYN

VLWRHYCEKIYTSNQDNNKKIKSINELYELLDLSIENKNVLDKENIYYDYILNDVVLIKP

TKKTEVIIYYEKDNLMLQGEIKVKTEVIEDTMRKLKKKIT

>34-9_0454

MIRLYTKFLQHADQYSTIYYTITDHFKNDIYISEDYPINFNYIDFSTSYKDDFENEVDIP

IDFFINNYINFYSNTDNKNAFIDSFVFNKIEHHNNKNYQLILQKNETTYSFIKVNSQHKK

SYSFNDLIDINNLLTKDIFDIFNDIKTLKASINNHKYHALYKYIYLDEITTPKPPKAIDN

IKTENEQLLLLSTKYELKSPTSTQLHEDHNALVKKRKQKLIKINEHLKTLLQLGTLEPPE

KIVNVIDSLKDEELMKFNCVKNTPVLQKSNICCQIQVCQFLRYHFKQ

>34-9_0455

MVKKKFYAIRKGFKTGLFLSWKEAEPLVTNYKGSQFKSFSTEGEAKLYLNGSKTIQKTSK

RTFDSKVLFQVDHEKKYGTLTTPYNKTKDTVKDSQGKIYDVYNCPINNLNFALLEKTVLY

NVNKTSFNWVSSPVYMFVDCFDNYSVIFDNYNLLKTHQNYRNKCVKANEKSATFQRKVPS

LQYAYNYLLNKLYVYEEKYMNKKNIEKLPYIGFKKSDQSIIHAYCDGSSDLKSMGRGIYY

DLQYVKEEDRNILSKLLKEHGGRPDKITTSDKQTNNIAELSACKRVFEDLLLMYQVLNKN

SIVLTLLKLLAKLFVEVN

>34-9_0456

MSDFDKYTTPLSSRYASEEMSSIFSLRNRFTTWRKLWLNLAIAEKELGLNIISDEAIAEM

EKNLEISDEEIKAASIQESIVRHDVMAHVHTFGETCPSAAGIIHLGATSCFVTDNADLIF

LRDAYDIIIAKIVNVIDRLSEFAETYKDMPVLGWTHFQPAQLTTVGKRATLWIQELLWDL

RNFVRARNDIGLRGVKGTTGTQASFLSLFHGDHDKVEALDKRVTELLGFDTVYPVTGQTY

SRKIDIDIIGPLSSFAATAHKMATDIRLLANLKEIEEPFEKQQIGSSAMAYKRNPMRCER

VCSLARHLGSLYNDAVQTASVQWFERTLDDSAIRRISIPSAFLTADILLSTLLNISSGLV

VYPKVIEKRIKMELPFMATENIIMAMVEKGASRQEVHEEIRVLSHQASNVVKNEGGDNDL

IERIKNTEFFKPIWNDLDTLLRPETFVGRAPQQTEKFIKNDVAKALEPFKSMINKEEVKL

SV

>34-9_0457

MSMDVSAELKKLIDDFTDPSKGYLDQDTIEIFNILPNRPHNPEYYNIIKKPMSFQKIYRQ

CKHYNIENIQNFVTDLCQIVYNCKLFNPNESIFYKKAEHLEKVLKEKIIPKIEESLKVKN

LKFVDFTEAAEKPAPVAESKTPEAPDGNEVIQSKESSEKNEVIEANANITMDTSLLDPSQ

LDGTDDGNGVFTAVDGRKFLTSVDVASKFTKVPIGSINRYPNFIKERTVNYERKLFDDIN

EHIARKKGRPTLKSNLELRFINFMNEEKLTTFPLLDNKAEFVFDGNVNTYEQVKFNIKRG

FIIDIYELYKSLEQVTAYQVENCLRKDREEITNFCTRVSKRAFAYFKDLPNEPDVNEKVI

VDRFLMDETVFEIGSFVLLKNPVDPTRPIPAQIFQIWKEPSDSSMWMSCSWYLRPEQTVH

RADRVFYRNEVVKSNQSRIHRAEDIISLCSVVHFTRYVRADPLDVVEPMFICEYRYNDSS

YKFNKIRTWKGAVPECIKEIGEVDVPLPQLRYMKKFESPLKHLVDASLPLNTDAAQKVFP

GLQTEDDREQPSIGNVYRDKIIDKDELGEYSTSRVYTQRHLILPQHFDLLPSNIRFDPVN

QSFIDSSSALSSFVYKVYDSGINVSNLLKTGGSNKIGTHSFSRALPQVSISSTGTKAQRL

ATLNSLTPGPTVFKTKHKQVNPGGAKSTLVTKPFRDEEMKFMTRNAENMKPSITKTILTE

ADKTKKKYTSLPYMQNKKTNKGEISGTKSHYLDALLQLNRHFKGNNTLINENREIINAND

MYLLNEEHSYVLNTPLLNNIEGFVRTDNNMHFEMIKNVESYNEQVVDKNKELILYNNKNL

KNEEEKLLQEFRKEDFKQKEAEEEEERNKVVNSDEEIDNLYLTKNERKRRNSESSMDSYI

DSELSDSEIIERKAYKKLQKTKLVKEEDIINKYYKYVDGMNKGDLIWFTGLSLNISKRVI

DYKDPMSNVGPSLEYLKYKLKKENSK

>34-9_0458

MPYQKVCKLQSGSFSTVYKGNLTENNQVFVALKCCYKPIFNVTNDNPDYQILHNKFQNKL

KKIFKLVNNEYEILNKLRQGSNISFYNNVLNDNNICHMVDFLESSEFYIFVLQYCEFGDL

YDHMKLLRNSNDNSHRPINYHKIINQIHQALIYAHNLGIAHRDIKPENILLDANCNAKLT

DWGLSTFNLTSLQQCIGTEKYLAPECFFPQYIKKEEYAKFDKIPNYIIHQNLTNINILKQ

DGYDTIVSDYWSLGITFLYILFASCPFKFAAIQHPSILKKLDKLPINERMNVTLPENKNF

QLYINSSSTFIEEYYFQNLLKQESLAISKNTIHAAMNIQDNSHYPAFWLTLLPSVQMVLD

MDLANMVYQNNVNDMSKNSYIFNTSANQFIDKNKIYFNDKITEIIGYTIQLLERSIPYRL

WLLHQISALITDNLILTLFMKDLNAILQIQVLVLKEFVRQLADLPAHFISIECIDLNQII

SNISHIYNNRNLYNNPEEEPEVLEEDIIETTSSDNDVFDENHNNRQFSLNAKNTRGFSNM

TLFETHKRSASEEKITIKMDKTSSDSTKTDEYTIEKPDYEDGEEIIQSFQGVRYPDFDTI

ANDGVFGKITFSEDNVPDLSTKPTTKAISDNGLYLAIALADNLKVYSGEKDFSSLIMEIP

LEKPLYSMKFSNTGRFLQTFQQFSVNKEENEGLKNCTVYDVINNKKLYEYTNKSLDNWHL

TFNKNDTFVIQLSNNRKSMKFIKLNKNCDNLSFDLSENIYNTFNFANSIENFEISPNCNQ

PPIVATFTPVKSGKPASLQIWVTSSLPSKKPIEQAVNTKTFFAVDSCIFYWNAKGTTVLG

KTSTNFNSNSYNGENKLYLLSHISNQALLLTSSKDSGPIHDINWNVDGLSFAVNFGYMPS

KTVFYNTKGDVIKQMEPDKKNTILHSPNGKYVLIAGWGNLAGAVDIFDITDNYKKIVSFE

QSNINFVEWSPSGEFLLMAITSPRLRVDNVIKILHFSGKLVYIKEFKELLQCGWRLKDLK

SKDPVYNFKDAKLKIHHHFTVTQYKIKNAGKEPNPGAPKKPATSWRASRGKVSSGLFEPG

ASSGSGSFAAKRQANAKKTVPGAVPGAVPVSNKKTPTNKNGGETKKEELTPEKKVLRNLL

KVYHGIQKIKEKQAAGERLELTQISKLEREEFVIKELKAVGWNGSDTA

>34-9_0459

MSVCVGIIKTVGVSSLGLYTGLLTSSYLISKNITTCDNSELKNKIICKLSKFALVLLSLS

TVFLGVGYFQVPLRYKQPYTLYGFLVGPLTGFYSLLLSKCPYKKTCPVTKKEGECPATKG

ECPMKKDINLSKHLLLLLTGTLPVYCLNILGLYGEGVF

>34-9_0460

MSNITDDYNSDEDLDYNPLLNVNNDSDLDEEDLYDLNNNISSNIKTRNNAKNDQNDLFLK

EQLNKYGNILIQDSNLTNSVDVNTIFNKLNKLTHSVYKQNDSNTHIDILNSQDDINIDNI

LVRNNTQQKIIEKIKIKRKYKFAGKVIDEEIEVPKNSAMAKEYFNSLKEG

>34-9_0461

MTLPSSLLSCAIFKSIYPILFPYNGNNFTDVENVYCQSIAVAVGTSALSLGFVGIVPAIQ

KFLTIEEAGGLDLSILSYKQLLYWGTGLAFFGVFFAVPLRSQVVIKEKLPFPSGQSTAVL

ISVLNGNTPIIQEVSQHEINMMKLKLDNNEDSNQSILTKEDSTKSNSVKQENSDYLYKQN

IKILLITFIISAFYTIFSYFIPQVKAIPIFGKFLSTHYMWNFQPSPAYFGQGIIMGHKTT

SYMLLGALLGWGFLSYIAYYKKYVDINVDPNDWQGGISGWVLWPSLTIMLVDSLIGLMIV

CIKACAKFYLQSKSKIGFNNENFKFNKKTFRASFFGTKSRDDFPELPLPMPINNLNDRNI

VMSTNMNKKSSILGTIRRSSSPTNNSQYGSSDNLDEIAIVDENAETKILKVKENGHTIIY

VSNEHINDDIIDENLLVSNTITITGIVVSFMLCILGMIYVFGIHIIPVSSVLLALFLSMF

LSILAVRALGETDLNPVSGLGKLSQLIFSLITKKNTPGAMTFF

>34-9_0462

MSLNNIENIDLEQKLSLQSICNFLKARTSYDVLAVSYKLIVLDTKLTLKKALTILNNNNI

VSAPLWDAENGKFAGLLTLTDFINCIQYQIKNNKNDGENIIDYLPLKELYKLETDKNFPY

KLDDVNIHPFQSLFQACLEMSKSNSRRISLIDFDEDTGKHIVISVLTQYRILKFLTLNCR

EIRFLKKKIHTLNIIKTFDTIKFCNIHTPVIDVIEILSKTGVSSVPILEQVNEKDYKLIN

CYESVDVIGLVKNGLYNDLTLTVGEALLKRNENDFEGVYTCQEDETLFNILDCIRTNRVH

RFYVIDKNGYIKGLLTLSDILNYILNAGTA

>34-9_0463

MKPHGNYVDQEDNENKNFATNSGNICNVKKSQILYTVKDSFESILPELKRRMRLKPNQKL

AILKQYKKNMLTKIDSKAKTESQSALDSKIVMFDYIPIVNEHEINIEFFKKIICYPKYRT

NYDLIIIKRDDSIPSMNHQIRCYLDKEVYFEKINEYKSIKENLYGYYIMKDINTALTGTM

MSNYNGNISFQSYMKFNQHRKFMSQSEYEEEERANSIDHEKSSLSGKFKTFPTGVEYFER

EDKSKEEDEKLKFMCYDLWVLDFN

>34-9_0464

MDFLKELDINSTPKKASKTIPNETNSRTDSVSGSPRKHSIINISTEDDDSNFLEKELNRK

LDEYINLEQEHVKIRKRRKESINDIKTRSILNENNDNDINNTFNAENYYYNSDLITKPSV

YQYYKTKVMKIYLEFCLTVLSYTPWIIKRIFFLIYSIFNNIIRLRFKAKRTYTELVNQLP

NLKTYDEYLKFAKSVDNLTGANIWRESFVSSSYDYETVLIMYMMLSNYDMNNKDDITKIK

DLLITDGPFMTRNFANIGDVSLYSKSLLGTKKLIEVLLKKCNMILDDLDKHNLVETSYFS

TCNLSLGHTALILNGGSLFGLYHLGVFKALFNNNYLPKIISGSSMGACMAAIICCLPIEE

VNKVLNEDEYVINKLINEEQELLKECGYGQQDLKFLDDGVDTDLNMTKMITNVIFKGYSK

DIFLFYKYVKLRICKKMTFQEAFELTGYKYNIVIYPKEYVITCPTLLNHITTPNVIISSA

IDCSLGSEMVPNCKLLCKNFKGEIEDYLITKNFKIEYLSPDRYNDSNSDNNYSKIFAGEK

KTAYEKLTELYNVNNFIISLARPYLSSLLVNDLKNEINVSKYYQYSKSYLKNIRRSSQVE

EEDLYENFDINTYTRYQKPFISTKWIYQLERKFKSLLAMEFKHRIEVMDDLGILNQWIRK

LTVDERIPSNYCDNMTIVPSISDISIFRIIEGRLDNINYWMSVGERSTNMFKIQSLLTLT

RSYTNDAKFKREFITTILQSTVTKREAKDYLKKYDLNGNKNNISLIILEDVNELLQHEQS

VKKNLNKLLNLGVSPIMLLLDREMNENKEMKLLNYYKISDLLNCTTMPNLDYVPPNYKPT

STNKIGQRMIMYRLGEENSKNQLVDFIKNISRNLGSLEKIIILNKNGGIPYEARSWNSHI

FMNMKDEYEVSKKELNDKLSKIEEKNMELIEKDKHGLDVVKQAIFNLDLMKESLDILPLS

STGLITSYRAIENNNPIIHNILTDRALLSCSLPMLKDYETKKDKKWYEMGINHKKELDEN

LQTTVLKKGVDIKIFKNRLLTQYNSIGLPQKYYTKEAGKEADKKLDLNKMQNIIKESFGR

ELDLNHYLDRINGNIFMIIIIGDYEGIGILTYEKYNEGEIIYLDKLGVLPHLKGSLGISD

LMFIEMFNRINDESLVWRSRKVNVVNKWYFQRSKGTIDLNKIDPDVDSIFRLFYYCNHNG

KDMFTTFDKDSLKSL

>34-9_0465

MSVNTTEEYLVKAINRSKINKKPLVVYLSKNNDLNWINSWINEDVVDLINKRNACILLKI

HINEMNTHYQFFKTYMKSLNNNENEMYEEVFSTKDNVLVIIHNNESQYVINDDMDEEEKN

EILQAFISDFKDELKVVSSTENENKKDDNDLSTVQRNEYLKAKNEDFLERQRLRKLIELD

RLEQKETLNNNENTDTLINEVIIADLKDNIKTKDNNSECKLSIKTPENKNIIRTFDKNDT

LDNVRKYLMEEENIKKEFFFHRIIPRVTYKDMDEFKTLEELDLLPRSVLLVEYKCGNQQK

ELKYFDSKTHNSNGIIRSLINWWNGDVSLPHEHEKEITEPKKKDDRETYNGNTTNVLDPN

KK

>34-9_0466

MQENRHFSQIDTLPRLKNVEISKTLGQGSFAFVKKARLLTPPNTVFAIKFIHLSTSKKFG

LSENDIAKEVIIHEKCSKNDNIIKVLTCDLNKTYMWICLELAEGGDLFDKIEPDVGISDN

EVVRFYFKQLISAISYLHIECGVAHRDLKPENILIDSKGNLKLADFGLATVFKIKKKQKN

GETNVVLKRSYELVGSPPYIAPDLLNKIDGYYPWKSDLWSCGILLFVLLTGTTPWFEPTN

NDIWYCNFIAKDSKPLDGTWNKLGMSELNLLRKILQPSPEDRIDLIGIKQHMWVNKPVSF

ADEYTGLCTDPELLFLKLTKKLKVSLSDYDYQQCTQQVKDSFDVDIQNNINNFTQPAKLN

NINDFAFTQNNIYNDSYLPKIENNFFSQVASDGLDSLELTDWLKNDPSMIQYTQQNIEEY

KKQKNLKIFNHNVNNIFTRFYSNRDLLEIVETLETILVQMKLNVKNNILSDLKSQFSGWN

EEEILPVTIKIKTFDTRKMPLLGVIRIEQNGEDYLKK

>34-9_0467

MLSRTIIKQFNKPALAQRFQSNIASLTNQQLVSKSLKALDPELNNFLDLEKKRQRESITL

IASENFTSTSVFDLLGSEFQNKYSENRPNDRYYGGNEYIDQVELLCEKRALEAFNLDPNE

WGVNVQPLAGSAANLIAFSAVLPVGGKLMGLDLPHGGHLTHGYSTKEGKSISAISKYFST

MPYQLDTKTGLIDYDTLEKNALLFKPKVIVAGASAYSRLIDYKRMKEITNKVNKNCYLVT

DMAHISGLVAAGVLPSPFEYSDIVTTTTHKSLRGPRGAMIFYRKGIRYTNKKGVETPYTL

EKDINFSTFPQHQGGPFNHTIAALAVALKQVMTPAYKKYQQDVVANCAFLAEELKKRNFE

LVSGGTDNHLLLIDLNNFGVDGARVESLLEKINISINKNTVFTDKSALFPMGLRVGTPAM

TTRNFGKEEFAKVAEFIEKGVLLAKKLQNDQQQSAEKVKGPRGQLKQFKQLVAESEEVKQ

LAEEVRLFVKQYPMPGDL

>34-9_0468

MTINPKQYKYQQFLSNKDSKRRLPAFWEHGPFSEELQKNLNALDPNEKIYLSSGVPNKEL

FSVKNISVNHTLDLGHENVGTPGNDVLTNTFHIGNEAFSKNTSSSDYNSNVPIESVFEYS

DQTHGLTSTVDLYNKLITKFNYFREDLPANKERRKNYDVMITSGTSASCFYLTQLLLDQN

KTILVEEFTYPVILANVTMTDGHYAPFNLNMHATEKNGENPIDVVYLDNLLSNWESIHPN

KPYPTCLYTIPVQNPTGLVQSSKHRQEVYNICKKHNVLIIEDDPYGNILLTDEIEKDVNK

TVTTYEDFSNQKDDYLNKLETAASYLSIDTEGIVFRCESVSKIFAPGLRVGVIIANKFFI

SRLKNIVECSTREISGASMTIFNECCYGMDKLLERQVQESNNIKELDGWIQWCMNLSHRY

KLRQDILLNKLYETKAYKEKNLFECIEPKFGMFLCIQLNLKEKKPSVLESAENMKMGMQY

MDYKLLEHNLSAIVGPKLSIDVEHSFKKSGFIRVTVAQATGKEQLSLAAEKIGKAIEDFF

DEYLTPKQKYDVIL

>34-9_0469

MDNRTVHGHIHNYKDFTLVHGHLHSNQQNEKSFDDSYKHFEFINLNKNVGQIDHQRANEL

PISSHSDNTDESNEYPSLKMLMEECHCDPQIIEICCENSDHNADEKSSHECDIEHLEPVK

KKVKSSKKDNLIEYTPKDLLFLDDCNTENKQTSKDVLMNFLDISKMYDIPVLNKTHGKIV

HQENLLNVGFTKKEESLIENGNHNHNHHHHKLELHTHRMHNQVKNEELEVDQIKKKIDQE

KHSLDCHFVCESPNSESFKVKSEEDKSHLDFKWNDLVSADKTHTTKCKWENCDRTFNSLM

ELHSHLIFDHLHNSKKHTHNNQNADASEALKTTNDDERCFWDNCGFEYQNDCDIVEHIND

THGIQFNIQMNPLENALEARPNETHRHNILMLPQTLENEFVCKWKNCGVTCKNEEDLDEH

LASHIPKRQAEYQCCWSECGKFFKQRQKILRHLKSHSKYKGCVCDLCNKTFSTSDGLLYH

KKKMHKIDEVEKQNTHIERNIANQ

>34-9_0470

MADPYLFCVSHLPPIHILPEYLVIDILEYLEPAPTVNFQEVEREFHLLSHPSHEWLMNSW

NGVPFHDRNEIEFYSEVLFSMVPSTSESEMIEYLTRLLGHPHVHSFEQSDEILDKISNLI

AKYCGSAVSGGSFRNFLLSNNDASIKVPIQIKELGIANDNIGWKTWGASYPFSQKVIRNN

TLFEDIASHPENVHRILELGSGTGLAGVSIVKKMLLDEVNNWTLHLTDLPEIVENLEENI

KLNKADIKDKVFVRPLDWKDPSDFKKETQNVKFDFVVVCDCLYSPEHPKLVANTIDEFLS

GNGLLYLEIPLREKYEEERNTLWNLLEEKNFEKILEQKDEGFDDYGETDYVFQCYKRK

>34-9_0471

MVEEDVNFTRRNNKTILGSLSEEIVPQVLMIDNINKNSLPNSRHRSNTAASALSTVGFKS

IATSSNNLSTAKLSSISANESTTPPNTKFNNETTIKNEGFKKRSNTLLSTNFQVSLSNSS

FDSFNDDQDNIKHVHKNDSLALKLSNTSNKEGNQRSSNSRNISGENKAKKFSRLFGRNKD

SAGSSGADKFHENWFLDKKFSSKNAVSLLDSNKILNEMLSASDPENELVNVSKILNHSAG

IGAYGSSNSQSAKNVGFNTNANLDHLMKQDAMLSEREYHYMYDLLKNYSGLESQFAFLKL

KSRTKIINDLILVINDINYAVINEDYMLPKTENLHKIIFLCIKLSFLNTSLEYKEEEYNG

KDQNFYYKSESSNKQNSGSTANGLDQISFQAQSSYSSGNKSAEEKHPKLKKKHVKGKTNT

GIESDKSKVNSNKDNSKDKQYTVNNGSSLSPSVISNSLLFGIIHATTVEELFAIKMPIKK

FEYITKKIYTFLINFYNKIFKDLIYMMENQLIYRNSDEKILNNPIKRLNFIWQNFNSTHI

PNMLILNNDFQIFTKMFLKKFGYANVKSDLKTMLLETFRDCVIMPIFENYDAIIFKNFLE

QQKSLTNNDKLLFLQCLGVLELSIVKDFDDKNQQILKVLIKTLRENMIVF

>34-9_0472

MDLTTYSKFAILGLVNNSLYVIILSLAMDLTSKNTPKSLILLLNILPALILKSLAPIIIH

NRVSHVKRVVCLIIINFLAMWLIAFKKVYLGIILASLMSGLGELTFLQIASSKFGENGIN

GWSVGTGFAGICGSLFVLIITSFFGFSITTCLLGSSLIPLGFFLYWTLPQNEYHQMDNID

LTQNSMATEENFEDDHIDKLGLVKELLYPFMLPLMLVYFAEYLINQGITPVLLFPLKDHS

IIFRKYRDQYVLYGTVYQLGVLISRSSGNYMRIKNVWFLSILQVINLFMLLIEALYFPIE

SVYFIIFCSFYEGLLGGSSYVNVFLNVNEIYRNSSSHKREFCMGVVTISDSFGILFAALT

GLVLEPNLCKHQVANGRNYCKSS

>34-9_0473

MSSVESSEDIKNAGQGTTTAFESHPSRDLSHQLSSFKSADGHRVNVVRSINNQLALEELG

YKQELERKYTYIDLFGIAFSVMSLLPSICAVFYQSTSAGASSAWGWLFTSLLGILPVGLA

LSELGSAFPSASAVYLSAWTFAPEKYKDISAYCVCILDSISLTAGVCSIFASCAGQILSC

AQVSNPDFVVTNGKKYGVYVAVILLGAGVGTLSGGIATRIQQISIFFNCFLLVLVFIALP

IGTSNKGIPFNKGKFIFGNVENYSNWSTGGNWILNALAPAAWTIAGFDSPIHMAEEAIYQ

PRDLTKSVLLDPKASPAAFGVILSIVSCGILGWCMLLCLYACMGPSVDDIMNSSFSSVVT

QIFMNSLGEKWTLAIMGLVSVGCFFMGVSLILATSRNFYAISRDRVLPPFAADWFAVVNK

KTKTPVRAMYGTCLASLLFGLLIFQEQGAASLFSIAVCGFYMALIIPMALKLTYARSTHA

VGPFYLGKTWSFIINLVAVGYQIFMIIIVLFPAVNKPNKQEMNYCIVCTFGILTLALISY

ALFQHKYFKGPRSNLSDNEFLEAVGENHIDDIVYDDKS

>34-9_0474

MDLHNPKDIIKTLKSGPDHENKDSKGNETLTKESKINKSKDVNNDDSGDVISLNTIRTQV

SETDVRRSYENPSKKGIFNFIYGKQENEEDKVADEDNMNYTPSHKEHGTKEENDIMTGHN

EDEEAGYKNLTFHGITSSLKGSLMKNKSSSQTSVKSLGDGSGKNFRNSLNFKRKLKSTQN

KFLSSEKLGKSPKTAEKENVYSEPLSQSEVFRSALQEDLFNMMLAGSPAALLFYSYFTLD

HKNNRRATLLLGALSVHVNKLGQDKHIEHNEEDEDSGIGFKLKQLQKETFRIDLEYGMSN

SKMKWSVYKSYLDIARLHSSLKAFTLTKNLRNSQNSSYIVGKKSLKIPKFPRSNNKKNNK

RNVKYLFAKTKNAATGASSKINEKTKQQMSKFKKNTNNKTLVNDKEVESDHEEEDINDVV

SEISENSDEADDLSSIGSFNASESSIESRIFNPNEHSLKQVQDIIGDHTTPPTSKGGNFD

DNNGQTEITENSLNIGSARLEDEPSDHDKKMTLSTQDNENENLNVRLEKYLKLLNVAVGL

NPIANKIFHFYELSPIGVLLSYEKGYAGKQGYLIMLSSAKANGWRVSHFRFNDWKAMVQR

HTKKWFLVRHSYITFVSDVHSTTPLDVFLVDSEFKVSYSELEGGTAKTLNNLQITLKNSE

REMKLICKSESILKEWVSSIEKMAASTIWSKPNRFNSFAPVRQSCYTKLLVDGRDYFWAL

SEALLSAKDCIFIHDWWLSPELYMRRPVGGNEPYRIDRILQKKAEEGVKIFIIVYRNVGS

TVGTDSSWTKHSLLSLHPNIHVIRSPNQWLQNTYFWAHHEKLAMVDNIIAFIGGIDLCYG

RYDTPEHALRDESMDIPEQNFPGKDYSNARHNDFFELEKPFESMHDRETIPRMPWHDVHM

MTVGEVARDLSRHFVQRWNYLLRQKRPSRPTPLLTPPSDITEEELKSLPYYDFIKDKSTC

ECQLLRSSGHWSLGLKETEDSIQKAYLKLIETSQHYMYVENQFFVTSSTWDSVSVLNSIG

DAIVDRIIKAHNDKTAWKAYILIPLMPAFPSEVDKSDGSSLRLIMLAQYMSISTGENSIY

GKLKKVGIDAGEYIQFYSLRKWSELGEANSLVTEQLYVHAKILIVDDRTCIIGSANINER

SMLGNRDSEVAIIVRDTEFVKSRMNGKEYLAGKFAFEMRQRLMREHLGCDVDLVEFNERK

FTKFKSLAQKHYKTLHTIELGSSKVSKKEMAISAYKELCTRDVLGIDFTKQWEKVHGSLK

GFGILKEDKESNDTNAFEEPTGDSNSLKKAKLPSVSNIFVHTFNNRVGVFNMGIKDDKPV

STDPRVKKENKNMLNPDTFTPEDGYSGSSKFVKDTSEQLNKWLHLNTHNYRRFKDDKKDD

ANKDELNNYAMGFLPTREDILEYLNADEEHISALEKVDMLKKISYLQFLSAKKKLQCTKK

DVISKTVQPVNFQESEEDYIEKVFMDDHRALDAELEQILAMVGIPEQLSVKNNFLNIDKT

KLSKEKYKQMLKEKIFANSSFIDPYMFEDPLCDDFFQDLWFTIAVRNTLIFRLIFHCQPD

SSVQTWSEYREFRDLEAAFKDSQKEIEGILNDQNSSSSKSQSSLNEKEDYVNHKVKNLRL

KLARGMIQGFDKKVYDKYTAVQMLNRIHGHLVLFPLTWLGREIEDNNWFYTADRIPPIDI

YD

>34-9_0475

MKISLLSLFFIFTTTVNFASSADVSIKNVCQQASINNDVACNYNKLSQFSFQEYQKLANW

MEISEHRTEYKQLLEEIYYYHGLKLYLDNNNLELDVISQWERITDSKTLLNRIQPKLDDL

YLKFGFISKIKGTDLLEEVRNYSELDIDEQLELSPYSEHLLHEKIKDSWGDIIDYTSNSN

IQTSGDDFLVIFANDLTTCYKNLLAKHGRKYSLEERSAFFSVLSDIEFLMLNNFKQSQLF

AKQCIELDDSNKHCIDLIKINAKLLSLKTRTKFDPLVIDKNINLKWRDDFTKMIKDEKGM

NTFTKISERLSKYYKSDTKSMFTKIFPDMPYHELLVIYKSIAVDLNKSNFKNHASGKKLK

ALLNSIKLKDIRSIRDVKESFTEEHLKNIWHDKSPYIGLDIIAYLIQNEQFINDNCAIKI

MNIFNFLNNERIFEKGNNDLDDFSYVTIQNVKKMFEEKQAKERILRERQQQQQQEQFFKQ

FQGGGGFDHFFQGGNHGQRQQAPPPEHSQFSKEKDYYKILNISPKASEKEIRKSFLNLIK

KYHPDKVSNLSDGEKVKMEETVHNINEAYEVLYDEEKRKEYNRFRSM

>34-9_0476

MAVSKNRTNLFLSYRKTYPRNVKNDFEDNFDLDLEVGNESIESLTINSNDRSQNIDLIIK

NIILPIEKNLEHIDTLLRQLDQQYKTAILPSFNDDQQADFKKVEKTSFEIMKLIQGNFKY

IKFVQEQIQNDKASKQNEFTIILTNMNTLYASKIQSRSNEYKVLQNKYLKFLNKDDFKPL

PSLKNKANDFSNTDILLLDEEDEQMKQLMIQKQVHSQKKDRFLAERDQEIVELSQNVLEI

NTIFKDLQTMIIDQSSIVNSIESNIYEFQNDVVEAKREIDISERYNKRENKCKITLFLVL

LCLFVLMLLLTKK

>34-9_0477

MKKSSSISTIILIFQFVTIACLVISFVTVPLFKFTSNKKSSTSNLLSLSEHRGIQYGVFG

YCETAKKECSTPSANYKAYEIQQKYKKHESDWKLGLNARKTLSELLIVVPVAAGLVFLNL

IGSSLNIITKSRDINENERFIFVQFIVSIIISFLAFAASAMSCIVVFLLFYPHINWPAWV

LIPAAVLSLVCLPLSFFQYLSKKGEVYNLETGAIIEEKQNDLYGNTRLLSDDVVFEEDKQ

SLDYFNNNDGHVSDESKSKNFFTELKKLNTNNMNSSSDFDNKASLNNSESYAQPSRLVDV

MNNGGSKNTSTSNLSSHYGNKKDSFNMYQEYKQNESEGFSNANSHPAKYDSAALSGNPYR

DASDSSSYYATANSYAAQKLPGSKLERENTFKTNDFKPESILLLDKKSPLENINGMSHKG

SNLPATTEEEVANYSDISDSDRDFVRKNIIPKSQRPDLDSDDGLHDDQGSNFTSVSQRAG

NPQYIQKKFNAQPTSNMNNYQGNTYQKQQVMPQQQQMRAYTHQYNQNNYYQQQQRSGPMG

GYAGQPPAQYQQYNGGQMNRVGINHQALNPQQRTRAPMVFQNNADFNAPIGSSYQQQQYQ

GNSAMQSKFQPPVYKPGYKKKPMGANNMMGPASLSNPYANGSGQQPYSGFR

>34-9_0478

MSSIEEEEKKNQVISQVHPILSGDVMSARIENVRSQLEWEPSPEKSGWQNFKDSFKRVNV

EEIDPNLSIAEKIAIKTARTPLKRRLKNRHLQMIAIGGAIGTGLFVGSGSSLRHSGPASV

INVVICIAVLSVGNAGIFGTSRNMVAMTEMGQLPKWTNFNYIDRRGRPIVALGVMAVFGA

IAFIAASPKEGEVFTWLLSLSGLSTLICWFGISLAHIRFRHGLKAQGRSTNELCFASPTG

IYGSYYACTIIAIIIGLTFWTSLWPYKTAPNVQDFFESYLSGPLFLVIYISHKIYRRNWK

LLIPASELDIDTGRRETDIEQLIADIAQEKAEIAAKPIYKRMYYTIC

>34-9_0479

MNNISKSSNDKLKIFKGIVVTTSTMSESRSNQVSNMIMEMGGNYHVDFPKTTNVLIIDIT

GLPSYDTILKSKKYQYTLTQKLDTIIIDFEILSMIHSLHKTNQLSKIHPILLGGGNGISV

KNDEFLKHIMLNLLRIKYSIKPFDLLKNKINIFFGKFNSSNQIPNYVKSIKNNMSGKKAI

GLNINEKHYVTQISDESRDSCLFITELNSGVRIAAAKKDRVPVVHIQWLIDVIQRNLMIS

YKPYMLNEVTSTDNKYLYDKMMANRCWRNTDLKTLNNDTALAKQVFDEFEMIDNKIIIKK

NLKNKLNNTVWDKIMKANDLPAPREDSVTKELPQLALVRNSLERDGTIEQFVVETNYFSN

CHILSYGFSHKHNKILNKTFDKLDCSTYKAYCVDEDTKENDASSMEADYERNFGDQSKKC

IIIIPHDMDLQTLPSFLIKQYLNSLNLFEQNKLNWNYTNTLIVTEFHVERCLFFKKYLDP

DCWSLPFFNCNLKFLTYKDNSEIFNWTMSGFEGVEILHMNKIIKLTDSLKEQTNFVYSKI

LNTTTDLLIVNLSKIMCVQKNKTGSLYESTNKYLNSFKPEEERLFSERQVNKNIDIDSNS

YLIKKLNFIRNSKNPIPSVTPGLIFQLFYKFNKCQLDQLSSFDQSTSNIVKINDTEWCIT

CPKKENFETKESSGQKAEPYEFLLQMEPKEKSVDIFENEKISQDPLIFLSNNFARPEAMD

ISPTKRTNKNSPIKDNGSEYESPSKNHGSISPHKLKKQKINRSPLRTMSEQNSDSSKKVL

TKEKLAYLFDGGTEGQSSQISYGQKKMSNTANSRLDHVSGNDHEINTTHHSLLRRSLRKR

STR

>34-9_0480

MSSQPTSPIKNQTKLQQDRQKLKDFYKNKEISNTKLKANEEIEQVKKVIKEKIEELKKNN

ENMEVFSNQINSLVKTFDNVDHQLETLNFKEQVESLLKDLDN

>34-9_0481

MSKVAEGIVETKPIGKPDQEELLLTKLVLGYDNDTEYLYENENDVNGLKNAMKMFDEFID

NEDFFNGNENEVFTAENEEEESALDFEIDNSDEENVQDDELFDIENSENEEEAVEEEESS

ESEEDAWNDSDDENIKINIYQNKRLRKLKRSYEETDMIGTEYSKRLRTQFEKIYPRPGWI

DAPVSDEDSEGSDVGVDEESNENEVNDYNALLKLLKESNKYMASVDKTSKILPTDKLTVQ

RLKDVNFKQISKSGIQSISYHPKNHNLVLTAGYDKSMRMYHLDNTENNIIQSVFLKGTPI

QTCQFYSEVSREAFKNCKLKQSQNNNLVFASGRRRYMHKWDLNEINNNNNIGISKISRLQ

GQEFSQKSFEQFKLGHIFEKEGTDGLKSHGIVALIGSNGYVNIVNATTGQLYTFLKISGH

LIDIAIDYKASTSNGIDTIIIAINKQGEVYEFNISKNCNNKVERYWKDPSVFNVTTIAIG

GGYMSDSYLHPVKDIVFNIKNGKMFNNLRSSKWLAIGLESGHVSLYDMNKIRQDSKKIVN

IGNVTPENHDSFKIIDNVLTSVSQLEFSHDGQILAIASRANKDCLKIVNVLHGKVYSNWP

NANTPLGKVTYMKFSNNSEVLATGNHQGKVRSWKIGNYY

>34-9_0482

MLKQTQTLLAGYKLNYSGEVVWSIHKKVHKNYKLAPNSTKYLKNRFHKGTGSSGIGKVDP

YSGKYVFSQSRIRTYPQPIDYDASLKPLVDPNLNRVYEKWDMAKYPKGHLSGSYFTNLVL

ENVENPSKDNTVELTTVTEGVHEGRKIVEKK

>34-9_0483

MAASPILKKATAPKASLGKKLNPLSYLFFTVERSSNKSELENASFLDDKFVYLLAFITIY

LLLQVVPVVGFPRDYKYDNLLNPLNFAGFDIDNKGSLIGLGLFPLVVSGGFIQLLTGCNY

LNFNFSKKADIYAFSNLQKITTILISLTITSLNVFVFDKNFEVVSLLGKALILTQWSIAQ

YIIIHIVECMDQGYGFQSGIMTLALSSYASVLVENLLGVTSSNAANGKSEKTGVLFFLFN

AITSTDYSWVELFQRFFFRNDLPNFSTQLISLIVGAGIVFWFNFRYELIIKSTKMRNVQQ

AYPIRLFYNGIVGIWISFAFLTLVDYIVINTLSLVPIELPPFATPILALTYKLIQGAILI

FLNLSISQQWTVFTGSVKTAIEQFKEQDIIIHSKRDSQQELTKTAEQASQTSALLLGLVV

AIAGIFGFRFQNDIVSGIVAFGSVMTLLEYVIIEMQENGKDSLIFKSLGWA

>34-9_0484

MSQENNGWKNEINIPSKDVRVQTDDINGFERQEFDQFPLNRELLMSIYEAGFEKASPIQQ

QSIPAIIEHYHCIARSKNGTGKTAAFVIPLVENIYKLHKENKSLKSDVNALVLVPTRELA

MQTVQVIRTLSKHMQSIGVMLSCGGNSLRDDIYRLKSMEINIVVGTPGRLLDLTQRGVLD

LSNCSMFMVDEADKMLNNDFRPVLENLLHLMNLQKTQICLFSATFPKSVKSFIDKYMADN

LKEVNLMEGSEELTLKGITQFYCFVEEKQKLHCLNTLFSKLNINQCIIFCNSISRVELLT

KKILELGVSCFYSHSKMPIEERNRVFHNFRKGEIRTLVCTDLLTRGIDISTVNVVINFDF

PKSAETYLHRIGRSGRFGHLGLAINLVNWEDRFNLFKIEQVLNTEIKPIPNKIEDDLYVY

NEEVLQSIPKPFETRIEDTKTYLNSKNQLVDDGYSKINSSVTNNVFVPPKTQN

>34-9_0485

MSDIEDKHKGSYETKVHPILSSDVMSARIENVRSQLEWEPSAEKSGWENFVDSFKRVHVD

EVDPNLSVAEKIAVKTARSPLKRKLKNRHLQMIAIGGAIGTGLFVGSGSSLRHSGPAGVL

IGYFISATFILCVVCGLGELAVEFPVSGGFTTFATRFIDESFGFAATFIAASPKEGEVFT

WLLALSGLSALFTWFGISLSHIRFRHALSAQGRGTDELCFVSPTGLYGSYYSCIIIFVVV

CLTFWTSLWPYKTAPNAEDFFESYLSAPIFLVMYFGHKIYRRNWKIFIPSSQVDVDTGRR

DTDIEQLKADVAQEKAEIAAKPFFKRFYYKVC

>34-9_0486

MSSSVIDKATELETGITNISSSESSENKIERVRSQLEWTPAEDKTTLQNFFESFKRIELE

EIDPSLTVAEKIAIKTARSPLKRKLKNRHLQMIAIGGAIGTGLFVGAGSSLSTAGPAGVL

IGYFISASFILCVVCGLGELAVEFPVSGSFTTYANRFIDESFGFAANWNYVWGCSVSLPL

ELVSASITVDYWGTPKKYRDAFVALFLLVLIVINLFGVKGYGEAEIAFSAVKVITILGFI

ILGIVLVCGGGGPDKKYVGGTMWKAPNGAFVGKNGGQRFKTIVSVFVSAAFAYSGVEMTG

MAAAETKNPRKSIPSAAKQVFWRILLFYIISLTLVGLLVSHTDPRLIGNSYVDAASSPFV

IAMENHGVKGLPSLFNVVICISVLSVGNSSIYGASRIMVSMCESGHIPKWTKLDYIDKKG

RPLMALLVLLGFGIFSFIAASNKQDDVFTWLLSLTGLSAMFTWFGISLAHIRFRHALKAH

NRGPDELWFSAPTGLWGSYYSCGMIGLIIVLDFWTSLWPYDSSPNAEYFFEDYLSAPVFI

LMYLGHKLFTKNWKLFIPSKNVDIVTGRRVTDVEALKAEVAEEKAYLASKPRYFRIYKFF

C

>34-9_0487

MKIITWPVFAYLISISCLVKSLDSDNGLEEKAEIKNTKKIFDFFETDVLELPITPFGSLD

KNLVRSLIIDYPKYEELEENSGFDLNDFSNMYEGYDNTYIRKLLRAENGNTDDKKNYHIN

DAFYCEESQDRCAYRQAHYLLNKVNSTQKDKHLALNILESVESTVPDALFLLGVLYENYD

IFFDDKSSEHEKLERNLKSLVYYKQGAEQGDSNCQLVMAHKYLMGQDVSKDITKANIIQY

SVLKKEIKEAANNSRFLWFMNHQEHNLWNHNYYKFLHDPDYKEFNYVAQDSKHSAISKDS

KISLANVNSKEMLADHDLIGTMVTLKNFHLIKVMDNKITEKVENQLKLSTFSSLEKQVLL

NVYRMDEIEYLSDEIIRFLYSDLFNSYKGNMFSSFKDYQYVFEFSKQLIIYLIDNQEFVE

KNISKVGKYYLKEMLIMFANIYKEYGIYDKNDLKKIDLVLSVYKEAQTDSPKSTAPWESF

KFLSYLRPNMNLTEVFYTYLALNVPDGYCRLASTAYVFNKEIEKIYKNKNFDEAVRTNAE

LYSVLQSKSSFIPLLYEDVNLSFMISTKHQAIDQSNASNKLRRLLNQMQMNCNIDIFKQN

FVNVMNYLLKNDQELNLFAMAQLASMGFVPAISNLANELIQPIKIFNDTNSNLSREKLNI

GVSKYMASYKFGDLNQGLCLSDIFEEFGMYSEMLSLNHLIAETDGTFYSYYNLARIYEYG

YGVKQNYDVAAEYYKKAFDVKSIQFFHNAYKCIEGLEISIQILLWKLKCKKIIDRIGLSM

LGNKVLNSILSFFSVINNKFLNFNILKLHPVSITMSLNRCYGETPLLYPILISLAVGSIG

YFFPAIIRYLARTLNWAIEINGVQFVDGAIVEREDTQ

>34-9_0488

MSTAIQNGQDHYADQTSTKNGETQVTITNAHADSIHDAVLDYYGKRLATCSSDKTIKVFE

VSQGSEESAGANNGYQLLDTLSEHTGPVWQVDWAHPKFGTCLASCGYDGKVCIWKENSYK

KFELISVLKCHTASVNSVQWAPHEYGPLLLCGSSDGSCSIFEFKDNGVVNEPIIIDAHQI

GVNAACWAPITFNGGNNNEKRFVTGGCDNLVKIWKSDVTDPKKFVLEHTLKAHSDWVRDV

AWSPSVLNKSYIASVGQDSQCVIWTKDQESKDNEWKQTVLKENKFDEVLWRASWSLSGDV

LALSGGDNRITLWRENLQGKWESAGEINE

>34-9_0489

MLSAKETIALQRENIAKAAPILNQKITSHFAPITKFEPKILIICGSGLGNLTNKLKLKSH

LIIPYADLPGFQQSTVQGHSGELWVGHLKDTPVIIMKGRLHAYEGYDIRDTVYPIRLLNN

YSEKFCNGSLSSLIVTNAAGGLNLDYKMGDIMLIFDHINFPGLAGKHPLVGPNWDEDGDR

FLAVSDAYDLTARKVFLQKYTELQNAKKITCSRTLHEGTYAFVSGPTFESRSEARFLKQI

GSDAVGMSTVPEVTVARHCGWKVLALSVITNNVVLDLPQKASDIANGEKEISLDTGKADH

EEVLQIGLAAAKDLEVIVEEIAAFL

>34-9_0490

MLRAAKNKVNGYTQSQVLVRDCTRNNDTEDVPIMELRQLVEYSYNHYEIVPMFEMLDKRL

NDKGKYWKHILKALIVLKFLVINGSEMCVTWCKENIYLIKTLREFMWRNPITDVDHGSQI

RLKSRELTSLLLDDARLKRERDMAAGNNDRNKNKPNKRNNFDDDLQKALDESMRTAREEE

ERRRNMKQDQEDMDVALQLSKEEEELRRLQELQRLRQLQLQQQQMQQQNMMYDINGNPIS

PEEYQQFLQQQQQQQQLQYQQYLQQLQQQQQMLLQQQQYEMYQQQLKEQEEANKQNILQQ

YATSSNPFKKEPEVPKPEPVVVPQPEQPIKRTRTGDAAISAKYSDLNNLLMSGPGIDTFG

NTGASRIPAHHTKTGTFINSQGTGYYQQQSTATKNPPSGNNSTANPFLKDQYTGLFSTIK

EQKTGFGFGNAPSMNTSQGNNDQSLIDL

>34-9_0491

MNKQRTLDFFSKKRSTPSIDDSVTDDSSETKTNLMSILKKPKLDVASKPTVTSDLPMKTY

DIKINFQDLAEICEVLENTPSRLAKLDKLEEFFDHIIEKDTSQLDVVTNFLLNQLGPEYI

QDLELGFGEHLIIKILQETFGYKIASLKMKLKEVGDLGQLTYKLRGEMKTVFAFKSKNDD

IKISIDEAWKALNEIAKTNTHKEKIGKIRPLLSRMSPVETKFFIRFLEGKLRIGSSNQTI

GVSMSRCMMKYDQVRGRSMELDAIEADRILRKVYSQVPNYSIIIDKFLEIGLKELEEKLT

LQPGIPLKPMLAKPCKSALDALAEFNDVKTGENDKFICEYKYDGERIQFHQLNDGTIKIF

SRNSEDMTAKYPELVNFSKFYKNKDEVQSLIIDGEIVAYDVVEDKILPFQILTTRKRKNV

EIEDIKVKVCIYVFDILSLNGKSLLEETLEERRRIYMDVFESKKGELQFANNLVSDDEAE

ISAFLDQSVQDKCEGLMVKALTGRKSIYQPASRSKFWMKLKKDYLQGVGDSLDLIIMGAY

YGKGKRTGFYGGFLLGSYNPDNDEIETCCKIGTGFSDEHLELLHKTMKEYESEISPSNYM

YDSQTKPDIWFSQPKLLLEVLCADLSLSPVYKSAIDLTGNNRGISLRFPRMLRIRDDKDY

TMATSSEQVLEMYQSQSNLQ

>34-9_0492

MLKFISSVSPNTFLKKAIFSNSITKRTLFAGVPKINKPLDEVYNKYTHIPEINESYNKLV

QTLVDENKQKLIDLGIKPVNNDTTLQLDSVWRKKTGMMTRHKHKKRRKANQILIVQKKAL

KKQKRANAEAKENYLLANGIEFYKH

>34-9_0493

MIRSNLVKKNHLIKNVNSKQFYGLFDRFKSNKLPEIDKSMDAALFDTTKNLKDINSHTDS

DDVDIIKKFGRPSYNKMNKADVIELKLKRHKPALSQEFDYKDTNQGLESSYPTLENRFIP

WKDLMHTNDYVNAAVNQIKTFSVCPITGGKIEYFDPISGLPTHSSEEAYMKDVEYTQSNK

PELMKLAVAYENDSISKRDFPEYDFSEEQSSNQAISYLNWESWFYTREFNSMETQFHVAQ

ASKYLTYPVTIAALLSNQSPYKLTSQGGPVTHEGLKSLSALRYSIFPRQRADLGFKEDDI

AELQDRAIRIFIVGSKFESLMPISVWKQLNYCMPDVNIEIHFIGPEALFDSKTNEYISYE

ENHKGVQVSPELKVFYHTNLFHVNHLQGDFFPYDPYLDSFYLFHPNISTDPESTKSWMYE

TMKGLLETKCPIFISGHSLDGFSSDWESVMEKYEDELDVLMEPTENAFGSTKWQINPYMP

TYAFQTNMIIGGIRGKRYQAVPVEE

>34-9_0494

MSSDSKDSSLYTLESLLQEYSINKANEKDSAIGIDLSTLISCTNNSFTEKYSNGSDESFK

TNRHQLDILQSPWLETSTNDVEPRYFIPMSFKNINVKYTNKHKNGVNDNKFNEITEESSK

LVESYNDETLFYLFYKHPGTVIQELSYLQLRARNWRYHKTLKLWLTKLPDIDPVMFPDNS

GERGFYYFWDYINWCKKKKEFELLYSAIM

>34-9_0495

MSSESTTVNYPEEFKGFAVSDPEEWSNPKLISFTPKKFDDEKDSYVHIEVETNGICGSDL

FVIKNEWSHNEMTYKKGTYSPKGQIVGHEIVGRVKYASPLAQKNGYNVGDRVGVGAQCNA

CLDCYECKNNKEQYCSKSNGTYNSIYPDGYVSQGGYASHVSCTTHMCFKLNDIKLESKYV

SPLLCAGLTVFSPIIRGIKNALEEKRTPKVAIIGIGGLGHLAVLLAKAKGCEVTAISRGY

SKKEDATKMGADDFIAMSDEKVDWSKYNRKFDLILNCAASTTSLDFSKLLPSLNIYSNFV

SVGIPEKTEVLKLHPVQFLRNGCGISASLLGSKDEAYELLKLADENDIKPWIEEIPICSE

GVHQGVQRLSDGDVRYRFVLTDYHKFFGTGN

>34-9_0496

MIVFEADTADTIEKALNVNTKKTEAKKTRFRPNIIITGTPGCGKSTLSKTLEKIPELKIN

LFNISEYAKDNKIFESYDESRDSHVIDEDELLDRLETPLREGGCIIDWHCNDIFPERLID

LVVVLKCGTSELYDRLKARNYKDSKIQENIDAEIMSVVLDDALESYDERIVIELQNDQKS

HTQENVDKIKAWYKQWLLDNEHGVTNEIDEFYKDREMPKDWDSDEE

>34-9_0497

MKYLVLETITSIANNNNGDNQPLRQNMNISKIFWKIIDPTEILETNPKKLLPHMLGSDKE

LSNLCNKPIFSNPMALIPYNRIEINSNMSLSQAVKTLENGALKELGYSKENIEEKNNLKI

NIVSLHSTWDIRVLLSYKAQQLNFKMPDWLQNPVIFDLCKEYERWCMQDATKISFFNAAA

LNINKRRKLHSNKIYLFTMLQLLGLTMDMYEMDEFGCIIRIFLALFWQNGAYALEKKMAE

NPDTIFTEEMEEVKNCGFTKPYDLGLDFSNFTNDASTVLYMNNLPSIVTQSELTRWFNSQ

DIMPLGFWTIKPAINSPLNYSTDKISFTYVPDSDTISGFAVFPSHEEAMKGLQMNGRSLV

LKLGSLPSTNQNYYQSQQKPRFKLIDRVIEVQPSSNTVLDQVSSYLVPFPQARSKPRPGD

WNCKYCGFSNFQKRSTCFRCQMSNIKQNSNENNTKAQQKPALNPIAYPYAPKTDEIRVKI

VKSALCHTDAFTLSGDDNEWPASDFKPVILGHEAAGVIESVGPNVTDLAEGDHVILLYTA

ECGECKFCIDDRTNLCVKVRSTQGKGLMPDGTTRFKNSKGEPIYHFMGCSSFSEYTVVSK

HSAVKVAKDFDLEKAALLGCGVATGFGAAGYEGIGAAKKNNQITKGSSVAVFGLGAVGCS

VLEGARAAGAKKIIAVDVNDKKEEWARSFGATDFVNPTKLAQGETIVSKLTSMSDDGYGI

DFTYDCTGNVNVMNDALMASARGTGISTVIGVAPSGAQISVRPFYLIVGRTWTGIAYGNI

KGKTEMNRLIEASDKGDLALDKYITHRRKFSEINTAFEDLHSGDCLRTVFEY

>34-9_0498

MEELQKAQDALGNFVQAREKLENQLQENNIVKSELQQLKDKKPSERKVFKLTGGVLLPVE

YDEAMGNVDKRLGYINNEIKNCEKSMKLEEEKMKEIQKKIQKARQEQMNLIQKMQQQVKK

>34-9_0499

MSIFKESADENLIQGRHILDSKVPAVETLVVNPLEEDIELPKVTFIKDVSGDETFKKMII

IPGRLSKIFLRIIAAYKANEEIEGIDDLVTVEYQGKLHKVQSYSKRNCKVPIKNKTRVIM

IDEAMPAIVVNYITKQLLKIIGEEPNGIIKTLVIGTSNDVSEGLYWKSSAAKNFKNGEFV

STCLANFLSYNNNSNIEYLIVPSEGPLSLPKVSAYEISEMCDQIIKTFKLDEDKSVKWKE

DALKMWNIMGGEVVRSLYI

>34-9_0500

MSSSDLEYILYEEPTGYAIFKIKNLQDIVNSSSQEVQKQINDFSSFTKVVELQSFTPFKN

AVEALLNINDISEGIMNDFLKNFLELNVPSSKKSKNGVVIGISDNKLGPSIKEVFPNLTC

ISNDFVQDLIRGVRLHSNKLLKSFNSDDATFIEKAQLGLGHAYSRTKVKFNVSKNDNHII

QAIALLDSLDKDINTFAMRAKEWYGWHFPELAKILVDNYSFAKAILFIRDKATINADSLN

ELVAVLNTTDSTIAEQIIDAARISMGQDLSEIDMQNIMVWAERVKSLYEYKQQLTSYLIE

KMHTVAPNLSELIGEHIGARLISHAGSLTNLSKQAASTVQILGAEKALFRALKTKGNTPK

YGLIYHSGFIGKAASKNKGRIARYLANKCSIASRIDNYSDQPNNVFGTVLRRQVEQRLEF

YSTGKATLKNEDAIKEAMSLAGDSEEVVEQKVDAPKRKLDDDDEEEKPKAKKEKKEKKDK

KEKKDKKEKKEKKDKKEKKDKKEKKEKKDKK

>34-9_0501

MISSTSWVSKGFPQQEPVMDYEVNDNEMNKINEMMKLSGISQDEDLEESEKPSKDDYKYA

DDEDLKKFNMDDYDDEPQTSAAQLFGSREFDKSFLKGANTYVDENGETFVELPKVSGTGD

NEGDEYDEEFENLSGDDDDEKDDMIILPTDNLILASRTDVEGDLSFLEVYLFDEGETTKD

FDREGSLYVHHDVMLPAFPLAVEWINYTPTSINYRKNLDEDRIGNFAAVATFEPTIEVWD

LDVPSKTIPTFMLKGHKGAVLSLSHNPMFRNVLVSGGSDGTIRVWDLNEADSTANEDQAL

SKGEKLKLKLHGKSKISSVKWLKSGMHILSAGYDSRIVVCDVSADKPKADKSWKVPNNEE

IESIELIGADEKLVLIGTDSGTVYCFDLESESGSKPLWALDAHNGGISSVTSNAVIPNLF

VTSAIAEKAIKVWKIDATTFKNPKLVTTRDFGVGNILTCNFAKDFEVSGYVNVGGVSGGL

IVWDLFNNKVFKRQMAEEFTALLKRKEVQETYPNFIYKYTHSNASNNEIFVSTNAEEIDE

EDSDKEDELEE

>34-9_0502

MVDKKDESNGDEKFDHFKKLQELLAQLTTEQYESIKKKHEEYMNKKDPDEPAKSMEEFLF

WKTQPVPRFDEKKSDSEKPAYGAIEHKKLSEVSTSPINLSQNIEGFEWCDMNINNESELD

EVHALLNENYIEDLSSTMRFAYSKEFLKWAMSLPGTDPSWLVGIRVKASNKLVAFISAVP

IQLGIKNPGMDENTVDEVKSVEINFLCVHKKLRSKRLAPVLIKEITRRVNLKNIWQALYT

AGLVLPRPISTCRYTHRPLNWNKLYDVGFVDVPEGIKETAMQEKYKVENKNLQLIEFEEK

YYEEVSVLFKAQQKRYKLQHVFQSKKEFLHQFLPRKDVLKSFVYLNDEGKVTDFISYYIL

PFNILGESEVDIVETLKVAYLYHYCSDCADTLDNDRSLTIRLNEIIQDMLSNALYEEKCD

LFNCLTSQDNNLFIEKQNFGMGSGFLNFYLFNYYSEKMNGGIEKVSKDNWKLQDKSDIGV

VMI

>34-9_0503

MGLTYEDEHIYANTTFNDFAFSVINRYPNPHALHIKSIDILSRTLIPISETNNEQHKLKF

TRLIRKLGSLPRWTPSVLRISDSFILETIIINPVEKVIDSRQQNIDHLNFLKVIEVNNYK

YIANKNEIQQQTKVEFISNIEHNEKKNTMWERLSSRVHLGNKIEKFCVNSYHKRIFKSRE

GIKWKIEFYKLKNGITAL

>34-9_0504

MSTELTNKKIISSDPYDPSTARDTYSGGKPFTQDDIENLKDNENISWANTMPKRVGLYNP

DSELDSCGTGFVANIKGHPSHKIVSDARPLLCNMSHRGATSTDGNGDGAGILVGIPHKFM

TREFKLDCNLDLPAAKQYAVGNVFFKKDSDLKSSIKAFEDLAAETGLNVLGWREVPRDSS

ILGSAALGREPHIMQPVLVFAKLEGQTISEADFIDQYEREFQCRLYILRKLISKQIGLQQ

WFYVCSLSNRTIVYKGQLTPAQVYNYYYDLTNAYFESHLALVHSRFSTNTFPSWDRAQPL

RLLAHNGEINTLRGNKNWMRAREGVMASKTFDKIEQDVLAKIYPIIEEGGSDSAALDNVL

ELLILNGELSLPEAIMLLVPEASHGHMDSNLKAWYDWAACLMEPWDGPAALNFTDGRYIG

AMLDRNGLRPCRYYITSDDRVICGSEVGVIQVKNDVVIKKGKLIPGDILLIDTDAGKLVD

TKKLKAGFAKRNDFKSWLSKVIKFEDLCEKTSKYIPSQLPLNTEFNSSNLHQDPRLLANG

YTMEQISLLMVPMAINGKEALGSMGNDAVLACLNQDPVLVYEYFRELFAQVTNPAIDSNM

ESTFMSMECYIGPQGNLLEMSPSQCDRLFLKSPILSWQQFESLKNIEKVYPSWTTAELDY

TFEKSEGLLGYAATIDRICKEASEAVEAGHKILFLSDKAFGENRISISALIASGAIHHHL

VRNKQRSQVAIIVESAEAREVHHFSCLLGYGADAVFPYLMMETLVKMNNQELVRNANNDD

MVFDNDTLLDNYIHAIDKGIMKNMARMGISTLTSYKGAQIFEALGLDNSVVDVCFAGTAS

RIKGATFEYLAQDAFSLHYKGFGGRSPYGYECISRSLNLPESGEYHWRDGGVKHVNDPTA

IASLQDSVRNKNEHAWDMYVKKEMEAIRSCTLRGLLDLDYDNSNEIPLDQVEPWTEIVRR

FCTGAMSYGSISWEAHTTLAVAMNRLGAKSNCGEGGEDPERSFVHANGDTMRSSIKQVAS

GRFGVTSYYLSDADELQIKVSQGAKAGSGGYLPGHKVDKHIAKTRHSTPNVGLISPPPHH

DIYSIEDLKQLIYDLKCSNPRAEISVKLASEVGVGIVGAGVAKAAAGDVTILVSGHDGGT

GAANWSEIKYTTLPWELGLAETHQTLVLNDLRGHCTLQTDGNLRTGFDIAVAVLLGAESF

TLATAPLISMGCIMLRKCHLNACAVGVATQDPDLRAKFKGMPEHVINFFYYLVNDFRKIM

AKLGFRTVDEMVGHSEKLKRYSKTTTKNINIDLDPILTPAHTIRPGVATRYTKKLEDKMF

ARLDNKLVDEAEITLDKGLPVSIDASIINTDRALGATLSYRVSKRFGPQGLPQDTIVVNI

KGSAGQSFGAFLAPGITFKLDGDANDYVGKGLSGGHIVIKPPQGSKFKSDENVIAGNTCF

YGATAGKAFLSGCAGERFAVRNSGATIVVEKIVGNNAFEYMTGGRAVVLSQLDSLNAFSG

CTGGIIYCLTSDQDSFKSKINSDSVELHSLDDPVEIAFVKNLIQEHYHLTQSELANRILN

NFNHYLSSFIKVFPTDYRLVLEKEAKEAAAAALRTKNAYLKKLERTEKVADATNGEVDVA

AIKRQKLAKAVVSSHKNTLHEAKVADLEDSIQDADQLEKQVEKLDKIKGFKKYKLRHEAY

REASARTDDWNEISDAITKKDAKYQTARCMDCGVPFCTSDTGCPVSNVIPKFNELVFKNQ

WKLALDKLLETNNFPEFTGRVCPAPCQGACTLGINNDPVGIKSVERVIIDNAFKEGWIKP

QPPVHRTGKTVAIIGSGPSGLASADQLNRAGHSVTVYERNDRCGGLLMYGIPNMKLDKKI

VQRRVDLLAAEGIKFVVNCEIGKDVTIAELKSKHDAVIYAIGSTIPRDLRIPGRDLKNID

FAMTLLSKNTKALLEKDLETVQKAIKGKKVVVIGGGDTGNDCLGTAVRHGAASVVNFELL

PQPAKERTNDNPWPQWPRIMRVDYGHAEVKHHYGRDPREYCILSKEFIGNEEGEVSAIKT

VRVEWKKSQSGVWQMVEVPGSEEIFEADVVLLSMGFVGPELVEDPDVKKTNRGTIGTVSD

ASYSVDGSNIFACGDCRRGQSLIVWAIQEGRKCAREVDSYLVGNTFLPGNGGIIKRDYKL

LEELAAQA

>34-9_0505

MSWDDEDFEVSVNTAEPILESWDDDFADDVDILASKANTPEPKKTAPNTDKAKGGKKKSD

KKDEEVLLEIDTLDPKTRADMMKKAELAADLNNAADLFEGLGVSHDHPRAKFTEKDFESS

LMKKASFTKDTPIETHPLFTQCETKKDWENLRKALSQAVTSMSEVSALNYSSLLAIDLNK

RSTLTVFMKEKERKERQARLARVKGGTASGGAGKKKAKGGNANMGAMKKSSEFDMSSIDD

TFEDFGDDDFM

>34-9_0506

MYRANQLLIKSSFKQASLKLARSQARPALCFTREYSIKSKINDTLHDLNESTKGASKIIE

KTEDLTHKAKTTITNAKEAISDSTDDLKSKAHEAKGQVKTEAKHAKKTAAKKADEAADKV

QDKADEIKKNLN

>34-9_0507

MSDENTQTPELPRSLSPSTSLNPYGSMLGGANFGMNSYPMYGGGMGNPYMGGPGMLMGGG

TGHLPSGLQQSIGLLETVLVTIGSTTQLIESSYLAAKGIMHTFREINHQARNIKEDFKST

IRTAIMFIKRALMISRENRKLNAKERSSIKRIIMVICLLLGVPYICKKVVMSGILNEQSS

LIDRNININPEEAKFAKVLYSYTPQDAKKGELKIVKNQIIAILSNTSLTGSESLWWKARN

KAGEVGYIPSNYVEVFDKK

>34-9_0508

MSRGRGRSRSISENDLTDQLFVPKNNVKNANGNLLNTFFLGASQQKRSNSLSASRDNNIL

NHRRLSDSFTTTGPTSTVNNNVSALGFLNFKNMNNNKNNNDTSKLYTSQNSKTSKQPKLS

DLLSNALTFNKMHVTEQKKPAQPKVLKPQKVLIPAKESSIPNQSVDYSNTDKSYISSTTS

TKVDLPTPRVLSQKFDIDYVAGSLPDEDQCESIGTPNLQNPVPSEFEQLCENSGIISEIS

SDSEENKNENINIIRYEKDEKKKNFYSKHNKQLSMTSQDLQNDLHQYESQQKKHQRQRSL

RLVQSVNDLQPSYGGNSEYNYHDEELNGMGIIGYTYADVVDNRMSHISNFSNLSWEEVPL

PVRDAHKRLTSNESPQTTKFSSENGSTNRHANNRKSLRKAKSSRALVEQDEEDDQEYINA

EMQLNLLKQQQVELKMKQMELQIQELKLTNDKLRYAMNDQRQIQDKMIYETLHDTIRTKE

SLETQMNRKVDNLESKINEYKAMISKLTDQNENLNGKIIQDEDQLSDMSSECRSLTPKSA

PALGRSGSLYVKKNRLSKIDPHQLEEMTKSRNTSRIEHSTSVNQASHKRMSTISSTSSST

NGGNSSYVKKKGVNLNLPIRIDSGDNL

>34-9_0509

MLALKNNQLKCFNNVLRFYSSHVASTVPVTSKQKTLFSELDKDELDPATQTPDYVRLTLR

SHVYDAIDETPITKGVNLSQKYNTNVFLKREDLQPVFSFKVRGAYNMISKLNEKYKGDIS

GVIACSAGNHAQGVALSSKLLNIPATIVMPVNTPSIKHSNVSRLGGRVVLFGQDFDECKK

ECSKMAEELKLINIPPFDHPYIIAGQATIAMEILRQVKRSNNIEAVFVPVGGGGLVAGVG

AYLKRIAPHIKIIAVETYDSPTLYNSLKAKKVVDLNTVGTFADGTAVKRIGTETFRICQE

VVDEVVRVDTDELCAAMKDIFDDTRTIVEPSGALSVAGMKKYISQRSDENHASKTYVPIL

SGANMNFDRLRFVSERAVLGEGKEVFMLVTIPDIPGSFKKLQNVLHPRHVTEFSYRYNEH

QQHHLKGSTALPKACIYTSFSCVKRDEDLKRVIQDLNKLGFQAIDISDNEMAKSHGRYLV

GGAAKIPNERIISFNFPERPGALTKFLNGMNTNWNLTLFHYRNHGSDTGKVLVGVSVPPK

ENMAFQKFLDDLGYVYEDVSDNIVFQKFLRY

>34-9_0510

MFAKNAVGPKLNSKKIDEVQLLQNTGYIALQNTGYYHYLPLGLKTLNKISAIIDKEFSIK

EDFKYDIEKLELTQSSPASLWEKTHRYPANKEEVFTYEESDKNLRLLNPTCEESITALFK

DNYMKGNSQLPKVFYQTSKKIRNEKRPRFGLVRTREFIMNDCYSFHDTTDDASNFFKIME

NKYKNIFKKLQIPVIAAKADSGDIGGDESIEFQYQDTRGEDTLLTCNSCNTGFNIEQFSG

DLNKSLHEKVTKNLTFKLCWSGDKSTLIAIYYPEGEELSISKIEEELGDNIDAVSFFAEK

EAGNNKIVDEINAKEDLQNFLFEPPIVRMIDLRINKRGDLPDWPFKIFSKYQFMNFDKVD

LCKPVSDVYSRLLQILAIENQDSFGLNLPKSVAPYKVNFIVDNYKKDITKICEEDPNMKA

FLIDINKKELSEDYHLDLACDIKQFEKAKVSAAVGIPINVYFNGKYTKLPYNEIEIRGDI

VEENWIAKIEAVVEDKDSFKVLKDKKTDTRLPLIKTNYKDTFRVCDELLKMIR

>34-9_0511

MSSVVPPGGARTLHKRAAKQKALKNSAENSKKQQNKSLTSAELDGSTNNILKLFSTGSLK

NSVNIKNEGDGFKVQPVAIIVLALAFILTVVGLHLLNKFVISYF

>34-9_0512

MNMGFSSQWADYILVPKDAPLYVGYDSKQHGADFPHFKLLDDLEKQITSNIEMSNGYNVQ

HYSEYLYTRLIENMLFNNPLSISELEWSFYDRVFCIYIISLFHLKKYSQILTKALPDYIR

FFSGSNSNTIDITSSMYTSTVLHYCTLSLKSIDQLKTPLATSNEFGHLYEILYSCNAPIF

EESISRSCLHIPYERLNALSEEELNQLNRKIKNDTKLFGAPDISSFLLSLADYSSQLKQK

TTLGQDVVDSEALNAEFLLNLSLTFNPYNLEAIERLNRVKLDKSVEISGKFHSTYSTTLE

KYGNLLSAKDIDLGSARWDIKFLVYNVNQIYLNFYNNNYYACKEIMESEAFKPFYYSEKL

HEISGGFKRDFWHNNHNMIYYYLKSLVEVYEFEAAFSFIKDVFDFSKNEIPLVDCEHTTS

TIDYITEIYNLISVLVWQCYKTTGDMKFLLYLKDLILFFSVRKENGKIHCMVALALMYSC

LDQTDKSIEILEKAILKHPRYAYTYYLLGNEFMVKEEFGDAIKVFDIAKCKGYLSYKVHY

SLGVVYLMLGEYHKSILHLSKGLQTNQVNIILLNTYGIVLEKVGNDEDAQKYFELVLELY

DDHGETSTFSRGNVFRENYINIALFKVANYKFYHQHDAKGALDVLSRFDSLNISSGAGKY

DSTLVNIYSLLQDIHENLGDMKSSIAFKNKVIALDPLSQGLNI

>34-9_0513

MNYNQEMSHNNVLSNSSNASQNTLKKENVNKVKEEDFLNIFDFSTTQNDTEENSADKPIN

NHKLDYLSYLTNNNDNAIPTQQYNTNENFNDLNKLLTNTVGNSEYLDIPFPMVFSNNDDY

KQNNIDFDDSGSLMSYTTNNTYTSNKTANGRRKYSKGINPGVAGSVKSYRSYTAESIDDQ

MAKERKKQIHNNVEKNRRELIKKKIKELSEIIPISVMKRVAANALNEGLDSSTSKVYTPD

TIDVRKIKYRKRDVLCGSVIYINLLREVVDGVNKKSDMYSVLINRLKDRLTNSSSHMSRA

DEATAEKGVRRFKFDETFDGNIEEKKDEFAELLQSLGNSDSTGQHPALKLENTNNGLDFS

NVLNNDINKSNNQTFDSMLNLNDVGYDDVFKNMMTYVNSDKVSGLSSASALTNAEENHNQ

NTNGNQDDNINQMIPLKLLAFCKDHTIEIELKQGCAASDKVIRGRFVNLDRAMNIILLDE

NTNREVFVKGNAVKAVVLPNYIINTIK

>34-9_0514

MNNSNINPTIGSVVRVINPYFKSLEDEIDLQVGDHIQIILDDEEYNDGWFYGKNLRSNLI

GLFPKCFTDSIEDNDVKESNNSQRIASHSEKHSENNVRKESNNSNHSLYRSTEKRLFSPY

GTLNRSHSSFINTSQTAPQVRTTSSNSVNRSLSRLNSKNLSSVSVDQEQQDEMLIQQLMK

LCNMDDVLECRNIDSWEPQDVTVFFYNKNIDLDSCLKFYDHKIDAKILFSLTQQDLKNEL

EILSFGSRFSIVKEIDYLKTIVNQGFSGKGVTNKKSLSSKMNEYDQISASPSMRSSRRSS

LLLPAASVRNRSSSQSTNDSVILNNTNMDTLNNLNKNFYNNVERTPSNKNAGYTDASITR

NGTQNYRSTSGNMVPTLSVSKRNNVDSTPVKAGLGLNLNFESPRKAPVPPIAPSPISQAL

ASSPDTHFSNSSKNKLYENNNNNQSSDKLYKNGSLNNSSSNMLKKSTSFLQRNLSFRGTP

SRSRNITPSGATNEANSLSQPKFPNNMNSYQTPQKADHDFNLQLTPKAAGYASKDIQPQF

LNTPNEISQPIYQPPNGNYKDSNNGADRSPHIPNDFILPNKHKKNESGGSFLDLFSKLES

MNVNSAVNTPTMASNHRFLQPTKNSLNQPGTADSAATSVYDNRSSVYDAPGMKFGHTRDT

SSVYVHSRNNTIGVPLNNQANVNDFLKNSVSSTSGNDLLPVDNSSLTKFNESDETSKDDS

FVDFSKDDIDSTLNDKEKIPTHKPLHEINRKLNSGQNLGNVGMTAAEKLKHFKANRAKKV

MNYSRTCKDAIKTANVHGWMGKRNSKSGVRGLNTWKRRYFVLTGTRLSYYVDPDNDIKEK

GIIDITGYSVIPVNVSADSNKNERFLTFISSTMSTKSNYFFKIVPPKPGSKKNANFTQQK

TYYFSVETVEEVKMWMNAMLKATIDIDTSIPIISSCDIPTISLEEAQNLLEKARRDVSLS

ESHISSAQSLDKLDEGEIIEENHKVFQESEDSEIFKSKSGSKELSLDPFKA

>34-9_0515

MHFHEPFHENGTALKKEKSTELLSYSPISDKNISQPNKKIKFIKPASAVYLEPLRGKQKR

PKFLLDFKIHGLSNIPLFDEKSLGESSKKGVCYLKWQITNKKPEEDGTVNNHNIFNKSKT

SKNNVVYMRDLEALKFFTSSSENKPSKNLNFDNMSREEIEESTLSMLPKNDHMHPSGTNP

GMYKIIAQNENKASSKRKVIANNTCLFDYQPDAPTILKFSIDETITNNSSLRTRSKITRH

LNPEYLVIECFSEILQLKEKVSANKGNYRKSTLGTVNADPFTLKQSETQISHGDTISVSS

LDSSLISSEVQATNANSVPSTSNKHKVVSREKLGTVSINLTDYISEDEAWQTHNFLLKDS

KINSVLKFSCHLKLIRGMYNDFDIPHELSSSQMPATLPKLYENKNQISQKFKERQDVSKQ

RRNSTVSSQSSKLVGELTEDLSKISTNYETTDTVKQDVAIDSTANVSQKQKGKEKDLQSA

KILNSPLLSTMKYKNYATNYLRNPYEYVDPKDCIKDILDGKSGWNIDAIFTQPLKQAVQI

IDHDKKQRINEDFLELEKMLDKYGSNRSADSTLKDKRNKSLVDSYKTKYKTTGGWLFSSI

VQKKLSDNSEVEEYPIKEIWEGYDDKLV

>34-9_0516

MFLSHEATVADFNEEIYNIKNFSSRKKFLYGKNIHKTLNQMIVEDSSKSENFEEEPTTRK

LNSWVLNHKNSKIEYAPQKVNKQPIFYHAEPKKKSVISYRIKDNLAVNKKIIKKLINQSY

KSWLRTEQVIQDTTILNNPMLMTLHVGLNSAIVSFDTDYFKNCSNYKGKIMLYGLHKNFP

DTRPMKCPYKSVRYSTHLNQGSTVFPRKNIPPNLQKRCICEHFTIIKSPLK

>34-9_0517

MDVIDVSQESFGDNDIIIVEEKHTNKRSASPIITSSSKRQKPNSTGEEVKNGPENLLLIP

DQSLEIDDDDLDEQIIEDSDVEIIEKPDVAISKDDWAKTQNGDDEVVCIICYGEVENATV

TKCGHIYCFKCLHEYINKGYVECAVCRQKLALKLNRLLKMKYRLVPVD

>34-9_0518

MAVKGSGSRLNRLPAASLGDMVMATVKKGKPELRKKVLQAIVVRQSKAWRRKDGVFLYFE

DNAGVIANPKGEMKGSAITGPVGKECADLWPRIASNSGVVV

>34-9_0519

MSDQNASNLSDDSNFEEWLNEESLVEENVSKEASAIEKTLIGIKDRVLNRNISTIKEKKT

IMNLFTSAINTSASPALYKKVKKEVVMETWKVLINFFNNLNKKNISDLIKIKSQYITENG

LDEYLVNKDSIIDKSITVRMTELLDFLNALAKLLDVAKHNFINGEGFKDLISWIIDQVIG

EDFTVFGDYVENFYDLNGKYSELYRYHYQNMLNVVLINPQALDRNLYDKLLESQYLIFTE

ENTSRFSSCYNLSIKILSNVMKPRLLGKVHGTWEKLLVNAFRKFLMDTYTVKKVNEDFIV

ITQYMLEWSFVFDNENYGITSRVLIDNFIKAKKFDDTVFNFLKNYLDFLPYDVEYQVTFN

KNNFFLDDMLRLLKFLLLNVIEVHNNDTDLIPETYTFKEFPKEGSDLRIYEYEYVNSTDG

LNIIAEYIGIYFYLKEKIKLFDDTSEKDIFVEDKSNIVEPLENSRVKKDVMNYVMPLLRH

SISLCEFIGSLKKACEESINFEKIVPVITAVLLKYQLNCPIDYNFVKHVNTNRNLGLHHK

YMLNSSLLFNVMQCKSDNCISAVRKDIAEALFLPYIEALSRLNENIPSMSYFVGLFTGWI

NREKTALNHEQLVKVRIKVIDILKNIKTQSPIGDSLEIFKVWKNFCLLIPYVDNNILQND

LAERESLIFEGFDFTTKNDSWLDKNTESQNKDLNSLKNYEISNGLTTFELILKDWLPIAL

ICEDALSFSNFFYWGYSQDTDTYIQDAFYAQQKTMDLCYSYSNSYKQKCNVCKEETTDDF

RNGLVNNNKGSTTLPFIKSKILFKNNDNMFLQNFLINISLKRKSKLMSVNRKVYLAENSC

SNKSCPQYTASYNSDVYEGRGMFRSYRSLFYLIKYAEKMYVDNTNNNGLGWMRLLIYLSE

DTEKMSGKIHSQLLSLIDRLVLPKKKYTADYMATIISNYDIYSIEYLNPKYFSIFECLGM

GSEKAAFYNVMDNENDLMYFCQLFQNISLQPFHQLTDRFTWVSFKKIFLESSTFWKSANQ

FDLFLETFFNKVSSLNSQEKSAIVNVLITNIKDDINSEKCHDIHMLKMFLLIVGKILASS

ILMEGQSKNKTLKLWISIHDMSYSGIYQSGTIESLESLFKRLDYKSQNSVLVNIQQSLIE

KQDVQIQFEMLTSLSKISKSLMVFCFKKVLTLLNKKTYTLFELMLSELCKWYNFKSKRKL

FAALNYDIMVVLMSDLLETDLTIVPDHVILSLNALFDEPTLLNVYEICEKLLKNENLGHI

FVCSCLILTTNIDKLKALGILEFQSNDVIKADVLYSSFKKESSICVVDQLRLTKVETLIP

KECYFMLAEEDIKLWKKTSIWKNIFKGLNSEYDIFLKTSIPMELELTEYLNNSKEYSKDI

RFSQSVLGFNLASLETNYQQNICLKNIQYSLENQGEHYLAKSLISQEKEDYINFNQKFES

YYTELQNWDIPLSDSCNEEAYLFNYSKSKLLFDKEYNHEVDTYGYGLIKNLMLKNKKNIE

EKVLGLNDIRLNILSDISLEKTNMLSNSFKASCVPTSEFFKKRNKSDLFSTISSGLVAFK

DILSYLDNQDQYKVLKYYNENLKILIYSNVFADNLKSAFKVDQHSPGYDEMQIPSKQMLK

ELGLLHLLSLSYMMERAENEYFENFNWPIKELIRCQKFIELLNTYYFDEHKEWEDHVNLM

NLICRWHSGDKISSFYHLNNMLERDDNVGNYDFKNILKINKIKWTLESKSETLKTTWTAY

HQLVNDIKTTHKYEDNLFSYHSKDIYTINAYYFGILNAKMDNYNPDYKEMDTLLRKHAEI

REQQISCKKYLTLFQNNAEKKKKMSETLKVISTNEKFVAERYNEIQRSMKNNKNEIINMT

VCLISILSDQICTLGNHAYKETENTDMFFSLWFDLINFFSKDYFDIDKLGNKEVEKLQIE

NIKVNFKQILEFQTIADMPMNKENKEANWIKNRCTIIGWKDILKKWVDSIENILSSNRAA

CLPWLGTIFAKISTGHIEVNENCNIFSVLLECYLNKIIINCIEEYPDYSIHLANSYMNYS

SDNNDKARLRSSLISQLMTSLVVKDSKLMRMADDYKSFSNIIKDLSLTPTLKKVARISLT

EKQNCKVIFKKTPDEIFEVFSEKRNIINPLVDYVKLVKSNSDLKQYHIVNIGDMINMSTS

GLSRPRIFNFQLKNGTQFKVLAKGNDDLKSDEIMLKVMRKIDNLIFKEKEGTDLKYRLLT

YQVFPMGHNFGMMEFIKDVSSLNDILKPMHKDDEISQEELRKKMDASYKAKDTSQARVKV

FTDMCELVTPRFNQFFRINFLSPESWYKAKKTYAMSLAITSLIGYVLGIGDRHMSNVMMN

CQTGDLVHIDFGITFDSGKKLAIPERIPFRYTADMRDALGIYGHYGAINRFFEDVYSTLR

KNHIVVLLSLMNLKLDPLYKWSLEVSTKKLEAMFDLSDDDLDDRNSSYELQDKIYDENYE

DWKHKAKKVAIKPMKNGKTTELFDVVVKPTESLNVKTALLVLRKSKIAYSISEEYSKKCL

DTVSDKILGGKDRQSVESCVQRLLNEAQSNDNLGLIFCGWCPFY

>34-9_0520

MYKDVIMPLMPDLEKKQELGRHAWYLFHTVLSRYPDEPSSDQQEKLQQYINLFAEFYPCG

ECSYHFQELIKKNPIQYKSRQTAALWGCHIHNLVNTHLKKPTYDCSNILEDYDCGCGDYD

DKIGKNTNQQVYARKRHYFDVSRLLGDPFGITTISLAIIGWLVAVVSGICFIYLPHNSLT

SEDIGFPKFTWWGLGFQIFLIFIYMILYCFDLLDHYRVFITCTTAISFVYNTNSTTNLIY

SSGKTKAASASGCIILSVINFTWLLYFGSDNSSPLNRFIDSFSLDGQRPSNIVSEYLQDR

RLANMKRLQQQKQQMSRYQELQKGMSSGYANSTQYVSNLNETSVPPSRSQQQDDNLIPEE

ENTGSISDEFHNPINVAAPSTSKFTNGQSNPRHNRNHTTQPSQSLSNARTSIISYADPSE

EEAIYPYKAVAIFEYVADASDPYEISFDEGEVMRVTDIEGRWWSCLKANGERGIIPSNYV

KLLD

>34-9_0521

MSYFKGSSHNDAQKYKDMTQMARESFEKNVLNKRFHQYNKDNVIKPKVKNKKMETIVNSI

NKTSFEIVIKNHTPILSYREIDSIEDELKMKLPEMTFVNNSIAIKYNDTYEYRIDSMSLM

DSVKREVDETIKVSNSKHWDKHKEKHGSQKNGMTTSNDNTEEDGLNFDWTYTPVEYIGKI

QPLNKNFDINQVEVMKEDKSKIPFLKLAQQLDIKKFDTILHFEDELNDNGISLSYSKIRV

VDTKVPDHNKIDKKKKMKYTYILTRFFLRVDNVMVRVIDLRTYFNLKKPNKIIIQQDVFE

KRFEENTNDLSEINKLKSFESVLDHEHQILKDLDKVDEKTQYLTINLNI

>34-9_0522

MINEYYLLLDKYIYIFNTGYYNQVSDTSVLDKYAGEIQSLLKVFDYQSINLKYITLIELY

LTINFLRYSSHSDDKTILAENSRYLELLKSKQFLSLDSTIYQYYNYLNQAFKLTVSKEKV

TGDVINQFEKNIENLLSGKLEKSTNSIQYLKMNKLFINFKMNFNSVSINSIIILVQSLID

KFPLDLECKWLLFKCYKSLASKNKALYTGYMKAVVEDIILIRPDNYLAWIELSKIIEDKV

DLYNCHLQVIKYTKYNKDSWVYLAKNSENEKIKTIAKRYV

>34-9_0523

MSFNINRANKDPFYRYKMPAITAKTEGKGNGIKTNVTNLLDVSRAINRPNEHVLKFFGFE

LGAQTKLDYKTDKFLVNGVHDPAKLQNVLDIYIKKYVLCDSCMNPETTFQFYGKANSKKV

GEIMKDCKACGKLTPVTMGDKLSAYIWKNQDEYLNLLASNNTTDNDDSKGKHRRAAATAS

ENIQGGGVSISDLAKQQQANQEAEQEEEEGIVADEKLQKDLEDLEEEDSDLEFANNDDWA

LDLSEDAVQKRQLLAQSQPLSANLQILHRLGEWIESEDPSDVEIYKKIISLNLLADAKIM

SVIIQALVESEEVASLTEFMTSFIEEHKAILIKLINERESFEVQLLGGLERLVAGKFYEE

KQLLPKAIIQLYNLDIVSEETILSFYTKCSGRFIEKKKSKELRRLAKPVIEWLQQAEEDS

DEE

>34-9_0524

MDNDSNDKSLSQEINRKSQRLPIYTNELRVLNALGQIQVTDEKNNRYVHNLRCSSCMDTN

CDFIYKFEKFNNITSKLSKNKKLEIFTSTLQSYFQSHHQDCKTKKNVLAFLEHYKIRNDM

STAFTPIDEATARYLFQLTENIHVFDKIRRYSKKLVCLNILELEKSYKIKIFCCNCRFEA

TVNTLRINLPTIIPQSLAVHNCETGSQMIPKKRNISDVVPINKENELNKPNDIIQKLKKQ

RINPNKKQINIMDILKKKKIKDYDKTIKKNNAENEVIPINEQESRLQRLSPSDLGGKRKD

ENLLLGTKNKNAIINNRSVSAGGALTLKKPTNPSAQSRSVSENKFGIDLSLLFDSEEEDV

VETKLDKFPAELDFELSSSENNPTKQGDLLGQLDLDFLDESDIEPEEQVNQNEPLNINNH

DGSTYAPKIKEENNLMQPLKKLQKSIPLSSATLKHVQDVSRITNDSDSLPNSSKYTHKEI

IQSRVISDIPVDFGKIQDFRNSPLNAKKHEQEIPEPTVAQRQVSVLENIKSPILKKTRQQ

FKSATKQHSKKKKYNGYLPQANNIMDSFEDFEDQRNVSLRDASIHSPKRLKKLVLSEEIK

SVDENTIRKLSQKDDILPASKKENSDISKVESNASQSPSVIINRPDDNIELGDDGNVSFN

IEELRPSTPQKVQQDQNISVQSDHKVRTDSPIKRPIIMESPVHSEIKKFNNLKSSPIRNS

IELPKEPVSENREESDSELLLSFNNNKSVLGDPKQNVLLDLKNKANENFVEILDSLDNQE

EIAYAGTTFKEINDFNIKELSLYMARRTLHINKIQSVVDLSLKNEDTLDFVEVLAKRKSV

FEDENNFNKSLLLKHYQLQKEFTWIFS

>34-9_0525

MAVQSKDNNIIPTSSDDSNDEFFEANSSFKLNVIDTNNESAINETKIKKDSAASQVENSS

NNKSILNNKKSLQNVRKNSNTSNQSTLSLRSNKDGFNDTQQKKNNKKRSSSSTFSKIQMF

RDRSNTKKSNVSSDAFLTDTGTTNNNDSRNKSNFYEQNIFEDDELSDGLHINTQDLNHSA

TASNLSINANNSGSNFDTTLSDANNYLLNGLDERGELSSPALSTNMNEELDANLKKNSNQ

KLPSKYRSGKFSSTITNLLPNISAKLHHNKNKAQGEGGSSQPPTPTADNIKFSADKLETV

SSTSPSKLKPTNNLDSYTAPIQQQILDNKAVNAQTVSTKFKILSMMGSSKKQNQILPGKI

YETSTARGTKTSPNMIKAEFITNTSQHQPIPFPNENSMLHSIPTNQSSYHQVGMSQDNTN

INNSLYTNRPRTETVSSQITSLSNVPSYSVWSNMGTNLQDVNSYQYDAGSIMSNNMIQQQ

QMAYSMSGYGNNTTSSSWRPRSKSNVSMTMMNDYIGYQVYPNGYNNPADTLSYQKSHQIS

VKNILPQLEHGESMKIAPILQDDVDHSSFNWVTNYSYAPKINLLPNITQPTDTICVSNIF

SLQKTQSFPTCLNLTSYALHGVFHQFGQILSIRTLYGLNIALIEFKSIDSAKTAHDVMQG

QTISTSIPSEIFYAQIIPLPVSMEADTQGNAKYLLNEQLHNGNLRFVEHGQWTIPVLNGY

IPAQKDSILTDLKNNTAHSNGNSAGGNNSNEIVIEQESCPFALPPPDLGQNQKNIDNVFK

YFQTHNSSNKGDNKSIKEAFDKVLEVLSNEKLSTDPINYGPQPNANGNSTTLANGNRKIF

DSPTLRDLRKALDNKQITNLEVEQICVCMLDEIAELCSDYLGNTIIQRIYEISSPLLKNL

IIENCAQYLTSISVHKNGTWAAQKLITGSTDIINNKISIVKGIEKYCLPLFTDQFGNYVI

QCCLKYGPPYNNFIYDNIIKNFWEIVQSRFGSRAVRAVLETSNLEDDKMLEKHDKDELER

CKVITKRQLYSITSLVVVYSEYLATNSNGTLLLTWYLDTCTIPNRLEILSKQLSKNIVPL

ATHKLASLTLLKLLNHRKDDGNSKAFILNSIFGDLNAQFDSLKNSIANSNLIKILKESNG

NGTQFILKVLSLSLIDVKYKQKAIARLHDILTSAEPEYKEAVDMMKKDKKMVEELSLGTT

STIFFDDTTGTILNTGAKRNRALSHVFNDSTNDGSFANNANGVHARNNSTGSTNSNSNTN

NGTARKNRGYSTSQRGHVNNSKASIWD

>34-9_0526

MSAKNEQIWLEDLERVSESEYLPQKRLFTKFLPKTLPVIPENDSERNLIPRHFLSPLNFF

FFSWVWPIIKTGYQRTIQPNDLFKVNKNQQIHKLHEDFNKNWDEALAKLEAKGKEPGKLF

LIMVLFRTFRWTYSMSIVFSVLSNAGVACVPFISKKLIAFVEEKHFLKSHLSPGRGIGYT

FGLAALLYVYTAFNSHMLVNAFIVGGFCRSVLQKAILVKSFKVSDKTKAKFSQAVIASMS

TTDLNRIENGVAMHTFFYAFPVTIGIALGQLIKNIGPISLIGCFYFILAIAVNVLAFVYV

FKFRIAANKRTDARIGLIREIANSLKIIKFYAWENAYFKNISERRSQEISRLGKMQMMFS

ALISFDILTPTVASMIVFVCISAIQGGLKSPANTFSSLAVFQVLSSVVFTIPQTLSSSMT

AYVAFKRVQEFLLSDEVDPDSLTELPCKDGQNSIEVDGCSFEWTEVQESDDAKKEDKKKE

KKGKAKEDDNEEPADVIVKSNFKGLQNLTFDIKKGEFVIITGPIGSGKSSLLYALAKLMK

KTEGELRVDGELLLNAEPWIQNTTFRNNITFGTKFNKEKYDTVIDVCALAGDLDLLDSGH

NTEIGERGVTLSGGQKQRLALARTVYKDADIYLFDDVLSAVDAHVGKHIMDNCLLDYLNG

KTRVLATHQLNLIEHADRVIFLNSDYTYTMGTVDELLASTPGFKTLMDNFTNEKVAKPAK

KLKYGDDSKELITSGDTSSETDSTFDEEEAEKEREREERKKGRIIAKEQRAVNSIGWIVY

KTYAIFGSGEKIWPLSVSLAAFSQTLNVFCTLFTTVWLSYWSEKKFKHRNEGFYIGIYIL

LAFGSMITTFFASVIVFAFGLNASKRLHNQAAYKILHVPMSYIDTTPVGTIINRFSKDID

VLDNELPMNLNFLLSMIFEIMGILIMAIIYLPWFAIATPFLCLLLIVVLDIYQSTAREVK

RLESVQRSFVVNNFQECMSGLNTIKMFKMQDMFVFKNDYTTNKQNEATIAFQSLQRWASN

NVMLLAVVTTILICVMCSCGVFSISAAATGVLINYIIQLTTSLRLLLIQSTELENYMNST

ERVNNYATELQQEAAYEAPETDPGSNWPNENPSISFSNVSMRYRENLPLILKNLNFEVKA

GEKIGIVGKTGCGKSSTVSTLFRITEIEEGTIRIGGVDISKLGLYNLRKKISIIPQESVL

FKNDIRKNLDPFGDYSDDQLWEALVNSGAIKEDELAAVKKQKFPEDQETAHKFHLDRQVE

EDGKNFSLGERQLLGLSRAVVRNSRVLVLDEATSSVDYETDAKIQRKIKENFGKNTTILT

IAHRLKTIIDYDKVLVLDAGEIKEFDTPYSLFSKSDSIFRSLCDKAKIKDQDFESIDIKK

>34-9_0527

MFVPEKVTSWMDKFNRTKAYYEKQLFEDETQFYEYLSEINLDSSLDEDYEYINKNRDLIF

NRIFELMLKKRDQFLVTVEKETSWKNMMRMKLILFFYLLFVNTLIFIFRESALFNIAVYT

LAEWIYVVLLDLLVFRWMYKIIKKKIFKYSYEVCDKDSQDYYQSYGKSVFGSNFLSEFDD

SDRGHKIQIFKDKASDDINSICVIQPIIELEDKITIKIGFMGTSTDKKLKTNILNKMVEK

VCLEQEKLKQDHPGLQVTIEITTRPFDEEFYKFLINFKEFKFAKNETNEFTLFQQFDKKT

YTRE

>34-9_0528

MFNQSANIELPELPRQLPSKHSYKGVLKDAETPLRLVSFNVNSIATVMQYYPMTKLSSSM

RNLNTFYNWLEADVVCLQELKITNPILPGVSQSMNSGSKLLQQHCFLSDYTCFASLPKAK

KSYSGVGLYTKKTFPFKIIKVEEGITGFLPVANISTKNYEIIHEGPFKNLQYKTSKGTLV

NCYRSDESLGLGGYENIDVLYNDDVERALELDSQGRAIAIELENGLVIFGCYIPANSQLT

EEGLLYKMQYLKVLFKRIENLIAQGKSVVLMGDLNVCKDIQDSEEALQLAKRFYSEAKGE

KISNDINFEFIKEKPERFFFNQQLMNSNFKIPNNYKGTMLDCIRYLHPKRRKMYTCWNTM

KNARAVNAGSRIDYVLLSKNYTDKKIRVLNADILPEIHGSDHCPVYSDLSLGFIFDDKTV

VSPQVNRFEAKTFYKLINNDITKMFGTRKIKASNNKENSLISGTKNKIVKPQKTIATMNA

KVDGFFKKKDSSIMSSKSSYPESNETSTVMTDVDLYNSDDDEFLPSIDDKVPIESSSERR

DFIQQKLDAIKSDVKLQNDLKKHNLNNDIPLFVEAQDAREVGLTVEDKLKRDKLRSLLDS

TNGKHAKIPLCRHKEKAIIKVSQTKGKNFGKKFYCCNRPLIVSDSKKRINTDTENEFSCG

FFKWVS

>34-9_0529

MSEEDADLTIDELTWKQYILQALGKNHGLFGQGVEHTILNTKKIFIGSSYVNIAYIQVSK

MDDKIFSNAINTYINYKLIDGKILTCAVLQKTDEFGKLEINDEEALWKKKLIEQLEE

>34-9_0530

MILNKKTKLSWLLFLNFVGLLCSILHAEDDKKFIPKVTRIDSLIDPKFDDKPKDSKKLYS

NKGGFSNKIIRFDDSHQLLSLGKSYELKYSSNAGDSWDKSNVPKQVKSLKDFSEKQLKKW

NDIVNDNQYFIPSYIEKDYFHGDKRSFVFKRGLADGFLVTENKGASFDFISMKSYIDDAG

KKIAKKILTLNLGSFKVIDYYLVGINTNSDFNNILVSYYISLKYEAQKEEDNLYTEAEVA

FISKDKGKSFTYIDTTSDDYLSIECEFLDKEIGDKVSTSELLCVQKSYDLVKESDDFSYI

TDVKREMVKSTNLGKTIEVVDGLKDFYVNWHSIEYPYILVYVPKDNYNVYGEQDLYISND

LGKTFNKAIIPADTTSKDEESYTSAICQDDLIFLSKNFFKKLPKPNSEIDGGHYEYIMTQ

KTYISDSSGYKFTSLDDATKNLVPNDDEGFYTLTNRLFNFKGVFTLSKTKVDFPSEANEE

DSFKLFMTPGFISFDDGNSWDKFNVKNDKSFICNTETSEKCSLRLQTRFSDLSFDFSGIN

YGDRLMTPGIASVIGVVSETGDFDPELIDSNTMTFITRDFGKTWEKAFDHAVDFAYGDYG

NIIVAFHNDPNSDGDFLQEFYYSLDQGFTWEEYELKEENGEEGLLFWSKIQPLVLDGSGF

QFIASGYKADGSKLSTDFHYIIDFTEAFDKKSCGNDDIEVVKLNSGSCIDGKRYSYHKRK

PDSKCLIRTEFKDLETIIDICDCTEDDFECSSEFIKDESGNCVLDKALITESGVCLKDNS

KSIELPVKRLKFNNECKNSNNFIIEKEKIDCEKLGLSKGDDKVIVHDIMQFDGKVVKYQY

FNTWERNSILVRTDTGSLYVSIDAGVSFEKFRSLESGEEIVEVLFNKHFGKVAYILTTKN

NLFVTDDAGENFVKHSNSIPSDSIQLMFPLDASAKDSSKLIYYGGSHCESIFSKDCHANA

YISKDSGKTFNILLENAVHCEFLGSTLPSADENLIVCEVKNRDQQFSSIVSSTDYFKNDK

KVVYDANSLGFVSYKNFTVFAIVEDDNLRAFITMDGKEYAGVQLPKKFKDFNQRSFTIAG

SSAGSIIMHLTTEMGPDKEYGALLKSNYNGTNFVTIETAVNRDKYGFIDFEYVGGLDGVF

ISNVVTNPGSKDKKLKTKISFNDGGVFRYLTPPKKSFKGIKYDCSGQGLEKCSLNLHGFT

ERQDIRDTITSGAGVGLLIGVGNVGDTLLPYDQSATYLSTDGGITWEEISDKPYQYEFGD

RGNLFVLASYEKTDSILYSKDQGKTWKTLKIADEPVEIHDLITNPTDASLNFVLICSDFT

IAIDFASMYKRQCSVDDSNDFGYKPVQHPDSKCIFGHEVDYIYKKNPECFVGMAPLENKY

KITKNCACTRDDYECDYNYELSENGVCKLGEGLSPLDPSDVCAKNPDLVEVFHPTGYRKI

SLSTCEGGKILDQPSSLPRACPGKEEEFKKLHSVSSLKAGFFVVLPLGLMLITLWFVYDR

GIKRNGGFSRFGEIRLSDDNDDDIIEENLTDVIINKIVSVGVIGFFGLSNIYRKVGKPMG

SIFNKIANTFGVRRQVFRNPEYFSVPNDNTYNTFDNDSIDDDLLFGNDADANDLSAINDD

DVNFDIDEMDDDTDSFKPFSDIPEADDAH

>34-9_0531

MAKVLFNITTDFQIESLLGQGAYGIVCSALHKPTGETVAIKKIEPFQNPLFAIRTLREIK

VLKHFQRCGSDGQGHENIVHLYDIQKPKSFADFDDIYIIQEYVQTDLHKVINSQVLSDDH

LQYFIYQILRGLKFIHGSGLIHRDLKPSNLLINSSCDLKICDFGLARLDPDLVDNKGDDE

QLDKMLMTEYVATRWYRAPEVMLTASDYTKSIDVWSVGCILAELLLKRPIFPGKDYKDQL

IKIFAVLGTPSMDSLNKLPNKRAKMYILNELPHFEKRDLMRFFQERSATQLHVNPLAVDL

IEKMLQFDPKNRISVDDALKHSYLASYHDPDDEPSGQLLTEDFFKFDKYKDRLNLKDLKT

LLWNVIFEDI

>34-9_0532

MSALFKSRVRNKLFLSKFVENPTDVVRAHFRDKQHIGFSGFTGIGGPKVIPNALCEYTRY

HHLNMPGDTERQMRFNLYVGASCGVEEDKMALENMIAKRTPHQVGKQIRKRINSGDTLFF

DKNLSLFPQELLSGFYNKDLNLPDPTKTDALPSNVLDIAVIEATGIDEDGGIILGPSVGA

TPEFLAGASKIIIEINTTIREMKGLHDLILQKNPPFRQPLMHTKVDQRLGSEKLYIDASK

VVAVLESKIEDHIPENSAADENSAKIASHLIAFFKDEVKHNRMPKNLFPLQSGIGNVANS

VIGGLANSGFKNLQVFTEVFQDSFLDLFANGSLDVASSTSIRLTPNGMKRLDKNWDSWSK

KIILRPQTVSNNPELIRRLGVIAMNTPVEVDIYGHANSTCVLGSKMLNGIGGSREFLTSA

KYSIMHTISARPPPTDPIGITSVVPFVSHVDQTEHDLDIFVTEQGLADLRGLAPRERALE

IINKCAHPEYKPLLMKYYSDSVKRSNGKMHEPHDLHKCFEMYFNLEERGSMRAFKTGDKL

>34-9_0533

MAKEYPDIAVFDLDYTIWPTYADTNISVPVKFDPENNHLVDSYGYVVKLFPDVLRIFEDL

QNHGVTILTASRTWAPEVAKEMIDEFGIKKYITASEWGERSKKGHIQDLLKSLGYTKSEI

PNLKIALFDDEGRNKDVRSIGVDFVYLNASNGVTWRDYQNYLEGKVK

>34-9_0534

MTDIVQVVQTVSESEKSIETQEENTTLRPAEFQTGFHELMFLVVCALSQLLNQGAQTQTL

TLFNTVSKDLNASKNAESWLMASFSLTSGSLILITGHLGDIYGWKKMIMIGYVLAFVGSL

VVAFSHYSKSVNQFIVARALLQAVGVSCILPNLVGIVGHIYEQGSFRKFVVISVIGAGAP

TGAVLGCFMPGVITHFNPESTWPWAFYAYAILCVITFFISLFYLPTGSEGNNRKLDIWGM

IFGVYGLAGFNFAWNQAGVDGWQKGYNIGILVSSVISLYLFYKVEKKSENPILDPKVIKN

FKIVTLLVNVLFGWGSFGVHLFYLYQVYLNVANDGPLEAGLKFLPFLVCGLIACALVPVL

ISKITAVGLTMLSSLAFFTGALLFSLTPPDLSFWKMQFIGVLILSFGMDFSFPSSQIILS

DLLPNGNAGSLVSTMTNYGMSLFLGIAVVVQREVSLAHPSNSLLKNIRAGQYFGIGVAVI

ACILSVILLIEMHMDKSNDILEDPENELISSSKNEF

>34-9_0535

MSKAVGIDLGTTYSCVAHFANDRVEIIANDQGNRTTPSYVAFTDSERLIGDAAKNQAAMN

PANTVFDAKRLIGRKFADEDVQNDLKHFPFKIVEQDTKPKIQVEFKGETKVFTPEEISAM

ILTKMKETAESFLGTAVNDAVVTVPAYFNDSQRQATKDAGAIAGLNVLRIINEPTAAAIA

YGLDKKDEGKGEHNVLIFDLGGGTFDVSLLTIEDGIFEVKATAGDTHLGGEDFDNRLVNH

FIQEFKRKNKKDLSTNQRSLRRLRTACERAKRTLSSSAQTSIEIDSLFEGIDFYTSITRA

RFEELCADLFRNTIDPVEKVLTDSKVSKGEVHEIVLVGGSTRIPKVQKLVSDFFNGKEPN

RSINPDEAVAYGAAVQAAILSGDTSSKTADLLLLDVTPLSLGIETMGGIMTKLIPRNSTI

PTKKSETFSTAVDNQPGVLIQVFEGERTKTKDNSLLGKFDLSGIPPAPRGVPQVEVTFDI

DANGILNVSAVEKGTGKSEKITITNDKGRLSQEEIDRMVSEAEKYKEEDEKEAKRIQSKN

QLESMAYSLKNTLSEAGDKLDEADKKTLEEKCSEVISWLDDNTTATEEEYSDKQKELEAV

SNPIMQKLYAAGGAPGAEGAAPGAAPGGAPEGPEEPIVEELD

>34-9_0536

MPSQLEELLLKFKYIRTLNQDEVNKSLSLYGFISLNGEDTVSKENRAILHINKQNLTASD

ILSYNNFKNTKDNDCYHWGEGSLKTKDNASDYKFNLIYPATDKHLSKYEYQNKNFQVIET

PEIYKNVVKPYIDYNINEGSIQWVYNIIQGKTEQEFVRFNEPDFLVLPDMKWTDETDIKN

MYLLLLFKDSKFKSIRDFNSEDHLLMLKNVRKYVEFEILKKYGNTSIEKVKLYFHYQPSY

YQLHLHIVHADNEINYKSMLLGKDCHFLDTIINNMEIAIDYYQRCNMVYYLTDSSELYRR

IKKAD

>34-9_0537

MPTDYKKYLKNISYEKLWFFGHCLTLASALLYALSFCTSTKLYKLIFVGVLLSFGVQLTQ

KYILTPKTKSTKKKSLKKVISDVNFQYFFIALIWLITASKGNLLSIPPFLIFSSFHILSY

VYKKNEKSSSKNTSFIPESAMTFLVNLEIILRNHRGKLVNASSMCEVYLFVQLLFKAITF

QSKSWIQLIGYLIFIKMRLIDYNDTKEVSQENNTQLLINNFISLDQRISIALTKFSSSNP

SVKTVQNVYEKTIKNVIIKQFINIKIPFLSSAPEEKVKKQN

>34-9_0538

MTEVEANIPTSELTEQEEVLKQILPSQPEFKVLKKKLTQGYVGFANLPKQYHRKSIRQGF

EFNLMLVGKDGLGKKTLINTLFNRDLTNEESETDETSDEQISRNLSEKLRRLQLQETLSE

TAGQENASLEENNINLKSINTVIEENGVNLRLNVVHTEGFGGYIDNTDSYVPIMKEIHRR

YDYYLEQENLIARKLNEFNDPRIHCVLYLIEPNGHGLTDFDLEFCSKISPISNLIPIIAK

SDTLKTTEEITAFKNRILKQLAEHNVNYFTPSVYQDDDKENSEYIQELLNKFPLAVMAGT

GLDSTGKRARVYPWGVLNVDNPKHSDFTYLKDLLIKQFMEELKEKTSNVLYEEYRSQKLV

SLGIKQDNTIFKEFDPSEKLKEEKSLHEAKLSKLESEMKQIFLEKVLEKEKKLQKSENEL

FLKHKEMKDKLMKQLKALEDKKYQLEMHLQSNNGSSVKEDNTKSPQASITKEKKKGFLR

>34-9_0539

MSEQLSAFRELTNFKFSGRNFSKEYLDLKSIYDSLSGYADDDGLFTNFMYAALPQSVINT

LPCVSFSSSDELYSMVAARLFEPKSFGPHTSQSFDPDAMEIDALSASGIVSNEIYDVITQ

YQDAMNDPDLAEDIIVALEAIRISRAHQNIRVSASACFYCQRPGAASSFITSDLADTLHS

PTHTADIPIKVKGAFSTNTNTASSIAEMNFTIRGCSQEFTISLYVVDSIGSDKPIIGNPI

LQQFPKLLTPSIATTVSRLMLFLLLQFPLRLTLINFLSIQFSKNIFLEIADELPTPTSTS

TYRHFIELLPDAPLPKSNPFTLSLTEEEEVTTQIDKLLELEHIVPSTSSFKDGTYRMVID

YRKLNKATVDDPFPILLISTLFAKLGNASIFSKLDLLNGYHQVQMNPI

>34-9_0540

MSSVQVYSLKIGNGYHEQSEKTIKNVIYTVLHETLEKLSISEGLDRDEVVSIQEKIDVTK

NSQNNDEYNVTIPLFLLPKNHESNVIEMIHVRFKEIGYSIDNVKKISSVLTEENIFSDKY

CISHSEGKMCEECLCGKECCSSKQEFDKDVEKVADDNNEYVVKALINGITCSACTSTINN

VCKELWYVKSSEINFITKIGIFTLKSNDMSVIDDLKDNIEDCGYDFELMEEPTLINNNSE

ENTQTGNTVFQLSAAIDGIYCAACVHTIENAIENEPKLSDVVINAEINPITKIGNFMLKN

YNDDIKNLLTETIEDCGYDFDIIDEVQNVEQNSLKTKLFRTVNLKIENMYCDQCPLRIEQ

ALKSKIDVPDTEMKLKWNESQVSKSRQNSNKNLVKKHEILKIIYKPSLTNGLNIRNFIEI

IDSIDSKITVNVAEKESLQDHMNKIASKELKNIAIRLMISLLFAIPSFIFGILVMSLLSK

NNSLKQKMETKTIGNASILMWILFAVSTPVYFGVNLIFHKKAFKEIRVLWKMNILFDQHN

GRSSFLSGFNWKAFFKRFVKFGSMNMLISLGTSVAYFASVAMVILSTKQKPGDEMYETYF

DSVVFLTFFLLIGKFLENYSKKKTVGMLNNLSAISGDSTVTIIDQVNSKITDIKYVKIPK

NQLEINDIMLIKPGESPLVDGILLVPLQQDSEFNKETVITQFDESSLTGESMPCNKVVGD

SIYAGTINLTFPIHSQIKDIDNLSKGSLLDKILESITMGQLNKRASLEKTADSLTSFFVP

AICFISLVVWFIWLGLGYSHKLPISYICKKEDLLRCSENWSLFSVGFSISVFVISCPCAL

GLAVPLSMFVGSGVLAKHGILPKGGGMALQNCSEIDIVCFDKTGTLTQGNVIVVDSIATD

TEKWNILRYMEEMSNHPLSNAVVNYINKELSVSTENLDTNKIGNVKEVSGKGLITDKGYI

VGNEKFLIENNYKFDTDMLDVFLKWKMKGYSIVVFGFNRRVLISLALADTLREETKFVIN

QLQSKGIEVYMLSGDNPITARAIGEQLVMDNVDKYVKGGLLPEDKADMVKKLRNDGEKTV

VMVGDGINDAPALSNATVGVAISTSKTANENKTSDLALLSCDFAILQRVNPLLSVLTLFE

VSKVTLRRVYLNLGWALLYNIIGIPIAAGILYKPLRFKLSPTWICEPFVPKFNEDDQAPA

QRNRSDSVSSTASYESMRDNGFKPLRLQQ

>34-9_0541

MSDDKLLSKSTKGVSFLILQQFFTKIITFVMNNVLIRYLSPKIFGIMAFLTFLHDTCFFF

KREAIRISILRISNVTEKEESDEKSKDMLNIENISRNNLQSIINFSYVSLIIGIPLSTVL

FIWQYTKINLYFKNLPHFQLSILILWVSIILELGVEFFYNLNQYLLNYNTRSTFEGMSLT

FGNLINFLTIYVTINSNKFSKVAISEEGVAILGFSLGKLAHSVILLGLYYKDYKKNFKPH

NKFSLSLSLIKPENNDDGVNAVYNSKKKTYYFNEEILTHFKKVYFQMCFKHLLTEGDKVI

INSLCSIEEQGIYSLLANYGSLITRILFQPIEETTRLFLTKVFSSTTSTFTSQKNFSKQT

LTLSFQVISNIIKGYLYLSILILIFGPINSSYLLKFLIGSKWSSTTVLETIRTYCCYLPL

LSMNGILEAFFQSVANGDEIVKQSYMMMALSAVFMTNSYIFIKIYNLSLEGLILSNAISM

ILRIAYCTVFISQFYRKNLPEDLVSSYTIKQLFNLRHIRNIGIISSLLWLFVYKCVGKFT

HNFKQLLFNATCAILLVLLMCYNEKELIMKIIKDKKKK

>34-9_0542

MVEEVKKLEEVDPTVEPVKKEVSEEVSSTAEPIKKEVSEEDVHVDDADDTNTELPNKRRK

LDVDPELKKFLTGLSEQMDSSVDYLRYLTSLTEQDKTMPLNNVANIMKSVLPPGTKISKE

AKQLMQDCCSELISFVTSGATEAVFEDKRKTVLGDDIIQSMYTLGFPNYSEIMTIYLQKY

KEFQILRQLTIGIIEQEGQGKKYKKRQEKEGKAENEEDDNHGN

>34-9_0543

MKSANKLLKSKIINHGGKRFITLDVITANNTIKIQLQNTPKNNHTIKNAVNKQRNVNLQS

LRSFSQTPSLMNKDDKDNKKDNEVNEEDLIKIKEMTGFKESNHTLKEKDSSKTKSSGSGE

GPEDHIPSVYPPVMALRIYKQPLLPGLAKPLVVSDPEIVETLTKCIENNQFYVSVFLNKD

KDGKSNGKFDKEHADRFTSMDDVYNVGTICEISHVYESQSATSTLLLYPHNRVKLSEIIK

VPELEEKSLSEEIAKLKENEGKADLPIDEDESFLMKENVTVAKVSVLNDNELESNPTIRA

LTSEILKSLKSCASVNFPLREEISALAEALDSLSSNQDKHPGKIADFAANVTASSPEEIQ

GVLEELDIEKRLLKALELVKQEMMNAELQTKLSKSMDERIRKRQHESLLYDQMKAIKAEL

GIDDGKPKVIEKLAKKASEMKLPEHVQVVFNEEMDKMKTMETSTEFTNTRNYVEWILNIP

WNQYSKDQFDVKKARQILDKKHYGMQDVKDRILEFIAVSKLQGKSKGQIVCLVGPPGVGK

TSIGKCIAESLNRKFYRFAVGGLSDISEIKGHRRTYVGALPGRLIEGLKQAKTMNPLIMI

DEIDKIAENGIRGSPSSALLEALDPEQNDSFMDHYIDIPVDFSKVLFICTANSLSTIPRP

LLDRMEVIEVSGYTQYEKLEITKRYIEPSSKKKHGLKNVDIQMEEDAIKELLKGYCPESG

VRTLKQHVDKIYRKLAFKIVEEHPEPEVSSKDETSHVKELAEKTAGEIEKETEDKVTTML

DSIDKKTVSNQTTAATESSKKTDDDEDEVKIEIVEGDENAAASKFEIPSDYSRTVKKEDL

KDLVGTPYFTKDRIYEQPPVGVIPGLAAGSMGGSLLYVESCITQPLIYSSSPHYQATGSL

KDVIKESTDIAYSFSKMYLAKKFPHNRFFEKANIHTHFPAGAVPKDGPSAGVTITTSLLS

LALNKPIVNTVAMTGEISLTGKVGAIGGLKEKTMAAKRSGMTEIIFPKDNLAQWDEIPDI

VKEGIQARPVDFYEEIYDYLFGKVTVDEGNTVWKKDFDVIDKKKKK

>34-9_0544

MNIDGIIDAVDYLENQDKRDYDAYEALPYKFDTCTFNVEDAKRQMIFSCLDCKVGICYSC

SISCEHYKHELIEVGYKKEFDCECGSLKNDKDFNCTLFDKKGIDYELENTKGRNFNGEFC

FCDGNPLSDGDDKMVQCTLGHSCNEDWFHLGCLNLGKYRLEEDDFDEFICSDCYNTYKSM

FDEIFNKYDKMAKLLLIKITTLQPEYELKPALLLKENYSKLFQYVLNKEKQSQHSSLETF

LNKIVYHDLVNPYKLYEPPKEEFTSIIKVDSTNYIADEDTYSKNETDIMRIAVEEKKLDV

EKFNVFKEGLKTFLTGFAKNKDVVKKEDIDYFFKNLKSESVETAVDNNDLSTFSNPESEL

EELESD

>34-9_0545

MSNSNNKEFNSDNNITNNNTSDLPSNLNNPSSELNSEDINPINRRRRRRNIQDTGSDLED

SDVENDSILNSQIISQQGFHTDINLEHSNNRNSNLQSSPVRPARRGRLVREDDALQSSLY

GTIGENSNINLDNIEGDEEEEDGLVDERIPDENQDIRSIDDVANEELIDEEDEEENDVDL

MNEQFMEKDYNENRKDDHYDVNDKMIDDKEINDDRNMDGAYRRAADRQLNQRDRLLERRR

RNEDLLSDFGSHSDMDFALPSNVGRRKPKKPSGNDDPNGYYNYDDDDMEAEDEGLFNYMT

LDELANATANSYADWVSQQKVADTIFYELQQFLIEYQDENGRSVYGNKIRILGEMNLEYL

EVSYRHLSESRAILAFFLIKAPEQMLKLFDAVAMTTVISQYPEYGNIHQEIHVRIVDYPV

LNHIRDLRETNLQTLIKVHGVITRRTGVFPQLKYVKFDCLKCNQVLGPIFQDSNQEIKIT

YCSNCKSKGPFRMNMEKTVYRNYQKVTIQESPGSVPAGRLPRHREVILLWDLVDTVKPGE

EVEVVGIYKNSYDGILNAKTGFPVFSTIIEANSIRRKEGFSGRKSLKDSSSKRKGKKSVN

DEDDVEMGLSSDEDNDSDDELSDDDHLMDSDDDVDLFEYTEKDEQDFIKLSKDKYVIDKI

ISSIAPSIYGHKDIKTAIACSLFGGVAKNINGKHSIRGDINVLMLGDPGTAKSQILKYVE

KTSHRAVFATGQGASAVGLTASVRKDPVTREWTLEGGALVLADKGVCIIDEFDKMNDQDR

VSIHEAMEQQSISISKAGIVTTLQARCSIIAAANPISGKYNSTLTLSQNVNLTEPILSRF

DILCVVKDMIDETRDRQLAMFVTSSHMRSHPENTNAQGSSSINKEQERLQKEAEISPIPQ

RLLMKYIHYARSRYYPKLLNLDQDKVSRVYAELRRESIQTGSFPITIRHLESILRIAESF

AKMRLSEFVSSSDLDRAIHVTVDSFIGTQKISVKKQLQKAFAIYTVY

>34-9_0546

MGRNKRSNNKPNAGKRSQTGNWKEIEKENIKWETYYKKQPIFKDDEEFETFKKFCKEGLP

ITFRFTNGNNITTDNSRQKEILNLFKKDILPHLDNVEYEDEVIAAPVVIPWYETEEVHDI

LWQYNISKTIIRKKEQFKKFQQFLVMENVLGNIARQELVSMIPPLLLKVQPSDKVLDMCA

APGSKTMQLLEFLHASEEATGFVMANDADFKRSHMLTHQLKKINSPNLIIVNHDAQFFPK

LFLGGDNKNFMSFDKILCDVPCSGDGTMRKNVNVWKEWTIQGGNGLHQIQLNILKRGLKL

LTPNVYTEDNQLVGNEARLVYSTCSLNPIENEAVVAAAIREFNSDPYNVKRGLKIRLLDS

SKDLNGLKRIDGISWWKVFNREAEEIAEYDENDEKCAKLSKSMWPPTKEEVEDFKLERCI

RVYPHLQNTGGFFITAFEKYKVGAENPAKKVKLDEKAEENEEETAVKEADTVSTAATEPV

KKSRMPRDFNEEPFIFLPSDHEITSKIINYYKLNDKFPQDTLLVRNASGEPVKSIYYTSP

IVKDVIQTNESRLKLVFSGVKMFIQQSAMKTVPDQNVADINSRFNWRIQNEALPHALKFM

DLDTNNLIYRTSNSDFVKLCMYHNFCKYEELYKLEPSFKEYMQKVTTGGCAFIYFDRKGS

SEEIKENLMFPVWLGKNCLNFMINKELQQELLYKLFQLSVEEIKLLQEKESAKAKAKSTQ

TEAKPVESVEEEKEETV

>34-9_0547

MDKQEHIENITKKKYLPPSVFSYLLNDNNYNKSYDEYLYKFIKSNIEKEEERENIFPFIE

QEISNNSTAENDQMVDVLSIYLHVYEYNLRNKKGSERSKEMMYDESAMLSLQLLLDDIVD

IEADRVYEQLKEEVTGTGSSNLTED

>34-9_0548

MALFTSGSDINELRQDPGLKKYFPEGTSDEDITKIVMKILKERQELKEKSVEPKKQAKPV

QETSNKKTKHEKKKTEKTCSFCQNKGHQRSKCPKKLYPGLDL

>34-9_0549

MLFFSLFKTLIDQEVTVELKNNLKLTGTLKSVDQYLNLKLDNISTPDSFFDENGDMEYIN

NPHFAGLKNLFIRGSNIRYVHLKKSFVDCNLLQDAARRELAMEKRDG

>34-9_0550

MDSLDGEEFDVIILGTGITECILSGILSSTQNKKILHIDKQNYYGGDTASINLKQLFERN

DKPTENLDNGKFGRARDWNIDMIPKLLMSNGELINILISTKVTEYLEFKQISGSYVYHKN

GKIYKVPSTEYEAVTSSLLGLFEKNRVKNFLQWVSQYNFENPKTHQGINWEKNTMEEVYY

KFGLGNSSQDFIGHAMALYPTDDYIKEPCKECIERIILYSQSVAKFGAGKSPYLYPLYGL

GELPQAFARLSALYGGIYMLNTPVSDIQYSEDGKFKSLTIPQGTVKAPIVIADPSYFPEK

VKSTGKKYIRCICLLNHKISSTDQRDSLQIILPQNQIGRKNDIYIAALGAEHFVTPKGWN

LAIVSTILEDTEGKNPLDEIKPALTLLGEIEDTFYEVKEILEPKEDGSKDNVFISKSYDA

MSHFESMTEDVKDIYKRVMGEDLVIE

>34-9_0551

MASVFTQSATKPSNSKNNQLTPNDRNAIFHYYMTLNKYFNTNKLENSPGADRSKSSRAVK

ARKRLLNLHTYQFFELITDVNDEVERRTSSDQDTEYLLPQEDLHLKRNQARQKLANLSLK

RFLDLVDDLSFEIRRRQYDLDPQFDDPVDVQEDTSEVIEPKENDFPAAVNNVGSSTTATD

NEEAEKSITNKPSVQPVNVKKADMDWSDDEEEHENNQIVAPNAEKINNDFSDLKMSAGFS

RQNVSVSRSASNDNIGIPLDNKADHENSTIKEKDLALEAANASINKRNDEIEKSLQLKID

DLGNKNSSLATKNQFLSEENAKIQEKLDLEIQLKNRLIDELSNNEEIINHLKSQVESNDK

SIDNEQYQAKNYINIVSDNKKLSIENEDLKQKITDLEIGNRLLNTSEGIKTDIFDEGNVS

NFGVEEYFSQGIIPIEHVNEFQTKVVDFSKKVQNYNPQQSNKDLFLSAIKLSDIVTNIVK

AVEMEDSNQIYTEYCILANTSSTHLITTLKYQSKFSAIFPKIIIQNALLEILGSLYQLIY

KAGCNLNKDSKVHSSPNNAQNTRNLANSSISDISNHSIKGIDNNTVKAQNEFTDSTVRPL

KITQRMSSLPQIDDIESSTNTNVANSSVHRKHSNTGLFTGMLVTSAKANADSTENLANNS

NKNVKGLSLNLKKDAEKSISSVKQGNNKSQGVLNRVKAFEDSSFSEDNSTIKDTTVGNVS

DISVDSRQHKRTPSKSFQEIDNLQKRNSLQSIEDRVSAFGEMKNAKVEKIKSDLRKSIDT

PQENINENAAADKSLDSNRSYEATEEEKDLNNLLHYLENESIEVIQTIQSLLTSIKNPKT

TIKELNGESEKIMTVVSKVCVNAMTSVEKNNDLKTIGEYIITSLQDCERRMKSLIDTDNK

GEYPDKSFKQRLAGIAFDIAKNVKKLVKVVEELSLKNEIAHLDQQLL

>34-9_0552

MFTGQVQAKRFLKAKIRLPTPHSNHKVNEFKLKEYKKNETIIPLFSHTNSENNKTNYLPD

FKNLEEFKLPYEETQLSNLYTNLSTLFGQNLVVKNIPKNLLVSEKYFNGSRLPQPFIRYS

NNQEVERTGNNAHFNAIGENYINFIKEKYGLEQTNLDKISDLEPTAVLGFLYLTNYNAGN

SVGFESKLLQVLKN

>34-9_0553

MDIFKVLTRGTNIKSNSINRNYTKTTKKTSLEEDKELDFFHNQTKKNTASNDQIEEQSID

DEDVSMKDLLVKNHFNGKLIYNIDNHLLNVDKPTEIQSKSIKIHTENEDIDIIGISPTGT

GKTLAFIIPLVSKIIANSNDSNMNSLQSIIIAPTNDLAKQIFQVTENLVQGIVLNHKVKK

SNNKLDVQLLNSKLSNKLLFENDDNSIKLPQILISTPKRLISVLNKENTESILENVKYFV

IDEFDQLFSSDFIKQTEKLLRLFDGKSNINKWMYSATKPEDKVFNDIIKNHMKQPELIEC

KYSSKLTSNIVKPKITESLVFVGNEQGKLLQLRQLQQNAQFIPPMLIFLESHARCMALYN

ELKYNSIPMATLHSKMTLNERENVINDFKEGKVWVIITTDIMARGLDINGIRTVINYDIP

KNKELYIHRLGRLGRGLTSYSDNKGKKKIECECITLYSKIDAKDVVPIAKVMKQHGAEGV

PDWLVEGRVREEKKKKDVNKLKREKISTVPGIIRKERRMKKEMIEASKKRKQEGIVRPPK

KQKKANGNN

>34-9_0554

MSSILSKVTTTAKKIYVTEGLAPPSIAQFKQVYSLTFDALKTSWKSSDATYDRLVKVTTR

QWLYYGLGALQVLGFFAIGEAIGRGKFVGYPIYLPKHEEEHH

>34-9_0555

MSPIPSDDDNFLPSSDPNPHLNSELEQRLPNNNIEDSSLGESNNLRNDGETVSSSLNVSS

QVGNNNLNSQQEAVSSIARHQLSSEINSNIRSNSSSFLNSEVGRTPRHQKRSDIGTINEQ

RLFNMTSSDLGSQSSSRNNISRAQEPYSMASDFEMNSSSLYNNNGNNLPSSDLGSDPAEG

YDYNNEDGDDQGPKSVIWGTSVDTAEVRRIFENFLMNFKYADRKDYEYSLINPDSQTAEM

YAFQDGDDDAYYVEKMKKMVQMKKINLNLDCRNLLVNTRYKKLYGWLLKYPNEVIVYMDD

ILKDCLMKICEYDEEWMTFRDELELKFPRVRPYNIENTRGIRELNPGDMDKLISIKGLVL

RSSAVIPDMKNAYFRCGICNHHEAVSINKGTIKEPSKCTSPSCTASNSMILVHNRCTFSD

KQVIKLQETPDLVPDGQTPHSVSLVVYDELVDSCRAGDKVVITGIFKCIPVRSNPRQRAL

RNIFKDYVDVVHVKKISNDKLDLDTSTIEQQLLQKEMEEEEAGGSGVQEIRQLSAEDVDK

IHILASRYDCYDILARSIAPSIYEMDDVKKGILLQMFGGTNKTFKKGGRYRGDINILLCG

DPSTSKSQILKYVHKIAPRGVYTSGKGSSAVGLTAYITRDVETKQLVLESGALVLSDGGV

CCIDEFDKMNDSTRSVLHEVMEQQTISVAKAGIITTLNARTSILASANPIKSRYDPNLPV

TENIDLPPPLLSRFDLVYLILDKVNEDLDRELATHLTSLYLQDSVKTGASDDILSIEFLT

MYINYAKQNVHPKLTPDAGEELTKTYVNMRRMGDDPDSDKKRITATTRQLESMIRLSEAH

AKMRLSDTVEVSDVKEACRLMQSAIKAYATDPKTGKIDMGLVQTGHSVDEQAIREEFQTW

LINYLQVNNYVLDSTISVSDLIKKFRELVEESKQDDEINEDEGSTNLSQLGSLTNTDIGV

IIDRMTGDGDLITKNVRSVKHISVSR

>34-9_0556

MDDGSEDMLEPKNEEFQIGFNLCLNNFFFDIYLPFTQSLINQLKINSCYSDNKILQHIEE

NYDALHEYLQDLIIPTANVSDADMSEPSVNYNFPSVNVDDFFKRITALQTELLRINHKQL

FESKLDSVVLKNLKRDAHKINDKSYTSVNTSKDSASRNNVLSEESKTSIQHRNHPVRNIK

IENDEVQKENKDFQKKKSIKLAETREIDMGEVDKVSLEGTDSGKNEISQTPKNESIKPEL

VENPVNKKVKRTKAEYLQELADKKRLKEQEVAEKRRIREKELEERRRLKEAELEEKKKAR

ERELEDKKKQKEKELEEKRKAREREIEERKKFKEQELEEKRKQKELELEERRKLKNQKEQ

ERLEKEKIKQQKDEENLSKLRISNFFKKKNDKQDNIENRNTKPGTFSRPITLNAVTDDSL

IEGENLTNITINDSSIVQIDEDTICDFDKEFQDFYLKTNYERFGDNTNKKSRNVEIDSII

NSQEDLTQTLKDSFVSLKNLKAEYLSKQVLITLQDNSLVVFHECDRGDMSDTELLKLLEK

VPHKYLKFYENVSLPFTGTYSESTLIPANNPFDVEKTDFEYGKDSDYEAVAYIDSDVDGD

NEFDDGEEGEELDSEDDDEDEDDDYDDSGNELDEFVEPDATGVSSKDTVTKNSKKKTLLI

PTIQFNNKEAAVFLDPLQQSFFNDNDREYIDSISATILYPTPININEPIGLPEEKEVEPK

KRTLEKAQSESTDIILKDGESENTENSTALSNETKTSKEEPDPKRAKIIISDTKALIKML

KKVSGCPYSKNTMVEVIGFDLESKFSRKIIKDTVDHYATRVKDIWAMNDPDIIKKLEEKI

ISDSSNQ

>34-9_0557

MSHIYKCSFKECSSELITFNKKDFNAEKQDITHLNKAFIEKYKLFTKPQDLNATDDIEFL

VTDSFWEFDNIGVSRSIETLKLEDHTQYKVTFNKAEYLLKSAKKYLICGDCDKGPLGLIV

DVEEIANPSNKRELCLLSTASVV

>34-9_0558

MATRTQFENSNEIGVFSKLTNSYCLVTLGASENFYSAFEQELGDSIPMAHTTIAGTRIIG

RMTAGNRRGLLVPTQTTDQELQHLRNTLPDEIRIQRVEERLSALGNVVCCNDYVALVHPD

VDRETEEIISDVLGVEVFRTTISGNVLVGSYCSLSNQGALVHAQTSVQDQEELSSLLQVP

VVAGTVNRGSAVVGAGMVVNDYVAVTGLDTTAPELSVIESIFRLHDAHPDSISKNLRDTL

IETYS

>34-9_0559

MNFYKNCDKLSLRSEAIEHLIQLAYEDEDDHSIIDTTKSQLTVSLFKGKNKETGPLLHSY

PLLFSEIEILFGICETTPTTALNGRSTLTDVIIPYFQVLSKQLFSDIVLNKFSASKLKNQ

KELFGSSIYEIMAIKLSKYLINCHNLFDELKPLVESTFAHFFNSLNSKFVTNDIFIILGI

FKAANQISYKSSIHEKLVISGWKFLSLYDTKFKLIVQNDIGNETLSAYYSSNEDITSDIF

LFEVLKVQTKIIESIFEFTNDCESFIEKMLVLKKNKEVEPETYASVYEKNLSLFEKNKEF

LTLITIHSKNKLQAFENIQLDISSFLYLNNFFEIQSLLLQTCSLNFFNFNIKNVNSCVSI

FNNFVGIFTTNTFLPSAGFLKSIVAFASLMNYYTDEITSELVSFFPVLVTNNYVTTEFVE

NISTIFAHGLPALTEDAVVNSVYDISNLFGEGRDKFLHKRKMTLTKSFNDNVSRKKSVSL

APFQSMNILSKVLNKKNKGFNESSSSLGATNGSEVSVSELANSVKAVDMDDDYVIISDAS

VEKAVVSLTTIGSIFKDNSITGLILTILIQKFDSVSSSLDTYILENLHTLAIVMDKNEFN

MIFKFLQQINIQTQDIDKKKALLSSKSKIAKTLKKSKKFNEDVYNAYLKFLLEDLDSVLP

ETSYEAKKHKKADTDQLFSNIKVFKEYVELLAEMLPDKNNDILIVETHTASLIRDLWIKL

ASYGFYFDIKLQRLQNCVDSKISIDTDALKIIALNTTQLAAFNPQNTSETSYKLNMILQR

PVLSRITDFQSKILRKNFNYSNNLSDDQILFLTSAIMLEYHRLMISPEKTIALTLEYLLD

DTIKQDFGNFFETFAKDMAHTICSSKKFRLQTSSKTYSDILNSAILKICSRNKEVQKSGF

YVADKLISYLPECLNTKKSVFLCLNLLSTLYQSVYDVAKNKYELNIEYTVGKNHIVSLPL

SEAWRKDTLRYFEQHCSSWFDILISRTPEATRALLFQYIFSKIEENYSSMDRVNYGVSFA

KNKATSLNIGATEPDSYLNQKIDNSLKSSNNYWSSVDHSISFLTATSSNHMFESKILQVQ

YTGLKNSIFDGGDATDKNVPLFLSTAAKLLSIDIEKEDKSKTASFICDIINASFIKSSSN

SNLSFGVSCWGKLLSQYPELSGLFLTELVKMWDIHIKIPGFTLNEDNGIGPCEESIMQYT

PSNTNAIMEKAEKLLKQSTGYVELFKFVEELSVKTMQFSDNNILSDCFVQLIKSTTDVME

SAGNINIHPALRFLKLRLFKTFFKIAESCHKRINKNSTETMYTIKNLSDVMKSLTTLCLK

MFSTKALWPFGNDELAWDELYYTINSIYKILSSNLESQSSTRWKKFLIASCGIEMKVLQK

VLVHELYTISIWSDLSSDSNSARTTAVSLTKLDENLVKDAFIIDVPLAASLIDRYVGWGG

VNASSSKERAKMNTLFAELVNLSPLRTAKTYGPGLKHLSNSNFVKYGLLFEKVDPMSTLN

YMLNVGEYNKFVKSSETLSVILQYFMKSLESFSSQVTFFYIPQIVQCLRYDNNGYVERFI

LDSGMINSYFAHQIIWNMKANKFKDDAMEVVDPVLGPKLYTIIEKLEASFSPKALEFYKN

EFKFFSEVTEISGTLKPYVKKSKPEKKQVIDSEMAKIKVLPGVYLPSNPDGVVIDIDRKS

GKPLQSHAKAPFMATFTIEKTNEEGDTFTEPLSAIFKVGDDCRQDVLALQLINCCKNIWS

EVGLDVYVFPNRVTATDGGCGVIDVLPNSISRDMLGREAVNGLFEWFVSKFGSEYTPTFE

KARLNFIKSLAGYSVISYILQFKDRHNGNIMYDDYGHVLHIDFGFIFDIVPGGVKFEAVP

FKLTKEMVSVLGGSKNSLGYKLFEKLTIKAFLALRKHYKFIQNTIEPMMDSNLPCFGGKT

IKHLQARLALDKSEADAAVFMKNKITRSYESNFTKGYDEFQKMTNGIPY

>34-9_0560

MLSSSFKCFRANAKRTLTRDSKVHQNLLEDHSYINYKSNIENVHIVKERLGRPLTYAEKI

LYGHLDKPHEQEIIRGSSYLKLRPDRVACQDATAQMAILQFMSAGLPEVAKPTTVHCDHL

IQAQIGGEKDLERAVNLNKEVYDFLSSSTSKYNMGFWGPGSGIIHQIVLENYAFPGALII

GTDSHTPNAGGLGQLAIGVGGADAVDVMSDLPWELKAPKIMGVKLTGKMNGWTSPKDIIL

KLAGMTTVKGGTGKIVEYYGDGIDTFSATGMATICNMGAEIGATTSVFPFNKSMTDYLGA

TGRSEIADFAKLYNKDLLAADEGCEYDETYEINLSELEPYVNGPFTPDLATPISELKKVA

VERDWPLDVKVGLIGSCTNSSYEDMTRSAEIIKDAAAHGLKAKSIYTVTPGSEQIRATIA

RDGQLKTFEEFGGTVLANACGPCIGQWDRKDIKKGDKNTIVSSFNRNFTSRNDGNPATHS

FVASPEITTAFAIAGDLRFNPLTDKLKDANGNEFLLKPPTAFSGLPTKGYDKGENTYQAP

PADRSSVSVKVSPTSDRLQLLKPFKPWDGKDAEKLPILIKSLGKTTTDHISMAGPWLKYR

GHLENISNNYMIGAINAENGKPNTLKNRYTGEYGAVPDVAAAYRDQGIKWVVIGDENFGE

GSSREHAALEPRFLGGFAIITKSFARIHETNLKKQGLLPLNFADNAAYDKIQPEDSVDIL

GLTTFAPGKNLVLKVHPADGSAPWTTELTHTFNAEQIEWFKAGSALNKLAKK

>34-9_0561

MSYHFDTLQLHAGQEPSDNTSRPRAVPIYSTSSYIFNDSKHGAQLFGLETPGYIYSRIMN

PTNDVFEKRIAALEHGAAALAVSSGQAAQTVAILGLAHAGDNIVSTSFLYGGTYNQFKVA

FKRLGIESRFVEGDKPEDFEKLFDENTKAVYIESIGNPKYNVPDFEKIVEVAHKHGIPVV

ADNTFGAGGYFVKPIDYGVDIVTHSATKWIGGHGTTIGGVVIDSGKFPWSKYPNKFPQFS

QPSEGYHGLILNEAFGNLAFIGHARTEILRDLGPALNPFGSFLLLQGLETLSLRSERHAS

NALAVAKYLESSPYVAWVSYPGLESHSHHEVAKKYLSNGFGGVLSFGVKELSTTSEDPFK

ASGPQVVDSFKFISDVANVGDSKTLAIAPWFTTHQQLGDEEKIASGVTKDLIRLSVGTEH

VNDIIGDIEQAFKTVFGDSTPK

>34-9_0562

MEVSSFFKNSIVKNSDYKAMQLQNEPKEQSINTNEFIFNKESVDETFNALVNKTEKEYNS

LINLNSSTNKKVKKPAHEATKKDIYLKYMDFKSQILKKFLEYDSFMEGIMADEDKPTLKE

ETNATTKLVENKEASASKVNSVSAVNSEEEDPTSKHEKTETGTIKHVTEEAKLKFVQVKR

VSPVNLLEIYSIFTDIEVGFKSTKYSEYLKVQKKASANTRKWYRKYFKYFFIVDGILKYL

DPNSKLPIEIIKDFEELMIQKATNPLPVKTGDADESKEASVLPSATTGKSQFSHEMFLTG

TNLIGYSLFVVSVEKAHLIDFVKHLQQIAIRKYGANSVRPELQQSELDELYDYLHGSKKP

EPLMKPSEYGSYNSNW

>34-9_0563

MTLAKRQAVENGINSNPKRKKHKNSKLGCKECKSRRIKCDTNLQLLNTNKYCCLNCLMKW

KRANENNPNQPFNDICSFSLLNPQEILALKKDISAKEFIDYHEMNQQRIYPVNNIQIILS

KEDIEQQSNLDLNEINIKRGFEITMIPHNQIEVPASIKMKRGTETVNQINSRVALAQLCM

SGLNFVDKTTVSYYTSLAVNSFIAKLKKYYVSELKGMDAIFDQQSSFTSNFLFDSSREDY

EKHLNILNSVPTFDEKKSLIIKYIKLSQNFLQEKLSMSYKNIRSSLDVIINKRRLISYNI

LINCHIGSYILKTFELYSLVTDDYAMNRFIEHTCSAYQICWELNSIFQENIQSGNNSSFI

DPSSITNSTYDYDFKNTINCLGGDSISFFALAMTGFNQNFLHMKSIPIKFLDEVLKMLLD

FGDTFILTNSNDVTIIDPLLKEDFKNLALFIEQFLKSSVFMDSTKALPLQPNLLWQLTYK

FFQIKPKKIFSSSLNKDENAKKLLGLLTIDKKDSYYKYSSKNIIDEYTKNGFLTPIEKVL

YSFYIAVDIAFVNILPSRMFVFDAEAYGVCGWNVFTFENIIYDLDLNFDVDKNVVPFANY

IFRISSYMNRRKVLFSGYTCLNNFYSDDSEEKQTKHDPNNFETSEKYLPIPCYASRIAHN

VKEVPISNFLTTTIKKENYFSLDNNYPIPLELLISFKNNHQQKTSMPFKSRFINKKGHYW

DGNFYLIKYEEYTKYQHNLCDLNEKYQRHLNFYFKNLLLFKKLFQSYNDITGTVFPDFNL

YDINILAIENLDSVYKFEEKIINDNFIKSLNTRLSKFKTLISNIYTYQKMHGTENIYMDF

ADEKLRVTDYHLLLNIFLSLDKDQRGSRSDTISIPSSNAVPSVSTNTVKSHLSEKTETES

QMSSTIDIMNDPDGIKKSHHNSMLVSNCGLFEEDYDPFLGCTVAEGINRDKANGDLKEGK

NKENNIPEFHFFMPDFNYIKMIRKDKNTLLFYDGRDEEDK

>34-9_0564

MPPLKALVKLAKPSLSLYPTNNSIVVFGRNSNKFINNLYNNICLNTNAEFFIDRKDLSIN

YLQFNKNSNKLNSSLYLSARFEHFKEDFDVSLKDFLLNKETSKKVSYTLNGVKEINDKNF

DAFFDKFVDILKLRDLLDKDLMLLSNGQMRRSRLAQLLIKIYFSRNKYVINLIDDFVIGL

DQESRARINKAIEIFQQTRINGINATTIVSLRPQDTVPEWCNRLIIINEDLSIEYDGELN

HDLIKTYKKLWDLKDVIDEAEFKLLNDTKNAGINSEGNPVYAKDLIKMHKWYDPIHQKDA

SSVNHIQLKNIKVQYRSVKEPVLNNFDWKIDRKSRWILKGNNGSGKSTITSLLTLDHPQA

WANDIYFNNEKIEVGKIDCEKHNANIACSSPELHNFILSKDLTVKQVILSGLNKDSNNQT

LIPNFVKNEDNQFYHLYDNHLAYWKLKDKEEVKFNLLSLNDQKLVLFIRSLIKLPELLIL

DEAFSGMSMETIKKCHFWLHNYWCGSLILIGHSENELIKGCLIKEL

>34-9_0565

MHNVLVDESKAYVNEKDSVSFTQDKVPLLPPLEITLAEKRELEYGTLSNKKYLVQQKSRN

LQPLKPIKFDPPGILISVITYLNYLIIITLGHLHDYIDYYLNRDKFRGILEQNGIAPWFT

TFESFFVRRMKQRIDDCFSRPTTGVPGRFIRCIERISHNMNEYFTYPGTEQLCLNLSSYN

YLGFAQSEGVCTEYVINNMEKWNVSNNGPRNLLGTTLLHRETEKLVAKFVGKEDCVITSM

GYSTNANFFNSFLNKNCLVISDELNHASIRTGVRLSGASVKPFKHNDMKKLENLLREQIS

EGQPKTHRPWDKILICVEGLFSMEGTMCNLPELVKLKKKYKCYLFVDEAHSIGAMGPTGR

GVCEYFGVDPKDVDVLMGTFTKSFGAAGGYVAADKNLVNRLRLDLTTQNYSEASPTPVLS

QVYSSMKIIMGELNPGDGLERLSRITFNSRYLRLGLQRLGFIVYGIADSPVVPLLLYTPS

KMPAFSRLMLERNIAVVIVAYPATPLTESRVRFCVSASLIKEDIDYVLRHVNEVGDMLFL

KTSSGIGGGSLDGKPPRWNIEDVIAKTPEDCKDDKYFII

>34-9_0566

MSSLLTIGDEAKNAIKKFRLTTNRIDKPEHIILKINGEQELLIDDEENNDEESDDGWSDS

DDEDELEDIKQIRKQLPSNQPRYVVLAYPYKNKIPLILIYYKPDTCRDEETRMLYVNGLE

FISRNNLVSPNKILDLVDKDELKKEFIEQENVPHWFQLTSDAVVEQISKYARKGLTPSQI

GVLLRDAHGVTQSKIVTGNKILRILKSNGLAPEIPEDLYYLIKKAVAVRKHLDRNRKDKD

AKFRLVLIESRIHRLARYYRTVAVLPPNWRYESATASTLVN

>34-9_0567

MSIRVNVDSTLGGVKLILNGYKNLCIQYRRHGNTDFHKEAFESMKKTVKYELDDMLNRNL

FVNSGHVKKTFNMDHFQPKANKNFKRRVLEDSFFNASKNLADDIKVNEEVTLKTENYELI

TDMNMLAHAYENKFNEELIKQIILEKNKRTNVFSAAFNKKPLLKRIKEHLDIAKENSHYV

PKHLEAFFFIYLKFLNRQYQALHHPLLEFDYPKTNTNVIPYKLRKQSSRLMKTIPIQGNP

LELHKLGILRLQHIIDRLQHGPSVRFSSFQSPIKNIHYCIPATDKNKPDDKIFTIYYERM

RYQSHLWEANYRSTLFASLPEIRNAFLPEIGEKVPKDIDYKHLFFDGEFDSTKNENLEIY

NDIKKHQFDERAKFGGFLSPKELTLLYIDAFERDHQNFCYAN

>34-9_0568

MNNYISSDISNIINTNKTYKTLLLDLILHFSDYESNTKDINYISRIINDSFIQDVIVERN

CNENKCGYLLCKEKVHTRRNSTLLKTIDPFDNAKINWTSILNENQNDFLYYNKKFCSKTC

YIKNNYMLIQIKEVDELLINREYSSMINALQDIAEKNENKIELWDDKLNEYDIIKRLNDI

AIKDSEEANNSVYTTPDVSMNEDNDSIIIKENKDVTLFDHDNT

>34-9_0569

MNKRKKNKSGVNGNKDLKSIDIEIENVNIIADTKTNQSYLKNTASLILNGFHRELNTSSE

EVAYTEEEKKLTLLEKYNFKVPLQYESTMMISIVLQLNTLLSNLLMPILNIFLIKKSNDV

SDKYRRLKLHLLNFFKIIIISHIIIKQVDTSRLYHMIKLQSYMKIFALINFLQMIEKLTS

TILVDITTEQKDKQNKDNERHKILKFLIQLPILFVNSLVIIMQALTFNVIINSKGNNKLN

TLLITISFNKLKSSTFKRFNGSSLFQVFLQDSNDRFQLNVALIIVLINNYFHFNKILILI

LVTTITDWIQQIFIVRYNSIDLKVYDAYKQVLVHDYLNKGIIKRFGMPVDGHIVSCIVML

YRKNINIKTWGIFLILFFATKVTCIYILESLLQIYDTEKDHTIDKNVTRFIKGLPKDTDV

SPKAVSKVIRYQMNGKKIF

>34-9_0570

MFMIKSNVLSLVNKQRSSYAIKTYTRQMPKLSTYFTPKRSFFYTPTMLNLQRKSVIPEKA

PFKIDLSDKEYEEIFGDKTQPQLVKGFDAENALRIKKNQRPLDCDKKVAYWLFFVASLVF

SIIIIGGLTRLTESGLSITEWKPITGAIPPLSQADWEEEFNKYKESPEFKQLNSHMNLDD

FKYIFYYEWGHRLLGRTIGLVFVVPMLYLLYSRKHTSRRVNFRLVSMAALIGLQGFIGWW

MVKSGLGEDELIDRKSKPTVSQYRLTTHLTTAFVLYLGLLYTGWGILRENKYLKNPEEAI

KSIKILQDPRLYKIRNVSRLLLVFAFATAMSGGMVAGLDAGLIYNTFPKMGETWFPSSGE

LFNNHYLHKNDDPKSDKIWRNLLENPVTVQLVHRYMAMTTFTMIMAAQWYTIRLRKQGIL

PRSLSKSMHGVAGFAVAQVALGISTLIYIVPIELASLHQGGAIMLLTWCALFNFKLGKPR

YAILRAVKMLMQKKAQVKA

>34-9_0571

MADDPIEKLRIRLINDPQTHAVLKEDFLFSLTQFKALIEFAKVQTNTENSPYFNRSLADV

LKNYEDMAEIAYQYKIHEQYQHSYTYDPESFTTSCMLYIPIRINNVPILAFVDSGAEKSI

LSSNFLEDLKLDKMLDKRFVGQARGVGTSNITGRLHHVMFTFDNEFLPHSLTVLDSLGSA

ILIGLDFMRKYKCIIDFNTNSFKITGLDVELKFLNEGQVEKYSNRVLSEEEFKEILSSDD

FKNKRKIQEDKPVSELKEDLELKKIRTDDSPELNKGKDEPVASFVANEQYIKQLIELGFD

KIKIVEALTLTNNDLDKAAELLLSL

>34-9_0572

MELNRYRTSALYTTPLQFQIVEPRLYRGIFPTKENLPFLKTLKLKTIITILPEEYDIKEE

SPYYQKFIDENNIELRQFSGDIKWKKKKDKKLKNEKKKVNEDVKRRDKTVGIGVDEIYKI

MDIVTDAEKYPIFVHDLTNETVTPLIVAVFRKFAYWNLVSILDEFIKYSGSVNIHERKFI

EDRKR

>34-9_0573

MQNNSNVNHEVVFPSIHELNHQSDQDVNFIFDRISQQSKNNPTKLFLLFTDQVAICISNM

KLFYKETEIYIKRNYSFKEMLEANSIKKHDLTKYYKNCKMSAKMIQILADIEKKYDFEEK

SMEIHSLLQLVLNDREKWSKTMYSMCLKFFKDNQFESQTITISKLKQMNTYEINERDLIN

LLDMPIETVQLQVWNVIKKSSISEDASDQIIVPSLVNWDLEETESWSNVQLELKNGIREF

LYERLERKKNGFSDIYFYIIFLQSYKNQNLNNAREQIFNWTSQIIKDLTNSNFMNDVEKI

NEMNIKLVTTDSTVEFFTSKYGSFDQLLSNKKSLLTRKNSINTSNKLSALMLEDTRPESS

LAKPMIPPPPPPKHSSMDLKPVLLKSKVSTDLKYVIPPIPSAPKLPVSDGKTDSVYLKRR

PPPPPINYQALQQDKYALPDLPKQVEDKNSVRYDNLNDIFKNIEPVVSDRKLSQSLSNQD

FTENPQKNKVQYPELDSVDKAKSLEPSSKLSNWKIFNRPSSSKVDLLDENYHLDHHAKLE

TGLVNLGNSCYLNTVVQCLLSYGNLTDVIIYKNALIKKQVNYESKFGSKGDVINKYIELL

EMIYKQTKMNTLKKNGKPISPMNFKISCGLKNGCFNNFKQQDAQEFLQFLLDILHEDLNN

CDPTLKRLPALTEEQEELRENLPMRLAAAIEWENFFTYNMSIIAKNFMGQYASRLQCLVC

KKTSTTYQTFSVLSLPIPVLNKAKADNISIYDCFLNFLKLETLTPSDFWKCSHCKKKQPS

TKKIIITRFPRNLIIHFKRFDNNLRKSNVLITYPNKLDLNYFYYDNDKNNYQVDEWPEDL

PNRDKELMLQTDKAFQYELKAVAKHQGTLTGGHYTSIVKKNDGNWKLFDDEKIKKVSGES

GYINKDAYMLFYSLKIKE

>34-9_0574

MEDIAQFNISKLPKKISIRPFLKKDIDELLHLESLGFPQEERCSKETVEYRIKNCPDICQ

GLFIKTDEDKNLLIGEIMATKIDTDFITLKSMEIGQHKESSDVIAIHSVVIHPDYQGQNL

ARLLMTDYIQKMSQQAVGKKITIIVHEHLFKFYEKLGFVKLNENQDVNDPNSDFSKNGVW

YNMDKDLFDY

>34-9_0575

MSEEKLTEFQQKNKDVFTSEAQEIWKKETANQEVIPANTAEEQGEDEEPAGIVPTLQNIV

ATVNLDCKLDLKTIALHARNAEYNPKRFAAVIMRIREPKTTALIFASGKMVVTGAKSEQD

SKLASRKYARIIQKIGFDSKFKDFKIQNIVASCDVKFPIRLEGLAFAHGTFSSYEPELFP

GLIYRMVKPKIVLLIFVSGKVVLTGAKKREEIYQSFENIYPVLSEFRKL

>34-9_0576

MNNKGLDVDTLFKIAEEYQLKGVEMALKNDHHVAATLINQSIVVYTFLVDKHDDPKETSF

YLSFEKLMHAYLKLIDILVKYTTNWKLVKKITQKAILKVHELTENSFTDDQKEIWYILHG

FYYYVIPMKMYGKSSNKDFHADDVKLAYKQFEQFLQSSSIATDWKVLFTICQISFYFKLE

DASDLHLSEAFEYLDKNLQSFKEGNPDVFIYVQLCKACYHISKNEIAKMDMVLKENGGKS

IIEKVIDNQQLLMSFYLILLYRELIAGVRSSTTISKIHKLLTSNKKNFVKTTKIFVIKSN

RSIIQCESSLLEYSFLKDLLLFFQGISYLDKGSIDLQREMSEMFFKRIFKGKSSMKWQNS

SLGDMITFYRKWQDEIIFGFEIDEDHPKDFKMEFAYAYMKFIQQETPTINDIDDFLKKHE

SYLKMPKYEDMATNLKMQKHILSVFNDENEEENLRFISELNGNETFEDTKTAIKMIYDKN

KYDLTGGTDQNTNILQQHDAFLKKLKTVSSKFKEDKVSEKSCFLVVIFSILRIDVEDNLN

EKFTKYYDIIKGLLINDMKRIFPLKFWMHIINYTIEIGTRNNESVNKVEELKKLSNTLN

>34-9_0577

MSDTKGRIINCVTRLPYSITKITHHNADDKTNQNVEYKIDSITGNTSLYTSLLNDDVQES

ALFDKTNISEEIILGWTGDIQYFDRYEVKTEFFEPEGADAEDSNESETTFDVNSSTMQRN

LSSVNSNKSKIINEDDNNKLKNIHSYYTLTDPNEQPKESKEDEVNDAFDLSSKEMKYLKT

RLNEEIVKINSNNQTRMEPIFLPTTTSKKNQWLKYADKILYPDFHYILSPFLQDLKFDTT

DVKKEKWIEFVKFNEEYAEKVAEIYQAGDIIIIHDYHLMLLPQMIRMKLNDRDTKKHLTI

GYFHHVTFPSFEYFRSLTYRKELVDGMLGSDVIFFQSPSFSRHFVSCCKRLLDCTWSFKS

PHKDKDCNSEDPDGLYHVSAYGNDVLIKCSPQGINKPLLIKDSFTDSVDNKVQALLKAYE

GKKIIFGRDRMDGCKGILQKLQGFDTLLEMFPEWKEKIVYIQVSTPKIKTAKKQVQGIII

GTEMDDETGVDDDDNEDYELERQIMEMVSEINTKHGSFNYQPVQHYHMKIPKDVYLALLR

VADVFFVGSIRDAINLSGLEYVTVQTASLLKESNTSQLKLKPLILSEFSGTPLTHEDCYI

INPWDSVAVAKTIDKALNEKFILKNKSEVQALFDKIPSLQKWSCDIITTLSSLLTVNNKI

TPLLSVNTLKRLYNKAEKRLFCFDYDGTLTPIVQDPAAAIPTAKLYNILTKLLKDERNEV

WIISGRDQVFLNKWFGDDRFTSSKHNKMGLSAEHGCFLKSPDSTEGWVNLTEKYNLEKWQ

KKCHAIMDFYTHKTPGSFIESKKVALTWHYRRAVPELGDFCAKELKQELETVFAHEEYKN

FELEVMEGKMNVEVRPKFVNKGEIVRNLYNQSKPDFVLCLGDDKTDEDMFKVLNSYGHKE

GIYPVSVGSASKKTVAKAHLSDPSRVLNTLGVLTE

>34-9_0578

MNTIPTVQPLNIQEVHDYKVSLLLNINSLLLNLISINETNSSINNVFLTKLHGNIKYLVM

MSKTQVDTKTLDLSHIPQINANDNKINQTISTLNKYYLLFNKLIELYP

>34-9_0579

MFNPLRLPIWFVRTTYKESLHNRYNFFTLILNFCINFFPVFLWLFLFKQSKNIPTKIRPK

INGQWLFYSDILFLGGDFWNEQENKFVTFLSFLTSWIGVFSGLLILLPYFLFRNKWGTNM

NSSFKTYSFLKPYHLLALFYFSLNILHIFTIQEEYNFKPYKDFITWFSYVLLHLIAPIVT

AVFLYIYKPPGTMGAFALTLGIQNTAGFFTHVCLPSAPPWFTHLYGMHLTEKLTKELNYE

TLGYAAGLTRMPFKLGTNLAAAGFKKSPIVFGAVPSLHGAMALQCGLWMFYKYGENLKYE

YNKSGDGINDNLINNKSKQSLEEYVLDDLDLSEDTNINETELEIPVLPLENKTKWWWHTG

RVIVSLYMLLQWFSTMYLDHHYRIDLFIGGIYAMIAFHIVRKFVMERNYNVYLEIHENND

KEAIKKLDLSSKVWWFNEYGVLARYKLKQIEPIGDREDMSIGERVFKKYKFHKIFKI

>34-9_0580

MQVKFIALTIFIFFTSTFAKSIEELQKKQEDILHYLGGVSPYVPMKNKRFIDMSLPETCK

LNSVQLLQRHGSRYPTKGANKGFKALYDAINPFKGSLNGSLSFFNNKQYKYFVTDADVQL

EQLTKLNNSKPNKYTGEIMAREFGYNFYSKYGHLVSENETLTVFASNSTRVKDTAKFFGE

GFVQNTNINFEVLVISENETQGMNSLTPAKSCDTFDSDYNDDLIATFFGNKTAMLNDIKQ

RIVDENPALNLSISNIESIYNYCTYELNVNGYSNMCNLLTKNELTMFDCEASLEHYYSYM

NGNTNSSAIASVLLNSSIELLTKSNGNKLNLAWTHDTDLNNFFSLALFNSTKLMPIESMD

FDYPFSRNDFTPMGANIILEDYSCGNETYVRYIINDSVYPLNDCNDGPGYGCSLDNFISR

AEKLMNGLDFAKQCNLNSSYPQELTFWWDYKNSTSYPCINQQVKVITQYSTYYTGTLIAY

DEFMNLILAGDVKERKLSSKEEIISSIGGDDKRVMICGFSVSIIVPGV

>34-9_0581

MNNQSITTSSDLIKQLQPILLHPEFNNYDTSALDEDITNTKLLKSKSNDAISLKTSLIIE

YILTYMQKQNMKTSDNEDIDMSTFNTNIIEQTYEDDVNDDESSISNGNDDTFNIVSELDN

DLNNPNKLKKMLILLAKTIKQISEDLENKNNLLLMKTPNTSRHSRLALLDNLNEKDVIME

DSNSYERLISFLISECVVNNINLQSTQNNNENEYTMLENSIKELIQLISNNKASNTVINK

DVVNSTVNDDDLKLKLEDLQLAHDFLSQKFSKERLHYNNDITIKEKQISNVNKQLKEKNL

EIKKLNDIIEDLKSNKQNNKPITPYSEDHENFNYIINSPSSISSNNSESINVLKTEFKSQ

LNKMRDYYESELDKLRN

>34-9_0582

MIFDIDYSIQLILNNSFSNSNDNNTLQKTDILKYFTPDIIHQICEDCIDIFINENNIVDI

EAPVTVCGDIHGQLHDLLNILNITYQDYSIKTLFEQINNTKYLFLGDFVDRGFYSFETIL

FLILLKLKYPDRIYLIRGNHESSNITKIYGFYDEIINKFGTSTPYLNITNLFNHLPISAT

INTSNKDPYFAMHGGISPDLLTLNDFDAIDRIQEPPHKGPMCDLLWSDPEDNINSDYKVS

PRGAGYLFSKRPLKEFNYTNGLSMVLRAHQLVMEGFKMMFDDELCTVWSAANYCYRCGNL

GSTLNIDSSGNYEFSLFEGVDINGTDKLILPVKKLSNSNEYFL

>34-9_0583

MNDSAITLDFSFLGANHQRLDQSKNKSLQSEATVIENRYKSNTLQGFLINHVLNNINPGI

YEVSYIPNISPINILLQNLDVQKTIWISCHKQPINLDSQLEHYVVRTIHELLYLLTKLKD

KDTSKNIVIVTHLSHIINNHLFDLIKHNNANNIKTSINDYKCKFLVLIFSKLSVLFDKTI

LINNFMNAKNSDYDSYVLKSELENGIVGKGVLDQVWKRMIHLKISIYNNVGNTYSMKLVV

EHGANTGVKRIHTLYQYPEKKQRKTAKEVEVEHTNIPAELEQKDSSVLANEYQYFNALAI

DDNYDDSDDMIDDSQY

>34-9_0584

MSNYNKNPQNALPSHEVPEFQGDSVFSNKKATYNKPKTFPAKKSAEIVNSNPSRGKRETH

FNTFSPNHQTQQMSMPSGYRPEGYHQHNPNMPIQTMGYHAQPFSMGYSNGYQYNYNTPNP

ALLPHQMAQMQMPQDRSNVQPVAKIEKPEVPKKLNFNEHISQEVARNISVNFEFTEVFDS

IKKNSEKTIISLGELIKAAEDQLIQENKNIEKQIKSDELGKKIIHLNKQKNDCDSKIKEL

DDELELLNKEEEDFDKLLKEQEELLKTKELELENNLQKVNSNPSISAGDWSSLSSGTLNK

NKGKPVTIVLDSSKTSVASEETKEEVKKPVVKSSKAKQNVKNTKAATVTSENNINLNPAN

VENDTTFLNENENFGKKAGMFLLEYMINPKKIYTFVDEKLKTFTPPKFMNNSNICFMNSI

LQSLLSIPEFLNLLYGLSRSSDYDIEKMPITFYTWNFLYECIKISNNYKPLSSLGSVDES

ANVSLDTSVLFDHLISLDKFKTLKKGRQEDAEEYLTLVLDSLNEEFISLIEKLDFKDVEE

FINSNIQKESEILTYLSNFVNNKNCSWLNEPLLGDSLKNRIEINLKNQSDWEVASTEKQE

IRYTEFYPTPITSIFGCLMMSELKKNKLNCKKGEYPVSITYDPHLVLPLNLQKDDNENSY

SIKGMLEKWASEELLEIDNNEVKKINKFHDLPKVLIIQIKRFNYHVQTGNLSVVDTYKNE

NKVNGKAEKLNDYIDFDFDFDIPASCLQDPSIQKQSYTLKSIVYHSGSDTHSGHYTTDSM

NNKNGWFRCNDIEIQKVTKKDVLELGKKKVNTPYILFYVRD

>34-9_0585

MFLSRYIINYGRIYSKLIKDDYKGINEEITTFLILIGNNDQFIDLYIMAISLLDSKNMFI

YNYKNISQKIIYKDILEQETQAKSIDDFDKNSNIITTDINYNSCSCKEYLQSFDRFVINN

NYTNPVDLKETVSLKKLLTNPEHLMVDLFGLISFEVVKGDETVQYVYDDYSKLVHFIKQF

VDNVIVDINLVYTTGEVMCPHLLSSFLILKNGFIDIKMLDQDTVLLDDSLHENKVLKYYK

EHVKTVYMLDVENLNDWLYLHYNII

>34-9_0586

MLRSTNKYIKYAFKHNKIKSLRLFHSPNVSVRKPITLKEEMQILQFISQKTLDPFENINN

VIDLMKPKDVILSNKVCESAILHVLEKIQGCTDNEIKNKYIKKLSELLLICLRKYYKNDK

YIDGIVKNKTFFITYNMIDQDRIFEIIKVLNTCSIKNDIQTANEILYKTIPLLYKYYYQT

YTWNFKYKDYNGETFDDIYVKLPKTNAKEELRQLIALNRHDNDFDLITYNYRYNNFEQRG

SGKKSIPNIEYEFVKQLPKVEMARKSFVPMKLNKNSDDTYVEFADYSTRTKLNAKFKTFI

SLQLLQSETAAKRYLMYLLNDKGNNELSNNEIFLVLAQLSHHLDRIVNMDGHQYRHKKNS

SVNYLLNDIKINDYPNLAFILKNSKALVKEYILKQLHYKVLKKKMLNDLKIKKYLPKIYQ

NCDLLEDGEIVEAKGEFEVLKLPYRHLTKAQRTNTKHKKNEQLVLLDYYPRILISIVNKD

GNFVYKYNTTFKTGYKRLYKKTKLIKKSKLHYLDHVQQSNLKFNKVFNPNTTKTKIFQEN

IIMSRNLFMTKILLKFLCVDKDGKLLISLDSPEDVNFLNETIALLEMFLGDPIFDDVNHK

YEEKSRLRAKNAMQFEKMQNLLLQFLEKTED

>34-9_0587

MLKTFKPLSSNLRLLNNNFKNINTFRSFSYINQSNSLNKYNPSQYQQISKRNNSNDAEKI

VEETSNKAVEFANTLPDFNSSIEAVTSLTDKSFGYLKSLGMVEHPFYWLPDTIQQILELE

HVFLGMQWGTAIIFTTLLGKLLLLPLTMKQRDVSFRQSKFQRSSEPYADRLYSGDLQNFQ

DMQLKINQLKVKHGIDGQMRYLILPVIANSAFAISMFSGIRQMCNYPVMGMDVGGWSWFT

DLSAVDPFLGLQMISSISIYLYTKYGMMQDMEASSPGQTSMLSSPMMQKFMRFLPFLTIP

FTMSLPSGVVLFITVSALSSVISSRLIKHTATRRLFGFQPFPTKEELAKLVKPKVPGAKP

LGFSDTFKKFNESQRKQRDFMEKYYK

>34-9_0588

MSNPFSKEYLAKRAEVFDKAQKSFHKQAPPLRITKQFKQRSFIEVFRFSLCVLAPILAML

YVGVDTKTKFSDWKMFPEKDLTVNDLPKNTVEIEYELSKLRKERLERRLKLQERLVNEFG

VEDLEAEKQRILGK

>34-9_0589

MDDDYIEIDLNKFKVINSLLPIKPKNTKTLTIVNPSLYNDFFANYFDEIRSKPDVYDFIS

YIPLSIETIEKYIKVSIVLNIVLKKKLKNTNINPNLDFMKQYKTFKYLFINTFPIKKIML

FGIITAHGYMQDVEQVPNFKFIFNENVLCLIKGSKIKMIKELINKNVKLKGEIRFNKEDY

NYYFMIDEIEFINHVQLTIYKNKIVKIVEEMLINRQWDMKETHIDEQLAHILDQWYQFSQ

LSYLPGIDQNCLIHLNKCMIPPVICINKSNSSMYDNLKVAVFFNLLHTFYENITNVDLIN

ELKTNYKICKMIINFIKKEHLHISDPEYRFFFKSLQHRIITDFSNIFVHYLNDGFINGIE

VVELCELMYSKINLYLKYSSNLKITVRLKKFLKDENIMMYLVKHVCYNYLTLKYDKDAVD

NDKCLRMARATKGTLIKCDPSIKQLIIKLNNENNNNIILQAIDNSYNNNEQESILLIDGV

YLDFLKMELDRMLSKNIFEEDDETNV

>34-9_0590

MKEYTVSCHVLNSNSGLPLSNILCELYTITDENPIKIAKQVTNKDGRVIKDTWEFLNDHK

ISQNNTYMIRFQVKTSYYDIVKENTLYPYIDIPFMIKDTNVSHYHIPLLLNNFGYTTYRV

LTMWFTLEQKVELYKISLEHPNWTQDDLASWFQRTYHSLKKPSQTTISRILNNGNNLIVQ

LNAKQNGSKVVKSNNPVKVNKVSKPKKQPKPIIPVNVITSIEEEEDDFDESIPKRQNNRS

LQNQKLRKILQEWIYQTSWNSIPITFPILKDTAASIWQKLPLEKRTGKGFFSYKWLLKFL

EKCQIDPPSQNKSFLELNKPKKIWSYEERDTLKNYLSKIPKKDLFTLDETLLAYNLPLDK

SYYGISKLKRQIEIMTIMFCCNVDASEKLNPLIIGKYENYQCFKSEFGMNKDDFFNDILN

KYDIEYYSNKESFLTSTIFHNWLMNWDDKLKLTNRQIYIILDDCCSHRISNIKLTNITLI

YTRSQNKFLPFNWGIIDEFKTKYRQQTYQALIDLQNTITTLEKSPKKVILTFQQSKINII

NAFKLIKKSWESITEASIMASWKASGTLPKAYINLNKPISMGLKKSDIFINLLNTLQSEL

KTFSKWDAEMLLDLSLEHRILNFVSLQELIDFNTILPWEPIYEEDDYNNVNNFNEIKQIN

FANDALDQEVLEILNLNSNKGFIDFNDLHNGITTNLQKGDLTINYDKENAHTNSSSNYYE

DDIFDEVNDFELEENMKIRSGIPILNNMINTPELLPKDDLFSEILEYNDLDIFRQQLGGI

GGYGAVNEEEISEEKTQAKKLLLMISDIINYQNQPAVLKTLDINLTSSLQLEFSLLYNKI

ISFLNNKNEVELKPNYFENVYVATKPRITLVSFDKQDIKSLVLKINEFINYCKIPEFAQI

YKIGSFKNLEELEMLSMDLVNILK

>34-9_0591

MSDTSSIASNNTTEYTEEEDLPLFKITKVKNTFINKIYASNETITKSLYHNNLVIFATDS

GSLYITNPSKNLILKEFRKLHKDYITSIHVRNDFLISCCIEGIVNIISLKENKITKRYNF

NRSLNYVKLHDFYDDVKNKCFFISSFNGLLVSCNLDTIENIQPQSNWLSYINFGSNLNGN

TDDLKIIELLSIDSILFVEILVKNKMLIVDFNEIFIIEFHKDGTIEVLFKKGFNIKNENI

NIVYQTSDLLVISIDNHQKILVLDLQTYDIIKTVELNMENVICINSFDTLSYSWNKNLMI

PPLLIIFSNMSISIIDINNDVVTIEDDIELPVSKNNKFINGLHTTENTLSILLKNEIFNV

YPFTKKMVFDWYVENEDLIDSWMISKDMPEVADIKRYEIGLNILKDDDDKVIMVLDKNNY

QKLNENGDMFIRMISEIEDKKFESISTKLIEVDTEKHDIKYLLNSGTLFILLSKLLKLNH

NLFYEFLRSFENHINLDTLITLNDLIKNEEEDIHLLKSQLTLSNRVMFSSNEILKIYYNI

LNYNIDIDHYKNEMLKYLKINSNLIKEVYNSHELLSKLETGCTSYREKTVYFTKLINIII

NNVDNEVILQYLLKNNDFVYFDTMLLTRLLLVLENDLFDLSIFEKSIDYYMFDKLYMFEN

NKEVSNTLILFLNKYFNDKYLLSNENKKNAFSYFLNKWSNVEDKDMFLEEVIFLYIQMNE

YNELLTLILNKKDIKECISLIMINYKNVNNVLLWERIFNFVHTKDTDKIILDYLFIMEFV

NKNNSKLHIDVLIFLYEKLLTKVDNFDAKINQSIKFKRLEVQKIKSLLKNELSNVYVKKM

TYLQIAEYHE

>34-9_0592

MADKKTSTQETQQLINNSLSFINKCKKPSNAELFQIIRAVGMGFLVIGLLGYVVNDKPDI

NKINDLLINKNIAFPSNKIKKIIQQSDEEIGKQTSITPAVVSHAMMLFMAKLVVESCETL

LEEEQGNKLDLNILEKGIRRDDEFDFLIGEVDDE

>34-9_0593

MNNDRIRRSNTVNLGTQNNQKSVHLNPQRMAHNSSPQYIKNPYAQDNNASRSNHEQNFKT

HSPEIKRHNTSPNLRINQPVIRSNTIAQNPNNMKKYDININIKDFDIKSMNNQNVLTTYA

QQQQYNQNYANMFDNNLKSFIFYGPNKQKNLIYVGQNPVLKNYFKLRRLKYDELVENLNK

GDPLGFNTNIKTNDSIKREDDNDGFIKVNVSGNIRIDNPDMYKQRVQQENAIPEKNHITQ

DKKVSLKKKMFGLFKSKPLVDEKRNDVNTLKVDTARNNSTIDKNNGKSGKVSNQNGGIQH

QMNNGTDQNLSYKNVPPQMHNKSLPQQPIYNRPAQQQPMPIRTAQQQPMPIRPAQQQPMH

IRTAQQQPMPNRRIKHPLQMQPNVSTESNSRSTNPKNTGRYQQADNNYRNMQPKNFNSNS

NNQKRIFQTPTINTSYQLNENRNSLKDVDMLLDVALKTYDVSDVNNKLVKSNLSPNTSLS

TSPTKSDSWSNLLNAQLMLQSESYDDDFDFLELYSPVDIKKNGNQSRRISICSTNSENSI

ISKNSYRNLSFTNLEKLKVLVKMNEDGYSNRNEENKSVQFEDPVLDRLFESGFKEIKVFD

KVRQKWVIYKDTNKAKDRDIPHSSKIKFSQSIEVFPLPPLVQDIDSDTDDSVDSLDSDNF

SDLSEEDLMKLKSMKELQMFQEIKTEVNNYKSQEMLVHKDSILNTHFFI

>34-9_0594

MSFLESFKNTVVNSTSPVVSSTYILFKISPIIIYLLAYNFASFTNITILLIIIKSMEFYA

VQNIFGRKLVGLRWSYDKDFKYETFKQYGLEEFGNSFDKIIFWYGMYLTILLWFVFSITT

LFGFKFIYFFIVLYCLFLEVYQYYGFRGSYYHKDETNNENGQGVNIMDVLSRYNNLTSFF

QTSSS

>34-9_0595

MFKVEGWNVNSNSIAKDASNNKQKKSKNNKKVHEQKQKEKFRNRVDNNSYDNVNRNEKEV

TKALNNTIKEDKKVSKKSDSTAKKEKKLVQQSTSAIDIASASNTKLTPLQQKLFNKLKGS

RFRWINEQLYTNTSSTALDLITKQPELFDEYHKGFREQVTTWPINPLDIMIKNIKTRFQS

RNLNAPGGLPAIDRTCVVADMGCGEAALSLEINQYYDQHKKKFEKQNKHLKVHSFDLAKH

NDRITVADIKNVPMENEECCIAIFSLSLMGVNFLDFIKEAFRILKPNGELWIAEIKSRFK

DQSLNEFIKIVEQLGFRHKLTKTLEEEGDDKEMFIRFEFFKPDERTVMEMKERQAKRTKF

LDSDRNEDEDVEARREKQPESEWLLKPCIYKRR

>34-9_0596

MDVQNKKFINNLASNDLKTRRKSINSIQERLKITKKKADYDRIWKGLYYAMWFSDRPLTQ

QKLADELADVFLIEEIFGKNDKNFEKFIKFSKSFWKIIALEWLNIDYHRINKFLLLLRRV

FNRQLEVVVKYNDITKTQEYCDRVLSKFVFSGDRRIYNGIPLHIIDILKDELVKVLNKLN

KKEEELDSEEIAKLIKESNVDPIFDMILNLVNNYSINKDYRAYIKDNLVNDEDFIEYGII

DRCYKPKYISKKDLEKEEEEEKDEEVEEWKGF

>34-9_0597

MNRYKKNRLEQYLESDGSTNIKKEARKSDYELKQASKRNNLDSIDIDKLNTINSKNAEKE

ARLSRYEILERVLLQIPLTKKLQYSDINTIRSIKDKPIYTTTSFMKKYSEKMKTMDMESI

EQNFVYDPKSKLVKDETQGYYNEKNGKFYRYLDTEGKELKELEDNVRRENIKKEIELKNK

IRKDKQNGKKLNRQEYESLMDKRSQVLFANPTLMAKQQKKTNFEAVKNTKRNLSDMKAKS

NTGKKMNMNLLSKYK

>34-9_0598

MSYRSIDTKHNSSKTLGFDELIMKDDLSEIPEEFFTDEATYINIIFNILNLIYATNPNFI

DKTHLSFIPIHLIFDRIYYQYSNLLSNPNIDRFVIKNYKKVEEALDFYLVNYCSFEQLKT

HEINLTFYKQSGIPMNIYTPNLMSKCIDEDKEDNGLYIIQHHIGGIRNSQCYITNDLSFK

ADSILKEVNQNNIKRKRFLYLDYANEIYWMLNIDDMLNDNAIISFLPLSLIIKENNRHSN

KITKKKKTNDMFLTNKILNFFFYENFQIHNMNNVTLYKYINENQMLMPSSDDTEDEFFID

RIIKTGGFNETYKDIHKLPKQTNFEDTTVKQLTEDVIISKSPLSSSPGLISKSPASSMGS

NLNSLGKSVSFKKKQLSRASTFENDFHNNNFHNNYDSIPNVYSNMSRSTSHYTYEETNLQ

SKKSFKKNFLLVISPVSKILIIIVLLEISYSGIFISFYIVHILQFVVVDIHQAGNNFIVI

GCIIGICTCI

>34-9_0599

MAINSAASTACGLIGTVCWCIQIVPQIIYIYKKKDTTGFNPIFMGLWLTATPFLATYAFV

SNQNIAIRIQPNVFMVFCLAAYYMSCLYPPHKKSKKFIYGSIAGFLLYFAIIEAGFIPWL

KPLYAKGTTWPALIFGICGGVLLAVGILPLYYELYKRQGQVVGVSFIFLFIDSLGAYLSI

LSVVLGTMDVMGIVMYSLIAVLEIGVFLSHFIWYMRFKVLKISKLPDSENPKLDSFEEED

TDQTCAKQDIKSTFEDRSTRGNISRTRVAIMNGNKLGKEYFELVRTAIKDDKQSETSTSS

RPLKRRKTRQSLKESSTATPSITKAGIDYNTLIDLTENTTGEVDLAEAQRLNSLKTKREA

QKHFASLKKHEIIKISSSEEEQDEYIPEDDEYIPEDDYEDDEFEDVDLDNFQDDENVDDG

FTFRINTDKNKNNTKKKNMITPELRDKRNTYYKTYILVNLALTGLKNKWCNNSELLKKLD

PLLPSDIFNALHPPKDLQLPVKSTNKLIFGLKEAIKFWNKYFIINEDRDIGYYMRDWNEL

EAKELYPVKDKQIITEQKFVDCVLEERYGSREISIMGFVSLLRSVGMNCRLIYNMQPPDI

TDIGRLPKEKVDKIDYADFNDCESIHFPIFWCEVWDKYTKKWLTCDPVFFKTVERHVSNA

NTLSKLCPRNAKEEQRLITRFVVSFNKTLKLRDVTPRYTNKYFSKVYSKKFKNDKDLEDW

YVNLLTNMSCKFNKENGTKSLKIDEYEDKYMEEKLSIESFPDSIKDFQNHPKFILSSKIP

ITKMFLNPEAKHVGFFKNDKVYLRDELQLLKSKNKWLREGRSVLESAKPRKVVKNHMLSK

RGNTNSNENVETELFTEDQTEIFKPSKPLADGTIITNKFGNIEVYHTNMIPVDCVLIEHP

LVIKAADFLKIKYAKAVVGWDFSTANKARSKGDNSKATAKFGGIVCLQKFEDAVWSIIEE

LESINKEKKKLDRQKQNMRNWVELFKRLKIKERLDGDINASGWLTHAVKEPDNFDEEAGN

IANNSELSDGAEESEEIAGGFEASDTGNESEEIAGGFEISEPESANEESKNSSELNFDDF

MNEMVNVMGYLDTLKKLNKPSQKKPALSRSLTDTEDEDDRIRLAKLAKSQIDPLDIKTNL

LPEDYQPVIPQHIIKRNLERKSKNLNKKNSSLKNSGNSSMNKDESSIVKQRLISKYKSKL

PTKAAPVIEKKDTTPEKKMSFKEMMRLAEGNKKHEVKPVEKKVTKEIVNKVDKKNRFLNR

EKINEQKRLEVYKKQEMMLSKSKSPKPVKHKTSEKEEEEYNYTPTGFDMLMEEELEAEEI

ARREDIKEAKLLKKRAMEKERLKNRY

>34-9_0600

MSDKSTRIAIVSDDKCKPKKCRQECRKACPVVKTGKLCIEVGPTSKIAFISETLCIGCGI

CVKKCPFDAINIIKLPTNLDAETTHRYSANSFKLHRLPTPRPGQVLGLVGTNGIGKSTAL

KILAGKQMPNLGKYDAPPSWEDIIKYFRGSELQNYFTKMLDSDIKAVIKPQYVDNLPRAI

RGPVQKVGDLLKLRIDDKGEDYNNMLIKDLQLYDVLDREIEKLSGGELQRFAIAMACVQT

ADVYMFDEPSSYLDVKQRLKAATMIRSLLEPTTYVIAVEHDLSVLDYLSDFVCIIYGIPS

VYGVVTMPSSVREGINIFLDGHIPSENVRFREDSLQFRLADSLIDNLKTEGTAQFQYPEM

TKTQGDFKLTVEAGDFNDSEILVLMGENGTGKTTLIKLLAGVSKSDKNETEQLSLNVSMK

PQKIAPRFPGTVRQLFFKKIRGAFLSPQFQTDVVKPLKIDNIIDNEVQTLSGGELQRVAI

ILSLGINAQIYLIDEPSAYLDSEQRVICAKVIRRFVLHNKKTAFVVEHDFIMATYLADRV

IVFEGTPAKAATAKTPESLVTGCNRFLKSLNVTFRRDPNSFRPRINKLDSQMDQEQKASG

NYFFLEGGV

>34-9_0601

MDTTDWEHFIPSPYIDKDINAINPKFLTFLQLCTVEIILSTLYVKDFINVFFKVGKQFEL

DQDIYKNKSKVLIFFKYSKNWILSSVVISLAFIYWKLYSLTDEVSFLISAMYIIGPDFII

QFSEFFKTPATATSTLIFHLINIGINLIRCVQYVVFEGKYSYLGNSQYPAVMILPLILHA

YLFVYNISYYIPESTIIDHFDVLKKDFKIFPPVNFLEDITFTWMNPIINDVYKKGGIDNP

YTMDLPPSNLNVDKAYGTFAIQWEKGQEGKSKSQLSTQYLLLTLFKIHYKLIIIAIAYNT

IADYTRVLQPMVIRKILMFLELSEKDLTDQPRIHLLSLGLYVCFLNILNSVLNNQFFIKI

FEVGLQVKSTVVLAVYKKSLKLSYKSRSTKKTGDILNLISVDALKIQRFFEQSQPLIGLP

VIIISTLLSLYQILGLAVLPGLIIMTILIPINSKLSARLSGLFKKNMIFKDMRTKLITEL

LKNIKIVKIYNWQKPLIDRLTAVREHDELGNYAYLNYFNSLITFSWSMVPLLVSCSSFLS

YSMITHKPLTANIIFTSVSLFNLLQECGQQLPSLITLLIESKVSLKRVQDFLYFEEADES

FIHYEVRNNLKPLIEVQNCDFLWESVSKETTIDNENDIENGLIKNSVAKVALKNINFKAS

TGKLISIIGRSGFSGKSTLLKSLLGILPVKASDPSKEAKVIYRTAKNEYESIAYVSQHPW

LMNASIKDNIIFGKKYDHALYEKVISIGQILPDLKIFADGDQTIVGGQGVSCSFGQLVRI

NICRALYSQAEIYVFDDILSALDASVGQAIIDNVLKDFLKDKCVIFATNSMKVMKYTDVI

YYLEDCSISRVTTYDEIMAAETSDKKLLSIKNLILEHDKTSDEKNNEHVSETESEEIKVY

DKEHVVVNKPEESMLESDDEGLENDVVRINSIRRRLSNASLHAKRPLIQTINENLKTKQN

IEKIGKGKIKYEIYLTYLKNCEWKNVFFFVLFSVLLQMTDVLQKLWLKYWSDVGEDSVIR

LNHYVMVYFILGLLGAFMESIRAVSLNVWCFLTAGGNLHRKMLKSVIDSPMSFFETTKAS

IILNRFTSDVSTLDEGLEWCVSSTLRNTMLYVCCVIIISISIPIWLLVNTCLVILYTYYQ

LRYVVLSRDLRRFWAISFSPVIASLEELINGVDVIKNYQQVERFNFFNSENMQFNVDIMW

SFRSTNRWVAIRLQSGSALVVLFITIFAIVKKDAKASLIAFLMNYAVQISNALMWIIRAS

VAIETNSVSIERIIEFCELPSEKPFELPVDKTLPKNWPEHGEIEFENYSTTYRENLDPSL

KYLNLKIKSGEKIAICGRSGSGKSTLTLSLFRILEPLTGTIKIDGVDVSKIGLSALRSGI

SIIPQTGDGQVFEGSLRYNLDPFNLYPNEKLIKALKLSHLQPHMEKLCREERFYADQSPE

NEPLTLQSPITDEDLLNVKIADNGANLSVGTKQLLCLARALLSDNSILILDEASAALDEV

TDFLIQQTIREEFKNKTVITIAHRLNTILSDSDKVLVLDRGELQEFDSPKALIDKKEGLF

YDLCKKGGYI

>34-9_0602

MGAVISIPINAAISFSSSFLGASASSMVKSALSNIETSSLGARILYAVGLLVNSIISWIS

LSANHTLWNPLNNCTSGIECGVSTAYRLSFTLGLYHILLMVVLLGVPDGNFKVLTKIQNS

YWGSKIFLYFVLLFISFKWFSNEFFTWFSKFISLPSGSIFVFIGLVLLIDFAHEYTETCL

DHIKEETENMIVEGEAEESMTLKFWRRLLIGGTIAMYASTLIMIVIEFILFCKNHCGMNI

FAWVLNILFLVATSVMSIHPVIQDYNPKSGLSQASVVGIYSTYLVFSAMAGEPDDKNCNP

LVRSTGTRRASIILGSIFTITAIVYTTLRAAGNSIFHITSQEGNQDIFLDENTYGDMSIE

DRRELRKKAIQSAIDEGSLPESAMSEYILEEEHNQAIRSENEAMKPNYNYLLFHVIFFLA

TQWISMLLTINVKQLDNGDFIPVGRTYFYSWVKIVSSWLCYILYGWSLLAPCVMEDKFDY

NF

>34-9_0603

MSATTLGTEQNKTSIIPVDELQNHGINVSDITKLKSAGIFSIQSVLSTTKRNLVKIKGLS

EAKVDKIKESAGKIISIGFIPASIQLQVRANVYAITTGSKNLDTMLGGGIMTMSLTEVFG

EFRCGKTQLSHTLCITTQLPVEQGGCAKKVAYIDTEGTFRPERIKEIAYRFDLDPEVALE

NVMYARALNSEHQMELLEQLSEELATGSYGLVIVDSILANFRVDYSGRGELSERQQRLNI

FLNKLCRISEEFNCAVFLTNQVQSDPGANSLFGPTSKPVGGHVLAHASATRIMLRKGRGD

ERVAKLQDSPDMPEGECVYVIKAKGICDPED

>34-9_0604

MSVAISENDTNILLTTKNVLDEPVLDKYQLAGKITQTTLQYITSLINSCYHDSNKDNNQL

FSISQLCFLSDLFMNQLLAKDFKGKTLEKGIAFPCSIDIDEIANGWSPELDDPTFIREKN

IDANEANTPSGAKASIKNFLSIGDLIKITLGCQIDGYTSQITHTLCVYPTEVNPESGLSQ

ATGPLLGAKADAAAVAQIAKDVIVTLLASAASPEKLPEHLKAVNPKGEAKVTGHLIRKVV

DHLIESYNCALVPGSKIRRVRRFLAGQNEGVVAEREFKGCVWYESNQENFLLAKSQQYQN

DLNNTESSDALTLLQNSENTHEDKQKSAFIYDSAIASDNFVVVPGEVYLVDLKVVSLSEA

TELGLVTVETLTEFSGSTSTGRNMTVRNSQFIRDYTQMKDLKLRNSRECLSKMDKQSVYP

VKLTYLSDEYKKLVQSPIELNFQNIDYIESQNVIIKENLKSRMGMNEIMDNFLATSKPVQ

IVKYIPWEVIVNVANPDGVKSIDAVNPTLPGYEIPLPKLNISALKLKSLLKNSRTITLPV

AREACTVAITDALTYKLTHAEPIYLQSKYELNPNSPLTQGIYQLNELSKDSRFGIKIRDV

KPLSEKAVRSSVPDLSE

>34-9_0605

MLSNIAKKNLTVFKQQTLLKPATAALRFNSTDAEELIDIALPESSFEGYLLENTPELNFQ

TTKGNLLQMYKDMIIVRRMEMACDALYKAKKIRGFCHLSVGQEAIAVGMEYAISKKDTII

TSYRCHGFTYMRGASVQAVLAELMGRRTGVSFGKGGSMHLYAPNFFGGNGIVGAQVPLGA

GLAFAHKYKSEDACSFALYGDGASNQGQVFESFNMAKLWNLPSIFICENNKYGMGTAASR

SSAMTEYFKRGQYIPGLKVNGMDILATYQATKFARDWAVSGNGPLVVEFETYRYGGHSMS

DPGTTYRTRDEVQHMRSKNDPIAGLKMKLLELGVASEEEIKSYDKAARKYVDEQVELADA

APAPEAKLSILFEDVYIPGTETPTLRGRIPEDTWDFKKGGFANRA

>34-9_0606

MSNKYNQINTSQKDILKKHLVSSYQLENNLNNFLTRHENSGSSSDFGKTVETQSQSNYEN

NQKSNFNFSSPIKKLKNLRNKSSAATSTSSKNKSSLGSRTTSSDKLTGLDDTMLFGLKSN

EVYSANNNVKSDIPIRTTSLSAFSNESGYNLFNNTSSLKPLVTTENPKNVNINFSEALKV

NTTVLVNKHPKNNLENKSELSSAYDLNKTPLQNNFNNGLNINDPPYFTPPKIRQLNSSKT

TKRFNLMKCCICEESVKEKTSEEKILTLRCDHVCHDSCLYLHMLMEKEKSHQLKAEMLFP

SCELCNDGITKCIPKDDASRDQLHLKILISDANEDITQINTTNNTESSMDSEKEKTLQLL

SKNIDYLDGLPLPNFTIQESAEITKPVKNELFHMTPPNQIISVFPNNDMSNLQTKSSLPT

PEIPSTIGVNAIKGEFMETQGTLKKQSILKKNLNSNKISNGSPNIHKLTRNRTSIYNQPS

ISRTSTLSKKTASINSPKPIVKKINSSLPTAAIFSSINTSVDSKPKNKPKSLILHKSFNE

DSDSDDELMFVQIEGDHQVTTKSTFIKKHSHSNSSDIQKVKSWGKFSDSVSTQTSENLIN

VVKDRLNLVHELIEKHSDQLNKKNIDSDLGLLRISNVFEVCKVLENKSKTTFYLCKCYLF

EKMLILDFFDKRIPKEFQMIKITSESINVDAVNRYVFRVSCLSSTDINIFQFRSLDKKDS

KVLEKWISALLNFNLEFYDKPFPVNNENKKFASTFNTTDKNEENNDNLLFQDFIIKKSNN

EVSTTLPIKSYSEAEDLILILQLDSEKKIKISENFNLVNSIKSLNDYFTKKQKTLKFVIL

DQKLKVLCVGSSKDVLNQLNGRMKFEQIRGKKFTESFWSNSVLQKYFMSTEDSKLNIVVM

SNTEMNVEQNCLFKDFYASSFEDVLKVHVGFLNIDYSEEINDLVEINTWFDLMEILCFSL

NLEFGQDDLDNDEEIFEVDKKNCSSAASSDYTVMTPLTPLDFDKEDDSSFIKEHLISNNK

TKSFFASEALYNYF

>34-9_0607

MSEFNRENYVFLAKLAEQAERYEEMVENMKKVASASTEGKELTVEERNLLSVAYKNVIGA

RRASWRIVSSIEQKEEQKLEQFLQQQKDLDGSNNAASTEEDKQQIALISKYRNGIEVELA

KICDDILNVLDSHLIPSASSGESKVFYYKMKGDYHRYLAEFSSGDVKNKTTDDSLKAYER

ASEIATAQLPPTHPIRLGLALNFSVFYYEIQNSPDKACHLAKQAFDDAIAELDTLSEESY

KDSTLIMQLLKDNLTLWTSDLADDVDDE

>34-9_0608

MYSKAVTEFANKSFSGQRYDNARPKYPPELYSKIKKSYNEYVQDQADRGITEPKIPIFID

LGCGPGTVTYDLSEHVSSNALVFGLDPSMKMIEAANEKSHMNHKTVKFDISDENSFQNYF

SSKYGNSKILNNTKVNLITAAQCSHWFNFPNFLKNSYEVLKEGNTGGKLFIFGYIQPVIW

NIPELDDIIQKLDNDLEQGFGKYWEQPGRDYLNNLLTDEYFTKALEESSFENIKVERFYT

SKNRDPFVDDGSHSYELKKTTTMRHFRNYVTSWSAYNKCKREKGEKYADGIIDQRFNEMF

QKVSTLSYDSKIDLVWATYIIEATCST

>34-9_0609

MPLIEIKSNQQFSELSSANPKTLKTLFFYADWANNCEQASQGFKQISEEYPDNEFFSIDA

EENPEISEIFDISAVPYFIFIKESSIIKELSSADPQEFQNEYKKIVSASTVADNKSVSKS

PENEEEEEEETEEQLNERLKQLTAAAPVMLFMKGTPSEPKCGFSRQMVGILREHQVRFGF

FDILKDDTVRQGLKKYSDWPTFPQLYINGELVGGLDIIKESLEEDPEFFTQSLAN

>34-9_0610

MSHEKSRLPLYMKYAPNTLDELFLHPKKIKDMRNILTNELFDSNHNLNMLIVSGPTGSCK

TSLIKLLINEYYDNNKFLYQTFDIQNVQKNDHFIEFDPLDKSINFEMFLNNCKLFKKNSK

MMKVVLIKYLPNIYFDQIHSQFLKAIKDYLNNDYYNEFPPLIFVISECDIPKDDTNDVSD

DLYTRFDIHSHYITETIFDKEILQNYRVKKITVNPVAKTYLKKILKRVIEKEIIQMNTSN

TLNRKIKISAFSGIIDELSNLKDVPSALSQLESYLQAFENKIDFKSLKIDNRDTGISLFH

AVGKIFFGTKEEGYTNDDIIKLLNKDYQQYIDPVFKYTIFENFKVAEIKGLNNFTNMVDV

MSETDIMNSSLGTEAYIRKIRYLCSDLKEDLQKAEVKANSIKFTPNFKILRKQREVRYQI

KDLQLLKIAKNNEFVDINDLLLYGNELESKLLTHYYREYQAWKEYRINVGKLTDGYAKPG

RLIDPKIDYMDIIGGKFGTINSIADMDINTDIGIYSNSKYREFLKQTKNDKTNMDVKKEV

DIPYDENLDDFDIESSSDESMSGSSSDEELFLLASQQIEKEKNFMSQSRTPSPLKKSIPI

SEDSDDSDEELFQLTSQHLNKIQKK

>34-9_0611

MSHKTSLLYSPSDEYNTQIYELLLHFLTTNDDIFNTVDNNSTQEILDKIINIILNDKIVD

KLKYLDQDLNIQFDPSDKENFDKYNRLYQICNNITDFKKKKTILSSNYDDERGDFSDVDI

IDDDLQDRFIDIPEKHEEDENVLENDIIDTSIISLPETIDKINENNVRAFISSLFKLDTN

DFEKSKAFGDKVIELIMSANDTDDLAKKLHSFINITKMRLIPKLLNYKDIIYYGLRYYRS

LNDEDKLNVIDEIKEDSLLHILPNSYTVSKRKLSESETLHTNTTIKKHKKNNNLLNINKF

KLPLDDSELMEKQDIKLPENSYKIKKDGYTELHIPAPNKIDEAFRLIEIKDLPEWSQKVF

PSKETSSLNRIQSEVFPTVFHTNKNVLVCAPTGAGKTNIAMLSVLQTISKHISNEKKGII

DTNDTSFKMVYIAPLRALVQEQVLEFQRRLTVLGIKVVELTGDSDTSRRELETAHLIIST

PEKWDIISRKFHNRNLIQKVELMILDEVHLLHEERGPIIESISMRMLNNNSRIVALSATL

PNYQDVADFLKVDKTGLFFFTPNFRPCPLKQEFISVNSFSSVKELININKACYDKVKESL

LNEHQVIVFVHSRKDTLKTAQYLLQQMAKDDEMKAFLNPEDPSIKNILSKEAKENCDDPQ

LANMIKNGIALHHAGLTRNDRSQSEDLFADGVIKLLVSTKTISWGVNLPAHTVIIKGTDI

YSPALNKLVKLSIQDMLQMLGRAGRPRYDTYGEGIIITNKEGVNHYLSLLMDQQNIESRF

IDKIIDLTNAEICAGNITNKVNLISWIKSSYWFIRMMKNRKLYKTEGLNIEVFLSSLSDT

IVDALMNTNMLAFHDENIFKSTELANVASDFYIPYTSIYEYYNNLNEKMTLIEILSLFSK

SKEFENMIVRPEEKYEMKILLNKVPIPVKESLDDIGCKPNVLLQSFISRLHMDGLSLNGD

MVFIKQNSTRLIQAIFQISKMKKLSRVSDLILNISLYIEKRVWITETPLRQLNLKPDILK

QIERSYIEWEHILQKEGDDVLDTFYEMAPHLKQYESLLDESLIKFPRLTDIKVSVQTITK

TFYRINVLFTPDFKWDHKIHYSYINFFLFLNDYNGDKVLYEDNFRVRKYHLQNEVSLSFD

IELLYPNLPPNLFVSFKAEKWLHCEYNVPVVLMNKIKQIESSLPQPTEVDSEYDTKKKTF

FVKEVEKLNIPEFSTIVKNEVPFIENSNRKYCNLFEIDALQIILKTDENILYSSTIMESK

TMLIKMAFLKAYEKGISRIVYVNDDNFILNELANWLDKHYPHNNNELKISKLGDDLDNKF

LFNRSHITLSSFKNFELITREWKNLPNMKDIGYFIVDNIETVFDANDMDRGYLFEELISR

MSLLQTQFDDRNTRFLCFSDPLSNYYEVGEWIGIERDNCFCYNNNLFIKEMDIKLEFLKY

TNGNVVDLMTDHALVKLLTDKSFSSKSKKKADVIYTTSRNESLSIAESVLAKSSTLKTNV

DFEKISGVSDELLGMFTDTEISRYFSYGIFIYYESMDTDDQMILKKFAKACKLYITDGSF

IFKANSALILKTVKVIESENSLTNQSTKPHLLTKILSSCERDAKLTIMTNEFFKIQKILT

SSQYSVESSLPYHFAETLINEIKSGVVSKKSDVLEWLPYTFLNSRLHKNPSYYGVSDNSV

LKLSKFITTLLNQSLSELEDMEILEYEENEELFKLSDAADGVYQYGLSFDDLQILKDNFF

AIKTDIQLLQLVTSLPSLEKDLNICYQNSFQQAEKHIGSFKALKQYRDKMPPLSFKIFGL

LYFLLFDIDISVLPLTLQLDAKAVYKNLLTLSFKIFKFLVVLIQTDQQKVKFKVLKAVCN

LAKGLHLEIGTFKKETNDLDSLKQIPYIDDDKINNLAAEGVLTVENIIKNGLQSKVNDEE

DEDIDDFFAKFPLATIKDISLNSENEYVIEISDFVSATEEDGINYTGDYYCVFYTEGEED

IYHAEIIKQVANKPNCIGRINK

>34-9_0612

MLFDIDEIPILFPYDKVYPEQYEYMRDIKRVLDNSDVNGNIQLNNGQNAAANSCILEMPS

GTGKTVSLLSITIAYQRYHFKQTGIKKKIIYCSRTMSEIEKTLLELENLIEYRNKMLPPD

EADENILGLGLTSRKNLCINEEVNSTKSGKLVDEKCRLKTNGELKTSLQDEAESKKINNS

LCEFHENMYQYDVHNYIPKGVYSFDKLINVCKEKKICPYFTSRRMIDQCDIIIYSYHYLL

DPKINSRVNKMLNLEDTANTIVIFDEAHNIDNVCIESLSIDLNNDILKRGNKSCNLLLKK

IDNMRQVDAERLETEYNNLLQGLNAANPQKATEEDDVVPNPVLSTDILKENVPGNIRKAE

HFVRFLKRFIEYLRTRLKVMHVISETPISFLKHCRQLTYIEQKPLQFCVDRLALLLRTLN

IQDNNHSAIEDIQCLKDIVSFATLVATYDTGEFQLIIEPFELENQQNIPNPILHFICLDP

SITMKPVFEKFHTIIITSGTISPLDMYPKILKFDPIISKSYKMSLPRQTFLPLVISKSTD

QQPLTTRFEIRNDPTIVRNYGDILVRLSQTIPDGLIVFFPSYLHMENLITKWQQQGVLDK

IWPHKIILTETPDAQESSVALEIFKKACDNGKGALLFAVARGKVSEGIDFSNHYARAVIL

IGIPFQYTESKILKARLEYLRNNHLIRENDWLSFDAMRHASQCLGRVIRGKLDYGVMILC

DRRFTKRVGQLPAWIRQGMEACNSEQDVDLEVGLNRCKTFLKSMAQKDVKTNTKDLSVWD

ENDVKAFKAENEKPIDYSKEGGFLLQ

>34-9_0613

MGPKKQRTLMSFFSKKKDPSVATVANSEQSNAPSELSEIEKNMVSSTNTTVVSSEKEESK

TPAIDNEDEDKVNTKTRKVKRALIVDDEDEEHEIVPAKKPKKELIMNETVKSDKSLDKTK

HVKSLAPPGIKKMVIPEKYDFLKNYTAGDKKVHIPAGSFSKFTDFEKQYWEIKKDLMDVI

LFFKKGKFYEVYENDAILANKLFDWKIAGQSNSNSLINSTANSKGRAGMLMGGVPEMSLD

YWINQFVSNGYKVGKVEQLESLLLKNITKANSKKVVQRELECIFTKASMDSIDDSLDNYI

LALTQTTEGTFSCHMVDLSVNKYFYQEVNDCKDLETFLLRSNPKELILYETEEFDEKIDR

ILKFYAKQAQWSKMEETSQRDSKTLLLKYLEYLKIEVPQDVNFLQFFTNDKDAHITHMKL

ESDTLRNLELLKSSIQTKTKGATLFSLINYTQTGMGERLLKKWLLKPLYQPKDIKMRQDS

IEFLLDESSNDCLSLIHDCLKKIPDLERLFGRLLVKKNLPFNVFHENVVLKFESIKQLVM

LLKASIEDHKGDNLFFQVVNQIDIDELDTVLKEWESQYRYTKVPTDNQVFKLELDEGIDE

EYDDLNQQVLDLERTLDDLLKEYQTRFSSRKICYKDQGKEILTLECPMEIVKKIPKNWTQ

TSATKYVKRYYSPELQKLANRMLELKELQKESQKKIIKTLYLKFLKHESLWKSTIDSVSV

IDCLYSLSVSSKNLGYPSCKPTIEFSSENFIEFEELRHPYTTATRKSFIPNDISLNINNE

KKMTILTGSNSSGKSTVLRMASIAVIMGQLGMYVPCKTAVFTPFSQILTRLGSNDNLLQA

KSTYLMEIEDVHELLKNVDKTTFVIIDELGRGGSSKDGFALCEGVIYELLTKSKCLGYLA

THYNGLYKSFDLNNTNFKKMDYIIDDNGNMVFLYKCVDGISESSMGLKVAKMCGISAEIL

QEGEKMAKDLEHTSRLMKIKQYEDNNNDGMISFPGLESDFLYMLDNRDLVTFGRDRGERE

ELEALTKLLSNIH

>34-9_0614

MSDNNKNSKPRKDSLDNIIPSMDKLQNDNNFDEQLLLANTLTSLSRGSSPVSHNQRSSHS

PLAGLLNNKPTNNDKFNSPLPSISTLSAAAIISNANNTSLTQNNRNNSIMSTTSSVTSNN

SMLFPPPSPNLLFFNPTNKDNFAQNNGSIPGPLGNVFPTSFNTQKKDDIKKIPVSNSTQM

LSLNNPISSTISTPINSNLFFNKNLSADTPPLGTLHNIKYSNVLKKGDRVSKAAKDSNRN

QQINSNDFIDEPIMSNNKSKKNNYYSNFNNNIAQLPGSGISYSPGPQPNNSFYNNPSVLT

NNLYSLSGPQSNSNLTFNSLIGSNNNVDNNLFLTSDSPLPNIHTNHSTGGSNTLATNASY

TGINSLLHQNPQLFMNSNMGRPRSPRQVNLKLNVNNSGPIKFESLTPTMGLSPELENDIM

NNMKRKHEDSSEVGKKKAKVDSNTKSKKGIKKRKKKKNIDDFSYMTQPSTGILYKVRLRD

LIKELQPSEEEILEEDHTIQPEEIIQDMKGNKKYRCMHCMKFFQSGHHLTRHKTSVHSMI

KLFECLRCGKQFKRKDHAWQHVCKKLSCPFLENAEEDLLLRGVICEEGDVESD

>34-9_0615

MEKIINTSKIRIIENIKMNTPSTKKTLIFTNFEYNFINLINFYLTHHNTENPNDIIVCRI

AGGWVRDKILNIPSNDIDIAIENKNVSGKLFLNNLVNFYKDNDNIHIKSFNEIHNTKFKF

KNIFNTNIHATKVNPEKSKHLETAMATIFNHDIDFVSLRKEVYTNENNRIPTIEVGTPLE

DCLRRDCTINSIFYNINTGYLEDLSNKGLVDLQNGIIRTPLEPMKTFTDDPLRIFRLLRF

KNKLNFQYSEDLMSCFKNNHDVLVDFVYKKVSRERVAIEANKILNTNSISICKNFLKDLE

YFGFERFLYSNNKNSYEDELDELEKLSNEKNSKKVNQKKVMLFDKITLHERTLNFFEIDL

KTHIKFFIEAIESHKDLASYKLDMTTVVHAILLFSLLDPRLQYSNKNKQTDLSIIKKDIE

LALLSNGIAKTKVRQIIALQELLDLVNKKDFEFIVGDFDNNIEIKNRAELCLFVKDQITD

SQIYNSFKNILHIFYPQHYTKFIKVLKENKIINDDNEDLDAIWNKDNLKLKWNGSMIMKH

LNKKPGVWMSEVIRLQWLYYFEKELDLVNDEDTNIEDFINWVNTQSNILKDN

>34-9_0616

MNFELNISDTSINDFDDLYNLLNHDLDLQLNSALDNINYDTPSRNDFFDSLEQKVDDNSI

HRETPKVTNVNERTMPKTVDVFITNPKQYQETNDINTLETKEVASTFDSRDISNLEEELY

KSKVLYQRPILRSVSSSKGKKLAALNIDTIKQNAGYSVIDNSIDIQAQYLNAKENDNASS

KTPMSAFSYNIFSNERSISPLVTINMADKDNSQKNSYPCFNYKKIIDYENIKRMSPKKED

FDIKSKIKSKIKSSIAKHVTKDKHQQVNNEYKEDKKLINVMSSKKISKKSSFSNLNNDNK

SLKEKRSTSSLRKKQSMTFDHKHMGNAELNLSSSLSLTNELEDEILMYPHYSSNVFNDRL

RSSSFLSDTTTCNSRSRNNSHFKPTIRDEKLSELAANEDEFDFTLDENKESDHLDSMLKM

SHIKLIKKDSNGTNSEEAVNNLSDLDISENDATSAFKLARKQSFKK

>34-9_0617

MVTHNNTSSPFDDDIGKKLFDISLDDDNDSDFNSSTKRNNNNIYNLSGNTEQNRPAGLFA

DTKTNIQTDFYSDIDDEDDDDSFTRQVKTPKEHQHARFVDEFEDDRQFLKHNDKADESSQ

GVKRLRFGTRRHKDGKPKVGRAKTLKWAKSHFSNPFESSGVASHDDEAFNTTDELSGESK

NRAFEKRSIFFHAELPPELLNEEGKPMVIYPRNKIRTTKYTPLSFIPKNLGFQFNNIANV

YFLTMVILGAFSIFGVTDPGLAAVPLIVIIIITMIKDGFEDSKRTLLDAKVNNTPTYILM

GIENYNVSDENISAWRRFKKLCTRLTSRSYKFLYQRLTKKGKADLSREKHKEQLRQMNEG

FRYSTNGRNTMEFRASMDEDNRRSIHLNASELEKFGTLVDPSIPTIDDPSIRFKKSCWKD

IKVGDIIRIHADDEIPADVILLSTSDPDGACYVETKNLDGETNLKVKQSLKCSHTIRTSK

DIAKSKFWVESEGPHPNLYSYQGTLKWYQRKNLSNNNYSDEDDDYITNNEINSSGPGLPP

VNGYYLKQESCNVNNVLLRGCTLRNTKWAMGVVVYTGEDTKIMLNSGITPTKKSRISREL

NWAVIINFIFLFVLCLISAICNGTYYGETGTSRNYFEFGSIGGNPWKNGIISFFVAVILF

QSLVPISLYISVEIIKTAQAAFIHNDMLLYYPKLDYRCTPKSWNISDDLGQIEYIFSDKT

GTLTQNVMEFKKCTINGKKYGRAYTEALQGLRKRQGVDVEKEAIIELDLINKDSQEMFKL

LPKLGDNSQFFKDEITFIAKDFVNDLLNASGDYQKDCNEHFMLALALCHTVLSEPSKTVE

GKLDYKAQSPDEAALVATARDMGFTFLQRTKTGVIIEVQGIEKEFEILNILEFNSTRKRM

SCIIKIPGDENTQPKALLICKGADSIIYSRLSKKNNDPALLENTAKDLEDFATEGLRTLC

IAQREISWEDYLVWNKKNEEAMNSLNDREGAMESVAEEIETELVLLGGTAIEDRLQDGVP

DSIAILAEAGIKLWVLTGDKVETAINIGFSCNLLGNDMELLVISIEETDAEDPGEYVDKV

ISKYLKDNFDMLGDREELAYAIKEHDVPKGEFGVVIDGAALKLVLSDPDTARKFLLLCKN

CKAVLCCRVSPAQKAAVVKLVKETLDVITLAIGDGSNDVAMIQSADVGVGIAGEEGRQAV

MCSDYAIGQFRFLTRLILVHGRWSYKRLAEMIPCFFYKNVIFSVALFWYGIFNNFDGSYL

FEYTYLTFYNLAFTSLPVIFLGILDQDVNDVVSLLVPQLYRSGILRVEWNMRKFWFYMVD

GLYQSAICFFFPYMLYKENGLVTKNGLGLASRYFVGVPVTAISVCACNTYILLQQYRWDW

FTTSWVLISNIVFFGWTGIWSSFLKAGEFYKSGSRVFGSGAFWGILFVGILFCLLPRFLF

DVMRKMFFPKDIDIIRECWKKGEYKMYPEGYDPTDPNRKKIVKFMPDLEDNESLERNVDL

FEKTTRVPDDKRKLSTNTIKNHFRHLSETVPIINGRQSSVISSPVLEKGNNFESLNKNGI

QLDEF

>34-9_0618

MLNLTSKQLKSKEFKRGLPHLYKRFLRLHSFINVSTTTISSSRFVGLYASIIRTKFQVVY

TYPFVTNIELVGDTDMFMKLRNIKQKYESIPKEQDRQKFLDKNLNTLILNNYLHYSIEDF

VSFYNMLLNDINKNEGKFLNWILTKNVSVIENSSDLTTVRNNLLYGSIFKFDSMFYTKQV

GDALAWRNLFKTSYSFVAGMKNYYNPTIIPYRKDLNDHVHITSDYQAKTKKTIMDEKNAF

LTHKFNSTDAMNEKNQQRSLMSTLLALRNFPKRTKQLLQTMSKKNDYNFLFMMLHNEIHL

HNNPSIQKNYLSYLQFDATSENKIVDNTIEPAGFNWNTKRRISAKMLKRMEEKKRKIEYK

VNNKTNENIKEMVAFLEENSKNLDTMKYGAIRYSHNILTSEYMKKKFILPYIKDEFN

>34-9_0619

MSAIPRRIVKETKNLIKDPVPGIKTSIPDDDNLRYLTVEMEGPDNSPFAGGVFNLELYLP

EEYPMEPPKIRFLTPLYHPNIDKLGRICLDVLKDHWSPAFQIRTVLLSIQQLLGTPNPDD

PLLSDVADLCKNDNAKYCEVAKEWTKKYAIPSPKD

>34-9_0620

MADITEKMNQLSVKETTPANAETYSLHISDLDKSVNESNLYDLFSQFGVVVSIKLCRDAV

TKESLCHAYVNYPEKEFAVNALEQLNFSEINGKQCRITWAIKDLDGLKNSASNVYVKHLS

PDVDDAGFYAIFKTFGEILSSKIQYDSEGKSKGFGFVQYKDKQSAENVISAMNGVELEPG

YVLSVCAHMTKEQRDTKLQNIKNEYTNVFFKNVSAEGSDEEIKQLFSEKGEVSSFTISRK

EKSPVVYGFCNFVKHEDAAKAVEALNDSEFKGFKIFVGRAQKTHERKAELQKAHNQAIQE

RNAKFQDCNLFIKNIPKSFKEEKLQELFSPYGEIVSCKISTRGDDSLGHGFVLFSKKEEA

AAAIEGLNGTTIEQKDLYVGLFQKKADRSNMLNREFEQKFNRFGGPGVPMGVPNFPQGMP

PQMMYPNVYPGQIPPNGFNQIPMRGFPIQQRMTQFPPQFKHQLGSKWFGHVLAAVGGSEE

KANEVTNMLLSLPETKLSAMIADETAFNQELNEAVRAFEGAAQ

>34-9_0621

MSSNLKDAALENPELYGLRRSSRKPVPSHSFYESDSDGDVVTKRKSKKPKNDDFIADDYE

EDDDDYEEDYEPESRSNRNRGRGTANSKTLDAALPTRFSNRGRTVVNYAENNNNDVYSDE

EIQPKTKRGSKTNYRNTTKAANPPPVRKLKLKSKKSTTKKKSKYYSESESSENDENSEED

EFDQQFLLSEEDLENYEEPLEDPRNIIDIVVDNRLKAEYTLNDLPQEVDGLTVHECKEKY

EFLIKWQGKSHLHNTWESYNFGTFPMDTEEDLENLPPLTSTTKGIKRVDNYCKKVLIEQF

NLIKSPYSVAEDLETLNLNNERIKEEWEQCKQVERIVNSQRNEETGKLEYLIKWRRLPYD

ECTFEDASLIAKIAKKDVSLYQLRIKSSVIPKNSDKFQKDRRPKFEKLLEQPKYVGGKDN

VLNLQLRDFQLTGLNWMAFLWSKDENGILADEMGLGKTVQTVCFLSWLVYSRRQYGPHLV

VVPLSTLHAWQETFEKWSPEVNLVVYLGGNKSREVIREHEFYNGKNLKFNVLLTTYEYIL

KDRDVLGGINWQYLAVDEAHRLKNDESSLYESLFSFKVANKLLITGTPLQNNIKELDALI

NFLMPGKFTIDQEIDFENQDKEQEEYIQDLHKRLQPFILRRLKKDVEKSLPGKTERILRV

ELSDIQTDYYKSILTKNYSALTNSTQGGHISLLNIMNELRKVSNHPYLFENAENAVLKTR

FNKGENQEHSRENILKGMVMSSGKMVLLDQLLTKLKKDGHRVLIFSQMVRMLDILGDYLA

YKNFKFQRLDGTVSSQKRRIAIDHFNAPDSKDEVFLLSTRAGGLGINLMTADTVIIFDSD

WNPQADLQAMARAHRIGQKNHVMVYRFVSKDTVEEEILERARKKMVLEYAIISLGLNNQK

TEADLKKMKGQPNNGELANILKFGADNMFKANDNQKKLEELNLDDVLSHAEDHVTTPDVG

ESHLGGEEFLKQFEVTDVNTNFEWDDIIPEEDLKQIKLDEEKAKDDIFVEEQMRVLARRK

QTLEKMRNFNAGDEFDDNSDDEGSGKRGKSLNNKIEDKDLKALYKAQQKYGSLDIRLIEK

LMSDTELPMKKAAGAYMDKYHDLIRKAEAEVVDQEEERKAVLKQYEERTLEFNEKLSKGV

IKAEEVDDKNNPIKQLTAKRREKKSIAFDFGCLKAINAETLLKRHNDMSLLRKTMNQMNG

NKELNDPELSFHFTVPCNQKVSWSVPWTNEDDEKLLVGLYKYGYNSWSQIRDDPFLDLED

KLFTDGRAISRRETKKETDDVKDPSPSSITDESSQTTKTAPIKKSPQPSHLNRRIDTLLG

HLRDLVEGNYQKVSEQAEQKKTSKEGSSDASSKRRKPASQKDSSSNSAKKLKSNTGAPVI

TSSNNKEKIKTQMNKVLPSLGNLKNSSSAKDKAEKLNILKKELSTVGNHIEGLLKAQKGT

LGNPVREDYWKYVATYWPSVVKFEALMAMYNKLTRRSNVLKWINPYAWVADNNINNLTYE

ISRLILCIQIFTTAIDTPERFLKYHWKTICMLIIPVMTYGWIVTGAFIKLIFPSMHFTDG

LLIAGCITATDPCLSASIMTGQFALKYVPEHLRHIIAVESGVNDGMAFPFIYLSLELIKY

SNSSKYNENAKARHVIKNWIVDVILYKIVFGAVFGIIMGYLGRKLFGKVVRNEEDKKFEE

SSNVSTDKLEAGLTDKKTDWFTQEALITIYLSFILFLIGCASTLGIDDLLVSFFAGSAFA

WTSWYSGHTQEFKFISSLDYLMNCCYFIYLGSIIPFDEFNNALNVKYVPELNIWRLILLG

LVVLCIRRLPVVCLLYKLMPDIKNFKESLFVGHFGAIGVGAIYSCMMAIQFLKEEMNKLS

DIDEAEKNYYTRLIYMMWPLITYIVFISICVHGGSIAVYSWGKNIYKKFVS

>34-9_0622

MLTNILPFYHTSEDIKGAHDILVNPSTKTESVATLCSPKIKNENKDFVFGNGSTKTIVPL

SAKGNMLERQQKYNLHSPSLLQNNTHSKLSGTNYLPFSGKTAVSNKPFSNGFNPISELSL

FGLPESYKVFSNHNYNVYDLKAKQYVDKYNISSPGINNSDIYEEISFNSNDSPNLQLHSS

KEDNNLFLDEVEESPLRMIINRNKVVYSLPDTSLHQEKFIKADDAYKNENIEEVLKSVRR

ELLFKDENIYSCNNVIFGQSLNKKDYQMDDADKLKHILNFEMGYMINFISEKIKEMEDTL

YSLRKDMQSPVDFVKCYINSFVCSNIKTNYFLLKESIYIAKKAPWCLFNETFWANYEEDN

IPLSKPVLLEYPNDNESFIKLENDFQIFLEKEAEKLLDEPYQLETN

>34-9_0623

MLLKQWVFMVPFLLITSQFDLSAFTPNTYKFIQTSDGKEDNNIHIMVNHLTTSKTSKKNN

HSIYDFFNKHIHMCKSDQNSVVKAQENLGSILLGDRIYELPYTQSEETVFGVDKFCQKIC

SSSISTEDADFINGLIVDNYQYQWFIDDLPLGSHYTLKHNENDKESVDYYSNSFGVGIYD

DKNEGSDFYKKPLIFNHFDIIIEYSTYDVLGSERIKDNTEYVIVATHITPYSIHRNEYKD

TNKAKKNYCSFTGQPKELLLDKETDIDYTVSIKYVENKTILFKNRWDKYLALQKMIISND

WNNILILLAINLVLSLTIYKLVMWNMNKYKQQNAIEKTLDMEFDEDQGWKLLLGDIYRQP

NKLSLLCTLVGTGIQTMVTALLISFLGASGILPMGDRGLLTTCSILVFSLLPFITSFTSM

KLYLMYEGRLFKRNMTLNCCLVPLFVFALLLLTNISNVIKLPSHIHSPSVIPLKTLFVFI

PIWLVLYIPSSVLGSVVAKKTTDTEKQPRKVNKIQRLIPAQPLFSKMLFLCILVSALPFS

SVFLVIKHTFGGDILVSGLIPKDQFVLISISLIVVAYNCFILGVIITYSLLIFENWKWSW

KVFMTLFVGMFTYTFVYGCFIIDAYHNIVKYLALGLLLGLICGSFSFLGGIVCVKKMFNE

KTSAPERLA

>34-9_0624

MNNENVLLIDNGAYKIKYGMAGETSVPKSCYNSIIKSKTPYGEIYCIGNQIENPKILQST

TNAYIKRPKEFGMLTSWEIQSEIWDYCFYNPDEMGFDLNNYIEHKKDGDVRCPHLVLSET

MLHMPELSKNTDEVAFEEYAFDSLYKSPSAGFIPFNRKHIKKTQFLLDSNDNIIKNDEKD

FQNDAEVSYNKFQLVIDSGFDCTYVIPVINGCIYYDAVRKSNFGGKFFTGFLKNLISYRH

IDLSQETLVVNKIKEQCMYAAPDSFIKHLNNAKYNVVKEFVLPSEEVRIENKSLDDRIDS

NMGYLLKQDEFLPNGRVSLKLNDELFTVPESILSPDKVQFDSSLKMSSHGLIELIQQSLN

LCPDMIKPLLVSNIVLIGGNFNIPNMKNRILKELQLNMPTEWKVRISCDEKKSDLFTYDS

MNEFAKTDEYLQTRYNMQNIRNQDTAAYSLFHKQMLADKASSPNPEKFISSHPDLNKYYI

GVDVGSGSARACCIDATGNILSLSEKVINKEVLKTNHVTQSSTEIWDAIRHCVKSCVGEA

DLQPHDIYGIAFDATCSLVAVDKDSGKPLGVGPNFDNTDQNIVMWQDHRASKEQKLINST

GHRVLKYVGGGMSIEMELPKMKWLHNNLDKETFAKYEFYDLADYLTFKSTGNKTRSLCST

VCKQGCLAPGVEGSTKGWEDDFFTAIGMDEFVKNDYVQVGGSAENFLTAGEYVGHLSHEA

AQDLGLSTDCVVGSSVIDAYAGFLGTVGANIDADIPALKEIDSKETGFNKAAGRLASVAG

TSTCHISIEKKDGVFCKGNWGPYYGVCFKDLYMFEGGQSFTGGLLNHVLTNHPAYTQLCK

EAEEAGINKFDFLNSRLETLKEKSDEKSVVALAKHLFFYGDFLGNRCPLNDPDMTASIIG

QTTDVSLDNLAITYLASCEFLAQQTKQIVDNLKNSGNVIECIFISGSQCKNGLLMRLFSD

CTQLPIIVPRYIDAAVCFGTALLGCAAYKNYKTFHGTDDKPINKAQTRVKQDTFIADRSP

SPYTQPSALGKTSLRPTIKKAVEKATDYFSQIPAKLEGSSDNSSSSGVDSLTNDSSKISL

EDKNVDPKMVAGDYLWDIMTNLTGTGKSILPNSTDHPDVQLLNTKYEIFLDQQKSQTLYR

KRVDDKLAETFDDSLLSSLQNTFDAESISFNKLDLSEYKNNQIEILSGSTGNQSNSNVTL

IKYQGEQNFTTYNNKGNHLIYYNYNDNSDLTNGSITKYINLLTDQDSADISYITNLRAFD

ENCFVITGTGSLNGKDLSKQVLLNLTDLSYTELFDSSITNINAVHFLGDTVYIGGNFTYD

DHHAVISYNFTKKAISPLPFGGFGDSSVVNTISNLNNDTVLFAGKFDTLDESKYLNKTVY

YKYQNVTVTNETITSNHSISTNSTDQVQQLIPLNFATWKDNGDSTFKNLDSFMCPESGDK

SSNWIGDNTGANFEVDFLNSITPSKIRVFMTDGSDSVSQFRLMLLNGGYLNMTYYDPFET

KLKSCDNNCPLYNNISTSGTYYLNDNITSISFDTNFQDFYFSPNVPMDGLQFQTIEGISL

VGIQLMQSGFAVYANNTFNNPGCSYINSHSQATLHGSGWTSEVSTSSGNSSFMQAPVESS

SERSSTSVIFSPQINVIGEYSLELYTPGCLSDNTCSARGIVNVTLTYENENGETQSKSQL

IYQDNNEEKYDTIYSGYLYSKPSVEVRYYQPIVVGNSDSLVMVADRLGVTPFNIPNPINY

IEKNKTTNHTTNTTITKHTNVQLPINGLFEYSISNFTNNKLNDSMVVGNTSLNYYPQQTF

VNNGIDDFSLFGSFYNHTLIVASGNLDGIVLLTLTENNQIESQNNLATGGNSTNALTLSN

GIQLLMGNFQLNGENLSTLYYDQSDGTFSSLGSQLPQDMSVNNFNTIYENYDSYLFSFDN

SVYFNWSSTERFENSSDFNLILKSSGFNKNGDTIFFGSIFNSKYSPYIEGSSYAINENMN

LQALSLPNDKSSSNTNGFEYDQGVYINDSFTGYAINTGDDYSMIFVPENSTYGSTKLAPF

SFKDPLKNLLFNSNNGLLSFTAGKHLYFYNLTQVETIADVTMPSNATNYDVNSMLYFEKD

NSMLFCGGMYFSTDGNQCNGTCLYGISEGSWYPLEFKSEKETIQGNVTKCLLQDEDLFYV

SGNFTYEKKNYNFLSLNMTTGKIVHKFGFDFEIGTVLDFTVDKSLNSVYVSTEKHPEVIY

YQNYSNESKWSNVTSTENIQSYVILNSASNKRDLDSNLNVLAVGESGKASVYNSDSWQPY

FQLSQTDLSKQADNIFFENKDNSESLVSKTETPLSNLLARIKHHQEKTHGVIKTGYVVLF

GLAMSTVTISCIALACSVLFYYTHMRKQNKLNASSLKLDKMFEDNLESKMIRNVPPEELM

KSLS

>34-9_0625

MSSIWSSSKHVSSANSIIPDLILEELKQRTNEFILLNNDDDINIITTFEEKTTKDIDFFY

KESLENAILSYYSNNKGNTHSEGINFFIHQIKKPIIESLYNTIDNKLVVLIQIESIDEIF

EQNKDNEDTSEIFQILLNSCVEKIDRYNTYFSNLVLFVFNYTNDSVKKHSTIINLMKTIR

NTYFVDCIIRDIKQESIADFNTNIEKLFLNKYFDKTYISYNESLQNKQTNILSKSISKLW

SFTSDSDSKKAQQKKLESEEEERNYESLSSAQKFEKFNKYKKLAVMNLLKRNANIRESNK

SIEQCMKEIDDSVIFNRYTNMIKVSEYHKHMFILQQYNKMYDHSITISDLYTTLNEFELN

YEFSTYLIPIIETVCHAGNYIEDFIRIYEFMLNKTPENSGNNIIRGFFIDRISSAYELKN

QNRKWSFNTLLSLREWKEALQNDTMLDENKHVINTICEFKSSMINSFYNDVVFSNELNKY

K

>34-9_0626

MSSQTATRLLEPKDGYAIVVGFSAFFCISMIFASFCLKKYKKEVMTSENFATAGRSIGKF

LSASSVVSSWTWASTLLTSSALTYQNGIGSAYAYAAGAALQLVCFAALAVAVKIRAPNLH

TYLELVRVRYGKITHIVFIIYALMTNILVTVMLVTGVIDSINVISGANIIGVAFLLPLGI

LCYTFYGGLKSTFLGDMSHTIIVVIVILMFLFKAYCMGSKDSDTGLGTPTLVYEALKKAS

SINPVAGNKNGEMLTFHSKPSFIFFVINIAGNFGTVFLDQGYWNKAISTSSENGQVFYGY

YLGFISWMVIPLAMGTGAGLICKALELSPKFPTYPNPMSSDQISAGFVVPFMAQTLLGKN

GAYAVMIMLISCFSSALSGELVAVSSVCAYDIYKVYINPKATGKQLINVTHLACIVFCFL

LICFGIGLHYGDVGMGWIYEFMGVIISSCVLPAVLVLFWKDMNIYAATISPIISTLLAII

GWMCSAKGLYKTVNVNTLFYDNPMLIGNVICLLGPLIFIPIFQIAFGKQNFNFKLFETDI

KRDDDDEEILKAELDIVKSTINENANEESNSDQDLEKKNLQAITSVTSYAKDIPKEALEQ

KKQENLAMLKRLSKIANWASLFFCLALCIVWPMPMYGIKGYIWSKQFFTGWVIVIIIWMF

ISSFIVVFGPLYEGRKSIWFVLKGLYWDITGQSARLSQYQMDHPEELLAVQSQVDKRMYE

IKDPFPSAYSCNLYMASNIIDKYYHYQNKNSTLLLKNKVKKSSNVVKKKTSSYQEFKNEQ

LIKDIELNLIPSLKVISIVSKKMHKEVADFNLEKEKYESSMKKYNDAIKKELEKQRIIQE

KKQKEEELKKQQEEEKEKELIASKEKERLEKLKESAKFMYSSVRVIEEFETNKKRINEIE

ETIKTPVENSADLKHVYAQRRLINPTFGQLTNSRRQLIELTTKVTGYINQTKVQPLAYQW

ILNFTAKALCKQAELECRVKPEMAIPLAEMAHLLNVQFPELIPNFLIPRLIKKCPVIIGY

MSNDKKEMGFKVKDPQNPEMKAVYLERMDGLLAFYSMLCKTQNTNNISLDLIWKMISRWA

NRNIKSLDGDLVFGSLVSVLECCGNEFDQRYGKQANKLINIFLVGDFKKEYSSLVNWKRL

MIMVEDKMASGRYKSFQGLLND

>34-9_0627

MKSIQKRDCDLLIEDKPVEPPYPIRLPYQEVKVGFGRGSSDLNCPTANIDIPKDNDDLNK

NLPTGVYFGFCKLRPNSHNLDSCKQKRVQNDNSEVEINKGIYLKEECKIDLKLPCVLSIG

YNITYDDNKIKSRSLEVHVLEKFEHKFYGAEMQLTILGYIRPEIKYNSLDELIDGIETDK

LVATEVLTWDSFKDI

>34-9_0628

MGLLEDDTRLRLLTSDLEKDVLSLNNWEILLNHILKTYYHNKRFQLNDDHKFDEDLKKSV

INLLNTTYESMLEVFPYLENYVIEYGNFKLEQNEIGQFHTIYKHYMKLMNNRSLLLWVNY

LKIIVNNQEEFNLSTKHILHTFEKAENYIGKHFYSQPFWSLYLLFLKKNYTSNPQIYLGK

LRRLIKNPIYDFAYNFRLWFKELASVSDMKQMKFFCDIKSFSKKIPRLLKPREKLQFVKF

KIDKMYKKLYKKIEEKVMKLYNSFELPLTVFKQSNQYYTSSKIPLNSSFVQTWIRYINHA

MNSKLRNKKAAKDKFYIMLIFQRALLSSSLSHNKIIWSLYIKWLSSIGDRKSAYEIYRQS

QKFVINQ

>34-9_0629

MLRHLQSKSISNKSHRHFSLSSLVLAQNLTEKIVQKYSVDLPANSELHSGDYVSVSPYHV

MTHDNSWPVALKFKGLGATEIKNKDQVVLTIDHDVQNKSESNLKKYSNIAKFSQEHGLQN

KTYKAGDGIGHQIMIENGYAFPNTLAVASDSHSNTYGGIGCLGTPVVRTDAACIWATGQF

WWQIPPVAKVELIGKLPHGLSGKDIIVSLCGIFNNDEVLNHCIEFTGSQETLASIPIDSR

LTIANMTTEWGALSGVFAVDETLKSWYKEKYEDKEHPFSKRSDLFKALSENESLKADLDA

SYAKVLKLDLSKLTHYIAGPNSVKISQPVSNLADMKVDKAYLVSCTNSRLSDLEAAAKVF

KDYGPNTKIAEGVKMYIAAASKTIQKQAEQNGTWKTLIDAGCTPLPSGCGPCIGLGTGLL

EPGEVGVSATNRNFKGRMGSKDAKAFLASPAVVAASAIKGKMAVPNDVSIDHDPEGIIEI

VPQKANSSSDSAAATVEMLPEFPKEIKGELIFCDQDNINTDGIYPGKYTYEDYVSKEKMA

EVCMENYDSEFQTLVKPGDIIVSGFNFGTGSSREQAATAILAKGVPLVVAGSFSNIFFRN

SINNALLTLELPSLIELLRKKYSNESNTALTRRTGYHINWNIEKSLVSVTNDVGEVVLTN

KVGSLSKNLQEIIVKGGLEGWVKSKL

>34-9_0630

MSLLLTFINRAVESGIIWKLLNILPIISKLLLVISIGIIFGFVPMDGSFRNTYISENALM

PNQAYSQFRETEWNIVRGFRNEISDLKIWNLDIRNRIVRNWLDDFGLETDLFQTDYDSAF

NNGIKSEVLYGILNGQRCDGTEAMLIAAPWYNSENNYNVGGVSVAVALTKFFTNWPVWSK

NIILVIPETPGYDLRAWVESYHEDLKLTGGSIETAIILDYAMDSDSFDHLDVEYIGLNGG

LPNLDIINTAIHVAENEGVKVSINGMQSFSKPSHEFSNRLQHIAVGLQKMALAGLEPLKG

HEAFSGWRIQALTLKAIGHGGNSDITTFGRIPEAVTRSVNNLLEKFHQSFFFYLLLSPKL

FVSIANYIPAAILLSVSYAAMSIDAIVQTGFLTSNEKMINIQTIEAFVLYLLSLVVSLLY

RQFITLIKNRNLSIEQQGKFIMVLQVFFVFTSYKVYKPKKTTKILMQSIAFMHFSLALTL

LLALNFGLCFGMGVLAYPMALAVDKNISKIRQRILLLVSNPFIAIFFMYSFTQDRWSWNL

FNVMVSSWDNMSCWTWPVVSCSWLTSWILIVLSCNGEQESVNKEPKSIKQE

>34-9_0631

MSSLESIFSSLVNTTSVFSYNWIFLVCFVVIIIAIIFYAYYFNRLFAFVFTKLLNFVLFK

RFHIIIDIEALHVSLLFGTVQFKNLAIITKDSTVSIVQGTFQWKFWMLRSWLSSSNYYAE

HLDQEKQHGKTFFSKDIYKLVKESRNFINIEGLEIFIYNCTSAYQRIFNEVMTERDREIL

MEKFPVLAEIFCNEPTVKTEESINKKTKLSNNGSSVYNVSLIDQLLDSLLPFVINIKKGS

LVIGTTATKYLLVTHWDMSQTLLHGIKSENSLDLYQVRLNTSHDNLNMILSPNPLYKNTK

NIDLSEFSHSILKNGRNLINFAKHRVLQKVNNIRDKKKDLKKVMKEQSSEWKGLAMYINM

INFEERGDNLLDIHDKTYLDNDEANVLPFVEEYAKYSTLLECSHLSHVYYFDIPGYIPDT

VIPTATDYQGPEIGNSGSAPEMRLDIQLSESQIIIGPWAYRNIMPIINLLFPKHSRDNKV

AEILRPGDRRSYTRFQLFVDIQENCNFRIPTREQSKDAEFISESELFQKSKMNANLDLPF

STKLIRPYGWIDLEVSEGTSFFFDFNYFPIDGQNKGSYSFDLLEPKITTSVNHEILYSAD

FHSLIWESLSPLAREETIVNFINQTTNNSKCNLLREHINLITDLVNDFSVPIEEVSLEDQ

YQVFAPQDYNFIWRFKDGYNLMIAVNDENIINSASEYDENNTISFVGDSSEAEIYLPFQM

LQQTSNNVDFNLKTRFYDICLTTPPYSTISGFSENNRIARGRDLEVSGHFEFFNELNFGN

IDNLELIFDTEELNVLFYGFVIKYFTNFYENLFGETSRFKTADEYQEQIFQNTERISDVL

SSPLDIETFSLNAENKKNISNEASTNGDFSTDSNIDKIDNQDDQNPLVLNKDDLKRTENE

LDISVKFNIKTANLYFPEKLLSLGDCLGLHTENMSVDIRALDYYFDLQIDAEPIYICEHP

SISGSNSELLLNTNFFNSNKFGTEEGILTHLNCHNHRLAGPVPINDSYFSKWDFSIGKLM

LDSKLRTLSKLTRTILNFDFSLDDFDNTLIVDKIESYGLEFDLFEIEGVDIKFREGDVIT

KIIADGITFDYQEYEKNGNSSVANVIVDLLEIVCEKGDDLVFKIQTEVNMEKTFRYSDYK

KRKQKQKQLLIKNDAPFHRIWFLLPMEFRNYYIYQNSLGFIKTGQSIPNLPTPITNETGD

SIIEDYINFAASSHFSLEERKQLLRKKILTTETLDLYPLDNSEQNDSISDGKNKRTMSLM

FDNVKTYLDIAHLDVFNILIEELAKNDCEGILDSIYVDIVKNLYLKEKIEASDNEFDVVV

ESSTLKVVNTAYSKEKYVILNTSPISTNMELLKRFELKNNSIASSVIKKFKLSTKKMSLS

LHDETNHNVAMNNNIKFVLESLDYDLKNDGQNDVKNFHFGTFNNLLNLKELEIFFEMFTF

NINALYSEINSFVSNIKNIQNTERELVLQLLSAGNQYEINYDPPAISKLSPIVRLSKTHV

RENRSWRICARLKHIYRNVPEGWESSFMNRIEKNEFTPQFLAREQFFDCFKKWINIEKQV

TDSTYLYRRIFLNENNVHEVKSKFNETFELFFKSVVLRISSQQDKVSDSVIFKKLKFSVG

NDADNAGKLKINSDFNFVGGRIGLGTLLLLKLKKKLMAKLNKNKLVVDTDDSLTPDAKSV

VSVGASLTSQVNEFMKFILAITIKKVKLDFDLDNQILEIINHDTKLDVDLNGLEDFSVNV

GSGLSNLAFKRNQLFLLKSSISDLSTVINRGFNKTSDKNELHISTEINTLNFINPEDMVK

TVFILENIYRTFEKYKDSVNDLFKDDENEDKSQSTTDFTDVIHYKGLIKNLKTSFKVLHP

LIIETKTSGLSFSVVNTEEKINMLFAVESFTVDVSSINTFYHYLLVTQKKLHIVTCIHNA

FNNKDLVCDLDFSLSKCKFLVSDLRSIGEKLVHDLLKVTDQINFIKDKIDSYGFNGNTNE

TEKVNSHTLFNIGLTVDYIGLLFDFSAQQLICEMTKNVFGIKNYHYIDAVLEMTKNSLWY

YAIDSLSASINDKILDQSQSKIFDTSFSGFVSETDDTKKLDFNLESEFFNVMFTPYSLIR

LIWFVNQFENIKQMAERDGFCKSSDVNMKSEEDRLSNAATFLEKFNNIKFVSKNVSLGWI

YSNESGNENNWNENGLVFGYQSLSLNHYSKKGEIALNGLFFGVTPFGKSYNFYKVMRSMN

FINYVKLTILKIGYTSSTLSVNNFRNLTIDVFGDDIEIFFGENIALVAEHVLESVYEFKK

MENRYINKKPVVKRSGSVDHVSDNIKLFESLGFYEIKCSALFTGGYCNILSHKDFLDSRE

PTISLHSPRVTVDAAYEFKYHEQFKHIIVLTTKIEDSENILYPTSVPIFQRIAKDVHHID

IFLKNMEDYKNSLDVIKTPLFDNDTSTESNINIKSILENIDIHCKVHCGSQEISLTCEPM

AKVQAVLGVKKMTFAADNSELDGARCVSLFFESEKLLTKFHHIFSNSVSTYFQLSKFSLH

GFLSAEYEIKEENFVITTANSLDAHVDITQIQNLDLFFDIWSSKSNLTKFDEPGTSDIAK

PESAMYNIRDDVSTDLKTSSLVNIFEKYYTESTFAIYSMFILEDVKCIIDFGPALGLVTL

TGNKLWFEFTQYRNWSKEMALNLNKISLSSKGRLQGLIELKGFSVETCLHFQDLKDFNVF

NYNLLDSMPDIKVYLQMQSLKSSLSFDYHAFLLTEFSDWSLKILNENENDVPSLKTFIEL

NKIELYATALVTANLLNIYSSFEKIKSENLRSYYDILHESNTVSNVNPKLRSSVQTYLEN

IKKTLHVKVGSMYIQISPHSLIYPQVLIIENKNTVLNFVTYGVNKIFNEVEVSTEELIGR

VTKAATIDMKEDELIDIPIKVYQEMAKQSKGGTILSFPAILTKMSTWNAKDSNTIEYIYN

SVFGGLVSIKWNLGPVSFIREIWSIHAVALKQVNEARIEFSMEEQKEVNAKKSFFEEENI

DQRIKKIKLNDRYEFKPLVVPIIDVPNLKDLGNATPPLKWFGINKEKFPGVIYEALNQPL

GALFKEVEQEYGKRLEKL

>34-9_0632

MTSNQTPTKKNLSSNSKKRKIQPTTPPRIKPLSHNEISAETILRSPQKQSSQFFNINNNK

QILSNKSTVDSSDSSRGRSLYESSQIKTQEVSPYRRLSPARKSPVSNNRKLSLVQLSPKK

QNNTLHQLQHAMERENQNIKTGGRRLYINQLTLTNFKSYANKQIVGPFNENFTAVVGPNG

SGKSNVIDSMLFVFGFRASKLRQGRLSELIHKSENYPNLEYCSVDVHFKYNYRDGFQKDD

ETNDELIVTRKAFKNNSSKYYVNGKESSYTQVTELLKTEGIDLDHKRFLILQGEVESIAQ

MKPKAEKEGDDGILEYLEDIIGTSAYKPEIEACLVQLETLNEVCIEKDNRLKIVENDLKS

LEPGKDKSLEYITKERQAVILNAKKINLELKEIEFQIKEITTKTGILEQEKSELKEELKN

IKNDSKNFEIDFQANVTKLKEFENKVSKLDQKLEVLSQKKIAAEEKIKNHEKTQQKDQEL

LVKTEDNVSKTAAELIKLEEFIVKDTEDTHALTIETNVLQEDYQHKKEALKNKSFDLEKS

IAEIEQELGPSQQNINSLETDITLKSQEIQLLQQRSNKSFEKSSFLENKEKELEKKVNLN

IEVMNSLKFKLKEKNDSKEKLKADFVRTKKEQLLLDEEYNKNKQKYLEAKSTMSSTDTSN

KIIKALSKLQEQGRISGFYGRLGDLGVIDSKYDVAISTAAPRLNDIVVETVDCAQKCIEY

LRSNKLGYSRFILLEKLSNFENAMRQNFENTLPSGSHRLFDLIKASKPRFLPAFYSILRD

TIVAKSLNEANKIAYGGHRRLRVVTVDGKLIDVSGTMSGGGKQKFKGLMKLINTSNSNEQ

SLQHFFMTQEELKTLEDSYKTSYSDYERITREVNSLQEEIDILSSQIPDLETELSSIEFD

LDNFREELKNTIKQLEVAKIQINDAKVNAEAEILNLQTIIDEKKREIENLKFVILPKTKE

IEELQLQIKELGGFELDKQKDLITSNEKQLHTLNTRLKKNKLLIKKKNSDKEKFAKTIET

LREKLINSGKVLSEYEDEVATSDEQINEINIEKEALTQKVSVIKEENAEIENQLVNFEKK

RKAISNKMSEIEHQVKTFDAEKSQLDKKSNLTRTELQALKIRDVYEALEVLSCLPTKEIK

VDESYSALQEENEDFNKMDVEEASAETTNEKIPMDIVAKEEPQDENVMDVEKESTKPLTK

QVPLSHIEFLKIESYSLSESSVSDYLSEIAVLQEFLDSCTVNLTALDDYVTNYKSFKQKK

DELTEAINNRDSVKNRCEVLKKNRYDEFMKGFNIISQSLKEMYQMITMGGNAELELVDSI

DPFSEGILFSVMPPKKSWRNISNLSGGEKTLSSLALVFALHNYKPTPIYIMDEIDAALDF

KNVSIVANYIKDRTKDAQFIVISLRNNMFELAERLVGIYKNENMTKSTTIENFDFFS

>34-9_0633

MISTVKYSKRQFSQASILSKAGSSLVHPTHHIERYKKNILSTTTDALKTHPNDIKSNHYF

PLDLRQDRLQEHYYNSVQSDMLLMAYDHSETFTPGPVYREIDNLSNPYLINKTKKVVFGK

TGEYLDRYPITPKNIPELLDIDVVCVAKDINVNKFNYISAQALLQQTTNVKPKHLKVKVV

DQKLNLRIGSIIGGQVALNGYNKSQFLLTVNELVLPKDENFTGIQEHLVDTKGNFTLHLT

SEHVSYFPEVMSDKDLWDKLFNIMININTTAQDPAHVKMLLSGLGFPFRTEKSLKEPKRV

>34-9_0634

MAQIAYTLVSKNSGITGSEGTSKNAPSFVSKAFQLKLESPHDEEKEAVMREILSVVNCYS

TSNTGSNEEELAASLLNDQDLLMAIIRYVMPSKSKSVKKLLYQYWEVVPKLDETTATLKQ

EFILLSANLHADLQHVNHFVRGSCLRLLMKFKEREILEHLVDDVFLLLQDKKKFVRKYSI

LTLLSIYQVDERLVPDFKAVIDEFLTKEIQNEKDGVVLRNGFLALWKVDSDAARDWLLKT

INDETTISDSNADDNDTDEVALPNNVHPLLTEIFTQFIKEDTFNSLTSSEIKLSYLDVIV

NKIDTVENREVKLDYVWALVNLTAVLISNSHEFDLIETIDTIVSNLIELFVQESDWNVKL

ILLSQVKSLISTSNNEQLATLYKQQMSLQVHTLISVINSSDPFLSEAALKLILDLVDFKT

SVEVVSFLKKELLVSFDIKKSQSVASKEFLNQYRTLLITSITKVASDYDKVAVEVATLLL

DGMAVLDTTACLEVVGFLKKIISKADSNNNSQLKSQLIEKLILKLDDIRHARVYRSCLWL

LGEYCNDKKLIVSVWNHLRESLAASKSIEKKSEAVPEVAQSSGPVILPDGTYSNAPLNAE

TVTAKDEEDEDSPATEDLDELPPIKLFLRKGDYYTMSIVSTCIVKLILQAVSLSPLTEKN

ITSTPVVNGMKQEAILMLINIIRYKNEVIVGKNIDEDSKEKIMQNIEILLNLNSVAIVEK

MISLYLETTMDAFQKQFKSLQFSKAVLQKNSKGDVLPKNKSYGVDSQIGFRLLPGSSNSK

LSENSILKDIGHAISGGKDLSKVSSISDASVKATATSKLAKIVQLTGFSDPVYAEACILT

NQFDLTLDLLIVNQTKETLKNLHLQFSTLGDVKILEQPSSINVIAHGFHRFIINCKVSSA

DTGVIFGNIIYDGAHGDDSKYVIMNDVHINIMDYIKKQDLKDDNKFRVMWNEFEWENKIN

VKTTNFQTCQEYLYKLCDFTNMEILNKNDINADDEADESARFLSCNLFAKSSFGEEVLAN

VCIEKLPETDEIVGNVRIRSEGQGLALSLGERIAMVAKNVKKLKLDKI

>34-9_0635

MSLQYLNPKAETLRRDAALKVNCSAAEGLQQVLETNLGPKGTLKLLVDGAGNLKLTKDGK

VLLTEMQIQSPTAVMIARAASAQDEITGDGTTTVVCLVGELMKQGFRLIQEGIHPRIITD

GYELARLETLKFLDTYKIEKQNAIQDREFLLQTARSSLITKINPELCEVLTPIVTDAVLI

SSKQQEDITIKPSLDLHMIEIMQMQHNNSNDTKLIEGLVLDHGPRHPEMPEHLLNCHILI

LNVSLEYEKTEVNSGFYYSSAEQRDKLAASERKFVDEKLKKIIDLKNEVCPLGSDKTFVV

INQKGIDPMSLDILAKNNIMALRRAKRRNLERLQLITEGQATNSVDELTPDVLGYAGQVY

CETLDDEKYTYVTKCKNPKACTILIKGPTFYTLQQIKDAVRDGLRAVSNVIKDESIVPGA

GAFFIACSEHLKQSIVKKTIKVKGKQKLGVQAFAESLLVIPKILIKNAGHDPLDVLALCQ

DDLEESIDEDETRYVGVDLNLGDSCDPTIEGIWDSYRVIRNAITGANGISSNLLLCDELL

KAGRSTLKQ

>34-9_0636

MDSTATTLKDHQIAAIKKMLLFNTAASKTAGDDQEINWKILIMDKTSTGIISSVLRVKDL

LKVGVTCHTTLFSPQRQAIPDIPAVYFIEPSRKNLEKIIQDLKNDFYDEYYLNFTSNISR

VNLEYLAEEAVKIGKFDRIKQVYDQYLNFVVSDPNVFEIEMEKIWSEFNLNNAITEEEIN

AKCQEIASRLYNVIITMNSSINSIPILRIQPGGPAEIVGKKLNNLLNEYIINSKQFLEGN

TTENSVADLDRPVLVLVDRNVDFTSVLSHSLYYECLVSEVFKLNRNTITLPSGQKIDLDS

NDFFWENNNQLPFPEVVENAELELNNYKKETEEITSKTGVNITNLQNLNVGKDGDIQQDT

DLIQKAMDKLPELTAKKQFIDNHFKILSELIKELGNLHLDKFYELEQEDIYNIKNRNEFI

EILKDPEAGTVSDKIRTYLYIFLKTNMNQLKGNRKQEFENFVEKCDEVFKQLCEKKGGEE

EQIDEQSVINSKLLDAIKNIYRYQKMNDMTYYSDDSNLDLKKKNHVSEDTTAKNASYFSS

KLYGLANDKLQDSMGSIISRVSNLLTSDNDNLPLTNIVSSIMTSSYADKTEDELNSYEQL

VEKYLYLDPTLKKNTLKINKNRKFNKCICFVVGGGSYLEHCNLQRYADKSSDLNPNSGGS

ENNNFNKMYTDKRIVYGSTTMVSPNEFLKELANL

>34-9_0637

MATTQVSIHHHIKNLKNENFIGQEQAKQATSIILDLIKEKKFNNKGLLIQGQPSTGKTAL

AYGMTKELGANIPFIKLVGSEFYSKEVKPTELLMMNLRRGIIIRMKELKEVYEGEVIELQ

PEESNNTVGQDIQGYGKSLSHVVITLKAAKGTKTLRLDPTIYDSIVKEKIAIGDVIYIEA

NTGIVKRCGRSDSFATEFDLELEEYVPLPKGEVYKKREIIQDLTLHDLDMANVKPSSNGN

DIISMMSNLQANKKTEITDKLRQEVDKVVHKYISNNQAELIPGVLFIDEAHMLNLEMFTY

INKILELDLAPLIILATNKQGLNTINGTEDIVSPFGIPQDLLDRCLIIKTKPYNVKELES

IISKRAQSMNLKINPSAMQKLCQIGDESSKKRGLRYALQLLSPCSILMEISGRDEIIRED

VEQCMDLFLDLDRSVEILKTNTQGFL

>34-9_0638

MKTLITKIDSRISNLNEYSSDSDTSSESSSDSNSSEYRFSNNHELTRKRTMSNKGLMINI

PNYLSKLSPTTSINSNDLSTRKVPEEKNIGRGRGLSFSQRLKNNNMLINNNMNKDFNTSN

SSVDIPIEFKEIIRSRSNSRTRANSMNNNLDQIMIGDKSRTTSFGEPITNNTKSNTNLMK

TRNSRNTSMTNLKKMTSSLDSINSINSLKKSSSSLLMTGSPSSGPISMKNLKKYLTTGDE

MFLTDELSDEDLFMNNLSHQQLKKRNGDVMVNMTSSANLYSLDNGSVMNDDIEIIKKNTK

VINNNIIENDYTMPFDTLTKTNKADSMSETESMDLNTGLTFSNSFSKQLKNNDKSHPESV

TSNTTIKANNLKAYKKITPIDLYGDNNDDLNDHVSNGSEWFLGGNV

>34-9_0639

MQLLTNTHTFPYTFEETIVAVWQKYPNDMSEHVKCIDILDRKYNKEEKKITTHRLITCQQ

KIPTWLQYIIGKELTKSKVLEISELDYKNKQFSLKSLNITGGKFLKVYEEVVYKPNMAGN

TTEFKQNAYILGNTNFNKINSKIEDWSISRFNFNAARGKKGFESILELLFGTRENN

>34-9_0640

MSDTVNPLSKTSKQQQDTIRKDSTRKKSLDRIEMMKFLENIIQEEDQYKYQQSFQNDASS

VVDFNLSPKSMGTTPTPFNNVPRRSFSRSNSITNRSHQLNKETTLTNNNLNDVIINFQYD

NRQNSIRIMDEERERLRQLIYNENKNEIIPPMFMQERGQSKQEDTGTKRRYSAVISNVTP

EPEQEDKPPKIIFHNSFLEKNKNMSISTPTISSPQLQIHKNDDVMKLKSNVMLLETEKEL

NTKKIKVLEKENADKNVVINELKKNEHKLISQIHKDKILIDKLVKELAFKDKQNFILAER

LKK

>34-9_0641

MQIFVKTLTGKTITLEVESSDSIDNVKAKIQDKEGIPPDQQRLIFAGKQLEDGRTLSDYN

IQKESTLHLVLRLRGGGKKRKKKVYTTPKKIKHQRKKVKMSILTFFKVDGDGNVEKLRNE

CTNCGPAVFLANHQDRYYCGKCHSSFAK

>34-9_0642

MNNLFELDTKWKNIIDYNDTFLKDYEIGTDASIDKTVIAFLNDMCNNLTKETFIEDSKII

DPKPYIRLLENCNKEILKKLSENSLDNYKHNKESLDEREFEETIKPNFDSLKKNYAFLEQ

KLNKVLINNNNKGLLNLIQNLQQKLERNNGYKNNVELLKHYLYFYKIVEENDVENKQNLE

DLYDTKISNIYKFEISELDREQIEKQSVVNKDMSLTLERLMSLSKKLEQKNVKLIKVSGF

INFIKNKFESSILEDFTLLHENLYNNVEHDSEVIYLGLNLIIDIYNKMNMNKKNNLIDIF

VDNHPILSNMTSANIHLFSSSLSTNNTRITLKDIDTYQDEKFVDFLDNNILKIIHKESDI

IKKIFEKSEKLYFDTTSKLITKILIDKVKPLTTKIIKHANERLTSESYLIIIKSLYKHLK

SSLQEVNEKFAFCEDLNQFIKVELLNFNKKQFQIDLNKTLNILFDKELKYITVHKLPTSS

LNGLKELMKKNENKDFIEIVKHINLIQNEEQNNNKLQNLGQKTMTQFNSLLKHNEMDTSQ

NSLMNVKLNIANFSILEFLNNLIYISTLQKELLINEADIKLSKEQMMVLINTMVEEYMMK

NFEIFYSVNKKESKLLSNIAEFFELVELTQSQIISYYNIMNEFFENSPNFGLLDKLENCI

NVLYIQYLLQFNESIEKILKTQNRKDYLIDNRNMLVNEGTQSFNNLEKYMNDLLLQFVNL

KSSSMMNFIDMLLQNLYKHLLKNYKKFDVNIAGGLVMNKDILNLYNCLENYSPNVVKVEV

LNKFKLLKELVGIFTMRDLNNVRYLIDNSIYLKKSREVKINDYVKKRV

>34-9_0643

MNTLNYRSSKQALPVYNDESKERLTIKQNNNLSFIKSNKSTILYLYTLITTVWLYTIKYF

EVKYVNKAISSSYKEQDNTKLLVYGDVQIMDKHSYPNRPYLINKITELILDNYHKKNWIS

AQNIIKPNYNLFMGDLFDGGRNWDNKYWLEEFKRFNKIFNKVNVFSKNVFNLPGNHDIGF

GDTIVKDSFYRFNKFFGNTNSRLEIKGTNYVVYTIDSIALSVSTAPEVQNLSIDYLNEVK

KYHNSNPLEKRILLTHVPLFRDPKTQVCGSMRESKNPFPIRKGVQYQTVLSSEITNLILN

SINPDLVLSGDDHDYCEITHSFNNREVQEITTKSAAMNMGIKYPQLTLLSIDKDDKLSYS

QIDLHVPYMSFAMYGLLAVLSIVYLFYYTFKIEHNDYRKKNINLFLYRFLVLSVIVLYLM

STTFI

>34-9_0644

MEPSFACQTPKHNSHCITISMSSDDEEPNVLFTFDDLNNIYNINQYTELQDIEDGIDDLE

TITMDKEDIMKLQDPILKVDTVIQKFDNQNFETINETLDDEKYTNHHKNLKKIEYNDYLL

QKYESKDLFEELFEDLNILNIDMNMNPKSKNYKNDLINSVLREIKTTVNKLCYVHDIDDY

DEVIKKKELCMHEIAKLLKYNKFIEFRRSLIIKRKSKKYNNDFIKKYEDINTYEAWDRKF

LYNNIEDLNDNIAYDSWNKIWEIGGKR

>34-9_0645

MENHKLDLKYILEGSCIKHLLPKNQLFELINVPGAGNINIDIQNYNGLENSLAVDNLNNN

SDINEEILEDLKKELLTYNHNAIKFEHFETEKDFIFDKKELGLSKVAEFLLPNELLSQEK

IQTLKPDSNKRKRDDTGSFEVKKVVIKSKIERDFKDSYDYNYKLLNETNGDVLSTIHKEL

ISILGNIDNINSSIINQMLYFVDILIKKETPNNEINEVLRKIVDGLKQDNYDINMCDNMF

LIIKILILTTNKILNFEYLDNIFIFISEYSQDIGTEENMENIIKFSELLSNLQILLTPNL

IIDSWDLNRIILSLQYILDYGTNNYNLNFPKFNKTIEKIKKSTTNILKMIFGKYPLQRDL

ILDLIMISFDKIPTLKEDTKFIGIKNTSITYFTYNIIQMLSNVAVGLDFEESLPGILEKS

KLHELEMLSYGVMLTDLIIEKIINNISLKGKLMVYSKDLVKSIEYVNDIVPDYLLSSLIN

KLINIMDNSSMGNVSLSVLSELGSFIIKGKDKLKKDEYFQTLLNDNFILLKEDLKQFNAT

VDNNYLLKKYNFINELKDYMSKNEYELFNKQIHNDFELLINAELKGDLVRNDKNDYKLSN

NHLNSLNYYEIYIKVLLETINNPKLQLGALKNFSILINQNKGLINTKFIKDLILKILKED

KVIIKEVILNFIKQDIKYIELFINEIEFNVLNDSLNIRKTVLSINKTIFEQSINEKLKFY

GLKGIFKLIDDDNSMVKNEVKAYLYENVFEPLLDEIDEVLLRFLIKLVNEDDTIVPQFKN

IMMTKDLIKMFKNINHDLIYSIIVYNQNDDKVQLNSNIAFINNLLQNHSNIINKQDLIVL

IPLLNLNNADLKLRLNIFKIFATNLAMIKTDVKIKNAINSILLNDLLKYQLNELEHLIII

ITYINSKDSLIKLYESLVVTLANSENDNKLKKLIVAIVSFIIHSNMVITSDSITNKLVMI

FNINKNMLVKSIIINQLLKLCNKTPNLYSNSKILSLLDNTLQLNNTFNILKKEIVIGLTN

YMKIQESNNDDIALNLIVKYTPKILIMVVHNNESMNNTIYKYIKNLVFFKIINPMIYLPY

IVPLVIYENYHIKTLDLEFNDINILNKGTNLMLKSFTMNDDIKNLKYYNFIDNLLLLDVD

INLILKIFIKLLRVNYKNKEELIFLTLNILTIVHKFNKNVIIELINITNILLDKLEQEEN

DSLNIPYRVLKIFLKHLNYQSNNVNFVNFKMENYDFEGLK

>34-9_0646

MLGFRSVAGSRSTLQTLTRKNQMTFNSLRYFTIPFLPTLPQNPGGVKGDINDNLKHPKSD

KLSSSIHWKIEKVLAIGLIPLVSMSLIQGAGVSTLVDLSLSYTLLMHCYVGFQSCIIDYI

PKRVYGHWHNRAMYLLTLGSLLSALGIYEMNLGDKNNGSVSLTSRLWTSKNEQVVEEAAK

>34-9_0647

MNLVSKSFISLSKPSLNTNISKRYRSFFNFEKKNAPVYTPPNYSLTSNISKINIESPISS

NTSDDILECSISTLPNGVTVVSKHIPNLKTATTGVFIKTGSRAEVYNNNGVAHFLEHLAF

KGTRSRTKLEMEQFFENNGCHLNAYTSRENTVYFSHTLKEHLETSVDIISDIITNSKFEK

KDIENERDVIIRESEFVDSQYDEVVFDHLHSIIFKESSLGFPILGPINNIKKIDRQDIKN

FIYEHYKGNRMVVVGVGCVEHNEFSSIVEKYFGKLPKAENNIEVEESKEVLHKRPTEFFS

GEKKIQKDEVTSTNIAIALRSASWSSNDYFKSILAQSIVGNWNKSDGHSTNSSSKLAQIA

AAGNKNLGLAESYMSFSTTYGDIGIWGIYIICDSNQNQNPEMIINEVMKDWKRLSNGEVS

DYEFEKCKALLISSLVLSLDGTTPLFEEIGRQVCTNGKTYKVRDIINTLEGISKQEVIDW

CKKSINKESPLAIVSFGQTQNVPSLDKLKSQLYD

>34-9_0648

MENDNNIELSNQIMDEILDILSINKDDAYKTMRKKDANGNDAIRYTHAIKNHQILNKEML

DKFTRNKLQFKLKTLFDRMNELIDKDDEKLKALNKDDSSNKRNRDTEDEEEPSNIKKFKK

TADKILENEDEARNNNISEEELKQHKLKKENDPLYKNPKLEYVEPQTLPKVMSLMGLFNE

NEDQKIEKFPNAQKKLTYLRQKYGVSRFPKRDLIADLPAKFPDLDLTQPKPPTQIQFSTF

LSYIEPFFRRFVDKDEEFLSLDYVVPNRILFEKQIVKTENIKKKYLKEKNSKEVKNEADM

MIDILFDDINDNKMYDPNVTPYLIPRLGKLYLIKWFEDNPNMISLLSNPSGFNLLDASSN

AYSQKNLQELKALLTKFPSIYNHFPQLVLPKGDSSLFISAKKSKDSTDTNNSLSSSTEIS

GKGKPKGRKPGVSAATEVDKSLDGDNVVSLGPLSTRLMQCLITDSNLQKEFAFLKHDDED

NNLEEEKVSLELMQQKLINENKLIDFKLEDKDNYDIDDDEDDEEENEDDDEYDEDEEELL

NDEEDFGFDEIYEADVKTLEDGRTSENFKPTIESSTVPFTWKIENPKILSSLNYESFEER

IKQELKSLGVFAVANKSIGRVETESEGKNLGINWVKEKEDDEVCSELRTLQKKLRVVNNR

NNQRKQILKPILKKHLAYQDYAQILDNLNKQVDHEYARRLKNSHSKKKKHKHTITEVHNV

NENAIAELSGDAGNTISNPDSTASQQFQQQQEIQHGIADINLKKLLEKRKKWMDAIGVIF

ANEESEEETEHLGNKTEDDDHIPLNIKKYMVSENEFLMDGVEEIGAKSIDPAVNMKRPPA

KDVFKNV

>34-9_0649

MFRLNKFNTFKGISSQMVRKYAQDTKQEAVNFVRDRTANPLNPSTWTHLNAAQVLDMYFQ

ACSMKYLHPSLPVQKHLEYHNEVELEQLQRIAPEIGFDTNYLTTLYNNGVLSEALTEVIN

ESLSDSRDITHVYSPGALEQFEKIRSDRHYFRIKAHELPLLVEKRVSYEHRSDEEYPLKF

IFSNALNDKTSKMNASCRVEFIVKNLKLDEDVKHVVRLLAGEHYNIKKDTVSISSGLFNT

GVQNASYLKEKVQTLVQSAKENLNEFKDIPLDVKEVEEIKAEKTLKEEKLSLERNFPKEW

AQSADHVEKRLDPRPLMKTYYEQMKHLERLQHTFSSQ

>34-9_0650

MSTEHKFKLAKDSLVSSLFELSKAASHSASSILDFYHCIQDENYDLDQAFLTLQQSLALL

GQGTAQLEGVVGEYGDKIGFKIIDPSVEGESALLEDGSKKKKLKKDPFAPKKPLTIFFAY

TAYIRDTIKQDRIDKGLPPLTQIELTKTISQMWRDLDEEQKEKWRLSYNHELEVYNKRKQ

EYLEAKKSGISINFNDPVALKNEGYLDDDNEKNQTIGSADDIQPDSTIITNLDAADENGS

PSTNDNVGIGSKRALDDEAEKKKKKKKAKKAEKKKKAAAAAAPPAATN

>34-9_0651

MISQQTLSILLLISSASALPIFNSKNGNSAYNANKPDQSHMFPFLAGSAPYFPYPISDNY

YKIDTAIPETCTLKQVQSLGRHGERYPGYGEGETIKEVYETIAKVSKKNLADSEYLSFMV

DPTYEFFLSTNASLELLTTTTDSINGVLNPYAGELTAKKFGTAFAEKYQSIIGDKLPIFT

ASNLRVFKTASYFSDMLNQYLGTEIDMQVLNELEQTGGNSLTPSETCNVWDVIVHQDIID

DVDDTYLKNIASRLNNGTKGLNLTSDDASVLFTWCAYEIDAAGYSPICEIFTQEELVWNS

IADDVYGYYVDGPGNPLIKSVGSVFFNASVELLKNPPKEYNAYVSFAHDVNIQHLVAAIG

LFDDEIPMTSDFDFSNTNNKKSWIVPMGARIQLENYECGNSSYVRYILNDAVIPIPKCST

GPGFSCELNDFYKYYDTRMSNITDFATACNITNLPTYQSFYWDYQNVNYTAPLAVGSY

>34-9_0652

MLSQIVLASLAASAASAASIQGTKSSYLANEEDQSAIFPFLAGAAPYFPYPLGENYYKIN

TSIPETCTLKQVQVMARHGERYPGTSEGNKLIKLYNTKFANLSKTELADNPYFNFLVDPT

YQFFLQNNASLELLTTTEDSLTGALNLHAGELTALNFGEAFTKKYKDLIGDTLPVFTSSN

SRVYETAKYFSSIINKYLGTTINLQVLNELEKTGGNSLTPSETCDVWDFPVHQDIVDAMD

TSYLDNLAFRLNNATEGLNLTSSDANMLFDWCAYEIDVSGYSPICNIFTQEELVLNAFSD

DLSSFYSDGPGNPLVKSVGSVFFNSTVSLLKNPPKEYSAYIGFTHDVNIQHMMAAVGLFD

TDELMDSKFDYRNTVYKKSWITPMGARVILENYDCGNSSYVRYVVNDAVVPIQDCNEGPG

FSCELNDFYKYAEDRLSNITAFGSACNITNLPTYQSFYWDYQNVNYTAPIAVQSY

>34-9_0653

MSLKHFDMAAAGHAGIMTNEEESLFFKPALEQEIAFYNTVKENNDRNDNLLSPDAQMRDD

VSPLDEWIPKYIGTMQKTSQVSKEELENLGQSVRTKMEVQELVDNANPNDQFIVLENVLN

GYKSPNVMDIKLGKVLYDESASTEKKNRLIKVSRETTSGSLGFRICGIKMANIQKANHSI

FLPAELSYQNNDSYISVNKQFGRRLQTETDIKSSLKVFFNNHEAGDEYFISLLTIFLERL

EALIDTLNNSRANFISSSILLIFESDVERIKEHGINDVLIDSDFDIMSYIDDISDSNETV

EIDGEQIGIQALRKEFSLSKLKLVDFGHSKIGNDLNIKDKENVISGLYALKKLLAELLNE

I

>34-9_0654

MSEENSNATYNPNSSRGGYRGNSRGGYRGNSRGGYRGGYQGNNYQAGFQGGYQGNNYQQG

YQGGYQGNNYQQGYQGGFQQQTASAPAAEEPAKGKVSLKDIKARAKQAKEAAAAAAAATP

EVAKPVETEEPKETAAEEPKEAAAEEPKEAAAEEPKEAATEEQKEAPVETVKEEPKEAAA

EEQKEAPVETVKEEPKQAVAAEVKEEPKSAPIEASMSKLSVKSSKPVVDIAKEQEDELDK

EIVSDMFGGKDHVSILFMGHVDAGKSTMGGNLLYLTGSVDKRTLEKYEREAKDAGRQGWY

LSWVMDTNKEERNDGKTIEVGKAYFETDKRRYTILDAPGHKMYVSEMIGGASQADVGVLV

ISARKGEYETGFEKGGQTREHALLAKTQGVNKMVVVINKMDDTTVNWDKERYDQCVSNLS

QYLRAVGYNIKTDVIFMPVSGFTGAGLKNRVDPKDCPWYSGPSLLEYLDSMQTLDRHINA

PFMLPISSKMKDLGTIVEGKIESGHIKKGHSTMLMPNKTKVEVVDIYNETEEEVDVAVCG

EQVKLRIKGVDEEDIQAGFVMTSPKNPVKVVKKFIATLAIVELKSIISSGFSCVMHVHTA

IEEVVITRLLHKIEKGSNRVSKKAPPFAKKGMKVIVEIEVENPVCVETYEDYQQLGRFTL

RDQGITIAIGKIIKVAE

>34-9_0655

MSPPSDADFIINHNKSSPDLKTASTILHSTSEDDIDSLNNLLSDIRLFTTLRDADIDPIS

LKWDSKSSDYKSRGPVVCNFYKEGTTQHNAIGAHSGCYSVYHGLAVAAKQLNPNFTPSFE

NTDPQFEVPYNEKWTDGKSIVSIDPFGHLAIPENYPELAENVDIRPSIAITKAVLQLPDI

QALVKEGKIPVDGKIVLNKEGDVAISKIALDSVWYLPGIAKRFGITEDELRLNLFKDTNN

MYPELITRPDIKTFLPPIGGSTVYIFGDPSYLSDPTKDLALRVHDSCTGSDVFASNICTC

RPYLIFGIQEAILQAQTPNGVGMCVYFQKEGRSLGEITKYLVYNARKKYGDTADKYFLRT

ECIAGVRDMRFQQLMPDVLQWLGIKRINKMLSMSNMKYDAIIDAGITIDKRIPIPDDMIP

GDSNVEIDAKIHSGYFTTGSVKTEIELKQTHGTEWK

>34-9_0656

MSDQITENISEHIKDAESPVDVESQAEAISIVSESGDEEFVDASDIHLETEESNNDTVIH

SPYNEAEVESNNSVSDKILDSLHEDEENEDIEENADIASVNEHEENKENDENADIASVNE

PTEIQLNSSEEEQEEPVTPEVSTEAQEEPIEQPNIDYEEETQPEEAFRYEEDATPEEISK

SKNIKSTLAFLKKSFEEFQESKDFKNLTSPSKSLNKSLTDAIEDIDKILKYPANTIASIN

SILIFEALRNCCRCRINTGKIMALDGLSKLFAFQLLDEEAMVNPPDALSVNEPNNENTDI

TPPPKQKLMDAAIDTITDCFEGESTNENVELQIIRCLTSLILMEDTLNICHGQSLLKAIR

TIYNIFIFSLNSSTQTIAQATLIQVVEKVFQRVRVFAESDNNTINEEGELTSFKSDASVA

SVNSKFVAEDGPLNLNDMKKLNDEEEANIDLNSISEESITEEQLSIKDAFLVFRSMAKIS

VKNIEDTLDMRSHAVRSKLLSLHMLHFILRDQVSLFLNPKLKLYSNGDKSLLDSIRQYLC

LSVSRNATSPIAPVFEITLEILWIMVNDMRFYFKREIPIFFAEIYLPIAELKTSTNYQKK

YFLQFLAKMSSDPSIIIEFYLNYDCDERFPNLTEIVVDFLSNNTLNKVENVSDSDKRQYI

DSIKGAISTYDLSQLPLLSISNLSGGSVSNATSLSESNPLYMLSSNTTKEKNTTSSGSKH

KSSASLSGSKIPLSSPNFPIAYHLKLDSLKCIILILRSLSTWCNINTSIKSNIKTGNENL

DITISRKTSVSSASLGKDDNDSIGYIRSRSGSDLKLTSTEDDNDIQHFEASKIRKTELSR

LLILFNKKPKKAIPELITSGFVKDDTPESIARFLLSTEGLDLTTVGDYLGEGDEKNIATM

HALVDQLNFKDAGFIDALRDFLKNFKLPGESQKIDRFMLKFAERYVEQNPTVFAKPDTAY

VLAFSLIMLNTDLHSPQIKKKITLEEFIDLLGGLKKEDELSNEYMTELYQEIRDNEIKLP

KDASEESNNTTEAASNSAFNFFNMRDLNRETYMQVSKEITSKTEDVVKNLTNETEEDTLF

HFANSKEHGILVFQNLWMSFLAGFTTTLNEIDYDTYPINLCLEGLQHGVRISATSRIDMA

KNAFIGALVQFTNMSNVAEMKPKNVLAIHKLLEVVLQVGDDLTDSWKDVLFAVSQIERLQ

LIAKGVDGLSVPDVSSSRLKNVNSRRTSKDSVRSATVDPSITDDSSSFFGFWQTKKPNPI

EEAQDKHSKQVLNSEISKILSSDDISVKIDMIFSQSSDLNAHSVIDFIKALTEVSQKEID

SSQDSSQPRMFALQKMVDVCYYNMERIRVEWKPMWEVMGEAFINIASNSNKNLAVVFFAI

DSLRQLSMRFLNLDELHGFEFQCDFLKPFKFIIQNSSSNYEIQQMCLECFNQFIVAKGDL

LKSGWKPIFESLQYLAVNTIMDKISFTVLNTANLILKSKILMEHVLKQVSSKTNEQDDNY

LQFIYVYRNMCKNSKFPKNSLKSLESLKKIRKYIFKDVNVAQGSKVKLDEEEFNKLWYPL

LFTYHDIIMSSKDMEVRSMALTEMFECLVGYGSLFTVPIWTKICRLLIFPIFAVLTMHWD

FDQFSSHEEVNVWLSTTLIQALRNSIALLTHYFESLVTLLPGFLDLLVSCICQENDTIAR

IGRSCLQQLIIENINKFTEADWVIIVDHFEKLFDLTTATELFESDPLKKGRRQSILGQQT

VPKIELDEKKDLNFDEIAKETRETVDFSSNRAEEEESDDKMIRKVSQSGAQIAPKEVLKS

NIGTIKNDLIRKRLSVKNTIVVKCVLQLMIIELLSELFDNEQFRNNEKITTEQMLRIVGM

LEKSYTFSRDFNDDIGLRTRLVEAEIVDKIPNLLKQESSSAAVLVAVIFKLYFQTEDDKK

NNVKLMDKAMQYCVDIIQRYVSFDENKMEKSIMTMRPVVIEILQGIYEFDDEDFKANSNK

LYNLVLQILDKNLSNVTLRACVKQFFERIGDLYFPTH

>34-9_0657

MSTNHVEVHEANGVSFRQAPEHFFKSQPSKSYIVDEAQYKAWYDQSINDPESFYGALAKE

YLHWDKPFTKVRSGDLNNGDISWFLQGELNACYNCVDRHAFANPDKIALISEPDEESEAY

SITFQQLLEKVCKVAGVLQSWGVKKGDTVAVYLPMTPEAVIAMLAIARIGAVHSVVFAGF

SAGSLKDRVVDAGSKVVITCDEAKRGGKTINTKKIVDEGLNGVQSVSKILVFKRTGNPDV

KMTPGRDYYWDEVCATQRSYVPCVPTNAEDPLFLLYTSGSTGQPKGVVHTVGGYLLNAAL

STRFVFDLHPEDVFFTAGDVGWITGHTYALYGPLSLGTASIIFESTPAYPDYGRYWRIVN

RWKATHFYVAPTAMRLIKKVGEAEIAKYDISSLRVLGSVGEPIQPDLWEWYNEKVGNNNC

CIVDTYWQTESGAHLITPLAGAFPTKPGSASLPFFGIAPAIIDPATGKELEGNDVAGVLA

IKSTWPSVARTVYNDHSRYMETYLKPFAGYYFTGDNAARDNDGYYWIRGRADDVVNVSGH

RLSTNEIETVLDEHSGVTECACIGINDDLTGQAVVAFISLKDGACDITNESDFTVFKKSL

VMEVRGVIGPFASPKAIIAVSDLPKTRSGKIMRRVLRKIASNEMEQLGDLSTMANPECVP

KIIAAVDSQFFVKK

>34-9_0658

MVQVKLSLGECIYISVKPIMRVYLILGTGFLLSKKGLLTPQMTKDISTLVVNALLPCLAF

NKIVNYISYKDLKMIGIIVLTSLFIYTLGILGGVLVYFGFKTEPKFFYGLLFCSSCTNIS

DLTISIMQSEGLQAIFTTEQLGKGVSYAVIFMSVQNFLLMNLGLMTIPYYDVKNDIEKEE

KHNETDANSSTSDDQHLTKDITDKHSKADSTFNNDVYDQQTSLSDAGIESIDSISTADSD

VVDSEEVEAEEIAEHEETFHQRRKRKSSISSVLTRPFTNSGTSHRVDRTTELVKEYSIIS

KVKSGQFDIKKPLDLYAEHPTENINSNHDENDKESIPEMEEKKNTLSDWCYSHRLGWLYY

ILINFERPCSIATVLGLLCALVPWLRILFVKQNGYHIHQGPDNAPVLSVFMDFTLYVSQA

SVPIALLLLGGSIAKIDLDKLDYKFVKAAIVLNLYRLCVMPIIGIAFVNRLVTIGWLDKG

DSIGRMSLVMTFAVPSATACLYFTAMFTPVTGPQIQMACLGSVYVVAYLMFFVTFAVVLT

YTIKHDLGY

>34-9_0659

MSFKNPFLQLGNDVEDSEILITQENIKTNTKNHKKEDVHPKSDASKATGKKSNKLESNNN

KAVSPVQGQRSNAKGSGRERQDKHSKSGKTDSNKKIRQGWGDAKTEIDAELDVDAERAAK

ASDESVATEAEEPKIPTISLDDFLASKQNTVVSRRANKAAPAPVASVEIKELKGKKSLFE

AEEKKVAEQKKSVEATGPVFQITHVKEEPKKRSPSNQSTRGGKRAPRGQSRGQSKPAAKS

SAPLPTL

>34-9_0660

MNSDKQLSHQQLLIQRNNKITSNIDTKMIIDILSKENDNNKTYHIRNITKGIANKYPDFD

NLSISKQRRVIMNILENDNTIFEKVGWGLWRLIDGSKSPSLSNDIPESKPRLPSISYDKN

IIKPLSNSTQNIIMNHHPTHNVVKIRRRSSVTSAGGTNDHILLTSPKSNNHFTQEKGFIM

PLYNSKTKERQPSISNINLQKHPHNQFNENALISSEEEDANNGTDEEDWKTLGAENLLVN

QRRKSYGGIIKLQSRKNSTVEENAAMLLVQLRRGSQ

>34-9_0661

MLLQNYDSKESILENVSSITFNALRELKCWFYPYIYNPLSIERLNTHIVSYFPLFKDTFG

LPTHASTSLFLSTPAKEDYEKIKQKIENDDSQNNYIFHIQTKETLMENIKLDASLHLKLE

IQNEKTEAYDHATASFIAKNSYSYLDKGVTNFYKVDTYYHHWQLKDNILLDPINKKDIAF

INNTKILSLDTTSNKVSELCDFRHKIVNFDINENYIVACGYRVMSSYPREYTSLFFNKFD

QPLGRPSERISSKSSLGKQIFFISSDEENKHYMACTKEDIDRGTINKGVIFVHNRKTGIT

YEFSVGSYMNNTAKILDSTYINDSEKENFLLTADLKVLVTNNDGSLYYLAFSNLDFEIIK

IKMLIAGGNSSYESLNNIAISDTSSHVVITTDSNYFMSFSMKQLIESLQNTTKSNSTEFV

LSNTIHYASSGSFAMNAIFLKKSAKVMVSYQNGRIHIVDLSNSGIQNIILPFREFEEAIR

NVVVDSKNNIYISQSSGAVHIYTNEQLLADSNSALNIVVSHNQLNFPKTSYLHHLEDTQI

SSEELDPIDNEYGFIKHKIAPYLTTSYDLYNTYSWLKDRNNVINREKTSEYAHTLSAIFL

DCVQDDNENTLKIVDHNTFYNLELGLSLQSKLRNEIYEQLFELERLVMLEKDNLYVGILR

ENEVKINRYYNSIAKLYPETYARIMNMCDFDLTQLLPFDKIMTRSYTPYKYDLNQYAKLD

ILKRDSTLFKAYRGKFPNKNKNLVINRPDFPKNYIISDKIKIESKCYEELIVDTQNEPES

IEFKKSSFGEKLGEYEDYQFSILNAFKSVKMDKKHRFEDRPNNKITGMSIRLFDHEEQLV

VGTSTGIYTIPI

>34-9_0662

MPIDYSKWDKLELSDDSDVEVHPNVDKKSFIRWKQQDIHQKREERKNEIKILEQQLEMYK

HLNKRMDYVLSLQKKDKDAVKVFMNVEDLEKLLNANFDKLEKCEGDRIDSNIPPYNEMVC

DLIEQFHSELKRDDKLTLQAVLFKIIEHRAKIEKVSEEANAKLAELYKEKELHISSEDMN

DAWNSSIINKSSPMTAAEVEKVKNEQQVKTQSLTKLGLKKFISFSGEENILTVDEKTLKF

GEIPCKNHEMGKSYLLANYEILSAQQVDALMMSSFNHELEGDTKKCYNVIYQSEVMSSIS

QIYDLRFKNDSSEFIHSEELQDIIVKFYMKLDDESSTNMARPFFMSEIEKKYNHVKTRAV

AMKEEDEDEREGQEIIQLKSVEPNTELKINVPEFKDENQTEDEVEKHRIQLFNMIPENMR

NALISGSLDKVNEIFASMSLEEGEQMLEIFNAGGFLGIEKTFENQEEFEEYQKNLKNEEA

Q

>34-9_0663

MSDDEFDDNVEGNIGVMGDGTKTEAQSNIGEKRKHEEEENSSEEDSEGFDSDASDYELDE

SGESKREKRKKAKTNPVFNAFNFSRKDKSLQDILEFLDEQPPVIPNVIVDYYLKKNGLNL

KDEKIKKLISLATSKFITDIAVDAYEYSRIRSGTAVYNATNGQQKARQLLMGQQQQKLLQ

QELNNNTDMTNMQNKGNSDANVNKANTQQQHNDKDRVVLRMSDLSSALKEYGLEIQKPNY

YR

>34-9_0664

MQILVEKVQFENDEDSLVIFEKQDINIGKKSLHLFKVCGDIMFSVYKININNKPNFLIQK

ISLQDPDIVTNLQHAFVQKKGNKQDYEKFLFNEVVDIFKVYNTKDYQNNILIIKTINGDK

FEYFKYDHNNSEIVYSLTELNKRSLDISCTSNLFNSASDGTDKILLGTANGKVYIYTLSR

NEIIKLNQYTATIKGLHYCSKLKELTCIIGVTIMKYKNIKEINEFNNDSIPLEIENYGSD

DVNKQEIENIQVFENIKREQFLVNYKNELVVNKYTESLSESRILKKNDTFTKDNYVIDSC

LTNYHILNITNQNKLNIINQFNYEDEINIEVEHSLADSMFLEVDYISNDLKPTYWLYDTA

NNVFEIILQDENLNVVDDLLKLNKFSEVLKFDLNDEEKKQSIYEQYFDYLLNEEEKNIDI

LIKVAGKIKKNFTLKMNQIIMLLSTERYKDTGAIKIIDFLKLRLINFNDSMTKNQIKLLL

SLIVIFMTQLPASISNDKILREFLVQNLEILDHEMTKQLLAKSQNNLIFFMKLTKDFKSL

IKYYLIENNNFIEAVKVINVYCDDTEIIYDTAPILLQNCPEEIVKTWVKLIVNKTISIDI

NRLLNIFLDYFHKVYSLKANNDSIKKMPNFCLWFLMWYHSTIESVNKNVNDLIMYMLIND

KNEFDLDKIISFIDNNWEYLDRFILLDMATKMDKESTQSKSIDVVVYILNRMKMYLESVK

LALKEGLLDTARDIINNIDEDEAINDFDEFNDIRKLKRKLWLLIVGHTLNNSYKNNMNLK

PIINDLIRESNDIINISDLFPIIESMNLTVAVIKDEFISNLKAQNRELSETNKEISNLIN

LRQKILEDIDYLNHNLSHEISESDVCAYCHDNLILKKKFYCFPCCHKLHVNCTLDLILKG

GDFGLKNQIENIMFKKEYKWLDKLNLLLAKKCPICTDININNIDEPFVLDVASSDKNNGQ

VVGGVSNSAIDYLKF

>34-9_0665

MSKVFFDISVNGSPLGRINFNLFKEVTPKTCENFRALCTGEKGFGYKNSVFHRVIPEFML

QGGDFTHGTGVGGKSIYGNKFADENFALKHDTVGLLSMANAGPNTNGSQFFITTVPCPWL

DGKHVVFGQVADKESYEIVKKIEALGSPSGATKATIKIEDSGEL

>34-9_0666

MAKTKAGVLGATGSVGQRFILLLANHPDFELVKLGASSRSAGKKYKDACNWKQTDIIPEY

VENLVVVECKSEFFQDCDIVFSGLDADYAGIIEQEFADNGLGVISNAKNHRQDPEVPLVV

PIINPEHLALVENKIVKSKEQGTNKGFIVCISNCSTAGLVAPLKPLVEKFGPLKYLITTT

MQAVSGAGFSPGVGSMDILDNVIPYIGGEEEKMEWETKKILGSISADGLTIENIPESELK

VSASCNRIAVIDGHTECVSFSFKSEEKPSVDEVKQCLKDYQCEATQLGVTSAPKQTIHVL

DQNDRPQPRLDRNRDAGYAVSVGRVRKDTVLDFKMVVLSHNTIIGAAGSGILIAEILRAK

KLI

>34-9_0667

MSEDKYIWKRIENEDLIYEHINERYALMSENSNKIKQDLGIINDKEEKVIIEIDYITKDK

NLTGTKKKLKQRASNYRIELNQSLTSLKSNTNGNSTTGFVVWQSTYFFITWLLNHDGLKF

LNWESKNINIMELGSGINCSNSIILSNYCKDFFILTDQKGILNKLQENCRNNLKEIKHYN

QSEKFLTETRIKEANFSGSKHPLFCSKSLNINDFLTNNSDSDLFHYEVGLLDWCDNTTYQ

EFIYNYGSIISGTNSNMTILAVDVIYNEFLIVPFLKTIKDILTYKKNADALVVLQLRDES

IIIDFLTETLEIGFNLETVEDVEDELVIGCSRHVIYRLSLSPTS

>34-9_0668

MSTNSTNFETKKLPKNLNAKKRNQKQTGNFPFGLYQTSPLVSNSDKLEKIDLTMKKPIES

PSFMKGKSMNIEPKKFVQNSWDLSNQKKMEKLMQVSKSNTQGLLDQLKAMRDNERKKMEA

LKLVDSADASKSLKDAITFQGSCMQMCPIFETVRRDVENSLSKIEKDSQGNVNKQKAMKV

FSRPAAFAPPPLPSDVRPPEILIKSLNHIIDNLLDLLPENESFIWDRTRSIRQDFTLQNY

FGPECIECNEKIVRIHILIIHTMIKSGIEYSKQQELEQLNKTIVTLCDIYDEGRKKGYVY

PNEAEIRSYRCLLNPRDASIDVQIQSLPEEILNNDMFDISMSFRRIMSNSLIKERGYLTK

KKNGLNLVNAFFQLIKNEKKVPLLLAMFLETYVNEMRYYGFLKIKVSWSKKLISTVSFDT

VTELFLFNNDEEIKEICNFYNIEYDTSNRTLNLSTLVSDSLASVDKKKVLSDVSLQFIDD

RLAKIGSRSNLINMNRKQINIKETFESPKEKKIVLKPLMPAQTTPLTNEQKKDLRTINKQ

EVKAEEPKTTPKEAKPLSITKQTEKKENYENSFNKNKVIINTPMTEFKRRSDLFDQTPSK

ITPNNPTKIVNLEVEKKVVSDSPGFNVDEVKKNTYKIIYEELLAETSDMLMNKLAKSIAR

NEVIKAKKEHKMKAIHTVEPTKDDVAFDVRDIVVDKQQREMLLLNFLKSPTIENKIKTFS

NKNENKNLFLTPLKKVKRKLKISKELFEKSKQDMLILKNYINHVSYNMKSNTWFNKFETF

DICWTFYTSDWSDVSIKAILKMLNINRIPLLKSQEKINLKVCKYGINEDMKNTQLLLFNT

GVNTNNIFDFKEHMESERNKLIDILNQLKSKSCIKIDVLILFWKNNGLDMFDLKFFEKYE

FVNSFKVIDIEDENVLKSLELELNKVSINFDFKLNGEIGKDIITAKYNDEIINNDDNLIS

MSNLDEIQRSLRKAKKVREFKHEGRLFDETSINKKRKLSTFENDQKKKKYFTNVTEPLSS

DSTFDSPSTSLLVTKNNIPTLNFGNQNQRKSTFNYKNNNNSRRMITPTSII

>34-9_0669

METESKTTQSPKTTKNDYNMMSRNLIFKRSLGLTHKLLKPTSITPSIAYKAAPSPDNYLK

TLNKSDLFSLFLIGCCSINKPILNTVIKIFPYVPLPLIKRFIYNLYCGGETFQEVLNTGN

KLKKNGINNMMLSLTIEDSEGTKNIDIDYIVNETISSVKNILKPHLLNQLNEKNINDIPP

GYIALKPSALVKDPYNVLLNYFLDSKDPHHKKQSLELFNNCSKITEVVKDLNEELSKIYP

TRASPFFISTIDAEKFDLQESGVYALQRDLFKKFNLNKYKHISVIGTWQLYLKGSKDLLL

KELEMAEKDGTYSLGCKLVRGAYIHSEKHRNEIIFDTKENTDINYDDTIDYVSNHLLNNK

DSKFGHLVVASHNYNSQLLVTKKLNANRSNVNNSKIVLAQLYGMADNITYDLINHHNCKN

IIKYVPWSNDSYETKDYLLRRLQENGDAIRTDNGLPLLKAIFRSLV

>34-9_0670

MLRNRISKICQQRLIKQSFVNKKDNIDGFNQELLAKLQSSFKQLLDDDPHRNTHYSSDFS

NDGYLKDIMAKNRLTDTVTHLYEDPELKSLNIDEIIKNNKDSKNAKSEKFNDIISHIDNA

NTFNNSKEKNSLIAVLKKQFNIDKIDEIAQDPKRKLKHLPFNNGIFDLKANEHEIVKNND

VMSNLETFNLDKLTFSDSLLNVLKFKGVLRGYLVNKFIHYKKNELVKYFNKNIKNLKKTN

KNLSLVTRKRTKDQIVDDYVDIILDGVKNPVSKCNYISLFIRKDLYSLMLSDREISKYVS

AYNSIDNSNASVAFIKYVPNLNDWIELICIYDSQMIDSSMINKIQNFKNTLIYQYMDNEL

LLDELKSTLQSNIYLLHLLKSSSQTFSISELFEEFKDYLRYGKDYGVMILNDSNNSEVLI

YTDTALQSGTWVKKFINYKLDELMSSQQGAIDNGMEVQYQNLFDVQDKESVEKKYYLVNE

KFSALEPEKDLSKFNKDHVYYSHEYSGSEVTLHSLLSPQIENLNMEKQASMDPLCSNTDS

NIKLEIDVESDNFISQKSIVPGVILKNEYSNKSIFTSRYKQIPSLMKALHHQTQLFGNEV

KNNDSLNYINMETETCIISHYPYARDNKLVTLDYGKVLNIGDVFLPPLDLLINLKKVQCS

RRTKKMLYQLDSNQITPMLLINQKVNFFKTVPLNYDVKYNQEFVYLLDMENVEIDNQYEC

LILNNAINECKLAEKSDMEEMIKYVRDEEFVHQYQENLELQENKTSEVSSEESSDEGVNV

IKVKLPVKIKIDNKWVKDHILVQYKMNSYKLHSSSPVQNIHDIKFDRNSLIYSKGFTSKN

EKYLSYQFENSESLLGIEAFFK

>34-9_0671

MTATETQNVLIQTDDEEDEYTGFISVRDYAYDTENPLHYGYLEEDDEEEEEDIRKSVSLP

KEYIINKKGIAMYDFENINDNELPLTKGDIIYVNYKHGQGWLVVQKLDDEEQTGLVPEDY

IKILDDDL

>34-9_0672

MAKSYTDDDVLVIIEKCNINNTNKQYKKTVKLIKPLLSSYNFNDINLKILILDGYLQALI

ELDLIEDIYNVTQELIQLDPLSKNSNNNKYFILAQIIGGKKGIDLYLNGINNITNSDKII

ECYLSIVDIYMTDLCFEVDAEQQCELYLNKSLELNSQNFMVYSYLGSFKISQQSFNNALE

FFKKSWSLFNENLDSIDMDKSKILQYCYNLIKFLLELGDLSLIDSILSYSKQLEDDNIEG

LYLEGFYNYLSLKMKLFNINNSVDINHSYTPDQFMENEMNVHILNYNIDAGLVESNGILN

ELFIDAKIAFSYLLLLVDNLNLQNQALISDDFLEFYEGSKNILINELYGIMDKDELIKLK

NGDFINEAEDVDLDNEILQ

>34-9_0673

MNNNKQVVIKSHSKIPVKILTESINYPVSVVTIKNKHNKGTLKSVVNNTLNVKVDDKIIK

GNDIKYIVLPDIMRFNPLLNEVLEYDNSKIRTKKTNSKKKNKKKEDKVDVKDREIKALMK

ELDKRENRKRQIEDEAQGTKKQKL

>34-9_0674

MDNLFEQYNINHVKINYDIEDNVDVNLYNEFSVNKLFLEDNIKEPISIINNTYNLDSYYK

NNKKVNTVKNDDPIDKSELDLKSEYFNVNKYLTAVKTTNLNPNNDLLKASINNNINQILI

LKKNVNSIFKNFNHTLNNDDHFNNILKKLNTSINDLSNYKNYKNNQLSQLVKFKNDFNKV

KSNQNMMLLFTLPNILLRMYELGLVNEMIYLLILYLNYKISNNRPIIINKLDSEIVKVII

KLKTKYYDDLDNMNNFNSLLILNYQCNVMNNFIIDYEDEVLNDKDIIKYWMKLKLDKLID

KIDNIDKVIFYNDYGVTVHQDNENIIKRYCKNYYNFLLLDEDHTSNIFNILNTSIINNKH

SNDFWKNWNNIITIIVPLLNELKDFEFVITELENKNLEDYVDDDNYNIINTDSESLNNDY

YKIIKFLSVAENNSFVNKKISINENINGDIEVIKTNIINKIILFTDSQLSKINDKSKYLN

WIKSLTLFYSDILSIHEDIPFDIMSIKFSIINILDGLLIKDIDNIFLLEDWVPLKAFNNM

TISSVLFYKIIEFHCNILITRFDSKDDKFIIDCINILITKYSTLDKELLILNNIESLLKI

EIISSNQEATNLLSDFKSKTINKYITKDCLKLKIILNTYTPYKTSEFGNYWTTFTINNNI

KISNNIVGALMNLDIIKNDIISRFSSSKLINNITENFLQIISDYFIQILDLNHDSNVSID

TLIQIGVDYLHILKHIEMFDDLDIKNISNDDDLVMKFLNKVWIIGFNRNFEDFNQYYNEI

CKYV

>34-9_0675

MLKHPVQKLASNQKGDLLYTLVKDTFQVYQTGTDKVSKIFEYKDNYNAETTVEETVAEEP

PVKKSKKSKKPSNYQPNKHQLENYEFLRNLNVSPCGEYIFVISDFDKSVLVFKQLSNEGS

YTYEFIKKQSFPKRPNCLTSTDKLLFIGDKFGDVYELDYRSPEILKAEDMKPILGHVGLL

TKLEIFEDGSKKLLMSCDRDEHMKLTHIPQTFIINKFLYGHKEFISNVLKINDYVLSSGG

DDFIMMSNWKTGELITKFDYLSLIEESDIVPEIHNALDRFQKKDEEKVKEFCISDMIYDE

DSKKVYFAIEGVKKIFALQFDEESKTLKLENTIKVSNIILNFAFLNIDNKKKLIFNTNRQ

VENQLVEVLNTENLELDAISTDLINNEIANNTNDEHYNVVDKDNDMCPININHNLRKHVD

FY

>34-9_0676

MPSTRIKYKHISNVISNVDVLIDFAQINRLYKAITILYHRNKNQYKTNFILLLKSIKQFR

LLLLKVIILINDKRYDELFTFLKQKYILKKKVLKKTLYQLYNVINNKTFINMALFMITLI

SDVHNILVVNFLNKIENELALKGLEVRYLNKNKFRIMKKLDNIVKKVEPKEEVTFQENVD

IGELIEEDIPPIEKEIEVVEKDLTKKKDKKKKKKKKSLIDDIFSGF

>34-9_0677

MSLIELQKEYIIKIIDDIKTFNNLKFLIIDKDVQEIFDHIFPKDHNNTHNNTLLKHVIHY

DLIDDVKRQGEHPIECIYLLKPTKYNIKIMNSDFSQFPTKYKKCHIRFLPGLTTSLKRFL

DNQDRLPYFIESFRECKLSNIIVQNHCFKSLNLSQAFQVIYNKECLDAVESYINYIVESL

ISICVITNEYPIIRYYKPSEHQQEKYNFKNVSKLVAEKLQVDLDNYARMNDDFLNLNSSR

QRSIMVICDRTLDLISPLIHDFSYESLVYNEIQNKNVFNNETDVFNYKVENETGDFDDKS

SIIRDIYDAIWTELKYLHIIEAKDVINKKIEKLINDNPLLVDRHNVKNSSDLLSVVAHLK

MFDEERRQLILHKTLIEKCLEIVKEKKLAENIADTEQIMAGFGKDIDGDNCSKNFTLEYL

IENVLSIEQININDKIRVLALYCIYRNGIIEEDLIKLLNFMEIDYQHPFFTHFIEFIKNF

ELLGMNLIKPNLKKSKLKIFKSNNYSPYLCDEVDGRRKFYHESIINNSEIYTTSRFIPAC

GVNISKSISNALLLPEDEFKFVKGIPVELYEMNEDLEKNDQSKEYPIEEILNSSNSRSYK

VKSSWNLKKENTNNIERQRIFYYVIGGLTHAEVKSAHDQGILKNKDVFIGSDEFMTSSKF

MEHVLNVNRPRNVLNLKIDEKENESIPDFFKFNNSRERIPNTESRRPFEQKMYKEETAHP

SEYMKHAEDEVVASNDDTKKKKSSRFRKMFK

>34-9_0678

MKFVGLISGGKDSHYNILHCIKNGHSLTCLANLRPSDKSQQELNSFMFQTCGHDLLSKYN

LVTSIPIEFHDIHEHTSEVTTLTYEATSKTDEIEMLFNFLKSIKEKYDIEAVSVGAILSS

YQRNRVEDVCNRLNLKVLSYLWQRDQNDLMREMCDFEEKTKMPFDAKIIKTAAIGLNSED

LGKSLSEVYPKMLKLNKMYDVHICGEGGEFETIVLDSPIFDKGYFKLKNVNVINNNAEND

DVYSCMLDVEVLTRERKLDNLKDMLIDMEQPGILSSKWLSVENQIKKENFNISTEVMAVA

NETPIAQDYEMNVCKVGGLLHIQNIQPTNPSSDLDAQCSQVFKQLFDVLKNHNISKRQIL

TTTLILKDMSNFQHINRNYIKFFNKIGALPPSRACLGSKLIKCDMQLSVILSLDDKLEKD

GLHVQGISYWNPCNIGPYSQFIYPKVKGNNNKVGYMAGQIPLISKSMILVNKFEELNSYD

RKDWIRDVVLTFKNYDFLKNTINIKNNLLTTIYISKEYTSLSLRDKIEVVKYIWDITCNP

YKVDIDYDTIDDHKFYYEKENFDEYDDLDIENLCLVEVDQLPAHAPIEMGGVVCQTLDSR

IDDGEEDLPELIKSQKGIDLLSDDNLRYNYSTKSFNSTDDFLDFMHLEHKYPFQALVIKN

FSNLKT

>34-9_0679

MNIDKITKKISNLGLHDIKNAARFTQNLLVQYEPYQVDIRRATNTDEWGPLQKHLMKIIR

HRYQVPMYLITEYTLKRLVDHMANDSKNLYEKARKDYINYGLEYRVVLKSLIVIEWLILN

PPTTKDLQDVLKCLKKHNAIFQQKLPQYKVLVAGDGKMEVHTRVIHNKNDNILMLMTDKD

FLRQERLKYKHQQEQINKSGTMGSNGENTYPTTVEEEDDDEEYYTPNKPARSGSIVDRQR

SQRRQILRDQIKLQEEKRKQLEEQQKQEKQTDLIDLLNFDPVAKPEVPVESNSFTKESSI

PSKKSVDTADENYENVWNTASTAQEDEFGDFQTESSTQSNVNIFADKKKSSDPFGDLLDL

I

>34-9_0680

MEEIREQQVQELEILQTIYPDEFFIAPNLTDYNLNKDDNDYEKIDGPLLSDDESESEYEE

ESDFEQEEIDWSAYNLENHPNPLIFQIKGLKVDFIPPDNSQYTVDSIENNFVHKVNLKFK

LPLNYLEDTEAVPFIQIKPYVEYNDEYIQSKYADQEDDDEVIFDDDGDVVDKDGIDIAFN

CHIKSSTFFNMLIGENTKDLTDLDNYDVVSWKSEMLDNYMLGGTPMIFNIISYLKENFEA

GLTKQLSNFDQIHQEKLNQKLEIQNAKFKGTTVTKENFAEWRLKFRDEMNLDRRDEERKK

LLHAGRLTGKEIFDNGLANEDHEDL

>34-9_0681

MSYSRAPMPLPNLTNELNIFGGAKITTNLEIPQNFVSNKETPTPNPVNHSNKFPLPTNDH

ITSLLVPSENDVPSYVQPFIYTLLQDPSLGKKTSLALSSSHQDQKVIGDHYAYNPVAIYE

LILKNLGICEVIEDSIVFGKGKVHMVQGQGMVTVEVANNPDIVSVDSQVGIRSVYDIKGG

EGRCYGLQLNNNKKWIVYQEGLSIKVERNEQTGMYQLNIMGNSGIIQLYPDTQDSNSLLS

MDFSNLYNLLKLGNYAQKLTISTSSTSKDTLQFNYTMKHTDREGLIFLLPHHHQDLKTDN

VKNSNLKLYSTVYGFMHGYYTTGIVQFDYSSKEDDLSDFDILSESKTSILHQKLNNLLDN

FSLIKEILVNNDVAECLNNLQNESNLDSMYFSGKIFNKYAWLLFISAELFHDTELSDKIF

AKITECLSKFIQNQQILPLLYDQKWGGIISSGNMSQDFGNSMYNDHHFHYGYFVLTFAIL

GWYNPEWFENNKFYSDLLLRDYCNIDDNDNKFVKFRNFNWYEGHSWANGVWPSLDGRDQE

SSSEDYNSVYAMFLYGSVTKNIKMQQIAKLQLNVMKRSNNKYFLYKDKDFNNEVSINEMK

KNKVCGIFFQNKIDYATYFGLNVEYIHMIHFIPGTFITRYIRDNEFVTEEWNQKLQNINI

DNNGWNGLLKLNKALISSDTIDFFKNKQFNKNNLDNGQSQTLSLILALLWNN

>34-9_0682

MTDEQNNQTQSLMHQTDAIFTILSNENVDSTIKYARTKVQSQDDDQISTKSETPNDFSFD

FKRLKFNNQPVFLIYVTVICVMLSQEILSSSSFLELQLKKITGHSATTQSTNEYLSYMKW

SGIVFTVLQLFSVYFFGSILNKHGVRFSAFLFLFFYLVSNVVNLFLMSDYYTFDFYSYTF

FRLIANVYGGLDVMVTIINAGIADLFESHPKRMLYFNYLYSIISVVTFLVPFVTTFVIKK

FGNYYTLKVQTVITFFNIAFVHLLLNNKLKSRIETTDDDSINKNQVKITKKKINTFSSTK

SKTEAITKNNVFTNIIKQFEVFILPKNTGIARRNVMLLELFNIFSTINQASISVGVSYLL

VAYNFNAIQLNYLTSFFGCYGSLTSFLGIKIFYYIVNKFTSLKASSHFFDRIDRIQLLMN

AGASTIGYLFPLIIRHSWLGQIFMMLCFDSVVFVAPVLNNSIIKIIENEKEYVKLQADEE

NDALLSVDDENELEEDGENQEHEEVSPPKSKTAIIFSCFTIQNKISTALFSAVLFQVLEM

TKDTRPWLFYVVSLILSSINFFIASLVKPVN

>34-9_0683

MSINSDITLHSNVSQINELNYILNTSNNTNVFDYYSNQNNSNKYQSNQYLNDDLLSYKNN

IDGMYMDNRRYSDNVSESSTVYLNQLNNYYHTEKQSPSLERFVNKPQIQQDNNKTNKSLF

KTELCTKFVLTNQCPYNEKCQFAHGIRELNVRTVNNKFKTKLCKKFSKTGYCRYGDRCQF

KHASAEDKSYSKIDMTSDSSRHLYVRENEIENGETNKKWVANVKYIGKVNNW

>34-9_0684

MEKNYSDDLKSEFDYDPIEYTAKNEHINSFILKPLSIPDDVDLTITRFLNVLTTTNEAKA

RSRELTLKPHLAGHDLLDVDSSSIPYIISKFEEYGLNVYNESYNVSLNTPIKTSLQLFGK

ELPHSNSSVYSSNASKLLYEASLFEDELPEDPYPETPIYKGGWHGMSKNGTVEAPFVFVN

YGTLEDFNLLKKNNVTTKDHVCIAKYGKIYRGLKVKFGQEIGECSAVVLYSDPGDDYYKI

KDGYKAYPEGPARNPSSIQRGSVLFLSDLKENVTDFENIVPKIPSVPCSYASIKPILEQL

NGHGLTCDKISALSKDKDWCNGAIEGLDYSTGPAPAHLGLTLKVENIQDYGVKTIYNIIG

NLSIGVGEPKTERGYILIGAHRDTWSAGGADPESGTTVILEILRSLKFLQDTYAWLPKTN

IVFASWDAEEYGLIGSSIYASTHAKDLKSNALAYLNVDVACSGSSLSVQSSPLLNEIIKK

SLARVSYPRDPSLNLYEHFFGHHERIGSLGSGSDYTGFLEHLGIPSLDIGFAAGPEDPAY

HYHSNFDSYYWMSTFQDPDYKYHNAISQAMGAMLLELSENELIQFKTHEYLELIDGFYYK

IAEEVPEEWYDFTPSHSHKKSKGHGKKKGGCHKNGKYKKEDHVDKKEEKEEKTTDKNFDN

KKEKEEKNADKKMYNKEEKKADKKMKKHALKSKESEKHKKHDTSKNGKKPHKGKHPKNEK

PLIELMNDVYDNILVKKNAALKIDYKSDNLNLDLKNIDWLPYWDQIAIKYRILGQNMALK

YFERKFLDKEGLKGRSFFKHAIYAAGHNYGYASNELPGLRDALDESDVAEFYSWLEKLNL

IFK

>34-9_0685

MTNNEDSVNPVKRKKSLKNILSKKDNVNTEQLENLITENKKLQEDKLLIEQMNKELKQQL

IEKEQEVFVDQANNVVLTTKNYNDLVNNTIERLGKDDICSYVRNKYKMKVISNAEYNVLS

KPVDESYLKNEASHLGYKLLERQDYDYMKSKMETPDEQTIRKTVDKLGLSILSTKEVEHL

KNPTKAEMERKLKALGFSVINENANIRHSNSDISQHSNNAISPPHSKNNSISNPNLNSDS

NRNSGFYNLRNSSRNSLNSSRVLASRDFFEQVIKEENSSNDVVLEAGRSLGFVRLSQEQY

KKLLDGQRDKILTKTDIYSGAKDFQLTVLPNEEYKQLLKNRKKRQSMSFEDLNEYAKKFK

LKLSSISKDETVPISDTDLSTSSKRASRIFSPVNDDNSNVDVDSKTLSRQTSNVLTFDII

KDWCKSNNYKLTSGQDFDQNVEEYCKESHQKLVTEEEYQQIKTPGVISLEQVKEYVHKND

LKLLPQNHYDALKAKKDGKFLADYETPEIIDHLKSKNANIHITDKETILNLNKQVKQLEE

KEVSSEEAKLVLLKTHHVLTNEELENLKLENKNAFTFEDINNKFPDYTVIHNDDYDDLNT

KLDKAILMSQKKKSLEDIHEVVKKEFPEYELIEKKEIKDSIENTKKLEQNQNKKPKTLDE

IEVELNENFPDHMVVNREEMFKALEDNTMSYEDLVTVVNEQYPDLVLAKKDFLQTYEKDM

KELEEEITQNHQKEIGNVKANYEDVIDHLKADILALNSLENLQEIVKSKFPEMALIEKDA

LNNANNNNIKNVDNIEHLLSEKFPHMTVVEKDDYKANLEELAKSIQNNEFLVQDLETCKE

KMKYLEEQVNDLDKLTNYVNTNYPGNSIVSDELYSTITENLSTVMKDHEGALNDLKEAAS

QHEALVEQLNDLSALKEIVNNKHPGHDIISTTEDALKDHVSYFHPKLSVIESDKLKSIRE

AGNNPVEALDVDKIKVEDVNVLERIIEEKHPSKMLISKQEHQDLVEAVSSKAEKSKESNS

EPIIQSHGITEDDLKNPEFVKLSANKIGLDVISQADMTDMSNHFAEIGKQQIQLNELEKI

ARIKFNAHVLSVEALENLKKNPEINLNLLSETAEEFGLILMSNEEAENLKNASFAQTTVN

HELSLEEIGDVATKNGLAVIPVDKFDDYQNLDLNREARRNNMVLLSIEDYTNLTTNQSVV

NDSNYNNTHANLNDLLEEPDYQKEDYDMEHQNDDFEFNNSEAVPDVHMNEKSIERTKAFL

NNIEDDTSSLSKVETQESKVVLTKNVYKELLDRPEMTKELLVQKARDYDMIAIDLEHFAE

ITKANMKQNMSVQDAMILLQDRGYISLPSQDYENIMNPSKDEVATLAYNKWNMVLVERSS

SLATRNLQDKSRIISGNGSRHASLIQIQPSVSNSGNNTFNNDTSFNNGMYESDQNKENGP

YLFQPFQHQRKRSLMVHQQEEVNLVDEAKKQNMLLIPQNMYLRDLHQIPPLNASGKDNGL

LLVERNQLEDLIMNEKTKQVEVPANNSAMTHETEVNTIESRGKFQNDKIIISLTQTVIGE

YLYKYYNRFGNMGNKPRHERYFWIHPYTMTLYWSLKNPSKDGQNVSSDSGSNHKTKGVGI

VGVEVVDDPNPYPPGLYNKSIVILTSDGKNVKITCPTQQRHKVWLRSLKYLQNAANSKGD

DGTGSILTDSRSVHGFETRGNKRLSFSGMRQPSNAYEGRGNNRMTPETEQHPKFFVNSLK

>34-9_0686

MKKKLKLKSKVDSSKFLYNEDLHKEEYDGQNTKLEPNIVDLEFNSSLSPEKKPQTLSKKK

NNTKSKKLFKLRRNGNTDQDSQTDAEKVYSKVNLDDYRSQFQLSYDKLSQKYYDALDSPS

KIPDIYESSTDEDRIYDDKKRIKQEDICDGNDSLLSEDIILPSQQDVEVVVYKKKKNLFN

ELYYTDFKPFSASENDIESNKLRKTNSVIPMTLSQVFEESSSEEIEEINLVDSFIEVSEE

IVVSDDSNTNDLVLLKQNDVLHHDNGKTEDVRTLKKTDLLIITSDDSDSEPIIMQSQFNS

PTKYNLEVLPSTVTDTVETKMSLNEINSMMTDEGIIFNNKDLLSIVSNNDDVKQFLLNSS

YKLDEQILTHLTKQKNMSLLKYELFEIFEKGKPMLVYDNIFKTMKKMIYQTTLSQSENDE

FTQIVLGLSLGRVFNFEYFTTILIKKLCEVDDIDSLKIKALSKLIQPKNLKSLYEKKLGA

NFEMDSKKK

>34-9_0687

MFSRSTLVSVIKQQSAAQLRKTLIQSNVTLKSFKAVSVKVPQMAESLTEGSLKAYTKSVG

DFIEQDEILATIETDKIDIEVNSPAAGKITKLSFNPEDTVTVGEELAILDDEASGSSAPV

AEKSASSESKPAPKTESKPAASGKNVTVKVPQMAESITEGTLKDFSKKVGDFIQQDEILA

TIETDKIDVEVNAPVSGTIKELLFKPEDTVTVDAELAIISEGGESSGSSEAAPAPAPTPK

AEEKPVEAAPKKAEPVKKAEPVKKAPETAAPSAAFSRNETYVKMNRMRLRIAERLKESQN

TAASLTTFNEVDMSAVMEMRKLYKDEILKNKDMKFGFMGLFTKACTLAAKDIPAVNAYIS

GDQIVYREYTDCSIAVATPKGLVTPVVRNAESLSVLEVEAEIARLGKKARDGKLTLEDMS

GGTFTISNGGVFGSLYGTPIINMPQTAVLGLHGIKERPVTVNGQIVSRPMMYLALTYDHR

LLDGREAVTFLRTVKELIEDPRKMLLM

>34-9_0688

MVYVSKFRNYFCALSKRQYKVLAMESSCDDSCVSIISDKGEVLYNKKETLADVIKSGGVQ

PNLATVHHLATIPILVQDALSKTNSKLDGEDIKMICVTRGPGMPTCLTASYQFAKGLAVA

NPKLKFVGVHHMLGHLLTSSMEFPELLEKPFLSLLVSGGHTQLVLTENITKHTILIDMEG

QKAVGDSLDKCARLLKFKGDMIAKEMELFIQSQNLPLDDPKYFKELARASNPFTKPLAKG

ENVYAKKFCFSGLDSQLRSHINKKVIKVDELTDFEKAEWAYRIQHVHFQHIMDKIKLQFA

ETPELEKLPLVISGGVSANMYLRGYCERQLNNKMYYPKQLHLCTDNSVMIGWAGLKMYEK

TKLVNTMNSTVISKWPLSDLMTVDGAVKDKTPL

>34-9_0689

MADSKDTNSNGLLSSLNTDSARFNSSSSIHKPKLYKRTTSSQVSLRRFSILQSDTEISDD

QIQPTLSHHDSDIEVFSNDRVITSNELLSRNELELNNEDSDSDYIELKPHERLHNSYTRR

SNSPLVSEHGLTSAVSTDSINNEKSTVLSNVLNVKFVDYFLDNVKYENSETNVDFKKEIL

QIIKLLFIKTWVKTDLVPEKLVLRKISGAMTNAIYQLTYDSVDKLPSLLLRIYGPNVDSI

IDRDYELQVLARLSNLKIGPKLFGCFNNGRFEEFLEDSITLTKEEVRKDEVSVKVSKRMR

ELHFFVPLTKDEKREGSITWKRVKNWLDNVHTIDEKTLKYVTQFDDFKTFEKHIEKYKQY

LEDNMVNSKLSFTHCDTQYGNLLNVHGEIVVIDFEYSGPNPPAVDIANHFSEWMHDYHSS

KPELLNPQKYPTEREIQLFLKGYNFADINTLKKDIALSRPLANLHWGLWGILQSGVLGND

KEISYNDDNANDEDHGEGEGANIETFDFLLYARQKFKVVLGDWITMGLIDKRDNYSATFI

>34-9_0690

MITTGKVINPDIALRFLDYNETFNANYSFVNIILKKEHIDKLLKLIQQRAQNLASLNFIK

QNYLFDDIESNDTIKYHISLNYNKKKETLNIKETSSLTLKTPLELNINKLDIKKTLSFRP

SNTNSKVFLSLNLTNKELSEIYKTNDLHISIAHVYTENNLEELNDSLENIEFTDELKNVL

EIINSDIQAYLDSNQMNIGISHY

>34-9_0691

MSFNFENMGNSPNDWNDLQSNHHLINNALIYDSSNEPNLNSNNLLDNIHFDTSKIFNGDV

SGIDLPEINDMIDENLGTKNVLFDDDSLLQNLNGDIEYLPNDFFLTKEISSNYLAEKNKI

NNKATRFVKSNSNLTSITPTKSNSFDFFNKNLVDELKDTVNNNNQFQYQLKENLFFNSKD

FKSSEDMLSADKKYMSGNDTSEYTNNNISNYATRDSPSKTKSYQNNKYSILDKYITVSDS

DDEVVPQSHTPNREYLDSADKFEEFDSPTRQPQNTNLYYSQGGQSPVANTNYSIQNSPIR

KNKTSSGYTHYGKYVSGNQSSNFFDAPKLQFDQNNNENFNMLNQASFRKQLIDSLEYEDD

LPTVPFNHNESNSELDNFYSKKQRGYDSDRSKPYINPNQVNSAMIYKNPAQPWNQKPISS

QNETMVSISNHNDHAYNRTNQRLKSHSSVTTSGSTILQENSPYQHENFKYNDYNDKNYRN

VSHGSSVKKQSLGLGLKMNSLTEERSRALNNALHEGPEFHGNENLNNQYQDYPNGVGSGY

NRSAKTNETVVNSGTRKLSKYETPQLQQIPFFANSNNFGLPDINNQANTNNTENSPSLIQ

NKMFQIVKISSPTQEQPLIGVINRSPKGCRKKTTLPPGSIDQHVAELPDKTFMCLFNNCQ

RKFPRRYNVRAHVQTHLEDKPYGCELCGSRFVRKHDMIRHSKTHLEKKFECEHCHRKFLT

EKLVTDHTEKMKCSVLYPKSKKSSPKKKDSDDELISFEEPIISKEFILENRPKPRKQSII

SPVEIKAPLENTFGKISKPVSPKKLQQQNQVKRGKVENALEFNNTMAEKSSVNKVVKKDN

YHFSSVNFGNELK

>34-9_0692

MARSYKRFVQKSQFGAIATTSAPIHVLDANIILKSTGPEVQKQNLKTDEVLQSYKPTQSQ

ITPAGSLEASNKAEPLATYLTYNEQTQLIAVGYNTGKIILFDEMTGEEMMSFVGHSTSIT

ALQFDNEGTILYSGGMDSKIISWDIVSEVGLGKYTGHKNVIVGLKVLQDIENQQDLTRIL

SLSKDGIFKIWDMNIGQCVESKYVGFETFAFNMNLELGIVVAMGPSQEIKIFDLDFINRK

EGEFLIEKTTIEKQSKQRAVSCEFVTFNNLNFLQVLSNDKTIEIFRIRSNEELDKGLKKK

AKRLQESEDHTEEEIAKIIQESRYSSIFHSVHILRSTYKIKASKWDTSLKTLSIVLTTSA

NSIEYYTLNIENKKQITGSEKKYDLQLKGHRTDIRTCDISFDGKILSTASNGELKIWNLQ

NDKNTCIRTFTTGYALCSKILPGNTLVVVGTREGSLQIFDLITSAMIIEVENAHDGAVWC

LDVSMDGRTIITGSQDKSLKFWKVEVIQGTSELQLVHDTTVELGEDVLSLKLSPSDNKFL

AVSLLDNTVKIFFFDTMKFFLSLYGHKLPVLNLDISFDNKVIITCSADKNVKIWGLDFGD

CHRSIFAHNDSIMQVKFIPNTHRFFTCSKDKEIKYWDGDKFEMIQKLVAHNGEVWCLAVS

DFGDKMVSCSHDHSIRVWEEVEDEVFIEEEREKELEEQYEDNLLDQLEDTGDKAKEDEEG

DEGKMTAVTKQTMESLKAGERLMASLDLGIKEIDAWDEYNSKMSLWETNKLTSGPQPLKP

SQNAILMALRKTPEEYILETLLKIKSSQLEDALLVMSFSYTLRFLKFINRLLSNDVKLIQ

HLQILLRVLTFIIKNNYRELTTQRDPALKLQLQQVQSKLKQAVKGNINDLGFNLKGLRIV

SNEWNNVHNLSYMDDDDYEVANKERLRARKRVFETTL

>34-9_0693

MSSNSNTPVNRSVNQYVFNEDIKVTFATKQITLSNKFLDTVTTKIKQMKAQIIQEQDAEK

KQQLMIKCQNIETMLQECLAEYKRKLNEYKNAQQKQQNENGDTNQQSNTNTENSANSNQN

NQNAGNISLKTYLTDEQKVHYVNINTQFQNKSNQLAQAFEQNKKQLELVLERLKNVIPET

EKEEYEELLKKKVKCEQAGKSIQLAYKSLEAQIQDIKKRFYIKASETNPTLKKAMMMFPQ

IQAANAKQNASTDSSNKAAAVKLQTETQSTPVVKAGNATPTQAQNTNNVPKAASAVKPNN

QQKTNAANNTANTSSNVPTQGILKSTISENTLLADAPKSVKPVEIPKTDFQKDPTQPTIF

GGYLGSNPILSQPLNRVLGVNDDINNSTQQTQTTTHRKNVYVPTPQDHVLTKRKLRDLLK

QYNGPSANIDGDVEELLLDLSDNFITQVVKFGGKVAKLRKSENLEVKDIKFALKKGFDLD

L

>34-9_0694

MFKITFIDSVKKSKQIWNIEEAERTYVFGRSCLSFFTARGIAENECYVDIFSDTDTVISK

DQLYVTLSVKNILEIKFKNKLRTFFADQEYSKIDNQKYEYYYIKANKGERLKFKADTNKY

IIRIEYDMNLKQSEKLNENNARKIDRRNKNQNHSSIAKRNFSIESLVNDELKFEKLENGK

SSLKEEILSEAESDGVIEASLQKKFPEQLSIPDVISNDVKIEIESNDNQIAKDTPIFKEK

KKPGRKPNQTTVKALAKKEQQKATVNNLMHTLYEAQKKNHTIQETNKEDNYKEDSIREIR

SNSFKIKEFTLKTVIKDPKLTKNDNDAESSSVNYKRFKKRKFGEIESYVNNTDEAPIKKI

PMKKYIDNSIPRFKTMKDLEVDTRHMSKKTVEKLKQNNTLTISRNKTTETKHLFLDD

>34-9_0695

MSFNSQIITLNDGNKIPQVGLGTWQNKNSEATIDSVYQAIKIGYRHIDTAAIYGNEEEVG

VGIKKAIDEGIVKREDLFVTTKLWGTQQRLSQQAIKQSLERLQLEYVDLYLIHWPVALKS

RTIDPSDPKNYLCIPMNPDNESARDVDVNWTFVDTYLALQHLKKEGLAKSIGVSNFSINN

LKELFKHPLFKIKPVVNQVEIHPLLPQDELIAFCKEHDIVCEAYSPLGSTSSPILTNETL

VSIAEKLKIDVAQVCISWAVQRGYVVLPKSSQVERIERNFNNLAKLSDEDFATVSALKEK

YGEKRFVKPDFTPFKVFE

>34-9_0696

MSVKLENDPLMSNLSLHDNLEDLVNLNISSFQRRFQSLQATDFYKNKFSKFKYIALSALE

NNELKYFNQVISNAIISPMEYNYVYQELTLPIVSYWINKQNHNLDSFVQRLSAIAMIIAF

NTDISSLAEHFVSQNSDFINANLLNNNDQSEALLLALYRLIKFDRLKFLKFSKAEILTQI

MNGEQYSSISKLLSLRIYANLVEMAEAKEQEHLNKIKNANVNNSATFIGLCDNICDYNYE

FLDFNEGNRISWGFSLPQSDNEISNPDDIFAIFPRELLTENVQMVYGKFVIYLEHKNSST

FSSINQQNLIANASFLQSEIQNIIDNLNSNKPTMITGSAGVGKTHIIQSLHKRMSHDNSS

IISIHLGEQTDAKLLLGTYTSGSKPGTFEWKNGVLTTAIKEGKWVVVEDINKAPTEVLSV

LLSLLENRSITIPSRDETIYCANGFNLISTITTTENNETYKDLIGYRLWNEVKIPEIPDL

LELLKWKSDEQLYTLLPNLISVFEEVKSIVSLPSFTSLNKGATSRPVTVRDLVKLCDRLS

ILIENKTIDLKWMTSESYDMIFLETISCFTSFVSEPQAINLLADKIGEILQIQPQRIKSL

ISSTYIPVFDITDDKIVVGRCSLSKNVINKQKKSINDTSFATTNHAKRLMEKVCVSIRNN

EPLLLVGETGTGKTTVVQQISKVLHKKLTVINVSQQTESSDLLGGYKPVNCRTLIVPIIE

EFEELFPITFSMSKNEKFYSLFHKCVKKQHWPNVIKVLNQAFKMAENLCLESINETTTSD

SNNNTTIKKRKMNSVQAETLMNQWQQFKKSYEIFEVQAQSLDKSFVFDFVEGSLVKAIKN

GEWLLLDELNLATADTLENIADLLNDDPENRSILLSEKGEAEAIKVHPDFRLFGCMNPAT

DVGKRDLPLSIRSRFTEIYVESPDSNMEDLLAIIDKYIGKYSISDEWVGNDIASLYMDAK

KLSEANKIIDGSGQRPHFSIRTLTRTLLYVIDIVSIYGLRRSLYEGFCMSYLTHLDLKSE

LILKPYIEKYTIDKLKNSAAVVNTIPKAPDSSRGEEYIQFKHYWLKAGSFEQRDVSHYII

TPFVEKNMLNLVRASSSGRFPVLVQGPTSSGKTSMIKYLADLTGHKMVRINNHEHTDLQE

YLGTYVTDDTGKLTFKEGILVEALKKGYWIVLDELNLAPTDVLEALNRLLDDNRELLIPE

TQEIVHPHPDFMLFATQNPPGLYGGRKILSRAFRNRFLELHFDDIPQDELEIIIKERCQI

APTYAKKIVDVYRELIVQRSANRVFETKNSFATLRDLFRWANREAVGYEQLASNGYMLLA

ERCRTPEEKVVVKKAIEKVMRVALDMPGVYNSLINSQLVNMPSQVVWTNTAKRLSVLVSE

CLKNNEPVLLVGETGCGKTTIVQLIAQFLKTELIGMNAHQNTETGDILGAQRPLRNRSAI

NEELFTLLSSVFNDKESELASMIEKFESLKDLSSIDEESLTKIRSLLKQSKSLFEWTDGP

LIQALKNGKFFLLDEISLADDSVLERLNSVLEPERSLLLAEKGTGDMQITAATGFQFFAT

MNPGGDYGKKELSPALRNRFTEIWVPSMEDFNDVAMIVREKLVSKNELVVNAIVKFSEWF

ALRYGNGNASSGIISLRDILAWVEFFNSSFESLNDDTKTLVHGAAMVFIDALGTNNTAFL

AANLERLKEEKKTCYTNIFELMECEYNEDIIVESKIEKTDEFLKCGSFKIPIDSVNSKED

TFNMSAATTSMNLMRVIRAMQVNKPILLEGSPGVGKTSLVQALADASGKTLTRINLSEQT

DLIDLFGSDTPGENTGEFVWRDAPFLRAMQQGEWVLLDEMNLASQSVLEGLNACLDHRGE

AYIPELDKAFTKKEGFLVFAAQNPQFQGGGRKGLPKSFVNRFSVVYADSLTSDDLNKIAQ

HLYPNINPDIVEKMINLMSQLENNVCVQKKFGSLGSPWEFNLRDTLRWLSLLNKGSIVGE
[truncated: 1,768,007 more chars]
