# Supplementary material for: The Shared and Specific Genes and a Comparative Genomics Analysis within Three Hanseniaspora Strains
Source: Int J Genomics. 2019 Jun 2;2019:7910865. doi: 10.1155/2019/7910865 (PMC6589277; doi:10.1155/2019/7910865)
Supplement: Supplementary 3 — File 2: All protein sequences of H. vineae. [file 7910865.f3.doc]

**Supplementary Table 2.** All protein sequences of *H. vineae.*

>model.g1.t1 Augustusgene.g1.t1 JFAV02000001.1:1-417(+)

DGQQQQQQQQVTGQVPSQNTSALPVTYLPYTNPAQGGIGGVSSIDDFYRRNTHSAVSSHL

KSWPSISHIDDLRAATYSANGGQVEYAMYPTSYPSSYPATTTTTTTAAIAPAAPTASSLS

TGMPQPPSLTNFAPYLDQ

>model.g2.t1 Augustusgene.g2.t1 JFAV02000006.1:1-140(-)

MSLDNFDFKFSQCFGDKADIVVTEADIITSVEFDHTGDYLATGDRG

>model.g4.t1 Augustusgene.g4.t1 JFAV02000007.1:859-1815(-)

MPALKEVVHQLPKRNKNKNTFNFLTAQNKGTIPYNKYGTYEVLYFTNKNGNELNFTNAWT

PADVVLEPENFLQSSDLQHIPKPRNKFIIFKNHVHSYVHQRNAEVAKEKELNKKNSNTLL

LTTELIVKLWSMMSPACKKYFEYLATLEDNLQQNLYPNYQVQKRSYNIPTKFMNSFLQEN

VRLTDKKIDKHATNDLNDSTQDFNLLKSLFKKAKSGQYVTSTHDLNFFYENEKKLEDIVE

KHKNKYKALRKTTYCNRFSVSQTNASNLKVDKKIKKFKSKKKAKIINSSNQSEINTFAID

PIFFVDSAESPNLFNSFQA

>model.g3.t1 Augustusgene.g3.t1 JFAV02000007.1:1-340(+)

PPKTTTNTSSIAQDVALFSKSKYVAIEPKFCNPAVDLDISNLIDNQKPKAKNSVNKKIVK

KCLTRKSNKRLNTQKTLQKNSGRLRINNVLKDVDWIKDSTEDVLQKIHGVLEA

>model.g5.t1 Augustusgene.g5.t1 JFAV02000007.1:2197-2496(-)

AGLNLKSQHGLEDCSKIVNDCCKKLVKHIQSLINAKSADQAGQDFVDFGKLSIHEHKTVE

MEQQVEILKLEAQLDKARKRLGEIRKFSYKDEQEEGVVQS

>model.g6.t1 Augustusgene.g6.t1 JFAV02000011.1:19-805(-)

RNVLQGVIPPKREYLVNCSLTPHQLDYYKYTLHSLLKEYLFVDGIKEFFLCNREKYGLNN

VSNEVLLKYMQYRNANYTENDYDSDDLREFFHKMDILVYNKYIVKQIKYKKYGNTIMQLR

QIVDSSLLMFSPYHHYNTIDGISLDKLLQESGKLQILNQLLTQLLLPQGHKVLIFSQFYN

MLDIIEDYLNLLGLDFYRIDGGIKNEERKKQIEEFQLNGEDEDRESIEKNLNKNLNKNLN

KNLNKNLNKNLNKNIKKSIKGE

>model.g7.t1 Augustusgene.g7.t1 JFAV02000012.1:1-507(+)

LNGQLPFEIKYGFNKKKLKTTEHSSQFIRLASEAGEISINSIQDTQAKCLVNFNKPGMEK

QFRDLSLKIHEKPTVTTSKGDVFEEDIHEGEQAEIIFTFEGEMPFQLKYVRMENEMTLLK

NLKNSNVEIFTVSDIYSYEYRFVTSLQGTYEAIEISDKYCTARNEEFYK

>model.g8.t1 Augustusgene.g8.t1 JFAV02000013.1:248-633(+)

MTKNEDLDFDLEFDLDNYNDNGSPEKKPTGKKTTSTPEQSSVPVPGTDNNSNTETRLTLS

EKPDNTGLYFLLASQFFNSVMVISTKLLETDPAFEDDPIEPFQILVVRMLITFIGTLLYM

YFHRHSIP

>model.g9.t1 Augustusgene.g9.t1 JFAV02000014.1:1-649(+)

TPISATATATATASATPTATANTAKRNRGSNKKTSSGSNSNYVGAGKKVPMHLLEKRRLG

RIKAAEEFSKKLKAVGIEKVEDNKTTSFIKPLNLINLKNISSNYLKNDDQLFAMRGKQQL

TKEKQVSEDSNGDKPAIVIDPGSRFLKIGFSNDEQPIIIPNCVASAKMAENLKSQKSESA

ESAESAESAESAESAESAESAEAHKFLDISNSPEF

>model.g10.t1 Augustusgene.g10.t1 JFAV02000017.1:1-1108(+)

EEEEEEEEEEEEEEEGESIQTYEPEEGNGDFTNRSKRESHKNQQGNHRSSAQQDNGGDDD

EEAKYKNLSFHGITNSLRPNFNNMGRRRSTFHELSNLGTAVKKKFQNTNQQSSNLEDERN

KLSKYVQERYDDSENVRDMSSDLIDTLLAGCPAALLSMTHILQDQRNVQRVPLLLNILSV

HVNPIKSTDSLHSAKFKIDLEYGVGPNRMKWSIYKKYKDLASLHGTFKAMILQTMVENKL

HKEKNTRPLIIPKFPRAFKNDFKNSQHYDEKRRADDRRANSDVLSTSSSNSSIVSVSPTM

DQEVFVPQRKNIFFKHLGKFNQSDETDDPDMPLNERLEKYLKILNLALSLRPAANNLFKF

YEISPISVL

>model.g11.t1 Augustusgene.g11.t1 JFAV02000018.1:1-769(-)

SIDFESFPTNRPTVGATLSADGSTISWSYVVPKNLYSSRVTFLSTVGTGYTFGGLSLFAI

SSDLTDSNPVSVDFTNESTDEELFGVFDAAFPDGAAALKWVITADVTDATLDSYSNTLVV

SFPAGTAVSKRDVEVSVTATAVRTGTAPAPQTLINSTAAPLVPPSSGSAGSSAASSASSA

ASSAASSASSAASSASSAASVASSSAASAASSAASSAASSASSALSASGSAAASSAASSA

ASAASASIYSSLSSA

>model.g12.t1 Augustusgene.g12.t1 JFAV02000019.1:1-302(+)

NNNNNNNNSNNSKHSKQVFKVRRKNGFSPMLTKTWMLLLPIQLHMQIPLLQAITTFKVQQ

VNPKQHSRHNSNSNKLSRKKLVLVVKNQKVRSLKAAVVLS

>model.g13.t1 Augustusgene.g13.t1 JFAV02000020.1:1-216(+)

VLKNAPHSLQDLILSKDWETTRGYTRELAAYPLPYLKTNKCWPQVTRLDDTYGDLNLLCT

CPSVEEVVQHQE

>model.g14.t1 Augustusgene.g14.t1 JFAV02000022.1:1-336(+)

LKRYGLGMFREIIRQTYKIDNNGAILQDKIFGKPMPIAYDYATKILPLENKRKSNIYMVG

DNPASDIIGAHNYGWNTCLVNTGVYTKGDPLPCKPTHIVKDLYEAVMKGLDD

>model.g15.t1 Augustusgene.g15.t1 JFAV02000024.1:1-198(+)

EDDDMEHELDKNLNHISDISGRLRKLAIATGQEVDKQQGRLRQIEDDTDNLDINIHLNTH

RLQHIK

>model.g16.t1 Augustusgene.g16.t1 JFAV02000032.1:1-245(-)

MPSTLAAFAPAVSSPAPSHTPVSAPPIDGFFNNKWNPPIASPAVQPIQQSFSSNPGTPLF

MHQQLNPTAGFHASQQQQQQQ

>model.g17.t1 Augustusgene.g17.t1 JFAV02000035.1:1-972(+)

TTVTGADGKATASTMYTVEVPSANFVATVPTYGDVSAVTTVGTAYSTFTGSDGSSSVSTI

YTVEFPGTAVVSSSVVTGDVTATTTVGTSVTTVTGADGKATASTMYTVEVPKASVATITT

DIYTAYNGTTPTTYSTTTVVATGSDGKETSSVVYYVSTPVGNAYSTVFTPGTVTAPYTYA

TNVFTTVGTDGLTTTSTIYYVQTPIVFVTTDVLTTSDVTSMVTYSTARVTTVDTDGAETI

STIYYVATPVGSSALSSAVSTVSTEYDGTVTSTYSTSVFTTTGANGRPVVSTIYYVEYPS

NGASSTYYTTYGGSVTSTYGTSV

>model.g18.t1 Augustusgene.g18.t1 JFAV02000039.1:544-918(-)

MSVDFNTLAQQFTEFYYNQFDSDRSQLGNLYREQSMLTFETSQLQGAADIVEKLVSLPFA

KVAHRITTLDAQPASPAGDVLVMITGDLLIDEEQNPQRFSQVFHLIPENNSYYVFNDIFR

LNYSA

>model.g20.t1 Augustusgene.g20.t1 JFAV02000041.1:2606-3867(+)

MSSPQVLAMSETKGCNKDNGSSNQQHSENQSGEVNQIATQEDFVSGTTVTTTYAQDSHRT

NWGSSLPETTPSSAIPISQLLGSHSTDNLSVPNATLMNNMNNSLGFGTPPQSFQSFFNSR

PNTFHVANNIQAAFPMTTANSPSISTPPFNEKPFPALSLPSLTSNSGPLPSNTDQTNIAT

ANNYNNNILPDLTKLQPISSTGKKKRNKLIKSCLYCRSKKLKCDKKHPICGTCKSRKLSE

CVYLTPVHSTLNKLKTKNHTVTNQTDNTLNKRKRDSGHFNEQTEDTEGSSENAVRKLKKT

LSKKSPSSNSNTMSVSSHLASGSPVGLYQTRRDIKYEPNKLLDLGCLTMKNERYMYFGAT

SKRASIINSDSTFLLKFFQGWKEFKNERVKVKRLTKRSVLQELRTIERDDGVPILERILA

>model.g19.t1 Augustusgene.g19.t1 JFAV02000041.1:29-1615(+)

MFYLALFLDVFVSIQYGKPLFVSSEVFDEELLLEPEEASETGELSCSTEAPAPAVWKRRL

TVLKAFLYHMRKVVATVYRPVGTPDIMGLVQHLNACYERIFLPLDKYFSFKEKEVDMFDY

LLLPVYSQLQIALSSIQLSLSDEFTIYFQNNIIKHAFSTMKICAATIKRCYLMQVDFEDD

NKKAYEKLTGTKFKDSIKPFILFALYVRYTNLMRAIIEYYKILYRTSTVIKDGNQSLKQI

LDAFVSESNFEVGIDSDVCEASSENDNNVVKIKHEKHFTNDFSLEHFSESAMMTTKYSGL

QLYKWFCVLSDKLFFYESKFFKATPKHFPDLYYMRAVEKITRMAFDSFINNLRNFVNGQI

SELRSFDSHAGTENFKNFEAAGRGSKAWRSVGFRQIIERGCPGEKEELILPKEKNEESDS

KLLNDDNSKNGKTSDTLTDNLETGKTSMSQEHIEKTPYDVHSFATSATADQPSEEANTEK

LLLLPEDLDFFSATFNSDEFSGDLAKEIDKIFESFDFDDNNDFDTILNM

>model.g21.t1 Augustusgene.g21.t1 JFAV02000042.1:1-1070(-)

MTQMLQYFWLALLLATQAMAGKTCTPFKQCQQNNMQYFQNVNSVPYYFDLTQVEYLSGTS

YQITYHVWSDANISLSSLNELKYLGSGISSSNTMIYSLNTGAGMDIDFDPTDFTMSVVVD

TSSANYGSELTGLPNSFGFQFDYCKGMNTYSSPKDSVFYTGNDYTCQCDWHYGATSFDYY

TSCNGDNNCNSQAAFSNWLWPKECADITDQKQQMSSLSSQNPQSYCTYTGTTNTDWVTQI

KNYYTPDYCSSYSTTTSKPTTATSTSSSTSTSTTTSSTSPSRSSTTVTSSSIISVSTQSF

VQSTTQSSASSSAPSSSAPSSSAPSSSATSSSATSSSATSSSAPSSSAPSSSAPSS

>model.g22.t1 Augustusgene.g22.t1 JFAV02000045.1:425-2469(+)

MGVEFNIDYLLGELTLQEKIKLLAAEDWWHTVAIERLNIPSIRVSDGPNGIRGTKFFDSV

PSACFPNGTALASTFNEDLLVRAGELMALEAKHKGAKVILGPTTNIQRGPLGGRGFESFS

EDPYLSGVATSAVVNGIQKSNEVAATVKHFVCNDLEHERFSSNSVVSERALREIYLEPFR

LAVKESDPKLFMTAYNKVNGIHCSSSKKLIHDILRGEWKSNATVISDWYGNSDIVDSIQN

GLDIEFPGPTRFRDPEIVQHLLGCKTETRQGKDFSIADIDARARKVLELVKYFVEIEGST

KLPTNEDDKNNTPETAKILREIGNETIVLLKNDNNVLPLNKKDDIVVIGPNAKAKNSSGG

GSASMNGYYTVSPLEGISKAVGKKLDEIPYIKGCDNHKTLSNLIEQCTNDVDPKRKGASM

KFYTKSSESRGDEKPFDEYVVDKSFITLFDYKHEKISTKDKLFYCTVEGYFTPEESGDYE

FECQVLGTALFYIDDKLVINNKDNQVAGNFGFGSGTAPKSNVVTLEKDHKYRIFVDYGSG

VTSNLSQSIAAGALQVGVNKVIDAEAEIKKAAELASKHDKVVLVIGLNGEIESEGYDRDN

MQLPRRTNDLVTAVLKANSNTVIVNQSGTPVEFPWLQQATTLLQAYYGGNELGNSIADVI

FGDANPSGKLSLSWPLKNEDN

>model.g23.t1 Augustusgene.g23.t1 JFAV02000048.1:1-510(+)

SYVKCAGTTVKSRPLNGAYCVTIGTGLNATNTAPSVSVQMWVDVINNELRKKKGFVEKDL

LPVLEHEKLLAVYLNYLNAIMNSFTSEGAKGILNEYYQHWLHSSQIITLTERNSVRAKLV

GITEDYGYLIAKELMVGSDDFFTGTVHHLQPDGNTFDIFKGLISKKVYNQ

>model.g24.t1 Augustusgene.g24.t1 JFAV02000052.1:42-781(-)

MSSTGSSFKEEKVNNTSEEDNAPKALNQEPKLDHDDVSSYAGSVQSYENEQEQFHPEGER

DIESQNIESQLSRHVTNILSETNGAERIESLARILSTKTKKQLEHFEVREGIDFDLQQLL

DYLRSHQLDQGIVPGDAGVAFKDLTAVGIDASAAVAPSVAEMVRGWVHHPLRAFSRKKTD

LVLRNIIQNFTGVIESGEMMLVIARPGAGASTLLKCIGGETSELVEVTGEFSYDGLDQHE

MMSRFK

>model.g25.t1 Augustusgene.g25.t1 JFAV02000053.1:867-2222(-)

MVYFGDIGENSNTLTGYFERQSGIKCGVSENPAEYMLNRIGAGATANASSDWHELWENSP

ECAAGRQELAELHEKLNARPVTEDKELSSRFAVSLFTQFKCVLWRTNIQFWRSPVYIRAK

FLECILCALYVGLSYVGTGVDYGVQGAQSSFASTFMMLLIALAMVNQMHVFAYDSRELYE

VREADSNTFHWSALLICHTWLEIAWSSICVFICFICYYWPAQFSGRASHAGYWFLIYVIM

FPIYFISYGSWIIYMAPDVPSASMINSNLFAMLLLFCGILQPFHKMPGFWTFMYKVSPFT

YVVQSLVTSVVHNKKVICNPHEFAILDPPQGQTCEQFLAFYRDENKGYIDNPDATDNCRY

CPYTVSDQIVEQYGVRWDQRWRNFGLLWAYIVFNFCAMCICYYIMRVKVWSVKGILDIKK

WYHGPRKERHEPETNVFKEKAHDKKNVTKNKV

>model.g26.t1 Augustusgene.g26.t1 JFAV02000054.1:975-1556(-)

IADVIFGDANPSGKLSLSWPLKNEDNPAYLNFKSIMGRVLYGEDIYVGYRFYEKLQKQVA

YPFGHGLSYTTFKFDDLKVSGDDETLKVGLSVANTGKVDGKEVVQVYIARNAPSAVPRPV

KELKKFRKVALKAGESAKVELTLSVKDSCSYFDEFHDKWHLEAGNYQVLVGSSSDDIHLI

GDFEVKKSKFWSGL

>model.g27.t1 Augustusgene.g27.t1 JFAV02000058.1:17-173(-)

MSRTRNYKINEKTKNKKSGSKSTNTNTNTNTNVNVNGVPQKNAHYFQHNGYY

>model.g28.t1 Augustusgene.g28.t1 JFAV02000059.1:520-2048(+)

MSTQESAGGNTDAAILFSKVLDITNILQYDGVGTKKNAKVTIHEVELDFTERFGQTKKTE

FASAVLLSRLKFNSNSDPTKYVEKWNNLLEDIPEFVGLPVRAKMLMFTTSLPFELGRSLM

HRDCSNLDEFFKMFVDRKSQYFVSSRSPEFDEDAMDIDAIATRKGSSNRTPFKEIMEVRL

FELLDPKVGGQRIVNLDCVGKKEIIGCPILLDTGACTSLVTSRFAKRLGCRKRLAKRAIS

VVGAIGTEKKIVTEVTGVCVKLPKGKNISIAAYVMDELKHDLLLGNPVLHAFPELLQCAT

TNGQQGVTSKNNSRNPIMLIESPSQFVDIEEGWVVQLYEIESPPLSKEDIRLPQVLATEF

GKVVRDDLPPLDERNYQQLLPQKESGEDGQRE

>model.g29.t1 Augustusgene.g29.t1 JFAV02000061.1:1-654(+)

KSSKKKRGSQTSNTEPIDIYLTATLPNDFYGSWTTYVGDSPSVSKSTDALETGTVDCSKG

GIFDSDLSSTFKKLDFPTDFAIQYLDGSQAQGIYFTDSFLINGILAPNITGALAMETDMT

MGILGLSFEASESSSRNANSSLQFTYPNFLSSLKNSGIIAKVAYSLFLNSLEQSEGSLLF

GAVDHSLYQGQLYTVPLINIYNKKYTPKPIEFDVTVN

>model.g30.t1 Augustusgene.g30.t1 JFAV02000062.1:719-1548(+)

MSLSSNLRKNFLTPFKGLTIEFDSYVIPDELLEDGQLRLLDTDTTVMIPTDTHTRFIVTS

GDVIHDFAVPSLGLKIDATPGRLNQVSALIQRPGVFYGMCSELCGVAHSAMPIKIEAVSL

AE

>model.g31.t1 Augustusgene.g31.t1 JFAV02000068.1:318-2501(-)

MSSVTTLKQTLVGFSNVLKDYQYSQLSEQPTDIENVVQEFKKISSREAEKSLLEPTDAEN

WELETKLWHLIAILLTFRSASQDEDALSDDMTIYDYNSNIVYWKKFLQNDKKLYEIWLIM

VWVQENLKAPARPDNMSGSKWPNSSISGELASCDVDCPLRDNVLLEKTDEVNDEIAFKYI

FDLLLCGKVEQAKRECELTENIALGMILCGIDEYCDPALDSQIEQADEASKTSPSGCEKK

VLWRRAVYSLSQNPSLGKYERAIYQYLAGTPPTDSQEGITEYNWDSYLLLYLNQLWNVCI

ESYMNSIGKFPKDQIVSDIHESKTSIQTILNTVALKSGSESEHPTRVLMAAIIMDDVNSV

IKSSVNELVNSVQALEQQSLFEKEPFLLRLLTHVIIVCDIYTPNIADNNDKTKLISSYIT

ILNMYELYEAIPIYASFLDPDSLLESYSFILTTMTKRELKKAQLSICTALQLPTSNILKR

LAQTVFDSTAKYYEISVGEETVVKSTVDSSDHKLINAADWLIEGHLYSEALDMIVAISRS

FLLCGKTEALSALMQKIDVIALIKDLKLSALAESNENKVAPETNSKVKEVSEYNSLVEGL

LLFKKWGSSSLLLNNQSNLPSLVGQFNEFSAHFYKLITNFLSELVSDDATLYQIRALYVP

YLVMELHKSLMSASEALQITSFAHEAMNLSCVVADESNQIYLLFQSSGKLKEFLTLVAQS

AIKVGIKQ

>model.g34.t1 Augustusgene.g34.t1 JFAV02000068.1:10498-15666(+)

MSLKDILNQDVVTTDHSGEVIMEPENYKNGGNNFGKDSNNSAMVNGNASVIDQTHHNINA

STVQEPSHKKNTSKIYSDINSFQKHLECLQKMDYNESVLQDWNFLNLQELELITIWNNQI

KDILEENDTVNEDQLDLSSSASATLNAKTHSNKNNKDTRSDLVNGDDNKANSLQLNKGKI

QVSNGKDRGDSVNPSITTSHKGLDNLCDNMDQVNKEWGMYQQYLNRKQGVIDKACDDIIQ

KHLPHVQKTLKEREKWELEQINKKKRKAMHGKSGRKERNEEEEESEEAESEEEEEGEEEE

EEDDDDDENTDEEDMDEEQNSQTIQKTKNKLIPTTTLNGKELLIKPKKLNKKSLKNKRLN

GTENTDPSSNVSNLLKRKKLNDSTASLINLSKSKKNLSSNGNVISNSYSKSDTDVDGDVK

LESRQTPDLQDLSKYFYGKNKKAKLISREGGLDKAGYMNQEDEDGNEERENENEEEEEEE

EEEEAQLVNGQPLRRSGRVAVTQEEKNRNLIRRMVSKQQKKASEERKRRFTQCVVSDFDE

TTKKVEIKITLKQHHMKMYKRHIREKEQSKRKEEKAAAAALAAAEAAKAAAAAKASSKSN

KSKNSKRANKDKDDSQKQDQQDLQSTNGKKAKRGKSIGDNNTEDQAQDDLEDEDEELNEE

QTNKEIPPLDTTNNHFDTENLPRYGLKLNPREARAIQRHYDNTFFAIWKDLARKDSNKYS

KTLQTMQQIRMQNTRKTASLVAREARKWQIKNMRQMRDVQVRARRGIREMSSYWKRNERD

EKELKKKREKELLERAKKEEEEKEKQRQAKQLEFLLTKSEMVSHFIAKKVKTSEHEKAHN

SATNLTELADTTHDEEDGRPNIELDLTGEIQAETQLHEIDFDNENKEMLDKRAMENASNI

LKKQQRSVQTYDYNEDAQDELNFQNPSTLGDSTLEQPKILACTLKEYQLNGLRWLNSIYS

MGMNGILADEMGLGKTVQSISMLANLAENHNIWGPFIVVTPASTLHNWVQEISKFVPDFK

ILPYWGNANDRKVLRRFWDRKHLRYNKDSPFHIMVTSYQMIVSDAAYLQKMKWQYMILDE

AQAIKSAQSQRWKTLLSFHCRNRLLLTGTPIQNNMEELWALLHFIMPDYFNSIDDFNTWF

NKDSKDGNMNGMNTDHLNRLHDILKPFMLRRIKKNVQSELGDKIEIDVMCDLTQRQKKLY

SSLRSKMSSNYEEIERAAIGGAAVADEENNSSSSSSNGSKNSNSSSSSDTRLINAVMEFR

KVCNHPDLFERADVNSPFLFGTFGNLNSFLNEKDNLDLMYSSKNSISLNIPRLIYDEMIL

PNFENNHVDIKNHILNEKLNIFNPTFENVEYWSFLCVLSGIENVNELKRMSQAQLLQRVL

RLSENPKEVAARTAFLFGMENSKVPSSSNLLIPTKNDILNNSQKVSPDSVLSNLLNVREC

YYNEHYTNNLKPAFSVVAAAPPIVPNVYNTKSLTNTLDNVLFDDKITQAFSSVPASIQND

LVVNKHAKIEELPKSEMWPEPLNKNFSTRHISMPSMDRFITDSAKLKKLDQLLVKLKEED

HRVLVYFQMTKMMDLMEDYLTYRQYKHIRLDGSSKLEDRRDLVHDWQTKPEIFVFLLSTR

AGGLGINLTAADTVIFYDSDWNPTIDSQAMDRAHRLGQKKQVTVYRLLCRDTIEERMRDT

AIKKEQVQKVVMEGKKKHKKIQDAAQVANTGEKIVSQVVNESN

>model.g39.t1 Augustusgene.g39.t1 JFAV02000068.1:23544-24587(-)

MMDDPRLHKTRHIAYFDRFLGFVPASHQSMGINKLIVIYYSLCGQSVLGVEKLYEKYPHA

QKCIQDMYCENEQLAGFIPSSLMDSKNQTNFSLPNTLFGLLCTLLLEDRQSLEKLNTKKI

FAFIQLCQDTVEGGFRSYIDECGVDPVDIRYSYMAVSILYILGCRTQDDFAKVIDTEQLM

DFIEKKCYCRGIGGFSKSLEPHAGYTSCVAGIYHLLGHQCPFKDETTNWLVHRQVCNTFA

VEHPNFDTNDHGGIQGRENKFADTCYAFWCINSINYFGDGNIDFFDIPQVIEYLLEKTQN

KVLGGFSKTNEDDSDIYHSFLGVATLGLVCGKFNGFLSLPKGICKQIE

>model.g51.t1 Augustusgene.g51.t1 JFAV02000068.1:46861-47169(-)

MSGRGKGGKGLGKGGSKRHRKILRDNIQGITKPAIRRLARRGGVKRISGLIYEEVRAVLK

TFLESVIRDAVTYTEHAKRKTVTSLDVVYALKRQGRTLYGFGG

>model.g42.t1 Augustusgene.g42.t1 JFAV02000068.1:30294-30821(+)

MKMINSNKNKDFNIKGKVHQSKKLNFNLNVDSEEKLQKKYDIDGKTGLKKRVVGDFNQDP

NQFDYDVDELIREDEEEEQRQENLKYRRNKSTSNGSVNLEELA

>model.g43.t1 Augustusgene.g43.t1 JFAV02000068.1:31000-33598(-)

MNNFWELQRVKISQYNPLEFFTKITNTSIPSFYSLSVIVLIFSLYYLSPLQFRKHLKNFM

SALQNHTTDYLTSKFRNSRIGRTTYTNALSSIVINEGGYVGGLVNEGNSCFMNSVLQSLA

SSKELMDFLDKRIINHQESTDSNDDNDNDDNDSDDQDGDDKVDENDVSSDVETIKQEESG

ESLKEKENESTSSTKKVVKRKYGKMKKKLNRFKDEQLLGKTDSSEKTNSEADITFSIALR

DLLDKLNAKHYNNRPNFRVNKLLRTMTKKPNSNVILGYDQEDAQEFFQTLLNELERNVKS

VSAATQNSGESSAPSTSGSNGTPEPVSIDSIPENALEEQEEDLDRVGTVYIPTNQIDPNI

KDTKLYTPFKLVTPLDGITCERIGCLECGENGGIRYSIFSGLSLNLPGEKFGVTLKLSEL

MNEWSKPEIIEGVNCNRCGLLAVKDYLLDEIKEKSGNLPDKLLNAYKDRLLQVEALLSKS

VIDDEDYQKFKTANMVQKSSKSKQILLSRPPPLLAIHINRSCFDPRTYTIRKNNSRVVFK

KKLNLLPWVCKPDEINLDARLPMSKKHEGLKQQFEISSSEDEKVEKYYKKLHKTFEREYQ

DSDEEDEDEEDDMDEEHEYEHKYNGFNGNRDFPDSSDSDDEDDDDDNDAESEIFEKPVDS

LGNTISSSDESADIEDLDEDNDRLVALDDLKNQNSGSESRFVEEKHRKPQEQRSSRDVVP

LPTLRSMSTNVPDGPFSYNLRSVIVHYGTHNYGHYIAFRRYRGCWWRISDENAYVVDEKE

VLSTPGVFMLFYEYDFDPKTGKLKDDLETARLEKELEKELEKEQKIHFQMESDKQNSDNQ

GSVDAN

>model.g40.t1 Augustusgene.g40.t1 JFAV02000068.1:24824-28744(-)

MSEKSFIVTLKESADPEKFKKSVSSLGGSISHEFSLIKGFTVKLPESLHIDSLTKKHESD

IATVEEDSKRKTNGGEAMTTPNNDPNFAQIKTLYEGRLNQFIDGNGQYAHLNLPKFYDVF

RESALRNINDVEWDNNKDDDAAIVDVTYYQVPYDKKNPVAPENRPAWKDIVQADREGTLN

FEKVYDNQPFGPSWTTTWFKVNLQFPKFAQPGETWVFEFDCENEGLVIDGETLLPITAFS

GKAERIEYLIKISDLDLDSSEKSATYKHFFYIECANNEMFGCGFPSQIDPPDNNKYFHLK

KADLVLPNWEARHLAIDFLQCQDAAKQLPDDSFEKHLCRRVCNEVMDLFDPYDPCSITNC

RKLIKDKLLGDVDDETVYNEKNHSQEYVPIFGFGNCHIDTAWLWPFAETKRKITRSWTSQ

IAIIEQYPEYQFVASQAQQFKWLKLEHPEFFHEKLIPKIKQGNFIPIGGSWVENDTNMPS

GEGLSRQFFFGQRFFIKNFNMKSNIYWLPDTFGYTSTLPQIIKLSQMDYFLTHTTLLSGK

SGDVIGGNLLLYGIGDGGGGPTTGMLEKLRRFRGLNNKLGSVIPKVSLGKLNSVEEFFRN

IESKNKGKTLPTWIGELYLEFHRGTYTTQAMLKKLMRLSEVQILELEKLCSWWSFENPDF

SYPHAKINDFWEKVLLCQFHDVLPGSCIGMVYDHEAVPMLKKVVKDVEKLMTQVLQQAKG

SYKFGSKSLDSPTNFTFAAEDDSFITLWNDNLKISIDKQKGVIISIQDDQYEYLDLKTGR

NTLGANQFVLFDDQPLNWQAWDTELYDVNNYHYINSVKSIDILDDNAVKISFAIASEKDI

ELITVISLDNKMVKMKTVVNNWYTGNKFLKVEFPVNVHNDYCSYETQFGITKRPTHYNTS

WDIAKFEVCHHKFADYSEYFKGVSIINDCKYGFATHGNLMRLSLLRSPKKPDAHADMGNH

EMEYAILPHRGGLNMQTVQKAIAFNNKNTFCLPSELATRFEKFVTLTPLQKHSGLSSIDS

LNIVVSSIKRGEDDFPDSEYSLSQANDDNTSKSLVVRVYEPLGGEISAQLNVSQKFTKVD

LVDNLEEPLQKDITALDSHSIAVHLRPFQIKIFKFYF

>model.g33.t1 Augustusgene.g33.t1 JFAV02000068.1:6966-8093(-)

MSTFGRIFKVTTYGESHCKSVGCIVDGVPPNMPLTELDLQPQLTRRRPGQSKLTTPRNEK

DKVEIQSGTEFGKTLGTPIAMIVKNEDQRPHDYSDMDNYPRPSHADYTYMEKYQTKASSG

GGRSSARETIGRVAASGIAEKLLTQYSKMEIVAFTSQVGDVSMNKNPLDPTFQQILNTIT

REKVDACGPIRCPDPKLAGEMVKVIEHYRGEHDSIGGVVTCVIRNVPTGLGEPCFDKLEA

MLAHAMLSIPASKGFEIGSGFHGVNLPGSKHNDPFYMDEESGRLRTKTNNSGGIQGGISN

GENIYFSVPFKSAATISQEQETATYDGKDGVLAAKGRHDPCVVPRAIPIVEAMAALVMAD

ALLIQKSREFSGKLFA

>model.g49.t1 Augustusgene.g49.t1 JFAV02000068.1:43240-44508(+)

MSNKGQADNGVQRTHATGEPILMSQQQHNTYAPSMKQTQHKPTKYKKKLSLEGKVNKIAN

KFIQDKDFHYKERLTRLQTNLTTMHQGNNVEFLRKVRDLEEERDMNLVGLRYFEEYRVSR

SSVEFDQDIEQLKKEHENLVTLCKKKLFEQIEYKIKMLQEEKLLIEVANSHAYSMDYSNI

YNQQSQQHGMVTRNNRHLTRGESLRLGGDQGSDSNATHSSSFAGLFNSNGGYSSTERTGS

PWLNDSGSSADDLAKESSANVFIGSRRSLRRRVTTKQAERMSKEQELESINENATGSYGR

AGGQHKFGNKASGNGNGKGSGSNNTNNSSNDPELSAEWVKLVSGYSQLHALLFGNTDEDG

KNDYYNGSYTHSAGNAKKRNVGRHSTKSAPPLPSLEKDEVTDDINLIRSLTGQDPSPFAS

SNS

>model.g32.t1 Augustusgene.g32.t1 JFAV02000068.1:2876-5821(-)

MQSSTSLPDINNQWPELPFTVKATYSWSGEQRLDLGFLEGDIIEVSKIKGMWFYGRLLRN

KKTGSFPANYVELFQKPEPITSESMNKRVLYTPQEQQNVQTRSRDVRTERSTPFSSRSRI

NVASTPSPAKLKTSSRSSNNNNNNSNNSNYINASGGSSKFSMHSPVSSNTSVSPLESPLN

SKQKIMHSQRSHFKSSMSPSSSANSPSSRIFPASKSSPNIHRYTQLADEQRLNGSLSNSG

RRSTERQIQSNRDSLSNFKTKQLPSIPSNYDHDDEQRYQSPHYDLNSSASSSFDKSIVSF

TQTKYWESSQDKKARNYRDSESSWNNNTYDSHPHHMYEEDDPYDGSLAVMSNFSATSAGS

FARHKFAKSFTDSKTKQDLPRLITSFNGNALNFNNSLTSLLQDSPMTPQPLGSANTSSNW

LMKKIKSSTSNGSSSNNSSPINPKKFPELPDLSQLSLQGIGDEVDGWAMVNKQLNRANTL

SGKEKYERRKRFLDEAAEDTDIVLEPHTVMIQSINSNEVTYDSQTGKPLTNPGMVNMMLQ

DLDNEYLDMKTHKRVVKNGVQNVDSFAVSHLGNMFKTTLERLRAIFIFCTETYQLIDDNG

STNFKKPPMNLDEIIHKNYCTPYELTWIFKKLANSVGVHCEIVIGFLKTPNTDNLDFQLN

HCWLTLIVNNEWRFVDVILGNISNPIHSYINGKQAEKAEDFYFLTEPLKLIHSHIPYRTK

DQHIVPEIDNVLPMCLPTTFPNFFKNELKFHRFDNSLRYLNDSEVFEMSLFIANDIEIFA

SVVIDEEDMKKKKHYEGADLCLVQVKWYKQKRIAFIKAVLPPGTKQGTLHIHSGPKGMQS

TFVNIHPLSIIVPLKHSGSDKNIEYEFVRILPAITAADVDMYIKKPENKYVYLNYEYNFQ

TILQPSDGLVASKSYGFSSTKKKMVAIQSPSGKVYKYKKNDAQSQYGTWELKFKLNEVGI

WTGLVTNDSGAGFAAYAEWTCI

>model.g44.t1 Augustusgene.g44.t1 JFAV02000068.1:34185-35549(-)

MSDKSSATAAPAIPGAITVEQVQKQKEQALKNAEAGKGDDSSEPSVVAAKATKGGRTASD

KILYVGNLAKSITEDALKQYFQVGGKVSSCKIIFDKTNEDVNYGFVEFFEKKDAGIAYSN

LNGQNIDGFDIKVNYAYQNTASATTQEETFNIFAGDLNIDVDDKILADTFNEQPGFVSAH

VMWDMVTGFSRGYGFVSFQTKEQADAALTAKQGFVLNGRPLRLNHAAKKQQQNQQRNNFN

NNNNNNNQYNNNNRSHHNNNNNNYNNNNNNRFRQTPLPPQALIPPPVDPSVVEEVIRSEP

LNVSTAFIGNIPPHMPHEQLIMLLQNFGNIRDFKYFAEKRHCFVRYETREQAALCIVALG

SGFQIRNRALRTGWGKDHPRPQHQQQQQQQQLQGQFGGEPSQFPPQQGEFQQQQQPQGDF

AQQQGQFVPQDAQFAQQQQNQFAPQQQEQAPHQQE

>model.g41.t1 Augustusgene.g41.t1 JFAV02000068.1:29460-30023(-)

MRKSITRTLSKTRLSPLSSPSRWYCTLYTLPSISQNKSNTLSITTKRCFQTSFIPKNTNT

GGNASTTGLSVPEEILNLTQEEYHKASDLYLETLLDELEAISEENYELIPEVELNQGVLN

FDIRGKGSYVVNKQPPNKQIWLSSPISGPNRFDLYQNDWISLRDNTKLTEIINKELQEAS

NGEYSLSV

>model.g48.t1 Augustusgene.g48.t1 JFAV02000068.1:41348-42751(-)

MLVVEVQDKKYVKQLKTLLESYNGNSLIKPIKTTDIPGSIAGENVKVFQLKTSITDIDEL

QKLLAEYKNSVSIAACGDFEEGTPTRTMKFNPSLLDYIRGYLKEQNSFSAEFIEDVIANN

CSSKYSLYPPLILFNHSPIKQMFVSPVWEDQIFRNSNERKTDFFAKMIQQVFIPSNSQNG

VAFTHIAVNNPIQETDNIWRKPLNITPLFGDFSPNDVNFDCPALDNFEKAFWCSTVQNGI

YQEWAPIYTMFSRGNIKEKARILDTEKTCYFKDIANNDVLDMYCGIGYFTLSYLKRHCRH

LFGFDLNPWSCEGLRRGLASKKNAKFVKQDFCHIFNESNELAMQRLDEYEVSSMSPADNN

KQLRIRHINMGLLPTSEPGWAIALDVAYHFHGSCSVTTLHVHENIHDDDIKNGSFVNKKL

LPALLSLKEELQYESIDSINVTHVEKIKTFAPDVWHVCIDTDIHFKCT

>model.g46.t1 Augustusgene.g46.t1 JFAV02000068.1:37540-38877(-)

MDDQEKLQVLINENPHYPVVLTSINTANLPFKTTILRDYVQQTIQNATTMAEYIQQQQIF

NEKLISNGLVENVDQNMTVQILNQAAKTSPQSIFTPSVPPLGLSPYYQFTPVKKFTAKTG

SKIGNGEGDGYLEFQLRNYFGTGEKFIMDVQKGTKTKSSVLLQYQQPFKVWYNTSLSFFQ

NNRKVSGCSYELAGLKLDFLTSYHRFSKKHVWNHAVSFESCARTMKNYSRTKSLTVLFQC

QEDWKNSVTYTVSNDTRDNYVSPTRGHLLKLSNELEVFTLWNKFQLEASKATSIMTSSGR

QISFIGTLKSGTLASFSSASTYVPYIDKFQNGGGNDIRGFDFMGLGDKDHVDSLGGNHYM

SYGFSMICPLKPDSNFKWHLFFNGGKLCNNAQKLTKVQEHSTAIGFGILYSHPSARFELN

FTLPVSASETDLQRKGFQYGLGMSFL

>model.g38.t1 Augustusgene.g38.t1 JFAV02000068.1:22510-23421(+)

MPYSSDKTTPHLIGGFVGGLTSALTLQPFDLLKTRLQQERNSTISKCLKSINHPLELWRG

SLPSCLRTSVGSALYLTSLNVVRSKIANTKQAGQGTSIASHGSSNLPKLSAKENLLSGAF

TRAFVGYLTMPITVIKVRFESTFYQYKTLKEAVLDIYRNHGMRGFVKGYGPTVARDAPYA

GLYVLFYEKNKSFFPKYILPANFIQTDPKTGKYVTSTATIVNTTSALFSAVLATTITAPF

DTIKTRIQLQPNVNKNSIMTIKNIIQKESIFNLFDGLSLRLIRKSFSAGIAWGIYEELIK

KFLP

>model.g45.t1 Augustusgene.g45.t1 JFAV02000068.1:36339-37481(+)

MIVPPHRLHSESPIHTSVTEYHTAIDHASDNNIVNADTLIEDEYFKTNPKPEYLDKLVGK

AEKFLKSIPKTCKIVLLTSGGTTVPLENNTVRFIDNFSAGTRGASSAEQFLANGYYVIFL

HREFSLTPFNRQFTHSTTTQFLDYFNEHGELKEDFKAQILEEKKLQLKYKKQLLLLPFTT

VNQYLWSLRSIALLMNNKNCLFYLAAAVSDFYVPYSKLPQHKIQSSNEHRSGTESEGGGS

SSSSSSSSSSSSSSSSSSSGSSSSNVLSSGKLILDLDPVPKFLRRLVQSWAKSSMIISFK

LETDSSILIKKAQQALKNYNHQLVIGNLLQTRNKEVVFVSEDANIKEQWINLTPDVSCIE

ALIIPEAIKRHQEWIATQESK

>model.g50.t1 Augustusgene.g50.t1 JFAV02000068.1:44829-46646(+)

MRLPRFLKNPHRKAQLKKYKDTDSPYSSKNKTHSRRSLFKKSRLVSRRGLLLLIAALILC

VYFFCTLLWPPIITFLKHKYLDYDFKLADIDIGSSIDAPFYYGCNDIKEYLSDPEYQKQN

AAFVMLTQNSELEDVLKTMESLELKFNQWFQYPYVFLNDEPFTQEFKDAVQQHTLSKVEF

GVIPSDNWDFPEHLESKTSDQKSSKNVQFESNFEDQEDRGILYGGMKSYHKMCRFYSGFF

YKHPLVQQYEWYWRIEPDVRFFCTTTYDPFYEMQKHKKRYGFTIFIDEIYWTIPSLFKHT

QAFLKNYKKNVDSSFEVGTLWPMFVDSSKQYDGDADEKFGDTKVLDIFVNRQEEVDEELS

RYVEKEYYLKNYEPDASQVQERIKEHGIGSIIRQGFIKPEIFEDKFDKQEYNLCHFWSNF

EISRVDTFDNPIYNAYFQYLDNLGGFYQERWGDAVVHTLGVAMLNREDIHYFRDMGYQHS

TLAHCPKNLANGQLDNPNDKNFESTCLPFYKTYGQHTSKNTANSWKTRIQDTLSTVFLQE

EPSATAYGSGCQCECPFLKDDIEDASSFCSSKFFEHMSDDYRPERKPKLNSLMNKVYQSY

ERYIQE

>model.g37.t1 Augustusgene.g37.t1 JFAV02000068.1:21168-21971(+)

MAHKAQVVSNLHKNHAVVVTVQAHKHVNKLQNDSFFFEECLRLLPLNNGVLKKRFFEDQV

TILLNRLLQFWGSQCAIGGDSLNNKLRFSRGPYGKPVLTDHHPQITFSMTNGKLITSMFI

TRDVFKDVGIDIASVEDHTGDDFDVFNDILSPEEIARFQDPQIPAVDKKKLFARIWSIKE

SYTKFTGQGLNCDLQKISLPDVSSTETVTVKTSHVGTENNQMTFVSRWIDPSNVLTVCYP

FGSNIDPSALNFLSVDLLDVIHDLQQWQ

>model.g35.t1 Augustusgene.g35.t1 JFAV02000068.1:15823-19419(-)

MDSSSIFDQFRNLIINSVENQCSPSQYFIFYNEFFNEKFGIALKKTEPDTSASEAQANPI

SVIKNETFAQGDIVSMKAATPPPIDDEQKKKIYEILSDCFIQTLRELSTRSEHFVTVTNY

LIQVCFKDYNSSFVNYITQKIFQLGTENEAVIIYFFTKATNYFKSLNDQLIIDDFLVNDV

PAFLLPNLVSNIGKMKSRQLFVSLGKLLISILSKYDLKENPKFQLKDQHFYENVMSFCDS

LSETSLVLSKNFYTLFDSKFETAELKKSQKHDDKSPFTIPVETPSITSPLPSALSGTSAS

TKQGSISQQAPNTKQFYDVKLVRYYKNMWLNNKIQHCTTDEIDFFPKYQGIENTLNSNNS

IPSFESAIVDFIDTSFTCFAQFLNNKKYHQSANSNFTLLEKKWYLFIVKRIPLIVEKYCG

VNRSYLAVKAVQDLDDKIIKVFRSYYATDRSNSADNDSTDLFDNFDSPSQPELDIRHDFL

KSLCMMKLQNPSVLNKVFSNGDEMIDTNTITDHDLVLIRNKQNNFETIHNLKEYVESSFD

SFDFESLCFHDTCISDKSECFDIIYMLQNFESIAPTSQLIIAETLISILETAVKNFDYKL

VAKICALLSFNLGDSMPGMFTMVNCDRFVIPLIKFLDESWDENKEQVFSTGEGNENDSDV

ESFNDYIYFGFALVFLIYLQEQYKVDVVQILVKITNVKKLESSFLLQYLQTYKISCKEFK

LPSTKEPYKAAKLLQDWTNSLFVENSISDELIKNADIKDLAFLIPFMFDQVISVFQGLVT

TSKQQQPQNKNELNAALTNFNRIVVNSVEYFSQPFMMIGLVKIVFTLDSTIKKLQSICIS

QDESLLDTFLNILTPLFNESGILSSESKVLQFILYRVTGPKLLLTMNNLKRLKAKELTEN

SATVNTKLDYLISRLDRICDGANMYDFNPSILELESNESSPYENQDLSPKSSGTLARPPV

YFDFEITSDAPMNKIMHKQCNSFWNLHSSTFYNYDYLQKVIQLLGPGKFFTDIHRTLVFK

VETNGIPTMGNTLKKGDKPEDDVDVAMQYLIYFSILFDFSNNNERLQILQFLESPLSSSF

GENLESKGTTTTGPKETKTENDTAKTSEETANDNDFNMLFGEDSTDNIDDVLMSDFSTKD

EETALTASDAAEKIAKFSFLKKNTFAYLFFRYHETKAYPFEAFEEFYERYLDLLKRCII

>model.g47.t1 Augustusgene.g47.t1 JFAV02000068.1:39107-41086(-)

MSAYHESKTTKPNKLPNSSNEQVQWSAQEKDIPAQSSSNQNFIDWTDINTDDQDLYSKFQ

KQDLQRASLSTPPMQVLSSAEQNYLKAQASNTDNSNDLFYSSDGRKETSELHSESSKTFK

SLSPVTTPTFNIIPPLEAPEFEKEPQQSNSQTIRNVPNIVLSSSGPTPHAHFSQKKSYQQ

TSKVSLSNKMSSENQQLSADDFSNQGYIDSTMTINNFSQNNLSSSPLLQTTEFARNDLFL

NPYAGNTLHPTTSNETSFVTANDLQNSPRLSVLEDPEDVLSLQNSPHIPALQVEDYSSNE

NLLDNFLSLDDSLAVPKNQDFESFLSEEELDRMINQSLSPARSASVQGTPQEITVSAYDY

NESNLKRETSPNMHLLSPKDALFDDSRRERPRSRSISKNSSTHSRSRSISRDRSKIEQLE

NDLSVGQNSLNQTFLENETRFSKNNPNYEVEKPESVMSHQRSSFDNVYNTDLSANAPLVY

EVDEVTGLQFMDKADKGVFSCLYCDKYFPQAYNLKSHLKSHTDIKPFKCSHCDKSFARQH

DRKRHENLHSGQKWYICGGFLEDGTQWGCGKQFARSDALGRHLKTKAGKRCLVPLYDEFS

RSRFLRNPDKEILVQNLDSYLDLSVKLDDFEFENTVQPLIEHNFLKLAQKRAHVLKNVNK

>model.g36.t1 Augustusgene.g36.t1 JFAV02000068.1:19782-20831(-)

MSTNVYQSAVEFLRDESIKDAPLDKKKEFLKSKGLSEDQIEQALLESAQTNHDLPNEPKS

SLNLNSENSTQDFHSSGKPQYEYYAVPPPLPRRDWKDYLIMATSTAGVLYGCYSLTKNHI

VPRIFPKEHSQLEQDKNDINQKFSKLDEALERLELQEKELKAKEEEKVKQLEETLKSLTE

TLEEAESSKRKMDTEFRLMKAELLSLQNTVDKFFLDNTKMNDMDNIAKEINSLKLLMEKS

TSLPVSSTATPADNTYNSANNSAETPKQERKSKVASPSSVPSASDILSKMNLKPKGSTPV

VQPPVSPVDHESKGETPAWLNSRNRNGSSEGQLEIPEWQKNLTSGNNNSA

>model.g53.t1 Augustusgene.g53.t1 JFAV02000070.1:809-1519(-)

MITYDTFCQQLSTNLLLDLDPFQKYQITITDYSKDCIRFQFFVKQSLMNTSGQKILFKCL

ICYNASYSEPQLHLQMYAVNEGCDETLDDAHEDEFTLENELKFQSSCDSPTEPELMSFSE

SLETMKFINISEFHHLLQSPLSHGIEQHVNNYFHQIYHFPLLLDSLSSGEIFYSVQNCDS

HEIVGGGNIERFSVNSTDQQENKGKDQHHLVEQNYLKRWISVYVLPILRDLCAVKKN

>model.g54.t1 Augustusgene.g54.t1 JFAV02000070.1:1929-2696(-)

MLRQAIMKRGLATAAAPATHVPKMKTFKIYRWNPDTPEVKPKMQEYKVDLNDCGPMVLDA

LIKIKDEQDGSLTFRRSCREGICGSCAMNIGGTNTLACLCKIDKNESKSTKIYPLPHMHI

VKDLVPDLTQFYKQYKSIQPYLQRKSFPKGKEIYQSVEDRKKLDGLYECILCACCSTSCP

SYWWNAEEYLGPAVLMQAYRWMIDSRDESFGKRKEMLQNSMSVYRCHTILNCTRTCPKGL

NPGLAIAEIKKALAVD

>model.g55.t1 Augustusgene.g55.t1 JFAV02000070.1:3467-13120(-)

MLESFAAAFLNKFLGSYVENFDPKQLKVGIWNGDVKLNNLKLRKEALDELDLPVNVKSGI

LGELTMQLPWSNLKNKPVKIFINNVFLLCEPRSWDSYDPEEDAERQFNLKMNDLDKLEAA

SSVTPVSDESNKNNANESFTTSLVNKIVDNVQVSIKNIHFRYEDFNSVFSEKPCSIGFSL

YEMSAVSTNDEWTPSFISITQQISKKLLSLNSLSFYFNTNDTESVFDEDLDVLIGKFQES

IQTEDENERNIYVSQNLVSPVTGSCKLSINKAGSTEEQPHLDALLDFSSFSLELDDEQYQ

ELLGIASKFQWFQKSWKFRKFRPLCSVEEDPRAWFKYTATCVLNEVHEKNYKQSWEYIKE

RSQQKKDYVKLWKLKLASGDINVPLADPTEESELQELHKKLPLEAIRFFRALARKEYSKE

QLELSHTLSSATLSTSESTNQSNKKNAQNSNTKGGGWFSSWWSAPTAEQSEEQSELIITE

EQKKEFYDAIDWDENKSISEAVDIPEDRITLRIKNFLGKGSLKLKSKSNNAVLGEMVFED

CQTDIFQKPKSFGVSFKLFQFTVEDGSPNTLNKHIVKVNNGNSAEKTPFFEVDFEQDPPH

SEADSSLSLKLKSMSVYYHVHFLNTIIQFFKPPKKHYDTLSALLSVAESTFEGWTYQTRM

GLEALLEEHKNVDITCDLQTPLIIIPLDAHAWNTPCAIIDAGHISVNTELIPKSRIAELK

KMSVDDYSKLETSDLNRLMFDRFLLTFDNAQFLIGPDIRSTISSLGYDGNTNQYAVLQKT

HLEMMVDVSIFPKALNFPKIKTAASLPFLKLSLNDYQYKIIMQLLEVCLPNGNFQDLEDS

QTQVSTNENEIQVALQDTVKRLANMSEAELKQRLFEFSLNVNSIEIKLHQCTAKEDMSSD

PVVNLSGYGFELNVSQYPKDMKVLLSLTELHLLDQGDGGERSLLSSRKGDKSLFKLDLER

SQRIVPFKNTLIEVFDQNIDLEMSSLKLNLNSNSILSILNYVLTTFTDINAPEMPADALR

HNDDSEDVAPQKMNVVLKMDEIVVVFEDETGKLATLNLSNGSVDCYLLPEKMKVVGKLGN

LELKDNLHEDLNLYVRQLISMQGGEVADFTYETFDQMVNNEIFTSKFKFNANSLKINFVE

QTFGRLFEYMNNFQKMKGIYDAAREAAYNQTPTLDSINNIKLDVLVKTPIIEFPTVVDPV

NGVIDNVTFYLGEFFMSNQFTKTPEKGTTNAIKTGLRSIKATSLFHLDNGDQNLELVEDL

DMTISIDCNEQSSLKTPAFKIDGQLSDMKLSLTDMQAKYLLSLQSKIVNSFVSQNPSQDF

EQVQTDAIKANAVIAPHKQHLADEKTFSEQSSSEIAKQPEALHLDMAFTAPSFLLSFYDN

TSKSTEIAECSISEISLLDTGVDFKMGNDGSFDGSAFFSTFTIKDTRVNKTSKHAYLVPA

ASSKEHQFETTVRRIVTDNNTELDVAVKLDNMRVILAIDFLISLKLFYDSCISAEPLNEV

VKSENFMDESSLVSHKKVTETTFSEEQNANNLNLSVDIINPTVILLAYPESSTSEAIVFK

IEKIMFTQKDIQHVSLSNIGMFMCNIDRFDKEHVRMIDDFSVSAILDDRDSSAQKSLTQI

RLDVEKLVMRVALRDVRLAMKILENTNKMLSASGLLGSAVVQAIQDNNEIKFSQEFKLAL

AKYAPSLLSSFSLQTKDSFLNDLVTEKDKIEICAQNFTSGFGGLRLVLIGDVSELPLLDF

NVNPFTIEAKNWSQEISVLGQLTSYTNVFNYSKSDWEPLIEPVPVTFKLAKGNKTDDASY

NIAVYSKEMSEITISAESIQLLSKIPKSLSQYKENFSRGDIKPFKIVNETGADLKIWIDQ

ALDDKRNETVVKNSESLPWEFEDWRKVRENLDTDNSAATLGFRVLDSRYTNTFKVNTKSE

LEELYKLEPPLDGVHHRIACHIYLDADGIKIIKFESPLIVTNQSGSKFELKINNEESVFI

APKSSFSIPFNIVYEAKYSIRPCLSGDEYEFSKKSFNWETLLGGPIFEKCSSVQNGSSFY

YRIDAKYEEKEKLAKVFPHMEVVVSAPLKIENVLPIDVEIELLSKNTKEKIVLNLHKSES

LPVHHINLNDFVFMKLNAPSTEYVPADYVLVASPNSSSLAVEKQIKMLKKNRTSILKLEL

KYSIDRYTQTKMMKIYSPYIILNRTERDIFIKAKGSNNIFTSKVSLQDGEYVTKPEMFSF

DYKDDSNSAQIKMKDSDWSPANTLDALEQNFDMALSIPSKTMQTELGLHISEGLGKYSLS

KLVTVSPRFIIKNEYSEDLVLGETRSIAEVVLEMNTAIPLYKMSTSSSKLCKLKFYGGNY

SEPFPIGEIGTLYTKVFNKQLTKHILLKIETVLDNATLFLTVKDSNKEWPYSIRNFTEET

YFVYQRDPNMLDSDRSYYTDANDDISEYDSYMYLRKPNEAKANYYLTGGKKFEPIRYKIS

PYTLMPFSWDFPAAKEKKIIVECRGIKRTVDLREIGNLKPFLVKKTTPDNEDAYVELNIL

ADGPVQALVLSKYNPDVSLYTLRSKAKNSSSDTVSRSSSQSPVESFEVSNHVDGNNNGKQ

VNKFKVQVNFEGLGLSVINQKLEELMYITAKGLELRYNDSKMYQSISWKLKWLQIDNQLF

GSMFPSVLYPTVINNVQEELDNHPCFSGSITKVNDKTRGLVYYKYATTLLQEMTIKLEEE

FLVELLQFIKFSQKGVSQDLASDHTTVEEHGESGVESAKISSEQIFDNYNHSDSLLRLPE

SSIKVENADIYFETLHIQPTILHLSFMRSDGLQIDGEHGLLSGKTNDNDMEVYEAEDGDD

VARQSGPSTLVNILTMTFGNVNDAPIQLNSVFLENLKVSAPMLIGLIREHYNNEFFRQVL

KIAGYADVLGNPIGLFNNISSGVSDIFYKPYQGYFMNDRPEELGISIAQGGVSFLKKSVF

GLSDSFSKFTGSVAKGLAAATQDKEFQRARLLQQRKNRINQDGSGVASGFSSLMNGISSG

ITGVASNSYEGAKKEGAHGFFKGLGRGLVGVPTKTVIGFLDMANNVSEEIRNNTALQDGH

GGKIVPVRYPRFINHGSGRANGVILGKDDGLIKPFNLREAQGQYWLKMCNGGQFIKDHYI

AHVVLPGKERILCVSQERVCEIDITTLTCISSVTIEEMCLAGTPIDDGRQIIVRILNRYT

RETVTWKFPVPRAESRKYIIDHIILAQNRALRYMDDRL

>model.g56.t1 Augustusgene.g56.t1 JFAV02000070.1:13682-19384(+)

MTAPSVQEKNELALIDKVELRLALANDAAKYTQFLETFLCPLLLKLASPHSTVRAAIFNL

IKDITSRLNSYPEIKLPVLKLIKQAKNPNVQSAQDSQSVRLYSLLLASKGVTNLQIECQD

EILLECCDGISDLPPTIAARMFLVFLKLVAKWSGPKLGTLEETSTKSFYSFSAKDTAFLS

SKFIKFFLLIPAKPVASGQPIPRGYVCPGLSLQDVAFFTFDAGVTYTKDQLKTSRKSLFR

FITHGINFDDIELVKNLSVISVDSSNNAEQAQTLLKRMQIPYENESFIGFLIELYTGNKQ

TGTPPVPNSVKEVILNVLLRSNIATKASSVKTVCSIGLNAEEYKIRSKTLKYIQHVAKYN

YEALLSDDNGSETNSETFHLNITSLIRSNLHTEGWPRIQIGQGTPNIHLSIEQRRTEYET

LGELLQHTKLEDLSYIEFLIQSLLKDLPDFRTTIQNVLTGLAVNLFQNLKEEQKTQLKKI

MKQYLNDDTEYLQMNKEEKDSFMSLRFVCLRFCNTIYSFNDSEARLLNVFGFSKKNRFDV

MEEAMKGLNPYWYKMNKTLSNKSFQDSLSVSTEEIKFPDFQSFVQLLLSEAKICLTYNNH

ALRESLLAGIRFALRTAISQAVFQKRTIIQQDQDWSLRVEQCLSVDFVVEKLFKDFILSY

KSQWYVDFLKLLLTEFCTSNNNEHILVTSNVVMGDILHTFIKYSKAEVLMQLGCFQQNLC

FFIGNQTNQAHDLDNAVEIFGMISCQLGVQNPVTQFLLEKIETSISERDFAPIIKAACSV

VARFVAKGEGNTFSIQINNLYDIIEAKLKTGKAVYRNDALKCLSLLSKYGCFDYLTDKSA

YSNTLNILATNIMNDEAALMAFSNMLIECKDDIEGPFEKVIIPTHQSKNVDFLFTVGEYL

TVIMGKWESEFLARQLDVSGVELSILKSKYSRSIKFGAVLNRILHLSKTTTSPSMIKALC

IWLLSIVQYCGSFEEVKNSVNEIHGVFMRFLSGRDEIIQEVASRGLSITFELGNADFKED

MVKQFFRSFTNSAASLGNNNNVGSDTELFDKGDLNTGDGSVSTYKDVMSLATEVGDPSLV

YKFMSMAKSSSLWSSKKGLAFGLTAIVSKTSLSNMLLQNGDLAKKLIPKLFRYRFDPNEQ

VSASMNHMWKVLVPNSSEIVEEYFDLIIEELLTSIGDREWRVREASAAALRDLLQLFPQS

KYEQNLERIWTMAFRTMDDIKDSVRQEGLRLTRVISNILVKSIDKNKGSAESKKVFDILV

PFLMGPKGLNSDAEEVRDFSLQTVITLVKDCNPQAIQKYIPSLIEDLCLLLSSLEPQMIN

YVALNADKYDINMNLLDAKRAHQANESPIMDTIEKLIEMVEPASYEQLFDAVVTTVRKAV

GLPSKVGAAKLIKLCCTKAPVEISPSSGKFLKVCLNNLKNSNNEMITSAYAVAFGCLFKN

APLSKMIKYSEKLVALYFEPSSSSAVDLNNKLASGVAIQAIVNYSPSQFEQVKGIFIPFV

FVSRNFAQSSLRGDETDQVSKTTRRINGVFQHIWTESTSNSEADTIQYYLDEIIKINAQH

IKSNDFKVRVMCLKSLADLVNKIKVSQTKENNISHIFDILTRGIEGRVWDGKEIVISTLI

QTCIKFETYYKTHDNVKKVTETVLSVELLKKTRKYAYSVVNSFCDYCVNFPSDWATDLLI

RSSIPLGLVEKKGEHFQNGGGSLDADGDAVMSDINLKSSQKNIDKETERNKLLENLSRAC

CWYSVTNPATGELGKRKFSSKLFGFIISNVFYAMTLKNPIVYTWRTELMSCTVGTILVNN

VFSTSHSNDNDEQSNEENLETSKMFEDYWKLLYQSIMVDMRNKSIENVQIQLVRLGGLLL

QKYPHSEQTIHVVVRDNLSLLLKADSTSSVVKNELKLAGVV

>model.g57.t1 Augustusgene.g57.t1 JFAV02000070.1:19568-19804(-)

MQIFVKTLTGKTITLEVESSDTIDNVKSKIQDKEGIPPDQQRLIFAGKQLEDGRTLSDYN

IQKESTLHLVLRLRGGASA

>model.g52.t1 Augustusgene.g52.t1 JFAV02000070.1:46-735(-)

MSTSLRASIVSMENKCSNLLNQYSTFAQATSSKKQPTEAKLDAQIEKNLQDRQTNIDDYA

QQLHALSSTNQAGLTGKMAQLERHKDVLFQHQKEFNSLRTCIQQERNNMNLLFDVKQDIL

TSRAEELERSRMYGDGAGTSADDYINEESRRIDKQHNVVDNLISQAWETRDQFSSQRRIL

NNANNKMFEVVSKIPGINTLIAKINTRRKKNAVILAGLISCCILVLFFTY

>model.g58.t1 Augustusgene.g58.t1 JFAV02000072.1:38-785(-)

MTNTSAYNEHTMADNESMSTTFYHEDLQLKDTAEVLHYAIGTLETKRDKYLTAVVVMPPS

EFFKKHGVLIVFVTSSGVILWFMFKKVLLIRALGQAIFDMSIYFFIGCFLRIKFETWIQD

NSFIAMDKEVNRTIADNGLNIFGFDIANKELGKSDLSNVQVFRDNESKEIVGLVVLKPLS

DVSSDDEKKFIVQIGIMAASETDEVCIQMFEEMLEWCVLKAQILRELRHETIYLQIHLLG

LDDESCVFF

>model.g59.t1 Augustusgene.g59.t1 JFAV02000073.1:1-1745(-)

MKLSSVLRLFAGATALAKAQSSDEVTDIVTVTKSLTITVTDNLDGTLVYPPSEVLAALTQ

ETSATTTSVDAQATTEVNAKQENRLKVQQKNIPKERCARTTTKYITYTVTTCPVCTATTP

APVTTTQTTYITLNSCTDSAAVTEQYTQVVPNKKRGALGKEEEKCKKTTTTTSSPKPTSH

TTSSTTCTQQKNVTPSTVTKVVTSTIYTCSVTPSPTTSCTQSHEHGTSCTHYPGTTSCTQ

SHEHGTSCTHYPGTTSCTQSHEHGTSCTHYPGTTSCTQSHKHGTSCTHKTSTVSTVSTNS

FVQPTTSSTPVVPPTSSTPVVPPANSSTSTTPCSQSSSFISTVSTHSFVQSTSSTPVVPP

ANSTTPVVPPTSSTPVVPPTSSTSVVPLTSSTPVVPPANSTTPVVPPTSSTPVVPPTSST

PVVPPTSSIPVVPPANSTTPVVPPASSTPVVPPANSSTSTTPCSQSSSFISTVSTHSFVQ

STSSTPVVPPANSTTPVVPPTSSTPVVPPTSSTSVVPLTSSTPVVPPANSTTPVVPPANS

TTPVVPPASSTPVVPPASSIPVVPPANSTTPVVPPASSTPV

>model.g60.t1 Augustusgene.g60.t1 JFAV02000073.1:3945-5674(-)

TPSSTLSSVTSSVVPSSSAPLNSTSTTPSSTLSSVTSSVVPSSSAPLNSTSTTPSSTLSS

VTSSSTISVSTQSLVQSSSSSTISTTLPSTFSSYNSTVSSTSPTSNTVVSSTSVSSALTS

SSVFVSTNSFVQTTNSSSLSTVSSTSYASSSMSSSMISTPGSSSSASVSSTPLNSSSTVL

PSTSQSSSFSTTSNSVSTSSATSLSTFSSFTIPTGSSIVYPSYGNSTYGSSTSSSTSSVP

GSSESSSTTTTLSSFTAPAPSCVVSTTVTAPTTVTSGSSTYTTLTTYVTSTTDSSCVASS

TSASGSLSSNALTSTSVFTSTVTAPCPVTSGGSTYTTYTTYVVSSTITTCPLSTPTDIES

TNTNNVYSTGQLSSATAVVVVSTVSSPYKVTSSGFTYTYTTFYTVSSTITKCPEGLCTTT

LASVSPKESSAVESSAFVGTVTSTAVYSGESSLNVPATPQTEGTSSTSSVDVVSTTPSPS

LAFSATLTASRNTAASETTKEVDSSIATDTTLSTFNTAKAASTTEASEYTYATSSVVTGT

LGTVSAQEAGAPKRVGSTMVGLLVSMASTLFYVLFN

>model.g61.t1 Augustusgene.g61.t1 JFAV02000078.1:1-523(+)

DYFLVRKGYVRLLHCYTNKPGSLYMYGKYGTNWRAVVAYLLALALNFPGFIAEVSTAKVN

VNENGRKIFYLNYYVGYLASFLFYFVLCYFWPIEGTRPNTKITDFKAFREVWVEVENFDE

KRRIFLETGEEGEYDEYNDEYMFDNDSTQQNDGDSSSSSDKVQMQTEIVADKKV

>model.g62.t1 Augustusgene.g62.t1 JFAV02000078.1:2073-3400(+)

MVHPTTSNVSEVESAFDPFHHANKSHIDDKIETDEKDSSIGYTNSATEENGLPMEDFEKA

DDTKWNRFLRFLEVKQNHVSGHSGARSEDVLESFLFNDDLKPVEEARRVWDWKNYVFFWI

SGSFNVNTWQISATGLQMGLSWGLSWVTIWIGYISVAVFVCLGSRIGNFYHISFPISSRI

SFGVYFSLWVVLNRVFMAVIWFSTMVYIMGQTIQLMLIAIFGTDLPVRIKNTIGDTQPIT

TFEFMCFMIAWGVTLPFMWVPVHNLKYLFLFKSIIVPFAAFGFLIWTLVKFDGKVAVGSL

ASFHPTGSLLGWNFIRAVMSAMDNFSTLILNAPDFSRFGKTAKSSVYSQLFILPIMYAII

AIIGIITTASAYTEYHENYWSPLDVLTRFLDHQTAGNKAGVFLIALAFCIAQLGTNIASN

SISAGTDMTALLPKFINIRRGS

>model.g100.t1 Augustusgene.g100.t1 JFAV02000080.1:75249-76325(+)

MTDIVKRRSLVFYTNAQKPELITTDLDLNSCYKPDEIVVKVHYAAFNPIDYILYKMASSW

FASKKSVKTFGRDFIGEVVKVGKNVPADEFSVGDKVAGLFQHLFGESGTMSDYLIFDPKA

HKSIAKIDSEKVPISPENGAWPLVFGTSYQALTSFELKKDVDWTKKDKRVLIVGASTATG

IMAVQIAKKELGAAAVDGICSAASFERNKEFGFDTLFDYKSGNVTQDVIDHVSKVGVKYD

VIYDCCGNSYFFNCMDQVLKPKNENSYYVSIAGDSKINYASPAFPSIVGAVKKWFIRCYR

YHQLLLQPNADVAKMGIKMITEKNLTVPVDTVYQPEDYEKAFDKLTSAKAKGKVVVSFV

>model.g106.t1 Augustusgene.g106.t1 JFAV02000080.1:84936-86915(+)

MQPSKLQLICCFIGANLVGLGSGTIYVYSFYAPQLLHRCNIPISQSSTLSFAITIGSSAF

GLLVGVIIDKVGPQIACFIGTVTTFAGYYILYLSYLHRYSYIYLLSLALVLVGLGSMSGF

YSAIKCCTTNFPNHRGTAAAVPVAIYALAGVLYSTFCKWYFGENILGVFKFFMIVCSSLI

FAGCLTLKVWDPKKYTEKLVSQQQQLQKTAIDGSKRNSVLHASDENGRTSYDSLVRNSSF

DNNNNNTSNTTSDSNAPHISKPMSIKAKRTESAIWSRELVGSLAYWGWGKVRDSPGSGTV

SRGTSFDKRNSRIASMGSSQRVNPLLQSAEFYANDNSHHRHDSSALDMEDAQDSNDETWE

DHQGMLPPQQQQQEQEFGSRHKLSDVSNSMTLKELEAHSSTRDIPLSSIIISKKFIGYYL

ILAILQGVGQMYIYSVGFLVATQLATHEETTNLNQETVQSIQVSLIATMSFLGRLSSGPL

SDFLVKKFKAQRKWCIFLAALLMAGTSVYASKDPSSVHTKTAISDVTNLNTTPDFVKRIS

ISSVLFGLAFGFTFGTFPATIADAFGTNGFSTIWGLATTGGLISVKYFTSVLANDLASHS

DLDELHHDGVCRLGSQCYMHTFRVSACFSLFAAAITLYMIFIKYRYRKARVADYRRRSSM

>model.g126.t1 Augustusgene.g126.t1 JFAV02000080.1:128966-129289(-)

MSTETALSYAALILADSDVEISTDNLLTLTKAAKIDVEGIWADIFSKALESQNIKDLLTN

FSAGAAPAGAAAAGGAAAGGAAAAEEEAEEEEEAKEESDEDMGFGLFD

>model.g95.t1 Augustusgene.g95.t1 JFAV02000080.1:69491-69985(+)

MSITAIIWFSIYVGVFAMTSLISLTFIVPMMSISFICAGLIILIGMFSNMAFRSAQKVYE

WNIAILQSLLVKLAEKMPPAHQSREGDATKFQANNKNSASALKSVEWVKTKVTHVSGRVQ

NKLVSIIHTFVDLFTNAVLFLHPKDKSEIANPTVPATPMAIQVIN

>model.g94.t1 Augustusgene.g94.t1 JFAV02000080.1:67290-68399(-)

MQRGGIRTKEITPNTQNPTTTTTTTFPSPSKMRPIQMFGHSRPVSQIKYNRDGDIIVSVS

ADESASIWYSNSGERVGTFNGHNGSINSVDITSNSKFCCTGSGDSTIKIWKLENGEELQT

IEYLPYIECVEFSPNDNKYLLVITAGVFKTHGCIDVYAFNNETGEIGDIIFKIERNPEVD

SFMEFKRASWSFDSKYIIIGCKDGQILKYDVEARKIVHQEQLHKLIINDIQFSPDRTYFI

TASKDRFSKMVDVMTMKTLKSYESTGPLNSACICPLKDFVIIGGGQEARDVTTTGSKEGN

FESRFFHKIFETEIGRISGHFGPINGLAVSPQGTNFASASEDGYVRLHHFEKNYFDFKFD

VEKTQAALGA

>model.g88.t1 Augustusgene.g88.t1 JFAV02000080.1:51144-52337(+)

MDKRARRQNSSLQHANKKVKKSGSQAYGLRNEGSNEGDRLEYRILQGYRTMDSEMVEQRL

EIDRGAGINLAMEKLKNADELFSQAGALMKDPEKSNNTILAYDSRALLNISELAQSSVRN

LKLDEAKQNINSIDVINGMKKWMLEEYLEETGLDHNLVDTDDEGGNSDSDNNGVEVDGDE

PLTQADIDKRLMTKRLKERSKLAKYEPFKQFEQFNWLKLGTLYRNISRKPNVADHLLGPL

QVEKKARAASKPRMRDDPIGKEVTAKNMSNEDMNAKGAATTEEFVRACFKILKKKKGYES

INLFEFIIDPDSFSKTVENLFYTSFLIRDGKLIMEEDEDGYPQIRIMPPAPKDPEARALE

LQKRNEKPLSHLIFQLDIPTYERLKQEFNITESFIGKV

>model.g125.t1 Augustusgene.g125.t1 JFAV02000080.1:127018-128290(+)

MYCVHQKNLPLLKNHFRKHWQERVKVHFDQAGKKVSRRNARATKAAKIAPRPLDLLRPVV

RAPTVKYNRKVRAGRGFTLAEIKAAGLTAAYARTIGIAVDHRRQNRNTETFEINVQRLKE

YLSKIIVFPRNGKVEENVEQVLSTAAAFPIAQPAADVEAREVVVPEQSAYRTLRLARSDK

KYAGVRAKRAREAAEAEAEKKK

>model.g77.t1 Augustusgene.g77.t1 JFAV02000080.1:32444-33547(-)

MQKYNSLRKHSTEAPKMLISRPRLKDILPKKSFFNRCLYDLKADHSFNNLYPTVEQIYKN

MGSEDSLGKIKYPKWFTSTDLMNFKHCMENYRVQHKRINKNLFELENTLLKIAVDMNDSN

AKALSAFYVLGKSSTLPKETVLQAQKHLQKLYKSEDNALSMKLLGDLAFNGNNSQASIKF

YNKFLTVENATPLAAEVYKSLGEVLFKLARFEEAEECFLQCIRNCKTQESVKAYYYLGQI

YTVSDPTKSKQCFEICASEGFVESLSLLGFLEMNYFGDMVKSCEWFKLGKELGDLKCYIG

YFDLQWELKDYRECMFTYKSIQDMAKTSENATKALQTFEESRQSKIKLVLDLNKEYTPVS

ATGDRWSV

>model.g97.t1 Augustusgene.g97.t1 JFAV02000080.1:71314-71871(-)

MLPTLNSTNDYVHVSKKYNNGKGIVMGDCVVAMKPSDPHQRVCKRVSGMPGDIILIDPSM

GSKLNYVSEKVACMRNTEDLAADPEGLLQETLSEYEPLLKNNSDMSNGDIDDEELRDNKR

RLESFDEFVQVPKGHVWLTGDNLSYSVDSRTYNFVPMGLIKGKIVAANDFNKPFMQSFRS

IENSYK

>model.g91.t1 Augustusgene.g91.t1 JFAV02000080.1:56319-59402(+)

MQYFGRALGSVTKSWSSINPSTLSGAVDIIVVEQANGDLSCSPFHVRFGKFQILKPSQKK

VEVIVNGQPTSIPMKLSENGEAFFVFETEMEDVPENLISSPVASAASSPMSSPETKTPLE

HMANKQRHDKKFASTDAINSLEEPDFLDINNNGTFSPSHPVINSSPDLTAAGNDTNQLAS

PVSFTSPTFTPIKKKFDKIKIPTKIDNQTGDLLLDIEGYKYNQDKAHDSDELVQQILKEE

FDDSVISKMIEKDSQGNIRIINRSTPSSPKSFDGNSLSPTLSSPSSVALQQNDTTDLGSP

PLRSSVSDNTGVMVSNSSTGNYDTATMGLNSPLEASNKEATPKAEKKYIKTIRLTSDQLK

CLSLNMGTNDIKFSIGNGKSFLTAKLYLWKWDTPIVISDIDGTITKSDALGHVMTMIGKD

WTHAGVAKLFNDIYNNGYNIMYLTARSAGLADSTRAYLKSIDQDGYKIPDGPVILSPDRT

FTALKREVILKKPEVFKMACLKDIRDLYLKNFTFDTDDISQTTPKKTSMNKNSGGVTALD

EDMPTPFIAGFGNRITDGISYRSVGIPKSRIFTINPDGEVHMELLELSGYKSSYLDMNGL

VDHFFPPVANHDEEEDFCNSATDYDNGRGTDLDTIQNQSTNDDNSQGDFVPGYGIFDTIN

GIDTVRSRSSSLGVANAHQVNVHKKTSQHSGQNDEDLNSLSKRISNTQSSAHKRNPSNNR

YGKKMSLYRNKEEKFTDVNFWREPLPDIDDLSDVSDEDASAASTSNGNISYQQPNSPVSP

RVSSADKSYTLSSRKSSMSHNRAKSIDSGFFSSFLRKEDPAESVAGSETDSNVNDDAISS

TPNPSPFMANDNSLSPVFGDGSLGSLRRSAPTTHVFSNNVGASGVSPEVGQKIYLNIGSP

LSSPRLDSLNYDDDEYPYTGHENGNEPDTPTKMPKPMFLPQSSFDLALTSTEGDLKKYRT

FEDDHCDNDRNNGLFNGLNNELTEDKLNQDDQADKKLQAPNGKIDFAHQNNMSDEFDEDE

FDDDEFED

>model.g80.t1 Augustusgene.g80.t1 JFAV02000080.1:36253-37473(+)

MFQRQIQLSTRLAYKLRSTPGSLQVFKRFSSFEPLHPSLLSRAVGYSKELRELEAEMNAG

QTQNFDVEKEKKYSHLSSIVGSFNKYISKLEQYVELKELMNADPSLREEAQNEIEEVLPD

LNRSKNALFNKLLPPHMFADKPCLMEIRPGVGGTEAMIFAQDLLDMYVNYCNHHRWPYRL

LSTTENEEGNGIIEGILAIDQPYSYDKLKFESGVHRVQRIPATEAKGRIHTSTAAVVILP

QLQDSNDQKSSDAYERSFPAKDIRIDVMRARGKGGQHVNTTDSAVRITHYPSGIVVNMQD

ERSQHKNKAKAFAILRARLAERERVEKEQSERKARTDQVSSTNRSDKIKTYNFPQNRVTD

HRCNFSLLDMENVLNGTKLDKMLDEMVAFDNSEKAKQLLAEIANEKN

>model.g121.t1 Augustusgene.g121.t1 JFAV02000080.1:116656-118113(+)

MTTDTVSDIKNIYAKAGQSHVFSSYDSLSSEEQEQLIQQLSKISDPAALIKSCQKSISHA

NAMKSTKTATIEPLPHTSYDSVIDNPIKEQEYFEIGIDAIAKGKVAVILMAGGQGTRLGS

SSPKGCYDIDLPSHKSLFQVQAERLQALQKMCGSHLGEGSTVEIPWYIMTSKPTYQPTMD

FFTDNHYFGLNPKQVKFFNQGTLPAFDLKGEKLLLQSESSLVQSPDGNGGLYKALLDNEI

LDDMLAKKIEHVHMYCVDNVLVKIADPVFIGYSIEHRFQLATKVVRKRDAHESVGLIVSK

DGKPNVIEYSEISNELCEEKDEIHPELLKFRSANIVNHYYNIDLLKRNLAKWCSTMPYHI

AKKKITYYDSETKKLVKPEEPNGIKLEQFIFDVFETVPLEKFGCLEVDRAKEFSPLKNSN

AAPNDNPNTAKAAYMALGTQWLRNAGAKIPEEVMIEVDSKMSYSGENLAQFSGFDFAGKN

DTYLSH

>model.g105.t1 Augustusgene.g105.t1 JFAV02000080.1:83945-84406(-)

MSGHLSNEAFLLNITSLFAEANKLHRNVRVSMKRIIPQNLVEKPKELDSSAHPEYDVSKM

SRSYLQIKPNSSKISQKKYNLLMRAVMGAGRGGKDCTKTKISTIINPDHLDQFWQNYSSN

VKAGMKGLSKKKKKKSTAGKGDKVSKKATKKVRK

>model.g81.t1 Augustusgene.g81.t1 JFAV02000080.1:37870-39852(+)

MLIQGYKTQSSLTIGNIDRYILKYKLHDGDLNHISEYLNASSVLKIRIHNLVHVSLRAAG

LVTGPYTLYCEVRPQDYHHHQHIFATANQPKFESNVQPQQTFHTDLQLNDFSKQELTWTI

DVISQVLLTSTATISYELAVEFEDGAGMSCDRLYVTKMNTQDIWELPFSQLQNNPLDKKD

HLVILTHGLLSNLTTDMLYLKEEIEKSDPRCIVTGFSGNVCKTEKGIKYLGIKLANFIVS

KTAQVHTHIKKISFIGHSLGGLVQTFAIAYIGLKFPMFFQKLQLENFITLASPLTGVLTD

NPKYIQLILAAGMIGKSGIDLGLASLDGYPNECLLSLLPCAHTRNLLRRFRRRTVYANAI

NDGVVPLYSSCLLYLDYEDVMNNLETCNSTDISVQELKGEQQELLQKSVIKPFSKAVSLL

APQATTTAEPRSIPKLSLLDTASHVLLQPVPPLSFIVNPSTRPNIILHDRLYTPADIEII

KIQEPFQALLTKNKKSKKDLNYMLEVIAGGSDDARHKEEVIASRWHKNLTWRKVVVGLEP

DAHNNINVRRRFSNGYGWQVIDHLTTEHFIHPYKEMTTYNDFESTDDVSDFNWLLEPEKQ

GFFDAGATGLIASMSNLLDQWTGKTEEYDSPRTSQEGKKSKPKESTYGQTVGHEDLDLLG

V

>model.g74.t1 Augustusgene.g74.t1 JFAV02000080.1:25437-26501(-)

MSTATPTPTSASPPSTTIYPSPTATGFVLYKYKLNRPLACVSIALFALWTLIIAVEILWR

YIKTIKLFRSSPLYAPNRKQIQKTLWSVSFGIYIPLILGGLIEVGGYISRLSSASDRLAL

SPFIAQTVLILIAPGMMSATVYMMFHRMIEFLNCPENSGVGVFIKDTVMVKLFVAGDVIS

FFLQAAGGGIMSQKNTLHTGEVIIIIGLFVQLVSFGIFVFVQLNFTLRYKKFSENYFYLQ

VDNKWKYLNYSLYSASIFILIRSVVRVAEFIQGFNGVISSHEYFLYIFDALAMFLTMVSL

SFSFLFTDMGEMYYNHYTITYSEKHQNDGCLVEETESRNSLSQTLEIPEWKDENN

>model.g86.t1 Augustusgene.g86.t1 JFAV02000080.1:47006-48754(+)

MSNHINRSCVRCVQKKIKCDRIIPCSNCVKRGLEDECVLPSEPSLSTEEMALKESFEMQI

LSVMHQWDYWVVQQGFCNGLLLPAKGYPLDYSEESWHPGVIKVIGLELSTRLLTHSVESI

GCLYLNFFPDVQELFSKLDTFFSVDESESSNIGDLMDEALMWSLLTLAMYYINNATLKEL

DMPNELQNFSSAELYREFLAYTIELIQKIQHYPDIRIIQMFLILSSTFMRFDFPLFYNQF

MTTVLQISQILNLNTCFKTGSGSSTTSMLIKNVSDRIWYKLLYWDYKTESFNRPISLHSS

LFTSLLRHAAFLKSQNPDKYVREDSLELLQWKITSLERDLDPYTHKKPSLKTLEVIRREL

EFLQTKFAQIKRLQFPESENDRFDLFVINLTIYSTYWKLLKMVIVYYYQDIFSLNELFEY

SKLILQTIEREPTFVKHPMVLPILAMLGSFHGFFYVFNKSQENHLLFKRIRKFLSSPVFA

QTKKHQALLALLSRFSKLKNMFKTRALTEIELMQSAPIVILENDIKILTNQLENRIPNVL

EDVGPVLDSVIEEETPYDNETKNYIKSEQSKIFQLLASFKDFI

>model.g82.t1 Augustusgene.g82.t1 JFAV02000080.1:39882-41966(-)

MLESVEAFHQIPDLLSGIEQKRIELSTQLQEIEQHNANLSTIENDLDDFSNGKTEAYESK

IVQLTEQLSQIKTLSDLESLTSSNQNLLEVSVVKQLKQLLQLQQANENFLRLAEECNNRI

IPEIKTLKEISEVENYLSTEISSNINYTSEEIAFIKKIISGELYKHFLSLKADLAQNFNQ

LLLDSNWDSHKFNNHDLIPAMKAKSNQLYHFSVLVQEWCPSEPMQYWNFESIANNFKIKF

IYHFSNTQQQDASQSIETFFSFMESYLNENLFKCIAVFHEEDIGLTKLTVHDQFINYILD

PLRKKIKKTLTSIVDNQKNLETLVRLITQIFKTDEILWEKYSYSGDGLITLLPNNVLKIW

IQFETDIVDKQYVKNFSNYKGLSKITQDGPNFVKYLLNLYSYFHPFLAVTAEKFFQYKIM

VFKKIFLNLLKSFRESNMNYQQSNETDELTHFEENVVRLKNMGSVYETMYVLEKQSDCII

LNTQFNEITESHYKTLFQAEMAEYQNSVNALLESLVHRFKKMFGNSLRTYFKVNTWSSLS

VTLENDRCSNELIGAVNAMSNCYKIVQTHLVGKMNEKYVHKFQLKVVNITVDYLMNYIIK

LNNFSTFGYKQIVQDYNFLKQSLGFEKNKIVSQDDGSLLETLQLYYTLTFEDHEEVPWLN

KDYAKFDSDFVRLRHTFDLKYAKNEDISYILYKCL

>model.g66.t1 Augustusgene.g66.t1 JFAV02000080.1:3401-5557(-)

MNDLFGDDILEVPQPYSEEDNEVRNLIKNEENDDGSSSKNGLEKIEKEQKISLNGDKLGN

SDNHSGNQSNGSKKLENETTNNKKKKLSSKNRSSKSSGFTKPEGLSNLISFDEMDDVQDT

NAENKKPLDLDISVNASASISKSQKFIGNGRFPQTVFIEEASKASSGQGSTTKYVLYTIK

YGEGTVKRRYSDFELLRKILVKLFPTTLIPPIPKKQGLKSYSQTITSTDRYKKTHTSYLL

PSQEEANGIDLSHSVIKCGVKDEKLVRHRIRLLTSFLNRLLKMDEITKTNIIFDFLDPDQ

QNYVDLINGLPSISNSLLTTSIYQLNPSNPLCTDMKIYSVLPIPHKQKRAGEASTSFISD

ASKTLQHLYLSSSPSSSSASLITPPTSDEDINANTGVSTNGHIKKTEPILEEQEHRPKQE

QQNSQNNHSTNADFKDFDENVHNPLSSVLDQHFSKYEHILKLQIFKYNKKSIQHLKELQL

EYAALSQLWGDFSGTSTYARSSMLLSAMFDTSTAFEESHLNMEKLAGTLYYNISEPLYEL

CGLAQSAQKLIEYRNLKRAQKNMVAHLISSKKQQLEKLKLKQKTQSLNNTLESAQQQQSS

QALSSEATRQSSFYGNRLLKRWNQITTIVKETINYVEVDAEELIPKIENEIETLQSVSKV

AQKDLLEIENHINLVELPKFLKTMDTELQILLKDYAKYMKEAAFNNLKYWKAVKNDIQE

>model.g130.t1 Augustusgene.g130.t1 JFAV02000080.1:137330-139318(-)

MDHEIRSLSSTPVSTQEITLAEYLFQRLNQVGCNTVFGLPGDFNLKLIDKIGNVPNMKWC

GNANELNASYAADGYARIRKIGVICTTFGVGEMSSLNGIAGSFAEHVGVLHVVGTPTIST

QLKKLLVHHTLGNGDFNVFHRIYSEITSYHVMLQDIDLAPLEIDKCIVSCILNQKPCYLG

FPANLSDITVPKNLLNIPLNFSSPISINKIETERDIIQSIISLIMASKNPIILADGTCNR

HDNIFKSIHDLIRLTKFPVFVTPMGKGSLDEQYPRFGGVYVGSMSSPEVKEFVENSDLIL

AIGSILSDFSTSTFHYSYKTKNVVEFHIDYVKIKNARFPDLNVKSILSKLLSSLNTLIKD

GKFKYNASKKVVPQPPKSKSNLLETSVLRQEWIWDQLTNWLREGDIIIAETGTSAFGIMQ

TRFPNHCLGISQVLWGASGYSLGACLGALFAQRDDLEVDKLRKYKRCIVFIGDGSLQFTV

QEVSTMLKWNFHPYIFVMNNHGYTIDRILHDSTVHEPTYHDVHPWNLQNLLSLFGGAVEG

QEDNPNGEAQALAKALNGPVYNPQDNTIKWHRAPVGPNCKNYRVELVGEFSKMVNDPAFS

IPDKLRLIEVILPTMDAPNNLIRHVRTMQQRKGSVGSSTVSTPATTSTVNSSIHPSSHNY

GMI

>model.g132.t1 Augustusgene.g132.t1 JFAV02000080.1:143653-144603(-)

MAISKFFKKTFNLIYSSTSGEALTIQEAPDPSQPSVSTNTTAVGPSITKLNTKGLEAFDQ

NFNDKKFFSSFHIEHKRVKHSGASKTTGKQDKPAKTNKTTTTTKKHKEHKPKNTDPKAPN

NSLTYIYLISEHLEKYKSASEERHSLSRGNSNSTSSVSSEATQEEIIISALTSNQRKAEE

RLKNQKTDILSFRSSNSIIGNNTEKIEKIDPNIVVIENHQKTGKQEKPKNKYNDQSTRKN

MNERIVKILNLEKEKQEKEENEGNNNYEELQKDGEYAVDHSTNGLNHETSDASKMVKMKT

IKGTYYEIFFFFIFYFR

>model.g79.t1 Augustusgene.g79.t1 JFAV02000080.1:35059-35997(-)

MTISTSYTKEKKVGEGTFAVVYLGHQQNGPTPNRRVAIKEIKTSSFKDGMDMSAIREMKF

LQELRHVNIIELVDVYNENENLNLVLEFLPADLEMIIKDKNILFGQADIKMWILMTLRGV

HHCHRNFILHRDLKPNNLLLSPDGQLKIADFGLARAIPMPQDILTSNVVTRWYRAPELLF

GGKHYTYSVDIWSVGVIFAELMLRIPYLPGKDDVDQIDLTFRALGTPTDKDWPAVSSFPS

YNRIKIYPPPSREELRKRFIAATENALDMMCGMLTMNPQKRWDVVQCLQSPYFIELPEPT

DPAELPKLTKNEA

>model.g101.t1 Augustusgene.g101.t1 JFAV02000080.1:76706-78307(+)

MNGPTGFFGNANNKVNPTTSGGLFSQTSAPSLNLSSQQQQQQQQQQQQQQQSAPSLFNPT

STQTLGSTTLGLNSNASNTLQQNQPLLQQQAPLYPTLQPQPIQTSQSNISRQEIKTVPTW

CTDNIKRTTPQTIVRRSNKETNDEQDPFSGSSAPSTAKDAFGTVSFGMKKSNTFNSIKSV

SSTFNSAKLNDSDQLGSPNKNNLSFHLDSTDAPPAKSIFDIQREDEFSNRAMDNGVVEST

GSSNATEERSKSPAKKMGIFKSKNIFDKSKRQEPLHEVGDNVNSFKKESHSPSADAQGYA

VLVFGYPESISNQVIAHFSKFGKVMEDFEVCREEPAFGLSRFKNSGESGVDKTKYPIFTG

NGWVKITYNTETSALRALKENGTLYGGVLLGCVPYSKDSVEGLTNCKIEKYQDSFSVSAP

ALPNDRSFPSSNSNASTTAQTQPVFTKGQKESIDVKNEPLFANARKLNVTSGANIFAHNN

SGSLYNNTSNGQSLKNGGWYNTLESVMTQDVASSKHPNGIVGKLSNWLFGWSEL

>model.g89.t1 Augustusgene.g89.t1 JFAV02000080.1:52478-53668(-)

MFVRRTLQKHFYPAKSFTYKCFRKYNVLAIETSCDDTCVSIINSEGEILVDLKKSLKTVE

QDGGIIPTKAHIHHQVEIGDLVCKALEDTQVSLSKDIKLICCTRGPGMPGSLGIGFDFAK

GLSIASGKPLIGVHHMLGHLLMPTMFDPSITFPNYSLLCSGGHTVLVYSENTYTHKIIVD

TLDIAVGDSLDKCGRELGLKSNMIGKEMEKYINDEFIARGRAQDVEDLQTLAKKYPITEA

LKYNLKQHPKFVSFSFSGYLSNLKKIIQDNNSAPDSELDKPGLCYLIQEHHFKHLINKIK

DARKYKMLPRENVPLILSGGVGSNMRLRAKLEQQLSSVFSSFHFPPIKMCTDNAVMIGWA

GIKFYDTLKLKTNYNTSPIRKWPLNELLTVDGWAKVE

>model.g120.t1 Augustusgene.g120.t1 JFAV02000080.1:109964-115462(-)

MKRFGSKFQGRKFSRSSVIHEESPSSSNIGPLQGSPYRNAVTGTALAEDFEPYLVDMDLN

SRTGANSMKREEDTSNTLEVDNSQIELDNYSTRDGAMAQNDSLPIQPSDNDNENDSYSQN

QQQNEQEETVDSRAFLSPRQSNFREKVTYFFSDLKTKVLNRSSLPPTANGRHIPLSLDKE

SIVYQDFHFDSEKTLLMDERKKFKKPYCDNVVTSSRYNIYTFFPRQLYAQFSKLANCYFM

IVAILQMIPGWSTTGSYTTIIPLCIFIAISMAREAYDDYRRHKLDKDENLKQTRILFKSK

QNKVFEKSKNTNTNHDLLPNVDHSLKTESGNPTDHFTNVHLLKSRHDIEYVQTEWRNLRV

GDFVYLKNDDWIPADMILLCSDGDNSDVFVETIALDGETNLKTKNPHPEISKRMSTATGL

VNTQGKVTVEDPNMDLYNFDGNIELLDSVSGTWAKYSLNSDNVVYRGSILRNTTNCIGMV

VYSGEETKMRMNAIKNPRIKAPKLQKSINLIVFFMVFIVASMSLFSFLGYKINENRFISN

NRAWYLMETQVTTAESVMGFIIMYNTLIPLSLYVTMEIIKAMQSKLMEWDIDMYYKPANI

SCESRTATILEELGQVSYIFSDKTGTLTDNMMIFRKFSVCGSSWLHNLDELDNQEDEEDS

KLALTQTSTGSNSNVSVVSQSDQHFVQDILTRPASLSARSVRAQQAGRSSGISTGRKSMG

RPSVEYRNYGSTNYTGRPSMASHIQRAEQLEEESLSPTTTGSSAHSSPNLNPSSTQKSKK

NTHKGIKTTLELIRYVQRNPNTFFSQKIKFFLLSLALCHTCLPKRRAAPMNNGNSEEQDD

EDLDDDEKITIEYQASSPDELALIVAARDLGFVMVSKNSNVITIKTYPSGFDKPPVTEEF

EVLNVIEFNSDRKRMSVLVKARAEPEKVLLLCKGADNVILERLYNNKVASTKSAELNEST

NERKREQAELVLDQRKSLSSASARHSGPFRSRVSVDIASRPSLSLQQVRKSMSKPYNARP

SFNPHNRTSFDTGVNINSIDAFVENVNKNKQDVDEIAFQSRKSLHKQQRERYKNSALGSD

SKGSDLTNNANNMNPVSASLANATRLSPAKSILPKSPSAQMGASSGNKSSIDRSIEAYIG

GQELCENEEYVIERTLEALEEFSTEGLRTLLYSYKWIDQADYQEWSQQYHEAKTSLFERA

QKMQTVGELIEKNLVLLGATAIEDKLQDGVPEAIEKIRRAGIKMWMLTGDKRETAINIGY

SCNLIHEYSTVVILSDSDENILLKINTMSQELDLGEVAHSVVVIDGATLKTFESNPVLMG

AFVDLCTKTDVVICCRASPSQKALMVSSIKETDKNMVTLAIGDGANDIAMIQSADIGVGI

AGKEGLQASRSSDYSIGQFRFLLKLLLVHGRYNYSRTCKFVLSTFYKEAMFYATQMIYQR

WTMFSGTSLYEPWSLSMFNTLFTSLPVLCIGMFQKDLKASTLLAVPELYTYGRLSQGFGP

GVFLRWLTLSACSAVLITFLNLELFGKNVLVDNSIYPMGFINFTAAVFIINIKIQFLEMR

NRSPIEFACVIISCGGWLVWCCFLPAIYSQELEYDVKYGFYHHFGKDYTFWAMILLIMAI

PLLIDMLFKVLKNSLWPTDSDIFAQLEQEDEIRKKLEMQAFSELEQGWTWDKDPSTFSRY

TDRVVGSVIPNKATDNVQNRSSAYSNESHSSLSLGSTEVDNSTNSNAAEKQRRTRKNTIL

SFYNNTNFNSNHDSLFDSTKFEVLPSGRLIKKRVTTNDGVSSNGEASGNEESNLSREGKK

LKNAFSRTLRFKLQTEDDEDIEAIIANRMKDLE

>model.g122.t1 Augustusgene.g122.t1 JFAV02000080.1:118197-120803(-)

MSTDVGLGLLNRDELNQSLSKDVARYKSARLENHNHGLRKTSNTSGNLSSFLSSIHEGQP

QQHQGYRHRGSDEHTVKFSDSSPTVVNEEVFKTEAHSSNAYSSLLNTIPFWQITKNSYHS

QNYVRNNGKTKKSSLQQFFFQKTHKHEREISNLVSKVLKVKPFGDDQFIDELQYTVISSS

LFSTQSENEYAKLLSFPVQKSVLDFRKNDNKTFFNQRNHQKQAITKYGYIFTGEKKLVLR

KFNSSYFNTHMFVYSMLRSIHIGKKRYNTLTKKLVICLFIQAYLNFQQESFRNRYIKYSA

LLSVKSTIKSLEKMNILLQRYENLYKELLVHRLMDNSNHSRPNIENTDENEGEFVQQLQM

LLKATIYPIYQKLIKTVELFVPLINQNILQKHCELFGVDLVCIHNYYKFVTLDQDLDYQV

SCFRNLVKLFLISMLSMQTLQVSSFAKEDQDFTHTNTTNHEIDVFFQNMFTFVEPRAAMN

DFDKLQFLKKHNLSVEKFITPFYETLLMKRYWFFGTSAFSPDKECFPNASRPPSPAFDYT

QILLHEQNQQHRNKSVQKMTFMMNQISNQLLTANKIKFTTQKFKSKIASEIKYLQDLWTE

ISSAPVEDTDLHSPFPDSSEVAQEMFEFPTCSSKKVSSLNKVHTHKNHIRQPSNTGFHLN

ILDVDTNGPVNMMNKNDSLQPLKSCVIDGMQTESIGSSSSIQPFSNPELKQSTEARKLTT

PIDFVQVDDELDIDYLGNHFDNEELENTFLLKPPAINKFKTLSDEELRKKLDEKIHSLAV

ENKMHKKSMLKLRKEQRTHGKLDDGGLLKQKSFTYAGRHPYQLGKSGNTSLKYSQSTPYD

LNIVEQPDMFKEESIPALYELNKLLNNEM

>model.g116.t1 Augustusgene.g116.t1 JFAV02000080.1:100084-102510(+)

MSDGNIATSSNAASKTTDPNSLPNSLNGSTSDPASPHASNAQYQVSPSDNSKNAVSTASL

KEENGANIDAQAPRGSISSYFPAFISSGINTTMQYLSKSNDLDEEYNKNMLLNGGIPFEL

LAPRFQSYIIKESQDLLKKEQESYFILEKVNQNTRKLGVCNSKSFHCLIPITDDSIIKLD

AESPNSAEKNDNMAQKPTPFLYVNNSLLLPGATISEMYHQKSLWSQIAMGVKDHYNLEPQ

RHLYLKNRTSSDSVYFAKSGNNSAKSLSKKIRIVSITCSLPEAYMLKTVASVPSSYELSA

NLKKTLLENNPHCDIQTLSISLVLQPVKNLLEVIQTIVARQNKFFKDVDALFFVGYYHTV

PLVIQTVEALIKKSIANQYNTEYSFKNCHIGLWGIESCLSSQTFFEHSSDVESNKKLYAN

CTKQEQEILKNIHKFASSASVQSSIKWICKNTNVKITLTAKLYDNFMTLSQKLGVAYKHP

NIMRNAWVDCNSFELCEPYISKSVENKELKISNTHCCKLLVPKERSFEISLINNLILAIN

LGHHEQASFLLQKLAPFFISRSFNENTYPNVLKKKIDRDTKEWLSKIEKDHPSWKNIVPT

ADSLHDIKECACNSFSDFIEFLKYKSYAKQLVLKRAIQNDEKIYKAFIQNTLFTSSLVDS

QKMGLFEKHELENIFDQHSQYEVVWSLHEFLSEFITFKTLPSDQGEKLYTKLCLSSSFLS

SKEGLGSSKNSTNDEVETQSLTLNFHEHESSKHTEYIRTTAESLLRVKNLYNQYWNWQPP

VKGLKTLKRIFSVIEMYSTSEDFIRDISV

>model.g78.t1 Augustusgene.g78.t1 JFAV02000080.1:33796-34983(+)

MFSRLRVPSTQFKTAQFKRFNSDFTHCVIGAGAVGLAVAAELSRIPDASVIVLEKNYKTG

METSSRNSEVIHAGLYYPSGSLKSKLCIEGKNIIYNELKPRLTGVDWIKCGKIVVAQNDY

QDSEMEGLYYRCKNELDIPAELIPSTKLKYIEPAIEALRGAMLSPSTGIIDSHSLMSYYE

SCLKENDGDIILNTEVKNIEFSENSAYSIVCEDTESKEEVVLTVDNIINCAGLHSHKIAN

MLLPKERQVQQYYGKGNYFQLNTSAVPKVKHLIYPIPPKHGKSLGTHLTLDMSGNIRFGP

DFEYVESPTDYSVSTKNLMPAYEAIIKYYPHITPDQLIPAYSGIRPKLLGPGQKGFSDFY

IKQEEGFKGFINLMGIESPGLTSSPAIARYIRKTFF

>model.g131.t1 Augustusgene.g131.t1 JFAV02000080.1:140114-141466(-)

MDISKITNNYTEQSGGSNTGNNNNNNNNNNNNSNTISDTHLTSNANIHTNQILRVPSGEQ

MAGQPRNFNPVASQSSLSRAPLPGNLILVTKTVFQGHPPVQSPAIQQPVNITYPKTEVVG

HGSFGVVFVTTIQETQEKVAIKKVLQDKRFKNRELEIMQKIQHINIVDLKYYYYEEDPQS

DSLYLNLILEFMSQSLYQRIRHYVHLQTTMPHDEVQIYMYQLFKGLNYIHGTLGICHRDI

KPQNILINPENFQLKICDFGSAKRLNPMEPNVSYICSRYYRAPELIFGSTNYTTKIDVWS

SACVMCELILGQPIFPGESGIDQLVEIIKILGTPNKQDICSMNPNYMEHKFPHIKPIPLI

KIFKREKDYLCIELISNMLKYDPFERFSALQCLCSSYFDKIRMNYNNEIVTNLKLLDFNN

DTDFDYLSQDEKLIVKQKLLPHLYSSINPSG

>model.g65.t1 Augustusgene.g65.t1 JFAV02000080.1:2452-3273(+)

MAEPGIVPDKDTVLITGGCGGLGRELVKRFDNANYKVIAVDICSKPVFVQNLDCKTKTSS

NIYYFSCDITNGISLQELKRNIENLNIGPVTVLINNAGIASHTPIKNFSLKSGASIEKIM

NVNFQACFGLIQLFLPDMIAAKRGYIVNVASVLGILSPAKLAPYGASKGALINMHELLSL

ELQRSRNMNRNIKSLLVLPGQIKTNMFANINTPNKLLAPVLKAEKLADSMYKAMVSKHKT

LCAPYYANFIPLIKNLDWPITFIARLFSRMDNAL

>model.g76.t1 Augustusgene.g76.t1 JFAV02000080.1:30612-32396(+)

MVDQRIVATSSFKKLLKPLIVFRVLNALLISSYFQADEFWQCLEPAHYKAFGYGELTWEW

KEGLRSYAFPFLFQIVYHLCKLISLVSTWALLYCEKSCQSIMMQSTSLKRYDFFWDVLDY

IHYLRLSFPADLEYYSVIYLPKIAMALIAAVGEYYTIGLIYKIYLLTFAKKYPKDANAII

KISLVLTLSNFFNCFFITRTFINSFEMSVTAIALYYWPWSENMAELNTPTFKLSLFLSAF

MCLQRPTNGLTWVCFGLCMVYRLLSKKRFAEINTLAFNIVAMGTLAVILNCCIDFYFYRE

ICFPILKFIKFNVTKSLSVFYGNAPWHFHIAQSFPIINGYSLPLVIYGLVTSFDTPNNYI

FKIIKLIIVFNTTIFSCIKHKEFRFLFPMQPLFMVFSTFSLFTVWKKYNLKLVSFPYLLV

PIASVIAALIIVRFQETGVIQVIDMLHNLPYPMESVGFIMPCHSTPWQSHLHRPDIGDLW

AITCEPPLHLLDEDDSDTLIKSYMDESDYLYDNINQFVYLNFPPVFNKKLRSPEKTFLHE

WPEYLVVFEHMDNEFMQKKLEDSPYFEYTRFFNSWSHWDSRRSGDVIVYHKPSWK

>model.g69.t1 Augustusgene.g69.t1 JFAV02000080.1:13069-14628(+)

MTVSANDKSGQPLYKEITLPSGKTYKQPTGLFINNEFVHSHLPADQVIPVHYPATNEEVC

SVEIADKTTDVDLAVEAAHTAFKTSWKNYSVLEKRECFLKLANLVNDNLQLLAEIESIDT

GKSVSNTSIHDMGRVVDCLKYYGGWIDKMCGQSFIPSKDKICYTYHQPIGVVGCIIPFNY

PLSLMAWKWAAIAVGNTTVFKAGDQTPLSILYFASLVAQAGFPAGVFNVINGYGQDVGDA

LVKHPKVSKVAFTGSTVAAKIIQQNCSSQLKACSLEAGGKSPIVIYKDCNMDQAIKWTCV

GIFSNTGQICSGTSRCYVEEDVYEEFLQKLKAYMEENYIIGDPFDSKTVVGPVINKIQFD

RVNSFIQSGIDEGCRLIAGGVGKPACIAKDSKLQNGFYVQPTIFADVEKHHTISQQEIFG

PVIAIGKFSSANGLQEIIDKCNDSPYGLGASIFTQDITRANVFAREVESGQVWINSSNDV

DMNVPFHGVKESGLGHELGSYGLENFTTVKSVHLTLADRL

>model.g83.t1 Augustusgene.g83.t1 JFAV02000080.1:42552-43004(+)

MLQTKPIKVNEFIIAIRSVSDEELVTLENEIKTSLKHLESSNARMQELKKKALAQIKGDD

TDFEAAHLPDEDVDQGLQADVKLYEESIRENESVIDNHHQRIAAVENEKEHRGLGQKKSS

SGKSASAGSKTGTGVSQDNDESGEQSNYVYL

>model.g109.t1 Augustusgene.g109.t1 JFAV02000080.1:91982-92833(-)

MSRPIGKHHKLVLLPHLIKIIPNKPKEPASLPNDAKFYGFDLDSTLIESKSKMKFSRNPK

DWKFVELVNPESPKKNPVNSLDALLQLFKNSSMQSQQANIVIFSNQGGVITTPTDSKSCT

NLLSKLQNVLEVIEQSFKEQDCVEFINHIWIYCSPKMPSSLKKKSSKPNAKTIMVSKKNI

FKKQNLTKEEDIEEIKLPLSQELLFAHMRKPEIGMFEMFQQDMNNYFHLKNGASEDIKLE

FYCGDAAGRPADFSDSDLELANNLQTEFLTPEEFMIRYTDKKSV

>model.g87.t1 Augustusgene.g87.t1 JFAV02000080.1:48862-50830(-)

MSIKPLDPDVVNKIAAGEIIISPTNALKELIENSIDAGATKIDVTVQNNGFKLLQISDNG

SGISKDDFPILCHRFTTSKISSFSDLSKIQSFGFRGEALSSISHIAHLSLITKRQDDKEG

DIAWKGVYEMGELVPGSVKPSAGNQGTIISITDLFYNTPSRLKSLNQKEEYHKILDCLGK

YSINNQSIGFTLKKADESKMALIIKSATPVIDRIRNIFGSSIANDLIEFEIKDIEKPVQE

LGFVSCKGLVSNLNYANNSNRKAAKPVFFINNRLIDCEPLKRAVYQIYANYLPRGAPRPF

VYLSVFLKPDHIDVNVHPTKKEVGFLNEEEIVNFISDNLGELLSKQDSSRTFKANAPSTN

TLSNIHVINPSQTPFGMSQGAQIKRDETRLVRIDSQQSKITSFLKAADVKYESQSYGLSQ

IEVIDEDEEGSLKTDTNDNSQSQHALPDKIVFNEEREKNDVNLTSIENLKEVVNLNAHSE

LTTILANLVYIGVIDSHKRLCAIQHDLKLFMIDYGAVCYELFYQIGLREFANFGKIHLSE

SLKVKDLLELLDILEADKITEIAQQLFEMKDMLDEYFSIEIDKVENSEEVVLKSVPMLLL

NYSPSLNKLPYFIYRLGIKVSWDDEQECLDGKLSGL

>model.g112.t1 Augustusgene.g112.t1 JFAV02000080.1:95033-95449(+)

MVRSTLTSVPYSKMNLQISYNLYKHGFLTSLQRGSTKGPDFEYTEVTPDNISTRRLWVGL

KYRNNQPVLSKISLISKPNLHIQLNHTDLLKVCKGLTVRKIKPLQPGELILVQHKGIVME

INEAIAKKKDNCLVLLRAK

>model.g102.t1 Augustusgene.g102.t1 JFAV02000080.1:78385-79449(-)

MFLQLSKQRLISLDNDDVQDLTLYNKNHTHKSKFTSKIWKNISNGILDFWIECNEYLVSQ

GISSQNVEFGSFAGHTFTIFYFLVRYIQDNLLKPNKDKLKRFEDFFDLSNSLTLQRYEFY

EQFASFSGIQRTLSASNWFDLYLKDINILVQVLAGFIVLSNVLITITFLVWSYKDYSLFH

IKTVPESNNIHARSLTELYGNDEHRPSFTQQLRKIYHFFTGSRVNQSAKGTDVFYQFSRW

TPSPLMTALFTSFNPVVVIFLYFNDTKSKSLISLFLLQYVMKCLVMDSFVRARKEEQVFI

QALVEESTSKLLTGYTGSTQSATSPSDRKIFITHSLTGEVIKEAYNYKKQEFEII

>model.g118.t1 Augustusgene.g118.t1 JFAV02000080.1:104486-107851(-)

MSKRPNVGENTALLNEADVEMGTTDVETHFTNTKRLKSFDHSTLSEPVNSINLIPSNDFV

KHKTNGYTTSTQDVSAGGLANANTVSSFEKELQTMANISPSKAKTPNSKFNNRQVWSRKP

LPLDFHPSTHDISFQQLDAEEATVSGLQDNNTSAVVRFFGVTDEGHSILCNVTGFKHYLF

VPCPPEFNANSHNVEQEIASFLKSLNEQYEKEAGKIDSIKIVQKQSIWGYSGDAKLNFFQ

IFVKEPYTIHKLRTGFEKGYVQYNSWYSQGCTTYDNIAFTLRLMIDCGIVGMSWITLPKG

KYRMIEDPMDKISPVQLEVFINYNDLIAHPAEGEWSKNAPMRILSFDIECSGRVGVFPEA

QVDPVIQIANVVSNQGENKPFVRNVFTVNTCAPITGSEIFDYEKEEDMLMAWKKFVVEVD

PDVMIGYNTSNFDFPYLVDRAQALGLPEFPYFSRLYHSRQKIKDAIFSSKAQGTRESKTV

NIEGRLQLDLFQFLQREYKLRSYTLNAVSAHFLGEQKEDVHHSIITDLQNGDSETRRRLA

VYCLKDAYLPLRLLEKLMSLVNYIEMARVTGVPFSYLLSRGQQIKVISQLFRKCLQIDTV

IPNMNSQGSDQQYEGATVIEPIRGYYDVPIATLDFNSLYPSIMMAHNLCYTTLCDKQTVD

RLKLVKDEDYIITPNGDIFVSSKLRKGILPEILEELIGARKRAKKDLKNETDSFKKNVLN

GRQLALKISANSVYGFTGATVGKLPCLAISSSVTSFGREMILRTKNAVEKKYSLENGHTH

NAVVVYGDTDSVMVKFGTTDLKESMRLGTEAALYVSGLFKHPINLEFEKCYFPYLLINKK

RYAGLYWTNPEKYDKLDQKGLASVRRDSCPLVSIVMNKVLKGILIERNVQGSLDYIKQII

DNILQNEIDISKLIISKGLAPDYTNPQPHAVLAERIRKRDGMGPNVGDRVDYVIIGGNGK

LYTRAEDPLFVLENNIPIDSKYYLNNQLQNPVVSIIGPIIGEKKAQAMFVVKSIKITTGK

TGALMGFIKKVETCKNCKSPLRNEPGPLCRNCASKTTEIYLKSLYEVRDLQEKFSRLWTQ

CQRCSGSLHNEVLCSNKNCDIFYMRVKTRKELQEKSESLSQW

>model.g85.t1 Augustusgene.g85.t1 JFAV02000080.1:44272-45783(-)

MLFKEITLPTGTKYEQPLGLFINNEFVESHSSERLPAYNPATGKVIVEVQEACAKKDVDL

AVEVATKAFKTWKKVPAVEKRDMFFKLAALVSENKQLLAEIESANSGKPVENNAKGDIDE

MIDVFKYLGGWIDKIDGETHIPDDKRLCLTFHQPLGVVGCIVPFNYPLSMMTNKFAAIAA

GNTTVFKSGDQTPISALFFATLVKKAGFPPGVFNLLSGKGATVGDRIIKHPGIVKVAFTG

STAVGKMIQENTAANLKPLSLECGGKSPAVVFDDCDFEQAIKWCALGIFFNTGQICSGTS

KVYVQDTIYEKFIEAMKKYVEETYIVGDPSVESTLVGPLISAKQHDRVTSYIKKSVEEGL

HLVTGGLEPPATIASDESLKQGYFVQPTIFRDLQPHNTIAKEEIFGPVMAIAKFSTYDEV

IDLCNDVDYGLGSAIFTTNLQKSIAFAKDIEAGIVWVNSSNDANSNCPFGGIKESGNGSK

DLGSYTLLNYMNVKSVQINLSSKL

>model.g71.t1 Augustusgene.g71.t1 JFAV02000080.1:17629-19230(-)

MNMNDGTPHDMAPPMELDLIDFFIFLFYTFGVMFFSLLCLVMMYVIVKNIIMRLFGLVVA

NSGDDSSTDHTRTISLLDPRYVDQRLNDQTRGSRSQRYDLTKYYDDPHDVELELEKMPTE

EQFYYKQGEEYMKHNPPFLLLNETDFDPIMTEQTMTYIEEEGAGAWEFQPDQNLPNDTVL

VDNKTELRFLNYGYEASIMTTLPIPYKNRVYYYECKVYEMNTSDSSQEDADNLDEDLEAS

RNKNEVVSIGFASNPYPSFRLPGKHYHSVAYESNGGRRMNSSFPLSPELQHLLPKVQKGD

VMGIGYRVRSGTVFFTHNGKKVKEVEVGGHIRKWRLKYLYPIVGANIPCRVSCNFGTSGF

VFVEANVKKWRFASMNGLKMPPPAYGNVGSDTILEEGHRDVTEGSIYMANRGSFSGSQTS

KKTVGLTEEHLRLLDDNHGDDDDYEDGDMASHYHDDENLPRIEGYNVDEEVENSDSNASD

MTNEDTDNDADSLGPPPPDFNFSNPPPNFHSDAISLNTLPPHNPPEYISDLERN

>model.g104.t1 Augustusgene.g104.t1 JFAV02000080.1:82482-83885(+)

MESKTEKRAKYINNLLLGRKRDVKYESSEEQADLVNAEDQEYIDTDQGKDQSDENSVISM

VKELRTETTESKEETLMAILDCYNSKFLHDFKPQYHTPYLNFFLQHTLPPQMLALDASQP

WMVYWLVNSLYIMQDGDIGDELRTKIKDKLLVQSSVSPLSSSQRPAPFGPFAGGVMQYPH

LASNYAALLSLVLCEDQEAFNKIDKDYVYEWLMGLKLADGSFETCEKCGETDTRGVYCAL

SIANLLGLMSEELVENSLDFLIQCQTYEGGFGGRPFEEAHGGYTFCAVASLVIIVDYLHG

CKKTLNDFIDVDMLLRWISSRQDNEAYGINGRSNKLVDGCYSFWIGGTGSILEAYGYTNP

IDKEKLRAYILQCCQSDEFPGLRDKPGMRSDFYHTNYVLLGLCCADSQFHLKSSTQSTLN

IEATITENKDMPGIAKINPMYSLPCSTISKVHSWLSEESGKKENLDSH

>model.g113.t1 Augustusgene.g113.t1 JFAV02000080.1:95724-96749(-)

MSDTEVKPEIQSAEQPVTGNPSEDVVIEDTAEKSPEQKDAGSGDKEEQERLKKLEEARKK

VEALRNRKKNKKSKKKDTAASAGETSTEGTPVPDSKQNETPRSTEKVAAESEADLKPAKE

EGKGGEKSDKEKVLEETSEANEPQQDGEALKENQEPVHDELDDLFGKVESQTGASSSVGR

AEATESFLATIQKEQESFELEQYKSKVSDLEQQVKKLKFDNMEYETTIDDLQDEVSELKA

QLAKTQQELQELKSKETVSAKPPLSTAPSTLQLSSFSTVQQQAKSYQPSSPYTPQPYGNL

PQQENTTPVQPVDQDLLYAKWKDWNVDMTSWRSIGTGPIVQF

>model.g72.t1 Augustusgene.g72.t1 JFAV02000080.1:20159-21187(-)

MSSTTTATSTATSTITSSAIASSSSSDFQLYRYQPKKGLAYVASILFGLWTVMIAIEIFV

KYVKASHLFKSTTSYNLNRGLIKKTLSSVCFKIYIPLIIGAMMEIGGYISRIFSSKDRYA

KIPYIAQSLLILVAPGMISATIYMLFHRMIKYLNVPDNSRVGIFIKDSLLVKVFVAGDII

SFFLQAGGGGIMSKESTVHTGEVIIIIGLIVQLVSFGIFLFVQLHFTLRYRNFSKNFYYL

EVSDKWKYLNYSLFAASIFILIRSIVRVAEFVQGFDGFIASHEAFLYIFDGLAMLLTLVV

VSISFLFTDIGEMFYQHFTITYAQNETADKDFAYKTDTSDSYN

>model.g73.t1 Augustusgene.g73.t1 JFAV02000080.1:23021-24073(-)

MSSTTATQTTDAPTFTDSVNNAPHEYKFYNYDPSKALTYVAFILFGIWTLIIGIEIFVEY

VRTVNLFKTTLAYDLHRAQIRKTIKSVCLFSYIPLILGGLMEMGGYIGRLASIYQTHTII

AFVVETVLILIAPVLISATIYMLFHRLLKYLNVPENSRVGIIFKNSLMVKFFVAGDIISF

LLQVAGGAMTAEESTMHLGEKLVIIGLIVQLVSFGVFLFVQTHFTLRYEKFSENFFHLEV

TGKWKIFNYSLLMASVLIFVRSIVRVAEFGQGYDGYIGTHEVFLYVFDALLMLMTLMVLS

VSFFFTDIGEIYFQHFTITYSQNEAQSKLTACKTSTIESYEENSNNDFSVV

>model.g84.t1 Augustusgene.g84.t1 JFAV02000080.1:43276-43848(-)

MREHRLRRMFYSIKDRFNKNKHLNKAGFLGKAMIPSYTKRDSQKWPDFRAFAKNHLDHDA

TAGKNDHHGNCQTKKRYEQENKHDNTKQLQEVMSDIAKIAQEIAQKKAKLASYKRELEYL

ANGDWCEDTILVKPDMIGDKLCQFLEWKKAECVYLMKEIERKEKREKKMILQAKRDFVSL

KDLCVKLESAS

>model.g128.t1 Augustusgene.g128.t1 JFAV02000080.1:132577-133992(-)

MSADSYVFQSVINKKPPHVIGGKGVRMTVEKDGVVYEDILDGVTGAAVGALGWGDEDVLE

IINDAAKNSSYTYPSLIGNQASEDLAKFYIDHSPEGAFASALWCCSGSESNENALKIIRQ

YWLERGKSKKTKFISRETSYHGFTLGALSISSNGRAEMFKEVLIDQDKTCLKMPVCFPYR

YQKEGQTTEEYVKYLLGALEKLILDNDPETIASVTVETLPGSSLGTAPPPPGYLPGIRRL

CDKYDILMHLDEVMCGTGRSNPNGGLNCWENFLEPGQFPDIQTVGKTLGSGYVTIAGVLV

SPKIKDAYVQGSGQIVGSHTYASHGFNCAVALGIQKKIMEKELTKNIFEKGNLMGKKLQE

ALLSGEDCSSNIVGDVRGIGGFWSIEFVKDKKTKEPFAKKLDVGHKMQDFCFNNKLNVMG

MQGSCSYKTGEGDFILLAPSFIITDEDVDEIVKRTVQSIKDCTEWLKKEGAL

>model.g63.t1 Augustusgene.g63.t1 JFAV02000080.1:1-299(+)

DVDLSSVDFGESDINVFQRFGQNTSWTSNFDVSGFDLNGNTFWDCNGLFSLDVQHCVVSL

GRAGFYPSEIGLTRRPLKAHMKVNKEGATERDKGYKEWA

>model.g107.t1 Augustusgene.g107.t1 JFAV02000080.1:87064-89934(-)

MAKKSAATSKPKEKQTGGKAKSPSPKNSSPNPASTHQEQTAPVFSLDYDPIEDFSIEKGP

VRQYLSVDVSDTLAKLRKPNTSFEWIVLTLLLILTCAVRLYGLSYPDSVVFDEVHFGKFA

AKYIKGTYFFDVHPPLSKMLFAGVGSLAGFKGNFNFENIGDFFPKDVPYYFMRLFSAFCG

VLTAIFMYLTLRCSGVKPWIGLLATTLFVIENSFVTISRYILLDAPLILFISGAAYAYKK

YEIYPEDSFKSFKSLIFTGVCLGMALSSKWVGLFTVAWIGILCALKLWFYIGDLNKPLIK

TTAVATKWGSILLGLPIVIYMLSFNIHFKALNTDSEGASILSPAYRASLDGNHVPNNIIG

PVGFGSVISVRHINTNGGYLHSHNLFYEEGSKQQQITLYPHIDGNNDWLVEDYNNTNVEN

YTTFEQLRHGTKIRLKHINTLKRLHSHDHKPPVSHQADWQKEASAYGFENFEGDPNDDFI

VEIDRKLSKKGEAQEVVKAIDTKFRLRHAMSGCLLFSHDTHLPDGGQQEVTCAHSGIPEL

TVWYIEANTHPLLANDPKVERVSYPKFGFWEKFVESNIKMWKVNNGFTESHVYQSDPTTW

PFLTRGIAYWGKDHRSVYLLGNAVVWWLSTATVAVFAGLILFELIKWQLGYELSSEKAVI

NFHIQITHYLLGWFIHYIPFFIMGRQLFLHHYLPAYYFLIMAFAHLLDLFVSHILSRKKI

IGLSICVALLSSTYYFFETHKSLGYGTPTLKETCKKGQWMSGWDYSCDAYLESWEEYSNY

TLSSEGPKQTSFQATNINELFHDDGSGNKQFDSNLVFEQDKRLAELNNQQPQQPYQQQAA

NQAGPAVEVGVEDVKNNQGPKKFIGPDGQEIDEEVALQMIAEQGGRLMKVEETSTKGGAT

QSAAPTQKHPNVGIEDIMKDEGPKKFMDQYGNELDPEVAMGYLNQGGHIVNVERHNQ

>model.g67.t1 Augustusgene.g67.t1 JFAV02000080.1:6577-11046(+)

MFAAIENSTSAALLAKYLPQNEQKKLITDLIQNEQYFELKNILQSVDFVSAVIEETAPIV

SQKVIEKLSKFVLITNADDLESNSEVSDVTLSYLSEIINYFPSVFDQLNEFIVSSILSYI

TNNKFTLFHPAFEAQFVNSVVSSSSVHHFQEDQHKEVDITKLFNFLEKVFLNFESYCNKT

LDKLLLLLINCKNDQISTLSSKLTRWRTTSILSYVSSDQEMDEFVWSLIPYLLSPTANEN

QKSFLKKNTYSVWLRLLMKMFPVVNTREKSRKVATTDNSICFCNFLKTEDYWHLIQLGLS

QDSAEIRKISLSILKVTLQACTAVPFYMDLKIENSYIKAHPSKNTDQWKKFTTLYEIVAV

DTALNQFKDATQDFLDLFKSSGVSMIDPSWGLIILSTGMKSSMEGVRKYSLQLLFLIQDR

SVFHSKLDEMQAVFLPSAMIAANFDITTTPETTSQLTCAYGSQFVQFVKDLIISSYTCHG

DITLSTERKNCMLDCLLQTLSDFRSSYDPARIYLVLGIYQALLSLSLNSSSTHLLLNNTH

IEMLEQAVFKVECEDKLFEMVLQITALKMLCFYNTNDDTSSPYQWLSAITSHAKKFGYQF

LEYVMEDLREFCVVNFNETDLAAATIDSQDVVFNVLSFCLLSKVDKTTLDITPEFLLELS

KAGTLSNFNKESAQLITEIYNGNATTAADNILPLLQNVENVNWNTFQLSQIEKNLLSSPF

EPHQFGNLVCLYEQVLKESMQYPSMKLEDLLDHYTRIGEHSEQNKHLNSKHKDSWFKTYF

QLVQLYIKSNGISEIEELQKIFQVMKKNLSDNGNYNANLEMIIICKYLFDKVIVFDSQYD

QVVVDDMRFVLNEIWEIVSCERLVLNQRALHLTFIETFFHKQMLHRCNSKGIDNSSKEEL

VKNLITNGKQIIDASTTRRTLMPLLSEQLLNFVNGDAKELDNNIWLIELVKHGMLTCPAD

ANVYKFKELMGTVFDAQFSNEFKFFFPQGLYQHIYGEPEIMGKVSLAGCVLKSSKEFQQA

LLEDLFLNEANMFKAVKRNDGNEEMQRIIKWQLATLVLPNADNDFLENHVCDQLIPSLVD

GESSPSCRYYNEWIVAFSIVKGQKESENKNLTCLMNMLQSETIIDSPVWLVSCVRVLFLI

MSYLEDLTLIKKFLTILISHCTSNKPLIRHFCNSLILSVWPKFKDVLNKNNEFELSQILE

KLYDFSKQTQVHGQYRSGDANVWDLFTDLTLTDIFGGLIYRISDHEVPFYIDASSFESKL

GSSTLNTPIKIGTDDKSLWLKKRNTKLHHEEMMNFKKTISLDALEAGKNATPLQTKSGAW

NLVFDVDNKKSNANIERSDLIVMASLVDKPPNLGGICRLCDVLGCGLLTVDDIKVKTHPQ

FKNVAVTADKWMPMEQVKIDEIVDFLTQKKKEGYTLIGLEQTDKSVKLDNNYNFPKKTVI

VLGIEAFGIPGYILEHLDLCLEIQQFGVIRSMNIQTATAVICYAYTIQHM

>model.g111.t1 Augustusgene.g111.t1 JFAV02000080.1:93879-94631(-)

MFKTLLRKKSTQQIKKLASQGSFARPKINIRSQRCNSTASSTNAQGKQSSGPGIKKIALV

GLIGTAIFVQAAESLEKAKPKNSFTEAEYNTYVMNGLRRKKLMLSPESFPPIKFLLNAQG

AAAAKTLSEDNVYVVDPVTVVEYYRSNETSKYEALLNEIFERNGSNKDILPEKLPQGMIC

MLIGRYIQDHSLAENIESKTSIIIKNFPKNLKEAIKFENDVGNVKSIIVGENDPAQSDIV

DYYDTVKKVEK

>model.g98.t1 Augustusgene.g98.t1 JFAV02000080.1:72172-73143(-)

MITQVFGFIVTFLYTIVQIVAENTSIRLHKTGKTLTTDQPESPSENVSVAKDEIYQFVTV

FGGSHNEYGRALCQKFAKKPHIFVINIDSPNGYFSRLPVSKYLQIDCTDFAKFELLEECI

DKMENVICATKRSMYPSTILVLNNIDIGVDNIGGNRGILEEKFSIFMHTTTCNATNVMLI

FKEIIQRFQENHCLYLINISNVVGLYPRNYKDHSYAYSCSKAAVIQLHQSLTYEENKTKK

TSSKGIKTLLIILPFSSREPSIWKRLFMISNKNKNNRFRKQVNESIHALTNGRRGELYLA

DEFAENLKVHAKYKFKQFNEINSF

>model.g70.t1 Augustusgene.g70.t1 JFAV02000080.1:15412-17430(+)

MNGNNDEYEYEAEGFSFNQAEPNNFLAQTISHHSMEHMPDDNNMEIENNESYYANFDNNN

GSNNDHNNNNSNNNYQPSDNNKNEYNAAVSVGSTSEEQSLPSLPPGESVSPEQQQQQIYN

QQQQAHSASMSKNKRTRATGHALDLLMREFEMNPSPNPELRKSIALKTGLDEKKVRIWFQ

NRRAKYKKTSSSTNLAYMGNQQPFGIVPNNGYENMNNNNSIGHLNIGTSTVPGNDEHYDM

EQMQQHGYSDNSNNNNAEENQMYRASATDMQYSDEFEFDGIPWDMNINYHFIDAMSLTVG

SWKRIKSGHLDDSSMPDVTRLSNLSPISINDIMADATDLLVLISKKNYEINYFFSAIAEN

SKILFRVFFPINSVVNCSLSLTKKDGSTTINSSSMSSPNGNKVKIEKKGFSSGPNNNNIN

QSSGNGRNANNELQKDNTADNTEENEDEDDYTELRLLLNRAPKFAVYFSDVVDEAASNQW

NICDDFSEGKQVSEAYLGSNEIPHVLKGTEESLKLMNSLILEYNNTNIYPSTMAMPPLSQ

SLHYQHQNTHQQQQQQQQQQQNQYPHQQQPAQHSPYPFHQQPTQPFQPASSQIPPTSFMS

PSNFGFHSSDVFMQSGFTPDPTSDLLMDNAGLQSSGPTPGHLGNTNMMGSTSNTIPETPD

FNSLLNFQTNDEE

>model.g123.t1 Augustusgene.g123.t1 JFAV02000080.1:121921-123252(+)

MSHEAEDDLLEYSDHEEQFQVPDSTQGVDAAGDSTAATTADSTGADGDKKGSYVGVLQTG

FKDFLLKPELSRAIVDCGFEHPSEVQQSTIPQSIHGTDVLCQAKSGLGKTAVFVLSTLQQ

IDAVPGEVAVVVICHTRELAFQIRNEYLRFSKYMPDVKTSVFYGGTKYQDDIDKLKNKET

CPNIIVATPGRCKALVRDKHANLSHVKMFVIDECDKVLENADMRQDVQDIFRATPREKQV

MMFTATLNDEMKPICRRFLQNPLEIFVDDEAKLTLHGLQQFYFKINENEKNKTLAKLLDD

LEFNQVIIFVNSSKRANELTKLLQLSNFPAITVHGMMKQEERIERYKAFKDFDKRICVST

DVFGRGIDIERINLAINYDLPKEADQYLHRVGRAGRFGTKGLAVSFVSSAEDEEILEKIQ

SRFDVKIAPFPEEEGIDPSTYLNN

>model.g124.t1 Augustusgene.g124.t1 JFAV02000080.1:123765-124190(-)

MFTIFPQTFGKKKSATAVAHVKAGKGLIKVNGSPITLVQPEILRFKVYEPLILVGLDKFA

NIDIRVRVTGGGHVSQVYAIRQAIAKGLVAYHQKYVDEQSKNELKKAFTSYDRTLLIADA

RMPEPKKFGGKGARSRFQKSYR

>model.g114.t1 Augustusgene.g114.t1 JFAV02000080.1:97261-98313(+)

MDFTVDPNLSDLINSTTHKWIFVGGKGGVGKTTSSCSIAIQMATKQPEKQFLLISTDPAH

NLSDAFGEKFGKDARKVTGMNNLSCMEIDPSAALKDMNDMAVAQNNNNGGNGGLADMLGG

GGALADLTGSIPGIDEALSFMEVMKHIKKQEEGEVSYDCVIFDTAPTGHTLRFLQLPTTL

TKLLEKFGEMTSKLGPMLNSLTGGGNMDLTGQMNELKVNVEKIREQFTDPDLTTFVCVCI

SEFLSLYETERLIQELNSYDMEVSSIIVNQLLFAEYDEEHNCKRCQSRWKMQKKYLSQID

DLYEDFHVVKMPLCAGEIRGLNNLKKFSQFLYKEYDPVADGKVIYELEDKK

>model.g96.t1 Augustusgene.g96.t1 JFAV02000080.1:70320-71234(+)

MQLLSWVFTALMAVSMLKSANAISNPQLLAGNSKQFLDLSQGVQKITLDYNTNELLKFQF

EYNPAPAPEPTNEEEGKEEEEEESAPVVPNTKPQHASLVLSVDNKIEQPYMIKDARDNVY

SWLLQSSQLPKSLVYYSAKWGKPISGKLYFKSAEEAIVDELFFEIEFDQTTISDQVLSSL

ISESPEQDKKTSVKPEIIHQFRAPPKQANGFIVSVFIVAVLLGALFTLGSWFAAVDFSKN

HITMKKQCGTFVYLAGFLIGILGLEFSFFQYYLGNNDIFRTLYNVFVLFTLPTAYAGSKL

LRRLY

>model.g108.t1 Augustusgene.g108.t1 JFAV02000080.1:90545-91912(+)

MSLEQADTYFQNADAYPQAIKEYSAVFQSNKDKIVDITSNNAVSSAISKILQEQEHVILR

LGELYGATHDLSALIQLIELSKEFMMTYAKSKIAKILKVLIDDFEMIPDSIDAQIKVIEE

SIDFADLHKRIFLKNSLSIKLANLYYDKQQYSLSLNLINTLLKNFKKLDDKSSLVDIHLL

ESKVYHKLRNMPKAKAALTSARTAANSIYCSSATMAELDMISGTLHCEDKDYKTAYSYFF

ESFDAFHNDSTKNTSALKSTNNGDNKINNKDRSNKLFHKSEQVLKYMLLCKIMLNLIDDV

NQILNAKYTKEIYQSKSIDAMKTIAEAYSDRSLLEFNKALKIYETELKTDDLIRSHLNAL

YDALLESNLSKIIEPFDCVEISHISNLIGLDYKQVEGKLSQMILDKVFNGVLDQGNGWLN

IYGNEVCDPTYDSSLELIGNLNKVVEQLFEKAGVLQ

>model.g90.t1 Augustusgene.g90.t1 JFAV02000080.1:54433-55602(+)

MTSSPDLSLSSTIPVGYQEHPHHYGSKPNAFEHGTNGPKRSEKSSNPPPSSTSSDKLSIW

YNVIAGGLGGVVADSAMHPLDTVKTKQQAPTLTSRSTAQRASFKSTFQQEGVRKGLYAGY

SAAVLGSLPSTAVFFGTYEYIKTILIDQKQYNETLVYLLAGFSGDLVSSVIYVPSEVLKT

RLQLQGKLNVGLFGYKSLRDAIHKIYKTEGLAAFFYGYKATLSRDLPFSALQFAFYEKFR

QYALQVQKNNNGCFDQDGHKHTHSAFVGEDEELNLVSELLTGASAGGLAGIITTPLDVMK

TRIQTDNSRSGDKSNFSPNSQTQAQSHQILRGIRTVYQKEGLLGLFSGVGPRFVWTSIQS

SIMLYLYQSALKAMNFESEVNKVKNKKNSY

>model.g110.t1 Augustusgene.g110.t1 JFAV02000080.1:93152-93820(+)

MSTQQSNFGRRTWDRDEYLQLKKSNQLQSFHANKLTTLTTDNKQLYDKLYQKYGTQNYET

LLSTQLKDVNKLMLKSSNDEGFTARRGKKFGFYCELCDFTFNNNMQFVDHLNEKPHFDNF

EKIFGEEYVRDKRDNDDIPLSEFNKTVSSLLKQYQKELKSESKKNLSSKNASSSTGASIQ

VTKPPNEDNLKKNKPKKNKKKQKKQPNAAKDDIAKIMGFKAFK

>model.g93.t1 Augustusgene.g93.t1 JFAV02000080.1:64049-65686(-)

MLKQLSRSSVSRRLLTLGSATSPKSQLLTTSSKRFITSTTPTPTPTPVKKTSFLKKFAYV

SLFSILGVSSYITYCLYKELNPKPQTAKSLTHENGSKRKNIVILGSGWGAVTLLKHLDTS

EYNVTIVSPRNYFLFTPLLPSTPVGTVELKSIIEPIRSITRSAKSQVDYLEAECTDIDPH

SKRIHVKSEANNYEMDLEYDYLVVGVGAQPNTFGIPGVMEHASFLKEIPDAQQIKSKILY

NIEKAASLHEDDPLRKSLLNFVIVGGGPTGVEFAAELRDYVDQDLNKWMPSISKEINIVL

VEALPNILNMFDKKLFNYAQDLMAKNKIDLKLKTMVKNVDGQIITAKIGDKIEEIPYGLL

VWATGNGCRPITQNLMSKIPEIQNSRRGLLINKKLELLGAEDSIYALGDCTFHPGLFPTA

QVAHQEGEYLGEVFHKKFLIDQLQYASEQKGVTQEVAKKNEFKVKKLQQKIKDFDYKNQG

ALCYIGSDEALADLAIGESKLRLSGGGLTFLFWQSAYLAMCLSVRSRMLVAFDWVKLHFV

GRDSSV

>model.g119.t1 Augustusgene.g119.t1 JFAV02000080.1:109004-109702(+)

MTEREYTKEQESVTLEVLKHDKHDFYKILKIEKTSNDLEIKKAYRKMAIKLHPDKNSFPR

AAEAFKRVNRAFEVLSDQQKRKIFDQLGVDPDDRHAASAAGRGSGFGNDAFRQQQGSPFG

GPGGMGFASPEDFFQHFAFGGPFGGGGPFGAGAGGPFGGGGSGFQTFTFDPSTRTFHSRS

SGAQRRRNAGGATTAGERQRQNRENQEEDFMPWRIIMPFLLLFLFQVFEKLLR

>model.g75.t1 Augustusgene.g75.t1 JFAV02000080.1:27092-30390(+)

MFGNLAGAECAVGQKIKWILELVGLFNQFASPIFWDKQEFFEDPPIVMNNFTGSARRRNI

NLSTADRKSRRDLLDKASKERERRAAERKEFLKAVVITKNIRRFLTLKRLYEQLFLQCRD

HTLDFSVYPVQSLISVFYPSFLQFDLPNEFVFTRFLLDSLDMQISNLGLLNKKLLLLLNE

MSNLHLPSTQPYISKIWSLISARGINEKKFVVEQINKFLENIAPYQLDDPNMILCLNGFL

QNDKFTTAFIRHDFVLKTNILLYPVFQKLVIPTAFFISNMNYKLDTMQEVLNVVINLSFF

ANSHQPYYYLDFVLKTLSTNENLIKASDSSTILKKYDPNIFLIYDYFCNNLINEYLNKDT

QSLEQIFLLLKLCSASAYQKSIINFLVQGNLLQKVYKNVVELNELQYLELLINMSNVYLF

TKTDFELLGNDSSARMKTNSENNLSLNQLLKLTICLKELLFNTLWCQNSDTISPAISKQT

IEKLLKLLKMLQVRDNRAKLFKDPSVWTIHNEEFDNVDITNLFIQFEEYYREISDSIEEE

TAYDDVLKTAKLRDQSSEIVDRFIERKKLMRQASKRQIQKFQIMGKFPSFISFEERVHWF

QNVLIGKEKERLGVDNDDFWLSWVRRTNMPRIEATISRNNVLLDSFNAFDFLGEKFKGKL

SLTFTNEFGEEAGIDGGGLTKEFLTSVTANGFLEHPELFTENVDHEVFCKPYNKITQNNY

KLIRFMGKCLGKCLYENVLIDVQFAPFFLNKLLKCDRFAGGYKSSLDELSSMDSILYKNL

MQLWTMRTDQLENMGLAFEITDDENPYGPMIELVPNGSHIPVDRSNVLDYMNKVADYKMN

SKRMLTSMKAFIEGFNTIIPQHWIGLFTPLELQKLISGGESHIDISDLREHTEYGGYLAT

DQTIVDLWDVLENELSNEERAKFVKFVTSVPKAPLTGFKALQPLFGIRNAGREIGKLPTS

STCVNLLKLPDYRDKKLLAAKLRKSIHSESGFDLS

>model.g92.t1 Augustusgene.g92.t1 JFAV02000080.1:59924-62635(+)

MSNNQNKANSGANPGAHQKMQNWMQFEQQQKQQQQQQQQQQQYYQQSQHARTHQQQQQQQ

QGAFPSSQTFNNKDAFEFNQQAVVSDTMAKNSRQLLYAHIYNYMIHNGHYESANTFLEEA

DVPISRPRNEVHLKPHELLETKVLMDSSDTFLLQWWKSLWNLNQFVKEQPLEYVTNMRPF

IDAITPILPISQQQRPPVNFNHPPPPPMQQQQKAQQFPQHQQQNAHPSQKQSYPMQPQQT

QQQAYFSRAQSDMGNPALRSHNTVENTNASSKKVAMNSSASSQPNGYRGPPLQQNYTSQP

GPPSSAPTSRTGPKEYHGQAQPQFDSQLSQPSHMHGFTSTSVPYQTEAVFKDPNSAVSTP

SISNRPPNDPQPLEQYQQHPFGQQQFAQSSSELSTPYNANFPGNFQRPNMVQQAPTPGHQ

MVYGPGGAVHGSNGNFQTKANQFVKSPPVYGSQNGESPSMFPQVAVENNGNSGVYNSQST

GPSTVPLAQQVAYQRKQPASTGASRQSSTLSAKQRQSLLTQIVKQRQPVHQSGLSGVNSP

GTQSSGFLEYIPEEPHNSNVQDPPSKANTHATNPSFLPNKKQNNGAAYNVYSGTTPSAVE

GNHPEMVQPAVGGHQVVVESPKISVKNSKGKSKKKTDRASNSNSGTKSRAKEDNVAQQQH

YQQRQKALKAQQHYQQLIMQQQAQKQGISSTPGPSDTDNFNTTAITPKATGETRVNNDSK

TPNIEASRISKQDSAENRTTPGTTNYTNKSTNSGVPKKVAKKQQRSGSTLSKTSAKSKPS

ASDSNSVAKNLDTGNAENAPIAKFSQTGGESSHKDAAVVNDENVNGDNGSVKPSSISFGR

HTPGLMEDGHMSAPFLLEDLNFDDLASSMDKDHGLGQNHQLDQFSKDAADLFHLEDFGAD

FMDK

>model.g103.t1 Augustusgene.g103.t1 JFAV02000080.1:79790-82024(-)

MDTYGSRDSLCKKAGDHEPKAPSQVSQPPTWTDLNDIRWQIYTSSRQNNKITLSVGSVGP

KLKENCPRALQKAILKVLQYNGKLPLQERFLWLSSFYLQRAYPPISLEETGGYTPQFLIK

ETEVPLDSRDFPDYKIRKPLFNEPSSKIGTPNLIQHPKVGDCSFVCSLINLERAHELQLS

IASKNAPNTYMVNFHFNGSDSRLVCTTSVADIPTTNTSDQLGLYSNDENHKILELAYLKL

KGSSYNHVGSNTAIDTFLLSGFIPEILKIRDLPTWEKLLHFYKSGLCTIAFGTKKLFSNA

STKFISGHDYAVHDFSDSQISLINPWAPDIPIEIKKWSTFIEYFDVAYVNWNSKKMFRLH

EKIHFSYNAETNNKNIRSTLNKPAFIMKNDSLSDETVYVLLEKHLSQEHPGIHLEVVDNS

IAVSGLTKYKNNTGFHLLKVLIGSKTQKKICCFSELSARFTLHMYSISDRVTIKKAEGKN

VCSVSNVFLNNYSLGSEKYCMNQTYKLEFADTNTDNKLHDNDGSKSAVFCTVQLLAAKSH

INFQLYHLHDFSLEHPLISENVYEFEMQTKFQTPLLRNKQYKLVCSSKDAHCGTAFSLAC

STDDANTRPKLTEYYNYFGGLYKAKISAADFTAEETRTGTKLSVLLKSEMGSSSDTWNIR

LFNVFGKNKNFTWSMKVIDQEYGDSLLSTPSGNPTFTSNTAANVEVNANQIRTEVIDLEM

LFTENIGLTLSNLVMEIGSQHKLSK

>model.g129.t1 Augustusgene.g129.t1 JFAV02000080.1:134970-136388(-)

MTQDAAVQETHIFQNTINKKLPQVLGGKGINITIEKDGKVYDDVIDAMTGAAVGALGWGD

EDVPKIVAKAVENSTYTFPFYVGNQASEDLAKFYIDHSPKGAFASALWCCSGSEANENAL

KIIKQYWLEKGQPKKNRFISRETSYHGFSLGALSISENNRANDFLDILIDQKNICLKMPA

CYPYRLKKEGQTTEEYVQGLMDALEKIILDADPATVASVTIETLPGSTMGTPPPPPGYLP

GIRRLCDKYDILMHLDEVMCGTGRANPNGGLNCWENYMEPGQFPDIQTVGKTLGSGYVTI

AGVLVGPKIREAYEKGSNMIIGSHTYSSHAFNCAVALGVQQKIMDNGLTKNIFEKGNSLG

KKLSDALIPTDNIIGDVRGVGGFWSVEFVKDKKTKAMFPKDLHVAYRFQDICFENGLTVM

GMQGCYSNKTGEGDIALVAPAFIITDEDVDEIAKRFVKSVEELSKALKEEGEW

>model.g127.t1 Augustusgene.g127.t1 JFAV02000080.1:129900-131822(-)

MGNVPGKLEDGSDGSVRSRSRSNSNLSITSSNGTLNSRSRRNSNQYAANASNFTSTNSTA

SIGNNGNTSSNHKSLGSRRHSHTAASPIATTYNASNAGRQRRAGSLVGSLLASTQHAFGG

GSSPTTTSTSLTMSSTTPNGTTPTRSRASSIFSSSNYLDNPYRKRSTRERQKFKEEHYRG

LVVRFDEVVDGGYLAPYGVHDDEHKLDYNDNVVKRLIIERKLQPFYLPLDDYEPEWDDSE

LLKWVHSLTLHQPYSASVEKYEDLPGVEGLSVKELENSENIENYLDSCLSKTERKIQRSQ

IFTARLYKRKIMWQQYETDLYLNEKLKVDAEKKEKLKGSQSQSQLQSYRCESWVPNDKLL

LDLYRNGTECPICFLYYPGPFNISRCCEQPICTECFVQIRRKDPHYPAHENDNENNEIDE

IDEEDDTKNNSNNQNNENGYSNASGSTPRRRDSTATGEGADKNGTLISEPTSCPYCATPN

FGVVYTPPPFYRRCGINAVLPGEYKGQNVVPSISATTITTGSAVTENSGIGSPCVSSTGV

SDVILEETEEGNSAFVSQIARRGSLPAHHESVVTSDTIRPEWEVKLNKARERQKKKYLKA

TTIHLNNRLVSASELARMEDEMLDKAIKLSLMSNGNGAAKH

>model.g99.t1 Augustusgene.g99.t1 JFAV02000080.1:73584-74741(+)

MSASETQHVAIEQLMGHGALTKLVNTSTASQRKEETSLESLITNHEICKSYIVAQCPYDL

FQGTKEYHGKCPQYHVSKYAVLFKTKYHDFATSKPAENSDNRETSENDLQVYLELHKQYY

QTLLGFIQKCDVTKELAIEKLESKTEDERLKISEVSKELDNIDESIALLSREIEVLAMNG

ELEKSMAQSIYLSKLTVKREEVAKRVREFASKIATSGKQELQVCNVCGAYLSRLDNDKRL

ADHFLGKVHMNYVYMRAEISRLKAIFKKYKVNLSSISVTERRDTRSLSRRYLHQRNNTAL

PSQSSTPGSGRYNSRFDRVQSGVGRSNFSSYPKRASRFQGGTGSSHSLYQSTTEAPTRPS

RPYPSARFETKAYRSRRSRYDSPPAY

>model.g117.t1 Augustusgene.g117.t1 JFAV02000080.1:102793-104358(+)

MSLKRKYDVFNDLEENEQSGGVQDGHKAKSFHLAENEVVGRLFNMSSGKEHNVFEIRYKS

VFIIGRSRSCDITLSGADISTKHCAFYFVENKAEPETPHLINIIDTSRNGTFVNGNRLVR

KDCILRNGDRIAFGKTSSFLFRYSNASFTANSQHDIKEDEAEPLREETVHGVEAVNEVFK

KPSQSTQPGVRKVLSLKNVVQRPTSVFDNYILGKELGVGHYATVKEGKNKATGQTVAIKI

FHAQKTNDRKKMEQFKTETNILIKIKHENIVRLLDRFIEPVSKSSIQTYLVLEKVSDGEL

FDRIIKKGKLPQEETKAIFRQILSGLKYLHKNGIIHRDIKPENVLLSIKKRTSKDQIQDG

PWGEDELSIRVKIADFGLAKFIGEMQFTNTLCGTPSYVAPEVLAKKTYSNKVDIWSAGVL

LYVCLCGFPPFSEQLGPPSMKEQILEGKYEFYSPYWDEIDDIVLDLIANLLVVEPEQRYS

VEDTVKHPWFADIIPQSQLRVIGRPPLSQNQTKPSSISEIMF

>model.g68.t1 Augustusgene.g68.t1 JFAV02000080.1:11153-12016(-)

MSLLSVTEQEYLRTSLESKNNNNVQPIRPDGRKTDQFRPMGISVDFLPTSNGSTRLVSSD

GTEIIVSSKVECVDATGLSLSDLLVIDLDIQGYRDDSIYVNQMKSILYKSVHSKLIESGC

LKINAKYSFKLCLDVLVLSKFSYPLSLISMAIYTTLQSTSVPMLLESEVEKNANISGRSG

DNQPKDIELNIDELPQFNDYDMKPLNFQSPLIFLIALIGDNCIVDPSELESSVADNCLLL

SFYDNKITAPIKSISLNDSYIKGFTPQHLQKSIKLVEKIAPEVVAALN

>model.g115.t1 Augustusgene.g115.t1 JFAV02000080.1:98816-99553(-)

MSSELNTMSNEKYTDTQDDVNSSKETGENQFPIASLVKHTLQLDIQPHANKNTQEETENL

KTNAEIKSDDENDHNAAAKEDLLEKADNAETNDTTIPNNTAPVKNTENTKVKQLNDSDII

NQLKTRDAIANNFSQFFVPRSELVLHFLPTNNKEDTATKQTQFLEEVLEKCKTQLLETEK

KNSLLDELIETQKLEIRHLQDEMLSMNIENNVLHQELQKKQTEYDNLVERWIAKAQQDAD

AMNAIF

>model.g64.t1 Augustusgene.g64.t1 JFAV02000080.1:718-1710(-)

MKTTTSDAPSIIRIKRKRNEESLQALCIEQAENNDGLNHGSNSNTNKRRKVIFRLKRVES

ANKYEEAPILKSTKQEHHFLFETQGERPETAKSVVPQEALQPELNDLLQEYLQQQQQQEQ

QQQHNNPNHDDQDYVFDVYFKEEINDDEDTFVFDKSTMGYIKIIEDPLVPEEETDISDAN

GLSDDQDSNEEDYYQNDYPEDEDDDRSILFGSDVSDQDSVIEDPNYTRDILVSSSGLING

EYDQAYSRMNTTGDFLDSLNNDRNHTSDEYEYLESDAEYGADQEDFSAPRVAEEDFEDFP

RNQFFATDQDDPLAIHRDKVMYKLQKMINKK

>model.g143.t1 Augustusgene.g143.t1 JFAV02000081.1:15531-16658(-)

MSSITSNKPIASQKQPVFFVSHGGPTFMYREETGSDKGAFDTTAKLGYDIKTKYKPDYIV

VLSGHWQPEYTGSASLNELKIAVPPELSDLNTVPDNHKLYENSLIYDFYGFPRHMYNVKF

KNYVTKDVVKRLDTIVQESGNNMKLVPEVRGVDHGTWVPLKVAKLDDYTDAPDGLNHIPL

VQMSILPSSPKKPSNPVSSFFASRPENMQTSKNGNEDTVDTFENHFHKLGPILQKIRQDN

GLVICSGMSVHNLQDLGYTFMDPFGNKFPYTDRFNQYLFDMITFHKKKLEQFTTGKEATN

EITPTYLLDKLTDLGKIPDLKRLLYQAHAPTIDHFLPFVAACGSLDNLEVVEELYNNKWG

SLGWGIYQFGVPVDKT

>model.g163.t1 Augustusgene.g163.t1 JFAV02000081.1:58998-60731(+)

MAGYIPIDAKKALKKLKEKKKPVSISAVAALALILLFTILFIRKGDLKLPECKDCGAYNA

ITPVFPGSLDEILNDKTFKLSSISHLSGAVQIPTQVFDNSPDPTGKDDDFLWRNFTVFQD

YLQKTYPLVFEKFTFEKVNYHGMLMTWETKNTKGKKPVAYLSHQDVVPVDPTSIDKWTHP

PFAGVYDPTNDYIYGRGVVDCKLLLISQFESLNKLIEDGFEPTTRPIILSIGFDEEAMGS

KGGAKLADFLYERYGDDGLYGITDEGGLTTKTPEGDYIGLVIPSEKGYVDMFITLDSTES

GHGSIPPVDGGAIVIMSKLGVSLYNDPYEPTITTNNPALEFLQCAAKYSPEYLPGLSKAI

KSKSQSKFLQATSSNLNIAYLFQTTQAMDIFNSGVKANALPEQATMVINHRIGLHSSVNA

TKDHVLEHVLDLAKENDLSVSVVYADGAKEVVKPDGKHGGFTVALYSSLEPTKISPTAGD

ALYEIIAGTTVNTYQQPLFNPHGNASVYVSPFLSTGNMDMKYYGKLSDRQYRYTAALMVP

DFNIHTVDEKTTSKAFLSSIAYLYQLIPNIDAMSSDED

>model.g170.t1 Augustusgene.g170.t1 JFAV02000081.1:73986-75422(+)

MNVNNYITTEKGEDGKDNVNHQGIHQYHQPNMTLSGGSNTTGLVNNYRYNFSYTYENPPF

TANNDLNNIGNNSSSSSNNNNNNNNNNSNSSTTTNTNRPQSMTRLPSIQYLLRAVEQTGI

SDVKTTDIGTNNIGSDRVSSDRVNSYGNSYDTNGNNGVNSYDISSDRINSYDTSNHIVNS

NTLISDMSSRITGFSTANSNERNTYTVNTTYRPNKISSLLHTESDNRGTTIMPITTKENA

GSKIDSILPSRQISLSSTTDASSGSSSSTPGSTSSNASSTSTPTQTNNPGKHMKKRKRRS

NLPKAVVSKLNQWLAQHMNNPYPTASEKLQLMNETGLNSVQLSNWFINVRRRKVFNEYYT

LAAQTPNHPPHKSIDENCVSVRAARKEQKKRENLEKREKLAYVVEKLGDKNGERAENGGN

GVVIKNDAIVHVHDDDDDDNKPEADEKDLQERFVMAPLTRRKKLIDRLEELKNMTNSRE

>model.g161.t1 Augustusgene.g161.t1 JFAV02000081.1:51565-54903(+)

MTSVMNNNPASTDNANTVKSYQDSTTSLKLNETNADETSTALQGETPVGNTPPPPLPHVN

VNQLPLSLLIRNLCIYSIGEIKQFFKLTKPNRRFEFLKLIVYLRNQFLRLYVLVKWCKTI

KNNNFHLLIDMLNYLRQQNMNIGHCIWHLKNSVISSTINAKLPNPDLETALEVFNLGRPN

LKNDIIIRNNNLGGSTKTFSTNTSINTFLNNNMIPTELILKKLRDLNVLLSIRVSMMELP

AKFSFGNHFIQDGRLIIRIPNEYEIHLSTATPQSPFYFVDFKILLMDVQASDNTIYLKKY

INDLLLRERDNTKVLHVLEHFLDKYLLSLKLYLCHKRLGRLSREKYSNNLMYHYDQKKSV

INIKYWVFSRIVNPADCKITIGISKNEETAEVELILRWEIGNLQKLPFTIPTVYKNDILN

NLFDAIDEIIFNHIQIIKHQLVSIDAGIFTDNTFDEDSNENADRVGNFGDTGSAGLNPSS

SALLRDTDNSVEGDLLPSSKNGFVKEETNMSIPTKSAVPTSDSTSLTVYLPSTCTTTTPV

KLKINPITGLLYFSNPNSPLLLSYMERLNKNVMKPADIVKSLYQLKLAKIVQILTNMFSK

CGWTVFNENVMKLPLETLHPHHNTWLKKSNNILIKDMFIRMNTWPANWYLVVSILSSPSS

CFVETKIAKIVSKQATWCLKYLSDNFKNPDDKEFINNANMMKLEHVSYQRVDFLSKTILS

KIINHIVLDSLNELGIEKNIVSESAKPKLPTYIQSLLSQDSNSAVIAVNVNSLMKTNVSI

LDTTLFLLINCGTKNHQNKMSLFGRFTNVIDTQLLRSSDCTKLMIEFIDEQCFVMTTSDE

KNERVVDHLSSLGKQLAIFKKKLSHLIVLSDVVTRLTESFHSPDFQIVSLKPKEIAFNYF

PKSNLSNVSAADCTITLKDIDEKSESVQNGKTQTKLEFALRTTNPQMELNQFLRGSNYGH

EFLFKYFQFTSKFFFTVEQFKRTCTHQCPLFFNIHSMSTYEIAYILFNKETSQPECLKIL

LEIRTKHINRKEELLYHISVGNSKASANTNSAIQKTISKITKSCFEISKPATSKDDAEKL

AKCKSVVKLNSGVCCSDEDIEEVLKEIHNIIVV

>model.g168.t1 Augustusgene.g168.t1 JFAV02000081.1:69828-70421(-)

MFSLNQRFQNVTNTALTYSVVLIALIVALTQLQLSIYNKVHDSISLSNFHINNIQTNMRS

SRMFGGSNKQNMKLKFDLSTDLTPLWTWNTKQVFVYLTGEYQDNTSSKNTNSTGKVTFWD

KIITQKQDALLDMQNIVSKYSVWDKNSQLKDKIVNVKLQWNIQPYVGPLNFGELVVPQEL

LPNTVFTVPDVKRKTAKA

>model.g133.t1 Augustusgene.g133.t1 JFAV02000081.1:268-1458(+)

MSALPTREEPSYFGAGPAVLPTHVLEQAAKDLINYDNLGLGVGEISHRSKEATHIINDTK

ENLIKLMDIPTDTHEVFFMQGGGTTGFSSIASNLFFHSYDPTKKDSEQKKGLYLIDGSWS

LKSFQEAQRLGYPAESIKLDTNNNEKGVSMLSEIQYPENLSYVYICENETVHGIEYPVLP

SNIPEGVELCADLSSDILSRKIDVSKYGVIMAGAQKNIGLAGLTVYIVKKTILNNIAENE

KNLNNESSSKYPKTPIAFDWPTVVKNNSAYNTIPIFTLHIMNLVFKNLLAKGGVAQQELE

NDKKSKLLYQTLDKYPNIFNLPVSKNFRSKMNVVFTLKDSSLNDEFLNNAEKQNLKGLKG

HRSVGGFRASLYNAVSLNAVEKLVKYIEEFASTVESK

>model.g167.t1 Augustusgene.g167.t1 JFAV02000081.1:66733-69738(+)

MYRPAGGLASTHIDRLLRSPFDIPRIIKHQEHPLEGRYYVFPLTQQYRYTSTSVALKSTK

AVKRNPQFPHYVDRIMTLTLTHGPNIHENSRQKLMQYLKSSPSISQISRKSIINVLFQCG

AYREILEIYGMPENYDLAQNTNIPKDITLDELRAIGRSLVLIQDFEGLDTFVRSAITKYS

TNFEAKRKVLIKLLNSVLKQLYDTCELELYFKKWIKYYSFMQGHVYFEKMYIANRLYLKP

FKFVVIEDCAKHNGDKLRYQSILQHLSSSTEISKHTLAQFLSTFLSVLINSQEWDIAKMV

FEYKMEHFEQSLTEFDLNCSLQVYAHFDQFKKLINVYRKYPQLHSMSAAQFDYLLVTLSK

LEDWQGLQKQFDELFYIGELPNIIHYGIIMSKVAKTGHIEIVDKLFTQLIGRGLIPNFHI

LQSLVVASYNSGDHYGLLKNFQLFEKYEVKPSPTTYLYMLRSYRRRNDMEGALKVLRNMD

SEMLNESHFLQMILIGTHLMNSKIAEEIFHVMQKDFKIKPSPVFLSKMMQLYNGCRLPEK

AIALFNENIASVKRNPKRISLYNQLLNSHIFLKNEEECENVLKDIENEGLQHDPEFNLMM

IRFFLSIKDNVSIAVDFAQKMYEKNSRMIGTVHLQEIMSYINCRMSSKNMDQSVNQIFQL

YKYQLDKRIPVSSEILRYVLKFAYIHNISKFKSGSQLDQFLKLARSFIDKFADKSLDTVN

PILHPNFIYFPVKSMSSIDPSKARELIEYYVDKCYSKQVDQDDSYKEIVLLKVKLFFSFN

TGEWNNVSYFFDKILENLLKLRKLPFYERSVNKNLRFYLIDIVDIKLRQLQAQNQISDGV

DLFKFIKKNLKMIINNKSHNLLAKLLVQNRTTIDLGLKMVENYLIHGHRVIHKLRSLKKL

KTAMESKTLMVLEEDPKKLQPKNYLQSEDYFEIMKSMDNYLIELNDNAAMEQTITKWCQN

YPQTMVRYLMVSRPFPNWAQYEKSHKEFLHKVRTEKYIEKLD

>model.g165.t1 Augustusgene.g165.t1 JFAV02000081.1:63109-65421(+)

MSAAVMKQFGPLATMRSALGRSVQSSFIKPTHRLFSLTISNRMEYEEEKEIVDKIRATQL

KPEDLARCAKLRNIGISAHIDSGKTTFTERVLYYTGRIKAIHEVRGRDNVGAKMDSMDLE

REKGITIQSAATYCSWDKNNEKYHFNLIDTPGHIDFTIEVERALRVLDGAVLVVCAVSGV

QSQTVTVDRQMRRYNVPRITFINKMDRMGANPFKAIDQIEAKLKIPCAAVQVPIGLESNL

KGLVDIINRKCVYNEGVKGEVLRIDENIPEDLKELVEERRSLLIEKLADVDEEMAELYLE

EKEPTVEQINRAIRNATIARKFSPVLMGSALANTGIQSVLDAVVDYLPDPSEILNKGLDL

ANEEKEVTLVPNDKLPFVGLAFKLEEGKFGQLTYIRVYQGKLKKGGFITNVKTGKKIKVS

RLVRMHSNDMEDVAEVGSGEICATFGIECASGDTFTDGNVSYSMSSMFVPDAVISLSIAP

KSKDGATNFSKALQRFTKEDPTFRVKYDSDSKETIISGMGELHLEIYVERMKREYGVDCV

TGRPEVSYRESIQIPSEFDHTHKKQSGGAGQFAKVVGNLTPVEVKEGEPIASENSFKSKV

VGGRIPEKYLAACEKGFIEACEKGPLVGAKVIGVDMTMIDGAIHVVDSNEFSFKFATMTA

FKEAFLKAQPVLLEPIMKVDVTAPIEFQGSCISLLNKVNAVIIDTENGQDEFTITAECSL

NMLFGFATTLRASTQGKGEFSLEFKHYAPTLPNVQKEIVDNYAKEQSKKNK

>model.g171.t1 Augustusgene.g171.t1 JFAV02000081.1:75736-76263(-)

MSLREFEQLNFSHSARRLDRPSLYLIKKARHHPEGEGYKELQKCLESKTIYIGNLSHYTT

EEQIFEIFHKLGPIERIIMGLDRFKYTPCGFCFVIFEDKASSINAVKYLNKTKIDGESIS

IDLDPGFEDGRQFGRGKFGGQKSTDDYQFMYQNMMPSTYTPGGTNAGGEDTYIPQV

>model.g136.t1 Augustusgene.g136.t1 JFAV02000081.1:5108-5872(-)

MKLPKVVVFGGHGFLGSQIVKQFPKDKYQLISLSRSLPSSKIPHVQYLPNIDVLKPETYD

HIIKDADHVVHSIGVIFPNEAYKKIINSKSICDTFTGVCNYLTSNSSATSAKNKEMYDRI

NYQSVRKLSETFVSHLRKRAPEANTVKPSFTYISAEETPMLKTFISPEYISSKRKSELQI

LQYENDLRPIIVRPGLLYDEHAKELTPRSSFAFLMKNVNPFANQFMCSTQSVAKAIVNKV

SSPDYKGGITTLKEL

>model.g148.t1 Augustusgene.g148.t1 JFAV02000081.1:28606-29106(-)

MPESVKVSIEFCNKCKWGLRSFWYLQELFQTFEDKIAELSVKPNNSNPGTFKVVGYIVSD

DSVENNIQEVTIWDRKVDGGFPDAKTLKKRIKTLLFHENNDVKIGKHNERGSNAVGEHTT

EAVQRDGLLVSDTIVTSTSQLSKSPSEQQPLETKEYVECAECQLAPK

>model.g160.t1 Augustusgene.g160.t1 JFAV02000081.1:47709-50624(-)

MGIENTSIETSSKVGPENELESSSLTYKGAEEDHKNEENSDKGNLFSSSQVIQPDTVADE

KSLNNKSSDSNEHWTSEGFGSPSRQSISVQTTPTDLHVVVPQNGFTTSSLSPVKADTDLS

ITPSAATPTTTTTTKKTTTTKGLLDTQSAGGLINSMWASLSFATGGSGTPDNSKTDLDSF

DQKKSNRVSSISHRKNDSISSYTTIFSGNAPESPIEDRTIKTKEKQAEESVDEPVAYYSE

TNTLKIPFASSKKNTDFHTTFKSVPADDRLLMDVFTCSLAKDTSNNTSNNNTIKNDGSWL

QTKDKTARDRSSSVSQKQTNFAANSSFMKQNSRRDSYTSSSPTTSSYTGFSSSMSALCNG

EFFLSENYISFRSNKNILSLWNTNIIIYIHDIELIDNMGKILKNKFRVTSTVLTSSEENS

DESNLEPTDAAKSASDSTAVIVKTVYGKTHTFFNFNKPEMISDAINTMWEKQINFKNPLL

LTSKEKESDKLLESNKPNNSSGLLVDTFPIDNYYLSNTRSNSNFSPSLANVQPFQKNVFD

LEDEILSVDESVDESYDDYHKRVSTDKTSSNGYTTKEKKVEEALETDSDGEEEDDEEEEE

EEEEEEEEEEDDDEEEEGEKTDGKELELDGSDHVEVFALKDNDTYNYVGPLFHEETFFVL

SPEQEQTEYLLAEVELDAPPGIVFEILFSSTNPKFSLDFLTNQNSSNFDPVHLGNFNEKI

NQHGQKYREYQYDKGLNYPIGPKSTRCYVRETILSLNYEDYINVLNVTSTPDVPSGSSFN

VQTRYMFRWSTGGKSVLKILYWIEWTGGSWIKSMIEKSCKSGQVEATKAFVKLLEETIAE

HVTKKNLSLAEATKASSKESNLGDISPLKQKKKKKGTALSDSSTSQSGGIGGLAAATNIA

KPAELSGKGQHSLESALSNEQTANINTMENPASVIAGSVDGAQIMKLIISSSRVLTLLLL

AVLILLVVNLYF

>model.g147.t1 Augustusgene.g147.t1 JFAV02000081.1:26337-27725(-)

MYTRPSSLFALILLLGVTQFLAVEAHSAAGKGSSSGQSDADLLNEVMSLPDLIKAKKNPP

SYDFFQSATLEEGRIVLTPEPSTSGAIWSKSSMAMPNAFTIEWVFRSVDFQGKTDGGLAL

WVTDSQINDPYLTSETVADFKFNGLMLYIDNNGPSGSSQIRGIINDDNAKQFTTMQEFYD

NEFGSCLLGGYQDSSVPMTARLTYDNADDTNFIKVQIDNRVCFQTRQISLAKGLNKKSIK

VGASAQNGQNNEESFEILKLHYYEGVVQDSLIPNIRQMQQPKYVTKVINQDTGDVTFVER

KPGILGDSELTLSAIYEKLNKIEGKVLANDVGVIYQEIENLVKINKQQSKRIEALINAIS

SSTLTSTSNSDDKTSATQGGSGLDMDNFKDFIKMDEKLEKLLAEQQKLREMTKLEKQNKL

ESHHFDDLFYKLLLWISPLVIIMMVMAYYTFRIKQEIVKTKLL

>model.g158.t1 Augustusgene.g158.t1 JFAV02000081.1:44739-45659(-)

MRLKITIGNGSKVVSIPNEKAPSVTLHDLVNEYINFDQDFKEQNVTSQKQLEKIRFGYPI

RTVDINDDPESYLSTLDSLGIGNGERIIITTKLDANPLVTQGTSSDNTSRKKKKSDIEYS

GLTIEDIPDDNSCLFHSISYCLFKPTNYNERMEYSNDLRKICAEYILSHQSQYNTVVLEK

SPVEYSRWIMKKDVWGGAIEISILSKFYKIAIYVLDISFDSFEKFNEDQYEDFIILFYNG

SHYDVCQKKIDKEVKKDELPTGKDPIDAFQTIFNKSNDYETQVLLTQCLELSKKLKQAGF

RYEGQKR

>model.g145.t1 Augustusgene.g145.t1 JFAV02000081.1:20300-23734(+)

MGFTNKNKTLPNPSKTESNDEEFEFYHVFEPANVKAVVHSITNNLKRDKNINIEYLFLPF

RKEQTNENLLRFLNAIFPLGNGKPINNVAKIDKIIEKTKGDTLFQALKYIWCRLPGASGI

VGWRAYNLFKEKEAELGFPQRAFLEIMPKNLESPDHASLVYDFFDLIVTMSSNANKNKLS

ARKISRMCALWAFPCENSSIAGSSSSSSSPSSISFQNGNDLDFSAGFPNDALPNNSFQEG

LKEWIPASNAMFHLTMAFIKSFLPEDGDISESFPKTLQHVLQDNEYPPAENSWITSSSTI

LQVPLVSLQTTEFSRKPWDLIKRLNDLLCSHDPEVEAGFDNFPKQDYMVLKNLFKSSSSS

SSKVVENISKKMSKQSKKLMKEMTTKHSTFQAGWASNRKCLPISTMNPSKDYLKTGICEI

DDYFIWTWMSSISNEQTSMKRKIFGRSLILEFEFDGFKKWVIFEECDSTIELLKNKNVSN

GQSANSKRNILSKNTNVASSVVQKIKKTEDRQITPVYEKFQKLNIQKNPSVVDASKNSTR

DFSTSTIASAPANSNVPETKKTVSARFETKNENSRNAVPRQEKTPASIPSTKNATQKAAP

QYNSSSTSNIYSSRTQPQSVSAKETYKLPEVNVDESNFKIDLPDIDASHITIEDDSSDTQ

GSSFYSENNNNGAKQNQYTSRDTRQQTRGPQVKMRNITDDSLNHAVQDLTEELQTVENSL

QQIDPPQQNHYMGNSIGSAVQPLQIDNIPNARNYNREHKEQLYQRVAPNNNKPVLAPPRS

EQPNEVYPQDYYQNQILPPQPYKTAQRSPVFANQPQFPPSANQHSPAYPKQHPQGHFSSH

SPAPNGYGPNSAQPQPRSSGSPTIPVSSSSQPPRHIPSKSMQMYQPKPPSKPPMQLNQYP

IEVAPVQPPAVFPPPPQPQPSPPNIYPQQHGKNVTPPPLQQQQPRSNKGVPFGGMPRTNS

MQNLTNVHHPRFSKHPVQPSTSDGSVPSQYLQQQQQQKPQQRSQQPHIYPPRMNTNMSSA

FPMPQQRHSMVGHSASPMLGRQPQPGMQPVPQQYNSPVPPAQQPGYLPPPSAYQQQAQQQ

QIYYPPQQQMPQQPMPQQTYGQQGFMPQSGLVPQMPHNKLHSANLNKTAGRKNLHAQIKN

GNFGI

>model.g142.t1 Augustusgene.g142.t1 JFAV02000081.1:14040-15341(+)

MSAIVSQFRLASSRLALRTAPLSPAFASRISIRNYAAAFDRSKPHVNIGTIGHVDHGKTT

LTAAITKTLAKTGGADFLDYASIDRAPEERARGITISTAHVEYQTEKRHYSHVDCPGHAD

YIKNMITGAAQMDGAIIVVAASDGQMPQTREHLLLARQVGVQNLVVFVNKVDAIDDPEML

ELVEMEMRELLSQYGFDGDNTPVIFGSALCTMEDRQPEIGEERIKALLQAVDEHIPTPER

DLNKPFLMPIEDTFTISGRGTVVTGRVERGEVKKGEEVEIVGYNKEALKTTITGIEMFRK

ELDKAMAGDNAGVLLRGIRRDQLKRGMVLAKPGSVKAHTKVLASLYILTKEEGGRHSGFS

ENYRPQMFLRTCDVTATFKFPPEVEDHSQMVMPGDNVEMVVELYHPTPLEVGGRFNVREG

GKTVGTGLITRILE

>model.g159.t1 Augustusgene.g159.t1 JFAV02000081.1:46484-47344(+)

MVKSSKQLQEEARNYKFNKSISLQVYMKFCISLIEKAQELATNADDTENAYVFYMRYLDL

IMNKLCNHPDILLAKTHSMQREGSSVNSLHVKTYHQILRLEVPAILKIVEEMQKKIETKY

QKQMAKQYIEPSKIHVRTSLHENSEENHCIGRNSPELNIEIALPSTFNEHAFNNSIKYMK

ESNMPVNSSNYKSITSLESHNDNKSELPSNFKLNNLSKFNPDKRTQKNDGNLRLNYNLPS

LTTAAPAPEQKTTFYKPQFDIGRSFPAPKPSTKQLDFDYPELPNICI

>model.g156.t1 Augustusgene.g156.t1 JFAV02000081.1:42690-43352(-)

MARRPARCYRYQKNKPYPKSRYNRAVPDPKIRIFDLGKKKATVDEFPLCVHLVSNELEQL

SSEALEAARICANKYITKTVGRDAFHLRVRVHPFHVLRINKMLSCAGADRLQQGMRGAWG

KPHGLAARVDIGQIIFSCRAKDSNKDIVVEALRRARYKFPGQQKIILSKKWGFTNLDREE

YVKRRDAGEVKDDGAFVKFLSRKGSLENNYREFPEYFQNLA

>model.g137.t1 Augustusgene.g137.t1 JFAV02000081.1:6659-8098(-)

MSSNVEPTTEPSEIDVLINPFIEKLKTRKIQGSYEISMQTLQLLKRFIGAVKFSSHDELF

AKLSNLGYKLEKAQPREFGSGNMIRRLLYVLKEELLTANIQHGFSNNPGAIGQTGTLMQH

QNNTGMYDSNKENKNEPMISSMFSLLQTPRPSELTSFKNGTLRASTPVTTAANANHTAFA

IGTSATVLVDEEATSNNSSTQNATALAGAATSGSGEDPAGQHVDSKSKKTSRFFDIRATL

INAIKDLMDEIKNIDEGIFSIAIDLIHDREILLTPTPDSKTVLKFLLKAKEEGYGKVRNF

TVLVTEGFPNNVQNAQDFAWKLSSHGIDTVIIPDSMVFSVMSRVGKVILGCKQVFSNGAC

IVSNTGVTSCCECAKEFKTPVFAVAGLYKLSPLSPFNITDYIEVGGSNTGKLLPSGNSNS

TDKTDYTRLTMINPMDDYVQPENIDIVITNIGGFAPSFMYRVVLDNYKVEDVNFKPTLSH

>model.g144.t1 Augustusgene.g144.t1 JFAV02000081.1:17659-19431(+)

MENVKAITKDHNIDINPLYRAPTMHPNSSEVLRTGEHGALSSDLEMCNELTYNKIILKYG

MESVNSADLAVPLGLCIGMVNFFNSGIGGGGYAVYTDSTRNNENNKWFDFRETCPLAWDN

DVHNLTKIGGMSIGVPGEIMGLYELHHQKGMGKVTWSQLLEPIIELGQTGWSVGPVLGAA

LSIYKDYFTENYDSWSFVFNKDKSLKKSGDWIQRPTLSAMFQELANNGSVAPFYDPEHWI

VKSMVKSVQKAGGILTSQDFANYKVMQGKPLTTTITTDNLFQYDVQTVGGSSSGAALIAG

INIMKSFPYREGGDYMQESVYELVETMKWMASSRSRLGDYFLEEGATDTNNYPQRILDVL

SDSWRINATHKMRENYLHTFDDFHEYDPKYEMNDPHGTAHFSIVDSHNGAVSLTTTINLL

FGSLVHDPVTGVIFNNEMDDFSDPHGKSNAFDLKPSIYNLPEPGKRPLSSTVPTVVIDEL

GRPDLVVGASGGSRIMTSVFQTIVRNYWYGMPLLESIAYPRVHHQLLPNQLEVESKSMIG

SQTIKQLQEIGYTDIVETNPKSVVNAIKRDRFSTLHAVSDYWRKRGISVAF

>model.g139.t1 Augustusgene.g139.t1 JFAV02000081.1:9792-10439(-)

MAEQKETPTFKLVLVGDGGTGKTTFVKRHLTGEFEKKYIATIGVEVHPLSFHTNFGEIKF

DCWDTAGQEKFGGLRDGYYINAQCAIIMFDVTSRITYKNVPNWHRDLVRVCENIPIVLCG

NKVDVKERKVKAKTITFHRKKNLQYYDISAKSNYNFEKPFLWLARKLAGTPQLEFVSSPA

LAPPEVQVDEALMQQYQQEMEQATALPLPDEDDADL

>model.g151.t1 Augustusgene.g151.t1 JFAV02000081.1:34378-35154(+)

MNALYSHATKQKQLLTKDLSKFEENIISAPISLQGSITTTLLSLEKTIVKYKQQLENFKQ

TSNVGGQDEEEAAELLKYETRLNILESEYANFKDQFTLLKERYQEEQTKLLQTPNSGING

MQGKSSSSTNPFTDTSNATMTNRRAQGHLKDFGDGSGTGQLNVDYQDSLRNEESILAKGN

QKLDYILEMGQQSLDDIMEQNQVLEKMQKTMTKSLSTLGVSNETIEKINKRVFKDKLVFY

IALALFFVGVYLVLKFLRR

>model.g154.t1 Augustusgene.g154.t1 JFAV02000081.1:40044-40724(+)

MISIFQNTIKIRCGSVPAPSHFIKPTVPHNFIRKNYFHQTHKLLDYEPSHIDTYLLRRKL

VAQGGFTNEQSDVIIKMITQSLNDGVKHITNQLTKREDLIKLAYRQKVDFTKLKDELLLL

DRNEFNKIDAEQEKLRHDLERLSRRLREEITKCNAGFKLDISLEKGRIKEEGSLHDLQIK

EIDSRIDKEVNNMKIQIDSVKTQVLQWMIGVSSGAFALVLAYIRLLS

>model.g169.t1 Augustusgene.g169.t1 JFAV02000081.1:70730-71722(+)

MSKKNDLNHSALNDNPFADSNDLNLDDDDFLSSAAPAYSTVDRDAAPASQTTPLTFTTTA

TPSTATTAPNTSKSSTGGFLNTYYFSYPTLSIKDLYISLISALSTSTSTNIVSSTTSIFY

PHWIIFAISLSTFISTTILKQLFEGKHNDNTKNITSNPTVQHFMTVYITLMCYTVFYSGF

HCFRINKLIKKTHNWLDISSMMTMMQNVSVSLLCVTVFIVLQFIVELVLVIVMPNNQWTA

FKRVNGLCKWGFACVDYLIRSRYLSVNGRVTEPSTTTTTTTTTTTTTTTSSPDLEAQETY

GSTEQEKSQLSWTEVFGVQLVSLFIVRFLVL

>model.g149.t1 Augustusgene.g149.t1 JFAV02000081.1:29459-31951(+)

MPYFTDSFWSPDFKEAIQTLFEGLFQNCQQNELFITFFANKMELEVMSAKKMISSSNDLV

KKCESLVPNGKSGSRIKHDNRNDDLNANSGYQTLIVLNQDLRIQGSQLLEIAELIETSVL

QPFTKWCKEHKQRLEYSEKLLNDNITNFHKFKGNLTNKLEQQYINKCKLLQDFKTELPQH

MIEEMDQILTQWKIWNKFLDKEKEYTHYTTLCNGSIDVDIRTMKMILRSLLLDLKKEDYK

TSVFGYLITNTNNGYDIAQWLTQIMSLNNVKQAELIGQELINLNFLKDCNHNLSLKKAQF

SSSKDLQYQWKPTAFDFIDIDPETLKGKIDEYNVPDFVTNTSKLSINEMESKKEITKNSV

SETQDKAENKISNNDGLVVQTNESIGSTENNGKENVDGKQNAETQHDFDLDLDDDDFERV

SIEVKSKNEKDQEKQESADLGTSKNAGTTTAKSDTIENDESGSTFVDEDLQPPPQQPKRS

LTELEIRYERILNDVKLANWKYLEGVEKADKLRCSLEELILDHLVFMEKCERDRLYALKR

ATDDLIHCYESKGQLMTLPTTNFKIEPELDLKNFVLKYRVGVYQPMPIIYRNYFGGGDPM

MDKQVFGIDLTVRCRLDHSKVPKIISKILQHFDDNVYPELPNDEVRCSIWTKSVKLSHSH

ELRLKLNDSFKLAKFQLNDIEPSIVASVLKIYLLELPIPLIPQELNDILKKIYHITDNNE

DEDSVVKSNEKFHAIANVLSRIPKLHLATLDAISTHFVRLLSIIETSEENKDLSKKFIEL

ISQEFANCIIRLTSFNDNVLGYKIFRDLLANKNQIFDLLKKNIKQINELNN

>model.g141.t1 Augustusgene.g141.t1 JFAV02000081.1:12232-13641(-)

MARFYCEYCRSYLTHDKRSVLKAHITGKHHIRLVKDYYRNKYVEQWNRARRLNKRKVIRD

HTNRNLAFAKSKNNPNGNVNKLSQSSSKRLQLQDIAPEYKFETMSKDDLQLSKLSKTRKF

KKIRKRQQLRTLLRNVDPFSHESILKERKLHSFGELSNVNGMENGLSVLNHLYKYSPGYN

KVFISTNRFDNPEYSGLKPVSNPSAQTSNSHSRGKGIKNFKGKSQFRSSKDNKPHNDVSM

KQLQRTSRNTIFHDDVNIFGYMKNSNKDRKKYNMGFLKPPKVVVNKRLKLNTDSNSVNKA

ITATAPGREFLFKQNNFFYKVFLRSMLPKRKFLQIFHGKRSNMGKKYRALELRKSRFQNK

KDTLVDKHKRVGTSRFSKSRPTSSHPHSTASGVPAPAKPQSRFVPQYAGASRFASHNTQI

TPTNTSVNTGSRFNQSSNQQQQQQQPRSRFTRGMHRNGAGNISSKSYRRY

>model.g146.t1 Augustusgene.g146.t1 JFAV02000081.1:24090-26210(+)

MFSYSPAPKDEQNLFLLKKALSYVSISESIQESNVKHTHAKTENVANTLSSFPEKAPKTP

NPFAFFIDIPENEFLLANSEPGQVQDAQPLALIGDYRIALTDKLGEHKIDAVGNLQGERK

YCMDSFTIPTVVTPKQPTRFLLLQRLLDILGDISKADFEATYPEIKIFQASNIKFKNQDK

MEKSLGEYFADWFPEISKDSYFFVHARVAFLRFNALLITGGNRILDDYWEQVFINQGFTF

RDRVFLVPNKIVNKAKTILGNTSSVLPCFKRNGKNKTQDLQRARLDVVRERDDPFNYISE

QPSMQLKKAYCNYQQQDLYFKNSTDLGVEDDDEEGFRNTENGAREKDENDADDEVSLDEI

PAYSFRRGAGRPALASDASQLSTVAGLQHVIVPGQNITGGIELSSQYKVPRYATKVAYMQ

WQNSLNVPIGEVSNDRSESAVSAELNEHEDGMLHGANLESGSASEFPNSSATLVPTHSDS

APHLQAHSGISSASSGKNKRLLSGLLGDDTFQQKKKAAYYVRRYFESQNGDGIVDKNVQL

NIDPWKFETLPLRKSYGSKKRGLKYNSKGLPIFEDEEQLKNRLKKLTPQQVMELQHQHDA

VQLNKGISKLVAQREEKWGKYWLYKAGLPLYKDGSAFSGDYPQNEDLTAHIIKHDIENYE

KVVLEKYEPDTNTDVITLEKRELGINFKNCSSIEEFRPPYSIFPDES

>model.g140.t1 Augustusgene.g140.t1 JFAV02000081.1:11349-11699(-)

MFRRQFLQNSKRFLSITLPKNNAVSELYLKELKSVKLPEVNLADANVKPFHALKVSPYAS

DILQANESEKDALKTYESEPVEVLKAVQQQTDAAEQTGAEEEDWLVIEDDVEEETHH

>model.g155.t1 Augustusgene.g155.t1 JFAV02000081.1:40896-41657(-)

MPIQEFAYKENPETLVLFDVDGTLTPARLTISDEVKETLQKLRQKVCIGFVGGSDLSKQV

EQLGPTVLNDFDYCFSENGLTAYRLGKEMASQSFIGWIGEEKYNKLVKFALRYLSDLDLP

VRRGTFIEFRNGMINISPVGRNASTQERNDYEKYDKEHKIREKFVAALQKEFPDYGLTYS

IGGQISFDVFPTGWDKTYCLQHVKNDGFKEIHFFGDKTFKGGNDYEIYEDPRTIGHAVNS

PADTVRILKEIFHL

>model.g135.t1 Augustusgene.g135.t1 JFAV02000081.1:2970-5009(+)

MQQRYGIHMLRSVTFCSVLARTFPAKTSILWHYKAFSSTSNLSNESTAHDTTLQEKILGS

KELQDRINAIPLENYRNFSIVAHIDHGKSTLSDRILEYTNTISAATDAESQKKRAQILDK

LEVERERGITIKAQTCTMIYPYKGQDYLLHLIDTPGHVDFRMEVCRSYASCTGALLLVDA

TQGVQAQTVANFYLAYSMDLKLIPVINKIDLTQTANIPRSKQQVQEMFELDPEGCVMVSA

KTGLNVKEDLLPAIVERIPPPDLAKVDPKAPFRCLLVDSWYDTYLGVVLLVYVVDGFLKK

NSKVVSAHTNIKYDVKQLGVMYPDMTPQDSLKCGQVGYIVPGMKNSKDAHIGDTFYQVGK

ETEPLPGFEEMKPMVLIGAFPAEGVDFKKLEDDLYRLTLNDRSVNIQRETSNALGQGWRL

GFLGSLHASVFKERLEKEYGSSIIITQPTVPYKLVYKDGTEKMITNPDDFPDLATKKSRV

EKLMEPYVECLMTAPQEYLGDLIELCENNRGKQLDLQYIDSTNQILLKYEIPLAHLIDDF

FGKLKSCSKGYCSLDYEDKGYQQSDLVKLELLVNGNSVDALSSVVHRSQVQSMGKRWVEK

FKEFLKIQQFEVIIQSAADNKIIARETIKARRKDVLAKLHASDITRRKKLLVKQKEGKQK

LKSIGKVAIPQDAYAAFLRK

>model.g162.t1 Augustusgene.g162.t1 JFAV02000081.1:56551-58581(+)

MFGVPALHVGENNVLKNMTNFFALLLCLSSMIHSATADDVETTNYKPKTHTFRFTAGWVK

ANPDYKHEKRMIGFNGQWPCPDIHVNKGDRVELYLTNGFPEEENINTSLHFHGLFHNVSL

GNSNQMDGPEMVTQCPIPSGETYLYNFTTDDQVGTYWYHSHSGAQYLDGLRGAFVIHDPK

MESKEWRVDEEYVIQVNEQYYKPYYEIVDHFLSRYNPTGAEPVPQNFLFNNTLNGTLNFD

YEKRYLLRFINSGVFVSQFIYSKEHVFTMVEVDGVYIKPENTHLIEVAPGQRVSVLVDSL

SKDQVDLKYYPIFQIADKTMLDTIPDDLEIIKRNCILYPDQKWPSKFPKPQENELPQLLM

DDFKLTTLEKQEILPSSDYKIELNVVMDNLGDGVNYAFFNNITYTAPKVPTLFTAITAPE

ELLFEPSIYGDNINAFVLQKNEVIEVVINNEDDNRHNFHLHGHNFQIVQKSKGFEDPTPF

NESAPLMDYPEHPMVRDTVFVEANGHTVLRFKADNPGIWFFHCHIDFHLEQGLSAVFIEA

PDEIQKYEGSVNSDMKRICKAANIPTKGNAAGNFNDWFDLKGLGKQAAPLPAGFTLKGYF

ALAVSIMTAIYGMYTITQYGLDDHIMTIEKEQEMLDTLTALVGATDSSSITSSGLHSNNS

GFTEQTTPLISDDRSAL

>model.g150.t1 Augustusgene.g150.t1 JFAV02000081.1:32064-33221(-)

MQPPSTPTRPGRTSKTGATTMAARNSSNNNPLQQTPSRTQNSNYYTESGDPDHSLNSMLV

TPQTVSKFKPTSSVLQNFSNTRTPEQKKSNPYENSKGSVFSPEYESLNSSGSIFMDSSIH

TRGFNRGPLEGQKVGLSYGQQQQQQQQQQQGITKRQHIGTVTPGHEEDARDILMSKLLLR

SPEYTPQTKPLRGLSLQQSQRGAESLIKSPVQGIKRKLAFENIPNMHENDLLLEKLSRET

SDIFESDEDSGEPAKRDNSKFFASNDQKQTSRELFKPMAVPEPEPLDFETPSTPSTKVIS

FEQAERWHNMSRLNSKGEPRRASNDKEDQEVFITKKQLKNPFLSDKKKSAHDAFLHSDEL

EDVYSDSDSDSEVETEKLRTFIYKKK

>model.g164.t1 Augustusgene.g164.t1 JFAV02000081.1:61111-62826(+)

MSGYIPIDANEALKRVKAKKKQVTISGLVLAFFIVFLILRKSSSSDFEIPECRDCGSYEA

LKPAFVGQINEILYDEDYKLQVIGKLSRAVQHPTQIFDDTPPPTKHTLLSDDWKNFTSFH

TYLEETFPNVFSTLKVEKVNFFGLLLTWEPETATGNKPVVLMAHQDVVPVEPNTINKWTH

PPFEGFYDSKEDLIYGRGACDCKQLLISQLEAVEKLIKDGYTPKNRPLIFSMGFDEEASG

TYGADQLSKFLENRYGKNGIYAIVDEGGSVETENGDYFAGPVGTEKGYLDAWITLNTPGG

HSSVPKAHTSIGLISLLNVLLENDPYEQAITPNNPVLEQLKCYAKYSDMNPDLKKAISRN

DIETIGKLINEDLSLKYYFQTSQAIDIIKGGVKSNALPEQVTVLVNNRIGIHSSVQDVVD

HILKHLQTVAEEYDLTITLEKDSGKQVTIREEGANGDFVFRMFSSLEPAPISPTGNDTFW

EILAGTTVSVYSHDLFNANGTSNVYVAPSLSTGNTDTRFYWRLTDRIYRFTGALEDIETN

EHTVDEKITGKSLISNVAYMYEFIPNIDMYTV

>model.g153.t1 Augustusgene.g153.t1 JFAV02000081.1:37268-38386(-)

MLYEKEIRIAVNAVRRASILTAKIQKQIIANRSSTTITKSDESPVTIADYAAQAIVLNAI

KANFPQDEIVAEESTEGLSESFLNNILSTINEYAPKEDDETLFNEDIALSNSEYPLKTTN

DVQKIINFGDSKGGAKTRFWCLDPIDGTKGFLRGEQFAVCLALIEDGEVKLGCIGCPNLK

LNDYVTKENSINVANPLNRSFRDPEQFGYIFYAVAGEDHLSYLHTTDVLSGSLKSVAANE

FTVSESKNLVSLEGVEKGHSSHDNQQIIKDHFHIDKSLHLDSQVKYCLLALGLGHVYLRL

PTSMTFEEKIWDHAAGNALIRAVRGNHTSSLSAEPLDFGQGRTLKTKGVIASIATPELHK

EIVSFAENVINKN

>model.g152.t1 Augustusgene.g152.t1 JFAV02000081.1:35215-36990(-)

MLRSYKTFGVLKTGLSVRSTIKARVINGSQLRFNSGNSLKDKIEKEHSVDASELFSDRIT

STPYYAKREHPQNEHVKQILKERQQAWDNHELAQTEGIKILSEQEQRDRMEKMTRLNSLF

QGALVLAVALGLAYYFTSDNMSFLNKHSSLIDEEKIEQKLNINNAKKDKKLKKISAQLTA

EAAKGQQLISNKSNHDLVQWKDGKANTLTAFSVGNGKKDLIRDVVECGEALLYVNKRGDL

YKLSTNSEATEDLKKDTLILKDQQITKLKSTSDNQIIAQTSKNEILVIPLTNSKYKEYVS

SKRSKLLPWKHYDGYSKKLPFSFESYDLGESHFIGIGAKDKLVYTCSLVSPENVKHKGQF

GLPKYAPIKICDNEELARELKPFDVENVELLNYLIKNDCLQERQFAKVACGKYHTVAMDT

KGDIYTFGNNTFGQLGVNVSYDTENIPYPKQLRKLPQLSRDKSYDWECQDIKCGDYTTYL

KVKDENDSKYTYFSFGSGMKGELGNSHFKSSQQAPTKMKYDPQDCIKEWEFQGSQCALVN

SENELYVWGSNNENSLPGILPNKNYAYPIDTGFKLEPNARVLVTKHNGTIIY

>model.g157.t1 Augustusgene.g157.t1 JFAV02000081.1:44110-44634(+)

MGRYSVKRYKTKRMTKSLDLIFDELTDPSKVSKLLNQPLDETKPGLGQHYCIHCAKYCET

AEALKTHLKSKVHKRRVKDLKGVPYTQETSNAASGLDVQKFLNRVQDIQTVKGPNKEAIE

KELQTHLDATLENSKAIDQMSYIDKVTNQENGISANVPVQNTAGPVEQQQQQQQL

>model.g134.t1 Augustusgene.g134.t1 JFAV02000081.1:1540-2688(-)

MRLQFNINSQDTPKQFSCFLKGLVLANSIRKSAIFKFKEEELLIILTTGSGFSSSSSTSI

SGNTGSSSQSDSQLWLKISVEPLLQSYVIKTKRVDRSVSMELALQPLIQILRQYDQMMKR

GDQAHTNAATMGNLEVRMTNVPKEWNLLMLDGSSGQGHQNTKGQNMKNGGYGALRVSFEE

QVPGKPTPLKHVTYIPLKLLSSQYELRQPVINADQVVYNLPQQNDMEEGKQFGRFMKRLD

RFSGLEHLKVCGTTQELKLVVDEMDWFMEVKWNGVIDVLNRDTLTQHEEEEEEEQGHEVR

LTTEEQSVIIRAKDWKMCTRLFDVLGEQTFLCISNKRYCLFNCMIKNTDSVDADSLTQSH

DSLAEHGNRFMDVSFYIANCRSF

>model.g138.t1 Augustusgene.g138.t1 JFAV02000081.1:8609-9343(-)

MSLVYDTNTKLISSNVYTTTSIDQINTLTRNLVENGQQSLLKITPEPNAKSTGLIKSMFE

KSVTSLTKNKNIEESYKYAKLAVEMAVKSRSLIESFQVQMQELQFILKHKVDVILALYQK

NYSKTSELLVNGKKLTKIEFIRTPESQDLLIEALQDIEMLLNTGLIADPNLSLRKCDVLM

KLTKFRDAKMEAERGLSLFTVKVQNKEFVQSIPQEQRPAFMQLSMKLKSLFAVAERYVKE

ENGDF

>model.g166.t1 Augustusgene.g166.t1 JFAV02000081.1:65613-66131(-)

MAPETVPSNKDKKFSATKHKKIQQSKKFTKEYKIAEIKKSLHKKARLKKNYFKALKDEGY

SLPDERENQSSGRKSRVDPSLSYKERKDIQKRENIEKLKALKQQKKDTKYQQKLTKEERR

QKELDRVNEIKKKNENRQLKRERLTQTTRRGQPLMGPKIEDLLNKIKTDDIYK

>model.g175.t1 Augustusgene.g175.t1 JFAV02000082.1:5477-8101(-)

MVYEEYALSKINNLLKDENALKNIKEIKEQLIKEKSTLDFKLKSVSDKNRDSVNSGLQEL

DESQKYVKRLKKQITEMTKLSQESTKSIERYDVIKRITKLQTMMEQTSSIYEKIVQFEDL

VNNVIVMLDKEFENNQNDTLATGVPQLLEIHHQLTVARDFSEQCDILGQISSDDVQNHLP

RIFRNLENATTKFNQLFQLIINDLLELLATKNYSLIVRFFKILEFEEKEDLRIQAIRTIV

CLRERELEASKIKKISDSDSDMGIPVSTPANNAYKRTSKIKDRLLKMKNDEDQQDFGKQP

KTATSNAAVHPATEEDDYNTPSDKALAEEILNGTITSRVYPRNYKKMFLNYLSEEIDLVF

KRCWDHFVQEEQNMFEILNGLDWINGELFAIKRFFPLYCPASLPIFHIYFQIYFKNLNKI

FTDLVNSEPETSMILNILDFDKEFFRFLNKELGCSKQEIDGATSLLGDAAKQQLLDDYLN

LILQKMQEWINNLEQSEFEIFEQRKQPPQSDSENLLYLDSTKTCFQMFTQQVEVAADSGQ

VKILLGVVESFCKLLVDRQNKWMTLINSEVKHWLDYTHQLETNPVLATMNKQEQAEYIAQ

NNSSDAAKLVTPSNGGLIEYLIAVANDQMKAADYAVAISTKFGELVSKSNSRIINDHIEG

TLDGFASVAKTNTNGLLRIIFDDLVIPYKEIFSKSWYTGNQAHQISDTLNEYLTDIKGQM

NPYVFSTFIENVIETTLLYYLNALKYGHSIKNKNGKFVDKLQDDASNFFKMFCEHVEPEE

VQSIVSEKFKIMEFFLELCVHPLSDIAKIWTQVLAKYHDCPMSLLSAILKCRKDVDSSLS

KQIMSQCLAITNERSKQVAAEMDMGPTFLNEFNLN

>model.g176.t1 Augustusgene.g176.t1 JFAV02000082.1:8433-10511(+)

MSAKNSSHTIFSIEDPMDSSVVAGMHTDVSTQLNNDESIELQDLPRENKTIFPDTPGNNK

NETEASRAREEWVMLEETNFLGRLKMFFHRFWHGPEVPVDETPTFPAHWIFINKLNEFPG

RYFENRFSSSMKISLIVLYCSIWFGIISSLIYPYLFHKPMFISHDGTKTREVFSMSCNSQ

LNWRGKNNACGLNAENCTPFEDQDYYIRCPALCDRGGWVYSPLVVGDERIRYKTYVIGGG

RIRDEEIEEELDAQSYPYRADSYPCAAGVHAGLVSPAHGACLKVSMSGAQLAFDSKHGKT

GDSISFSTFFPSSFVLREMYDGIASGCYDPRVLVVVLNIVFGLPFFYISSSLIGYWVTTM

VGYWSIVLVFDPPVIANPNDMNTVYDLFSVGFQRLLPLCFVLYVMWMCSIKRCLEKGSPL

LKIVFWYPLFWLGTLNNVTFDRLPVDRLTPEDLKDQPAALASVLFIISIIAVSACFQGYS

IWKSGRFQKYFKIYITIIITLFVFAYLPHLSLRIHHYILGMMLAPGCATRGVSAYVLQGI

LIGLILSGVGRWDFASILETRRALLRGGAGDAAKPPQFHFDSSSEFAISWEDVSPEENTA

YDLSSFTGYSLLANDVEVYVGNNQTVFIDELLRGNDFLQDLVEGALQYQDDVKLFFRVAH

ANLANSAKRGDYTNAAVYYYPSGNWTEPESGVS

>model.g172.t1 Augustusgene.g172.t1 JFAV02000082.1:106-1710(-)

MLRNKTQTLFKTPSTRPFAQGLFNNKNVASSSATLFMRLKSSLYSPITLPNGLKYTQPTG

LFINNEFVESYHGKTFEVINPFNEETITKVQEAREQDVEKAVSVAKHVFETEWKNSKPEF

RAQCLNKLADLVDENAELLASIESYDNGKALSSSKIDVQLVSKYLRYGAGWCDKLSGKVM

QNQPGQFSYTVREPLGVCGLLSSFNFPLMFVSWKLVPALVTANTCVFKPSEHTPLSTLYF

GELIKKAGFPPGVVNFLPGFGKYCGNAICQHKDVKKVSFTGSGPTAEKILQTCGIKKTTM

ELGGKSASIITKNAKDLDKVVANVALGIFFNSGEVCCAGSRVYVQEDIYEDFLNRLKIYA

ENLKVGNAFDDPFYGPLTNKAQFDKTMHYIDVAKSNDKATLLTGGQRIGNEGYLVQPTIF

SDATNDMRFVKEEIFGPVLAVSKFKTVEEAIAMANNTEYGLAAGVFTDDVNEALDISSRL

EAGTVWVNTYNDFHPALPFGGHAMSGFGSEMGEEALHCYTAVKAVKINFDSSLNK

>model.g174.t1 Augustusgene.g174.t1 JFAV02000082.1:4894-5274(+)

MVQDIFQDNEYDLRVCCEKLTQSVSDMSSKHKFIVNVTEVKHKEVEEEVEEEVEEEVEEE

EEDEDDDDDDDDDEEKRNEKSNSISLSVNNSFGTNWDNDKDGVVNCSVIDEKNGRIFMIS

VFWITTV

>model.g173.t1 Augustusgene.g173.t1 JFAV02000082.1:3421-3789(-)

MSSAPLLQKAPGKKIALPTRVEPKVFFANERTFLSWLNFTVMLGGLGVGLLNFGDKVGKI

SATLFTLVAMGTMIYALVTYHWRASAIRRRGSGPYDDRLGPTVLCFFLLLAVIVNFVLKL

NYQ

>model.g177.t1 Augustusgene.g177.t1 JFAV02000082.1:10709-13450(-)

MHVYKRDGRKEPVQFDKITSRIARLCYGLDPNHIDPVSITQRIISGVYEGVTTIELDNLA

AETCAYMTTIHPDYATLAARIAISNLHKQTKKQFSQVISDLYFYVNEKNGLKSPMISDDV

YQIVEKNKDVLNSAIVYDRDFQYNYFGFKTLERSYLLRINGQIAERPQHLIMRVAVGIHG

SDIEKVLETYNLMSLRYFTHASPTLFNAGTPRPQMSSCFLVAMKDDSIDGIYDTLKECAL

ISKTAGGIGLHIHNIRSTGSYIAGTNGTSNGLIPMIRVFNNTARYVDQGGNKRPGAFALY

LEPWHSDIFDFIDIRKNHGKEEIRARDLFPALWVPDLFMQKVKENSDWTLFSPSEAPGLS

DVYGADFEKLYNRYVSEGRGRKTIKAQKLWYAILEAQTETGTPFIVYKDACNKKSNQKNL

GTIKSSNLCCEIVEYSDNDETAVCNLASIALPAFINVSEDGQQQTYDFQKLHDVAKVITR

NLNRVIDRNYYPVETARNSNMRHRPIALGVQGLADTFMLLRIPFDSEQAKQLNIQIFETL

YHAALESSNEMAVEEGPYQSFQGSPASQGVLQFDMWDRAPTDLWEWESLREKIVETGLRN

SLLLAPMPTASTSQILGYNECFEPFTSNMYSRRTLSGEFQIVNPYLLRDLVDLGIWNDAM

KSNIIANNGSIQHLPNIPQELKDLYKTVWELSQKTIIDMAADRSAFIDQSHSLNIHIMAP

TMGKLTSMHFYGWSKGLKTGMYYLRTQAASAAIQFTIDQKVADQAAFSVADLSELKRPSY

NAQPFKQTENYVARKETSFTKFVPTASAPVTSASVTSVPVTSAPVTSAASTSAPSLPNEE

STREATPVSIANSNENTLVNSIENLSVSDKPAAQAVPKPAAPLNMEDEENWDIFNSKVIA

CAIDNPESCEMCSG

>model.g185.t1 Augustusgene.g185.t1 JFAV02000083.1:15573-19790(+)

MGRPPKNVSKENIERFQLELKLANNDSKLLLGDKRGRSKSCLLCKRRKQKCDHMVPSCTA

CLKSGVKCIQPSNYGGVTKPNNNGTFSANDHEKTNAEMKNNNSMNEFDSDSQSNSSFSVP

TNEKDSSSSTSAQKGKKMSNKDKQKELVNNYSAKNEQKNDYTKFLEKKLIYLENLVSISP

ASSAFEKKLQNYKKITQFLDVKLKDYEEIMNSIDSLRETEQQEQRLLLQQQKNLLLQQHP

NSTENHESSENNSQLVKPILPPPPLTNFIPMPSSMKPSVHRSAVPSNYIRPFDSARELNG

DFNSSAYYNTNKVTGRSEGGLLMANSLSTVKNIDDSLLVSRDCLGSIDFSKCVFSKYNLK

DFLSYDPAFDFEEELSRQFLDTYFTRLQFKYCLLNQKEVYDFHEYYINLNNNSLSMSQAK

PQNVYSPVHFHYCCSRMWLIFAVSACLHKTTGKYRGLPPNRYFSTAIRHITKCQDKLNNW

QKVEILSLLLLYIIRTDRDSLELYDILKDVVHIVLHELHLNQYDDGDALKNKRLRVFWCV

YLLERMICNAVEKPYLIQESDISPNFTLFTYESFRDAILPIDISASANPSSSPNFSKSEE

LTTSSNILNERKVHFINQTLRLRRLESRFVTELDIMSFKTKNKSKKFLKSQLPRVEKYFH

DLKIWRSSCNLQYVRNFENETLKLYYYRSVRLLIQPYLELLKPEHRLFRECQASAGQICQ

LYKIFHQKTVFGHSTPAVHTVFTSGLTLVYCLWLARNADDQRRRNLGDNAKHTRPTISAT

LFAVFDDLRACSVCLYVMAERSKFAIIFRDTFDQLMNCTIGNLIERCGPDSSELITLRND

DKKRSGVFASVPLGESLSNEESQFAEESNQTGSLPKKNGEPYSKSSKSSSTLHKSNAFNA

NDVKKGMPPAVSRTFGINQESEHAGFLQNLHVDLEEQQEMDRKKIALKKNMVPRSMTHLL

AAYDDHENKERFKKGGFANMDGQRLKTETISKEDQQSNMNQRPSKQEPNETPTGTRLSHF

YAASLNNNSNPKEHSAQTLPVKTVDFSSPSTMQGEQVENLSSSSRSPSSTKFVVQKPSLN

IPDEEWKDFQTQALLQQQQGQQTLKAYLSSMFTNADFLNMDLSNAGWPLEGQDVQHTTNM

YETQNHSPLHTQRSSLHENLHQLSQDQYQMTYEQQQKQQQPHAREFSSSEAAYQQQQQQQ

QQQQQQQQQQQQQQQDYYYNNFKRNDSMLQSSHQTKKQKTVADSTILPSASTIENAANEN

NAFLENIQANGVPLNTGVQNMINNISYWTSVNNEGFESFSMSEAPIIQENGSFYPRYESS

ASMNFQVDDLKQQHIQEQEQAYNQQLQSKQSVYAQPSYQHSPSQYDAHQHENTQHQPTTK

YNEHLQHGTRKPDYWNTNNGYGGCNW

>model.g184.t1 Augustusgene.g184.t1 JFAV02000083.1:13681-14187(+)

MIRLLPKLSTLTNRLVFRTNSSFHTSHNLSIKAHELPPRPKFTPDLESEIEESFLHGGRG

PGGQKINKCNSKVQLKHVPSGIVVNCQETRSRDKNRKIAREKLAMEIAVWKNGGQMVDRQ

LKMIELKQQRKLSKLKKSTKKHELVKQQKEEQKLKQEQEDREFLAKMMQ

>model.g189.t1 Augustusgene.g189.t1 JFAV02000083.1:25639-28182(-)

MSSFDRPAIYSAPVLPGEELASNENTEIVKSFKKFILEFRLGSKFIYRDQLRNNLLVKQY

FLKLNIDHLINYNEELFKKMYEEPVDVIPLFETGITEVAKRISYLISPNNNNNNSNNNDN

GDNTQNASQDGGFETGMAISEDTITDFNNGITCQLILESGGHSTVTSATANANNDIIPIR

QLNSSNVSKIIKINGIIVSSSLLASRASKLTLMCRNCKHTVRITLNNFNSISGNTSSSIP

KSCLAEKSNNMANNPGDFNNTGVNSAAQLTSASESCGNDPYLIIHENSTFIDQQFLKIQE

LPESVPIGEMPRNILMTSDRYLTNKLVPGKRVSIIGIYSIYQSKNSQLNGNNNKAVAIRS

PYLKILGISTTISDTMGAGDTTDQQQQQQQQQNETDNGPQAGIVSSTNFNEEDEQLFLSM

SRDPQIYEKLSQSIAPSIYGNLDIKKAIMCLLVGGSKKMLPDGMRLRGDINVLLLGDPGT

AKSQLLKFVEKAAPISVYTSGKGSSAAGLTASVQRDPTTRDFYLEGGAMVLADGGVVCID

EFDKMRDEDRVAIHEAMEQQTISVAKAGITTVLNSRTSVLAAANPIYGRYDDMKSPGENI

DFQTTILSRFDMIFIVKDEHNEQRDISIANHVMNIHTGRGNNNNNNNNTEDNNQNENSDF

EEVPIETLKKYVSYCRAKCAPRLSQPAAERLSSHFVNIRKQLLINELEAREKSSIPITIR

QLEAIIRITESLAKLELSPVATEVHVNEAIRLFQASTMDAASQDPVSASGSGAGANSSAS

YQDILRIEQELKRRLPIGWSTSYSTLRREFVDSRGYAQSALDKALYVLERQETIQMRHQR

QNIYRAGV

>model.g180.t1 Augustusgene.g180.t1 JFAV02000083.1:4508-6556(-)

MGLLTTADKEKIKIALPKAFNKIIDITIARLYIAYPDPNEWQYTGLWGAVALVDDLVGNT

MFLKLVDIQGFRGVLWDQELWVNFEYNQDRTFFHSFEMEECYAGLLFEDLSESAHFYKRV

AKRQKYASKKTLNNKNAMALKKKVDEENQLNVVHGPRGETLISDQRKRYNYDLPQESHTG

NHETTYNENNDEYDFAPPAESFSSKKAPPPPPPPLTMAASGTESNTLQQESNRVASPQRV

ESYASMDTYESTPQSESSTPAPSTNSVVHNVPPPPTQYMHSPPPVRPEMAQAGAKSEAAQ

NGGFAFPIPQQTGFGQTGLPPPPPPPHPPAASGYGQPPAPPSMAYGQPPPAPSLPSRSNM

PLHHQQAPQPNNFASFSGPAPGLPSRGSVPPPPPARRGAAPPPPPRRHGTAGATAALQPS

RTGRAGPPPPPARRGAAPPPPPRAGVRPAMASPQPPLQVAPQSYQQPQQYQSSQVPPPPP

LPNMQGQSQSIVPPAPPAPPMQQQQQQLSSIPIPPPLLPHMQQQSSIPAPPAPPPMQQQS

NIPAPPPPPPMQQQSNIPAPPPPPPMLQQSNIPAPPPPPAMGMPSVTSAPSSGLSEATGD

AGRDALLASIRSAGIGSLKKTDKSQLEKPSVLLQESGSSGSGPSAPSGGSGGGPPSMADA

LAAALQSRKSKVAYNDDDDEGDW

>model.g181.t1 Augustusgene.g181.t1 JFAV02000083.1:8904-10805(+)

MYSLITKIAILHFLSAVFLSAAASTTFDKNANNHVALYWGQNSAGSQKSLATYCQSGEAD

IFLLSFLYEFPDPLNVNFANACTDSYADGTLKCTQIAEDIKTCQSLGKKVLLSMGGASGA

YGFSDDDTAVAFASTLWNTFGEGSADERPFGDAVVDGFDFDIENNINIGYAALTTELRSI

FADQGSKEYYISAAPQCVYPDAGVGNLLLNADVDFAFIQFYNNYCNVGANFNWDTWTDFA

SNSAFNKDIKLFLGLPGSASGAGSGYISDLSLVDSTVANMAQDSHFGGVMLWDASQGYAN

IVDGENYVVNMKNIVDKYATSSTSSSSSSSSSSSATTSAKSTSAASSAASTKSSATTSAS

KSKSTSTTASSTSSKSSSSTTSAASSTSSTFSAATASSTSINASSSSTIKTQTTTSVFFS

SSTFTTTTRNWDEPLTTETVTLANGQVTTSHIWWLPETTTYAAATTATTGRNWDEPLTTE

TVTLANGQVTTSHIWWLPETTVSFTSASTTASTTKSTTTLAPTQASTTSTLSSSKATTTT

ASSTVTAASTASATSSAQASAIALNEQYAAGKMSGKDTCSTGDMACDAEGRFALCANGVW

SYFDCSAGTTCFAYTSNEEVLLGCNWSSVKSQFI

>model.g186.t1 Augustusgene.g186.t1 JFAV02000083.1:20126-22582(+)

MSVRVSPEAASDSSFKFFGLGGSNEVGRSCHIIQYKGKTIMLDAGVHPAYRGMASLPFYD

EFDLSTVDILLVSHFHLDHAASLPYVMKKTTFQGRVFMTHPTKAIYRWLMKDFVKVTNIS

DRSVNDYDDSNLYSNVDLADSFDKIETIDYHSTIEVNGIKFTAFHAGHVLGAAMFQIDIA

GVRILFTGDYSREIDRHLNSAEVPPLSSDVLIVESTFGTATHESRQTREKKLTSLIHSTV

SKGGRVLLPVFALGRAQELLLILDEYWTQHAEELGHGQVPIYYASNLARKCLNVFQTYVN

MMNDDIRKKFRDSQTNPFIFKNISYLKNLDSFQDYGPSVMLASPGMLQSGISREVLEKWC

PEQKNLVLVTGYSVEGTMAKFLMLQPDTIPSYNNPDVNISRKCKVDEISFAAHVDFRENL

EFIEQINAKEIILVHGESSPMGRLKSALLSNFKDLKNTKNQVRVHNPKNCVLVEVEFHGI

KIAKAIGNIVDEINGQLTELIDEPQDEVMKTEENTEDETAEKLKNQNGNLKPEEKVNEVE

SNVRVNEDGLIEEETGISVSGIVVSDESHFDLNLVSLSDIRSHHSDLKTTILKERQSLKI

SCKKELIYWHLCQMFGDLKVLIDDDNIANEKEQEHKDFAKTTDDSGEFEVQIMNSIKVTI

YNSIVTVEWVQNAVNDTIADSVIAVLLGVESSPVSVKMSSKSCEHDHSHNEVCQKLASEG

DEKITNHQTSDKDAENEALWKIAEMAKLFKEQFGETFKLSLNNEEYKAGDEIIGTVSLGQ

NVAKINFTKMNVEDCPSKPLKGRIETILQIASDLLTPLV

>model.g188.t1 Augustusgene.g188.t1 JFAV02000083.1:24783-25130(-)

MSHTAYRDLSNVSRSELTANELKKLEKYEFQHGPLSLLNEAQVSKTPVIISLRNNHKIIA

RVKAFDKHCNMVLENVKELWSEKHHKNSKLIPRERFISKLFLRGDSVIIVLKAPSN

>model.g178.t1 Augustusgene.g178.t1 JFAV02000083.1:2217-2579(+)

MKYDLLTSFSFLFPLFAQKVYRLYWDISLNQFEYSTTPTSQKVNILQNTLGRKKRHYSLQ

GKVHGSLARAGKVKSQTPKVDKQEKPKKPKGRAYKRLLYTRRFVNVTLTNGKRKMNPSPS

A

>model.g179.t1 Augustusgene.g179.t1 JFAV02000083.1:3181-4395(+)

MAPTQDVTEESLKDITTGLRTNLQAFTQKQDLHTKIETKFDKSKNENVNELEELAKLAKL

IKAHTTKLGILLSKFGESTKPDQEINTVEQDLERLGTPLLTEITDLNSSVWYLLSLLHII

HANTVSSSSKDNLPKGKNCYPKIYADEVDENVLYLVQNVEELISIVQRVLTNDNNEKKTG

EEISQQRLISVGKVWASCDKLVALSLSIPKVIIDYINSVTMKLLSDTLLEIEEWLEDPTL

EDLDDPFGLDYSDEEGNESDNEANNGSKDAPSEKMIEFMELWNTKIRLIKLLMNSLKLSL

TKATSTNNTANASGLPAKMCLHLQIIYELQKQITLKVDDLVSTVFMSSNKDFDDPMKDED

IKDLITELNENTLVKICNTVIALNSDSAKSEDTKKWIATWKLKYF

>model.g182.t1 Augustusgene.g182.t1 JFAV02000083.1:11096-11857(-)

MSDSESLYGDGMFEEPEDFRPLPPQDHYTSYKRVNSSEEPSEIKLKLIGTSPLWGHLLWN

AGIYTANHLDKYGKELVTGKDVLELGAAAGLPSVICGLHGARKVVCTDYPDSDLMANIQY

NVDQYNTKKNVVVEGYIWGNDYEPLTKHLSSDSGNDKKFDLIIMSDLVFNHTEHHKLLKT

GRDLLKENGKILVVFSPHRPWLLENDLQFFETAKEEPYGFTVEKIEMVNWKPMFDEDEET

VEIRSRVYAYYLSL

>model.g187.t1 Augustusgene.g187.t1 JFAV02000083.1:22869-24659(+)

MTETSNHVSDAYLDESVTFDSFKLDARLLQSLKSNGYHHPTLIQSSAIPLAIQDKKDIIA

KASTGSGKTLAYLIPVLQTILEFKKTNPDHSLQRDGSSSMGIILVPTRELSEQVYQVLQK

IVTYCSQDIKSVNLSSSTVSDNVLKSLLMESPDVIISTPSRLVQVLDNNNSQRSNFTLED

LKFLVIDEVDLVLTFGYQEDLWKISEYLPLKKNLQTFLMSATLNDDIQNLKEKFCRSPAV

LKLNDEEISKDQSKLAQYYVKVTEFDKFLLCYVIFKLNLIKGKTLIFVNNIDRGYRLKLV

LEQFGIKSCILNSELPANSRQHIVEEFNKNVYQLLIATDDTEYIREEDEDQLEEIEDSEE

GKQEKDKSSSINNKESKIVKDKEYGVSRGVDFKNVSCVLNFDLPTTAKSYVHRIGRTARS

GKSGTAISFVVPLKDFGKHRPSSCPTCKKDEKILSRIIKQQSKLGFEIQPYAFDISQMEG

FRYRMEDGFRAVTQVAVREARIKELKQELLTSEKLKRHFEENPQDLQSLRHDKELHPARV

QQHLKRVPEYLLPENARQDKKKISFVPFHNNKKRRVHKKKGNNNRRQGKTDALKSFK

>model.g183.t1 Augustusgene.g183.t1 JFAV02000083.1:12215-13357(-)

MKSVLKKCVSSGFKIRPNGVTRKAFHTTSVCPLRISSPTDEPHLKSSGSVDLQNGGGSTK

LYSSLSSKSIPVSNTDNGSNTASIEKTKKGFFSNKNTKETKTFGSSSTKFLNDNEGDGVM

LNSLALLENANQTPADIIRQGNLLAATTARKQVDTMKMYDELIKTKQFSHLQAKKIVKLL

VETLDEQFYSTYNYKFVRNMELDNFSYLYDNLSNDLKFELAQLSDNHLMKRNIQILKLQE

DLANITEELNQSVITELQKGSKVDFTNHKIENTLLHKDISMRLKQLDYKINTHLFGHLQS

QTEQLRWYTMKNGLLALLALVSMMIVGANFNKFINTEKPPPSVVLKTVEKEEDDLDEKKN

VLGIIGGDSIRHDADLISSEE

>model.g190.t1 Augustusgene.g190.t1 JFAV02000084.1:1363-3216(+)

MSTSGQKNLSGSRAQRFFKFLEIPHDKRTTISVLRNPDLLPIPVEHQTWGFFSNFAYWGL

LSFNIGTYSAASAASSIGMSYANIILSYFVGDCLTLCFTIANSFQGLNYKVGYTLTSRFV

FGIYGSGLGILIRILMSIVNYSCNAFMGAYTVNLMIESIFPQYLNMKNTLDHNVAMNTRE

LIGFIIFHGVCVVAYFFKPYKMNYLLIWSSVASMASFVGLCIYLTHLAGGVGDGFKGSSQ

TIHGATFAQQFVYMASYWFGSVSPGTTNQSDYSRFAKKPWAMSLGIALGLLIPTNVVPIM

GVVGTSAALKAFPGTTADDVWMPNQMCELILENTYTKGARAGVFFCGLSWTASQLAYNIS

SCGVPAGFDASGIAPRYINVFRGAMACALLSWACQPWNFYNSSSTFLTVMSAFGIIMTPI

LSIMICDNFCIRRQRYAVSEGFKVKGEFYFTKGVNWRAMFSWAAATAPGLPGLYYQIHPD

QKHTQGIDNFFYANSFVAFLISFFTYWIICLIWPLKINIKQDTKDYYNAFTDAQALKKGM

IPYSQLTEDDLNEVGLSKGEDRVSMVTESDSEASKHESMVYGKDEETDLKKSDLETEEST

ESKKKSGIFSSTGFKWFR

>model.g195.t1 Augustusgene.g195.t1 JFAV02000084.1:11789-16435(+)

MSLYSTPLKTTPGVRRNTEDNNIPLSRKNLKSGSNPFLASKTGPSHTNMTLAGKITKPAT

AGTHSGTAASRLTSTKFFNPQSLSMMRVPYGNMFGMVASGRSENIEIKNMAEQSPLELAS

QYIESLQELDNSSPILDMNSYYSNGVEYNFSEEIGGLGPLTPFERVKVLNIPDELLQKFN

ESSIKKPTTTQCGIFPEIDRCWFTIGNKLILWSVKQPNVFQTLDDDFEHDIVKVALAKPH

KNTFIHTIEHLLIVATHKDMHVLAISYDDNSEDLKVFNTGLSVPLNGLLVNEIVSNSKTG

QIFFTATNDYNVWEFQYSNNDDWFNSKCSKNCLTQSFLSHLIPTGFVSKISSFFGTDHNL

EHITKLVIDESRGILYTLSSKNVIKGYGLNKNNKGIDYTIVTNSHDISTKLRMNNNWRSR

LFFNELFTIVNIVPVSRKENNSLCLVAITVTGCRIYFTGNQGYSSASLYSNVNNNENVRF

TSLTIESVKFPPVTSEQVAKEMNRDVSERGRLMLSNNVYSSFDWWNYKLQRESKTLLSTS

TNATIISPGIFFAPVLRKSSKKNEKVSHKLYCSVPDYGILKNYSRYTENATFLDTTGDIK

CVTPVTQLFNATETPYGYANEFSTQYTREPLQVAVLTSTNLEIYKYRTPDEVFETFVESG

NVEPFVMNNGQAEACSTALFFACKLNKSEQSRISALNFFLLGIPNVIELRPTYNTRMAAP

SSGPTIAGSSTTKPSVTGHLGSGSSAGGVHGTLHNELSLDNVILSPRFYGIALLISRLCR

DIWSKPIFELMGNIKIVDGKIVQLSTDSSDDSTLNSANANFDAKNDGKQLISKISVSKDE

LEYYISSIIVLEEFLNKYHDQIICTKATSSPATSSTAVGGSSAVSGGHLTKQEEIAYQAE

NIGINSLIMLIKSIKEALSFLNVLYEESEINGFEGQYLAFKDIIKYLKLDVQLKLSELKF

RDFFTGSNSLEVKTLIREVSSSIINRNLLRGRSIDYITNALQERCGSFCSSNDVLGYRAV

EHMKKAREVGLRDVEVLDYHLKNATIYFTKICDDISLDKIKEACWIMLDLNYYPKAIEFL

LKMCHSVDKGNLALQYVNDGRLEHDERKKYYDHRMVIYDLIFQILIRLDNEKIITRDGGN

SASTISKTPLRGSQLKEVQSKSFSTIPSLGALEKLRKETYHIVFQNDDKLFHYSLYDWLF

EQHQQDKLMEIDTPFILPYLKEHSVDSLEACNMLWIYQSKRSRFYESALILLSLATSNFD

IELTKRIEYLSRANGFSKSMCSPNERSKMIKLSSDIKEIFEVAEIQDEILKMVENDSRLK

AESDSKEVGKPGKNKVTVQSKMVSQLNNKILSVSDLFNDYAEPLEYYEICLLIFQISDFR

VREEIIKKWKQLFVSIQQTQQNDSNPNDLKYLLSNLMVSLGKKLYTNDFVFPVGELIQLI

VEFFYNGNADGKSADQQISYKTGEIIDMFINCGVPYDKLYYILKNLIETNNFVITIYKKE

MIYLIKNWYKEDRNLRDIIKYEQIRSLPEVYELSKDPINEYTEKTGDRI

>model.g192.t1 Augustusgene.g192.t1 JFAV02000084.1:6441-7856(+)

MTHSIQRIPFLSQIEDMEAYVSQYKDMKNNNNTNMIMNLNNASNNYNYNNLSNNNGSSYG

SNQNHINNPSSSVMHTVQSYNNIHHNQQNNKNGRHHNHHNYHNHHSTDNNNNSNNNNNNH

YQNGYKGFSTNHKNNNVNGGMNGGNGTSMQSYGSQLQVQRSGGVLNPTSYSSLYLNNDMA

SAPNYLNVPAHFANGIYNGSALDLDSVTATTTPSHPTSATPLYQNTNGYDQLLNQRSKLV

SPALSTSDSLITPQSQVSRGQPSYINDFYQTQSETGLGQSNSLYSLQNSMQPNAFDSTKN

MVNSTNNNNNNNNNNNNNSNNNMMNESTQGTASSNLGKMSGLYSMNSASPFGSSGATFND

TGLYAGNQSSSPFSYTQHMGASSTAGITVNGSSSSNGQRMPLQQFEQLQTLQSMNQPQQQ

SHQTSGQQQFLPQQPNLQPFSSAGSSSSGQLNNSSMLSPYNKIWSDNMSVWS

>model.g203.t1 Augustusgene.g203.t1 JFAV02000084.1:29355-29603(-)

MSSFYDEIEIEDMTFDNDTKTFTYPCPCGDKFQVYIDDLFYGDDVAVCPSCSLMIQVIFE

KEDLQEYYEQADHTPPEPFMIAA

>model.g191.t1 Augustusgene.g191.t1 JFAV02000084.1:3886-5235(+)

MSFFGSNLNNNNATNSALGSTMNNGLAGGMNNTMGGLNSMNSMNSMNSMNNSTMGGMNGM

NTMGANTMSGGFNTMGTMNANANTNFGTLVPELANDITIANPANDSVSDISFNPQQDLIF

SSSSWDGTVRIWDVQSGVPQGRSQYVHNSQTNTPVLSTRWTDDGSKVASGGCDNNIVLYD

VMSQQQQVIGTHDQPVQAIRFAQVGPTNQSILVSGSWDKTIKYWDLRQPQPVCTVQMPER

VYCMDAKSKLLVVGTAGKQVAVINLDSPQTIFKTMVSPLKWQTKSIACFNQANGYALGSI

EGRCSIQYIDPEEHRKVGFSFKCHRPNTQTVGGTSSNLSSANSGPVSKTSPAYVYAVNSI

SFHPTYGTFATAGGDGSINFWDKDLRHRLKGFPSFKAPISVGTFNRTGSVYAYAISYDWA

QGHNGRKPEYPNVIRLHQCSDDEVKERKKR

>model.g202.t1 Augustusgene.g202.t1 JFAV02000084.1:27247-28557(-)

MSEQGWTDPKALEQPLELHRVARPLRPVKYVPTKSIVFYSKNSPPQFTYDIKIKMPIKAT

SVIVQVLYVALNPVDLKIYNGYTTNMNSFTGMGREFVGKITHLGKDVKDYQIDDVVMGTF

FHPHLGKGTLQSSIELDVNKDVFFLKPEGLSMQEASGGSYALGAAYSLLSTVKQLRKTSN

NNNNMNNNMNNNNNDNINILINGGGSSVAMFALQILKYHYKITSKITVICSAYATNVIKA

QFPELIDELIFINYLSFVKNNKPAKTLSAMIQDQEIIEYVNTPGSTETAVLSVPYTQGKY

DLVLDFVGGYDLIENSNKIMNKNAEYITTVGDYKFNYRKDVYAFNNLQKNNLEMFGRSLW

SKMSWDFQYTHFQFDPALKYNTSYYNDWKTHVRDMLNHQTVKVVIDKVYDWKDYTDALNY

LRAGHAHGKVVLKVEVF

>model.g200.t1 Augustusgene.g200.t1 JFAV02000084.1:24510-25466(-)

MSSNSIKILAGNSHPALAEAICKKLGLPLAQINVYQYSNKETSVTIGESIRDEDVYIIQT

GTGQDEINDFLMELLIMIHACRTASARRITAVIPSFPYARQDKKDKSRAPITAKLIANLL

TTAGADHVITMDLHASQIQGFFKIPVDNLYAEPSVLNYIRNKTDFDNAILVSPDAGGAKR

VASLADKLDLNFALIHKERQRANEVSRMVLVGDVAGKSCLLIDDMADTCGTLCKAAEVLL

NEGKAKEVIAIVTHGIFSGNAFEKLANSKLSRVVCTNTVPVNSNLAILDQIDISPTLAEA

IRRLHNGESVSYLFTHAPI

>model.g194.t1 Augustusgene.g194.t1 JFAV02000084.1:9904-11160(-)

MYHSSPKKKIDIASRVLAEKNVNESIVAQSRLKAEHAHIITKQKARKRTLYVKKPPTYDV

TKKVNIAPSHVPSMPLLISSDKINIHSTLFRSTISKTPSKRTLKNTNNSFVLNGVSLQTK

AKNNSNESFKLVSQHLHDKHGNSFSTNDTPLTEKLHAHNLNLNSNLKLVVNPLTAQNIAH

LQYAIKQTEAKLTGDCGHAMCHSFYAFSQNPSADATGFSAEWELRCQMFHDMDLFADQES

LLKGNIRQTCYLNFVYTKVTSMFRTSVDLNSSSWPVMKTIADCTDEFPFPPEFMIVPEID

EQFLNVETGQGENNENEDEIIVHITSFDDCAEGRYQGFAGRKSRFIQKPNLKTSNSLFPK

KSDYYSLFSSRKNSLSRRKNLIDESSKRLNLKENLANRPEPCLLLKRLNKNAFNNIHDK

>model.g193.t1 Augustusgene.g193.t1 JFAV02000084.1:8004-9614(-)

MLRSKRYASSWATANSKFKLLSEYKLKCGLEIHTQLNTPHKLFSMAENQPFQSMTEPNSR

TSYFDVSLPGTQPNLNPEVVLYALKLAKSLDCEINLQSTFDRKHYFYGDQPLGYQITQHY

NPFAINGVIRLNKQFDGILENEHPIKIIQLQIEQDTGKTVYDEKDQISLIDLNRSNVPLV

EMVTAPDFYDLKQIRAFIKKYQSLVRHLKVSTGDLETGAMRIDVNISVNDFPRIELKNLP

NTSSVMNSIKYEYMRQVEIIRNKEAASKLQQSETRGWTGVETVKLRSKETTIDYRYMPDS

ELPPLSLDPSVIEDVSSLIPELPEEIMKKLMAAPYNLSLKDAKILTVSSSGHDALYSNEG

LLEYYLQTFHKFLSLFEECAKADLKPRINYKLPSNWIIHVLLGNLNKLQMHLDDCKLTPE

YFAIFLQLINDNKISKTSGDLLLFHIMENNISSPDFSALISEFELNKVEEVDETELNEIC

SSIISDINDPKLLDQIKSGQKKNSLKFLIGQGMRSTQGKIDAKLFEETFKRLLNITW

>model.g199.t1 Augustusgene.g199.t1 JFAV02000084.1:21420-24398(+)

MGLRRWIASNSNNNSTVSLNDLDSENNKNKSSASVGNRSHGNGVTTDYESDGGEREKSSK

NQSKNHSSKGKKKRSMLKGLRSSFIGGSSSSASDNGSSDDDTEMNSNVISGISKKSSHFS

LFPQSKNSSQSLSTVKSDSDDTVDDGEEAEEEAESEEDNSEKRKVDRDTQSCKDNSKSDT

QDKETISANESPLTKLSTNSSDATNETFINETDATFNKETLNSTTANDVKDNGKLVSCSN

TANTDSGISNASKYGTKNGSKLKNEITKNDFEHDEEDQIFNDTSATDEQKTIKDTFLNDT

FPQEKENTENATSSADSSRGTTMDNSMTMSKHSDLSSRTNSAGLWLPSDPIAVPKPKKLI

QGGDEAMLNLSKIPSASAPATPTGNNSGIGAKKSGMDNTMNHHHHHHDTDHINHSTGHNG

RVHKMMHGVAHKFAFDDSSHHNNSNSGTGKKFEISTDSEKVFGLENFGNTCYFNSMVQCL

FSIDEFRIQMMTPLIPEDNHERKLYCHPLKPRYFTKQSLTKNKQKDSGASNSSGNGVNSG

YKIISTDDVLAKLNTSYERIIVGRSQVDKKRHSSETRKRNALIKGPVVNIDHLSTNYHHI

TMSWEKDDSKIMYYCLKDLFEYIIENKYSTGVVSPIKFLNSLRIVNCMFNTSMHQDAQEF

LNFLLNSISEYLDANHHSNFIKSLFQGEIVNKTKCLNCQNITFRNEPFLEFSVNISKKYK

DLQSYFDNYYEKESLCGANKFHCDNCQSLQDAERVAEIGSLPKILVLHEKRYKYSERENS

MIKLFDKFQYSRNLKVCTTLSNSTNDSNEEPVNVCKNYELHSIVIHLGGGPSHGHYVSIV

KNSSLGWLLYDDESVESIDEKDVFKFAGSHEDLSAAYVLFYEEVEAGKTDNLNDEKSRNK

YYQKRVADFIKKDELMRFKIDESRKNSVSSSASMDSMATSGSIMSNASGKTNHSNGSTSS

SSKSKIKRRGTKLFSGFKKNNNNSSTASIKEES

>model.g201.t1 Augustusgene.g201.t1 JFAV02000084.1:26317-27111(+)

MATIQANKRLTKEYKNIVANPPQYIIARPNEDNILEWHYVISGPPDTPFFGGEYHGTLVF

PADYPFKPPAIRMITPNGRFKENTRLCLSMSDFHPDSWNPTWSVSTILTGLLSFMTGDES

TTGSIVTSDELKKRLALQSKYYNSRQNTRFKKVFPELVESYEEELKQKEEDFKNGGGNSN

ASENYKLNFLGIDQQNELESRKQMERAKNEKVIPLSEIEDPEDRIRAAELASGENDSSSS

FKIFNIVSMAVGFASLAFLCKYLLD

>model.g196.t1 Augustusgene.g196.t1 JFAV02000084.1:16792-17157(-)

MKSAFKSQFPFEVRKAESERICSKFINRIPVICEKADKSDIPTIDKRKYLVPNDLTVGQF

VYVIRKRIKLPSEKAIFIFVNETLPPTAALMSSIYQEHKDKDGFLYVSYSGENTFGKMSD

SE

>model.g198.t1 Augustusgene.g198.t1 JFAV02000084.1:19624-20619(+)

MPETTKLARTVTLNNGLKMPIVSLGCWKIPNDVCANQVYEAIKLGYRSFDGACDYGNEVE

VGQGIKRAIDEKLVERKDLFVISKLWNNYHHPDHVEYALDKTLKDMGLEYLDLYYIHFPI

AFKFVPVEERYPPGFYTGEKDEKEGKFTFEKVTYLQTWQALEKLVHSGKIKSLGVSNVRG

VLIQDLLNGCSIKPQCLQIEHHPYLTQENLVQYAKNEDIQVVAYSSFGPASFLEMDVELA

KSTPSLFEHEDVVSVANKYKVSPSDVLLRWATQNDVAVIPKSSKPERLLQNLQFEDKVTL

SKEDWDRISKLNKNVRFNDPWSWNNADIPTFA

>model.g197.t1 Augustusgene.g197.t1 JFAV02000084.1:17931-18626(-)

MLSVMNRNTPRVYPANTATPSSNKDSNDKRGSGCNNLIEDRKHKYSKYERVHGVTATNAH

LLKKQTILGEMRRRYKDMKTLEIRNKMKTNDVMVGSVDDQSGVQLTTKSSTPGNTDNTPS

WYDAFVSDDDHDENTEIAFTRMKDEKKEQEKEYSPHMNYDYINKMSAKYQISVEEAELYY

SQMLNEELQEEEQYLKDQENKLKKEQLELQLQDLEEQEQMDIEYLLENFKIE

>model.g204.t1 Augustusgene.g204.t1 JFAV02000085.1:1084-1683(+)

MGISRDSRHKRSATGAKRAQFRKKRKFELGRQSANTKIGAKRIHTVRTRGGNKKFRALRV

ETGNFSWASEGVAKKTRIAGVVYHPSNNELVRTNTLTKAAIVQVDATPFKQWFEAHYGQT

LGKKKAASTEEPVSRSKSVEKKLAARAKDAKIEYSVESQFNSGKLYAAISSRPGQSGRCD

GYILEGEELAFYLRRLTAKK

>model.g206.t1 Augustusgene.g206.t1 JFAV02000085.1:7704-10046(-)

MKLNNVLCAFMALPLLVSNSQTVGATPITVTDSTTDTLTSCSTTFSSSAPSTITDHTTVT

SVTCTELHCTASQSSSPVTTTDISIVTTTSCSTTSSEPTTLTPSTSNTPSTPTTSCTTTS

TPETPVSPSIPTTSCTPNTLTTLKTSTASVTISTSASSSSSASFSGSSGHFSFHSTPQSS

QNLVQSTSSTSSATFSAISFTSSTQPTSSSTNAVNTTNTLISSTQPSSSLTASANTSSTP

ATLTHPTTPTSSNFMSSISTTSNATVPPRSIFSSTLSSTYSSVSSEITTWPISSVSSAVT

PSSHNNNPSSSSNSSSSVSHSSTPESTSSLSTTVTVSLVTLPSVRPHTSSSPGHGSSSAL

STFFTNTSGLASSNMISTTPTFTLSSTSNAFSSVLVSSAFSKSEDTSNTLSSFFPRSSTY

TTTPSFSSKVSSSTLTAVSSGTVTSQYPTTVNGSTTTMTNTYVYSSTFTTCPFSSGSFSK

TTATVLVTSLASTTEASSKELSTTIFSSLTSSAKLSTTSPVVSSTEFFGQSSATTTIPMA

ASTFSGPVGSTTSISSKTSTTEGITTTYAHGTSKTSDISTHVLEEVSSIFNTDTLPRATL

STESMSGPSTTTLTSEPESSKPTGESATTETFTTVHSKSTGKVLTSSVSGFSVASSISTA

SVETSKLLSSGSTSTTANVKQTTKTFTTSYVSSGSVVVRPVTDSSSMVTFFSSNIAFGET

TASIVTSRSAEFKSASSATGYSSATFSASQANAGCKNKQVFVKTLFNSFIGSTLLALLIF

V

>model.g205.t1 Augustusgene.g205.t1 JFAV02000085.1:2816-4648(-)

MRTSQILGFSAASAFLTQQAASAPVSSNIVVPTIELINAQSTVVSSNSSLDNAQTTIVVP

TIQLGNVKTNLGSDSVSTFSTSSNAIPTSNNIISFPTLEFLDPRTTISASASTPSFIVVP

TIQLFDPRTSIPPASTLSSSSLSSAAASITSESTASSSFSDALATSSFITVETIGLLDPR

TTISQSSSTTESTATSGFITVETIGLLDPRTTISQSSSTTESTATALSSFVVDISSSTSS

ASVVSSSSATLSNSTGVAALAAPFAAPFIFATQSTSVDVSSSVAAFSVVPSSVAPLNSAT

SANSTDVDALVASFATSSVVPSSSVAASALASIVAASAEASSEVSSAEATHSVASTLSAV

SSSGGVNISSAALVQNTTFFASASTTSDVISSSTVTSATSAYTETVVQTATTSYPTFING

NLTFTTETFVVTSTTTFCPDSATASSLTPATDASSTTSTPTFVNNVASVLGALSFANSTS

VESANAISTASTTILAPSSETVSSAAEHVATTTPIASSQVASSQVASSQVASSQVASSQV

ASSQVASSQVPKPSSGTTLTTSVAASGSSSASVSATVAVAQANDGVNNKITFNNLLTLII

STSLATLFMFL

>model.g207.t1 Augustusgene.g207.t1 JFAV02000085.1:12936-14583(-)

PPTSSTPVVPPTSSTPVVPPTSSTPVVPPANSSTSTTPCSQSSSFISTVSTHSFVQSTSS

TPVVPPANSTTPVVPPASSTPVVPPTSSTPVVPPANSTTPVVPPTSSTPVVPPASSTPVV

PPASSTPVVPPASLDSSCPTSFLDSSCSTNLFDSSLVPPANSTTPVVPPASSTPVVPPAS

STPVVPPTSSTPVVPCSSFVSTVSTDSFVQSTSSVISSTTGPRYMNSTTTAPTSSSVVPP

ASSSVVPPASSSVVPPASSSVVPPASSSVSTLSPQSSVSTTAPSSSAVSSKTFITSSTVT

KIYPTIVSGSTTTTTERFVSTATITTCPLSSASTATAETETSEQTKGIKTTTTETESASS

TASGSPSGASPSGAAPSGASPSGAAPSGASPSGASPSGAAPSEVSTSSAENSDVTQSSTS

ETGVFTSFSTTVLASTTAAPEQSTASASSSLNESSSSVSASVSAAIISSSSSSPKTTASI

TTQQNAAHHNQVSNGILFKSILTTGLFTFFMLY

>model.g208.t1 Augustusgene.g208.t1 JFAV02000086.1:838-5637(+)

MSYHHYLNKKYPVRNNKIKPDHVVDGVPVFKPNMHDFADFHQYVQDINHYGMESGIVKVI

PPQEWVEMQQITPQLLSNIQITKPIEQNISSNKPSGHPSGIFITNIEKNKKYNIIQWKEL

SYDYPLPSNIVIDRSSQNSPNASRSNSLKKASTPTLPNNNNNNNNNTPKGKSRSMSPTRK

RNSKITSKKIADFTLQDFKDFIDNKDYICDEDKLEDDFKQEDYNFKKEDTQDEKLDDSMD

KENEDATGKQQNTHKSIFHNSEKLKALEEYYWKTILLSTPMYGADSLGSLFDPQMETWNV

SKLPNLLDELHEKVPGVTQSYLYAGLWKATFSWHLEDQDLHSINYLHFGAPKQWYSISQK

DAEFFYNLMKELFPNDYQNCKEFLRHKTFHCNPKFLQEKGVTVNKIVHYPNEFIITFPYG

YHSGFNYGYNLAESVNFAIEDWISLGLKAENCKCIDDSVSVDVQKLKNIVRENKLKRQAV

QTLLDASKREDLPQRKKLRSQGNLLDSSDKLSNDSNDKMPSAEIKQEGQGLRNEKEKKDS

PLLATATTNVAETTDNKKLKSQETNIKSPSPLHSSSNNMQMKVQLPSLQSPSKQNSSQTL

PVPRGIFEKNDLSALNTPSVAPVSASTAPVISRLSSPLLTRIFTEADPLLDFQKKPNGPN

SMLSTKNISSPLSMGSTPFQLSKIPSATNPSTKTPTTNASNLASFGNTEQTDKGETSKST

AQSQISSLNTRFMNNLEGMPSPTFNLFKNDSNQVTRFNTPSSIPKLNLNSNNSLNSEQPK

SATTQLNPFIFKTDSNNLNSKKASNADADNNSAKSNTKNTSTAATNGSNTNYNANLLSNK

NLALQNGITGLNNKENNANNADTMSTGSSFFNVDEDEDNMLALSLASMANSRISSPRLNF

MPLNTNNSSAVSMSNFNNNTGPLNNINTPFMMGNNVNSTNDNVTSNGTNNNTASINTANI

TNTKAANSPLTNYFNSSTIESDLAARGAGNTGGNTDGTTNSTTGTAAGSAFAQPSQPQPQ

HSIHPNHPINQILHLQNGNTGNRAPSLVSPRPNYSNINSLPSLGLSNSGTPIVGQSGPNA

NTGLLNQHNSLLTTATTQAAAAAAAQAASSSNSVDQQQKQQQQQQQQTPSAFPQQQLHQP

RSNSNTSVNLYSSNLGSVAFGSTSPGPGLFGSVLQNNRGYTSGFGTTTLVNPTSTSIPSS

ATGGIIRPKSPQSPYLVRGRSPNRVMLNISRDGSPLSNFHLNNPNVSTTPGSTNLLNLMN

PHQPSITTSLSQIIADKNGQTGYQAANPTAANNLAKDNQEPDTKGKPAGSQTRTPTKENG

QVKKRKYTRKNPTKKQLLQMQKQMGSDLQTQNQLKSGRDSSFSVAQNPTAAPPIGSIGVP

ATSKGADSFMSQVGPNEHSNLRSTSQGIMSAISPVIPSSDAFLSAQRYHLEPQQKSNYST

KNNSNNSSMISEQPSPGLPPQSNQNNSFSSAGALTGSFGSLYQSTLLAESVDIPTNENEL

NNANLKVQDDEVVVAEDGVKSYVCLLCNKSFSSGHHLTRHKNSVHCEAKPFSCPKCGKPF

KRRDHVLQHLNKKIPCTKNDSSETTAGSAEDESAIKKVII

>model.g210.t1 Augustusgene.g210.t1 JFAV02000089.1:3125-3694(-)

MYSTNTGVSGPYYYLESMIKKGTAQGKNIKFYWSIRNWSTLEMFSDELKLLSEQDSIDVF

VYISQYDSDRYSESSSNSQSELEIIEQSNSNNSSNKSQIREKMAEKSASTDSLSMKNFES

LLPQVTFRPGRLPVNDIVLADISESLDNTNITVMTCGHPELCDDIREIVLQNVKIQKNKN

IRLIDELQTW

>model.g209.t1 Augustusgene.g209.t1 JFAV02000089.1:1-1403(-)

MWPKFLIQTTALFFLLFDVGAALDVTWVKANQQSVGIACKAAVSGTALYCSKDSTKVYTC

QCSDVVGMGAYAYCGYTETEGNEGSRKRFQDYFISMCPGLTRQEIEDSYNNVTKYITTTA

KIPNFNKTQPIRTPVKYTKKTYINVYNTNIQRWTNVDWGNYFGIGLMGYWAAIFLIFGVF

RVLELMNLTEKCVTNTTLYKFLQSKVFFKAFLGKNYKKSYFFGYMPITMDVIIILGYVIL

NTVGCFVNIHFLPNNTTWPKHSTQIGRLIGDRTGRIAMFTINLTFLFAGRNNFMLWCTGW

NFQSFITYHKWVSRVTLVTVFVHAIAFYISSVTAGNIGTRAGRSYYRWGSVATVAGALIV

LQAVYPLRKRFYEIFLYVHITMAVLFLVGTWLHLVYFDLQQYVFASVGLWSLDRLLRIIR

IAGFGGYKKANITVVNDEFLVITVKSYNSKAFKPQPGNFVFAYFGLK

>model.g220.t1 Augustusgene.g220.t1 JFAV02000090.1:21919-23760(+)

MSDGVFQGAIGIDLGTTYSCVATYESNVEIIANEQGNRVTPSYVAFTPEERLIGDAAKNQ

AALNPKNTVFDAKRLIGRRFDEESVQSDIKTWPFTVIDQQGSPLIQVEYLGETKTFSPQE

ISSMVLTKMKEIAEAKIGQTVEKAVITVPAYFNDAQRQATKDAGAIAGLNVLRIINEPTA

AAIAYGLGAGKSEKKERHVLIFDLGGGTFDVSLLHIAGGVYTVKSTSGNTHLGGQDFDNN

LLDHFQKEFQKKTGLDISNDPRALRRLRTACERAKRTLSSVTQTTIEVDSLFDGEDFETS

ITRARFEDINAAQFKSTLEPVEQVLKDAKIAKSQIDEVVLVGGSTRIPKVQKLLSDFFEG

KQLEKSINPDEAVAFGAAVQGAILTGQSTSDETKDLLLLDVAPLSLGVGMVGNVMGVVVP

RNTTVPTIKRRTFTTCADNQTSVTFPVYQGERTNCAENTLLGEFDLKGIPPMSAGEPVLE

AIFEVDANGILKVTAVEKSTGKTANITISNAIGRLSSEDIEKMLHDAEEFKAADEAFAKR

HEAKQRLESYISTVESVVSDPVSSAKLKKGARSKVESALSDALAALQIEDASTDELRKAE

IALKRAYSKATSSR

>model.g212.t1 Augustusgene.g212.t1 JFAV02000090.1:2968-5241(-)

MSTPKKAASPLIYCKSHVAIHPSTKKNDNVFGFLICYRNVASSSTPSYSSGLGGENNHDF

NVAWVRDDNISLSLKQQLLDADLYFADEYVTVNKHQGPGTSLSLTKKVLPNVDVQMLEYK

SDWYCNINAVYSIQFRLPSQWYKGSVIINTKQHEYTSNIPVLFFHDEKCESTKLISKRLM

LESFDPFTSERNLYWGGDDFKKFLNKNCDLQHANEVAGLNDNVYLINPSLNDLRNFQTTN

NSSLKSSGLFDSSKETKKPGLFESFEKSKWSVLGKIADVTNKLTNDDPKINPILQTLYSS

ETINKLSNDKYVKKLLKKSQPQINKISSDFDGAKIYLAKWALNVKEQVEKQENVYKKLTN

EMDNMVTEEEVHVALERNHPLSLVKWESMFDEQGRLKFTVGEIKDYIFHGGCVDDKVRAA

VWPFLLNVYPWDSSEDERVQLYETLKTIYELNYKNQWILDQTPDDFEEEVWKEEKFKIVK

DVFRNDRHLDLYKHNTDDGNAVSNDGNESAQAEEESMEDDDESTWVIKNPHLRALENILL

SYNVYNPDLGYCQGMTDLLSPIYHITQDEVVSFWCFIEFMNRMERNFAVDQSGIKEQMVT

LGELTSLMLPDLMAHLSKCDCSNLFFVYRMMLVLFKRDLDEEKVKQIWEIIFTNYYSSQF

HLFFVLAIFQKNKVAIEHLEEFDDILKYFNDLKILDNVELMTRAELLFIKFKKIVMNVDE

TKTLSTYNSTGAENNNKLEISPRLRKLLSKEVILKNDT

>model.g216.t1 Augustusgene.g216.t1 JFAV02000090.1:10767-11453(-)

MFILKSGIQRFSSCSILEKSSEARKLLTLVNSSSPTSAYDDVDRSSSTHKINQSDLNKTK

HENAVEKGVTLVQNASDANKTKKKPEWLVQKLALKSKFEHWNPTKKLSRDEMDNVRLLKE

KMPYLNNTDIGNHFKISPEAVRRILKSRFSPKTERERQGLEDRWIRRGERIQAQLGELAQ

QSRKASPGQKTQFSTKQTRYFKKDSEEISKKFQNKKQLKVLENLKKISL

>model.g214.t1 Augustusgene.g214.t1 JFAV02000090.1:7258-9822(+)

MGGTSGRLPDNGEATYNNITPHTGFFNDNDSFAPHNSEDLRTRQTYDVGYGQYTLKEQIE

KDKMGVPGSYQQPQQQQTCISNGQQANEYNTQKAFPTEQQQQRQRYQQQQYQQQQLQQQH

HQQQQQYQQYPQQHPQQHPPQAQPAKTPQHSSANINVPRTNRVTKNTVPQQSPVYRTMAQ

HNSTKLSPQLQQQIQQQMKQQQQQIQQRQQLHIQQQQQQQQQQQQIQQKQKHQQQQQQQQ

QYLNSSNATAKNAPGMNMANQQESTNETPKPAPYNSQLQDKNSKQAHGKQEPTQSLQNIP

SHMLGLLNPENAAKATIVHPESLTNQPPLPYPVRKYFANMAILRVHEIINMLLMVRMGPN

GKDPQGLTNFVNETFAPYCNIKYTVRNQAKEIRTYDLSVLVFADFLKNWLSSCTVRVEIV

PQQLRSQVLNNGTIMFECPCCTFTRYFDDGSYITRFFQMKGIFDSWLKLEWLDLNGHSFI

TGVEWGSIERLLLNPQETKDIFYSMISKEGTTEEESAIGEFSVEKKTENTKEDPQKEAKS

TLENDGQIPPGKSGTSLDASIEKLRSKFEIFQNLNSFGTTESFMKILQVNDVMQHLKSLM

LYQRLNNVNSPLESMYQLVNNMKLQQKQAAEANKKAAPAFSPPSAEIQPQAFSQASPTPS

VFQQRPPNTFAGATPQNSPPPGYAANRYSASSAQAVPQNFQPQGSPQEKYFSGQNTPQEP

LKNYPNNPPYTSTTTGPAVRQYAGSSEQLNNSYMHSVHPTSTTGQAPSGPYGESLHPSQT

QPGQNQYYEQPSQQHSGMSSTSNLNDHSFGPGSGSAEPSVMPVPQNRSPSGLSPHSQVQI

DYNADTFKQNKPEKY

>model.g219.t1 Augustusgene.g219.t1 JFAV02000090.1:19871-20956(+)

MTDPNSKTPWYLDQPLGDLKMKYQYIQYKEDLRLRDATLNPETSNYSLGVSVLRENSRRN

RYCDIMPYERNRVTLNTLHSTNNYINASYIKLDFLANTKKYYIATQGPTKRTINQFWTMV

FQKCVLGPDVVIVMVTPLVEQNREKCFPYWPSSISEEKILHIPKVQNVDGKNDISVFEEE

LNLEFISEEKYYHSPLPHAKEQHHNHIYTKLKLTNLQTGEYRNIHHLYFDKWSDMSSPTS

VDNILGLITHCNELNAKFGNPVISHCSAGVGRTGTFIALDYLYTSFLGNHRKLVSSVKQE

TSSTGEHAFLNTADSEQQQKQNLEDLVQKTVLKLRSQRMHMVQTFDQYLFLYKAVKTFYV

AD

>model.g215.t1 Augustusgene.g215.t1 JFAV02000090.1:10107-10670(+)

MSQLDANVNIIWPTKNDRIIRTNQTLQTIRYLAKLGKFSSLLVSIVYLAFRNILIPSYEK

LLEQRSDFNMNTLFALRKTLSKAQKLTKNAPSSKTTTDNDQLAESSLVIFTDNCCKTYIK

RIQVVNESFASDSQDANGLQTSIQTFAENLHNMQYKHDKDKLEVLKKSNAIVADLRSLKG

KIIKEGLA

>model.g211.t1 Augustusgene.g211.t1 JFAV02000090.1:272-2860(+)

MPASESPSKQPALAVSGHPKENDDAEFTDDLEHYSSAHETLETQESKTGIPEENSELQSP

EKSANKEKQTESPRVVTDTARQGHSSSKNSLLFHGMLFFIDSSLPHDMFVKLIRKISKNG

GQIIDDNEDSDDDNDDESRLNESGNDSQKTRDIIKITTIPYTNSSSTSLAQSPPGTKVKY

VVPPEFIFQSIKLKQSLDLTNHLLDKIEEEAVPLDQESLPLIPSDSRKRSINAITQDDEE

DDEEKEYASENNKSEKNSTSEIDNEHPLNGEKSVAQEEIFQGNSENTASFDLEHLGDTVD

EPTASEGKPESKKRKKPKTTVKQININKNSFSPEEDELILAYVRKNPKLRNTHSLYHRIA

QVMENHTGNSVRYRFRRTLASRLDYVYEVDDTGALLRDENGEFIKTSVLPKQLKSNYKAE

EDYILCLAVKKQFYSDIYHVDPDYAEVKEQQESLLSQHKQNTDQPIESGEQSNQIALSDE

HNTHSTSHDQAPRAEDSTYDTAYLNDILDHEQLNNIPSFKDFKIGNKRGPLKRFFFKNFQ

NIYPAHTENSWRDRFRKFCVPYGMDDYIAYYEGCLAENIEPEPIKNMTTKHQTPFKYTNA

VGGSEEEAKKLFPRLRTGPGNFPKNFDKNTSEKIPAAAKTFLEETSPAALMHVAANAVNA

AENIDNLLPSSVPEHQDSAEADRNGSAQSDKNDTTNYFDMFSEVELPMEYVEESKQIEPN

EQLCEYFEIDMAKFENDLSKFLLTISNCMVECIGATESHLINTIHKETNIPKTELAKILF

RCSNDSSLLTRYFFYIFRDEVTEPAYVEGIFGSEDDKVILENSNASTFTPEFEKLLSVHS

AEGVMKRKLFLSTSQQNEIVQEN

>model.g217.t1 Augustusgene.g217.t1 JFAV02000090.1:11992-14097(+)

MEGLLYSSDNELSSENCILKVIVSDSGKKLCVELPEAKRSLEFPVNSQFLLGFDMSSVII

FWMHREIQYDFFPLSEAGDFEQTYKYFSHLVPCEKVFDDEEEKEKKGQEGQEAEFTLGDI

PASQKLASAKNKPISVKQEKVPAPDASPDSFQAKLKTPAKQSLAKNKATKSKQATKNDEN

VDNLLSDVNKKMNALSLKTEAKFDNVQISVFDTVREKEFVQAKTGSVAFHFSEKSDTWLQ

LSNEKHESLANTDCNEDIEPEFDIFRLTFKFKYYLEEQIQITYIVTFSNLLQYTKFKAAY

LGYQEANRTGSSPIQVSRNFGDSFFTRSDDEDDEASDSEYEEASDEADQTSKIIDSFYRP

GSTTKSLTVGLNSNRSYVVHDNNISVIKEDVINDELEYISTFENVSYNDSKPFVPSKPMM

YLQDQALIFQNENAPTNLYKMDMNKGVVVEEWSMGENPIVNYSPARKFDQLNLEQTFMGI

SKKSVFKVDPRISSRSKIVQDSNFEYKTNPNFSSIATSQSGFVSLGSKNGSVRLFDKLGG

RAKTLINGFGDTIKHVVVSNDGRWLLATCSQNLILYDLLIAEGKPNAGATGFQKSNSDPN

FFFLTVPESFEEEQEETSTKNPVEFSKAYFNTGVEVRETTIVSSYGSYVLSWKLSNVLKG

HLEPVVLKKFADNKVRENNFIFGSNDKVVIALNKGVTLASRK

>model.g218.t1 Augustusgene.g218.t1 JFAV02000090.1:14865-18395(-)

MSGLEEIEDSRQGSGSYRQRSFLSASLRNHHGSGTNTHNNSFSELPTQETIFKNSLIKKF

GLTSLSQNNNNKDENFSQLPLASAGSQKLSFANSNENFLHSRTSQSQLQLNQLISSNEAL

EKTYLNAQPSFLSHENSFVHKSNVELNQNGTGLDDYELEELRDGFFDPVFPKHTPIRPII

DENFAETSKGSSAERADEEEKNTAESSSNGAESTVDSLPIFHASAGPPSHLAFSKVFSTF

APRKIDVHKALQKKKKILKFFIAYFIAMVLSVIPRTRAWFGSDRYIWFLPLATLIHHPSR

TVGVQVEIAVWAILGGSLSIAWSALALYIATCNNVALRGYGGILWLSMTLAIILSVWLGS

LFRRLVYFSTAFNTGILFTHTVSFINAFPQVADFQIDNSIRNWKIMWDFGMSYLFGILIS

TVVSCIVFPDFGNKIITDTFAETIDSIENFLVLFIDPENFENQEELASKEKTMVKKMNYK

CTQAYREYSNQLKLSKYDENLLKNVRNSLTNIVAPLRCVPLNLQNVIDQKALEKLVSGVN

ANGINFKVEETDYEDLQDDITALRKTLQKPLFDLILQMIICLDYNKEMFKNPKVQNCDTL

HKYDVVLQDKIQNTDKAYKNFIKSKFFNKELLRNQNCVEMFLFLRYLVHSANHLVVLNST

TIETIGTQKWHIVTPSYPLSTSLRRLPHQCALDQGNGDQMNYSDTKRATDEIFENIYNAN

MSKHIFQASTSDKHVKAISRNDFNKKTTTSSARWIFWNFKCAFLCDEMRWALKVCAVMFF

FCLPSWLPNSYKWYQKYQVFTGAILLNILMNRRNIDNWGSFAIRLINCVVGVFWGWAANQ

SRHFASPYVIMTFASLICAVYAYVFFVNRDTKASYTALLTFTIIVLEPLGNKSVYSHHSV

NTATIWKSTWVTGLALFFGCALSIPINWVLWTLSARTQVREGANALVGHIGKSYQFLVDR

YLYRDVDDEPSLLELKYSNVSEIRLSQSIDALEELLNQAKKESEYIMKFKAAQYEQLIQC

SRALVEKLLESRLSAQFFDVWNTDADSRVTRSLLSFRRDSVSTVCFVFFMLSNCFQSKNK

VPPYLPNPILSRKKLYDAISRFDTLDWNGRQDSEQSSLFSTTFNFTPEGKVSFKLFEKKH

WQEVHAIAFSSAFTGVTAELQRMIELSKQILGEETRY

>model.g213.t1 Augustusgene.g213.t1 JFAV02000090.1:5623-6825(+)

MPINRGQRKRYISESENSEDDDEGIVARRTSKRSTAGKKSSPVYGNDDDENGAGDETLYS

NSGRPIRSSSRLSSRKIDFKEPNEDEFDSDAEDVSANNGSAEWNVAQELEKDDSKLNDSE

EDMKEEEEEEEEEEEEEEEEMTNDAMGSKQNDEEEENDEDEDDEEEEEMDEDIEEEEEEE

EDDIPIDDDDNDDTSIPALVDEDDIDAIEQADNERDAVSSRNKRRFDKASTNNDVLNNQN

AAGNENEMEDEEDVENKLNEKELRRTENLRRRKVLHDKRMEEEKRDTINKLLKRRAGKSR

GKDAGSKKRAGLQQPGGMASGDRSSSSLASVGTENDKDEEEDEEEEGYKVNKPRRKYDNR

GMRRIVYGKDNTIRYAFEYQGKSAGLQPEAREQQNSTIAAD

>model.g223.t1 Augustusgene.g223.t1 JFAV02000091.1:6409-11718(-)

MNKTNETPRLVPFVEKKTANIDYKEKQFSSNVEKALQQFDSVDDWADYISRLGKLLKALQ

SWSPKFQNVSYYVPHPYQVARRLASSLAPNLPAGVHSKTLEVYSIIFDKIGIETLSNQCN

IWIPGVLPLMTYATLSVKPQLIEVYEEYILKMNARTLKVLVKPILASLLPGIDEEGSEFQ

AQVLQLIQTLQEALNNDALFWQSFYLIMIKNKDHRLGGLAWMTKQMPSLNAIPHLATSSD

EKNTQKEKNSTSTREDVSTKEQRKAALELLLPSAKPLVSLEVGLLIRCLVACLSNDNEVI

IQRGILDLILQRVHLDSPVLETLIFPSEKEMLVMACCEQLLKKDMSLNRRIWSWFLKPSS

KNEEAPDQQYFVDHCLDTLIRGLHNYIKNGRVADALKISQILMHKWEIGSLVTERMFLPL

TKASIGENEKTVLAASEYFDSIDTKLIWNSILSTLLKNDYSVLNFVLDNYHLENEEEMVV

KHLPMIFLELLVLPQQRDYSLLMKLIRLIPGRAYLLEPQGASPNSLPNDEEIAAEIFEYY

SNDNAMSPFSPITLTHIILSKLHALLLSSLETGKNVTESIDLFVTLYSLLPEAELMTDFD

YNKISDRLVEAKYRIGEDDCLTIIPLSNLFVAILLSKIDTPSMIKNLRFLFQRLWVLLTN

PSKQLEALQALENLQRVVPQKYMEASIVSVFMEEKEITKKLLVMNGLWTQSNKTSIVTNT

STSNYSLAAKPLELILDELADESKSEYLYAKKWLLNILEDTKSANRLFEIYAWNLLNFGF

INKNEEFEEFDNIDIFTYYVNTLINVLNCSDGQSLALLNSELTSSSCANLFLSTSKDKKV

NISTYKNLFVAILLNFLCIKSNKNSRSVRVVLKFLHMVLNGSEYNFKSIVSLFLDLSSSY

IQKSSNHETDLIIVALLDIVSNVLSLSQKQNIKLEIFDNSQSHLKYVDFLVSSVLLIQKP

SIMNAYVKLLTESILYFREAVFNIILPLISAMVASVESLYSEEYGTYENYDNYLSVYSLL

DGIEELLQVSHSYMSVSLSHLNGGNKSDFLSNVVSNVFSGGSTYSASGSNGSPESNRILV

NRQVVVQSFAATIKCCFKIWKSCNSYEQDLDRDKMYSLSLYQQSCKYKYKSRKLLEKLYL

LEPVESSQCLLDITNEPASIHSIYKLVYALDGNRPTLTLPHFFRMCVYKHNPGSTLLTQD

FKVKMSTSSSFYHVNSALLMDFVLIYVANLEFSAVEDINTEFLQFAKEVVGNASAYENIS

VKFLELVATVCYKMGHCQFGDDKRIRKEYSDLFTKYLGSLDYNLVQLKNKDVYRESLQKC

VQYTEHIVADAKFSEKYNLLLSSLVGTFVAPFMKKINANSSINTEFLPVESITSNDENAK

EKSVSKAASKRLSRQNKGNSIDIPNYVAEFMSFIAPLGSKVKPWKAIVSDFFSMPPNDDY

SKSSKIFGNAFSNCLFMVDQSSAWRTIIYEWSQYSDNKGKLINDLVVNCSAATSNPFGSW

SQTEVGTKCLDYFKICYLLLISPKDEHILQFKPLMNEVERVCLNETGDIENEIVCIGFKL

LRCMFLKFNSMQFLNNWGSITFILQKNLQMVLESDGKFDIHLVFQIFKTLDMLVTLDFEE

FDSTQAWLFIVDSLKGVSSDDYVSLIDLLAQSETALDINVQGSDSASKEKLPSSSSATAT

TSFTNSSFVSTTAVSTPTPSSLLSMIEKQKMPLLYGKHDAVAQTDLKNFVEIFSYANYES

LFNDDGAHYSIEEILEDTLQDLFYSPQFFF

>model.g224.t1 Augustusgene.g224.t1 JFAV02000091.1:12018-12578(+)

MDIHSQSATPKLPPKELSAENSNKQVQKTPEKAYPLPPNTEFLSQAQLNSLLQNIEKLKQ

YVHELNVSQFDFHELDEYKAKFENLLGSFQRLYKEKDNLETKHFETKARYQNMTKDLAEL

QEFINRNLSQSAYRDKIDLHLKDIRRETDQMADELLNDYSDKLLDQFFQARIHYHNESQK

YEFFLNY

>model.g225.t1 Augustusgene.g225.t1 JFAV02000091.1:12648-13652(-)

MLRYNTPQFSGSSVQYSPYFDNKIAVGSGANYGLVGNGKLSILDIQPNGSLLLTNAFLTQ

DNVFDVSWSEMNENQIITAQGDGTIRLFDINLSKYPLKIIKAHDKEVFSCNWNLVNKQSF

VSSSWDGTCKIWDPERGQCVASWMPVKGKQMGNGMQLQNKATCMHQAIFSPHDPNLVLSS

SSTSHITLKDIRSPDQTSIIGHHGLEALSVDFNKYQPFIFCSGGADKLVRVWDYRMMSSE

SNSGGVHHNRRKPLHDHVGHRLAVKKVAWSPHCDDVIMSTSYDTTVKIWGKNRMRKSFRK

HTEFTTGCDWSLWGAPGFVATCGWDGNIFVWNALQ

>model.g226.t1 Augustusgene.g226.t1 JFAV02000091.1:14442-16784(-)

MSESNEVNQENSNNNPNSSNDVDQSSNTSNNQSNAPVHDDDNNVAGDTTNTNQTNPTEPE

GTGTPSAPSEEPFTFHPGTNVIVAINYVLQGTGTAAANLRTTGDGQQGSNSNSTNETSGE

NGTANDSTTGDTTDNNTNNNNNNNNNETVGSFYLNFTGLPANTSPERFSQLMSIAANVAL

SRLSRGLNRPRKLKKEFFDRLPVKKFIEVDGEVCSICYDPYVKDDKEIAAEKEEAEAAAS

STGLANNLKDKDRDTIKKRKLKDGEMEEEHRSNSNKSRKLNDEQGVSTTSQTDTLEAGQE

GRSEMQTAESTTNTEVLNAVDEASNNNNNSSIETPGATEETANAPSEQQETEKNDDDDEY

KHSAVELPCHHIFGRSCIYEWCKEHNSCPVCRAQIGSDDDLEPLQLMNHIQNLGEDPTFD

LIRRLLYGTPTEGNDNETNGNANGDANGNAASNASNESNGQDATDTANATTDGNSNNERQ

NATGIPGFERFTTIGGGANRPSIIYLRRARDETTPTNDRNDGLPNFIREMFGSDTVGNAN

QQGNPNTNATDGANINAADNNGNANGSETGTRPSTPTGSGAARGGGISTLLRDLFTRSNS

RDTVNTPPPAPQAPPIPPFLATGTGTGAGTATSLGTAAVPATSTPIPTEAEVEERRENFR

RSALNILDTIFGHNSSEDTSRVPEPIVGQQGVASYRDSSGRVSTVNIPRTTAAASTQPPS

SPAPNFTEPANDADAGSATAATSNSTSETNATHGNQQSNQDNSSNSSGESEYTSLPGSPA

L

>model.g221.t1 Augustusgene.g221.t1 JFAV02000091.1:1455-3638(-)

MQAKSVATFLIACALYFGNVQSAAINDLTPKKAGVRFGIERGSKYIGSKSKFVKRDDGGD

VLKFVIDNQENYYSVNISVGTPPQHVSALLDTGSSDLWLLGDFKNVTACEAYLDAIGIDD

SSSASSSSSSNSKNDNIIENDVVYSNFHRVLNKYDKKQQEERQKEKAQQKKQARDVNGKA

ATTAARSSHSATGSATQTVDLESYFNTVSFQTSGMDSTNTGDSFQSYDSAAPTETDFDYS

SDYYGSYETGTATTSGANYASMVQELEDAEASQVLEGCSDSGVYTPADSSSYKKNESTAP

FLIQYGDLTFASGWWASDSIAINGVDVALQFAVANISNSSSVLGISYEASEASIAYSDDD

DGQSTSTTKRRKRSGNHEKKELDSFTYPNLPVALKNQGLIDKISYSLYLNSLEQPYGEIL

FGAVDSTKYTGQLYTIPLVNIYAASGVDKPIEFDVTLQGTGISLGNGKNKTFSTTKLPAL

LDSGTTLLQLPLDLAEMFAESVDAKWDDDMESYMMACPSTKEQNEISFVFDMGGFLVYTP

LSNYILTTSDSSTCALGIMPSDELYVILGDNFLSSVYVVYDLEDYEISIAQANYDGAAQS

GDLEAITSTVPNAKKAPMYSSTWSTAMQISTGGNIFTASNASNPWTATNNNGGSTVSLSA

SDITKTASASAKALSTGSSTGSSSSSSSSSSSSSSSSSSKSKNAGNSIMNYSYESSFAAL

ILLLSALM

>model.g222.t1 Augustusgene.g222.t1 JFAV02000091.1:5341-6282(+)

MLFWCGKSQNKLSMSFCLQETLKNFTQIFFSNTIKEQFSQEKRNFFNKMLPTPYVQCKEY

DKVYEPAEDSFLLLDSLETEIPFLQKQFGNDNMPSYVPVIVELGCGSGILSTFMLQNSIP

MQNSLYFPIDVNPHALKVTKDTIDYNMSMAEKSCNNNLGNLDSKAHQAQQPSATPNFYST

LQMDLFFGLRPKTIDVLVFNPPYVPAEYVPKIPETEKGLNTQEMQCNTNRTGNPDDAWLD

LALLGGPQGMDITVKVLENMDKMLTSDGVAYILFCARNKPEQVVQKWIEKGWKIDLVEKR

KAGWEVLSVYRFTR

>model.g227.t1 Augustusgene.g227.t1 JFAV02000092.1:1-218(+)

MGKIQLCFSSCFQRWSCFVFHCDFLCCTIQRQIHQLVGKQRSICWPRWIRFAIMEHHRYS

KRFLWCCSRSLS

>model.g233.t1 Augustusgene.g233.t1 JFAV02000092.1:19430-20221(+)

MSWTGFKKAINRAGNSVMIKDVDKTVDKEYDTEERRYKVLERTGGALQKEAKGFLDSLRA

VTASQVTIAEVISNLYDDSKSVNGSYNVGNYYLQCVRDFDSETVKQLDGPFRETVLDPIT

KFSNYFNEIDEAIKKRAHKKQDYDACKAKVRRLIDKPSKDASKLPRSEKELKFAKDIFDN

LNDQLKAELPQLVSLRVPYFDPSFEALVKIQLRFCTEGYTRLAQIQQYLDEQSRDDYAHG

RLDTKMEQVLQQMSSLDICALGFK

>model.g234.t1 Augustusgene.g234.t1 JFAV02000092.1:20840-23641(+)

MFQLTENRKVKKFCSQLQRYHLVLFNRIIKSPERYIRWFVLLNATLAYPTLNDFFIWLVF

FPKHGMLGSTTDALNGNGPPTYYWLFGFFSTIFLISAIFWKIFETIHNVSRGKLLNCSFL

LSENLISFCCSFSLCRLAIMLLGIAHERPLTKEQVYKAYINTVMDTPVIPLVLISAFTMF

QSVQFLEVLQKKALLIKYNPTKVVFGDFSLRDALHIGFMELMNFGLLLLLNFCLSVGLYW

CSAITFHTLIAIFVGLIAHHFFNMIYLPAVVILSVKLNYVSFEDKKQVTTKNSNKSFKIF

FYLRDWCLLTGGFQLLYYLSKQSATFRKLSNNYHIYHENAFFWKYKNSYATFASDILEFF

VYIGLIGCISYFVVEICTLKLKDVYELGSETLTTSSVASSANHEEYHGYVLCSEETTEPD

VDKALNNTFQDFLAHPSTNSVIIHKVYSLQILKLFQDEIECSTEKSVVTIPLFFQQNTDF

LPPKVVKLEIDETFNRFLSILYDDGTITIFELIKHQFVIKNFSYSDDSNRVRAVESYFKL

RSEGSSSSNSSIISKNQDYDPLVGNILGQYIIIDSNGMMHTISFMQENTFHINGNNTANP

SQWNITFISNRISSNKITQCRKMITPRANDRLIFQTEDNLVYVCQEIVNKPWRSRPLIVL

ANAFNTIKKGRMLASSSIGINNKMYPGDIKIINLSYFGFTFIFNPSNMKGELVDVQTGTL

MKTIKIDQRYCFDDSLHIYKDVIPTFCKFCGSASIKKLCLTYNIKASTPSTAAGPSEQKV

LMKTHIFKTNKKNKKLICLRVERDPREIRCFGLESVSEKTCDLDVTGKPWFLKKYSLTHP

SKIFVLQNGDGAQKSDAEHSELLSIIDTTKFKIQKYDVMKSSHPWDRNNIGDDGTLPGFA

ENQIIKAYDDNNILFLRKNNVTKSVALEFIYNIQ

>model.g230.t1 Augustusgene.g230.t1 JFAV02000092.1:8793-9830(-)

MLLTAKLLLLAVLSCACAGKAKDDFPKIESLLSLTNATESLLLPFNKTRLPGSLGSFEVQ

THIKNFFASLPVSWTVEEDSFAEQHHNFTNLVFSLNTDSLSIFSESEFAHEQAIETDQTF

FVVAAHYDSKREPDGFIGAIDSIASCSIMMYLAFHLSEQIEQAPLATLSRGIKFVFFDGE

EALEEWSETDSIYGSRHLAKKWHAEDLLGRIDAFVLMDSLGGVKQGLRDVKVFPFFEQTV

EFYNQLAAIEQRVNTLDSLKTQYFEPEVLAFLNYNKQDPNYLPIEDDHIPFYKLGVPILH

LIPYPFPAYWHTIEDDFEHLDIPGIHKWAKILGIFLQESLFEDDLV

>model.g228.t1 Augustusgene.g228.t1 JFAV02000092.1:2508-5141(+)

MSDIEKKAATTVDVASKSTDDNGSSSNEHYVSGEKPIEFITSIKSYSDEQVWHLLKVLNF

DDVKDLNDLPPEVEFLGTKVHEITIDESLEIMKDAIEYHDNDPNIDEDQYRSFIRYAEEG

VDPNNDVAVFELKALAVLLRDHSPYPEVRAVCPPAMMDDPTIPIETFRAYFFALIWLIFS

AGFNELFSHRLVSISISTSVIQMFLYPMGRFWEKWIPSWGFSVKGHRFALNIDTPWTDKE

QMFCTLIIAISMGTFYTSYNILTQKIYYGQDVSFGYQFWLSLSVQFMGFGFAGILRRFVV

YPVKAVWPTSLSTIALNKALLTPEDPNAKGMSRYTFFFSCMVFMFIYTWFPTFIFNALNT

FNWMSWIAPNNWNLAAITGGVSGLGVNPIASFDWNVITSSSLLTPWFSMSTQYAGSFLAI

IITIACYFTNYQNTKYLPIYSNSLFTNTGEKFKVKNILNSDYQFDEAKYQAYSPPFYSAG

NLVCYGCFIATYPFLITYYLIMDHKMFYSAFKQWFSALWAMRKKDSWISLWRDEARALDD

FKDPHSRAMARYKEVPDWWFIAVLLLVIIVAICTIEKYHTSTPVWSLFMSLGFNFVFLIP

LAILQATAGVSLGLNLLIEMIMGYALPGNPMALMIIKAFGYNIDGQADSYVSNLKLAHYC

KIAPVALFRGQLIMAFIQIFINLGVINWSIDNFEGFCESTQKAKFTCPDIQTYYNASVMW

GGLGPTKIFNDVYPILKWCWLIGFLLGAAFGFAKKFGGRYYPTWFNPVIFLVGMLIGPPY

GLMYYTPPLIMCFFSQWYAKKYHLKLWEKYNYVLAAAFNAGLVLSSIVIFFAVQYKDRSL

DWWGNNVPYAGQDGLGLPLWNITATAKGYFGAAPGHYP

>model.g231.t1 Augustusgene.g231.t1 JFAV02000092.1:10326-17444(+)

MEQQNINLDANISGSSAGEAQFVSNHEKPASVQSIFSSSESVPNPQNNLENYNSELCASN

PDMQGMVVPNDIRGPNHDLLYSRPKNSLGQPDDEDEEEQHIFDFANDNDESEEDADQPAN

TRADASFFMSDKFAVNELIKGNNFGISDNFTKQATNATMANESKQSPVNLNKKSGEQENS

MGSSGNILRDNVSFAKHSSEPLLDSTENKIAGVHDHGLLQESNGSSLHFAKTSANTKPLH

QSDKTSSESPRSGDFSRAKNGQKKYSESKNKPDMLRQAAESPLKSRVLQGVHASVLEEEK

QYTEPFTAQAGSKGLESDYVINEKFKMQAPQNLNAVQRSSVYENKSTVTAIPVRTPSYKR

NNTTTNSIYGSKIGHLRGRSKDLDDTASLHSLSSSLTASFSKNFLSSFYKNKHSKRRKEQ

IALLPQQYWMKDENCKECFSCGKNFNTFRRKHHCRLCGQIFCSNCTLLISAEKFGYEGKV

RICESCVEHFENDYYEDSSDDEVSMINDTHDNSEDTMDVSDHLLLHPTISESRNHNDYFG

ALGAQDGYTKANSANVRYGNATDVVSLFGEDDSKVLTESLNAPKMTIPATKQGEALEIGL

PDYGKNQSPFRPSLQFQSPASHHAPLSKLNNELSRVKHLFRGSSKDTHKDSASTFTTLPE

HKHLSALKSHSGFVQSNIFPENNSYSADNSSSSNSVAHNLSNGNFKFEFRMNNPYFGGTK

NKSPVPRVTSEMSLEGLNAETAQKQKVYARNLLSLSNKNNSSTTISDSTNYGKPLNLSHG

DSNPKIISPEPVSNAQEEAEEEEEEEEEEEKEEEDDDLSSGEDERSMSIFAALNDAHADY

NHRRHYQNLEQANNTPKAFEDHRSRDQYSVGAHFQNSLEPSHARLVPRSESKSSQRAEAS

LQRIRTRRKSKSKSVLASNKNMQIFSHSTPNLMSILSNEKEKPTSGDSRRRQKHDSFSKR

LVNINSQYSSEQKSTLSEVHQVHAEALLNQVLLDQETLNVSRWTEFFGPVFRKIEAIDVS

AKTTGSLDFRQYVKIKRIPGGDISQSSYINGIVFSKNNPLKHMPRFLQNPRILLLMFPVS

YQKNDNHFLSMQIVLAQEQEYLNSLVSRITALNPTIVFVSDTVSGYALDLLQKAGIVVQF

NIKPQVLEKIAKFTEADIANSVDKLVANMKLGRCETFEVKSYLYNDISKTYTFLRGCRPS

LGGTVLLRGLTTESLRSVKDTAEFMVFVILSMKLESSLLKDNFIQLLLSEYNKLVVLKKN

RKSIGYFSEFVEKFNRRLLSISPSIDFPLPFLLSKTRSMEESIDEMKRQMQRIHEFNDDQ

FQKYIDTDIFLKELSSDKFVTLKDVRYFANFIIEKNIESLTEACYAKKRQWEIFFSLSYN

MLGTGTHQSISLLYSMVSNKTGTPCIGPQVVTVEFFWSNDVTIGQFIENIVSTLHFPCSA

ACGGTFLDHYRSYVHGNSKIDVVVEKLQSKLPSLKNIILMWNYCKKCRMSSPILSMSDDT

WNYSFGKYLELFFWGVPSCMSGLGNCKHDVLRDHVRYFSYNDIVIRLECSAVDIHQLVTP

KTKVSWRPFFAIKSKVDLANTVRSKIDRFYGSILKRLVRVKLDSKENTAEGITQVENMKK

MCVSEQASFHALATSIYKNTPGDECLQLNSVIQQLHSKCFDWMNDFAEFEKKYLPSEKDI

ARITASQLKKMFVESGSSDEEESKKVETLPFDQEKLTDSNGLMKVDFADKLQVEDNDVIS

SLNENDKRLKSETPTGEVSDGYQVNQKAPSIRTEGSVARNFSRQESDLSKHLRNMSNVGA

DEQESKVGRLTMFFDQMHFDALSKEFEHQRELERKQMSRRYYKNQMRSESLKPIVDIYKN

VDEAVKEPLHLEKGTKKKDTVIASQGPTTKHGEATFPESNPGWTTVTLGDETIRSKLKNA

EPIKHNLPLPPVTTTATTTADQASLSDLETDEELCEAGQQAQELPSSLLNKNDLKQQETQ

ETQAQQQEKTSLLKILSSFWEDRSATLWEPLESPLGPNEHIFAENNVLIKEDEPTSLVSY

CLSTPDYVDKMNQSFAHANAEISAFDPFENPEVLESLLNKKTGMHLRYQLQDGNVVMSCK

IFFAEQFDAFRRACGCTTNFIQSLSRCAKWDSSGGKSGSAFLKTLDDRFVIKELSHVELD

TFIKFAPNYFHYMGQAMFHDLPTALAKILGFYQIHIKNTETGKVFKKDVIITENLFYDKQ

NLRIFDLKGSMRNRHVEQTGKANEVLLDENMVEYIYESPVFVREYDKKLLRASLWNDTLF

LSKMNVMDYSLVIGIDSENQRLTVGIIDCIRTFTWDKKLESWVKEKGFVGNSTKEPTVVT

PKQYKNRFREAMERYILVVPDPWFKENMSQAET

>model.g229.t1 Augustusgene.g229.t1 JFAV02000092.1:5249-8383(-)

MIVTQGKQSLSRLIVLLLAVTVIATAANPFENSNSGKIKTFELQNIPRRKPTPGDDVCPP

CFNCMLPLFECKQYSSCNEYNGRCECIPGFGGEDCKEALCGGLSSENDQRPIRNVTEGFC

DCADGWSGINCNICQSDDVCDNFLPGEVKTGAVCNKNGMIVNKMYQGCDVTNEKIRSILG

GKSPQVTFSCNRTSEICDFQFWVDQVESFYCGLDTCKFEYDIAKNTSHYNCDNAKCKCLP

GEFLCGKSGSIDISEFLTETIKGPGDFSCNLETRKCIFTEPSMNDLISTIFGDPYIMLQC

ESGECIHYSEIPGYLSPEKPALLLKQKIVLIGTSIALVMFICVIAKCILISPLFTSENKA

IEDTKTDSDRIADLEFLSNTMGATLTFENINYFAGSQHILKHVSGVIKPGLTAIMGGSGA

GKTTLLDILALKNKCGKTKGSIKINGEEVGHTNKKFKKQISKTIGFVDQENFLLPTLTVY

ETVLNSALLRLPKNMSFKEKQKLVIKVMQDLRIYDIKDHIIGDDFARGISGGEKRRVSIA

CELVTSPSILFLDEPTSGLDSNNAKNVIECLERLAVLYNRTIVVSIHQPRSNIFQKFDKL

ILLGNGEMCYSGTTLDVRDFLKNCGYECPKDYNIADFLIDVTFETPITVSSENQEENFED

SAHDHAHSHALVSPNTDTAIQGEWAHLAEHRDELRSLLAPTNKSNYNTIDDASKKNILTS

SMLSSKFLESQFYADLEFQIRNANASISETTSELAKRQVPKASFWQQLIILSSRSFKNIY

RNPKLLLGNYSLSLIMGMFLGFLYFGVSNDISGFQNRLGLFFFIITFLGFSSFTGLSSFN

LERLIFLKERSNNYYHPLAYYLSKIISDIFPLRVVPPILLVLIIYPLVGLNMGIEGGIAY

PFFKCIEIFIMFNLAIAFLIQSIGFFFQEMSNSIIFSVLVLLWSILFSGLFINTQQITNV

FFKYMKNGSIFYYAYESLIINEVKDLMLKEKKYGLNIEIPGATILSTFGFQVQHLTLDIS

MLFAFMVFFFIFGYIILKWRVVENR

>model.g232.t1 Augustusgene.g232.t1 JFAV02000092.1:17773-18798(+)

MNFNADMMSELSDPAKGLYDVMISQARGTIFNQDEITNATGKQSSDNRLLLQELINKNLV

KVVKHENSIKFQAVDVSEASKKSTMSRDEQLVYSYIEASGREGIWIKTIKARTNLHQHIV

VKCLKNLETQRYVKVVKSVKFPTRKIYMLYNLQPSIEVTGGPWFTDGELDDEFISHLLTV

IWRYVGQRTFPDGFNNFNDVDFMTGKGETLYTKSVNNYASSYEILEFINGANISKVDLSP

LDIESLCNVLYYDNRLCKVGLNSYKVTLQSVLDLMGEDKIGVKSEDEDALASGKDSAENG

NHVSRNECSTIVESSFNIFQSKNFITPSLNDKEAVFFDEWML

>model.g256.t1 Augustusgene.g256.t1 JFAV02000093.1:52851-55148(-)

MSNSSAFSPNLSSKKLKNNDENTETPSKLLHKASFSGKSASKQFNRGFGGALANSHAPNI

LGNTPASKLNSLSGSYRPPKVFKKTTSISIPSKQPCLKRQASMSSLSISNPKLKKANSLA

MLNKTLSKIGDSNKGAMQHMHVEGKQHSNTVTYSNHTVGSSSTSLNTNGIRKFNRPTMQK

ANPTLQKTRSFDISSTFFGKSSFDSISSTTQNNNGQKSDDASSSFSIQNQGQDGCGSSGF

MMQMPDRFIPASTNDKANSTLASVLASTNLSNKSVAQNITEALKPHSTAHTFSDDSEEDI

NQSASSIIDSPPPPANASPETHLKSINKKFFQKTVAQACGIDANQRILQYLPKAPLPSLK

RSQSYSLENSRNKYTYDYYSGVRMDEFTNNSRSSSPNLPTDFMADRTKVNYQIRKINQSP

ERILDCPEFFDDFYLNLIAWNKRNVLAIALNQAVYLWNGDSGDVLQLLDYSEQPLMKVTS

VTWSDDNFHISVGFEQGFMEVWDTETMKKIRKMHGLEKSRIGVQSWLCNLIASGNKDGMI

KINDVRVKNHVVQSWDDHHHGLEVCGLAYRSDGLQLASGGNDNSMIVWDTRTSQPQFVKR

HHQAAVKALSWNPDISTLLASGGGSNDQKIHFWNTSSGIRVNSIDTGTQISSLHWGQSTS

NKREIVATGGEPKNAISVYNYENRLKVAEIINAHESKIISSQLSPDETTLCTIGGDENLK

FFKVFDSKKPKYSSTRAQDDFIDDDHLDGMDTDGKLKSPHKNITIR

>model.g261.t1 Augustusgene.g261.t1 JFAV02000093.1:63466-65127(-)

MDLVEKLKNKQNRGKKISFDINSTNKSNLQNKKNKKKAFTISDRRFKVYKTCCSTIHNDN

QYGQKIVDKRLSKLLNDLDTLSEHASTNTTILGPYSVFKHDGCVKIFATGNQGISNANAT

PASQSNTKNKKLVQSCNTQWQSQQQHPHQQHQQQQQQQQHPHQQQQQLNTYLQNNTPQLF

SFPNLLQPAPVMPGLLPGANAFPLSRKDSNLSLSISPSVSSLETDDKSAAHDDDDDDDDD

NDDNDDNDDNLFDDSHDFDNDAANSEALQRHVRDFANSITTVSLGDLGKQHDLPPLEPQF

ETVDVNLDELTRLLTDDDHLVKTFTRKISNLTIELKYNISTTILNNLNTYIAQHNYENYN

GLIDIMRWCLCNCEPNSKIPSTIIKSIYYHDMQVDMHFINLTFFILMLKDYNINNYTEWL

YQWIQKCYTSLKTIAAANASNTSSFNANVHSTTLPKRLNFSLFLVLIEFYVCKDSMGYGI

QIIPMDRLVTCFKLLNFLSAQQVSDMKFDVDLLKLKVQFDKELMRRSNSNKEMQKYFKIQ

LDIDSTLLHDVYDV

>model.g250.t1 Augustusgene.g250.t1 JFAV02000093.1:36972-38954(-)

MVFRLNLLSSFQCEAAAYLMANLDTKDYVLSNKFEIQGIQTSDLHPESKLLVEDFSSGER

YILEYFVNEELILYRVSYRDSEKLLRFLQKFPRPVQPPKTMVKPLELNSIPSFLQYIKVE

KQPSQKVPRPVLDLLKVTFFRPKTPASDRNLSKALDEVDAKQYLKDRYYTFIYSSELKMS

HFVKNTLSKVKLLCKNKSSGSTEATIFYQRLLEETALSFTAFDMKYENLSTKLIKEEDNY

EKLRQSLITQQWDISDHEELSQMKDALAKLNCIHKLRELKLQIIICLELLTFTTLDKNFI

NFESRYKKKLTQKAFLYSSNLIGRKRKTKTKKPKHEKSVPVDLCDTLDIWVDKLCITETI

LGINSSPMHPTELKIDTYIKTKLLRAASESSSPGFHKQVLLPFYSRKVPNAVNFVSNKMK

GTSFNKKITENNNNKRETSHSQRKNSATVTPSYSISVRKRRSSSKLSDLLQGQPVNTLLQ

RTKSDLNLLEKRSTPVSDTPVEHTSSSFYKAKSQMNDVENSGNQNGATLHRKHSELYDVD

DHNSVFVNGKSVKLARLSSSVSSFQRVGKKQLDSSQALKAISSVRKASQVQVEETPKAKS

NNIDVIESPQTAQIVNSPLMQFASTRTSPYDKLQTETQKIVDEHEKGSTAPVRRVLFPTS

P

>model.g270.t1 Augustusgene.g270.t1 JFAV02000093.1:83068-85005(+)

MPPKRPEPKKWKAPKGPKPIQRKNKNTIGLGKAIITQRKKENQVNILPDGELQFTTDKHE

ANWVKLRSVTQESALDDFLSTAELADKDFTADRHSNVQIIRMDQNTDNVRSQGFMMTNEA

KSELNAKQREHLKDLIVPRRPHWTKEMTAYELQRLEKEEFLSWRRKIASLQESNEDLLLT

PFERNIEVWRQLWRVVERSDLVVQIVDARDPLLFRSVDLEKYVKEVDERKQNLLLVNKAD

LLTVKQRIAWAKYFISRNISFSFFSAKKANEILEDQLEFGDDYIAKKDYYSEINNILDEE

EVDPELLAKIKILKIDQLEDLFLTNAPDEPLLTPLPGQESLIQIGLVGYPNVGKSSTINA

LVGSKKVSVSATPGKTKHFQTIKLSDDVMLCDCPGLVFPNFAYNKGELVCNGVLPIDQLR

DYIGPCTLVVERVPKYYLEAIYGIHIQTRSVEEGGDGETPTAQEFLVSYARARGYMTQGF

GSADESRASRYILKDYVNGKLLYINPPPHMEDDTPYTKEECKEFNKDLYTFDHLPENRKE

QLSEAVKSKNIEDFDLSRDLGKLTFSAHVGAEGDNTAKSVNHGGKQAALYNAAVDLDKEF

FNMNSVEGKFLSPFHAKNAEAGSSRKKHNKKNKKAKKKAEMLYSKD

>model.g241.t1 Augustusgene.g241.t1 JFAV02000093.1:20414-20659(+)

MSRVISKIKTKLGSEKNKDTKKSQTSTASKSNTKSTLKTSKKSNLSSKTDRVGGSSVEPH

PEALSPREAARRAAEQRLKTQK

>model.g271.t1 Augustusgene.g271.t1 JFAV02000093.1:85710-87452(-)

MAKYVPIAASNAFKKLQAKKKQASIIFTSLVVLLVLVLSLYNRSTKDIPDYTQDCKSYGP

LKPAYDVSLDQIFTDKDYQDSVIEKLLGAVRIPTEIYDDSPNPDLSIPLDQDPIFKNFST

FHDYLEKAFPLVHKHLTVETVNSAGLLFTWETKHTNTSAKPLLLMAHQDVVPVNKDTIDR

WTYPPFEGVYEKKTGYIYGRGASDTKQLIIAQLEAIELLISTGWEPTKRPLLVAYGFDEE

CGGELGAQSIAKVLFNRYGANGLYGLLDEGGGTTYFGDGDYLAAPAIAEKGYVDTVITLN

TKGGHSSAPYIWDFDQTSIGILSELNYFLENDQFEIDIAPNNPALGWVQCKAKYDKTFST

DLVKGWKSGSLKKLGQALTQSRLKFLFKTTQAMDIIHGGVKANAMPEQVSELINHRINTK

SSVGETLDQVLSSTVTVADKYDLGIILEWTNGTETSVLKEGTKGDFIITVKGALEPAPIT

PTGDDEMWNLVGSTVVNTFKNPFFGEDQDVYFEGTISTGNTDTKWESRYKLTNRIYRFSA

ALETPGSNAHTVDEKSSDKSLISALAFIYQIIGNVDAYSTD

>model.g263.t1 Augustusgene.g263.t1 JFAV02000093.1:70405-70752(+)

MAGGQHHHRINLDKYHPGYFGKVGMRYFHKQQNHFWKPVLNLDKLWTLVPEQKRDEYLKN

ASSSSAPVIDTLAAGYGKVLGKGRIPNVPVIVKARFVSKLAEEKIRAAGGVVELIA

>model.g239.t1 Augustusgene.g239.t1 JFAV02000093.1:14871-15299(-)

MSTTKTYKDIFTLFDKKNEGKIGTNQLGDFLRAVGYNPTNALVNELSGTNSSFTLNEIDQ

LVLDNKQVLEATTQASVQDFMKAFQVFDKDGSGKISIGDLKYMLTGLGEKLNEDEVDEVL

KGVESDQDGMIDYKKFVEDILKR

>model.g252.t1 Augustusgene.g252.t1 JFAV02000093.1:41356-42378(+)

MSWSAEKVQVTNSAQNEQNQPDNGAQEDINQRERIAQSDSEYDEEDDEDYNPEAQPEEKV

GRLKNGAADDDEDKTLDNDKEYQKLEQQYKSIESGGTAGGLVKTRRARLLEKEAEQKQKY

DNVNQHLVTTSNVSSVWDNLKKRANVRLQTTTPVSALREATQPDENTRDSHPTFGETDET

NDMSNKIKIKRTYEFAGQQHTEEKYVLKNSAEAKEYLHSLKFNSSLESSSASDLNKDDKS

KGETKERQNGKISGLRRPLKRPPILEKIIAGSLKPKLSTLEKSKMDWAGFVDNEGIYDEL

QQNNKAGYMQRQEFLHRVDKHKDVQYKKMRQKELAEKFKSK

>model.g255.t1 Augustusgene.g255.t1 JFAV02000093.1:47037-49055(-)

MENRDSVNSKDFCHKHTKYANKLKSLEYNEDGHFFSNQPCKEELNLKVENGSFLNVSKTN

LPALVVSECTSTDNQAYLQPYYTPTFPSHNLPPPPRYRQSHSVPAPVIPNYRHHQHFDNF

SRGKSLGESVLNVGKHKFEQYSSPVNTHLVQPSSTISNNTPIQHQVSLPPITTLFRDIQM

NEARRSWYSSCSSSVSDLQLYSPHTIDSSEHSKPVGNNDRHESLENPSGDNKQAPIDSSA

PLYIQQTAQIEPERISMKNNKLSSSTSENSVFQKINAGKISNAHSPTDEDSTSDTNEQNK

LKKLKQGRPITLDISVNLLFYESLADYLTFLIQKSTNHTFSSKKQHNRSKENLNNMQNNH

ATSKSSLPMSKTKQSFLQENIEGDAKSISISVDSSIIRFDENSTSKSMLINKQHYLNLPN

IEASSSGQSITSHVKVRPIRKPLLILVQNQSDLVLSHIKAALNLEPLDGICLVRNYSRST

SPNRASSSDAPDENHDALYRDGQKQARKDRPTGTNDMNHPVTASNGKESSHYIKFDYIPL

ANEAVIDIEFFKKHLCYPKYRTNLKLVLIKKTPKFPNTLRVYLKSNLSYAEAASSSKKEN

ISANFKKSTTDQSKDDIGNKKEKELIKEHHQSYTLEEFKRDKSHKLMSAMAKHSEKELFL

LEIWKINKYVLES

>model.g266.t1 Augustusgene.g266.t1 JFAV02000093.1:73791-76976(+)

MEDYLQRIKPFHNGLPTANPEARQSRYLLDKELIKKHKTVGIHQISMTVKRFEHLLEISN

LFRHFLLNKVEQKDEKYVKVYEILKKNSPQLLSSHHANGKDKSAIHRGRMSEKDEDKQLM

KGEEQSTALNETLAQDEEANEIDEEEIQFTESPLYINGSLRPYQIQGLNWLVSLHRNNLS

GILADEMGLGKTLQTISFLGYVRYLEKKPGPFLVIAPKSTLNNWLREFNKWTPEVNAFVL

QGDKEERAKLVSERLLKCDFDVVIASYEIVIKEKAHFRKLDWEYIVIDEAHRIKNEESLL

SQVIREFTSRNRLLITGTPLQNNLHELWALLNFLLPDIFADSSTFDQWFGGSSDESEASD

KEKEEKDSVVKQLHAILQPFLLRRIKSEVETSLLPKIELDVYAGMSAMQKQWYRKILERD

IDAINGETGKKESKTRLLNIMMQLRKCCNHPYLFDGAEPGPPFTTDQHLIDNSAKLKILD

KLLDRFKEQGSRVLIFSQMSRVLDILEDYCYFKNFGYCRIDGSTDHEDRIAAIDEYNAPG

SEKFVFLLTTRAGGLGINLTSADIVVLYDSDWNPQADLQAMDRAHRIGQKKQVKVFRFVT

DKSVEVKILEKAKQKLRLDQLVIQQGRTSIEKDKKLKGKNDGKDELLSMIQHGAKDLFEK

NGKSNDEDDEFDLEKLLQDSEQKTKELNAKYSKLGLDDLQKFSNQSGGDTYEWDGESFKK

TNVSDKVIDPLSLLNGNGEGGRRDRERNANYSIDEYYRDVLNPKSAAKPAAQLRMAKPPY

FPAHQFLPIKLKDLMEKDRLYNAKVNGVTPTMDDVKLTFGELSDDKEENKKKLEIFKLSI

ETAEPLTDKEVREREDLKLQGFTNWTKNEYRKFLTASGRHGRTAIREITREIGTNKTEDD

VYEYSKAFWANITLVDNYERVLKNIEAEEDKLNKLAVQQEALRRKVSQYRDPLRQMKVKY

VSASSTYSLEHDRFLITMMLKHGLGNNNLFDLIKFEIRKSPLFHTDFFFQNRSSTDINKR

CQVLLGYIEKEMNTAIPIDAKLKKRLSDEDAAYGRNQKKAKK

>model.g264.t1 Augustusgene.g264.t1 JFAV02000093.1:71336-71992(+)

MTTTTWKPEDHIPEEVQESFKNLSSSPSAYVISLLSVVEQLKIQRRTGWVDHNVSPCESI

SDHMYRMGVAAMLLKTPGIDKAKCTQIALVHDIAESLVGDITPFDTVDKEEKHRRELATI

EYLCEKLIKPYNEVAAQEMYENWLAYENISSIEARFVKDLDKFEMLCQCFEYEKRSKGAV

DFTSFWNAAASIKTEEVTQWKDELYKQREAFFKSLKEGK

>model.g267.t1 Augustusgene.g267.t1 JFAV02000093.1:77385-77870(-)

MSKFYELSAIDNAGHEYSFKQLEGKVVLIFNSASKCGFTGQLDGLEKLHEKYSEKGLVII

GFPCNQFGNQEPGDDTQIQSFCSMNYGVKFQMMKKIDVNGSNESPVYHYLKHEKSGLLGF

KGIKWNFEKFLINRKGEVVSRHSSMTKPESLEKDIVALLDAK

>model.g268.t1 Augustusgene.g268.t1 JFAV02000093.1:78382-79455(-)

MKPFISGHEDIVHDVGYDFYGRHLATCSSDQHVKVFKLDKETNEWLLSDSWKAHDSSIVS

LDWAPPEYGRLIVTCSYDKTVKIWEEDPEAPENSGKRWNNVATLNDAKGPLFCCKFAPGH

LGLRVGCIGNDAVLRVYDALEPSDLRSWSLTSEVKVLSEVPVSHLQSDYCFTWCPSKFYQ

ETIAVCALDKAVIYQRNKKDQLYEAGELKGHSGLIREISWAPSIGRMYHLVATACQDGNV

RIFKISSYQSSDLHKNSINSSQDETSNKSDSSLLVELVSENDDHKAEVWSVSWNLTGTVL

SSAGDDGKVRLWKSSYANEFQCMSVISPQEQQKLQQQPQQPQQQLSQEQEGQNSSELP

>model.g251.t1 Augustusgene.g251.t1 JFAV02000093.1:39312-40940(-)

MTKIKRKGELANKHHKMDIEIKLPAKATETLNGFGSGNYDVLTNGALPLVFPELLHMEET

DTGRTTSGSSDLSNDTEEDYTRNETKNLNPEYSNLHLSSLDGADKRSSLSTSGITPSLIK

KRKNIETDEAEISLEAEIKRVALEDTTHSDSTQSYEKKYKLMKTNTMLSKKQLDILEPKK

EEESFTMRLLCHVKEAALIIGPKGSFINKLKTATKTHVIISENIEGVQERVITVKGTLCD

VAHCASIIARKLLFPHCDFSELSCVSESDFDKCGLKWLVPHELVGFVIGKGGNTLKRLEL

ANSSQISVSSHSLPSSNERLVTLSSTTYLDTAIIDIAKILIENKNRVSFKNSYYNPTSRS

AHHQEMTQDQRYGFPHNHGNKDSSSVTGTHSITPHVRQSEQANHKKDFPFEFPPFTLNGP

GPLMPNPGLQNGVSALPPSSVPLTGAPIPNTYSVFPFIKLAPYSSAPLGIIHQDCFILDV

YVGKIIGKGGKNINFVKKNSGAEILIDQSPQQGERKFTIIGTPMSTQIALAILNRKIESI

LRG

>model.g260.t1 Augustusgene.g260.t1 JFAV02000093.1:62063-63181(+)

MADLVSKFNALDIKQATDLSNEQRAIKAQWESVVQSGSITEHFQDLNLQLRDQTFILPGT

TELEEVDVKTLEVVLPILKDLLASSKDITNNVATYRHIVRWALFILNKLNVEDEILSGMN

LDLDLPKEIIEKKKKEDGKDKKAGAAAPAAAAENKKDSKKDGQQPRGKPDEEALKKLREE

AAAKKAAKKAAKAQAQPQQQEAVKPVVSAIDFRVGFIQKCVPHENADSLYVSTIDCGDKE

GPRTVCSGLVKYFPLEAMQERYVVVVCNLKPVNMRGIKSSAMVLCGSDEEKVEFVQPPEG

SKPGDKVFFEGYSDQEPLNQLNPKKKIWEAFQPNFTTNENLEVVYKDEDGVKKLTSKLGQ

PFKVASIVNANVR

>model.g247.t1 Augustusgene.g247.t1 JFAV02000093.1:32573-33709(+)

MSKDVVNVFKLVKTPIYSHCFNHDNSLLAITTENKVLIFNVSQPSKPQLITTLADHDKTV

TAVDISIHGRIVTCSQDRNAIVWEPMSDGTYKPTLVLLRINRAATSAKWAPNGFKFACGS

SARIVAVCYYEHENDWWVSKHIKSEIKSSITSLDWHENGVILACGGTEGIVNVYSGFIKG

LDKKEQVLNSPWGEGKYPFGLLLKQFYEGSWIHGIKFNSALDKLAFVSHDARLTIADAQN

NTTTASNPSGLPLNDIVWVSDSKLIGAGFDCHPVEFSYDPNASAVKFTKNIDKIVQSGAA

RNISLDNFDNEDDDQENGQQTFGISALRKFKDMDLKGKVSVVDNELGTTHQNAITQLRVL

NNSTVSSSGLDGKVVIFNV

>model.g246.t1 Augustusgene.g246.t1 JFAV02000093.1:30423-31886(-)

MKAVVSTDDGSVKQFVFNKGTDTSVLESLKPFYSETLFRKPTEKKVVKIHILDSSTSNLD

QITGKKIVLFQNDGSIELRAVENTKFDNESFIESSDVASANKSKENEKDTAKKTPAFAQP

IPQAFDILKLADAKPLDVITGMFDATRLEEVQKKSKKRSHIHDEFVSVQTLNHTENETFC

IGTKSGLIHIIQVENDKFKFIASHEVRAPMDFLQYNDVTKNTTSESSHVLAYGGEENLIK

LIQISSDLKSMDQVWEAKNVKNDKLDLKVPIWPMSVVFTPTNDTQDETKLNLEFLEITKT

GFIRHYKTQHGRKPLDSIDIIHDRPSQRQQWPSILRAQLFQSNITPNGNSTSNFDDTSRI

VLADSSKNLLQIDTIKGSLKGKYGHRDIYGVTSCIRNFENKYLVCGGIDGYLRIYDAHTR

KRLAKVYCKHKVLECIIEDDAEVDTVESLKKAKKLLKKRKLTQEEEDQENEKLWNNLESQ

GSSKKVKK

>model.g254.t1 Augustusgene.g254.t1 JFAV02000093.1:45633-46640(-)

MTTVQNPISQSTGDVTHTGQNPLEIEQKLAIQSICRFLKSKTSYDVLPVSYRMIVLDTQL

QIKKALNILLQNNIVSAPLWDSQNSKFAGLLTSNDFINVIQYYFSNPDTNFDLIDKLKIT

GLSALEQHLGVPSALDDVSIHPFQSLYEACCKMIESRSRRISLIDKDEETDRQIVVSVLT

QYRILKFVSLNCRETRFLKRRIHEINIVATDLLHCYMSTPVIDVIQILSSKSISSVPILD

ENTGELINVYEAVDVLGLIKGGIYNDLSLSVGEALLRRGDDFEGVYTCQDTDKLATLLDT

IRTSRVHRFFVVDSKGFLKGIITLSDILNYILFGEE

>model.g269.t1 Augustusgene.g269.t1 JFAV02000093.1:79792-80907(+)

MSDLETETATLSTIKTLFPPCCLQILPDNEHILVGTYELQKESGNRIGTIDLYRFDARSN

TLELVYRFSENVSAILDLKLNGLDKFVTAHSTGELRLWKYSFKGDAFELELIQKFQVFES

DVLVTSCHIRNSFGSSKNEGNEVLCTGTNGELRLVDLLHYTESKVSEGAYDFDSAHSLEC

WTAEFGKLAPLENCILSGGDDSVVKLFDTRTKNEVWSNSRVHEAGVVAIKCASPTFRTNH

PTSILTGSYDDNIRSFDLRMLSPTVVYPGTMPPTNVKSKNLGGGVWRFVENPSHAQSREV

DELLVCCMYNGAKVISVFNENEINDEESEESIFQEQNYVKKGHDSMCYGGDWSESFIATC

SFYDNSLQIWRP

>model.g236.t1 Augustusgene.g236.t1 JFAV02000093.1:5409-7133(+)

MVKVLYGELKWGFIPVKRIVEDYPEDIVKTVETDESSEYSNVEKKELDQQTSSAASAPVA

EKSGAETTETGEGANIIEYEYRDEAERKWWKFFDEQEYTIPAHIKNKKRQWNWFEPNTNG

AEKKLLVKLDILLAFYSCMAYWVKYLDTVNLNNAFVSGMQESLNMTKNDLTNTQNMFSIG

NTIFQIPFLFVLFKCPLNYTLPILDIVWSLFTVGACKSQTLGQLQACRFFVGAAEAPAYF

AYQYLFGSFYQSTELVRRSTFYYFGQYIGVLSSGALQGALYKYGTATFEGWRVAFLVDAM

ISLGVGFLGFYMIPGNDPYNIYSLFLSDDEIRLLRKRTKRNVFKASDNLLDWKVWLRLFS

QPKIYVLSLFNAFLWCCSNASSGSYLLWLQSLKEYSVVKLNQLSMTSAGIGLVLLAVFGL

YADLFNSKYQAIILSQCVNFTGNVILSCYSRASKGAKYFAFNIAYSAWAAAPTGYMYMAI

MSRDSEERSKILVVANIFAQTMSIIGNKFAFNAKDAPSYKHGFPFAAAMAVCLSLCSLVV

LFMYKRQERALAKSNGIILYNSKTGENYPLDKEEA

>model.g248.t1 Augustusgene.g248.t1 JFAV02000093.1:33863-34132(-)

MDQDDSLTALQETVLGKYRLLAEELHTLDETIKRIINNDHKDENGNTLHCSPQELLQLVR

ELEVKMGIAGTLIKGSVYSLILQRAQQEQK

>model.g257.t1 Augustusgene.g257.t1 JFAV02000093.1:55723-56637(-)

MILGDIETKVLSGASTNEKIITEKKACVCSQLDSEFETKNLCCIYSTQETELSQENHNIL

NQLIEQLSNKLRQSCLEYADLLLEDLMYPKGVENSVPLFSTSDSGILNENYSYLVSDLGN

TTTLEVLHIKKLLKKEYSRDCAAELKQMEKLHVCFVVLLRNLSAYLEIPNAFHFFKELLK

KNMINFERLFKLFKKYKQIITILLDCAFSHDSNLSSSGNEEEKSKSGNAEDCDIFQIKEQ

DLQLIQDFSLENSKWLEEMLHGSKAYKEYLQQNSQVSKKLKYDDIEFGSFLKNRKNALRI

SSRTL

>model.g242.t1 Augustusgene.g242.t1 JFAV02000093.1:20800-23175(-)

MSTEEQKKNALLEERRAKLAAWKKQREKKLAQQQETAQQPPLNVERPQQSVTSITLEKKT

DKRKLKRKKKITFNENDEPTLKPTEHEKVEPSIKNDHLLLSNNEDPLDAFMSTLSPINDQ

LHTKERFLLSSDSESISSNESSEGGESTGAFKQSLRKKKKKRKIVSRLRDSDLVPFEKNF

YHEPEELKNMTDEEVADLRLSLDNICVEGSGVIPKPITKWSFLGLSSDIMNVITLDLNFS

TPTPIQAQAIPCIMSGRDVLGISKTGSGKTVTYLLPLLRQIKQQPKLKKTETGPIGLILS

PTRELAFQIYEEVLKFTPPEVTSLVCTGGGELKEQISTIKKGVHIVVATPGRFIDLVTVN

SGNLLKTNRISMVILDESDRLFDMGFGPQVRNIMRGIRKDRQCVLFSATFPESVQKFATM

FLDDPVSITINSRDVVNENIHQKCSVFSDQRAKFEGLLKILDSSSNASSKTVIFLSSQEH

CDALFEHLLNEGLDSEYKLFAIHAGKSSAERMDGLQEFKVTKNKALLLGTEILSRGLNVP

EIELVIIYDAIKAFPQYVHSIGRTARTSSSTGVAHTLLLDDDCDLAASYVLFKSMRQHEL

DEMVPSELAALTAMYKKFDAGLKTGKYKIILGLGGKGLDNMSASLANTMKKAKSKYAAEV

GSGDEEATDNESGDDNVQPFEYKVDPVYDGEKPVSYKCQIVINDYPQTVRWEMTKQTTLM

EVKRETGTSTTTKGKFYANNGGPLFSGDEPKLYLLLENKTEEDLKSALDIIQKACTHGYK

LLESNESKKFSI

>model.g253.t1 Augustusgene.g253.t1 JFAV02000093.1:42464-44935(-)

MQTLSDQDMPLQDMSSTTPASSLNASTAGIFGASSSKNYSNTTLGRTPANSQGLEGSTSK

NSDPGLIDWNEKPYLKQVTLRGTIVGLFIGSLVLISNFQFGLQTGWVSMMSLGSALLACA

IFKQVMPHIVPKSMNQTPFTDVENVYIQSIAVAVGTGPLCFGFVGVVPAIEKFMTVEEAG

GIDFKGSISLFKLLVWSTSLAFFGVFFAVPLRKQVIIKEKLPFPSGSATATLIGVLNNSE

IIHEVSRKELNRLREYSEVLETEMANESTAHDHYHHQHLSENEASESAFEGKSTNKLSKV

YYENIKILAKTFAVSSIYTIGSYFLPILKELPVFGNYLSKNYLWNFQPSPAYIGQGIIMG

LPTTSYMMFGAILGWAILGPLCKKLRLVDPDADVHDWQNGIQGWVLWCSLTVMVVDSCIG

FIVVTVKSLIKYYYENKRKPGINLERVQVPLLTASDNAQEDAESRADDYGSLSDGSDVLD

DDLNEDGRNNVTSPRALVASESQDDLNNNKPVKMNRSGSSVMYVSKDFDHDVKDPSLLVG

KVTVISGIIISAFVCILSMIYIYGMAIVPIYSVIWALILALFLSILAVRALGETDLNPVS

GIGKLSQLIFAVITPKERGGAVLLNLVAGAIAEAGAQQAGDLMQDLKTGHLLGASPKAQF

IAQMIGASWSIVLSSVMYKVYNKVYTIPSANFRVPTAVIWIDCARLVMGDGLPKGAGTAC

LVLGIIFGLISFVKNVYRDHPNLSKPFSKFLVILPSGVAVGVGIYNSPSFTIARFIGGLA

AFLYVKRQHANRTKLIVFSSGLVLGEGICSIFNMVFSSLNVPHM

>model.g258.t1 Augustusgene.g258.t1 JFAV02000093.1:57476-57706(-)

MSPLQKWIGIAKKEARNMRRAAKTIANEILQPVEQQAQPQLQRVPIPVHNNNGQSGLPFP

VNGGSLNGNNNGGSTFN

>model.g244.t1 Augustusgene.g244.t1 JFAV02000093.1:24737-27970(-)

MESTFYQLPGSHKPSNSSEATYSTPLLTKKGVQAQSESSFSYFTGNAAGNDMMQRKISSK

FDPENPHKHKLGTYDGVFVPTTLNVLSILMFLRFGFIIGQMGILGTLALLVVSYLIDLLT

TMSVSAIATNGTVLGGGAYTLISRCLGPEFGGSIGIIFFIGQILNSGMNVVGIIEPIMYN

FNSQSGVIAPVLPPGRWNEFMYSTILLIMCFGVCLIGSQTVSKVGNVLFWLLLMATVSIP

ISALCMPAFSSPIEYTGFSMTTLRENLLPHFTSGAAGSLLEKKETLNDLFGIFFPATAGI

FAGAGMSSELRKPSRSIPKGTLWGLLLTFSCYFLVIISLGSTVPRSSMHKDVSIIQTVSG

CQPLILIGEMATSLFSVIVGIIGAAYVLEAIAKDQILPGLDCFHKHPNLALFVSWFLTQL

CLFSDVNQIATFITMTFLMTFIVTNVACFLLEISSAPNFRPSFKYFNRFTASMGGLLSIV

AMFVVDGFSALGVMFSLIALCLLIHYTCPPKQWGDVSQNLIYHQVRKYLLRLKQDNIKYW

RPQILLLVDSPRSSWNLIKFCNHMKKGGLYILGHVITVKKAHQFQKNYNELQLQKTSWEK

VRDYLQIKAFVHIGLGQSLQWGVRNVFLGSGLGGMRPNITVLGLMESEKKSEKYLKNGEI

PAFLPTDNCKNESRITKQQWVQLVEDLSLMKSNIAIAKGFYDLEIPLKNHTPKQKRYIDL

YPIQMTANLTNGKVSTKTTNFDTYTLILQLGAILTTVPAWHKSHILRCIVFVENEFEKID

EYKRITQLLSVLRIEARVHVLVLSQFKVYNTIIKGDPIALKYVNQQLKDDAWWADIVKAR

KDTNFSSRRFSYSDYNNKYRIDKKLKPKSTVSELQRFGVSLTMSSNIPVANDMFLFDETV

NGLDESADETDLESSTPSVSTAQRRPKTVKKEDKPNIIPVFSSDAIPQTRVIEEATGVEP

SLIPVENPYVPIKKHIKPQLSPCQSMDNLHLKELDFNSIPRKAQLLILNDMMTQLSGHKL

KEQCNTNLILSTLPIPMLGTHMDEKASEDYINDLNLWLDELPPTLLINSQSMTVTTAL

>model.g238.t1 Augustusgene.g238.t1 JFAV02000093.1:12304-14238(+)

MSNLNDEPKRKRKNTKRACTNCAKAHATCDSNRPCSRCIQKNLSDSCVDKARKKSKYLQD

IPDEELAKTLRSAGSSFTLQNDLHQRSGVMNNAFQQQQGFPQSGAANALNMQFAAQVQSL

SSQQLNDTSSQPNIRHTNFHEQQFIATPQHIIHKPVFQSSAIDSEYSSLSNILSKNADSM

LNKIPVNILNNRSSSELIASLDTTSPGSSVGSAMGPGNMYGGNMSHNSNHYIQQNNNNNN

NNGSAMSSGHNSYSNFHHINAMNAPQNPAMAAMPGAPPNNSFYNSMNTQGSIYSPSYKNM

SENLVRYNLEEDKYVERANICKYLGPQGAEVLSNLEINLLEQHFPLVPLDVAPEADSHRM

RFSASLSSTDTQIVEEDHLTIKSKRSESSSSKNDHQNSKCHQTDLVFCGLKKNKFVVFNL

SKDDDPLHTCAYSLQVSPENKIYSNTRWPHSLQYDTPRDIYTKIQEPFFQTQSFVALLEH

VKSRFKKEDVLAMCRDLSAIRPILIAGCIDLKEEDMIFMEHSYQRTLLQYVSFINQVGTP

TIIWRRSGLISYVNDEFCVLTGWSREVLLGKMTFILEILSDECCLEYFHNFKSICYSDYK

GSKSALTLTVQSPVRGKLIECVTWWTLKRDVFGLPLMIIANFLPK

>model.g249.t1 Augustusgene.g249.t1 JFAV02000093.1:34486-36894(+)

MAKPKQAQTAANSTITQANNSSFTVWSPQDTVRDVAESLGIGNISDDVLRSLAMDVEYRI

LEIIEQAVKFKRHSKRETLTSDDIAKSLKVLNVEPLYGYAPGIGVQNFTKTQVGVGPQQQ

NLYCLNDEEVDFDELINEPLPPAPRIPSFTAHWLAVEGVQPAIPQNPPLTEIRHNYPPFV

RGAIVTSINENSLQTVVPEEDLNEEITEKSGALNGKQQDSSVAVQQSQSSQAGSKIAKSI

IKPGSKELEVKPRVKHVLSRELQIYFEKVCEALLDQEITDAKTRVKTAALHSLKNDTGLH

QLVPYFISFISEQITYNLDNLALLATILEMMYSLLCNESIFLDPYIHTLMPPIVTLLLAK

NIGNENNTLEQTLGLRDFASELLNHVLHKFPKLDKTLKPRLTRTLLKSFLDTNRTMGTYY

GCFRGVMVLGPETVRFFMGNLHNWAELVIPSLQSSVDSPKTEPLLELPEKEAQKNYVSDN

NDDLPTKEAQDQDVNMTSPPTEQGETADQDVNITLPSTEDAAVEKVQKDENENNNVVSEE

RKNETVGEELPEADEPPLEDNNAENKIAVSSKENAVNETSDEKTDVINSKEKVGGVTGED

AEQQENAKQSDEMEPKSENSGSQQQSETPSHIANDTEKSVVEANSNTTDQQVANDVEMAD

ASSSQGVPSETKIADTNARPTDASLPEPTTENPVPESSSDVISQENKKPEDTVTVSSVEK

MSSKFTNEEFDHLVKIIIEFLKTLEQELPPMEQSGSEPLSVEEFTNLQNRVGQVVAQRIK

SFSDHSEARKLYKAIFFGTLGDA

>model.g237.t1 Augustusgene.g237.t1 JFAV02000093.1:7510-11496(+)

MSTHDPPEKPKKASHHLRKELNWLKKTQAEKPNQELLLNTFKFSKNVDLRQEPNASIGVS

FPTPSVSSTSLNTARSTVVQSKPVSSSSSPAVSVEPVVPANSRAFERPSVTSSVRTINST

NPLISTSRNATSKSDPTPSINSSKPRPFVNLTRPPARNVVDFFSQNKSIPQTLRFNAREH

IEPPLGPAAPRNLNSNSSVNVSKAKTIESDTSSAQVNLPNMQQSNQSEQFQSRNTTAERS

KLFNDRISNFVYDGETSSSLSEHLANRSDNGKVISSSVNHSAGNNSVITILTPLQQGTSE

KPNPLVSSSSGFIDLTDDMPTKKRKISEVSMIAKSTDTGNTEPSPPEKDIIKDKLIAALL

EQNELLLKKCSILQSTSLSEDHKRKRIKYDVDPLIETQSLDISLLQKQVALSDTQKESFL

PSTVQSPALQISSLSKSASLHPKDLSSEKNNTNFNNHIEDSFAESESDLEELVNQYKSAP

NIPQSSTGTFDPISQGPLSSAPYNFEQPLLQSNAGVNKRTDLSDITDEAEYEDSNYFNSG

IEEDTRSSDRGFIDDDGHTTNFMDEDASYKDEAETSPDQLYRNDNHGSLVEIIDSSPLKK

YPHHEQQQSDTEMYLSENDFELLMQKNNPTENASDDDNVSDIDDLMELENDVFDMEREHF

TQNIKDVDDDLKIISETKLTDEQLDHAAEIAVKREAQTLKQQPPQKSSSSDLGFDEEDDD

EDILNMAQPRYPWTAEVNKQLHQVFKLSSFRSNQLDAINGILSGRDVFVLMPTGGGKSLC

YQLPAVVKRGNLHLTTIVISPLISLMQDQVEHLQKKNIRAGMISSKNTSEDRRHMFNLFI

DGLLDLVYLSPEMVSASQQCKRAIDKLYCEQKLARIVVDEAHCVSNWGHDFRPDYKELKW

FKEKYPTVPIMALTATASEQVRLDIVHNLKLKDPLLLKQSFNRSNLIYKVATKNKNTIFE

MAKEIKIKYKNMTGIIYCNSKNQCEKTSQFLSTQGISCSYYHAGMENDERSNVQRQWQNG

QLKVVCATVAFGMGIDKPDVRFVYHFTLPRSLEGYYQECGRGGRDGKTSYCTLYFNYADF

KSIESLLKRDKDLSKDNKEKHLAKLNDVLQYCNNTIECRRKLVLTYFNETFDPKACRKTC

DNCRANNLVEQRDITKESKDIIALVGALDNQQVTATYCMDVFRGSKNSKIVNAGHDNIPQ

HGLGKAFRKDDLQRIFQHLISAKYLGEYTKFNKSGFATTYMKRGTRVLRDKVVMNFALSR

PSSSSGSNSSRRPPSAAVALDKEEQKAMQQTYKKIASQSQANSYGNKRKKGYRGGKSSKR

YYKKRSRTG

>model.g265.t1 Augustusgene.g265.t1 JFAV02000093.1:72095-73342(-)

MQGIVAPLSVSVVSSALGYAATKWLIPRVSDSFIKIGLFGKDLSKVGKPVIPETIGAIPS

VIFILLMFINIPFFFFKDLTDSTLFPYGKFSEFLSAILCLESILLLGIADDLFDLRWRHK

FFLPAFAAIPLLVVYYVDFGGTTVMIPPFIQNFFELPHNYLDLKGLYYCYMASVAIFCPN

SINILAGVNGLEVGQVVVLCVIFLLNDFLFLFGAVEPAKHSHILSSTLIIQLLGCSLALY

HYNKFPARVFVGDTFCYFAGMVFAVCGILGHFSKTTLLFFIPQIFNFAYSAPQLVKLVPC

PRHRMPKFNEKDGLLHTSRAVLGKSTKDHRFDFVLRLLYKVGLIDLEFEANDEKTIVSCS

NMTLINLVLVWSGPLREDKLCQRILILQLAIGILAIVGRHLGGAMLFGHDNLFSVI

>model.g235.t1 Augustusgene.g235.t1 JFAV02000093.1:156-3263(+)

MLRRSNKLFSLKHIRLNTTLASVDSISYAKVHSPSSNTLQNLDTFQRRHLGPNVSNVSEM

LKTMKYDDLQKFVESVVPPQILTRRPLKLHNVPPQGFTESEMLQSLKKIASQNKYTVRNL

IGKGYYGTHLPPVIQRNLLESPEWYTSYTPYQPEISQGRLESLLNFQTVISDMTGLPVAN

ASLLDEGTAAGEALLLSFYQHKKKKTTYIIDSNLHEQTKSVLQTRAEPFGIKLVEVNFIE

KSAEEYSSILNNKDVFGCIVQYPGTNGEILSQDKLSKIGELVHANKGLFSVASDLMALAL

LPPPSTFGADICLGTSQRFGFPTGFGGPHAAFFSVVEKLNRKIPGRIVGVSKDRLGGKAY

RLALQTREQHIKRDKATSNICTAQALSANVAAMYCVYHGPEGIKSIAQRIYGMTTLLAQQ

IQNFSGHKLLNKNWFDTLTIELQAGIDSDSFLELALTKYNTNIFKVDNNTVSLSLDETVE

EKDLINMIELFTGLKKTSVDIAQTGLPVFSNARTEGFLQSPVFNTHHTETAMLRYMHLLQ

SKDLSLANSMIPLGSCTMKLNATVEMMPLTWPEFTNIHPFQPVDQIEGYRQLITSLEKDL

ANITGFHSVSLQPTSGASGEYTGLRVIMAYLKSQGQYNRNICLIPVSAHGTNPASAAMCG

MKVVPVKCLENGSLDLQDLEAKAQQYKDNLASIMITYPSTYGLFEPGVKDAIDIVHKYGG

QVYLDGANMNAQVGLTSPGDLGADVCHLNLHKTFAIAHGGGGAGSGPIGVAEHLTNFLPR

HDVEPIFDDLVESKEVVQAKKDNAIKAVSSVAYGNAPLLTISYAYIKMMGSLGLPYASII

AMLNANYMMSKLQSHYPIMFIGSSNEKTSSDVATSTADTIKHCAHEFIIDLRDFKKYGLE

AIDVAKRLQDYGFHGPTLAFPVPGTLMVEPTESENKEELDRFVDAMISIKQEIQYLIDGD

SRGNVLKNAPHSLQDLILSKDWETTRGYTRELAAYPLPYLKTNKCWPQVTRLDDTYGDLN

LLCTCPSVEEVVQHQE

>model.g245.t1 Augustusgene.g245.t1 JFAV02000093.1:28501-30363(+)

MSKVLEINSNPEESLVSLPIVTFPPFKLRAALVEKDPVVWVHLLEAYNMFMAHLIQHREL

MRLNEKTWEQLCIFVRSFLSETSSEMGQLLSLGSNVDVTRNLEHLKRWVLKLVEIGGGAH

KLQMNASTLWDFVRVYVETDANTVRKIISEGGKIDCTYMLHNHLEFLVGSGKFDRYDLKT

FESLIDRKTTTRSSNQSVLAKNNAENSKVHTRISQRTKKYDSNKFIDKFVTVKWIEILEG

LYNHGEGVHSKTCKQLMIVTLLNCSTQNISSLATQLGISNLPALKLYPLFGGVLWVGFTE

KSHFKGLETRLPFLKASVINTNAQSSGELSTYDSRNKQSQHSVDIETVLQVQEMFPELPV

SKIQQLCPRFDSAEAFIDALFENPTFVEDLEAKNTDFLKETMGLNEFKNKLNISKKKTSD

RIYDRGVPDEVRNKTMAAALKLLYDPNEDERDDTYDDAERQQPGNGAADNNKETEKYNKT

EEYLWGLLKTDAQLFAKTSRKSKSRKELKDKTKWSDEQIEGWARMIERSPARALRLEEKY

MFQNPNRKPIIKEEEEEEAEKDSTNMTPADKRKNKDKGRDKKNGTTNDAIQSESSNDSKR

KNKTAKRGHNRRGQYSGSNSR

>model.g240.t1 Augustusgene.g240.t1 JFAV02000093.1:16730-19213(+)

MLRFVQGPSNTFLKQRVSNNIVHQASKNYLHPGNTSSAAAASQQPTFIPQSIHLDRSLSQ

ERQYTTGASTTTGTTTSNNSTNTYQHNPAFNNNKTNSYSQNYNYNKNKKFSKNENKKNQL

KSTHMQKHLNFTKIDTESPLYEQFQQFDKYITQSLQISTQQSSSLDHKGRTKQSFQFQKN

PLYWDSVTQSMRLYREINASQEFGPERLSSLIHLLHNGLRINRFQLARMSKKPDYDSSSF

HKQITNFIYESLKEISDDILINDMKINEFGLMHLITCFKELLLEEEAVEVWNKGLQKINA

PGITPEQVHQIKNIYLNSKVVGTVLPLLNNQGLPFEESYKLYKESMSLSSPTQYSNLILG

MLLVCLRMGANKKALDLFSDLFSIAQKNHFGYLIEAHLAFIGECKDLNVANDFFQKAILG

DLPYKVDLQVSFVKKFMNNTWTLTRDFSKVYDIWLQASKFYGSYVHHGISSSLNDEFFNI

FFEAFPGQARAETSEANTATSDASVYTSRANSLNSATSTREQLLEQGFQQLQDIIVTYNS

IKQIDEPFFNIILSKASAQWRDAKILEFIDQSYQIYNTKKTIIAYRISLKSLGFINNVTQ

QAIWKKWDELLAKIDETGQSYIANADWAALRDSTLTWANENILKLNSLKQRNLNEKLITP

GVTSQDVTTGDMSYTGNNAAATSTASSGATTPTYTEEYNPALQALKESGAFEMETSDDEL

SSKMQEDDAAERSFEFISGSATPVSHMEQEIAQLETDIRMRIDTYWDVFAQYSKYCRDLN

QLKKLSNGYVKKFGALDPYLTSSKNMFETKHREEPVNTVFVNIKPFNE

>model.g262.t1 Augustusgene.g262.t1 JFAV02000093.1:66974-68587(-)

MSENTPLLGVPPVQAASDMPDITNNTNNVVFTNHGHRSQSFSKRHNLTWRLGLFIMITCM

GSMQFGYHMAELNAPQRYLTCDYMHGDISKLSSLPTTPRLKTCVKMTDQQYGVITAIFSI

GGLVGSFYAGRFADYMGRKNLSIINAIMSLTGSLVLSLSNSYWQFIVGRLIVGMAAGSSI

VVVPLMITEIAPVSMKGIFGSMNQGSINIGILVVQFFSLFWAKPKKWRYLFYAGIVIAAI

QMMLLVLFFEESPKWLYSRGELRKAERILLGLRPGSRTEVKREIDEWGHQSQSGNSNQAS

GSVGGGIGGGIGATSTLRNTRDVTFWEYLTGSRYFKTRLLVTAILVGQQFTGINSIILYG

VSVVGNLAKSSRTPIKVNFGISILNVVVTIGASPLIDHFGRKPLLISSSILMGIAAYMVS

ISLHFEKLIFLILMIFAYIGFFALGVGPIPFLIIPELSPSNALGAAQSYGTVCNWIATFI

VAYGFPVLSNMIGISVVFAMFATFAILFAIYLFFTLPETRGKSTYNEVWGIDSGTYEA

>model.g259.t1 Augustusgene.g259.t1 JFAV02000093.1:58125-61406(-)

MSPVKEESEINEVSPELIEQNCDLCRKRKLKCSRELPQCKSCIKFNKKCVYSPKARRSPL

TRSRLTALEKKLFKLEKIMKRAFPNMSIEEIAEKVEQKDQLDIAESLISLQSAPSPSVST

LVKNGEQQPPLNADNAKAQVSSAIKNETDQSQSKKIPARTTDPTLPNMSQESQMIDRINE

QASKSKKMTRDKKNDSGNQKIVPTDTPNDPLFGFDWTEVDEDQKFVQNDGMAALTTDPNN

KGYFGQGSSAILLRAALKNNDSLTQNKKKIEDLIVTTELEQQLGSLEIREMFVNAYFEHY

HTSYPFLDKEFFMMKFKEYEGADIDLQPHSADPDDAHTTERYLDWKILLNTGCAIGCWCL

HGESLLNIDLFYYKEVKKCFKGTLFERGNILLVVSFTLLSNYAQKRNKPNSGWNYLGLAV

RMAIGLGLYKEFDWHVILGNSKYTHELEVRRRLWWGIYIFDAGVAITFGRPINLPNNFIY

ELALPSNIDDYSGQEVETKPEGFPTKREKGEKSLPKPPQVVDYPTIYSGLIEQTKFTKIS

VEIHNRLLARPSPSAKECLQLNEKIKSFVENLPSWFSKNDNTAYSCCEQLKKNSNNNEIP

HWFHLTRYRLIWRYLNLQIILFRGFVWQNILGKNDANFKKFFENPTTSECIEICLDSSGQ

SIEYVAKYIEICKTPTNVSKDVYFGSDPRQTSPNANGLKNETEQYSELSVLGSWYATYFL

FQATLVPVICLVYYDARYKNFESWKKQILVARKLLSILQVKNKTAGDFIKIIDSVCGNIL

SGKSVSKNFKANEPISKEELSKTGLSKNSKATTNKTKKVAKATGSSKKESSMKEKVLKKN

LEKTGNAGNINFNSPDIEKFLDETMPIDSASWENTMDGSNTNLNSLLSLFNVPTPGMPNV

NTPDYFKMNNYDFDFSPNSQSASQFDPSRVGPYFGGSNSEASNLLLVPGSGKTQFPSTLG

SNENDGTQLAGNEQNPSSVLIPVPPFSSEMSNDDQQTGSNNTNFQTDLLNPTTKNDNSEL

HKSISNSSFFPIWNEQSFYSASINENAIKKDGEEIYRYIFNGTSASTPTPTPPVREFDSE

KTQQTQQQNSLQKS

>model.g243.t1 Augustusgene.g243.t1 JFAV02000093.1:23426-24664(+)

MNSAPPKAPWMSDAEYQRLYGHLLTTKIEEKPELTSAVAEPVEKGPPTIQSEDKEKYSIS

KKRRDHDASTYRDEEYLQRQKREKHLAEQIKKQEINLTAGGKVNVDHMVIQHYNERTIMT

KNENRDLSPIIKLKNFNNAIKYMLIHKFTKPGNVVLELACGKGGDIRKYASCNISQLIGI

DISEESIREASRRYQKMRNSIPYEVVFITGDCFGESLGPTVEPFPACRFPCDAVSVQFAL

HYAFETEAKAKTMMLNVTKSLKIGGYFFGTIPDSEFIRYKLNKFPKDREEIKWGNSVYRI

KFANNEYLKNDYEFPSPFGQMYTFYLKDAIDNIPEYVIPFETLRSLADEFGLELRLKKGF

NEFFAQEFPAWYNQFSPRMKEGLKRKDGKYGIEGDEKEAAAYFYTTFAFQKVR

>model.g273.t1 Augustusgene.g273.t1 JFAV02000094.1:4075-7344(-)

MPLLPSSRNSNNHTVKGTVSASYASSTGSTNNVSNVSLEDQKCSEASQVPSSSTSRWRLP

HYRRHHKLKASISSPVLPQSTDKANGNSLSLDTGFNATSNNALDDLNESLAKFKGYMHQS

MSENPSPVNSVGTSSTNSTNVSSMTVDMNTQVLQSKFSPNGIEPAENQHHHNQHHHHNHH

HHHHHHHHHRTSRVLKLFKYKHNEHGSCSSDDISTCKEAFGNVGDDRNIDVQANFESLKY

QQQASELKDSSKQKKTKRSYSSRILHYATGNTLRSKSPGTNFVNDNEQTTAGKFSETAQN

DANFVYHKSHQVQGSKSGFVVSPSVASSFSSISGKPMLAESFQSKSSSSPYVTEGQLVTS

FNNQHTQSPLPSPVLYNTRAGSSPLVDQSYGLNKQIYSRPTSALKRSKSYTISDPPYSPY

GFNHTSNRSFAATADGTSPVYAASTLSASSARFGDSSESTFSPVLSRFSQAPIIHSPLNT

STSAFPNAQANIYNNKNGMTPGSTTETGASKNARYKRSNTFGETSLMNMQQHHLKQGLLA

KNLATESRSGSNTNTNVTCNSDTQDENELFEYGETESAKNIHLIADADDDENDASVAFNK

VFSSASNSFSNLNLPSATSTGTQFLDSVPTPHTNKYNTFSAASGAPAGPVSMSTHSKTHY

ISRRKRTNTCGSMNSRASVSTNASSVVRLSPIRRSTSPNRCVGNTTLNPGSTVSSLKSQH

QQQQHWSSISSGSSSIMSSNIRSNSSSNLLQISTASSIITPNTATSKVLGTDTYSDQNTN

MGNHLCGASQPLPPVPQLLSTHVQTAITQPMLQLPSSVVTSSKSRKLKSANSQKTEKANA

GTQDKHKSFSVHPGTKNLGSEMQNYSDPSEEYLQPPNSATTITSTGHSLPMDDERTVYSH

STTTPVAASQGSGLGDVGEDPEFMNLMSLMEITHPSSSEELSSVLDGKSTQNTQSIPQAT

VQGLEDLESELDLHPGLNHKDGEHGNGAANVEHTQYFTNDALDRHKDDVLMFQDDGMEDL

PQDHKNGTKHSTFIHTHSESESPLNGGGANESEEMMQMMLDLPELREDFDEDYFLINEMD

DLKSSHMNSN

>model.g274.t1 Augustusgene.g274.t1 JFAV02000094.1:8991-13907(-)

MSNKDNLFDVSLDDDANTNNYSRLNGNNNNNTRSSPTNPFSNDNSIDNSESSSDHAKKTN

QHITISSTVKAEDGLHVIENDENEDEDHDDDDSIFGDHVKTPTTHQHARFADDFDDFSDN

GDNDSDSDTAKKTLKAQNHHQHTGMKRLRWATRRHKDGKPKVGRAKTLKWAKNKFPIHSM

NQLYTSDDVDVNDLAGESKNRSAEKRTIFFNLDLPPNMVDEEGKPLVIYPRNKIRTTKYT

PLNFVPKNLLLQFNNIANGYFLFMVILGAFSIFGVTNPGFSAVPLIVIVCITAIKDAIED

SRRTILDLQVNNTTTHLLTGVENFNCSDDNISAWRKFKKSCTKVTWASLKYISARMTKKG

RAELLREKHKRELRQMNDNFQMPRISGNGRGSFDSVQDYRPSLDDPRTSMHYTANEMKKF

GNLIDYNMPYDEESKFKKSFWKDVKVGDVVRIHMNDEIPADVILLSTSDPDGACYVETKN

LDGETNLKVKQSLKCSNLIRTSRDIVRTKFWLESEGPHPNLYSYQGNLQWNERQQNEDSH

SGSAPVLRQEPVTINNMLLRGCTLRNTKWAMGLVVFTGDETKIILNAGVTPTKKSRISRE

LNWSVILNFFLLFCLCLIAGVVNGVFYDENGTSRKYFEFGTVAKTPAANGVVSFFVALIL

YQSLIPISLYISVEIIKTAQALFIYGDLLMYNEKLDYPCTPKSWNISDDLGQIEYIFSDK

TGTLTQNVMEFKKCSINGVSYGRAYTEALQGLRKRQGIDVEEEGTREREGIKQDKEFVIE

KLPKIGSGNSQFFPDEITFISKDFVQDLEGSKGDYQKNCNEHFMLALALCHTVLTEPSKT

VEGKLDYKAQSPDEAALVCTARDMGYSFITRTKTGVVVEVQGVEKEFEVLNILEFNSTRK

RMSCIIKIPGDKPKALLLSKGADSIIYSRLSKTGNDEALLDATAKHLEEFATEGLRTLCV

AQREIEWEQYLEWNARHDAASSSLNDREGAMERVADEIEQELILLGGTAIEDRLQDGVPD

SIALLGEAGIKLWVLTGDKVETAINIGFSCNLLGNDMELLIINDEVSEEDRENNAHRVME

DDPVTIVERNITRYLHDKFGMSGSEEELEEAKKEHEVPKGEFGVVVDGNALKLALMNKEV

SRKFLLLCKNCKAVLCCRVSPAQKAAVVKLVKDTLDVMTLAIGDGSNDVAMIQSADVGVG

IAGEEGRQAVMSSDYAIGQFRYLTRLILVHGRWSYKRLSEMIPCFFYKNVVFTLALFWYG

IYNDFDGSYLFEYTYLSFYNLAFTSLPVIFLGILDQDVSDVVSVLVPQLYRSGILRTEWT

MKKFWGYMIDGLYQSVIAFYFPYVLYSKTGLVSSNGLGLTARYFLGVPVTAISACACDLF

VLMKQYRWDWFTSFWVAISILVFFGWTGIWTSALRSGEFYKAAARVFGAPSFWAVLFVGI

LFCLLPRFTFDVLQKMFFPRDIDIIRECWKRGDFDQYPPDYDPTDPNRRKISKYSLDALS

QIEDSYLVEDQEKSSGGILDEDVNPMVKNNKNSHQRVKSVLSMIDESVPILSGRHISSAT

TSSPFENKLDNDIGLSNGNRINSGNNKYHQSMDTIQTEEIPLDIMENHIRGASTNDRRTS

TDQYTRYSQDYHRRTSPFN

>model.g272.t1 Augustusgene.g272.t1 JFAV02000094.1:2100-3977(+)

MSVPLFNNIKANGNSSNTINHLQPKLSSEIQLNEKESKLCILIKNYIDYYNSVSSNAPRS

PSSSSSSSSSSSSSSSSSSSSSSPHNGELLVARISGGWVRDKILGKQSHDLDIAINLMSG

EEFVHNMLTFYSENPLPDCFTNYQNDSSNNDNDSKSSGKPTGKPTGKPTGKPTGKSKNSP

FFHNVNTIKKNPEKSKHLETATTKILGMDIDFVNLRNETYTENSRIPIVTHGTPQEDAIR

RDATLNALFYNITTNQVEDFTQRGLQDLHSRILRTPLDPLQTFMDDPLRILRLIRFSAVY

NFKLHESIEQCFVTNHRDLMDALQKKISKERIGVELMKMLDRDLECCKRGLKICCDFNFL

TSIFYHCFDANILDINKDNENEDLTKIEKIYQYENNMESGGPAKNILNHVCYTKIINNSE

FPWLKDSSSTSSLKSETDENQGNKILVFSCLLSVFEEIHIYTTSKNKNVKNLKNSMAEWI

LRDTLRFGKQYYQPVALIVENISSLHNFIVENLADLHNSSGHLKRSDICEKIIPYEEYLF

IALEVEKILFGDETYQLIKQQVDSLKLEQAYLVKPLINGKELASILSRKPGVWMSAFNKQ

ILNWQYDHPNATKEDLIQNLGAFSLE

>model.g278.t1 Augustusgene.g278.t1 JFAV02000094.1:25552-27372(+)

MSDKNTRIAIVNSDRCKPKKCRQECRRACPVVKTGKLCIEVAPTSKIAFISENLCIGCGI

CVKKCPMDAITIINLPTNLENEVTHRYSANSFKLHRLPTPRPGQVLGLVGTNGIGKSTAL

KILAGKQKPNLGKYDQPPEWTDIIKYFRGSELQNYFTKMLEDDIKAIIKPQYVDNLPRAI

KGPVTKVGELLKIRIEKGEEYILGMIKALQLNHVLKRDIVNLSGGELQRFAIAMTCVQKA

DVYMFDEPSSYLDVKQRLKAATVIRSLLDATTYVISVEHDLSVLDYLSDFVCILYGVPSV

YGVVTLPASVREGINIFLDGHIPAENLRFRDESLQFRISDTSDFIESDASKSFQYPDMTK

TQGDFKLTVKSGDFSDSEILVLMGENGTGKTTLIKLLAGAISPDDGDKVSKMNVSMKPQK

IAPKFPGTVRQLFFKKIRGSFLSPQFQTDVAKPLKIESIIDNEVQTLSGGELQRVAIVLA

LGIPADIYLIDEPSAYLDSEQRIICSKVIRRFVMHNKKTAFVVEHDFIMATYLADRVIVF

DGIPSKDAVANTPESLLTGCNRFLKNLNVTFRRDPTSFRPRINKLDSQMDQEQKASGNYF

FLDSGSV

>model.g280.t1 Augustusgene.g280.t1 JFAV02000094.1:28968-29882(+)

MISEKGSTIAATLGTVCWCVQLIPQIIFLYRKKDCTGFPPIFMFLWVICGIPFACYTLIS

HANIVIEIQPHLFMFFCSISYAQSLYYPPYGQRSIKKVLLHLIPIFLIDIAFECGFVIWL

RPMYAKGVTAPGIVFGTVASILLAAGLVPPYWELAKRNGEVVGISFIFLGVDSLGAYLSI

LSVILGNMDIMGIVLYSIVAALELGIFLSHFIWCCRFKWFGNKEKLLDQEKDAERYSGGE

GDNESLATATNGEPLVIGKTESDNYGTQSLVDGTRNERTRSNSFLKDNSVSVQVQEETRS

SRADV

>model.g275.t1 Augustusgene.g275.t1 JFAV02000094.1:15137-15586(-)

MSNPLPKRIIKETSRLVSDPVPGITAVPNEENLRQFQVEIQGPSESPYAGGLFKLELYLP

EDYPMEPPKVRFLTKIYHPNIDRLGRICLDVLKNNWSPALQIRTILLSIQALLASPNPHD

PLANDVAKEWLENENAALQKAKEFTEKYAK

>model.g282.t1 Augustusgene.g282.t1 JFAV02000094.1:33073-34092(+)

MGYLDQLKAIQQNKKTQSQNQRSRNSYADSCNKNGTQSNGFTDPLDMKSSILPERYTREV

DPAVKRLKELRRQKQLEEDAKRGGTLKVRKPSAARKARQSPTDKDSSITSTRFRKKIENK

RQSMTAPQPKAPVVKEKKLSFEQLMAQAEGSNGPNGAPSHVNSAAVPQKRTPVEISGWKK

KKKKKSTTTPSSNGSLQERLKTASSQGGASNPSEKQETLMKIPSSFKSGPSKKILKKLEK

QKSSRGGYYGEEEDDSEFDDFIEEDDPGYDRSEIWKLMSNGRNRSSRPYEYDDESDDMEA

DGFEILEEEEASRKYARLEDKKEEAWLKEHEKAKRKRLNK

>model.g277.t1 Augustusgene.g277.t1 JFAV02000094.1:19186-23916(-)

MVSELPDDVLEHPDLYGLRRSGRTSANSKSTSYYDSEEDDSDSGGAMDKSDKENYSDAAG

VDMDDMDDDDDDDDDDDEIVVKKRGSRGRAAKSNGSTRSSGRTASKRTNYNEQDYGDDDD

DAMEVEEQGYDSMDDFLADDDDDVYGDNYGSSKKAKKKQSLKVTSNKTKITTKSKSTGKK

NNSRGDGGYSSKPHQEQIPTRFSNRAKSKVVSYKMYDEDDEDEDEIEQDWLVTDDGDEVD

DYADNDNGEPQESIDCVINHRWRDFDGNDDKSKNDTNSESQDQDDQAIQKLTHEPDLQEC

KEKFQFLVKWNGKSHLHNTWENFDTQTLSTQRENNKKPIKGLKKVDNYCKLFIIQNYLFR

NDPYTTREEIEVMDIEKERRLDEFEEYKKPERIIDSERNEHNQLQYLVKWRRLNYDEATW

ELASEIVQLAPLEVKHYQTRTNSSILPKNSSNYNQRPKFEKLIEQPKFIKNGELRDFQLT

GLNWMAFLWSKNQNGILADEMGLGKTVQTVAFISWLVFARRQYGPHLIVVPLSTLPAWQE

TFEKWAPDLNVIIYMGNTQSRDTIRDYEFYSGANKKSGLKFNVLITTYEYILKDRQELGS

IKWQFLAVDEAHRLKNAESSLYESLFSFKINNRLLITGTPLQNNIKELDALVNFLMPGEF

SIEREIDFENQDDMQEEYIRDLQKRLKPYILRRLKKDVEKSLPSKTERILRVELSDIQTE

YYKNILTKNYSALTSNGAQGHFSLLNVMAELRKASNHPYLFDSVEDMVLNKFGDGQMTRE

NILRGLIMSSGKMVLLDQLLTRLKKDGHRVLIFSQMVRMLDILGDYLKIKNINFQRLDGT

VSSQKRRVSIDHFNAPGSDDFVFLLSTRAGGLGINLMTADTVIIFDSDWNPQADLQAMAR

AHRIGQKNHVMVYRFVSKDTVEEEILERARKKMILEYAIISLGLQEDSKKKVKDQPVNSE

LKEILKFGANNMFKANDNQKKLEQLNLDDVLNHAEDHVTTAEVGESHLGGEEFLKQFEVT

DYKADVDWDDIIPQEELEKIKEEEKKRKDELYVKEQMDIAQRRQQTLEKMRNSNGQAKNK

TSDNNDSDSDDNDSLSSSNERKPSARRNGAMNKLADKDIKSLYRALQKFGYLDAEKIEQL

MLDRHLAPKNVNTYLSKYDELLKYAQEECIVQEKERAEILKDMEEKARVFQDKVKRGEIT

NEEELSASENPVKLLNAKKREKKSISIDFYCLNGVNAETLVKKNNDMKLLKNYVNRYFRE

DPLKFKLRKPCKTAVNWSCEWDQFDDEKLMIGIFKYGYNSWAQIRDDPFLGLTEKILLDG

KTNATKESTPLPQPSSADDNSSTTSLAKLANAQSALNKKNVTPQASHLNRRVDTLFNHLR

DIVENPDLPTGPKAHSNSPGSTPPIAHALKRSAPVSKSSGANGNTSNSNKKMKFLPLNIP

IAPKALLQQQQHKQQQHQQQKSTANSEETQIKKLMNPVRTSLVKLKQSGKTMDKKERLKI

LKKELLKVGNHIKEQSVAAAVNKNKANDHEKNSLFLKLWNYAAHYWPSRVESKDLIAMHK

KILNSVPQLPTGPAKKG

>model.g276.t1 Augustusgene.g276.t1 JFAV02000094.1:16254-18053(-)

MSDITEKVTEQLDNLTVNETPAAASAAAATTTEGSDVPENSTSSLYVGELNPEVNEALLF

DLFSPIGMVASIRVCRDAVTNQSLGYAYVNFQDNESGARAAEQLNFTPINGRPCRIMSIQ

RDPSKRRQGDGNVFIKNLDPAIDNKALYDTFSVFGKILSCKVAVDVDGQSKGFGFVHFED

PEAANDAIESINGMPLNGKELYVGLHVSRKERQAKLDDIKKNFTNIYIKNIPLDVTQEEF

TELFSKIGPTFSCALALDDESKSKGFGFVNYEKHEDAVKAIEELNDFELKGSKLYVGRAQ

KKFERAQDLQKQYEVFKNEKLEQYKFVNLFIKNLADSIDDEQLNELFAPFGTITSAKVMK

DENNNSKGFGFVCYSTREEAQTAISEMHQKMVLDKPLYVVIAKTKEERRAEYLQQRQAKN

RLRFAGAPGVGPHHGMPNMMAKSIPQQQMMYNMYAGGAPMGQFTPQFQRGFIQPQMIYPM

QQQQQQQQQHAQQFNQQQAQEARKRALGQPLFEKVQVKTNNDEQASSRITGMLLETSEPE

VLQMLQDEAFFEKQFSGAKAAYDNMNSQQAAAAAAAAAATTGAAPVPAPAAADAPAPAEK

>model.g281.t1 Augustusgene.g281.t1 JFAV02000094.1:30196-32742(+)

MSKRLSKEYFDLVKDALAEERGSRTTRQSKRKKPNEATPKIDEIVDLDSDSDADLAHGNS

QDVITLDSSEDNPPSSEDNFDDEEFNSDDFEDVPMDEHVSSGDEQDDNMTFTINTTKQTS

GTPKKREVNSVSKEEKRRRLFFHKTYLLTLLVHGYLRNKILNDPKLHKKLDKLIPDQVFD

LLHPPKDNELPLRSTRKLLDGLKKSIEIWQKYYTCSYDNNHYLMGLYMRPWNEIGHSSSK

KRALLMSQQEFLTTVLKKKSGSREIGTQGFVALLRAVGLNARLVFSLQPPDLTIMKKELD

NKDDFDLQLLLKQTNVFIADKKLRKMRINTDAKYEVQTKPENDIAILPVESLKYPLFWCE

VWNKFNKTWITVDPMNFKTIEQHTMNQNSRSKLEPLSHRQANKFNLLRYVIGYDRKLGCK

DITPRYTTQFHSKVYKKRCTRDDEAKEWYENSIKFLNLRGGRGTLKIDEYEDEFFTNKLN

LETIPDSLQDFSGHPKFILENQIPSDSVLRDGAKHCGFFRIKNRNKSLKVYLKSDILKLR

TARQWYMEGRVLKAGAQHLKIVKKRKFGPTKNYGDSDDDDMERLYSVDSTELFVPPLASP

RGEIQTNVYGNIDVYQPNMIPMNCVLIEDALAIRAAKFIRVPFAKAVTGFEFEKGFTVKP

KISGVVCLRDFKDAVCSAIEGIKVAEEEEKEENREVAALSHWVTLLKKLKIQQRLNMLHG

SVDADNQNVQEVAAGGFVAADRNDDQDFDQEKEAGFLHEDEQEGEEGGGFVVNEEVYKDD

AGEASDNAGGFVQDVLVEKKEESTHPEDEGGFLPDSANSVGDEKTEDKQGDEVNLSDEYN

AFLEELNSD

>model.g279.t1 Augustusgene.g279.t1 JFAV02000094.1:27775-28521(+)

MTHSRKGLWIVGYGSLIYKAPIHYQYRVPGLIHGFIRRFWQSSSDHRGTPASPGRVSTII

PYEDIVSNPSILQDTKNYLHLAQDHELTKDDLTILATAYYIPPEKVAEVVEYLDEREQDG

YTTHKIEVHLMPPSPMVDDQYMEVTQVLNETLPLHEDPHRNMRFYNLQSTVYIGTLQNES

FIGPEDINSTAKIIAKNVGPSGPNYEYLKKLYDCIERDLVTFKGQYHLRHRDHYLIDLME

KTEKLRVAA

>model.g283.t1 Augustusgene.g283.t1 JFAV02000094.1:34212-37088(-)

MKFGSQIFDKSVPEWRYNNLDYNKLKKLIKLCTTTQYSNPDHLAIMKQHFEDQFLNVNIF

INLKLNEITSRILVLEEQLLALKQKKDAMSSASTINSNQQIDSHRLDTQHKASLSKTKRV

FLKKIDKCNQLLTNLSRFLIVQKIAIRKLFKKFVKHYPDRQRGANFIDELQSSDIFTKGY

NGVSIINVDLDPYLLEVSLIMDYLYSDNTEQELQEQEQEQEQEQEQEQEQRKKQIRSNDQ

VPQKSHHKNKKTSKVFHDECEFDGLMLKNSFKSKKLVVSGESSMQLKFMLMKLGFLLIAP

GFFDKALTTTVSGTSSFANAENTASSNAHNLSVPENNTLNRSLSVQNLRKQSEVSLLSNQ

NLESDFEKLSLKLLNRPIANNGHQKLDIETSKEIDTHPNLIISYLKESLQSLPNDTQNNE

KCCVQCHIGGIRQFIVTDQVPYSSIINRDSEELLDPSNTMHSSTHTNMSSSTTSNTTHTS

SSSSHPLNKMIIEWIQSKNLQVIHPTLEFKRTRFALFDKTTKNIKYLCTLDENINNNEFI

ENNGFLTLINLDPESKTSSTSGNASHSSSPASNKKQIDTRLNNEIEFFIDFYKNLQANNI

MAFPTNNTLWLSMYHSQLYDMQDFYKFTMQDYKDSYLYTSDLITNDIFYFVGGKVIESEV

QELTKRQKTLNDDHSYILKKPHRNVVALLNTDPNSQNLLYQQESGQSGENDNQQSSQTQQ

LRYWNEFDDGDEYENGNSNGYDYYDENEDYDDLENGVLYPDERFIKISKNFIDTAYKVFF

PHAPLPSQSTLEHLRLGRIRSSGNSENSSLLDSPRLFDHPYESYTGEQSHNRNGSMVKKA

NFLSMEDSMIIYEYKHDQVFSVLYILSLVLSCLTCGVSIGIVLSVFDLIDEHSSELEISS

LFIAILICSLLGALVFGIISLLLLFSRFTKAPLWHYMSGFSLFIAVTIIACYGILGLVI

>model.g297.t1 Augustusgene.g297.t1 JFAV02000095.1:23045-23515(-)

MSTALYQISPETKSRINKFRTATSRAEKLEHLVIKIQPKPSYEIVVDEQDDEDDDDDDDD

WGVEVNELKDLPEALPDNLPRFVLLAYPLVTRDGLKKTPLVLLYWKPPTVVSQEWKMVYA

GCLEMVRNFTNPNKFLEVNNGLEDEDDVQDLKEQIEA

>model.g300.t1 Augustusgene.g300.t1 JFAV02000095.1:28976-30607(-)

MSKFALPPIVSIKNALFKRSNVSQAPFIFKNPINFEIPNRNAQDSERKWCVWGTHASQFL

NVLSRREYLAFPSPSFVKYHKIGKDDTLNFTTNVLKFKPPPTSPHLSARFEFFKSEFDEK

LATFIKNVGNTSTLVSYELTNSTRKIDTELYGELLNRLHLKDLENRLVIGLSNGQMRRSR

LARALLTKNDLLIVDDLYLGLDPTSRSLISETINWYNSSYKNESMVIVGLRPQDTIPNWC

TDLIIADETGVVYQGPNSNQIVNEYRGKWADLKKQQSLKTLNDVGAHIPSVEQLVSRHEW

YNADHTHQPCHFQIKDLSISYKGVPVLKDIDWTILKNSMWHIQGNNGSGKSTLISLLTAD

HPRSWSSNIVDENNKKRKSGSSSYFDINKSIATSSPEIHSIALKFSDMSLNDLVVSGMNE

DSSNNFTVSSSLQKSTSPERFELCDEYMRFFGLDSLKKAKFSQLSVNDQKLALFVRCLVK

LPKLLILDESFSGMDMPTIEKCHDFLKQWPGTVLIIAHLPEEVPEFCGSSIKLIQPGKYE

ISGQ

>model.g294.t1 Augustusgene.g294.t1 JFAV02000095.1:19007-19651(+)

MTTLIDLKTIVQPYTKHKQLSIRESEDLTLDLLLNFSEEDAADAETLQKMGLLLTKKSYM

DLIDERNINKKCGYPLCPSQVNRVTDLYSRKNSIMSKSNITNPYAYLTQYCSKWHSQCSQ

FYHIQLSDEALFSRIGEHLKIYEDENEYTDVELLEDMMEKNNRRDIVDLVSNLQQLRLSG

DSEVNIETEQLAEMMEDFKIVEHNDPEFYGDYEKD

>model.g284.t1 Augustusgene.g284.t1 JFAV02000095.1:557-1268(-)

MQTSEFPVFTFASMNDDDDDDDDDETLSLLINVHSAQSMNQSMNQSMNAPTNATSSTQQQ

LQYKIQILLHINSLLLSKLYTQQQTLLMQQQQQQQQQTQEQPPAEQTIEQKIHSDYLKRV

HANLQCISQLHQGLKGAKPSIMEPPVSYYGGGNSAGGINSGSNSASSAGGQGGQTPGNTG

IINDALIKQQEEGLRRLYLLLNRMFEIW

>model.g290.t1 Augustusgene.g290.t1 JFAV02000095.1:12238-13011(+)

MFDSAIYTTPLNFSQFQPKLYRGTHPHEKNFPYLKLLQLKTIISILPEPITEENDPEFVK

FIQVEKIKHVHIPCDGKKKKKKDKNKKDKDKGKAKDVDGQEAKEADGCAEKLKDAQKKDK

SAPTSSGDPAESKLQDEAELAKQEEEKKVKRRDKAVPITEQSVYEIIDILLDNRNYPIYL

HCATGETITPIVVACLRKLSYWSSVSIVDEFIRFSGSINVHERAFIEEFNVDDFTPKLTD

KTHYASWIKRTQGWKTRS

>model.g286.t1 Augustusgene.g286.t1 JFAV02000095.1:5684-5992(-)

MSTNPDTAKQVLLPLELIDKSINEHVLIITTGGREYYGVLKGYDDFVNVVLQDGIEIKHE

TTTGQGAQLEEDPQGHVVNKYGKEMLVSGQNIVMIVPGQRDYV

>model.g296.t1 Augustusgene.g296.t1 JFAV02000095.1:21253-21675(-)

MSSSALPYSRAAPSWFKLSSDAVVEQVIKYARKGLTPSQIGVLLRDAHGVTQSKVVTGNK

ILRILKSNGLAPEIPEDLYYLIKKAVSVRKHLERNRKDKDAKFRLILIESRIHRLARYYR

TVAVLPPNWKYESATASTLVA

>model.g298.t1 Augustusgene.g298.t1 JFAV02000095.1:23814-25541(-)

MHGIITDRKYKHECSITYDNTKVPLVEPEHISVDEKRELEFGRLTSPEYLHTQKSRNRKP

LTPNKFDPPGIIISLITYLNFLIIMVLGHVHDFIEHIVDPDKFRGITEQNGLAPWFSRKD

SFFMRRMKQKIDDCFARPTSGVPGRFIRVIERVSHNMNEYFTYPGNSKLCLNLSSYNYLG

FAQSEGICTDYALKSVDDWGIGSPGPRNLTGTTDLHREVEKLVAKFVGKEDALLCSMGYS

TNANFFNTFLNKNCLIISDGLNHTSIRTGVRLSGASIKVFKHNDMVYLENLIRDQIIQGQ

PKTHRPWSKILIVVEGLFSMEGTMCNLPKLVELKKKYNCYLFVDEAHSIGAMGTTGSGVC

EYFGIDPNEVDILMGTFTKSFGAAGGYVAADKKIIDRLRVDMSTQNYSEPSPAPVLAQIY

SSMQIIKGNLNPGEGEERLARITFNSRYLRLGLQRLGFIVYGIADSPVIPLLLYAPSKMP

AFSRLMLERNIAVVIVAYPATPLTESRVRFCVSASLTKDDIDYVLRHVDEVGDMLFLKTS

SGVGVGDLNGKPPRWNIEDVIAKTPNDCKDDKYFII

>model.g299.t1 Augustusgene.g299.t1 JFAV02000095.1:26347-28482(-)

MNFSNSEDEPSSNISVVSSRSNNTTEYESLTSLHTLHPTFAQPTTNTDSTYRPEKEFVSY

IASVVNEICRFKPHSLRKLTEEEHGMINYKLNGRHNSKEFKLAVALSAQHPIVHLDYYRE

FDDAVGNSQGDKLVRKRAFTQREDQAHIELEKKYSPKYEIHDFVRCFKLLSKSDYQNMLK

VIHAGQYIVYRTVMKFFRFRNNIKYTNVVIPGVTKILNIEDISECYGISKYHFHRMFKSF

ANVTIKEYETISEIFIRDHAKQLQEIISSNKIFHNHGSVLFNMNESKFWNTVSTMVIVNR

ESSEKTVKETMLPGLFADFNTNANNRASKKRRTASMVEAGVYSGNGRDSSIYDQQVITRD

FRRGFFGEQAKKYVSYDLDHCLLFGFSNTDNIPHFRYLEETRKLAKPKRNHVPTPGHLEK

EENRTIRICKTNADFKNQGLTSSYFTPLHQQRELPALAFNGEHKDLYEELQLLIGRNTEQ

ELTNATGKHSNNTIYAFHQPAASNTLTDNVVIDNVVATCLKNIDERKHDLNCKPIHEDTA

QHHQTDKNNEVACRKDTFDSIIDIFDNGFPGSAFDLNNKYSLVMDHIQTDNSHSSGNASS

MDLEFSSPGNKNAHVSSNLNMTSLSQEYITSAESSNKATTTMDTAGTATTTASTNISRTD

NQNLDMQSSSMQSAEAHDESDHQPLNSIVFFPAEEEEDEVYKHILNTKLFLE

>model.g301.t1 Augustusgene.g301.t1 JFAV02000095.1:30990-32351(+)

MEENYDVIVLGTGLTECILSGLLSIEGKKVLHIDRQEFYGGESASVNLTELYKRYKPEYL

AQHGGKIPEKYGRDRDWNVDLIPKFLMSNGELTNILIHTDVTRYVEFKQISGSYVYHKNG

KIYKVPANEMEAISSPLLGIFEKRRVQKFLEWVAKYRDNDTSTHHGLNLDNNTMEEVYYK

FGLGKSTQDFVGHAMALYPTDEYLQLPCRPTVERIILYSQSVARYGKSPYLYPLYGLGEL

PQGFARLSAIYGGTYMLGTPVEEVYYTEDESTKIKKFAGVRTKEGIAKAPLVIADPSYFP

DLVEPTGEEVIRCICILNHPISGSSSSDSVQIILPQNQIGRNHDIYIAMVGESHHVSGKN

YYLAIVSTIVETKEPLKELEPGLKLLGDIEDKFVGISKLMKPKEDGTKDNIFISKSYDAS

SHFESMTDDVKDIYFRIHGSPLVLKTRQDGENEE

>model.g295.t1 Augustusgene.g295.t1 JFAV02000095.1:19721-20695(-)

MIVAHDTLYLKRFTMVNAVPYGKQHPKISSRQLDLQVAQKAVENPAITYEKKILNEFIRY

ERYKSGSIPLPGKEHIDPKALLPLATHRYYMNRLKYVEHFLSRPPRTTVRGTRSYFGRMW

FPSNFLTNRHKSSRNLSMFLGLTRRSGEYHAALWKNLGNRNLDYTIMEAQWETELEKTLN

AKAMEAGERDRNHTVPFKKYQNEAQKILGDTKGPIFQWKRNKDYVSKFPILEEWLQPVLL

NYNAVYDRTMIKTENLSRFRVETLPKIFQRRHSLAIKVYNSSIAKWNKMCAEDLANCNPF

DPKNDLKSILLKYGFLKRKKSTSND

>model.g287.t1 Augustusgene.g287.t1 JFAV02000095.1:6786-7451(+)

MSTELPMHMYVRPLTLEDVEAIKDLESVGFPPSERCSEDNINFRITNCPELCSGLFVRKV

SDPYAENNSNTGSKDEEGDESMLGVVGPYDAGAPQVLGEELIGHILATKIKSTLTAITLE

SMSAEHDESSNYIAIHSLVIDPKYQKKNLATLLMTDYIQKLSNQEVGTKITIIAHEHLFP

FYERLGFKKLGPNENVNNDKEFSAHGVWYNMERDLVKDEFEE

>model.g285.t1 Augustusgene.g285.t1 JFAV02000095.1:3758-5368(+)

MMIAHKLLQIPITILQFLITVYFSGLHNKYNVLTLAFNFIINFSPIIIWLTIFKKAGEIP

ITWRPDIHGKTSFMADMFMFGDYWGEILVQYKEHNSHGPLLHKLSFIVSTLGFAFPVLIC

IPLALWYYIYYIKKYTKNLVSYHLPLQNNNPKTQLHIVFIPLMVWLALNLDHQFASQQES

NFTEWKNMLSWIFYVLAHLLAPIFTAIYLYVFKAPGTLKCFSFAMGLQNICGVFTHLLLP

MAPPWFTHMYGLFNNSELNYNTPGYAAGLIRVSFKAGTHLASEGFHKSPIVFGAVPSLHS

AMAFQCFLFVWFNKVQAVSETTSSVEMDDLESGLPKITVNADSEADSETETETQEETHSV

HSTKDNLSVEEFDMEDMLLKADSGSRTSLSRESSTPSEMLFSATEQDTPITSAPEINTDL

RAQPSRVVKIVRSGWIPRVIVSGFIMVQWWATMYLDHHYRFDLFVGMFYSLCSFLIVNHF

VLQKRVLTAFAEKRKMMMDPSKEDPKDDEGMSMGMRVFKGTRVEWVFDSYDNKHYER

>model.g291.t1 Augustusgene.g291.t1 JFAV02000095.1:13088-14254(-)

MNVTVINEISKSIYGPMEYSDDLTLENLQALLQFEAEYDEKRHELVYGQQVLDVVNDRNK

TLKDLQIKDTSTITIRMKKLDPIEQLRLKMLDDVETPEILKSEILIDLKGFKQFIEFIKF

SPISRSKPLAQVLRNNALMEELAYARKIDEQMTHAMEYTPENFTTVSMLYINMEINGHPV

KAFVDSGAQNTIMSTKLAKLTKLDRMVDKRFQGEARGVGVSNIVGKIHSALVKIETQFVA

CSFTVLDINVDLLLGLDMLKRYQACIDLEKNVLKFAGIETPFLGEADIPREPVLEKIAGA

DVNAGSGARGTAGQGFNANKRATPTQAKPASSVKQPKQEERTLLGMPEQFSESSVKTLMD

LGFPREKVIEALKVANGNADLAASILLNM

>model.g289.t1 Augustusgene.g289.t1 JFAV02000095.1:10767-11813(-)

MDYAYEPTVDDQIRENSDKDNTGNSASTIMQDAKTEETFSKLEQKLDQSFTKTTNFLYNL

WNEDQHVSLFREEDKPGPANSKTPSDDDSSDGIVTRGERAAQNVLDAFDTKLQAVEDFVH

SKDQQQKAAENATRLIKSKWTSWSTGLTQFSESLKTKLNDISDVIAFPENEEHEREVTER

LKVLGSDENVYLNYKGDVKDCKYSNDDISREVARDINLKVLVDSLVAGNKIDYKTFWAIY

FIKKQELLEASSQNNESEKSALKTNLPSWDDEEEEEEEENETEDRNHNENNTVSDSSNEP

FENNDIRLESRESMVIVNKGKDNAVENSATAGQSESQDSEDDDDDEEWQ

>model.g293.t1 Augustusgene.g293.t1 JFAV02000095.1:16588-18621(-)

MSEKQSTPASLRKLKNFVLNELNNPETSKTNRTRSGSVTTHGLGLKQEREDSFSSFVNDS

PIIPKKSNPYTQEKSLIGARSKNLQKEDDTLKEKVVKVQGLGIHSLNDSALRTPIAQKVL

LSDQPNTNKADSPSDRPKLTIHPGALHTEARGVSSRSGIPNISNEHELELLINMVKLPVY

FEKLMAFSLLGCFNLFLYYFTMIPLRFVYLNYEFCRIIGINFYNKLFRNSFAPVVLAAEA

AQSGTGTSGRRQSRAREETQKRHAFKSTKQIVSFLQVFRMDFILLVEMIITCVILLLSLD

TSKAYHKIKGQNAIKLYALFGVLEMADKMLSTIGQDFLLAIYRRVSDSTLLLNNKTNIPS

RYFLLLIIARWFYLTIHSTILIYQTIALNVAVNSYSNTLGTLLLSMQFGELKSSVFKKFD

KEGLFQLAIADVAERFQLVSMLAIIALRNLVANKKSFTMSSSFPQSWNLNLIIFNQYTTG

IVGVLCGPMVKVIGSEFIVDWLKHGYIGRFNRFKPEIYDKVFRKILVQDSVNHTNFKLQA

RLGVPVQAQVVTFIVLVLPKFKRMFYQMSTVKRTAVLFGAWLFLLTVKLATQLIIKKWCR

HITKKHEQQTSAASDKYYVPGELSGSQGMVDEPMRRIIHNDSANMPASAAEFRLKKDAKD

PTSLEKVERYKMCSKKIW

>model.g292.t1 Augustusgene.g292.t1 JFAV02000095.1:14631-16118(-)

MFRNIVSLKQGVRSLSLFSSTASRRAKTNTSGLLLRNLKPSLKPMNANSSPFSITLQSRF

IQTRNSKLYSTAQAEIPHARPQRKTRRLMNSHSNVGYWLIGTSGLVFGIVVLGGLTRLTE

SGLSITEWKPVTGAIPPLNQKEWEEEFAKYQDSPEFKQLNSHITLDEFKFIFFMEWFHRL

WGRAIGGIFVVPTLYLLASRYTSARVNRRLLGLSALLGLQGVIGWWMVKSGLDEENLEER

KSKPTVSQYRLTTHLGAAFMLYMGMLWTGWEIVRENKWMKNPAESIKIFEKLNNPQLKPL

KRMSLALVALTFLTAMSGGLVAGLDAGLIYNTFPKMGDRWFPSKRELLDKNFSRKEDNSD

LVVRNMLDNPTTVQLNHRILAITTFSAVLAFHMYAVKRKALIPKNVNRTIHAMMGLVTLQ

AALGIATLVYLVPISLASAHQAGALALLTSALLCASQLRRPRPIMQQLIMNLNKNGIPQK

TGSKILKQINGMPMKS

>model.g288.t1 Augustusgene.g288.t1 JFAV02000095.1:7895-10654(+)

MEPVVCKSLTALKLEVESKIQEEHYEQLSFKQILDKCMINLSTYQQWWKKIEEFQATSFE

VLSESQQRQLVLAYENAYFYSKLVVKGSAQIIPRMQEFKILKQQQQLQGKQSDLLQVYDE

FMHLLFKYQPNFDTVKSFLNLHSFTKQQKMQLNQQLDLLDSTNNNTISCKLLHFILHHHK

NSTLLIDIRPKVEFERYHIKFEKCISIEPISFKETYTDQDVLDRSMCTSPSKDKSLFQSR

DMQKFIILYTSNRNPQYYTQLLAFKNILLKKSFEKTLDLQHTKVLILGESVEQWVNMYGY

ESCTVGSLQNENYEKHTNGVAKTSLLSSPPSSPPSLNYAPLKQQQILQPRGISQGREVLN

GSSSNESLKTSYPDPLLSFGNANQLKPALFTNNSPRSSPVPMYSSLPDSQQQQQQQQQQQ

PYQLPTLYPLPAQQSPLPIKRSPSFRNVYNNLLNKGAKLNRETSPQRHTPSQSAVDLVAS

YPETPALSPSIHKSPSLSMDSSLSAPSTQPAVMQSALSKMLRNGTGKVETSSTAAAPPVS

SKFFCTGLINLGNSCYMNCIIQCLLGTPELVQLFLGDDYKRYINMRSKLGSQGVITKNFS

DLIHTMSDKNSSVVQPRKFKNACGLVSSIFRNNEQQDCQEFVQFLLDALHEDMNMSEPSS

GRKEVSEADELRREQMTMRDASIIEWERYLTTDFSVILDMFQGQYSSRLQCKVCQRTSTT

YQPFSVLSVPVPPASSMAQNGGPSAPYKININDCFREFTKVENLGRDELWNCPACKKKQP

STKKITITRLPKNLIIHLKRFDNSLNKNSEFIYYPPVLDLTPFWFDHKPEFYKSAAKEWL

PNIQQRIQEPPFKYSLNAVACHTGTLYGGHYTAYVKKENFQNQLNNTQWVYYDDASARKV

KNNAEMITSSAYVLFYRKID

>model.g302.t1 Augustusgene.g302.t1 JFAV02000097.1:55-1016(-)

MFLLKLETLWGFLLWFSDYAKAARHGDSTSSTTSTALETSTVSVLTTTTTETCPSCSVTS

FLTSKPVWYFTSFTSYNGKVTVTYSMTSNVVANVTSTTFFVQTPIASEDLTKLSSFTGTV

PSTYSTDVFTYGDATDATVSVIYYVQTPTSHATMFLTSSWTGLSVTTYSTFYSTFFGKDS

TETIETIYYIETPIARTVSTFLSWYDGSSVFTYATDVFTITGTDNLETISTLYYVQTSLA

HTLDLTTTFWTGLSTSTYATSFSTFAGVDGHETVNTVYYIETPVSRSSSTYYSYWDGATD

STYSTSVFTVTNGSSPFIST

>model.g308.t1 Augustusgene.g308.t1 JFAV02000097.1:15588-16865(+)

MSKYGNRDYTTGHPNTQHDRANNFHSADGIAADNNLSEGYYIDNTDGRAAGSTGLRGNQG

LEVEDNNNNNNDNDDDDDDTEATHATVLLMGLRRCGKSSICKVVFHNMQPLDTLYLESTS

KPTLTSNKLNKNLINLSIMELPGQLNFFEPNYDHERIFKKVGALIYIIDSQDEYLNAITN

LAMIVEYAYKINPRINIEVLIHKIDGLNEDFKLDTQRDIMQRVGDELLDLGIDNQNGSGI

SINFYLTSIFDHSIYEALSRIVQKLIPELPFLENMLDNLVLHSDLEKAFLFDINSKIYVS

TDSSPVDIQTYEVCSEFVDVTLDLEDLYCVNLDSQQHDGRSQNKLDKDKEIMSFSKLNNG

LCIYMKEMVRGLALVALVRLKNEDTTNNANAGTVQSSTKSINEYLTVIDYNVEIFKKSLE

QIWKTR

>model.g303.t1 Augustusgene.g303.t1 JFAV02000097.1:3221-4465(-)

MTSIELNEQMFDNTMKNNMDTANTTSTTATTTTTTKSSLKPDVVVNHCTPIPSQGNLSKK

IPEFVKRRKSAPSAFLSSTSSSGSAAAHSTEQIVNQMEKEQDRMVITLLREIQHLQYENG

IYKQKLNSIMDTKDMNKFMSMHQLGSGCSTPKNNYATTTTTTGSANNNATTPSYHNTSTG

NGHSYFHTLPTSVSPLGMSNITRQSSLSRQSSLSRQGSASVGRQGSIGVIHNNPVNNAVF

STGVNTILNTGVNTVFNTTSHASYTSRQSRSRFNSLSGSVNGSVISGVITQEESPLTSPM

PSRRSSWNKQQGTGQVVGYAGQGVNSYRRRSSSTSYGNANGPTAQVPKLGEVLKRNKKNF

DNSMEKGNTQKSKEENGACTAEEEEEEEYDDDDDDDDDDDEEEAVMDSHSDYTTA

>model.g304.t1 Augustusgene.g304.t1 JFAV02000097.1:6757-7524(-)

MVNVYVTVGATVPFHSLIYELIHNLDILILNSCKAFPDFQSNTINIVIQYGDNYSERFQS

LLSTYLSTHSAFFKEISIKSSEPVFAQLETNTTEYNLQYTTPTNNTITKQVNICGFALSN

NISSFLHEYQPQLFITHAGTGSLLDALKLPNPSLSSSSSSSSSSSSSSPSHPLKTIIFCI

VNNNLMDNHQLEIAHKFSSLKLVTCVESPKQLSHYLNQLSDIVRDTNSRGGASSSGDHLH

MGYNTEFQNEVLLGNW

>model.g305.t1 Augustusgene.g305.t1 JFAV02000097.1:7910-9091(+)

MAGVKPFFEAQIQDCEILIRNKQENLHRLEAQRNLLNDKVKFIKDELVYLQEPGSYVGEV

VKVINDKKVLVKVQPEGKYIVDVATSIDIKSLKVNQRVCLKSDSYLLHKVLENKVDPLVS

LMMVEKVPDSTYDMVGGLTKQIKEIKEVIELPVKHPELFESLGIQQPKGVILYGPPGTGK

TLLARAVAHHTDCKFIRVSGAELVQKYIGEGSRMVRELFVMAREHAPSIIFMDEIDSIGS

SRGDGGSGGSGGGDSEVQRTMLELLNQLDGFESSKDIKIIMATNRLDILDPALLRPGRID

RKIEFPPPTVAARTEILRIHSRKMNLTRGINLKKIAEKMNGCSGADVKGVCTEAGMYALR

ERRIHVTQEDFELAVAKVMNKNDETAISVAKLYT

>model.g306.t1 Augustusgene.g306.t1 JFAV02000097.1:10495-13560(+)

MHIRDNMSAEPSQPQQEQQKPQEAGVEQQPQDFKSSQQQQQQPYGKKPYNNNHNNTHRRK

SFTKNGSNYNNRPYHNNNNSTGPYKKNQYNNNASKVVSNYNSNVAMNTQGMMADPSQVYV

AQQGAPYMQAYPYPNGYYAYAPAGSIPQHQEEALNSDAQKTFEKEGSEQPEQEAGSDVSQ

GKEAEHASAQSQAQAQLQQTILERKLQLQQAKQQQQQGASPATSASTTPTSPPPAAASTG

STAPAAAAGTSLSELLKQKALERKKQRDLEKQQSEAKAKAEQESKEQTAEPVQTESTTVA

AVPVAAEKAQDVTESSDSEAEEVEEPVKEAEPATETKEQEEAQPEAQPDAEAPVDAPAEK

ENTEEQAEKEEDKKEGEEESTEKKPLTFAERLKLQRAQKEASAAAAASAAAESEPSKDQE

ESNENETKEATPVAEEPALAADYVYLKDYRSQLETATPVEKTGAKFLYSLEDLFDISQNV

KVSADAKWKAFCEDSLLVEASHLPGNNGAYNKKGGAGYKGGPNGPYNGPGGRNMSNRNMS

KDGRSMSKRGGMGDRSGSSRSDSKRRSKKKGGRKDGDYHGSRFDPATNPHGLPPSIRAQQ

QAHNQNGGASEEAEFKKEENAEPAPVVKLNKTGKGWKPKPKTVVKITTADGTEILPPSEV

EKQFKSLLNKLTLEKFDAISSQVVDLMNQSKYPEDNGESVAIVVKFLLAKACDEPYWSSM

YAKLFGVCVHQADHELVSPKEENVKKGQFLVVHEILDKVKTQFDEGWVDKLPTNEDGTPL

DVEMMSDEYYELAAAKRRGLGLVKLIGFLYESNIVSDAIALQCVKKFLADINSNPQDDTI

ETLIELLNTIGAKFENAKLGTLTGGIVLDLVFECFQKIIDQEQCSVRIRFKLQDMGDLRL

KGWVDEEKLNSGPKTIQAIHEEEQIKKEKQMEERQQQQYRQQSNRGGSMRGNRNNSNYRD

NSYNNNNNSSRRSNNNNNSGFGGHSQGSTFTSSYQKTKEEPKEESKPATAANMFSILGEH

DD

>model.g307.t1 Augustusgene.g307.t1 JFAV02000097.1:13978-14952(-)

MPPKQDSFLQKKQSILNSNIDLSPKGSIDALCIPIIKLINDNSDDMVTTSSCSGRLSVFV

EGLKTKSQAKQYENSTPQTATGQSSQAPEEAVTSAMLEYKLGGKGDGGEWLYITHNVDEV

TGWLQKVQTSERAIRFLSQDNKVETKNIDMTTKRLILYKFEPLILHIKCRNFETASRLYN

VAMGCGFRESGIGSNFNVALRINIKLDVPIGYLNEADGSLQFIVPEVMLQNVLDPLTILK

FKENEAKLNELYIKINTEIIQKETKDQVSKESSPRESSVYAQETKEERRVRKKQEGLARQ

LELAKLKKEQHVQQNEEPTSTEHNN

>model.g311.t1 Augustusgene.g311.t1 JFAV02000098.1:4964-7312(-)

MLASRNLLTKARTTATRGLASSSASSSTITRDSKVHQNLLEDHSFINYKQNLENVNIVRE

RLNRPLTYAEKILYGHLDKPHEQEITRGVSYLKLRPDRVACQDATAQMAILQFMSAGLPQ

VAKPTTVHCDHLIQAQVGGEKDLSRAINLNKEVYDFLSSATAKYNMGFWGPGSGIIHQII

LENYAFPGALMIGTDSHTPNAGGLGQLAIGVGGADAVDVMSNLAWELKAPKIMGVKLTGK

MNGWTAPKDIILKLAGMTTVKGGTGKIVEYFGDGVETFSCTGMATICNMGAEIGATTSVF

PYNKSMIDYLNATGRNKIADFASQYHRDLLIADKNAEYDEIYEIDLNTLEPYINGPFTPD

LATPISKMKEVAVKNNWPLDVKVSLIGSCTNSSYEDMSRAASIVKDAATHGLKAKTIYTV

TPGSEQIRATIARDGQLETFKDFGGIVLANACGPCIGQWDRKDIKKGDKNTIVSSYNRNF

TSRNDGNPSTHAFVASPEITTAFAIAGDLRFNPLTDKLKDAQGKEFMLKPPHGIGLPPNG

YDKGENTYQAPPKDRSSVQVAVSPSSDRLQLLKPFEKWDGKDAMNMPILIKSLGKTTTDH

ISMAGPWLKYRGHLQNISNNYMIGAINAENKKANCIKNELTNEYGTVPEVAGYYRDHGVR

WVVIGDENFGEGSSREHAALEPRYLGGFAIITRSFARIHETNLKKQGLLPLNFKDPKDYD

KINPEDHVDILGLTTFAPGKDLTLRVHPKDGSAPWDAALTHTFNEEQIEWFKAGSALNKL

AKK

>model.g314.t1 Augustusgene.g314.t1 JFAV02000098.1:17150-17638(-)

MAQTSNDNKLRATCPFEKCASKIISYQDNQLVELPSYLSELYKLMRSSESENDATRTQGT

KFLVTQDIWDFDNIGVSKSLDSLDLEHRVQVLTDAGHAQYSFKNIVIPYKNTNYKIVKAL

KYLICADCDKGPLGVILEVFPVGTLDSTSHIQLNLLNVDVCKS

>model.g321.t1 Augustusgene.g321.t1 JFAV02000098.1:29810-33970(-)

MSQTNQSGQLTPRERVAIYEYFQGLHSYFMQIEDRRDRSGNSRAIKARSKLLNLSASQFF

ELGTDVYDELQRRSDPEEMGTYLVPKLALHVKRNQARQKLANLSTTRFNDLIDDILYEIK

RRNYDEEPKLASEASTASKASDNADASHPQVSDSTLAKKKSENTLVKKKSDNSLLQKLSN

QSSSNLLNNTGSNNIQTTQVIPKKAAMDWSSDEEEPAKKETARETSEHHAKDTKTLPDTP

MPTSKSVHPAQSMASIGATSTSQTQLFTPSYKKSQEDLQDKDTFEYHASTPTAPDGFVKR

GLMIKNISNKPSMDSINTNLHKTVTAATTTTPTTATTPLSAPKSPLPMATPNRFETLVSS

AMHSPPSIANKVVSSPTFATNHSTPFDKNLPHKQVNVENEDVHKENKEVHLDNGKSEGVA

VKKTASKLEDKEVTSQRSASNLELKHQESISKLENLQKTFNEQKTTLLDLQNLNTEYKKT

IDSLTNMDSQSKNTIVKINEENTHLTEQNTQLTQENTELKQENTELKQENTELKQENTEL

KQENTELKQRVDELTNKHAQLNTDYETSREQATASKSAITNLEKALQLKTTELATFQSGN

QHTKEMSSLSQNLNDLLIENETLKQKLADLEFSHNSLQQEKNELLQKHRELETTAKSAAI

PLANKEPQVQAPPVKNETNVDYSQYIQPDGLIPLNLVEEVESKIVATFEHLQSKPALLDQ

PVGEILFEDVALISDCVSKIIAVVEKSTTNTMGSRESALSTSSNVMLNDFDGNSNNIPVD

SKNKILKDSSVLLRAAVSHCITNIRYYAIYHTLLPKITVQSALSEILFTLCNLLNVVRIN

DGESDEFGNTERNNKSNPSLAYNRDISGSASSSKGNQAMLQKLDILNHTSSDLNDEDVSS

PVRPLKITQKLASPLKDQPARQKNSRSSSTSSSYNTGKPIQQRKPSNTGLFTSMLSSSVT

TSTAHLGSTLNLSNHKRQAESSEGKRNVKNLMLDLQKPVEEVEPKLDSRKVPSTLDRKVS

YQENTVDRKDTPLGLNERPEINYINEDKLQESIDPSFSSETLPTAQSYSFAGNAERDNDE

ESTEKQKPLQNKKSKLINSFHLEDDGIETGVDSGNANFSESDYDEDFNKPVQNYLQEEPL

DADENDDVDDDDMAYNQLKSKLSANSVIQNLDSAGMNNSIDKITTDSTHAERKPLMGSSN

NASDEESLTRAPFSPENFNVQTFDIANPDNNLNELLLYLEHQTIDVISTIQSLLSSIKQP

SSKTGELRYESFAINKVVGQMVEATRVSMDQTRHTQLKEYGGWVIQSLEDCRRRMNILCT

LGSDYTSDGFERKTLEKLDTDSSYADKHFKQRLAGIAFDVAKCTKELVKIVEEATLKEEI

EILSSRH

>model.g313.t1 Augustusgene.g313.t1 JFAV02000098.1:16059-16793(-)

MATRTQFENSNEVGVFSKLTNTYCLVTLGASENFYSAFEAELGDAIPIAHTTIAGTRIVG

RMTCGNRRGLLVPTQTTDQELQHLRNSLPDSVKIQRIEERLSALGNVICCNDYVALVHPD

IDRETEELISDVLGVEVFRQTISGNVLVGSYCALSNQGALVHPQTTVQDQEELSSLLQVP

VVAGTVNRGSAVVGAGMVVNDYLAVTGLDTTAPELSVIESIFHLQDAQPESISKNLRDTL

IETYS

>model.g315.t1 Augustusgene.g315.t1 JFAV02000098.1:18518-20728(+)

MSRKADIFSFFKKSSNEKEKSDPPSVPTQTQIDESEPRADQSKTRTLSSSNNLTNELQQV

DLSVPEDQNQSDSESDLSDLCEGQGDELLFQLESPQKTDNNSDNFVVEENGVSLMNEDVT

EYHTAIDISKVDDISSSPIKQSSQELTENKKDTKNKKNKRTREEYLELLKQNRLLKEAQK

EKERQEREAKKEQERQEKEARKEQERQERELKREQEKQERDARKEQEKRERELKREEEKR

EREAKKEQERLAKEKKLEEERLAKEKILEKSRISNFFKVSSSNLKAKTETVNIPSASRVI

DIESSPISEKTQFSNTSSNKSDFQKAFSAFYVKTGYQLPASLKLGNEDLEKSISSIDQQL

SKDSIISDTSDSIQFLKNAKQDFLSNSSSISSHITAIELLKEIEKGKKPEQELLDMLKRI

PHKYIKFYENVRPPYTGTFSDKTIIPKHDPFSTSETKYDYSYDSDLDWAEGSDGEAEDLD

ELDDDDEEDNEEGQDDGEFDGFLDKDSNENATEAGDQNANSNQAFKKKVFLTALIPSVQL

HNDVTTNFDEDERAYLKAVSVKLFFPTPIDTHSVIGNPSTQASICGEKENSKRKLNADDA

QDNASNASNAVNTSQKEKSKSPSKEPKTKKLKPQPPIISELKDLVNILREVEGSSFTIGT

LAEVAKVKTETKYSKKMIKDTIQHYAKKDSGTQKWKLINPSEFASLQQKLISSDSATWIK

SQVSDSTVGSQKEDEIQ

>model.g316.t1 Augustusgene.g316.t1 JFAV02000098.1:20967-24038(+)

MYDHENLQRSGKRSNRSVKIIDSPHSSNKQKQKQKQKQKQETKGIMSEPSSPIGGRNAPE

DSSPLPSSDLPSQTQPIANSSSSTTRRNNAHMSSPMQGIVSSPRAFDSPLIRRGTQERDT

YSDASSQSQRRPMASVSSPMAFPSSSLGSNIHSQRNIPASSSSMIGSSRAARNRTLNSQN

SRASPSSSFYSGSERARNTGRRNDISSSDISAHRRMVDMSDLSSSPRPLHSNALPSNGLG

SSSSVISSDDLSGPNEPVRVIWGTNVSIQECVNKFREFLMSFKFKYRKELDSRGETVDFN

GDEELFYIDKMKKMIQARTTNLNLDTRNLLAYRNTEKLYHQLLNYPQEVISIMDQTLKDC

IVQLCVDEGMSDSILDDIESKFFKVRPYNIDNRRGMRELNPGDIDKLVSVKGLVLRSTPI

IPDMKVAFFKCSICNHTTVVEIDRGVIQEPPRCPSPSCSQPNSLVLVHNRCSFSDKQVIK

LQETPDLVPDGQTPHSVSLCVYDELVDSCRAGDRMEVSGIFRSIPIRANPKQRSLRALYK

TYLDVVHIQKVDNTRLDIDTSTVEQEVLQHEVQADEAGGSGVQELRKLTDSDIRKIEEVS

KRFDVYDLLARSIAPSIYELDDVKKGILLQLFGGANKVFKKGGKYRGDINVLLCGDPSTS

KSQLLQYVHKIAPRGIYTSGKGSSAVGLTAYITRDVETKQLVLESGALVLSDGGVCCIDE

FDKMSDATRSVLHEVMEQQTISVAKAGIITTLNARTSILASANPINSRYDPDLPVTDNID

LPPPLLSRFDLVYLILDKVDENLDRELATHLTNLYLEDRPQNGSSASYDILPVEFLTMYI

NYAKEHCKPVITELAKDELVRHYVEMRKLGDDSRSEEKRITATTRQLESMIRLSEAHAKM

RLSQEVGLEDVHEAVRLIKSALKQYATDPKTGKIDMSMVQTGKSAVQLQLQEEIARNVLS

ILNSSTEDQIAVNDLIKQIKESGQNVENAAVVEALSRLQQEDKIVIKPVRGTRVVSLNTM

STHL

>model.g320.t1 Augustusgene.g320.t1 JFAV02000098.1:28835-29542(+)

MLQNISRGALFARSGTIHPLNNVLRLYSPCHHRSIATYLHSGSRVRGLKKNSDEYLTVQH

NDKKLHIDRVDLSAYKNEIFDKLNIHKYGIDLDENLVLQCLTHKSFAHGLKPYNEKLSVM

GYHFMKYFAIVKDMTKLQNSESANKTNIIGTAFSKRVLSNDVLNKVFEQHQLQDLVFWKK

RALDHNEGNFNGEFTVHSTCLKALIGAILLKNGQEKTSQFFEQEIYHKMIQLASKQ

>model.g318.t1 Augustusgene.g318.t1 JFAV02000098.1:25732-26070(+)

MSAFVSNLTSKVNVLVSKSIYYSKVVGEVAKQVYVKEGLQPPALADVKSVYSTLFAKAVY

YSKNTAEFTPILKNLNKNNYITYGAIGVQLAGLYNLGEIIGRRHIVGYKNHHH

>model.g312.t1 Augustusgene.g312.t1 JFAV02000098.1:8812-15372(-)

MFVLDGYLKVKRSGQTKEPNSEQKKNTHHSYCVMSSFRRHNFSIRGECLKKLTQLSLKQD

SATGEAVDDPVPYWKATTDQLSVSYTTDRTKVYTIPLTLNELEILFAVCSSVPETEDQAT

SLIDNVISHYFTTCTKQQFNDICKNKFKDVHNVRPDGRSVYECLTYNLCTFLIRCYAKYN

NLQPRIEAMFLESFTNYQFTTYSLCSLLGLLNAFIKTATDDAPSQLHDVVLRQFWDWNFS

DGHFDARFHELITNVLQNENYDDTLMSYFDLNYELSSFMFGERIAALQILILKSKFSFSD

DYSFIEHILRLKKCESSNENGTAKFDSFLETIKKHGSFLSDCLKFSLETLDYLQHKSMDL

STFSKVEFAFMNKDCCLQVSSLLLFMTDDVKTDLNIARVVDIILEFMCAYTDDSYLFYEG

FLVSIMVFASLLNYYTEQLTTELLNMFPIFISNKFVSNDLVVRISKNFSLGLPPLTEDAV

VSSVYDITNLLGENSMAHLASLNQRKAVVDDSINSKFLRNFHEQPTFDGLSLDEPSGNAT

TKKKTPYQEKFGLKKRANTLTTLQSMNIFKNFAEKNMLDNLSIQSVDGSASVSNGATAQR

DDASDETAELFKKMTLDTKDNEKNDKWKFWNNVVTALVHVASLYNDQSINVLTITILTQK

FGTVSETLDSIILHKLPILVNQVGETEFQLLLKFIQYHEKNAKTKAGITSCMESRCEISK

VLHQLGPKHPLYMIYLKDLLENIVAEGENQASETLSLVDYDGRSVSQKDGLARPELQKLA

SSYRSTRSRGHQNQHTAKTEHEVMSSGGRIMVYLKPVSLLLPGLNERKINYQKDLDETLV

NLFRNSWFNMVIHGFYYPFPQLDRRLVSKNSSAHGSLSTMSGMRDKYLQVFAYNSPPLAS

NYPKSTRETSFEMNTILRRDTSESNLKLQNSVIHHFYKLENPLISNNSVSNQVKTLFLSS

SIMLELYRCDSLSDESVSSCILYLTDPQVKSHFSKFFQNFSLELVKWVSKKAKLTKNAAS

RVPFDLQKMLICTSSRDVDVQKQSFESCNSFIQHCPDILIRNETSIFTMLGLLSVVFKSC

VDMKQTTMSPPFQYTIHTGDTHTETIFLPMNDTWRIQTLQLMEQFYSKWISKSFKYYQDD

IEILLQKFISLKTKRGNNAGRVSGHADFYTDEIDYAVSFAMTHATGISTREKPILKTVFP

PRAYHPSTGTSVTRFDSAYKFLSKQSWSSSFWSDNMGFFSRDEIVKTRELMVRDGLHLSQ

DLIEYLNLTSTLLCIGGSDHQDPQDGGLRLIRELFEVSMVELSNSSIDFEQQMDSLSVIK

HSVDIWANVMREKTGFKNLFLSEFIHCWSLAFSGGFESCMGSSFPVFSKKFEIVGSEYNE

MEYKPSGTEQIQHRDRITMAHFEKMHHVLQFWCQFIKKEYSGSSSSGLDATVHVFLSVIA

SPKVFATLSTHPFSLKIRLDILEILLICIEKLQGDFTQLTQLCNWLVYDGCLWFSLPNGQ

GSVYGENEIEMIFCVEQLQKIQTKLAYCFNKIAKFVDPLLGDASSSKNQVIILQKMVALE

IKNLRDWMSLDNSGGVMSIELGDSGANNTDITESLVKTSFAMDCGLCLQLLVRFRLGEKF

GALVTRLLVTMPHVTDSVLRNGDLILAYLLSSGASKDVSRFLYKNMVFFKPIAPIQSINI

FKMLAQGGSTKSLNNVILQFNLKSLEYHDSRVVFFYIPQIVQCLRYDPLGYVEKFIIDTG

KVNMYFQHQIIWNMLANMYKDDEGVVPDEVLKPLLDKLYNKILNNFTVREREFYDLEFGF

FNEVTAISGKLKPFIKKSKMEKKLKIDQEMALIEIKPRVYLPSHPDGTVVDIDRLSGKPL

QSHAKAPFMATFLIRGVASTEKSTTETSTTETSTTETSTTETSTTETNEGTCTEITSSTR

SPSSNTRWQSAIFKVGDDCRQDVLAIQLINVCKNIWEHYCLDIYVFPYRVIATNAGCGLI

DVLPHSISRDMLGREAVNGLYEWFVNKFGDENSMDFEIARLNFIKSLAGYSVISYLLQFK

DRHNGNIMYDDMGHILHIDFGFIFDIVPGGVKFEAVPFKLTKEMVKILGGSKNSKYFLLF

EKLCCQAFLQLRWQHEFIIKCIEPMLDSGLPCFKANKTIKNLKNRFVLGKDDLQAAQYMK

HLINKSYESVYTKGYDEFQKLTNGIPY

>model.g310.t1 Augustusgene.g310.t1 JFAV02000098.1:3277-4050(-)

MIILERLNDLLNFKRTGFDECLVKNDHLPIESILENFQKEGRSFEHIEIAEVEKKPLLQE

YERQLEIVQKRYQQILLLNNNPLYSDLKLKEKHTINQRNGSSGCPPELDILSTEITGQTV

KLSDGKRVSRCVDCSIIVDPQVCLQKPINIFDCRNCKIQIDCVQQYIGSVYLNKIKNSEI

HLSWDPSLKNQIRMSNLENCQIRISKGELSESTEDFVVILEGCENLVFDIQSQGTVTIKD

FDNIMGNKKSFSFSELPS

>model.g317.t1 Augustusgene.g317.t1 JFAV02000098.1:24423-25235(+)

MLNNLAVANSACSDNDKCVEKLQLIYSSSDEDDASPIPVTHRVPGNSCFAQCFGLQKESG

FETKVHSIKSKPQKSLLDVHWSSQLNHYQLAEYDSEMESLSEFETLQNQAPEKVKKTVSF

AEKHEKHIFDPDGIIETRQHSFPKSNTMINLKSILRLKNNPNAELEGKKALESGEVSVYD

ILQDVISRHKACKTREELANIQAHQNLQREKELVNYFICEFSSIMETQKFGRATLVSESL

RATEKNFKLRLDFAKFYSATGDTEMNKDIWA

>model.g319.t1 Augustusgene.g319.t1 JFAV02000098.1:26377-28170(-)

MDIFRVLTRGANIKNTSNTSKKTTNQLKKQTDNVLTNDESQNKDLSKDLDFFHNKKIVSK

VAFSKQEQNKVQKESSSDGKNQSESENALGQEDTAEVPPTILSREDAMKLRKTYKGNVTG

SDTTLPIGSFEDLISRFQFNGKLLDNLITNGFTEPTPIQCEAIPATLLNKDILACAPTGS

GKTLAFLIPLLQQVIDESRTTGQNKKSGKTQKNVTNGLKALIISPTKDLANQIYEECIKL

SSRVVLNHDSDNVEQQRHLQVSILSKSLASKLKQSGSANNKTRKPIDILITTPLRLIDMI

KEENALDLSEVRHLIFDEADKLFDKTFLEQTDHILAHCTSSFLQKSMFSATIPSSVEEIA

STVMSSDMCRIIIGHKEAANSNIEQKIIYCGNEEGKLIAIRQLVQEGEFKPPVIIFLESI

TRAKALYHELLYDKLNVDVIHAERTQLQRNKIIENFKNGSLWCLICTDVLARGVDFKGVN

LVINYDVPNTAQSYVHRIGRTGRNGRQGKAITLYTKQDALAIKPIVNVMKQSGSEVSEWM

EKMDKLTKREKQLIKSHKNKQNQDIKRKQISTVPRIVKQKKRQRVDMIEASKKRKTME

>model.g309.t1 Augustusgene.g309.t1 JFAV02000098.1:753-2087(+)

MPSHFDTLQLHAGQEPDNDNNRPRAVPIYATSSYVFNDSKHGAQLFGLETPGYIYSRIMN

PTNDVFEKRIAALENGIGALAVSSGQAAQTLAITGLAHAGDNIVSTSYLYGGTYNQFKVA

FKRLGIETRFVDGDKPEDFEKLFDEKTKAVYIESIGNPKYNVPDFAKIVQIAHKHGIPVV

ADNTFGAGGYFVKPIDHGVDIVTHSATKWIGGHGTTIAGVIVDSGKFPWGKYPEKFPQFS

KPSEGYHGLILNEAFGPAAFIGHVRTELLRDLGPALNPFGAFLLLQGLETLSLRAERHAS

NALKLAQHLEKSPYVSWVSYPGLESHPYHEAAKKYLTNGFGGVLSFGVKDLEDEKAIAAA

KEDPFKDSGAQVVDALKIVANVANVGDSKSLAIAPYFTTHQQLSHEEKLASGVTKDLIRV

SVGTEFIDDIIADFDQALKKVFNDK

>model.g335.t1 Augustusgene.g335.t1 JFAV02000099.1:38637-40616(-)

MKTTTVIPISLALLAATANGLVIPNKIEKTNTDNDNTDVAVAKKGKGCHGMKDSSSSSSS

SSSSSSSHSSLKKLSFESATDLFRQMVESSTETVESLADSASETLQNNYHFKQEDIKDFI

GKLKDQIAKVSSKSKNAPKDLATFLIQDLIDSASSRGVEGLEVAPMINKNEFNPKQQDSH

PLIPNRYMVVFKKNVSLNEIMFHQELINSINLDFVESTSNENYDFAQPNEYGIASVDKNG

GIENTFNIAGLLQGYVGYFTKEVVDIIRMIPIVDYIEQDSMVFASDFDTQNGAPWGLARI

SHREKLNLGSFNKYLFDDDAGKGVTAYVVDTGINIEHEQFEGRAKWGATMPVNDEDEDGN

GHGTHCAGTIASKDYGVAKNADLVAVKVLRSNGSGTMSDVVRGVEYAANAHIKASKDSKN

KNFKGSTANMSLGGGKSPSLDLAVNAAVDTGLHFAVAAGNENQDACNTSPASADKAITVG

ASTLSDARAYFSNYGSCVDIFAPGLNVLSTYIGSDSATAVLSGTSMASPHVAGLLTYFLS

LQPDKDSAFYDESLDKVLTPKLLKQKIIDFATADVLDDIDAETPNLLIYNGAGEDLSDFW

GPKKNKSLETQETTEHLENTVEANKLGSQFSIDTLVESIGDSTDNLFEDVRHALKNLNIL

>model.g332.t1 Augustusgene.g332.t1 JFAV02000099.1:32098-35199(-)

MSLLNTKSISSRGTAVVNKPSAPADRQHDTNSAEELNILVAVRCRGRNDREAKSKSPVVV

TIPESDESDKKPKEICLNTTGDIGITAQLNAKTYTVDRVFGPNSGQERVFTDIANPLFLD

FLKGYNCTVLVYGMTSTGKTYTMTGDEKLQNDQLSPEAGIIPRVLFKLFETLEQEALDYM

VKCSFIELYNEELKDLLVDGTASDNAHKKLKIFDSSLKKTRSQTPELTHSYSQPRAFRSV

SLSQPNLSSRFQSSLKNSRRNASGSLRRTQSGTYDDLSSTTSLASNTASGNKFDGVNNHS

KSDSVGLENSSGIFIQNLQEFEITNAKQGIKLLQRGLKLRQVASTNMNDFSSRSHSIFTI

TLYKNHNGELFRLSKMNMVDLAGSENVSRSGAINQRAKEAGSVNQSLLTLGRVINSLVDK

SVHVPFRESKLTRLLQDSLGGNTKTALIATISPAKINSEETASTLEYATKAKNIKNKPQI

GSVLTKDILVKNLALELSKIKTELYNTKSKVGIHLDSEQYDELMRDMENYKTETQENSRC

ISKLKQQNESLAKEKKNAALIIERQTKEIKQLEGTLSYIYDKMDKQHKNELNLVDHAKQL

VKAINLLKDSKRFYNEAKQKSKSSLSDIVRTSLFVLQEQALKEFSSLEKQASSYATFGEH

SFTNIKNGFAEMVSKMSESTNNLCSTVIDKIMGDNSALNESILSSLNSLDASISEHVQHT

NQSLSVISDYCNDCNYYINEKLFTSANEKVISSSAEKAYAQLQSNSSQMVENFQKMMEQQ

LKSSKKIIFGSLNDVTAEVKRIERANFEPNKKKLLEAIESINKCDSSNIKFEETYKKNIK

TLSASMDKYSETSSKVEEYMSTELSHLEQYSANLIKDSSLLTELEKLQENHVASVSTVAE

SLQSAETIKSLATKIEAALVELANEPSDSQQRKNEVLLQDILSDLESKQFKPVESTGKTP

SRPQYAQDATVARTLLDPIEEKNTSNEGQTASKSDNYSTALAKENGSPVAPVHFKRRSTS

SLDTDMPAKKKTSF

>model.g339.t1 Augustusgene.g339.t1 JFAV02000099.1:49974-51146(+)

MSDVEDHQPETVEDEYELWKSNVPLMYEFLSETRMLWPTLTTEWLPSAADSLRQELIIGT

HTSGEEPNYLKIAAIELPIDVLSVKQSVSDKDQPHASKPKLKIARKFEHELEVNRARYMP

QDSNFVATINGKGKVFLYNRSVKDKKNALVSEFEIHKDNGYGLSFNPNTKGQLLSSSDDS

TIALWNVESSLIHLYKCHSDIVNDSKWHNFYSYYFASVSEDKTFKFHDTRTSAVVNTIKT

GVPFNTLAFSKHSKNLVAAAGNDTLVYLYDTRNTAKPLHVMSGHADSVTSLDFASHKDGI

LCSSGEDRRVITWDICNFGAEQSPDDAEDGSPELLMMHGGHKSNVSDFSINPNIPWLMAS

AEEENVLQIWKPSGKLTHPSAYTSFGISDLE

>model.g341.t1 Augustusgene.g341.t1 JFAV02000099.1:52605-54158(-)

MLARSLSEVEKTIALETLNNKRNGILSAVVFSSDQEEALREDDTATLASIRSYRVGSSLL

KKALQLLVYENPELQVCINDDLKYEQIKTINYHDVLNKLDFDNVIDEKINCYHSIPLYLL

REIFNKTKFELNKPLWRIFIVDENMLVFHGHESLFDNFSVMNLQKKLYNTICSLEPQKID

QQRSGQALFESKSAQNFDFPKSIFDSSKLYLPAVAKDLLQLQTQSFFKNVYLQTIKKPID

FFNSTPQYEILNHKTSIVFNFNDLCGNTIFGNISPKQFNNLKKALELENISLRTFMASLS

MMCLQPFTASMNKDDFISFCFPMDLRKDLLYYQKDTASLGGLVYKKIIVECPLAMISDSA

YDNATFHNGYDPKNVKLSKTDPKFAEKLFEYKFNQVAAHIQSSIDQRVRAWRRNKFNDDD

FKRMKFGKKDGQEQKYFEINDLTAFKLEDKPIKGKTCEKYKPKEIFFVKSENPESFMSLS

FTYCELSGMNICIQYPEGYNMDDYVSNFETLITRLVES

>model.g330.t1 Augustusgene.g330.t1 JFAV02000099.1:26701-29592(+)

MNQISPDFKWVVSELLLKLQNNDSKSFTRTELFEYRERQVRFFIKEWRLTVGTDFYPLFR

LLLPQRDSRQYNLKEATLIRAVCSVIGLPKKSELELNLLNRKKGTKKDKLSVLLANELSQ

RKLTNEKGSQITINQLNERLDELSFRALGHRFGYQSLSKDPAFILFVKNMSAVEVSYLFD

ILLKYRVLSSTLEKKLLHAFHPQAELAAGNAFAPHLCKKIDDFKEILAKMGEENFFIEEK

LDGERMQVHYLDYGSDIKFYSRNSLDNSFLYGNSTQTGIISQYLKLHKNVRNCILDGEMV

TVDVLTGDILPFGIVKSAARKEIVAERASSASRGNAMDAEEGDNAWSSQEGESIESINKE

YRPLFVVFDVVLVNDVSFEKYPLYKRKDYLENILTPFPGRVKINKYYEASTEQDIKDALD

HAIRVDAEGIIIKNRVSSYKIGARNDTWIKVKPEYLQYFGEEMDLLVMGKIPQKKPAYIC

GFRVPIRNNKSESDEDNISNDSFDESETYKYISFCKIANGISHYEDELIAAKTKNKWLDY

KHEKPTMLEFGTEKPVQWIKPEESFVLEIKGRSINYSEQSVKKFKAASTLTGGYCRSVRF

DKDYKSCATFASFFNSRKGTETQANNLLSKKFQKNKRKNDTGRKRISLLDTEHLPKKLKL

DDYLFQGLEFYVLTDYFPEGGSKIPKAIICQQIILHGGKVINNVKSNTSSLRVRILSSKR

TYECAYWIKKRFDILNISWVLDCIKSKRLLHLEPQYCFSVSRSLYKKSLKMIDKYGDSRQ

TQTNKEMLSDILQSYDAKDPILWDKEKYKSEMQDLSPLPLLLFSGLKFYVATTGTPAALV

LYLQNLIKMFGGELTAEMGKSHIILIAFSRDNKCYSADSIAKAQNCVSDLLHRTESIREG

KIPVVLKAEWLVKCVDAMLLQDLNGFRVTDHDK

>model.g337.t1 Augustusgene.g337.t1 JFAV02000099.1:45449-47257(+)

MIYNKEKLRELYCKYLKDKNANFEYQYGTAGFRYNHTLLDPVMFTTSIIAGLRSLTLDGK

AIGVMITASHNPPQDNGVKIVEPMGEMLIESWEMHSTQLANSLQTFEIFEKNLCDIIDVT

VQKTIVEKSLIKDNLKLNIVLGKDSRASSDRLSEIVYATITSVFSNLKVKVKDLGLVTTP

QLHYLTRYANKKNVDIASLSLQATYFPHFKNAFTDLLKLHYENIDPKQVHDYLSHFPFGR

LVIDCANGVGSYQFHQMIKDDPWFRSFVEIINDKYLVPSSLNVNCGADYVKTNQRLPENV

DGLLPQTQGDSLFNLYCSFDGDADRVVFYYLKQLEPQQFDDPHEKQQFHLLDGDKISTML

AKFFHDMLTQAKIPQDELRLGVVQTAYANGSSTLYLKNSLKIPASCTKTGVKHLHHEAVS

EYDIGVYFEANGHGTVIFSPKVYEVVEARLESLHKSSTKHEYEQEQEQIIALKTLKLFTE

LINQSVGDAISDMLSVIAVLIISQQNAEDWNKEYKDLPNRLIKVIVDDRTVFKTTNAERQ

LVSPPGLQKKIDEIVAKYGPLARSFVRASGTEDAVRVYAEADSTEKVEALVAEVGALVKS

SSS

>model.g338.t1 Augustusgene.g338.t1 JFAV02000099.1:47655-49619(+)

MAKKASKKDKEAKKARAAAKQKKNESKAEVKNKKLSEKKKFVDEEEDDDLDIETILANFK

KEQQAFEEVSIENIERPANRISPCMFANPIHGKSELLIFGGENTVLKPSQSNKSATTTVF

YNDLLSFNTANHVWKKITSQNSPMPRSSAASCAHPSGIALVHGGEFSSPKQNTFYHYSDT

WLLDCSTKEWTKIDQKNGPSARSGHRMTVWKNYILLHGGFRDLGTSTTYLDDLWCFDITS

YKWKQIGFPSNHSVPDARSGHSFIPTQDGAILWGGYCKVKAKKNLQKGKILSDCWYLKMS

SDLGNIRWERRKKQGFQPSARVGCSMAYHKGRGVLFGGVYDFEETEESLDSNFYNDLFSY

QVETNRWYNCSLRPQRKAKKVAINKNKNKDDELEEILNSILKKNNINTDEEDSEAVKSEL

AKLNDESDAEESDADEAAEKPETTFTTKLPHSRFNAATTVVDDNLFIYGGIWECGDREFS

LDSFYSIDLNKLDGVTVYWENLDEVERAEQEGVVDSDYEDEEDEEDDEDEEDEDEDDDSE

IKDEKLVYENEDEEEEEVAEDEPEIPDPRPWLPHPKAFENLRAFYLRTGADFLTWAIANN

RDAKGKHLKKHSFDMCQDRWWERREQVRIEEDQLEELGGVGEVIEKDASKITKRR

>model.g323.t1 Augustusgene.g323.t1 JFAV02000099.1:6349-9040(+)

MASKLTNRVPPSDLDKSKIGQLKTQMADAAHKTETVIAKAFLATWDELPLWQKDNEYIIT

GYVRETNNFIKTFQSLFYLHNESVNIYTHLIPGLIFLGIVFLSLDNLVVPIHSTTGVTDY

MMFNLFFAGVFTCLLMSSTFHCLKSHSERIAIMGNKLDYLGIVALIVTSMISIMYYGFYD

CKKAFYCFSTVTLLLGIACSVVSLDGKFRSREWRSMRAKLFVAFGLSALIPVVGGIFIYG

LAETYVKIGLKWVLLEGVFYIIGAVLYGLRFPERLSPGSTSTHLKSEISNKQIAATTMAV

KFTKRPWLGIAIPCLIISFIGYNAHYFILQNFLALHKQLIFELQMCMIWISYYLAIITNP

GTPALSPRANKTEELENFCSKCNRVKPQRAHHCKTCKQCVLAMDHHCPWTMNCVGFYNFP

HFMRFLFWVIVTTVYLLVLLVARACQLYQIRSRPSYLIHTSEIVFLVLLIPFDFFVFFTI

FLLFVRCIRNQIFRGMTQIETWEWDRIESLYMNKRLMPLMLDSAIKKFSITRSPRVDEQI

HYLLKNQLKIPMDEFVNFPYDLDICGNAKLFLGPWWSWLLPWGTPTMSDGTYFTKNELYE

YDENADIVDKLLSLPWPPDGRRNTSLEEAASSSMQSLGSIYDSVEQELFIKKRADSRLKL

KRNEWFNDWGESLEDFGVDVSTDS

>model.g349.t1 Augustusgene.g349.t1 JFAV02000099.1:70452-74138(-)

MSLITTPRDEDGDIYILDSPPPQESVNLEESKTSNAVSTPSFQRTIMVPNKSFPSNEFLG

NTPVTKNATHGNNPHFNNHMDKILTKNRANSEKIQRTPVIDFTKDDSSLPNTSTGSFTHI

NSPINELQTRVYDVDNDSFVDAPVSANANLSSVSSASPSLSASSSLFSSIVPLSSTAKQD

HDDSLAMQQSLSHINQKFKKYDELRAVNSTKSKVLQIKITKKIEKLKEYQKEIDSLPPGN

DPTRNQEIRAHYAAKIKFEEEKLKINQQKLATTNKNLSLIQNGLNTLINQKTDVKLKLDK

LKNGLNHTPSEMAALKKRTNDIKNALPDAAQQKRTAQEQLLQQHLKLNNIPTDQSPVAIK

QQEQQQVPKPYSPYANYIASTTQKLQKTIENKTKLNQMLLQLTRMFQNKMIPTGLYNSRR

ENIIKNLNVLNKEESNNKRLLNQLLAFESGYLRMNTNNTNQPVLHNRNIPNYMSQYNTEK

REEDEAQMQEYTESLGINPTAFGGVYSAEERESIRSFLEEFKTRETEIEGETMTPEELTV

NLMKHQRLGLHWLLKMEDSVKKGGLLADEMGLGKTLQMVSLVLCNRSEDKDCKTTVVVAP

VSLLDTWQGEFETKVKDSANISTLMFHDKTKVKSFRELSKYDVVFVSYHTLRSELTKTWP

RRVSPNGVYVDRDGTERAIPKNGMDLESLLSLKEPGEYYSPFFTMDSKFFRIILDEGQQI

KNKLSKVSQTCCTLMSKYRWILTGTPIQNNMSDLYSLVRFLRIPPYNKETYFQKEISNPL

SLKKDSSSATSKSRVAAMNKVQVLLAGIMLRRTKHTQIDGKPLLELPPKTVSEEQCSLVD

AELEFYQDLETKNKRIAEKLLKRHAKGNYSSILTLLLRLRQACLHSELVLIGEAKAEQAR

VASGRDFDKDWVRLFEICQDIPQSIVSRTNDFVETDMICERCTNPMELSLASVFPSCGHM

ICFECLKEYKEDCSERGVSKYNNAGQLVFPCHICQTHQVEDNVMSFKFYDQVVNQNYSLR

ELRDEFDVEQRAAKEKLKEGYEIDFEKLEPSPKIEQCLDLIKGIANNDPTEKVLIFSQFT

TFFDILQYFIGKNLGMRSLRYDGTMNVRAKTAVLKEFQRDPDTNILLISMGAGNAGLTLT

AANHVILADPFWNPFVEEQAMGRAHRISQTKEVHVHRLLVRSSVEDRIVELQNKKQELVG

AALDNKKITEISRLGSRELGFLFGLNQLS

>model.g346.t1 Augustusgene.g346.t1 JFAV02000099.1:65353-68058(+)

MGIDQTSKTRSFSTDNIDEDPLAPQHSNESVCSLETQNSLSFGNKSFFTPNIFQVIRRKS

GVFSRKNSAFSSIDDFNKIVPPAAKKNAEKIATSRSRSSSISSELSSMNLMRTSSSGCLT

HSYHFDAAPAQQLFENGGYTLYGTKDHQTKCRYNENTFQQGVSLIRVTRKKSVNYKFTLI

NDQLLHWKDKYIDIDTIKDIRVLDSANNYREQLGVSSEKGQHWITIIYQVPPNKLKALHL

YAENAEDFQFFYAAIYYKTKQRHDMFQTMAFPSSNEFTTFHWNLNKTAVSTKSDKNSQEN

THNLNFENVATICSKFNIFCSDSYLHTLYKRADLNDDGLLNHSEFQYFVKLLKNRPEISR

IMQDISKNGKYLDFDDFKKFSTNIQNEELAENDLEAVFEKYKHGKLGIITTEGFLKLLAE

QPYLFAVDSLSYYDRPLNQYFISSSHNTYLRGNQVGNSSTVESYIEALQKGCRCIEVDVW

DGENGPVVCHGILTGSISMKSVMEVVRKYAFITSPYPLLLSFEVHCKDANQIIMIYLIKE

YLGDLLAPKFETELPSPNSLKNKIVLKFTKRGKIYNSEGLPSSTTSLESSDDEDLLEQKS

RNIKNPFHLGKKNKVAILPMLLEFSNIEGIAFKNFSLSESKTPAHSFSFNEKRFQELTRN

KYQEYAVDKHNRKFLMRVYPHALRYKSTNFNPIDFWRLGVQMVATNWQTNDLGQQLNNAM

FLSPMAGSSHWNSGYVLKPDYMLPPVKIKQMSQYRNSVQFSLLHVEVDVISAQWLHKPSF

SRKDSPDTLLDILTEVEFISDMKVKSPITIQSGNAISDTKAQTKYIRENGVSPIWNTHVS

IALYDTGLNFVLFNVKTKNSIVLGTCCIKLHNLRKGYRQIPLFNAENGEKFIFSTLFIKI

DY

>model.g347.t1 Augustusgene.g347.t1 JFAV02000099.1:68210-68863(-)

MCEPVFSTRISIKWESDPKPHEPTSTWVFTAGNEEFVDTRINMANSNPDWLITGYEKSLA

TKAGYESTIMFCHDLDSLCKPGETPGADVGHFKSIEGSSDRLEEGAMMNPDLGKTLEYQE

VWRTLDPIYSTVSELKPIAASTKQVESEVWKLENGKGVFIKIGRFAQGVAVNANNEYQCI

RLFEKDVIYQHGNDSSQVFHPFIAGHLQQSSWVKTFQG

>model.g328.t1 Augustusgene.g328.t1 JFAV02000099.1:20913-24305(-)

MDAAAKSFQSSTNGQKPAVPHMLNPIVDTIFSCQDPSTFEFANTFKNILDKDFIILVPPA

DSVLFGKDAALVKTDEKCKTSNYCSRELLELHILIPLSKEKIRKHKGSSAYCLLADQRNL

VEISNCELRYKAQTCKILHTQYLPIANDYFCNKSHILLLHIDNCVKFEQKKPNPSVVQHL

KPATKASEQSSLELSKHKLELIKQEISSFELLLALNPKVKHELKSKIFHEYAVLEHTSFQ

SAFDMASVYFGALNKVIEWCLDEPFFEGYNQLGRMVYKYIESQFSNMILQKLHFFIKETG

TNMCEISPIFLNLSFVDIPSFSEGLQRDFVMSTREMIVFERNLNNAIPIFQKMITSKNCD

TKSDYLLSTLQELSRSQDADADVLVGLLILCICRANPKNVYGELLYLQNFMNDTSKVTFG

SNAYAISTFEVAVKYLVSSENNSYLIARSVRNKEFWNVLQNGKYEDFVRYIDSQTVSDVD

DILLGRHFAGKSCFSLMVENKSDGHRMFSHIILDSQKLKAIDINRLLDDQSSNNETLLLK

SVKLRNDHFTKVLIEVLLSACTDHEIEAYVNAKECDTNRTVAHYIGLDCENLLQLGHYFD

WEILDSQNHTPLFALFRFYDHLTYNSLVEQGFRACLFWYQKKQRQFNFSSHMDTKGNTLL

HIAKSNIAALLSCLPTPPLNQQNEAGLTPLMFYCRYSRLENIEEIIENGKSSLLYVRSKG

NQKYLGYSCFDFAKNEQVLRVLGKYSLCSKDIIGKTKILAIRCEKACWFVYLVIKTSESK

IEVVKHKFKTLKAWVAEYFRQHPKSFLLRSKDFMEIFPDSLSSLSGIFSKFEFDRKLMNW

GQIITAINIIQPDFLKLFFSTNDAHVSEQAHGSDGGCNALTRITSSCAQKVNTTSEEVFS

IKSFLKMSLDECVALQHYLKDIRKLLVLKMMKHFDLEYAKTLLSGHLFRKEKSQQELYCD

VFKSEYFDTLSTSCVSFLLECLDAIVANLQNILHGSLVNWWKLYGEYLQLQRKLQDANFC

LSSAKKFGIPSTTNDEIVPETVNNCYSVQNNTSSKFSSFFDNYFEGNKRRYRETLITDLS

LEAQKLKVADTFLRKDYKHMAEEMSKFMTMKDTFLHSYCTVEIASNAALFC

>model.g342.t1 Augustusgene.g342.t1 JFAV02000099.1:54687-58016(-)

MSQPSKEKNPVTKVNRDLFYTLASDLDFERAQSVISIIDQLTSIQKHDNDTFIKEINYTI

DRLITGLGSNVGSARLGFSLCLTEIVNISLEDGITADAHQYIKKLQEKLPLNDSSNESKK

KVKGKDERGFVFGRLFGLQVLMNEPLFSKLFVLDNGKEKTLNNGFLVEFASLLIDLSSFK

SWIKETALFTLYQFLHRLEQTGLNDEQNVVQILGLLDSANITMTMEGLAIYLLFTANEKP

YDLTLFKPKNLHWENNDPLNKTNMSLLVKVMKDVPVADSDSSGDVQLKQKGFWTPRLHFV

FPILIETLLKTEDREEQDLEPPTKKQKKNDKSGKKHKKQVAAQKRFTIDDLYKNVVDESY

FSEKSSNERKYIGFLIFKLCLETFPVEKVELLFKQQNFLRTLINHSSNKQRMLYKLCNSL

LNKQIPAILQNQPKKITPILENIWFYKHNGSMINFDQLTKSKFTSSLLNHESLKEECLVA

LSEMLIHHLHKFLENIYLLKSKDLAGDEVANSEDYTKNLGMYKFCIDSLLHLVRSHKAQL

SEKWILPIIKALAKISYFRDRKEKTLPATLNDREGTKDEAESAADQNDVHTIALERLYSI

LFDVLSSQSEISLKEKPVVWSLQLLEEITSIEEQLGAGFLLHSVDSSLAQIKNAALKHLQ

DIQQSLSFGKTTDGMKNVLQGLQQLLAINLLQLYSGNSEAIAILEDLSSFYADYAEKSSQ

DGVHGVVNFVSITEILMSSMSDDKAIFRKLSLNSWEMFVKEIGTQELEILLETLTTRENK

SGFSQLFGGDAELEDGSDEDGEEEYDVDDDDASEEEEVEEDDEDADTEFSDSDNDDNGKH

TNEKNEKSSKIEKEAASALAKALDLPEGIVDENGDVNHDGYVDMGSDDGSGEEEEDEDLD

DEKMLELDKTLSMIFKTRKEALSGTPTGNQRKIEVQKARENVINFKSRVVDMLEIYVKYI

DKDASSDQKLENLLYMLPPMIQCIKMTLSKPLVDKLGKIIKTKITKIKVSAAPLDAQLID

VAVTTLQTVHKLALSSKSGQFSQLFYSVCSICSLFVSKIISLASENKSFDTVIEIYSDTM

KEWSNNNNCKIPATFFSDYVNMLATKKQQK

>model.g334.t1 Augustusgene.g334.t1 JFAV02000099.1:37441-38184(-)

MNPAQPAVYPEDPPPPYPNDIDGAFTLRVDRNAPRVLRHNIGPSSHAPSSQPGASSSQTT

EGKLGKRQRSLLKVATYKKKFKDMFLSQQDPVGSPASHAQRASPRETARTSSFSSRSNVA

RNTAGSNRAIRNQRSETQSIRPTGGSPAGLRKATAFKWTSFCKKFNWRSKTSGRLSWKAI

DLFLQALANSLWEDAQRHHENFYERSSVQRENLVLRYRVFATLSVVLLVWFYLKAADHAV

QWIRLLFV

>model.g322.t1 Augustusgene.g322.t1 JFAV02000099.1:3562-5811(+)

MSTPKKDFSSLLTQVTSLVKTTTALASQDIEFHSAVQPAVQDTVDKTKERILGLLNDLFL

NIDEQSETLAFDEGLSVNESWRQVGDALDTVLEKADKYIDETKRARNSLVNEQSLQYLDD

AANSKVSSTRKRITKPQLAFATPVDNSETGPFKPLLSTKPHALKSLQDSLVLIPAEEGVP

EHYGNPYEYEILNQEYNTSLLEKSEPIPSKDWTSTKGLWVDSVTTLRAMIESLKVQTEIA

VDLEHHSLRSYYGITCLMQISTREQDYLVDTIALRDSLQELNQVFCNPTITKVLHGAFMD

IIWLQRDLGLYVVGLFDTYHASRALGFPKHGLAYLLETFAHFKTSKKYQLADWRVRPLTD

PLLAYARSDTHFLLNIFDQLKNKLIEAGKMSEVLYESRGVARRRFEYNKYRPKVMSGNVY

CTEERATPWKSLMNNYNIPYELEPLVISLFEWRDAVARREDESPAFVMPNQTLVTLVSAK

PVDAAGVLSVSSVVPDYVRTNAKALANVVKSALKLISSGASANKSFSMEPSSLSEMSNVV

SRETVEYFSTLFASLKKNEDSEMCKVPSAQNVFDGSSKFFGNSEFWNNGFVSYELTKRVL

ANEKDKEDRKSEMYKAFEANDEKLAFGQPVAATEDAHHLETPVVAQSKPAIVQVKPQVKE

SKDEVVVLRSRNAQSQKKPSKKVDTFAADDVIPVDYGQVENVMEASKKHKQAPSKKRSFD

PYAVENEGPKAYSKKHKPNAGKNVSFKQKK

>model.g329.t1 Augustusgene.g329.t1 JFAV02000099.1:25453-26391(+)

MGKGKNKTHDSRSSHTSSRGSNGHSIRQNHKRMEMKHTSTDAEGKKRFPVKLAMWDFDHC

DPKRCSGKKLERLGLIRSLKVGQKFQGIVVSPNGKGVVCPDDLAIVEECGAAVVECSWAR

LEEVPFSKIGGKHERLLPYLVAANQVNYGRPWRLNCVEALAACFAIVGRTDLAAELLSNF

SWGLGFLELNKELLEIYQQCTDSDSVKAAEEKWLQRIEDEHRERKNQSRNEDIWLAGNVN

RKSKNEAHADDESEYFSSEYDDDNESDETSAVYDALGNIVEKKEAKYDSLGNLIENFDEL

ELQDSEDEESEEI

>model.g325.t1 Augustusgene.g325.t1 JFAV02000099.1:16030-17175(+)

MEQKAAELGEPNIDRRKSFSFNSFSKFTSFIIYFLIGILIVHVAFNFQETKNQNKQQEQL

EQLEQKNQLQKQQANMTRSVLKSFLAVEQSEGVGAKVRRSIGTGQMRNFLPFLMLDHFNV

QPPAGFPDHPHHGQETITYITEGMVAHEDFTGSKGVLRPGDLQFMTAGKGIVHSEIPVDS

GDGKSAIGLQLWVDLPENLKNCEPRYRDLRKEEIPEANPSDFLKIKVISGKSYGIESLQD

LAYTPVHFYHFLASKKGTAFEQHMPTDFNSFIYILKGSITIDNKLYPQYSSIFFNTDSDI

ISGVSASETTEFAIIGGQIMDQPVVQYGPFVETSKSKILEVIQNYQLGKNGFEKAPYWRS

SISDGIDKKRAHEFLEKDGLQE

>model.g331.t1 Augustusgene.g331.t1 JFAV02000099.1:30048-32072(+)

MNSFSDITPIHTVFYSVFHPTEGTKVLFEFPPNNLAKSDIDFDTIKNYIIPKPQLCNKLI

TFKYGSFRMVGYPVNVRASYYARNSYNFNIVFVFPYDSATTPYEPSIERLGKMLAVLEEQ

SQLLSKAQRDAVFYQLKRDVSATADPGVSTDLITQKYQELITEISSSKKQLSIPDFLTKM

FQDLNNYSECLIPIDAGNSIDIKLFSMNPAPTTNISIEDVPVTTVNLLKFVTPDWDPTML

KIVPYINGVNSIARIAKCSDSDTVLVIECIKHLIYYECATIVDIFQFQNVYAPTNNLSTF

LTNKILAAECQKYIISTDSHMMSLEFSKHHNSGQKRPIPVHQNSASSYASSKNRVPSFSS

GNSNQANASKKSFSASSFGSWNKTKSQDGTPLTLPTKSCIFELYLSLSQGQVLTEWYKRN

ADLIKDSCIDVRKFIVFGITRGLIYRCHSHPILKNSASILDTTSINFKNKLDGMKRKKKT

LNLFSTADIKPDIENMHDKQNSSTHNFLEPTTSGSLNGSKSKLTSAADLNPDKIADDLLK

EVYQKLTFKTHEPQGHNLITNPKSILYSTQSNSSNGRGLNRLKGRTSGSTSRSKVVFDTI

SNSEEALIDNSSVYTGENSRLQHQQEEKLQQDILALVNCLKNADSIDRICTKLEKNRDEV

ETMLANIGSYNIVNT

>model.g333.t1 Augustusgene.g333.t1 JFAV02000099.1:36025-36918(-)

MLVYNHTFKFRDPYQVLLDDQVIIETFKSNFNLIKAICRTLQVLDVKPMITQCCMQKLYE

TKDQEMISMAKELFERRRCNHNIKEPKEPLDCIYDVVNVNGVNKHRYVVVTQNIDLRRKL

RRVPGVPLVHINRSVMVMEPLSDASKEISERQEKQKLTSGLNDAKFAGFVKPEETEDASN

NGSAKSGAPLKKRKAPKGPNPLSMKKKQKKVPAHNHESKEKAASSPKDQDSHHVAEKSEE

KKSSLGEDSTDDAKNEGAVSNRPEVVATDAEGASEKADTPKRKRKRKHKSAKKISGDD

>model.g324.t1 Augustusgene.g324.t1 JFAV02000099.1:9181-14385(-)

MSFSSNHSTNNTNTNTNVSANASTNVNAILNNAGPVSNGNITPHHHSQHQRHHSASAGMP

SRAQGPVVLPPASSLNLNRHESMESNHSGGLGGNEGAVGASTSNNMFYRLPSINDVGHSR

KSSHGGSSNGNASMYSANVQLPSVSKTFMAGTASSLMASDDNNAKNMSANTFNNNTSDSI

NVSAHKKSVSHNDISHTTGATTTINGIPQSMSPNQHQHSLSFNSHISSGHQSPTLPSINS

IGHMQPSVSLQEHQQHQQQHQQHQQQQQPQPQPQPPQRANASSANASPSNSVEVPSQESA

DADANSDNYRPLNVKDALSYLEQVKIQFNHRPNVYNSFLDIMKEFKAQTIDTPGVIERVS

TLFAGYPSLIQGFNTFLPQGYKIEYSNNPNDPNPVKVVTPYTGGGAVDLNTTAMHEKHYY

EQQQQQQQLEQQKQLEMQQQQQQQQQQQQLEQQQLEQQRLEQQQIEQQRLEQQRLEQQRL

EQQKMEMQKQQEAQTNPAANSNGDVEFSQAINYVNKIKTRFQSQPDIYKKFLEILQTYQR

EQKPIREVYEQVTVLFQNAPDLLDDFKKFLPETPESHLQEQQYAEQQHQLQLQQQQLLLQ

QQQAYAEQQQRELAYGKDVSALNNNALAGVQQQPQLPPLGSFSPPSKFQKEEFKQPVVSE

NVESHQSYPLQNHIVTQGLSNDNIPVSNLREELISDAQSSQLYMQQQQQLAEQQQQHEAA

MYQQQQDTMVARPEIDLDPSLIPVVPEPLAPVEAEISLSYEATFFEKVKKFIGNKPSYGE

FMKILNLYTQDLLTKSELVYKVEFYIGSNAELMTWFKQFVGFEELPKHIENIVHEKHKLD

LDLCEAYGPSYKRLPKADTFMPCSGRDEMCWEVLNDEWVGHPVWASEESGFIAHRKNQYE

EALFKTEEERHEYDFYIEANLRTIQTLETIANRILNMTPEEKENFKLPPGLGHTSVTIYK

KVIRKVYDKERGFEIIDALHEHPALTVPVVLKRLKQKDEEWRRMQREWNKVWREVEQKVY

YKSLDHLGLTFKQSDKKLLTSKQLLAEISTIKLDQQSSLNNHPAAHHAQGNGGHNNSAKR

SHPLTPKSKPQLDYSITDYEVLYDIVHLCRGSIYNASSYSYADKDKLLQVLVWFLSTFFG

ADHASIKASLDSREGVVQNNKNGLAEYGENGQMDNILPADEEIINENGKRFRETEEDSRS

VFGENLSFKEIISKSKYKKLNNLDDTNERELPDHTKETESDLDQEIFKNESQKPWIHGTL

LDKVNENSVVTNRTVFNAFTNTNIYIFLRFFITAYERLVAMKEIDAKVTSDVNGREDNEY

VKDMTLSSAQLADMGLDFKGTSAYKRLLELAEDLIDGKIEHQWFEESLRQAYRNKAFKLY

TIDKVIQSIVKHAHTISSDNKTCHVMELLEKDRMSKNTSFKEQILYRLQVRNQMSPTEPM

FRIGFDTVTKHLCIEYVAVDGTTVQHGSGTDEKWKNYITSFALSHPTEGVLQDKVQSVVL

DQVSTEEAAFSKNTHSDGSASGDKVSDENEKGDADAKALEAKNSISGVSYSDVALEINKD

DYTLQTECGSQDVFSRASVNKFPRFSLTAAQTVDKNETVKTVHTWLDASVQNYQSQQLAK

SSDNEAHSDDTPVDQQSNAEPKSLQDGHKQEPSSTSETANVGISKSQESAASSGSEHIAK

AAHVADSAPINTSKDLAISHAEASSKREGSSPETTTSAAEPAESSTAFSPAREEN

>model.g327.t1 Augustusgene.g327.t1 JFAV02000099.1:19751-20722(+)

MSSNKTISALIIDFLNTVIEKKEVQEEFTDSLNVAIDCIAEAFEIEREDVKSLTGGKTLA

QLASGATVTTGSSAASSSKVEVKVEDDPKAKAEAETLKLEGNKAMASRDFALAISKYTAA

IEAYPSNPIYYANRAAASSSLKDYESAVADAELAIKIDPNYSKGYSRLGFAKYALDEPQE

ALEAYAKVLEIEGDKATAAMKKDYDTAKAKVESTLNLEKTEKEATPGSTPGGNPFGGAGG

MPDMSSFMNGAGLGGLGGMLNNPQVMEAAQNMMKNPGMMQEMMNNPAIKQMAEKFSGGNG

MPDLSSLMNDPSIKDMASKMFGPK

>model.g343.t1 Augustusgene.g343.t1 JFAV02000099.1:58339-58773(+)

MYEPISKSISTCCVKASKEKLISTTHDTKPQIPKEIDMSAVWRKAGFTYNTYASIAASTL

RNALKQEFKTTAVLERAQTDVKVLKFTNGVQADPVPLKKD

>model.g344.t1 Augustusgene.g344.t1 JFAV02000099.1:59780-62200(+)

MLSLPTHSRLVFQPSSFLIKNILHTEQASTLLGNRTSWRGFTGRALFHTNTLQSNNTKNF

HNTHNGIQSEQLRLLNRNLFSCSMKTTKQHFHISSKSLLNDLSQNNGDQKQNTKVAANKT

DTVKDSKSHPKAEANHEDMEEESQAGHKELLRLFRLAKYDWKLLSLALTLLVVSCCIGMC

IPKVIGIVLDVLKDNISKTTDISEIKIYDMITLPNFLWLFALSLLIGQICSFGRIVLLRV

LGEKLVSRLRARVMKVTLHQDAEFFDRNKVGDLISRLGSDAYVVSRSITQNISDGCKAIL

CGGIGVGMMFSISTSLSAAVFIFAPLLMIGATIYGKKIRVLSKSLQMATGNLTRVAEEQL

SSVKTVQSFVAENKELNKYNESIRQLFNVGKQEALANATFFSSTNLLSDMSFLIVLAYGS

HLALQGAMSIGDLTAFMIYTEFTGNAVYGLTNFYSELMKGAGAASRLFELTDRKPLIHST

KGQSFVSKGDNAACNVKVENLSFAYPTRPNNQIFNKLNFEIEQGSNVCIVGPSGRGKSTI

TSLLLKYYKPSEGRILIDGQDLSQISAKSIRRSLGVVQQEPVLMSGSIRENISYGLDYEP

TMDEIRAVAKRAFCYDFIAKFPHGFETQIGPRGALLSGGQKQRISIARALLKNPKILILD

EATSALDVESEGAINYTLGKLMRSKQCTIISIAHRLSTIRRSENIIVLGHDGSVKEMGKF

KELYNNHESALYKLLNEPKKKEKQPLRAPEKDYKAESPIEDKDKPQENQPQSKEPSSNEA

EKKIKQEVLEHIIEDIDNDVKSSKLHP

>model.g336.t1 Augustusgene.g336.t1 JFAV02000099.1:42354-43841(-)

MLRNTSKLVNSNVLKSTQQSILAKRMSSSSTSNQYRIETDAFGEIKVPADKYWGAQTQRS

FENFKIGGIRERMPEPVIKAFGILKKSAAVVNTELGTLDPEISKLIQKAADEVISLKLID

HFPLVVFQTGSGTQSNMNANEVISNRAIELMGGEMGSKKIHPNNHCNQSQSSNDTFPSVM

HIAAVLEIEQKLIPALVELKKSLELKSQEFSKIVKIGRTHLQDATPLTLGQEFSGYVQQL

ENGIKRIEHSLINLKFLAQGGTAVGTGLNTKIGFDTKIAKQVSLETGIEFKTAPNKFEAL

AAHDAIVECSGALTTVACSLFKIAQDIRYLGSGPRCGYGELSLPENEPGSSIMPGKVNPT

QNEALTQVVAQIMGNNTTITFAGTQGQFELNVFKPVLISNLLSSIRLMTDASHSFKIHCV

DGIKANEDKIGKLLNESLMLVTALNPKIGYDMASKVAKNAHKKGITLKESALELKALSEE

EFDQYVVPEKMIGPKP

>model.g348.t1 Augustusgene.g348.t1 JFAV02000099.1:69272-69982(-)

MVQLFQRKLIQALPNPPCETTSTFSSVTNAGHFISLRPFVNKAGHEDFPFEWCFGGLSKY

AQASQLDADTVQIIFTMELDTNTIRNVENVDKDPVTTKWATWPSGDVEETGKIFPFGKDK

EGVEFRELWQPINPDTYETEQKFNILPKSADASNFTSIALYADNSEFHGLITVVGNFVQG

FLHKKGTESSSFIRAAYKGGEYEYSIKFGPNVDVFPANFHNGLKVGEKVGIFEVIEA

>model.g345.t1 Augustusgene.g345.t1 JFAV02000099.1:62674-65043(+)

MPAHHGNGYHRMIKVKNLLAETHSVSGYTVTDYTSSKASSTKLSSPKLQAQGSRSQELLN

LSLSIQELQRTLKERVQLIKDRSPEDDSPSSLQMHYREIVNCLKDLLKKDHQFFELILFQ

KSQLSMNDYCKWLNQGKTQFHAYFESLSSIEEIVLEILDRVEQLHTKNEIFTDGEFFYEI

EKCSESYANLYNLCSYLKVQFSASSEFNEIYYNYMESIKDEIMLMFENLNDLNDTNLTLS

NNVPSLEELIRTLSSSVWSSKKYRGTMSGSSDTHNSSSNVSTSSSSKPGLVLVNAQDSLM

HEHYKTLLSNFDPLTTSLTEILALKIEDFTSTHKTADYLLELIDLIKIKYNELVAIYELL

ETELKNLKRKIFFDRWCQVFDFHFSGLDHIVNVELPSVLVTVKNFSSEKHPKILSFYKTV

IANYAQMADKYFKFADLIIGKNISHELTKKVTYKKNDLNFKWHKFVRDAPVDFAECFTKP

NEFSKTFLPKSSSPSKRNVQSVRDLMSGLKKTENITGSLQKTNVIRRNEGLTSKRKLRNS

RRLRRSLLSDVQSSKLSNVETDELLTKLNRLSTMENIEEWKTLSKEFGRQSAGALIHQRL

NIRPVVINDSDDLISPTKKDFTDSEEQLSDLENLLEADLNNVPIENDEETSDEAYRAHVE

KMKEVDKSLMKQASTSTLRISEEDRTLLGENFVLSPSAITFAGSRPTVSIFQTLKFKDTL

LHFSKLDTKIKAPFDSGLAGLFAVKSPTSEDFNNSEYSFHQIIQRLLKPESQLNPLTKHT

LRLPKTYKRI

>model.g326.t1 Augustusgene.g326.t1 JFAV02000099.1:17642-19336(+)

MGASPQKTETSAVEARNNTKKQSPTTITQTGIERKEAFYASPLYDFLSPFKLVTHQWEAK

YIIVLFALILKCAVGLGSYSGYNTPPLFGDFEAQRHWMELTINLPLSQWYWFDTHYWGLD

YPPLTAYHSYILGYLGVFLNNKKGNWFTLNESRGQEELDLKSFMRFSVILSEVLLYIPGV

IYLCKWIGKQTKQSPINQYIAAAAILLQPGLILIDNGHFQYNCVMLGLTVYAINNLLDEF

YAAAAACFVFAICFKQMALFYSPIFFAYLLRKSLFQWNFGRFLLIGLATVSSFVLCFLPL

YLLGGGLSNVVQSIHRIFPFGRGLFEDKVANFWCVTNIVVKYREVFSNEELKLYSLVLTV

AGLLPSMLIIFFWPKKHLLLYALASCSMSFFLFGFQVHEKTILLPLLPITLLYTSSNRSV

VSMVSWINNVGLFSLWPLLKKDGLQLQYVVIFCLSNWLIGNFSFATPKSLFPKWLTPGPS

VSQVSATYKRRSLLPKSVFWKLVIIVSYVAMGITHMLDFWVIPPGKYPDLWVVLNCFVSC

VCYVLFWLWTNYKLFTMRNQSFQSH

>model.g340.t1 Augustusgene.g340.t1 JFAV02000099.1:51340-51537(-)

MARGNQRDLARAKNLKKQQEASKNQKKDGSKQKRMESDADILRQKQAAADARKAAEELER

RKKEKR

>model.g355.t1 Augustusgene.g355.t1 JFAV02000100.1:15696-17330(-)

MPSLERLPLIERQDENCWICLESKPDAWEKYNKNNGWLDCKCSLTAHESCLKTYLLTAIE

DGRAKPLHPFFLASQSFIGDISHQDTELMYRTENHAQSLSNRSISQNFSGINKYFLNYYE

DQENVLLSLFRKGCMDYWRPSFWVKTKFYYIGCPQCRAPIVIRISEWFREQLELLIEKGN

LIARVGFTVAVPLYISQTAVQSVLDIFAKYGEFCMHLLIPNTVRVARLKAYKNILNGNDN

LAFQRADYLIFAASSFIASAFDWNVFFFIPWFGIVYKVRQSDFWICMTEASLYFLVAHSF

NGYKIMSYNSFAYMIERLIYCFCFNPIYKRLTSDNSADMFTGEGITIQAPENVSYFAKIK

AFCQKQASKVLEFLTTDYFPQTHNFYFGFLIELFLWPRIGCFINRNFFMHSRCAKAFLNK

FSDTPDEAVLLGNMAGASVYRIGKQALICAWQYYTHNYTQESATATVNSNNMLFFKAEGY

GQLFGFATSLPNSENQFPQTILFTKYFPRDLQGKGLSMGLTYTAFRKSMRSSVLYNFSRV

VNSVE

>model.g357.t1 Augustusgene.g357.t1 JFAV02000100.1:20791-22950(+)

MGLSNQSNRYTPQASPSLNKDTIKDSSHSVTDMKLTNKLGTQNLKKGNLSISRNAINNLA

YFTNKGILIKNEVPESANRAAGSATKSAMSGSSRDGAALQDDYEHGVEYNGVDSDDEDGA

DAYETTSLISCVTCLSGHSLSTLSFADSQTGYGDDNSRLLSSSSSSNSIYTDALTGEEDA

HGVKTLLNDFKIKSVHLPNNSVIPFSKVLNCKPVVDIEANSTVYDSTGNLEPVHLVEITF

AKDRRQDLVPKSSVLKVTHAEYSKLLKEYQTLTNSTDVKELSDLVSWKSFPQGEANRPKR

ILVIINPKGGKGKANKLFLTKARPILLASGCLVDYKETTYHREAYHFTKKYPFLSKYDVI

TCASGDGIPHEVLNGLYKRKHDRVELFNKISLTQIPCGSGNAMSVSCHGTSNPSHAALNL

LKGRPTRTDLMLITQPSYFTNEHGIDDDDEEPHRVSFLSQAYGIIAESDINTEYLRFMGP

SRFEIGVTSCVLRRKKFPCEVFVKYGSKTKKELRDHFNKYSRYVQSFPNSSSSLPLSSEE

DQSLVDIDSPFENTNSSSKANSDMLTEEDFKPEYEFPENSIKYTDDIPDDWEQIDSSLTK

NCGIFYTGKMPYIAEDCKFFPAALPDDGVMDLVITDARTSITRTASILLSLDKGSHVLQP

EVVHSKVKALRLIPHVTKSVISVDGENFPLEPMQVEVLPRICKVIMLSNGNFVDTKFDSM

>model.g362.t1 Augustusgene.g362.t1 JFAV02000100.1:32450-33865(+)

MDTESDIDFKNFYAVEEKLKSHYNQENLQEDNTPTHHNLGLDGTSSESFRHRKENTSSVS

TQIDEALKNFQKSSSNSKYSLKEMEDYLQSHVNDNEGDDPMTTVNLQSSEYDLISKYIEN

ERLENFHDIEFANDSKKKETPEAPLTDVGEQNLYFNNGAAQRSSTYYQAFFVVDTNVLIS

NLKTLEELRTHANTQLAYQIIIPSVVLKEVDSLKKYSKDEVLSQHESQHAGRQRGRPVSI

QKSSKTANDWLYNNLARKDPTIRVQKSKETLNTPNQINNDDAILDCCLYFKEKVEGTPTT

IVLISNDRNLCIKCLSEEIQTISFIDKNIITTESIIEITKQQFAMNSVKTLATSVKSDAL

KLLNSVTEAFFPHNEKFSSLVDFIEYVSNHWDANNGGIQSLFLSDPVLMHLQYWITDLHS

ITTTVHSNMEAFALCQCWSQILTLLYAGFQQDQTVLHSNFARWEQNINNLPN

>model.g361.t1 Augustusgene.g361.t1 JFAV02000100.1:29484-29684(+)

MDSKTPVTLAKVIKVLGRTGSRGGVTQVRVEFLDDTSRTIVRNVKGPVRENDILVLMESE

REARRLR

>model.g359.t1 Augustusgene.g359.t1 JFAV02000100.1:24992-27460(-)

MTTTTSNNTPSVDQLTESFSKIGFEDGKIKEILKNNKLSVILLELIQLSDKTASTESFGK

IERGLVHNLASSINKSKNVGAEDIPKFKIVVDYILDGKLKTNLQVEAAFKYVADDLSKTT

NETFDQYCGVGVVVTEDQIRSRVMDYVQQHLDEIKEKRYVIVPALFANIKALPELKWAEP

RYFKPIIDAEILKVLGPKDERDFVKKEKKKTTNNSNKNGEKKDNKNGNSSGALHKERNMF

AEGFLGDLHKVGENPQAYPELMEEHLKVTQGKVRTRFPPEPNGYLHIGHSKAIMVNFGYA

KYHNGVCYLRFDDTNPEAEAPEYFESIKSMVSWLGFKPWKITYSSDYFDELYALAEVLIK

NGKAYVCHCTAEDIKRSRGIKEDGTPGGERKACVHRSQTVEETLSKFRDMRDGKYQPGEA

TLRMKQNLESPSPQMWDLIAYRVLNAPHPRTGTKWKIYPTYDFTHCLVDSFENITHSLCT

TEFYLSRESYEWLCDQVHVFRPAQREYGRLNITGTVLSKRKIAKLVNGGYVRGWNDPRLF

TLEAIRRRGVPPGAILSFINTLGVTTSTTNIQVVRFESAIRKYLEDTTPRLMFVQDPILI

EIENLDDDFELDCSIPYRPGCPEFGERIVKFTNKVYIERSDFSNNADDKEFFRLTPNQPV

GLIRVPHNLIFKSLETDPKDPSKITKIVCHYDVEKATKKPKTYIQWVPVATGIKVHETRI

YNQLFKSENPLSVENYLDDLNPESEVVLTNSVIEPNFREVVAKSPWVMEAVKKSEFYVEE

ADPTRKEVVRFQAMRCGYFCLDSDSTDDKIVLNRIVSLKDGSK

>model.g358.t1 Augustusgene.g358.t1 JFAV02000100.1:23131-24624(-)

MSGRQGGKLKPLKQKKKQNNDYDDEDAAHKEKLRQEQAAKKAMMQNIKAGKPLGGGGIKK

SAPTKKRKLWSNTGILSYISFMSGLQVEGAGNSVTKYKIVFLGEQSVGKTSLITRFMYGT

FDDHYQATIGIDFLSKTVYLEDRTIRLQLWDTAGQERFRSLIPSYIRDSHVAIVVYDITS

KKSFEYIDKWCEDVKSEREDDVILCVVGNKSDLSDERQVPIEEGERKSKILGASIFIETS

SKDGYNVKTLFKQIAKSLPDFQSSNKNKEDLINENSTGGSKQGVIDLSSANADEDSASCQ

C

>model.g350.t1 Augustusgene.g350.t1 JFAV02000100.1:896-2230(-)

MQISSLLPLVPLFASLAAAAPVFKKHENDIQYYTKRNYMDYDAGLIRGVNIGSLFVLEPY

ISPSLFEPFYDETKPAYEDGYSYGIPVDEWHYCEALGYDECASRLEAHWDSWYTEQDIVD

MASMGINMIRLPIGYWAFQLMEGDPYVTGKQEYYFDRIIQWCGNNSINVWVDLHGAAGSQ

NGFDNSGLRDSYGKFQTEDYMNVTKSVMEYMLTKYSAEPYVDTVIGIELVNEPLGPILDM

DLLKDYYQWGYDYTRNNLQSNQILIMHDAFEPFHYWDDFLTVDDGNYGIVIDHHHYQVFS

NPEVQRNIDEHVEVACSWGMNIKNESHWNVAGEWAAALTDCAKWVNGVGFGARYDGSYVN

GDTTSSYVGTCEGINDISTWSEQQIENQRRYVEAQLDAFDQRGGWIIWCYKTETLLEWDF

QRLVYNGLFPQPIDDRKYPNQCGFD

>model.g360.t1 Augustusgene.g360.t1 JFAV02000100.1:28160-28978(+)

MNSQTTRYKIYKSHKLIRVRINPLNAQQQQQQQQQQQQQVYSIDENKNSETMSCSSSSSS

FSLFSSSSESNELTESETIEIDQENTSHILLQNDLVKSNVHKPLDFTEKQTLFSSIEANN

DNSKPVSNNNLSHSFDSNSQICQQDKIKNQNSEPSTSSKKELETLQHLYFNMQLNKLQSD

VNSLISSFRDKINNFRTDFALQDGSTTTTIIPAFTRSYETIKTDNSNGDNKEKEEKKEKH

NESVKNVSKAHECSQVDRALLHRYLLKQKLKKR

>model.g364.t1 Augustusgene.g364.t1 JFAV02000100.1:35041-37800(-)

MNHDQANNLLNDARTTGLNSPPSSPSKSFQTVDTAASPITAAEVATMLEHPVEGKIGPTT

ETTTTTTETAPKGAAHNVEEETVTVTTVEAPNEVKTITKTETPYEIKTETKTETPTETIV

ETVVETKLNKNDLPIQLISEEKQYNTSTLDFFKKTIENKADIDKVNYHVISVFGSQSSGK

STLLNNLFHTNFDTMDAQVKRQQTTKGIWLGHAKEVATTASEHMKPASDLFVLDVEGSDG

AERGEDQEFERKAALFALAVSEVLIINMWEHQVGLYQGNNMELLKTVFEVNLSLFGKNKI

EGHKVLLLFVIRDHIGVTPIDSLKASLLSELEKMWQALSKPQGTEDTTIFDFFDFEFKGV

GHKYLQYDQFMADVKTLGDSFTSKNPAKSYFKPEYHHNLPLDGWNLYSESCWLTIENSVE

LDLPTQQILVAKFKTKEFSDAAFDKFKTEFENYNHADLTHEEIAKHLSSLKMTCFMAYDE

FASRYNKQVYQDERQVLSENVYHVLVNFVMQKAKELEHELLGSFKELLSTDKKTKNFVTR

SQVAKLKAVERFEAMLSIFFKYELIREADSAEIAKTFNELLIKETETERAKEFNQLVKRF

EKNMKSKLQESLELMLMEPKVTFWKDYNDKFHELFDELLGKRFKSAEKDTVDFKLGFSTE

ENEAKFVAFKKQSWKIWKDVIAGQLTISKLPGILEETFKINFLYDKNESPRFFTNVAHID

EFSKEAKDFALQMLDIFSVARLGDEPGKKIVPDVDMMSDDIDREENDDHEEEEERENEFE

TKKDFGDILTTNEKEKVRIEFNKRMQLVILQTKRSILTSNTKIPPYMFLLLGVLGFNEFM

MILRHPLLLTLCLLTLAGWYFVHRTGSYRAVQHVAISSLQGVKDATVEKLRDTLASEDTS

SAKSKRESFEMHEMSSKKQD

>model.g353.t1 Augustusgene.g353.t1 JFAV02000100.1:7837-9093(-)

MHSLFNFRKSSNKANLPALPIILANVSNSKLLPSSAAPDANNLGTNETTDKNTTASPHPH

VSRRKTAKRKYYEKFKKIFKITSSKKAIPDYQPISENVGATTTTTTTTTTTTTADQEQEI

ATITATTTTDQNQETTAQEIVSSDKNQQSKSILRKSSRYDQQATPVSPEREAQNMQTALE

NLSVEELMTSYEDNLCRLENYEMCIDSRIREQKEPPDWRSLRFPVKQNGPEHTPKTVIEW

QLVHLRVQYLMSHIEKQAFVDETRAPYMKEASAGAWNRLNKQLDKAKQRKVQLAKLLTAE

TEKLERLQPGLITDFDPQHYFNNMEKPAKLFSPFLANVTGTFTLPFSEKQEELRMHPDER

NDPKLLSDLVFADEKYRLMLAELNYFKFLLQKKCSAYSHGHLSGLERFSDLVSWGFVSA

>model.g356.t1 Augustusgene.g356.t1 JFAV02000100.1:18172-19995(-)

MGKFYSPLSQTFGSRPDDFSYSAGSDFRKSHLKLNHKLVKLRQYCHVLNKNTKSYDSSKL

GQFSAETYAKENKLQGALVLHLIERDLVYVESLRLNHRVGKKNQRANTRLKKASKYAAQL

KQLVKLEKVWQTKLEYLVYSQLATIEYYSATSKKSGVSESEISLRYFKVLMGLDLLLQDR

RAKHTDEDYKFYEGIVEKIKDKYRYIPLPSTVHNKKDVAKLLSQSKKNSDKACQIFQILN

LNNYNPNYYFLGVTSEAMKDKKYIKTVEWRSFKADVNDEDLAKHLTNALNLQKKKHIKTK

QNANNNVDASSQNDNELYDEMLMNYSLAEEHHQKIMDPEDNTDQILSTFISYNKLFVMIQ

RDALIFENLLKSWNTAATSGKIRYQDIGEIYAKLEKIANNLSATLTQVTALPGVYSDDDL

LGNLHLSAAFYKSSLYAYCLAPLYKIKGQYVESMALLNHSLKALNECHPFNSSLALPGNI

VNGRKVASLKKNIELACLDVESLAIYQQKLRTKASQNCKYNNGMTLLESGFKLSPSVIEL

NSLFPLKPTIKPVMAKPTLFDLAYNYIEFNSPQATTAPKEIESKTELTEPMKAELPVQKK

GFLGFFRS

>model.g363.t1 Augustusgene.g363.t1 JFAV02000100.1:33947-34840(-)

MTNNTVFWKSYEIKDDRSHDWLMKVFFDAEAKDSRMSFTTFDQQAAQFYSFEISYGTIDK

ALKSLHVLSSKEQISDIISQVYSYVENISLLAPCSSDFCFQLCFLELPQEMEVSISIIDE

LDMIFSVPCNPQKENLIFYKELLDVSGTYLQDSQHIINELIDLVYDKTEVLHECRDMLVE

SGRHSFLDQLEKTNANAFDNFHYNKWVVKVMEKHSHSRKNANRWGRNILELDAYNLKQSP

QKKQSPLKIYSGNTASKRSSDMAEFVNPDNTKKVLMSSLKANDELSSARNSPVKKDFK

>model.g354.t1 Augustusgene.g354.t1 JFAV02000100.1:10645-15630(+)

MAATLLGSPEKSHSSLKQRYLQSLQNTGMSVEPLKPASSTRINSLSGSPRKFSTSVLRKS

PVKNDLYKIKQSYEKEPSFVTQTPVKPSSVLNSRKIFEMSSSNKPLASELSLKAKVQKQT

LPNKSSAKSPSSTTQMPNLNEFSSKSTIDHAKSVTKISSNFEKVDTERFSPIELKYYEFL

CRVYEAKEWIESIIDEELPSVMNLVTRNALQDGVALAKIAQKVNSSLVPQVHPSTTKFKY

KLLENIENFLLLVDTVEVPTNFKFESTDLYDGKNIPQVFETLHALAFTLHKKWPKKVPAM

INEFGLVNFSRDDLRDCKIKFPHLKNFQFFGHDHHSPTKEKSQNHEHDLIPNLDDVDRQI

STKIQDTQNYEQIQQPEITEPKKLQHTSIQPTTSFSAFSPKSMKESALDDDFEEQLKFTQ

TIEKISTDRVSSVVVKNPLPIELLDEKDTNDFGNSSSSWFNYNSKLKDIPVVNYAPRQDL

SYYSPSVSAQMSPRRRRRRRANMYHPENQFDSTASFSGSEYSNPFSSTSSYAASDQYSRS

DYQPYSVHEYDPFYTKSRNGDLTQNSYSSGNTDLTCKVQAICRGSNTRYALYALDLKLEF

LEEEIIAIQACLKSKILNQSIKDKLHIVAVSNQSHILNQLQGYIKGSQIRFEYDRKMFDL

SRHHLSVDALQAMTRAYAVRQRTSKLQSSIFEATASIQKLQSLIKGGSARLKYKETQNIL

ITSSANIVNLQAILKAGTVRKTFHALVNCYTKSQGAQRSVVYLQAYVKGLLYRKKAGRTD

RSLNKSRNKSALLIGCIKGALFRDNFYEILYNMESFAEEASNLQAHMKGVLARYAVQLLD

EVIDNSSVSLLQSQIRGGLERSKLKQTSDYYNQNVGAVIILQSRIRSFALEKAYHELMDF

ANPSLWALKKFAFLLNGVQPTYETQNTLDNLKEAIDKKNLVINKSDSKLRNTAEKLSVLK

SSGVIKHSLSSEVTSILQSLRGSVNFENCETIAYFEKIFYLLQVDPFYFRLLFSVEPQTV

KKYLMLLFFKRDGSINNREGLLYMKLVSELLLVDVESRAHVSEFLLEQFDDEPWKQLLNI

FLLAKHKHLMNSLFSGLIKTLDSLDVSFEGNPSKIYQSLYGTDKDMDPKEAIEDKATNAQ

YITNMCTLWSFIEQAHGIIQSNIEYLPVEVLHLCTKAYTAVAGKSSDEFDALEAISKVII

GAFLNSYLLQSDAYGVQLQHSDHTVANLEILSDALSTVFAMRRFKGFYSQLNSYVEATGN

EIAATLQHLQITPAYEEQMESMVYEDMNKNNRPILTINIKYLSEIVDFIYQNIEMLPQDD

PLVELLENVQDMENTALFTKSSVVNLELNPSAYILCDVSDRGRPLFNNVMWGLIYVIQIC

SAHCSSVLDVLSKPEEENEEVLESRFQTLLLKYPSLKENAVFKELPSVSYFELKMYVTEK

TLELSNMSFIDRSGDFQCLLNDIANTIKCHVYVKTRNMKEIDIAEHTLDMLGKKHQKILH

EYQLLKQSFDTSLGGVKPVVHFNAKKRGLGSKLKEMVHTKNNKDLSSTRYKWSLRQLYEK

GVLLGIEGEKLESLPVNYFGGGGAKFPHVDFVFSTSDGKEYIIEMIDNRKNHGSARISDK

FNFESILERQMNSSAKEIMLVNNKAKFNTKRLFNMIVETFLS

>model.g352.t1 Augustusgene.g352.t1 JFAV02000100.1:5930-6919(-)

MGRADKKQKYTTATGKQVDAEKHLNTVFKFNTNLGQHILKNPLVAQGIVDKAQLKPSDTV

LEIGPGTGNLTMRILEAGVKKVIAIEFDPRMAAELTKRVRGTPYERNLEIILGDFIKIDP

LPYFDVCISNTPYQISSPLTFKLLNCQPPPRTSILMFQREFALRLIAKPGDPLYCRLSCN

IQMYGDVQHIMKVGKNNFRPPPKVESSVVRLVPKLPRPTIKFEEYDGLLRILFNRKNKTL

NSNFKNSSVLELLEKNYKIWKSQQQENNNGMVEDEEMDVDEGKGKNKEIKDLVDKVLHDL

DMGDKRPQKLDQVDFLKILFAFNKVGIHFA

>model.g351.t1 Augustusgene.g351.t1 JFAV02000100.1:5005-5520(-)

MATVDSKHIDTPHENVITTDKSQIQSSPLKNDITANQNTKQENENVEQDNTNTVDKQSKK

WTLSQDSLKIKSFTGYTLSLPQWQSTPSTSSPKVAESSASTTTSDKSSSSDKNKADLVQK

SDESDKKTLFNVDNTNSTENNNSTDEPQIGDKRKPDHGESSAKKIAVPDKKD

>model.g366.t1 Augustusgene.g366.t1 JFAV02000101.1:3382-3648(+)

MIFGFGKLFYVILLLINAIAVLSEERFLRRIGLSGQHQQNTSSFTYGTQPQDASVKSKLI

TLISAVQTLLRIPLIGINVLVIVYELVLG

>model.g394.t1 Augustusgene.g394.t1 JFAV02000101.1:70205-71344(+)

MQHNNKSNSESNGLLKSENNNTTSPEQIIKKRTGVPENGQHHESYNLSVGKLRNLTENTL

LLKTSKGTDTSKKAQSTSGKSSWNKKFTETCLHIFFNILLATFYCVFAVTRYCQYVVHRV

KIKIFNLAYNPANTPQLIRRDVNSLSKIPKNLAAILEYKPEEDVGGGILGLMDGSSDLVA

WSLSSGIKHLVLYDYNGVLKKDVDMLRYTIYNKLAKYFGPNNVPKFAIRIPRTDSIYYNT

AELEAEDEGYGDSADNTGDNSSAKSNTNKKVSIEITLLSNVDGRETVVELTRTMAELCYK

KELDIKDVTMDLVDKELIQLVGNEPDLLLYFGPNLDLQGYPPWHIRLTEFYWEPDNDEVT

YSVFIRGLKKYATCKINVGK

>model.g388.t1 Augustusgene.g388.t1 JFAV02000101.1:51069-56204(-)

MQQSYSSAPLRTVKEVQFGLFSPEEIRSISVLKISTADTFDEKGVPIINGLNSPLLGTVD

RNNMCQTCHEGMSDCPGHFGHIELAKPVFNIAYLSKIKKILECVCVHCGKLLLDEHDPNM

KQAMKIKDPKKRFNAVWTTCKTVNVCETDVLTSDNVYVSRGGCGHIQPQIRKNGIGFTVV

YKKGKDADDDDEPEQKILTPDEVLNIFKHIISEDCWKLGFNEDFARPEWMILTVLPVPPP

CVRPSVAFNESQRGEDDLTYKLGDILKANINVQKLELDGSPQHVIQDFEKLLQYHVATYM

DNDIVGQPQALQKSGRPVKSIRARLKGKEGRIRSNLMGKRVDFSARTVISGDPNLELDQV

GVPMSIAKTLTYPEVVTPYNIDRLTQLVRNGPNQHPGAKYVIRDNGARIDLRYSKRAGDI

QLQYGWKVERHIVDNDFVLFNRQPSLHKMSMMSHRVKVMPYSTFRLNLSVTSPYNADFDG

DEMNLHVPQSEETKAELSQLCAVPLQIVSPQSNKPCMGIVQDTLAGIRKMTLKSTFIDYA

QVLNMLYWIPEWDGVVPPPCILKPKPLWSGKQILSIAIPKGIHLQRFDDGTTLLSPKDNG

ILVIDGQILFGVVDKKTVGSSAGGLIHVVTREKGPYVCAKLFSNIQKVVNFWFLHNGFSI

GIGDTIADEKTMREITEAISVAKAKVEEVTKEAQANLLTAKHGMTIRESFEDNVVRFLNE

ARDKAGRSAEVNLKDLNNVKQMVSAGSKGSFINIAQMSACVGQQSVEGKRIPFGFADRTL

PHFSKDDYSPESKGFVENSYLRGLTPQEFFFHAMGGREGLIDTAVKTAETGYIQRRLVKA

LEDIMVHYDGTTRNSLGNIIQFIYGEDGLDAGHVEKQSIDTIPGSDKAFERRYKIDVLDK

DADLIKNILESGSEVVGDFKVQTLLNEEFRQLIKDRRFLRKVFVDGESNWPLPVNIRRII

QNAQNTFKIDITKPTDLTIEDIINGVRKLEEKFIVLRGKGHILEEAQDNAITLFCCLLRS

RLASKRVIFEYRLNRQTFKWVTDNIEAQFFKSVVHPGEMVGVLAAQSIGEPATQMTLNTF

HFAGVASKKVTSGVPRLKEILNVAKNMKTPSLTVYLEPDYADDQEKAKFIRSVIEHTSLK

SVTVASEIYYDPDPRTTVIEEDEEIIQLHFSLMDEETEAQLEYQSPWLLRLELDRAAMND

KDLTMGQIGEKIKETFKNDLFVIWSEDNAEKLIVRCRVVRDPKTMDADMEAEEDHMLKRI

ENNMLESITLRGVEDISRVVMMKYDRKMINPSTGDYHKVPEWVLETDGVNLFEVMSVPGV

DASRIYTNSFIDIMNVLGIEAGRAALYKEVYNVIASDGSYVNYRHTSLLVDVMTSQGFLM

SITRHGFNRSDTGALMRCSFEETVEILMEAGASAELDDCRGVSENIILGQMAPIGTGTFD

VMIDDSTLAKYMPEQKSAFDVEGGATPYSSDAGLANTDIDIKDELMFSPVTEAGAADAMA

GGFTAYGGGEFGDATSPYSGYGSTSPGFGGSSSPGFSPSSPSYSPSSPSYSPTSPSYSPT

SPSYSPTSPSYSPTSPSYSPTSPSYSPTSPSYSPTSPSYSPTSPSYSPTSPSYSPTSPSY

SPTSPSYSPTSPSYSPTSPSYSPTSPSYSPTSPSYSPTSPSYSPTSPSYSPTSPQYSPTS

PQYSPTSPSYSPDSPSYSPGPHHDHQNNNNQN

>model.g383.t1 Augustusgene.g383.t1 JFAV02000101.1:40718-41797(-)

MTLKVLVIGAGGVGVLTAYSLFHRAKCEVSLVVRSDYEKAVKDGYEIDSCDYGKIHNWRP

HHLYRSIEEASASGQFFDYLVVTTKNIPDGPSPVRKFIEPVLKSCFTNWDPKSGKSLNVF

LVQNGIDIELEIIDLLKADHLCSDGVHGPKVTLLSGVQMIGSTKVGNCQILHKGQDYLIM

GAFDSKDSLAISKVDEFRDLYLNEGKNHFEFDENVKWSRWRKLVYNATINPLTAIVGLDF

TRTLLFGTDGSTEFGILRPAMKEILAIAKSDGHIIPEEVMDYMIDCDRTMVYKPSMMVDV

EKTQLMELEVILGNPLKVAAQNKVYCPNLTLLYNIGKIIQGRTKEKIGMITFNEKTGNLE

>model.g368.t1 Augustusgene.g368.t1 JFAV02000101.1:5299-7494(-)

MQASRAAVRSIHLTCVRNETQLMTKLRKFEKDHVLLKKTHKELRKSQTYKHIKLKKQVKT

PYSRQKVVQLLNEKYKIPLESLKGVEIGPTSNQDVEVFRKISNNKDIFLRDKRLLFTILG

VSGEQLRDAKLISDDVEKFLKRDQPEKALLLIKLSNTKKSVVAMNKLIEFYLIKRHDCNT

ALSLFNWRKKWGIIPNEESFTMLFDGISKLPHKISHKNASNLINIIVGLIQNTNLDFQIN

DIHFNSAMSALINSKDGKAIFELFDMRPKHVKKTAITYTILLKGLTSYYNTLLGESENNS

SDSQHGAKNYVPNVKEFPLEEDSGEEQNNLSTDTEVNSNFQKYHQFFKGIVDQIPLKQMD

DKLCYEMIYSLSRRGILESENKLLKYKILSDCCNGLAHYFDFKGLLKEQRSPEFLPTWDK

LLVRAHSKHEPQFKKFKMNQHVIALAIDLLGRNGSYKKMDEFFQQMISDTTLNTRVTPLV

LEHYFKNVLDGYTEICDSKMLDVLNKCDENGTRRVVSKEVQYLMNTAFLMKLQNAAVLRD

YDRISSTFENFFKYYDQYGSINTTGHHSDFAVNDPSRKKWYIYFEMIKRAKFLEKFGQDK

GKLQKYFLDEYIKDMKKGEFVIADLEQQINLKAVKFKKDIDLKAIMMLNHIIYDLNTEDA

NKQAKTHGTLLMRLKDYLLKRCTLMDVFLKSKNKAKFPSSSDEQTPRAPEYTFEQLDESI

KPTLSSLATRSL

>model.g391.t1 Augustusgene.g391.t1 JFAV02000101.1:59558-59827(-)

MDSKYYQEALEDYKEQQREQAQTKEEVDQWDKRINSTGCYVENMALQLCHADTNDWRQCM

KEMALFRQCWDQHGNNKRVGTVDLVDSEKK

>model.g374.t1 Augustusgene.g374.t1 JFAV02000101.1:23946-25271(+)

MSRDAPIKAEKDYTKILDEELPNIQSLGINDYKEALSKLLVLEKKTRQASDLASSKKVLV

EIVDLLAAHNDWPLLDEQLQVLSKKHGQMKVSIQTMVQRIIVHLEKLKDQEIKIKSIENI

RTVTANKIFVEVERARVTRDLTHIKREEGKIDEACDLLCELQVETYGSMDMSEKIDFILE

QMDLSILKGDYQQATVLSRKILKKTFQNPKYETLKIEYYKLLVKNAMHSNDYLTIAQYYL

EIYNTPSIKKDFAVWSKILSSAVYFLILAPYGNLQNDLVHKIQLDNNLKKLEPQESLIKL

FTTPELMRWPALKSEYSSVLNTIDEEILAFKQSKHWDDLYKRVIEHNLRVISQYYTRIKL

GRLNELLDLQENQTEQFISDLVNQGIIYAKINRPLKIVNFEKPKNSSELLNEWSSNIDEL

LSHIETIGHLVTKEEIIHGLNA

>model.g367.t1 Augustusgene.g367.t1 JFAV02000101.1:4124-5029(+)

MSKFLKSAKATSNLLRNSFISSVYTKPVVSTSSLMYLNKATVNTTSKNANNKVWSRCFSS

LNGVLFNENSKRVHDVLQSEIALEVEDPSVTELPEQLSQYMSNHKYEIVNRKPDTSLVEL

FKSTDVENIHIFFDVSQIANLPSNFQNEAQSDLANGSSAASGSSGANGASSANEEMEFDE

DELNEAFANVNVVVVKKSDNSAISMELLMNVMEGSFYIDSITPFKDSNIALDESADMEVK

RDLLYHGPPFTNLDEELQDSLELYLQSRGIDSELANFVYEYADFQENNEYINWLKNMKSF

FN

>model.g389.t1 Augustusgene.g389.t1 JFAV02000101.1:56723-57853(-)

MTHRKTYFDVSIGDHPAGRIVFELFDDETPKTCENFYQLCKGGFGNCVSKPDIPLSYKNS

IFHRVINDFMLQFGDFTNFNGTGGESIYGEKFEDENFTHKHTGPFLLSMANAGPNTNGSQ

CFITTSNAPLTHLDNKHVVFGKVIQGKRIVRAIEKTECDQGDKPAKDVKITDCGVLPDNY

KVPADAEILQKDQYGDDYEPSIADEPRVDLGSFESAVAAVNAIKAIGTTCFKEKNYKVAT

EKYSKCRDYLADYAKACNDKYATEENTNTIKSLKATIYNNIAISALKDGNYKEVLAAATQ

VLHITDLEDDSAKAKALYRRGLAYFHLNDPEMTLTDLEFATMYQPNDKSIHKAIADAKAL

KKQQLAKQKKSLSKMFA

>model.g371.t1 Augustusgene.g371.t1 JFAV02000101.1:13350-14894(-)

MFPAGNTNKFDKHNSFANSLSSPAQSFHDISSLTKDTIYEKQEIINHKRTNATSNTNSSN

RFHKLYTLGSAEIDLTKGVPTPQVSPVMEAWHSSTPSNDCSNTSKLTSQCNPKRKRSSVY

DLSTILNDAYNKDHSLPPDSSLLDCSEKNPKLDQSVDNVLEEQEAKRCCCKQANKKKIIV

KPQNPFILFRHNFQKLLTKYITLEHEYLQEQKRNAGKKFFVELDSTKNFFTSQELERCCF

KNLFELDIISHECLYANVNFASKTDEKSNISSPLRKKRRNHETSPYTPRVKVSKISQFAS

KKWLGILPKTKLYWKTQANDAKKEYELKHGYFGNGHRVKKVDASMLKNNRLSERKNQVTE

KTTLNDNATAQDRFELCSFCKKQLELNPNVTRSTNWNQVYYEVKNTTIDIKEIMERNNVP

SNLGLSLQNYDSTERFVFMSNKAPTDNQIIRRNAVAEHEILQNTHKQGYTFVNVSNTFLG

TESEFHETFTKKEMEGSILTDYLNGIRMPHESQDL

>model.g384.t1 Augustusgene.g384.t1 JFAV02000101.1:42644-43837(-)

MTTQTYQPLSDIAVTKQDIDRCRYSNWYKKFVKYTPNSIIFENVPLEFIEYLKQDGFHLP

ETADQSFYDMKIVNDASNNDYSDWEDAEEDVDNSNIQLKKDQSDDNADNNKKVALNPLVD

FPDFHEKVKQTIKEYRNIGLTPKLNWSAPRDSTWILPYNNTMVIKEINDLYLLLNASNYI

MHDLECPYEGCGDENLNDSKSDSYDLVLREWFNINPALEFRVFIYHGEIIGVSQRDMKTS

YVFLPALVPELKDRIDEFVYDTFLPQFCDTDKTEDGKASEKDNAHFDLSIVVDVYIPRPY

DKVWLIDCNPFGRCTDALLFSWNELVSVHAENNASKTCEKDYELRLVDNTSVGHRDHTEN

QMPIDIVQASLDPESLKELTLQWQQTLFLQKQEDAEDN

>model.g365.t1 Augustusgene.g365.t1 JFAV02000101.1:1854-2852(+)

MNTNFAGIQSDVITIRTRKVISNPLLSRKQFVVDVLHPNRANVSKDEMREKLAEIYKAEK

DAVSVFGFRTQFGGGKSTGFGLVYNSVADAKKFEPTYRLVRYGMAEAVEKASRQQRKQRK

NRGKKVFGTGKRLAKKVARRNAD

>model.g373.t1 Augustusgene.g373.t1 JFAV02000101.1:21090-23540(-)

MAGSQLKKLKEALKANGLSGPVQQNKKGRGKKGKQSKDYDREERQRVISKIREEFNPFDI

KTNRNKHGSANDQATNTKSSKIAVGKPGISKQVGEEQRQQIYSAKKGSKGKASAFIDKRF

GERNKALTDEEKMLERFTRERQHESAKKRSLFDLEDDGSDSNDFENETDMYGTKLTHYGQ

SLSFEDDFQEDDLAADEPMSGRKRVNQGQDNDLELEPSEEPQRKKTKAEVMKEVIAKSKF

YKQERQKKQEALQDQIQDLDDNFDDLVSELQKIPKKQLEPSSKFVEQAKAVEEGFKDYDL

KVKELGLEKRAAPSERTKTEEELQKEKDDQMKKLEQDRINRMNGIFEYDEDEENQEAVDV

EKLDDGFWGDSEEELEMDDANIPDSDDDVHFSDEDQTTESASSHKARNIPSVPCPQTHAE

LLNFLESYPTEEHVLLIKRIIKTYQPKLAEGNKQKLDTFTGVLLRHIMFLASKDYSKDLQ

KFESVQNSLITILKLLSEKYNRGLTESCREIVEEIQARYEEYGFEGLDPGVLVFFSVVGY

LFSTSDHYHLVVTPCNILMGEFLDQLVYTSTNMKPLVFSTILSNIAVKYQNLSKRYFPEI

SYFLVKSLSILLPLVPSSEYEKQKTGCLPIHSLFQTSYSASTSALLLNNQLNLVSLLVTN

FWRDLSAFPEILLPFQALFERHLENKHVSEIFEKVKKLLKLNIHNPLTLQNHRPLAIPTY

APKYEENYNPEKKSYSTDRTKNEIDKMKAQLKKERKFTMKELRKDARFEARQQIDEKKKS

YEDYHSKMARIVNTINTEEGAEKNKYEREKKLRTGKK

>model.g378.t1 Augustusgene.g378.t1 JFAV02000101.1:30076-31215(+)

MVFEEGFEIEEFTHKYETKVDCNLAEACCYSLSLNDVYKLTGERFEIDNNSRFTYGAIDG

TAKLRELVANLYSDESVKFTKDNVLITNGGIGANFLVYYTLFGKGDHVICVSPTYQQLFS

VPKMFGAEVELLRLQKENGFVPSVDQFKKMIKPNTKAIILNNPNNPLGSVMDTDLLQEIS

KVAEEHGITVICDEVYRPLFHSNDHVPKSVVHLSFNAISTGSMSKAFSFAGVRLGWIVSQ

NIDFLKAARSKRHYNIISISMIDDQISQYVLQNKEKILEKNKQLCLKNLDYLNNWVKKNE

KVEFYDFPKGGSVCLIKIKGISDTQKFATWLAENKSFLVVPGETFECPGTLRLGYANLEE

DVAKGLDILSKSIAEYTNQS

>model.g369.t1 Augustusgene.g369.t1 JFAV02000101.1:7743-9236(-)

MSTRKAIKSLLHKNFQSTLSATRPPRLINLGQPLHENRPLLVKAGDITPGISAQEYYQRR

LKLLEKLPAKSCVVIPGSQVKYASGPVFYPFQQNNDLLYLTGWNEPDSVMILEKVNENAH

DSVLHMLVPPKDKAKEQWEGHRTGVENVKEFFNADESGDIASLASYLNKLFSRNTNIFYD

FEKKPNAFATMFTSSSTNSNENTVTIESIAKHHNKNISGVKSLIADLRLVKSPSEIAIMR

RAGQISGRAYNMAYAERIANERSLHAFLEYKFISGGCNKSAYVPVVATGSNALCIHYTSN

NDVMYNDEIVLVDAAGSLGGYCSDISRSWPVSGKFSQPQRDLYEAVLNVQRKSIELCKTE

NELSLHDIHTWSSRYMIEELKHIGIHNADNYINVLYPHYIGHNLGLDVHDVPECSRFAKL

SENQVITIEPGAYVPNDARFPEWFHNIGIRIEDDIAVGKNDYLNLTVEAAKEVVDIENIV

KNGVTTKIPQDVISPLNI

>model.g382.t1 Augustusgene.g382.t1 JFAV02000101.1:39169-40278(-)

MSTTSPQCLVIGAGGVGVVTAYSLFLNKGSAVSLVARSDYDRASSLGYKISSCDYGEVTN

WKPHHLFRSVEDAALSKTFFDYVVVTTKNIPDGPTPVSSIIKPIIENNDKIWKERDDFKK

QKKTTNVVLVQNGIDIENEIKEKFDLASRKQSTEAPQVALVSGIQLISSTKIGPCEISQK

GHDHLTCAAFDKNDELSVEKAQEFVSLYTNPGQNQAHFDLNCRYTRWKKLLYNSSINSLT

AICGLDFTRCLQFGTAEGSTEFEIVRPAMLEIIELAKKIDNITIELDLVDFFVDITRTMV

YTPSMGVDIQKGQLMEIEVIVGNVVRSAKAHQIAVPHLSMLYNILKILQGKLKEQRGLLT

FDESSAKIVE

>model.g393.t1 Augustusgene.g393.t1 JFAV02000101.1:66220-69633(+)

MRGRSARKNYTRSITNSSHSRTTSDIILRQKPAYIITQLLKRRNRQADGYKLFQQQRQLI

QQRQQYLRSRNKLLPNEMGKASINSQGNDSGNVSADIKQMEHVSTVPIISNDKNAHAKFE

MCHLPENNAHYDYDLEKKLGPNARCKVGTQKVIPDAFNTQSKVLNVEEKFNSKMDFEYSD

NNNYTTRNSENNGNINYIPANNNVLTNYDEVLPYLQLSATVSQGSYTSSNDRQRVSFDNE

GKGYGANGKYLTSAYPGDQESYLDPKIFDKEPQKQSLKMSFLVGIFVAVGGFLYGYDTGL

INSITDMPYVTKHFTTTASHEFTNGQHAIIVSSLSIGTFFGALLAPLLCDRYGRRPTVMV

ATFGVFMVGNTLQICSQGIPLFCCGRVVCGLGVGLLSAVVPLYQAETVHKSVRGAIICCY

QWAITWGLLVSSAVSQGTRLIEGSASLKIPLGLQFVWATLIGIGMYFLPESPRYYVLKDQ

LNDAAASLSYLRGVPIDDTGLLEELVEVKANYDYEMSFHSKSILDCFRSSPTRYKQRSRI

FTGIAIQAFQQFSGINFIFYYGVNFFSRTGIQNSYIISFITYAVNVVFNIPGLFLVDLIG

RRKLLIGGAILMFVGNIIVAIIGTVVGDTVQSAKIRIPFICLFIASFSATWGGGVWVVSA

EMFPLGVRSKCASISAASNWLANVICAAITPYIVSGEISDSTPYAHSTGSKIFFIWAALN

IIGAVIVFFSVYETSGLTLEEIDELYKQSPCAYRSAKVNKEIKMDDSHLIGFLSLAKNYN

NNNHHNNSSANKKNQKNKKTKKKKVRGKFFGKNSPDYDATLNSGTTHVDTSNINSDKTNS

GNTSNDSFGYRRLGPVDFEKDYKQSDTFLTKSNSNSSSSKSNSKPVVLSSERSCAENNPT

TNVETDTFSERKEVQKGTFQNPQQIGSEHFVDLGHGLGLNTHNRGPPSILSEDSDLGNTF

DLQPDLVNSLVLPQTLTLPDDTTKWGPSLVTAEDPAKRVLESRKSFIHKDVDLDDASIAE

IGVQADGSAYNSNIKEYMAQLVDPLNNSQPGEFQAKQVVDTPEQAEFFRSSPLNYNEMFA

RVRQEPPSVFMDDNDISDSGDGVEDEFDFSDDDDDDDDDDDDDGSPLRSQFDFSKIIP

>model.g385.t1 Augustusgene.g385.t1 JFAV02000101.1:44233-45963(+)

MATKAQTSTPSNSVFNNREKPQEVRKANIIAARAVADAIRTSLGPKGMDKMIKSSKGEVV

ISNDGHTILKEMSILHPVAKMLVEVSAAQDAEAGDGTTSVVILTGALLGACEKLLNKGIH

PMIIAESFQNAAARCVEILHEMSHKIDLVKDRDALIRAASTSLSSKIVSQFSSKLAPLSV

DCVLKVAGSNGQSTNNVDLNNIRLIKKVGGTIDDTEMIDGVVLTQTSVKNAGGPTRMEKA

KIGLIQFQISPPKPDTENNIVVNDYRQMDKILKEERAYLLNICKKIKKSKCNVLLIQKSI

LRDAVNDLALHFLAKLKIMVIKDIERDEIEFLSKSLNCKPISDVDFFTEDRLGTADLVDE

IDSDGSRIVKITGIHQSNNQPQQQEQESFVSEVANSVENTLSGKGASSSKTNYYNNFHTN

NTVSVVVRGANNMILDETERSIHDSLCVIRCLVKQPALIAGGGAPEIEASRILMKEARAL

QGVEAFVWQEFAQALEIIPTTLAENAGLNSIKVVTELRSKHERGEKNAGISVRRSGTTSD

AYVEHILQPLLVSTSAITLASECVKSILRIDDITFSR

>model.g372.t1 Augustusgene.g372.t1 JFAV02000101.1:17833-20985(+)

MDSSSIMLPRGDESRSEKPTSQKAQEKTSNRNTFLSGIFNKTNPNDDGSDSARLNTQPTN

SVNFESVDSIALNSLSGSNSSMQSTLDDNLITQHFTTDRMIESDQESSSSSEFGEEQDGR

EEEEEEEEEDEDKDEEGEDEQSDNESMKYFRANIHTLDVDNGDMTEQQQGQESHMNLGKE

PIRKNGLTNKTGFSEDDEPFVSVKKNKTQNNQIMFQKNIAKQDTNDIQKSFLFRKKSTFP

TTENMEDNYSNIDLESNQNGKRDFVQPAYTATMRQNIVSLSKKEKALWKWANIENLDLFL

IKLYNYYLGSGFKCILMEKFLNLFTLIFITFFGTFSTNCIDYSKFHQAQKLQDIVVDQCY

KNSMTNSTKFLIYLCWLYVVLKLITIYNDYKELLEIHDFFCYLLEIDDLELQTISWQNIV

SRIMELKDSNALTANVHEIKAKNRISAHDIANRIMRKENYLIALFNAKILDLDLDVPLPL

KFLPANARTINLSSNLTKTLEWNINLCIIGYAFNEQGYLKHYFLKKSQRTKLVAELKKRF

MVAGFLNIILSPILVSYFVLLYFFRYFHEYKSTPGAISTRQYTLRAEWKLREYNELYHLF

QKRLGLSIEIADDYINQFPNEFKNLILKFIAFITGSFVAILAIFTLFFDSENFLNFEITK

DRSVLFYLSIFGTIYTVCKSSIGDQYKVFDPELYMLELIKYTHYEPKKWENKLHTQYVRD

DFCRLYDLKIVVLLKELSSLLITPFILWFKLPSKSDEIVDFFRNVSIYVDGLGYVCKYAV

FDKESDTFGRDAVTGGTSRKLTKGEKKQSIRTILKKRYESNAGMQDNASKTHEPAKQKSK

YEIGQNDKHDTEEINDKIMQSYIHFVDNYSNVENNVGKTAIPRHVELNGNYTWKKQFGFS

NNLNRSQLRTFENGLRPTESLSPNSSSYNFEHKNKLGHDLSNEGRGTANMNKKSKFEGAQ

QGFGKQSRRPNLLDSDSLINQSSSYQDQHSSIMTDRTFGPNTTSYNNFTNQNHSMTDNSV

YQEHGNLNQETNTKRVLGLLKQYYRDADVGR

>model.g381.t1 Augustusgene.g381.t1 JFAV02000101.1:37458-38810(-)

MIHALSLLIFIPVLTRIVLAQTQNLPFQPQPIIKCSQTQSCPEEWPCCSQYWTCGSGPYC

VTGCNPKMSFSTNSCLPQAAFLPPNVMRRKPFIKSEADVYNLENSGNDNELTDEKEDAKK

SWVYNFSNDMDGLNKRAAEKKFESAQNSQKDYFRVGNTNIMSWTKYLIADDAEAATAQWD

NVDFTYSGTLKISNAEDSEIYLTMPKKTAGSLLTTTQTMLYGKVAVKLKTARSRGVVTSI

VLFSSVHDEIDFEFLGGDLHNAQTNYYHQGELIHTRMKLARVSSDTYENYHMYEIDWNEN

RIIWLIDGKPFRTLTKEETWDPVENIYKYPQTPMKLHIAVWPGGDAQNHPGTVAWAGGLI

DWDNAPDITEKGEFDCKVKEILVQPFETNQLQELQQTLLNFPISKDSRIYWSFDAKNNNP

QNFDAKDVALNAGPVPYLQNSRSNGANRQKL

>model.g390.t1 Augustusgene.g390.t1 JFAV02000101.1:58230-59243(-)

MNRHSHKSRKKSGKKIILKKLNNAVKGLLEQEKQLKETPKFSNTRVVLTTPSANEQIEVK

HKADCKDSIAISKESSPFVTKLRTSELPWSRKDFIDLDSESESGGEVNKKSGLSNEGTEE

PHQEDIQPINKHGVLYTMSKENKLIPKFTDDEIMEKHKQADTNMKEAWMNIIDKYSINEE

EEDYNGIKSDVIDIRTGQIVEDNGHLRSLYKKNKTNGYLDDKNTIRNDKYVSKLLQGYEF

RLEKSNKHLSRNHLNKGKTSLSERSGKQNTESSQHDSDSDSSDSDYIDESDMDDSDIWGD

EKEDYGPQEKSHLEKKITKRDASDEESEYSESDANYTE

>model.g387.t1 Augustusgene.g387.t1 JFAV02000101.1:48611-50722(+)

MTSAKMISHKTASTAHSMNVLVYNGEGASPVCVQHTVNTLKYLLEPHYAVTTVSNKALLT

EPWMTKTSALVFPGGADLPYTKDCKPVISEIQKFVKKKGGLFVGICAGGYFGTSRCEFYL

NHPTMEVSGNRDLKFYPGVARGPTFPGFQYKSQSGARVVTLDVDNGTKVKSYYNGGASFI

SPESFDNVEVLARYCDEKNDMLVETLNENEKSRDAAVVLCTNGNGKALLIGAHPEYVPEL

MVRSKEEGHYDEKLLKELASGNQNRKEFLRYIFSKAGLKVNHVDSETGVTPLTPMYISVD

KIPSEKLKEFKAHLTPNLVDSDHYHVKDNADEFDIYEGYANPKVKPFTSSPLDSTYSEPK

VIVLPTDGESCVPFEKTPKFDVAKFFKHRWPTTEYGSILLYGEVVTSTSGILDSDKTLLS

MLPQNSVVFLGTKQVLGRGRGGNVWVNPDGCLGATISVSFPLKSSATGNPSSIAFVQYLS

SLALCKTVKNYAKGYENIPIKIKWPNDLYIVDPDYYFDKKLNIFDLKSVPLNDIEQPYVK

TAGTMINTHFINGAYNVLIGTGLNATNDAPSVSVQMWADIINEEMKKRKDFDEKNLLPPL

EHEKLLALYVNYLNAFINSFMENGSKSILSEYYEHWLHSSQIITLTDHSVRAKIVGITED

YGLLIAKELRAGSDDAFTGNVYHLQPDGNTFDIFRGLISKKIYH

>model.g377.t1 Augustusgene.g377.t1 JFAV02000101.1:27909-29324(-)

MTGEILTLQVGHCGNQLGKHFWSKILNEHAINKDGSSKIDNEDPEFTREDDPSVFLRQIE

ANKYTPRALLFDLEPSSINDTQNTLPGLFDERNVWISRDSLGAGNVWADGYDYGTQYIED

MINMIDKELDSTDNFEGFQLLHSVAGGTGSGLGSQLLEMLSDRYYKKLTTTYSVFPSTES

DVVVQPYNTILTLRRLIENSDACIVFDNNSLMNLSSSVFRNSNTSFNDANQLIASVMSAT

TNSLRFPSYMYSSLSSIFSTIIPSPNLHFLIPSFTPFTTDFIPEAKNFKRKNSYDVILDL

LDTKNSMFSGAQTPGYYEQQQASYIAIWDSLQGNVDQSDCMRALLKAQQRINYVPWGSKT

INLNIGKKSKYNESGNSDCVTGLMLANSTGIKPILNQSCKNFDALFKRNAFVTKFENGKL

FEDGFDEFKVSREVVEDVIQEYTLCDNSSYLDDILLGDEDDMMVGIHDGMQI

>model.g386.t1 Augustusgene.g386.t1 JFAV02000101.1:46158-47213(-)

MSCLLLKNQLSLQKKFILNAYTRTALKSNYKPLKRHYISISRNHFKTITPQRFFSHTTRC

SVDQHHSNVFEAQKKRTLDSESILRKQNEALPVSNPQKSDTLFTIPNILTMSRIATTPFI

GYFVLQENYILAGSLFIYSCVTDFLDGYIARKFNMKSSAGTILDPAADKLLMLVTTLSFT

LPPGPQIVPLSVASFIIGRDVILLINSFFVRYKTLKAKIDKITQEKFWDFYHYPSVEVQP

TRISKWNTFFQMCYLGPGVILVLLTLQKDNVKNTENEESAGAGAAATATAAATEVDQQKA

SQGESALPSEASKERPDWFTYFGYFVSLTSVLSGLSYIGPAKYSKLTVKILK

>model.g392.t1 Augustusgene.g392.t1 JFAV02000101.1:60613-64752(+)

MVVIADYQRTATFAWSHDKIPVLATGSISGSISADFSSESKLEFWSLLDGKNEKPFHSIE

TSSKFNDLDWSIDNKIIAGALDDGTVEFYSAPTSSTDSSNSKIITSLKKHSTSVKTLKFN

PKLGNVLISGDSKGLINVWDTNKITDSGYSPTQPGQSITSIDEIKCLAWNKSLSHVFANA

GSSSGFASVWDLKTKKEVIHLSYTSPITNSKVPLDIVEWHPSNSTRIATASNTDLDPSIL

IWDLRQPNSPITTLSNVHSKGILSLDWCTKDSDLLISSGSDNTVVLWDPETLKTSLTVYP

ARSNWLFKTKFAPELPDVFASSGFDNKIEVQSLQNLPTQLDEKKSTEIINETEDDFWNTV

SVKNSAQDFNMEKPKVNHIYAPKWLTLQKSPAAQWAFGGKLVYIGSDGKSVHITKPENLI

NSDSNTEMLTTALTSKDFNPIINRRLNNSINPINEEDWSMLEKLSMDGKQDFLKELLAFD

DEESGDEETEDKDQEGKDFFENLETEKFSPSSEEPFKLDITSSDLVKLLANHDFTKSIHL

SLEAVDNTDKDKELLLESFIIALISKQDSLIKKVVEKYFAKYGPKSSLARYLFSLSSSNF

DDLIENSEVSQWKYVAKTINNYVSDNDLKSKLLTKLGDRVLDNGERQDALILYMASNNID

KISGIWLKEFNELEIDLQKKNGKKTTNYESHLQCLTEFVERFSCFSNTFNDGSKFKLENE

ELISKFLEFVSLISSNGDFDLALQFLNALPGDNADVNTEKQRVLIASGKAVSSTKASAGA

SAASKARTVAAKRTSVAPSTFIGSQSSSMFTAPVQPQPALSSYAPQASVNVPQPYMPAAG

APVASSVVNPYAPARSSVSGIPANQSYAPPPTVSAPRKDSSVVPPFGQSPLSLSNATVTA

PAAPVPNTINPYKPVASVIPATQAASTPGTPGGANGLANNSQFGGQPAQAGYYGNGSSSS

PAPSSTMNGSYPMSGQTPSLNKKANEGWNDLDLPVKDKIVRAKPVITKPVGGSSDFDAAG

GIKTSASPLSATGIKVPPPPTSSRISSQAQTAPPPVGKAPLKRVTSEASVKYAPPPQAAS

ASQGYSGLNGVAYAPSSSGVSTPSPTIINPYAPPPPAAAAAPALANPYAPPPQKAATPHG

HGTSRSSANKFVPPTPASSNFGGIAPPQTSGAPSQASMMPPPPPMKRKSHAVPAQNVEQA

LSQLDSIKQNTPGSAAPGVPEPVVEQPQPVSATPAAASNIVASVAGDIVGPSAAQQVSQD

EVPAKSTAISEEELQIIEFFKQELLRVTPLVPQEYSKQLKDSNKRLKLLFQHLENQDLLS

STVVSKLSEITNLMKEGKYTEAKTIQVEIATTYPQEAGNWLTGVKRLINIAEATAGTSSA

>model.g379.t1 Augustusgene.g379.t1 JFAV02000101.1:31940-33082(+)

MPYQEKFEVEEYMDEYETSIEYNLAETCCYSMSLNDVEKVSGEKFELDYDTRFTYGAIDG

SEPLRELIAGLYSNEDVEITKDNVLITNGAIGANFLTYYTLFGKGDHVICVAPTYQQLFS

VPKMFGADVDLLHLKKEDGFVPSLDELTKLIKENTKAIILNSPNNPLGSVIGTDLLQKIN

GIAESHGITVLCDEVYRPLFHSVEDAPKSIVQLSSKGISTGSMSKAFAFAGVRLGWIVSQ

DLEFLKAAKARRHYNTISVSMIDDQLSQYVLRNKEAVLQRNNQLCSTNLAYLKQWVSESK

FAEFYNVPQGGSVCLIKFNGIPDTYQFTCWLAKMKKVLLVPGETFQCPGTVRLGYANSYK

ELVKGLDILTASVDDYFKEFK

>model.g380.t1 Augustusgene.g380.t1 JFAV02000101.1:33226-36874(-)

MLTKFESKSTRAKGLAFHPSRPLVLVTLFSSTIQLWDYRMGTLLHRYEDHEGPVRCVDFH

PTQPLFISAGDDSVIRVWSLDTHKCLYTLTGHLDYIRTVFFHHELPWVISCSDDQTIRIW

NWQNRKEIACLTGHNHFVMCAQFHPTEDLVVSASLDETVRVWDISGLRKKHSAPSHANNF

EDQMQSSQNLLDGGFGDCVVKFILEGHTRGVNWASFHPTLPLIVSGGDDRQVKLWRMSST

KAWEVDTCRGHTNNVDSVIFHPFEELIISVGEDKTIRTWDLNKRTPVKQFKRENDRFWLI

AAHPHINLFGVAHDSGVMVFKLDRERPPFVNHQNQLIFVNKEKQVQIFDYKKKVASLPLV

SLKSIGNPWNAFRSISYNPAQHSILINTGEHDKFALCVLPKEPTGAVQPTNVIHDIGNFA

TFVARNRFVTYDKSTSSLEVKDLNNKKTKTIKIEGSVTNIVAASPGSILIMQPKQVVHFD

VQQDKILAQVEALNVKYVSWSHDGQFVALLGKHSITICNRNMEIVNAMHETIRIKSAAWD

ESNVLIFSTLNHIKYCLLNGESGIIRTLENTVYITKVHGKHVYSLNRSGEVEILTIDPTE

YRFKRALVNKNFPEVLQIIKTSNLVGENIIGYLQKAGFPEIALQFVQDPQTRFDLALQDF

NLEIAYTECLKLKDEGIWTRLGASALSQGNADLTELVYQNKEDFDKLSFLYLSTGEQNKL

SKMGKIAERRGDVNSALLNTFYSNNTIKRANLFAEHGSLPLAYAVSKANGINVASILEEA

GISESDIVLPDGYSTQSFVKSPGKLKIWIFKMTKVKKINDGTAAEEQLTFEDEAEDFHEA

EDDNGQADEENAWDLGDEDLDIGDITDGNEELGELNEKELQGEDSITTSTETELSKWVQN

SKLAAVLASSGAFDAAAQALNKQAGIYNFEPLKEKFIDLYESSKLYMTGTPSELPSIEGF

IRYYEEDNTKVLPFVPDITFINTKVSKGFKSFKANKLEEAIEVFRDVIYTVCLLAVDNEE

DEEKAFEALKLASQYILGLSIELTRRALPPTDIKRNLELASYFTKVKLLPAHRINALQVA

MTQSFKAKNFVQASHFAEELLSIVTSGPRAEQATKVKAKADSIASDAQEIDFDPYGTFDI

CPATYTPLYSDSPTAVDPLTGLKYKASEKGKIDSISLISKIGAPASGLRIHL

>model.g375.t1 Augustusgene.g375.t1 JFAV02000101.1:25408-26061(-)

MSLEAIYQWIDQAEDFVVSRSFSKAAQKYELAQKDILKLLDSLKKEKTKLDENLYLVVQS

LADEIDSRRSKLVGLNQKLNEQHKQSDDDGNKATQEHPSQNSLLNAGVSKSFLETQDKER

NDKLNDSNDLMTLGDPVLDGIINELYAGISKTLPPIYNSALKSEINKHKVRLASHILKVQ

NAQSLREQLLIEENRDLKQYSEKLAARWETMKENTKQR

>model.g370.t1 Augustusgene.g370.t1 JFAV02000101.1:11661-12986(-)

MSFENDKQMNYKNSTSSIILLVQMLLQEYAKQRKLFSSSFFARKLFKSNKAVEEEHITVH

TLKRDKTPETDLFIDWIAKDIPRLIEQKSLAGLSFDLYRDGPNTIENRPFETVMLRINYA

DNNTFSKNNEPDEENTLHMTRSYLETKKHMMTQVKSLVSRITLLKPLPSCYSISLKVLTN

ENLHKKPMKNSFMLSSSKLENSLFNHSAFFLDENKLATKFHQFKLCLFSSLEKNNFYPMP

LLDGYTEKLSSSGTNDANKKAELRTRADMCDILTEKSLHKFLDGDVTAVNAETQLVCFDE

IKRLVTQDKNMLNSQIFDGDITVNNCSDEKNKKVTAYISEDSYLKCGKKNACKSKTPDSI

SKKRKRTLIDLGLNLIETETGCFSKEIDLYTRSTMFQKSCEDKTKNGFHSKNLPKAIGKR

KILITKKQLQSTNKKLKSTFAS

>model.g376.t1 Augustusgene.g376.t1 JFAV02000101.1:26424-27800(+)

MSFATNLWLELHALVSNSNTQNTINLILVAYLSKCSEYENALFLAEEPSRFYKAALILVE

AKIFQQNVDFCLSKMLSLMNVDADVETPYLKYFVSYALLCEIKMRDTSLDILCNYQGFRV

LYNNLYSNFAYLSEYGDDSSKLSQKDSSLDNVEYEIMDDFRKISTLQMDILFQIIKFSKC

SEQDIRLIDNFFVFFLMNTMVSSTTEDLFNNTKFKLLLSINEQYMIINKKCHDSFAQKIE

NKVFKFLLNTSVSQSFIELLFLFFNRTNNDRSLQIMICKLLYLVLTSNSEIVMNFFYLND

LKVLTDVLIREIDNLSISEEYVRNTFLRLLYRLLKYTELSTIEYRKQDLQTVLTQLTKID

NETKNADQLECQNCTINLANKCLSDISWLALKSKTDTDDNASDVSSFSDSSSVFLAKQNS

NTESFAHMYTDPNVNVSIDSLRLKKRPPPPIPRKSKRCM

>model.g402.t1 Augustusgene.g402.t1 JFAV02000102.1:19348-21009(-)

MSELYINEFTGKDATTQTGTKELPFQTAAYALFSSASAGSAEPKLFVFKKTEQEDKSIVE

EYVLITASALKKAKKGAEGLKKKAIKQQQQEANVQEITKKIDVLAFEDLKLDESLPKAVP

AKIYQSYELVDKRIKVSGWVHRLRVSKKLAFLVLRDGSGYLQAILSGDCANAAQALPLES

SIELYGTITKLPEGKSAPGGVELVADYYKIIGLAPSGDDAFTNKVQENADPSILLDQRHL

ALRGETLSAVMKVRAAFLKAVRRFYEEEHLTEVTPPSMVQTQVEGGSTLFKLDYYGEEAY

LTQSSQLYLETFLASLGDVYCVAESFRAEKSHTRRHLSEYTHIEAELAFLDFHTFLQHIE

NLLVRSVKYVLEDPIAGPLIQQLNPGFKAPEAPFMRLEYKDAIKWLQEHGIKNEDGEDFK

FGDDIAEAAERKMVDTIGVPIFLIRFPVEIKSFYMEKCTDDPRVTESVDVLMPTVGEITG

GSMRIKDTDALLEGFKREGIDPAPYYWFIDQRKYGTCPHGGYGLGTERILAWLCNRFTVR

ECVSYPRFSGRATP

>model.g399.t1 Augustusgene.g399.t1 JFAV02000102.1:10374-11501(-)

MFKKAGLCMQWLDRANSNALLFTLQQVRSATKRASGSKTSMKDSAGRRLGPKKGNGAYVQ

PGQIIYRQRGTKFYPGENCSIGRDHTIFAKEPGWVQYYLDPFHPKRNLIGISLFREKKLP

TPHFAPRIRRLGRRVIGDEKAAEKEASSLTRKQFFAKDEFVAQHEGRENKRLELLKGFEG

FLTNNFPSNEIFNKNSEFCGRALLLIRSNLRQGFSRDESFFNASAHFKHSAKINSHIKNN

DMPDIKVIESCLAEIKKYVDFNHDYDIVEYVIFEDKLAKQQEIRAQVAELLPLNTKDKVK

KAQEIFNKPELKTCFTKSEYVKFKKSIIKPVLPESVEGTVVSKTTKGATATRRFDYKTGR

INTIYRSKHAFLEPLV

>model.g417.t1 Augustusgene.g417.t1 JFAV02000102.1:58373-59134(+)

MKLAYLSVLPFLSSLAHGAIIGEDADAKTASSGGSTKDSNAMIDETINVDVESPPEGMEW

QDWHMLQEHQLDSYSPETFFALHDISKKGYLDSKDILTMYGLNRDEVIGSGDGMGKHDES

EVVDPELADRVVKFIFKLFDIDDNDKITKQEFLNIAERGTKFPDLGVGVGHHGDFELEYE

FHHWNEHHKDADPDVKNVHREDIEHDLLHHEHEIEHEENVPRGGSRATVITDDELEARIK

AKNIPAAFKANVYQ

>model.g409.t1 Augustusgene.g409.t1 JFAV02000102.1:31020-31394(-)

MNRQLTAFAKIAPISKTVARSPIRQFHTSLSHMQSYQKWADLDKTGKQNFIRSFVNLHVE

KHPCSKSNVSYQALAHDMEEYDDTPYIFGILYDEIRAVAQKESVHNTDGSGFTGDPDFAK

LLSSK

>model.g411.t1 Augustusgene.g411.t1 JFAV02000102.1:38608-40530(+)

MTAESSQQSSYREATYKDSELQRISFENTSKELTDTSNSLDEKDFQNNYEEESVTSKEVI

RTPDPAAPQYYEGWRLAIVQLSLNLILFIAALDIVIVTSAIEKISEEFHDYAKSGWIITG

YSLPTSVCCLIWSRIAQRLGKRLSVAISVVLFEAGSLIVALSNSMNMLIGGRVLAGIAGS

GIQVLMFLVGATLVPEQKRAFVLSLLSFSFTVSSVVGPFIGGAFTNAHSGGVLSWRWCFY

INLPIGFTALAVFYFAYDDHDTSIMSILKYWRANLKRDVKKLKTKNLYTKLMLELLVSFD

MIGFITSSAGYILALLSFTFSNSDDYSWTSGLVIAFIVVGMLLIIFSFLFEYFLYHKMVS

HLEQRYSENEALAFNFKRAAPLFPKEAVRNMYIGCANATATFVTMAYGFQANYVIQYFQL

VFNQSAMKASINFIPFMISISIVVFLSALVMSKTGAMKPFIILGGVSAVLGNALLSTMDG

SSNEAKKIIYLIIAASAFGFVAQSTLLSSHLQLDKTDPMYMLKFAGVTGVNSFAKTMGIS

IGSVLSTMTFNFSVINKAKSLSPPVTLTANTIVSYITGNYTSPTSPLSNMVSSAIHNVFY

VSTAISAVALILSLFTSNKKVMTAKKAKEIEEQEEKQDLDV

>model.g415.t1 Augustusgene.g415.t1 JFAV02000102.1:50914-54051(+)

MSDSAESVKVLNELFGKLTVATPEQRDDVAVEISSFLNGNIVEHDVPEQFFQELTKALQG

KKTAANALAAVQHIASANDLSASVEPFVVALTPDVCAATGSKDKATAELAASTLIALTKA

INPMSVKVILPHLTNALSTTDKWQTKVAVLAAISALVDTAKTQVALRMPELIPVLSESMW

DTKKDVKIAATNTMTKSTETVENKDIEKFIPQLISCIANPKEVPETVHLLGATTFVAEVT

PATLSIMVPLLSRGLAERETSIKRKAAVIVDNMCKLVEDPQVVAPFMGKLLPGLKNNFAT

IADPEAREVTLRALKTLRRVGNVDENDTLPEVSHAGDIATTKSVFEELLKAINVTVAPRF

APVVTYVAAIAGELIDERVIDQQAWFTHILPYSTIFLHEKNAKEIVDDFRKRAVDNIPTG

PSFDDEEDEGEDLCNCEFSLAYGAKILLNKTQLRLKRARRYGLCGPNGAGKSTLMRAIAN

GQVDGFPTKEECMTVYVEHDIDGTHAETTVVDFVFQGNVGTKEQIVAKLTEFGFSDEMIN

KPITALSGGWKMKLALARAVLKNADILLLDEPTNHLDTVNVAWLVNYLNTCGITSIIVSH

DSGFLDKVCEYIIHYEGLKLRKYKGNLSEFVKQCPTAQAYYELGASDLEFKFPEPGYLEG

VKTKQKAIVKVSNMSFQYPGTTKPQVEDISFQCSLSSRIAVIGPNGAGKSTLINVLTGEL

LPTTGEVYTHENCRIAYIKQHAFAHIENHLDKTPSEYIQWRFQTGEDRETMDRANRQINE

DDAQGMQKIFKIEGTPRRINEILARRKFKNSYEYECSFFLGENIGMKSERWVPMSSVDNA

WIPRGELVETHSKLVAEVDMKEALASGQFRALTRKEIESHCSMLGLDSELVSHSRIRGLS

GGQKVKLVLAAGTWQRPHLIVLDEPTNYLDRDSLGALSKALKVFEGGVIIITHSAEFTKD

LTEEVWTVKDGKMVPSGHNWVTGQGAGPRISKKEEEGDKFDAMGNKIASAKKKTKLSSAE

LRKKKKERMKKKKELGDAYVSEDDDF

>model.g395.t1 Augustusgene.g395.t1 JFAV02000102.1:1-965(-)

MSDIEKKAATTVDVASKSTDDNGSSSNEHYVSGEKPIEFITSIKSYSDEQVWHLLKVLNF

DDVKDLNDLPPEVEFLGTKVHEITIDESLEIMKDAIEYHDNDPNIDEDQYRSFIRYAEEG

VDPNNDVAVFELKALAVLLRDHSPYPEVRAVCPPAMMDDPTIPIETFRAYFFALIWLIFS

AGFNELFSHRLVSISISTSVIQMFLYPMGRFWEKWIPSWGFSVKGHRFALNIDTPWTDKE

QMFCTLIIAISMGTFYTSYNILTQKIYYGQDVSFGYQFWLSLSVQFMGFGFAGILRRFVV

YPVKAVWPTSLSTIALNKALL

>model.g400.t1 Augustusgene.g400.t1 JFAV02000102.1:12009-17135(+)

MSEGQAKKATASKKPAAFKTKDTLDDGSVKKRAHGKSCLICRKRKIKCDQTKPVCLACVK

HACSMDCSYTKETKKSVSNSSAGASAMFSANETATKELEQKLYELQAKRASPVSLETGIS

SNVMSNTNNIDQTLLDGRPTVLLTRGDSTRGPQSESLMQLLNKQSNEIKLQHRLVDANHE

HNTMSPLSNATYSKLSSKIRKTVRKQKMIKEFSEFKKKLESLEALLEGSIKEEEFEIEHY

SPSSDASLSPYATNSLTASDYPELKYIPKMSFYDGFEQVNVLSARLLFLGPLSPSTIVKK

DSFCIVLSAMINKTRDDIAKALEVQTEEKYQGLRVTARHMAEQVNIHIKSDSKIVNEEET

DNDLLENVRINGAARGQQRMQDLSTPAEAQQQVSASERYSTFGIPENNDNNVEQNEDRLR

KANSFPVMVADGTKHVQEQINFNTPSLEASNKSKDGRAHSISYNSGPHPGSVDVSKKRTY

SNNVQNKNNPKFNKKSKSSISHLITSGAGDDEDEEAEEVFEQKFLENEGLDEMDTILQEG

KIANNPLSVDNLLNKKIGSSSNSRSGSTTHLSPSNPMLISSNTSDIRSEKLPPRNMQSHM

PSASWRGSLQNLRESPRAQAVSLDESQVLTGQHSFENDAGNSMNAGSNSTNQAFDQPHTD

DQQKLLDLKTQAIPQTESKELFSETFQPNLPQASRNAEDDINMWRALDNVLTKIQKQNSK

PQDRIPEGLFGQSNDIKGKQELDVLMRIRCILPSAKLVRMHIDNYFKSPIHGLYPILNED

WFRDTMQTVISGLDKNSDQQPKINATRRFDLSKLGSLLVLLRLSYLQCPETLEQCQTDTE

RFLLSHPIGPEFIDTAQMCLNLFKMLRKGILPVLHCALLLRVYRKYAPEDGDIVDGTDSE

TFTGLLVRSSTSIGLNTDVARSHWVQGQPMYIDQWRKCWYVVYFFDFMEALNMGNTLSID

VDTFDTKLPEFKLDPHTGVNPTYILDPQLEDVVVGNMQRDFQMTLICRELLCAITNKRQK

FSVLELQSSIDRLDQFIRKEYGADMKSIIEMPANTLPQTVAKVNCFKNFVDINSLIFMVY

LHIFIHIDKNIVSNLNAIKADHSVESNMELFHFYLKKVISLYIEMEPALILCYESKKKEK

NKVEEIFGKSTKLLVIQSCEYVLTRFRQGLYVVISRTLHFHYNFLCNVEETSMEQINPER

KQIAAMVSEIISCAMDKLEYSDSICRLLSQKYFQIWRISKHSQFMFQLLSNMENNLFDKA

SKINNDHRNECNNTRFAIYPFKYIPHINLFSHYRLIHFQEIFSTLSSVKWNVFSNFLTPE

ELAEAKDYRRHDRKAFIKNTRRSKKCGSNGKSAAVGLEKSVKKKNLKNAIKKNEVHNANG

FKETGFENEKNISPNMHSSQSSSTPVVGQIDIPIDEIDKFWYSTVINNSNVFAQMPNRLA

STGVPELNATNGDSSHFDDANIKHLQRNLQVTANVHPNTLQEGQPHSTHFPTDIFDKQEL

SKNASLGQSNTSNSNATPFDPSFGNNEALDEIFNNKDMLKAHVFQKLQELNAQTEPPSLE

SSMAYSGNRTFENSNNFRHTASYAETELMQNSHLSELATNPASNLNSTMDNKVIGTNNGF

SNSANASHTADFTRSYNANEPSSGYVQPANTNHANFVANDADPSMGSGHKDGGIQVFDNI

ADILKMQEMFNTDFLFPGSNSYYSDEINF

>model.g404.t1 Augustusgene.g404.t1 JFAV02000102.1:23660-24838(-)

MVTSNGVNEKWDTVISLLQDLFNANEVIPFDYHAAKNANAENPSDAQSETTLSKQKELQH

KDNCSRGKRILQEIQINLDGLLKDLDKNFAANLGNQNGELQTNGTEGADSKHTLVHDEHG

MAESGRGGRKRRKLLGTPELQELPASLGKRGRPSVHEYKNDKSDVSAQAAVSIAEGAKSF

KDGSLVPEDTAALPGRRRKHASEDATDQNANTKTSNMLREDETKVPAHHGTTSSKNVQHG

KSFYASEYNANEPVLLGSQVAYKPMKKNSGITDWFQCEVTNILNSEGTKFEIKDPEPDDF

GNPGASYKCNWKEILLIPPHPNIASARKLKAYPVGTKVLAKYPETTTFYSALVIGKTQTK

NGLPTCILRFDGEEEVDKETEVERFWVLPFPKK

>model.g418.t1 Augustusgene.g418.t1 JFAV02000102.1:59237-60808(-)

MYTTKIQDTEKKQEFLSVTTDKPVKESDNKKDIVVLSGGTATNSLIDSCFSPALYNKVTF

ILPISDNGGSSSEIIRLFPGAAPGDFRSRIVKLIDPKCKDDIELARIMNYRLNQDNIDLA

KREWNDIIEGDHPLWDECSSILKHMIRSFLINVHIEILKKQQLNPSAKQKFDFRNANIGN

FFLTGCRLLIGASFDSGLELMMRICKVDSNKTKVYSCINTNYTHHIAAILSNGQIIIGQS

QISHPSSVSVLSNSDEGLNAVSSSQPHQQDTLQQKAHEEDDVITDDEFDVDIKSPDEDDN

QKRIHPDLKISQLNFTKSMTHSKLPSPIEKIVYINSYGEIIQPLGNPRCVHSIKHANTLV

YSIGSLMTSLLPILILPNVAEAIVCNKKMKKVLLVNSEVDRETEQFVDTLTNPQNITDTS

TACSTNTNAFVHMIVKSIYNNLPPKYKPLNLSDNSTTTLQQENEIPISFYKEVLTDVIFL

KKGDIPIDGKDLEKKGLNVHCIDSDSQKFEGEDLQEIFKTIGAY

>model.g421.t1 Augustusgene.g421.t1 JFAV02000102.1:64561-66126(-)

MLKNQMSLMTKRAVCTQVVLAQYRGLASAAAKTAAKTAATIVQQQEEHAHRLRDDRYKDF

RLSVSTQLQNIGSKDASAIATEAIDTGVTSLLEEAESKEASGIPGFSSVLKHAYQEQSDR

GTRPIYLDVQATTPTDPRVVDVMMKFYTGLYGNPHSNTHSYGWETNNEIEKAREHVASLI

NANPKEIIFTSGATESNNMVIKGVARFYKKTKNHIITTRTEHKCVLEAARAMKNEGFDVT

FLNVDDKGMIDLQDLENAIRPETCLVSVMAVNNEIGVIQKLKEIGTLCKSKKVFFHTDCA

QAYGKIPLDVNDMNIDLLSISSHKIYGPMGIGAVYVKRRPRVRLEPIISGGGQERGLRSG

TLSPPLVAGFGEAARLMKEEFEADTQHIDRLSKRLTKGLLSIEHTSLNGHADQRYPGCVN

VSFAYVEGESLLMALRDIALSSGSACTSASLEPSYVLHALGKDDALAHSSIRFGIGRFTT

EEEVDYVIKAIKERVEFLRELSPLWEMVKEGIDLNSIEWSGH

>model.g396.t1 Augustusgene.g396.t1 JFAV02000102.1:4351-6621(+)

MNNTTYYRDAPVVHYVNFNQTGSCVSVGTNHGIKIFNCDPFGKFYSSASSTHPATDPDSS

LPTQADGGYGIVEMLFNTSLLAVVGLGEEVSLSPRKLRLLNTKRQTCICEITFPTSILSV

RMNKSRLVVLLQAQIYIYDITNMKLLHTIETSSNPNGLIDVSNNIDNFDYLVYSSSQKLI

TSEVKDHSIPARRSSFNNNSTTSLSSSINSSSSASSSMGTIKEQQQQSTQIITSNGTSSN

LHKDIFSTSPDTRIVSVSNQEYNGSSGSTTNTSGYTGSETAGSSNSTASVTGGNNNNNDN

NNSNNNAPISKNGDVIIFNLKTLQPIMVIEAHKGEIAALSLNHDGTLLATASDRGTIIRV

FSTETGNKEYQFRRGTYKTRIHSLTFNSTGQFLACTSSSRTVHVFKLSSNSEPSGRSTNA

LHNSPSQQSLTAKMMHKNKSLSISGSTSSSIRSNHTGHANFDESDAESANLIDEDEDEDD

EEENQHLNIDDEYVGKSDNEDALGSKMGDADGEEEDAQQKEVVPVVDSSRSTVARMIRKS

SQKLQRKAAKTLGQYFPQIRVASILEPSRHFASLKVPNFDNVQHLHLTSAANNDVLIFDN

MESSANNGASSSPTNNSLTPTLTNNNNNNNNNNNNNNNNNAHINSINLRLKAAFGEKLLD

IDIRDYPEMLKHKRTTSSHADEHHRIASGASSSNNANDVSPINSNNSHASLKSEASNFIQ

VLPIYVISTEGFLYKYLVDPDRGGDGILVSQYFLLYD

>model.g397.t1 Augustusgene.g397.t1 JFAV02000102.1:7337-8617(+)

MEGILVLDKTPKFLIASFYYNYLVPILGYTKWPLHAAYALLGKCVRYFGMLPWLAKTLLR

GIFMKSFQVISWVLFKPFGILLEFVKVFTKLVFDFIFFPINVVLSVFLGTSLSKMLAGAQ

RLDKRDFIIIGELFKIFFIHWSALLFLGAVLGSLNSLVMFKLLNNIFNVSFLKFEFTLKV

SDITNYLAGPVRTALRFIKKMASHAKSEISNDDLRDSKFTQNKPLPAPSQAFIESSETVS

TLPLDGQAEKIAVHPEDHPQTVLSSRGSLSSVSPGKKLSQPSSSKGSRKPSIHPTEPVLS

LNPNTGGIAIRRLSSTREELATQLPEDYFQQLQTPANSDTEKEGPGVEKASVRKVRTYSY

TYSESQLSRSELGEDEEAEFDDACESVFSYSGEETAKTKVGTHRGSAKTSGVFVPRIHAT

NAVHKPK

>model.g401.t1 Augustusgene.g401.t1 JFAV02000102.1:17264-18652(-)

MSEQKNKLWGGRFTGETDPLMNLYNASIPYDQKMYAQDLEGTRVYTQGLHKLGLLTDHEL

SEIDRGLNEVHKEWESGNFKIIPVTDEDIHTANERRLGEIIGKDISGKVHTGRSRNDQVA

TDMRLYCRDVLSSKLLPFLKSFIEVILNRAREEIDVLMPGYTHLQRAQPIRWSHWLSSYA

TYFTEDYKRLVQILERLNKSPLGAGALAGHPYGIDRDFLATGLKFSEPIGNSLTAVSDRD

FVVEIMFWSSLFMNHISRFAEDLIIYSTAEFGFIQLNDAYSTGSSLMPQKKNPDSLELLR

GKSGRVFGSMCGFMMSLKSIPSTYNKDMQEDKEPLFDSMTSVEHSILIATGVISTLNINK

EKMFQSLTMDMLATDLADYLVRKGVPFRETHHISGECVALAEKTSLSGIDKLTLEQYQNI

DSRFSEDVFKIFNFEESVERRDAIGGTAKSAVLKQLQNLESQL

>model.g410.t1 Augustusgene.g410.t1 JFAV02000102.1:32539-34503(+)

MGWLDIHKSNSTRVHHEEAHRGSPEASHDVDKTYSTELNHLASADTACFPDENEEKKIQL

KEETEELLNYSASLNLILFIAALDIVIVTSAIEKISEQFHDYSKSAWIITGYSLPSAVCC

LIWSRIAQKLGKKLSVALAVVVFEVGSLIVAVANSMNMLIGGRVIAGIAGSGIQTLMYLV

GATVVPESKRPIVFSILSFSFTVSSVLGPFIGGAFTNAPSGSILSWRWCFYINLPIGFFA

LFIFCIIYKDPECDAIKIYKYWAANLIPNLKKCKTRKFYTLLLLETLVTFDMIGFLSSSG

GYILTLLAFTFASGENWTWRSSIVLAFFILGLILIIFSFIFEYTYYDKMVAMLRKKYSQN

RAIDFHFDQAAPLFPQVAISNTYIGCANAAALFVILAYGCQANYVIQYFQLVFNYSALKA

SINFIPFMISISIVCMFCGVVMARTGHLKPFIIIGGVAAIVGNALLSIMDGASNEAEKIV

FLVIASSAFGFVSQSSLISCQMQLDKEDPKYMTNFVGVTAVSNFCQTLGISIGSVISTMI

FNLSFIDKAKKLDPPVTLTADDLVSYITSHFESPKAKTSNMVSSSIHNVFYFSTAIAAVS

FILSLFTSSKKSPTDKKLKKGFQDAEEDA

>model.g403.t1 Augustusgene.g403.t1 JFAV02000102.1:21576-23147(-)

MRESDYQRSRSRSPVRRDPEDRRSFREHSRSFDSRRDSRGSTNDHEHSTGSTYRHSERRN

SGSRPGDRRSSSLRDNGRRSSDHSGVSSSNNRHGSSNGRPRNHNHNNNNNRGSTSSTSSY

VNKKPQVGYSPPRVEGASYEDKMNRNYSKSVFVGNLPYDCAATDLKDYFSKVGRVIRADI

VTNRGRHRGMGTVEFESRSAVDAAIRQLDHSNFMEREIFVREDNPPPPSSQSTFSASSSR

SENDRGRDSNRTRNGNHNNSYINNEHTNNNSIRSSSKDMGSKQLYDGFEVFIANLPFSIN

WQALKDMFKKFGDVKRADISLDHNQRSRGFGTVYFKTAEEAEDSIAYYAGYEMEGRKLDC

KKGRYGWGATSRAANSSEHSQNEHDDVKLIDTDANISSGRQIRNGNSRQMNSDFVAGARS

DGPTKSKTVYVSNLPLSTAQSDLYDLFDTFGKVSRAELKFVDGNMNGEAVVKYDNLDSAE

MCVDRLDNYVYGGCELHLSFATYDDNNSSIVETTEGEDAAVHQQ

>model.g416.t1 Augustusgene.g416.t1 JFAV02000102.1:54498-56561(+)

MKSLLFYMLWWCCFVDTAIGKTGTRETDNTAKIICKYAPQKVACPERSLIRNGETGLCRA

ETEYMNKRYELHIRKNLMTFLQKCDIPGLSVSKYMHEVKRAPPAIGVSLSGGGYRSFLIS

AGVLQQMERLELLPCINYMTSVSGGTWLLAKLILQEFEDLQLTTWDFQSSLLEGVPNFEL

SDEEVGDSDLIAYKPLLKDNNSEDSSSLVTTIEKRGFLDQYKILKIKLNLFINNKANDQV

TNITPLIDFLDNLATLQKTFHFYESIHTELKEKRLLGFPVSFTDYWGRALSRRMQKPIND

ASNEHLALSVGKFHDVTVKNSKKFKNSGMPFPIIVANCKNKPQMTNVVFEFSPFEFGSFH

YSLNKFVEIKYLGSKIENGKSVECYSGFDDLGFIAATSSSLFNNILAVVWEMVRTCSKET

IEAMSTLLNSFGLSVEPPPLGNQNASEETKIKPGYAVFHPNPFYISEDEQTKNKDSTLTS

ENHLYLVDGGEDGENVPLRPLLKAERRMDIVFAVDSSSDAKTWPSGKNMHSLFLHMQAEN

DTRTVNWKNKLIQILNIPRIPKAITDRSKPMVFGCAVEKYIYSVKNKGIQSEHKVNVLPP

MIIYIPNFPHVFSSNTSTFKTQYNRTEIAKMMQNGGAMLLPETADSLEAAYFQKCLACFI

IKRSFDNGIAFGNPPKFCDLCYKAYCYN

>model.g408.t1 Augustusgene.g408.t1 JFAV02000102.1:30327-30890(+)

MIELQYMLLLSRQGKTRMIQWYDSFTSAEKQIIIKDVTALVLSRKSKMCNIIECNLHGST

GNTHRVIYKRYASLYFIAGITTAGSARDKKLAGQNNTGANTSEDYVDGDSNELLVLEAIH

RYVEALDSYFGNVRELDIIFNFEKAYHIMHEMFSCNGSILESSKNDILSNVAKMDQMESV

DNLSTVLS

>model.g420.t1 Augustusgene.g420.t1 JFAV02000102.1:63271-64368(+)

MSEVKKIVVLPGDHVGQEIVNEAVKVLKAIESSTKSKDVKFEFQNHLIGGAAIDATGVPL

PDEALEASKKADAVLLGAVGGPKWGTGAVRPEQGLLKIRKELGLYANLRPCNFASDSLLD

LSPLKPEIARGTDFIVCRELVGGIYFGERKEDEGDGVAWDSEKYSVPEVQRITRMAAFLA

LQHNPPLPVWSLDKANVLASSRLWRKTVEETMKTEFPQLTLQHQLIDSAAMIMVKSPTTL

NGVIICNNMFGDIISDESSVIPGSLGLLPSASLASLPDTNKAFGLYEPCHGSAPDLPKNK

VNPIATILSAAMMLKLSLDMVEEGAAVEKAVKQVLDAGIRTGDLGGSNSTQEVGDAVAKA

VSELLN

>model.g419.t1 Augustusgene.g419.t1 JFAV02000102.1:61925-62650(+)

MVKSVVFSDFDGTITCEDSNDYIADKYGMGKEKRLEIFKSIIAGTGSFAVGFREMLESIN

KPFDECVQILHDNITLDPGFTKTFQYCEQNDIPLIIISSGMKPIIRSLLGKLIGEEKANK

IPIMSNDVKIDPKDNSKWEIVYKDTTPHGHDKSISINECKAEFDKKYPNEKITYFYCGDG

VSDLSAAKECDLLFAKKGKDLITFCERDHVPYHEFETWEDILQAMKDVLEKGVGVGALMQ

NK

>model.g398.t1 Augustusgene.g398.t1 JFAV02000102.1:9213-10118(+)

MIRAQLSCQKKAILSHLRFKSSSTSALAYKQLHKNKKTPPLPTLETPSWNVNSAVSSILY

ETPAAPKIPSKKKQHVLNCLVQNEPGVLSRISGTLAARGFNIDSLVVCNTEVRDLSRMTI

VLQGVDGVIEQARRQIEDLVPVYAVLDYTQSDIVKRELLMARVSLLGAEYFEDLLHHHHH

GGASATEAQQDVRSKKFHPKNLPFSEVLRLKHEHLNDITNLATNFGGRVVDISDANCIVE

LSAKPQRVTSFLRLLKPFGVLEVARSGMMALPRTPVEKDSTEEEDVEGQINDIVDVSQLP

PG

>model.g406.t1 Augustusgene.g406.t1 JFAV02000102.1:26801-27832(-)

MGKADSTTPLLSTNTGSNKVQTHQPQQTETGLDRPGPEESTRSNHNSYAAISTPEIADEQ

FREMEESFALSKKRSRVYTYLRTVLIFFLTICFLLPIGCMIFSVPLMRAVDNKYGDNYLQ

AFVENSTNIDVQDIDWRILNSQHIQLNITADVSFDYLSSVNDASFTATSSSLGLLPSDKT

LRKLMRFSMNHAVRNMCIMVDNIEVVGEVHKDNEKSYHSDSVKWSLSNLKPGSQDELLCF

SVMENVTTRVYVPILLELNWTNLYKIIKVINSEKDREALQRNIEIFSVLDLNVYKETFLG

HKLPILKNLHIDKFDVTKYILDTGYYTEFAEPYLRPSSSGIGEE

>model.g407.t1 Augustusgene.g407.t1 JFAV02000102.1:29142-30038(+)

MLYLIGLGLAYPEDITVRGLKAVQKCERVYLEHYTSILMNSSMEELEEFYGKKITLADRE

MVESGCEEIMANADKVDVCFLVVGDVFGATTHTDLVLRCKQQNIPVEVIHNASVMNAAGA

CGLQLYNFGQTISMVFFTDSWRPDSWYDKVMENRKIGLHTLVLLDIKVKEQSIENMARGR

LIYEPPRYMSIAQCCEQLLEIEETRNEKAYTPNTPCVAISRLGSNKQAFKAGTIEQLSQY

DSGEPLHSLVILGRQTHELELDYILEFVDDKDNFKKMVIEDQEFFKPAPWVPEEENMSD

>model.g413.t1 Augustusgene.g413.t1 JFAV02000102.1:46424-46996(+)

MFKLKNIFKREKDRSNAGSTTSLKSASTKKNVHTSTSPPVSKEHTPASSVRSGDERHSAE

KTQQQQQQQQQQQQKQQPAPAHSSTPETAASSQHSENVPTPNTNNESSKVSSQKDTEKIT

FVLSDEQLHKKELQQQQQQCQQNNLNSGDNSDRISGISGISSSIHSLNSDYVDMDFDEEE

DDEEEERRRRR

>model.g412.t1 Augustusgene.g412.t1 JFAV02000102.1:40703-45289(-)

MNFNSDGQRATVYHIDDYALLNDTRNGDDQLLSANDDANQVSMMYGNFLNKNKAYSHKSI

EKLIFTNQDTKSNSDHKNEQVLLSENILNVDEYDAYENAGLQYNQQKNSIIIRLPQTTND

KSNWKTVLEINLNEYYSLDHCGNTSLAFLVSIMIAFSNKNADKGYSTPRNGRSKKKKRKI

SSADELVEFVYKTPLDYKSVNVFSTFYLQKPTTLCNGNSKRKWCLKLDYLLYYKPNDFNT

YTKDTINFIYPQPDNEVKVESNVNVRQLFEEKIIKHTTDQLGSTGNGNKSHYFLTTEQKN

ALQPLNLLPFQEKTVSWMLGKEQKNRSSLIGISVLDFLNQHLAYGYQNFVGVGYYNIYTE

YLISQKQATNYYNEYVPKTRNEPLSKKRLGAKGLLCEEMGLGKTIELLSLVILNPRRKPG

NNASLAQRLGPYIKGKTTLIVTPQNILNQWIDEITLHSPQDKDLKIFHYRGFNFLKKDFP

DITPTLIAENLAQYDIVITSYHTISSELHYTEFSKRHHGQDIENSEDNGGKNLRHFLKYD

YSSPLSLIQFWRIVLDEVQSVGRSINNNIIKCCSNLQRVHTWGCSGTPIVSNNYNKSVAD

FLTILAYLQIAPFIIPQVFKKINQILTLGKQGSYSDLQSQMTVDKLIDDTLIKFSIPIRH

SKKSVIHEINIPKQQKLLLPLQFQHIEQDKYKNLFQKFLQETGYTETGVPRFSSSSSFSS

FSASSSSSSGEQRPVWKPDNDLMNKWLKELQMACINFQIPESKTFSKNLINTTQTKSSME

DILAEMQKDIYQEIDNSLKRQLTTLLYKGRYELETLSQVDVGMETLNKCIEMIQQEQETL

LTKPKQESSSSVEQSTTRVKDTNHTLLNLLHQCYFFLATGYYLKGSQRLEKDEALVKDEA

EMSDIYKCQELEEHYYNEAELIRRQLLKNKIQQVDSERSKILEMNAKNESRFTALSLVGF

SKNDNYSSDPAMDKIFKELSYVFENLDKQAAAINTYIKESEELLSKPILLDSDTAADEVK

VKEYETSIENQDLVFSIFDCIEAILENRAKVIDDKTLESIKKYKPATTKQYSKQHLTMLK

GLPMVQGHSLKMIFNEIENIRIVKNYKLINDFEYFLLQYSRNLPAMFKEIQRVRDHLKFL

NVIYNAKTDYYSSLQKISDALIPLYQLDLTLFNKVQNDYRNFDSVWKSANTQISNYKSRL

KYLQTLQNLKENEKLTCLICLDYITLGSMIKCGHFFCKNCILEWFKQKTKTNLKCPACST

KTSSLQELYDFKFTSTEDNSNLANAHENNTSSPNVTNLNQNTQSKLSASNTSQNSLFGDY

EECKDFHKIMTLNIEVDLGSKINYVFKIIKYIELQHGGSAQILIYTSNLDHMAYLKDLLN

RSGVPTLSTMGLLQTEFNHTISEFKKTKNYKVLILNSKRQVAGLTLINASHIIFLDPIIN

NSDELQAVNRINRIGQSTETYVWTIMMKDSVEESILRYKSSLEESFKLNSGDAHSSNNGR

KRKNHHGEVETKIDQDHIWNCFFFRESTQ

>model.g414.t1 Augustusgene.g414.t1 JFAV02000102.1:47199-49022(+)

MAQVQTLNYAPRKLFKEQEGLMDYLLLEKIGEGAFSKVYKAVKFDMNKIYSNTGDYQEQS

SDSDSDSESESENELESESKSKRSSGKSPRQEFFAIKVIIKKMMDGEGTQQHQTKDNEPK

NSSKEQVLKEIMIHKLASQKRKFYSAGGINSRKSSSKMTQQTHVNGHRGMDFGTDENNGP

AHIVEFVDFIETEQYYYMIQELLTGGEIFGEIVKYTYFSEDLSRFVIRQLAIAIKHLHSL

GIVHRDIKPENLLFDRIPYQPRDNATDPPLKLRKSDDPNNKRDEGIFVPGVGGGTIGTVK

LADFGLSKQIINILNNDTTKTPCGTVGYTAPEVVKDERYSFQVDMWGIGCVLYTVLCGFP

PFYDDKIDVLTEKISKGQFTFLQPWWDEISLGAKNCVSKLLEVDPKKRYTIEDLFQDPWF

MTYDCLENEKILRAQQMKTSDSNSSSQSAKARTSNRHKHSKKLHSKKKHLMSFDVGDSAL

YSPAAVAMRDAFDISNAVARQEEEFMEHGGSKSHAQSKNGHRTRNNNVNNNKGNSSSLAC

LNESEELDTSDYNTTITAPNTPEPGKIHENFFQLRLNSSTIVKRRNKNGPANATSKGPAM

SSPLRVEV

>model.g405.t1 Augustusgene.g405.t1 JFAV02000102.1:25642-26709(-)

MIYEERAESFAQQFFASTINTSLINHFLRKFSWQPVFPDCNENLNIIFPSTKTFFQVALV

EEEEEDDEEKEVEHAKKDSAKALRIFGSIYDEGFPFEFTNGVCYADDINTVTPMNLFLNK

VFNSSEAIRLGVVETSFLSLYDPLHQSDIHDKSMQDPPSVKNILNPALTPLPIDVNFTLN

SSDFLREVGIDKKTFKIIKDPNNKRNPNSYKVQGQGYAEIFIPYVTDLEISVEKLKGETK

IIDQISNKHFLTVTMDVWEDCKVEYINDDEDATSGATLHKDHHAQPVHMKLIFKLDDAEV

EVVDSAVLGRVMSRTFWQGESPVLIDGVMDLIVKNDILGEFMLSDIPGTGSTVITR

>model.g473.t1 Augustusgene.g473.t1 JFAV02000103.1:129336-130901(+)

MNTPPGQLSPRIHPGAVLMSPNGNYSTGTSGASTANLSHKKNTSVNMQGHYFPHSTLDDL

IEKTDHPLSPTTSNPSVQHMNGTFLESFASPSQAPLDSDLISYKSQYQNTNDPLGGNIID

ANFGNSKSSTVVNFTQDVNQLIYWMEKLSVQQQNTVMDNLLSSLNEEVLQYTKLKLDSLI

HTGYISPSPIGSPAMLPHNFVSGNNNNNNNNNNNNSNVINGFDLNSSAGASNVLGSFMPL

ETTMSAPAMGPAVSMGMSPSLSMHAQPQMNSLDYIMGVNEQPVGYVNSEYSKQQQQQWGS

TNPMAPSLSNGDQPNSSIYDFLMGNRPRSADPFKDKSQQLLNEKNSGNSVDAAGPAFHKP

SSKYPNDTSSSKNSGGNSYIGKKLGIKNFLNGMKPGSSSSSSTAPSQGSAHANESNSSHV

AAVSSSASSATSSNQNQAMQPSNLTDVELLQNIPMWLKSLRLHKYTDNLKQYNWEELTAM

SDADLQKNGIAALGARNKLLKAFKIVLDCKEQGLIAKEAYEH

>model.g463.t1 Augustusgene.g463.t1 JFAV02000103.1:103491-105692(-)

MLRASVNPALMRASLRAQSTTAAKYTPTLNQVIINQIQTGKQKPINEIVNNLNERLSKNG

TTGQASKTKFYSNDFFTNKNISELISRSKYDYKMYLKNEYSFSDVPPTPYELFTKLDNLN

LIQPYHYTLLIKYYFDEKNFKELLSFYVNNSNQKEVFDKLRSNNKRESNTLRDMIIISYL

QTCSSKENVDFNILLKFLNNNFLQIDHATFRSIVEMFYYKGSAKNDPLDAQSMSKFHEFY

KQFMDTQSKARFAKSIPPNRTGLSYLIDLKNHYLPYVYEKNKTPKLSWIMDSMMDHYSYK

LFTELYKQYPNDVYSNRSLLYALGKNTQDPKEVRSQRIQAVWNTFFLQRADPPLCKEDYL

AMLRSLGELDYENTLEHFFKNSVDIPTSLKKDIQLKNCYHFYLLNYDGKSNFHSLKQMEE

IEKTIETKSQQTLEFLHSHLKELTDFPNDEHLQKLVLILGMRLVEHKTFDKEMEMNSKGS

DGKFLQILQTLGLENVEKSLIVYNKLLNTENLQDKSLMIEEAHFDFAQISSYTKNKKFYE

NLLRYLPNNYINPTNYLMNIIETCVIQLCRRNEEDLAVELFKLNFQKFIKLANVKNTKNV

AKDIKTLINAMIQGLSKNLKYCSPNDPAHISKCLKSLDEFMFLYLKSFEVLFENQQSNAQ

TQNPRKNGVFLDDRSMKQILMAVVELKFAKLTHKDKQLLKTFIDHYNQYKNIYDKNLTSV

EKAKIENLISKLAV

>model.g425.t1 Augustusgene.g425.t1 JFAV02000103.1:9965-11218(+)

MGINNPIPSSLKSETKKAAKILNSFVKPNQVLGAEEVIPPNVLRNAKGLAILTVFKAGFL

FSGRAGSGVVVSRLRDGTWSSPSAICLAGGGAGGMVGAELTDFVFILNTYDAVKSFSQFG

SITLGGNMSVAAGPLGRSAEASASASMKGVAAVFAYSKTKGLFAGVSLEGSVLLERRETN

RKVYGSNCTAKMILSGRVEPPAFADPLYRILDSKVFSYNGRRRGGDEYSDEDDSLYDDLP

SSLGSEDSRSFRSSNDAGRTRGSRRARSSSFRGGRNSGDSYDSYDNDSDFGRDDDGFGDG

RLSHQDTSRDYYRRARATSQSTKTRWEDDIYDTPAGGSRSSAQPSRPRTEKPDFGLSNNG

LPKAVALYTFKGEQGGDLSFKKGDVVSIIKKSESTNDWWTGRMNGQEGIFPANYVELV

>model.g475.t1 Augustusgene.g475.t1 JFAV02000103.1:135417-135929(+)

MLRLQSPFLKNTASLLQRSVRFNSTTIGNGLTKTELPYTFTASGPIQVKYTPQHEWLAIH

PNNIAFIGITKYAAVALGDATYIELPEKNSSLEIGESFGSVESVKSASEIYSPVKGEVLE

INEELNDKPQLINHDPMGQGWLVKVVFEELEREDHLLDLERYEQSLKNDTY

>model.g469.t1 Augustusgene.g469.t1 JFAV02000103.1:121993-123312(-)

MSSASKLLHLDAFSKTQEDVRIRTRSGGFITLLCIISVFFLLISDWKQFNTVSENPSLVI

DRDRNKKLRVSMDITFPHMPCNLLALDVMDDSGNIQLDLTQSDFVRQEIDSETLQPIDHD

EEGNAGFVSAEEALRNLPADYCGPCYGSKDQTKNDELPQEEKVCCQDCDTVRRAYLDVGW

AFHDGKDIEQCEREGYVERVKREINQGCRVQGSAFLNRIHGNIHFAPGKGFQHPTRGTHT

HDLSLYESYENLNFDHIIHDFSFGPVLMGYNAQTKEEEFVTSNPLIGVEMKEGNHFLQYS

YFAKIVPTRYEDHTKTIGSKHTISEDKFKSDVENDDKDVGKSLNSLETFQYSATMHSKPV

FGGRDEDHPNTFHSRGGLPGIFISFEMSPLKVINRKEYRMTWTSFLLNAVSSIGGVFAVA

TVLDKMTYRTMQYVRSKKDA

>model.g447.t1 Augustusgene.g447.t1 JFAV02000103.1:69126-70172(-)

MPNLLATQSHVVHGYVGNKAATFPLQCLNWNVDCLNVVQFSNHTGYGFFVGSTISEQDLT

NILEQLIKNFRYNALLTGYLPNKDCVRTVLTKYNEYKNQLNAANSGKKCLWLLDPVMGDE

YQLYVHANVVPEYKKFLQMNNIDIVTPNSFELQLLYNENLTFIEEHKSLPKLITMEEIYE

MLAGLHRNVNFIVITSLDNVDVGEEADRANFIHCLASWHQESDKKYLFKIPKIKSYFTGV

GDLFSSILLNSLYNSAVFSTSITAEEREAEIIYCINYTLTVLQKVLEITIKQRLLASPGI

TSTSIESASIGGDPMVMKNMELKIIESRNVFLSTDKTNILNFLCKKITH

>model.g438.t1 Augustusgene.g438.t1 JFAV02000103.1:46623-46946(-)

MAFKEGSAKKGATLFKTRCLQCHTVEEGGANKVGPNLHGVFGRHSGQAAGYSYTDANIKK

NVLWDADNMSDYLTNPKKYIPGTKMAFAGLKKEKDRNDLITYMLKACK

>model.g482.t1 Augustusgene.g482.t1 JFAV02000103.1:146264-149794(-)

MDYNNNNGTNHLNNNKYNIMHNHIGYNYSGAQNGSSHQPPSPATSRNFHQQSMYVLPNGQ

NQQHWNTQQNPNFNRSNVHSLLNESNLLQNNMPNTQLAQLQPQSILPRGNLPNGHGNQVF

QTFPQQPPPPPIGLSSYSSGQPNMINQYSSGISSSSSSLNNTSSNNSAEKFKETQPSATS

STPVQSQSDSSADSLTGRNNLSVANLLSANSEVKIAQDNGTPSSNGSDLKQDEASKTEKT

AESDKVSKQTTKKRNKLSFNCVNCRKRKSKCDRLKPSCTKCLQLGTSCFYDTDTQVGPKR

PNKDAMIARLSKQVEYWKNKALKEGNGGPAFHHDHNNENNRLYNHSVALSLDRQGNLSQN

YDTLTPPPAGADGVTYLSSSPKGQTGDNTIGNTSSYQPSESDLSTLTINPEHIYIDFKTQ

APSFMRSNGVTKHFDIFSDIAAVQKDVFMRVFMSSIMGFSSINNQKQTSEERKQTIAKIL

ESTVYSEFELSDVERASCEHYAQKIVSGKNDEQQGTVNIQYALLSGLTDVKTVFIEDSIP

SGDEQSPLLKKSLEMLKNVLPPKTKIMQYKYFFYSKIYSSLVALNIETFEKSLAATLSDD

PENPEFYKIDVGTSGIREKMENLVLLCIIMKLSDVALQQILLKNSIMGKVNSPGVQFACS

EDHVKRFSNIIDVSIKILTFLNATAWTTENTLCCLMYLWSYLVVSPGEADMFSGKPTDLL

ANLIVTLACNIGVDVDPLDIVYFQKNKKSPLINYRRILNVSLSTILVIEDLFKGKMYLNT

AVHEQVLSVKHQQFLDKMLETRPEMSHLDRKILSLSFKRAQLYALICEFYHNSRKDKIVS

LFSVETNMLKINGFLEKNFLLSNLKKPSSNSEEVLTGTNTKISSTLVENTYCIELVFFAK

AHTLMVNNSIFISLQNSSKEAKSSKNYFPFYKKYFIVLIHDVCSSFKLLEDFFEGKYNYA

LNNRESLSMLKSVVILLNRVIFIILALLVRFSFTKQILLKKLSNRLENVFNSKEIQLRID

LITKMSDQTSQLLKRLCTYSTKKLRFSYFQSFKTSMFFDYIKKLTEKNELCNILLRILDK

DTQKEKLTPKAVDLLQNTMGFDINHSEDFKEDLELSELLSFVPISLLEEYLNTFVVTEVL

ETEEDEAMEQSSENTSSIPFDVDEFNIFDYDFLFKGT

>model.g489.t1 Augustusgene.g489.t1 JFAV02000103.1:161751-164369(-)

MNLDRFKNDYKPLEYVAGYISGYELLNPSSLETERKQQLHNLEKYYMSKTAEKTTSSLTS

LTSLTSLNSLNAVPTISTPLKQNNNKDPPKKRNNQDLHQSKLVLQSDIDFFHKEILPTRL

TSNTDIIAHLKTGSRQRLYYKALNNLCKETVTKELNSYLNYSNNLRVTTDIFSHQIKKQN

IWIPNLQIESKYIAQDAISSNHKLNSFSDPNEIFKPLSKDIFNGVAYYPKLLGETKIPGL

VYHNSVEINDTVYIMGGLRSNFENTQCIDDISNFYVDGLDDLPAPLDKDVINNPNMVGNK

KFYSFNTISNSFREHVPRGDVPSALLCCKGSKISERYIFYYGGFEIITELINYKKDPKTN

EKLFFLKSRALINNTGYILDIATFTYTKVELVARPSKYFSYPTTVPRFGHSQTVVLKSKS

LDKFSLSCTNTIDDSINTNKKQFPDNIMIIIFGGYKQVGDSKYEPMNDLWSVELPVLEKG

VNNYMRFTDTAFATMHKNSLMNITFSTPSTTGNTPVMSLTSIPTINSSAENSKNTTTPNN

IQWPCARGFQSATIVQRSNFEKKSIKDTILQNLIDNYKVKTARRKSSNHYHNFNLRNGSS

YGALQSDGSTPSISSIKTASAQNNSSHHSTNELSAVRKKHVQNMSVIDDSDMEVLLPVSS

SSVSFDDDYEQLNVDNSYLVIHGGTYNEDTLGDFWCFDFSSETWEQKFLEAPNLIEPSLY

KPINLNLCGHEMFSSGEYAILMGGLSSSCKNYTRENHSLVTLVHLPTLRVLNVKQIDDFE

VEDQDIRILGSPIKGSNGIYYMLGGVFMHIVKRDKMDPAVLPSLGEGKLVGQEIKTPLMN

AVVSDDTVTQNYLHGAILASLSPTVSVKSQTFF

>model.g440.t1 Augustusgene.g440.t1 JFAV02000103.1:49774-51348(-)

MEYKHSKKRKTALVAILSSLFTLLFLYEFVTNIYKIPGLSSVLLSTSGGSTTSSPSWFPS

FNDKFQVYEPDFSKTPQVEYYDLQKYKGNKDGWQTQDRIVFCVPLRDAAEHLPRFFKILS

QMTYPHNLIDLTFLVSDSKDDTMGALKNELEFLQANNDKSKRFGNIEIYEKDFGQIIGQS

FSDRHGFAAQGPRRKLMGRARNWLGSVALKPYHSWVYWRDVDVEVVPDTIMEDLMHHDKD

VIVPNVWRPLPDWLGNIQPYDLNSWKESGGGIQLAETLDEDAVIVEGYPEYATWRPHLAY

MRDPSGDPEVEMELDGIGGVSILTKAKVFRTGSHFPAFSFEKHAETEAFGKLSRRMGYNV

VGLPNYVVWHIYEPSSDDLKHMKWMADEEERILEENKIKDFYDKIWEIGFEDMRSSWEKD

IHNILKNSDSTFQRKINVDWSENEDEDYIKEEEPKREGKGEGEGEGESEGEGENSEEDIA

VLEEEAKKKAESLDLDKSSKSGKKEKSEKKEEEDDSVWDMLSNLE

>model.g437.t1 Augustusgene.g437.t1 JFAV02000103.1:43239-45518(+)

MSFPENANAHSSSDVPDGSMADAFKTDLKITSAPASPVLSPKTGLIHGSPFSSSSSPTTS

SIPPLTSNTENNNTLSDDIEGATAENTMKNRKMSHNDLEVQHLKEELLDCAREVDEVKFG

SNKKLSRSHSRRSSVAQILSLDSSNNFDSDVPATSFVTAPTTTTTTNTDNTHKNAMFTLS

PTPTNSGATSGASTGTTTPNVLDTNTSSYGGTGVPGNTFSGNSLASIMSNGGLSSATNSN

NNSQLEMKTKFPRVPHFHHSSHLNKQFTVDPQLYEHYHHHHQQQQSRTGEEISYSSADLR

KSSSVNGESRRTKSHAQLSETAHGVRLLSKDIFNTKMTLEVENLMIVTKHYDDSLIYLTR

DLVEHIFVTYPNINIYLEKSMFYGNELFQLEDICKDICKDYSNSADCEKKNESLDSKPKQ

NGILKNKTKRASIAEGKTPQTFEERVKYWDLPFIQENSELFDLVITLGGDGTVLYVSNLF

QKSVPPVMSFALGSLGFLTNFKFEKRLNSLRKILKHKVRTKMRMRLSCTLYKKGGRKILE

RQVLNELTIDRGPSPFISNLELYGDDSLLTVAQADGLIIATPTGSTAYSLSAGGSLVYPS

VNALVVTPICAHTLSFRPIILPDSMVLKVKVPQDSRSTAWAAFDGKNREELLKGDYIVIK

ASQYSFPTVEARPTEFIDSISRSLNWNQREPQKSFSHMLSDKNKSRYENDVMQNKVKLED

ANGARSLLAKRSRDLEDSMTDYSSGSSSEDEADENDYACV

>model.g453.t1 Augustusgene.g453.t1 JFAV02000103.1:80608-83838(-)

MQQSLNMMGSIEPSLNVPIQNQMNLGGQIPLNYSSSSFPQSSSSFPQSSSSFPQSKSQSP

EYTPLNDDKNESNRRTHVKKACDACRAKKIKCDGLQPCGSCKKKAESAAMPNEIQCTFSF

ECKKKEPSKPRTSNKQILGMLTDKLASLEKMLNVIQENTIKRIKTSSPTSLSSAASSSSS

ATLIGHQQQQQQQQQKYPITIKQEEFANMQIPHIINKTATVPTVPKTIQKTDFKVQKKTN

IKHLFSKASNHKQLSVATTNKVKPPFGAEKISGFLHDNEDARKYHIRTKCCFLILTQEGF

NWVKKKISLPDCDALLTKIAKLNELLRKDNELYIQKVFQRVESPTLVKPPEAILDVLCKH

LPKTVSYAYSMLAVKSTQALIETYKTDLNTMEEEKLFLVNAVLLLTCSVAKCNKDLVSLS

SKKELEFWEKVFLLNTVSFYQKFTLSTYNPETLKSGLLCLQSLWYLCIYLQNSPSPKVAV

MIASAAITMAQELNIMAPSVVNSLENLEERNECRMLWFHWFAVDTNFSIWYGKPNYISIE

QLTVCDRDCHTDFLAAFNMTMDPALFAIDKCQQRYDFLSQSALGHYFIMFDLKFRLMGIT

SLAYKDILSVQNERRPLNDLKKDVDATNAKLLEFIASLKRAFKLSEDCSVEDVFERLIAI

QAHNNTDFISLQFFGLNILLDSYTLLLIVNSKLLSDLTILDQQETSDITKSTILNLFKLC

DLALNKCWSPASGCQVLFSLACAFCMGTMIAISDKKFLKEHVKTFVQSLKTITEANEKKG

FIDPLIWSVITIYNIHLLRLLFLHGRDILDKSMVLQWEKVFAEDTKKLYNKVITISKSMV

EIVEESNVVRRQAGRPIFKNHFSPTSLDTLLKPSPLDWMSQMSPLTLNALNNLDSNALQN

GMLTNSPGLLGAGLQGLTPFTNNGLNNINMSREPSIMGFSKSNNWGDSMKSPNQISMMNS

RSSALDMPPLIPQSPIKRPSVTSQMSDNLLRKHTLNNNALFFEQANMQKPGLLKADNKSS

LQMPSNLLNTSAPSKTIEIPEALRDLYFDDIFGLNSFNALEFLDDEMFTHANSQGKE

>model.g422.t1 Augustusgene.g422.t1 JFAV02000103.1:1348-2205(+)

MNTNDMMISLLSGACAGTSTDLAFFPIDTIKTRLQAKGGFFHNGGYKGIYRGLGSCVIAS

APSASLFFVTYDSMKKFLKAKYGNDSVRGNYTLIHMVSSSMGEISACLVRVPAEVIKQKT

QTTVGSTSWKTFTNILHSSAGWNELRLNLYRGWSTTIMREIPFTCIQFPLYEYLKFYWKS

KEQMKQNLNGNSKHVTLSPIKGAMCGSIAGAIAASLTTPLDVLKTRIMLSTDKQRDSLWT

ITKNLYKEEGGIRVFFKGVGPRTMWISAGGAIFLGVYETVHALLDH

>model.g430.t1 Augustusgene.g430.t1 JFAV02000103.1:24841-25899(+)

MFRDRTNLYVSYRRTYPKAFRTTTATRSGLSNAQEFDEEEGFPMLDSVDVSNEYTQENNS

LKLIIDNILKPIDMKLTSLDTLIDDDLVKLYKKVMLPGFQDRTEDINKIEKLNYQIIKYL

QICSSSIQLIKESQQQQQHGQGNSPIRTAGIDIILENLGKAYARKIQARSTKFRKLQNNY

LKFLNKDDFKPLPSSSVTQQESNPQDLLLLEEGEEVVEDDAEYSKSVLVQQKQQRSQQMK

SEYLKKRDEEITELAQGVLEVSTIFKELQELIVDQGSVIDRIDYNIEMTSTNLQQAQKEL

VTATKYQKREGKCKIILFLVLLCILMFFIFMLKPSGGGTKTVVIEKPATPNTL

>model.g445.t1 Augustusgene.g445.t1 JFAV02000103.1:65886-67823(+)

MLSATRNNSAIKSLVAKRLQSTKVQGAVIGIDLGTTNSAVAIMEGKIPKIIENAEGSRTT

PSVVAFTKEGERLVGIPAKRQAVVNPENTLFATKRLIGRRFEDPEVQRDIKQVPYKIVKH

SNGDAWVEARGEKYSPAQVGGFVLNKMKETAEAYLGKSVKNAVVTVPAYFNDSQRQATKD

AGQIVGLNVLRVVNEPTAAALAYGLEKAESKVIAVFDLGGGTFDISILDIDNGVFEVKST

NGDTHLGGEDFDITLLREIVKKFKEESGIDLGNDRMAVQRIREAAEKAKIELSSTMQTEI

NLPFITADASGPKHINMKFSRAQYETLVDPLIKKTVDPCKKALKDANMATSDIGEVILVG

GMSRMPKVVETVKQIFGREASKSVNPDEAVAMGAAIQGAVLAGEVTDVLLLDVTPLSLGI

ETLGGVFTRLIPRNTTIPTKKSQIFSTAAAGQTSVEIRVFQGERELVRDNKLIGNFTLSG

IPPAPKGVPQIEVTFDIDADGIINVSARDKASNKDASITVAGSSGLSESEIEQMVNDAEK

FKEQDQARKESIESANKADQLCNDTEASLKEFETKVDKLEAQKVKDLVAALRETIAKVQS

GAEDVKPEDLKAKTEELSASSMKLFEQMYKNQQQDGSAPKDEPKKD

>model.g468.t1 Augustusgene.g468.t1 JFAV02000103.1:118410-121337(-)

MSRQISTQSNISLPDVSAQIMNVKVTEEHSLFHVCAALLKRLDQLPALKPYISLSKSTAE

TACEKQAFLITQNNNNTHNLHNNLNTLNNNNNNNNYQSQSKASNSIASPGNGGGTTRNHF

ENSRSNSHRASSSSSLDSASPTTDSPRMSSSGARFSTTSVNSASSSFTSKNDLLTFSIGV

LPVSVDCDPVTQLSKLFQLGSPLCIVFNSVKPQYKLTTVSSDDIKICKKSIYDFICACKV

HLAFNDEELFTISDVFSNSINNLMKIIDVVNLLLDSAPKIFPPVPIQRTASHGVSSVPSF

SQPNSPMYDFSQQSSSPHATFQRLRSTDSTKSELPSPSYSREKASHNNFMSSSLSNVSSP

TGPPSTLSKKDDRYGVFREFLETERKYVYDLEILQDFNEQLVDLNIINLDESNMLFPKLH

DIIEFQRRFLVSIESNNNVNYDEQRVGSIFVHSQNFFKLYEPWSIGQTAAIDFISSLPVF

QSQSSNLIIKNKLELQAFLLKPVQRLCKYPLLLRELIQATPANCANLKELESGLLISKTV

AQSINENQRKTENHEVMKALHKRVTNWKGYNIAKFGDLLFFDKVVITNASTNDNGGVSAQ

LDTNGTQTNKQFEVFLFEKIIVFFTEVSDPSDTKASLSTSSHNDSSTKNSLMKTASSTLN

LKKNKNHTTGSSNNTSNSSTAARNSQEPAKLDLRGRIMVPTINKVMELPLHSLMIQWEMP

KEHGNFVFKFKNDESKDNWFICLNNLMNQYKFANDFSSSPSTQSHQSVGSSALNTGKIRS

ASLSSSTSSLSSPMELSARPTNSTISTASNGSHQRRNSNRSSANNLRLSSSSNQHVKRHQ

HYNSIISVNSLQHISESSPHSSMVSASRQTTTTSAFESEYRNISENYKHSISENDLLCRV

QFHNDFYTILISIDFTFEQVFRAIHRKVGHMGEMQKLKYQDEDGDYVTLQSDEDWDMVKE

WLQENNESMLNVIAYN

>model.g444.t1 Augustusgene.g444.t1 JFAV02000103.1:63464-65194(-)

MALSSRNVIDLHFIADHDDQSLMDLHFTEIIERIFFHHNLNWFVVKNYMLFEKLLDDLYD

LNGIENFLSGGDNEDIDVLENEKYFSVQYHPKGDGDSKSKSKNSPPNVQCGIVYKKLHSL

LTFFEKIITRVLRQSSSSHDLDMPANRKEHVQMRTLLTKPDEAATPDTQTEASLSFLPNH

LFDNHEFLWSYISLVPVVQDLKIKKPDNDNELVTLSCDDELLLFHVNHELLKRDFVNFKY

FTNSSTSLLPFTVLSYANEYDKMQMCAYNDALAKDLQIDLPARLIENKEDAEVYNQHKLR

STELEMIKLIKKIDSKILFAKFDFANNDSKLLRLDDVDLELLKKLLYVWPTCYTIATGDS

LKARNRTLPKDNKIYIDYYVLPQDLNSQQKRLRMFKHNFRQIKHFKDIERGALQGHSTSA

KSSVSAKNINVSPGKVTKPRTASSPQKRTNNTVSTRRSIFKTQDNSTHAELLQKVKAKEE

FNRLNFAKNQQEIQENYNRLFEHNLQKIEKILFVLNKSQPYTINKLMDLITDSLSNMEML

STEEIERILMELHNRNVVECVDYNGLKIFNIIDRIKN

>model.g465.t1 Augustusgene.g465.t1 JFAV02000103.1:109894-111489(+)

MEPQLKKAKLSQNDDKKLVKNAYKGYTLPGYTAGDDSSENQDEYKIDAISMHDILKLGKD

SSNWFYENYIKLRKPCKITGCESYKDMAQLYELLDRENIAKNLQNHNENDDEEEEEEEEE

EEELLDVEKEVDGGFGSGKKRLSMTFGEFWETIGKNDSKETLYLTTQYKNDPYLDHEEDD

DEGEAEDDEEEGDLFPLGSDISDAESISFDPSKDDYVDSDADCGGDESQSDKTEEYVQRM

VELYQRPLTSIADKIPPNLANLTNTLLAQQINLWIGSTKNNTFKNADFFQKLLDNSKKGT

NESSTNTLGKHVPGNKLSSGLHHDHADNIYIPVEGTKQFTIYPPNTVFDMYTVGDIKNVY

SNGVINYVRNQKAPNWNDLKEDGTINDECGSNSDKFDNKTTAAIPDPPSFSRINPIYLHL

DELRSESEDLYNTLLLKGQAEYPNFFAHNLHEADNKLVINLNKGDALYLPSGWFHEVSSI

GDLHIALNYWFTPPTINANNFNDCSHLYSDDLYSNNKENMAAREWYIGHFKG

>model.g481.t1 Augustusgene.g481.t1 JFAV02000103.1:144335-146207(+)

MQSKHAVKVVVCGDENVGKTTLIANFVKNQYIPHLQKRLTPVSISKDFSDSYSFPETTVV

IDTTLEDTSHLQQSLETADVILLIYSDDKSYDRISSYWFSMFRSLGLNLPVIVCRNKCDL

LSSEDYELVDDEDFLPILNDFKEVGSCIKASCLDNHNVDRTFFLCQRSIMFPLAPLYDSR

TQALKPSCASALKRIFILSDKDQDGFLNVHEIAVLQKKSFNKSIKTSEIQDLIQNIQELN

IPNTCSRTRGLTKEGFLLLSTLYIEKGRYETVWGMLRAFHYTDTLEIDASILFPNIGIHQ

NSTSSIELSKIGYRFLVEVFKRYDKDNDGGLDDFELRQLFKSCPEGIPRLWIENSFPNSA

PTNNDGHITLQGWLAQWAMTTFLDYKITTKYLVYLGFEQNAKIALEITKPRKMKKRYGNL

YRAPVSDRKVVNCFVLGKPNSGKTALMESFLNKQIVDNGLYSPTIRPQIAVNSMELKGGK

QFYLILQEFGGSETAVVENEAKWSECDVMCLVYDSSDPGSFAYLVEMIEKYSFLKKIPMV

FVGLKADLDRQQQRSHFQPDEFTDSLFLDHPLHVSVYWQSSLSKLFRGQLQWPLQV

>model.g455.t1 Augustusgene.g455.t1 JFAV02000103.1:86642-87048(+)

MLASYKRLGQIRLTNLNTKPQNPKELCLQMINFVSIIDANDKPLIIECNTSDTEASNTTT

TTNNNNNTNNNELAWQVKYHKLTNMSLDYFESPYFQPTGRPGEQNEVCYWDQ

>model.g484.t1 Augustusgene.g484.t1 JFAV02000103.1:152876-155344(-)

MPTSKSLLKIVFSFFLLLQIFNPLVHADDAATTTTTNTATTTTAGGDNVLGISTSTVNVK

NKSPSKLLRTSSLLTCMEDSQFSSSFFDVTYYPGNTSIVFNIEAVTTISSKVEIEFQVIA

YGLNIIQRSINLCSLNEKSLCPLSAGRIDISSTYQITSDVVDQIPSVAYTVPDLDALVRV

YAYSLDDDGNADTSDPVACVLATLTNGKTVQTKYASWPIAAISGVGVLTSAFYSVMGHSA

TSAHIASNSISLFVYFQNLAITAMMGVAKVPPIAAAWTQNFQWSMGIINAEFMQKIFKWY

VQATGGTSTNILANRSILSIYVQKLKNKVKHKLAKRAITLSGSFDDSVFDDANVYTTNER

NITAVASKTLLVRGIERVAFKADIELSNFFLTGIVFLLFFIFCMCCLLFCLKSLLELLVR

TRAMNPDNTRFFEFRKSWGNIIKGTMYRISLIAFPQVTLLCIWEFTQQDSVACMVDSAVI

LLIILCLMVYGTTRVVIRGFQSSKIYKNPAYLLYGDAEFLNKYGFLYVQFKATAYWWLVP

LISYSLLRSLFVAALQTKGKAQSLIVFIIELFYFVGLCVFRPYMDKRTNIFNISIHLINL

LNSIFFLFFSNLFGQPMIVSSVMAVVLFVMNAVVALYLLIFTIVTCVLAVLHKNPDSRYQ

PMKDDRVSFIPRAGGMAVMDNKAAELNDLSKAVMVTNETENQKLQRDNAFQLGSQSDSSK

RKLLLDDDNEDYELESNSIWSKKDSFGRQQQTQLDDPDVVTRSESYLDVQQPLSSLNNQN

HNQQQQHPYSNSAQNYPANNNNPYQNFTSNTRNTNNNPYSGYR

>model.g490.t1 Augustusgene.g490.t1 JFAV02000103.1:166045-168249(-)

MPNPSVIRDSNDIDLLNSPNTTQNKTTGMRSTVRASTGSTSTNDEKAMENGNLMSNAEAT

ENAQHLPHLMTPYSLGHNSNLDDTFILQRLLNSGGPNNVSNLAPPATGNRNITDFNTRPS

SVFQWDKISNDFLLSSPDHIRDVLQQSISNHPPFSNNGSLNNNNNNNNNNFFNTDSPVSN

MLNSLFKTPAKSTLGNNPNSRMINNSGFSMWNLNSTIKKLTPLKFFNESNKKMDPLLLQT

NDIKIESTALNNNKSNEDSMKEVENTIQGNNVLHGPTDLQSFIPVTSYTGEAVTIPNIGN

MSHPLSIQYQHRSLVCASPSVNRVKQPNLKRKALEPIDNVLSNISDNTVLQVMDPSSSKP

AAQNKRKPVLKKKRKLNSTTTSLPDFSQTMKKSTFTNNTFTNNTFDQNDKESIENEANYF

NKTISKKQVSHQSVQPAGVPSNNTTATEDDINSQEDDLDACTDINSSQATIDLDGSASKP

REVEFSNTSDNTFMNVTVNKLNTSPTPKFKKKTGNVLPKLRRTVSSAGSSGNNNNNNNNN

NNNNNNSNKRISSVPELPKMGSFSNGHGSTTFSAGQEGMGNLQYLPQKKQGNKRITSLPI

NHQSHSSSNGKPMHSNKKRRSSASGHPNTHSNNNNSNNNNSNNNGTSHNKSFQIVFTDPN

LFESCNTSSSTLVNTSFNSSMNHGTHHHHNSSFSTMNTPIHSTSSRVSSSNKKFKKQKNS

LKRSKSENIKFVQRK

>model.g460.t1 Augustusgene.g460.t1 JFAV02000103.1:97312-98343(+)

MQAENNIAAGVYPTDEPGTPLAEEGAPQPELAELDQDDLMLLDVPSCDNIYLKGNPAALN

ATETLNKRAFEEVNQSDLEMDDVSEENDTYKPSIQFNDKIMTPPVYPTVLENSHREASKK

KYSSNQQIKFMNFCENEFMKVQRKYVQSRGLKLGEIPKYSYYDALLPLLQDYKRIVDFVW

YSMDTSCKNTENLIPSPLSTHSMQLDIDAGESTDEGYDESHYSSKSLRRLLNEAQNIGQV

YILLKIADDLMDYIEKFNTKGDSDKETIKYIFMLHFILDRIFSLLIVEDPELRYGVYMTP

TEIVRCKGICERTRISLSKYFQDCGIKGFHYELSKIYELTLERI

>model.g451.t1 Augustusgene.g451.t1 JFAV02000103.1:77766-78611(-)

MVEDENLATPNACSSRTHLQLTRNTYSLRSVTMSGGNTDVLARIQEIEAEEAENSQYKNY

SRYARPKNIRKITSLQECYNWRKQIIKDCDQAYSKLFETTLSEDDVRNYNDLYNQYMDEL

NKWDKHIVQDLNGHAFKNKRTEQSVSKSRSSRRGQGKARFMVRKSIRKINGKWYVGKALE

LPEIKDELREIEAIQIEKRAHLNNKAFLKRIGKSLDKKEYFKEAPTGLVLPDALLDQVAL

SKNEVDELQKQHKIPEFAVPTRKEMEQWLVNKRKEKLKFVLE

>model.g459.t1 Augustusgene.g459.t1 JFAV02000103.1:95392-96924(-)

MNSQKGCTALTGKDIIYGYIELITKSTKTIKKIPIHKAKILKFGRNSELCKLHIDNRVIS

GTHCKFWGIQFDENTLPVCYVQDVSLNGSIVVSKATKNNLNLYTHHKLVKYECHPLQNGN

YVKFPTIEDFYLRFTSHSQYANGDCDDEHVFKDLKINKYVHDEWVIQPIFVGAGTYGHVM

ITKAKNSGRVGAVKIIKNMSAKDRSLTQDQRNNHTMRETKLLLELDHPNIIKMYSTVISN

GISPNYKDYYIFQQLVFGGDLFSYLAKSDSLMPVEEEECLLIVYQILLALKYLHARNIVH

RDLKLDNILLRTPEPCTKIILADFGIAKRLTFADRLHTVIGTPEYCAPEVGFQNKQVFCK

SLTKTVAKQHEKEGYDFKCDMWSLGVIAYIALTGVSPFFQGAESTTENMFDKMLSGNLNF

GAIQWANITSEAKHFVQSLLTVDTKKRLGAEQALQHKWISQQSVILQKIYKKKILNEEPC

FEIQTAVKYPEPPRKKTKVQPSVVKSYSCSL

>model.g432.t1 Augustusgene.g432.t1 JFAV02000103.1:30314-32638(+)

MPAGSYSYVDPNAQSEVANFNKFTTIDWPEERVKEAKEKKSPHLSSTDLEGPDLRTHHEY

VNRSVWNSVHKYFGHIFRQNWTTFLTLTAMGITIGCIAGFIQIFTESLVSWKSGYCSKNW

LLNKNFCCSESKEFESQCVAEGFWINYSNGGLKFVVFVVLSIVYAVISAIMVKLVAPMAA

GSGISEIKVTVAGFIYNTDFYSLSTLLIKSLALPLTIASGLSVGKEGPSVHYATCCGFII

AKFFLQKGRQHLNKWSSRSSSLSQYGVNFPELSEYMVAGAAGGVAVAFGSPIGGVLFALE

DIASSSDYNLSTLWKSYYVTMMGIATLKAINPFRNGKIVQFEITYDKDWHIAEIPIFVLL

GIFGGVYGIIISKLNVYMVHFRKKYLSNYALQEVLILTVLTSLISYFNQFLQLDMTESME

ILFHECIGEDDATWDHQLCSISNASAFKFVQMCSSLLFATVVRAALIIVSYNCKVPCGIF

VPSMAVGATFGRFISILVEKFITGPNVITAGTYAFLGAAASLSGITHLTLSVVVIMFELT

GAFFYIIPTMIVVAVTRMIFANYGTGMGIAEQMIVFNGFPHLEDKYDHHLSVDYNAQDIM

CPKLQTLPEYISIRELKTILSSSVQDKVHGFPVITVANKCIGYVKRQNLETFLYMNNIDD

LTIDFNKATLGENAQQVDQLESPENDFLMDKSVIVVKSAMKITLIHDIFKKVGPKVVMVE

NENGDLTGLITRKDLMSFESSKHTELNGPLYTFNESWDDFLWSAITKIKAKLGSP

>model.g477.t1 Augustusgene.g477.t1 JFAV02000103.1:137873-138208(+)

MFLKTVPSKLRIHCFRVGHFPQLATLRHNSTLSFQTDEEKNIYNKLKQTLHNPKELLVQD

ISGGCGSMYKIIIQCAEFNNLTMIKQHKLVNSILKDDIKRWHGLQLTTKKDL

>model.g429.t1 Augustusgene.g429.t1 JFAV02000103.1:22185-23924(-)

MAKKLYLDNTIEFLRGRVYLGAYDYIPEDTSDVTFFTMEEELFYNSFHLDFGPFNIGHLY

RFAVKLHDLLTREENLSRAVVFYSSTSTRQRANAACLLCCYMILIQSWTPHQVLQPISQV

DPPFMPFRDAGYSNADFEITIQDVCYGCWRAKECGLIDLQKMDLDSYEYYERVENGDLNV

LTPNFIAFASPKENIADMNNNHNTKINRTFRKVLDIFQKTNVQLVVRLNSHLYNKVHFEN

NGIQHLDMIFEDGTCPDMSIVTNFIGCVETIINEFDDLNDQSPKIAVHCKAGLGRTGCLI

GAYLIYKHGFTANECIGFLRVIRPGMVVGPQQHWLYLNQQTFREWKYTMRMSIKPSDLIN

GMYPLISLEEYKKQKKERLQQQQQQQQHGKKLQLLQHERKMSNIDEGLNGNNKDIDQADL

SGIIIPPETASPMTHNPRHGVPMRSPGQPRKGQHGANTIEDINNFPGKRTKSKTAIVNKA

SSSNNNANMTQENVENEADVSMTSRRNITPIDDQDMKMLYVAKQQQRRSGGRNLSSSSSA

ITNNMHSANHLGQNGSNFSRAVSGGSVRKISGSYRKDTKY

>model.g448.t1 Augustusgene.g448.t1 JFAV02000103.1:71167-72303(-)

MSHPYTTAANDFRSDTFTTPTKDMLDAALHCSIGDAVYNEDLDTICLENKIADIFGKPAG

LYMCSGTMSNQIAIRTLLNVQPPYSILCDYRSHIYTHEAAGLAILSQAMVTPVIPQNGAY

LTVEDVANWVIPDDGDIHGAPTRVVSLENTIHGIVTPLNELRKIKEFCLEHNLKLHCDGA

RIWNAAVKSEVDPKVYGSIFDSISVCLSKSMGAPIGSVLVGGKKFITKANHFRKQQGGGI

RQSGIICRMALVAVQPGWEAKLEYSHELAKSLGDFCEKELQIPLEVPVDTNFVFIDLQQA

KINPDVLVKKGLKYGVKIMGGRISFHYQISRDSLEKIKMAIKETWEYSKKHPFDHKGPTQ

IYRSESVDIINTDIQTYKY

>model.g446.t1 Augustusgene.g446.t1 JFAV02000103.1:68573-69034(+)

MAITISPLTKIIFLSGLLALGFLLVILSCALYSNYYPLFDLLLFLLSSVPRAMFPAGRGG

AGSAGYSSFLDDSQGAGGALKDTGTYFTSWFLTLAIGLPVVMEHSNVINKRAMILSITGG

GIIYGTIVLFGWFFSNGGSWLDEQDDDEYDGFGY

>model.g467.t1 Augustusgene.g467.t1 JFAV02000103.1:116336-118297(+)

MINSSVKRSVARLGAKQARFAAPRLRVAATSARFYSIAQFNSLINGRKQFHTFTPTLKSV

ETPFNKLTEEQQALLTEERAIDRADVVIVGGGPAGLAAAIRLKQLDAKHNGNLRVVVLEK

SSEFGNHTVSGVILEPKALRELFPEECEGKPATYIPLPEDLVTPVTSEGLKFLFQEFGAS

VPVPEPPAMVNKGKNCIASLSQVVRHLSECAENLGVEVYSGISVSELVFDQAKENVIGVA

TKDMGISKRGEPKDSFERGMEFHARQVFLAEGCHGSLTKKAIKKYDLRKGKQQQSFGLGV

KEVWEVPPENFKKGYVAHTMGYPLSNAVYGGGFQYHFGENMVTVGLVVGLDYKNPYVSPY

QEFQKMKHHPFYSKVLKGGKCISYGARALNEGGYQAIPKLHFPGGALLGASAGFMNVPKI

KGTHTAMKSGMLAAESIFDVLKNEETSFSELEDESTIAALPTIDLKSYEENFKKSWAHDE

LYEVRNIRPSFNTKLGNLFGMCWSGLDSFVLKGRPGFSFKFHETGDSGITEAASQHKPID

YPKPDGVLSFDILTSVSRTGTYHDEDEQCHLRVPDQDLEKHAEASYPKYKGIEQRFCPAG

VYEYVEDPESPIGVKFQINSQNCIHCKTCDIKVPTQDIDWVVPEGGDGPKYTIN

>model.g442.t1 Augustusgene.g442.t1 JFAV02000103.1:56344-59328(+)

MTDNSDSLFNDKVRRFQDFLDSYSNYKSDIKSILVHNAELELQKQKPNINYSTSLYEDEG

RKNAEQEDEELPQRLIISLDDLREFDRVYWNGILHYPHEYIPPAEHSISLLAMTEQPTGA

MRPRHQTNVQWTLGFKGSFGANTLTPRTLNSKYLNKLISIQGIVTKTSLIRPKLFKSVHY

APKTSRFHYRNYKDATTSLNTPIPGGASSAGAETGEASTSQDQMALTSAIYPTEDTEGNK

LITEFGFSEYKDHQRITIQEMPEQSPAGQLPRHLEVIIDDDLVDSVKCGDRVNIVGVYKS

SGGGGLMDKKGLMGLQGFKTFILASTVWPLHARSTGVGAMENVSAKDIAKIIKLSNNYKN

ENIFDLLSRSLAPSIYGHESIKKAVLLMLLGGVEKNLENGSHLRGDINILMVGDPSTAKS

QVLRFVLNTASLAIATTGRGSSGVGLTAAVTTDKETGERKLEAGAMVLADRGIVCIDEFD

KMSDIDRVAIHEVMEQQTVTIAKAGIHTTLNARCSVIAAANPILGQYDVNRDPHHNIALP

DSLLSRFDLLFIVTDNINDIKDRQISEHVLRTHRYLPPGYLEGEPIREQINISLGVGVED

EDNGADDDDDDIDDHNMDDIIDDEFHADNMSSVFEKFNPLLHAGAKLAVNNGDHNGTNVP

LIVSIKFIKKYIQYAKERIVPQLTQPAIDIIVKAYSDLRNDITNKKLPITARTLETLIRL

SSAHAKVRLSQLVETEDATVATQMLRYALLNEDPANEKNNGIPSPQKKSPRKKSPRKKMK

PSPKKRTRTAATTGEEIASEPEQDDEQLISEGRTDLREAMETADLEERLERGLRISPRRQ

EQMQTRRGVLSPLKDTNILESPSKKGRTSRSDDVNLGPGPYSGDENVMPSSPLVPVAVPF

EDPEEDDDQDSESAEISQERLSLFNSVFAPLLSDSSLFVDDHCEMGKLFEAVNTHLKNRD

PDAEPFSQGEYMAALEALQATENIMLADGLVLRVV

>model.g454.t1 Augustusgene.g454.t1 JFAV02000103.1:85057-86517(+)

MSASQLPVVVVGSGLAGLSAATQLITKYKIPVVLIEKADKIGGNSVKASSGINGVQTRTQ

LENYHITDDNAEVFYQDTLKSSKKLGIPKLMEKLARDSTKSIEWLQNDFQIKLDLLSQLG

GHSKMRTHRSSGKLPPGFEIVSTLQKNLEKIKETTPELIDIKLNSKLSNIKINSITKQIE

YVEVTNTQDTTASSSSNGVQQIHTNVLVLCTGGFGFNKEMIYEVTKSKFLSELPTSNGQQ

TLGEGQQLMQKLGAELIDMDQIQVHPTGFVDPTDRKSNVKFLAAEALRGLGGILVSPVDG

QRFVNELDTRDHVTDAIFNANLPDNVALLVLSEKVYEVFANNINFYIFKKLIKKTTVGEF

HKEHDVAGKWGSQEKFITQLIEYSNESTQDKFNREVRVNSFGDSINADTVIYVGEITPII

HFTMGGVKINEKAQVLNNKNELVAHGLYSAGEVTGGVHGANRLGGSSLLECVVFGRQAAD

SIASSYA

>model.g435.t1 Augustusgene.g435.t1 JFAV02000103.1:39237-40231(-)

MSDLCPVYAPFFGAIGCAAAIIFTSFGAAYGTAKSGVGICATCVLRPDLLFKNIVPVIMA

GIIAIYGLVVSVLVCYSLGQKQALYTGFIQLGAGLSVGLSGLAAGFAIGIVGDAGVRGTS

QQPRLFVGMILILIFAEVLGLYGLIVALLLNSRATQDVIC

>model.g466.t1 Augustusgene.g466.t1 JFAV02000103.1:113485-115593(+)

MASATPIQPAVGSMDPQQHEFIHNSQSPVFAMSPAPAEAINSGTLVNSSAEATQVKDSSS

DAVALPEKTKPASNSAENKAEEPDANGDSTTESTDISKKIPKSPKSPKSPKSSKAKKASN

TDEAGASALSDTSNSKVKTAKPVKLPAPISDISKNIPVTGEKPKPEKDTPAMEDEVLHAV

FVILYEFDTKEEGMTVKQMCDHLLERHPDMAQLSTKLSNLISAKLNAYVKKVEKGEKTLT

YCLSREWADTSPRRMVYVYRGLLAPDYQTFAQEATKKMQEAQEQQRQKQLENGDLSGSVG

LNYSKMSNSPMITNAENIAFSGMGNSTLLGSEPTYGASSLGGNPKGNNNHMDYHGFGGNS

RMSKTPVVSNAFHFAMDGMNNSTMQNINRFSITYDPRFSSLPFQSTNNSFSSTSISSTDH

AGGVVHNFSDSSLMSLNSHSNTLKSNNNKHKVKGFSSHHTQLDPLAEYGNENDAVRNQEN

DDDEDEENNEDEYIYNTDVDAIASKRGAFKQLNQKKGKMTKKNGASLSSDSSKQSYTTNT

LFNGKNSHIGGKNDSSSHLPQAKYITAAAAAPRLTKNSFQNDHFNSPQVAAAVAAIQKAV

ITESPVAVDLSYTSSSFSSVSSQKSTTSSSLNASSIAKMSQSTSSSSPGSLNSASSLTPN

SSKSSSPEPVCAQWLKAVRAGFLSEDIVKPENVSIEELDGLFD

>model.g427.t1 Augustusgene.g427.t1 JFAV02000103.1:13644-15692(-)

MLNSFKGMQSIPRLCTRQSTLKTSQFISSPYTRSQLFHTSHVHHNSIKRSAIKVAKYGIF

ATGLLVSSAALSMIGFFIYDATTYKTDTKCDAIRVPQLAISPETGGPENLPILSTSLDSL

DTKDKASIAVNKPKLVILGSGWASVGILKSLRPGDYDVTVISPTNYFLFTPLLPSAATST

LEVKSLMASIRSIVKKISGHYLEASAEKIEFADKLVKVTSNYNTNDSFYVPYDKLVIAVG

CTANTHGVEGLEHCERLKYARDVIAIRKKIKDNLERACLPTTSDQERQQLLSFVICGGGA

TGTEFAAEISDLLNEDLPEQYPKILRQEASVHIIQSRSNILNTYDERISEYATKKFLKDD

IDVLINSRVEKILPDKVIFKQMNVETGEYDMKELPFGLCLWSTGIAQNPLAHSVVETLGP

YFQKNKRAIETDSHLRVIGCKDVYAVGDCSTVRTDLANYAVEEVKNFIIHKHHPNAKWSN

HKDFPDEEIEKMSLTYDDICQFGTGLMKKFPQAREHLITLEEILPDYDINNTGQLSYSDT

VKLLKDVESKVTTLPATAQRAHQQGKYLGKNFTKLSKKMEALDQDADSTITSATSLVPST

TTFPLSDEEFAKPFKYVHLGSLAYIGNSAIFDMPGYSFVGGIVAMYLWRGIYFAQTVSLR

TRLLLCMDWMKRGLFGRSIVNED

>model.g450.t1 Augustusgene.g450.t1 JFAV02000103.1:74814-77684(+)

MMGSGNSMSEKTNASFLTMILLHLNMINKETLQIEFSTNVLLVILSVFWLLYRLKQHLAI

PLNALVEKYHTISIPHNPTKISLDYIDDTSVLIRWENQSYNYNNKKNEVFVTKDNESSSS

KMPSGSSTDTLKNTRKTNGLNKTKKKSATVTNTTTTTTTTTTTTTGEPLSQEHTEKTETV

ENANNCSKSSHSDSQVDGTFISHYFLYINNTLIGQFFNNPNNNNLYTSCSIKNLKINHFY

KIDLVSVNKNGFRNQPLSVIVNLNNLKNEHGKSQANHKTKLQNVNRNFNTQGTFSASLKN

NIHENSTTAKENIGSSTLHWKPSLENDLTNGSDNASSQPGFSSSTSYSSLTSMHSLEKFS

LEQLKEILIGAQEELHGVIKYREELFEQYKENELTLGKSIEENKKKYGNYQELKKQQKAE

IIQLENQKNNIDFQKTRLTNKLIKLKTSLDNVIKKNEDLSRKVNEIKDTKPMSDLINIRD

KLLDSVSLKETSLKEVQKEMHGLVQKLNDLKKKLSFEITDPSSSQANGSKFGQTSSSKSG

IASNHSMIDQFDKENMNDSENEEEKANDDDDKEKTFDSVKELDMNDFNENYVNKSNGTID

TAAKQELQKLLIPNHYTEVMTKSDELKTHLFDTEETINSLNEKCNNLNRKLEQYTEYGIT

LTNKLIDLQKQQHQQQLKQQQLQQQQRQSQQQLQHSHPSPPMSSNIPLSGAQTGTRGGAG

TLVLHDPTTYEDNQSINLMRPANSSNQYLSYLLNNYQQQQQQQQQVSGGASEFMPLSTSS

SSLLLPEQNNLYGSKNSNLWNQQPPPTSLNSQTQPLNIKSSLNSLSSLYAAGVNQQSQQQ

QTLPPSSAPSSLTMSGMPIPSSLESQQQQSLNLPYYPMQTSLSNSYSQSSFQPTPSGSAG

AHAQSSVWNDSSNQKISPSSAQASLHSDAQSSNDPTSFRPYSSSSRFEMPKWSSSNR

>model.g478.t1 Augustusgene.g478.t1 JFAV02000103.1:138320-140674(-)

MTAAVEIENLSTLTEFDNIKLDDIQIDPEELAAFEKQFEEEYQISGNGELLDIDNFVVVD

GAPVAPESKIPALTRVLTKLFSQAGEIVNFELPVDEEKKTTKGYLFVEYKDSLSSKKAIK

LLDGKKLDAKHKLLVNSFRDMDTIASDDYNVEYQEPVLEKPVSYSELQNWVLDGKDQFVM

SKDQMSLLAWYKGAQKPMPVVEPRVNWSDAGNLKFSPKGTFLISMHEQGVKTWGGDELVE

LNKFAHYDVKAVQVSKTEKYLVTFSPTPINDDVFFPKESQNHNIAIWDIQTGVLLKTFPL

PPQDLVPFHWPMIKWSFDDKYCGRLGPNAIAVYEVENNFKLLDDKIMKIENVRDFQFAPA

GIALGRNPQDLSSVLCYWTPEMQNQSCKATIMEIPRKRVLRTINLVQVTDVQFHWQAQAE

YLCVQVDRHTKSKKSNFTNLEIVSFTGRDYPVEKIELKERVLKFKWEPKSNRFVTISIDE

ANLDPNVAVPQNIVQFFATTVGSDEKAPSWTVVYEITGKHSNTISWSPIGRVVVIGTLVS

RFVKNSEFLFFDMDYAGEHPLNSAQWTADYNKRKQAAADAAALAAQISLGINPNQNNRNR

NQPATNFNKKKATIGSTGPIKVNAAIKEIGISKFSSVTNVQWDSCGRFFAIWSSADKHKT

ENGYKIYTYGGQLHREEATVGFRHFTWRPRPNHVLTNAEIKKIRKNLKEYAAKYEEQDLM

ESNEELRLAILDRKKKLKEWIEYRTEAAEAVKPYETFDNFEYVSTFESIEVVKEDILEEK

KEKIE

>model.g493.t1 Augustusgene.g493.t1 JFAV02000103.1:173032-174396(-)

MSYEPEFEQAYKEVEDSLVQSTLFKHHPEYANVLKVLRVPQRIVQFRVTWENDKGEQEVA

TGYRVQLSNNLGPFKGGLRFHESVNLSVLKFLAFEQVFKNALTGTSIGGAKGGLNVNPQG

RSDKELRRICHAFMAELHRHIGQDVDIPAGDINVGGKVIGLLFGAWRQYSNTFEGVLTGK

GTNWGASPFRPEATGYGVVYITNDMIEYATKGKESIKGKRVAISGSGNVAQFAALKVLEL

GGTVVSLSDSKGAIVSESGITKEQIEAVAAGKSEYKPLSAIFAESQSDIKFIPGARPWTN

VSKVDVALPCATQNEVSGQEAEYLVKAGVKYVAEGSNMGSTPEAIEVFEKQRLAAASSSS

DSAVWFCPPKLANMAGVMVSQFEMAQNSQRVTWEPEYVDSLIKKHMKEGLMLSIETASKY

SNEGNSDALPSLLKGANLAGFIKVADAMIDHGDVF

>model.g424.t1 Augustusgene.g424.t1 JFAV02000103.1:3878-9394(+)

MKSPLAKEGNAEFPTADFAIKSVVKTEQDNSEIFDDSVYSTHSESNDTPVHPSEDTIKVR

SEETLPLPESSSIATEKILFEHKDTQEDLTDSTNSMTLPANLFDVLLPDEKLMAENFEDI

FNSAVFIKSNDPKVFGEFIAIVYNITGKPDTFCCYLFDKFGCTYHENVDISPVSKYYLAI

ENLNEANKKLNVPKVVAVSLLQKYAYAQKFNAGFFPNILSFDPTDAGMALNAHKLVETNS

KLTPLQLGQKMQQMSCLQNRTIKSLFIDVLYENTSDVIDDNNKLVFHLGNQLEQLFNPLT

EYSPEQTEIVYKPVPPADGSSLMLQDSTLIQSIIKELVDIQTIFTVELVNFLQKYIIPIR

IKVSNREIPGISFSKLNKLFPPTIDEVTRINCIFLDALKLAQNYGSLQILKACSVTIAYF

YKAYTRHEAATKKFHKDIKLFLAKFNDQLPLNQVYTELKLETIITGPQEKITKYKLILQR

LWKEKQWESEAVEEEATKYYNNIIDVIDTFGNQATPSSKTSAYQNRVFTPSGKILTELAE

KWPIELQYKWLKRRVVGVFDAAHYTDNKRDVVVIFNDYVVFLNVIDGDLYYSNGSNKPLI

SDILMNSLINEVPLPNNIPQLTVRKHVHINQLLVTTYGNTGKQIRFDILDGDSSPLYYQL

VSPTDTCEYICELIVKAGILCKSTAFHLFSKSFETSLVGSEVAQSSKLKTYVTAHEINTY

KGEKIKSPFAMFLNIDPSYDVLKENGVYFGAFLKFVDSSKKLVKLQILTAKKKNNEASQN

STTEFDESPIIVSLSEYTKVILNFLAKVHLKEFYYSLLSPLAAQIFSINEQVVKQVQEPV

KFFARGLVKSENLQKLPTLSTSTADTSTNMDKSANLQSTSLEDSAIHSSSFMDKSKSNST

IATFSSSSNQLNKKFPLFHTKNGEVETGIRHASTVITRPTDQTALTDKGSTSRSKIAAKN

DNIQKQVIKPPSKIVNSIHKKEKVVLQKAPKAKLVASNEPPTKEKNKNKNKKNRLSKVFA

SIFGSSSNSTKTDSSSKAPTAVSNKAAKRQSLPAIPSAAVTLAGAGNNEVPFAEASDKRH

AINKKRFSLTSIAPPSSATSPFKKTYDTGLSKGVTGSSNTSQSRNNTSPLKSTGPRKYSS

SNFSGISKTPISSPLKIDRDHLDADLEIPRMQSNIEQDKMFSNTKVLALESPLSVTQNTV

KEKETSKKQTTAAVFNDDLYGEILNHNEKYFVKESAEPSISETSSSHKASSVVQKEQKAA

LVPEKKSFDSLVGDDLTAGSNNKISHEDNEETKQKSVFDLPEQTIQGQRKSTGEMVFEKL

QSLNESKPNTNFSAVPTEKRCHFFESFQKNGTLPQSTSYRELFQDMEKVLTPYEKNWNWV

SLEKSASLENVKNAAEEGYWNQQPETDLLERYYEKVPISPKPDSHTADEDEQEAQNVEPS

ESGKRADSLHDSNLAESLDKNLDGQDDHQVNEEYGKSFVSESHPTNLTSVNDVKALQAVG

YTEAKSSKLGVEEQSTFSPPSNNSQILEDSQRVRMRINYQSKFRVVKSSSPTRTPLDSAY

STTTRPSQKLNLDEAIPVLAPTQSRSTATDSELDTKSDTKAKVNARFFSAGSESSELSSM

SMSSGKNNSGVFKRVMDLQNHNSEQLPSRDISFHESDLEKDLGDSTLEFSDFNITFNKND

TLDEKTEEDFATPKNVILQNMASPEFEDPIFYDFNTTQDYSNGGANSFGEKAMDYNNNAI

WVSPSKLDVFDLSKKSPSMYERLNIAETNKTGKLVFHDAPDNGNSDSGAISSKSGSNISH

EYQIEKENLDMTQTQNRLKKDENVKVLRDESYGYLSEVL

>model.g474.t1 Augustusgene.g474.t1 JFAV02000103.1:131122-133704(-)

MQENNNNNNNNNNSNSNNSNNSDIKDSSDKNGCNKELPVTNVYLVNTTINTTFNTTINTT

INSTINTTINTTINTTINTSKSISNEVSSHEESIKERKKEDENHLSQTIDAITTNNPNIK

VTMFETITQLIVHIFEMRLKGSLHFENYSTLIILLSNSQMSNTETRFHKENQSTTSNQQQ

TTKSHTNGKNKIDSITNGSNARSISTPHSQHRLDTLDSQELQIVFNKIFGLLQFHITGVP

FNETSTTLKQTILNYNALNMSNFHPVLKNRMRRVTHWCFDTTDSQQESKNLQNTKTSTTC

SDSTYSMYTQLSKLITANTKHSVPDSYKDLNISELLAHIDLSQILHLPQGKSAKSYNSCL

NTWDFCATELTPSELIQIGVNILYPLITNDYKDSSNSLQILQTQDIVGKENSSSSSSSSS

PSAPSASKSMSVSKNEVFLLMFIVECNYHQDNKFHNFKHAVDVLQASHYLFLQLVGESCK

KSNPDSAQSSSISNHICACSKKELTSMLLLLSLSALGHDVGHPGTNNKIMQENTQMLQKF

YENSADSILEKFHYKIFWNFLKDFSNILDNCYAGKHHINDIILATDMAKHYQYIEDLSTT

NSCCSENLDGNNKCLSDLANCNKRSFSSLNIDSMHLSQNTQNSITTKNSCSVSESCHLSK

NSLLVLELIVKAADISNVTRPLLISTKWAYLICLEFAECTKLTEQNEKYAEALAKADAAE

AKAADAEAFAAEVCGNEDEAKEMKKVAERAASKARLEATEQTEKIKETSQSKNINTHRNS

VTYSSSSSDDEDTDPEPDLMDQCLSLQPSIPQGQLFFIKTFAAELFSKLSAKFPQHFAFL

EKNLNENRLFWEEYIKENEKK

>model.g436.t1 Augustusgene.g436.t1 JFAV02000103.1:40660-42006(-)

MTTLDQFVHESLLNNVSLEKHNSSDVSVVDIAQKFDWNIAQSRKAMYDYYTTEGQKMSEK

LTFNMMCVYTEEKEIGEGQHSHIKNFKLIKSLKDEKFVLEQVAPENIIDMFVYSIGLKHH

GASEGEDALIAASLKPVQKVFVSHGRKLDLSENAMVQDKKSTGSKPTTKSTADITKRSAT

EPAKSFEKRKVTSVADIRAAGAASASSAGTSKTKGSDVNAKLNTNSSSSSTPPKKASSFM

GLRSMDFLAKRKEQDELKEQQRLKELREKRAGTTTAKNTLSNAQSVAKKPKIDVEKQKKL

KELESMFSDSDEGEDNEVKALSTTNSNKIEISKDTKAQTDLAEVENLFDTTNDDSEMVEL

LTKEKPQQQQQQAEEVPPVKRSSPLKKPTTYVDEEGYIVSTNLNANKRPAKRTASTLPIV

SSTRTGGSSDTANKKRKQGNLMNFFSKRK

>model.g443.t1 Augustusgene.g443.t1 JFAV02000103.1:59696-63319(+)

MIEVKSPLVQDSKLLKSRALLARPYVFPFIPLYAFFYRVYTQLYDEYIGGQEWTFVYLGS

ILSLNLLFWLMPAWNTSINLLFNYSEVSTLEEAEFIYVKTTPNNGSDDIVKIESAFEQNT

VQIFFQFQKKRFLLDDAKQTFASPKFSIDEPLKLEFFQKNKGNSGDLTHARRLYGENTFD

IPIPTFLELYKEHAVAPFFVFQVFCVALWFLDEMWYYSLFNLFMILMMEAATVFQRLNTL

KEFRTMGIKPFPINVFRDGKWTLLQTNELLPNDLVSVVRSNDETAVPCDLVLVDGSCIVN

EAMLSGESTPLLKESIKLRPQNEFIDLEGIDKNSILHGGTKCLQITKPDSPIVKQAPDDG

ALAIVTKTGFETSQGSLVRVMLFSAEQVSANNKEAFAFIGFLLIFAIAASWYVWVEGTKM

GRVHSKLILDCVLIITSVVPPELPMELTMAVNASLAALSKFYVYCTEPFRIPLAGRVDVC

CFDKTGTLTGEDLVFEGVAGLKSEDGNALTQPDASCEETSFAIGSCHALVKLDDGEIVGD

PMEKATLKACGWTVESETKVSHQELGTARVLRRFQFSSALKRSATVSSYKNKVCVSVKGA

PEVLRERLSSIPSNYDDIYKHYTRNGSRVLAIAVKTLDSNAKVDKLIRQDVESGLKFVGF

LVYHCPLKDDAVETIKMLNESSHRCIMITGDNPLTAVHVAKEVAIVERDVLIIDKPSESH

EIDYSADDLIIRNVEETYIKKFTPSVDEFEGIFGKYDLAVTGYALNLLFGHKQIRELLKH

TWVYARVSPTQKEYILNELKDMGYQTLMCGDGTNDVGALKQAHVGIALLNGTEEGLKKIA

EQRRIDGMKQMYDKQCDMFNRWGKPAPPVPEPIAHLFPPSQLNPHYLKALEKQGTEITPE

MRKEVDKAMKQPLKAKENKPLRPEDIKAGDMSDLFMNALNSAGGDDDEDSPVLKLGDASC

AAPFTSKLSKVSAVTNIIRQGRCALINTIQMYKILALNCLISAYSLSIMFIMGVKFGDGQ

ATVSGLLLSACFLSISRGKPIEKLSKERPQAGIFNVYIMGSILSQFAVHCISLLYLTNEI

YKLEPREPVIDLDKTFEPSLLNTGIFLIQLIQQVSTFYVNYQGEPFRENIRNNKGMYYGI

VGVFALAFAGATELIPELNEAIKFVPMEDSFKGKLTGALLLDFFGAWAGEQFFKFFFMDD

KPSDIAHK

>model.g470.t1 Augustusgene.g470.t1 JFAV02000103.1:123707-124390(-)

MKPFQSFSTSIAQALPPDQHRMVSSTATYISTTPGSCSANNNSSSGAHPEGGVAKSTKHT

PTPPNTETAKKTALSNSMILNQDSINYDEPENFLEVEIINAKTHYSQESNSRDGSITPQY

TDYEIVCRTNIPCFKKKVSKVRRRYSDFDYFRKCLIKELAVSGNKASSKIAVPSLPGKIY

LSNRFNGDTIEKRRLELQEWLGFVAGHPLLQTGSKVLVRFVQDDSFVG

>model.g452.t1 Augustusgene.g452.t1 JFAV02000103.1:78918-80510(+)

MSQHIFRSYRFIIIAVTAIMLILIIKTSTDHHTSITENKHYTADDVSINLAGQSNENVPS

GKVAIVEESASPSASADALLATSSADAKKTPSTSSEVCNKDHQYIVIIDAGSTGSRVHVY

EFDVCTSPPSLIHETFDMLKPGLSSYPDDASGAALSLNPLLDLAVETIPKKFRDCSPIAV

KATAGLRLLGADLSNAILKEVRSHLETNYPFPIVEDKDAISIMGGDEEGVYAWITTNYLL

GNIGAPERLPTAAVFDLGGGSTQIVFEPTFANKNEKMIDGEHKYKLEFGSEKYTLYQFSH

LGYGLMQGRNKIHSLLINTSLKNEAVTVSELASGDAELVSPCLPPNTTFSDKVKMEDGTS

YSVTFKGPKTPAGPQCRYLADKILNKNAVCESKPCSFNGVHQPSLVKTFNEHNDLYVFSY

FYDKTQPLGMPLSFSLNELKDLARMVCNGEEVWQSVLSGVEGGVEELQKAPEYCLDLSFQ

VSLLHTGYDIPLHRELKTAQTLDGNEIGWCLGNALSVLDGNNWKCKVNQLE

>model.g491.t1 Augustusgene.g491.t1 JFAV02000103.1:168871-171588(+)

MNGKPHNLRPRASEDMDADSSMAHGGAHSENEDSLDAGSPLKNLSKSYQLGSIGHPDSKY

IALEIDNMPSLQEENDKYSRQKPMSEQESVLNDTSNNNYSSSFAEYDTVINSPEKRPASA

QEEIDYADGSPQAPAQGKMENAEAQWKNYFQSRNRPQLVNRENILGSYKHNNEHGAGTIG

NRVENKGDHQYYHNFLPDVSSHIIKHDPSEDEEDSKSGRSHSFSKLTESHDENDKSIFHY

EFAEKTDDYTSSFYNEREVNENSSKFLKTSKQSGHEEQKGREEESNEEPNNTFLKRKKGI

SAFNVPQSIPEYHMTNASDHSNVSGDPAPDARNVFANLFASNIKPLDNTNSNVQKHYVNA

DAVQHPTTSGLSNATTAIASATSNPAFQQNAALKRDPEMKFSGHKKALSTTTPQDVGMFY

DEKTGVWANPHNSSSMGSANASANTMGDPTSESSRHTVNMSDVQSGNLSRKGELRQPSLK

TSSKADVAYDDSLDYADTSVIPDPVISPLDQELGKDAKSFLNEQETKCKAILIERLIQLA

GTSLGSVYELDISNKMLGLDVQDYFYLIFEILPNLLELASSNTNLHEVLLPTYTNLEVLN

LSNNKLREINSLLSGTHLHLRSVDLNHNFLTRFQVSKPETQFFRLEKLDLSHNSLSGHFE

LKILENFPNLETLDLSHNQGLKHITLTPGTKPLNLLLNNNSNLQKVSIFEKELHPNTTVF

HKLEMENCLPTFIAYMNTKFHDDIAHMVVSSITPPTSKTSWRFARSLKSLVVEKYWSEDY

WYRLNKKLEHLVIRNIVSDINLSFSNAGDSCLRNDLGNLHALSIANTNLSFPLSSKYEFL

FKNLQIRNLHELNVKENVEIKSKIYTPFDIYDQDRVERLYRHGTQKKFGGSLTVLDGEAG

FFDWSV

>model.g431.t1 Augustusgene.g431.t1 JFAV02000103.1:26025-27980(-)

MSYTKAFSISAVITVLQFASMAALIIACVTAPVFHQISLSSYNGYRYGVFGYCTDEGSNC

SKASANYNAYSFQNLKNEDTWKMASNARKTLSELLLVLPVSAGVVFISMVFNIAYGFISS

GNVFLFLVDLLLTFLAFTGSAFSCIVVFLLFYPHVNWCAWILIPSAVLNLVSLPLTFLVY

TNKRNAYFDNNDSDNEDTESTDGYSSRLDRGLAKTNLLNREDTAVSNEKNEVSITNLNYK

DDDFFTQGIPKAYQSNNNSSTVLGSNLALKTTAHTNNSDSFDFTNNNNNNNNNYNSSSNI

NDEKDDNDFDMSLDDVNKQQPFSAVANKRFSSSSNAYPPITEQVPTSANSGKTFPQSVRD

ISDSSSKYSEMPINNQNSYPSLRQPNNKFGMQHPPTRTDTLNTADLKPGSVLQLKDDAPP

TLSNYMKQNEAIDEEDEEFIRKNTIDPAERPEIEDDGISDPGSDFTSVSQRGINPEYFRR

QQMQQVRPGPLSAQQQPQIPLAQNRQRFNNQPPMPQQQQTRLAYSQQPQQPQQQQLPMQG

AYRQPYYNPAQQQYQQGPNSNPNPYQANYAPGYAGQQQTMRYNNNMNTATNYAMQNNQQF

PNASMGPANRFPPHQQQQPMAYRPGYKKKLPGNNMNGFTPASMQNGNIYNFR

>model.g423.t1 Augustusgene.g423.t1 JFAV02000103.1:2255-3454(-)

MKYKLCSKRLNQIDDGTAYKLLQLTPEMLSHLEDDTITTKSLTFKPMDGPQSELVLCTDS

CTFVMKQKMHSNCVLLMSRSDTPVKESAFTTDNGGNSISGNDDSSKEPLCTYEAFSQQCF

ELEPRKIEGKINVAQLPIYDGPDKPFLSTPRTLKISLLQFSESSPCSTKEFYDQWYAFNG

CVLADQACVLSEKFIDKQLHLLLLCIVGAELDVANGLTMEQCKVAMEATFQESRIERTDF

DQVLKTILMKFSENPETTGAKQDLQVFVLDLGKIAQWYGKKCLSKYCSNFQYIGIDDFMI

KWKSSFPPFFSCDIDLEMLLGFYCKDINNNKLCYLDDSTLPQEPKLRFNSLFSIQKSWLQ

QEIWPFVKDLNHQNLKPDTFLIKYARRKRVKRDKFIITSR

>model.g457.t1 Augustusgene.g457.t1 JFAV02000103.1:90011-93082(+)

MDHDTHLSNIALIRDGLVAVQDNLSNIIISIEYFQQFPDYLVKKLSSLRVFESEIWKIEQ

DGHLGKFTPDNTKQFYQPNQNLIIAAQLFHKLSVVIKYKKLIQYLPNDLELIPVLVEMIS

HYRDTPAGSSGLEQQEKWLLLNYLSHYLLLNIMNPFNLSQEINETILQTVISLDKNFAIA

CNSFLVATLLHRNPSLIHKYYLHLSYKMINQFLKVSERDFEQGLQLPEAVSKDWKYLIYA

GNISSSAEHVDYLSLLKIVPRVAKYYFYSDQSYLKELLQWYITIFSGENELSGDILENTE

HRFKLAKSFVLFLDTFEMQTVEQIYDYMIAPNGSDDLNTLHLKLLCIAAMIQNQSPDQNT

RMDMNTLNVFPIISKYFDYQSLDYNNCLDAAYNKIRDAVNFITWAWIRSKNKVTSMQLVP

YTKAVFLKLLFSTLFDYDLNIRRSALATLHELLGRFAVLDNVATVELMTFLNVINLQHIY

SVVIPQIFDLFRKHGYETYNMQLLERMILVNIEKLKKINITKQAVSVLLILYEKQEWNKH

ITEISNKFVLDGKNTTEYLYYFTSIKLLQPVCDIETCYNSFKTVSVTPCSSETSFRYLTL

LKSLNFMLKSPTNKLLSEKDIDHVYNIFLYTNKIINKKPDYTEFHNELAISINIMADIKT

SKYNSKSSYEHWQKLFVKLLSSNNNMLFQLLPLMDQIYKNSDILTDYLKKCNHLTKATLI

NALGDCFPRLVSCNPDAVKVLLLSLDDYTLTQEGDVGRKVRESGLNLIKKNIIYFQAKKS

LITSKLLKLACEPIESLQKLSFSVLLQLYGEPTEEHISASFYERIIDFHLKYDSEHKSSF

FNGYVATAGAIHCPDFQITDSLNAFLDYFLRLQDKNKLQVLNDLVRIFPTGETIKKYHPK

ANCNEINSPQKDLIKHTLTIFKFWERFLQNDIVLNYKTFNYKGFFARIFNIHFFCKNFGI

TEAWISLSAWLCCNILKQTDDSDFMNIIKAKITKVAKASPRLEKKCTESLNLIELTSENY

GAIK

>model.g472.t1 Augustusgene.g472.t1 JFAV02000103.1:126498-128711(+)

MSVQQLKQAKQLALTSKNPEELLPQVLDTTFQLVDVPISGAPPNFELVNFAVDLLQDTLQ

HSSISKNEHQFIALKHIGKLSQVFQCGFHYYQELELSMINIVSIMALVYEQVFNLVATTS

NQELWDLMQLLSLQFKKLWDILTGKGLKYISELKDDSFLTFNQITMKYRHTRMKVMLMKF

FSKIIIIHTMGDSGGTNSNDDNDPNSSGSTNNNSGNLVSSTSSKNTVGQVSLSNVSDSHP

VIKNKSKIDSDAKKLLDMLLNYLVDEEIMMCTQVFQCLLTNLVFVMQKRPQSSSRIFQAV

LRFNLDRKFQFAKEDTLQYRLSKRFMEREYKNFVNFCYKTQLLNKNMNSLMSKLTKIAST

LQMIGDDTKQKGILEYDENSVKNIIPLEILQKFENPKPVVNLVRLRNSALKRPFAEMADT

ADEEDANKGKYTKDLYSTHKKSFDLTKISKDNLIKLSINALMDGYKTQPLVTKLLETVLK

YTQVSNEVPGVKLQTSDSSISPTPIDAQDLQQELPADQLNDTSLLSLVSDISMNKEEGLQ

HFEFLVNNILSSEGSLDGLNSKKGEEQNDQSLIYKKAAPAVNILIRLASKGIPRENIEYS

NIIREKLYEYVIQDFSNRMDYVIAWMNEEWYFESDPLKPVYNEWCNKVFNGIVPFVDNSH

RRQFIRLVSELPSMNEQHINNFKSLCLDPLRNQLGFVTLKFMIMFRPPVVSLIKSFLTNL

KSEHADLQDQCNNLLSKI

>model.g462.t1 Augustusgene.g462.t1 JFAV02000103.1:102387-103370(+)

MGLFWAESTIKDQTSTNKTSTSNSSWFWSSSVTGNSTQKQSSCPHKPVDTAGGKSEQVLG

SANSQAVSACPVMHKNSSSTQPAADISACPVMHKTTHVGDTVDSNHINPLNNMPSHLPDQ

KQAWQKLDLPTERTLSSIPKGDGSPWEYPSPQQMYNAMIRKGKIDPNTGEEIPEDAVESM

VFVHNFLNEGCWQEVLDWEEKYASKEVPTKLLRFMGRPDDLSPRARLFHYLSYVFPSKFN

RTLPFDRHDWYVLRPDPSSQLNGNHPGYKEVRYVIDFYEGPDDEEGMPTFNLDVRPALDN

FENGKDRAIRWAKPIMEQIFDTDKKNKD

>model.g480.t1 Augustusgene.g480.t1 JFAV02000103.1:143111-143971(-)

MTSIGTGYDLSNSVFSPDGRNFQVEYAVKAVENGATSVGLKCKDGVVFGVEKLITSKLLV

PGKNRKIQTIDRHIGCVYSGLLPDGRHLVNRGREESANFKKMYGRPIPLDAMADRLGQYV

QAYTLYNSVRPFGITAIFGGVSKEDGKPELFMLEPSGTCWGYKGAATGKGRQTAKAELEK

ILTVEAEQDDEVLLSAKDAVKQVAKIVYMAHEDNKDKDFELELSWCSLSETDGVHKFVPK

ELFDEAVEFAKKEIEGEDDSDEDSDDEPAAGSGSNAQDAEGDIELEN

>model.g492.t1 Augustusgene.g492.t1 JFAV02000103.1:172072-172650(-)

MAKKISKHSRAARKAEAEVHEIKELADLPRAEKTDIAGSLIRTASKNEDLLNRKMERKQR

GKNRIGKNSGKNELGLKDKLKNVDLGFLEKEKLQKSLNFTNVLDGKISKSISRAKYVQGA

RKAGWDATNSRIRAELQLAQGSVNPASATTADKETTEEMDTEDVVTFGDLEEQEKQKKLQ

TNLFGSLEDDVEA

>model.g433.t1 Augustusgene.g433.t1 JFAV02000103.1:32755-36159(-)

MASLPTNSETLSKYLRSKQIKVPELISIVDKSYNGIYFPRGDLFVFELLVDRFNEQSNNT

FLGNAEIWGLFNNLWDKVLCKYEATFLIRVYKKLRMFQILTNLQPMLSGNSALESQVGSF

LDKVKESSLSVDLPQPDMAFKLVCGFAQQTPVSADTIDKLLLVVDFDSKADQSRFLQYLC

FKAPRSLLDLLALDDTKITSILYRFVLDTGKVDGPQTLTKLLEKTIKTATPKDSATLVKL

FEMVVKSNSSFTESVFQQISKISQNTVPELLDSLAHITKKNLVSEEILNEVLTATLSSDN

IQWALVTDVLKLNIELGIKNSTLILENVEKASTTLTSVWEQFVQCYVNAREFHVFVTDVL

QTFSKKHSNSKLIFDSQYASIISKCVSNLSSTQLLSTFNKLLDAPQLLLLIIRGSFNPVS

LQYIKDSSDLKTVLHNIIFSDTFTQSAESTNWELIFNVMELYYSCFNSQEDTDDIKDHYE

SLVETQQQQTARQQCRYSFFTLFKLRELIDLPDKLIFQRFMDWFSTVSTDKEAILFDLFT

RWSSLINFVFDKTNLNFLCDELVTRYPNVLHRVLTASTADSNDFFEEEEVIYELVSIISN

SIIEKREFSSVDFVCSIPVQCWNKNIRVALINFLAENLQHLSNNNAIIYLLKDSPTFKST

IETNFSELTKSLEFGSDIFECVWANHLTRWKESISYLNESLSILTKNFENDDCALKASLV

VILSSHSVNDGMFIESLDKLLHAFVAYCYQTIQSKETSIVYLYKILSSCEFLNKEEFIQD

KIKPLFDSDTLGTFSVEIEKLDLANEARVCDIFSLYLTTLSTESEILTGIAHYLVLKSNG

FNPQGFSAFIKNSTKSDAELFQGIFEKTVQIIGTCGNYKLPILEVYQSLLEHIPSKNEKV

VMPLARSVGQLISWSDDQPSVAPLVPVLETIKNLLITQQWVFSQYTIETLFPLCCKTVSQ

CNPNNETELGNIIEVTTQLLSIVLNFHSFRLTNRYHLINSIICFYMDVLSNSSHVSVNAV

QNVSRLIINYCEPSTSSTVSKNSDAGLHSKVSLLKKNLRRSVGPVLLKYIKLCVSNFNPL

SNSVRKELIGSIYSVFALLSQDELVTINASLDHAGKTYFKKIYEDFKTNGKWRED

>model.g458.t1 Augustusgene.g458.t1 JFAV02000103.1:93108-94977(-)

MFQYYFKRNIVTQDKTSPVRKIILSCLPDVRQTMLPNQKTTLMPYQSDDICDNLFRSSIK

AGAKSEQLPVIKAPLFLVNLRSFIGNSANSRQNLTFSTNIVALLHRLENFPYRWAGSFRA

SLGRSNKYFNLKFKESSQSSICAVVPVEILDQITFDISKKWRDLLRSSKTDENTESVAQL

KRAVQILFSLVEKNYKGKDLPLSFWKKLCNLACSVDEVDLILKSINGSNLQAVKQDYIEM

AIYRHKKDWLKFIAKFHQLEKSYSDEIVYNELFGSLYFDSLERLMKAGIHEDDCIEVYNR

CLKRLGLHGQFKLLHVSETHGLTKLNAHIQASLSSLAGHLKDYSDFIQDYLLELENMNVD

LDFSKNPHLLHELEFLTVKFPDFEEAKSYCSLTDQVFTKVSNQNLRFLYAHFLLKNVSFT

SSFHDFSFIMKDITERLFRTESHSPELSDLNKLSGFVNSENDVFYSIFENLSRTRDCQLT

TYKIYQYLKKNMTRDFTAKDYYCLIKANLQGDSFLAAYYYIGELILNLGASFFDKNNNWS

LPTKIGKDLIEKGLIDFFGDPEIESVLYHVGKYFEKVQRPISREELLRIIGT

>model.g487.t1 Augustusgene.g487.t1 JFAV02000103.1:159119-159538(-)

MSDVEEVQEVQEVVVESQQITIEDALKVVLRTALVHDGLARGLKESAKALTRGEAQLVVL

VDTVTEDNIKNLIEALANNSENKVPVIKVSDAKQLGEWAGLGKIDREGNARKVVGASVVV

VKHWGADSDARQVIINSFSQ

>model.g485.t1 Augustusgene.g485.t1 JFAV02000103.1:156529-157146(+)

MKDFGHPGKADVTSLDEDLYIKRNAKFTQENIQEIQNWIFNDILHQTPESNDADLIDLLK

DGCVLCELANVLSTGSACETPLIKYKKSAMPFVQMEQISLFLQFAQRYGVPQDELFATVD

LYERKDPAQVYQTLKSLSRYANTRNPERFPVLGPQLAQKRQAPLKPKKPSYLTNASWSTM

EFGYMKGANQKTEGISIGTRRNITRD

>model.g428.t1 Augustusgene.g428.t1 JFAV02000103.1:21169-22092(+)

MKTLQRKSQRKTIKNVPKKLKQSTIATKTNNMKSSLLTSNKLSPCTKCDVSITRCEFCLM

KYDKLDPVATHQHKEFHDVQTKGLKWAVPNYLKNSKFVSKPLMSPFQKSQGSRDIMSMLS

ASQDQLKTPDNSQRTAKCSSQDYKDCGDCIVEIQKTNMQEIKLALQLLDFVNEELTAPKD

ENDFWISGKGKCLVFIKDGRAVGVITLEDLVSSHYNMKWMILSSKQIVEHVNPRFLAGIS

RIWVSKHYRRKGIALKLLEAAQKNFHNGVELKPLQIAWSQPSESGSKTAKKFNSILHKSG

ELLIPVYF

>model.g486.t1 Augustusgene.g486.t1 JFAV02000103.1:157658-158854(+)

MSLTSDILFTATTVHLEHITTALNCLVPFGAKEDVLIIIDEEGLSFASEKSHIIKIQLFL

SKDLFTLYHFDSSRAPFEKVCVKLNHILDSVNVANNANNSGNGGNTISNNNDETDFLECT

LSYDGDGTPFVFIFEDALITERMEYSTYLIKEMDVFDILQIDLDRIEYECIIQGDILFNT

LQDLKELDCKECYLYVSAKKNCKPHFSFIFKNEQLGLSRISLPSERTCLEKIEFMNSVDS

QNNQSALQEESISYFDFQMLDKISKSVKIASKVLFRKDTRGILSVNILSKTNNIIPVGNF

RKSSTTLQNKKKTNVLPKNYSGIVIDITMIEKESDSDFQDIIEMMNRDESTPSPKKKASR

KRARVDDLSLDDEDDQTANATSVNLGPTQNKKPGVPLFF

>model.g426.t1 Augustusgene.g426.t1 JFAV02000103.1:11763-13250(-)

MPEFTTNSKQKMQSSTFFKHSWKQTEAEKKKTTYNTSKAQNSSFFESSQIQKKQFNLGSP

SLQSEYKANYTLTNNESPVFQYKTPSLKSTEKNGAEYVKMSPALAESDSGSATSSEDGVI

LCPIMASSSSSKRSTARTLTQKQTQGSVEHQKFGNKKAVVHEKTAESAVPLFIGDLDQII

DEKYLRTHFQRFKSIESIKICKNPKRPTELNYAYINFKDPAEAAAALKHFNYKPIMGKDV

RMMYSFRDKTLRHKIGTNVFVSGLPLTDEKLTTRAFYEKFSSFGNILSCKLDKRKSIGFV

YFEKKTAALAAIDFYNNNVFFGKPIKCGIHFEKSVRDQSGFDKQLQLTSGNRIFKEDIDT

ARLNSCIPAITKSELNPMSVESFVQSSSSVSVSNVSKFVQVDEILDHFCKTGPIKSVYIF

KKNCKNNAAVAYITYKKTADAFQCIKLFDGSPFQGLKIQVKLSLPKNSYQSLKEKFQSMS

LLVDESSKANVDSNGD

>model.g434.t1 Augustusgene.g434.t1 JFAV02000103.1:36504-38987(+)

MITSASNEDQLNKSDTVAKKSSPFQQLLMDPNALSFLDNNEDSNEKQTEHLQDEEMVENT

SQEIPDEVVSDSQSVDMDDDVEAEKETLDGTEKADADNIDIELESEEKQNGLKQNVKEDV

EQKEEIARDTEELDGSSKNIPSFFFKFNPCFNAGIFGSDDGRQLTEITDLDCEDEDELNE

LLKTHPTEPAFNTADVDYISKVFEVYYGLYKSGDKNFLSSNEVLPYGVVNKQEISEKLSL

IDLSFEQVSNELDFYLTKKLNEPENDAKFTFRLQELNNVLNCLKTFQFIQFEEYLGTEFF

TRLHEFINKLDGQPSTGVIYEFLEGEPTKLAETEIFYKLVIQLLLRGLIEQAVNLLKTWQ

EKTKSQYISNVDAFNSVVQLLENYPYDNQSHFREWKNYVAELANYLENNLDEPDTKWFRK

MVQCMNGNQNVISSVSTKWYETLSGLFHFYIPTIKLLPEYLAISLEKYTDNQLTILEENL

IYILENRIVNILPYLESLHQPSSAFIAALCEHRGLLPFLPSLGNDKSERTVSSYMLYQLG

LYLATPSAKINDDLLKRLYSLAFGVLRVSNYEPYLRDTVSELLLRYPVVTNEDMEYCLTI

AAKWKLPATISVIYQRLGMELIHTSWLESMIFFSKSMEYSDSSIVENVGESKLIYYSLLL

LEHSLINGKPVNNALINQVLEDDEQYNALPKALRQSLAPYRVLYRIFQLTEDLKANPQSI

SLSYEILRLSNELLRFQYLKNYQKVLLTVKIVHPLLKEVSSTCAKFDSQLLIDVMESVNK

LEKSIFTQHSKDAAVSDKLLGEQLESGCVEFLKALNKQLNQLFCTSVI

>model.g483.t1 Augustusgene.g483.t1 JFAV02000103.1:151897-152685(+)

MASNPPAACCFEGFLHEGTPKGVHKELFGLNTYISTPTDPSKCKGCECGGSEPKQCEKVI

VILTDVFGNTLTNCLLIADDLASQGFKVLIPDILLGETVDINHVGEVDFQGWMSRHGNSK

TKPVVDAFMQKVKAEMKPKFLGAIGYCFGAKYAVQQLDHSAENKGVADCVAIAHPSFVTM

EELAAIKKPLLISAAESDDIFPAELRHQSEAKLKEIGATYQLDLFCGVSHGYAARGDVSN

PKVKYAKEKTLLDQIYWFKHFSA

>model.g476.t1 Augustusgene.g476.t1 JFAV02000103.1:137175-137585(-)

MLKAPLRSQRLLYTQVARMSSQTTLDPNRCTDGPICQQIIAKLQKGDPSSRISSANISKL

EIYNDSYKHVAHAAQKNLPNKIESHIRLVIVSDGFKRMPVVKRHKLIYSLLAEELARDVH

ALQLTLKTEEEYKKLSA

>model.g488.t1 Augustusgene.g488.t1 JFAV02000103.1:160054-161598(-)

MTTQQSGNFTRPTAKLQTAMADHMDEVDVVIVGTNIAHSVLASALVWQGTSVLHVDCNEY

YGDSTPTFSISQLREWNDKANGSLYSNLNFEMQEELFKQYPNYEHKHFSIDLHPKVLFAE

SEMLALLIKSRVHQYTEFLPLSNYYTFCRKTESFSKLKTTKNEIFIDESLPLLTKRNLMK

FLKIVVNWEEEGGKWERYRDAPLIDFLREKFKLSGTQISEIVYTLGLAFEKSISTQMAIE

RIRKCLTSYEVYGPFPTLFSKYGGVGELAQGFCRSAAVGGCTYKLKTRITEYNASKKEIT

FQDGTKTKFTKKIVHSPTQESCLLNGGDHSTDQLFHRLIAVIKKDFSQQWFKAGESAAVL

VFPPGVLEEANKKNENSVQVVLYGSSSELCARGTTLVYISSIDSQDCLVAGLAQFLQSSS

ALEEDVILKQTYSQKTSIPFSIPDGFDFETAIKLGDKSDVILTPLPSPDASYEDGSLGVA

KGVYKKLLGQFDDFFAIDLEEEAAEDEESRKYSNI

>model.g456.t1 Augustusgene.g456.t1 JFAV02000103.1:87255-89066(-)

MSKTAPNSSGNTKEPKKIHLVILVHGLWGNVSHMEYLCKKLNNLNNSNSSANEQLYVHIS

NSNEHYKTYDGIDVCGTRLYREVESLIKELSKTGSSDNNNTQGQTTVVKFSMIGYSLGGL

IARYAVGLLHNNKVFDHNQPQMELVNFTTFCTPHVGVFAPGLNTGVKIFNNIVPKCLGNS

GKQIFLKDSDKLLYLMALPNSIFMNGLKKFANLSLYSNCINDIRTSYWTSGISLNDPFFD

LIDNENPSIVEKFQYVGNYAPVVLDNKKPFNIVSFKNETENHQQQQSGSIATLDYKNSST

KVNEKCVKKKKHHRASPTSAQNVDVMTEFYFFDYWCSKLYKWFKVLLNFFVLLVMTPFWI

LNSTTKAILENCKSYIRVSKHYRSAALLDYFEHSSSSSSSSSLSSSLSSSSASLPSSLSS

SFESSSSLSLASGRSSYRSQQDLQNDTNQDKQSVEDSLEENFENGLDTFVDSVYQAIERV

EIRNAGIGDSIAELNNAKSLQVAITIQELENHSIEQLTAKYCKTSHSSAENVEKLLENFQ

IHINNKEQLAIIENLNNSLKWNKFPILIRNTKATHAAAIVRHNDPFFNEGKVVIKHYINE

VFKV

>model.g461.t1 Augustusgene.g461.t1 JFAV02000103.1:98464-101742(-)

MANNIAENTAPQQVLSGGGTDHNPTLNPTLNDQKLVEKEYSDQKVIKYISNELKTDSVVR

LSHINLKTLPMEAVTLMVTNNTDKLYMQKNKLESLPDNFGKLNSLRLLDLKDNKIAAFPS

AILPPSVEILDISHNLISDLDVSLAETLPNLKYLNLKHNKFTSINKLAPLLKLPRLRILE

LDEYDDPETSIKDLTALKEYGMDTDPLQALRNYFEKKSLEKKTKNIRNTVNDRNLSSDQQ

ENDAYIYSHSKYNDYFKRLSVLPEELNEDSVEKTPKSIHLNTFRSVDHNESEYSDRGTRL

FFHSGKNDATALTTSQSLKHENKSQLAEDSKQNSDAPSSFNKWNHIRNSRLKTQVHRGHD

LYRTPPPKSHAPQNFQQQHNAPASVLESADETMDTKTNLPQISKISNTTSDRHETGQKLA

IFSNTETGLHLSDASSLPKKVQQDHMGTGIQEETSKQNTDNPLKIANDHSPSAAHHKSVI

TKKEKISKATYLQIKAIRYNRLLICCRKLLFVFTECQQTIRRITSFGKDKTVAMNVVSLL

YGVTSHIDNLVEVLEKVESSEKQVDSLILIQSCTAIIPSFKQIFKLLSTNFDSFFQGNEI

CFLRMFFVTITSSYNELCNAWSLIKQESAEAMALKKGNLVHQRAHGQRKTSMFSPKGAKS

QPTSSTMLSQLSQSFQSTPNLQAQLLKRKPSQNAKLPKLTRTTSLNEAPVQGGPSVLPSG

RHRSSTMLVTKKNDENSQTKKSLLHKSEADSQTAVANGQDLGNEPNFIRKDIHLSRNTLT

PEVGLSGHSSNVDALNSDIHGKSKEQTPKQLATSSTSDHAPSAATDPIAVVPPARSVRRN

KSLSRSVIPNDSSVSQLSKNPKAFDLPSNDSSKSLHDTDQALYKTLQTVVSTASYVNAEL

NSTVSKAAIAHAQNVSSNDESSKSSELLAPKIKLLMGTCVSCMDLSTHMKSRLAILMQNE

ASLISPIEKRKTWELVNTFLKSIIALLGNAKSVINEVDGFGDLRPGLGNLAKVTKEVTLI

LNNSSYKNAGSIPTSQIITKDTKGTSSYGPPPAQQNSQTTASSGVYTSVFPDLKTTPSVA

SASAPAMNYFDQH

>model.g441.t1 Augustusgene.g441.t1 JFAV02000103.1:53590-54060(+)

MADEDHTFETADAGTSATYPLQCSALRKNGFVVIKGRPCKIIDMSTSKTGKHGHAKVHLV

ATDIFTGKKLEDLSPSTHNMEVPVVKREEYQLLDIDDGFLSLMTMDGETKDDVRAPEGEL

GDNLQAAFDEARDINVTIISAMGEEAAISFKDAPKQD

>model.g471.t1 Augustusgene.g471.t1 JFAV02000103.1:124778-125758(+)

MSGNEEREAHLATNNTKLPTREQVQKHSTPSKPTFMNENSKKNKEHATLANVTESRQPEE

EHFDAEMDNGVIVIDTYQPEKKDIADENKTKETKEYSEHPEAHGSPKVEPLAHDQMSEET

TAEATSHPQTETAEHEYPGEQDGDGEMSPSTYNEYYEYNDENEVIVKDEEDVFKNVALGL

EGKYRDLLIDFWQTLINQIESLNENDLEEAQTNSETDSNAPPSSSSSSSSSSVVVGIQDS

FKTHNLPLARIKKVMKIDENVKMISAETPILFAQTCEIFITELTLRAWTIADENKRRTLQ

KQDVVEALQKSDMYDFLIDIVPRQLNK

>model.g464.t1 Augustusgene.g464.t1 JFAV02000103.1:107795-109177(+)

MPNHYAVMSMEIFSHEQACQEYGVEFLQHLLALDKKYDLQVKHTSANYGSPAAMVYSSQS

IENVMFLNYYSLRKLIFDFMMCCHTKLRLSTPTLILSFQILDKYVTLLRLEQEMLLDRLR

FQMIGVVSLWIASKFMDPKIKHPDINILLQLISYNSGKCSVATTADKAQEKMKLETAGKL

KNLELDILKHLNWCVSDIPTHDFFVDILLRSIDTFDDSQQQRFENETHSCFSMEDINQIK

YGAQMLCELACFYPHINNNHSVSSIAYSSVTLIQKCMQDFYSCGEKEKAGANISLDGISF

RLLEMFRDCHSALNEDGFFALPFSFKLKYFPKNACHNPLFLQSLLNFVSKQYCSESDTSS

LDSGVSEKQDTCASVGATPSAALLMPTKAMPPTPNTPSLSDMSKPVKNTDTETETGSRPP

TRGKRSLYELDPAVDEEQEHKLNPGKKKLHQELFIELNNQS

>model.g439.t1 Augustusgene.g439.t1 JFAV02000103.1:47901-49124(-)

MSSITVKFKDFKKNIHPVSLGADSSVEETKQKLCDTLGNNTTLDQIKLIYSGKVLVNSKS

LSTYNIKDGDQIIFMVSKKKQATPTPQPQEQAKEETKVTDPILSANEQPTTATTAAPDAA

PAPEPAPEAAAPASAAPASTAPASTAPPDEYITRLMELGYSRDESVMALQLANNDPNRAC

ELLLMGLTEEMVAGFNAPAAPQPHAETQQDGEDDHDMEQEAGADDDDLFAQAAAAAGGDS

TDAGAGAGGAGARGGNAPRSISLTMEDLMQLRRIVNGDPESLQPFLENMISRYPQLEDQI

QSSPEMFLAMLLEGLGGNIPGDLGDEQMGEQEGDAGVLGESGDAAGANLPGQAPSNTIQL

TQEENDAINRLCELGNFDKNLVIQIYFACDKNEELAANLLFTEHSNFD

>model.g449.t1 Augustusgene.g449.t1 JFAV02000103.1:73886-74437(+)

MDKKLDFIRECIEHNNKNDATRDKLKSHKWLNHTNKSSLERKNNSSSSSKRGRKPSNFTK

RIVSKSARQVLSDEWKRINNLNNLDGQVNGQVNGTANEENKEKSSDAAAQKQRNEVTYFN

VEAPPSLLPVKRYCDITGLKGVYRAPSNNLYYHNAEVYTNVIKPMLPGNDQQYLKLRKAD

VVLK

>model.g479.t1 Augustusgene.g479.t1 JFAV02000103.1:141095-142990(+)

MSRFGTLERDHRNFRDTELFDNSEDDNSSTNAPVHPPESFELRPDANSTNIMESYETELN

GGLNALGEHISRGLLEEDRASSQSSPVENAHSQQNLETDQSSSKGKSASEPSLDKISPKF

RELGIWEPSQSAGSSKIGVEQKEKKRSILNQKSATISNSGVFTNHDARTVSASNSNSIEH

LQQELARSQMQIKILTDELKQAQPFSKDSSSSQPTTTSFKPLINNNNVEIENNNWKHEQE

LSELHEKHEKEINELNKKLELSCAMQEEMHIEYEEMFKFYTDIIKEYETNMSQLFANLNK

FIENNEEFLTMKKNYKALKMTKNCDENFEQLNKFFYENLTTIREYKLEQQRVREEALHEV

SELTDDEEENKEKRDTKGNTHEHAQKEVSFVDGDGISNEEASIYQSSKEPGEETRYEIEI

ENIHTEYHNFFSHIFELLNQSDQYGRELEHKIFQQTTLLKRFDSLLNDELEYTSSTVKEH

LEQENLGLLTDLSEVNQLARHLDSRLAHHLKNTSNFQIDVNEICGDILVQFGKVFEPDSI

FLAKEKLYKHKLEIANDKSRQNSVRLLRVLQFIRAGTCSLIDEYISQLKLKNKHNAFIEI

KELNKKWLLEKYKREKDLRDFQQRLSSLETSE

>model.g556.t1 Augustusgene.g556.t1 JFAV02000104.1:153236-157099(+)

MAMKKLKIAFNKDIVSTIEHHLSNKEIIDRLGDLHEELSHIDQENTKLESLDKYRAQLID

KKLLKNKDGGIQAFTACCLSDILRIYAPDAPYTESELTDIFKLFFTQFKMLTKPDNGYYI

QQVYLLTKLVEVRSIVLITDLPQAEKLVHELFKVFYNPENNDFPTKLVSIISDLLDEVIS

ETDAVSVDVLKLIFNKFLTYNPHAVPKGLQIAQDSSFQFSLHICENGSGRLGRYLTKFYT

EMLYDVSRPSSSSSSRKNIQEDENEDTNGDRTFIDEITSVSHPGYKVLTKLHTLVLNLWE

YVPELVSAVSGFLNQELYSTNELFRISTTKLVGDILRLKNPSLNFVTTCPEVFKAWLAKI

ADSSPKVRISWVKTLPGVLNSGSSYVDEQLCSNICQGLSKTLIDTDFKVRYISIVSIKEI

NTETIWTYIKSPVIFNELLHLTREKNKDIRETAIKFTCEFYRVSKSKQYHLLIPNEKLSE

VFSKIPSTILSLYYINDKSINALVDTLFVESILPFEEEDDEKRVETVLELVSTLDEKAKQ

SFFAFNKRQVELHSVVYKFMNMAEELNSIDNTESAEYKNLKFSIDQIIKWLAVGLSDSFN

ITTILETLAHLNNKRLFELLKLIVSSETSYTQLLKHFRELREMLKDNKLFASVKLNSMFT

KDEFSAIVQLLSYRSAFIIYNKSNISIFLEKTTSSGNKNEETRLACTEIVEHISLNIPSA

FKHEVSSLMAIIKTCDIGDKSALETLKTLYRIGKVKLEYIDQNDSFFIDRLMDFMIKGTP

FEAQYSCKLLLLMDKSEKHIKSLLDLLLPINDTVTPSQIMILSEVFAFQEYELLSDRSTD

IVTYVLANILLSNSPIISAVENDENEWIDDQKLCLPGYAEIAKKSFGLKLITNHLISLSK

NLKENEEEKEILITKSLKLFFFLIESGGELIVRNEDEEEGDDQIITPVQYQKKLRLLAGL

QILKLAEHPVLNNFITSLNVEKLVNLVEDESLIVRTSFLNALKKCITDERISIKFLPLMF

FVAFEPDKELKKQVKLWINFTSSKKSFQKTTFFERGFSRFIHYIAHHPDIYDQLSCSSSE

DTNKQVLSNAVEYVLYYFENILKKENITLLFYLSNRVKQYQDIDETVPESSNIYIISEII

QLILVEYKEIKNWDIPVYQGRLNLPTDLYVPITDPKKQNSIIMTNFLKDEQVAEIKKMLK

SKSIGSRIGGAMKKKATNEFKKNQKKSTVPTKRRKQTSKNDDSDDEDSYTVSKIKADYTK

VRQSSRVKQNVNYEENDSTDGEDEDEYE

>model.g572.t1 Augustusgene.g572.t1 JFAV02000104.1:188851-189684(-)

MVDFPPDPVLKFLPAIFPAENKVKLNDGVGVTDKIHKLTREVKTGSTLSNDELDQHLTLL

KGNLGGYYDRHGSYMYEYDEDQPKKPSSNAVHGKSKWESHKLEEMKLNELIYVSYYHCTE

DSRNLAYYVSFLITDEDMRDTFNTGPVHKLQSRRLRNKHLEVYKKILYVYELQLDENYRR

LGIGFEMLNTLLPQVLDSYIRNERLEKNAGTYGKDGGGAELDKAVVEYIRAIQLCCFEDN

RLALGFYREKCNFINLFSSKRLQNGHFYIFHKDICDEL

>model.g533.t1 Augustusgene.g533.t1 JFAV02000104.1:99276-100511(+)

MLIPKEERTKIHSHLFQEIDTKNLYVIKAMQSLTSKGYVKTQFSWQYYYYTLTEDGVDYL

REYLNVPEHIVPGTYLHDSSSSNERQSRRY

>model.g496.t1 Augustusgene.g496.t1 JFAV02000104.1:5274-5846(+)

MNQLRSAKSLSEQELELGILDVQDTWHEEYKDNAYVYFGQFNKNLTEADILTIFSQFGNP

VDLRLFHDSQTGCSSGFGYLKYEDQRSTILAVDNLDGVHLLGNFIKVDHAFYRPTKKMQL

EKYRSCIEAELTKDFVSTKNTKREQNPILERTQAPKRLEQSVEEADVNDPMASLLGSDLL

LPYEESTEKDE

>model.g535.t1 Augustusgene.g535.t1 JFAV02000104.1:102694-104631(+)

MTREKPSGIEGGTSRLLIKGSTTQKSEKLGSKNLAKLKEELMNDANKCLTETVEGLESVT

FEEETATVQSSVTEHIHNQSRDAEKTVALSNQKKQGGLLKKLFDSNEPNHLARPKASRNL

TMEKLKNKLESCAESETNDSQVLANTVDKQKATAMITSKNAFKAEKKLPGEAHSKEPGKS

RTNGDFDKASNYKLVDEIDNRDTEKLREQIACGIETLPSQRVKLNRKKLITYNIMCVGQS

GLGKSTFINTLFQSQLMTTTEQNHEKESTSKSAEIEFKTTRICHKTHYLEQDDGMCIRLT

CIDTPGFGDYANNQYAWSGITSYINDQYTQYFLQEIQPERKEKTDTRVHCCLYFIPPNNK

GLSSLDVEAMREIAQRVTLIPVIAKSDGLLAEETGIFLKNIRETMQKQGIQVCKYLREDL

RQDLSMANMEELIPFRVIGSTTKVLNSEKQMVYGRSYKWGTCEVENPEHCDFIKLRNLLI

SDHLMCFFGLTEEYYATWRANIIFTQSRQSQRLLFSKDLWKKLSHWNPVYNHQERALKEK

YDNMTTCEFQLLKNKKTILFNRQDALNEDINDLRGKIVCLENECADIEALIEITKTEKIA

AEVDRQSESGSGNDIKIDASNDFTKLSRTNSFGAVLGSLFHKNPQS

>model.g500.t1 Augustusgene.g500.t1 JFAV02000104.1:12747-14036(+)

MSEKLFRLDAGFQQYGWGKIGSSSAVAQFAHHSDPSVNIDESKPYAELWMGTHSSVPSYN

HDTKQPLSKIITEHPEEYLGSSIINKFESKTQLPFLFKVLSIEKVLSIQAHPDKALARVL

RANDPKNYPDDNHKPEMAIAVTDFEGFCGFKPLEDIADELNKIPELRAVVGEDLAAEFIA

GIKPCAAENSAEYETNRKLLQKVFGKVMNTNDEFIVEQSKLLIKRAQEQPECFTEKDLPA

LLLNLNKQFPNDIGLFCGGLLLNHCQLKAGEAIFLQAKDPHAYISGDIIECMAASDNVVR

AGFTPKFKDVKNLVEMLTYQYGSVESQKMIPANFDRSSGQGKSLLYNPPIDEFAVVETTF

KGAKGGVRSFKGLQGPSIVITTKGNGYLTSGDVKLRAEPGYVFFIAPGTEVDFVSEDEDF

TTYRAFVEAN

>model.g498.t1 Augustusgene.g498.t1 JFAV02000104.1:7082-10171(-)

MYNNSAHQFGQLNGFGNTPQMNSPGMYNQPQSTGFVSSQGTDYFSTTQPPQQQQHQTGGF

MQAQNTGYFQQQSQAPSLPLRTSTTTEFSDFKQNKPESYGTGPTTFGQPMQQSLTGYSSQ

QPQLSQQQQQQQPGFNSAMPTTSFGNFATGQQQNTPAPLQPQQTGFYSSQLQPQQTGYYS

SNQQVEPLQPNATGFVNSMANAGTFNDTLKLPNRRLSFVTLVDQQKFEKLFRSKVPLGSN

TISGKNCRDILIKSGLPPKQLAQIWNLADSNKAGELLFPEFVLAMYLVNSVLHGEPLPYA

VERKIGNEVTAFVDAINFMVGEEANERSAPPPPSNPTPFDDLTVGLQNLQPQPTGMMPSI

TFGGGSSSQLPQQYTGGMLPQQLGVMPQTTGGALQPQNTGFMPQTSFGQEAQMQLTGAPQ

TSFGFAGQQQTGLLQPQMTGFGLVAQPTGVLPPSSFVPTAPLVAQKTGFGNNELYSQSNF

ASKFSAENDDFITAEEKALFYKIFETYDLENSGLLDSPTAVEIFRKSGLNRSDLEHIWNL

ADANNSGKLNRQEFAVGMHLVYRKLNGYVLPTVLPRSLVPKSTQIIDLMKDQLKESNGLG

NQKDKKSSPINSSGSSYRNQDELPQFKSRRSENVLPTQKQGRDEAMKNQEEQEIKELKKL

IRDKKILIDGEIARNEQRASRNDEEKIQDLTEIENLKNQIASLPSPEGGSAFSAERKARY

DSLKGKVSQVLAEITSTEEAITNAKIHLYRKKNPSSIVGTGPNGEVTDFDRKKAKRKAIL

AERMASLTGKPVAKAENLEEENQRLEQAVQQIKQESLKNAAVVNDIKTTIDEIVSGVSDL

YQNNADTPESAKYDLGLGLRPQVKTFIKQLQATKLKTSYFKNNLSQPSSPAVASVSPSLQ

ASSPLGSETQKEASYSEFKTPAERAAYIKEQAQKRMNERLAKLGLNRRVGRRDKIPDESN

TPPSPAVVSGPEKASSNIFEETGVQENVVAGVSSAASFESSSNVPPSLNREDHVSEEEEE

EEDEEEEEEE

>model.g545.t1 Augustusgene.g545.t1 JFAV02000104.1:125679-127148(+)

MTTKTPATTLPLPIKVWTDHILPHLPLDTILNVTTLLNKETSSVFDMYEDDLWLNYLKKQ

HIYLVGLRRACEYYYPSVKGISWSTYAKGCFSREYPQENYAMMVGNCHAILCGSRYDLIP

QALTRLKQLRDELKSVDFQISYYETWLLKTNDVFNNFAVFLDMFDPAFSALYTDSMTILH

EVTQDLKTKIENNSNPSWSPLTNCVNEVSLFLRSMKQTIPLLPPLDSLIGVLRLICLPGS

QKNNGDILFIIFRELMQRLGYAIDSNQVGFKSNGEFLTYWFRFNSTKHSFYSLDFYEDGD

VVWNEQESEQGWKFEDFELFIKEHQSSNQSSTLLLRYSWGHNFMDEMTVNSHYDYIINNN

RDLIFFLRCRYGMNMLPGMFYDTTLLKSINWNSDICPAHGCFAWSSKNMVVILGFNTVEQ

KYYCLNMVSKLFLESPINLVIVDKLCDPFKDSVEQAAEPIEVLHGIFKYNTQIGQFFKSY

ENNRFTNFLL

>model.g518.t1 Augustusgene.g518.t1 JFAV02000104.1:57834-58661(-)

MSVIGTFGNGKNNEYDDLTGDLQDLVVGDVNKELFQEYINGKNNGKALHDYKDISDFLIS

SNLQYMMLDELVGEMGRLMEDCSNDTYSELEELVIPDYLELMHLSDTKNDENPMLLHELY

LDITTFSNKITHHQNTDLLQNKKLIQSTVKYLQKLDTLLMLNQCHIYIHQLVVFLQNAEI

GSLVVSDNNSTSLQLFYHYYQLIKHVTLLFDKVGSTCDSKFLHQLRSTFENDVLVDFNNQ

LENLVLQINTTSGNGKGSLKQLCSLVYTILKDQLDG

>model.g567.t1 Augustusgene.g567.t1 JFAV02000104.1:173902-174708(+)

MDRRFNPLQRAFLEQNESENGDNKQNSFLKPRVPVPVPTQQSRFRKPKITQTLTTPGGAE

THSSSPGVARQPGCSTLPVPVPAPAALQTSSSKISPGAAQKRNKVVLQPGHGPLDWADLQ

AKKGSTGETTMPALISIRDWSTFTRDNEVYEHNLIPVLKQLAETHSVTSPPSAQVLTRYP

PILPWEFKPALKIRLEEVQKHNTRNDMWCVLNGKVYFLSRYLDFHPGGQDILMKYCNGKI

ATVMFNKYHRWVNYEKLLQTFYLGDLLSA

>model.g534.t1 Augustusgene.g534.t1 JFAV02000104.1:101228-102454(+)

MGKKVNYAAGDNLPLYATPEQSLEHGKQWEEEKLEEERRQRLSQHLMMQRQQQQQRKEKL

NKMPESTSFPSHGSSTNGSSRHSSGVSGGQRRPVPLNALPNTFPQQNYQQQQQQPSMTRP

LPVIPVQQPHQQYSPQYVPQQPGQQTSGMQIPYGQQPSPMEQQHYVPKPLHQQQQQQQQQ

QKQHRVQQPGTPISGLSQASPSGASFNSAHARLGVVSASSNRSSSSSLNGDKPDVKEAAS

ALFHKYDVSKSGRLGANELQRLLQNDDTTPFSYSSVDSIINLFGVSRSGTLNLNEFIYMY

HKIKTWRDVYEQTDANRSHTLTSLEFSDAVQKMGYKIPIEVVENLFDQFAEYDNGIKCLK

FGKFVESTVWLIRLTKVFRKFDEKSNGVAVIDYKDFIDVSLYLGKFLPH

>model.g529.t1 Augustusgene.g529.t1 JFAV02000104.1:84600-85679(-)

MNFFLSKPINTISIAVLFVIASILYHAFATKCKQNPSVRTSQNLTANLEKLLLLRVVSTD

PDQATNYYKMYQLENLTSSDSFTYARTIDDFLEIIYLNSSDHKSLTTSSDKTTVDVSLRA

LLIFCFGAICQGALNIMFPYFTMQMVVLDTKIKALIFEVWFKLKKGVLQLRNKYANKASQ

KSFPKFESLFIKWSEETLAIQKELRSDINACLCLVSDLLAQPKCKEKNMKADYCAKNSSS

LVNEFAKALKSDDEEETSVTCENCQASSRWNSGNEPNKQLYHSNCFKLRKLSSCQTFQMV

KNDSARLSNDITTKPTFFIGTSSSSAHSTPKLFDSNGKPYRRIFVPGRGWISRSNYFEQY

>model.g497.t1 Augustusgene.g497.t1 JFAV02000104.1:6109-7005(-)

MAQLKREIEEAEEADEKEASGNSQGTSATSAPAVPSISTKALLPEVGQAADENDGWSDGE

MAVHASPPPSLIPVLSNASVAQHHNGNPFSKPKGDEIKTSTADSSKKPFFQQSPSVKAST

SSDFDTKAAELQRRMQRGLDNGNDDGWSDDEVKSDAQNSEHVSGHQPTSVASQSSNVTSD

IGVSNAAEAPVVEQVASTSLSEVPPPSQVSGSSNLELDAFPLAPSLTGQPARPQVSEAPV

PVAPPLPSMDQQPYLANSEQTPSDVDDDDLSIPESVSSDDDDADFAGSKIPAPPSLPHF

>model.g514.t1 Augustusgene.g514.t1 JFAV02000104.1:52195-53381(+)

MGNGKPRGLNSARKLRVHRRNNRWAEQNYKKRLLGTAFKSSPFGGSSHAKGIVLEKIGIE

SKQPNSAIRKCVRVQLIKNGKKVTAFVPNDGCLNYVDENDEVLLAGFGRKGKAKGDIPGV

RFKVVKVSGVSLLALWKEKKEKPRS

>model.g552.t1 Augustusgene.g552.t1 JFAV02000104.1:143054-144016(-)

MVATNNIKLLAPDVHRGLAELVAQRLGLRLTECKLKRDPTGEVSFSIGESVRDQDIYIIT

QIGSGIVNDRVLELLIMINACKTASARRITAVIPNFPYARQDRKDKSRAPITAKLMADML

TTAGCDHVITMDLHASQIQGFFDIPVDNLYAEPSVVRYIKEKVSIEESIIISPDAGGAKR

AAGLADRLDLGFALIHKERAKANEVSRMVLVGDVTDKVCIIVDDMADTCGTLAKAADVLL

DHGAKKVMAIVTHGVLSGNAIQNINNSRLDRVVCTNTVPFEEKMTLCPKLDVIDVSRVLA

ESIRRLHNGESISYLFKNYPL

>model.g569.t1 Augustusgene.g569.t1 JFAV02000104.1:178698-181073(-)

MESSGKAVTGTGSTANSNKSIYSYWMTKKLQMSTFLGDLQNGESFSNAMTRLQTMGALME

QVIQQNIDLYDDEDEELHNEQEQDPVTKHENQDGTHTGTTATTAGKDDHSRKKQKKLRSR

SNTVNSSLGMDEDEVVLYNDVVIPRDKQYQEILDSITSEMPTGVEDEQQALRMKIQKIRT

LKDISPKIKSLMIQKLMMGKTNLSDLDLSIKATGPSASQSNIKKSQSDVEEVVPTISKKD

LEQVPFSDPFGCEHYLRNCISQCPTCQEWVMCNFCHDENVHSHEMQRNKVDWIACLYCET

VQHPTSQYCTNASCSQMLASYFCDVCKLYDNDDEKDIYHCDKCGICRIGLGLGLDYFHCD

TCQACMPIELFDFKSDDEESEENDADHKHDGVNKRGKTLKNLEKNSAQDNVLNDVMKMIG

NTTSNSLLSIFEYAQQDRLKMAEKQHICIDGNTKSSCPICGEYMFTSLLPVMYMQPCSHA

IHKHCFDEYTKHSYKCPMCQVTIIDMEIQFKILDQEIQEQPLPEPYSEWKCVYKCNDCNA

RGICQYHILGLKCGDCYSFNTVQLKLIKTDEFIEEELQNLNDYRRQVHGGLHPGLGNSTT

RPVTSSSSTNHAISSGSKTSENSSTRGHHNSDSESRMIESILQRNHELLESHFSISDHRT

PSINKDLNDEDGNIDNFMNSYLKNDEDLKFSSLAQGFKLYLTGYKKMVSEQHSTSPSLTA

PPATHSLLSDNDEVHCQDNDAEPQHVANDTSNTVDIQNEETSFTSMLVDVKRAFTEFIKQ

QRQLGEDYEDWA

>model.g568.t1 Augustusgene.g568.t1 JFAV02000104.1:174973-176283(-)

MNQGIKTSPNSKDQQQQQQILQQHFQSQQQHPQLHQNNVNVLGSTNGNTNHNLMGSQLSG

NAAFGHLSPGGLPHGVDASGLTQQQLQQYQQLQQYQQLQQLQQQQQAQQQAQQQYQQQQQ

SQPHMQQHYQQSQQGGAGPGNAANNLGNMNITEAMFNSFLSQISQRSNPNMMGQQGSPNA

TAAMSTQASQFQQPNAPQQMNFPRQFHNTAAPFLDATAAFMQPQATNYAGKQETGTSRGS

RGSAAGAAGADGARASGSSKKNAAAQAEVKKLSASQKRVEKRKELVKQGPKRPSSAYFLY

YQEIREKLKADNPEMKVPDLSKMAAERWRGMTEEDKKPYQDRVAGLWSTYRINKAEYEAK

LPPKKPSGPFVQFHRDIKKELQSDNPGKNLLELTKLASERWKNTTPEVKKKYTDAYKEQM

QEWKEAYESLDPAEETE

>model.g577.t1 Augustusgene.g577.t1 JFAV02000104.1:199802-200551(+)

MSDFHKHQRKQQNSNRLSKLAYYCQICQKQCRDDNGYRQHLRSPHHLKMKSKITAQDIAQ

YNEMFEKSFLQYLRLSHGEKWVNANKVYNAFIINDKDHVHMNSTKWSSLTKFVQHMGKQG

KVRVQVEDQEHAKHEEADVYSRLEDNDAVNSNSLLIAYVDRSSETLLRKAQVEQLERDNL

TEQESRTLLLQKQMDNAKKLMQEEEAEEAQDASEETRPVEPLGKISLVTSNRVGKPRAKK

PARKKKNLFE

>model.g509.t1 Augustusgene.g509.t1 JFAV02000104.1:38753-39886(-)

MSVGESRSELIAWVNELLQLEFTKIEQMGTGAAYCQILDSIYGDVAMHRVKFGYPLAEYD

KLNNFKILQQTMAKYNINKKFMVEKLINCRMQDNLEFLQWIKKFWSKNFSNYRQDRDYDP

QSRRKAVSHSGNSSPAQSRKVSLAGSNGAVDTTASGTSSYSGAQAPRRVSNSNGLVRSGL

RNSGVPERRHTSANQIYSENVSLREELELKNQELQQKNQELKQENEALQQKHQEFELIKK

ELFQFQDGFDQVVGERDFYFDRLRSIETLTKTTESLLLDQFAQSRMPNGGISDDAQLNGS

STNYNNSNTLYEFIKKINRVLYDRPVEAESEVTNQVEHGSHPAQSEPDNIDIITPEPDLA

DQEDQLNNEKNILLEDAF

>model.g510.t1 Augustusgene.g510.t1 JFAV02000104.1:42405-45962(+)

MRLVGNDVDNVVNTKDIIDIKKTQGPFVSSKESSECEHSFQRSSEDDGHWELQDQKKRRK

RLRDYEDDDNDDVANRDLRKKVSKTSSDIDEGSDPVSSLNKKIGFLSESDDFIRKPRSVD

HNNNNNNQSANTTADVRTIEQQKAYEFSLQRQIQVLEKKLESLKKKVTYKKTTYDRICKN

LHVIRELLWNSYKKYSNDSFDKTKSRNKDKDRRRASVIQSLIEANKSSHAPTNITGTNRS

TEDHVSAQNQNVTDYINSLHLKDLLSSEESTDNSSSDAESQSVGRGRIGKQLSPSSILRA

TSKSSGKRINAIDSDSDEVNGDEINEHGSRGSSNKGYSCYKIPGSSLSMPNLSSLNKSES

FSAQSEHTELKRSNSKPSSFLTKANGNDVINSTNKDLSKEQSTTTTTNNNNKDTNTLNET

INQGHGNANSIHLNKNNIANDHKSPEYDVDNDNDDFHGENDQGDDNYDAREELRSNNSNQ

HVSPHSQAHSQDKNPHLFANNEKEVGVLSKKKFKDDLELASNSNTNNSTETNSTSTLGNN

SHGVASSNDENTISASSLKSSDNSKDVIENSSLDEDSAKTSEFSLAKSKHVINPIKERFI

KTEADTKTTSYNIRTSSQTNSPQNMAVYLNSSHGNKNAGHINMDFKTNPTSMSTTNNNNN

NNDNNNNNNSDNNGDLAYSEDSINQNTHQQGQNAIRVTLDGSGSLSMINIKNGQGINDTN

NNESHGNSNNSQISGTSNRTNARNEELIYENFVNDINSNNNTENISANNANNNVTTTYHH

HHHHHHLLHQSQWNLGQNQPFLSQNYYSSADNSNGNDLMIPKATNIQSFPYTDFNASNSD

NQLGASNVTTITTTTTSKANNISDSKRNSSSVSSNGNNFVSGERPSFPPSFRAPPSGSIT

SSVVNPTILPPPPSTFGAQNQMGVSSGVNAAGNLPGQDNVFNNASMTTNNPSVSEQPKQH

AFSVNNQNPAPLWVQTMGNNMEGLVLPTGNTMSNEDALRNMKAPAMNVETSASSNTLQST

SSQRGSSGQGTNEHKDRSMSAASNNSGKSIAGSNPSTATRTTNAQVGTLTESDVFERWGV

FFNERIKKVEDVWAEYHRVSEKGYSFEKLDLLWSNKWKLHCEPKFVKKFNRRLVMIKAIQ

NAVIESGQSASKFVDFMDQVLVDRKKPISHFYKKTNIPNLLEGNVA

>model.g549.t1 Augustusgene.g549.t1 JFAV02000104.1:136563-138098(+)

MNSTKHHVKTHSTYQFESNTNNVAASQMRNALNKLIDSTTSNSKLSNETRVKFEKEMDSY

FSLFTRYLNEKSNPSNKLQWDKIKSPTPEEIIPYKDLQSTDASTDKSTDLSSNLNKLAVL

KLNGGLGTSMGCVGPKSVIEVRDGNSFLDLSVRQIEHLNRKYDSDVPLLLMNSFNTDKDT

QHLIKKYSGNRIRIRSFNQSRFPRVFKDSLLPVPQDFDDALDSWYPPGHGDLFESLYHSG

ELDALIEQGVEVLFVSNGDNLGATVDLHILQYMLETGSQYIMELTDKTRADVKGGTLISY

DDHIQLLEVAQVPKQHVDEFKNIRKFKYFNTNNLWINLKAIKHLIETDTAKMEIIPNEKQ

VKRNGQDYNVLQLETACGAAIRFFQNAHGVVVPRSRFLPVKTCSDLLLVKSDLFQLQHGE

LKLDPSRFGPNPLIKLGSHFKKINNFQERIPHIPKIVELDHLTITGNVFLGKNVTLKGTV

IIVCSEGEKIDIPNGSELENVVITGNLQILEH

>model.g530.t1 Augustusgene.g530.t1 JFAV02000104.1:86027-91210(-)

MAVKRKRDNTESPLTRSDETKAPAKSSLVQQDAATEMSFPRGGASVLTPLELKQVANEAA

SDVLFSKEAQATSSKDGKPLSKKSKKNSKKSQDNGAENDEDIKTSSQIVQQQHISFKTLP

VGSLVLGQISLVHNHGLTISLPDNLTGYVPITNVSKQIDDILDKLNEDQMKDDEAQDSGY

ESEEEDSDIKKSAEQEIPRLNNFFKVGQWLRCVVTKNTALESSKKVSKRIELTIEPSAVN

KGLEKEDVQLKFSTIQCSVKSVEDHGAMLDLGLETASELTGFIAKKDLIDSSVKLVPGFV

FLGTVSKYSGRTITITEKQLSSTKPISTISSVDAIVPGQLVDFLVDSVKENKGVFGKVFG

LANAFLSISQMKTKNSKKFKDTFEIGSNIKTRVIANILYKGERVVLLSQLPATTGFDQTL

KSSESLESFPIGYTFDSKMAIIAKDSQYLYVQINDSLVGQVHFSKHSEDSEEFIEKARVL

GYNTVDNLFVLTNDKDQLSMEYLRSQDIPIGKLLTGCEIVTVSAEKGIELKILNGHFKAM

VPPKHISDIKLVYPERKFKIGSTVKGRVINVNPLNGKVFVSLKKTIVNADEDETPLVSNY

DQLAPGLKTLCTVENFKDNGAVVTLLGNLRAFLPNKEISESFVRRPQDHLRLGQTVPVKI

VNFDKENRKISVSCKIISQEKLESQKHIIDSMQLGRTIVQCVVVEKTKDSAVVELKSDHT

IRGVVYAGHLSDQRIEQNRSNLKKLKIGAELEGLVIDKDPKTKVFNITCKNSLIKDSQSG

DLPLTFSDVTSKATNAELHGYIKAISSTGIFVAFNGKFVGLVLPSYSGEDRNTDLSKKFY

INQSVTCYLLRTDEENERFLLSFKNPTNNTDAKTKPDASNTAIVNPIDKTVKTMQDLTMG

KIVSCKVKQVKKTQLNVILADSLQGRVDISQAFKSFNDIKDIKNPLAQFKKDDILKVKII

GTHNIKTHTFLPISHTSGKNAIFELSAKLDEEEIPKLSSFKVDDSELFGFVNNYSKDFLW

LTINPQVKAKLNYIDLSDNTSDLNDIQSAFPLGSAISCKTISVDTEHECLVTRSRFSSII

TSYQDVKVGDKLPCRITKIADSYVLVNLGNGVKAISFITDALDNFKDDLKTTYHINQVVV

GQIMAKEDEGEKINVSLIKNPDSIIKSASDLKVGQVVDALVKNVTDKGVFVYLSSTINAF

VPVSKLSDSYIKDWKKFYKPLQHVIGKVVTAESNKKISLTLRESEINGEIQILKTIKDIQ

KGDIFEGVVRNVTDFGVFVKLDKTVNVTGLAHVKEISDSKIEGDLTDLFGEGDKVKAYVL

KTNPEKNQLSLSLKASRFKIQDADNDVDMDAEAENEDAEDAEEDEVMDIDYNDEGSDDED

VKASSTNGATRKQISLSDDGLSLSAGFDWTGSILNQANEESDGEDNGEDEDFTVEKRRSK

KSRASQIVEDKTIDIAARAPESVADFERMILGNPNSSVVWMNYMAFQLQLGEIDKAREIA

ERALKTINFREEMEKLNIWIAKLNLENTFGSPETLEEVFKQSCQYMDSYVMHMKMIGVYQ

MSAKVDEALSLFKAAAKKFGSEKVSIWVNWGEYLIDQQRADEAHAILANALKSLAKHDHI

EVVKRFAQLEFNKGDPEQGRSLFEGLLADAPKRIDLWNVYVDQEIKHDDKTKVEDLFERI

ISNVKITKKQAKFFFKKWLDFEENKNDTKTAEYVKAKAAEYVQNAENK

>model.g540.t1 Augustusgene.g540.t1 JFAV02000104.1:118876-119154(+)

MPQALGKLNKNKKGHRVTKRQTNLRKAAPLQLKPKTVHKGLHLHKLSNKYSITQQTEKLV

ASKVGHLEILKGTRKEIEKQKKQKALKDAKKGK

>model.g564.t1 Augustusgene.g564.t1 JFAV02000104.1:168154-169227(-)

MPRKPPTTQPSMTKPTVFVSGATGYIAQHIVKQLLDSQKYNVIGSYRNSEKADQLRANFK

NNSRLSLVKVEDLGHIDAFAKVFQEYGPRLDYIMHTASPLDFTAKDLENDMIVPAINGTK

GIIQATVNYAPNVKKFIMTSSVAAVMGPLPADRDPHKKIVYTEESWNDRKLEDSLNNAHN

AYSYSKTAAEKLAWELHKELHPKFSLTTINPVLVLGPQCFDANAVGKLNVSSGIIYDLLH

TKPNDENLSTRNGSYIDVRDVARAHLECIANDKLDGHRLILSEAKYTMQTFADIVNKNFP

QLKGKIAKGVPGSDKKAIEELPTVVDNRVTKELLGFEFKKIDEIVKDVIQQVLKVQQL

>model.g499.t1 Augustusgene.g499.t1 JFAV02000104.1:10741-11484(-)

MSSSKEYTAFVLGSTGLCGGAILKHCMGSGKFAAVTTLTRRELPDYLVSSPELANQKGTL

TSIVDAGSENWATKISAPERGKNNVFFSGLGTTRAQAHGFANQYKIDHDLNVALAQKAKE

AGYNTYVLVSSIGASKDSMFAYTKMKGEIEDAVEKLGFENLVVLRPGLLLGDRHGNKSQS

FMSSAAESIGSYFYRSKLQKLFGCPVYGEEVGEVAVKLALETSSNANPSGKTKVRIVESK

DILDLAAK

>model.g576.t1 Augustusgene.g576.t1 JFAV02000104.1:198691-199665(+)

MFKIFQRAYHRRVVYKSWNGASTSSGPANVINMISTPRGKRNLALFVGGSTVFYVSNLHE

APVSKRVRFLWIPRSWELKIGDYSYNSMMAQVQNKILPQSHPEHRRVARIFHRLVEAAQN

DAQDPEVREQLKDLHWQIHVINDPQQPPNAFVMPGGKVFVFTSILPICANDDGLATVLAH

EFSHQLARHSAENLSKSPIYSVLGLVLYGLTGMSVFNRLLMDACLRMPASRQMETEADYI

GLMIMSRACYNPNESIKLWQRMANYEKTVSGYAGNVEFLSTHPSSANRIENMRKWMPQAD

AIYEQSDCGMFKSFNSIPKPFGLPF

>model.g543.t1 Augustusgene.g543.t1 JFAV02000104.1:122503-122871(+)

MSTLSTERFYFKRSLITNNVLFFTISFANCENSLFSAGYIGEFEYIDDHRSGKIVVQLNG

RLNKCGVISPRFNVKIADIEKWTDNLLPARQFGYVILTTSAGIMDHEEARRKHVSGKILG

FVY

>model.g527.t1 Augustusgene.g527.t1 JFAV02000104.1:81032-83089(-)

MPPKKTTKTTTPKPRRKPNAAKIAKPKLLKPLNQTAIINNATSTSTTPSRPFKFTLGKTS

HQAQQQPSGNTSASATSEPSVQASSTGAGGGGGGGLRIRFKPVRAFGEGYDSEDEDVEDD

PLIEEGVILRIDAGVEGPLTSELIRNCIETGDFSSLNIKWVDQRRAVLKIKQVMMDSGDG

AANGMGNELWSNYACVLLDLPTIIECNKSVDKGKNLLKTFDITQVLLCYSKLENEKDIFS

IKYEEKEDVEDPVLKNFERYAGDIYELRKAQNKMKRRMSGPDNDLVDGNHSDAELTRYYA

YKHGITPPLYNVKNRRMRKILNPEEIDYIEDYVKMLVDQDRNAEEFETQLVLEHEVETAA

SDKLARNGGEGVSEERTATAQPQRVSFSTNDTVHVYSGTKGTEEDESDAFDLELEQALQS

DEEMDDAPSAKESLVDRGEEKANTSDNGDEDLFGEDSDENGASEVADTANGKTSSAAEKE

SSAFTQSHAQDDMADEEDEDEDEDEDEEDDDEDENSDEENYSSMRTEVNEKMNHLKLVRE

EISEIENVLNINKKNYDAASNPLLKTRYGDMVKKLEKELEMKRKQLKADEDEMQGSGNGA

SGSGDGQSRDTKGNAGGQEEEEEEEEEDDEDDEEEEEEEEEEEEVEDAPEEQEEQDELPE

EPQAQDNHSDLDQDDMNMMLLFGADE

>model.g513.t1 Augustusgene.g513.t1 JFAV02000104.1:48309-50111(-)

MDDITETSTQETYHNDHKTIVNLLIAKYLIKQNYNQTFKKFIDESESIDLLELLNNKMSE

IDLNKNENENENDIEENKKTIVNEDLETLIYERLKYNEYIDEKKKKKKKKKNGSDKTEAL

GEEVNQDNIKEQKEKDSIKKILSKNYDMPNLHGSFHKIMLTKEEEDGEMTTTSIENALMS

QLSTGTKRLSNLRIIKLVVCGPYLLCSTSSKHLAIFDIKENYQIVKIFQFNSIIKFISTK

LIVQRHASDKSNSTTNYIYCCGIDGKFNLINCNTWEIVVANHGLSTSRAISHLEVYPVSS

LNRCNDHSTIENHHINIVVCGMNKHVYISQLDMNSLELKNVENTQTPMLSGITNMRVFQR

TQEEEINGIKTPFLKTYMALSLVDNTQLFVYEIPNSKETHKFTLVGKIALNNAQFSTHSF

NISSFSIFNAQSNFENETEVEGDTAKQDWKILITTSHIPYMRVLVVDLDEKLFFNNLQIT

TTVPIYYDLVKVNFVTDIEQSNYSTSEIFYNHSYGNFTIVTQFCMYRVIGFKTFPISREI

ATAEGKNNENYSSINNNNIIQSRSDDELKTITSCSITSDSNGLIISGTSNQSSEPIIRLY

H

>model.g494.t1 Augustusgene.g494.t1 JFAV02000104.1:936-3014(+)

MNEPDEVHATPNEPVASLSTPPIPTKRPERHAKIQSTKHSEINKDECISESLSALKGNDF

SSAESSRSHAVEAPQESQKLNEKQAEESMQKERSTELNGQNGGESMPELAESIKEFLPGE

KSLSDVHHTGTDGKVEHLIKPDSEVCQKEDESNFVETSPNVSSQKKRLDTDFSEETNGTK

NEFETEAKIKPDESFLNSTDSSGVETQENAKDNLISKEDVSNDRHEEAQRQVSGESNLTS

LEIVSENDSEKLAPGPAGLAKSSEPKDCNNKELEKTVELTSTTFEPKRAPPPVRPKPSSK

IAAFQKMLQQQQEEENFFVPKRPASAARPHSFSNRKTSSSSFNSVSSVAPEAEGETKMQH

KDNEETLGKKASSNPFFNNNPNFVSNLNGILAGGFGGASDSSAPATPHHPPTKNNTSASF

SSVPAGERVTTTDASSEKENSNTEACPVSQSTEEEKLADSLRTRRVRGPKGRKLPSKLQN

VAKVKIEATQHISVLTNWTVSFGVNTDQKRLTEQRRRSTEQQLDEILDGYVNLSEDSSQN

VSEKPLQLAHKEPLHETSKGILNAPSSEGLLQHVSSEVCVNVSANENHLTKDSESQENVR

EIQNNVSNQREDSTYDETDSRTKGPEPLNNGPDSREEEAVNHDRTDAACTSFLQRQNEKD

DKLNSREHCIPKVPLKRPVKRLSDNLKLPADSC

>model.g544.t1 Augustusgene.g544.t1 JFAV02000104.1:122996-125257(-)

MFSLQNTSYSKNYKHLNNKEQHQADKNNARPISAKSEETVPGNRPRKESSSITTYSNTSS

TLPINASLTGSSSASHSSQNPSYFDIRLRTNYKDMIVIAEDELEESKVFISGALIVSLVE

PMSVKKISMQLVGNFKLDFIQTTGKHGYTTIVKESRPIFESLWDNLLSSSIGTITDLNAQ

KKKKKSGKKNALTLHLTHSNENLSNLGHPGRSSSPVNADRPGLSSSGSSMSLSEDDNSVP

QHPKPHKKRTTSSASLWKKLGLDYISTGNSRSEISPSLNPLNSGSLTQDGKKKIVKVLNP

GDFNLGLDFFSDVDSSNGVDYYHLEAGNYEIPFSLEIPNNAMIPETVEGLQSGSVLYTVQ

ATIDSVDSGKISNTKYVRVFKTLKVNNWAIHENLYVGQSFQDKLQYDIRLPSRAVPVGGE

SPLSIKLFPFQKHYRIEKINVNMMQYYAVTDQHNQIFEEEQPIMQLTMQNFRNIDGVDPN

TGELTGDVHLRSTIRIPKNLKKITQDCTILENDTVTNTYKKLIMVRHQLSLKVVLISPTN

AVTEINCNMPIILYVSPYVPIETRRVVLDKATKIHFRPNEITPFFKSLLEPSMTNKRQEG

VFEGNSNLIRYFPQEEFSAPPTYNERVSDRIIDGETVNGLSHHIATAREMLVRRPLDMKS

LQNINKTPSYDHTFYDSDNIATQQHNITATATTTTTSSVSQDILGSEMTNGERTSTAGNT

SDDAATAHQAVHANPHLTAGFFQFAADLSPEYQN

>model.g546.t1 Augustusgene.g546.t1 JFAV02000104.1:127820-129024(+)

MMLGHCHATIHGSRYDLISQALVRLRQLKNEIGPKHFEKFFDLETGLLKFDEVFNAFAVF

LDMFDPAFAALYTEAMTLLNAVTLRLQTEMKNLPNSSIPHLNKCVRTVTIFLQIFFKKST

GLTEGSEFPETYLNGFKKSKHGFYGLTLFKGKKLKSIYQECDQDCTFDDLVKHVQAKQNS

RDCWLPKIKVPLMLEYHIRNILFMDLFLPTTYLENVIKEDPYFIFSLKFPYGMNVLPKKP

EFKILLRTIKFYPEQCRAHGQFAWSSQNMVVVVGLDIESHKCCCLNMMNTLFLEELDNLI

LPDHPAYPLNLNLEPDPEATKVLHRIFKYNTMIGLFFRTYENRRFTDFLL

>model.g565.t1 Augustusgene.g565.t1 JFAV02000104.1:169758-170303(+)

MNSNLNHKQQSPSIPTGAVSHDGAPAPQPKINEMIIPLLESSAYQRLTRILMVRPERVRQ

VEQYVIMLAQQGQVNQNHKLTDTELKGILDGLSRTETAKSAERAKTSGNNSASNGSNSTK

NNGAFTQGITFNRRGGFMDDSDDDLDFVMPSGSSSTTTATAANNTNYNDANAGDDSDDDF

FD

>model.g553.t1 Augustusgene.g553.t1 JFAV02000104.1:144743-146605(-)

MSLPDYYSIKIELNSGYTTSFAYRSFQSACHLKARPKRPGINNISSRRKHIKLESIIDPT

MSISLDQNNSHDFDIFGSIFEGKIRLFKLSGSSAAYNQHFGPLIKDEVVKKVVDEHNVMA

CIMFEPQINLLDVLKKVFPTKESLKTVTHVRCLKQSLFHSNVKDSSLFLNEFDEEQGYHN

NLLLVNFTTAENKQQFSLEFDGYEIQQTLLKVINIKEVVFTAKLFQSLPMGHKREISMKK

EYNKNSSYQNQPVLPYLIEDPFTKEHNSSVSNIELPTCPVCLDRLDSATTGIITTNCQHA

FHCKCLMKWKNSRCPVCRYTLDQTNQNEKKCSQCDAKTNLWCCLICGHVGCSRYSSKHAI

DHYRDSGHCFSVDLNTSRVWDYAEDKYVHRLVQNFTDGKLVEISGSSGSTQNQRDRAGGE

SAKKEISNDEDNLEYIQLLLSQLESQQTYYENKLTAIKQERETDLDKVSEKWEQKFKSLE

QKTFASEVQFKKLQESIEVGKQTNINLLATLKNMKDEIKTLKTHNEKLEREKLEQEENTK

DLMFHLEAMSKLNHLDGPLTSDDPASNLAGHIVIRETSDTKMSTLEAKKRKNKKKNEKKK

LKKLQLQGQVEDPVHEEDRDV

>model.g531.t1 Augustusgene.g531.t1 JFAV02000104.1:91950-96215(+)

MTAPNPTRQHRPSISSSRSSYLSETNDPWDDDHVTVPSSISTFHHNDHHIPQVVINESNH

YESSSGRPSITGSSIHSERESFLNERISGSLQNAQFKFFTNQQIENAEGFTSTIENISFL

DDEDPFYEGSQTSLLSGSQVGRRRHGSFYVRPPTTYGSIHSQRSSNSSSSSSSLVYNDQH

RVIPDGEGHESDGISHDEDSLLGEAYDNENFDNADLHVANLREIMDLNNYSKLSAKEHEK

YFKFNPQLQVQRLYIAEEDLVVGIAGYTTITYRVWLYYLLCIVTFGISYLFCRWFPKWKL

YITSKKTALGKADFVAIETEFGDFELAYVKRQWYNRALSTVLNRFNTEEEIDYDADTNFE

NYDSRRGSNNSNTAYVSRRRRSSLHEDPDLPLLITFEYRFFKLFYSPIEDIFKTNTNWVD

KNWKNVNKLKNGLSSNVHQDRLQVFGENKCELEIKSIFELIINEALHPFYIFQVFSIILW

AYDEYMSYATCIFIISLFSIASTVYETRKSSQKLAEMSHFEYSVRCYRNGFWISIASSDL

VPGDIFELSDPNLSIVPCDCVLLNGVVLVNESMLTGESVPVNKVACDKEVMSQLMFDFQN

GRISSPVAKSVMFNGTNLIRTKATDGSPVVIAMCIRTGFSTTKGSLIRSMVFPSHPPVTS

SSNGNKASKSKDLQTDAFKYIGYMFIIAMFGFTISAVNFKRLGLPTKLIVVRALDIITIV

VPPALPATLTIGINFSLKRLKDKLMFCISPTKINIAGKIDVLCFDKTGTLTEDGLDVHGI

QENYIARGKFSKLMNNCADIQNKIFQMCLQTCHSLNYIDGEGLVGDPLDLKMFEFTQSRF

KEHHDSGFIVNNKTHAFKIFEFASHLRRMSCLVKEMGSSTEAQTYSVVKGAPEVMIDICK

PGTIPHAYDSILKDYTHRGYRVIACAGKKVQLASGQSFQDLSRDYVESDLDFLGFIIFEN

KLKTKTKSTLAVLQDAQIRTIMCTGDNILTAVSVSRECGLLPKSHRCFVPIIDENAQSAN

EMIIWEDVDDPEIRLDEVTLVPSSGIGEYSLAITGDVFRLLFKNQDQKSFDSESKDGENN

IDEDIGNFLFSEHYIHTILLKSCIYARMSPDEKHELVEQLKQIDYCVGFCGDGANDCGAL

KAADIGISLSEAEASVAAPFTSQNFQIDCVLELIKEGRSSLVTSFACFQYMSLYSAIQFI

TITILYGQGANLGDNQFLYIDLFLIVPLAVTMSWSKPPSSNEKIVPKRPSANLVSPKIIV

PLVLHMLIILLTQIVPWMISKKMPWYIKPVVGDQDTIDSTDNTVLFFISSFQYVFISIIL

SLGPPYREPLMKNTWFIMNCVVAIMFNVYLLFIHDPANSWIAGSVMQLTYTSVPFKIGIL

VICVASYYLHYEIPNKFNRLFKKKQSSKLYKNLIKRCENISV

>model.g516.t1 Augustusgene.g516.t1 JFAV02000104.1:54561-55094(-)

MNKRATLNKPIAQTTPNRKMSDSGVPEERVRKHIDESEDDEDVDMNNNNNTRVIQRGDDG

EGQGYAGGGDHTEPDYQAEGVVMDEHARKRHDLEVRMTQALKRQGRGSSRRSRRDEEDLE

QMLDDKILRLKDEMSLAAQRDMETIERRINDDEEEEEDEEEDHEEDDDDDDDDRRRRS

>model.g526.t1 Augustusgene.g526.t1 JFAV02000104.1:80170-80931(+)

MANHHASIELQGFQKLNEYEKIIHVYKNCSWNCYPIMSDNKVNKNYEGKLGQKKATVIIT

NMRLVLASETSLASMPSLSSIPSYNSSLPSSSSSSSSSKPPSSTFSSTSSSTLTVEYPQV

IYNNKVFQKGKKISVKMLPPELVIPWIGENYLLLKFQPGKEQQPYLNYIYPWVLEIYCKT

DIFALKDCFSKVILESRMSMGGSGNNDFGSANANANANENAHPNANTQANHDSSRCTPDC

INQDNFVPLPKYEP

>model.g574.t1 Augustusgene.g574.t1 JFAV02000104.1:191687-193402(-)

MSGKLTLSVQGEIQCGLEGFELLNSPIFNKGSAFTEEERDIFKLNGLLPPHVDDLEKQIE

RAYKQLHDLRDPLRKNDFMTSMRQQNKTLYYALVNRHISELVPIIYTPTEGDAIANYSNR

FRRPEGVFLDITQPDTILDRMSKFGNDSDIDYIVVTDSEGILGIGDQGVGGIRISLAKLA

LMTLCGGIHPGRGLAVCLDVGTNNETLATDELYMGNRFARVRGKQYDDFVEKFINVIKEH

YPSSVLHFEDFGVTNARPILEKYKSQVACFNDDIQGTGAVVMSSFLAALKHTSRDLKTAK

VLIYGAGSAGLGIADQIVNHMVNHGLTKEQARGNIYAMDRHGLILDSMGDVSYEAQMFYS

KKDEDFKDINTKSLLEVVGQVQPTCMIGCSTQKGAFTEQVIREMYKYNDKPIIFPLSNPT

RLVEAVPEDLLQWTDFNALVATGSPFQPVEGYNIAQNNNCFSFPGIGLGAVVCKASTISD

TMISAAVDQLADLAPINNGDPKSGLLPNLDLINELSSKIAAAVVLQAVKEGVAQRKDIPL

KFEDTLKWVENQMWRPVYRPMKKTLGKYTHQI

>model.g506.t1 Augustusgene.g506.t1 JFAV02000104.1:29576-31111(-)

MNHTPKNGNSTKSVYTASQTPTMQNVVIVTPLDVLILLQIVLLDQHTTSDNKAAQQSKFP

LCMKRLDETCIKQLCQHGNIFVFREKESKMKRWTDKLNWSPSRIVMNKFLVYKQLVNHDI

AQKPNNYENDHGFMNLHGIINKSTLNQKQGDKLMYTGLCKKTISLKFQNDIVHVVAYYNE

KMQSLQYAPLENRFEIHNNQHYLKSLTEIYGASYPINKELQENMEKCSVGTTTSSSTSIS

NSTTTSVCSSSGGGNRSRSNSKTKTKRRTSSNSSSNNTIKNSRYGASNSEESLVGKFAMP

LSPPHSIMESSFATHHAPNHPTEHTYNQVSPLHYPSTPYHTLVGQNNVLMSNTSNTHKVQ

KQQVSLPKPSKMQNGLQILQQQLQKPYMSGDGSTHMYLHPTTTSIQHNYNPQNALHYATT

ATPLYQHLNTSGTQLSLHPPPPPPPPSFPPLQQQQYGSLEQRSISQQHRPLVCTQQFFLQ

PAPLSKSPSANSGSYQPNQLPSVQDLKLPTIQ

>model.g561.t1 Augustusgene.g561.t1 JFAV02000104.1:163944-164978(-)

MTKQTVFVSGATGFIAQHIIKQLLDSQQYKVVGSVRNSAKADKVMKDFKKNADLTLVIVE

DLSNLDAFDSVFQKHGATFDYVLHTACPILSADNDFQKNLITPAINGSKNIFEATVKYAP

NVKKFVQTSSYAAMRNPDANHNPDITVNEKSWGSLTLEQGLQNGWSAYFYAKTSAEREVW

ALHKSLNAKFVLTIINPTFVFGPQCFDSSVSKTLNSSCEIINSIVHSKPTDPPQKDIKGN

FIDVRDLARAQVQCITDHNLDGHRLLLSAGKYCWQLIADIVNTEFPELRGKIAKGEKGGD

EKVLNSFAQIDNHVTKELLGFEFTDLETSVTDTVKQILKVEAAAR

>model.g520.t1 Augustusgene.g520.t1 JFAV02000104.1:62441-64162(+)

MAITDRKRKRSSKPSRAEKNDTASTRSAANDEDITDPSSGEESSDEQQDSVEDVEDELES

EEEFEGENPADKRRRLAKQYLSNLQQEANKIVTESVLDERGQPQQEIDDYNNFDAADLER

EIIQSRLKQDVAEQEGRVYKFIAENLSLKNAKSTFTRVGQRNLTGLSCYQPMTIDNTFSM

EDTNKRQRLYAYTVSKDLQLTKYDITDFNKRPQIMKSVKGGKEFAPAEPLRTEYDNNSEG

HYDEILSVAACPNGRYVVTGGRDRKLIVWSTEALAPMKVLPTKDRRGQVMGLAFRKNSDQ

LYAACADYKVRTFAINQFAQLEILYGHQDHVVDVSALAMERCVTVGAGDKTVMLWKIADE

SRLTFRGGNEDPDRLKKKWLKNNEGKTEKDCPHFYTENYIDCCSMIDDSHFVTGSDNGSI

SLWSTSKKKPIFTMRTAHGILPKIKNDKISAETNKEKRNLQLQGQNLTKPYWITSIYAIP

YSNVFVSGSFNGSLKLWKLSDDLREFELLKEFKNAKGVITKIQVVTSGPPNKETYRILCT

VAKEHRLGRWISNVPKARNGLYSVVIEQNASKGK

>model.g571.t1 Augustusgene.g571.t1 JFAV02000104.1:182479-184614(-)

MIAENQNSFRNSNVNTAFIRNASTNGKTKSNRGSKTKAKNESYTQTQTPSPSAQHNNNHN

HNHNHNNKHSNKVINFGTPVHSFVDGDGLSREDKKVTNILNTDSSSSQDEEMADVASSVS

GKSNDSTESFKSSVDSNDMILINTRNDDDGDNNDNNNNNNINTNGEELTVNKLPSFSTVL

DKINNAQPNNNSNNNNNTQTTAMSSSNMRKHDLSSNPLGMHVIGTSLPSNQSGSNVLSGG

NLSSNLSSNTGSNTSSNVTDSNTDSVLESNSNGTNSRSGRSSISSGNSGNSGNSLIGNTN

ADQQFEQKYSQPKQSFFTVSNPLNDQNGGSMKPPRTQPNTNNVSGQYNLVPSYQTMLSSH

SQNSSNNTNSTGIRPSSSVGSFTSSNSAFGSPITMNMANIQQSPQMDAQSFWNAQQDHSG

GLNNGKGDVPMHSSNSLQSVSTMGSTNQFPKLNTPFGNGTQMSPQINGSGFSSSNSSTSN

VANPDMISPLSGKYPMSNPQQVVGKFAPAPSLVSMGRKKLQNKDEFLTPKNVATTSSCHI

CGKVFDRKSWLERHLSCHNDMRVYRCLWCNSRHKRKDNLLQHLKAKHPVELIKALFEEMS

ANHYPQGVLDKFQERAAIVLQEVTNSEKGGSNAQQLDGNDHNSIASNTTSTISDIFDTEK

NSDGSLEPHSVHNKIIKVLVREGVLSKDLLRNVLNTLVVKQADYPDDIYHVS

>model.g524.t1 Augustusgene.g524.t1 JFAV02000104.1:70933-72525(-)

MSYNWSIDEELNHTPNSVQPITYIGYSKQKMSKDSDMATSEERPLCKKELNFFQKFYYYR

TSLQFTTNVYVGVQYNILPSLTDLYAALAQLAAENPQLCLQAFSEQPSEVVEGKDFVPPE

SLYVAQLRKIDFAEVVEVHEDFDIFESCAMELLLSKKFAYGKDDKPLWKVFILKDNWLVF

NYDHLFLDGISAPIFHEKLINSLEKKELNVKLENECVYYPKKAETLREFDLAEIYEKPQV

NVSFLAKKVLPTVAKPVIINALNAIVDRYSLSPALIGTLYRYSCGPKYLVKRHKFYENCL

YINIPQTDILRLIKVAKDCEKISLNSLICSLLSIATKNALITKEQSLKFCVDANCRGIKN

VASDKMGLIIKTMDVVSPLLKNNLLTETGDLNLKEFWSFSLAIHKDLVCQIPTADGIDNI

NLLSLIDILSYLNDKEGQSPEFAFELSNLGLQFKDHAATSQKSLDKKYAIKNAIFNQCIG

ITTCFTVSSIATPIGGMNLSIQYPQELSGEVENVIANFKAYVKLLVSLGDI

>model.g508.t1 Augustusgene.g508.t1 JFAV02000104.1:36148-38577(+)

MSTLISRKICGSTQAIKSGAGGLRLPGINGLSKIARNTRILQNANDLLKNRQDRAFSTQL

MRENFCYKSTLNGSTLVRTRTFHKSSHFFNQKNKDQDGKSNEKDEHKQQDKENNQKQKDE

ANYKNMMDYFKSKEFQKTLIYTFTFSFLYTYFTAVLLKNSEENGMHEPLTFQDFKVKYLE

KGLVKRIFVVNKQYINAELVTSSLGSDVQGAEYSDGSVLSSSSHSTLGQTNYENHGQMVS

FTIGSVDFFEDEMDKIQDQLHIPPHERIPIIFVEQTSMLQYLFPFVPTILLLGGLYYISR

RFQAGGAGAGGFGGGKNGGIFQVGKSKAKLFNEETDIKVKFQDVAGCDEAKQEIMEFVEF

LKTPEKFTKLGAQIPRGAILSGPPGTGKTLLAKATAGEAGVPFLSVSGSEFVEMFVGVGA

SRVRDLFAKAREMTPCIIFIDEIDAIGRSRGGKQGGGMGSGGANDERESTLNQLLVEMDG

FETNEQIIVLAGTNRADILDKALMRPGRFDRHVEVDNPDVEGRKAIYRVHLKNLKIQPSL

LENLDLLSGKLSALTPGFSGADIANCCNEAALIAARHNDNFVEMKHFEQAIERVIAGLEK

KTRVLSPVEKKIVAYHEAGHAICGWFLKNADPLLKVSIVPRGQGALGYAQYLPGDMNLMN

NLQIRDRMVMALGGRVSEELHFSSVTSGAHDDFKKVTGMATSMVKSLGMCSRVGYINYDE

SKSASNGGGVLINKPFSEATSQKLDEEIKKLVDECHEACKQLLQSHMPHIEKVAQLLLKK

EVLTRQDMIDLLGPRPFEENNPAFTKYLDK

>model.g536.t1 Augustusgene.g536.t1 JFAV02000104.1:105436-106764(-)

MLPDDIKIGKERTKGRTKEALIAPENNESGHSATLHDSQNESNESKRRTKKFNRKRAVKR

DELKTETSGPNASGPTTSGPTTSKDTSTSKKSAKRRNNNKRKQGKGKPQDVANKELPQKE

EEKELIFIVDPTCFTKGLGHVMQWVSYIEDMEKEAATSSAKRSNTTGPQKTKITLYLSTY

TIEELNFLKTNRNFTSAIKSLKFIDTKFEFFDMEILEKKSVVEEMSWSGVLRDLFFSTDQ

STSSSNVLERASAEPESHSLQAQDYAETENTVDAFDAVLDKPYQRQLVKKLFQATMKIPM

RYKKLIKSYHLLQKELLQRSREDPKVLENTKVYIVSADIYVLQDLNQIFNGAVTSTDSEL

TESDQQDNKKIEILDILKADIMIDEIYHSKCHKVLSAENGSSQHSGNGYSSHNIMSNNNV

TSESMYFKKNFFAPRGKGELWTP

>model.g562.t1 Augustusgene.g562.t1 JFAV02000104.1:165434-166471(-)

MQEYHSHKPVVFVTGATGYIAQHIIKQLLDSKRYRVVGSVRSEEKAKRLAKLFECSQDLS

FEVVQDFRKPNAFHDVFQRCGPQIEYVMHTATSVDLSCGDLEEGIIEPAIHGTDNILAAT

SSFAPNVKKFIYTSSYGAMRSPEEDRDDMRIIDESSWNSLTMEEATQDGKTAYHYAKAQA

EKLVWKWQKDWKNKFEVVIICPTFTFGPQCFDAYVGKTLNASCEPINLLVHGAPVNNAIK

GYFADVRDVARAHVESLTNDNLNSKRLLISERRFCSLNVAKVIYSVLLQLKGTIAEEPTL

TDAQNSSRFAHLANKATRNVLPFELMSLEQSLKDTVEQILRVEDAK

>model.g557.t1 Augustusgene.g557.t1 JFAV02000104.1:157264-158292(-)

MSQPTVFISGANGFIAQHIVQQLLDSKAYKVVGSVRSTAKADKLKSDFGNNPDLSLVIVE

DLSKLDAFDSVFKEHGASFDYIFHTASPHNFSFEDFENSVIVPAVNGTKNIFEATVKYAP

NVKKFVQTSSAAAMINLGPGEEAKKPVTEESWNLLTLDQAMHSVLTSYMYSKTTAEKTLW

NLHQALSPNFALTVLSPAYTFGPQCFDSAASDGVNVSNRPINDVVHSTPQDFTLENTTGN

FVDVRDVAKAHLEAITNDKLNGHRLLLGEAEFTSQDIADIINTNFPELQGKIIKDVHISG

ESGPDFSFKMDNHVTKELLGFKLIDLSTSVTDTVEQLLRISKN

>model.g528.t1 Augustusgene.g528.t1 JFAV02000104.1:83339-84517(+)

MSKIAQLTESLLQEVPAVKKLAKFTQATDYHFLQKLYQGKRLNLKDKYPHRGETKILDLY

PGLGFPSLALYETLNPKQQLLLDTNEKFNEFIDKNLQMVKTAQQPSKSDGNKFKYYHTQI

PELSNMQHAHLNPYQWATYSTITNNDIATNKTVNPEKAIFLPSQKNTDKLHDEFLIHGNL

TGLVCARYSGEALLMQLYGCVHRKNWMQRFGRAKMLFWVREATALKLIARPGDVGRSKAS

LIAETFTNTSLVAVNKNTVENPKSKRYALHPELIAKHNPVIFSDDEAKIVTEDKERIALI

EIDPVTFPSFDFEQWDYVTRQLFVLHNHPLTASISTLGPGAKEWILPRLPKEWHDSKPTQ

LSKEAFLKIYEIFSKWPFKPDINLEFLDIYQDI

>model.g503.t1 Augustusgene.g503.t1 JFAV02000104.1:24295-24657(+)

MSQRSSNNSNSNLNDVSDSNRGLDYNGSMLSQLESQSEEQMSLMGTKVKQLKNLTLKMGD

EIRSSNSTVSQLGETFEGTSLRLKKTFNKMMIMASKSRIPLRTWFLIFVVIGLLFFYVWV

T

>model.g558.t1 Augustusgene.g558.t1 JFAV02000104.1:159140-160156(-)

MSKPTVFISGANGFMAQHIVKQLLESKAYRVVGSVRSTAKADQMKADFKNNSDLSFVIVE

DLSKLDAFEGVFQEHGASFDYILHTASPVNFAAKDLEKDVIIPAINGTKNIFEATLKYAP

NVKKFVQTSSVAAMYDITSDTTPKPIVTENSWNPITFEQGLENQLNAYLYSKTAGEKGLW

ELHKSSNAKFPLVVINPTYVFGPQAFDSSAKGSLNASNEVINAIVQSKLDDANVTNVKGG

FIDVRDVARAQVEAISNANFNGRRMILHNRNVASQEVADIINNNIPQLKGKIAEKVTVPE

VGPGFSANIDNHVSKEILGFGFIGFDKTVIDTVEQILRV

>model.g501.t1 Augustusgene.g501.t1 JFAV02000104.1:14254-14985(-)

MGSVRARKMARSSVKKATRRTKDKQREANIRGNAIIAENWDYSQTLAQNYKRLGLKSKLG

TAAGGQEADLSSKVMKKSLVRAQTIADMDSDDEDVDDAASGADFINPEELNSDGEFDEAK

IPAGEARIKRDSDGQVVKVIYGSKKFDIDESVEVLKKAEQNKNAQKNKAETQVVQQLEEY

ANRPIVRHERVESEREDEWLEKLYKKHGSNYKKMFFDSKLNIYQQSEGDLKKRVEKWKAR

KGLN

>model.g578.t1 Augustusgene.g578.t1 JFAV02000104.1:200940-201935(-)

MADQYEARVSNNMGQNVFEDDIDDTDFFFNNNDNNNRERSPNGTNFQSIFSNDLFGDDDD

DDDDDDDDMLELYNLTPRQRLYYKLKHNLYTKNVKRFKKLRLWQKIILIILLVLFLSFVL

TFVVMHKKILEYLVETSNELYLKWYTSWVLFVLLYIVAFPPMIGYSLLSTSVGMIYGVSF

YGWVLLASGTILGSISSFYIFKKFLHNQAENLVHSNRKFEAIASVLQENNSYILLALVRL

CPFPYSLTNGALAGIYGIQLKNFVIANCIVSPKLVLYLFIGSRFKNLGEEDNPNKWFDLM

GIVFALSVLSVTAGVLYSKTRKRYRELQSDVL

>model.g538.t1 Augustusgene.g538.t1 JFAV02000104.1:112610-113557(+)

MSIAAAVFPNNSTLNNLLTNVDGTKSFSQVLNNVSQYQPQLSLMEKYWASWYCYMNNDIL

ATGIMFFVLHEFMYFARSLPWYIIDCIPYFRKWKLQPTKIPSNKEHWHCLKSVLLSHFLV

EAIPIWTFHPMCEKLGITMEVPFPNWKTMTAEISLFFVLEDMWHYWAHRLFHYGIFYKYI

HKQHHRYAAPFGLCAEYAHPAETLSLGFGTVGMPILYVLYTGKLHLFTLCVWITMRLFQA

VDSHSGYDFPWSLNKFVPFWAGAEHHDLHHHYFIGNYASSFRWWDYGLDTEAGPEAKLER

EERMKRKAEEKVKKTN

>model.g522.t1 Augustusgene.g522.t1 JFAV02000104.1:66373-67863(-)

MSSTEIPKGEMSFWAKYFYTKVCLEMGTCVYVGVQYTKLPELPELYAALKRLVDENPPLG

LNIFAAGVEDMNCNIVQTPNGPVKPDPPQNLYAAQLKEIDFSTVVENLTTFEIHNFETVK

YILQRKFEYGSFTKPLWKVTLLKDNWLIFSYDHAFIDGMSGANIQKKLVVILNDMNKNND

SYSVNDSTYKITPGSTDDNFGIINIYEKPENTWGFATRNLLDSKIYPVLKSCAKAIVQKI

GLKPTWLGRFNKFECDAKYVVQKNHFIDNYQNVSINAKDVEKLIGVGKKHGNVGFNSLIG

ALLSVSAKNTFVLEEQSVPFMFLVSERLKRKLDPESIGILIKAEEETTPILSNTLFDSVG

LNHTEFWKFASQVHADMTRAIDTGNGLEHFNMVGLQDLVTKVKAQEGSFPHFLFCFSNLS

LQFKEEKFKEGNGLKYAIKDAVFNQSQVYADYFTVSSIATPKGGFNMQMVYQDEVKKETE

MLLANFEKLYRELINSV

>model.g537.t1 Augustusgene.g537.t1 JFAV02000104.1:109075-110871(+)

MQDAAFTQNAFKEVTLVDEKQYDTNESTHSVYTPNNSKTDAKPRTFWQKVKIGMRPNTLD

DVHARSKTLTEEEHLNLENENPLEYGQHFLQHKPSMFGRFFDRLTNFAGDTVVFLITCAI

LVMWMILGIVYRAPDNWQIAMQDGSSIQAYFSDSFLMRQQQNEYMDLLQFLSEFKKNAVN

YLKIFEHYNATTEESRRTTFKPLEQDEIDTIEEELTAVLGDAQKMQTYNWFDRLTDFASK

IMGSFYTSVLYWIGIFVWIGLGALPYLHFADEWQLYINTATAVVITFASMFTQNIRKRHN

KYLQRSLSVILHTDADIDYKLRNYVGFYESDDESVAGCSGSGSGSGSDSGSDIDNVSEDY

KTIKAKPLNKRVRAIEYYAHFVGGGVGAAISSCVFIAWICIGKTMKFSDNWWLIIGTYTG

LVGFVDGFTLRSSYYRYLRYCMKQIYELESLEENLLTYLHLPVTKTRILTNKERSFNYRA

SAWLGKWISYSWGVVASIVVVIGLIILASGMRWSTTAQLICNTPTMIVEGFCLLALLEGH

NQNNEKLRILAHDSLVRKLYLKTYVEHFVQNHGEQDSDEKKMQVEITTDTRDNSYDSEL

>model.g523.t1 Augustusgene.g523.t1 JFAV02000104.1:68697-70163(-)

MSFWAKFFYTRVSLQLSTCVYVGVQYNKLPELPELYAALGKLVEENPPLGVNIFADGNVD

VNCNEIETKNGLERPDPPQDVYAAQLKEINFSEVVEDMKHLDIHDYEAVKTILQRRFEYG

NFSKPLWRVHLLKDNWLVFCYDHAFLDGMSGANTQKKLAVILNKMAKSSETFQKNAIFEL

KPYSAADNLGIINIYKAPKNTWGFAARQILDSRVYPILKSSAKTVVSKLGLSPAWLGRLH

EFQSNTKYVVQKNHFIDNYQNVNISAKDTKNLIQLGKKNGSIGLNSLITALISVSTKNAY

VTEEQSVPFHFAISQRAKFKTDPESLGVIIKTENVVSPVLKNNLVTPSGLNVYEFWSFAR

GVNAEMATAIKSDNGLEKFNKLGLVDLVPSIKGKESKTPTLLFYHSNLSLQFQEKKFRDD

DDDDLKYTIKNAVFNQSQVFSNNFTVSSIATVDGGINLQITYPDELIAESENVISNFNTY

YKNLLESLK

>model.g575.t1 Augustusgene.g575.t1 JFAV02000104.1:194932-198465(+)

MTLNESSISQILEKREGGTDERRLSGKQKKRRKKKNNMNDLEKLVSTIETYLQKQKSADV

NITRPLCVVIAKVFMKVSGGEDGESKFTQSVGDLNSSLRKINSSELYAVLTNRLNKPDIE

EQRKKDEPPEGSIETHQNNDFNNNKPPTEAKSMFKKRKKFVVANVEPLPNKKVKSPLLGN

DDEDDEEEEGIGATIMFNRQLPNKQKCTESMQKSPEPPSESSRIDLLQLESEINGIKQKI

QHCLSTANKHFQNSKGSTSYIENEHYKLTPVSLKERLQLVPPFLQTLNERYMPVKATKTS

QLNDTNYLLDIIMGSLLPSNFASTDGPHTDLIEGKIEISNDENGELTQSCNKSSKMLVEY

KKVVEQKKHVKNIVLNDTLQRVTEQDQNSSVQEEEVVDDDEVINTDEDEEEKDSTDISES

PRFIDNSHTLPVYACKQDFLKVLQENQVVLVIGETGSGKTTQLPKYIYEQDRETHKIAIT

QPRRVAAMSIAQRISDELQCELGKEVGYAVRFDDKTDKNLTRIKVMTDGILLRDFLTMSS

SSQIDYTHIIIDEAHERSLNTDVLLGFIKQLLLLKQNRLLKIIITSATINFKTFLQHFPN

APVFKIPGKTFPVQSIYLKRPAMDYIETIIQSIVKLHLSNPVEENNDVLCFMTGQDDIEI

SCAMLEDKLTQVYAQAVTETPMTTTDEKENKNFVILPLYSTLSPKKQALVFQKFPGKRKI

IFCTNIAETSITIDGIKFVVDAGFHKLKLYNSKIGLNVLKTVPISKDMSVQRQGRAGRTQ

PGVVYKMYTESCFNFELLKNSIPEIKRTNLSNVLLMLLSAQEGSSNLQENHNSVEKILKF

PFMEKPSKLTLLSSFLELYNLQAVDNTGDLTPLGFKMSRLPIPPNLSKMLILSKQATFDC

FDEILIIVGMLSIESNSGAGYFQRPKNFEKLADSKRLNFVVPYSDHLTLLNLYSKWQYNN

CSMKWCERNFINHRALLKVGKIVEQLQRVANGIGVGTFKSNKKANRKHHQNTWENVKKCV

TTGYSYRNIGKKIGLNKYMSLQNGMTLQIHPTSSLFGLPDLPPYIIYHDLLLTSKEYINC

VTAVDPVWIMEYQPFYFNIHAAVSEENGEVVHPTNNLEQLEALQNLREYSINLLEKQKGL

ALIKNKEERNTAAHQEHQTYMSNLSSLKTFKKRKKMGF

>model.g555.t1 Augustusgene.g555.t1 JFAV02000104.1:150798-152879(+)

MVDDTYYITPHETALAVVATSMKKARLRLDTLIINSTVGGLLFSAGGMLYLYSRAENPAM

AETNPGIINFLGALTYGIGLFYVVINGADLFNSNILFFSVGFLRGAVTICDLLVSWSISW

LFNLGGSLFMCYVICYLSGVATSENMVAGSIYVASSKASFSFIQTFIKGIAGNFCVCLAV

YLQLMAKPIHVKYILITLPIFTFVAMGFTHVVADMYLVPMGMMNGAPVSVGEYIWKLLIP

ATLGNMVGGTFFGAVIPYYLHLFVVEQDRKKLELPDYDARDEQPELNMDSRVVRTDPKNI

DEELDSEVEDFDQNEKTASRATDSFGSGETYTNDEENASMQPQRYFPKTHSSVFSSALNK

TKSAGFKKRAMRSPAGVFPVQGMGDPLLREMSIADPSIVEKVKSKLSAQETREQGNEKAL

ESAEDSANHARLMNEEDIYDPRAAGAPSLENLDDYSIQSYYEPPLSTAQSSQIAQQASSH

LTSPKLNKVPTFAALEKEEEQEYKQDGGYDVNANKLGTKLEKALSHIAGRDRSLSKSAKH

SPVAHVLPLTNQDTENFTKRHPDPQITRAYSEVQKPHPALLKSLRKSFNMSSASISANEL

QRRLSEVGVTQRAARAADNIAGLENFQDMDLPVSTSPFYTLRKTSTSTSTSSRRADGDYD

QNIYKNSKKPRAPSNLKNVVLYDNDDMEEQSIAD

>model.g502.t1 Augustusgene.g502.t1 JFAV02000104.1:22606-23481(-)

MSQDQEGLPRSLNNVKHIILILSGKGGVGKSSVTTQLALTLALKGLKVGVLDIDLTGPSI

PRMFGLEGKEVFQSALGWLPIEKKFNASSNKAHATGSIKVMSLGFLLTNRGNSVVWRGPK

KTAIIKQFLSDVYWGDDDLDYLLIDTPPGTTDEHISIAEELRVIGMLEEAGLGQTKIDGG

VLVTTPQSISVADVKKQINFCKTIELKILGIVENMSGFQCPYCPECTDLFSSGGGEQLAK

ELDLKFLGKVPIDPKFVEMIETQSATTDLLEEYASMKLYDIFKAITAAALQG

>model.g539.t1 Augustusgene.g539.t1 JFAV02000104.1:114097-118110(-)

MTVLILPGPQALSAFRVDNLVKKISGATVINKDLVSTCFVHYVELQENAELTEKELGLLN

SLLIYDSPCLDSNLSTIVSQNTSTQDETLIRVLPRTGTISPWSSKATNIAHVCGLAGKIN

RIERGMAIYIKSHKPQNLTLTEDSLKSVFDRMTQSLFVNEIPNKESLFNHESPRPLVHVD

LLAAQNQGSSQTAYDILKQKNVELGLALDEGEIQYLINAFNQRNPTDVELFSFAQVNSEH

CRHKIFNADWTIDGELKNLTLFKMIKNTFNNCPDYTISAYSDNAAVLDCDHPEDKQFYYA

PDFQTKEWKSTLEKVHLLIKVETHNHPTAVSPFPGASTGSGGEIRDEGATGRGSKTKCGL

SGFATSDLLIPNHKQPWELDVGKPGHIASALDIMIEAPLGSAAFNNEFGRPAINGYFRTL

TTEVKNNEGNTEIRGFHKPIMLAGGMGSIRPQFALKNKPITPGSHLIVLGGESMLIGLGG

GAASSVASNDDNVALDFASVQRGNPEMERRCQEVINACVSLDKANPIQSIHDVGAGGLSN

ALPELVHDNNLGAVFDIRKVLSLEPGMSPLEIWCNESQERYVVGVADGHDLEIFTNICKR

ERAPYAVVGVATEEEKLVVEDPLLKTTPIDLDMSVLFGKPPKMTRTAITQDLQLTKPDYS

QIQLNDAIERVLRLPAVASKSFLITIGDRTVTGLIDRDQFVGPWQVPVADCGITATCLSS

DFINQPGEALSMGERPTLALISGGASAKMSVAESLLNILSADVKSLKHVKLSANWMSPAS

HMGEGSKLYEAVQAIGMDLCPDLDLAIPVGKDSMSMKMKWGDKEVTAPLSLNITAFAPVN

STSNTWTPELANKTAPLVLVDLGKFAMGGSCLLQVYNQLGDEAPTVYNNKQFKGFCEAII

ELHVLQSSVVGAYHDRSDGGVLVTLLEMAFASRCGLKIKASANKSGNLIAELFNEELGAV

FQVLDMAKFKQVMNKHGITDDLISVVAEPLQDSLDVEIVDGQQDQVVYKAVLNDLLSAWS

ETSYLVQELRDNPNAAKEEFENLKDTTNPGLFYDLTFSPAEPSKIANSTENKPKVAILRE

QGVNGYVEMSFSFEQAGFAVYDVTMTDLIEGRFHLKDFVGLAACGGFSYGDVLGAGNGWA

KSVLFNENLRAQFIDFFQVRKDTFAFGACNGCQFLSRLSELINPENPQIWPIFERNKSEQ

YEARVCMVEIVSDNQVFFKNMKGSKMPIAVAHGEGQATFASAQDLSNFESNNLCGVRYVD

NYGATTERYPYNPNGSPSGITGILSPNGRVLAMMPHPERVTRLESNSYYPKDKYTEWAAR

GPWVKMFKNAREWIESLN

>model.g512.t1 Augustusgene.g512.t1 JFAV02000104.1:47615-48265(-)

MTSINPFEMTDLFNLQNVNLDYLTENFPVEFYLEYLILWPTLFFQNTETLNTIPPIATQS

LSSFQHPGTTTHTKHPISGYIMGKTEGKNLEWHSHITAVTVSKLYRRISLASQYLCVPFQ

LLSDSLNEVEFVDLFVKCDNQLAMKMYEKLGYSVFRRVVGYYNNSNDINTASTNLKILKN

NDEKDAFDMRLSMPRNNNKSIRKDGKNHRCLPQDVRF

>model.g548.t1 Augustusgene.g548.t1 JFAV02000104.1:133879-135966(+)

MTNFKSKPNLISNLISNQQGESKGIKKRHTEHTEFDENRDNIDFLLKDSVITATTTVAAN

KNTIKSNSGYHHLDTQPDYRSISIGDIILDHSELLMDSEKKMSQIDIDSQKSMATIDIDS

QKSMATIDIDSKKSMSIKNQKTKDKKKANKKDIQKQIQRKKSTRSCDYCRLKKIRCFFKQ

EGEDKDGSTQCQNCIKNGLECTFTRVQLKRGPGMRSMTKSMTKSMNKASASKQSTPNLKE

QISPEKGKTAHRHQSKKQKTQKEQVAAYQIQEPYSQQRVNSIGSASGVALPPLSTFQSEA

ISSTAIPSEARDYVTSNKNTNNSNTTSDPNNHTYSPSPSLTAQYAYTNGFSGLLQNNVAS

LGSINYNINTELHHRGSRQENLHQGGTLPQSHMPGQHMNNAINTETVHTDTLNSGNLNND

TLSNTIINNNSDNNNNNNNNNNNNSVAQQFWKVPYNRRDSIVSLTSGISSSSEKSSREDH

TNLFPPGLASGVNVERLLMNLHATGMTPNNTTTTPKTTYTDHTNHPNHTTRSQSTTTIQS

LMNRDGEEPARTVTGVSPRQQEQMNNIFQANPMLQTFLEHQNQQNIMHSAEQGQRHGGVE

SQDDGLSGIIMMFLKTVEGLNKKIVKLENRVRVLEANDRPPGTTTSEASNTFAPNNNTYT

GKTNTSKDNNTNNTNKPLQEQTPVHNGNTSNIDTPK

>model.g495.t1 Augustusgene.g495.t1 JFAV02000104.1:3575-4951(+)

MVVDTSYYDVLGILPTATKLEIKKAYRKKSIIEHPDKNPNDPEATIRFQAVSEAYQVLND

DQLRSRYDKFGKEESKPEQGFADAAEQFKMIFGGEAFESYIGELQLMKNMQEQSDLEKEL

MKAEEDSNAAAKEFEKDGAGAMNQSVDGEFSPAEGTPAYPINNNVDSKKVKMISHVDSIS

ASMSDLNISEKQKQKQLEEEKKKKIKEIQDKLDESQRKAKEETVEKLVKTLIERISILTE

SVYDKACKEAFKEKFEMEANLLKMESFGVDILHTIGKIYCVKANIYMKAHNTWGIGGWLP

SLQSNCDTIMDTFKTVSAALDAQHMMQEVEKAKTIMQAQQEENTKADEEKSLLVDPSGTP

IKVPTAEEYAEMEHLLMGKVLSAAWFGSKYEIVSTLKTVCDRVLNDKEVSASKRLKRAEA

LMILGNVFKSVYRTKSEQEEAQIFEELFSQATKKKAAPS

>model.g550.t1 Augustusgene.g550.t1 JFAV02000104.1:139422-141899(+)

MRIDGNTVLFIFFIIFFVILPNNERDQGSTQYEVKQIQYAKDQLRYELAFLNNNITSIHN

IPGNITGFKYTQEDLSLNHDNYETSFVKNESLGTPWLGYPIEGKDYRNGWVNYHSKGNIL

PQNVLETVNEQVWTNKTKVDEANLLESVYPWNLTSALRGIFGKPESKNTDSVLYLPRPKY

YRQQLTFTDPDYFNPPDNNNMNEKREDDQEISLADLELPQFGDEFLNYSTESINYREDYV

NFTDRPFQVTIDEVYQYNHEWKIYSLDFKDSKDLSRNQLPMIGIYNVLKGQLLTFTQSSK

FHPLFVAPSYFFNENEDDFHNLKDMVNTFINKTNYIDTFNREDLNTWETRANMRCEYMGY

FQVAKWQGFNARDMDMFERELAKPMGRPIAHKNPPKIEISNGVLYSPDCGLYYQFDKFAG

DREEIAVQKIRWDIIFFVTIPYMLQLFIFLKQMNFTNTPSRLNKVSRTTIDMLTLTDGTM

AMTLFISAIVMDSLFVPLMVTSFFAFFLTLVFGIRYMSNIIVSQINERIISWRSLFRSSG

HDNADGGGTETAQTGADATTTAPAAAATPVANVIPDESQLTSDLNAKFMFKLFLSTFTMM

TIASWSSDSRNVLEGIAIVFCNSYWLPQISRNFLIDISPDLVGLNFKWFVVGTSYVRLVP

VVYIYGVKANIFHHHQSNMFILLMFVYVTLQIVVIKLQHTVGGRNVLPKVFMKHLNKYMN

SKKKYEYHRKFTTGEVEEHGAVFSCPICMDEDDPIKVMVDVCDDSSSENDQHSNSANEAE

DHESQKTSLDKYMVTPCYHVFHTTCLVTWMNTKLQCPVCRQPLPPL

>model.g551.t1 Augustusgene.g551.t1 JFAV02000104.1:142135-142866(+)

MTKDIKQIRACLFDMDGLLINTEDIYTETQNELLSRYGKGPITWDVKIQLQGLPGPVAAA

KVIKHYDLPLDLTQYETEAVEIQTARWGTSAFLPGAKELLHFLKEEQHLPIVLCTSSGKA

KYESKTGHLRKDFDLFDAIITGCDSRIPKGKGKPSPDIYQLGLKVLNEKYHKDGKDLIKP

QECLVFEDGVAGVLAGVSFGGYVIWCPHPEAADHLPTPESIMHEQGEKVDTLNHLDKSKF

CFQH

>model.g554.t1 Augustusgene.g554.t1 JFAV02000104.1:147053-150190(+)

MNQDPYDFSAIREVCVEVSKYAFLPTNIFNPNDPALLSSLDNLVDVLQSQEGLKHQPAIT

DAKLADYVFFPISQLLKHNIPQTCQPLLIRILKGLTLNFPLWGQLDHAHFKQFLPLALVL

IGTKENIQQFEVVVNFLASFLPCFTSFDTNDLALLAFVAQLVQLILHILETQNIDTELRL

KCLSQNLFTLFSKVLRSDGDILAFILPGVISSLVRVLLGRTKNASSPMHKPSPVLVAKVL

ELLGYLITTVYGDSDLESILQLSDLSDSADVVQLLENTANELDVSLNLELRELSKPHDSM

SRNHNQRDEKWLKGTRHQILKTLQAFISKLLKRNSPVIDQALCDFMKRLLKECNQSLHVC

ERYLSESFLQLQNTEVNHALIKQPVVREILKSLDLTKTVFFQAGYHEETTLVSGLKYMDK

NSDNDNAILCKLITDLCENIMNFKNSLIGNKLSRAYKSEETILHPHVNMKKLKPVHESDT

KLLLEHATVSGANLVVLSQLDEQEECILGKYFNKEMELKLKNILQNLGSKLEERNLNELL

EALFSTFESDGMIIWIGSNLLLGQLPATETDPLSDFLVYEDEEKDKDSGYLLEEPCCLIL

DTSSKKLNDILSNNAITYDKKLQLNLTASLHGISCMVDLVPHVVEEELMDLLPAIVECLT

LSDDATRHYAQFLITKMAQTYTDSKNINELIYENISYIVDYCATKLNNSIINRCSVILSV

ICQLGGYEMISSFGDVLESIFEVLDYHHDAEYTSVCIEIFQLFLTIIREIDKKYMACSEK

YLISGKVEFSNSSLKPWSMTSMKQVIELIKPSEADIHSNQDSEQTQIQEVDSDDEEIDEE

RFRSTENSEQNASTQEERPWTSPIPQQAYKLIVQITNYGERMLTTTTSLHLKTQILNCIS

ESISLLATQYDSFLPQVAQSLWKIVYDMSTENVYLMEKSFQSLRMMIHYAQGFLIDRFLN

AWAMYQKSALIKKLFLQEIQPKSKDSVTGVSPSDALSTAVNSSYPTALLETVKAEFSKML

VEGLNVSFLRISDVEYCKIMKVIDEH

>model.g515.t1 Augustusgene.g515.t1 JFAV02000104.1:53741-54484(-)

MEKVKLLPKVAAALNKVSLADTILDNNLLQAVKFWLEPLPDGSLPSFEIQKTLIHAIAQL

PIKTEHLKESGLGKVMIFYTKSSRVEPSLKRLSDKIVAEWTRPIIGASDNYRDKSIMKID

FDVQKYKEKSTQELKKNLAKEKSARLAIANGGADISGSVTKSSKKSKRGSTKDGKKRSSL

YDPGQQRKNRTVIPQQTTTDYKYAPVSNIENLKDHQLSFKTSGVGSSLSNSELYKRINSR

LGNTKKRR

>model.g505.t1 Augustusgene.g505.t1 JFAV02000104.1:27408-28556(-)

MSLNNTFKNKENIPLITRNLYSTEIVALSGALSGFLAGVVVCPLDVAKTRLQAQDNKFAK

TNPLSSSDKTSTTHATKAFAKKKYNGLLGTLKTIVKEENIKGLYKGLAPITLGYFPTWMI

YFSIYEHCKHSPLYRTHLGEGYQLHSLSAMTAGLVSTVLTNPIWVVKTRLMLQTENINKS

IVVEVVPVFAPSNIINNNNNNSSNKVFVDNYENKYKGTLDCFIKMYQYEGWRSFYKGLLP

SFFGLFHVAIHFPLYEKCKAILHVNTSAQNILPINDNESPQYSPIMSSKDQYLGKLLLAS

CVSKMIASAITYPHEILRTRMQVRNSQSLVKTIQKIYSTNGGLKGFYQGFFTNLIRTVPA

SAITLVSFEYFKSFITKHNTHFA

>model.g521.t1 Augustusgene.g521.t1 JFAV02000104.1:64269-65777(-)

MSTPSEISATCKQELAACNKFFYTRNCVDLSTNVAIGVRYNKLPSLPELYAALANLIESY

PPLGLQVYAEGDADANCGGGKTTEAGIKPLPPRDIYAAQVSEIDFSTVVFLKESLDVQDK

AAIDLFLEERFEYGSLKNLLWKVFILKDNWLVFNYDHTLFDGISGISVQQHIISFLNRLE

PSTDNSFKHSSIFTPKQLTNPKDLGVVNIYKPVENTWGFVTSRAIDTKLKPNFKYYANAI

VKRLSLNPSILGRLNQFENSSKYLISKNQFADNIFVLSIPQKDVTTLIKMGKKHGNISMN

TLLCSLFAAATRGSFVLSEQSINFFFPVNVRSIGNMPQDSIGIMIKGEEVVTPVLKDEIF

ASTSLDLNKFWEFALEIQYEMSSAILSPKGYQSFCLLEQADLLSLVRSKEGKPPTSVFGH

SNLGVQFKENASTTSEKNQYTIDDALFYQSNVFSNCFTLSSISTLDSGLKTSIVYPNDLK

QEMETLISSVQQYLKQLLQAEQL

>model.g560.t1 Augustusgene.g560.t1 JFAV02000104.1:162248-163279(-)

MSKPTIFISGANGFIAQHIIKQLLDSKAYKVVGSVRSTAKADKLKSDFKNNPDLSIVVVE

DFTQLDAFDSIFKEQGASFDYIFHTASPVNFTITDFQKDMIDPAVNGTKSIFEATVKYAP

NIKKFVQTSSVASMIDVSSDISYKKVITEKSWNPMTLEEALQNPSFAYQYSKTTGEKTLW

ELHDSLHAKFPVVVINPTYVFGPQMFNSSVQKTLNVSNEVINSIVHSTVENSNTISNTVA

SSIDVRDVAKAHIDAITNDKLNGQRLILDNQRFSSQSVADIINNNFPELKGKIVEKVTSK

TEGPAFPAEIDNHVTRELLNFKFIDLSKSVIDTVSQILSVEAAK

>model.g532.t1 Augustusgene.g532.t1 JFAV02000104.1:96346-97806(-)

MPVQLFGRDQIVVEYQNDARTNSNESIEQRASKHHSLELETPPKRHLHRPKDKKQYDHMR

SNMILATPPSQKQTRLSPTPKRPHRERSGSRKSSFPRLFTPYTDEETSDVSNTATPTKSP

NDAIIANSVHTTPNLVSPPTSKQHSPLPVSLLDNTRNYQTHAPVPITLKDKVKYHSYHYS

VKIRQFLDNVNLSNVPKISNVSILHWFLLFGFITLLLYHFIFVFSKLYSYSPVLNNICTN

VLLMGISDNMAQIIEIKYNNRSAVMSRNGDLVKPEYYDGMDSFTMHPFRVRSVAGSVADP

STVPSTTVHGEDPQIVDPSLDDLFDLENQKMKSLNNELPLTISLDQSIGKENFKCYRWVC

FIFWGAMIAVPQCFWYKILNFYFTEDPSIVQILERMLSDQLVYSPILLYMFFYYSNYVME

HGTVEKFQQKLKDLYLSTIMINYMIWPLAQIINFSIVKPHYQPIFSSTISLFWNCLLSYR

NSLHHER

>model.g517.t1 Augustusgene.g517.t1 JFAV02000104.1:55772-57652(-)

MFVFLIRFDDVVPQITLHSDKPTAKPQKTTTMFGNSGTSGSTGFSFGSGARNTGTSTSGG

LFGKSAATTTTPGGGLFGQQSTTSNNTTTTGGLFGNNNTAATAGAAPSGGLFGKPAGAAT

TTTTGTAPSGGLSGNTGASTGATTANSTTTGGGLFGSNNANTTTTSTGGGLFGNKPAATN

STGGGLFGASTASNTTGGGMFGKPAPSTTTGGTGLFGQQNQQSQLATGVTQQPAQPQTGA

IFGKTTQPSFAWSNNSAHLQQQQQQQNSLLQQQQQQHLQQQQQLQQKQSTQYSANYPQQI

QEQLLKLQQQWLPVTDNCKFKGFVYNKMDTTQQAVLDLQKPSNVITQNEWEQALKDRPGE

DYLPLLITSPTDLQERIALQTKTVAQYRQIMNNVIYEQHLSQLKDKHNLDTTIRLSKCQT

KLRDLESRLWKLNCTIKTILEQTKNYKFEINSDVLHSDYLENHTHASNKDDNRHDYKVGG

VAKSHYANNMFGIPVSGIDNNAVPLDEYVQHLVKQSEDPSSLGKVQSGELWARLSLLKER

CKVVLERLDGTLGSSNGGANIDAKDEEQQEREEDSKDAGNDEAKSLEANIDAKLASVLNV

QQDGLMYLNKVLLQDLASLKELNTSST

>model.g541.t1 Augustusgene.g541.t1 JFAV02000104.1:119248-120069(-)

MSQPDKEDLRSPQAFPSELLNTPKHEYPETYPEIPKFITFLKERSGLSWPVFIPVSTIIV

RTCITFPLSIWQRKRLIKQNELKPIVKAMSPVFKSKLAYSNNVSNHKLSPEQIILLSVKE

TRKRQKQLFKTCNVSMWKNLVLPLVQFPLWITISYGLRNNPLVLDHDPILTSLNSGLPLS

PIVFSSVLGFLSLSNCEMYAKILKLRNHFEISQRSTNLRNSFTNISRFFAMLLTFTSFQQ

PELLVLYWISSQTYSFVMNLVLNKWWPFEYKLIK

>model.g570.t1 Augustusgene.g570.t1 JFAV02000104.1:181761-182291(+)

MLGFTDIKSLINFGKLISNLKSFNFSLYGRYMGYINVLLCFALGISNLFHVNLVIIFAII

AIIQGLVLIFVELPFLLKICPLSDNFVQFIRRWETNGARVIFYAIMAIIQYCSCIFKVTS

LLAVAIGLTFSSCSYGVALIKHQDFAKAAPESVIKNPIQDDDAFDIESGLDTSRQVL

>model.g563.t1 Augustusgene.g563.t1 JFAV02000104.1:166676-167710(-)

MSKQTVFLSGATGYIAQHILGQLLESGDYKVVGSVRSQEKADALKANFQNNKDLSFVFVD

DIAKEDAFDEAFQKHGAELDYILHTASPFTFEIKDTEKDLIIPAINGTKSIVKAAVKYAP

NVKKFVVTSSYAAVASPSSDYIKSNTLTEESWNDQPLEEAVKSPIDSYYYSKTAAEKTAW

DLHKSLNAKFKVTSINPVFVFGPQQFDSSVTKKLNTSCELINGIVHSQLDDPVSNDMRGA

FIDVRDVAKAQVAAITNDNLDGHRLILSEGRFSLQSIADVINKKYPQLKGKIARGVPGSD

KEAIDSLATLDNHVTKELLGFKFIGLTESVMDTVNQILKVEAAKN

>model.g559.t1 Augustusgene.g559.t1 JFAV02000104.1:160733-161764(-)

MTKPTVFVSGANGFIAQHIIQQLLDSKFYKIVGSVRDATKKDKLLRDFQCNPDLSILVIK

DITKPDAFDSVFEEQGSSFEYIIHTASPVNLAKTDFQRETITPAINGTRNIFEATVKYAP

KVKKFVYTSSVAAMLDVASVTSTKITINEKSWNNMSLAIGLYNASNAYVYSKTASEKTLW

ELHKNMNAQFAIVVINPTYVFGPQVFDSCVRPKLNLSNQVINTIVHSTLNNSSGVKITNG

SAVDVRDVARAHIEALTNDYLNGQRLLLDNQAFSTQSIADIINTNFPQLKGKIVKIVNLG

ETGPDYSSFFDSRLTKILLGFPFIELSTSVIDTVSQILRVEGRD

>model.g507.t1 Augustusgene.g507.t1 JFAV02000104.1:35270-35572(-)

MIYSDHGSGFQSGLSKTLMSELAIAHVVTTERDNRPNRNTERFNRTIMNTAHTLSIHNGL

EVKCWPEEAAFATMVLSALQVSFGDCLKVLLGLDALSVSFL

>model.g542.t1 Augustusgene.g542.t1 JFAV02000104.1:120357-120665(-)

MSSSERACMLCGLVQTEAEFVSKGCANCQSIFDKAGVDTIDCISPSFEGYVGMCKPNKSW

CARWLRIDNFHQGLYAVKVDGRLPVDVVDLLPHYIPRDGSHVE

>model.g504.t1 Augustusgene.g504.t1 JFAV02000104.1:24714-27059(-)

MKLSSSGFVFLPFWVWLVAIFHPAFGTLEGTTKETSVNAIENTKNPVFDIPEFELPPTLS

TEDFDLVTQSSLVVMEFYSPYCSHCKTLAPIWKDTVQNFTLDNKYEDFRDKIQFHQVNCV

EMGDLCARENIRAYPAIKLYGPEGFIKDYPRDFKRNLEDFLRFAQLEVLNLEQLPTKQTS

NDATAEKENKDSLLNEDRSHFLNPMALAGLIAGELPVNVKQVGNLDVSQPCLVSFWPSSE

FESPDAFVNSKAKMDSLFSECPNCFYSYKNWKILSNKLKANNIQTGVFNCFDRDSSSDEN

KKVCQDLGIMDSLTEPQIFMIVPGKAYNNIFKYKKQETISTVKETIFNIDRVVDFSVRII

QNAKIPEINTMELRSFTTPKVDTLEKNTTPEKIYLVYNYDAESLVQEELDCLEHMIESVT

EFPNMYLYKTSQDLSIFSEKSVDSLYRKINYNESEPIKTLNLEVYHNKMLTQRPTFYLFK

ENSLIPIVYKMYATVEVRDLYLVMNWIEQDALPNLIELKQTNAQLTFQFNKENFPQVALL

FVDSSKESSKDFLYNYRLSSFEYEMLRWENNYAALQSARDEKHARIKELKQSSSSGNRKK

MFEEMQKEFYLNLDKRCVFSYVDLSVTTDLAALGIELPKDITYNVGDILVIDNSTPTIKV

YDTDPFGQKLTASSPYYLKELLKSLNLDGINKVAYRYKLPETVYLQGFKNSSSSVSLFFL

SFKQIICLALVVIVLLRVIVVKKKPRFIYSKLTLLQRKLRAGKFNNKKNGNLGILGSKNA

RL

>model.g519.t1 Augustusgene.g519.t1 JFAV02000104.1:59152-62079(+)

MATNQSPEIVEKEVFSLKGKTFVNISLNDVLLTINEKSICKIFELNNSDKEEPEVVDTCE

SIAAFTPIFKSPTTNKWAFCLADKIQDNVYLYTHNENIAMATPKTIFRSFLDIRDICIVH

NGKILAVASDDLEVKLLSLEDILKDDGGVTTGSMSPSGTTKKSQAIKVSKPVVNISFSHI

SNILAISCNDGSISLWSLSSDSPRLVHGGDDSEEQLFNYVTPNYTLGLDEDDDDDEDEPT

DGIQSATRIADKLDEVSTSRVAFGDNNGVFACCDNMTISTFAISNVRKKVKSFTHDNKIK

DFKYCGNDNYLCLIDYSDRLLLWHIASETLVFNKLITKDENKLTNVAWKKSTTNSNNFNV

YCGTKNGNVVFIENILSPNNVSVLSTKTADKSDSGNYGLFVNDSDNEDVDDLDEVNQRKQ

KLNDTISRGNELFNDEAEEDDKDNNEKHGPLSGKRRAHFDDYDDLDEELKNLNNDSFADD

LDENDDQEDDGEMNDGQEDSDDGGSLFNDYSGRPRKRVHFGPTSESLKPWSSAGTEFHLS

DRRYLTMNSIGYAWTVKSSNTQSVTVSFFDRSTYREYYFEDYNMSDVCSLGEKGILLANS

KKGEIAFRSHSTDSNKWSKTIPLQKKEIITSVALTNKKCFIGTSLGNFYELNVFGYIINI

ERLSPVAAMVAYQNRVFIVHRSAAASFGNEFLTFSLFDDKTYFQREFILPIKKFMKNIFF

TQYGDPAIFTNDNTLLLLSKWRDSKKSRWVPVLDVGFEIWRLSGGNQTSNDIHCWPLGIA

GNYNNTLNYIMLKGSKKYPEFPLPLPTDMEIKIPIFNKPLFEEVKKQKDLEDKVRKEEGN

GADGDENEDDDLASIAYGTKELEIPQEINAEEEFLRSKISHTMLQDTLENDGELFGDEQT

TLDQLNTIYDKSLLRMFAIACSDSDIKRAYSLVIELKQDRAFGAATKIAERAELIQLAEK

INQLREARSEHEMDLL

>model.g573.t1 Augustusgene.g573.t1 JFAV02000104.1:190064-191419(+)

MNVIGYISNYIPQSPTSNKQHQESTNESLLDLAQHPENGTNQQENFENAQDLNTHHQESL

EIDQLIRTSADNNLTSAYSSGTLPKRTNSLNKRLSQVYDNTADQLALQGQPPMSSKPETT

APTAPTTVSTSYKIWRVIIFVPNLLIVRPILFVWYIVTFPLTIIEMISSYALKSNESTPP

SAHPGTPNIPSNTNDLSNVHSVTLKQDFIKDGTPTPSQQPCRDFGGKFFFPKKLIPNSIM

NNPNKKKTLVLDLDETLIHSVSKTSNMSNKAHVVEVQFVPSAIPPIFQNTTSLKQRRSAN

NNSSASVISTLYYVNKRPYCDLFLNKVCKWYNVVIFTASIKEYADPVINWLESSTRARFV

QRLYRQDCTLKDGIGYVKDLSILCHSLDDVIIIDNSPISYSMNIDNAIHVESWINDPSDS

DLLQLLPFLEAMRYTTDVKTVLCLKRGEEVFH

>model.g511.t1 Augustusgene.g511.t1 JFAV02000104.1:46367-47470(+)

MSQYIGKTISLISVNDSRYVGVLQSIDSEEGVVSLQQVRCFGTEGRKGGWGSPYEIFPNP

QVFETVTFKGSDVKDLSILDIPLDQVQPVLIQPPPPPPSSSSSSTQQPQQTTDTQEPPSA

ISSYGAYQPAPGMTSAASHANKESDTEETAAGTNHKESLAKEEDNKGDVQEFESRKPRQN

RRRDPSSFQMEVPKEDFDFAGNNSKFTKEAHLKHSNEENEEQRDNHGTGKTEGGAEDDDD

EEEEVEEVFYDKKSSFFDSISNSNEESNSMRWNEERNVNMDTFGQERVRTNGNNYRGRGG

HRGRGRGRGRGRGRGRGGYNNYHNNDSGNFRGGSYGNNYRGNYNNNRGGRGNYQSSQFSS

APTENIQF

>model.g547.t1 Augustusgene.g547.t1 JFAV02000104.1:129614-131938(+)

MLRTAFKANYQRIIHNSCSVFARCSSQTRSSPRNTKVLGIDLGTTNSAIAYHNPHTHTTD

IIENEFGKRTTPSVVSYIPKTSTSTSTSSPGTPWKVLVGDPAKRQQILNPKSTFHSTKRL

IGKSYTEIVESGKLGEHAKHDQMQYDVASLASGECGVVLPLPKSTKLEGTATTADNTRPT

QIVKSPAEVGSEVLKYLLAQARHHFFGQTNASFAEPSSEPSSEPSSFPEKLDKCVITVPA

YFNDQQRQATKHAGALAGLKVLRVINEPTAAALAFGMDETTGAGESGKDGIIAVYDLGGG

TFDVSVLDIEDGVFEVRSTNGDTFLGGDDFDLLLVEYVIGEFVRGLPLDSKITKDVIYGD

AQLMQRIRDACEQCKIELSHVEETSVDIPFAVEGKHHLHVTVSEAQLDAMSMPLIEKTLL

CVQKAIKDCDLDIHRDIDEVLFVGGMTRMPRIRTEVARLFNGAPDLSISSAPASHTTRMN

YSINPDESVAIGAAIQGAILSGEIKDILLLDVIPLTLGIETYGGVFAPLVPRNTTIPIKI

KEVFSTGVDGQTGVDIKIYQGERTMCMNNQLIGDFKLSGIPPMLKGEPQIEVTFEVDADG

IINVSARERRSGVAMEISCVSKQVFEMDSTQVENILHESEANAERDEQLKTSVELLTKLD

IITNDCLKLVNSDYLKPLLQKAERAERAERAERAEKAERAEKAESAAGSALEQEFALLKD

QLHMINKDIGHLKHCSQDASLVDQLLQYDVNSLKEKLMGIQQSLFKSIKQVNDMQ

>model.g566.t1 Augustusgene.g566.t1 JFAV02000104.1:171443-173500(+)

MSYSTPSVDIFNPITDDEIRITTSLIKKSFPNDNVQFIQVDRVDPPKKLATQLLNYVRYG

TGTKPRISRRAYAYFYKNSEMPLYKSLVNTTERHITCTCAVDAVGPLSPEFVSECDEKVF

EIPEVQAEIAKLKLDSQFYDHPQLGKLKYKVLSEAWMYGISKEEDAEKPLCQVFMYLKLD

HPNANHYSIPLTFSPVLEYLTAKYIRMDYLPNDALESFNPVVKPFVGKDLDHVPFEYHPD

VVPNYKPAEGLKPLIVEQPEGPSFTVDSNNKITWQGWEFYVQSNVREGFAIYDVHFQGRS

LFYRFSLNEMCVPYSSKNETFSRKMAFDLGDCGFGNAANSLKLGCHCLGVIKYLDCRRSD

RDGNPVVNPSTICMHEQDYGILFLHQNYRSGASTTTRRREFVVQTIATVANYEYIVNFVF

DQGGAITVQVRATGILSTTPLDTEVTSTDFGTIIAPGVHAPFHQHLLSFKFDPRLDGDNN

TVCYDDYVPNEEDPEKWNVGYVQKRTFMEKSGHIDQSPFTNRIYKVINEHSINPTTKKPV

AYKFEMPARQMIMAGKNSYHLKRAHYATQQFWVHKYDDERRYAAGEFTNQSRKDTGIAKW

CDGTENVRDEELTVQVTLALTHPPSSEQFPLMSSDFMQFGVSPASFYSKNPALNVPLATN

KFNQSVYYEDSAKTCSKAAGSCCSKI

>model.g525.t1 Augustusgene.g525.t1 JFAV02000104.1:73159-78549(-)

MSANTSIPSGNNNNNNNNNNNNNNNNNASTTSNTNKNHLIREFAIPDKHLTKEEINRCYL

RWQQLRAKHEMNAANVPEFVYYTEVLKRAAKQQQEQKQAMLAQGANTTTTANTSETSINN

NRINGHNSNTHSNTNISGMSTPKIAPNIVISPNPSTSNSLPTVNTNNGYSQQANNGYPQQ

ANNGYPQQANNGYPQQANNGYPQQANNGYPQQANQSEYPLNGNTQGKSDFLNAQNISNHN

TNISNATNHKTIGRKESVAVSPKSDGHGNMGQNDDLSTTTITTTMNNTDNNTTIMNNNTN

TMNNMNNAHNQINANGQTQQQAQARQPGKVSIFTPQQTSLLKAQISTMKFLLAKQQVPIN

FQTTINDSVTHPPNYKEMLMALSETMRQRKAGVSQQNAQQQNAQQIPQQSVPQQNTQQNT

QQNTQQNTQQNTQQNIPQDIPQQSMSPQGPIPQKMSPQASAIAEAQQKALNQKLEQEAKR

KEEEAKKLALEKERKAQEEERKRREEEIKQRAFKLEQEKLERKRKLLNEIPVPLDKFKQN

NPDPQLKIVDVMDPEMKVESYTLPVAFPKYKDSLVPFNELFSPSSKIVSSLLPLGIDLQN

SLEIYQTLIALNTDTEVNNTLLQILNCDEENDKDYKKKQDKLILKLNSLNLIPLQKAVRG

HVLSFERFQRTLISNAYPTFVSKQRLINLEDANITNTLFTNHEILQQEIIKQNHSARVEN

IVDDCARQLKARQDKKNKRVKFGMKITSLHANLEKEEQKRVERNAKQRLQALRANDEEAY

IKLLDQTKDTRITHLLKQTNSFLNSLTKAVKDQQVYTKTLITDHIKHDKKKSEKNPASLD

LQTTASGLEDQSSGLQAHEIDGGEGEEEDDDEDEEEEEDEDGGDGSIDYYSVAHRIKEEV

TVQPSILIGGTLKEYQLKGLQWMVSLFNNHLNGILADEMGLGKTVQTIALLTYLYEVKKV

HGPFLVIVPLSTLTNWNSEFLRWAPKLRKIAYKGTPNERKALQKQIKAREFDVVLTTFEY

IIREKNVLSKIKWVHMIIDEGHRMKNAQSKLSLTLNTYYHCDYRLILTGTPLQNNLPELW

ALLNFVLPKIFNSVKSFDEWFNTPFDNTGGQDKIELSEEETLLVIRRLHKVLRPFLLRRL

KKDVEKELPQKVEKVLKCRMSSLQQKLYEQMLKYKKLYIEEDDGLNEEDSDSVVKQQKKK

GLTARGFNNQIMQLKKICNHPYVFEEVEESINPSRDTDVNIWRCAGKFELLQRILPKFKA

TNHRVLLFFQMTQVMDIMEDFLRFIGLKYLRLDGGTKADDRTTLLNLFNAPDSEYFCFLL

STRAGGLGLNLQTADTVIIFDSDWNPHQDLQAQDRAHRIGQKNEVRILRLITQNSVEEAI

LERAYKKLDIDGKVIQAGKFDNKSTSEEQEQILRQLMKSEEERKRKRELGEDDDDELEDD

ELNEILSRGDEEVAIFKKLDEERMKVDMEQGVLSRLMEESELPEVYKKDIDAEIQREAEE

AAAFANENNGRERKVATYNEESEEQWLKQFEVSDDESSGYRTRHGRKAAQETVKSEEQDV

EMKDEEDDSKDLKTTEASTEPETVDLSASNNDALHDSAANKDNDDHEYEEPEHKPIGSKR

RAGKNKAPAATKKIKSAVLGRKGRPRKTGRDYIRTVVAEGDEEETDITEKAQELHDALVN

YTNSENRRLSDIFMEKPPKSVYPDYYRIIKYVVAMDNIEYHIENHCYSSLMQVLEDLHLI

FANACTYNAEGSVLYNDALELHAFAIDKFVEMTGTEKSEIDFSRFDEEFLTKPLDFK

>model.g579.t1 Augustusgene.g579.t1 JFAV02000105.1:853-2859(+)

MDLFKVADYYIDKIVNTPSKRLQATGSTNNGNFVTSHIKVLLLDDTTVQTISMCTTQSKL

LEHEIYLVDKLENQVNQESMRQLRCICYLEPSEETVDLLCAELRNPKYGEYQLFFNNSID

KPQLEKIASSDELECVTAIQDFFQDYLIINRHFFSLELSAVQLFKNDGVHKIWRSQYELN

SVVDKLLSCLLSLKVTPMVIYEKSNDSVAKLLAESVANKMQHSHKTLFDSNNKNRDSAPL

LIILDRFQFDPITPLLQPWTYQSMVKEYLNIERNVVDMENSIGEDLDDEDEELKKIVLNC

RQDPFFEKTMFSNFGDLSDQVKTYLNDYKKQVEMRKNKIDDLQDIKKFIANYPEFKKTSF

NVTKHMTLVGALDNFLKNQEIWSISEMEQTISTTNMTLIDEDYQVMLRMLQDSNIEERYK

FKMVVIYICVYYMLQRKQQEQQKNNPVSLKMDSHIVALMDTIKPLFNVNDVNFLYHLIKK

FTAAPARRGSTGVPGGGSLNGHANSVHSRARGSSTTSNINNNVHEDLNSTITRSFNESDL

FSGLAKRFNQTIHMKDTRPEAENVYMQYEPKLKSLLYDLNGHNLQKMGFYDKYQIVSGNK

NTNKLDLPQDVIIYIVGGVTFEENRIVEEFNNDKKKNHNMRCVLGSDKIYNTSEYLGSLK

TLYNLGNEV

>model.g583.t1 Augustusgene.g583.t1 JFAV02000105.1:9555-11402(+)

MNKHGRSAFEDVKSHWVNKKLKASSPDVTPSAKKTKTETQEAKNTSRPTVIELSSDDDDD

DDDDDATDFTVVKSSQTNPTAVITTTMTPNREQSLVYPRMYLLYSPVYDPLTCIDNTNKA

VNPFYITEQTMFSSLATELQSDKFYHKETLFLWSFQFDFEALLPHLELHNTRVSRVIYAF

HQQGTVLPPEDPVLLEIFEDLVHKKLLLFRQFQMPPYSSFHCKMILKVNKQLQKCKIIIP

SSNFTKLEPKYVQQMVFDSGVMSLRSDSGGSEMQKNDQPHGFRKNFLKFFEIFHHKFKDD

HLINEVFRIMNHRADFTYLNGKYDFVFSSSKDMQSGLQLLKKTVSARGSPDQALTSAPKT

KFTVQTSSIGAPLSKFGESTLLETSFPQALPSLLTQTTKCEVVFPTSRSILRSPLGLLTS

GWFHFHHKQSGKIASMYKRWKQSKMLFIHSHGYRTESSLNSDLNVSSTARQYVPSHTKFY

IVEDVDDAGTCGGNSNNSNNDTKIVRFWMFTTANLSKAAWGVYHIKPVNGVHKFTPPTNF

ECGVLRVCKKNSGDKIILRNIIEYQQLWNNTLEVNKPAFRDFQKEYEDGVVYCIFDPSKL

VPYSKTDTAYCPDEST

>model.g582.t1 Augustusgene.g582.t1 JFAV02000105.1:8876-9175(-)

MFGTSIKNITTPTGSTPKIAKTEDIKDIIEDLEEVHLPSKFLCTIKKKATEKQLKDGKRK

TNSLDSKFDAKGDLIPDDADELLMAQYLDKNPNRDFNDIK

>model.g584.t1 Augustusgene.g584.t1 JFAV02000105.1:13476-16280(+)

MGNSPSRNNEYINNVSANNNNSNNNGNAGSTGLDDGSNAESLDPDDRKCISSVNDNLKSK

SDNFRNHDEHKHTSENKSKSKSKSKSKSKSKNKDKDQDKDQDKDQDKNSSLNASNRASKK

NLIGGYSTTAHSKSDNIDIKGTITKSGVSSSFSNEKKKSKKTHGLINDLYAGGTPYKQQL

QTYKSSGKSSSSSGGGSGGGSGKKHKKSFGVGSNGYYSTTTNNINININNSSTPTSPNMK

TNDFEVDLEMAKAFSNYNFNLNNNNHNIDNIDNSVSALGSENNTSSYSSGTNDDDDDEDD

EDDEYDDDDDEGIFEGEGELEKRKKGTKNAAPINSLERPSVVSKLRAGSNGSTSSSSSEG

STSTIESALSTASTSGESNSESNSESNSDSTDSTVSASLDSGGNMAHVSSLNGVKGEVQG

AESNAIKNGKSADAVIDGTDSSPDSPISPVSNSIMSHTSNATTTAVPSSSPSSVSSNDSS

NHSYHVLSAKNVANLPPIDEESLKDKDGVENLTKSATTLNATNSNGSTEGQPLLLQRRKS

SSSTSKYLNGLKNLSSSPTLIPVASHLSDLSVSKHSSRSKSHGSTSVPASASTPSSSSSS

SSSTSSSMRHSHKHKSKKSNADYLNVDECIEKLLVLGDLREYKSKSFPFELWEIQLICSR

AREIFLSEPSLLKLKAPIKVVGDVHGQYQDLLRVLKLSGYPGKTGYLFLGDYVDRGKQSL

ETILLLLLFKIKFPKSFFMLRGNHESANITKMYGFYDECKRRTRTSKTWKNFIDCFNTLP

LAATINDKIFCVHGGLSPDLHDFHQINKIARPTDIPENGLLTDLLWSDPDSNVSDWCSNN

DRGVSYAFSKKNILEFCDNFNIELIIRGHMVVEDGYEFFARKKFVTIFSAPNYCGEFNNW

GAVMSVTNNLTCSFELLKPKLHTSKSSGGSKGKRR

>model.g580.t1 Augustusgene.g580.t1 JFAV02000105.1:2915-5359(-)

MSAQHTDNTNEPNTASPVINVNTDNSKSVEETNNITESNNQSTSNENLEDQPSLSVETNS

INTDNFASTTPSNVLTNSKVVTNDTLIEPNTVKSTTGGGDKTIVNTIEPHENDQPDTLNA

PDKLSSKDGTAKDEQQAGVSEENVDASENLHADAQKLPEPNVLTSSEPDISNEVHGENSE

QPENNKLENDTVETPATNITDSQNEKENNVPKENTDSVLQENDGTNKEIELKKLAEIEPA

GEDNGGHEHEKKEVFEQEKKKENGEERQEEEEEEEKGEEKEEEEEEEEGETRCVCGEIDP

PDERGAYIQCDKCSTWQHQFCVGFKIDSELPEKYWCENCQPSFHTLYLNKKLNLINSRYD

PSVQTKTKGSSSSGKDDSLENSRSTRRSARYNDKNSEQEDGADFDQSKSKNHAKQSSNDT

HDNEGNHDQKQLVKSLKRKNAESNSNSPSKKIRKKGASEVSTDTRSSFRSRATSLAREEV

QYQMMLEKAIEESKKSSSSHFSPRRKTDDEENGEGDDTEGENEKKLLLTKELLNGVNAKL

ESDQQANADGINEGIDGGKNEALFSDTGKGTTHVGEKHGEDETEDGADGDEELDIEEDNE

EEEEGEEDNEEEEEEEEEEEEEGDNEEEGEEDETNNGTGRFVKPKRPSRSTDRRALGRTK

RMRNSKSAPGSPSARRTKSKRTASLSSSPSSSSTNLSSKYRANEKNITNKDNRISGKKSS

KEEIGLDKPSKPRLPTPNVTLGEMHKRVGAIMEFVSRTKKEIEELQSQDSELLKYVENQE

FIEKFKEFNNIELLNKMDVLTEKLLQWKENFATNL

>model.g581.t1 Augustusgene.g581.t1 JFAV02000105.1:6797-8110(+)

MPIKIELSQQEKNNKIQEILDKYTSTNTLDKPIVPGIVAGYATSNEIKYVGASGVLKHKE

SNKPRSAVDGDDSATDTTNKMQKDSLFLLWSTTKAVTVTALFQLTERGLVNLHDPVEKYL

PEFEFKKVIKSMNSETAEMEVEELKTKPTIHHLTTHTAGFGYFFFNKYYKTIMDVKSRGN

VLNCTYGEFDDVPLNFQPGTQWEYGTNIDFVGAIVMKVSGLSLEEYFQKNIFDKCDIHTM

KFNLVDEEDHPTLSNIHIRDTNDPTRFIQITEKLTPSTPEFQCGGHGMFGTVGDYLKFLQ

IFINDGVSPITGQRILSHETLVNHAFKDQLKEMSLQPGNENCHFKDLPSADATVTNPVSV

YPGVKKGWTNFFMINEDYIEQVNRPKNSLMWCGLANLYYVINLEKKLVMYWAESVFPFAD

KSSFIGFSEFEKFVNSEC

>model.g608.t1 Augustusgene.g608.t1 JFAV02000106.1:59604-63434(+)

MSAEQKDNSLFDLDFLENDTDKRVTNAPSGSNHEFPMETLDLDDISIENDYGDPFSDRNT

YTQNVSNQGGDEPLHRAQNNGFFKRLFGKKTAVSDSDNAYEMHTYSNIGSSANDSFLNSS

KQYDLKALFEKYLLGRGDHDSVSLEPRTILINDAHLNSHFKDNHISTTKYNFATFLPKFL

FQEFSKYANLFFLFTAIIQQVPDVTPTNRYTTIGTLTVVLFISAIKECVEDIKRANSDKE

LNYSPVEVFDSMLKGFVLKKWIDIKVGDIIKLTSEEAVPADIVLISSSEPEGLCYVETAN

LDGETNLKIKQARPETSKFLDVQQLAQMAGQLVSEAPNSSLYTYEATMTLNGKSFPMSPE

QLVLRGATVRNTSWVYGLVVNTGHETKLMRNATATPIKRTKVERVINVQIIMLFGLLILF

SFISSLGNIIQITAQSKNLGYLYLDRVNKVGLFFKNILTFWILYSNLVPISMFVTLEMIK

YYQAWMISSDLELYSEETDTPTIVRTSSLVEELGQIKYIFSDKTGTLTRNIMEFKSCSIA

GKCYIEKVPQDKKATMEDGVEVGYRSFDDMFKKLADTFDDDSAIINEFLTLLSICHTVIP

EFQDDGTIKYQASSPDEGALVEGAAKLGYKFLIRKPTSVRILIESSGEELEYELLNVCEF

NSTRKRMSAIFKFPDGSIKILCKGADTVILERLGEDGMEHVEATTRQLEEYASEGLRTLC

IAYKDISEEEYQKWNAAYEEAATTLDNRAEKMDAQAEIIEQNLILLGATAIEDKLQEGVP

ETIQTLQEAGIKIWVLTGDKQETAINIGMSCRLLSEDMNLLIVNEETKEDTRKNFIEKFQ

AFKEHNISSTDLENLALVIDGKSLSFALEPDLEDYLMQLGKMCKAVICCRVSPLQKALVV

KMAKRKTKDILLAIGDGANDVSMIQAAHVGIGISGMEGMQAARSADFAIAQFQYLKRLLL

VHGSWSYQRISQAILYSFYKNIALYMTQFWYVFANVFSGQSIMESWIMTYYNVFFTVLPP

FVLGVFDQFVSSRLLERYPRLYTLGQKGQFFNVTIFWGWIINGFYHSAITFICSILFYKY

GDSLNMHGETVDHWTWGVAVYTSSIITVLGKAALITNQWTKFTFFAIPGSLIFWLVFYPI

YAAILPHWDISYELRGTISHCYRSATFWLMCIVVPTFSLLRDILWKYYKRNYNPATYHIV

QEMQKYKITDNRPRVEQFQKAIRRVRQVQRMKKQRGFAFSQSEEGGQEKIMRMYDTTQKR

GEHGELLDASANPFRDM

>model.g585.t1 Augustusgene.g585.t1 JFAV02000106.1:371-1546(-)

MAINTNDKFATLAVHAGSHIDSHGSVIEPISLSTTFAQSAPAKPLGEYEYSRSSNPNRKN

FEDAVAALEKGKYGLAFASGSATTAIILQSLPQGSHAISIGDVYGGTHRYFSKVANTHGV

ETDFTNNLLEDLPKLIKPTTKLVWIESPTNPTLKVTDIKLVSETIKSINKEILLVVDNTF

LSPYLSNPLTLGADIVVHSATKYINGHSDVVMGVLALNDQVLFEKLQFLQNAVGAIPSPF

DSWLAHRGLRTLHLRVKQASESALEIATFLENATDKVVAVNYPGLKSHPQNDIVQRQHRN

GLGGGMISFRIKGGAEAAAKFSSSTRLFTLAESLGGIESLLEVPAVMTHGGIPKEAREAS

GVYDDLVRLSVGIEDTEDLLEDIKQALEKALA

>model.g590.t1 Augustusgene.g590.t1 JFAV02000106.1:11230-12045(+)

MEYAKLSYEIDKLKNYVEERNRLIDVLRIEPSNKDTILVKKQLNYILQLIDETPDLQQAE

KYNEILASVSTEDTHFTKEQKGLYHYDTGKLKREEELAAKVVLPEPKDLVKKVRFSDHND

VKENYDNSQQLTKLPDSDKGSQDISNIVNFAPYKDAVPMEDDQSAYSMMSNDELLAMQQD

TMHDQDTLIDSLSTTMHRTHEISVGIQEEVHYQNDGLLVDLEGMVDATANTLNKAHLKLN

KFNKMNRDSGGSYCRGVLIFVLIILLVVLFAL

>model.g594.t1 Augustusgene.g594.t1 JFAV02000106.1:18002-19372(-)

MDMSQSLQDLLSSDKFDNPPPPDFPCTPPNADVNGETITHSYSAGITQDNDEDQKSKPSS

IAFQQDGDSIAYSECTKLYIVGDFINVLEKMESLGKFIEPGVLLSVDVDDLSALSFSSIE

KAKKWDTLFFKTIISMDITELSKSHKDTLISLYDLVFLYTNTLFHAIVQDSVKQSLSGNK

QVTKFTIEYIFGLIQAYYVLAFNATTNYQQLKDFSKIVQGYIKYTSKSDLTESQDDDTFI

YYLYKMCEFYIFELEINGLHKVPNKGLYHDLISSNKGASNVAGYLKGTKYESAILEKLEK

KSDDTTANTKAGSGFGSTKLKRKDTKSGAHFKLHSVSPIRYSHTREHDIDRGIVDESEPL

ETKKNKDNNVLKKLNYSVSLIQDKLLNYSNNAKSFYSRMSALNKTVFNSSIVFSLVLLLV

SIVKYRRNKKLRTSALSSTSGSKFKLFEMLKILAKLL

>model.g591.t1 Augustusgene.g591.t1 JFAV02000106.1:12287-13207(-)

MNYSGPNGDISHQGLEPPPRQSSTHQQHSQTPPQQSTDILPQAASSYMHPQAMANGTHNN

ANNNASNINAPTEVSAEVVQQLNIKMNELNNYRSFMAQSKLFLDADLFYTPEMNINHVTP

YQQIQDQTLTPHQKQQHEIEHQQLKNNELLEELQMFKSLRQVEKSAGRLPPHYVPSATQS

LRRVLSKKDAQAQAQMQAQTQAQIQLQAHAQAQIQAQAQAQAQAQAQAQAQAQAHAHSQA

QPHQQLNNMSRMGGTTAGLNGVPTVDHHSGASHFPFLQQQHKQAPQQETFGTFAQQQQQH

QKVAYRL

>model.g612.t1 Augustusgene.g612.t1 JFAV02000106.1:69518-74407(+)

MSTTKTSYDVGTRCWYPNKEQGWIGGEITKNSFDEASGLFSLELKLENDEIVKIETKNLE

SVEANLNATTSTDSPSKETLPLLRNPPILESTEDLTSLSYLNEPAVLHAIKTRYAQLNIY

TYSGIVLIATNPFDRVEQLYTQDMIQAYTGRRRGELEPHLFAIAEEAYRLMKTDYKNQTI

VVSGESGAGKTVSAKYIMRYFASVEDLHNNPDQSMNSQLNTQNEDSDLDIDGIPSVDGIT

SETSSTNSLNFGPEMSETEEKILATNPIMEAFGNAKTTRNDNSSRFGKYLEILFNENTSI

IGAKIRTYLLERSRLVFQPKSERNYHIFYQLLSGLDDEDKEKLSLKGIDDYKYTNQGGEP

VIAGVDDKEEFSTTVKSLKLVGVNKDTQFEIFKILAGLLHIGNIEITKARNDASISSEEE

NLKLACDLLGLDPVLFAKWLTKKQITTRSEKIISNLNYQQALVSKDSVAKYIYSCLFDWL

VENINVTLCNPEVEHQIKTFIGVLDIYGFEHFAKNSFEQFCINYANEKLQQEFNQHVFKL

EQGEYVKEKIEWSFIEFNDNQPCINLIENKLGILSLLDEESRLPAGSDQTFAQKLYETLD

KPPTNKVFSKTRFGQSKFIVKHYALDVEYDSDGFIEKNRDTVSDGHLEVLKSSTNKTLLE

ILSCVDRAAEKLANEQEQQSQSQTKPVGRLGSARAANKKPTLGSIFKNSLVELMNTINST

NVHYIRCIKPNEEKEAWKFDNVMVLSQLRACGVLETIRISCAGFPTRWTYNEFVARYHLL

TPAKYWENLYSTDITEEDIKNLTQKVLEYTDTAPAKYQLGLTKVFFKAGMLANLERLRSD

KLHESIVKIQKNVKKVYYQKQYQQILSSIKVLQSSLSAYYVRQKVELEIKTNASLLIQSL

LRSSLVKTKYNGIIDSVIVIQSLLRMQQSKKLLIKQKELDSAVKVQKNIRSFQPRKAFLV

QQKSGLVLQNLIRRRQAQIKFKKLKSEAESVNSLKEVGYQLENKVIQLSETIALKIKENK

ELNSRVADLQEKLLKNENLKEVLNLEKLNHSKELEFLQKSKETNDSEYQQKLLKAKEETD

KVIQEIDSIKAERDQLKKDVQDKLAELQEARDQLGNSKTQNSDLQNEVNSLKEEIQRLQQ

SLQLASTSSASVKANGVAYNDGIQETPKSNRLSISATDYIDPNTPGAYSDLYDGDDRSIN

SVLSQINDELYKLFDDTKALNKEIVFGLLKDFKVPEAGVAVEMSRKEVLYPARILIIILS

DMWRLGLTKQSETFLAEVLSAIQNIVTNIKGDELIPNGAFWLTNVRELYSFVVFAENSIL

NDETYNSGLTDDEYEEYVKLVTELKDDFEALSYNIYNIWVKKLEKDLEKKVVSAVVLSQS

LPGFIANESSQFLPKIFSQPIVYKMDDILTFLNNIYWSIKTYHVEMEVFREVITTLLKFI

DAICFNDLIMRRNFLSWKRGLQLNYNVTRLEEWCKSHYIQEGTECLTHMLQAAKLLQVKK

ATLEDIDIIWEICSSLKPAQIQKLISQYSVADYESPIPEQILQNVAERVKKDAAENPKKP

SSSRSGGKSHSSDIFLTVNTGPFDDPYATLDTREFGKIEAYIPAWLNLPITKKIVNLVTS

HVKVQESGNI

>model.g609.t1 Augustusgene.g609.t1 JFAV02000106.1:63523-64863(-)

MSAAEQIAKSARKAGNVLKTIDDEHRSLVLQNIHDALKANKDLIKAANEQDLKVAAENNL

ASSLVKRLDLFKSDKFDTMLQGILDVANLEDPVGKVLMARELDENLTLYKVTAPVGVMLV

IFESRPEVIANITALAIKSGNAAILKGGKESLETFREMSKIINTCIEESFASTGVPAGSV

QLIETRQDVSDLLNQDDYIDLVVPRGSNALVKNIKNNTKIPVLGHADGICSVYCDSEADL

EKAKNITVDAKTNYPAGCNAMETLLINPKLDNWWVILDNLIANNVTLHVTKDVKAEYFKH

ASDESQKKVVDVDESKDFDGEFLSFDCAVKFVNNVSEAIEHINAHSSKHTDSIITENKET

ANKFLKGVDSAGVYWNCSTRFADGFRYGFGTEVGISTSKIHARGPVGLDGLVTYQYQMVG

NGQIAGDYLGAGGQRAFVHKDIDIKSL

>model.g619.t1 Augustusgene.g619.t1 JFAV02000106.1:92146-92649(-)

MVRFKSRYMLFEILYPPTAEEYSTFSEEEKFAIEPALLQHHKVTPPDVNNRVLVYEIKKS

LQYFFGDYGSGRGTSMFQMKYFSNSTSTGILRCSKDDYEYVLMTLNMMKRIGSLENVIIN

VVKVSGTIKKLEEFAISRSDELMKAFHQDKKDFLQSMSSIPLDENDDI

>model.g600.t1 Augustusgene.g600.t1 JFAV02000106.1:33633-36896(-)

MANYGNDTDLDSSPLVSPNKQVSAYDTDIKNLKNEFPTFSETLIRAYYKTNGYSYAIARK

KIKGIVEFKKQKMKLSSQEKDTNIFPKKSPVKKETIFGKYKFNIQKYQNENGSHLNEDMP

VVDTGSDADIGDSDEEEIISFARSRKKQPLNRFGYNKETVASSAPYSNKKRKLVRSDQLE

RTKPKKALKLESDEGYYSEGTDEDQQDVDDDAEDEEYQTIETSKKPKETDILKFMNKANE

KDIADIADIHIAQALEIIKNRPFSSVKLFKSMNFPYDAGNGQTKKSNGKRKARVTKTDGE

KIFDKAHQTLEGYNAIESLIKKCSKYGDLIANEIKKWGVEVNQKKGAATDGALDDGPGLD

FLVVKHQNDVEDQEEEQPKEDEKADANAAGADVKTDETNDVKQDPSIGTNEQEEAKEDKE

EKEEPKGAERADQAIEAEEANEADEANEADEADEDFVNMSDENVSDNEDMDDDDYDENFE

KTSTSKTEDVTFTNSLDASGPTSTKFFTKPPKYLDTSIQLKDYQLSGINWLYLLYCNDMS

CILADEMGLGKTCQVIAFLAYLKQMKAKGPHLIVVPSSTLENWLREFAKFCPALKVQPYY

GTQNEREELREILDDSSQYDVLVTTYNLASGNKYDQSFLIKRNFNCVVFDEGHMLKNSLS

ERFDKLIKIRAHFRLLLTGTPLQNNLKELISLLNFIMPSLFTSQKENLATLFKQKTSTTV

DKGYNPLLAQEAIMRAKVMMKPFILRRRKQQVLQHLPPKHIKIVHCDMLDHQKSVYDKQV

QEMIKNKFDVEDSEENSKTTKKDVAAKKQSNVNYIMALRKASIHPLLFRDIYNDNIINEM

SFNILQEPRYIKDGNQQYIKEDMSYMNDFELDNLCHEFPATLKSFKLKEDEFFQSGKVKK

LLSILNDVCVVKNEKILVFSMFTQVLNILERVLKHYKYQYLRLDGQTSVNDRQSLIDEFY

ANSDIKIFILSTKAGGFGINLVAANNVVIFDQSFNPHDDAQAMDRAHRVGQTKDVSVFTL

ITKDSIEEQIYKMAQNKLQLDSHISEDQKKESQKPGMKVTAGVSNKIQNLLQDMVGVPPS

SSTASQDL

>model.g611.t1 Augustusgene.g611.t1 JFAV02000106.1:66519-68639(-)

MGDNLLSEASKTKENIEYKPAGYSTSQSPSNADISNIYKKDVSNALKPPIVFKSNNNSIP

SLKNYEGHNFGTYKPVVNRDYLNSSKSFSIHQKTNTDINSNTAPKPYRNLEMDNGGLANS

KNSNTSASKKNLLDLLGSKDSPGKSHQESLHLNPVGVNTNFLNPDGPIRRGSVSSNSYKR

NTECYAPVSNLSRLKNLTSTSKGSTFSDMVFNQNDIGLVPESMGIASRSNSNNTSTNRIA

TPVPPAGTPVNESSTKPALKKTFSGGLGTQKKYTMNSPPNFHKRNISNTHSAAGSQMSLP

HAHSHMHSSAHPNASSLAEDSTASFSHLNHPNPSQTPLTFIPNQREDSSKLVQQFYHDDS

LYNNVDIFGSNHSDSSLKRIISHTGNNDLMNARSATLNPTFGNSSRSMSNTQCEFDDEEE

MKMIKQSKHIMDDYRDDLLIPFVSQTLATLNSIYVQKHETHSKHYLLRKEKEDSATRRHH

SRSNPVPATNPNGAISYDYTNTTALNARKEAEQSNSKKLNTFSNKNENYGVLVECSVDDE

LYKFNNVVLKKLSVSLRSERLHLSKFHDLFQVLHIEFEKTVDDMQVLQENLVSGLQELES

LNQSNIAYYENTMLSKIVSNNEKLYYFKMKTTKNLEILKSLHAKIQTLEKFNKLFIEIDN

FYNDMGVFQRIRFWIKPYNRYFTSLGFFVDLAVFAIILLYYYRARIM

>model.g597.t1 Augustusgene.g597.t1 JFAV02000106.1:26663-27754(+)

MSFQVIIQNHKSYPNALKVWPPVDNLQNEDNDKTNVNLKSTNLHKNKAAYKKSTESKALA

IKNMLRNVSSYTMKEISLVTSNLPKDFFFKMTYLYPIKGLIEFLTTPLYWKFLLPMVYPY

FLLTTVVGIIYYFLIFPIILANLTMTMGIIGFIIAHVHFLLETNAVSMAIAKIILFPSTN

AIFFNLVLENHKQQDYLVEAKKRFISITAFQKPENHGIFWNVWNHVLGDDIYIVFSKNKH

DYHYWVHVAPLQILSLVYKGVRFCFLFALSLIPIIGPLLVLCLTSPDRAQNCLQEYFLLE

NYSKDTINNVRRANYGQFSAFGLVAGLLEAIPYLSLIFEYSNLNGAALWANDLIKKKTTF

KKKV

>model.g616.t1 Augustusgene.g616.t1 JFAV02000106.1:83484-84185(+)

MSATAGHPLNDTQVNEELNKMQTFIKKEAEEKAKEIKLKADQEYEIEKTQIVRGETIAID

TAIASKLKKATLTQQITKSTIANKMRLKVLQAREEILENIFEESEEYLKTKVSSDKKKYG

PILKNLILEGLYKLLEPEVLVKVKEEDVKLVESLIPDLVKEFTSATSLDSVSIEISKKDF

LPKDIAGGVIVTDATGKISVDNTLEERLKLLREESLPAIRLELFGPSESRKFFD

>model.g614.t1 Augustusgene.g614.t1 JFAV02000106.1:75751-78336(-)

MSYEWLDKPTIDQTVPSYFNPSNQNATASGNSQHGFPQSTYTNQNQPLFQSAQQTSYQAP

FVSQTSHVPQVPQVPPRTNMGTLASDIFKNPTYSSPNQPSYTQTTSHKSDFAQLPKEPNA

AAIFNYFSNSPASSNLNSQLPSKPLNVNNPFRSTNNNTANSFALQNQKTGPAKLPLRETN

TFQSSRSEASLYTINSTPGQPQNPEIPLALSTYQLSLEESKNYLRWYQYISDKSNFKSYV

TLQDIFNFLKTNFEITDHIKKVVNNFFKNIKDKIGLENFYAVLRFISYLLLKGQIPTKDM

LLLAAPVLKPKSILSKGEKEEVYEEIEDSQNDQPFDFDNFASLLLTGKSHRVKRVVKRSD

NRPSKNVRFSEKLETFEKNHAPEIESPVVGTYSQSVINNLSTSSNDQKNEDSKNEPLDFS

LPMDQLLAKLQQSKQPATKEEEEELADMKESLTHFQNLPKIDSVSLHIPSRNYQNSNEIE

PLKPTATGSANHLFGQQPLQPTATGSANRLFGFSGNKSGLENSTMEPLKPTATGSANHLF

SNQNGFSSSNANFNSSNPTTTFGNFESNTLKPTATGSANFYMKHSNDYIQSHPPTFNSSL

PANNTGNLSSNANGYLNQVQLSPNHTNNGNFAHSSNFSGNTPSVFYDSAPNNVNQNNNVQ

NATNEYNFNLLNKNNMAENSSSGLQTNFSGGNNSSGRPFSNVSQQSSTNQSNVQNNNKAS

DYFSMLLQHNDNSSQNNSGSSLHLPNSISPPSLQQSPMYQGGFMQQQHTVQPQFQPQQPQ

PKQNTGPLGYQYTGYTNSSFQPNGTPQLQPQQQQQGFSSGYQSPNSTFQGNRPRTNSTAI

LGDMSQMQQQLNNIQNNRYGNA

>model.g587.t1 Augustusgene.g587.t1 JFAV02000106.1:4920-5345(+)

MAEVEQRKKRVFKTYSYKGVDLADLLTMPTEEFVKLTPARVRRRHSKGVSHKQVNLLKKL

RAAKLAAEENEKPAPVRTHLRNMIVIPEMIGSVVGVYNGKVFNNVEIRPEMLGHYLGEFS

ITYTPVRHGRAGATTSRFIPLR

>model.g595.t1 Augustusgene.g595.t1 JFAV02000106.1:20111-21682(+)

MSYLSLPVSNNESGSSLDKEKNEDDQPHLLNSKQVTSLIINRIVGTGIFTTPALIFQMCQ

GSIGISLLLWLIGGLTTFSGLAVYLTFGLKIPKNGGELNYLNKVIPRPKYLIESIYAFAI

IILGFSSGNCFAFGKYILYVFGVESNDDLSRIIGCGVITSCVLLHIYSPKWAQKLNVFLG

GFKIFILVVIVLCGVISLIAPTWITPQYSNFHNIWGTTTTTNSTTNSASQSTKNSIDWYL

LSVALLQVIYSFKGWENANYVLGEIENPHKTLISATSYGVGLTTILYFAIVLSYYLIIPK

EEISETGVLICGVFFTKIFEQYYAQSFAVKFVSKFLPLIITFSNYGNVLSVSYANARVNK

ELANDRVFPFAKFFAKKSINASLWLHCFITCAILIIPRNNTNLYELIINLYSYPGVILNI

IIGVGLLHLTYKNIDEWNEIKVPFTSNTLLTGFFIFTNVFLTVVPLIPPSKKSLDFLQNQ

GYPYWVFPVTGISILSMGAIYWVLAIKPFQKNLRKNEYQTLLVK

>model.g618.t1 Augustusgene.g618.t1 JFAV02000106.1:85905-92113(+)

MQKFSAATNQNLVSPFASVHTNIRGLTTASALSQKEQSLGTLRHQLDQYTLSLNPLKPVV

PNDLFVSCTIMKKNGTVVGISQKFPKWQFLKDHDLYPRDLRKIDTSTIDNIPSIVVKPNK

CILVNLLHIKALIKADEVMIFDTVNPVAAHGLGELMYDLEVRFQHSPAQITLPFEFQVLE

IILINVLNSLEIQLNNLEKQCGGILKDLENEIDRNKLKDLLIRSKNLSAFYKKTLLIRDV

LDEILDNDEDLQGLCLTNNLVKHPGNSTNESHGNLLESAEDVEMLLETYYYQCDEFVQKA

ESLIQNIKSTEEIVNIILDSNRNSLMLFELKITIYTLGFTVATLLPAFYGMNLKNFIEES

ELGFASVILVSSLAGIIITMTNFKALRSVTRLTLMNNHSGKNTKTHIDNAEMKTQKFVPT

VSSSLKNWKRKFHYLMKGENSKIAQVDRITSKREAIRQWLIQHKTDKNAARAAASASTPS

IQGGNIFDHLDAKNVQFQFSVDHLKQMLELLNAMHDSIISSENHQNIVPLTSLISTLCSG

WFFNVNSTLKKRFALILDGKLIKLTTIKPILSTQLVEYSYEFLSRAESEKEFDFDKYKNK

FYNFNHQLKSLESLISIIHNALEFYEVKLKQIIMERSSNRKPDASGVAPVIDHQSLAEFT

HVDETRFSLDLAVLVEDKVKDTTDESFRNLQLQVLKKTLDVVKKFHDGTLSDFSKTMAAL

YNKETTVSQLPYWQFTLHRMYAFLLRCIQHCTFVAEIMKQLYHPSKAFFNNNRTKLFSKN

LIEYEHMLGLLDDTTDVVFLENLVKDFCANNVIIFFQTNTITSLYSRYIFEVEKCIEYRH

SVLSLWIDHWEYLEKNIGENLEEYKDIESSTQLLKLVEERRVNDRITCKKPLLRNMVNTK

GPAQGTEFKAAILRKPYRASNSTNKNVLRSGSTKKGSASSSATSSRNRSKLASPQANSNP

GSPRPGPVHAPFARAGRSRSSSLQHAKHTTNKESFNMKLAAAAAGLSTNMIGNDSAQKSH

SLQLASLETQAEIQKKYANQSISSHQPLKPPPPSVLMKKSVSENSRGTRQETSYTSQSEL

IPKLANVHLNDETRRSSGKPSRVNSKRQEGKSRYTSSSESKQSDASVVVSDLDLRKPENS

ENEEGYFDSEEYPHEIIVKRVRFTGVPPFSKKEDPAPTRRG*PLSSLLPKLQNEKRKKNI

NNLNNEPKETVVIPTFMIQREKHLLQKMMLSSNGKIEKNEEPERDKHETNDHLLQYQKSL

LPLRSVNPDYKCPLPTKEAIRECTEATKKTIEKLLGQNAPAALSNTSSSAARLINSNEFS

HTVPNVSNDKDTETLVKVIERHKDPLAPSTKKILKKIPLADDNTELLETPILHSENASGQ

KLGEKEKALLNIPSAVSSWENSKGFSISIDKRLQYTQSDRKNVPKEINNKFVELTKALDA

TSEKKRIELAEKNERLLKAKEDQEEFEKIKEKIAKTKSLEQKETQRNSRYSQHNQVGRRF

GGDNREAIRAQRRREIQHTSRFNNKPKQAPTATLDQRDMQTASVSVNDESENEVQYDSRF

FTRAADATWKRSEDQMFDTPLFRQRDETVSSVYKNRRAGKRTERDADNDVVHLKETPVEF

VSAGTKDEPRQHNAGDSHFANSNKKRKTNE

>model.g589.t1 Augustusgene.g589.t1 JFAV02000106.1:9365-10846(+)

MASSSLKNLFGNSTNVSSTQSIMQKLAADRQKSGNKSTIEKDLAKVADAEGESDNDSSDS

EEASDLEENEEIEEPAPKKLKKRRNDNDDDIEGKYMAKLMKETEKQEKERKSEQPVAAAQ

DSENKDESDSDSDSDKEDAETAAEAKPASSIDLKEKELEKAEKTIFVGNLPVSVLKDKKA

YRRFKKLFSTNPHKPDDIVVEDTKEEKEEEEEEEEERSGSAKESVNPYTIESIRFRSIAF

SEALPRKVAFSQLKFDEKRDSLNSYIVYKNKNVKKLNQLIKTLNGTVFENHHLRVDSITH

PAEHDKKRSIFVGNLDFEENEESLWKHFSQIGKIEYVRIIRDSKTNMGKGFAYVQFNDSS

DVNKALLLNEKPISSTVAKKARKLRVSRCKNLSKQGSSNDSTLSESRNGTLSVKANLNNT

QKTKIGRANKVLGKMDKKTLGKQLTIEGERATKIDKNVNSVLGKRKRKPRSKDGRAAKRS

AAHKEKLAAKETKK

>model.g599.t1 Augustusgene.g599.t1 JFAV02000106.1:29820-32795(-)

MNTGDWRSSFSEQERAKYRTSLAQLLVQISKTNNGKSADIEKLKKTAADFEQNLYDSSSS

AANYIEAIKKRIELTANAKQNAQKQQLQQQAIAAKNGGTAAGMQDPRSPNNGNANNNSNS

SSSSNTKNYAMNMVMNQKALMNQNLQLQQQQQQQYQQPGAANAVPNAAPNGRQQSSRYHQ

LTPEQQQLLHEIKYTPIPRELLSRIPNLPPNVNTWQQIMELQLGDNEITKKIYRYHQQML

LRDRQKNQALANANNTNSTNKAGTIAESSSAPQPQQQQQQQQLQQFQLLQQQKQQRLQLL

QQQKMQQQQQDNSSNKTANGASFNNSNSSSSGNVSSANPSASQAARFISPEQQKALLENA

QLLIKKLQKTGRLPPKLTAEEYQTFVKRYLNEVASKRINQIRQQQAQQQALNKAESGTNA

NMNLPNSNMTAIQAAALAQQRRLQQQQQQQQQQQSNSETRIAGGSNTLPGSAAVSNGTTP

QPNPNMLPMNNLTPQQQQQLQAQYLLQQQKAQQQNMTKQQQQQQAVAAASAAHRTTQSSS

HTPGQASQASTQSVASSSSGPAVNILSKIDELFNPQEQKMLYENAKNAIKAGQQQGKLAA

VLSTQQQNFYIRKYVQQMCIKKLAAMKQASAAAAAQKLQQQQQQQQQQQQPQLPQSQLQQ

QEVVPQSSVPQQADSTPQEANMQARKLSHQKSVSHQASVSVPTSAVSKTKGPAKAKGASP

SVGRTSVSAASPLQGAATSNVKASSGNYVNPFKEDEQALRELLIKKREITARHKKRQEVY

CYSEADIFLGTLSDCLGLFDEPHEAITPIQPYIVDFINNTGKKKLTKTLLKARDQEMLVT

SIKDNNKLIMESKNNKVMYTKSIFDSDGGLRLHSNEIGSLFSFLDCENPVIHNLSNDENP

RMETETTKLTEKISNGKKRANEIHEDANNKKIKSGSASTSPEDILSSASSTESAISASTT

VDVKPNNINGYEKANLESDIWDYNFWQSLTHD

>model.g586.t1 Augustusgene.g586.t1 JFAV02000106.1:2397-2726(-)

MKYLAAYLLLNAAGTTPSADSIKSVLESVGIEIEDDKVSSLLTALEGKSVEELIAEGNEK

LASVPTAGPAGAASGASGAAAAGGADAAEEEAAEEEAEESDEDMGFGLFD

>model.g601.t1 Augustusgene.g601.t1 JFAV02000106.1:37399-38751(+)

MPFICIRCLGSNGNYQLGLNHDEDIDTPETTLRCYIRPEFVSQVDKTFLHRQESNLSRES

MENCDLHRLGFTSGSVFPVKIACGGNHTVILLSNGAVIGTGLNEQGQLILDSKVPENKVL

KFWRVIYDDPTHPAIDLACCWESTVVAVLNQPHTTGGDFKSINIKSFGTGMKGELGLGNS

TTFNADHSRNVLELKNSSKISLLSSMANVIVLDKQAKMCYGWGNNTKNQLLVPNANSHSP

ANTDKLSKKRTKIVWEPVQIDVVAEFDDCLYSLGKDFLAWISRENTDLTFEATGSLDVQL

SSQKISKSKVDSILSANSNFQISKMEGMWSSLHIISKQKNDTVKLTSLGKGRHGQLSMNG

RLLQQYKDFSCGSEHGIFCNKKNEVYCWGWGEHGNCGMRNPGLIPDGHRGKITQDTTVWE

NLNLVFRAESKEHVLFVQGGCATTFIVSIAP

>model.g615.t1 Augustusgene.g615.t1 JFAV02000106.1:78864-82778(-)

MRISNSEHIQLSQAIVRSVLRSQCFLIPRRNLSNVNKRNFSFSTPALFTESKPDGAKDSK

VKLNAFGIQYLAPSLKSQVFISGEKSKQKKPQDKPLTASASRILDDEPQFLNKLAVKSLK

NHNLYGKKTSINTPIEMELPKLQGSSLEEHFQKLGKHMSEPYLSMAKSKFTNILPKPDKW

EMESGAWIRYAPGFEPEKVDFPMEDLYVFDVETLPKISPYPVLATALSDKAWYSWCSPFL

TNDVENSTDGFEHLIPLNTQDPQRKQLIIGHNVSYDRARVLEEYNYKQSNAFFLDTLSLH

LGTAGMCSRQRNLWQKATKLLRNNDKLEKKLAKKEGSETRMEEDNENDEFDDELIGLLPQ

SGGSSINEVEADDILKNDPWVKVTTMNSLQECALFYCKIILEKDSRDVFVTSTDKRDVIN

DFQNLCNYCADDVVATSMVFDKVFHNFLQKAPHPVSFGALKMISQCFLPTNTDSWTSYIK

HAEDLYQNSKIDIENKIIDIVQDLVKLKDSPEVFSNDPWLKQLNWTIKPLRLTKKGAPYK

NQKLPGYPEWYRNLFPSASSEKPVITIKSREIPLLFKLSWENKPVKWCNDDGWCFDVFEK

EEIEVMRKKSYQEVKKTKDCEFTGVRFRVPHPNGPDNRTTTLLSKPFVHYFEKGVLTAVS

PIAQKALQINASGSYWMSARQRIMKQWVVSRQSFPTEFNFQPTDGDGQHGATKSLNLNSN

VGDRGMIIPKIIPMGTVTRRAVENCWLTASNAKKNRIGSELKSLIKAPEGYSFVGADVDS

EELWIASLVGDSVFKTHGGTPVGWMCLEGSKSEGTDLHTKTAQILGCTRNEAKIFNYGRI

YGAGVKFAAQLLKQFVPSMSDKETVDVATSLYKETKGKKITIPKVGTIWYGGSESVLFNK

LEEISDQDIPQTPVLGASITNSLLKGNLKKRSFLPSRINWVIQSSGVDYLHLLCCSMNYL

IEKYQIDARMVISIHDEIRFLVNNKDKYRAAMALQISNLWTRAMFCEQLGMTDLPQNCAF

FSAVDIDFVLRKEVDMDCITPSNQVPLEHGETLDIYKLMSMDNSALGKPVTDIDLSKYEY

EPRAKVVETLKKQLDPLLNKYLIATEVQNQKTVADALIKEYKENQVQSKFVNEYNKLLDE

TIDFNDPLRKKKIEQPEKETQISFEVLEKSLEHKPQKQAKTKTTRESEFLKEAALIEEPL

KRRKTARTKMFKENRHSVKQVADKKMLPEESILNEILQSSYQSALDLGSYPTTGLSTTGK

MMSTKNKKKFSQYDKFIQNQKEENSHLNTLRSSNIPNPKTTTVSS

>model.g606.t1 Augustusgene.g606.t1 JFAV02000106.1:52063-57789(+)

MATVPRKSVLSEKKKNHFLSSIRNSCTTKDEYESEIERELDHYRIYYPVFEDSNYYPKPT

KNVMEIVSKGNSSNDSGKYSIIKSTVEGILIQFSNPVDPINYQQFQDFFIIYRVFLDPLK

LLDICLSRLKWCIDKIQEIIKENKLSYLPASRNVSNDGNPNVASRYKSPVPFAIDKMEVI

LIRTFVLLRHWILNYYIQDFMNNESLSLKFIDFMNSFDYNSTLKRKIVLNCLKNLKKSWI

DQHNKVFDDVEHKININKLHSEKDWLNFRIPVDFEENIDESENFENVGYENNKQTRLSFY

ALQSSSNPAFRNQNLLSTYGAQQIFKIPQQEDKSLANKLNKISKRQSKIFNILFPQDYNN

AFSKNDRAMKGMSDTSVNSDQELHNANSSKRQTYSFNEYTNNSVNSSIIGNIIQSKNSNV

LKHLHTNYPESSTISKLAPLTPAKNLEFTISLDEMSKNSDMNEELSAKNNSPTSFKSKEL

QENSSANNIPSNNSMLENQQVKKFVKYVFSIGTINVPNNDSDGTKDEISEKKFDILGARS

IDQVQFLIDMETYLLNQLEKKTTPRNSLNGMDDYANDSSLTDTFPESPGIVKNYNMQETP

NTVNGMDNLNLYQTVTSIANSVLSSARKNQIDLNESLLTNSVTKNFYFDHHLKNESNSKT

VQNDPNGDDENQIPSHLKISPFLRSLDKSNVYNHRRVSSSFFSNNLDTPSKSSKLKTNSA

TLKLRPLNSNDDFTSTSFDSDSNPANLTIANSSPSRSNRASARSSLKKKQTLKMSDSAYD

LSKHISSITSNGETESNSNVSGSINLSNEFNILHESNDVISTRSNVQHRNILQSSLPGIP

KNKTQNNRLSKVNANHLSKVSKSTTSVAYQPKALNSGRISISAAPPTARTSRVASSSSNP

YRSPTLGNQFDKGHKKTKSSEARYGDLFRNDLSSNANAQVRHKSADFNEVDYNRANKISR

YPSVFANPSFVSKDQFFEEQALEVKILQRKTSRITLREAKNEGQESIKEEYETYELNAPP

SHLSNRGVFQGTPSSEEHSLNTRPDKFSKLNGIPQITEYNENDCEENKPEFESKKSSAHA

SLSSSPRSEKSFATLSSINTNMIMGSPNASESLRRSYPNYTEVEGLQVSEEFVGKTFSNL

LSDKSNKPMESKRLTSPVSPGGLFLNGAPDVNRISTFISDSDDDLSLRMSKTSLSTYHKS

QNKEKRDTFNETNAKLDLRRQFHQQHEQSQNSNNESHSGNGARFSYMVYGKNTPEPMPLG

IEQHQPKLYGAVRDDEYNDSFKHNLQQELDSTSSSLDFSPIHSSPEHFDSVNGIANGSRP

SQANRKSISSSPFKTQNTNSVLYNENASEKLSPSHSARSSQISKTLSPSKGNNPYFIVTS

TSYEDQQDQRKTTLSTQSNSTKGKKDSFPNFTMINGKRYSNVSANSPSPNKAELLRQKYL

PSQNSASSLSGLNLQKSNESLDDTRDITEASIAENNNAGRKKSHSLQSNPKMLELANLPD

KSFSGVDPVDAAMRKLEGAYSTSNDSSMKLKSILKDSDKPESSSSLTQNEAEQAKESAIV

EDSKEMQMPNLKKRDDKKRQTVLIEARRSTRLFNVFDQSQKSSKSFLARGSHVSALQQKE

DTKVDGVLVNEQITDLLKHYRLTDPNLLLENSDEHIPFILMYSSYDIASQLTLIEQDVLN

EIDWFELLELNMQSDLPNFTSWLEVLFQNEKLSGIDLSIARFNLMVDWIISEIVLTADTK

MKRNTLQKFIHIAEHCIKLQNFNTVMQIVLALGSPLVQGYASSWRLIDPGDLLTWAELQK

TFSVENNYSEVRDFMNELDPIHGCLPFVVLYLGDLISLKEVPNHFHSNSQIINYSKFDNM

VNIIKSFIQRVQWGTEFYQIKTIPQLLSKCVYISCLSAEEIELVASSSG

>model.g617.t1 Augustusgene.g617.t1 JFAV02000106.1:84783-85403(+)

MSLKPQFFKSHVLTAVSAVQGTTQAVPPNLVELYLDYNCPFCFKIFKKWDDAKLFDKETL

TKYNLAYRFNHVIQPWHPFGTLVHELGIVAGLLKPEAFWTYSMVLFRDSKELWSEEYTQK

KTTIELYKELSEHCEKHTGLKADIVYDKLNGSGKEETIRAVKYFTRYHRQNGVHMTPTIA

VNGIILGNIESSTDPEKVLEIIQNQTA

>model.g605.t1 Augustusgene.g605.t1 JFAV02000106.1:48000-50600(-)

MVFSRLGFKSLSSDKKSSGSSSKHSQKLNSNDLDNTKINNLKKKLKYDPKGPVELAISIE

SPPCVMYGTTSNSTGALLSGLLKVIIKPLPQPQGQDSHQQLQKQQLVHLTCVKMRLVQRL

TYSKPFVPSDNDVLNNCHDCLTKEVELASWDIIKEKLLNKGTPTSSAENLGTGNKEQTEA

EQQQNGVPILPGEHSYPFSRLLSGRLPPSSKYGSNADSQLSYELVAQLQYFVPSSINKHV

GKNNKKTIDLTLPIQISHSILRGHDKNSVRVFPPTDVTATCTLPNVVYPKSKFPLELRLE

GVATENKRWRMRSLTWRLEEKIKIRQHSCDPHKKTLKNFENKIREDQKNGKICTNGNNGK

QKNIGQRRPLKRNALTGPIVTTSVIDEGYEQILLGPVRSITNEEAGPNDNNQNGNENDNH

NDNDTITNNIDETLQNAASGGNLSSPVPSSGLLNTHSSSFFNTLSSNNEDYNGTVHPNDY

VLRQQLINQQRLLREQQIEQERKKDISLYTEEVRTIAHDTIKNGWKSDFENGGKIELITD

IDCMHLNSGITNSKRNISSKNYMKNQHFLNFLKANKSNIACDVEDPVLGIYISHILVVEI

VVAEEALQYANGAPLHPKSSASSSHSSQSKKASSSNPNVMFTREELHQHHPHHSNRQQDI

TPMHSSSSAKTNGGQSGRSNSNTSTNSSTSSNAYKPQLISVPTGAARVLRMQFRITFTER

SGIGIPWDDEVPPTYQDIKYLSPPSYDVAMSPVMSNEITFNHPINNLNSVISASYLDSLN

NRLESLTMRGSNVENDGNSTDDEDQDIITIRANEMNPPPLAHFNLNLNNVQSIDGNVVGA

NDMMLSPRNTNVLNRGNSISNVFNSTQ

>model.g603.t1 Augustusgene.g603.t1 JFAV02000106.1:43243-44712(+)

MDATGNSSAPTSAYEIKYITFVLLGLCMLWPWNAMLSLTSYMSHDVFKDTTIYAKIYTST

MMTVSTITSVFANYQLSKQQYGYSIRIKRGLIWQFFVFVVLAALVGLVKKFKLLLDFFII

NLLVLISSVGTAYTQNGSMAWANVYGSEIYSQAIMMGQAIAGVLPSLVFFFLSFIEAGGD

ARDTSPVGIFFYYIATAAISIACYVLFNISKIGHEEKPVAQGNAVSLSPTANSAAYASHE

GQENDTGASFRETSKSTSKSNTESEDFANKFIADQNEQDPFNDAEQLEGFTTAENVMSTL

DEKASHIPLSVMYAKLKYLVLAIFLCFAATLLFPVFAANTYVSNFPLHNAQFIPLAFTVW

NVGDLYGRYIASKYHYIFKNPRIVNPLNLFWYSIIRMTIIPFFFTCNIFGHMDSPFVLQD

VWYLFLQFLFGVTNGNCISLSFMNIGDQLGDDDELKAASGGFSLIFVSSGLTFGSVLSYL

FVYIINTVYK

>model.g593.t1 Augustusgene.g593.t1 JFAV02000106.1:15017-16846(-)

MDLSHIDESDDLYPLALLMDELKHDDIANRVDAMKKLDTIAIALGPERTREELIPFLTEV

AQDDEDEVFAVLAEMLGSFVPLVGGYQYATCLLPPLEILSSTEETIVRDKAVASLNMIAK

ELSQDQILHDFIPLIEHLASTEWFSSKVSACGLFQSVITRIQDDKLRIKMFTIFYNLCID

ETPMVRRAAAKNLPAIIDLLTDNMGISTDQDWEFISNMFQKIITDNQDSVKFLAVDILIS

ILKFFNKKQNASHLPDLLNSTIQLISDEAWRVRYMAADRFTALAENFQSKNEYLQQLTPP

FLHLCEDNESDVRKAIAKQIPGFAKLIVKLDPNIVLDKIIPCVQSLSMDDNETVRTALAS

EITQMASLMNKTECTEHLIPILLNMLRDECPDVTLNIISNLEVVNKVVGIDILSENLLPA

ITDLSIDVNWRVRLAIIENIPLLAKQLSVDFFEERLSALCLSWLWDSVYSIRHAAVENLH

KLTEIFGSDWAKNEIINKLLKSKSQLLENFVYRITLLHALTALIPVVNDDVVLEKILPFI

DHLSEDKVPNIRFNVAKSYSIVVEKLDKQKYANLIGNTIIPKLQNLSKDSDPDVIFYARE

SLGKVENLVN

>model.g613.t1 Augustusgene.g613.t1 JFAV02000106.1:74971-75297(-)

MSASAPYDPYIPQEEQSQNKTAALQAQIDDTVGIMRDNINKVAERGERLTAIEDKADNLA

VSAQGFKRGANRVRKQMWWKDLKMKMCLVLAVIILLIVVIVPIAVHFAK

>model.g602.t1 Augustusgene.g602.t1 JFAV02000106.1:40013-42751(+)

MNFNNQFPQAAKQQQMGYSPFTAQQQQQQQQQQLGAPPGVLVGNNNAHANGMPMQSNAIY

SQQQLPNHPPGLSQQNQQSHQAQQFLNQQNHQGQQQQQQQQQQQQQQQQLPAQQQPQTEQ

SQRFMFAEMGETLSQFLGVQDASPNNLSHQHYNQIPGQGQVQSPPQFQHMNQMQQQQIQQ

QLQQQQQQQQLHQQQQLGQAPNQPLQGALLFENDPEYHPHLKNPQLLNHPNWELQMFLAM

ISRHMMGSESNIYAIESAINAIRGNLGGGTITNSHTEVSNRLVNATKASLIEMVNSLPKT

GSSVALANGLDDANTSLAGSSSSGSNGMSAGTPNPLSLSPESLPGTPSFEPVTPLTHNAQ

HGAPGMGAAAHPGSILLQHKKLSQYNIDEDDDNDVTSKQHAYDEQLWHVLDMANLKLSCI

SERLTSYTFLNRLYLNGNKISVIPKSIKNLKKLRVLDLSNNNISMLPAELGMLFNLKYLY

LFDNKIKSFPYEFGNLISLQFLGFEGNPIDPEVLKTYMDKGLTGLLFYLRDNAPEVKLVK

QRQIVEIDADGETTGNEYPTLSEAVQNQPLGPNSFSLVTYNTLCQHYATPKMYRYTPSWA

LSWSYRRDKLQKEILGFKTDIICLQEVETKTYEEFWLPLLSKQGYAGVFHSKTRSKTMQL

KDSKKVDGCCIFYKESEFKPVFYEHQDFSTLWMSNKNFLKTEDFFNRAMSKDNVVIYLKL

QHLKTNETVWIVTTHLHWDPRLNDVKAFQTAILLDHLEKLVKEQSHITREDQYKKEPIII

CGDFNSQRHSAVYELFSEGNVQKDHVDLHGRDYGHLTKNNFAHSFQFQSAYDCVGELNFT

NFTPTFTDVIDYIWYSPDVLRVRDVLGDIDSNYTSHFIGFPNADFPSDHIPMLSRFEFTK

NKADNISSRSRKV

>model.g588.t1 Augustusgene.g588.t1 JFAV02000106.1:6263-8755(-)

MSQGPGELAAVASSNGTLESKNSTQTKSNEGSNNNNDENNSNSNPNTELNNNTKSSLNTN

PDSTLSAVGNTTRSNSLIWKTGLSNRSGSSKDYLSDNESELSYVGLSPEKDKYSLPFEEE

DDDADEDNNGEEKPLQEELVLKTGVDDVNDNSKGELNGKGLVKGAIAKEVLDAAIDEVGS

DCETEKGAIVEEYERRGKEEVIRESSVADNAPVVHREDFEEPNPNNQLFEQEETKTEEKQ

ISDKNDGKQNEEPTDIDTNILKRKFEQVEDATEPSDSNKRVQTPVKEINEKLSTETKEEV

IDEVRSIEPPVDAILAPVTENTVASPGLADEQETNTVSLPAVSEETATIEDPLAANVALN

QIEEQTAKDITQEILAEKEEALSTGDKPVDAESKLETKSLSTVSNASMSITNDDKTQEPP

VETEVLIPVLPAADVDADADADADADAQVLSNEELSLAEEVNGELDSKILPEPKELFEAD

QESQRLKGYEAICDIETDFANLRQEIYENKIQKLKMELQMCLDGSHSALQKYLAQVQEIR

DEKLKKIYLKQQYRLDSIDRETRATRYILHQDFYKKTTDLKEALLNETTKQWYDINNERR

DIDLMSNVSAAAAAAAHKDTHGANDVDDFEEERINLMNMLYNGNTSILHQENKYHVPIKI

LNKSLSMITGYSTQPAIEIDHRTPHTFDLTHSGSLSSLESAAPYPFGITNQNGNINGDVN

ESSNGDEDRVCENINWNFVNNPVDKLEVIVDRMRFNNMISDFRGLKKYYGGFPSAPELPT

IKELELVQDFTKIHNQLVKKQQRLNGNLVLDNGTELHADGGAGKLTKQKRR

>model.g607.t1 Augustusgene.g607.t1 JFAV02000106.1:58148-59077(+)

MSDEVIWQVINQNFCSYKLKTDKNQNFCRNEYNVTGFCNRQSCPLANAKYATVKNIDGRL

YLYMKTAERAHTPAKLWERIKLSKNYQKALKQVDDHLLFWNKFLIHKCKQRLTKLTQVAI

TERRLALREEERHYVGVAPKVKRREQNRERKALVAAKIEKAIEKELLDRLKSGAYGDKPL

NVDEKIWKKVMGQMEDEQELEEDEEEEEDWDDEDLEEDEESDVGEVEYVEDEDDELVDME

DLEKWLGDESDSAASDSDDSDEDSEDDSDDESEAGAAKKRSKKASQGKKRPRVEIEYEQE

TEPQQQELAQ

>model.g598.t1 Augustusgene.g598.t1 JFAV02000106.1:27820-29283(-)

MQYKYPNLSDEQLKPLLTEIHHWGLTNGLVMYPQTGNPENTTAVAPITVYPTIIPEKCFD

QAIGVQTVYNELYAKIVQDYDWLHAETEKLSKHDSEFTGKLWDVYLKAKKLGIPQKLELG

IFRNDFLIDSVKNEIKQVEFNTVSVSFGGLSTKVSQLHRYLKLHGFYSDDLLISNVPVSD

SAEKLSDGLFAGVHAYEEQQHGSFDATTIVAFIVQDGERNVFDQRILEYNLASNHGIKAV

RLTIQDVPKLLKKCEKTSKLFYTPTNDEISVVYFRSGYAPNDFKSEQDWESRLLLETSLS

IKAPSLKVQLSGTKKIQQLLTDENILRKFISDDDKISELQSTFVQIYPLDDSPLGVKAKE

IIKTGNTDNYVLKPQREGGGNNIYKSDINPFLADKDEKDWSAYILMEMINAEPTEGNVIL

RNNELYREKILSEFGVFGVILFDENQIHHNEYSGWLLRSKFSSSNEGGVAAGFGCVDSVA

VMPKSSVV

>model.g610.t1 Augustusgene.g610.t1 JFAV02000106.1:65185-66276(-)

MPPIIAYVKINKQTSLTLRVFVNRKEVLSNFKKTTTLANNDEFDINTLNDVQKSVFEAPV

LSSNSIIRLKKPLVKLFLSNKDLNDLVENLRNDLILILYEFSDELVVSQILNKMKIDSSI

ELFKVTQFVVSKNNLKIKDQNVNIDGLPVHGKSIKKLGKYKYIIDFDDKWGCDIVISDIR

LLKSFKRVMDLNGYTLLSNAGSADLLGTKLSHKVADFVGQQPLHPDTQGIDQFGESGEVQ

QMEAEDDNSDDDDEASEYNYIQGASNSSRLILRSLRKGKAKKSSEEKFAPLILQEENDDG

EENNGLSAFDREVEALQKQNAVGASFDDQDSDVKFEASYSYKPIANLGKCIDIHVLKRPK

HRRG

>model.g596.t1 Augustusgene.g596.t1 JFAV02000106.1:21813-25847(-)

MFNTNIFKNEKHANNNLTTNKIKTSEDGASSNYTTKVLPKTRKERHSIPTFDIQEELQQQ

RPLAIQATLQGEAIPEEQSLQASSNQTSALPNETLLAQEEVFMNELITFPSLSTHSFSYN

PLSPNSLFVRLIILKRTLEVMLHNPSLIKEPTETQLAKNKAADLKEESRPNMIISRSTND

LYRSPAYQNFSPMRRKISQQLVQDPSATSKRNASSAAFDAYVKEPTSTSSYSGNNSNRSS

IIGPSVSSHNLSRVLSSQSGSEDEQELDFAQPIAPFLKRHYSAGLTNHNNGNNKSESHLT

GSTFNNHIFTTNSNNNFHVKTNVAKNDYTKGRRSITEENDENSESLNDLDDYNDLLSSYS

SDSDNHSIATHLNKNPFDTHERKSSFYKLEDFQDSSEFLQTKTHDLQSLLDLLNDTLENN

VVDLPATDDEKQVSNLHMMSVLNITKLLTGSSSSHKESERTETLRKNLINSLSQPFYELS

HRAEQDYSQRVLHDFTAGKNTSPQAIVLCENENPWSFKNVNDIACLMFGVARNSFRALNL

LDLIHKDSRNFVLNKLVVTELENDNLVFTGEIIGIAQPSLKEPVWTSLWARRKNGLIHFL

FQKVPCDSIKCDFSLEHLKVDNFTADKAITFRVKTNQKDDNETPEGHNNAIKKVKSVHDI

EDLFEISNSISEFISNIKQTGYDDHEMNVAEIIKGVNDTRYFTLNSLDYNIPCAVTAGYA

FGSDIKLNIHSMPYIAGAFIIDSSTNSYELISFNKSVSKNLFGIHAKDLLKKPIKTVIPR

FNAIIEHIHKYYPEYNINKETNKSLVLTEHFFRKINTELEVYANLITSQEYETKFYNSKG

INGLHRDGSLIKLDVQLRVLNTKYCLLWITHSRDIVSKNYNIMPSQIKILRENERFVSHQ

QNETGENLVSNDTEYDRSIKEKLESVKKTGISGGSSTTTLSGKNSRGPSVKSLTKLLTSL

SNKHSDTTICDESTVAGKENDDPDETQSYQHAQSSCDEEESPVDIARKYKQNKESFLKSS

NFNFDRQLIISATSRPSSANTVEAISKVPEANGHVDSYANLYKNVAGMETFKLGQLKHKK

KYTDFTLLESMGRGAYGLVDLCMHKELKYIVVLKRIIKERILFDTWVRDVRLGTVPSEIH

IMALLNESPHENIMYLLDFFEDDNFYYIETPIHGNTGSIDLFDLIEVKTDLKEIEVKLLF

KQVVSGVKHLHELGIVHRDIKDENVIVDGNGCIKIIDFGAATHVAKGPFDTFVGTIDYAA

PEVLSGEKYAGKPQDCWSLGVLLYTIVFKENPFYNIDEIMDAALKIPTGTNVSNECIDLI

KKILDKNVKQRPDINEIFHDPWLKL

>model.g604.t1 Augustusgene.g604.t1 JFAV02000106.1:45545-47872(+)

MSKVSAAQKTVKTETTKVSAAKTEKFNEKNVDSTIDSDESDDELISEKDELLGVPKDSND

KQLDTAGAEDAATAAKNPLYRMEAILVPLLLTALSFFVRMYKIGASDRVVWDEAHFGKFG

SYYLRHEFYHDVHPPLGKMLVGFSGYLAGYNGSWDFPSGETYPEYLDYVKMRIFNAAFSA

LCTPLAYFTAKAIGFSLPTVWLFAILVCFENSYATLARFILLDSMLLFFTVASFYCFVMF

HNQRFRSFSRKWWKWLILTGVSLGCAISVKMVGLFVITLVGIYTVLDLWTMFGDKSIKKT

TYLFHWIARIVCLIMIPFAVFMFCFKIHFDLLSHSGTGDANMPSLFQASLQGTDVGVGPR

DIAIGSSTVTLKNQHLGGALLHSHVQTYPAGSQQQQVTTYSHKDSNNHWVFQRMRGAEPW

SAAVDGDSELEYIEDGGIYRLVHVNTGRNLHSHTIPAPESATAWEVSGYGDDTVGDRKDN

WVIEFAEQVGDESKDKLHLLTTSFRLRNEEVGCYLAQTGKILPQWGFRQTEVACVKDPFK

RDKRVWWNIETHENEKLPTPESFKYPKPKFLKSFVQLNLAMMATNNALVPDPEKDDQLAS

SFWEWPSLHVGIRMCGWDENHVKYFMLGSPASTWLSTVGVLAFMGLIVVYLLRWQRQFSD

FADSVKLNQFLMGGLYPLLGWGLHYTPFAIMGRVTYVHHYLPALYFALIILAYWFEWVFY

NTKKSKLNNILKIAIYGTYYAVVIGGFWYFSPISFGMTGPSSDYEYLNWLSSWRIA

>model.g592.t1 Augustusgene.g592.t1 JFAV02000106.1:13641-14867(+)

MKRKRVEVEIEQGTTAIEDEVKLKYEAHVQKIKFLPTTEYLKMMTRELTLMEPLDLLLQQ

DPEKAAKLPKNFIPIYSKIREMRKTVSAAVDAQGCASMPFYLNFKLPHEPIWPTPENFRL

QVLVSLMLSSQTKDEMNCKAMSQLMIYCLDNLDSVHGISFKSLSAIDEHTLDTLIQPVGF

HTRKAKYVKTAVETIISQHATASLSDQDSPLDVPYHYNDIIALQGVGPKMANLLIQCAWA

YGEHGIGVDVHVDRLSRMFGWVPGPKERKNPEETRVALEKMLPVSLWTEINSVLVGFGQS

ICIPRRQKCNECFANDICPGRIRPKIDKTYSSYEEWFSQTKNVLPIRTVEGEIEDMVDFV

KKGIKGEENHASFGVKLEPVPFKFSSYEHAVEKKLTTSKYFNKKKKPTP

>model.g621.t1 Augustusgene.g621.t1 JFAV02000107.1:898-2310(+)

MVWRHLDVDQQHINYLCISLTGIIFCLTARFFKSRLYVGEALFFTTTGIILGPICLNWCD

PFSWTTNVDKITIELSRIILCIQLFTSGLELPVKYMQYNFKSVFLLLVPVMTAGWLIVGS

FIYLLIPGLKFKHGLLVAGCITATDPVLCQSIVSGKYAQKYVPERLRNLITAESGANDGL

AFPFVYLSLFLITLDSQKGEILKEFFVVTILYTCILGTFVGLVLGWSSSKVVQLSSRHGL

IDEQSVHIYYIFLSLLMAGMGSLLGMDELLISFFGGCAFGWDGFYQKNVANSASSLTSGI

DYLLNILYFAYFGTLIPWQKYTNSYLSLSAWRLVLIGVVVVFLRRLPVVFTIFRWVPAVK

NLKESFFVGWFGPIGVGAIFTSLLAKDYLEKTLEKDIALDSHSNDVHLGIDTVWVLCSFI

VVISMIVHGFSVPAIMFSKCFSKQTKKLGTPSRSSCDVSVFDKEEQILEKC

>model.g640.t1 Augustusgene.g640.t1 JFAV02000107.1:58961-60799(-)

MNPFEGLDNAFLASNKQLKSSYRRLTWNNLKSIEQFLKEAQEILVKYSKPNEKVKLCVKK

CFEVVLQKYPLFFGYWKKYAAITLQLYGAEEQTKVLKKSVQRFPTSLELWQDYLTVLKEG

STFTPEIKKQFDTAASLIGNHFMSEPFWDLYISFITESITNSEEKDELLQQMYEKVTSIP

LYSYAKFYKQYQTFIQKHPQLNIDSQKTTSVFQKVRTTVNTVWAFESKIKQPFFTIGPMK

KPLLDNWDTYLTFLLGPPERHTLQISDDYIQCCFERCLIPCHLYEFFWVKYHVWFGTRYP

DDDKGITKLFERSISAVPQSIKLKQLYLVYRKSKIAGSADLGGMMKYLKLLQRFLQQNCS

SKDLFNEYVRVHKTIHFSNKTPAEYEEFLEFTINDFFKITNKKIRVSQDEFLSKNLINDD

NIDMLVIELVRSSCFSSQNIAQTRKYYKVFGNRKQLRKSHEFWLEYYKFERSNKNVQSLL

EFIERLGVDIFLPTVLMNDIVHDFHQFFLTQSSVEQYTKLDLSKNIDPLFFLDMKLNNPK

LFGHHQPKMLKQYYKSPECKELGHPGVQSERPIVKNSIMKKNSKRRTKQKFDLPAIKNIN

KISTGLVQGSKRK

>model.g637.t1 Augustusgene.g637.t1 JFAV02000107.1:52042-53553(-)

MESKLEWLTKLNPTASATAPALRRSSIIGTIGPKTNNPEVLVELRKAGLNIVRMNFSHGS

YEYHQSVIDNARKSEELFPGRPLAIALDTKGPEIRTGTTTNEVDYPIPPNHEMIFSTDAK

YAKACDDKVMFIDYANITKVITKGRIIYVDDGVLSFEVLEVVDDKTLKVKSLNAGKICSH

KGVNLPGTDVDLPALSEKDKSDLRFGVKNGVHMVFASFIRTANDVKTIREVLGEDGKDIK

IIVKIENQQGVNNFDEILKVTDGVMVARGDLGIEIPAPHVFAVQKQLIAKSNLAGKPVVC

ATQMLESMTYNPRPTRAEVSDVGNAILDGADCVMLSGETAKGNYPVNAVKMMAETALIAE

QAIAYGPTYDDLRNTTPKPLSTTETVAAAAVAAVYEQEAKAIIVLSTSGNTSRLVSKYRP

NVPIILVTRNERAARFSHLNRGVYPFVYSEAVASEWTVDTEKRFQFGIQKAKEFGFLSEG

DAVVTVQGSSAGIGHSNTLRIINA

>model.g631.t1 Augustusgene.g631.t1 JFAV02000107.1:30397-35403(+)

MDVSKPVGSEIVSVDFSVMTSSEIKALSAKQITNPTVLDNLGHPINGGLYDLSLGAFLKN

LCLTCGLDENSCPGHQGHIELPVPCYNPLFFNQLYIYLRSTCLYCHHFKLRKSESHRFAC

KLRLLQYGLIDDCYEFDNISVGNSSLASSVEDEDNEDDEEAMVEGMPVSNGNNKEKINEQ

LFKELKLKRNEFVDLKIAQALSTGRTTEYGTFTAIVNDERKKLVHDFYKKLLSRPKCDNC

GMFSPKYRKDGITKIFENPLTEKQLTNNRIKGLARRDMLKKQQQARKLNGDNSDGKMDDE

MMDIPITKPKIGSTYILSTEIRNILKSVFKNEQVVLQYIFHARPNLSKKLVKSDMFFMDV

IVVPPTRFRLPSKLGDEVHENSQNQLLSKILTTSLLIRDLNDDLSKLQKDKVSVDDKKII

FNRLMNAFVTIQNDVNSFIDSTKAPTNGGKTPTPGIKQALEKKEGLFRKHMMGKRVNYAA

RSVISPDPNIETNEIGVPPVFAKKLTYPEPVTNYNVAELRQAVINGPDKWPGAIQIQNED

GSLISLIGMSLEQRKALANQLLTPTNNNNLKVHTLNKKVYRHIKNNDIVLMNRQPTLHKA

SMMGHKVRVLPSEKTLRLHYANTGAYNADFDGDEMNMHFPQNENARAEAFNLANTDSQYL

TPTSGSPVRGLIQDHISAGVWLTSKDSFFTREQYQQYIYSCIRPEHGHATRSRIVTLPPA

IIKPQPLWTGKQIISTVLLNVTPSDIPGINLKSTNKIKNDYWGKGSLENEVIFHNGELIC

GILDKSQYGASKYGIVHSMHEVYGPETAGKVLSVLGRLFTHYISTSAFTCGMDDLRLTAD

GNKARRDILQKSTDIGREAAAEVTNLESDVKANDPELLKRLEEILRDDNKLAILDAISSS

KVNGITSEVVSTCVPAGTMKKFPYNSMQAMALSGAKGSNVNVSQIMCLLGQQALEGRRVP

VMVSGKTLPSFKPFETDARAGGYIKGRFYSGIRPQEYYFHCMAGREGLIDTAVKTSRSGY

LQRCLTKQLEGVHISYDNSVRDADGTLIQFLYGGDAVDITKESHMTQFNFCADNYDALLK

KYNPAALIDHLDIESAIKYAKKALKSRKKAAKVPHFEQNNKYDPVLSKYNPAKYLGSVSE

KFQDRLEKFIDDNADLFKKKKAVVSDKKFKALMQLKYMRSLINPGEAVGIIAAQSIGEPS

TQMTLNTFHFAGHGAANVTLGIPRLREIVMTASAAIQTPQMSLPVLDDVSDEQADAYCKS

VAKIILSEVIDNVQVTETVGSSESSNAPSRSYIVRMNFFDEKDYDEEYDVKKEQLETVIN

KKFLHHLESAIVKEIRKQKHKSSLPDELNVAVPKSQTALGAYTSSSRAGDGDEDDEESGS

KKSKAAASYDDPDEDEIDTMRQAEKSDDEDLLNSSSDEEDNASASDDEMDVDDDNDNEKS

AELSKNMTQEQLDRQSAVVTSHKYITKYQFDDSEGKWCEFKLELSADTEKLLMVNIIEDI

CKVCVIREIPNIGRCVFPKPEGGKRTLITEGVNFHAVWDHDAFIDVNGIKSNDVAAVLRT

YGVEAARNTIVNEINRVFSTYAISVSFRHLDLIADMMTRQGTYLAFNRQGMETSTSSLMK

MSYETTCGFLTKAVLDHEREELTSPSAKLVLGKLSGVGTGSFDLLTQSL

>model.g627.t1 Augustusgene.g627.t1 JFAV02000107.1:21352-21858(-)

MTQNQREQQNVTQRLQTELVQMMTKGTPPGLSAFPIDDEDITQWSGIITGPEGTPYEGLK

FKLLFKFPVEYPFVPPKVTFTSPMWHPNVDMSGNICLDILKTQWSAVYNVQTILLSLQAL

LEEPNNNSPLNAVAANLWKTNMVEYKKKLMARYEEFDEDEDDDDDDDED

>model.g624.t1 Augustusgene.g624.t1 JFAV02000107.1:9993-14129(+)

MKFVRQGAPILSIALIACTFLLQVVKSAHNTEIDISQSLYKERNNETTYDAAIDNLRVPE

ASVLWQCVKQLKNIILLLDEERDASEISKSDSVINNLYGIIYGLDLSDDFEQDDSNEDDL

FKKAHNYLKSVNPSYAAFFKSYYQYNVYDLPSSDPPKIDVEKLTNFFIINNKVYEKSDDI

FYLKSHEIKPGEDTADIFLINKDELVIGSNNEAPLALFYGCDSPENQDEFEDFNSNLWNE

AQGGKIRYVLRRLCTDSSNNYPWNNNQKFVDVSFLKSNLHNYTFNGEIININHNDNTTQF

MDLQLTDFIIKYHNSLENNKNAWGTLNFMKKIINKLPFESIQNKCELLHTGNVEDYSSIT

EDLDALMKLGVNYDMLGVFVNGVNIRLTDFSMRSLVESIQREFRFLKTFKQIASKHNLEL

SVEDMKKLITDFTVASQLFTKNNQPRRFDVTQTIDKNVDVVIFANDLENDSQYATTLSTD

PLEFMTQKSHFGEIPAYAENWNDVIFITDFQNKEMVDGLIRVLEVVQNGYPQRIGILPLP

KSFESMENDQEYMKVISQLQKIGNSNIDGLLKFLNDGKGLKFDKKLNLLNPTSPVVSSIL

STIQLMKITENSIMVNGQLFGFRENAWQYLISTIIKEDVGFLKSALPVVMKDWEEQEHSM

RKLLYAEAQAFVSRDLVLTPNIHGDSTYSIENLDGLDEIKNQGRVFEYVKSDVYNVVHTL

SVVDDFESPSAWDSLSNIMALHLYGVKLRLIHNGAESKHWNVLKKLLSGTDVVNSEELLQ

HAPNGKSNKEKYPLDFSRWLVDLSHEQLSSTKFLVLNGRVINLENHSFSTLHPITSNDYF

NLIKHESIRSLQTIKFVEKYHLGQIIPPPLIEEFSAFFTKIYYDDMLLSNGIDFTVESIL

PRLPLRKVLNKKSVLKSNSLFLHNKSKKRPIDLTLVLDPMEERSQTFISNVAQLMDKSFQ

KFVNLEILFLPTKDLKFFSVQRLYYDSQELEKQETFDIDTEELEVHVDMTAPDLTPDGKS

IWCTAYVQDVTHEISRLNVESVGSNICVNVVNVRGDIVTSFQTMQTFGFGQFKLPLNETN

EKYHFELCPTNSGYLMNGFTLDVRADMSFWAEFSVHDFFSKRAYIQVTRTDDELVFSSVD

SDTLNVIVTLQDAESEGAFMSLYSTLLENFPSGFKIWVWKQDFHGVDQVAERISVLENVE

LISYKWPKWLRPTKLDTNKLNLGKFLFLDMVLPADTMNVVYLDLKELDEGFIQSYVSDID

VVKSIVVGEENFTVGMVPYADDEHGYWEQGYWKEYLQNNKLNFFKADNFIVNLKKFREAG

LGDILRVHYQQISQDVFSLRSYGQDLINNIQLKAKIYALDAVVDEVADLQEDDVVHDEL

>model.g620.t1 Augustusgene.g620.t1 JFAV02000107.1:59-467(-)

MVRFKSRYMLFEILYPPTAEEYSTFSEEEKFAIEPALLQHHKVTPPDVNNRVLVYEIKKS

LQYFFGDYGSGRGTSMFQMKYFSNSTSTGILRCSKDDYEYVLMTLNMMKRIGSLENVIIN

VVKVSGTIKKLEEFAI

>model.g625.t1 Augustusgene.g625.t1 JFAV02000107.1:14614-17904(+)

MNDKQPISIKEELSPSVKLPNTQQNNILHAQTTGDKSNSEDQDVEKKSKKRMACTNCRKR

RKKCDFQFPCSSCVKLSLDCNINVNDLRKKRYKNKYVEELESKVQFLERKLQEAQSLRSS

SFANSASPASSSSYNIISPSNSNGLLQETRNSPLYQNTYEQKQLKPQTSTSSLPALSNSA

LKKSSSRINDLKTTVIIRPDSFLSSEGFQNNKESSVPLGLDQGQTRSKDIKLQPIIPSLS

TLQAKVALTNGSTNNGSDNTGTAPIPNHSVQLPHLPFHATKPAHLSNDSQILQSLKLFFQ

WLYPGYFIFVHRESFLYGFFTDYQDGYEHSRYCSSELVYAVAAVGSRLSPELRSKSAEYF

ETARLEVLNKIFQQSYTAEITTVQTLMCLAFYELGNGNFSSAWYFSGLAIRIGYDIGFQL

DPQAWVVESSGKSGVEDDNLGNKSNQSGMGHSSEDDFNDSDKLTRSEMAIRSRIYWGCYV

ADHYICLILGRAPSLSVSNSTIPDSDEMPDIAGTEDFKFESKLILQVSLPLKNLIILSRI

VQIFTSKLFIEPNISKLEKLEYLRKFNSKVYSWRQSLPNFLKWSKNTLENNLDYSTDPTI

CYFWYHYYIVLLTFNKPFIGDSEVSQVAVKEVLDDLKMLFTNFKLKMGKDCLNKCSLNQL

FSCSLAIQILVKLKSITANEAETQPSGSKEKELEFRKKLKNIEQSLKFFSDMFELMSPTY

DIPGKMMGGTLSRNTKGNTYLLRSKDSLSVIKKSSSSVPPSFNGTTSVSMGLSPGGSGFL

QDYAHPPSPFGMTEKLQFATSNSNHPSPPPPQLSFFKTNNLSMKKHFFTSGHPMPNGQSH

LHSLSPQPGLQHPQHQQPLSQLQQTQTQTQSQTQTHSINSYPPSSSQPSASVVIGMNVNN

QYVNNVNDLNNMISYHHAMKRYDLGNTAPVSAATTNNVHEQNSTHTSSKDILVPSLEHPD

FDPTTAITPTNTTKDVVSTTMNNPVNNSLGDTTTDTNIHKMALPTLNMPVMSTFKTTTPN

NKNSGLSEATLLSNTNSAGLSSAIPNNHIPQSNNNINALFINNNNIEYTYDFSLSDEIDG

IIRETFGIDNFNTHHTL

>model.g636.t1 Augustusgene.g636.t1 JFAV02000107.1:48734-51772(+)

MGLTKEELQLLLESFNSDRSGGNEQKEKVNLENVDTSEVSSVSDLNKHTQAVTDLNVLLS

DVDSELINDSDHISTDNYEFNEPQKEKINHSVKNYFQLSTAKENKNESADVQHLQKNENA

DFSPFGSNSFLTLSDKITQSPTKIPPNTKLFSTQSLETSPFKKKNKEGNSGKQNFQLLEN

INIYVNGYTDPGRLQLHRLMTMYGGKFTERLTSKKAVSHIIATNLTPKKRIEFAKYKVVR

PQWITESIKAQKVLNWQEYSLFYETKTNITKKFAKAGSGNSADPPPMHTIDCNHPDFLAS

FYSKSRLHFLSTTKMLLQSKYLKYTSPPLASTPNAVSSSLRQKLTSKTASTPLKTIYYID

FDCFFAKVSSLMDPSVDIDKDCIVVSHGQNTADIASCNYVARQKFGIYNGEWVSSAKLKC

KNDLKILPYHFAKYKEISEYLQLYLKNKFDCIIPLSCDECIAYDYGELDYEKSRIKCENI

REGVEKITKCTISIGVSTTLYVSKLALKYAKPDGYKIVDPERDNVSEFVSLFDLTDLPGI

GYNIVEKINHLASSCKKDQSTKGTKIFTIADLIEFIETQRQRSQLSFRQVTIQILSSIGE

KLLKKFINYLADCKDDSESLQKLDQPLKYFERKSISVDINYGVRFQNIDQVYKFLERICN

YLLTQLQEISMCTNQLVLKLAKRLPGEPIEPSKHMGMGKCEFMSKLKNLRKYTNEKFIIL

PELKSLFHQLYKAYAISDIRGISVQFMRLSKTDAAIPKTSLLDFGMFKLKKLTDNDALVM

HNAKTPTTKDKKCASEPAALPVTPQFGSSKRSTSPVKDFFDNYSKKKRKLSIPDHIDMSV

LRELPVNLQRELLAEYHLINTINTSKAKRILEKSTGKLSSSLKKSHSSETIYMQNNDYET

ANSSKERLPRKRITFQTKQRPKEIRQLLQAWVQYSIEIGPLDEDKALFLKFCQKLLTQRK

FSNIILYITFLQNLVDYYRDRSSQGSESSEGVDAWSRYLLLQLQPLLSQCDNL

>model.g623.t1 Augustusgene.g623.t1 JFAV02000107.1:6373-9237(-)

MTVGNKELWTAPHVRDTFLKYFIEKKGHKYVKSSPVIPYDDPTLLFANAGMNQYKPIFLG

TVDPQSDFASLKRACNSQKCIRAGGKHNDLEDVGKDSYHHTFFEMLGNWSFGDYFKKEAI

QYSWDLLTNVYGIPKDRLYVTYFEGDEKNGLEPDLEAKQYWKDVGVEEDHILPGNAKDNF

WEMGDQGPCGPCSEIHYDRIGGRNAAHLVNMDDPDVLEIWNNVFIQYNREQGGSLKSLPA

KHVDTGMGFERLVSVLQDVRSNYDTDVFQPLFAKIQEITGCRPYSGKFGDEDKDGIDTAY

RVLSDHVRTLIFALSDGGVPNNEGRGYVLRRILRRGARYVRKYMNYPIGDFFSKLAPTLI

DQVKDIFPEVNKDPSYLFEILNEEELSFAKTLDRGEKLFEKYAEVASQSAHKTLNGKEVW

RLYDTYGFPVDLTRLMAEEQNLKIDEEGFEKAKLESYEASKKSNKKNQGTLIKLNVHDLS

KLNEDSVSKTNDEFKYGLENIDATILAIHDGENFVDEITETGKPYGIILDKTCFYAEQGG

QEYDTGKIVVDGVSEFNVENVQLYNGYVFHTGVLQEGKLSKNEKVIASYDELRRFPLRNN

HTGTHILNFALKETLGADVDQKGSLVAPEKLRFDFSHKKALSFEELEKVEAICNKLIKEN

MTVYYKEVPLELAQSIISVRAVFGETYPDPVRVVSVGKPVEDLLADPKNDEWNNYSVEFC

GGTHVAKTGDIKEFVIIEESGIAKGIRRMVAVSGSEAYEVQRIAADFNKELDHVETLPFG

PMKEKRIKELGVELGQLSISVISKNKLKERFNKIEKEVKDEIKTRAKAEIKQTVDEVTQF

FEENEAAPFLVKHIEIPTNAKAITEAINYIKNNKKEKSIYLLTGNDASGRVAHGCYISNE

ALTKSVDGSALAKAVSASIGGKAGGKGNVFQGMGDQYAGIDAAVEEVTKLLDEKL

>model.g641.t1 Augustusgene.g641.t1 JFAV02000107.1:61176-63332(+)

MPLKPALRDLGASTFEETELSTENIQHSNMKSSTIPKSASVNFQIPKSGDDFFGYQSLKG

NAITPPQRVKNSTGNRRSYNISPDLLKPFPRPNSLAPPPPSVSSAGSTVFSNTKQYPTLY

SESEKGEFDDADHAEISFRKLKAKQQNLPTLNVNPQTRQFLDDFEFETDEENTDETEEEE

YINAKAAQSNPSSRSKKYLNKMDVELNDIQENFEKGFRISSDIHQLNPALYTAGSFHQEQ

QKQTPAIHAQQHMHHNPLLLQPKSMMNLRPASGRAFSSSPDKLATAYEDQTYKPTPFDKL

ESIREVRRKASRSKLPKYKSMSNLKSNTISAGNGNNGMTTTRKLQNKQSFPSLKVVHKDS

FFPSQKNLHQFQDKNDNFQDDTFGEDFNDVIPQYLVSSSPRKSTKPQAVNILENYDEVEQ

VNVEKNEDYQCDYPELSTPQLRKPAAKKKWKSKKQVLNSFRENSDAEEDDLSDTGTVYRT

SIRKPSNIQLLKQEIDSNTPLTKGEMVYNPETFKWEGNTDALSEFPENYKYKTANRRSSV

VKSEGIQLRKSRSTLFPRSNNNKIEGASTESGLQSRKVVGNMQFDSKNQRWVSLHGDSGE

IDPFKNIEDHVLAQSSSYGNSRFTSNGTDSNLKRYVSASSTAGNGRASSTGKVYSTSRPQ

FNPTFVISSKKLEVFYHEENKWLDKLSGWINQDLEHADSETRFEYAYEIRNMVLSSTKK

>model.g632.t1 Augustusgene.g632.t1 JFAV02000107.1:35631-38660(-)

MAKKGKKNAPNYWDEEVEEDQQLQDQPQDLGTEPTSAGSEVSGAESASASASASVEPETA

GADDIESDFMSTLKKSKNKKKNNNKKNDDEEGQDNGGKPVLKSKKEKEKEKKEREKQKKK

EQAAKKKAAGGAKNNDQSDKKNGAANKDKKDAETQDSGSASTSAPSKPKKKVPAGLAALR

KQLELQKQYDEQERLAREEEERLEKEEEERAAKELAQKEEEKKLKKEKEKARKERLKAEG

KLLTRKQKEEKKLLERRRAALLAAGNIKVAALEKANNGEDAADQESKKPKKVVYGKKKKK

SKEELEAEQLAKKAAEEAAKKEAENVEGEEDELVDDWEALLSGEEEEEQGEEASAVEETP

KEETKAKAEAPKAEVSKEVTPKPAAIDPKYDLYSAAANASRTKPKGNKELRSPICCILGH

VDTGKTKLLDKIRQTNVQGGEAGGITQQIGATYFPIEAIQQKTAVMAEYEEQTFDVPGLL

VIDTPGHESFTNLRSRGSSLCNIAILVIDIMHGLEQQTLESIRLLRDRKAPFVVALNKID

RLYDWQTIPNNSFRDSFEKQTRSVKQEFEKRFNEIKVALAEQGLNSELYFQNKNMSKFVS

IVPTSAVTGEGVPDLLWLLLELTQKRMSKQLKYVSNEIEATVLEVKVVEGFGTTIDVILS

NGKLKEGDRIVLCGLNGPIVTNIRALLTPQPLRELRLKSEYVHHKEVSAALGVKIAANDL

EKAVSGSRLLVVQDEEDEEDMMEDVMDDLTGLLDSVDNTGKGVVVQASTLGSLEALLDFL

KDMKIPVMSIGLGPVFKRDVMKCGTMLEKAPEYAVMLCFDVKVDKEAEHYAEEQGIKIFN

AEVIYHLFDAFTAYQKELNDKKREEYGSQVVFPCVLNTIQIINKRGPMIIGVDVMEGTLR

IGTPICAVKVDPATKQRQVLPLGKVASLEINHESVADVKRGQTNAGVAMRLDDPSSQQPI

WGRHVDEKDTLYSMISRRSIDTLKDPLFRDQVPKTDWLLIKKLKPTFGID

>model.g634.t1 Augustusgene.g634.t1 JFAV02000107.1:40932-41855(-)

MSFQIQYELANMKNQIQPQQLTQNVFFGPVNSLSRLDFLMENNIKHFVCVDVPTAMAVKY

SEKIPLDKYAYNFVNFDSKFNKAEHTNEKLFEIMCFNQNFSSQLFNFIKQTLPSSSDLLN

NEIYNMNSLRVLQSNIVTCGKGYERFEVFNDLLTIIKYAKNGNVLVVSSNGNDESLITLL

ISQVLRENCTADVMDAIKYVKALRPTIQELNLQQIQFFTGFIEYSEKIKSKSWNNIMDYN

RKAHMKKTNMRTSSAYDLTQQDDDEEQVSSMQQQQQQQECFNYRPVNAMETSTDFSRSYK

RARRTDYD

>model.g626.t1 Augustusgene.g626.t1 JFAV02000107.1:19656-21050(+)

MSVTEFNTPQPEHVELNVCKESNSQNTIKTNHRNPMAALLSKESSDLSDSMTAATAATIH

NSSSNNLNANSHNNESVLNDTELRSSQGSMGYGTKELLSPPLSPNINSHGTNTDFMSSDF

TNADYTKHYTSFPSSKTKFLNPTNYLLLTPQSHPAQLSAHPLTHKSAHTTFNDNSFDSIF

LEPQDTHKDVYVPCAPDYKISSHKPLVVDLQWSKKEYASDLKFFVSKYRKFRQSTQNGTH

MNNRLEKRYLTRSSSNMVNDYTNENTFNGSILTSNNSYSNGLQPVRTYRTRLRTIAEGNK

DDEPHAREPSMPKKFAKPPHIPNKTAKPHLSKPHVSHPPLNEWRQMPDYAPPLSSLDNIH

NGSSKGIMKVEWKGSTMDLSQDPLRNKLHPAELQLASILRLSCDSYLASKRRLFMEKVSK

LKRGQGFRRTDAQKVCRIDVNKASRLYQAFEKVGWLDDKNFVKFL

>model.g633.t1 Augustusgene.g633.t1 JFAV02000107.1:39530-40633(+)

MSTVDKIKSIEEEMARTQKNKNTEFHLGQLKAKLAKLKRELLAQATGGSGGGGGVGFDAK

RSGVASIGYVGFPSVGKSTLLTKLTGVESEAAAYEFTTLVTVPGMIKYRGAKLQMLDLPG

IIEGAKDGRGRGKQVIGVARSCNLLFIVLDVNKPLTHKQLIEKELEGVGIRLNKTRPDIL

IKKKEKGGLSITNTVPLTHIDADVVRAVMHEYKINSAEVAFRCDATVDDLIDVLEDQTRR

YMPAIYVLNKIDSLSLEELQLLYRIPNAVPISSGQEWNLDKLLEIMWDRLNLVRVYTKPK

GVMPDFSDPVVLRNDKCTVKDFCNSIHKSLVDDFKNALVYGSSVKHQPQYVGLNHVLEDE

DVITILKK

>model.g635.t1 Augustusgene.g635.t1 JFAV02000107.1:42808-44823(-)

MNNLTNTATPNTNDLKPMSNFEDWIYQNVSDSTTMPYSEKMNVSQSDSTLLDRGYVLDNN

GHHNVGNQANAITHSSNDNINQFTNFHSMNQFTNYNHNNTNHHTNNSHGFTHPNGMNKSL

SLDGTSKNYTFNTIDDTRNLSTLSNTNGLGLNVSDINTQNTPFNIHDLDYQTVQKNMQAR

NASLLTGHDGLYKIEENSGVESCAGGDAFANYQTYSKLTSDGMIDEHDMRYNFLNTPVST

VMSATASNMSNTAIPTQNAPTSYTSSSNTTISPTPESTLKKTKKAKNKGKKPRNKKLVSG

SSGENKEQMISAKNASSEGLHNDTKKEQNLANADHNASGLNNNKSGNTQDVMVKLEKPDS

DQSSVLQLPDPHDIMGIKLESGNPGNLDLLLEEMPAEVLGIDVGMGASAADAAATAATTA

SNDYLKTEQDNDADAISKNKPASKKGKNKKKIMTAQQKEVHNRVEKKYRININTKIARLQ

KVIPWLASANASTAFEIHESEQMGNCSNGAPSSSSSSSSSSSSSSGSSVSAKSSETLVPE

ENDIHTDSLKSKTDQAKKNDNDERKDSVTSLDNQPKLNKSIILEKAVDYIMYLQNNEKLY

GLEVQRLKAEIKRLKKMNPNSNNNNSNSSNFESPSNQSDCSSRSTHKEETSSACVSPTNT

SLSQSVKTSHQA

>model.g622.t1 Augustusgene.g622.t1 JFAV02000107.1:2848-6024(-)

MLGENDNLPPPNEGATQSSFKATKAVKRKRSRAIVSCLYCRERRLKCDQARPKCNSCESR

NLDACFYFEEATPELIASERAKIRARKTKITKSRKTLATVPNNVLESSSFINRILPPPSN

SDPLQGNQIFSNVSKEYSRDGSSLMVQAQRRNYAMQSSLIEPIVPQMNSNESENSSFLHE

HQNQLPKQNQLSESSEMAPTNSHTRNDPLQNKTADKEFYFHSINLFSNKKTPTSLGGSSP

AYQRVPMAIDSSVTTVDPIHSDPLAQDISTKKAQTHAQSLGFSAHQLGNSTLIRDSATAL

NQNLFTEKTAQHFPRKALDEPSTLEANDHDDASRDIGLSNTANTSQASTSKDTATSCASD

HDLVNSCNLDNSNGTSLTSVAPQEEGKCPYIHPFTLDFAQSVEPVNPLRQYTIVFATEKA

ACMVGATSSICLFKKSEFMEENKAIWDAFRVSCEQSSRGDPNGLDGVFKERYVLLRDRGS

SLMQEIVATLPSFSSFKETLFSYFQTAYHSFTNVIDEKTVRHLLEDFVEVDNETDCIISL

NVDDRTNFFSVGIILLLYKIVKGCDVDDDCFTEFFCYIRGITSGLTFSLHRIQFLLLNYF

QRQFDPKRGAHAGSVHLLAESLGSMTDMLGFRAGVQKHYKDKFEHVGLIFTLQSIWYWSL

YADVLNSFENGKSLTVSYGSFDEEELHSLESGRVGKLKRFLVLSRKILFDLNKPRGIPDI

EGMLMQIDHYLSSELHPLKVYCAKESLNEVDIFDYVVLAPLIAMKISLWYLQIRAFKNDE

AVHKNKLFRTLVTSFKMITALSAHCSHLQASSSGVSSQWNTAEAATNIAFWLKKPLAFRS

LITLFDLMYERGKTDQKVKRGNGKYKKNLAMENLLDFEKLDAEIDCTNVQYSSSDMFGKY
[truncated: 2,586,140 more chars]
