# Supplementary material for: The Shared and Specific Genes and a Comparative Genomics Analysis within Three Hanseniaspora Strains
Source: Int J Genomics. 2019 Jun 2;2019:7910865. doi: 10.1155/2019/7910865 (PMC6589277; doi:10.1155/2019/7910865)
Supplement: Supplementary 4 — File 3: The result of all gene family analyses of K. apiculata 34-9, H. uvarum DSM 2768, and H. vineae T02/19AF. [file 7910865.f4.doc]

**Supplementary Table 3.** Result of all gene families analysis of *K. apiculata* 34-9, *H.* *uvarum*

DSM 2768, *H.* *vineae* T02/19AF**.**

1 0 13 1.000 1 3 KKA02400.1;34-9_3558;model.g3606.t1;

2 1 13 1.000 1 3 34-9_3637;KKA01304.1;model.g3640.t1;

3 2 48 1.000 1 3 KKA01318.1;34-9_3651;model.g3627.t1;

4 3 12 0.753 1 6 model.g239.t1;KKA02392.1;model.g1300.t1;34-9_1860;model.g3614.t1;34-9_1526;

5 3 14 1.000 1 4 model.g1551.t1;34-9_1095;KKA03962.1;model.g2071.t1;

6 5 19 1.000 1 3 34-9_3630;KKA01298.1;model.g3648.t1;

7 5 49 1.000 1 2 model.g2269.t1;34-9_1888;

8 7 31 1.000 1 2 34-9_0382;model.g2010.t1;

9 8 23 1.000 1 2 model.g3929.t1;34-9_0062;

10 9 76 1.000 1 3 34-9_2504;KKA03059.1;model.g3248.t1;

11 10 73 1.000 1 3 KKA02759.1;34-9_0238;model.g4587.t1;

12 11 57 1.000 1 2 34-9_3599;model.g1793.t1;

13 12 57 1.000 1 3 34-9_1002;model.g1033.t1;KKA03876.1;

14 13 65 1.000 1 3 34-9_3563;model.g1773.t1;KKA02406.1;

15 14 99 1.000 1 2 34-9_1148;KKA01236.1;

16 15 16 1.000 1 2 model.g3644.t1;34-9_3635;

17 16 4 0.376 1 10 34-9_2349;model.g3364.t1;KKA03326.1;34-9_3474;model.g459.t1;34-9_3148;34-9_0394;KKA02312.1;model.g4617.t1;model.g117.t1;

18 16 7 0.604 1 24 KKA02149.1;model.g1499.t1;34-9_2664;model.g1647.t1;model.g2247.t1;KKA01658.1;34-9_2017;KKA01611.1;34-9_1384;KKA03050.1;34-9_2497;model.g3445.t1;model.g1742.t1;34-9_0428;34-9_1484;model.g4646.t1;model.g1012.t1;model.g4654.t1;KKA02671.1;34-9_3436;KKA02576.1;34-9_1822;34-9_3347;model.g3216.t1;

19 16 25 1.000 1 3 model.g1234.t1;34-9_0173;KKA02432.1;

20 16 24 1.000 1 3 model.g3426.t1;KKA02988.1;34-9_2429;

21 16 51 1.000 1 3 KKA01102.1;model.g2587.t1;34-9_1625;

22 16 11 0.843 1 34 34-9_0920;model.g3855.t1;model.g3595.t1;model.g1120.t1;KKA02907.1;model.g79.t1;KKA03631.1;KKA03208.1;KKA03800.1;34-9_1734;34-9_3118;34-9_2181;model.g3720.t1;34-9_3163;model.g2463.t1;KKA02885.1;KKA03500.1;34-9_2759;model.g2850.t1;34-9_2720;34-9_0531;34-9_0741;model.g131.t1;KKA02251.1;model.g4251.t1;model.g3781.t1;model.g2497.t1;34-9_1028;KKA03238.1;KKA03901.1;KKA03597.1;34-9_0702;KKA01433.1;model.g2220.t1;

23 16 7 0.502 1 3 model.g2980.t1;34-9_2111;KKA02625.1;

24 16 35 1.000 1 3 model.g414.t1;KKA02730.1;34-9_0267;

25 16 7 0.667 1 4 model.g4008.t1;34-9_0129;KKA01424.1;model.g2275.t1;

26 16 6 0.420 1 8 model.g3667.t1;KKA01305.1;KKA02878.1;KKA01892.1;34-9_3638;34-9_0338;model.g3100.t1;34-9_3171;

27 16 23 1.000 1 3 model.g1269.t1;34-9_0194;KKA01092.1;

28 16 10 0.667 1 4 34-9_1754;model.g2836.t1;34-9_3332;KKA02266.1;

29 16 36 1.000 1 3 34-9_3524;KKA02364.1;model.g2440.t1;

30 16 41 1.000 1 2 model.g3955.t1;34-9_1757;

31 16 43 1.000 1 3 model.g3049.t1;34-9_0466;KKA02074.1;

32 16 66 1.000 1 2 KKA01834.1;34-9_3277;

33 34 28 1.000 1 3 KKA01307.1;34-9_3640;model.g3665.t1;

34 35 37 1.000 1 3 KKA02391.1;34-9_3549;model.g3616.t1;

35 36 36 1.000 1 2 model.g3702.t1;34-9_0684;

36 37 27 1.000 1 3 34-9_3546;KKA02387.1;model.g3620.t1;

37 38 31 1.000 1 3 34-9_3539;KKA02380.1;model.g3650.t1;

38 39 39 1.000 1 3 KKA01310.1;34-9_3643;model.g3662.t1;

39 40 46 1.000 1 2 model.g3617.t1;KKA02390.1;

40 41 18 1.000 1 2 model.g3656.t1;KKA01322.1;

41 42 36 1.000 1 3 KKA01301.1;34-9_3633;model.g3645.t1;

42 43 21 1.000 1 3 KKA01313.1;34-9_3645;model.g3628.t1;

43 44 19 1.000 1 2 model.g3670.t1;34-9_3587;

44 45 40 1.000 1 3 KKA02386.1;34-9_3545;model.g3657.t1;

45 46 10 0.502 1 3 KKA00956.1;34-9_0013;model.g3659.t1;

46 47 15 1.000 1 24 34-9_1120;model.g4020.t1;model.g3612.t1;KKA03149.1;KKA01428.1;34-9_2199;34-9_0923;34-9_2589;KKA03953.1;model.g1743.t1;model.g4433.t1;KKA01420.1;model.g2111.t1;34-9_3552;34-9_0137;KKA02394.1;KKA03987.1;KKA03479.1;model.g2056.t1;34-9_1085;model.g4230.t1;KKA03802.1;model.g305.t1;34-9_3673;

47 47 6 0.584 1 11 model.g3554.t1;34-9_2816;model.g729.t1;model.g3924.t1;34-9_1263;KKA02713.1;34-9_3373;34-9_0065;KKA03268.1;model.g3130.t1;KKA02293.1;

48 47 16 0.835 1 9 model.g3758.t1;KKA02808.1;model.g508.t1;34-9_3455;KKA02658.1;model.g2241.t1;34-9_1656;KKA01743.1;34-9_1559;

49 47 9 0.502 1 4 model.g1883.t1;KKA03796.1;34-9_0917;model.g3726.t1;

50 51 44 1.000 1 3 34-9_1096;model.g2074.t1;KKA03965.1;

51 52 4 0.388 1 9 model.g3608.t1;KKA03641.1;model.g2356.t1;34-9_0752;34-9_3501;34-9_3556;KKA02398.1;KKA02337.1;model.g1988.t1;

52 53 6 0.502 1 3 KKA01324.1;34-9_3656;model.g3633.t1;

53 53 18 1.000 1 2 KKA01323.1;model.g3632.t1;

54 55 10 1.000 1 4 KKA01299.1;34-9_3631;model.g3516.t1;model.g3647.t1;

55 55 29 1.000 1 3 34-9_1394;model.g1674.t1;KKA01663.1;

56 57 98 1.000 1 2 KKA02851.1;34-9_1601;

57 58 28 1.000 1 3 34-9_3547;KKA02388.1;model.g3619.t1;

58 59 20 1.000 1 3 34-9_3081;model.g2784.t1;KKA02925.1;

59 60 98 1.000 1 2 KKA02399.1;34-9_3557;

60 61 58 1.000 1 3 KKA01303.1;34-9_3636;model.g3643.t1;

61 62 16 1.000 1 13 34-9_0704;model.g3714.t1;KKA00998.1;34-9_1291;KKA03613.1;34-9_1290;KKA03599.1;KKA01248.1;model.g3772.t1;model.g3716.t1;KKA01246.1;34-9_0718;model.g221.t1;

62 64 6 0.502 1 3 KKA01316.1;34-9_3648;model.g3630.t1;

63 65 45 1.000 1 3 34-9_3629;KKA01297.1;model.g3649.t1;

64 66 56 1.000 1 3 34-9_3653;KKA01320.1;model.g3623.t1;

65 67 45 1.000 1 3 34-9_3542;KKA02383.1;model.g3651.t1;

66 67 26 1.000 1 3 KKA01855.1;model.g2266.t1;34-9_1885;

67 69 27 1.000 1 3 KKA02395.1;34-9_3553;model.g3611.t1;

68 70 51 1.000 1 3 KKA02396.1;34-9_3554;model.g3610.t1;

69 71 38 1.000 1 3 KKA02397.1;34-9_3555;model.g3609.t1;

70 72 37 1.000 1 2 34-9_2738;model.g924.t1;

71 73 21 1.000 1 2 34-9_0099;model.g4478.t1;

72 74 18 1.000 1 3 34-9_0840;model.g2429.t1;KKA03727.1;

73 75 18 1.000 1 2 34-9_3182;model.g1620.t1;

74 76 49 1.000 1 3 34-9_0692;KKA03587.1;model.g3710.t1;

75 77 54 1.000 1 3 34-9_0777;model.g2388.t1;KKA03668.1;

76 78 27 1.000 1 3 KKA03678.1;model.g1880.t1;34-9_0787;

77 79 23 1.000 1 3 34-9_0689;KKA03585.1;model.g3706.t1;

78 80 35 1.000 1 3 KKA03577.1;34-9_0679;model.g3699.t1;

79 81 38 1.000 1 3 KKA03560.1;34-9_0662;model.g3676.t1;

80 82 39 1.000 1 2 model.g3709.t1;34-9_1378;

81 83 45 1.000 1 3 KKA03568.1;34-9_0669;model.g3686.t1;

82 84 21 1.000 1 4 model.g2457.t1;KKA03763.1;34-9_0881;model.g3692.t1;

83 84 7 0.553 1 41 model.g1241.t1;KKA01897.1;model.g1240.t1;34-9_3535;model.g642.t1;model.g3873.t1;KKA02085.1;KKA02434.1;34-9_0169;KKA02436.1;model.g4221.t1;34-9_0724;34-9_0170;model.g1239.t1;KKA02375.1;model.g648.t1;model.g1238.t1;KKA01898.1;model.g1237.t1;KKA02437.1;model.g643.t1;KKA02090.1;KKA02435.1;34-9_3646;KKA01312.1;34-9_0478;KKA02438.1;34-9_1222;KKA01314.1;34-9_0486;KKA02459.1;34-9_1223;34-9_1969;34-9_0168;model.g1208.t1;34-9_0485;model.g649.t1;model.g1207.t1;model.g1811.t1;model.g1242.t1;34-9_0167;

84 87 22 1.000 1 3 KKA03574.1;34-9_0675;model.g3681.t1;

85 88 29 1.000 1 3 KKA03576.1;34-9_0678;model.g3685.t1;

86 89 18 1.000 1 3 34-9_0667;KKA03565.1;model.g3696.t1;

87 90 11 0.835 1 5 model.g3260.t1;KKA03579.1;34-9_2569;34-9_0681;model.g3683.t1;

88 91 16 1.000 1 2 model.g3673.t1;34-9_0659;

89 92 19 0.502 1 3 34-9_0668;KKA03567.1;KKA03566.1;

90 94 75 1.000 1 3 KKA03564.1;34-9_0666;model.g3697.t1;

91 95 22 1.000 1 3 KKA03571.1;34-9_0672;model.g3691.t1;

92 96 76 1.000 1 4 model.g2329.t1;KKA03556.1;34-9_0657;model.g3671.t1;

93 97 13 1.000 1 6 model.g2736.t1;34-9_0687;34-9_2894;KKA03583.1;KKA01480.1;model.g3705.t1;

94 97 29 1.000 1 3 model.g4209.t1;KKA03531.1;34-9_2152;

95 99 25 1.000 1 3 34-9_2861;KKA01290.1;model.g1516.t1;

96 100 15 1.000 1 3 34-9_0664;KKA03562.1;model.g3678.t1;

97 101 21 1.000 1 5 34-9_1957;KKA01563.1;KKA02467.1;model.g621.t1;model.g3693.t1;

98 102 33 1.000 1 3 34-9_0677;KKA03575.1;model.g3684.t1;

99 103 38 1.000 1 3 34-9_0636;KKA01574.1;model.g2351.t1;

100 104 17 1.000 1 3 KKA03578.1;34-9_0680;model.g3700.t1;

101 105 32 1.000 1 3 KKA03561.1;34-9_0663;model.g3677.t1;

102 106 21 1.000 1 4 KKA03557.1;34-9_0658;model.g1025.t1;model.g3672.t1;

103 107 98 1.000 1 2 34-9_0685;KKA03581.1;

104 108 17 1.000 1 8 34-9_1388;model.g1651.t1;model.g1650.t1;model.g1646.t1;KKA01657.1;model.g3695.t1;KKA01660.1;34-9_1383;

105 109 97 1.000 1 2 KKA03580.1;34-9_0682;

106 110 6 0.514 1 16 34-9_0218;model.g3698.t1;model.g389.t1;model.g1517.t1;34-9_1285;model.g2471.t1;model.g755.t1;KKA02779.1;34-9_2507;KKA03409.1;34-9_0665;34-9_2267;KKA03563.1;KKA01937.1;KKA03062.1;model.g3251.t1;

107 111 13 0.776 1 6 34-9_1702;KKA03794.1;KKA02234.1;34-9_0916;model.g495.t1;model.g3728.t1;

108 112 26 1.000 1 5 KKA02302.1;34-9_1216;34-9_3463;model.g4339.t1;model.g3740.t1;

109 112 6 0.557 1 9 34-9_3126;KKA01277.1;34-9_2872;model.g422.t1;KKA02876.1;model.g859.t1;model.g1610.t1;model.g90.t1;34-9_0274;

110 112 11 0.918 1 10 KKA01541.1;KKA03969.1;model.g2507.t1;34-9_1101;model.g2079.t1;34-9_3591;model.g1781.t1;model.g1782.t1;KKA03214.1;34-9_1519;

111 112 4 0.361 1 9 KKA01492.1;KKA03932.1;model.g1048.t1;model.g2034.t1;34-9_1063;KKA03982.1;34-9_1116;model.g2721.t1;model.g865.t1;

112 112 5 0.396 1 11 KKA01479.1;KKA03970.1;model.g2737.t1;model.g1871.t1;model.g505.t1;34-9_1102;34-9_0857;34-9_2893;model.g2080.t1;model.g2916.t1;KKA03740.1;

113 112 23 1.000 1 3 model.g1078.t1;34-9_2657;KKA02153.1;

114 112 83 1.000 1 3 KKA03745.1;34-9_0863;model.g2399.t1;

115 112 72 1.000 1 3 model.g2253.t1;34-9_3268;KKA01842.1;

116 112 33 1.000 1 3 KKA01493.1;34-9_2908;model.g2720.t1;

117 112 46 1.000 1 2 34-9_3101;model.g38.t1;

118 112 26 1.000 1 3 model.g1611.t1;KKA02875.1;34-9_3176;

119 123 10 0.667 1 9 model.g3736.t1;model.g4234.t1;model.g3313.t1;KKA03482.1;KKA01338.1;34-9_3517;KKA02352.1;34-9_2196;34-9_3757;

120 124 54 1.000 1 2 model.g3725.t1;KKA03797.1;

121 125 25 1.000 1 3 KKA03145.1;model.g1721.t1;34-9_3679;

122 126 14 1.000 1 2 model.g3738.t1;KKA02354.1;

123 126 37 1.000 1 3 34-9_2955;KKA01167.1;model.g1327.t1;

124 128 44 1.000 1 2 model.g3727.t1;KKA03795.1;

125 129 38 1.000 1 3 34-9_3513;KKA02348.1;model.g3731.t1;

126 130 22 1.000 1 2 model.g3717.t1;34-9_0960;

127 131 15 1.000 1 3 KKA02349.1;34-9_3514;model.g3732.t1;

128 132 25 1.000 1 2 model.g1104.t1;KKA01062.1;

129 133 56 1.000 1 3 KKA01850.1;34-9_3260;model.g3718.t1;

130 134 53 1.000 1 3 KKA02353.1;34-9_3518;model.g3737.t1;

131 135 25 1.000 1 3 KKA02351.1;34-9_3516;model.g3733.t1;

132 136 33 1.000 1 3 34-9_1356;model.g2609.t1;KKA01945.1;

133 137 15 0.502 1 4 KKA02356.1;34-9_3519;KKA02355.1;model.g3739.t1;

134 138 33 1.000 1 3 34-9_2437;KKA02997.1;model.g3744.t1;

135 139 78 1.000 1 3 KKA03000.1;34-9_2440;model.g3747.t1;

136 140 26 1.000 1 3 KKA02995.1;34-9_2435;model.g3742.t1;

137 141 18 1.000 1 6 model.g946.t1;KKA02998.1;34-9_1882;34-9_2438;KKA02218.1;model.g3745.t1;

138 141 5 0.467 1 23 model.g525.t1;34-9_1060;KKA02228.1;model.g2745.t1;KKA03325.1;model.g2009.t1;model.g266.t1;KKA02317.1;KKA03929.1;model.g905.t1;model.g2474.t1;34-9_3479;34-9_0313;KKA02538.1;KKA01473.1;34-9_3666;KKA03156.1;34-9_3214;34-9_2350;34-9_2886;34-9_0418;model.g600.t1;model.g3365.t1;

139 141 27 1.000 1 3 KKA02914.1;34-9_3104;model.g34.t1;

140 141 35 1.000 1 2 34-9_0621;model.g277.t1;

141 145 7 0.667 1 4 34-9_0915;KKA03793.1;model.g1675.t1;model.g1975.t1;

142 145 18 1.000 1 3 34-9_1345;model.g349.t1;KKA01955.1;

143 147 53 1.000 1 2 34-9_2434;KKA02994.1;

144 148 84 1.000 1 3 KKA02996.1;34-9_2436;model.g3743.t1;

145 149 27 1.000 1 3 KKA02999.1;34-9_2439;model.g3746.t1;

146 150 17 1.000 1 2 model.g3752.t1;34-9_1565;

147 151 39 1.000 1 2 model.g3762.t1;34-9_1555;

148 152 17 1.000 1 6 34-9_3087;34-9_1570;KKA02923.1;KKA02818.1;model.g2794.t1;model.g3749.t1;

149 153 7 0.502 1 3 model.g1211.t1;model.g1210.t1;34-9_1961;

150 154 39 1.000 1 3 KKA02802.1;34-9_1552;model.g3765.t1;

151 155 41 1.000 1 3 34-9_2840;model.g2950.t1;KKA01151.1;

152 156 29 1.000 1 2 model.g3766.t1;KKA02801.1;

153 157 21 1.000 1 3 KKA02819.1;34-9_1571;model.g3748.t1;

154 158 31 1.000 1 3 KKA02804.1;34-9_1554;model.g3763.t1;

155 159 20 1.000 1 3 34-9_1560;KKA02809.1;model.g3757.t1;

156 160 31 1.000 1 3 KKA02812.1;34-9_1563;model.g3754.t1;

157 161 30 1.000 1 3 34-9_1561;KKA02810.1;model.g3756.t1;

158 162 49 1.000 1 2 model.g3751.t1;34-9_1577;

159 163 36 1.000 1 3 34-9_1562;KKA02811.1;model.g3755.t1;

160 164 85 1.000 1 3 KKA02806.1;34-9_1557;model.g3760.t1;

161 165 11 1.000 1 6 KKA01408.1;model.g4001.t1;model.g4343.t1;34-9_1213;KKA01763.1;34-9_0158;

162 166 45 1.000 1 3 KKA03154.1;model.g3140.t1;34-9_3668;

163 167 87 1.000 1 3 KKA02813.1;34-9_1564;model.g3753.t1;

164 168 35 1.000 1 3 KKA02805.1;34-9_1556;model.g3761.t1;

165 169 51 1.000 1 3 KKA02807.1;34-9_1558;model.g3759.t1;

166 170 23 1.000 1 3 34-9_3073;model.g2775.t1;KKA02928.1;

167 171 32 1.000 1 3 KKA03622.1;34-9_0730;model.g3798.t1;

168 172 16 1.000 1 2 model.g3805.t1;34-9_0736;

169 173 30 1.000 1 3 34-9_0696;KKA03590.1;model.g3787.t1;

170 174 5 0.502 1 3 34-9_0707;KKA03602.1;model.g3773.t1;

171 175 37 1.000 1 3 34-9_0706;KKA03601.1;model.g3774.t1;

172 176 43 1.000 1 3 KKA03629.1;34-9_0738;model.g3807.t1;

173 177 22 1.000 1 3 KKA03593.1;34-9_0698;model.g3783.t1;

174 178 6 0.557 1 6 KKA02659.1;KKA03767.1;model.g2240.t1;34-9_0885;34-9_3454;model.g3809.t1;

175 179 16 1.000 1 8 model.g410.t1;34-9_3033;34-9_3035;34-9_3034;KKA02953.1;model.g411.t1;model.g976.t1;model.g975.t1;

176 180 12 0.965 1 16 KKA01430.1;model.g3780.t1;34-9_0045;model.g1001.t1;34-9_0601;model.g2397.t1;34-9_0526;model.g2398.t1;model.g2952.t1;model.g3826.t1;KKA03598.1;34-9_3446;34-9_0703;KKA01431.1;34-9_2833;KKA02665.1;

177 181 6 0.502 1 7 model.g2963.t1;34-9_0184;34-9_1340;model.g344.t1;34-9_2065;model.g1223.t1;KKA01960.1;

178 182 39 1.000 1 3 KKA03617.1;34-9_0723;model.g3790.t1;

179 183 73 1.000 1 2 model.g3792.t1;34-9_0726;

180 184 87 1.000 1 2 34-9_0699;KKA03594.1;

181 185 10 1.000 1 3 34-9_3759;KKA01340.1;model.g1629.t1;

182 186 10 1.000 1 2 model.g3803.t1;34-9_0734;

183 187 25 1.000 1 3 34-9_0732;KKA03624.1;model.g3800.t1;

184 188 11 1.000 1 4 34-9_0735;KKA03626.1;model.g853.t1;model.g3804.t1;

185 189 66 1.000 1 3 KKA03620.1;34-9_0728;model.g3795.t1;

186 190 11 1.000 1 3 KKA03625.1;34-9_0733;model.g3802.t1;

187 191 46 1.000 1 4 model.g3810.t1;KKA03762.1;34-9_0880;model.g3811.t1;

188 192 16 1.000 1 3 34-9_1712;model.g2226.t1;model.g2884.t1;

189 193 17 1.000 1 2 model.g3414.t1;KKA03124.1;

190 193 33 1.000 1 3 34-9_2574;model.g3415.t1;KKA03123.1;

191 195 50 1.000 1 3 34-9_0709;KKA03604.1;model.g3771.t1;

192 196 19 1.000 1 3 KKA03600.1;34-9_0705;model.g3775.t1;

193 197 29 1.000 1 3 KKA03616.1;34-9_0721;model.g3789.t1;

194 198 24 1.000 1 3 KKA03592.1;34-9_0697;model.g3786.t1;

195 198 29 1.000 1 2 34-9_2679;model.g1664.t1;

196 200 14 1.000 1 3 34-9_0720;KKA03615.1;model.g3788.t1;

197 201 30 1.000 1 4 34-9_0722;KKA03596.1;34-9_0701;model.g3782.t1;

198 202 32 1.000 1 3 KKA03619.1;34-9_0727;model.g3794.t1;

199 203 40 1.000 1 3 KKA01982.1;model.g1887.t1;34-9_1320;

200 204 17 1.000 1 3 34-9_1912;model.g2288.t1;KKA01883.1;

201 205 14 1.000 1 2 model.g3806.t1;KKA03627.1;

202 206 22 1.000 1 3 KKA03621.1;34-9_0729;model.g3796.t1;

203 207 14 1.000 1 3 KKA02292.1;34-9_0064;model.g3926.t1;

204 207 76 1.000 1 2 34-9_2548;KKA03101.1;

205 209 27 1.000 1 3 34-9_1012;KKA03885.1;model.g3816.t1;

206 209 28 1.000 1 3 34-9_0540;model.g3602.t1;KKA01438.1;

207 211 6 0.502 1 9 model.g4442.t1;model.g3483.t1;34-9_2774;34-9_3257;KKA01047.1;KKA03249.1;model.g4133.t1;KKA02512.1;34-9_2403;

208 212 88 1.000 1 2 KKA03883.1;34-9_1010;

209 213 35 1.000 1 3 KKA03882.1;34-9_1009;model.g3813.t1;

210 214 52 1.000 1 3 KKA03884.1;34-9_1011;model.g3815.t1;

211 215 34 1.000 1 3 KKA02751.1;34-9_0247;model.g3819.t1;

212 216 19 1.000 1 3 34-9_0246;KKA02752.1;model.g3820.t1;

213 217 30 1.000 1 5 model.g521.t1;model.g524.t1;model.g523.t1;model.g522.t1;model.g3823.t1;

214 218 53 1.000 1 3 34-9_0243;KKA02754.1;model.g3825.t1;

215 219 50 1.000 1 3 34-9_1632;KKA01112.1;model.g3817.t1;

216 221 37 1.000 1 3 34-9_0242;KKA02755.1;model.g3824.t1;

217 222 6 0.490 1 16 KKA03666.1;model.g2383.t1;model.g3601.t1;model.g3821.t1;KKA03941.1;34-9_0774;model.g3828.t1;34-9_3021;KKA03280.1;model.g2041.t1;34-9_2832;34-9_0245;model.g960.t1;34-9_1073;KKA02963.1;34-9_0538;

218 222 14 1.000 1 2 34-9_0303;model.g535.t1;

219 224 37 1.000 1 3 model.g3283.t1;34-9_0014;model.g3818.t1;

220 225 41 1.000 1 3 KKA03279.1;34-9_2830;model.g3830.t1;

221 226 15 1.000 1 5 34-9_1693;KKA02231.1;model.g1322.t1;model.g2936.t1;34-9_2968;

222 227 28 1.000 1 2 model.g4189.t1;34-9_0710;

223 228 93 1.000 1 3 KKA02553.1;34-9_3194;model.g3851.t1;

224 229 66 1.000 1 2 model.g3839.t1;KKA03158.1;

225 230 22 1.000 1 3 34-9_2829;KKA03278.1;model.g3831.t1;

226 231 50 1.000 1 2 model.g3829.t1;34-9_2831;

227 232 22 1.000 1 2 model.g3846.t1;34-9_3690;

228 233 73 1.000 1 2 34-9_3664;KKA03157.1;

229 234 26 1.000 1 3 34-9_3689;KKA01041.1;model.g3845.t1;

230 235 14 1.000 1 8 KKA03818.1;34-9_3195;model.g1918.t1;34-9_2274;34-9_0940;model.g3852.t1;KKA03401.1;model.g2131.t1;

231 235 14 0.875 1 19 KKA02602.1;KKA03834.1;34-9_0956;KKA02584.1;34-9_2067;34-9_3775;model.g2145.t1;34-9_2727;34-9_0093;KKA01355.1;34-9_1707;34-9_2034;model.g480.t1;model.g849.t1;model.g2962.t1;model.g1111.t1;model.g2923.t1;model.g3295.t1;KKA02103.1;

232 235 29 1.000 1 2 KKA02104.1;model.g1112.t1;

233 235 59 1.000 1 3 KKA02975.1;34-9_2415;model.g4121.t1;

234 235 82 1.000 1 2 34-9_1613;model.g3527.t1;

235 235 63 1.000 1 3 model.g793.t1;KKA03349.1;34-9_2325;

236 241 19 1.000 1 3 KKA03159.1;34-9_3662;model.g3840.t1;

237 242 35 1.000 1 3 34-9_3193;KKA02554.1;model.g3848.t1;

238 243 51 1.000 1 3 model.g4472.t1;34-9_0101;KKA02047.1;

239 244 14 1.000 1 2 model.g3841.t1;34-9_3661;

240 245 60 1.000 1 2 model.g3858.t1;34-9_1025;

241 246 21 1.000 1 5 34-9_1015;KKA03888.1;34-9_1017;model.g3868.t1;KKA03891.1;

242 247 28 1.000 1 3 34-9_1026;KKA03899.1;model.g3857.t1;

243 248 19 1.000 1 3 34-9_0393;KKA01531.1;model.g456.t1;

244 249 17 1.000 1 6 KKA01517.1;model.g4152.t1;model.g81.t1;34-9_0372;KKA03281.1;34-9_2398;

245 250 32 1.000 1 3 KKA03892.1;34-9_1018;model.g3866.t1;

246 251 17 1.000 1 3 34-9_1024;KKA03898.1;model.g3859.t1;

247 252 35 1.000 1 3 KKA03896.1;34-9_1022;model.g3861.t1;

248 253 12 1.000 1 5 34-9_1016;model.g4707.t1;KKA03889.1;34-9_0348;model.g3869.t1;

249 254 10 1.000 1 3 34-9_1023;KKA03897.1;model.g3860.t1;

250 255 5 0.502 1 7 model.g14.t1;34-9_0244;34-9_0772;KKA02753.1;model.g2380.t1;model.g3865.t1;KKA03664.1;

251 256 21 1.000 1 4 KKA03886.1;34-9_1013;model.g2154.t1;model.g3871.t1;

252 257 22 1.000 1 3 34-9_1021;KKA03895.1;model.g3862.t1;

253 258 33 1.000 1 3 34-9_1014;KKA03887.1;model.g3870.t1;

254 259 25 1.000 1 3 KKA01899.1;34-9_1224;model.g3882.t1;

255 260 8 0.667 1 6 model.g3874.t1;model.g3875.t1;34-9_3496;34-9_3495;KKA02332.1;model.g3876.t1;

256 260 25 1.000 1 2 34-9_1995;KKA02441.1;

257 262 36 1.000 1 3 KKA02329.1;34-9_3492;model.g3878.t1;

258 262 22 1.000 1 8 model.g1668.t1;KKA02130.1;model.g1978.t1;model.g4447.t1;KKA02345.1;34-9_2770;34-9_2693;34-9_3510;

259 262 44 1.000 1 2 model.g1392.t1;KKA01565.1;

260 265 20 1.000 1 3 KKA02328.1;34-9_3491;model.g3877.t1;

261 266 17 1.000 1 11 34-9_3162;KKA02886.1;KKA01780.1;34-9_1156;model.g1445.t1;34-9_1189;model.g4392.t1;KKA02651.1;KKA01801.1;model.g3880.t1;model.g130.t1;

262 267 5 0.435 1 33 34-9_1865;model.g3343.t1;model.g2376.t1;model.g3881.t1;KKA01973.1;34-9_1773;KKA01998.1;KKA01637.1;34-9_2772;34-9_0131;model.g4446.t1;model.g4012.t1;34-9_3012;34-9_1326;model.g2759.t1;34-9_0768;model.g1885.t1;34-9_1225;KKA03660.1;34-9_3020;model.g1019.t1;KKA01900.1;model.g1311.t1;model.g3190.t1;model.g139.t1;KKA01206.1;model.g4373.t1;KKA02208.1;34-9_2367;KKA03248.1;model.g358.t1;34-9_2796;34-9_2601;

263 267 6 0.463 1 14 model.g1027.t1;KKA02014.1;model.g1598.t1;34-9_0822;34-9_1790;model.g3147.t1;KKA01930.1;model.g4667.t1;KKA01929.1;model.g1828.t1;34-9_3528;34-9_1269;KKA02369.1;model.g2449.t1;

264 267 58 1.000 1 3 34-9_2846;model.g1535.t1;KKA01156.1;

265 267 7 0.502 1 3 KKA01928.1;model.g4716.t1;34-9_1268;

266 267 24 1.000 1 3 KKA02255.1;34-9_1739;model.g2845.t1;

267 274 42 1.000 1 3 34-9_0437;KKA02497.1;model.g3886.t1;

268 275 12 1.000 1 3 34-9_3232;KKA02528.1;model.g3897.t1;

269 276 38 1.000 1 2 model.g3900.t1;34-9_1869;

270 277 23 1.000 1 2 model.g3898.t1;34-9_1867;

271 278 10 1.000 1 2 model.g3885.t1;34-9_0436;

272 279 59 1.000 1 3 KKA02191.1;34-9_1835;model.g3893.t1;

273 280 24 1.000 1 2 model.g3896.t1;34-9_0536;

274 281 17 1.000 1 2 model.g3894.t1;34-9_3233;

275 282 59 1.000 1 3 KKA02210.1;34-9_1868;model.g3899.t1;

276 283 44 1.000 1 6 model.g2392.t1;34-9_1830;KKA01144.1;model.g2814.t1;model.g3892.t1;34-9_3744;

277 284 61 1.000 1 2 model.g3888.t1;34-9_1863;

278 285 17 1.000 1 3 34-9_2469;KKA03026.1;model.g4107.t1;

279 286 11 0.773 1 25 model.g3028.t1;KKA02346.1;34-9_2909;34-9_0769;model.g1458.t1;model.g1977.t1;34-9_3281;KKA01494.1;KKA02743.1;model.g2719.t1;34-9_3511;34-9_0254;model.g2377.t1;model.g2352.t1;model.g385.t1;34-9_0635;KKA01573.1;KKA01245.1;model.g4261.t1;KKA03505.1;KKA00997.1;34-9_1289;KKA03661.1;34-9_2177;KKA01830.1;

280 287 95 1.000 1 2 KKA02207.1;34-9_1861;

281 288 51 1.000 1 2 model.g3914.t1;34-9_1505;

282 289 40 1.000 1 3 KKA03203.1;34-9_1501;model.g3910.t1;

283 290 27 1.000 1 3 KKA03204.1;34-9_1502;model.g3911.t1;

284 291 46 1.000 1 3 model.g4298.t1;34-9_2217;KKA03460.1;

285 292 40 1.000 1 2 34-9_2711;model.g1133.t1;

286 293 25 1.000 1 2 model.g3909.t1;34-9_1500;

287 294 11 1.000 1 3 34-9_1495;KKA03199.1;model.g3904.t1;

288 295 33 1.000 1 3 KKA03205.1;34-9_1504;model.g3913.t1;

289 295 43 1.000 1 3 KKA03373.1;model.g3353.t1;34-9_2300;

290 297 8 0.667 1 12 34-9_1759;KKA02267.1;model.g3963.t1;KKA02342.1;34-9_1750;model.g1982.t1;34-9_1499;model.g3978.t1;KKA03202.1;KKA02264.1;model.g3908.t1;34-9_3507;

291 298 33 1.000 1 3 KKA03200.1;34-9_1497;model.g3906.t1;

292 299 21 1.000 1 3 KKA02277.1;34-9_0041;model.g3962.t1;

293 299 47 1.000 1 4 model.g1546.t1;model.g1547.t1;KKA02276.1;KKA02275.1;

294 301 27 1.000 1 3 KKA02280.1;34-9_0049;model.g3946.t1;

295 302 28 1.000 1 3 34-9_0743;KKA03633.1;model.g3982.t1;

296 303 97 1.000 1 2 KKA03040.1;34-9_2484;

297 304 6 0.502 1 5 34-9_0048;model.g1455.t1;KKA01827.1;model.g3949.t1;34-9_3283;

298 305 35 1.000 1 3 34-9_0053;KKA02286.1;model.g3938.t1;

299 306 15 1.000 1 2 model.g3933.t1;34-9_0057;

300 307 4 0.420 1 7 34-9_0056;model.g2193.t1;KKA02288.1;model.g1544.t1;34-9_0992;model.g3934.t1;KKA03864.1;

301 307 52 1.000 1 8 KKA02889.1;model.g128.t1;KKA02887.1;34-9_3161;34-9_3160;model.g129.t1;34-9_3159;KKA02888.1;

302 309 18 1.000 1 2 model.g3921.t1;34-9_0068;

303 310 36 1.000 1 3 KKA01125.1;34-9_2633;model.g3919.t1;

304 311 12 1.000 1 2 model.g3972.t1;34-9_1758;

305 312 31 1.000 1 3 KKA01630.1;34-9_2983;model.g4056.t1;

306 313 99 1.000 1 2 KKA02033.1;34-9_0118;

307 314 62 1.000 1 2 model.g4271.t1;KKA03510.1;

308 314 31 1.000 1 3 model.g4272.t1;KKA03509.1;34-9_2172;

309 316 21 1.000 1 3 KKA03076.1;model.g3224.t1;34-9_2523;

310 317 16 0.502 1 3 34-9_0252;KKA02746.1;KKA02745.1;

311 319 11 1.000 1 6 KKA03870.1;34-9_1764;34-9_0998;KKA02272.1;model.g2200.t1;model.g3961.t1;

312 320 19 1.000 1 2 model.g3952.t1;34-9_0044;

313 321 22 1.000 1 2 model.g3950.t1;34-9_0046;

314 322 51 1.000 1 6 34-9_0495;model.g3981.t1;KKA02098.1;model.g3979.t1;model.g3091.t1;model.g3980.t1;

315 322 32 1.000 1 7 model.g1076.t1;34-9_2658;KKA02155.1;model.g2257.t1;model.g4360.t1;KKA02152.1;model.g2874.t1;

316 322 37 1.000 1 4 model.g4100.t1;KKA03022.1;34-9_2465;model.g4101.t1;

317 322 65 1.000 1 2 model.g3087.t1;KKA00983.1;

318 322 28 1.000 1 3 34-9_0497;model.g3088.t1;KKA00982.1;

319 327 22 1.000 1 4 model.g3927.t1;KKA02279.1;34-9_0047;model.g3936.t1;

320 328 26 1.000 1 6 KKA03048.1;KKA02287.1;34-9_2495;34-9_0055;model.g3213.t1;model.g3935.t1;

321 329 23 1.000 1 3 KKA02274.1;34-9_1766;model.g3958.t1;

322 330 22 1.000 1 3 34-9_0613;KKA01721.1;model.g1411.t1;

323 331 33 1.000 1 3 KKA02283.1;34-9_0051;model.g3941.t1;

324 333 22 1.000 1 3 34-9_0058;KKA02289.1;model.g3932.t1;

325 334 45 1.000 1 2 model.g3956.t1;34-9_1756;

326 335 13 1.000 1 7 KKA03780.1;KKA02294.1;34-9_0901;34-9_0066;model.g1962.t1;model.g3923.t1;model.g1298.t1;

327 336 28 1.000 1 2 34-9_3080;model.g2783.t1;

328 337 41 1.000 1 2 34-9_2649;model.g1066.t1;

329 338 99 1.000 1 2 34-9_1751;KKA02265.1;

330 339 39 1.000 1 2 model.g3969.t1;34-9_0042;

331 340 47 1.000 1 3 KKA02270.1;34-9_1762;model.g3966.t1;

332 341 37 1.000 1 3 KKA02263.1;34-9_1748;model.g3976.t1;

333 342 24 1.000 1 2 model.g3939.t1;KKA02285.1;

334 343 31 1.000 1 3 KKA02296.1;34-9_0069;model.g3920.t1;

335 344 9 0.557 1 6 34-9_0060;KKA01845.1;KKA02290.1;34-9_3265;34-9_0059;model.g3931.t1;

336 345 8 0.502 1 6 34-9_1420;model.g323.t1;model.g1709.t1;KKA01680.1;KKA01979.1;KKA01978.1;

337 346 80 1.000 1 3 KKA02295.1;34-9_0067;model.g3922.t1;

338 347 14 1.000 1 2 model.g3984.t1;34-9_1752;

339 348 18 1.000 1 4 KKA03434.1;34-9_2243;model.g747.t1;model.g3925.t1;

340 349 10 0.753 1 4 34-9_0063;34-9_3110;model.g65.t1;model.g3928.t1;

341 350 8 0.667 1 5 34-9_1237;model.g2748.t1;KKA01907.1;KKA01470.1;model.g2005.t1;

342 351 23 1.000 1 2 model.g3970.t1;34-9_0043;

343 352 10 1.000 1 2 model.g3977.t1;34-9_1749;

344 353 41 1.000 1 4 KKA02271.1;34-9_1763;model.g674.t1;model.g3967.t1;

345 354 77 1.000 1 2 model.g3915.t1;model.g1057.t1;

346 355 90 1.000 1 2 KKA02268.1;34-9_1760;

347 356 23 1.000 1 6 model.g966.t1;KKA02262.1;KKA02959.1;34-9_1747;34-9_3024;model.g3975.t1;

348 357 9 0.373 1 12 KKA03991.1;KKA03990.1;34-9_1125;34-9_1123;KKA01212.1;model.g1055.t1;KKA03992.1;model.g4265.t1;model.g4059.t1;34-9_1124;model.g3986.t1;model.g4263.t1;

349 357 28 1.000 1 3 KKA02357.1;34-9_3520;KKA02358.1;

350 360 28 1.000 1 3 KKA01406.1;34-9_0161;model.g3998.t1;

351 361 50 1.000 1 2 model.g3993.t1;34-9_0166;

352 362 43 1.000 1 3 KKA01422.1;34-9_0132;model.g4013.t1;

353 363 16 1.000 1 2 model.g4016.t1;34-9_0135;

354 364 19 1.000 1 2 model.g4028.t1;34-9_0145;

355 365 82 1.000 1 3 KKA01410.1;34-9_0156;model.g4003.t1;

356 366 9 0.776 1 6 model.g1073.t1;KKA01402.1;KKA02156.1;34-9_0165;34-9_2656;model.g3994.t1;

357 367 19 1.000 1 2 model.g4005.t1;34-9_0126;

358 368 19 1.000 1 2 model.g4026.t1;34-9_0143;

359 369 62 1.000 1 3 34-9_3582;model.g1754.t1;KKA01535.1;

360 370 34 1.000 1 3 KKA01404.1;34-9_0163;model.g3996.t1;

361 371 33 1.000 1 4 KKA01426.1;34-9_0125;KKA00986.1;model.g4004.t1;

362 372 68 1.000 1 3 KKA01416.1;34-9_0144;model.g4027.t1;

363 373 30 1.000 1 3 KKA01418.1;34-9_0140;model.g4023.t1;

364 373 21 1.000 1 2 model.g104.t1;34-9_3136;

365 375 10 1.000 1 3 34-9_3102;KKA02915.1;model.g39.t1;

366 376 31 1.000 1 2 model.g4015.t1;34-9_0134;

367 377 79 1.000 1 2 model.g4029.t1;34-9_0147;

368 378 43 1.000 1 3 KKA01421.1;34-9_0133;model.g4014.t1;

369 379 84 1.000 1 2 model.g4006.t1;34-9_0127;

370 380 71 1.000 1 2 model.g142.t1;34-9_0443;

371 380 8 0.714 1 10 34-9_0975;model.g1042.t1;KKA01767.1;34-9_1204;34-9_0654;KKA03851.1;model.g2333.t1;model.g4362.t1;KKA03554.1;KKA01043.1;

372 382 39 1.000 1 3 KKA01407.1;34-9_0159;model.g4000.t1;

373 383 6 0.502 1 3 KKA01409.1;34-9_0157;model.g4002.t1;

374 384 22 1.000 1 4 34-9_0331;34-9_3173;model.g1608.t1;model.g3987.t1;

375 385 18 1.000 1 2 model.g3992.t1;34-9_0335;

376 386 16 1.000 1 2 model.g4010.t1;KKA01423.1;

377 387 16 1.000 1 3 KKA01405.1;34-9_0162;model.g3999.t1;

378 388 18 1.000 1 5 34-9_0332;model.g4068.t1;34-9_1132;model.g3989.t1;KKA01220.1;

379 389 40 1.000 1 2 model.g3990.t1;34-9_0333;

380 390 17 1.000 1 3 KKA01403.1;34-9_0164;model.g3995.t1;

381 391 30 1.000 1 2 model.g4019.t1;34-9_0136;

382 392 13 1.000 1 2 model.g4022.t1;34-9_0139;

383 393 12 1.000 1 2 model.g3991.t1;34-9_0334;

384 394 32 1.000 1 3 34-9_0128;KKA01425.1;model.g4007.t1;

385 395 13 1.000 1 2 model.g4047.t1;34-9_2010;

386 396 21 1.000 1 2 model.g4057.t1;34-9_2984;

387 397 30 1.000 1 3 34-9_2012;KKA02574.1;model.g4049.t1;

388 398 20 1.000 1 2 model.g4051.t1;34-9_0071;

389 399 45 1.000 1 2 model.g4031.t1;model.g1293.t1;

390 400 24 1.000 1 3 34-9_2001;KKA02564.1;model.g4035.t1;

391 401 15 1.000 1 3 KKA02573.1;34-9_2011;model.g4048.t1;

392 402 42 1.000 1 2 model.g4050.t1;34-9_2013;

393 403 27 1.000 1 2 model.g4037.t1;34-9_1999;

394 404 23 1.000 1 3 KKA02571.1;34-9_2008;model.g4045.t1;

395 405 35 1.000 1 3 KKA02569.1;34-9_2005;model.g4041.t1;

396 406 68 1.000 1 3 KKA01629.1;34-9_2982;model.g4055.t1;

397 407 72 1.000 1 3 KKA01628.1;34-9_2980;model.g4053.t1;

398 408 22 1.000 1 6 KKA02455.1;34-9_0123;34-9_1977;KKA00987.1;model.g1200.t1;model.g4042.t1;

399 408 31 1.000 1 2 KKA02454.1;model.g1198.t1;

400 410 33 1.000 1 3 34-9_2865;model.g704.t1;KKA01285.1;

401 412 70 1.000 1 2 model.g4719.t1;34-9_3320;

402 413 58 1.000 1 2 model.g4044.t1;34-9_2007;

403 414 18 1.000 1 2 34-9_2003;model.g4033.t1;

404 415 11 0.667 1 4 KKA02568.1;model.g4039.t1;KKA02567.1;34-9_2004;

405 417 16 1.000 1 3 34-9_0070;KKA02297.1;model.g4052.t1;

406 418 67 1.000 1 3 KKA02565.1;34-9_2002;model.g4034.t1;

407 419 48 1.000 1 3 KKA01231.1;34-9_1143;model.g4083.t1;

408 420 14 0.800 1 8 34-9_0780;KKA01239.1;KKA03671.1;34-9_1152;model.g2394.t1;model.g4092.t1;model.g1410.t1;34-9_2550;

409 421 30 1.000 1 3 34-9_1140;KKA01228.1;model.g4080.t1;

410 422 26 1.000 1 3 KKA01229.1;34-9_1141;model.g4081.t1;

411 423 99 1.000 1 2 34-9_1127;KKA01214.1;

412 424 56 1.000 1 3 34-9_1130;KKA01218.1;model.g4066.t1;

413 425 66 1.000 1 2 KKA01240.1;34-9_1153;

414 426 14 1.000 1 4 KKA01221.1;34-9_1133;model.g462.t1;model.g4069.t1;

415 427 49 1.000 1 3 KKA01233.1;34-9_1145;model.g4086.t1;

416 428 9 0.667 1 6 34-9_3709;34-9_1128;KKA01460.1;KKA01215.1;model.g888.t1;model.g4063.t1;

417 428 20 1.000 1 2 KKA01216.1;model.g4064.t1;

418 430 27 1.000 1 3 34-9_0189;model.g1261.t1;KKA01101.1;

419 431 53 1.000 1 3 34-9_1135;KKA01223.1;model.g4074.t1;

420 432 26 1.000 1 3 KKA03206.1;34-9_1507;model.g4070.t1;

421 433 18 1.000 1 3 34-9_1126;KKA01213.1;model.g4060.t1;

422 434 12 0.502 1 3 34-9_1134;KKA01222.1;model.g4071.t1;

423 435 29 1.000 1 3 34-9_1137;KKA01225.1;model.g4077.t1;

424 436 27 1.000 1 3 34-9_1146;KKA01234.1;model.g4087.t1;

425 437 28 1.000 1 2 KKA03503.1;model.g4258.t1;

426 438 34 1.000 1 3 KKA01230.1;34-9_1142;model.g4082.t1;

427 439 11 1.000 1 2 model.g839.t1;34-9_2091;

428 440 21 1.000 1 3 34-9_1139;KKA01227.1;model.g4079.t1;

429 441 18 1.000 1 2 model.g4090.t1;34-9_1150;

430 442 29 1.000 1 4 34-9_1131;KKA01219.1;model.g1597.t1;model.g4067.t1;

431 443 11 1.000 1 3 34-9_1147;KKA01235.1;model.g4088.t1;

432 444 75 1.000 1 3 KKA01224.1;34-9_1136;model.g4076.t1;

433 445 51 1.000 1 3 34-9_1129;KKA01217.1;model.g4065.t1;

434 446 77 1.000 1 3 KKA01237.1;34-9_1149;model.g4089.t1;

435 447 13 1.000 1 3 34-9_2553;KKA03106.1;model.g4168.t1;

436 448 48 1.000 1 4 KKA01050.1;KKA02968.1;34-9_2406;model.g4130.t1;

437 449 33 1.000 1 3 KKA03110.1;34-9_2557;model.g4164.t1;

438 450 20 1.000 1 4 KKA01051.1;KKA02969.1;34-9_2407;model.g4129.t1;

439 451 29 1.000 1 4 34-9_2405;KKA01049.1;KKA02967.1;model.g4131.t1;

440 452 86 1.000 1 2 34-9_2388;KKA03288.1;

441 453 13 1.000 1 3 34-9_2408;KKA02970.1;model.g4128.t1;

442 454 74 1.000 1 3 KKA02971.1;34-9_2409;model.g4126.t1;

443 455 15 1.000 1 3 34-9_2474;KKA03031.1;model.g4112.t1;

444 456 31 1.000 1 2 model.g4134.t1;34-9_2399;

445 457 24 1.000 1 3 34-9_2547;KKA03100.1;model.g4147.t1;

446 458 97 1.000 1 2 KKA03099.1;34-9_2546;

447 459 15 1.000 1 3 34-9_2541;KKA03094.1;model.g4139.t1;

448 460 65 1.000 1 2 model.g4120.t1;34-9_2414;

449 461 41 1.000 1 3 KKA03029.1;34-9_2472;model.g4110.t1;

450 462 58 1.000 1 3 KKA03283.1;34-9_2394;model.g4156.t1;

451 463 30 1.000 1 3 KKA03108.1;34-9_2555;model.g4166.t1;

452 464 17 1.000 1 3 KKA03021.1;34-9_2462;model.g4097.t1;

453 465 43 1.000 1 3 34-9_2404;KKA01048.1;model.g4132.t1;

454 466 78 1.000 1 3 KKA03112.1;34-9_2560;model.g4162.t1;

455 467 11 1.000 1 3 KKA03020.1;34-9_2461;model.g4096.t1;

456 468 69 1.000 1 2 model.g4155.t1;34-9_2395;

457 469 15 1.000 1 3 KKA03092.1;34-9_2539;model.g4151.t1;

458 470 16 1.000 1 18 KKA01703.1;model.g3245.t1;34-9_0582;KKA02133.1;34-9_0427;34-9_0908;34-9_2502;model.g2867.t1;model.g1096.t1;KKA02493.1;model.g1968.t1;model.g4534.t1;KKA03057.1;34-9_1722;34-9_3382;34-9_1273;model.g1556.t1;model.g3141.t1;

459 470 44 1.000 1 3 model.g1097.t1;KKA02132.1;34-9_2690;

460 470 98 1.000 1 2 KKA03291.1;34-9_2386;

461 474 66 1.000 1 2 model.g4125.t1;34-9_2410;

462 475 74 1.000 1 3 KKA03098.1;34-9_2545;model.g4149.t1;

463 476 19 1.000 1 3 KKA03286.1;34-9_2390;model.g4146.t1;

464 477 64 1.000 1 3 34-9_2412;KKA02973.1;model.g4118.t1;

465 478 53 1.000 1 3 KKA03284.1;34-9_2393;model.g4157.t1;

466 479 36 1.000 1 4 model.g4497.t1;model.g4498.t1;KKA03161.1;34-9_1437;

467 481 22 1.000 1 2 model.g4141.t1;KKA03290.1;

468 482 44 1.000 1 3 34-9_2401;KKA01045.1;model.g4136.t1;

469 483 19 1.000 1 2 model.g4158.t1;34-9_2392;

470 484 6 0.502 1 6 34-9_1265;KKA03282.1;model.g3128.t1;34-9_2397;model.g3506.t1;model.g4153.t1;

471 485 53 1.000 1 3 KKA03027.1;34-9_2470;model.g4108.t1;

472 486 87 1.000 1 2 model.g4099.t1;34-9_2464;

473 487 16 1.000 1 3 34-9_2476;KKA03033.1;model.g4114.t1;

474 488 75 1.000 1 3 34-9_2556;KKA03109.1;model.g4165.t1;

475 489 57 1.000 1 3 KKA03107.1;34-9_2554;model.g4167.t1;

476 490 16 1.000 1 3 KKA01046.1;34-9_2402;model.g4137.t1;

477 491 5 0.502 1 5 KKA03028.1;34-9_2471;model.g1853.t1;model.g4109.t1;34-9_0800;

478 491 17 1.000 1 6 34-9_2879;model.g2752.t1;34-9_2878;model.g2751.t1;model.g2750.t1;KKA01270.1;

479 491 11 0.945 1 9 KKA03979.1;model.g4280.t1;34-9_1113;KKA03443.1;model.g1045.t1;34-9_2235;KKA03442.1;model.g4283.t1;34-9_2236;

480 494 27 1.000 1 3 model.g4218.t1;KKA03466.1;34-9_2211;

481 494 15 1.000 1 6 model.g3565.t1;model.g4254.t1;34-9_1160;34-9_2725;model.g1115.t1;KKA02106.1;

482 496 31 1.000 1 3 34-9_2561;KKA03113.1;model.g4161.t1;

483 497 52 1.000 1 3 34-9_2473;KKA03030.1;model.g4111.t1;

484 498 5 0.341 1 43 model.g844.t1;model.g4222.t1;34-9_1873;34-9_2212;model.g2981.t1;model.g4262.t1;34-9_2039;34-9_0553;model.g768.t1;34-9_2468;34-9_3534;KKA02374.1;34-9_3154;model.g1330.t1;34-9_2805;KKA00969.1;34-9_2176;model.g123.t1;KKA02874.1;model.g1616.t1;KKA02089.1;34-9_3178;model.g2421.t1;model.g1810.t1;34-9_2958;KKA01444.1;model.g4105.t1;34-9_2124;34-9_0484;model.g319.t1;model.g3099.t1;KKA03506.1;KKA02892.1;KKA03025.1;KKA03465.1;model.g2234.t1;model.g187.t1;34-9_2294;KKA02631.1;model.g2215.t1;model.g2540.t1;34-9_1008;KKA03380.1;

485 498 33 1.000 1 3 KKA02045.1;model.g4474.t1;34-9_0103;

486 498 37 1.000 1 3 model.g1661.t1;KKA01333.1;34-9_3752;

487 498 15 1.000 1 2 model.g242.t1;34-9_0446;

488 498 44 1.000 1 3 34-9_3240;KKA02522.1;model.g2541.t1;

489 498 63 1.000 1 2 34-9_3714;model.g1739.t1;

490 498 31 1.000 1 3 KKA03037.1;model.g3232.t1;34-9_2480;

491 506 10 1.000 1 2 model.g4655.t1;34-9_1808;

492 507 56 1.000 1 3 34-9_2475;KKA03032.1;model.g4113.t1;

493 508 14 1.000 1 6 model.g3351.t1;KKA03024.1;KKA03372.1;34-9_2467;34-9_2302;model.g4103.t1;

494 509 11 1.000 1 5 model.g531.t1;KKA02827.1;34-9_1580;model.g443.t1;34-9_0308;

495 510 9 0.502 1 3 34-9_2552;KKA03105.1;model.g4170.t1;

496 511 24 1.000 1 3 34-9_2286;KKA03389.1;model.g4173.t1;

497 512 26 1.000 1 3 KKA03390.1;34-9_2285;model.g4174.t1;

498 513 28 1.000 1 3 KKA03391.1;34-9_2284;model.g4175.t1;

499 514 68 1.000 1 3 34-9_2631;KKA01127.1;model.g4178.t1;

500 515 90 1.000 1 2 model.g4184.t1;KKA01008.1;

501 516 9 0.667 1 4 KKA01000.1;KKA01004.1;34-9_2641;model.g4180.t1;

502 517 39 1.000 1 3 34-9_2640;KKA01007.1;model.g4183.t1;

503 518 100 1.000 1 2 KKA01002.1;KKA01006.1;

504 519 51 1.000 1 3 model.g4192.t1;model.g3287.t1;model.g4191.t1;

505 520 34 1.000 1 3 KKA01122.1;34-9_2636;model.g4190.t1;

506 521 12 1.000 1 3 KKA01119.1;34-9_2639;model.g4185.t1;

507 522 27 1.000 1 10 KKA03075.1;34-9_2629;model.g3469.t1;34-9_2626;KKA01132.1;model.g3471.t1;model.g3470.t1;KKA01129.1;model.g4177.t1;34-9_2521;

508 523 61 1.000 1 3 34-9_3764;model.g3305.t1;KKA01345.1;

509 524 5 0.510 1 20 34-9_2179;34-9_2418;model.g3420.t1;KKA03502.1;34-9_2975;KKA02298.1;KKA01838.1;KKA01626.1;KKA01627.1;34-9_0073;34-9_1409;model.g4225.t1;34-9_1492;model.g2092.t1;34-9_3273;model.g1694.t1;model.g3421.t1;KKA02978.1;model.g864.t1;model.g683.t1;

510 524 37 1.000 1 3 KKA03747.1;34-9_0865;model.g2401.t1;

511 524 27 0.929 1 9 34-9_0012;KKA00977.1;model.g1943.t1;model.g1941.t1;KKA02367.1;model.g1940.t1;model.g1942.t1;34-9_1998;34-9_0353;

512 524 38 1.000 1 3 34-9_3526;model.g2447.t1;KKA02366.1;

513 524 32 1.000 1 12 34-9_1218;KKA00967.1;model.g1745.t1;KKA00976.1;model.g4337.t1;34-9_0155;34-9_0122;model.g785.t1;model.g1058.t1;model.g4338.t1;model.g2828.t1;KKA00988.1;

514 524 19 1.000 1 3 34-9_3660;KKA00957.1;KKA01328.1;

515 524 9 0.502 1 7 KKA01116.1;34-9_2644;KKA00991.1;KKA00950.1;KKA00963.1;34-9_0121;KKA00959.1;

516 524 28 1.000 1 3 KKA00953.1;KKA03552.1;KKA00990.1;

517 524 9 0.816 1 12 model.g1737.t1;34-9_1650;34-9_3785;KKA01363.1;KKA01746.1;34-9_2586;KKA03135.1;model.g2595.t1;34-9_1651;KKA01747.1;model.g2593.t1;model.g2594.t1;

518 534 99 1.000 1 3 34-9_2642;KKA01003.1;KKA00999.1;

519 535 57 1.000 1 3 KKA01120.1;34-9_2638;model.g4186.t1;

520 536 15 1.000 1 6 KKA01545.1;34-9_2180;34-9_3596;KKA03501.1;model.g1788.t1;model.g4250.t1;

521 537 34 1.000 1 3 KKA03529.1;34-9_2154;model.g4204.t1;

522 538 17 1.000 1 3 KKA03549.1;34-9_2133;model.g4195.t1;

523 539 5 0.427 1 11 model.g1954.t1;model.g309.t1;KKA02545.1;KKA02909.1;34-9_3205;34-9_0561;34-9_3115;model.g2051.t1;model.g585.t1;model.g4267.t1;KKA01690.1;

524 540 51 1.000 1 3 34-9_2227;KKA03450.1;model.g4329.t1;

525 541 40 1.000 1 3 34-9_2229;KKA03448.1;model.g4312.t1;

526 542 22 1.000 1 3 34-9_2149;KKA03533.1;model.g4328.t1;

527 543 67 1.000 1 3 KKA03473.1;34-9_2205;model.g4223.t1;

528 544 56 1.000 1 3 KKA01114.1;34-9_1634;model.g4198.t1;

529 545 32 1.000 1 3 KKA03457.1;34-9_2220;model.g4290.t1;

530 546 14 0.918 1 11 34-9_2241;model.g4288.t1;model.g4255.t1;34-9_2452;model.g2542.t1;KKA03319.1;model.g3277.t1;34-9_2355;model.g3368.t1;KKA03010.1;KKA03436.1;

531 547 67 1.000 1 2 KKA03518.1;34-9_2166;

532 548 42 1.000 1 3 34-9_2163;KKA03521.1;model.g4315.t1;

533 549 18 1.000 1 3 34-9_2262;KKA03414.1;model.g4335.t1;

534 550 13 1.000 1 3 KKA03520.1;34-9_2164;model.g4318.t1;

535 551 22 1.000 1 3 34-9_2197;KKA03481.1;model.g4233.t1;

536 552 6 0.557 1 6 34-9_1228;KKA03468.1;KKA01902.1;34-9_2210;model.g1995.t1;model.g4216.t1;

537 553 33 1.000 1 2 model.g4308.t1;34-9_2150;

538 554 61 1.000 1 3 34-9_3490;KKA02327.1;model.g2480.t1;

539 555 17 1.000 1 2 model.g4239.t1;34-9_2193;

540 556 22 1.000 1 7 KKA01661.1;KKA03469.1;34-9_1391;34-9_2209;model.g1671.t1;model.g4215.t1;model.g2249.t1;

541 557 11 0.769 1 13 34-9_0827;model.g4330.t1;34-9_2146;KKA01265.1;KKA03536.1;model.g2419.t1;KKA03451.1;KKA03715.1;KKA02301.1;model.g2018.t1;34-9_3462;34-9_2226;34-9_2145;

542 558 29 1.000 1 2 model.g4305.t1;34-9_2159;

543 559 27 1.000 1 3 KKA03524.1;34-9_2160;model.g4324.t1;

544 560 24 1.000 1 3 34-9_1889;model.g2270.t1;KKA01858.1;

545 561 29 1.000 1 3 34-9_2143;KKA03538.1;model.g4201.t1;

546 562 29 1.000 1 3 34-9_2225;KKA03452.1;model.g4331.t1;

547 563 25 1.000 1 2 model.g4217.t1;KKA03467.1;

548 564 30 1.000 1 3 34-9_2201;KKA03477.1;model.g4228.t1;

549 565 13 1.000 1 3 34-9_2343;model.g3390.t1;KKA03330.1;

550 566 35 1.000 1 2 model.g4257.t1;34-9_2185;

551 567 22 1.000 1 3 34-9_2192;KKA03488.1;model.g4240.t1;

552 568 32 1.000 1 3 KKA03530.1;34-9_2153;model.g4205.t1;

553 569 12 1.000 1 3 34-9_2147;KKA03535.1;model.g4326.t1;

554 570 54 1.000 1 3 KKA03471.1;34-9_2207;model.g4213.t1;

555 571 99 1.000 1 2 KKA01115.1;34-9_1635;

556 572 19 1.000 1 4 KKA01624.1;model.g1316.t1;34-9_2973;model.g4231.t1;

557 572 37 1.000 1 2 model.g1000.t1;KKA01625.1;

558 574 44 1.000 1 3 KKA03395.1;model.g1911.t1;34-9_2279;

559 575 50 1.000 1 3 34-9_2221;KKA03456.1;model.g4291.t1;

560 576 79 1.000 1 2 34-9_0302;model.g538.t1;

561 577 16 1.000 1 3 34-9_2610;KKA01198.1;model.g4242.t1;

562 578 60 1.000 1 2 34-9_2663;KKA02150.1;

563 579 41 1.000 1 3 34-9_2132;KKA03550.1;model.g4197.t1;

564 580 28 1.000 1 3 KKA03511.1;34-9_2171;model.g4270.t1;

565 580 16 1.000 1 3 34-9_0005;model.g573.t1;KKA01039.1;

566 582 86 1.000 1 4 model.g808.t1;KKA03462.1;34-9_2215;model.g4211.t1;

567 583 50 1.000 1 3 KKA03416.1;34-9_2260;model.g4333.t1;

568 583 25 1.000 1 2 KKA03417.1;model.g4332.t1;

569 585 22 1.000 1 3 KKA03463.1;34-9_2214;model.g4219.t1;

570 586 22 1.000 1 6 model.g1657.t1;34-9_3748;KKA01329.1;34-9_3766;model.g3304.t1;KKA01347.1;

571 587 38 1.000 1 3 34-9_0753;KKA03644.1;model.g2358.t1;

572 588 18 1.000 1 5 KKA03484.1;34-9_1679;model.g2905.t1;model.g4236.t1;KKA01732.1;

573 588 24 1.000 1 3 34-9_1232;KKA01904.1;model.g1999.t1;

574 590 16 1.000 1 6 KKA01948.1;KKA03526.1;34-9_1352;34-9_2157;model.g2600.t1;model.g4303.t1;

575 591 26 1.000 1 3 KKA03476.1;34-9_2202;model.g4227.t1;

576 592 50 1.000 1 3 KKA03464.1;34-9_2213;model.g4220.t1;

577 593 30 1.000 1 3 34-9_2189;KKA03493.1;model.g4246.t1;

578 593 4 0.443 1 6 KKA01580.1;model.g2338.t1;34-9_0377;34-9_0647;KKA01521.1;model.g650.t1;

579 595 38 1.000 1 2 model.g2825.t1;34-9_0154;

580 596 26 1.000 1 3 34-9_2261;KKA03415.1;model.g4334.t1;

581 597 10 0.749 1 7 34-9_2842;KKA03499.1;KKA01154.1;34-9_2182;model.g1539.t1;model.g4252.t1;KKA01153.1;

582 598 48 1.000 1 3 KKA03478.1;34-9_2200;model.g4229.t1;

583 598 89 1.000 1 3 KKA03809.1;model.g2119.t1;34-9_0931;

584 600 15 1.000 1 3 KKA03455.1;34-9_2222;model.g4292.t1;

585 601 40 1.000 1 3 34-9_2174;KKA03508.1;model.g4274.t1;

586 602 5 0.502 1 3 model.g1083.t1;model.g1381.t1;model.g4282.t1;

587 602 12 1.000 1 6 model.g4483.t1;model.g1084.t1;model.g3334.t1;model.g1081.t1;34-9_0474;KKA02082.1;

588 604 67 1.000 1 3 KKA03534.1;34-9_2148;model.g4325.t1;

589 605 75 1.000 1 3 KKA03480.1;34-9_2198;model.g4232.t1;

590 606 20 1.000 1 4 34-9_1892;KKA01861.1;model.g2276.t1;model.g2523.t1;

591 607 6 0.502 1 5 34-9_3235;34-9_2190;KKA03491.1;model.g612.t1;model.g4245.t1;

592 607 50 1.000 1 3 KKA01852.1;34-9_1883;model.g2261.t1;

593 607 65 1.000 1 2 KKA01851.1;model.g2260.t1;

594 610 44 1.000 1 3 KKA03537.1;34-9_2144;model.g4202.t1;

595 611 43 1.000 1 3 34-9_2203;KKA03475.1;model.g4226.t1;

596 612 18 1.000 1 3 KKA03513.1;34-9_2169;model.g4275.t1;

597 613 13 1.000 1 3 KKA03437.1;34-9_2240;model.g4289.t1;

598 614 33 1.000 1 3 KKA03539.1;34-9_2142;model.g4200.t1;

599 615 5 0.502 1 3 KKA03523.1;34-9_2161;model.g4322.t1;

600 616 13 1.000 1 3 KKA03528.1;34-9_2155;model.g4203.t1;

601 617 11 1.000 1 3 KKA03525.1;34-9_2158;model.g4307.t1;

602 618 7 0.667 1 4 KKA03449.1;34-9_2228;model.g1232.t1;model.g4311.t1;

603 619 33 1.000 1 4 KKA03515.1;KKA03517.1;34-9_2167;model.g4320.t1;

604 620 18 1.000 1 2 model.g2728.t1;34-9_2901;

605 621 11 1.000 1 3 KKA03445.1;34-9_2233;model.g4278.t1;

606 622 5 0.459 1 19 model.g4266.t1;model.g1834.t1;model.g220.t1;34-9_0535;model.g547.t1;model.g445.t1;model.g767.t1;34-9_0295;KKA02034.1;34-9_2175;model.g3598.t1;34-9_0816;34-9_2249;KKA03507.1;KKA03427.1;KKA01272.1;34-9_0116;34-9_2876;KKA03705.1;

607 623 24 1.000 1 3 model.g4575.t1;34-9_3450;KKA02662.1;

608 624 30 1.000 1 3 34-9_2206;KKA03472.1;model.g4212.t1;

609 625 8 0.667 1 22 model.g4314.t1;model.g85.t1;KKA02911.1;KKA01514.1;model.g3586.t1;model.g172.t1;model.g2803.t1;34-9_3112;34-9_2165;model.g2551.t1;KKA03514.1;KKA02905.1;34-9_2168;KKA01136.1;34-9_0364;34-9_3737;model.g4306.t1;KKA03519.1;34-9_3624;34-9_3122;KKA01293.1;model.g69.t1;

610 626 20 1.000 1 3 KKA03856.1;model.g2180.t1;34-9_0983;

611 627 17 1.000 1 3 KKA03527.1;34-9_2156;model.g4304.t1;

612 628 44 1.000 1 3 34-9_2186;KKA03496.1;model.g4249.t1;

613 629 59 1.000 1 3 KKA03439.1;34-9_2238;model.g4285.t1;

614 630 16 1.000 1 3 KKA02870.1;34-9_1623;model.g4296.t1;

615 631 48 1.000 1 3 KKA03532.1;34-9_2151;model.g4210.t1;

616 633 16 1.000 1 9 model.g4248.t1;KKA02836.1;model.g435.t1;34-9_1588;34-9_1783;34-9_2187;KKA03495.1;KKA02006.1;model.g4695.t1;

617 634 98 1.000 1 2 KKA03454.1;34-9_2223;

618 635 29 1.000 1 2 model.g4321.t1;KKA03516.1;

619 636 33 1.000 1 3 KKA03444.1;34-9_2234;model.g4279.t1;

620 637 15 1.000 1 3 34-9_2178;KKA03504.1;model.g4260.t1;

621 638 20 1.000 1 3 KKA03474.1;34-9_2204;model.g4224.t1;

622 639 12 1.000 1 3 KKA03483.1;34-9_2195;model.g4235.t1;

623 640 44 1.000 1 3 KKA03458.1;34-9_2219;model.g4301.t1;

624 641 63 1.000 1 3 KKA03494.1;34-9_2188;model.g4247.t1;

625 642 12 1.000 1 6 model.g801.t1;34-9_2331;34-9_3745;KKA01145.1;model.g2815.t1;KKA03343.1;

626 643 46 1.000 1 3 34-9_2741;KKA03223.1;model.g4400.t1;

627 644 78 1.000 1 2 model.g4364.t1;34-9_1210;

628 645 13 1.000 1 5 34-9_1166;model.g1460.t1;34-9_3279;model.g4372.t1;KKA01832.1;

629 646 22 0.502 1 3 34-9_1190;KKA01778.1;KKA01779.1;

630 648 21 1.000 1 3 KKA03264.1;34-9_2799;model.g4379.t1;

631 649 26 1.000 1 3 34-9_0228;KKA02769.1;model.g4593.t1;

632 651 34 1.000 1 2 model.g2757.t1;34-9_2991;

633 652 19 1.000 1 7 34-9_3768;KKA03252.1;KKA01349.1;34-9_2781;model.g3303.t1;model.g4429.t1;model.g377.t1;

634 653 53 1.000 1 2 model.g4396.t1;34-9_1533;

635 654 31 1.000 1 2 model.g4424.t1;34-9_2784;

636 655 5 0.502 1 5 model.g3321.t1;34-9_3192;KKA02555.1;KKA02556.1;model.g4378.t1;

637 656 13 1.000 1 2 model.g4374.t1;34-9_2797;

638 657 89 1.000 1 2 KKA03225.1;34-9_2744;

639 658 50 1.000 1 2 model.g4363.t1;34-9_1211;

640 659 40 1.000 1 3 KKA01798.1;34-9_1161;model.g4383.t1;

641 659 5 0.431 1 8 34-9_2881;KKA02414.1;model.g2729.t1;model.g2753.t1;model.g1386.t1;model.g1764.t1;34-9_3572;KKA01268.1;

642 659 48 1.000 1 3 34-9_0237;KKA02760.1;model.g4588.t1;

643 662 47 1.000 1 2 model.g4448.t1;34-9_2769;

644 663 40 1.000 1 3 34-9_3234;KKA02527.1;model.g4395.t1;

645 664 8 0.502 1 3 34-9_1155;model.g4381.t1;model.g4382.t1;

646 665 36 1.000 1 2 model.g4367.t1;34-9_1192;

647 666 58 1.000 1 3 KKA03244.1;34-9_2766;model.g4436.t1;

648 667 18 1.000 1 3 KKA03255.1;34-9_2786;model.g4422.t1;

649 668 10 0.502 1 5 34-9_2684;34-9_2768;KKA03246.1;model.g4439.t1;model.g1641.t1;

650 668 23 1.000 1 5 model.g3267.t1;model.g1644.t1;model.g1643.t1;KKA01655.1;KKA01067.1;

651 668 37 1.000 1 2 34-9_2933;KKA01185.1;

652 671 39 1.000 1 4 34-9_0419;model.g267.t1;34-9_3238;model.g4445.t1;

653 672 17 1.000 1 2 model.g4402.t1;34-9_2743;

654 673 30 1.000 1 3 34-9_2822;KKA03274.1;model.g4417.t1;

655 674 84 1.000 1 3 KKA01764.1;34-9_1212;model.g4345.t1;

656 675 36 1.000 1 2 model.g4538.t1;34-9_0577;

657 676 31 1.000 1 3 KKA01768.1;34-9_1203;model.g4358.t1;

658 677 20 1.000 1 2 model.g4359.t1;34-9_1207;

659 678 15 1.000 1 8 34-9_0933;34-9_2775;34-9_3628;model.g1808.t1;KKA03811.1;model.g4441.t1;KKA01296.1;model.g2121.t1;

660 679 17 1.000 1 4 34-9_2778;KKA01388.1;model.g2575.t1;model.g4432.t1;

661 680 49 1.000 1 2 model.g4365.t1;34-9_1209;

662 681 10 0.714 1 10 model.g274.t1;34-9_3221;34-9_3151;KKA03772.1;model.g120.t1;34-9_0891;34-9_0617;KKA02894.1;model.g2013.t1;model.g608.t1;

663 682 38 1.000 1 2 model.g4341.t1;34-9_1215;

664 683 20 1.000 1 3 34-9_1193;KKA01776.1;model.g4368.t1;

665 684 19 1.000 1 2 model.g4377.t1;34-9_2798;

666 685 65 1.000 1 2 34-9_1454;model.g2662.t1;

667 686 26 1.000 1 3 KKA03222.1;34-9_2739;model.g4398.t1;

668 687 35 1.000 1 6 34-9_0026;34-9_1169;34-9_1457;model.g2947.t1;KKA02647.1;model.g4411.t1;

669 688 13 0.859 1 8 KKA00964.1;KKA01026.1;KKA00962.1;model.g2940.t1;KKA00965.1;model.g4336.t1;KKA00952.1;KKA00979.1;

670 688 14 1.000 1 3 model.g17.t1;model.g302.t1;model.g2492.t1;

671 688 7 0.502 1 4 model.g4482.t1;model.g1634.t1;model.g3423.t1;model.g2709.t1;

672 688 43 1.000 1 2 KKA01040.1;34-9_3691;

673 693 15 1.000 1 2 model.g1633.t1;model.g1635.t1;

674 694 52 1.000 1 2 KKA01769.1;34-9_1201;

675 695 32 1.000 1 3 34-9_1171;KKA01793.1;model.g4413.t1;

676 696 12 0.502 1 3 34-9_2742;KKA03224.1;model.g4401.t1;

677 697 14 1.000 1 3 KKA01775.1;34-9_1195;model.g4371.t1;

678 698 19 1.000 1 3 KKA03253.1;34-9_2782;model.g4428.t1;

679 699 19 1.000 1 3 34-9_2764;KKA03243.1;model.g4434.t1;

680 700 81 1.000 1 3 34-9_2785;KKA03254.1;model.g4423.t1;

681 701 77 1.000 1 3 KKA03245.1;34-9_2767;model.g4437.t1;

682 702 20 1.000 1 3 34-9_0223;KKA02774.1;model.g4351.t1;

683 703 23 1.000 1 3 KKA01765.1;34-9_1206;model.g4347.t1;

684 704 45 1.000 1 3 34-9_1198;KKA01772.1;model.g4350.t1;

685 705 15 1.000 1 3 34-9_2748;KKA03228.1;model.g4408.t1;

686 706 14 1.000 1 5 34-9_2800;KKA03689.1;model.g1854.t1;model.g4380.t1;34-9_0799;

687 707 19 1.000 1 2 model.g4366.t1;34-9_1208;

688 708 7 0.502 1 11 34-9_1064;model.g4420.t1;model.g911.t1;34-9_1743;model.g910.t1;34-9_1828;KKA03933.1;KKA02259.1;model.g2841.t1;KKA02187.1;34-9_2787;

689 709 27 1.000 1 2 model.g4353.t1;34-9_1200;

690 710 58 1.000 1 2 34-9_0612;model.g1409.t1;

691 711 50 1.000 1 5 34-9_2777;model.g3051.t1;34-9_0467;model.g4449.t1;KKA02075.1;

692 712 38 1.000 1 2 model.g4415.t1;34-9_1173;

693 713 7 0.667 1 4 model.g4637.t1;KKA01335.1;model.g1632.t1;34-9_3754;

694 714 35 1.000 1 3 KKA03628.1;34-9_0737;model.g4421.t1;

695 715 13 1.000 1 3 34-9_2745;KKA03226.1;model.g4405.t1;

696 716 18 1.000 1 10 34-9_1728;model.g2948.t1;model.g4412.t1;KKA02247.1;model.g1152.t1;34-9_0025;KKA02648.1;34-9_1170;model.g2857.t1;model.g1763.t1;

697 716 11 1.000 1 2 model.g2858.t1;KKA02246.1;

698 718 69 1.000 1 2 model.g4406.t1;34-9_2746;

699 719 39 1.000 1 2 model.g4416.t1;KKA03275.1;

700 720 72 1.000 1 2 model.g4414.t1;34-9_1172;

701 721 98 1.000 1 2 34-9_1532;KKA03221.1;

702 722 14 1.000 1 4 34-9_2783;model.g2762.t1;34-9_3060;model.g4427.t1;

703 723 14 1.000 1 4 34-9_1604;KKA02854.1;model.g3519.t1;model.g4346.t1;

704 724 35 1.000 1 3 KKA01461.1;model.g1727.t1;34-9_3712;

705 725 33 1.000 1 3 34-9_3004;KKA01647.1;model.g1022.t1;

706 726 12 1.000 1 3 KKA03241.1;34-9_2763;model.g4450.t1;

707 727 39 1.000 1 3 KKA02043.1;34-9_0105;model.g4476.t1;

708 728 20 1.000 1 3 34-9_3464;KKA02303.1;model.g4462.t1;

709 729 13 1.000 1 3 KKA02305.1;34-9_3467;model.g4454.t1;

710 730 7 0.667 1 4 KKA03298.1;34-9_2379;model.g3162.t1;model.g4467.t1;

711 730 81 1.000 1 2 34-9_1221;KKA01896.1;

712 732 10 1.000 1 3 KKA02086.1;34-9_0479;model.g4460.t1;

713 733 42 1.000 1 3 34-9_0097;KKA02050.1;model.g4480.t1;

714 734 99 1.000 1 2 34-9_0259;KKA02738.1;

715 735 10 1.000 1 3 34-9_0104;KKA02044.1;model.g4475.t1;

716 736 26 1.000 1 2 model.g4463.t1;34-9_0381;

717 737 29 1.000 1 3 34-9_3465;KKA02304.1;model.g4461.t1;

718 738 36 1.000 1 2 model.g4479.t1;34-9_0096;

719 739 32 1.000 1 2 model.g4451.t1;34-9_1226;

720 740 28 1.000 1 3 KKA01524.1;34-9_0380;model.g4464.t1;

721 741 23 0.502 1 3 34-9_3468;34-9_3469;KKA02306.1;

722 742 43 1.000 1 3 KKA01522.1;34-9_0378;model.g4466.t1;

723 743 38 1.000 1 3 KKA02739.1;34-9_0258;model.g4470.t1;

724 744 16 1.000 1 3 34-9_0100;KKA02048.1;model.g4477.t1;

725 745 22 1.000 1 3 34-9_0257;KKA02740.1;model.g4471.t1;

726 745 5 0.502 1 8 34-9_1109;model.g2090.t1;KKA02575.1;34-9_0424;34-9_2015;model.g165.t1;KKA03975.1;model.g1010.t1;

727 745 25 1.000 1 2 model.g2292.t1;34-9_1905;

728 748 8 0.502 1 3 34-9_3699;KKA01457.1;model.g4456.t1;

729 749 61 1.000 1 2 model.g4457.t1;34-9_0385;

730 750 38 1.000 1 3 34-9_3231;model.g137.t1;KKA02529.1;

731 751 24 1.000 1 3 KKA01523.1;34-9_0379;model.g4465.t1;

732 752 28 1.000 1 3 KKA02546.1;34-9_3204;model.g4494.t1;

733 753 9 0.502 1 3 KKA02547.1;34-9_3202;model.g4489.t1;

734 754 39 1.000 1 2 model.g4488.t1;34-9_3201;

735 755 6 0.502 1 3 34-9_3203;model.g4492.t1;model.g4490.t1;

736 756 14 1.000 1 3 model.g555.t1;34-9_0291;model.g4491.t1;

737 757 54 1.000 1 5 34-9_3198;KKA02551.1;34-9_2804;model.g4485.t1;model.g2233.t1;

738 758 13 1.000 1 6 model.g800.t1;34-9_1772;34-9_2330;KKA01997.1;model.g359.t1;KKA03344.1;

739 759 18 1.000 1 3 KKA02550.1;34-9_3199;model.g4486.t1;

740 760 17 1.000 1 5 34-9_1442;KKA01967.1;model.g335.t1;model.g4505.t1;34-9_1332;

741 761 99 1.000 1 2 34-9_1963;KKA02463.1;

742 762 77 1.000 1 2 model.g4503.t1;34-9_1440;

743 763 19 1.000 1 2 model.g4495.t1;34-9_1436;

744 764 45 1.000 1 3 KKA03164.1;34-9_1441;model.g4504.t1;

745 765 33 1.000 1 3 34-9_1452;KKA03170.1;model.g4511.t1;

746 766 53 1.000 1 3 KKA03167.1;34-9_1445;model.g4508.t1;

747 767 42 1.000 1 2 model.g4510.t1;34-9_1451;

748 768 51 1.000 1 3 34-9_1439;KKA03163.1;model.g4502.t1;

749 768 8 0.667 1 5 KKA02719.1;model.g1590.t1;34-9_3362;KKA01287.1;model.g1515.t1;

750 770 43 1.000 1 2 model.g1595.t1;34-9_3367;

751 771 24 1.000 1 3 KKA03169.1;34-9_1450;model.g4509.t1;

752 772 10 0.867 1 18 model.g1052.t1;34-9_1119;KKA01651.1;KKA01682.1;34-9_1371;KKA01741.1;model.g575.t1;model.g1673.t1;34-9_1641;34-9_1663;34-9_1422;KKA01755.1;model.g2918.t1;34-9_1393;model.g2630.t1;KKA01662.1;model.g4496.t1;KKA03986.1;

753 773 42 1.000 1 2 model.g1572.t1;34-9_3390;

754 774 32 1.000 1 3 KKA03165.1;34-9_1443;model.g4506.t1;

755 775 23 1.000 1 5 34-9_1438;KKA02482.1;KKA03162.1;model.g2327.t1;model.g4499.t1;

756 775 17 1.000 1 3 34-9_1933;KKA02483.1;model.g2326.t1;

757 777 10 0.706 1 20 KKA03928.1;model.g993.t1;34-9_3047;34-9_2141;KKA02945.1;model.g2149.t1;KKA03540.1;model.g992.t1;34-9_0468;model.g3453.t1;34-9_1059;KKA02943.1;34-9_1916;KKA02076.1;model.g991.t1;KKA02944.1;34-9_2672;model.g3454.t1;34-9_3046;model.g3052.t1;

758 778 23 1.000 1 3 KKA01709.1;34-9_0590;model.g4521.t1;

759 779 28 1.000 1 3 KKA01713.1;34-9_0594;model.g4517.t1;

760 780 32 1.000 1 2 model.g4513.t1;34-9_0596;

761 781 37 1.000 1 3 KKA01706.1;34-9_0588;model.g4526.t1;

762 782 23 1.000 1 2 model.g4520.t1;KKA01708.1;

763 783 35 1.000 1 3 34-9_1481;KKA03188.1;model.g2681.t1;

764 784 43 1.000 1 2 model.g4518.t1;34-9_0595;

765 785 17 1.000 1 3 KKA01711.1;34-9_0592;model.g4515.t1;

766 786 10 1.000 1 2 model.g4532.t1;34-9_0584;

767 787 27 1.000 1 3 34-9_1229;KKA01903.1;model.g1996.t1;

768 788 75 1.000 1 3 KKA01700.1;34-9_0575;model.g4536.t1;

769 789 19 1.000 1 2 model.g4528.t1;34-9_0587;

770 790 13 1.000 1 2 model.g4551.t1;34-9_0330;

771 791 41 1.000 1 3 KKA03231.1;34-9_2751;model.g4548.t1;

772 792 19 1.000 1 2 model.g4544.t1;34-9_1167;

773 793 28 1.000 1 3 KKA01794.1;34-9_1168;model.g4546.t1;

774 793 27 1.000 1 2 model.g963.t1;KKA02961.1;

775 795 11 1.000 1 3 KKA03234.1;34-9_2753;model.g4550.t1;

776 796 13 1.000 1 3 34-9_1164;KKA01796.1;model.g4543.t1;

777 797 14 1.000 1 3 34-9_2755;34-9_2754;model.g4552.t1;

778 798 23 1.000 1 3 KKA01840.1;34-9_3270;model.g4560.t1;

779 799 43 1.000 1 3 KKA02660.1;34-9_3453;model.g4580.t1;

780 800 11 1.000 1 4 KKA01015.1;KKA02627.1;34-9_2118;model.g4565.t1;

781 801 61 1.000 1 3 34-9_3451;KKA02661.1;model.g4578.t1;

782 802 26 1.000 1 5 34-9_3449;34-9_3560;model.g1776.t1;KKA02403.1;model.g4576.t1;

783 802 35 1.000 1 2 KKA02402.1;model.g3604.t1;

784 802 24 1.000 1 3 model.g981.t1;34-9_3039;KKA02950.1;

785 805 51 1.000 1 2 model.g4572.t1;model.g62.t1;

786 805 10 0.502 1 5 34-9_1551;34-9_2685;model.g3035.t1;model.g190.t1;KKA02800.1;

787 807 18 1.000 1 3 KKA02585.1;34-9_2042;model.g4577.t1;

788 808 25 1.000 1 4 KKA01866.1;34-9_1896;model.g2281.t1;model.g4584.t1;

789 809 43 1.000 1 3 34-9_3342;KKA01609.1;model.g4556.t1;

790 810 20 1.000 1 6 34-9_1076;34-9_3448;KKA03944.1;KKA02663.1;model.g2052.t1;model.g4579.t1;

791 811 21 1.000 1 2 model.g4573.t1;34-9_2110;

792 812 31 1.000 1 5 34-9_2095;model.g162.t1;KKA02201.1;model.g4570.t1;34-9_1848;

793 813 98 1.000 1 2 KKA02624.1;34-9_2109;

794 814 23 1.000 1 3 KKA02617.1;34-9_2093;model.g4568.t1;

795 815 68 1.000 1 3 KKA02767.1;34-9_0230;model.g4595.t1;

796 816 14 1.000 1 3 KKA02772.1;34-9_0226;model.g4601.t1;

797 817 70 1.000 1 3 34-9_0236;KKA02761.1;model.g4589.t1;

798 818 17 1.000 1 3 KKA02895.1;model.g119.t1;34-9_3150;

799 819 36 1.000 1 3 34-9_0229;KKA02768.1;model.g4594.t1;

800 820 18 1.000 1 5 KKA02758.1;model.g4639.t1;34-9_0239;34-9_1817;model.g4586.t1;

801 821 18 1.000 1 4 model.g3456.t1;KKA01758.1;34-9_1639;model.g4604.t1;

802 821 14 1.000 1 2 model.g577.t1;KKA01757.1;

803 823 48 1.000 1 3 KKA02757.1;34-9_0240;model.g4585.t1;

804 824 54 1.000 1 3 KKA02771.1;34-9_0227;model.g4602.t1;

805 825 31 1.000 1 2 model.g4599.t1;34-9_0231;

806 826 23 1.000 1 2 model.g1826.t1;34-9_0823;

807 827 15 1.000 1 3 34-9_0234;KKA02763.1;model.g4592.t1;

808 828 37 1.000 1 2 model.g4598.t1;KKA02764.1;

809 829 16 1.000 1 2 34-9_0307;model.g532.t1;

810 830 98 1.000 1 2 KKA02766.1;34-9_0232;

811 831 32 1.000 1 2 model.g4596.t1;model.g353.t1;

812 832 34 1.000 1 3 KKA02313.1;34-9_3475;model.g4618.t1;

813 833 57 1.000 1 3 34-9_1593;KKA02843.1;model.g4616.t1;

814 834 16 1.000 1 3 KKA02846.1;34-9_1595;model.g4612.t1;

815 835 20 1.000 1 2 34-9_0022;model.g3547.t1;

816 836 30 1.000 1 3 KKA02847.1;34-9_1596;model.g4611.t1;

817 837 25 1.000 1 6 34-9_0351;34-9_1824;KKA02948.1;model.g4703.t1;model.g4647.t1;model.g984.t1;

818 838 21 1.000 1 2 34-9_2692;model.g1669.t1;

819 839 23 1.000 1 2 model.g4619.t1;KKA02841.1;

820 840 6 0.502 1 18 34-9_2417;34-9_1355;KKA03047.1;model.g4608.t1;34-9_2494;model.g2608.t1;34-9_0619;model.g875.t1;KKA02705.1;model.g3211.t1;KKA03196.1;model.g627.t1;34-9_1598;model.g3256.t1;model.g666.t1;KKA02977.1;model.g275.t1;model.g721.t1;

821 840 31 1.000 1 3 model.g201.t1;KKA02865.1;34-9_1619;

822 840 75 1.000 1 2 model.g2525.t1;34-9_3246;

823 843 24 1.000 1 3 KKA02737.1;34-9_0260;model.g4622.t1;

824 844 17 0.502 1 3 KKA02845.1;KKA02844.1;34-9_1594;

825 845 31 1.000 1 2 model.g4636.t1;34-9_3058;

826 846 15 1.000 1 8 model.g1313.t1;34-9_2994;KKA02922.1;KKA02965.1;34-9_3014;model.g4624.t1;model.g2795.t1;34-9_3088;

827 846 27 1.000 1 2 34-9_0572;model.g290.t1;

828 848 53 1.000 1 2 model.g4627.t1;34-9_2997;

829 849 40 1.000 1 3 KKA01644.1;34-9_3000;model.g4631.t1;

830 850 77 1.000 1 2 KKA01643.1;34-9_2999;

831 851 48 1.000 1 3 KKA01639.1;34-9_2995;model.g4625.t1;

832 852 46 1.000 1 3 34-9_3057;KKA02938.1;model.g4635.t1;

833 853 26 1.000 1 3 KKA01645.1;34-9_3001;model.g4632.t1;

834 854 68 1.000 1 3 34-9_1776;KKA02001.1;model.g4688.t1;

835 855 98 1.000 1 2 KKA02175.1;34-9_1811;

836 856 46 1.000 1 2 model.g4697.t1;KKA02010.1;

837 857 38 1.000 1 2 model.g4650.t1;34-9_1815;

838 858 28 1.000 1 5 KKA02005.1;34-9_0637;34-9_1782;model.g2350.t1;model.g4694.t1;

839 859 50 1.000 1 3 KKA02009.1;34-9_1786;model.g4664.t1;

840 860 8 0.502 1 3 34-9_1777;KKA02002.1;model.g4689.t1;

841 861 27 1.000 1 3 KKA02165.1;34-9_1799;model.g4677.t1;

842 862 52 1.000 1 2 model.g4691.t1;34-9_1779;

843 863 36 1.000 1 3 KKA02003.1;34-9_1778;model.g4690.t1;

844 864 9 0.667 1 6 model.g3263.t1;34-9_1793;KKA03002.1;KKA02017.1;34-9_2442;model.g4670.t1;

845 865 30 1.000 1 3 34-9_1818;KKA02179.1;model.g4640.t1;

846 866 12 1.000 1 3 34-9_3703;KKA01458.1;model.g1734.t1;

847 867 20 1.000 1 4 KKA02019.1;34-9_1795;KKA00994.1;model.g4672.t1;

848 868 52 1.000 1 3 34-9_1804;KKA02170.1;model.g4682.t1;

849 869 84 1.000 1 3 KKA02016.1;34-9_1792;model.g4669.t1;

850 870 34 1.000 1 2 model.g4658.t1;34-9_1810;

851 871 24 1.000 1 5 34-9_1806;model.g415.t1;34-9_0268;model.g4684.t1;KKA02729.1;

852 871 20 1.000 1 6 model.g2481.t1;34-9_2383;KKA03294.1;model.g3177.t1;KKA02326.1;34-9_3489;

853 873 55 1.000 1 3 KKA02171.1;34-9_1805;model.g4683.t1;

854 874 17 1.000 1 5 KKA02011.1;34-9_2992;34-9_1787;model.g2758.t1;model.g4698.t1;

855 875 47 1.000 1 2 model.g4645.t1;34-9_1821;

856 876 58 1.000 1 3 34-9_1794;KKA02018.1;model.g4671.t1;

857 877 62 1.000 1 3 34-9_1295;KKA01252.1;model.g380.t1;

858 878 24 1.000 1 7 KKA02749.1;KKA00993.1;model.g484.t1;KKA02020.1;34-9_1796;model.g4673.t1;34-9_0249;

859 879 23 1.000 1 4 model.g1751.t1;34-9_3580;KKA01028.1;KKA01017.1;

860 881 7 0.502 1 3 34-9_1780;KKA02004.1;model.g4692.t1;

861 882 83 1.000 1 3 KKA02181.1;34-9_1820;model.g4644.t1;

862 883 48 1.000 1 3 KKA02013.1;34-9_1789;model.g4666.t1;

863 884 16 1.000 1 4 model.g3587.t1;34-9_0543;KKA01440.1;model.g4674.t1;

864 885 29 1.000 1 3 KKA02168.1;34-9_1802;model.g4681.t1;

865 886 10 1.000 1 2 model.g4642.t1;34-9_1816;

866 887 7 0.502 1 3 34-9_1813;KKA02176.1;model.g4662.t1;

867 888 42 1.000 1 2 model.g4661.t1;34-9_1812;

868 889 34 1.000 1 3 34-9_1788;KKA02012.1;model.g4687.t1;

869 890 79 1.000 1 3 KKA02174.1;34-9_1809;model.g4657.t1;

870 891 13 1.000 1 3 34-9_1785;KKA02008.1;model.g4665.t1;

871 892 46 1.000 1 2 34-9_3280;KKA01831.1;

872 893 5 0.502 1 3 34-9_3306;model.g3325.t1;model.g1444.t1;

873 894 45 1.000 1 2 model.g4711.t1;34-9_0344;

874 895 6 0.502 1 9 model.g4710.t1;KKA02785.1;model.g402.t1;34-9_1536;KKA02776.1;KKA01887.1;34-9_0345;34-9_0221;model.g2466.t1;

875 896 17 1.000 1 6 KKA01482.1;34-9_2441;model.g2734.t1;34-9_2896;model.g3262.t1;KKA03001.1;

876 897 32 1.000 1 3 34-9_0809;KKA03698.1;model.g1840.t1;

877 898 29 1.000 1 2 model.g4738.t1;34-9_3333;

878 899 33 1.000 1 6 34-9_2273;KKA01601.1;KKA03402.1;34-9_3336;model.g1917.t1;model.g4739.t1;

879 900 6 0.557 1 6 KKA01013.1;KKA01599.1;KKA02626.1;34-9_3334;model.g688.t1;model.g4737.t1;

880 901 10 1.000 1 3 KKA01598.1;34-9_3331;model.g4724.t1;

881 902 15 1.000 1 3 model.g4746.t1;34-9_3318;model.g4715.t1;

882 902 38 1.000 1 2 KKA02710.1;34-9_3376;

883 904 21 1.000 1 3 34-9_3322;KKA01592.1;model.g4721.t1;

884 905 15 1.000 1 2 model.g4718.t1;34-9_3319;

885 906 12 1.000 1 3 34-9_3321;KKA01591.1;model.g4720.t1;

886 907 44 1.000 1 2 34-9_3597;model.g1789.t1;

887 908 7 0.667 1 4 KKA01597.1;34-9_3328;model.g2774.t1;model.g4730.t1;

888 908 22 0.502 1 3 KKA02929.1;34-9_3072;KKA02930.1;

889 910 80 1.000 1 3 KKA01600.1;34-9_3335;model.g4736.t1;

890 911 13 1.000 1 2 model.g4729.t1;34-9_3329;

891 912 71 1.000 1 2 model.g3546.t1;34-9_0021;

892 913 45 1.000 1 3 34-9_3337;KKA01602.1;model.g4740.t1;

893 914 11 1.000 1 2 model.g4744.t1;KKA01605.1;

894 915 6 0.502 1 3 KKA01606.1;34-9_3340;model.g4745.t1;

895 916 11 1.000 1 6 model.g3661.t1;KKA01603.1;34-9_3650;34-9_3338;KKA01317.1;model.g4741.t1;

896 917 82 1.000 1 2 model.g4735.t1;34-9_3324;

897 918 37 1.000 1 3 34-9_3326;KKA01595.1;model.g4727.t1;

898 919 70 1.000 1 2 model.g870.t1;KKA03191.1;

899 920 23 1.000 1 2 model.g4734.t1;34-9_3325;

900 921 25 1.000 1 2 model.g4712.t1;34-9_3309;

901 922 14 1.000 1 8 KKA00968.1;model.g2992.t1;KKA01012.1;KKA02419.1;KKA00966.1;34-9_0001;KKA00955.1;34-9_3062;

902 923 99 1.000 1 2 34-9_0006;KKA01038.1;

903 924 24 1.000 1 2 34-9_0019;model.g3544.t1;

904 925 99 1.000 1 2 34-9_0020;KKA02650.1;

905 926 36 1.000 1 3 KKA02649.1;model.g812.t1;34-9_0023;

906 927 11 1.000 1 2 34-9_0024;model.g2949.t1;

907 928 65 1.000 1 2 34-9_0027;model.g2946.t1;

908 929 54 1.000 1 3 KKA02613.1;model.g2955.t1;34-9_2089;

909 930 12 1.000 1 3 KKA02646.1;model.g2945.t1;34-9_0028;

910 931 29 1.000 1 3 KKA02645.1;model.g2944.t1;34-9_0029;

911 932 39 1.000 1 3 KKA02644.1;model.g2943.t1;34-9_0030;

912 933 17 1.000 1 2 34-9_0031;model.g691.t1;

913 934 82 1.000 1 3 KKA02643.1;model.g692.t1;34-9_0032;

914 935 69 1.000 1 3 34-9_2420;KKA02980.1;model.g3430.t1;

915 936 20 1.000 1 3 KKA02642.1;model.g1194.t1;34-9_0033;

916 937 99 1.000 1 2 34-9_0034;KKA02641.1;

917 938 98 1.000 1 2 34-9_0035;KKA02640.1;

918 939 99 1.000 1 2 34-9_0036;KKA02639.1;

919 940 51 1.000 1 2 34-9_0037;model.g697.t1;

920 941 98 1.000 1 2 34-9_0038;KKA02638.1;

921 942 19 1.000 1 2 34-9_0039;model.g2989.t1;

922 943 26 1.000 1 3 KKA02637.1;model.g2988.t1;34-9_0040;

923 944 60 1.000 1 2 34-9_0050;KKA02282.1;

924 945 98 1.000 1 2 34-9_0052;KKA02284.1;

925 946 50 1.000 1 2 34-9_1266;model.g3132.t1;

926 947 100 1.000 1 2 34-9_0061;KKA02291.1;

927 948 19 1.000 1 2 34-9_0072;model.g592.t1;

928 949 23 0.969 1 11 model.g2564.t1;34-9_3610;KKA02792.1;34-9_1545;model.g1882.t1;34-9_0852;model.g3535.t1;KKA02299.1;KKA03738.1;34-9_0074;KKA01553.1;

929 949 24 1.000 1 2 34-9_3609;model.g24.t1;

930 952 52 1.000 1 2 34-9_1202;model.g229.t1;

931 953 16 1.000 1 3 KKA03551.1;34-9_2131;34-9_0075;

932 953 55 1.000 1 2 34-9_1154;KKA01242.1;

933 955 48 1.000 1 3 KKA02068.1;model.g3043.t1;34-9_0076;

934 956 98 1.000 1 2 34-9_0077;KKA02067.1;

935 957 93 1.000 1 2 34-9_0078;KKA02066.1;

936 958 56 1.000 1 3 KKA02065.1;model.g3058.t1;34-9_0079;

937 959 71 1.000 1 2 model.g379.t1;model.g378.t1;

938 960 27 1.000 1 3 KKA02064.1;model.g3059.t1;34-9_0080;

939 961 62 1.000 1 2 34-9_0081;KKA02062.1;

940 962 16 0.502 1 3 KKA02060.1;model.g3061.t1;34-9_0082;

941 963 17 1.000 1 2 34-9_0083;model.g3062.t1;

942 964 8 0.667 1 5 KKA02059.1;model.g716.t1;model.g3063.t1;34-9_2105;34-9_0084;

943 965 51 1.000 1 3 KKA02058.1;model.g3064.t1;34-9_0085;

944 966 97 1.000 1 2 34-9_0086;KKA02057.1;

945 967 23 1.000 1 3 KKA02056.1;model.g3067.t1;34-9_0087;

946 968 5 0.502 1 3 KKA02238.1;34-9_1706;34-9_0089;

947 969 99 1.000 1 2 34-9_0090;KKA02055.1;

948 970 21 1.000 1 3 KKA02054.1;model.g492.t1;34-9_0091;

949 971 99 1.000 1 2 34-9_0092;KKA02053.1;

950 972 62 1.000 1 2 34-9_0094;KKA02052.1;

951 973 54 1.000 1 3 KKA02051.1;model.g429.t1;34-9_0095;

952 974 100 1.000 1 2 34-9_0098;KKA02049.1;

953 975 93 1.000 1 2 34-9_0102;KKA02046.1;

954 976 30 1.000 1 3 34-9_0114;model.g809.t1;34-9_0106;

955 977 100 1.000 1 2 34-9_0107;KKA02042.1;

956 978 98 1.000 1 2 34-9_0108;KKA02041.1;

957 979 48 1.000 1 8 KKA02081.1;KKA02040.1;model.g3021.t1;model.g3022.t1;34-9_0473;34-9_0109;model.g3023.t1;34-9_3356;

958 980 50 1.000 1 3 KKA02039.1;model.g3037.t1;34-9_0110;

959 981 30 1.000 1 3 KKA02038.1;model.g3038.t1;34-9_0111;

960 982 99 1.000 1 2 34-9_0112;KKA02037.1;

961 983 30 1.000 1 3 KKA02036.1;model.g3033.t1;34-9_0113;

962 984 36 1.000 1 3 34-9_2712;KKA02118.1;model.g1132.t1;

963 985 33 1.000 1 2 model.g1391.t1;34-9_0632;

964 986 64 1.000 1 3 KKA02035.1;model.g3025.t1;34-9_0115;

965 986 33 1.000 1 3 KKA03584.1;model.g89.t1;34-9_0688;

966 988 36 1.000 1 2 34-9_0117;model.g481.t1;

967 989 99 1.000 1 2 KKA02032.1;34-9_0119;

968 990 34 1.000 1 3 KKA02031.1;model.g478.t1;34-9_0120;

969 991 53 1.000 1 2 34-9_0124;model.g2222.t1;

970 992 96 1.000 1 2 34-9_2752;KKA03233.1;

971 993 99 1.000 1 2 34-9_0138;KKA01419.1;

972 994 98 1.000 1 2 34-9_0142;KKA01417.1;

973 995 11 1.000 1 2 34-9_0148;model.g2819.t1;

974 996 29 1.000 1 3 KKA01415.1;model.g2820.t1;34-9_0149;

975 997 49 1.000 1 3 KKA01414.1;model.g2821.t1;34-9_0150;

976 998 25 1.000 1 3 KKA01413.1;model.g2822.t1;34-9_0151;

977 999 22 1.000 1 2 34-9_0152;model.g2823.t1;

978 1000 29 1.000 1 3 KKA01412.1;model.g2824.t1;34-9_0153;

979 1001 17 1.000 1 3 KKA02433.1;model.g1235.t1;34-9_0171;

980 1002 11 1.000 1 5 KKA02431.1;34-9_0175;model.g1233.t1;34-9_0174;KKA02430.1;

981 1003 86 1.000 1 3 KKA02429.1;model.g1231.t1;34-9_0176;

982 1004 99 1.000 1 2 34-9_0177;KKA02428.1;

983 1005 13 1.000 1 3 KKA02427.1;model.g1228.t1;34-9_0179;

984 1006 81 1.000 1 2 34-9_0180;model.g1227.t1;

985 1007 43 1.000 1 2 34-9_0181;KKA02426.1;

986 1008 100 1.000 1 2 34-9_0182;KKA02424.1;

987 1009 37 1.000 1 3 34-9_3498;KKA02334.1;model.g1993.t1;

988 1010 41 1.000 1 3 KKA02422.1;model.g1222.t1;34-9_0185;

989 1011 37 1.000 1 3 KKA02421.1;model.g1221.t1;34-9_0186;

990 1012 99 1.000 1 2 34-9_0187;KKA02420.1;

991 1013 22 1.000 1 5 model.g1260.t1;model.g786.t1;KKA03085.1;34-9_0188;34-9_2533;

992 1014 98 1.000 1 2 34-9_0190;KKA01100.1;

993 1015 36 1.000 1 3 KKA01099.1;model.g1262.t1;34-9_0191;

994 1016 8 0.502 1 3 KKA01096.1;model.g1265.t1;34-9_0192;

995 1017 31 1.000 1 3 KKA01094.1;model.g1267.t1;34-9_0193;

996 1017 18 1.000 1 5 model.g1268.t1;34-9_3716;KKA01463.1;KKA01093.1;model.g883.t1;

997 1019 39 1.000 1 3 KKA01090.1;model.g1272.t1;34-9_0196;

998 1020 36 1.000 1 3 KKA01088.1;model.g1274.t1;34-9_0197;

999 1021 35 1.000 1 3 KKA01087.1;model.g1275.t1;34-9_0198;

1000 1022 64 1.000 1 3 KKA01086.1;model.g1276.t1;34-9_0199;

1001 1023 85 1.000 1 2 34-9_0200;KKA01085.1;

1002 1024 93 1.000 1 2 34-9_0201;KKA01084.1;

1003 1025 68 1.000 1 2 34-9_0202;model.g1279.t1;

1004 1026 57 1.000 1 3 KKA01060.1;model.g1280.t1;34-9_0203;

1005 1027 20 1.000 1 4 KKA01059.1;KKA01761.1;34-9_1219;34-9_0204;

1006 1028 98 1.000 1 2 34-9_0205;KKA01058.1;

1007 1029 33 1.000 1 2 34-9_0206;model.g1284.t1;

1008 1030 71 1.000 1 3 KKA01057.1;model.g1286.t1;34-9_0208;

1009 1031 45 1.000 1 2 34-9_0209;KKA01056.1;

1010 1032 98 1.000 1 2 34-9_0210;KKA01055.1;

1011 1033 58 1.000 1 3 KKA01054.1;model.g1289.t1;34-9_0211;

1012 1034 16 1.000 1 12 KKA01123.1;34-9_0212;model.g2993.t1;34-9_0213;model.g2994.t1;34-9_2635;model.g2995.t1;model.g1290.t1;KKA01052.1;KKA01053.1;model.g2996.t1;model.g209.t1;

1013 1035 60 1.000 1 2 34-9_0214;KKA02783.1;

1014 1036 66 1.000 1 3 KKA02782.1;model.g398.t1;34-9_0215;

1015 1037 16 1.000 1 3 KKA02781.1;model.g399.t1;34-9_0216;

1016 1038 84 1.000 1 2 34-9_0217;KKA02780.1;

1017 1039 34 1.000 1 3 KKA02778.1;model.g2470.t1;34-9_0219;

1018 1040 70 1.000 1 3 KKA02777.1;model.g2468.t1;34-9_0220;

1019 1041 98 1.000 1 2 34-9_0222;KKA02775.1;

1020 1042 75 1.000 1 2 34-9_0224;KKA02773.1;

1021 1043 99 1.000 1 2 34-9_0233;KKA02765.1;

1022 1043 22 1.000 1 3 model.g1923.t1;model.g19.t1;model.g1922.t1;

1023 1045 61 1.000 1 2 34-9_0235;KKA02762.1;

1024 1046 30 1.000 1 3 34-9_0810;KKA03699.1;model.g1839.t1;

1025 1047 99 1.000 1 2 34-9_0241;KKA02756.1;

1026 1048 99 1.000 1 2 34-9_0248;KKA02750.1;

1027 1049 99 1.000 1 2 34-9_0250;KKA02748.1;

1028 1050 51 1.000 1 3 KKA02747.1;model.g487.t1;34-9_0251;

1029 1051 99 1.000 1 2 34-9_0253;KKA02744.1;

1030 1052 51 1.000 1 3 KKA02742.1;model.g3029.t1;34-9_0255;

1031 1053 82 1.000 1 3 KKA02741.1;model.g3030.t1;34-9_0256;

1032 1054 47 1.000 1 3 KKA02736.1;model.g408.t1;34-9_0261;

1033 1054 30 1.000 1 3 KKA02683.1;model.g1472.t1;34-9_3414;

1034 1056 82 1.000 1 3 KKA02735.1;model.g407.t1;34-9_0262;

1035 1057 7 0.667 1 4 KKA02734.1;model.g396.t1;model.g1844.t1;34-9_0263;

1036 1058 42 1.000 1 3 KKA02733.1;model.g409.t1;34-9_0264;

1037 1060 6 0.427 1 8 model.g872.t1;KKA02732.1;KKA03697.1;model.g1841.t1;KKA03193.1;34-9_0265;34-9_0808;model.g2187.t1;

1038 1061 12 1.000 1 3 KKA02731.1;model.g412.t1;34-9_0266;

1039 1062 58 1.000 1 3 KKA02728.1;model.g417.t1;34-9_0269;

1040 1063 52 1.000 1 3 KKA02727.1;model.g419.t1;34-9_0270;

1041 1064 14 1.000 1 9 34-9_0271;34-9_3078;model.g2781.t1;34-9_0969;model.g2791.t1;model.g420.t1;KKA02726.1;KKA03846.1;model.g2096.t1;

1042 1064 100 1.000 1 2 KKA02924.1;34-9_3085;

1043 1066 64 1.000 1 3 KKA02725.1;model.g421.t1;34-9_0272;

1044 1067 6 0.502 1 6 model.g1864.t1;KKA00954.1;model.g21.t1;model.g1865.t1;34-9_0273;model.g3534.t1;

1045 1068 19 1.000 1 3 KKA01401.1;model.g423.t1;34-9_0275;

1046 1069 18 1.000 1 3 KKA01400.1;model.g424.t1;34-9_0276;

1047 1070 25 1.000 1 3 KKA01399.1;model.g3070.t1;34-9_0277;

1048 1071 42 1.000 1 3 KKA01398.1;model.g3071.t1;34-9_0278;

1049 1072 5 0.443 1 6 model.g3155.t1;KKA01397.1;34-9_2370;model.g3073.t1;KKA03305.1;34-9_0279;

1050 1073 95 1.000 1 2 34-9_0280;KKA01396.1;

1051 1074 61 1.000 1 2 34-9_0281;model.g659.t1;

1052 1075 48 1.000 1 2 34-9_0282;KKA01395.1;

1053 1076 38 1.000 1 2 34-9_0283;model.g2259.t1;

1054 1077 42 1.000 1 2 34-9_0285;model.g570.t1;

1055 1078 12 1.000 1 2 34-9_0286;model.g569.t1;

1056 1079 13 1.000 1 2 34-9_0288;model.g567.t1;

1057 1080 31 1.000 1 10 KKA02690.1;34-9_1621;KKA02867.1;34-9_3704;model.g1583.t1;model.g200.t1;model.g552.t1;model.g1733.t1;34-9_0289;34-9_3404;

1058 1081 35 1.000 1 2 34-9_0290;model.g551.t1;

1059 1082 66 1.000 1 2 34-9_0294;model.g549.t1;

1060 1083 42 1.000 1 2 34-9_3152;model.g121.t1;

1061 1084 12 1.000 1 2 34-9_0297;model.g550.t1;

1062 1085 20 1.000 1 2 34-9_0299;model.g541.t1;

1063 1086 25 1.000 1 2 34-9_0300;model.g540.t1;

1064 1087 56 1.000 1 2 34-9_0301;model.g539.t1;

1065 1088 27 1.000 1 2 34-9_0304;model.g534.t1;

1066 1089 36 1.000 1 2 34-9_0306;model.g533.t1;

1067 1090 37 1.000 1 2 34-9_0309;model.g530.t1;

1068 1091 56 1.000 1 2 34-9_0312;model.g2510.t1;

1069 1092 33 1.000 1 2 34-9_0315;model.g520.t1;

1070 1093 45 1.000 1 5 model.g3017.t1;model.g57.t1;34-9_0641;34-9_0320;model.g2346.t1;

1071 1094 29 1.000 1 2 34-9_0321;model.g3018.t1;

1072 1095 29 1.000 1 2 34-9_0323;model.g3011.t1;

1073 1096 13 1.000 1 5 model.g3009.t1;34-9_3632;model.g3646.t1;34-9_0324;KKA01300.1;

1074 1097 43 1.000 1 2 34-9_0325;model.g2997.t1;

1075 1098 10 1.000 1 2 34-9_0326;model.g2999.t1;

1076 1099 21 1.000 1 2 34-9_0327;model.g3001.t1;

1077 1100 74 1.000 1 2 34-9_0337;KKA01893.1;

1078 1101 33 1.000 1 3 KKA01891.1;model.g3101.t1;34-9_0339;

1079 1102 31 1.000 1 2 34-9_0340;model.g3102.t1;

1080 1103 27 1.000 1 5 KKA01890.1;34-9_1305;model.g3103.t1;model.g3105.t1;34-9_0341;

1081 1104 12 1.000 1 3 KKA01889.1;model.g1946.t1;34-9_0342;

1082 1105 14 1.000 1 3 KKA01888.1;model.g1945.t1;34-9_0343;

1083 1106 90 1.000 1 2 34-9_0349;KKA01886.1;

1084 1107 45 1.000 1 6 34-9_1823;KKA00958.1;model.g1944.t1;KKA02182.1;model.g22.t1;34-9_0352;

1085 1108 68 1.000 1 2 34-9_0354;model.g1937.t1;

1086 1109 14 1.000 1 3 model.g1939.t1;KKA03104.1;34-9_0356;

1087 1110 99 1.000 1 2 KKA02319.1;34-9_3481;

1088 1111 20 1.000 1 3 KKA01508.1;model.g1935.t1;34-9_0357;

1089 1112 43 1.000 1 3 KKA01509.1;model.g1934.t1;34-9_0358;

1090 1113 20 1.000 1 3 KKA01510.1;model.g1933.t1;34-9_0359;

1091 1114 78 1.000 1 3 KKA01511.1;model.g177.t1;34-9_0360;

1092 1115 30 1.000 1 2 34-9_0361;model.g176.t1;

1093 1116 14 1.000 1 3 KKA01512.1;model.g175.t1;34-9_0362;

1094 1117 74 1.000 1 3 KKA01513.1;model.g173.t1;34-9_0363;

1095 1118 19 1.000 1 2 34-9_0365;model.g1947.t1;

1096 1119 28 1.000 1 4 KKA00971.1;KKA01885.1;model.g2287.t1;34-9_1914;

1097 1120 99 1.000 1 2 34-9_0368;KKA01515.1;

1098 1121 14 0.890 1 12 34-9_3091;34-9_0370;KKA03770.1;34-9_3010;34-9_0888;KKA02919.1;34-9_3180;model.g50.t1;model.g1309.t1;model.g2454.t1;model.g1618.t1;KKA02873.1;

1099 1122 16 1.000 1 3 KKA01516.1;model.g656.t1;34-9_0371;

1100 1123 100 1.000 1 2 34-9_0373;KKA01518.1;

1101 1124 61 1.000 1 2 34-9_0376;KKA01520.1;

1102 1125 67 1.000 1 3 KKA01525.1;model.g2011.t1;34-9_0383;

1103 1126 13 1.000 1 3 KKA01526.1;model.g1950.t1;34-9_0384;

1104 1127 32 1.000 1 3 KKA01527.1;model.g231.t1;34-9_0386;

1105 1128 59 1.000 1 3 KKA01528.1;model.g232.t1;34-9_0387;

1106 1129 87 1.000 1 2 34-9_0388;model.g233.t1;

1107 1130 56 1.000 1 3 34-9_0973;model.g2093.t1;KKA03850.1;

1108 1131 97 1.000 1 2 34-9_0389;KKA01529.1;

1109 1132 36 1.000 1 2 34-9_0390;model.g2577.t1;

1110 1133 33 1.000 1 3 KKA01530.1;model.g454.t1;34-9_0391;

1111 1134 15 1.000 1 3 KKA01532.1;model.g460.t1;34-9_0395;

1112 1135 20 1.000 1 2 34-9_0397;model.g464.t1;

1113 1136 23 1.000 1 3 KKA01533.1;model.g465.t1;34-9_0398;

1114 1137 75 1.000 1 2 34-9_0399;KKA02022.1;

1115 1138 55 1.000 1 3 KKA02023.1;model.g467.t1;34-9_0400;

1116 1139 27 1.000 1 3 KKA02024.1;model.g468.t1;34-9_0401;

1117 1140 38 1.000 1 3 KKA02025.1;model.g469.t1;34-9_0402;

1118 1141 39 1.000 1 3 KKA02026.1;model.g470.t1;34-9_0403;

1119 1142 21 1.000 1 3 KKA02027.1;model.g471.t1;34-9_0404;

1120 1143 98 1.000 1 2 34-9_0405;KKA02028.1;

1121 1144 81 1.000 1 2 34-9_0406;KKA02219.1;

1122 1145 55 1.000 1 5 KKA02220.1;model.g3276.t1;model.g948.t1;34-9_2451;34-9_0407;

1123 1146 71 1.000 1 3 KKA02221.1;model.g949.t1;34-9_0408;

1124 1147 46 1.000 1 2 34-9_0409;model.g950.t1;

1125 1148 44 1.000 1 3 KKA02222.1;model.g951.t1;34-9_0410;

1126 1149 17 1.000 1 3 KKA02223.1;model.g952.t1;34-9_0411;

1127 1150 12 1.000 1 5 model.g268.t1;model.g3097.t1;34-9_0488;34-9_0412;KKA02092.1;

1128 1151 37 1.000 1 3 KKA02224.1;model.g269.t1;34-9_0413;

1129 1152 41 1.000 1 2 34-9_0414;KKA02225.1;

1130 1153 46 1.000 1 3 KKA02226.1;model.g260.t1;34-9_0415;

1131 1154 44 1.000 1 3 KKA02227.1;model.g264.t1;34-9_0416;

1132 1155 62 1.000 1 2 34-9_0417;model.g265.t1;

1133 1156 57 1.000 1 3 KKA02229.1;model.g2536.t1;34-9_0420;

1134 1156 51 1.000 1 2 34-9_2084;model.g831.t1;

1135 1158 11 1.000 1 2 34-9_0422;model.g2534.t1;

1136 1159 33 1.000 1 2 34-9_0423;model.g2533.t1;

1137 1160 11 1.000 1 3 KKA02492.1;model.g170.t1;34-9_0425;

1138 1161 37 1.000 1 2 34-9_0426;model.g171.t1;

1139 1162 43 1.000 1 2 34-9_0429;model.g581.t1;

1140 1163 57 1.000 1 2 34-9_0430;KKA02494.1;

1141 1164 23 1.000 1 2 34-9_0431;model.g579.t1;

1142 1165 25 1.000 1 4 KKA02495.1;model.g2522.t1;model.g2513.t1;34-9_0432;

1143 1166 54 1.000 1 2 34-9_0433;model.g235.t1;

1144 1167 99 1.000 1 2 34-9_0435;KKA02496.1;

1145 1168 74 1.000 1 2 34-9_0438;KKA02498.1;

1146 1169 29 1.000 1 3 KKA02499.1;model.g147.t1;34-9_0439;

1147 1170 14 1.000 1 2 34-9_0440;model.g144.t1;

1148 1171 31 1.000 1 5 KKA02500.1;model.g143.t1;34-9_1687;KKA01727.1;34-9_0441;

1149 1172 47 1.000 1 2 34-9_0442;model.g1566.t1;

1150 1173 99 1.000 1 2 34-9_0444;KKA02501.1;

1151 1174 29 1.000 1 5 KKA02502.1;model.g2339.t1;model.g919.t1;34-9_0646;34-9_0447;

1152 1175 21 1.000 1 2 34-9_0448;model.g926.t1;

1153 1176 99 1.000 1 2 34-9_0449;KKA02503.1;

1154 1177 33 1.000 1 2 34-9_0450;KKA02504.1;

1155 1178 58 1.000 1 3 KKA02505.1;model.g921.t1;34-9_0451;

1156 1179 22 1.000 1 5 model.g3045.t1;34-9_3368;model.g731.t1;34-9_0452;KKA02716.1;

1157 1180 59 1.000 1 2 34-9_0453;KKA02506.1;

1158 1181 48 1.000 1 2 34-9_0455;KKA02507.1;

1159 1182 89 1.000 1 3 KKA02508.1;model.g930.t1;34-9_0456;

1160 1183 14 1.000 1 2 34-9_0457;model.g931.t1;

1161 1184 7 0.502 1 3 model.g932.t1;model.g934.t1;34-9_0458;

1162 1185 21 1.000 1 2 34-9_0459;model.g935.t1;

1163 1186 6 0.502 1 3 KKA02509.1;model.g252.t1;34-9_0460;

1164 1187 26 1.000 1 2 34-9_0461;model.g253.t1;

1165 1188 56 1.000 1 2 34-9_0462;model.g254.t1;

1166 1189 73 1.000 1 2 34-9_0463;KKA02510.1;

1167 1190 30 1.000 1 3 KKA02072.1;model.g2961.t1;34-9_0464;

1168 1190 24 1.000 1 2 model.g3039.t1;KKA02071.1;

1169 1192 99 1.000 1 2 34-9_0465;KKA02073.1;

1170 1193 65 1.000 1 3 34-9_0874;KKA03756.1;model.g1387.t1;

1171 1194 99 1.000 1 2 34-9_0469;KKA02077.1;

1172 1195 19 1.000 1 3 KKA02078.1;model.g3054.t1;34-9_0470;

1173 1196 17 1.000 1 2 34-9_3386;model.g1511.t1;

1174 1197 100 1.000 1 2 34-9_0471;KKA02079.1;

1175 1198 26 1.000 1 3 KKA02080.1;model.g3057.t1;34-9_0472;

1176 1199 11 1.000 1 2 34-9_0475;model.g3036.t1;

1177 1200 25 1.000 1 3 KKA02083.1;model.g430.t1;34-9_0476;

1178 1201 17 1.000 1 3 KKA02084.1;model.g431.t1;34-9_0477;

1179 1202 34 1.000 1 3 KKA02087.1;model.g3074.t1;34-9_0481;

1180 1203 24 1.000 1 3 KKA02088.1;model.g3076.t1;34-9_0482;

1181 1204 9 0.502 1 5 model.g3077.t1;34-9_2867;model.g701.t1;34-9_0483;KKA01283.1;

1182 1205 99 1.000 1 2 34-9_0487;KKA02091.1;

1183 1206 56 1.000 1 3 KKA02093.1;model.g3098.t1;34-9_0489;

1184 1207 34 1.000 1 3 KKA02094.1;model.g3094.t1;34-9_0490;

1185 1208 38 1.000 1 3 KKA02095.1;model.g3093.t1;34-9_0491;

1186 1209 34 1.000 1 3 KKA02096.1;model.g3092.t1;34-9_0493;

1187 1210 23 1.000 1 3 KKA02097.1;model.g3090.t1;34-9_0494;

1188 1211 40 1.000 1 3 KKA00981.1;model.g3089.t1;34-9_0496;

1189 1212 37 1.000 1 3 KKA00984.1;model.g3085.t1;34-9_0498;

1190 1213 13 1.000 1 3 KKA00985.1;model.g3084.t1;34-9_0499;

1191 1214 30 1.000 1 5 KKA01366.1;model.g897.t1;model.g3083.t1;34-9_3729;34-9_0500;

1192 1215 39 1.000 1 3 KKA01367.1;model.g3082.t1;34-9_0501;

1193 1216 40 1.000 1 3 KKA01368.1;model.g3081.t1;34-9_0502;

1194 1217 17 1.000 1 3 KKA01369.1;model.g3080.t1;34-9_0503;

1195 1217 39 1.000 1 3 KKA01576.1;model.g2348.t1;34-9_0639;

1196 1219 76 1.000 1 3 KKA01370.1;model.g3504.t1;34-9_0504;

1197 1220 46 1.000 1 3 KKA01371.1;model.g3505.t1;34-9_0505;

1198 1221 10 1.000 1 3 KKA01372.1;model.g3508.t1;34-9_0507;

1199 1222 92 1.000 1 2 34-9_0508;KKA01373.1;

1200 1223 64 1.000 1 3 KKA01374.1;model.g3510.t1;34-9_0509;

1201 1224 21 1.000 1 3 KKA01375.1;model.g3512.t1;34-9_0510;

1202 1225 33 1.000 1 3 KKA01376.1;model.g3513.t1;34-9_0511;

1203 1226 99 1.000 1 2 34-9_0512;KKA01377.1;

1204 1227 20 0.502 1 3 KKA01378.1;KKA01379.1;34-9_0513;

1205 1228 100 1.000 1 2 34-9_0514;KKA01380.1;

1206 1229 99 1.000 1 2 34-9_0515;KKA01381.1;

1207 1230 100 1.000 1 2 34-9_0516;KKA01382.1;

1208 1231 99 1.000 1 2 34-9_0517;KKA01383.1;

1209 1232 96 1.000 1 2 34-9_0518;model.g3572.t1;

1210 1233 79 1.000 1 2 34-9_0519;KKA01385.1;

1211 1234 33 1.000 1 3 KKA01386.1;model.g3574.t1;34-9_0520;

1212 1235 99 1.000 1 2 34-9_0521;KKA01389.1;

1213 1236 99 1.000 1 2 34-9_0522;KKA01390.1;

1214 1237 39 1.000 1 3 KKA01391.1;model.g2572.t1;34-9_0523;

1215 1238 63 1.000 1 2 34-9_0524;KKA01392.1;

1216 1239 17 1.000 1 3 KKA01393.1;model.g2569.t1;34-9_0525;

1217 1240 22 1.000 1 3 KKA01432.1;model.g3590.t1;34-9_0528;

1218 1241 20 1.000 1 2 34-9_0529;model.g3591.t1;

1219 1242 34 1.000 1 2 34-9_0530;model.g3592.t1;

1220 1243 55 1.000 1 3 KKA01434.1;model.g3596.t1;34-9_0532;

1221 1244 36 1.000 1 3 KKA01435.1;model.g3597.t1;34-9_0533;

1222 1245 36 1.000 1 4 KKA01436.1;model.g2105.t1;model.g2107.t1;34-9_0534;

1223 1246 15 1.000 1 2 34-9_0537;model.g3599.t1;

1224 1247 34 1.000 1 4 KKA01437.1;KKA01016.1;KKA00972.1;34-9_0539;

1225 1248 45 1.000 1 2 34-9_0541;model.g3589.t1;

1226 1249 29 1.000 1 3 KKA01439.1;model.g3588.t1;34-9_0542;

1227 1250 18 1.000 1 2 34-9_0544;model.g3585.t1;

1228 1251 12 0.820 1 18 34-9_3619;KKA02110.1;model.g1119.t1;34-9_0545;KKA02212.1;KKA01558.1;34-9_0555;model.g2556.t1;model.g189.t1;KKA02829.1;model.g442.t1;model.g3582.t1;34-9_1581;KKA01441.1;34-9_2721;KKA01446.1;34-9_1871;model.g316.t1;

1229 1252 42 1.000 1 2 34-9_0546;model.g3581.t1;

1230 1253 63 1.000 1 3 34-9_2990;KKA01635.1;model.g2756.t1;

1231 1254 29 1.000 1 3 KKA02926.1;model.g2782.t1;34-9_3079;

1232 1255 43 1.000 1 2 34-9_0549;model.g3578.t1;

1233 1256 61 1.000 1 2 34-9_0550;model.g301.t1;

1234 1257 18 1.000 1 3 34-9_1578;KKA02825.1;model.g488.t1;

1235 1258 12 1.000 1 3 KKA01442.1;model.g321.t1;34-9_0551;

1236 1259 100 1.000 1 2 34-9_0552;KKA01443.1;

1237 1260 23 1.000 1 2 34-9_0554;model.g318.t1;

1238 1261 11 1.000 1 2 34-9_0556;model.g315.t1;

1239 1262 24 1.000 1 4 KKA01449.1;KKA01686.1;model.g314.t1;34-9_0557;

1240 1263 92 1.000 1 4 KKA01450.1;KKA01687.1;model.g313.t1;34-9_0558;

1241 1264 20 1.000 1 3 KKA01688.1;model.g312.t1;34-9_0559;

1242 1265 50 1.000 1 5 KKA01689.1;34-9_1940;model.g311.t1;model.g1158.t1;34-9_0560;

1243 1266 12 1.000 1 5 34-9_0629;34-9_0958;model.g2147.t1;model.g2414.t1;KKA03836.1;

1244 1267 25 1.000 1 3 KKA01691.1;model.g300.t1;34-9_0564;

1245 1268 11 1.000 1 6 model.g2971.t1;KKA01692.1;34-9_2068;model.g298.t1;KKA02603.1;34-9_0565;

1246 1268 55 1.000 1 3 KKA03753.1;model.g2407.t1;34-9_0871;

1247 1270 12 0.667 1 4 KKA01693.1;model.g296.t1;model.g297.t1;34-9_0566;

1248 1271 21 0.502 1 3 KKA01694.1;KKA01695.1;34-9_0567;

1249 1272 100 1.000 1 2 34-9_0569;KKA01696.1;

1250 1273 42 1.000 1 3 KKA01697.1;model.g292.t1;34-9_0570;

1251 1274 24 1.000 1 3 KKA01698.1;model.g291.t1;34-9_0571;

1252 1275 16 1.000 1 2 34-9_0573;model.g288.t1;

1253 1276 18 1.000 1 3 KKA02791.1;model.g199.t1;34-9_1544;

1254 1277 21 1.000 1 2 model.g979.t1;34-9_3037;

1255 1278 40 1.000 1 2 34-9_0574;model.g287.t1;

1256 1279 100 1.000 1 2 34-9_0576;KKA01701.1;

1257 1280 24 1.000 1 3 KKA01702.1;model.g285.t1;34-9_0579;

1258 1281 22 1.000 1 5 KKA01583.1;34-9_0652;34-9_0651;34-9_0580;KKA01582.1;

1259 1282 98 1.000 1 2 34-9_0583;KKA01704.1;

1260 1283 97 1.000 1 2 34-9_0586;KKA01705.1;

1261 1284 83 1.000 1 2 34-9_0589;KKA01707.1;

1262 1285 55 1.000 1 2 34-9_0591;KKA01710.1;

1263 1286 91 1.000 1 2 34-9_0593;KKA01712.1;

1264 1287 39 1.000 1 2 34-9_0598;KKA01714.1;

1265 1288 16 1.000 1 2 34-9_0599;model.g281.t1;

1266 1289 83 1.000 1 2 34-9_0600;model.g278.t1;

1267 1290 51 1.000 1 3 KKA01715.1;model.g1399.t1;34-9_0602;

1268 1291 37 1.000 1 6 model.g3530.t1;KKA01716.1;KKA02859.1;model.g1400.t1;34-9_1614;34-9_0603;

1269 1292 51 1.000 1 2 34-9_0604;model.g1401.t1;

1270 1293 79 1.000 1 3 KKA01717.1;model.g1402.t1;34-9_0605;

1271 1294 64 1.000 1 2 34-9_0607;model.g1404.t1;

1272 1295 15 1.000 1 4 KKA01718.1;model.g1406.t1;model.g2829.t1;34-9_0608;

1273 1295 9 0.502 1 4 KKA01719.1;34-9_0609;model.g2561.t1;34-9_3613;

1274 1297 15 1.000 1 2 34-9_0610;model.g1407.t1;

1275 1298 16 1.000 1 5 KKA01720.1;34-9_2060;model.g1408.t1;model.g2974.t1;34-9_0611;

1276 1299 12 1.000 1 3 34-9_2498;KKA03051.1;model.g3215.t1;

1277 1300 41 1.000 1 2 34-9_0614;KKA01722.1;

1278 1301 16 1.000 1 3 KKA01723.1;model.g272.t1;34-9_0615;

1279 1302 90 1.000 1 2 34-9_0616;KKA01724.1;

1280 1303 67 1.000 1 2 34-9_0618;KKA01562.1;

1281 1304 26 1.000 1 2 34-9_0620;model.g276.t1;

1282 1305 15 1.000 1 3 34-9_3486;model.g426.t1;KKA02323.1;

1283 1306 94 1.000 1 2 34-9_0622;KKA01564.1;

1284 1307 22 1.000 1 2 34-9_0623;model.g1396.t1;

1285 1308 22 1.000 1 3 KKA01567.1;model.g1394.t1;34-9_0624;

1286 1309 99 1.000 1 2 34-9_0625;KKA01568.1;

1287 1310 36 1.000 1 3 KKA01569.1;model.g3192.t1;34-9_0626;

1288 1310 16 1.000 1 2 model.g2410.t1;KKA01570.1;

1289 1312 34 1.000 1 3 KKA01571.1;model.g2411.t1;34-9_0627;

1290 1313 16 1.000 1 2 34-9_0628;model.g2413.t1;

1291 1314 48 1.000 1 2 34-9_0630;model.g1389.t1;

1292 1315 21 1.000 1 2 34-9_0631;model.g1390.t1;

1293 1316 36 1.000 1 3 KKA01572.1;model.g2409.t1;34-9_0633;

1294 1317 33 1.000 1 2 34-9_0634;model.g2408.t1;

1295 1318 99 1.000 1 2 34-9_0638;KKA01575.1;

1296 1319 66 1.000 1 2 34-9_0640;KKA01577.1;

1297 1320 100 1.000 1 2 34-9_0642;KKA01578.1;

1298 1321 29 1.000 1 2 34-9_0643;model.g2343.t1;

1299 1322 80 1.000 1 2 34-9_0644;KKA01579.1;

1300 1323 15 1.000 1 2 34-9_0648;model.g2337.t1;

1301 1324 7 0.502 1 3 KKA01581.1;model.g2336.t1;34-9_0649;

1302 1325 27 1.000 1 2 34-9_0650;model.g2335.t1;

1303 1326 28 1.000 1 4 KKA01042.1;KKA03553.1;model.g2334.t1;34-9_0653;

1304 1327 60 1.000 1 3 KKA03555.1;model.g2331.t1;34-9_0655;

1305 1328 41 1.000 1 2 34-9_0656;model.g2330.t1;

1306 1329 99 1.000 1 2 34-9_0660;KKA03558.1;

1307 1330 100 1.000 1 2 34-9_0661;KKA03559.1;

1308 1331 99 1.000 1 2 34-9_0670;KKA03569.1;

1309 1332 98 1.000 1 2 34-9_0671;KKA03570.1;

1310 1333 100 1.000 1 2 34-9_0673;KKA03572.1;

1311 1334 99 1.000 1 2 34-9_0674;KKA03573.1;

1312 1335 92 1.000 1 2 34-9_0686;KKA03582.1;

1313 1336 99 1.000 1 2 34-9_0691;KKA03586.1;

1314 1337 58 1.000 1 2 34-9_0694;KKA03588.1;

1315 1338 12 0.855 1 21 34-9_0695;model.g798.t1;KKA02169.1;model.g198.t1;model.g3126.t1;KKA03918.1;34-9_1803;34-9_2328;34-9_1049;model.g657.t1;model.g2062.t1;34-9_2810;model.g2162.t1;model.g3127.t1;KKA03346.1;KKA03589.1;34-9_1261;model.g2161.t1;KKA01394.1;model.g2163.t1;model.g797.t1;

1316 1339 24 1.000 1 4 KKA03595.1;model.g695.t1;model.g2302.t1;34-9_0700;

1317 1340 88 1.000 1 2 34-9_0708;KKA03603.1;

1318 1341 78 1.000 1 2 34-9_0711;KKA03605.1;

1319 1342 70 1.000 1 2 34-9_0712;KKA03607.1;

1320 1343 95 1.000 1 2 34-9_0713;KKA03608.1;

1321 1344 99 1.000 1 2 34-9_0714;KKA03609.1;

1322 1345 95 1.000 1 2 34-9_0715;KKA03610.1;

1323 1346 98 1.000 1 2 34-9_0716;KKA03611.1;

1324 1347 98 1.000 1 2 34-9_0717;KKA03612.1;

1325 1348 99 1.000 1 2 34-9_0719;KKA03614.1;

1326 1349 92 1.000 1 2 34-9_0725;KKA03618.1;

1327 1350 97 1.000 1 2 34-9_0731;KKA03623.1;

1328 1351 42 1.000 1 3 KKA03630.1;model.g2462.t1;34-9_0740;

1329 1352 73 1.000 1 2 34-9_0742;KKA03632.1;

1330 1353 32 1.000 1 3 KKA03634.1;model.g2543.t1;34-9_0744;

1331 1354 100 1.000 1 2 34-9_0745;KKA03635.1;

1332 1355 22 1.000 1 3 KKA03636.1;model.g2545.t1;34-9_0746;

1333 1356 98 1.000 1 2 34-9_0747;KKA03637.1;

1334 1357 22 1.000 1 3 KKA03638.1;model.g2548.t1;34-9_0748;

1335 1358 70 1.000 1 3 KKA03639.1;model.g2353.t1;34-9_0749;

1336 1359 20 1.000 1 3 KKA03640.1;model.g2354.t1;34-9_0750;

1337 1360 29 1.000 1 2 34-9_0751;model.g2355.t1;

1338 1361 14 1.000 1 3 KKA03645.1;model.g2359.t1;34-9_0754;

1339 1362 28 1.000 1 3 KKA03647.1;model.g2362.t1;34-9_0755;

1340 1363 99 1.000 1 2 34-9_0756;KKA03648.1;

1341 1364 13 1.000 1 2 34-9_0757;model.g2364.t1;

1342 1365 15 1.000 1 3 KKA03649.1;model.g2365.t1;34-9_0758;

1343 1366 99 1.000 1 2 34-9_0759;KKA03650.1;

1344 1367 96 1.000 1 2 34-9_0760;KKA03651.1;

1345 1368 23 1.000 1 3 KKA03652.1;model.g2368.t1;34-9_0761;

1346 1369 11 1.000 1 3 KKA03653.1;model.g2369.t1;34-9_0762;

1347 1370 27 1.000 1 3 KKA03654.1;model.g2370.t1;34-9_0763;

1348 1371 18 1.000 1 3 KKA03655.1;model.g2373.t1;34-9_0764;

1349 1372 75 1.000 1 2 34-9_0765;KKA03656.1;

1350 1373 99 1.000 1 2 34-9_0766;KKA03658.1;

1351 1374 34 1.000 1 3 KKA03659.1;model.g2375.t1;34-9_0767;

1352 1375 27 1.000 1 3 KKA03662.1;model.g2378.t1;34-9_0770;

1353 1376 8 0.780 1 6 34-9_1413;KKA03663.1;model.g1698.t1;model.g2379.t1;KKA01674.1;34-9_0771;

1354 1377 61 1.000 1 2 34-9_0773;KKA03665.1;

1355 1378 16 1.000 1 3 KKA03667.1;model.g2384.t1;34-9_0775;

1356 1379 15 1.000 1 2 34-9_0776;model.g2389.t1;

1357 1380 43 1.000 1 3 KKA03669.1;model.g2390.t1;34-9_0778;

1358 1381 11 1.000 1 3 KKA03670.1;model.g2396.t1;34-9_0779;

1359 1382 90 1.000 1 2 34-9_0781;KKA03672.1;

1360 1383 99 1.000 1 2 34-9_0782;KKA03673.1;

1361 1384 47 1.000 1 2 34-9_0783;KKA03674.1;

1362 1385 87 1.000 1 2 34-9_0784;KKA03675.1;

1363 1386 70 1.000 1 3 KKA03676.1;model.g1877.t1;34-9_0785;

1364 1387 13 1.000 1 3 KKA03677.1;model.g1879.t1;34-9_0786;

1365 1388 98 1.000 1 2 34-9_0788;KKA03679.1;

1366 1389 100 1.000 1 2 34-9_0789;KKA03680.1;

1367 1390 98 1.000 1 2 34-9_0790;KKA03681.1;

1368 1391 74 1.000 1 2 34-9_0791;KKA03683.1;

1369 1392 11 1.000 1 3 KKA03684.1;model.g1861.t1;34-9_0792;

1370 1393 98 1.000 1 2 34-9_0793;KKA03685.1;

1371 1394 87 1.000 1 3 KKA03686.1;model.g1859.t1;34-9_0794;

1372 1395 58 1.000 1 3 KKA03687.1;model.g1858.t1;34-9_0795;

1373 1396 29 1.000 1 3 KKA03688.1;model.g1857.t1;34-9_0796;

1374 1397 83 1.000 1 2 34-9_0798;model.g1855.t1;

1375 1398 69 1.000 1 3 KKA03690.1;model.g1852.t1;34-9_0801;

1376 1399 30 1.000 1 3 KKA03691.1;model.g1851.t1;34-9_0802;

1377 1400 28 1.000 1 3 KKA03692.1;model.g1850.t1;34-9_0803;

1378 1401 19 1.000 1 4 KKA03693.1;model.g1849.t1;model.g3638.t1;34-9_0804;

1379 1402 55 1.000 1 2 34-9_0805;KKA03694.1;

1380 1403 98 1.000 1 2 34-9_0806;KKA03695.1;

1381 1404 16 1.000 1 3 KKA03696.1;model.g1843.t1;34-9_0807;

1382 1405 36 1.000 1 3 34-9_3130;KKA02903.1;model.g94.t1;

1383 1406 33 1.000 1 3 KKA03700.1;model.g1838.t1;34-9_0811;

1384 1407 99 1.000 1 2 34-9_0812;KKA03701.1;

1385 1408 30 1.000 1 3 KKA03702.1;model.g1836.t1;34-9_0813;

1386 1409 6 0.502 1 3 KKA03703.1;model.g2469.t1;34-9_0814;

1387 1410 47 1.000 1 3 KKA03704.1;model.g1835.t1;34-9_0815;

1388 1411 25 1.000 1 3 KKA03707.1;model.g1831.t1;34-9_0817;

1389 1412 98 1.000 1 2 34-9_0818;KKA03708.1;

1390 1413 17 1.000 1 3 KKA03710.1;model.g1830.t1;34-9_0819;

1391 1415 27 1.000 1 3 KKA03711.1;model.g1847.t1;34-9_0821;

1392 1416 98 1.000 1 2 34-9_0824;KKA03712.1;

1393 1417 99 1.000 1 2 34-9_0825;KKA03713.1;

1394 1418 24 1.000 1 6 model.g2477.t1;KKA03714.1;34-9_3482;model.g2418.t1;KKA02320.1;34-9_0826;

1395 1419 98 1.000 1 2 34-9_0828;KKA03716.1;

1396 1420 21 1.000 1 3 KKA03717.1;model.g2422.t1;34-9_0829;

1397 1421 56 1.000 1 2 34-9_0830;model.g2423.t1;

1398 1422 30 1.000 1 3 34-9_1898;KKA01870.1;model.g2309.t1;

1399 1423 55 1.000 1 2 34-9_0831;KKA03718.1;

1400 1424 18 1.000 1 3 KKA03719.1;model.g2425.t1;34-9_0832;

1401 1425 16 0.776 1 6 model.g2620.t1;KKA03720.1;34-9_1365;model.g2426.t1;KKA01025.1;34-9_0833;

1402 1426 14 1.000 1 3 KKA03721.1;model.g2427.t1;34-9_0834;

1403 1427 15 1.000 1 3 KKA03722.1;model.g2428.t1;34-9_0835;

1404 1428 73 1.000 1 2 34-9_0837;KKA03724.1;

1405 1429 31 1.000 1 3 KKA03725.1;model.g2433.t1;34-9_0838;

1406 1430 72 1.000 1 3 KKA03726.1;model.g2431.t1;34-9_0839;

1407 1431 58 1.000 1 4 KKA00974.1;KKA03728.1;model.g2430.t1;34-9_0841;

1408 1432 8 0.835 1 7 KKA02861.1;KKA00975.1;model.g3532.t1;KKA03729.1;model.g2438.t1;34-9_0842;34-9_1616;

1409 1433 73 1.000 1 3 34-9_2660;KKA02151.1;model.g1080.t1;

1410 1434 56 1.000 1 2 34-9_0843;KKA03730.1;

1411 1435 60 1.000 1 2 34-9_0844;model.g3233.t1;

1412 1436 97 1.000 1 2 34-9_0845;KKA03731.1;

1413 1437 69 1.000 1 3 KKA03732.1;model.g1819.t1;34-9_0846;

1414 1438 98 1.000 1 2 34-9_0847;KKA03733.1;

1415 1439 20 1.000 1 3 KKA03734.1;model.g1823.t1;34-9_0848;

1416 1440 24 1.000 1 3 KKA03735.1;model.g1822.t1;34-9_0849;

1417 1441 99 1.000 1 2 34-9_0850;KKA03736.1;

1418 1442 42 1.000 1 3 KKA03737.1;model.g1806.t1;34-9_0851;

1419 1443 39 1.000 1 2 34-9_0853;model.g1866.t1;

1420 1444 51 1.000 1 3 KKA03739.1;model.g1868.t1;34-9_0854;

1421 1445 75 1.000 1 2 34-9_0855;model.g1869.t1;

1422 1445 57 1.000 1 3 KKA02653.1;34-9_3459;model.g3539.t1;

1423 1447 75 1.000 1 2 34-9_0856;model.g1870.t1;

1424 1448 68 1.000 1 2 34-9_3731;model.g900.t1;

1425 1449 13 1.000 1 3 KKA03741.1;model.g1872.t1;34-9_0858;

1426 1450 21 1.000 1 2 34-9_0859;model.g1873.t1;

1427 1451 22 1.000 1 4 KKA03742.1;model.g1875.t1;model.g1874.t1;34-9_0860;

1428 1452 100 1.000 1 2 34-9_0861;KKA03743.1;

1429 1453 24 1.000 1 3 KKA03744.1;model.g1876.t1;34-9_0862;

1430 1454 36 1.000 1 3 KKA01962.1;model.g339.t1;34-9_1338;

1431 1455 13 1.000 1 4 KKA03081.1;model.g3152.t1;model.g3154.t1;34-9_2529;

1432 1456 60 1.000 1 3 KKA03746.1;model.g2400.t1;34-9_0864;

1433 1457 100 1.000 1 2 34-9_0866;KKA03748.1;

1434 1458 99 1.000 1 2 34-9_0867;KKA03749.1;

1435 1459 39 1.000 1 3 KKA03750.1;model.g40.t1;34-9_0868;

1436 1460 29 1.000 1 3 KKA03751.1;model.g2404.t1;34-9_0869;

1437 1461 25 1.000 1 3 KKA03752.1;model.g2406.t1;34-9_0870;

1438 1462 30 1.000 1 3 KKA03754.1;model.g2415.t1;34-9_0872;

1439 1463 98 1.000 1 2 34-9_0873;KKA03755.1;

1440 1464 99 1.000 1 2 34-9_0875;KKA03757.1;

1441 1465 100 1.000 1 2 34-9_0876;KKA03758.1;

1442 1466 100 1.000 1 2 34-9_0877;KKA03759.1;

1443 1467 18 1.000 1 3 KKA03760.1;model.g1382.t1;34-9_0878;

1444 1468 97 1.000 1 2 34-9_0879;KKA03761.1;

1445 1469 64 1.000 1 2 34-9_0882;KKA03764.1;

1446 1470 13 1.000 1 3 KKA03765.1;model.g2460.t1;34-9_0883;

1447 1471 98 1.000 1 2 34-9_0884;KKA03766.1;

1448 1472 98 1.000 1 2 34-9_0886;KKA03768.1;

1449 1473 42 1.000 1 3 KKA03769.1;model.g2455.t1;34-9_0887;

1450 1474 53 1.000 1 4 KKA03771.1;model.g2015.t1;model.g3511.t1;34-9_0889;

1451 1475 62 1.000 1 2 34-9_0890;model.g2014.t1;

1452 1476 21 1.000 1 3 KKA03773.1;model.g2012.t1;34-9_0892;

1453 1477 18 1.000 1 2 34-9_0893;model.g1951.t1;

1454 1478 99 1.000 1 2 34-9_0894;KKA03774.1;

1455 1479 32 1.000 1 3 KKA03775.1;model.g1956.t1;34-9_0895;

1456 1480 22 1.000 1 3 KKA03776.1;model.g1957.t1;34-9_0896;

1457 1481 69 1.000 1 3 KKA03777.1;model.g1958.t1;34-9_0897;

1458 1482 49 1.000 1 3 KKA03778.1;model.g1959.t1;34-9_0898;

1459 1483 47 1.000 1 3 KKA03779.1;model.g1960.t1;34-9_0899;

1460 1484 24 1.000 1 2 34-9_0900;model.g1961.t1;

1461 1485 53 1.000 1 2 34-9_0902;KKA03781.1;

1462 1486 97 1.000 1 2 34-9_0903;KKA03782.1;

1463 1487 41 1.000 1 3 KKA03783.1;model.g1964.t1;34-9_0904;

1464 1488 37 1.000 1 3 KKA03784.1;model.g1965.t1;34-9_0905;

1465 1489 98 1.000 1 2 34-9_0906;KKA03785.1;

1466 1490 17 1.000 1 3 KKA03786.1;model.g1967.t1;34-9_0907;

1467 1491 6 0.498 1 9 34-9_0909;34-9_2614;KKA01194.1;model.g3493.t1;KKA03947.1;model.g1969.t1;KKA03787.1;34-9_1079;model.g2048.t1;

1468 1492 98 1.000 1 2 34-9_0910;KKA03788.1;

1469 1493 83 1.000 1 3 KKA03789.1;model.g1971.t1;34-9_0911;

1470 1494 19 1.000 1 3 KKA03790.1;model.g1973.t1;34-9_0912;

1471 1495 62 1.000 1 3 KKA03791.1;model.g1974.t1;34-9_0913;

1472 1496 99 1.000 1 2 34-9_0914;KKA03792.1;

1473 1497 98 1.000 1 2 34-9_0918;KKA03798.1;

1474 1498 100 1.000 1 2 34-9_0919;KKA03799.1;

1475 1499 17 1.000 1 3 KKA03801.1;model.g2110.t1;34-9_0922;

1476 1500 47 1.000 1 2 model.g881.t1;34-9_3718;

1477 1501 17 1.000 1 3 KKA03803.1;model.g2112.t1;34-9_0924;

1478 1502 12 1.000 1 3 KKA03804.1;model.g2113.t1;34-9_0925;

1479 1504 20 1.000 1 2 34-9_0926;model.g2114.t1;

1480 1505 17 1.000 1 3 KKA03805.1;model.g2115.t1;34-9_0927;

1481 1506 34 1.000 1 6 34-9_2137;KKA03806.1;KKA03545.1;model.g2116.t1;model.g739.t1;34-9_0928;

1482 1507 14 1.000 1 3 KKA03807.1;model.g2117.t1;34-9_0929;

1483 1508 36 1.000 1 3 KKA03808.1;model.g2118.t1;34-9_0930;

1484 1509 37 1.000 1 3 KKA03810.1;model.g2120.t1;34-9_0932;

1485 1510 23 1.000 1 3 KKA03812.1;model.g2122.t1;34-9_0934;

1486 1511 96 1.000 1 2 34-9_0935;KKA03813.1;

1487 1512 58 1.000 1 3 KKA03814.1;model.g2125.t1;34-9_0936;

1488 1513 12 1.000 1 3 KKA03815.1;model.g2126.t1;34-9_0937;

1489 1514 99 1.000 1 2 34-9_0938;KKA03816.1;

1490 1515 96 1.000 1 2 34-9_0939;KKA03817.1;

1491 1516 56 1.000 1 3 KKA03819.1;model.g2132.t1;34-9_0941;

1492 1517 35 1.000 1 3 KKA03820.1;model.g2133.t1;34-9_0942;

1493 1518 13 1.000 1 3 KKA03821.1;model.g2134.t1;34-9_0943;

1494 1519 50 1.000 1 3 KKA03822.1;model.g2135.t1;34-9_0944;

1495 1520 12 1.000 1 3 KKA03823.1;model.g2136.t1;34-9_0945;

1496 1521 30 1.000 1 3 KKA03824.1;model.g2137.t1;34-9_0946;

1497 1522 39 1.000 1 3 KKA03825.1;model.g2138.t1;34-9_0947;

1498 1523 99 1.000 1 2 34-9_0948;KKA03826.1;

1499 1524 98 1.000 1 2 34-9_0949;KKA03827.1;

1500 1525 11 1.000 1 3 KKA03828.1;model.g2141.t1;34-9_0950;

1501 1526 12 1.000 1 3 KKA03829.1;model.g2142.t1;34-9_0951;

1502 1527 23 1.000 1 3 34-9_2134;model.g741.t1;KKA03548.1;

1503 1528 14 1.000 1 3 34-9_1243;model.g2028.t1;KKA01913.1;

1504 1529 39 1.000 1 13 model.g559.t1;model.g558.t1;model.g557.t1;34-9_0953;model.g1914.t1;model.g560.t1;model.g562.t1;KKA03831.1;KKA03830.1;model.g563.t1;model.g561.t1;34-9_0952;model.g564.t1;

1505 1530 75 1.000 1 3 34-9_2617;model.g3490.t1;KKA01191.1;

1506 1531 15 1.000 1 5 KKA03832.1;model.g1699.t1;model.g2143.t1;34-9_1414;34-9_0954;

1507 1532 99 1.000 1 2 34-9_0955;KKA03833.1;

1508 1533 30 1.000 1 3 KKA03835.1;model.g2146.t1;34-9_0957;

1509 1534 5 0.502 1 3 KKA03837.1;model.g2148.t1;34-9_0959;

1510 1535 31 1.000 1 2 model.g2686.t1;34-9_1473;

1511 1536 99 1.000 1 2 34-9_0961;KKA03838.1;

1512 1537 63 1.000 1 3 KKA03839.1;model.g2106.t1;34-9_0962;

1513 1538 95 1.000 1 2 34-9_0963;KKA03840.1;

1514 1539 29 1.000 1 3 KKA03841.1;model.g2103.t1;34-9_0964;

1515 1540 32 1.000 1 3 KKA03842.1;model.g2102.t1;34-9_0965;

1516 1541 57 1.000 1 3 KKA03843.1;model.g2101.t1;34-9_0966;

1517 1542 19 1.000 1 3 KKA03844.1;model.g2099.t1;34-9_0967;

1518 1543 22 1.000 1 6 KKA02587.1;KKA03845.1;34-9_2044;model.g3111.t1;model.g1561.t1;34-9_0968;

1519 1544 98 1.000 1 2 34-9_0970;KKA03847.1;

1520 1545 38 1.000 1 3 KKA03848.1;model.g2095.t1;34-9_0971;

1521 1546 22 1.000 1 3 KKA03849.1;model.g2094.t1;34-9_0972;

1522 1547 14 1.000 1 2 34-9_0976;model.g1041.t1;

1523 1548 93 1.000 1 2 34-9_0977;KKA03852.1;

1524 1549 19 1.000 1 2 34-9_0978;model.g2172.t1;

1525 1550 39 1.000 1 2 34-9_0979;model.g2173.t1;

1526 1551 49 1.000 1 3 KKA03853.1;model.g2175.t1;34-9_0980;

1527 1552 19 1.000 1 3 KKA03854.1;model.g2176.t1;34-9_0981;

1528 1553 36 1.000 1 3 KKA03855.1;model.g2177.t1;34-9_0982;

1529 1554 76 1.000 1 3 KKA03857.1;model.g2183.t1;34-9_0984;

1530 1555 12 1.000 1 6 model.g2199.t1;KKA03858.1;KKA03869.1;model.g2184.t1;34-9_0997;34-9_0985;

1531 1556 46 1.000 1 2 34-9_0986;KKA03859.1;

1532 1557 79 1.000 1 2 34-9_0988;KKA03860.1;

1533 1558 28 1.000 1 3 KKA03861.1;model.g2190.t1;34-9_0989;

1534 1559 78 1.000 1 3 KKA03862.1;model.g2191.t1;34-9_0990;

1535 1560 24 1.000 1 3 KKA03863.1;model.g2192.t1;34-9_0991;

1536 1560 31 1.000 1 2 model.g2279.t1;KKA01864.1;

1537 1560 35 1.000 1 3 KKA01865.1;34-9_1895;model.g2280.t1;

1538 1560 15 1.000 1 3 model.g1695.t1;34-9_1410;KKA01672.1;

1539 1564 94 1.000 1 2 34-9_0993;KKA03865.1;

1540 1565 11 1.000 1 3 KKA03866.1;model.g2195.t1;34-9_0994;

1541 1566 24 1.000 1 3 KKA03867.1;model.g2196.t1;34-9_0995;

1542 1567 34 1.000 1 3 KKA03868.1;model.g2197.t1;34-9_0996;

1543 1568 20 0.502 1 3 KKA03871.1;KKA03872.1;34-9_0999;

1544 1570 20 1.000 1 3 KKA03873.1;model.g2202.t1;34-9_1000;

1545 1571 40 1.000 1 2 34-9_1001;KKA03874.1;

1546 1572 45 1.000 1 3 KKA03877.1;model.g1032.t1;34-9_1003;

1547 1573 44 1.000 1 3 KKA03878.1;model.g1031.t1;34-9_1004;

1548 1574 15 1.000 1 3 KKA03879.1;model.g1030.t1;34-9_1005;

1549 1575 7 0.557 1 6 KKA03923.1;KKA03880.1;34-9_1054;model.g1029.t1;model.g2156.t1;34-9_1006;

1550 1576 17 1.000 1 3 KKA03881.1;model.g2214.t1;34-9_1007;

1551 1577 98 1.000 1 2 34-9_1019;KKA03893.1;

1552 1578 99 1.000 1 2 34-9_1020;KKA03894.1;

1553 1579 65 1.000 1 2 34-9_1027;KKA03900.1;

1554 1580 85 1.000 1 3 KKA03902.1;model.g2213.t1;34-9_1029;

1555 1581 96 1.000 1 2 34-9_1030;KKA03903.1;

1556 1582 98 1.000 1 2 34-9_1031;KKA03904.1;

1557 1583 75 1.000 1 2 34-9_1032;model.g2211.t1;

1558 1584 31 1.000 1 3 KKA03905.1;model.g2210.t1;34-9_1033;

1559 1585 18 1.000 1 3 KKA03906.1;model.g2209.t1;34-9_1034;

1560 1586 21 1.000 1 3 KKA03907.1;model.g2208.t1;34-9_1035;

1561 1587 77 1.000 1 3 KKA03908.1;model.g1040.t1;34-9_1036;

1562 1588 86 1.000 1 2 34-9_1037;KKA03909.1;

1563 1589 57 1.000 1 2 34-9_1038;model.g1036.t1;

1564 1590 71 1.000 1 3 KKA03910.1;model.g1035.t1;34-9_1039;

1565 1591 46 1.000 1 3 KKA03911.1;model.g2205.t1;34-9_1040;

1566 1592 17 0.502 1 3 KKA03912.1;KKA03913.1;34-9_1041;

1567 1593 27 1.000 1 3 KKA03914.1;model.g2207.t1;34-9_1042;

1568 1593 16 1.000 1 3 34-9_1328;model.g326.t1;KKA01971.1;

1569 1595 9 0.667 1 4 KKA03915.1;model.g2170.t1;34-9_1044;34-9_1043;

1570 1596 18 1.000 1 2 34-9_1045;model.g2168.t1;

1571 1597 96 1.000 1 2 34-9_1046;KKA03916.1;

1572 1598 67 1.000 1 2 34-9_1047;model.g2166.t1;

1573 1599 100 1.000 1 2 34-9_1048;KKA03917.1;

1574 1600 75 1.000 1 3 KKA03919.1;model.g2160.t1;34-9_1050;

1575 1601 100 1.000 1 2 34-9_1051;KKA03920.1;

1576 1602 100 1.000 1 2 34-9_1052;KKA03921.1;

1577 1603 23 1.000 1 3 KKA03922.1;model.g2157.t1;34-9_1053;

1578 1604 98 1.000 1 2 34-9_1055;KKA03924.1;

1579 1605 11 1.000 1 3 KKA03925.1;model.g2153.t1;34-9_1056;

1580 1606 43 1.000 1 3 KKA03926.1;model.g2152.t1;34-9_1057;

1581 1607 99 1.000 1 2 34-9_1058;KKA03927.1;

1582 1608 31 1.000 1 3 KKA03930.1;model.g2008.t1;34-9_1061;

1583 1609 12 1.000 1 3 KKA03931.1;model.g2036.t1;34-9_1062;

1584 1610 99 1.000 1 2 34-9_1065;KKA03934.1;

1585 1611 14 1.000 1 4 KKA03935.1;KKA03936.1;34-9_1067;34-9_1066;

1586 1612 94 1.000 1 2 KKA03261.1;34-9_2792;

1587 1613 49 1.000 1 3 KKA03937.1;model.g2037.t1;34-9_1069;

1588 1614 19 1.000 1 3 KKA03938.1;model.g2038.t1;34-9_1070;

1589 1615 100 1.000 1 2 34-9_1071;KKA03939.1;

1590 1616 75 1.000 1 3 KKA03940.1;model.g2040.t1;34-9_1072;

1591 1617 37 1.000 1 3 KKA03942.1;model.g2042.t1;34-9_1074;

1592 1618 99 1.000 1 2 34-9_1075;KKA03943.1;

1593 1619 100 1.000 1 2 34-9_1077;KKA03945.1;

1594 1620 58 1.000 1 3 KKA03946.1;model.g2049.t1;34-9_1078;

1595 1621 33 1.000 1 3 KKA03948.1;model.g2046.t1;34-9_1080;

1596 1622 22 1.000 1 3 KKA03949.1;model.g2045.t1;34-9_1081;

1597 1623 37 1.000 1 3 KKA03950.1;model.g2044.t1;34-9_1082;

1598 1624 15 1.000 1 8 34-9_2795;KKA03951.1;34-9_1286;model.g2054.t1;model.g388.t1;34-9_1083;KKA03263.1;model.g631.t1;

1599 1625 90 1.000 1 2 34-9_1084;KKA03952.1;

1600 1626 34 1.000 1 3 KKA03954.1;model.g2058.t1;34-9_1086;

1601 1627 35 1.000 1 3 KKA03955.1;model.g2060.t1;34-9_1087;

1602 1628 11 1.000 1 2 34-9_1088;model.g2061.t1;

1603 1629 99 1.000 1 2 34-9_1089;KKA03956.1;

1604 1630 72 1.000 1 3 KKA03957.1;model.g2066.t1;34-9_1090;

1605 1631 13 1.000 1 3 KKA03958.1;model.g2067.t1;34-9_1091;

1606 1632 24 1.000 1 3 KKA03959.1;model.g2068.t1;34-9_1092;

1607 1633 59 1.000 1 3 KKA03960.1;model.g2069.t1;34-9_1093;

1608 1634 15 1.000 1 3 KKA03961.1;model.g2070.t1;34-9_1094;

1609 1635 52 1.000 1 3 KKA03966.1;model.g2075.t1;34-9_1097;

1610 1636 22 1.000 1 3 KKA03967.1;model.g2076.t1;34-9_1098;

1611 1637 31 1.000 1 5 KKA03968.1;model.g586.t1;model.g2078.t1;KKA02544.1;34-9_1100;

1612 1638 14 1.000 1 2 34-9_1104;model.g2082.t1;

1613 1639 63 1.000 1 3 KKA03971.1;model.g2084.t1;34-9_1105;

1614 1640 99 1.000 1 2 34-9_1106;KKA03972.1;

1615 1641 99 1.000 1 2 34-9_1107;KKA03973.1;

1616 1642 33 1.000 1 3 KKA03974.1;model.g2089.t1;34-9_1108;

1617 1643 24 1.000 1 3 KKA03976.1;model.g2091.t1;34-9_1110;

1618 1644 19 1.000 1 3 KKA03977.1;model.g1043.t1;34-9_1111;

1619 1645 12 1.000 1 3 KKA03978.1;model.g1044.t1;34-9_1112;

1620 1646 48 1.000 1 3 KKA03980.1;model.g1046.t1;34-9_1114;

1621 1647 63 1.000 1 3 KKA03981.1;model.g1047.t1;34-9_1115;

1622 1648 18 0.502 1 3 KKA03984.1;KKA03983.1;34-9_1117;

1623 1650 75 1.000 1 3 KKA03985.1;model.g1051.t1;34-9_1118;

1624 1651 100 1.000 1 2 34-9_1121;KKA03988.1;

1625 1652 56 1.000 1 3 KKA03989.1;model.g1054.t1;34-9_1122;

1626 1653 99 1.000 1 2 34-9_1138;KKA01226.1;

1627 1654 98 1.000 1 2 34-9_1144;KKA01232.1;

1628 1655 82 1.000 1 2 34-9_1151;KKA01238.1;

1629 1656 13 1.000 1 5 model.g2227.t1;34-9_1307;model.g369.t1;34-9_1157;KKA01263.1;

1630 1657 35 1.000 1 3 KKA01800.1;model.g2225.t1;34-9_1158;

1631 1658 26 1.000 1 3 KKA01799.1;34-9_1184;34-9_1159;

1632 1659 100 1.000 1 2 34-9_1163;KKA01797.1;

1633 1660 31 1.000 1 3 KKA01795.1;model.g634.t1;34-9_1165;

1634 1661 37 1.000 1 5 KKA01792.1;model.g638.t1;model.g715.t1;34-9_1174;model.g3437.t1;

1635 1662 85 1.000 1 2 34-9_1175;model.g637.t1;

1636 1663 84 1.000 1 2 34-9_1176;KKA01791.1;

1637 1664 36 1.000 1 6 model.g1450.t1;KKA01790.1;KKA01824.1;model.g633.t1;34-9_3287;34-9_1177;

1638 1665 18 0.502 1 3 KKA01789.1;KKA01788.1;34-9_1178;

1639 1666 54 1.000 1 3 KKA01787.1;model.g3561.t1;34-9_1180;

1640 1667 42 1.000 1 3 KKA01786.1;model.g3559.t1;34-9_1181;

1641 1668 14 1.000 1 2 34-9_1182;model.g3558.t1;

1642 1669 29 1.000 1 6 KKA03321.1;KKA01785.1;34-9_2354;model.g3556.t1;model.g3374.t1;34-9_1183;

1643 1670 39 1.000 1 5 KKA01784.1;model.g617.t1;34-9_1186;KKA01783.1;34-9_1185;

1644 1671 98 1.000 1 2 34-9_1187;KKA01782.1;

1645 1672 52 1.000 1 2 34-9_1188;KKA01781.1;

1646 1673 29 1.000 1 3 KKA01777.1;model.g615.t1;34-9_1191;

1647 1674 44 1.000 1 3 KKA01774.1;model.g616.t1;34-9_1196;

1648 1675 99 1.000 1 2 34-9_1197;KKA01773.1;

1649 1676 18 0.502 1 3 KKA01770.1;KKA01771.1;34-9_1199;

1650 1677 94 1.000 1 2 34-9_1205;KKA01766.1;

1651 1678 99 1.000 1 2 34-9_1214;KKA01762.1;

1652 1679 83 1.000 1 2 34-9_1227;KKA01901.1;

1653 1680 22 1.000 1 2 34-9_1230;model.g1997.t1;

1654 1681 16 1.000 1 2 34-9_1231;model.g1998.t1;

1655 1682 35 1.000 1 2 model.g2889.t1;34-9_1668;

1656 1683 19 1.000 1 3 KKA01905.1;model.g2000.t1;34-9_1233;

1657 1684 21 1.000 1 2 34-9_1234;model.g2001.t1;

1658 1685 13 1.000 1 2 34-9_1235;model.g2003.t1;

1659 1686 20 1.000 1 3 KKA01906.1;model.g2004.t1;34-9_1236;

1660 1687 99 1.000 1 2 34-9_1238;KKA01908.1;

1661 1688 52 1.000 1 3 KKA01909.1;model.g2032.t1;34-9_1239;

1662 1689 14 1.000 1 3 KKA01910.1;model.g2031.t1;34-9_1240;

1663 1690 18 1.000 1 2 34-9_1241;model.g2030.t1;

1664 1691 72 1.000 1 3 KKA01911.1;model.g2029.t1;34-9_1242;

1665 1692 12 1.000 1 3 KKA01914.1;model.g2022.t1;34-9_1244;

1666 1693 20 1.000 1 2 34-9_1245;model.g2024.t1;

1667 1694 26 1.000 1 3 KKA01915.1;model.g2025.t1;34-9_1246;

1668 1695 4 0.388 1 9 34-9_1247;KKA02311.1;model.g228.t1;KKA02308.1;KKA02310.1;model.g2026.t1;KKA01916.1;KKA02309.1;model.g395.t1;

1669 1696 74 1.000 1 3 KKA01917.1;model.g2021.t1;34-9_1248;

1670 1697 38 1.000 1 2 34-9_1249;model.g2016.t1;

1671 1698 12 1.000 1 3 KKA01918.1;model.g2017.t1;34-9_1250;

1672 1699 82 1.000 1 3 KKA01919.1;model.g3118.t1;34-9_1251;

1673 1699 59 1.000 1 3 KKA02450.1;model.g1216.t1;34-9_1983;

1674 1701 38 1.000 1 2 34-9_1252;model.g3117.t1;

1675 1702 61 1.000 1 2 34-9_1253;model.g3116.t1;

1676 1703 64 1.000 1 3 KKA01920.1;model.g3115.t1;34-9_1254;

1677 1704 19 1.000 1 2 34-9_1255;model.g3114.t1;

1678 1705 11 1.000 1 3 KKA01921.1;model.g3113.t1;34-9_1256;

1679 1706 10 1.000 1 3 KKA01922.1;model.g3112.t1;34-9_1257;

1680 1707 13 1.000 1 3 KKA01923.1;model.g3123.t1;34-9_1258;

1681 1708 100 1.000 1 2 34-9_1259;KKA01924.1;

1682 1709 19 1.000 1 3 KKA01925.1;model.g3125.t1;34-9_1260;

1683 1710 20 1.000 1 3 KKA01926.1;model.g3131.t1;34-9_1262;

1684 1711 69 1.000 1 2 34-9_1264;model.g3129.t1;

1685 1712 98 1.000 1 2 34-9_1267;KKA01927.1;

1686 1713 34 1.000 1 2 34-9_1270;model.g3145.t1;

1687 1714 54 1.000 1 2 34-9_1271;model.g3143.t1;

1688 1715 31 1.000 1 3 KKA01931.1;model.g3142.t1;34-9_1272;

1689 1716 96 1.000 1 2 34-9_1274;KKA01932.1;

1690 1717 22 1.000 1 2 34-9_1275;model.g3139.t1;

1691 1718 20 1.000 1 3 KKA01933.1;model.g3138.t1;34-9_1276;

1692 1719 20 1.000 1 3 KKA01934.1;model.g3137.t1;34-9_1277;

1693 1720 87 1.000 1 3 KKA01935.1;model.g3136.t1;34-9_1279;

1694 1721 20 0.749 1 5 KKA02891.1;34-9_3157;model.g126.t1;34-9_1281;model.g3134.t1;

1695 1722 38 1.000 1 2 34-9_1282;model.g394.t1;

1696 1723 18 1.000 1 2 34-9_1283;model.g392.t1;

1697 1724 44 1.000 1 2 34-9_1284;KKA01936.1;

1698 1725 25 1.000 1 6 model.g3335.t1;KKA00995.1;model.g2830.t1;KKA01243.1;model.g387.t1;34-9_1287;

1699 1726 16 1.000 1 4 KKA00996.1;KKA01244.1;model.g386.t1;34-9_1288;

1700 1727 32 1.000 1 3 KKA01249.1;model.g384.t1;34-9_1292;

1701 1728 45 1.000 1 4 KKA01250.1;model.g383.t1;model.g382.t1;34-9_1293;

1702 1729 38 1.000 1 3 KKA01251.1;model.g381.t1;34-9_1294;

1703 1729 30 1.000 1 3 KKA01828.1;model.g1456.t1;34-9_3282;

1704 1731 78 1.000 1 2 34-9_1296;KKA01253.1;

1705 1732 100 1.000 1 2 34-9_1297;KKA01254.1;

1706 1733 15 1.000 1 3 KKA01255.1;model.g374.t1;34-9_1298;

1707 1734 27 1.000 1 3 KKA01256.1;model.g373.t1;34-9_1299;

1708 1735 18 1.000 1 3 KKA01257.1;model.g372.t1;34-9_1300;

1709 1736 14 1.000 1 3 KKA01258.1;model.g3110.t1;34-9_1301;

1710 1737 33 1.000 1 3 KKA01259.1;model.g3109.t1;34-9_1302;

1711 1738 11 0.776 1 6 KKA03078.1;KKA01260.1;34-9_2525;model.g3107.t1;model.g3200.t1;34-9_1303;

1712 1738 14 1.000 1 3 model.g3199.t1;34-9_2526;KKA03079.1;

1713 1740 30 1.000 1 3 KKA01261.1;model.g3106.t1;34-9_1304;

1714 1741 99 1.000 1 2 34-9_1306;KKA01262.1;

1715 1742 19 1.000 1 3 34-9_2877;KKA01271.1;model.g219.t1;

1716 1743 39 1.000 1 3 KKA01264.1;model.g3120.t1;34-9_1309;

1717 1744 14 1.000 1 6 model.g416.t1;KKA01991.1;model.g1896.t1;KKA01993.1;34-9_1310;KKA01992.1;

1718 1745 23 1.000 1 3 KKA01990.1;model.g1898.t1;34-9_1311;

1719 1746 29 1.000 1 3 KKA01989.1;model.g1894.t1;34-9_1312;

1720 1747 14 1.000 1 3 KKA01988.1;model.g1893.t1;34-9_1313;

1721 1748 23 1.000 1 3 KKA01987.1;model.g1892.t1;34-9_1314;

1722 1749 15 1.000 1 3 KKA01986.1;model.g1891.t1;34-9_1315;

1723 1750 69 1.000 1 2 34-9_1316;KKA01985.1;

1724 1751 98 1.000 1 2 34-9_1317;KKA01984.1;

1725 1752 42 1.000 1 3 KKA01983.1;model.g1889.t1;34-9_1318;

1726 1753 52 1.000 1 2 34-9_1319;model.g1888.t1;

1727 1754 68 1.000 1 3 KKA01981.1;model.g1886.t1;34-9_1321;

1728 1754 38 1.000 1 3 KKA01166.1;model.g1331.t1;34-9_2959;

1729 1756 25 1.000 1 3 KKA01980.1;model.g324.t1;34-9_1322;

1730 1757 26 1.000 1 3 KKA01977.1;model.g322.t1;34-9_1323;

1731 1758 34 1.000 1 3 KKA01975.1;model.g327.t1;34-9_1324;

1732 1759 48 1.000 1 3 KKA01974.1;model.g329.t1;34-9_1325;

1733 1760 23 1.000 1 3 KKA01972.1;model.g1884.t1;34-9_1327;

1734 1761 32 1.000 1 3 KKA01970.1;model.g332.t1;34-9_1329;

1735 1762 27 1.000 1 3 KKA01969.1;model.g331.t1;34-9_1330;

1736 1763 33 1.000 1 3 KKA01968.1;model.g333.t1;34-9_1331;

1737 1764 93 1.000 1 2 34-9_1333;KKA01966.1;

1738 1765 73 1.000 1 2 34-9_1334;model.g336.t1;

1739 1766 81 1.000 1 3 34-9_1537;model.g401.t1;KKA02786.1;

1740 1767 33 1.000 1 3 KKA01965.1;model.g337.t1;34-9_1335;

1741 1768 22 1.000 1 3 KKA01964.1;model.g342.t1;34-9_1336;

1742 1769 37 1.000 1 3 KKA01963.1;model.g341.t1;34-9_1337;

1743 1770 41 1.000 1 3 KKA01961.1;model.g338.t1;34-9_1339;

1744 1771 67 1.000 1 2 34-9_1341;KKA01959.1;

1745 1772 33 1.000 1 3 KKA01958.1;model.g346.t1;34-9_1342;

1746 1773 33 1.000 1 3 KKA01957.1;model.g347.t1;34-9_1343;

1747 1774 37 1.000 1 3 KKA01956.1;model.g348.t1;34-9_1344;

1748 1775 25 1.000 1 3 KKA01954.1;model.g2605.t1;34-9_1346;

1749 1776 61 1.000 1 3 KKA01953.1;model.g2604.t1;34-9_1347;

1750 1777 92 1.000 1 2 34-9_1348;KKA01952.1;

1751 1778 17 1.000 1 3 KKA01951.1;model.g1540.t1;34-9_1349;

1752 1779 13 1.000 1 3 KKA01950.1;model.g2602.t1;34-9_1350;

1753 1780 22 1.000 1 3 KKA01949.1;model.g2601.t1;34-9_1351;

1754 1781 53 1.000 1 3 KKA01947.1;model.g352.t1;34-9_1353;

1755 1782 98 1.000 1 2 34-9_1354;KKA01946.1;

1756 1783 98 1.000 1 2 34-9_1358;KKA01944.1;

1757 1784 17 1.000 1 3 KKA01943.1;model.g1715.t1;34-9_1359;

1758 1785 76 1.000 1 3 KKA01942.1;model.g2614.t1;34-9_1360;

1759 1786 19 1.000 1 3 KKA01941.1;model.g2615.t1;34-9_1361;

1760 1787 71 1.000 1 2 34-9_1362;KKA01940.1;

1761 1788 41 1.000 1 3 KKA01939.1;model.g2617.t1;34-9_1363;

1762 1789 79 1.000 1 3 KKA01938.1;model.g2618.t1;34-9_1364;

1763 1790 38 1.000 1 3 KKA01024.1;model.g2623.t1;34-9_1366;

1764 1791 65 1.000 1 3 KKA01023.1;model.g2641.t1;34-9_1367;

1765 1792 27 1.000 1 5 KKA01022.1;KKA01669.1;model.g2793.t1;34-9_1368;34-9_1404;

1766 1793 28 1.000 1 3 KKA01021.1;model.g2640.t1;34-9_1369;

1767 1794 18 1.000 1 3 KKA01020.1;model.g2632.t1;34-9_1370;

1768 1796 15 1.000 1 3 KKA01652.1;model.g2633.t1;34-9_1372;

1769 1797 62 1.000 1 2 34-9_1373;model.g2634.t1;

1770 1798 22 1.000 1 2 34-9_1374;model.g2636.t1;

1771 1799 27 1.000 1 3 KKA01653.1;model.g2629.t1;34-9_1375;

1772 1800 73 1.000 1 2 34-9_1376;model.g2628.t1;

1773 1801 13 1.000 1 2 34-9_1377;model.g2627.t1;

1774 1802 15 1.000 1 2 34-9_1379;model.g2432.t1;

1775 1803 33 1.000 1 3 KKA01654.1;model.g2625.t1;34-9_1380;

1776 1804 19 1.000 1 2 34-9_1381;model.g2624.t1;

1777 1805 56 1.000 1 3 KKA01656.1;model.g1645.t1;34-9_1382;

1778 1806 98 1.000 1 2 34-9_1385;KKA01659.1;

1779 1807 19 1.000 1 2 34-9_1386;model.g1653.t1;

1780 1808 23 1.000 1 2 34-9_1389;model.g1655.t1;

1781 1809 20 1.000 1 2 34-9_1390;model.g1672.t1;

1782 1810 22 1.000 1 2 34-9_1392;model.g1670.t1;

1783 1811 11 1.000 1 2 34-9_1395;model.g1677.t1;

1784 1812 29 1.000 1 2 34-9_1396;model.g1679.t1;

1785 1813 26 1.000 1 3 KKA01664.1;model.g1680.t1;34-9_1397;

1786 1814 15 1.000 1 2 34-9_1398;model.g1681.t1;

1787 1815 31 1.000 1 2 34-9_1399;model.g1685.t1;

1788 1816 66 1.000 1 2 34-9_1400;KKA01665.1;

1789 1817 47 1.000 1 2 34-9_1401;model.g1682.t1;

1790 1818 32 1.000 1 3 KKA01666.1;model.g1687.t1;34-9_1402;

1791 1819 34 1.000 1 3 KKA01668.1;model.g1689.t1;34-9_1403;

1792 1820 58 1.000 1 3 KKA01670.1;model.g1690.t1;34-9_1405;

1793 1821 18 1.000 1 2 34-9_1406;model.g1691.t1;

1794 1822 18 1.000 1 3 KKA01671.1;model.g1692.t1;34-9_1407;

1795 1823 14 1.000 1 2 34-9_1411;model.g1696.t1;

1796 1824 7 0.502 1 3 KKA01673.1;model.g1697.t1;34-9_1412;

1797 1825 19 1.000 1 3 KKA01676.1;model.g1701.t1;34-9_1415;

1798 1826 76 1.000 1 3 KKA01677.1;model.g1703.t1;34-9_1416;

1799 1827 23 1.000 1 3 KKA01678.1;model.g1705.t1;34-9_1417;

1800 1828 27 1.000 1 2 34-9_1418;model.g1707.t1;

1801 1829 39 1.000 1 2 34-9_1419;KKA01679.1;

1802 1830 85 1.000 1 2 34-9_1421;KKA01681.1;

1803 1831 44 1.000 1 6 KKA02610.1;KKA01683.1;34-9_2083;model.g2656.t1;model.g829.t1;34-9_1423;

1804 1832 99 1.000 1 2 34-9_1424;KKA01684.1;

1805 1833 58 1.000 1 2 34-9_1425;model.g2654.t1;

1806 1834 98 1.000 1 2 34-9_1426;KKA01032.1;

1807 1835 46 1.000 1 3 KKA01033.1;model.g2650.t1;34-9_1427;

1808 1836 33 1.000 1 3 KKA01034.1;model.g2649.t1;34-9_1428;

1809 1837 34 1.000 1 2 34-9_1429;model.g2648.t1;

1810 1838 14 1.000 1 3 KKA01035.1;model.g2647.t1;34-9_1430;

1811 1839 47 1.000 1 2 34-9_1431;KKA01036.1;

1812 1840 99 1.000 1 2 34-9_1432;KKA01037.1;

1813 1841 18 1.000 1 3 KKA01069.1;model.g2645.t1;34-9_1433;

1814 1842 38 1.000 1 3 KKA01068.1;model.g2646.t1;34-9_1434;

1815 1843 99 1.000 1 2 34-9_1435;KKA03160.1;

1816 1844 99 1.000 1 2 34-9_1444;KKA03166.1;

1817 1845 26 1.000 1 2 34-9_1447;model.g2657.t1;

1818 1846 67 1.000 1 2 34-9_1448;model.g2660.t1;

1819 1847 8 0.502 1 3 KKA03168.1;model.g2659.t1;34-9_1449;

1820 1848 17 1.000 1 2 34-9_1453;model.g2661.t1;

1821 1849 18 1.000 1 3 34-9_2090;KKA02614.1;model.g1585.t1;

1822 1850 98 1.000 1 2 34-9_1455;KKA03171.1;

1823 1851 35 1.000 1 2 34-9_1456;model.g2664.t1;

1824 1852 40 1.000 1 3 KKA03172.1;model.g2666.t1;34-9_1458;

1825 1853 20 1.000 1 3 KKA03174.1;model.g2669.t1;34-9_1460;

1826 1854 12 1.000 1 2 34-9_1461;model.g2670.t1;

1827 1855 19 0.502 1 4 KKA03175.1;KKA03176.1;model.g2672.t1;34-9_1462;

1828 1855 16 1.000 1 4 34-9_1569;model.g2671.t1;KKA02817.1;model.g447.t1;

1829 1857 23 1.000 1 2 34-9_1463;model.g2673.t1;

1830 1858 99 1.000 1 2 34-9_1464;KKA03177.1;

1831 1859 7 0.502 1 3 KKA03178.1;model.g2676.t1;34-9_1465;

1832 1860 10 1.000 1 3 KKA03179.1;model.g2687.t1;34-9_1467;

1833 1861 57 1.000 1 3 KKA03180.1;model.g2688.t1;34-9_1468;

1834 1862 57 1.000 1 3 KKA03181.1;model.g2690.t1;34-9_1469;

1835 1863 33 1.000 1 2 model.g308.t1;34-9_2587;

1836 1864 15 1.000 1 8 model.g427.t1;model.g2691.t1;model.g93.t1;34-9_3129;34-9_3485;34-9_1470;KKA02904.1;KKA02322.1;

1837 1865 99 1.000 1 2 34-9_1471;KKA03182.1;

1838 1866 99 1.000 1 2 34-9_1472;KKA03183.1;

1839 1867 78 1.000 1 2 34-9_1475;KKA03185.1;

1840 1868 17 1.000 1 3 KKA03186.1;model.g2684.t1;34-9_1476;

1841 1869 11 1.000 1 2 34-9_1477;model.g2683.t1;

1842 1870 22 1.000 1 3 KKA03187.1;model.g2682.t1;34-9_1478;

1843 1871 21 1.000 1 2 34-9_1479;model.g2696.t1;

1844 1872 69 1.000 1 2 34-9_1480;model.g2680.t1;

1845 1873 100 1.000 1 2 34-9_1482;KKA03189.1;

1846 1874 30 1.000 1 2 34-9_1485;model.g867.t1;

1847 1875 56 1.000 1 2 34-9_1486;model.g868.t1;

1848 1876 47 1.000 1 5 model.g869.t1;34-9_2291;model.g1900.t1;KKA03383.1;34-9_1487;

1849 1877 20 1.000 1 3 KKA03194.1;model.g873.t1;34-9_1488;

1850 1878 44 1.000 1 2 34-9_1489;model.g874.t1;

1851 1879 61 1.000 1 2 34-9_1490;KKA03197.1;

1852 1880 44 1.000 1 2 34-9_1493;model.g862.t1;

1853 1881 62 1.000 1 2 34-9_1494;KKA03198.1;

1854 1882 77 1.000 1 2 34-9_1498;KKA03201.1;

1855 1883 56 1.000 1 2 34-9_1506;model.g2493.t1;

1856 1884 36 1.000 1 3 KKA03207.1;model.g2494.t1;34-9_1508;

1857 1885 44 1.000 1 2 34-9_1513;KKA03210.1;

1858 1886 97 1.000 1 2 34-9_1514;KKA03211.1;

1859 1887 14 1.000 1 2 34-9_1515;model.g2501.t1;

1860 1888 68 1.000 1 2 34-9_1517;KKA03212.1;

1861 1889 26 1.000 1 3 KKA03213.1;model.g2505.t1;34-9_1518;

1862 1890 47 1.000 1 3 KKA03215.1;model.g2508.t1;34-9_1520;

1863 1891 26 1.000 1 2 34-9_1521;model.g2511.t1;

1864 1892 83 1.000 1 2 34-9_1522;KKA03216.1;

1865 1893 23 1.000 1 4 KKA03217.1;model.g1303.t1;model.g1304.t1;34-9_1523;

1866 1894 17 1.000 1 3 KKA03218.1;model.g1302.t1;34-9_1524;

1867 1895 24 1.000 1 3 KKA03219.1;model.g1299.t1;34-9_1527;

1868 1896 89 1.000 1 2 34-9_1528;KKA03220.1;

1869 1897 38 1.000 1 2 34-9_1529;model.g1296.t1;

1870 1898 17 1.000 1 2 34-9_1534;model.g404.t1;

1871 1899 18 1.000 1 2 34-9_1535;model.g403.t1;

1872 1900 36 1.000 1 3 34-9_2594;KKA01211.1;model.g3339.t1;

1873 1901 52 1.000 1 3 KKA02363.1;model.g2441.t1;34-9_3523;

1874 1902 22 1.000 1 3 KKA02787.1;model.g400.t1;34-9_1538;

1875 1903 18 1.000 1 2 model.g3424.t1;34-9_2427;

1876 1904 44 1.000 1 3 KKA02788.1;model.g425.t1;34-9_1539;

1877 1905 79 1.000 1 3 KKA02789.1;model.g2484.t1;34-9_1540;

1878 1906 60 1.000 1 2 34-9_1541;KKA02790.1;

1879 1907 24 1.000 1 2 34-9_1542;model.g2489.t1;

1880 1908 21 1.000 1 3 KKA02794.1;model.g195.t1;34-9_1546;

1881 1909 15 1.000 1 3 KKA02796.1;model.g3407.t1;34-9_1547;

1882 1910 27 1.000 1 3 KKA02797.1;model.g193.t1;34-9_1548;

1883 1911 99 1.000 1 2 34-9_1549;KKA02798.1;

1884 1912 33 1.000 1 3 KKA02799.1;model.g191.t1;34-9_1550;

1885 1913 99 1.000 1 2 34-9_1553;KKA02803.1;

1886 1914 66 1.000 1 2 34-9_1566;KKA02814.1;

1887 1915 98 1.000 1 2 34-9_1567;KKA02816.1;

1888 1916 21 1.000 1 2 34-9_1568;model.g446.t1;

1889 1917 97 1.000 1 2 34-9_1572;KKA02820.1;

1890 1918 93 1.000 1 2 KKA02821.1;34-9_1573;

1891 1919 5 0.502 1 5 KKA02822.1;34-9_1709;model.g452.t1;model.g2927.t1;34-9_1574;

1892 1920 70 1.000 1 3 KKA02823.1;model.g448.t1;34-9_1575;

1893 1921 100 1.000 1 2 34-9_1576;KKA02824.1;

1894 1922 99 1.000 1 2 34-9_1579;KKA02826.1;

1895 1923 87 1.000 1 3 KKA02830.1;model.g441.t1;34-9_1582;

1896 1924 16 1.000 1 8 KKA01454.1;KKA02831.1;34-9_3696;model.g440.t1;model.g2703.t1;34-9_1583;model.g2451.t1;34-9_3530;

1897 1925 19 1.000 1 3 KKA02832.1;model.g439.t1;34-9_1584;

1898 1926 74 1.000 1 3 KKA02833.1;model.g438.t1;34-9_1585;

1899 1927 12 0.835 1 5 KKA02834.1;34-9_3247;model.g437.t1;model.g2524.t1;34-9_1586;

1900 1928 66 1.000 1 2 34-9_1587;KKA02835.1;

1901 1929 90 1.000 1 2 34-9_1589;KKA02837.1;

1902 1930 98 1.000 1 2 34-9_1590;KKA02839.1;

1903 1931 28 1.000 1 3 KKA02840.1;model.g432.t1;34-9_1591;

1904 1933 98 1.000 1 2 34-9_1592;KKA02842.1;

1905 1934 25 1.000 1 3 KKA02848.1;model.g1574.t1;34-9_1597;

1906 1935 64 1.000 1 3 KKA02849.1;model.g2490.t1;34-9_1599;

1907 1936 12 1.000 1 3 KKA02850.1;model.g3515.t1;34-9_1600;

1908 1937 99 1.000 1 2 34-9_1602;KKA02852.1;

1909 1938 72 1.000 1 3 KKA02853.1;model.g3520.t1;34-9_1603;

1910 1939 20 1.000 1 3 KKA02855.1;model.g3518.t1;34-9_1605;

1911 1940 69 1.000 1 3 KKA02856.1;model.g3523.t1;34-9_1607;

1912 1941 20 1.000 1 2 model.g3243.t1;34-9_2501;

1913 1942 26 1.000 1 3 KKA02857.1;model.g3522.t1;34-9_1608;

1914 1943 24 1.000 1 2 34-9_1609;model.g3521.t1;

1915 1944 23 1.000 1 3 KKA02858.1;model.g3525.t1;34-9_1610;

1916 1945 36 1.000 1 2 34-9_1611;model.g3526.t1;

1917 1946 22 1.000 1 3 KKA02860.1;model.g3531.t1;34-9_1615;

1918 1947 76 1.000 1 2 34-9_1617;KKA02863.1;

1919 1948 87 1.000 1 3 KKA02864.1;model.g204.t1;34-9_1618;

1920 1949 18 1.000 1 3 KKA02866.1;model.g202.t1;34-9_1620;

1921 1950 20 1.000 1 3 KKA02869.1;model.g2592.t1;34-9_1622;

1922 1951 99 1.000 1 2 34-9_1624;KKA02871.1;

1923 1952 70 1.000 1 2 34-9_1626;KKA01104.1;

1924 1953 43 1.000 1 3 KKA01105.1;model.g2588.t1;34-9_1627;

1925 1954 82 1.000 1 3 KKA01106.1;model.g2581.t1;34-9_1628;

1926 1955 64 1.000 1 3 KKA01107.1;model.g2584.t1;34-9_1629;

1927 1956 38 1.000 1 3 KKA01109.1;model.g2586.t1;34-9_1630;

1928 1957 17 1.000 1 3 KKA01110.1;model.g2580.t1;34-9_1631;

1929 1958 27 1.000 1 3 KKA01113.1;model.g1662.t1;34-9_1633;

1930 1959 31 1.000 1 2 34-9_1636;34-9_2643;

1931 1960 99 1.000 1 2 34-9_1638;KKA01759.1;

1932 1961 37 1.000 1 3 KKA01756.1;model.g576.t1;34-9_1640;

1933 1962 59 1.000 1 2 34-9_1642;model.g574.t1;

1934 1963 9 0.667 1 4 KKA01754.1;KKA01753.1;model.g3460.t1;34-9_1643;

1935 1964 99 1.000 1 2 34-9_1644;KKA01752.1;

1936 1965 60 1.000 1 2 34-9_1645;KKA01751.1;

1937 1966 47 1.000 1 2 34-9_1646;model.g3465.t1;

1938 1967 65 1.000 1 2 34-9_1647;model.g3466.t1;

1939 1968 98 1.000 1 2 34-9_1648;KKA01750.1;

1940 1969 21 1.000 1 3 KKA01749.1;model.g515.t1;34-9_1649;

1941 1971 84 1.000 1 2 34-9_3674;KKA03148.1;

1942 1972 87 1.000 1 2 34-9_1652;model.g514.t1;

1943 1973 43 1.000 1 2 34-9_1653;model.g512.t1;

1944 1974 78 1.000 1 2 34-9_1654;KKA01744.1;

1945 1975 10 1.000 1 2 34-9_1655;model.g509.t1;

1946 1976 18 1.000 1 2 34-9_1658;model.g2911.t1;

1947 1977 24 1.000 1 2 34-9_1659;model.g2912.t1;

1948 1978 31 1.000 1 2 34-9_1660;model.g2914.t1;

1949 1979 22 1.000 1 3 model.g2915.t1;model.g2921.t1;34-9_1661;

1950 1980 31 1.000 1 3 KKA01742.1;model.g2917.t1;34-9_1662;

1951 1981 33 1.000 1 3 KKA01740.1;model.g2919.t1;34-9_1664;

1952 1982 100 1.000 1 2 34-9_1665;KKA01739.1;

1953 1983 20 1.000 1 2 34-9_1666;model.g2886.t1;

1954 1984 27 1.000 1 2 34-9_1667;model.g2887.t1;

1955 1985 29 1.000 1 3 KKA01738.1;model.g2890.t1;34-9_1669;

1956 1986 8 0.702 1 7 model.g892.t1;KKA01737.1;model.g2749.t1;KKA01266.1;model.g2891.t1;34-9_1670;34-9_3724;

1957 1988 40 1.000 1 2 34-9_1671;model.g2892.t1;

1958 1989 15 1.000 1 3 KKA01736.1;model.g2894.t1;34-9_1672;

1959 1990 24 1.000 1 2 34-9_1674;model.g2897.t1;

1960 1991 100 1.000 1 2 34-9_1675;KKA01735.1;

1961 1992 29 1.000 1 3 KKA01734.1;model.g2899.t1;34-9_1676;

1962 1993 73 1.000 1 3 KKA01733.1;model.g2900.t1;34-9_1677;

1963 1994 30 1.000 1 3 KKA01731.1;model.g2904.t1;34-9_1680;

1964 1995 49 1.000 1 2 34-9_1681;model.g2907.t1;

1965 1996 100 1.000 1 2 34-9_1682;KKA01730.1;

1966 1997 90 1.000 1 2 34-9_1683;KKA01729.1;

1967 1998 14 1.000 1 2 34-9_1684;model.g504.t1;

1968 1999 22 1.000 1 2 34-9_1685;model.g496.t1;

1969 2000 30 1.000 1 3 KKA01728.1;model.g498.t1;34-9_1686;

1970 2001 34 1.000 1 2 34-9_1688;model.g499.t1;

1971 2002 73 1.000 1 2 34-9_1689;model.g500.t1;

1972 2003 44 1.000 1 3 KKA01726.1;model.g501.t1;34-9_1690;

1973 2004 30 1.000 1 3 KKA01725.1;KKA01976.1;34-9_1692;

1974 2005 30 1.000 1 3 KKA02232.1;model.g2934.t1;34-9_1694;

1975 2006 21 1.000 1 2 34-9_1695;model.g2933.t1;

1976 2007 29 1.000 1 5 model.g502.t1;model.g2519.t1;KKA02517.1;34-9_1697;34-9_3251;

1977 2008 25 1.000 1 2 34-9_1698;model.g503.t1;

1978 2009 13 1.000 1 4 model.g2932.t1;model.g3205.t1;34-9_2488;34-9_1699;

1979 2010 13 1.000 1 2 34-9_1700;model.g2939.t1;

1980 2011 85 1.000 1 3 KKA02233.1;model.g18.t1;34-9_1701;

1981 2012 54 1.000 1 3 KKA02235.1;model.g2930.t1;34-9_1703;

1982 2013 13 1.000 1 3 KKA02236.1;model.g749.t1;34-9_1704;

1983 2014 98 1.000 1 2 34-9_1705;KKA02237.1;

1984 2015 30 1.000 1 3 KKA02239.1;model.g2920.t1;34-9_1708;

1985 2016 5 0.502 1 3 model.g2928.t1;model.g1100.t1;34-9_1710;

1986 2017 51 1.000 1 3 34-9_2054;KKA02593.1;model.g834.t1;

1987 2018 22 1.000 1 2 34-9_1711;model.g2929.t1;

1988 2019 19 1.000 1 2 34-9_1713;model.g2883.t1;

1989 2020 16 1.000 1 3 KKA02240.1;model.g2882.t1;34-9_1714;

1990 2021 21 1.000 1 2 34-9_1715;model.g2881.t1;

1991 2022 69 1.000 1 2 34-9_1716;model.g2877.t1;

1992 2023 18 1.000 1 2 34-9_1717;model.g2875.t1;

1993 2024 23 1.000 1 3 KKA02242.1;model.g2873.t1;34-9_1718;

1994 2025 21 1.000 1 7 KKA01348.1;KKA02243.1;34-9_3767;model.g2872.t1;model.g3311.t1;34-9_1719;model.g2871.t1;

1995 2026 15 1.000 1 2 34-9_1720;model.g2870.t1;

1996 2027 32 1.000 1 2 34-9_1721;model.g2868.t1;

1997 2028 91 1.000 1 2 34-9_1724;KKA02244.1;

1998 2029 97 1.000 1 2 34-9_1725;KKA02245.1;

1999 2030 17 1.000 1 2 34-9_1726;model.g2860.t1;

2000 2031 20 1.000 1 2 34-9_1727;model.g2859.t1;

2001 2032 22 1.000 1 2 34-9_1729;model.g2855.t1;

2002 2033 97 1.000 1 2 34-9_1730;KKA02248.1;

2003 2034 24 1.000 1 3 KKA02249.1;model.g2853.t1;34-9_1731;

2004 2035 25 1.000 1 2 34-9_1732;model.g2852.t1;

2005 2036 14 1.000 1 3 KKA02250.1;model.g2851.t1;34-9_1733;

2006 2037 99 1.000 1 2 34-9_1735;KKA02252.1;

2007 2038 53 1.000 1 2 34-9_1736;model.g2848.t1;

2008 2039 33 1.000 1 3 KKA02253.1;model.g2847.t1;34-9_1737;

2009 2040 31 1.000 1 3 KKA02254.1;model.g2846.t1;34-9_1738;

2010 2041 66 1.000 1 2 34-9_1740;KKA02256.1;

2011 2042 20 1.000 1 2 model.g44.t1;34-9_3096;

2012 2043 42 1.000 1 2 34-9_1741;KKA02257.1;

2013 2044 48 1.000 1 3 KKA02258.1;model.g2840.t1;34-9_1742;

2014 2045 16 1.000 1 3 34-9_2941;model.g1348.t1;model.g1349.t1;

2015 2046 98 1.000 1 2 34-9_1744;KKA02260.1;

2016 2047 27 1.000 1 3 KKA02261.1;model.g2838.t1;34-9_1745;

2017 2048 27 1.000 1 2 34-9_1746;model.g2837.t1;

2018 2049 99 1.000 1 2 34-9_1761;KKA02269.1;

2019 2050 76 1.000 1 2 34-9_1765;KKA02273.1;

2020 2051 67 1.000 1 2 34-9_1768;KKA01994.1;

2021 2052 22 1.000 1 3 KKA01995.1;model.g364.t1;34-9_1769;

2022 2053 93 1.000 1 2 34-9_1770;KKA01996.1;

2023 2054 76 1.000 1 2 34-9_1771;model.g361.t1;

2024 2055 35 1.000 1 3 KKA01999.1;model.g357.t1;34-9_1774;

2025 2056 23 1.000 1 3 KKA02000.1;model.g354.t1;34-9_1775;

2026 2057 99 1.000 1 2 34-9_1784;KKA02007.1;

2027 2058 98 1.000 1 2 34-9_1791;KKA02015.1;

2028 2059 93 1.000 1 2 34-9_1797;KKA02164.1;

2029 2060 67 1.000 1 2 34-9_1800;KKA02166.1;

2030 2061 99 1.000 1 2 34-9_1801;KKA02167.1;

2031 2062 21 0.502 1 3 KKA02172.1;KKA02173.1;34-9_1807;

2032 2063 99 1.000 1 2 34-9_1814;KKA02178.1;

2033 2064 74 1.000 1 2 34-9_1819;KKA02180.1;

2034 2065 58 1.000 1 2 34-9_1825;KKA02183.1;

2035 2066 90 1.000 1 2 34-9_1826;KKA02184.1;

2036 2067 15 0.502 1 3 KKA02185.1;KKA02186.1;34-9_1827;

2037 2068 18 1.000 1 3 KKA02188.1;model.g909.t1;34-9_1829;

2038 2069 99 1.000 1 2 34-9_1831;KKA02189.1;

2039 2070 26 1.000 1 3 KKA02190.1;model.g249.t1;34-9_1832;

2040 2071 13 1.000 1 2 34-9_1834;model.g240.t1;

2041 2072 99 1.000 1 2 34-9_1836;KKA02192.1;

2042 2073 38 1.000 1 3 KKA02193.1;model.g153.t1;34-9_1837;

2043 2074 98 1.000 1 2 34-9_1838;KKA02194.1;

2044 2075 12 1.000 1 2 34-9_1839;model.g152.t1;

2045 2076 21 1.000 1 3 KKA02196.1;model.g154.t1;34-9_1840;

2046 2077 87 1.000 1 2 34-9_1841;model.g155.t1;

2047 2078 93 1.000 1 2 34-9_1842;model.g156.t1;

2048 2079 59 1.000 1 3 KKA02197.1;model.g157.t1;34-9_1843;

2049 2080 98 1.000 1 2 34-9_1844;KKA02198.1;

2050 2081 22 1.000 1 3 KKA02199.1;model.g161.t1;34-9_1846;

2051 2082 94 1.000 1 2 34-9_1847;KKA02200.1;

2052 2083 13 1.000 1 2 34-9_1849;model.g168.t1;

2053 2084 58 1.000 1 2 34-9_1850;KKA02203.1;

2054 2085 33 1.000 1 7 34-9_3381;model.g913.t1;model.g665.t1;KKA02453.1;34-9_1980;34-9_1851;KKA02706.1;

2055 2086 21 1.000 1 2 34-9_1852;model.g914.t1;

2056 2087 33 1.000 1 2 34-9_1853;model.g915.t1;

2057 2088 65 1.000 1 2 34-9_1854;model.g916.t1;

2058 2089 49 1.000 1 3 KKA02204.1;model.g917.t1;34-9_1855;

2059 2090 25 1.000 1 3 KKA02205.1;model.g237.t1;34-9_1858;

2060 2091 23 1.000 1 3 KKA02206.1;model.g238.t1;34-9_1859;

2061 2092 22 1.000 1 4 model.g236.t1;model.g810.t1;34-9_3357;34-9_1864;

2062 2092 14 0.502 1 4 model.g2310.t1;KKA01869.1;34-9_1897;KKA01867.1;

2063 2094 32 1.000 1 4 model.g1571.t1;model.g1570.t1;model.g2405.t1;34-9_3103;

2064 2094 29 1.000 1 4 model.g3202.t1;model.g3203.t1;KKA03041.1;34-9_2486;

2065 2096 99 1.000 1 2 34-9_1866;KKA02209.1;

2066 2097 40 1.000 1 2 34-9_1872;model.g188.t1;

2067 2098 58 1.000 1 2 34-9_1874;model.g186.t1;

2068 2099 50 1.000 1 2 34-9_1876;KKA02213.1;

2069 2100 75 1.000 1 3 KKA02214.1;model.g182.t1;34-9_1877;

2070 2101 22 1.000 1 5 KKA02216.1;34-9_3615;model.g943.t1;model.g2559.t1;34-9_1879;

2071 2102 60 1.000 1 2 34-9_1880;KKA02217.1;

2072 2103 10 1.000 1 2 34-9_1881;model.g945.t1;

2073 2104 17 1.000 1 3 KKA01854.1;model.g2264.t1;34-9_1884;

2074 2105 23 1.000 1 3 KKA01856.1;model.g2267.t1;34-9_1886;

2075 2106 24 1.000 1 3 KKA01857.1;model.g2268.t1;34-9_1887;

2076 2107 32 1.000 1 2 34-9_1890;model.g2271.t1;

2077 2108 100 1.000 1 2 34-9_1891;KKA01860.1;

2078 2109 46 1.000 1 3 KKA01862.1;model.g2277.t1;34-9_1893;

2079 2110 100 1.000 1 2 34-9_1894;KKA01863.1;

2080 2111 45 1.000 1 3 KKA01871.1;model.g2308.t1;34-9_1899;

2081 2112 11 1.000 1 3 KKA01872.1;model.g2306.t1;34-9_1901;

2082 2113 49 1.000 1 2 34-9_1902;model.g2305.t1;

2083 2114 11 1.000 1 3 KKA01874.1;model.g2303.t1;34-9_1903;

2084 2115 99 1.000 1 2 34-9_1904;KKA01875.1;

2085 2116 26 1.000 1 3 KKA01876.1;model.g2293.t1;34-9_1906;

2086 2117 19 1.000 1 7 KKA01554.1;KKA01878.1;34-9_3612;model.g2298.t1;model.g2297.t1;34-9_1907;model.g2563.t1;

2087 2118 35 1.000 1 3 KKA01879.1;model.g2299.t1;34-9_1908;

2088 2119 8 0.502 1 11 KKA01617.1;34-9_1909;model.g271.t1;model.g164.t1;model.g163.t1;34-9_3236;34-9_3354;model.g2300.t1;KKA01880.1;34-9_3237;KKA02526.1;

2089 2120 27 1.000 1 3 KKA01881.1;model.g2291.t1;34-9_1910;

2090 2121 72 1.000 1 3 KKA01882.1;model.g2290.t1;34-9_1911;

2091 2122 100 1.000 1 3 KKA00970.1;KKA01884.1;34-9_1913;

2092 2123 94 1.000 1 3 KKA02491.1;model.g2284.t1;34-9_1915;

2093 2124 83 1.000 1 3 KKA02490.1;model.g2283.t1;34-9_1917;

2094 2125 14 1.000 1 3 KKA02489.1;model.g2314.t1;34-9_1918;

2095 2126 98 1.000 1 2 34-9_1919;KKA02488.1;

2096 2127 20 1.000 1 2 34-9_1920;model.g2312.t1;

2097 2128 18 1.000 1 2 34-9_1921;model.g2315.t1;

2098 2129 67 1.000 1 4 KKA02487.1;model.g2316.t1;model.g543.t1;34-9_1923;

2099 2130 71 1.000 1 2 34-9_1924;model.g2317.t1;

2100 2131 26 1.000 1 2 34-9_1925;model.g2318.t1;

2101 2132 10 1.000 1 2 34-9_1926;model.g2319.t1;

2102 2133 12 1.000 1 4 KKA02486.1;model.g2320.t1;model.g899.t1;34-9_1927;

2103 2134 95 1.000 1 2 34-9_1928;KKA02485.1;

2104 2135 41 1.000 1 4 model.g2323.t1;model.g672.t1;34-9_3398;34-9_1930;

2105 2136 29 1.000 1 3 KKA02484.1;model.g2324.t1;34-9_1931;

2106 2137 28 1.000 1 2 34-9_1932;model.g2325.t1;

2107 2138 6 0.502 1 4 model.g1166.t1;KKA01477.1;model.g2741.t1;34-9_1934;

2108 2139 17 1.000 1 3 KKA02480.1;model.g1162.t1;34-9_1935;

2109 2140 87 1.000 1 2 34-9_1936;KKA02479.1;

2110 2141 19 1.000 1 2 34-9_1937;model.g1160.t1;

2111 2142 73 1.000 1 2 34-9_1939;KKA02478.1;

2112 2143 29 1.000 1 3 KKA02477.1;model.g1159.t1;34-9_1941;

2113 2144 49 1.000 1 2 34-9_1943;model.g1168.t1;

2114 2145 99 1.000 1 2 34-9_1946;KKA02475.1;

2115 2146 28 1.000 1 3 KKA02473.1;model.g1173.t1;34-9_1947;

2116 2147 13 1.000 1 2 34-9_1949;model.g1175.t1;

2117 2148 42 1.000 1 3 KKA02472.1;model.g1176.t1;34-9_1950;

2118 2149 60 1.000 1 3 KKA02471.1;model.g1177.t1;34-9_1951;

2119 2150 42 1.000 1 2 34-9_1952;model.g1180.t1;

2120 2151 18 1.000 1 2 34-9_1953;model.g1182.t1;

2121 2152 14 1.000 1 2 34-9_1954;model.g1183.t1;

2122 2153 22 1.000 1 2 34-9_1955;model.g1187.t1;

2123 2154 39 1.000 1 3 KKA02468.1;model.g1188.t1;34-9_1956;

2124 2155 90 1.000 1 2 34-9_1958;KKA02466.1;

2125 2156 26 1.000 1 3 KKA02465.1;model.g1190.t1;34-9_1959;

2126 2157 94 1.000 1 2 34-9_1960;KKA02464.1;

2127 2158 12 1.000 1 2 34-9_1964;model.g1213.t1;

2128 2159 84 1.000 1 2 34-9_1965;KKA02462.1;

2129 2160 98 1.000 1 2 34-9_1966;KKA02461.1;

2130 2161 29 1.000 1 2 34-9_1967;model.g1189.t1;

2131 2162 32 1.000 1 3 KKA02460.1;model.g1209.t1;34-9_1968;

2132 2163 43 1.000 1 3 KKA02458.1;model.g483.t1;34-9_1970;

2133 2164 40 1.000 1 2 34-9_1971;model.g1206.t1;

2134 2165 25 1.000 1 2 34-9_1972;model.g1205.t1;

2135 2166 18 1.000 1 2 34-9_1973;model.g1204.t1;

2136 2167 33 1.000 1 3 KKA02457.1;model.g1203.t1;34-9_1974;

2137 2168 36 1.000 1 2 34-9_1975;model.g1202.t1;

2138 2169 23 1.000 1 3 KKA02456.1;model.g1201.t1;34-9_1976;

2139 2170 29 1.000 1 2 34-9_1978;model.g1197.t1;

2140 2171 31 1.000 1 2 34-9_1979;model.g1219.t1;

2141 2172 42 1.000 1 3 KKA02452.1;model.g1218.t1;34-9_1981;

2142 2173 17 1.000 1 3 KKA02451.1;model.g1217.t1;34-9_1982;

2143 2174 18 1.000 1 2 34-9_1984;model.g1215.t1;

2144 2175 40 1.000 1 3 KKA02449.1;model.g1258.t1;34-9_1985;

2145 2176 99 1.000 1 2 34-9_1986;KKA02448.1;

2146 2177 36 1.000 1 3 KKA02446.1;model.g1252.t1;34-9_1988;

2147 2178 25 1.000 1 2 34-9_1989;model.g1251.t1;

2148 2179 15 1.000 1 2 34-9_1990;model.g1250.t1;

2149 2180 32 1.000 1 3 KKA02445.1;model.g1249.t1;34-9_1991;

2150 2181 35 1.000 1 3 KKA02444.1;model.g1248.t1;34-9_1992;

2151 2182 53 1.000 1 3 KKA02443.1;model.g1247.t1;34-9_1993;

2152 2183 99 1.000 1 2 34-9_1994;KKA02442.1;

2153 2184 47 1.000 1 2 34-9_2000;model.g566.t1;

2154 2185 99 1.000 1 2 34-9_2006;KKA02570.1;

2155 2186 95 1.000 1 2 34-9_2009;KKA02572.1;

2156 2187 12 1.000 1 2 34-9_2014;model.g1008.t1;

2157 2188 33 1.000 1 2 34-9_2018;model.g1013.t1;

2158 2189 19 1.000 1 3 KKA02577.1;model.g1014.t1;34-9_2019;

2159 2190 66 1.000 1 2 34-9_2020;model.g1015.t1;

2160 2191 52 1.000 1 3 KKA02578.1;model.g1016.t1;34-9_2021;

2161 2192 42 1.000 1 2 34-9_2022;model.g1017.t1;

2162 2193 31 1.000 1 3 34-9_2854;KKA01163.1;model.g1524.t1;

2163 2194 59 1.000 1 2 34-9_2023;model.g1018.t1;

2164 2195 21 1.000 1 2 34-9_2024;model.g957.t1;

2165 2196 36 1.000 1 2 34-9_2025;model.g958.t1;

2166 2197 99 1.000 1 2 34-9_2026;KKA02579.1;

2167 2198 49 1.000 1 3 KKA02580.1;model.g956.t1;34-9_2027;

2168 2199 51 1.000 1 2 34-9_2029;model.g855.t1;

2169 2200 75 1.000 1 2 34-9_2030;KKA02581.1;

2170 2201 23 1.000 1 3 KKA02582.1;model.g854.t1;34-9_2031;

2171 2202 79 1.000 1 4 KKA02583.1;model.g851.t1;model.g857.t1;34-9_2032;

2172 2203 49 1.000 1 2 34-9_2035;model.g848.t1;

2173 2204 38 1.000 1 2 34-9_2036;model.g847.t1;

2174 2205 73 1.000 1 2 34-9_2037;model.g846.t1;

2175 2206 25 1.000 1 2 34-9_2038;model.g845.t1;

2176 2207 45 1.000 1 2 34-9_2040;model.g840.t1;

2177 2208 12 1.000 1 2 34-9_2041;model.g841.t1;

2178 2209 35 1.000 1 3 KKA02586.1;model.g1560.t1;34-9_2043;

2179 2210 70 1.000 1 3 KKA02588.1;model.g1565.t1;34-9_2046;

2180 2211 17 1.000 1 2 34-9_2047;model.g1553.t1;

2181 2212 45 1.000 1 2 34-9_2048;KKA02589.1;

2182 2213 38 1.000 1 2 34-9_2049;model.g1550.t1;

2183 2214 62 1.000 1 3 34-9_3071;model.g2770.t1;KKA02931.1;

2184 2215 40 1.000 1 3 KKA02591.1;model.g1549.t1;34-9_2050;

2185 2216 57 1.000 1 2 34-9_2052;KKA02592.1;

2186 2217 10 0.502 1 3 model.g2953.t1;model.g2954.t1;34-9_2053;

2187 2218 12 1.000 1 2 34-9_2055;model.g2960.t1;

2188 2219 47 1.000 1 3 KKA02594.1;model.g2959.t1;34-9_2056;

2189 2220 57 1.000 1 3 KKA02595.1;model.g2958.t1;34-9_2057;

2190 2221 16 1.000 1 3 KKA02596.1;model.g2957.t1;34-9_2058;

2191 2222 35 1.000 1 3 KKA02598.1;model.g2973.t1;34-9_2059;

2192 2223 46 1.000 1 3 KKA02599.1;model.g2968.t1;34-9_2061;

2193 2224 29 1.000 1 2 34-9_2062;model.g2967.t1;

2194 2225 18 1.000 1 2 34-9_2063;model.g2965.t1;

2195 2226 84 1.000 1 3 KKA02600.1;model.g2964.t1;34-9_2064;

2196 2227 76 1.000 1 2 34-9_2066;KKA02601.1;

2197 2228 35 1.000 1 3 KKA02604.1;model.g2975.t1;34-9_2069;

2198 2229 22 1.000 1 2 34-9_2070;model.g2976.t1;

2199 2230 72 1.000 1 2 34-9_2071;model.g2977.t1;

2200 2231 46 1.000 1 4 model.g817.t1;34-9_2730;model.g1107.t1;34-9_2072;

2201 2232 38 1.000 1 2 34-9_2073;model.g819.t1;

2202 2233 99 1.000 1 2 34-9_2074;KKA02605.1;

2203 2234 82 1.000 1 2 34-9_2075;KKA02606.1;

2204 2235 19 1.000 1 3 KKA02607.1;model.g822.t1;34-9_2076;

2205 2236 99 1.000 1 2 34-9_2078;KKA02608.1;

2206 2237 12 1.000 1 2 34-9_2079;model.g826.t1;

2207 2238 99 1.000 1 2 34-9_2081;KKA02609.1;

2208 2239 16 1.000 1 2 34-9_2082;model.g830.t1;

2209 2240 48 1.000 1 2 34-9_2085;model.g832.t1;

2210 2241 47 1.000 1 2 34-9_2086;model.g833.t1;

2211 2242 27 1.000 1 6 34-9_2874;KKA02611.1;KKA01275.1;model.g2969.t1;model.g1666.t1;34-9_2087;

2212 2242 23 1.000 1 2 KKA01274.1;model.g955.t1;

2213 2244 29 1.000 1 3 34-9_2671;KKA02144.1;model.g3452.t1;

2214 2245 29 1.000 1 3 KKA02612.1;model.g2956.t1;34-9_2088;

2215 2246 92 1.000 1 2 34-9_2092;KKA02616.1;

2216 2247 98 1.000 1 2 34-9_2096;KKA02618.1;

2217 2248 29 1.000 1 3 KKA02619.1;model.g705.t1;34-9_2097;

2218 2249 99 1.000 1 2 34-9_2098;KKA02620.1;

2219 2250 87 1.000 1 3 KKA02621.1;model.g707.t1;34-9_2099;

2220 2251 16 1.000 1 2 34-9_2100;model.g708.t1;

2221 2252 39 1.000 1 2 34-9_2103;model.g711.t1;

2222 2253 17 1.000 1 2 34-9_2104;model.g713.t1;

2223 2254 52 1.000 1 2 34-9_2106;model.g717.t1;

2224 2255 99 1.000 1 2 34-9_2107;KKA02622.1;

2225 2256 66 1.000 1 2 34-9_2108;KKA02623.1;

2226 2257 35 1.000 1 2 34-9_2112;model.g2979.t1;

2227 2258 30 1.000 1 2 34-9_2114;model.g690.t1;

2228 2259 16 1.000 1 2 34-9_2115;model.g689.t1;

2229 2260 48 1.000 1 2 34-9_2117;KKA01014.1;

2230 2261 41 1.000 1 3 KKA02628.1;model.g1415.t1;34-9_2119;

2231 2262 98 1.000 1 2 34-9_2120;KKA02629.1;

2232 2263 69 1.000 1 2 34-9_2121;KKA02630.1;

2233 2264 29 1.000 1 2 34-9_2122;model.g1414.t1;

2234 2265 28 1.000 1 2 34-9_2123;model.g1413.t1;

2235 2266 64 1.000 1 3 KKA02632.1;model.g2982.t1;34-9_2125;

2236 2267 64 1.000 1 3 KKA02633.1;model.g2983.t1;34-9_2126;

2237 2268 58 1.000 1 2 34-9_2127;model.g2985.t1;

2238 2269 26 1.000 1 3 KKA02634.1;model.g2986.t1;34-9_2128;

2239 2270 35 1.000 1 2 34-9_2129;model.g2987.t1;

2240 2271 99 1.000 1 2 34-9_2135;KKA03547.1;

2241 2272 98 1.000 1 2 34-9_2136;KKA03546.1;

2242 2273 16 1.000 1 3 KKA03544.1;model.g738.t1;34-9_2138;

2243 2274 28 1.000 1 2 34-9_2139;model.g2020.t1;

2244 2275 37 1.000 1 3 KKA03542.1;model.g735.t1;34-9_2140;

2245 2277 93 1.000 1 2 34-9_2162;KKA03522.1;

2246 2278 98 1.000 1 2 34-9_2170;KKA03512.1;

2247 2279 7 0.502 1 5 KKA03498.1;model.g367.t1;KKA03497.1;34-9_2184;34-9_2183;

2248 2280 70 1.000 1 2 34-9_2191;KKA03490.1;

2249 2281 77 1.000 1 2 34-9_2194;KKA03485.1;

2250 2282 99 1.000 1 2 34-9_2208;KKA03470.1;

2251 2283 86 1.000 1 2 34-9_2216;KKA03461.1;

2252 2284 59 1.000 1 2 34-9_2218;KKA03459.1;

2253 2285 94 1.000 1 2 34-9_2224;KKA03453.1;

2254 2286 99 1.000 1 2 34-9_2230;KKA03447.1;

2255 2287 99 1.000 1 2 34-9_2232;KKA03446.1;

2256 2288 18 0.502 1 3 KKA03440.1;KKA03441.1;34-9_2237;

2257 2289 92 1.000 1 2 34-9_2239;KKA03438.1;

2258 2290 54 1.000 1 2 34-9_2242;KKA03435.1;

2259 2291 11 0.776 1 6 model.g781.t1;KKA03433.1;34-9_2596;model.g748.t1;KKA01210.1;34-9_2244;

2260 2292 76 1.000 1 2 34-9_2245;KKA03432.1;

2261 2293 69 1.000 1 2 34-9_2246;KKA03430.1;

2262 2294 100 1.000 1 2 34-9_2247;KKA03429.1;

2263 2295 40 1.000 1 3 KKA03428.1;model.g766.t1;34-9_2248;

2264 2296 96 1.000 1 2 34-9_2250;KKA03426.1;

2265 2297 43 1.000 1 3 KKA03425.1;model.g752.t1;34-9_2251;

2266 2298 46 1.000 1 3 KKA03424.1;model.g764.t1;34-9_2252;

2267 2299 13 1.000 1 3 KKA03423.1;model.g761.t1;34-9_2254;

2268 2300 12 0.875 1 10 model.g1928.t1;34-9_3434;34-9_3217;KKA03382.1;model.g604.t1;34-9_3138;34-9_2292;KKA02672.1;model.g107.t1;model.g1494.t1;

2269 2301 23 1.000 1 3 KKA03422.1;model.g762.t1;34-9_2255;

2270 2302 64 1.000 1 2 34-9_2256;KKA03421.1;

2271 2303 26 1.000 1 3 KKA03420.1;model.g759.t1;34-9_2257;

2272 2304 19 1.000 1 3 KKA03419.1;model.g757.t1;34-9_2258;

2273 2305 25 1.000 1 3 KKA03418.1;model.g756.t1;34-9_2259;

2274 2306 31 1.000 1 3 KKA03413.1;model.g742.t1;34-9_2263;

2275 2307 46 1.000 1 3 KKA03412.1;model.g744.t1;34-9_2264;

2276 2308 15 1.000 1 6 34-9_2898;KKA03411.1;KKA01484.1;model.g745.t1;model.g2732.t1;34-9_2265;

2277 2309 15 1.000 1 3 KKA03410.1;model.g754.t1;34-9_2266;

2278 2310 36 1.000 1 3 KKA03407.1;model.g1925.t1;34-9_2269;

2279 2311 76 1.000 1 3 KKA03406.1;model.g1926.t1;34-9_2270;

2280 2312 15 1.000 1 3 KKA03405.1;model.g1920.t1;34-9_2271;

2281 2313 20 0.502 1 3 KKA03404.1;KKA03403.1;34-9_2272;

2282 2314 99 1.000 1 2 34-9_2275;KKA03400.1;

2283 2315 26 1.000 1 3 KKA03399.1;model.g1916.t1;34-9_2276;

2284 2316 28 1.000 1 3 KKA03397.1;model.g1915.t1;34-9_2277;

2285 2317 19 1.000 1 3 KKA03396.1;model.g1913.t1;34-9_2278;

2286 2318 29 1.000 1 3 KKA03394.1;model.g1910.t1;34-9_2280;

2287 2319 31 1.000 1 3 KKA03393.1;model.g1909.t1;34-9_2281;

2288 2320 22 1.000 1 4 34-9_2353;KKA03322.1;model.g1907.t1;model.g1908.t1;

2289 2321 98 1.000 1 2 34-9_2283;KKA03392.1;

2290 2322 21 1.000 1 3 KKA03388.1;model.g1902.t1;34-9_2287;

2291 2323 67 1.000 1 3 KKA03387.1;model.g774.t1;34-9_2288;

2292 2324 14 1.000 1 3 KKA03386.1;model.g1903.t1;34-9_2289;

2293 2325 12 1.000 1 3 KKA03385.1;model.g772.t1;34-9_2290;

2294 2326 32 1.000 1 3 KKA03381.1;model.g1927.t1;34-9_2293;

2295 2327 36 1.000 1 3 KKA03379.1;model.g769.t1;34-9_2295;

2296 2328 20 1.000 1 3 KKA03378.1;model.g770.t1;34-9_2296;

2297 2329 21 0.502 1 3 KKA03377.1;KKA03376.1;34-9_2297;

2298 2332 100 1.000 1 2 34-9_2298;KKA03375.1;

2299 2333 16 1.000 1 3 KKA03374.1;model.g3354.t1;34-9_2299;

2300 2334 14 1.000 1 2 34-9_2301;model.g3352.t1;

2301 2335 68 1.000 1 2 34-9_2303;model.g3350.t1;

2302 2336 14 1.000 1 3 KKA03371.1;model.g3349.t1;34-9_2304;

2303 2337 18 1.000 1 3 KKA03370.1;model.g3347.t1;34-9_2305;

2304 2338 79 1.000 1 3 KKA03369.1;model.g1931.t1;34-9_2306;

2305 2339 28 1.000 1 3 KKA03368.1;model.g1930.t1;34-9_2307;

2306 2340 11 1.000 1 3 KKA03367.1;model.g1929.t1;34-9_2308;

2307 2341 15 1.000 1 3 KKA03366.1;model.g3357.t1;34-9_2309;

2308 2342 98 1.000 1 2 34-9_2310;KKA03365.1;

2309 2343 97 1.000 1 2 34-9_2311;KKA03364.1;

2310 2344 54 1.000 1 3 KKA03363.1;model.g3196.t1;34-9_2312;

2311 2345 69 1.000 1 3 KKA03362.1;model.g3197.t1;34-9_2313;

2312 2346 94 1.000 1 2 34-9_2314;KKA03361.1;

2313 2347 34 1.000 1 4 KKA03360.1;model.g807.t1;model.g3148.t1;34-9_2315;

2314 2348 23 1.000 1 3 KKA03359.1;model.g3149.t1;34-9_2316;

2315 2349 89 1.000 1 2 34-9_2317;KKA03358.1;

2316 2350 23 1.000 1 3 KKA03357.1;model.g804.t1;34-9_2318;

2317 2351 29 1.000 1 3 KKA03356.1;model.g3174.t1;34-9_2319;

2318 2352 97 1.000 1 2 34-9_2320;KKA03355.1;

2319 2353 37 1.000 1 3 KKA03354.1;model.g3150.t1;34-9_2321;

2320 2354 49 1.000 1 3 KKA03352.1;model.g788.t1;34-9_2322;

2321 2355 13 1.000 1 3 KKA03351.1;model.g790.t1;34-9_2323;

2322 2356 35 1.000 1 3 KKA03350.1;model.g792.t1;34-9_2324;

2323 2357 14 1.000 1 3 KKA03348.1;model.g794.t1;34-9_2326;

2324 2358 17 1.000 1 3 KKA03347.1;model.g795.t1;34-9_2327;

2325 2359 13 1.000 1 3 KKA03345.1;model.g799.t1;34-9_2329;

2326 2360 90 1.000 1 2 34-9_2332;KKA03342.1;

2327 2361 42 1.000 1 3 KKA03341.1;model.g3395.t1;34-9_2333;

2328 2362 80 1.000 1 2 34-9_2334;KKA03340.1;

2329 2363 99 1.000 1 2 34-9_2335;KKA03339.1;

2330 2364 13 1.000 1 3 KKA03338.1;model.g3381.t1;34-9_2336;

2331 2365 63 1.000 1 2 34-9_2337;KKA03336.1;

2332 2366 12 1.000 1 3 KKA03335.1;model.g3384.t1;34-9_2338;

2333 2367 15 1.000 1 3 KKA03334.1;model.g3385.t1;34-9_2339;

2334 2368 99 1.000 1 2 34-9_2340;KKA03333.1;

2335 2369 15 1.000 1 3 KKA03332.1;model.g3392.t1;34-9_2341;

2336 2370 45 1.000 1 3 KKA03331.1;model.g3391.t1;34-9_2342;

2337 2371 20 1.000 1 2 34-9_2344;model.g3388.t1;

2338 2372 48 1.000 1 2 34-9_2345;KKA03329.1;

2339 2373 17 1.000 1 3 KKA03328.1;model.g3376.t1;34-9_2346;

2340 2374 32 1.000 1 3 KKA03327.1;model.g3377.t1;34-9_2347;

2341 2375 15 1.000 1 2 34-9_2348;model.g3379.t1;

2342 2376 100 1.000 1 2 34-9_2351;KKA03324.1;

2343 2377 97 1.000 1 2 34-9_2352;KKA03323.1;

2344 2378 99 1.000 1 2 34-9_2356;KKA03318.1;

2345 2379 13 1.000 1 3 KKA03317.1;model.g3363.t1;34-9_2357;

2346 2380 10 1.000 1 3 KKA03316.1;model.g3362.t1;34-9_2358;

2347 2381 11 1.000 1 3 KKA03315.1;model.g3361.t1;34-9_2359;

2348 2382 100 1.000 1 2 34-9_2360;KKA03314.1;

2349 2383 65 1.000 1 3 KKA03313.1;model.g3358.t1;34-9_2361;

2350 2384 100 1.000 1 2 34-9_2362;KKA03312.1;

2351 2385 99 1.000 1 2 34-9_2363;KKA03311.1;

2352 2386 31 1.000 1 3 KKA03310.1;model.g3185.t1;34-9_2364;

2353 2387 83 1.000 1 3 KKA03309.1;model.g3187.t1;34-9_2365;

2354 2388 18 1.000 1 3 KKA03308.1;model.g3186.t1;34-9_2366;

2355 2389 99 1.000 1 2 34-9_2368;KKA03307.1;

2356 2390 23 1.000 1 3 KKA03306.1;model.g3156.t1;34-9_2369;

2357 2391 100 1.000 1 2 34-9_2371;KKA03304.1;

2358 2392 89 1.000 1 2 34-9_2372;KKA03303.1;

2359 2393 29 1.000 1 2 34-9_2373;model.g3157.t1;

2360 2394 99 1.000 1 2 34-9_2374;KKA03302.1;

2361 2395 22 1.000 1 3 KKA03301.1;model.g3179.t1;34-9_2375;

2362 2396 48 1.000 1 2 34-9_2376;model.g3180.t1;

2363 2397 99 1.000 1 2 34-9_2377;KKA03300.1;

2364 2398 39 1.000 1 3 KKA03299.1;model.g3161.t1;34-9_2378;

2365 2399 88 1.000 1 2 34-9_2380;KKA03297.1;

2366 2400 15 0.502 1 3 34-9_2687;KKA02135.1;model.g1094.t1;

2367 2401 98 1.000 1 2 34-9_2381;KKA03296.1;

2368 2402 54 1.000 1 3 KKA03295.1;model.g3182.t1;34-9_2382;

2369 2403 99 1.000 1 2 34-9_2384;KKA03293.1;

2370 2404 98 1.000 1 2 34-9_2385;KKA03292.1;

2371 2405 98 1.000 1 2 34-9_2387;KKA03289.1;

2372 2406 86 1.000 1 2 34-9_2389;KKA03287.1;

2373 2407 99 1.000 1 2 34-9_2391;KKA03285.1;

2374 2408 99 1.000 1 2 34-9_2400;KKA01044.1;

2375 2409 100 1.000 1 2 34-9_2411;KKA02972.1;

2376 2410 89 1.000 1 2 34-9_2413;KKA02974.1;

2377 2411 12 1.000 1 3 KKA02976.1;model.g2509.t1;34-9_2416;

2378 2412 43 1.000 1 3 KKA02979.1;model.g3419.t1;34-9_2419;

2379 2413 72 1.000 1 2 34-9_2421;KKA02981.1;

2380 2414 87 1.000 1 2 34-9_2422;KKA02982.1;

2381 2415 23 1.000 1 3 KKA02983.1;model.g3434.t1;34-9_2423;

2382 2416 19 1.000 1 3 KKA02984.1;model.g3433.t1;34-9_2424;

2383 2417 96 1.000 1 2 34-9_2425;KKA02985.1;

2384 2418 34 1.000 1 3 KKA02986.1;model.g3422.t1;34-9_2426;

2385 2419 62 1.000 1 3 KKA02987.1;model.g3255.t1;34-9_2428;

2386 2420 42 1.000 1 3 KKA02989.1;model.g3427.t1;34-9_2430;

2387 2421 55 1.000 1 3 KKA02990.1;model.g3428.t1;34-9_2431;

2388 2422 23 1.000 1 3 KKA02991.1;model.g3429.t1;34-9_2432;

2389 2423 70 1.000 1 2 34-9_2433;KKA02992.1;

2390 2424 22 1.000 1 3 KKA03003.1;model.g3264.t1;34-9_2443;

2391 2425 29 1.000 1 4 KKA03004.1;34-9_2446;KKA03005.1;34-9_2445;

2392 2426 28 1.000 1 3 KKA03006.1;model.g3282.t1;34-9_2447;

2393 2427 11 1.000 1 3 KKA03007.1;model.g3273.t1;34-9_2448;

2394 2428 36 1.000 1 3 KKA03008.1;model.g3274.t1;34-9_2449;

2395 2429 15 1.000 1 3 KKA03009.1;model.g3275.t1;34-9_2450;

2396 2430 28 1.000 1 3 KKA03011.1;model.g3278.t1;34-9_2453;

2397 2432 98 1.000 1 2 34-9_2454;KKA03013.1;

2398 2433 18 1.000 1 3 KKA03014.1;model.g3272.t1;34-9_2456;

2399 2434 23 0.502 1 3 KKA03015.1;KKA03016.1;34-9_2457;

2400 2436 76 1.000 1 3 KKA03017.1;model.g3270.t1;34-9_2458;

2401 2437 78 1.000 1 3 KKA03018.1;model.g3269.t1;34-9_2459;

2402 2438 96 1.000 1 2 34-9_2460;KKA03019.1;

2403 2439 48 1.000 1 2 34-9_2466;KKA03023.1;

2404 2440 96 1.000 1 2 34-9_2477;KKA03034.1;

2405 2441 50 1.000 1 3 KKA03035.1;model.g3234.t1;34-9_2478;

2406 2442 81 1.000 1 2 34-9_2479;KKA03036.1;

2407 2443 97 1.000 1 2 34-9_2481;KKA03038.1;

2408 2444 98 1.000 1 2 34-9_2482;KKA03039.1;

2409 2445 31 1.000 1 3 KKA03042.1;model.g3204.t1;34-9_2487;

2410 2446 99 1.000 1 2 34-9_2489;KKA03043.1;

2411 2447 28 1.000 1 3 KKA03044.1;model.g3206.t1;34-9_2490;

2412 2448 18 1.000 1 2 34-9_2491;model.g3208.t1;

2413 2449 80 1.000 1 3 KKA03045.1;model.g3209.t1;34-9_2492;

2414 2450 93 1.000 1 2 34-9_2493;KKA03046.1;

2415 2451 18 1.000 1 3 KKA03049.1;model.g3214.t1;34-9_2496;

2416 2452 41 1.000 1 3 KKA03052.1;model.g3217.t1;34-9_2499;

2417 2453 21 1.000 1 3 KKA03053.1;model.g3237.t1;34-9_2500;

2418 2454 24 1.000 1 3 KKA03058.1;model.g3247.t1;34-9_2503;

2419 2455 99 1.000 1 2 34-9_2505;KKA03060.1;

2420 2456 31 1.000 1 3 KKA03061.1;model.g3250.t1;34-9_2506;

2421 2457 35 1.000 1 4 KKA03063.1;model.g3252.t1;model.g3253.t1;34-9_2508;

2422 2458 50 1.000 1 3 KKA03064.1;model.g3236.t1;34-9_2509;

2423 2459 37 1.000 1 2 34-9_2510;model.g3235.t1;

2424 2460 63 1.000 1 3 KKA03065.1;model.g3425.t1;34-9_2511;

2425 2461 91 1.000 1 2 34-9_2512;KKA03066.1;

2426 2462 23 1.000 1 3 KKA03067.1;model.g3219.t1;34-9_2513;

2427 2463 80 1.000 1 2 34-9_2514;KKA03068.1;

2428 2464 19 1.000 1 3 KKA03069.1;model.g3221.t1;34-9_2515;

2429 2465 28 1.000 1 3 KKA03070.1;model.g3222.t1;34-9_2516;

2430 2466 100 1.000 1 2 34-9_2517;KKA03071.1;

2431 2467 99 1.000 1 2 34-9_2518;KKA03072.1;

2432 2468 31 1.000 1 3 KKA03073.1;model.g3227.t1;34-9_2519;

2433 2469 99 1.000 1 2 34-9_2520;KKA03074.1;

2434 2470 67 1.000 1 2 34-9_2522;model.g3225.t1;

2435 2471 13 1.000 1 4 KKA01195.1;model.g3494.t1;model.g256.t1;34-9_2613;

2436 2473 16 1.000 1 6 KKA00951.1;KKA03077.1;KKA01837.1;model.g3229.t1;model.g682.t1;34-9_2524;

2437 2473 16 1.000 1 3 34-9_3274;model.g679.t1;KKA01836.1;

2438 2475 45 1.000 1 2 34-9_2527;model.g3198.t1;

2439 2476 69 1.000 1 3 KKA03080.1;model.g3151.t1;34-9_2528;

2440 2477 99 1.000 1 2 34-9_2530;KKA03082.1;

2441 2478 16 1.000 1 3 KKA03083.1;model.g2086.t1;34-9_2531;

2442 2479 96 1.000 1 2 34-9_2532;KKA03084.1;

2443 2480 22 1.000 1 3 KKA03086.1;model.g803.t1;34-9_2534;

2444 2481 62 1.000 1 3 KKA03087.1;model.g3172.t1;34-9_2535;

2445 2482 98 1.000 1 2 34-9_2536;KKA03088.1;

2446 2483 62 1.000 1 3 KKA03089.1;model.g3167.t1;34-9_2537;

2447 2484 87 1.000 1 2 34-9_2538;KKA03091.1;

2448 2485 100 1.000 1 2 34-9_2540;KKA03093.1;

2449 2486 90 1.000 1 3 KKA03095.1;model.g3164.t1;34-9_2542;

2450 2487 90 1.000 1 2 34-9_2543;KKA03096.1;

2451 2488 99 1.000 1 2 34-9_2544;KKA03097.1;

2452 2489 58 1.000 1 3 KKA03102.1;model.g3441.t1;34-9_2549;

2453 2490 86 1.000 1 2 34-9_2551;KKA03103.1;

2454 2491 71 1.000 1 2 34-9_2559;KKA03111.1;

2455 2492 98 1.000 1 2 34-9_2562;KKA03114.1;

2456 2493 99 1.000 1 2 34-9_2563;KKA03115.1;

2457 2494 39 1.000 1 3 KKA03116.1;model.g3439.t1;34-9_2564;

2458 2495 18 1.000 1 4 KKA03118.1;KKA03117.1;model.g3408.t1;34-9_2565;

2459 2496 10 1.000 1 2 34-9_2566;model.g3410.t1;

2460 2497 12 1.000 1 3 KKA03119.1;model.g3411.t1;34-9_2567;

2461 2498 31 1.000 1 2 34-9_2568;model.g3412.t1;

2462 2499 100 1.000 1 2 34-9_2570;KKA03120.1;

2463 2500 100 1.000 1 2 34-9_2571;KKA03121.1;

2464 2501 13 1.000 1 3 KKA03122.1;model.g3418.t1;34-9_2572;

2465 2502 21 1.000 1 2 34-9_2573;model.g3417.t1;

2466 2503 37 1.000 1 3 KKA03125.1;model.g3413.t1;34-9_2575;

2467 2504 98 1.000 1 2 34-9_2576;KKA03126.1;

2468 2505 99 1.000 1 2 34-9_2577;KKA03127.1;

2469 2506 100 1.000 1 2 34-9_2578;KKA03128.1;

2470 2507 99 1.000 1 2 34-9_2579;KKA03129.1;

2471 2508 22 0.502 1 3 KKA03130.1;KKA03131.1;34-9_2580;

2472 2509 33 1.000 1 3 KKA03132.1;model.g3397.t1;34-9_2582;

2473 2510 10 1.000 1 2 34-9_2583;model.g3398.t1;

2474 2511 99 1.000 1 2 34-9_2584;KKA03133.1;

2475 2512 28 1.000 1 3 KKA03134.1;model.g802.t1;34-9_2585;

2476 2513 80 1.000 1 2 34-9_2588;KKA01429.1;

2477 2514 19 1.000 1 2 34-9_2590;model.g304.t1;

2478 2515 99 1.000 1 2 34-9_2592;KKA00980.1;

2479 2516 53 1.000 1 2 34-9_3049;model.g997.t1;

2480 2517 62 1.000 1 2 34-9_2597;model.g782.t1;

2481 2518 92 1.000 1 2 34-9_2598;KKA01209.1;

2482 2519 39 1.000 1 3 KKA01208.1;model.g3345.t1;34-9_2599;

2483 2520 7 0.667 1 7 34-9_3581;KKA01207.1;KKA01029.1;model.g3344.t1;KKA01018.1;34-9_2600;model.g1752.t1;

2484 2521 14 1.000 1 3 KKA01205.1;model.g3341.t1;34-9_2602;

2485 2522 57 1.000 1 3 KKA01204.1;model.g3501.t1;34-9_2603;

2486 2523 23 1.000 1 3 KKA01203.1;model.g3502.t1;34-9_2604;

2487 2524 13 1.000 1 2 34-9_2605;model.g777.t1;

2488 2525 60 1.000 1 3 KKA01202.1;model.g778.t1;34-9_2606;

2489 2526 18 1.000 1 3 KKA01201.1;model.g779.t1;34-9_2607;

2490 2527 26 1.000 1 3 KKA01200.1;model.g3498.t1;34-9_2608;

2491 2528 99 1.000 1 2 34-9_2609;KKA01199.1;

2492 2529 97 1.000 1 2 34-9_2611;KKA01197.1;

2493 2530 75 1.000 1 3 KKA01196.1;model.g3495.t1;34-9_2612;

2494 2531 28 1.000 1 3 KKA01193.1;model.g3492.t1;34-9_2615;

2495 2532 88 1.000 1 2 34-9_2616;KKA01192.1;

2496 2533 19 1.000 1 3 KKA01190.1;model.g3474.t1;34-9_2618;

2497 2534 15 1.000 1 3 KKA01189.1;model.g3475.t1;34-9_2619;

2498 2535 57 1.000 1 3 KKA01188.1;model.g3479.t1;34-9_2620;

2499 2536 16 1.000 1 3 KKA01187.1;model.g3480.t1;34-9_2621;

2500 2537 33 1.000 1 3 KKA01186.1;model.g3486.t1;34-9_2622;

2501 2538 67 1.000 1 2 34-9_2623;KKA01133.1;

2502 2539 58 1.000 1 2 34-9_2625;model.g3489.t1;

2503 2540 37 1.000 1 3 KKA01131.1;model.g3481.t1;34-9_2627;

2504 2541 41 1.000 1 3 KKA01130.1;model.g3478.t1;34-9_2628;

2505 2542 99 1.000 1 2 34-9_2630;KKA01128.1;

2506 2543 36 1.000 1 2 34-9_2632;KKA01126.1;

2507 2544 29 1.000 1 3 KKA01124.1;model.g68.t1;34-9_2634;

2508 2545 100 1.000 1 2 34-9_2637;KKA01121.1;

2509 2546 14 1.000 1 2 34-9_2645;model.g1061.t1;

2510 2547 53 1.000 1 3 KKA02163.1;model.g1062.t1;34-9_2646;

2511 2548 81 1.000 1 2 34-9_2647;KKA02162.1;

2512 2549 24 1.000 1 3 KKA02161.1;model.g1065.t1;34-9_2648;

2513 2550 29 1.000 1 3 KKA02160.1;model.g1067.t1;34-9_2650;

2514 2551 24 0.502 1 3 KKA02159.1;KKA02158.1;34-9_2651;

2515 2552 13 1.000 1 2 34-9_2652;model.g1069.t1;

2516 2553 25 1.000 1 2 34-9_2654;model.g1071.t1;

2517 2554 23 1.000 1 3 KKA02157.1;model.g1072.t1;34-9_2655;

2518 2555 37 1.000 1 2 34-9_3063;model.g2764.t1;

2519 2556 45 1.000 1 2 34-9_2659;model.g1079.t1;

2520 2557 50 1.000 1 2 34-9_2661;model.g3443.t1;

2521 2558 23 1.000 1 2 34-9_2662;model.g3444.t1;

2522 2559 83 1.000 1 2 34-9_2665;KKA02148.1;

2523 2560 99 1.000 1 2 34-9_2666;KKA02147.1;

2524 2561 13 1.000 1 5 model.g3447.t1;KKA02113.1;model.g1123.t1;34-9_2667;34-9_2717;

2525 2562 71 1.000 1 2 34-9_2668;KKA02146.1;

2526 2563 52 1.000 1 3 KKA02145.1;model.g3449.t1;34-9_2669;

2527 2564 50 1.000 1 2 34-9_2670;model.g3451.t1;

2528 2565 50 1.000 1 3 KKA02143.1;model.g3455.t1;34-9_2673;

2529 2566 14 1.000 1 3 KKA02142.1;model.g1086.t1;34-9_2675;

2530 2567 29 1.000 1 3 KKA02141.1;model.g1088.t1;34-9_2677;

2531 2568 19 0.502 1 3 KKA02140.1;KKA02139.1;34-9_2678;

2532 2569 24 1.000 1 3 KKA02138.1;model.g1663.t1;34-9_2680;

2533 2570 55 1.000 1 2 34-9_2681;model.g1637.t1;

2534 2571 55 1.000 1 2 34-9_2682;model.g1638.t1;

2535 2572 18 1.000 1 3 model.g1639.t1;model.g1640.t1;34-9_2683;

2536 2573 34 1.000 1 3 KKA02136.1;model.g1095.t1;34-9_2686;

2537 2574 61 1.000 1 2 34-9_2688;model.g1093.t1;

2538 2575 6 0.502 1 3 KKA02134.1;model.g1089.t1;34-9_2689;

2539 2576 100 1.000 1 2 34-9_2691;KKA02131.1;

2540 2577 8 0.753 1 4 model.g1156.t1;34-9_3275;model.g681.t1;34-9_2694;

2541 2578 24 1.000 1 3 KKA02129.1;model.g1155.t1;34-9_2695;

2542 2579 13 1.000 1 2 34-9_2696;model.g1154.t1;

2543 2580 31 1.000 1 2 34-9_2697;model.g1153.t1;

2544 2581 31 1.000 1 2 34-9_2699;model.g1146.t1;

2545 2582 23 1.000 1 2 34-9_2700;model.g1145.t1;

2546 2583 97 1.000 1 2 34-9_2701;KKA02126.1;

2547 2584 52 1.000 1 2 34-9_2702;KKA02125.1;

2548 2585 15 1.000 1 3 KKA02124.1;model.g1141.t1;34-9_2703;

2549 2586 94 1.000 1 2 34-9_2704;KKA02123.1;

2550 2587 26 1.000 1 2 34-9_2705;model.g1139.t1;

2551 2588 5 0.502 1 3 KKA02122.1;model.g1138.t1;34-9_2706;

2552 2589 33 1.000 1 2 34-9_2707;model.g1137.t1;

2553 2590 62 1.000 1 3 KKA02121.1;model.g1136.t1;34-9_2708;

2554 2591 99 1.000 1 2 34-9_2709;KKA02120.1;

2555 2592 18 1.000 1 3 KKA02119.1;model.g1135.t1;34-9_2710;

2556 2593 39 1.000 1 2 34-9_2713;model.g1128.t1;

2557 2594 99 1.000 1 2 34-9_2714;KKA02116.1;

2558 2595 31 1.000 1 3 KKA02115.1;model.g1125.t1;34-9_2715;

2559 2596 52 1.000 1 3 KKA02114.1;model.g1124.t1;34-9_2716;

2560 2597 100 1.000 1 2 34-9_2718;KKA02112.1;

2561 2598 49 1.000 1 3 KKA02111.1;model.g1121.t1;34-9_2719;

2562 2599 51 1.000 1 3 KKA02109.1;model.g1118.t1;34-9_2722;

2563 2600 55 1.000 1 2 34-9_2723;KKA02108.1;

2564 2601 39 1.000 1 3 KKA02107.1;model.g1116.t1;34-9_2724;

2565 2602 56 1.000 1 2 34-9_2726;KKA02105.1;

2566 2603 73 1.000 1 2 34-9_2728;KKA02102.1;

2567 2604 100 1.000 1 2 34-9_2729;KKA02100.1;

2568 2605 68 1.000 1 3 KKA02099.1;model.g1106.t1;34-9_2731;

2569 2606 25 1.000 1 3 KKA01063.1;model.g1102.t1;34-9_2733;

2570 2607 46 1.000 1 3 KKA01064.1;model.g1706.t1;34-9_2734;

2571 2608 41 1.000 1 3 KKA01065.1;model.g1099.t1;34-9_2735;

2572 2609 81 1.000 1 3 KKA01066.1;model.g1098.t1;34-9_2736;

2573 2610 70 1.000 1 2 34-9_2747;KKA03227.1;

2574 2611 98 1.000 1 2 34-9_2749;KKA03229.1;

2575 2612 87 1.000 1 2 34-9_2750;KKA03230.1;

2576 2613 99 1.000 1 2 KKA01812.1;34-9_3298;

2577 2614 62 1.000 1 3 KKA03235.1;model.g2224.t1;34-9_2756;

2578 2615 85 1.000 1 2 34-9_2757;KKA03236.1;

2579 2616 98 1.000 1 2 34-9_2758;KKA03237.1;

2580 2617 17 1.000 1 4 KKA03239.1;model.g2219.t1;model.g928.t1;34-9_2760;

2581 2618 57 1.000 1 2 34-9_2761;model.g2218.t1;

2582 2619 7 0.502 1 3 KKA03240.1;model.g2217.t1;34-9_2762;

2583 2620 99 1.000 1 2 34-9_2771;KKA03247.1;

2584 2621 69 1.000 1 2 34-9_2776;KKA03250.1;

2585 2622 53 1.000 1 2 34-9_2780;KKA03251.1;

2586 2623 75 1.000 1 2 34-9_2788;KKA03256.1;

2587 2624 11 1.000 1 3 KKA03257.1;model.g628.t1;34-9_2790;

2588 2625 9 0.502 1 3 KKA03258.1;model.g624.t1;34-9_2791;

2589 2626 29 1.000 1 2 34-9_2793;model.g629.t1;

2590 2627 52 1.000 1 3 KKA03262.1;model.g632.t1;34-9_2794;

2591 2628 60 1.000 1 2 34-9_2801;model.g2229.t1;

2592 2629 22 1.000 1 3 KKA03265.1;model.g2230.t1;34-9_2802;

2593 2630 19 1.000 1 2 34-9_2803;model.g2231.t1;

2594 2631 30 1.000 1 3 KKA03266.1;model.g2235.t1;34-9_2806;

2595 2632 19 1.000 1 3 34-9_2809;model.g2237.t1;34-9_2808;

2596 2633 28 1.000 1 2 34-9_2811;model.g52.t1;

2597 2634 79 1.000 1 2 34-9_2812;model.g54.t1;

2598 2635 28 1.000 1 2 34-9_2813;model.g55.t1;

2599 2636 49 1.000 1 2 34-9_2814;KKA03267.1;

2600 2637 36 1.000 1 3 KKA03269.1;model.g3555.t1;34-9_2817;

2601 2638 6 0.502 1 4 KKA03271.1;34-9_3616;model.g2558.t1;34-9_2818;

2602 2639 72 1.000 1 2 34-9_2819;KKA03272.1;

2603 2640 93 1.000 1 2 34-9_2820;KKA03273.1;

2604 2641 29 1.000 1 3 model.g619.t1;model.g620.t1;34-9_2821;

2605 2642 15 1.000 1 3 KKA03276.1;model.g639.t1;34-9_2823;

2606 2643 80 1.000 1 2 34-9_2825;KKA03277.1;

2607 2644 82 1.000 1 3 KKA01803.1;model.g3567.t1;34-9_2834;

2608 2645 23 1.000 1 3 KKA01802.1;model.g3566.t1;34-9_2835;

2609 2646 20 1.000 1 3 KKA00989.1;model.g815.t1;34-9_2836;

2610 2647 19 1.000 1 3 KKA01148.1;model.g816.t1;34-9_2837;

2611 2648 17 1.000 1 3 KKA01149.1;model.g814.t1;34-9_2838;

2612 2649 52 1.000 1 2 34-9_2839;KKA01150.1;

2613 2650 18 1.000 1 3 KKA01152.1;model.g1541.t1;34-9_2841;

2614 2651 58 1.000 1 2 34-9_2843;KKA01155.1;

2615 2652 81 1.000 1 2 34-9_2845;model.g1536.t1;

2616 2653 98 1.000 1 2 34-9_2847;KKA01157.1;

2617 2654 97 1.000 1 2 34-9_2848;KKA01158.1;

2618 2655 48 1.000 1 3 KKA01159.1;model.g1533.t1;34-9_2849;

2619 2656 52 1.000 1 3 KKA01160.1;model.g1532.t1;34-9_2850;

2620 2657 98 1.000 1 2 34-9_2851;KKA01161.1;

2621 2658 46 1.000 1 2 34-9_2852;model.g1529.t1;

2622 2659 64 1.000 1 3 KKA01162.1;model.g1526.t1;34-9_2853;

2623 2660 99 1.000 1 2 34-9_2855;KKA01164.1;

2624 2661 72 1.000 1 2 34-9_2856;model.g1523.t1;

2625 2662 47 1.000 1 2 34-9_2857;model.g1521.t1;

2626 2663 75 1.000 1 2 34-9_2858;KKA01292.1;

2627 2664 33 1.000 1 2 34-9_2859;model.g1519.t1;

2628 2665 37 1.000 1 3 KKA01291.1;model.g1518.t1;34-9_2860;

2629 2666 31 1.000 1 3 KKA01289.1;model.g1513.t1;34-9_2863;

2630 2667 24 1.000 1 3 KKA01288.1;model.g1514.t1;34-9_2864;

2631 2668 38 1.000 1 3 KKA01284.1;model.g702.t1;34-9_2866;

2632 2669 25 1.000 1 3 KKA01281.1;model.g700.t1;34-9_2868;

2633 2671 98 1.000 1 2 34-9_2869;KKA01280.1;

2634 2672 58 1.000 1 3 KKA01587.1;model.g3328.t1;34-9_3314;

2635 2673 10 1.000 1 3 KKA01279.1;model.g2991.t1;34-9_2870;

2636 2674 85 1.000 1 3 KKA01278.1;model.g858.t1;34-9_2871;

2637 2675 74 1.000 1 2 34-9_2873;KKA01276.1;

2638 2676 59 1.000 1 3 KKA01273.1;model.g954.t1;34-9_2875;

2639 2677 19 1.000 1 3 KKA01269.1;model.g218.t1;34-9_2880;

2640 2678 75 1.000 1 3 KKA01471.1;model.g2747.t1;34-9_2884;

2641 2679 21 1.000 1 3 KKA01474.1;model.g2744.t1;34-9_2887;

2642 2680 28 1.000 1 3 KKA01475.1;model.g2743.t1;34-9_2888;

2643 2681 34 1.000 1 3 KKA01476.1;model.g2742.t1;34-9_2889;

2644 2682 16 1.000 1 2 34-9_2890;model.g2740.t1;

2645 2683 99 1.000 1 2 34-9_2891;KKA01478.1;

2646 2684 83 1.000 1 2 34-9_2892;model.g2738.t1;

2647 2685 36 1.000 1 3 KKA01481.1;model.g2735.t1;34-9_2895;

2648 2686 89 1.000 1 2 34-9_2897;KKA01483.1;

2649 2687 78 1.000 1 2 34-9_2899;KKA01485.1;

2650 2688 98 1.000 1 2 34-9_2900;KKA01486.1;

2651 2689 45 1.000 1 2 34-9_2902;KKA01487.1;

2652 2690 28 1.000 1 2 34-9_2903;model.g2726.t1;

2653 2691 21 1.000 1 3 KKA01488.1;model.g2725.t1;34-9_2904;

2654 2692 35 1.000 1 3 KKA01489.1;model.g2724.t1;34-9_2905;

2655 2693 14 1.000 1 3 KKA01490.1;model.g2723.t1;34-9_2906;

2656 2694 36 1.000 1 2 34-9_3124;model.g87.t1;

2657 2695 10 1.000 1 3 KKA01491.1;model.g2722.t1;34-9_2907;

2658 2696 42 1.000 1 3 KKA01495.1;model.g2718.t1;34-9_2910;

2659 2697 13 1.000 1 3 KKA01496.1;model.g2717.t1;34-9_2911;

2660 2698 75 1.000 1 2 34-9_2912;KKA01497.1;

2661 2699 44 1.000 1 3 KKA01498.1;model.g2715.t1;34-9_2913;

2662 2700 38 1.000 1 3 KKA01499.1;model.g1848.t1;34-9_2914;

2663 2701 20 1.000 1 3 KKA01500.1;model.g2713.t1;34-9_2915;

2664 2702 100 1.000 1 2 34-9_2916;KKA01501.1;

2665 2703 99 1.000 1 2 34-9_2917;KKA01502.1;

2666 2704 39 1.000 1 3 KKA01503.1;model.g1380.t1;34-9_2918;

2667 2705 98 1.000 1 2 34-9_2919;KKA01504.1;

2668 2706 97 1.000 1 3 KKA01083.1;KKA01505.1;34-9_2920;

2669 2707 36 1.000 1 7 KKA01081.1;34-9_2921;KKA01082.1;34-9_2922;KKA01506.1;model.g1378.t1;model.g1377.t1;

2670 2708 43 1.000 1 3 34-9_3620;KKA01560.1;model.g1824.t1;

2671 2709 16 1.000 1 3 KKA01080.1;model.g1376.t1;34-9_2923;

2672 2710 45 1.000 1 3 KKA01079.1;model.g1374.t1;34-9_2924;

2673 2711 98 1.000 1 2 34-9_2926;KKA01078.1;

2674 2712 100 1.000 1 2 34-9_2927;KKA01077.1;

2675 2713 57 1.000 1 2 34-9_2928;KKA01075.1;

2676 2714 44 1.000 1 3 KKA01073.1;model.g1366.t1;34-9_2929;

2677 2715 23 1.000 1 3 KKA01072.1;model.g1365.t1;34-9_2930;

2678 2716 70 1.000 1 2 34-9_2931;KKA01071.1;

2679 2717 33 1.000 1 4 KKA01070.1;model.g1363.t1;KKA01095.1;34-9_2932;

2680 2719 69 1.000 1 2 34-9_2934;KKA01184.1;

2681 2720 79 1.000 1 3 KKA01183.1;model.g1358.t1;34-9_2935;

2682 2721 22 1.000 1 3 KKA01182.1;model.g1357.t1;34-9_2936;

2683 2722 14 0.502 1 4 KKA01181.1;model.g1356.t1;model.g1355.t1;34-9_2937;

2684 2723 24 1.000 1 2 34-9_2938;model.g1354.t1;

2685 2724 11 1.000 1 3 KKA01180.1;model.g1353.t1;34-9_2939;

2686 2725 11 1.000 1 3 KKA01179.1;model.g1351.t1;34-9_2940;

2687 2726 100 1.000 1 2 34-9_2942;KKA01178.1;

2688 2727 78 1.000 1 2 34-9_2943;KKA01177.1;

2689 2728 12 1.000 1 2 34-9_2944;model.g2164.t1;

2690 2729 17 1.000 1 3 KKA01176.1;model.g1345.t1;34-9_2945;

2691 2731 99 1.000 1 2 34-9_2947;KKA01175.1;

2692 2732 15 1.000 1 3 KKA01174.1;model.g1342.t1;34-9_2949;

2693 2733 31 1.000 1 3 KKA01173.1;model.g1341.t1;34-9_2950;

2694 2734 25 1.000 1 3 KKA01172.1;model.g1340.t1;34-9_2951;

2695 2735 19 0.502 1 3 KKA01171.1;KKA01170.1;34-9_2952;

2696 2736 59 1.000 1 3 KKA01169.1;model.g1338.t1;34-9_2953;

2697 2737 40 1.000 1 3 KKA01168.1;model.g1337.t1;34-9_2954;

2698 2738 34 1.000 1 2 34-9_2957;model.g1329.t1;

2699 2739 16 1.000 1 3 KKA01619.1;model.g1332.t1;34-9_2960;

2700 2740 35 1.000 1 2 34-9_2962;model.g1324.t1;

2701 2741 33 1.000 1 2 34-9_2963;model.g1323.t1;

2702 2742 25 1.000 1 3 KKA01620.1;model.g1335.t1;34-9_2964;

2703 2743 32 1.000 1 2 34-9_2965;model.g1326.t1;

2704 2744 20 1.000 1 3 KKA01621.1;model.g1325.t1;34-9_2966;

2705 2745 66 1.000 1 2 34-9_2969;model.g1321.t1;

2706 2746 86 1.000 1 2 34-9_2970;KKA01622.1;

2707 2747 30 1.000 1 3 KKA01623.1;model.g1317.t1;34-9_2972;

2708 2748 61 1.000 1 3 34-9_3149;model.g118.t1;KKA02896.1;

2709 2749 30 1.000 1 3 34-9_3652;KKA01319.1;model.g3624.t1;

2710 2750 56 1.000 1 3 34-9_2977;34-9_2978;34-9_2976;

2711 2751 97 1.000 1 2 34-9_2985;KKA01631.1;

2712 2752 13 1.000 1 2 34-9_2986;model.g212.t1;

2713 2753 5 0.502 1 3 KKA01633.1;model.g214.t1;34-9_2987;

2714 2754 24 1.000 1 3 KKA01634.1;model.g2754.t1;34-9_2988;

2715 2755 27 1.000 1 2 34-9_2989;model.g2755.t1;

2716 2756 30 1.000 1 5 KKA01636.1;34-9_3695;model.g2705.t1;34-9_2993;model.g2704.t1;

2717 2757 96 1.000 1 2 34-9_2996;KKA01640.1;

2718 2758 79 1.000 1 2 34-9_2998;KKA01641.1;

2719 2759 16 1.000 1 3 KKA01646.1;model.g1024.t1;34-9_3002;

2720 2760 30 1.000 1 2 34-9_3003;model.g1023.t1;

2721 2761 22 1.000 1 2 34-9_3005;model.g1026.t1;

2722 2762 88 1.000 1 2 34-9_3006;KKA01648.1;

2723 2763 45 1.000 1 2 34-9_3007;model.g1028.t1;

2724 2764 57 1.000 1 2 34-9_3008;model.g1306.t1;

2725 2765 28 1.000 1 2 34-9_3011;model.g1310.t1;

2726 2766 34 1.000 1 3 KKA02966.1;model.g1312.t1;34-9_3013;

2727 2767 17 1.000 1 2 34-9_3015;model.g1314.t1;

2728 2768 75 1.000 1 2 34-9_3019;KKA02964.1;

2729 2769 53 1.000 1 2 34-9_3022;KKA02960.1;

2730 2770 51 1.000 1 2 34-9_3023;model.g965.t1;

2731 2771 98 1.000 1 2 34-9_3025;KKA02958.1;

2732 2772 50 1.000 1 2 34-9_3026;model.g968.t1;

2733 2773 6 0.502 1 4 KKA02957.1;model.g969.t1;34-9_3028;34-9_3027;

2734 2774 65 1.000 1 2 34-9_3029;model.g971.t1;

2735 2775 43 1.000 1 2 34-9_3030;model.g972.t1;

2736 2776 100 1.000 1 2 34-9_3031;KKA02955.1;

2737 2777 5 0.443 1 6 model.g1621.t1;KKA02954.1;34-9_3183;model.g974.t1;KKA02563.1;34-9_3032;

2738 2778 39 1.000 1 3 KKA02952.1;model.g978.t1;34-9_3036;

2739 2779 50 1.000 1 3 KKA02951.1;model.g980.t1;34-9_3038;

2740 2780 56 1.000 1 2 34-9_3040;model.g985.t1;

2741 2781 22 1.000 1 3 KKA02947.1;model.g982.t1;34-9_3041;

2742 2782 97 1.000 1 2 34-9_3043;KKA02946.1;

2743 2783 44 1.000 1 2 34-9_3045;model.g990.t1;

2744 2784 17 1.000 1 2 34-9_3048;model.g998.t1;

2745 2785 82 1.000 1 2 34-9_3050;KKA02942.1;

2746 2786 77 1.000 1 2 34-9_3051;KKA02941.1;

2747 2787 29 1.000 1 2 34-9_3052;model.g995.t1;

2748 2788 35 1.000 1 3 KKA02940.1;model.g994.t1;34-9_3053;

2749 2789 24 1.000 1 3 model.g986.t1;model.g1090.t1;34-9_3054;

2750 2790 47 1.000 1 2 34-9_3056;model.g1003.t1;

2751 2791 5 0.502 1 3 KKA02937.1;model.g2761.t1;34-9_3059;

2752 2792 36 1.000 1 3 KKA02936.1;model.g2765.t1;34-9_3061;

2753 2793 40 1.000 1 3 KKA02935.1;model.g2763.t1;34-9_3064;

2754 2794 72 1.000 1 2 34-9_3065;model.g2767.t1;

2755 2795 98 1.000 1 2 34-9_3066;KKA02934.1;

2756 2796 21 1.000 1 3 KKA02933.1;model.g2769.t1;34-9_3067;

2757 2797 44 1.000 1 2 34-9_3068;model.g2773.t1;

2758 2798 99 1.000 1 2 34-9_3069;KKA02932.1;

2759 2799 17 1.000 1 2 34-9_3070;model.g2771.t1;

2760 2800 26 1.000 1 3 KKA02927.1;model.g2776.t1;34-9_3075;

2761 2801 31 1.000 1 2 34-9_3076;model.g2777.t1;

2762 2802 19 1.000 1 2 34-9_3077;model.g2778.t1;

2763 2803 17 1.000 1 2 34-9_3082;model.g2785.t1;

2764 2804 16 1.000 1 2 34-9_3083;model.g2786.t1;

2765 2805 22 1.000 1 2 34-9_3084;model.g3403.t1;

2766 2806 14 1.000 1 2 34-9_3086;model.g2792.t1;

2767 2807 15 1.000 1 3 KKA02921.1;model.g2796.t1;34-9_3089;

2768 2808 24 1.000 1 5 KKA02920.1;KKA01616.1;model.g2797.t1;model.g1427.t1;34-9_3090;

2769 2809 20 1.000 1 2 34-9_3092;model.g49.t1;

2770 2810 43 1.000 1 2 34-9_3093;KKA02918.1;

2771 2811 27 1.000 1 2 34-9_3094;model.g46.t1;

2772 2812 46 1.000 1 3 KKA02917.1;model.g45.t1;34-9_3095;

2773 2813 34 1.000 1 2 34-9_3099;model.g41.t1;

2774 2814 85 1.000 1 2 34-9_3100;KKA02916.1;

2775 2815 77 1.000 1 2 34-9_3105;model.g33.t1;

2776 2816 32 1.000 1 2 34-9_3106;model.g32.t1;

2777 2817 10 1.000 1 2 34-9_3107;model.g31.t1;

2778 2818 84 1.000 1 4 KKA01472.1;KKA02913.1;model.g2746.t1;34-9_3108;

2779 2819 18 1.000 1 2 34-9_3109;model.g64.t1;

2780 2820 89 1.000 1 2 34-9_3111;KKA02912.1;

2781 2821 20 1.000 1 2 34-9_3113;model.g70.t1;

2782 2822 7 0.667 1 5 KKA02910.1;model.g2250.t1;model.g71.t1;34-9_3345;34-9_3114;

2783 2823 25 1.000 1 2 34-9_3116;model.g76.t1;

2784 2824 12 1.000 1 3 KKA02908.1;model.g77.t1;34-9_3117;

2785 2825 26 1.000 1 2 34-9_3119;model.g80.t1;

2786 2826 98 1.000 1 2 34-9_3120;KKA02906.1;

2787 2827 11 1.000 1 2 34-9_3125;model.g88.t1;

2788 2828 34 1.000 1 2 34-9_3127;model.g91.t1;

2789 2829 34 1.000 1 3 KKA02902.1;model.g475.t1;34-9_3131;

2790 2830 15 1.000 1 2 34-9_3132;model.g96.t1;

2791 2831 20 1.000 1 3 KKA02901.1;model.g97.t1;34-9_3133;

2792 2832 97 1.000 1 2 34-9_3134;KKA02900.1;

2793 2833 12 1.000 1 2 34-9_3135;model.g101.t1;

2794 2834 28 1.000 1 2 34-9_3137;model.g106.t1;

2795 2835 29 1.000 1 2 34-9_3139;model.g108.t1;

2796 2836 15 1.000 1 3 KKA02899.1;model.g109.t1;34-9_3140;

2797 2837 99 1.000 1 2 34-9_3142;KKA02898.1;

2798 2838 27 1.000 1 2 34-9_3143;model.g112.t1;

2799 2839 76 1.000 1 2 34-9_3144;KKA02897.1;

2800 2840 65 1.000 1 2 34-9_3145;model.g114.t1;

2801 2841 99 1.000 1 2 34-9_3153;KKA02893.1;

2802 2842 85 1.000 1 2 34-9_3155;model.g124.t1;

2803 2843 74 1.000 1 2 34-9_3156;model.g125.t1;

2804 2844 30 1.000 1 3 KKA02890.1;model.g127.t1;34-9_3158;

2805 2845 98 1.000 1 2 34-9_3164;KKA02884.1;

2806 2846 99 1.000 1 2 34-9_3166;KKA02883.1;

2807 2847 46 1.000 1 3 KKA02882.1;model.g1602.t1;34-9_3167;

2808 2848 99 1.000 1 2 34-9_3168;KKA02881.1;

2809 2849 23 1.000 1 2 34-9_3169;model.g1604.t1;

2810 2850 99 1.000 1 2 34-9_3170;KKA02880.1;

2811 2851 26 1.000 1 2 34-9_3172;model.g1607.t1;

2812 2852 81 1.000 1 2 34-9_3174;KKA02877.1;

2813 2853 19 1.000 1 2 34-9_3175;model.g1609.t1;

2814 2854 21 1.000 1 2 34-9_3177;model.g1613.t1;

2815 2855 19 1.000 1 2 34-9_3179;model.g1617.t1;

2816 2856 28 1.000 1 3 KKA02872.1;model.g1619.t1;34-9_3181;

2817 2857 28 1.000 1 3 34-9_3573;KKA02415.1;model.g1762.t1;

2818 2858 99 1.000 1 2 34-9_3185;KKA02562.1;

2819 2859 16 1.000 1 3 KKA02561.1;model.g1627.t1;34-9_3186;

2820 2860 9 0.667 1 4 KKA02560.1;KKA02559.1;model.g3557.t1;34-9_3187;

2821 2861 67 1.000 1 3 KKA02558.1;model.g3315.t1;34-9_3188;

2822 2862 70 1.000 1 3 KKA02557.1;model.g3319.t1;34-9_3190;

2823 2863 63 1.000 1 2 34-9_3191;model.g3320.t1;

2824 2864 99 1.000 1 2 34-9_3197;KKA02552.1;

2825 2865 100 1.000 1 2 34-9_3200;KKA02549.1;

2826 2866 73 1.000 1 2 34-9_3206;model.g587.t1;

2827 2867 14 1.000 1 2 34-9_3207;model.g588.t1;

2828 2868 32 1.000 1 2 34-9_3208;model.g589.t1;

2829 2869 46 1.000 1 3 KKA02542.1;model.g593.t1;34-9_3210;

2830 2870 99 1.000 1 2 34-9_3211;KKA02541.1;

2831 2871 19 1.000 1 2 34-9_3212;model.g597.t1;

2832 2872 15 0.502 1 4 KKA02539.1;KKA02540.1;model.g598.t1;34-9_3213;

2833 2874 38 1.000 1 2 34-9_3215;model.g602.t1;

2834 2875 42 1.000 1 2 34-9_3216;model.g603.t1;

2835 2876 23 1.000 1 3 KKA02537.1;model.g605.t1;34-9_3218;

2836 2877 14 1.000 1 2 34-9_3219;model.g606.t1;

2837 2878 63 1.000 1 2 34-9_3220;model.g607.t1;

2838 2879 65 1.000 1 3 KKA02536.1;model.g609.t1;34-9_3222;

2839 2880 79 1.000 1 2 34-9_3223;model.g936.t1;

2840 2881 98 1.000 1 2 34-9_3225;KKA02535.1;

2841 2882 14 1.000 1 2 34-9_3226;model.g136.t1;

2842 2883 20 0.502 1 3 KKA02533.1;KKA02534.1;34-9_3227;

2843 2884 44 1.000 1 3 KKA02532.1;model.g133.t1;34-9_3228;

2844 2885 5 0.502 1 3 KKA02531.1;model.g179.t1;34-9_3229;

2845 2886 22 1.000 1 3 KKA02530.1;model.g180.t1;34-9_3230;

2846 2887 22 1.000 1 3 KKA02524.1;model.g2538.t1;34-9_3239;

2847 2889 13 1.000 1 3 KKA02521.1;model.g2532.t1;34-9_3241;

2848 2890 13 1.000 1 3 KKA02520.1;model.g2531.t1;34-9_3242;

2849 2891 49 1.000 1 3 model.g2530.t1;model.g2529.t1;34-9_3243;

2850 2892 50 1.000 1 2 34-9_3244;model.g2528.t1;

2851 2893 8 0.502 1 3 model.g2527.t1;KKA02519.1;34-9_3245;

2852 2894 99 1.000 1 2 34-9_3249;KKA02518.1;

2853 2895 19 0.502 1 3 KKA02515.1;KKA02516.1;34-9_3252;

2854 2896 19 1.000 1 3 KKA02514.1;model.g247.t1;34-9_3253;

2855 2897 29 1.000 1 2 34-9_3255;model.g244.t1;

2856 2898 50 1.000 1 3 KKA02513.1;model.g243.t1;34-9_3256;

2857 2899 72 1.000 1 2 34-9_3258;KKA02511.1;

2858 2900 21 1.000 1 3 KKA01849.1;model.g675.t1;34-9_3261;

2859 2901 98 1.000 1 2 34-9_3262;KKA01848.1;

2860 2902 44 1.000 1 3 KKA01847.1;model.g676.t1;34-9_3263;

2861 2903 58 1.000 1 3 KKA01846.1;model.g677.t1;34-9_3264;

2862 2904 100 1.000 1 2 34-9_3266;KKA01844.1;

2863 2905 17 1.000 1 6 model.g2808.t1;KKA01843.1;34-9_3739;model.g2254.t1;KKA01138.1;34-9_3267;

2864 2906 99 1.000 1 2 34-9_3269;KKA01841.1;

2865 2907 99 1.000 1 2 34-9_3271;KKA01839.1;

2866 2908 55 1.000 1 3 KKA01835.1;model.g678.t1;34-9_3276;

2867 2909 98 1.000 1 2 34-9_3278;KKA01833.1;

2868 2910 99 1.000 1 2 34-9_3286;KKA01825.1;

2869 2911 26 1.000 1 3 KKA01823.1;model.g1449.t1;34-9_3288;

2870 2913 37 1.000 1 3 KKA01822.1;model.g1448.t1;34-9_3289;

2871 2914 24 1.000 1 3 KKA01821.1;model.g1423.t1;34-9_3290;

2872 2915 49 1.000 1 3 KKA01820.1;model.g1422.t1;34-9_3291;

2873 2916 72 1.000 1 2 34-9_3292;KKA01819.1;

2874 2917 97 1.000 1 2 34-9_3293;KKA01818.1;

2875 2918 85 1.000 1 3 KKA01817.1;model.g1419.t1;34-9_3294;

2876 2919 100 1.000 1 2 34-9_3295;KKA01816.1;

2877 2920 21 1.000 1 3 KKA01815.1;model.g1430.t1;34-9_3296;

2878 2921 57 1.000 1 2 34-9_3297;KKA01813.1;

2879 2922 99 1.000 1 2 34-9_3299;KKA01811.1;

2880 2923 26 1.000 1 3 KKA01810.1;model.g1436.t1;34-9_3300;

2881 2924 13 1.000 1 3 KKA01809.1;model.g1435.t1;34-9_3301;

2882 2925 98 1.000 1 2 34-9_3302;KKA01808.1;

2883 2926 23 1.000 1 3 KKA01807.1;model.g1437.t1;34-9_3303;

2884 2927 29 1.000 1 3 KKA01806.1;model.g1438.t1;34-9_3304;

2885 2928 99 1.000 1 2 34-9_3305;KKA01804.1;

2886 2929 29 1.000 1 2 34-9_3307;model.g1442.t1;

2887 2930 99 1.000 1 2 34-9_3308;KKA01585.1;

2888 2931 54 1.000 1 2 34-9_3310;model.g3333.t1;

2889 2932 53 1.000 1 2 34-9_3311;model.g3332.t1;

2890 2933 56 1.000 1 2 34-9_3312;KKA01586.1;

2891 2934 19 1.000 1 2 34-9_3313;model.g3327.t1;

2892 2935 59 1.000 1 3 KKA01588.1;model.g3329.t1;34-9_3315;

2893 2936 17 1.000 1 3 KKA01589.1;model.g3330.t1;34-9_3316;

2894 2937 98 1.000 1 2 34-9_3317;KKA01590.1;

2895 2938 65 1.000 1 2 34-9_3323;KKA01594.1;

2896 2939 100 1.000 1 2 34-9_3327;KKA01596.1;

2897 2940 49 1.000 1 2 34-9_3330;model.g2812.t1;

2898 2941 36 1.000 1 2 34-9_3339;KKA01604.1;

2899 2942 98 1.000 1 2 34-9_3341;KKA01608.1;

2900 2943 98 1.000 1 2 34-9_3344;KKA01610.1;

2901 2944 33 1.000 1 2 34-9_3346;model.g2248.t1;

2902 2945 55 1.000 1 2 34-9_3348;model.g2246.t1;

2903 2946 44 1.000 1 2 34-9_3349;KKA01612.1;

2904 2947 34 1.000 1 3 KKA01613.1;model.g3537.t1;34-9_3350;

2905 2948 61 1.000 1 3 KKA01614.1;model.g3536.t1;34-9_3351;

2906 2949 75 1.000 1 2 34-9_3352;model.g1446.t1;

2907 2950 13 1.000 1 3 KKA01615.1;model.g1424.t1;34-9_3353;

2908 2951 99 1.000 1 2 34-9_3355;KKA01618.1;

2909 2952 16 1.000 1 5 KKA02724.1;KKA03056.1;model.g836.t1;model.g3242.t1;34-9_3358;

2910 2953 99 1.000 1 2 34-9_3359;KKA02723.1;

2911 2954 23 0.502 1 3 KKA02721.1;KKA02722.1;34-9_3360;

2912 2955 38 1.000 1 3 KKA02720.1;model.g1589.t1;34-9_3361;

2913 2956 11 1.000 1 2 34-9_3363;model.g1591.t1;

2914 2957 89 1.000 1 2 34-9_3364;KKA02718.1;

2915 2958 100 1.000 1 2 34-9_3366;KKA02717.1;

2916 2959 23 1.000 1 3 KKA02715.1;model.g732.t1;34-9_3370;

2917 2960 25 1.000 1 2 34-9_3371;model.g1596.t1;

2918 2961 100 1.000 1 2 34-9_3372;KKA02714.1;

2919 2962 68 1.000 1 2 34-9_3374;model.g725.t1;

2920 2963 14 1.000 1 3 KKA02712.1;model.g724.t1;34-9_3375;

2921 2964 84 1.000 1 3 KKA02709.1;model.g660.t1;34-9_3377;

2922 2965 22 0.502 1 3 KKA02707.1;KKA02708.1;34-9_3379;

2923 2967 5 0.502 1 3 model.g663.t1;model.g664.t1;34-9_3380;

2924 2968 75 1.000 1 2 34-9_3383;model.g1555.t1;

2925 2969 25 1.000 1 2 34-9_3384;model.g1567.t1;

2926 2970 53 1.000 1 3 KKA02704.1;model.g1557.t1;34-9_3385;

2927 2971 44 1.000 1 3 KKA02702.1;model.g1512.t1;34-9_3388;

2928 2972 33 1.000 1 3 KKA02701.1;model.g1573.t1;34-9_3389;

2929 2973 87 1.000 1 2 34-9_3391;KKA02700.1;

2930 2974 87 1.000 1 2 34-9_3392;KKA02699.1;

2931 2975 23 1.000 1 3 KKA02698.1;model.g1464.t1;34-9_3393;

2932 2976 98 1.000 1 2 34-9_3394;KKA02697.1;

2933 2977 27 1.000 1 2 34-9_3395;model.g1462.t1;

2934 2978 41 1.000 1 3 KKA02696.1;model.g670.t1;34-9_3396;

2935 2979 31 1.000 1 2 34-9_3397;model.g671.t1;

2936 2980 11 1.000 1 2 34-9_3399;model.g1730.t1;

2937 2981 13 0.502 1 4 KKA02694.1;KKA02695.1;model.g673.t1;34-9_3400;

2938 2982 20 1.000 1 5 KKA02692.1;model.g668.t1;KKA02693.1;model.g669.t1;34-9_3401;

2939 2983 99 1.000 1 2 34-9_3402;KKA02691.1;

2940 2984 50 1.000 1 2 34-9_3403;model.g1584.t1;

2941 2985 15 1.000 1 2 34-9_3405;model.g1582.t1;

2942 2986 75 1.000 1 3 KKA02689.1;model.g1581.t1;34-9_3406;

2943 2987 16 1.000 1 3 KKA02687.1;model.g1579.t1;34-9_3407;

2944 2988 37 1.000 1 2 34-9_3408;model.g1578.t1;

2945 2989 13 1.000 1 2 34-9_3409;model.g1568.t1;

2946 2990 12 1.000 1 3 KKA02686.1;model.g1576.t1;34-9_3410;

2947 2991 33 1.000 1 3 KKA02685.1;model.g1577.t1;34-9_3411;

2948 2992 100 1.000 1 2 34-9_3412;KKA02684.1;

2949 2993 32 1.000 1 2 34-9_3413;model.g1471.t1;

2950 2994 66 1.000 1 3 KKA02682.1;model.g1470.t1;34-9_3415;

2951 2995 11 1.000 1 3 KKA02681.1;model.g1468.t1;34-9_3416;

2952 2996 42 1.000 1 2 34-9_3417;model.g1473.t1;

2953 2997 14 1.000 1 3 KKA02680.1;model.g1475.t1;34-9_3418;

2954 2998 72 1.000 1 3 KKA02679.1;model.g1476.t1;34-9_3419;

2955 2999 40 1.000 1 2 34-9_3420;model.g1477.t1;

2956 3000 55 1.000 1 3 KKA02678.1;model.g1478.t1;34-9_3421;

2957 3001 15 1.000 1 2 34-9_3422;model.g1479.t1;

2958 3002 31 1.000 1 2 34-9_3423;model.g1481.t1;

2959 3003 97 1.000 1 2 34-9_3424;KKA02677.1;

2960 3004 22 1.000 1 3 KKA02676.1;model.g1484.t1;34-9_3425;

2961 3005 60 1.000 1 2 34-9_3426;model.g1485.t1;

2962 3006 33 1.000 1 2 34-9_3427;model.g1487.t1;

2963 3007 35 1.000 1 2 34-9_3428;model.g1488.t1;

2964 3008 59 1.000 1 2 34-9_3429;model.g1489.t1;

2965 3009 73 1.000 1 2 34-9_3430;KKA02675.1;

2966 3010 30 1.000 1 2 34-9_3431;model.g1492.t1;

2967 3011 100 1.000 1 2 34-9_3432;KKA02673.1;

2968 3012 41 1.000 1 2 34-9_3433;model.g1493.t1;

2969 3013 20 1.000 1 2 34-9_3435;model.g1496.t1;

2970 3014 97 1.000 1 2 34-9_3438;KKA02670.1;

2971 3015 21 1.000 1 2 34-9_3439;model.g1502.t1;

2972 3016 99 1.000 1 2 34-9_3440;KKA02669.1;

2973 3017 64 1.000 1 2 34-9_3441;model.g1504.t1;

2974 3018 14 1.000 1 3 KKA02668.1;model.g1506.t1;34-9_3442;

2975 3019 98 1.000 1 2 34-9_3443;KKA02667.1;

2976 3020 26 1.000 1 2 34-9_3444;model.g1508.t1;

2977 3021 100 1.000 1 2 34-9_3447;KKA02664.1;

2978 3022 18 1.000 1 2 34-9_3452;model.g1559.t1;

2979 3023 13 1.000 1 3 KKA02656.1;model.g2243.t1;34-9_3456;

2980 3024 46 1.000 1 3 KKA02655.1;model.g2244.t1;34-9_3457;

2981 3025 21 1.000 1 3 KKA02654.1;model.g3538.t1;34-9_3458;

2982 3026 95 1.000 1 2 34-9_3460;KKA02652.1;

2983 3027 24 1.000 1 3 KKA02307.1;model.g230.t1;34-9_3470;

2984 3028 35 1.000 1 3 34-9_3472;34-9_3473;34-9_3471;

2985 3029 99 1.000 1 2 34-9_3476;KKA02314.1;

2986 3030 15 1.000 1 3 KKA02315.1;model.g3069.t1;34-9_3477;

2987 3031 54 1.000 1 3 KKA02316.1;model.g2473.t1;34-9_3478;

2988 3032 77 1.000 1 3 KKA02318.1;model.g2475.t1;34-9_3480;

2989 3033 100 1.000 1 2 34-9_3483;KKA02321.1;

2990 3034 41 1.000 1 2 34-9_3484;model.g2479.t1;

2991 3035 36 1.000 1 3 KKA02324.1;model.g2483.t1;34-9_3487;

2992 3036 19 1.000 1 3 KKA02325.1;model.g2482.t1;34-9_3488;

2993 3037 16 1.000 1 3 KKA02330.1;model.g647.t1;34-9_3493;

2994 3038 48 1.000 1 3 KKA02331.1;model.g646.t1;34-9_3494;

2995 3039 53 1.000 1 3 KKA02333.1;model.g1994.t1;34-9_3497;

2996 3040 51 1.000 1 3 KKA02335.1;model.g1992.t1;34-9_3499;

2997 3041 31 1.000 1 3 KKA02336.1;model.g1989.t1;34-9_3500;

2998 3042 12 1.000 1 3 KKA02338.1;model.g1987.t1;34-9_3502;

2999 3043 74 1.000 1 2 34-9_3503;model.g1986.t1;

3000 3044 53 1.000 1 3 KKA02339.1;model.g1985.t1;34-9_3504;

3001 3045 31 1.000 1 3 KKA02340.1;model.g1984.t1;34-9_3505;

3002 3046 100 1.000 1 2 34-9_3506;KKA02341.1;

3003 3047 22 1.000 1 3 KKA02343.1;model.g1980.t1;34-9_3508;

3004 3048 64 1.000 1 3 KKA02344.1;model.g1979.t1;34-9_3509;

3005 3049 56 1.000 1 3 KKA02347.1;model.g1976.t1;34-9_3512;

3006 3050 88 1.000 1 2 34-9_3515;KKA02350.1;

3007 3051 72 1.000 1 2 34-9_3521;KKA02359.1;

3008 3052 9 0.667 1 4 KKA02362.1;KKA02360.1;model.g2443.t1;34-9_3522;

3009 3053 16 1.000 1 3 KKA02365.1;model.g2439.t1;34-9_3525;

3010 3054 91 1.000 1 2 34-9_3527;KKA02368.1;

3011 3055 38 1.000 1 3 KKA02370.1;model.g2450.t1;34-9_3529;

3012 3056 100 1.000 1 2 34-9_3531;KKA02371.1;

3013 3057 100 1.000 1 2 34-9_3532;KKA02372.1;

3014 3058 36 1.000 1 3 KKA02373.1;model.g1809.t1;34-9_3533;

3015 3059 10 1.000 1 3 KKA02376.1;model.g1812.t1;34-9_3536;

3016 3060 41 1.000 1 3 KKA02378.1;model.g1815.t1;34-9_3537;

3017 3060 51 1.000 1 2 model.g1813.t1;KKA02377.1;

3018 3062 24 1.000 1 3 KKA02379.1;model.g1816.t1;34-9_3538;

3019 3063 29 1.000 1 3 KKA02381.1;model.g1818.t1;34-9_3540;

3020 3064 35 1.000 1 3 KKA02382.1;model.g1817.t1;34-9_3541;

3021 3065 10 1.000 1 3 KKA02384.1;model.g3654.t1;34-9_3543;

3022 3066 29 1.000 1 3 KKA02385.1;model.g3655.t1;34-9_3544;

3023 3067 36 1.000 1 2 34-9_3548;KKA02389.1;

3024 3068 38 1.000 1 2 34-9_3550;model.g3615.t1;

3025 3069 11 1.000 1 3 KKA02393.1;model.g3613.t1;34-9_3551;

3026 3070 90 1.000 1 2 34-9_3559;KKA02401.1;

3027 3071 31 1.000 1 3 KKA02404.1;model.g1775.t1;34-9_3561;

3028 3072 71 1.000 1 3 KKA02405.1;model.g1774.t1;34-9_3562;

3029 3073 27 1.000 1 2 34-9_3564;model.g1772.t1;

3030 3074 22 1.000 1 3 KKA02407.1;model.g1771.t1;34-9_3565;

3031 3075 19 1.000 1 3 KKA02408.1;model.g1770.t1;34-9_3566;

3032 3076 90 1.000 1 2 34-9_3567;KKA02409.1;

3033 3077 22 1.000 1 3 KKA02410.1;model.g1768.t1;34-9_3568;

3034 3078 89 1.000 1 2 34-9_3569;KKA02411.1;

3035 3079 46 1.000 1 3 KKA02412.1;model.g1766.t1;34-9_3570;

3036 3080 21 1.000 1 3 KKA02413.1;model.g1765.t1;34-9_3571;

3037 3081 90 1.000 1 3 KKA02416.1;model.g1760.t1;34-9_3575;

3038 3082 67 1.000 1 2 KKA03152.1;model.g903.t1;

3039 3083 39 1.000 1 3 KKA02417.1;model.g1759.t1;34-9_3576;

3040 3084 13 1.000 1 3 KKA02418.1;model.g1758.t1;34-9_3577;

3041 3085 56 1.000 1 2 34-9_3578;model.g1749.t1;

3042 3086 55 1.000 1 3 KKA01027.1;KKA01534.1;34-9_3579;

3043 3087 12 1.000 1 2 34-9_3583;model.g1755.t1;

3044 3088 54 1.000 1 3 KKA01536.1;model.g1756.t1;34-9_3584;

3045 3089 98 1.000 1 2 34-9_3586;KKA01537.1;

3046 3090 67 1.000 1 3 KKA01538.1;model.g1778.t1;34-9_3588;

3047 3091 47 1.000 1 3 KKA01539.1;model.g1779.t1;34-9_3589;

3048 3092 99 1.000 1 2 34-9_3590;KKA01540.1;

3049 3093 20 1.000 1 3 KKA01542.1;model.g1783.t1;34-9_3592;

3050 3094 44 1.000 1 3 KKA01543.1;model.g1785.t1;34-9_3594;

3051 3095 80 1.000 1 3 KKA01544.1;model.g1787.t1;34-9_3595;

3052 3096 17 1.000 1 3 KKA01546.1;model.g1790.t1;34-9_3598;

3053 3098 10 1.000 1 2 34-9_3600;model.g1794.t1;

3054 3099 36 1.000 1 2 34-9_3601;model.g1795.t1;

3055 3100 45 1.000 1 3 KKA01547.1;model.g1796.t1;34-9_3602;

3056 3101 20 1.000 1 3 KKA01548.1;model.g1798.t1;34-9_3603;

3057 3102 54 1.000 1 3 KKA01549.1;model.g1799.t1;34-9_3604;

3058 3103 25 1.000 1 3 KKA01550.1;model.g1800.t1;34-9_3605;

3059 3104 99 1.000 1 2 34-9_3606;KKA01551.1;

3060 3105 39 1.000 1 2 34-9_3607;model.g1803.t1;

3061 3106 5 0.502 1 3 KKA01552.1;model.g1804.t1;34-9_3608;

3062 3107 69 1.000 1 3 KKA01555.1;model.g2560.t1;34-9_3614;

3063 3108 98 1.000 1 2 34-9_3617;KKA01556.1;

3064 3109 30 1.000 1 3 KKA01557.1;model.g645.t1;34-9_3618;

3065 3110 29 1.000 1 2 34-9_3622;model.g1825.t1;

3066 3111 60 1.000 1 2 34-9_3623;KKA01561.1;

3067 3112 73 1.000 1 2 34-9_3625;KKA01294.1;

3068 3113 38 1.000 1 3 KKA01295.1;model.g2553.t1;34-9_3626;

3069 3114 12 1.000 1 2 34-9_3627;model.g1807.t1;

3070 3115 98 1.000 1 2 34-9_3634;KKA01302.1;

3071 3116 44 1.000 1 3 KKA01306.1;model.g3666.t1;34-9_3639;

3072 3117 79 1.000 1 3 KKA01308.1;model.g3664.t1;34-9_3641;

3073 3118 99 1.000 1 2 34-9_3642;KKA01309.1;

3074 3119 99 1.000 1 2 34-9_3644;KKA01311.1;

3075 3120 99 1.000 1 2 34-9_3647;KKA01315.1;

3076 3121 99 1.000 1 2 34-9_3654;KKA01321.1;

3077 3122 35 1.000 1 2 34-9_3655;model.g3660.t1;

3078 3123 37 1.000 1 3 KKA01325.1;model.g3634.t1;34-9_3657;

3079 3124 72 1.000 1 3 KKA01326.1;model.g3635.t1;34-9_3658;

3080 3125 75 1.000 1 2 34-9_3659;KKA01327.1;

3081 3126 27 1.000 1 3 KKA03155.1;model.g906.t1;34-9_3667;

3082 3127 66 1.000 1 2 34-9_3669;KKA03153.1;

3083 3128 95 1.000 1 2 34-9_3670;KKA03151.1;

3084 3129 99 1.000 1 2 34-9_3672;KKA03150.1;

3085 3130 98 1.000 1 2 34-9_3675;KKA03147.1;

3086 3131 32 1.000 1 2 34-9_3676;model.g1724.t1;

3087 3132 29 1.000 1 2 34-9_3677;model.g1723.t1;

3088 3133 18 1.000 1 3 KKA03146.1;model.g1722.t1;34-9_3678;

3089 3133 69 1.000 1 2 KKA02128.1;model.g1148.t1;

3090 3135 49 1.000 1 3 KKA03144.1;model.g1720.t1;34-9_3680;

3091 3136 63 1.000 1 2 34-9_3681;model.g1719.t1;

3092 3137 20 1.000 1 3 KKA03143.1;model.g1735.t1;34-9_3682;

3093 3139 51 1.000 1 3 KKA03141.1;model.g1729.t1;34-9_3684;

3094 3140 64 1.000 1 3 KKA03140.1;model.g1741.t1;34-9_3685;

3095 3141 98 1.000 1 2 34-9_3686;KKA03139.1;

3096 3142 100 1.000 1 2 34-9_3688;KKA03137.1;

3097 3143 98 1.000 1 2 34-9_3692;KKA01009.1;

3098 3144 21 1.000 1 4 KKA01010.1;KKA01452.1;model.g2707.t1;34-9_3693;

3099 3145 40 1.000 1 4 KKA01453.1;KKA01011.1;model.g2706.t1;34-9_3694;

3100 3146 71 1.000 1 2 34-9_3697;KKA01455.1;

3101 3147 11 1.000 1 2 34-9_3698;model.g2701.t1;

3102 3148 7 0.502 1 3 model.g1254.t1;KKA02447.1;34-9_3700;

3103 3149 18 1.000 1 2 34-9_3701;model.g1717.t1;

3104 3150 10 1.000 1 2 34-9_3702;model.g1718.t1;

3105 3151 74 1.000 1 2 34-9_3705;KKA01459.1;

3106 3152 10 1.000 1 2 34-9_3706;model.g885.t1;

3107 3153 53 1.000 1 2 34-9_3711;model.g1731.t1;

3108 3154 20 1.000 1 3 KKA01462.1;model.g1738.t1;34-9_3715;

3109 3155 17 1.000 1 2 34-9_3717;model.g882.t1;

3110 3156 98 1.000 1 2 34-9_3720;KKA01464.1;

3111 3157 41 1.000 1 3 KKA01465.1;model.g1736.t1;34-9_3721;

3112 3158 66 1.000 1 3 KKA01466.1;model.g889.t1;34-9_3722;

3113 3159 35 1.000 1 3 KKA01467.1;model.g890.t1;34-9_3723;

3114 3160 43 1.000 1 2 34-9_3725;model.g893.t1;

3115 3161 47 1.000 1 3 KKA01468.1;model.g894.t1;34-9_3726;

3116 3162 72 1.000 1 2 34-9_3727;model.g895.t1;

3117 3163 65 1.000 1 3 KKA01469.1;model.g896.t1;34-9_3728;

3118 3164 11 1.000 1 2 34-9_3733;model.g2799.t1;

3119 3165 73 1.000 1 2 34-9_3734;KKA01134.1;

3120 3166 98 1.000 1 2 34-9_3736;KKA01135.1;

3121 3167 18 1.000 1 3 KKA01137.1;model.g2805.t1;34-9_3738;

3122 3168 55 1.000 1 3 KKA01139.1;model.g2807.t1;34-9_3740;

3123 3169 15 1.000 1 3 KKA01140.1;model.g2806.t1;34-9_3741;

3124 3170 53 1.000 1 3 KKA01141.1;model.g2809.t1;34-9_3742;

3125 3171 59 1.000 1 3 KKA01142.1;model.g2810.t1;34-9_3743;

3126 3172 98 1.000 1 2 34-9_3746;KKA01146.1;

3127 3173 40 1.000 1 3 KKA01147.1;model.g2817.t1;34-9_3747;

3128 3174 10 1.000 1 3 KKA01330.1;model.g1658.t1;34-9_3749;

3129 3175 84 1.000 1 3 KKA01331.1;model.g1659.t1;34-9_3750;

3130 3176 29 1.000 1 3 KKA01332.1;model.g1660.t1;34-9_3751;

3131 3177 24 1.000 1 3 KKA01334.1;model.g1636.t1;34-9_3753;

3132 3178 22 1.000 1 3 KKA01336.1;model.g1631.t1;34-9_3755;

3133 3179 94 1.000 1 2 34-9_3756;KKA01337.1;

3134 3180 14 1.000 1 3 KKA01339.1;model.g3312.t1;34-9_3758;

3135 3181 68 1.000 1 3 KKA01341.1;model.g3302.t1;34-9_3760;

3136 3182 18 1.000 1 3 KKA01342.1;model.g3309.t1;34-9_3761;

3137 3183 25 1.000 1 3 KKA01343.1;model.g3306.t1;34-9_3762;

3138 3184 99 1.000 1 2 34-9_3763;KKA01344.1;

3139 3185 98 1.000 1 2 34-9_3765;KKA01346.1;

3140 3186 67 1.000 1 3 KKA01350.1;model.g3301.t1;34-9_3769;

3141 3187 96 1.000 1 2 34-9_3770;KKA01351.1;

3142 3188 99 1.000 1 2 34-9_3771;KKA01352.1;

3143 3189 87 1.000 1 2 34-9_3772;KKA01353.1;

3144 3190 10 1.000 1 3 KKA01354.1;model.g3296.t1;34-9_3774;

3145 3191 36 1.000 1 3 KKA01356.1;model.g3294.t1;34-9_3776;

3146 3192 24 1.000 1 2 34-9_3777;model.g3293.t1;

3147 3193 6 0.502 1 3 KKA01357.1;model.g3288.t1;34-9_3778;

3148 3194 14 0.502 1 3 KKA01358.1;model.g3292.t1;34-9_3779;

3149 3195 40 1.000 1 3 KKA01359.1;model.g3291.t1;34-9_3780;

3150 3196 24 1.000 1 2 34-9_3781;model.g3290.t1;

3151 3197 42 1.000 1 3 KKA01360.1;model.g3289.t1;34-9_3782;

3152 3198 99 1.000 1 2 34-9_3783;KKA01361.1;

3153 3199 60 1.000 1 3 KKA01362.1;model.g3285.t1;34-9_3784;

3154 3200 90 1.000 1 2 34-9_3786;KKA01364.1;

3155 3201 98 1.000 1 2 34-9_3787;KKA01365.1;

3156 3202 13 1.000 1 2 KKA03964.1;model.g2073.t1;

3157 3203 18 1.000 1 2 KKA03723.1;model.g2435.t1;

3158 3204 51 1.000 1 2 KKA03963.1;model.g2072.t1;

3159 3205 32 1.000 1 2 KKA03875.1;model.g1034.t1;

3160 3206 23 1.000 1 2 KKA03606.1;model.g222.t1;

3161 3207 20 1.000 1 2 KKA03543.1;model.g2019.t1;

3162 3208 12 1.000 1 2 KKA03353.1;model.g787.t1;

3163 3209 38 1.000 1 2 KKA03408.1;model.g1924.t1;

3164 3210 12 1.000 1 2 KKA03184.1;model.g2698.t1;

3165 3211 19 1.000 1 2 KKA03259.1;model.g625.t1;

3166 3212 68 1.000 1 2 KKA03260.1;model.g623.t1;

3167 3213 24 1.000 1 2 KKA03173.1;model.g2667.t1;

3168 3214 10 1.000 1 2 KKA03209.1;model.g2498.t1;

3169 3215 15 1.000 1 2 KKA03090.1;model.g3168.t1;

3170 3216 10 1.000 1 2 KKA03055.1;model.g3241.t1;

3171 3217 28 1.000 1 2 KKA02939.1;model.g1002.t1;

3172 3218 41 1.000 1 2 KKA02868.1;model.g2591.t1;

3173 3219 46 1.000 1 2 KKA02793.1;model.g196.t1;

3174 3220 16 1.000 1 2 KKA02688.1;model.g1580.t1;

3175 3221 26 1.000 1 2 KKA02657.1;model.g2242.t1;

3176 3222 42 1.000 1 2 KKA02597.1;model.g2972.t1;

3177 3223 32 1.000 1 2 KKA02523.1;model.g2539.t1;

3178 3224 50 1.000 1 4 KKA02440.1;model.g1291.t1;model.g1292.t1;KKA02439.1;

3179 3225 24 1.000 1 2 KKA02469.1;model.g1184.t1;

3180 3226 20 1.000 1 2 KKA02470.1;model.g1179.t1;

3181 3227 10 1.000 1 2 KKA02474.1;model.g4.t1;

3182 3228 23 1.000 1 2 KKA02361.1;model.g2444.t1;

3183 3229 19 1.000 1 2 KKA02230.1;model.g2938.t1;

3184 3230 16 1.000 1 2 KKA02241.1;model.g2878.t1;

3185 3231 32 1.000 1 2 KKA02195.1;model.g151.t1;

3186 3232 21 1.000 1 3 model.g1747.t1;model.g2827.t1;model.g3284.t1;

3187 3233 52 1.000 1 2 KKA02154.1;model.g1077.t1;

3188 3234 32 1.000 1 2 KKA02069.1;model.g3042.t1;

3189 3235 18 1.000 1 2 KKA02029.1;model.g473.t1;

3190 3236 55 1.000 1 2 KKA01894.1;model.g3079.t1;

3191 3237 67 1.000 1 2 KKA01805.1;model.g1440.t1;

3192 3238 20 1.000 1 2 KKA01853.1;model.g2263.t1;

3193 3239 28 1.000 1 2 KKA01829.1;model.g1457.t1;

3194 3240 100 1.000 1 2 KKA01685.1;KKA01448.1;

3195 3241 50 1.000 1 2 KKA01566.1;model.g1393.t1;

3196 3242 16 1.000 1 2 KKA01559.1;model.g2554.t1;

3197 3243 33 1.000 1 2 KKA01507.1;model.g1936.t1;

3198 3244 15 1.000 1 2 KKA01456.1;model.g2700.t1;

3199 3245 16 1.000 1 2 KKA01411.1;model.g2826.t1;

3200 3246 29 1.000 1 2 KKA01387.1;model.g2576.t1;

3201 3247 35 1.000 1 2 KKA01165.1;model.g1522.t1;

3202 3248 75 1.000 1 3 model.g2813.t1;model.g2391.t1;KKA01143.1;

3203 3249 44 1.000 1 2 KKA01117.1;KKA00960.1;

3204 3250 11 1.000 1 2 KKA01103.1;model.g2583.t1;

3205 3251 22 1.000 1 2 KKA01108.1;model.g2585.t1;

3206 3252 68 1.000 1 2 KKA01118.1;KKA00961.1;

3207 3253 52 1.000 1 2 KKA01098.1;model.g1263.t1;

3208 3254 30 1.000 1 2 KKA01097.1;model.g1264.t1;

3209 3255 26 1.000 1 2 KKA01076.1;model.g1369.t1;

3210 3256 18 1.000 1 3 KKA01019.1;model.g1753.t1;KKA01030.1;

3211 3257 100 1.000 1 2 KKA01005.1;KKA01001.1;

3212 3258 100 1.000 1 2 model.g7.t1;model.g1656.t1;

3213 3259 24 1.000 1 2 model.g27.t1;model.g2181.t1;

3214 3260 100 1.000 1 2 model.g51.t1;model.g2798.t1;

3215 3261 31 1.000 1 3 model.g2942.t1;model.g3336.t1;model.g58.t1;

3216 3262 37 1.000 1 4 model.g72.t1;model.g73.t1;model.g2766.t1;model.g74.t1;

3217 3263 22 1.000 1 2 model.g350.t1;model.g3372.t1;

3218 3264 23 1.000 1 2 model.g545.t1;model.g546.t1;

3219 3265 11 1.000 1 2 model.g595.t1;model.g3568.t1;

3220 3266 50 1.000 1 2 model.g775.t1;model.g3503.t1;

3221 3267 40 1.000 1 2 model.g1623.t1;model.g1624.t1;

3222 3268 17 0.502 1 3 model.g2818.t1;model.g3346.t1;model.g1932.t1;

3223 3269 34 1.000 1 2 model.g2621.t1;model.g2622.t1;
