# Supplementary material for: The Shared and Specific Genes and a Comparative Genomics Analysis within Three Hanseniaspora Strains
Source: Int J Genomics. 2019 Jun 2;2019:7910865. doi: 10.1155/2019/7910865 (PMC6589277; doi:10.1155/2019/7910865)
Supplement: Supplementary 5 — File 4: Results of the shared gene families. [file 7910865.f5.doc]

**Supplementary Table 4.** Results of the shared gene families.

**Multiple-copy**

4 3 12 0.753 1 6 model.g239.t1;KKA02392.1;model.g1300.t1;34-9_1860;model.g3614.t1;34-9_1526;

5 3 14 1.000 1 4 model.g1551.t1;34-9_1095;KKA03962.1;model.g2071.t1;

17 16 4 0.376 1 10 34-9_2349;model.g3364.t1;KKA03326.1;34-9_3474;model.g459.t1;34-9_3148;34-9_0394;KKA02312.1;model.g4617.t1;model.g117.t1;

18 16 7 0.604 1 24 KKA02149.1;model.g1499.t1;34-9_2664;model.g1647.t1;model.g2247.t1;KKA01658.1;34-9_2017;KKA01611.1;34-9_1384;KKA03050.1;34-9_2497;model.g3445.t1;model.g1742.t1;34-9_0428;34-9_1484;model.g4646.t1;model.g1012.t1;model.g4654.t1;KKA02671.1;34-9_3436;KKA02576.1;34-9_1822;34-9_3347;model.g3216.t1;

22 16 11 0.843 1 34 34-9_0920;model.g3855.t1;model.g3595.t1;model.g1120.t1;KKA02907.1;model.g79.t1;KKA03631.1;KKA03208.1;KKA03800.1;34-9_1734;34-9_3118;34-9_2181;model.g3720.t1;34-9_3163;model.g2463.t1;KKA02885.1;KKA03500.1;34-9_2759;model.g2850.t1;34-9_2720;34-9_0531;34-9_0741;model.g131.t1;KKA02251.1;model.g4251.t1;model.g3781.t1;model.g2497.t1;34-9_1028;KKA03238.1;KKA03901.1;KKA03597.1;34-9_0702;KKA01433.1;model.g2220.t1;

25 16 7 0.667 1 4 model.g4008.t1;34-9_0129;KKA01424.1;model.g2275.t1;

26 16 6 0.420 1 8 model.g3667.t1;KKA01305.1;KKA02878.1;KKA01892.1;34-9_3638;34-9_0338;model.g3100.t1;34-9_3171;

28 16 10 0.667 1 4 34-9_1754;model.g2836.t1;34-9_3332;KKA02266.1;

46 47 15 1.000 1 24 34-9_1120;model.g4020.t1;model.g3612.t1;KKA03149.1;KKA01428.1;34-9_2199;34-9_0923;34-9_2589;KKA03953.1;model.g1743.t1;model.g4433.t1;KKA01420.1;model.g2111.t1;34-9_3552;34-9_0137;KKA02394.1;KKA03987.1;KKA03479.1;model.g2056.t1;34-9_1085;model.g4230.t1;KKA03802.1;model.g305.t1;34-9_3673;

47 47 6 0.584 1 11 model.g3554.t1;34-9_2816;model.g729.t1;model.g3924.t1;34-9_1263;KKA02713.1;34-9_3373;34-9_0065;KKA03268.1;model.g3130.t1;KKA02293.1;

48 47 16 0.835 1 9 model.g3758.t1;KKA02808.1;model.g508.t1;34-9_3455;KKA02658.1;model.g2241.t1;34-9_1656;KKA01743.1;34-9_1559;

49 47 9 0.502 1 4 model.g1883.t1;KKA03796.1;34-9_0917;model.g3726.t1;

51 52 4 0.388 1 9 model.g3608.t1;KKA03641.1;model.g2356.t1;34-9_0752;34-9_3501;34-9_3556;KKA02398.1;KKA02337.1;model.g1988.t1;

54 55 10 1.000 1 4 KKA01299.1;34-9_3631;model.g3516.t1;model.g3647.t1;

61 62 16 1.000 1 13 34-9_0704;model.g3714.t1;KKA00998.1;34-9_1291;KKA03613.1;34-9_1290;KKA03599.1;KKA01248.1;model.g3772.t1;model.g3716.t1;KKA01246.1;34-9_0718;model.g221.t1;

82 84 21 1.000 1 4 model.g2457.t1;KKA03763.1;34-9_0881;model.g3692.t1;

83 84 7 0.553 1 41 model.g1241.t1;KKA01897.1;model.g1240.t1;34-9_3535;model.g642.t1;model.g3873.t1;KKA02085.1;KKA02434.1;34-9_0169;KKA02436.1;model.g4221.t1;34-9_0724;34-9_0170;model.g1239.t1;KKA02375.1;model.g648.t1;model.g1238.t1;KKA01898.1;model.g1237.t1;KKA02437.1;model.g643.t1;KKA02090.1;KKA02435.1;34-9_3646;KKA01312.1;34-9_0478;KKA02438.1;34-9_1222;KKA01314.1;34-9_0486;KKA02459.1;34-9_1223;34-9_1969;34-9_0168;model.g1208.t1;34-9_0485;model.g649.t1;model.g1207.t1;model.g1811.t1;model.g1242.t1;34-9_0167;

87 90 11 0.835 1 5 model.g3260.t1;KKA03579.1;34-9_2569;34-9_0681;model.g3683.t1;

92 96 76 1.000 1 4 model.g2329.t1;KKA03556.1;34-9_0657;model.g3671.t1;

93 97 13 1.000 1 6 model.g2736.t1;34-9_0687;34-9_2894;KKA03583.1;KKA01480.1;model.g3705.t1;

97 101 21 1.000 1 5 34-9_1957;KKA01563.1;KKA02467.1;model.g621.t1;model.g3693.t1;

102 106 21 1.000 1 4 KKA03557.1;34-9_0658;model.g1025.t1;model.g3672.t1;

104 108 17 1.000 1 8 34-9_1388;model.g1651.t1;model.g1650.t1;model.g1646.t1;KKA01657.1;model.g3695.t1;KKA01660.1;34-9_1383;

106 110 6 0.514 1 16 34-9_0218;model.g3698.t1;model.g389.t1;model.g1517.t1;34-9_1285;model.g2471.t1;model.g755.t1;KKA02779.1;34-9_2507;KKA03409.1;34-9_0665;34-9_2267;KKA03563.1;KKA01937.1;KKA03062.1;model.g3251.t1;

107 111 13 0.776 1 6 34-9_1702;KKA03794.1;KKA02234.1;34-9_0916;model.g495.t1;model.g3728.t1;

108 112 26 1.000 1 5 KKA02302.1;34-9_1216;34-9_3463;model.g4339.t1;model.g3740.t1;

109 112 6 0.557 1 9 34-9_3126;KKA01277.1;34-9_2872;model.g422.t1;KKA02876.1;model.g859.t1;model.g1610.t1;model.g90.t1;34-9_0274;

110 112 11 0.918 1 10 KKA01541.1;KKA03969.1;model.g2507.t1;34-9_1101;model.g2079.t1;34-9_3591;model.g1781.t1;model.g1782.t1;KKA03214.1;34-9_1519;

111 112 4 0.361 1 9 KKA01492.1;KKA03932.1;model.g1048.t1;model.g2034.t1;34-9_1063;KKA03982.1;34-9_1116;model.g2721.t1;model.g865.t1;

112 112 5 0.396 1 11 KKA01479.1;KKA03970.1;model.g2737.t1;model.g1871.t1;model.g505.t1;34-9_1102;34-9_0857;34-9_2893;model.g2080.t1;model.g2916.t1;KKA03740.1;

119 123 10 0.667 1 9 model.g3736.t1;model.g4234.t1;model.g3313.t1;KKA03482.1;KKA01338.1;34-9_3517;KKA02352.1;34-9_2196;34-9_3757;

133 137 15 0.502 1 4 KKA02356.1;34-9_3519;KKA02355.1;model.g3739.t1;

137 141 18 1.000 1 6 model.g946.t1;KKA02998.1;34-9_1882;34-9_2438;KKA02218.1;model.g3745.t1;

138 141 5 0.467 1 23 model.g525.t1;34-9_1060;KKA02228.1;model.g2745.t1;KKA03325.1;model.g2009.t1;model.g266.t1;KKA02317.1;KKA03929.1;model.g905.t1;model.g2474.t1;34-9_3479;34-9_0313;KKA02538.1;KKA01473.1;34-9_3666;KKA03156.1;34-9_3214;34-9_2350;34-9_2886;34-9_0418;model.g600.t1;model.g3365.t1;

141 145 7 0.667 1 4 34-9_0915;KKA03793.1;model.g1675.t1;model.g1975.t1;

148 152 17 1.000 1 6 34-9_3087;34-9_1570;KKA02923.1;KKA02818.1;model.g2794.t1;model.g3749.t1;

161 165 11 1.000 1 6 KKA01408.1;model.g4001.t1;model.g4343.t1;34-9_1213;KKA01763.1;34-9_0158;

174 178 6 0.557 1 6 KKA02659.1;KKA03767.1;model.g2240.t1;34-9_0885;34-9_3454;model.g3809.t1;

175 179 16 1.000 1 8 model.g410.t1;34-9_3033;34-9_3035;34-9_3034;KKA02953.1;model.g411.t1;model.g976.t1;model.g975.t1;

176 180 12 0.965 1 16 KKA01430.1;model.g3780.t1;34-9_0045;model.g1001.t1;34-9_0601;model.g2397.t1;34-9_0526;model.g2398.t1;model.g2952.t1;model.g3826.t1;KKA03598.1;34-9_3446;34-9_0703;KKA01431.1;34-9_2833;KKA02665.1;

177 181 6 0.502 1 7 model.g2963.t1;34-9_0184;34-9_1340;model.g344.t1;34-9_2065;model.g1223.t1;KKA01960.1;

184 188 11 1.000 1 4 34-9_0735;KKA03626.1;model.g853.t1;model.g3804.t1;

187 191 46 1.000 1 4 model.g3810.t1;KKA03762.1;34-9_0880;model.g3811.t1;

197 201 30 1.000 1 4 34-9_0722;KKA03596.1;34-9_0701;model.g3782.t1;

207 211 6 0.502 1 9 model.g4442.t1;model.g3483.t1;34-9_2774;34-9_3257;KKA01047.1;KKA03249.1;model.g4133.t1;KKA02512.1;34-9_2403;

217 222 6 0.490 1 16 KKA03666.1;model.g2383.t1;model.g3601.t1;model.g3821.t1;KKA03941.1;34-9_0774;model.g3828.t1;34-9_3021;KKA03280.1;model.g2041.t1;34-9_2832;34-9_0245;model.g960.t1;34-9_1073;KKA02963.1;34-9_0538;

221 226 15 1.000 1 5 34-9_1693;KKA02231.1;model.g1322.t1;model.g2936.t1;34-9_2968;

230 235 14 1.000 1 8 KKA03818.1;34-9_3195;model.g1918.t1;34-9_2274;34-9_0940;model.g3852.t1;KKA03401.1;model.g2131.t1;

231 235 14 0.875 1 19 KKA02602.1;KKA03834.1;34-9_0956;KKA02584.1;34-9_2067;34-9_3775;model.g2145.t1;34-9_2727;34-9_0093;KKA01355.1;34-9_1707;34-9_2034;model.g480.t1;model.g849.t1;model.g2962.t1;model.g1111.t1;model.g2923.t1;model.g3295.t1;KKA02103.1;

241 246 21 1.000 1 5 34-9_1015;KKA03888.1;34-9_1017;model.g3868.t1;KKA03891.1;

244 249 17 1.000 1 6 KKA01517.1;model.g4152.t1;model.g81.t1;34-9_0372;KKA03281.1;34-9_2398;

248 253 12 1.000 1 5 34-9_1016;model.g4707.t1;KKA03889.1;34-9_0348;model.g3869.t1;

250 255 5 0.502 1 7 model.g14.t1;34-9_0244;34-9_0772;KKA02753.1;model.g2380.t1;model.g3865.t1;KKA03664.1;

251 256 21 1.000 1 4 KKA03886.1;34-9_1013;model.g2154.t1;model.g3871.t1;

255 260 8 0.667 1 6 model.g3874.t1;model.g3875.t1;34-9_3496;34-9_3495;KKA02332.1;model.g3876.t1;

258 262 22 1.000 1 8 model.g1668.t1;KKA02130.1;model.g1978.t1;model.g4447.t1;KKA02345.1;34-9_2770;34-9_2693;34-9_3510;

261 266 17 1.000 1 11 34-9_3162;KKA02886.1;KKA01780.1;34-9_1156;model.g1445.t1;34-9_1189;model.g4392.t1;KKA02651.1;KKA01801.1;model.g3880.t1;model.g130.t1;

262 267 5 0.435 1 33 34-9_1865;model.g3343.t1;model.g2376.t1;model.g3881.t1;KKA01973.1;34-9_1773;KKA01998.1;KKA01637.1;34-9_2772;34-9_0131;model.g4446.t1;model.g4012.t1;34-9_3012;34-9_1326;model.g2759.t1;34-9_0768;model.g1885.t1;34-9_1225;KKA03660.1;34-9_3020;model.g1019.t1;KKA01900.1;model.g1311.t1;model.g3190.t1;model.g139.t1;KKA01206.1;model.g4373.t1;KKA02208.1;34-9_2367;KKA03248.1;model.g358.t1;34-9_2796;34-9_2601;

263 267 6 0.463 1 14 model.g1027.t1;KKA02014.1;model.g1598.t1;34-9_0822;34-9_1790;model.g3147.t1;KKA01930.1;model.g4667.t1;KKA01929.1;model.g1828.t1;34-9_3528;34-9_1269;KKA02369.1;model.g2449.t1;

276 283 44 1.000 1 6 model.g2392.t1;34-9_1830;KKA01144.1;model.g2814.t1;model.g3892.t1;34-9_3744;

279 286 11 0.773 1 25 model.g3028.t1;KKA02346.1;34-9_2909;34-9_0769;model.g1458.t1;model.g1977.t1;34-9_3281;KKA01494.1;KKA02743.1;model.g2719.t1;34-9_3511;34-9_0254;model.g2377.t1;model.g2352.t1;model.g385.t1;34-9_0635;KKA01573.1;KKA01245.1;model.g4261.t1;KKA03505.1;KKA00997.1;34-9_1289;KKA03661.1;34-9_2177;KKA01830.1;

290 297 8 0.667 1 12 34-9_1759;KKA02267.1;model.g3963.t1;KKA02342.1;34-9_1750;model.g1982.t1;34-9_1499;model.g3978.t1;KKA03202.1;KKA02264.1;model.g3908.t1;34-9_3507;

297 304 6 0.502 1 5 34-9_0048;model.g1455.t1;KKA01827.1;model.g3949.t1;34-9_3283;

300 307 4 0.420 1 7 34-9_0056;model.g2193.t1;KKA02288.1;model.g1544.t1;34-9_0992;model.g3934.t1;KKA03864.1;

301 307 52 1.000 1 8 KKA02889.1;model.g128.t1;KKA02887.1;34-9_3161;34-9_3160;model.g129.t1;34-9_3159;KKA02888.1;

311 319 11 1.000 1 6 KKA03870.1;34-9_1764;34-9_0998;KKA02272.1;model.g2200.t1;model.g3961.t1;

314 322 51 1.000 1 6 34-9_0495;model.g3981.t1;KKA02098.1;model.g3979.t1;model.g3091.t1;model.g3980.t1;

315 322 32 1.000 1 7 model.g1076.t1;34-9_2658;KKA02155.1;model.g2257.t1;model.g4360.t1;KKA02152.1;model.g2874.t1;

316 322 37 1.000 1 4 model.g4100.t1;KKA03022.1;34-9_2465;model.g4101.t1;

319 327 22 1.000 1 4 model.g3927.t1;KKA02279.1;34-9_0047;model.g3936.t1;

320 328 26 1.000 1 6 KKA03048.1;KKA02287.1;34-9_2495;34-9_0055;model.g3213.t1;model.g3935.t1;

326 335 13 1.000 1 7 KKA03780.1;KKA02294.1;34-9_0901;34-9_0066;model.g1962.t1;model.g3923.t1;model.g1298.t1;

335 344 9 0.557 1 6 34-9_0060;KKA01845.1;KKA02290.1;34-9_3265;34-9_0059;model.g3931.t1;

336 345 8 0.502 1 6 34-9_1420;model.g323.t1;model.g1709.t1;KKA01680.1;KKA01979.1;KKA01978.1;

339 348 18 1.000 1 4 KKA03434.1;34-9_2243;model.g747.t1;model.g3925.t1;

341 350 8 0.667 1 5 34-9_1237;model.g2748.t1;KKA01907.1;KKA01470.1;model.g2005.t1;

344 353 41 1.000 1 4 KKA02271.1;34-9_1763;model.g674.t1;model.g3967.t1;

347 356 23 1.000 1 6 model.g966.t1;KKA02262.1;KKA02959.1;34-9_1747;34-9_3024;model.g3975.t1;

348 357 9 0.373 1 12 KKA03991.1;KKA03990.1;34-9_1125;34-9_1123;KKA01212.1;model.g1055.t1;KKA03992.1;model.g4265.t1;model.g4059.t1;34-9_1124;model.g3986.t1;model.g4263.t1;

356 366 9 0.776 1 6 model.g1073.t1;KKA01402.1;KKA02156.1;34-9_0165;34-9_2656;model.g3994.t1;

361 371 33 1.000 1 4 KKA01426.1;34-9_0125;KKA00986.1;model.g4004.t1;

371 380 8 0.714 1 10 34-9_0975;model.g1042.t1;KKA01767.1;34-9_1204;34-9_0654;KKA03851.1;model.g2333.t1;model.g4362.t1;KKA03554.1;KKA01043.1;

378 388 18 1.000 1 5 34-9_0332;model.g4068.t1;34-9_1132;model.g3989.t1;KKA01220.1;

398 408 22 1.000 1 6 KKA02455.1;34-9_0123;34-9_1977;KKA00987.1;model.g1200.t1;model.g4042.t1;

404 415 11 0.667 1 4 KKA02568.1;model.g4039.t1;KKA02567.1;34-9_2004;

408 420 14 0.800 1 8 34-9_0780;KKA01239.1;KKA03671.1;34-9_1152;model.g2394.t1;model.g4092.t1;model.g1410.t1;34-9_2550;

414 426 14 1.000 1 4 KKA01221.1;34-9_1133;model.g462.t1;model.g4069.t1;

416 428 9 0.667 1 6 34-9_3709;34-9_1128;KKA01460.1;KKA01215.1;model.g888.t1;model.g4063.t1;

430 442 29 1.000 1 4 34-9_1131;KKA01219.1;model.g1597.t1;model.g4067.t1;

436 448 48 1.000 1 4 KKA01050.1;KKA02968.1;34-9_2406;model.g4130.t1;

438 450 20 1.000 1 4 KKA01051.1;KKA02969.1;34-9_2407;model.g4129.t1;

439 451 29 1.000 1 4 34-9_2405;KKA01049.1;KKA02967.1;model.g4131.t1;

458 470 16 1.000 1 18 KKA01703.1;model.g3245.t1;34-9_0582;KKA02133.1;34-9_0427;34-9_0908;34-9_2502;model.g2867.t1;model.g1096.t1;KKA02493.1;model.g1968.t1;model.g4534.t1;KKA03057.1;34-9_1722;34-9_3382;34-9_1273;model.g1556.t1;model.g3141.t1;

466 479 36 1.000 1 4 model.g4497.t1;model.g4498.t1;KKA03161.1;34-9_1437;

470 484 6 0.502 1 6 34-9_1265;KKA03282.1;model.g3128.t1;34-9_2397;model.g3506.t1;model.g4153.t1;

477 491 5 0.502 1 5 KKA03028.1;34-9_2471;model.g1853.t1;model.g4109.t1;34-9_0800;

478 491 17 1.000 1 6 34-9_2879;model.g2752.t1;34-9_2878;model.g2751.t1;model.g2750.t1;KKA01270.1;

479 491 11 0.945 1 9 KKA03979.1;model.g4280.t1;34-9_1113;KKA03443.1;model.g1045.t1;34-9_2235;KKA03442.1;model.g4283.t1;34-9_2236;

481 494 15 1.000 1 6 model.g3565.t1;model.g4254.t1;34-9_1160;34-9_2725;model.g1115.t1;KKA02106.1;

484 498 5 0.341 1 43 model.g844.t1;model.g4222.t1;34-9_1873;34-9_2212;model.g2981.t1;model.g4262.t1;34-9_2039;34-9_0553;model.g768.t1;34-9_2468;34-9_3534;KKA02374.1;34-9_3154;model.g1330.t1;34-9_2805;KKA00969.1;34-9_2176;model.g123.t1;KKA02874.1;model.g1616.t1;KKA02089.1;34-9_3178;model.g2421.t1;model.g1810.t1;34-9_2958;KKA01444.1;model.g4105.t1;34-9_2124;34-9_0484;model.g319.t1;model.g3099.t1;KKA03506.1;KKA02892.1;KKA03025.1;KKA03465.1;model.g2234.t1;model.g187.t1;34-9_2294;KKA02631.1;model.g2215.t1;model.g2540.t1;34-9_1008;KKA03380.1;

493 508 14 1.000 1 6 model.g3351.t1;KKA03024.1;KKA03372.1;34-9_2467;34-9_2302;model.g4103.t1;

494 509 11 1.000 1 5 model.g531.t1;KKA02827.1;34-9_1580;model.g443.t1;34-9_0308;

501 516 9 0.667 1 4 KKA01000.1;KKA01004.1;34-9_2641;model.g4180.t1;

507 522 27 1.000 1 10 KKA03075.1;34-9_2629;model.g3469.t1;34-9_2626;KKA01132.1;model.g3471.t1;model.g3470.t1;KKA01129.1;model.g4177.t1;34-9_2521;

509 524 5 0.510 1 20 34-9_2179;34-9_2418;model.g3420.t1;KKA03502.1;34-9_2975;KKA02298.1;KKA01838.1;KKA01626.1;KKA01627.1;34-9_0073;34-9_1409;model.g4225.t1;34-9_1492;model.g2092.t1;34-9_3273;model.g1694.t1;model.g3421.t1;KKA02978.1;model.g864.t1;model.g683.t1;

511 524 27 0.929 1 9 34-9_0012;KKA00977.1;model.g1943.t1;model.g1941.t1;KKA02367.1;model.g1940.t1;model.g1942.t1;34-9_1998;34-9_0353;

513 524 32 1.000 1 12 34-9_1218;KKA00967.1;model.g1745.t1;KKA00976.1;model.g4337.t1;34-9_0155;34-9_0122;model.g785.t1;model.g1058.t1;model.g4338.t1;model.g2828.t1;KKA00988.1;

517 524 9 0.816 1 12 model.g1737.t1;34-9_1650;34-9_3785;KKA01363.1;KKA01746.1;34-9_2586;KKA03135.1;model.g2595.t1;34-9_1651;KKA01747.1;model.g2593.t1;model.g2594.t1;

520 536 15 1.000 1 6 KKA01545.1;34-9_2180;34-9_3596;KKA03501.1;model.g1788.t1;model.g4250.t1;

523 539 5 0.427 1 11 model.g1954.t1;model.g309.t1;KKA02545.1;KKA02909.1;34-9_3205;34-9_0561;34-9_3115;model.g2051.t1;model.g585.t1;model.g4267.t1;KKA01690.1;

530 546 14 0.918 1 11 34-9_2241;model.g4288.t1;model.g4255.t1;34-9_2452;model.g2542.t1;KKA03319.1;model.g3277.t1;34-9_2355;model.g3368.t1;KKA03010.1;KKA03436.1;

536 552 6 0.557 1 6 34-9_1228;KKA03468.1;KKA01902.1;34-9_2210;model.g1995.t1;model.g4216.t1;

540 556 22 1.000 1 7 KKA01661.1;KKA03469.1;34-9_1391;34-9_2209;model.g1671.t1;model.g4215.t1;model.g2249.t1;

541 557 11 0.769 1 13 34-9_0827;model.g4330.t1;34-9_2146;KKA01265.1;KKA03536.1;model.g2419.t1;KKA03451.1;KKA03715.1;KKA02301.1;model.g2018.t1;34-9_3462;34-9_2226;34-9_2145;

556 572 19 1.000 1 4 KKA01624.1;model.g1316.t1;34-9_2973;model.g4231.t1;

566 582 86 1.000 1 4 model.g808.t1;KKA03462.1;34-9_2215;model.g4211.t1;

570 586 22 1.000 1 6 model.g1657.t1;34-9_3748;KKA01329.1;34-9_3766;model.g3304.t1;KKA01347.1;

572 588 18 1.000 1 5 KKA03484.1;34-9_1679;model.g2905.t1;model.g4236.t1;KKA01732.1;

574 590 16 1.000 1 6 KKA01948.1;KKA03526.1;34-9_1352;34-9_2157;model.g2600.t1;model.g4303.t1;

578 593 4 0.443 1 6 KKA01580.1;model.g2338.t1;34-9_0377;34-9_0647;KKA01521.1;model.g650.t1;

581 597 10 0.749 1 7 34-9_2842;KKA03499.1;KKA01154.1;34-9_2182;model.g1539.t1;model.g4252.t1;KKA01153.1;

587 602 12 1.000 1 6 model.g4483.t1;model.g1084.t1;model.g3334.t1;model.g1081.t1;34-9_0474;KKA02082.1;

590 606 20 1.000 1 4 34-9_1892;KKA01861.1;model.g2276.t1;model.g2523.t1;

591 607 6 0.502 1 5 34-9_3235;34-9_2190;KKA03491.1;model.g612.t1;model.g4245.t1;

602 618 7 0.667 1 4 KKA03449.1;34-9_2228;model.g1232.t1;model.g4311.t1;

603 619 33 1.000 1 4 KKA03515.1;KKA03517.1;34-9_2167;model.g4320.t1;

606 622 5 0.459 1 19 model.g4266.t1;model.g1834.t1;model.g220.t1;34-9_0535;model.g547.t1;model.g445.t1;model.g767.t1;34-9_0295;KKA02034.1;34-9_2175;model.g3598.t1;34-9_0816;34-9_2249;KKA03507.1;KKA03427.1;KKA01272.1;34-9_0116;34-9_2876;KKA03705.1;

609 625 8 0.667 1 22 model.g4314.t1;model.g85.t1;KKA02911.1;KKA01514.1;model.g3586.t1;model.g172.t1;model.g2803.t1;34-9_3112;34-9_2165;model.g2551.t1;KKA03514.1;KKA02905.1;34-9_2168;KKA01136.1;34-9_0364;34-9_3737;model.g4306.t1;KKA03519.1;34-9_3624;34-9_3122;KKA01293.1;model.g69.t1;

616 633 16 1.000 1 9 model.g4248.t1;KKA02836.1;model.g435.t1;34-9_1588;34-9_1783;34-9_2187;KKA03495.1;KKA02006.1;model.g4695.t1;

625 642 12 1.000 1 6 model.g801.t1;34-9_2331;34-9_3745;KKA01145.1;model.g2815.t1;KKA03343.1;

628 645 13 1.000 1 5 34-9_1166;model.g1460.t1;34-9_3279;model.g4372.t1;KKA01832.1;

633 652 19 1.000 1 7 34-9_3768;KKA03252.1;KKA01349.1;34-9_2781;model.g3303.t1;model.g4429.t1;model.g377.t1;

636 655 5 0.502 1 5 model.g3321.t1;34-9_3192;KKA02555.1;KKA02556.1;model.g4378.t1;

641 659 5 0.431 1 8 34-9_2881;KKA02414.1;model.g2729.t1;model.g2753.t1;model.g1386.t1;model.g1764.t1;34-9_3572;KKA01268.1;

649 668 10 0.502 1 5 34-9_2684;34-9_2768;KKA03246.1;model.g4439.t1;model.g1641.t1;

659 678 15 1.000 1 8 34-9_0933;34-9_2775;34-9_3628;model.g1808.t1;KKA03811.1;model.g4441.t1;KKA01296.1;model.g2121.t1;

660 679 17 1.000 1 4 34-9_2778;KKA01388.1;model.g2575.t1;model.g4432.t1;

662 681 10 0.714 1 10 model.g274.t1;34-9_3221;34-9_3151;KKA03772.1;model.g120.t1;34-9_0891;34-9_0617;KKA02894.1;model.g2013.t1;model.g608.t1;

668 687 35 1.000 1 6 34-9_0026;34-9_1169;34-9_1457;model.g2947.t1;KKA02647.1;model.g4411.t1;

686 706 14 1.000 1 5 34-9_2800;KKA03689.1;model.g1854.t1;model.g4380.t1;34-9_0799;

688 708 7 0.502 1 11 34-9_1064;model.g4420.t1;model.g911.t1;34-9_1743;model.g910.t1;34-9_1828;KKA03933.1;KKA02259.1;model.g2841.t1;KKA02187.1;34-9_2787;

691 711 50 1.000 1 5 34-9_2777;model.g3051.t1;34-9_0467;model.g4449.t1;KKA02075.1;

693 713 7 0.667 1 4 model.g4637.t1;KKA01335.1;model.g1632.t1;34-9_3754;

696 716 18 1.000 1 10 34-9_1728;model.g2948.t1;model.g4412.t1;KKA02247.1;model.g1152.t1;34-9_0025;KKA02648.1;34-9_1170;model.g2857.t1;model.g1763.t1;

703 723 14 1.000 1 4 34-9_1604;KKA02854.1;model.g3519.t1;model.g4346.t1;

710 730 7 0.667 1 4 KKA03298.1;34-9_2379;model.g3162.t1;model.g4467.t1;

726 745 5 0.502 1 8 34-9_1109;model.g2090.t1;KKA02575.1;34-9_0424;34-9_2015;model.g165.t1;KKA03975.1;model.g1010.t1;

737 757 54 1.000 1 5 34-9_3198;KKA02551.1;34-9_2804;model.g4485.t1;model.g2233.t1;

738 758 13 1.000 1 6 model.g800.t1;34-9_1772;34-9_2330;KKA01997.1;model.g359.t1;KKA03344.1;

740 760 17 1.000 1 5 34-9_1442;KKA01967.1;model.g335.t1;model.g4505.t1;34-9_1332;

749 768 8 0.667 1 5 KKA02719.1;model.g1590.t1;34-9_3362;KKA01287.1;model.g1515.t1;

752 772 10 0.867 1 18 model.g1052.t1;34-9_1119;KKA01651.1;KKA01682.1;34-9_1371;KKA01741.1;model.g575.t1;model.g1673.t1;34-9_1641;34-9_1663;34-9_1422;KKA01755.1;model.g2918.t1;34-9_1393;model.g2630.t1;KKA01662.1;model.g4496.t1;KKA03986.1;

755 775 23 1.000 1 5 34-9_1438;KKA02482.1;KKA03162.1;model.g2327.t1;model.g4499.t1;

757 777 10 0.706 1 20 KKA03928.1;model.g993.t1;34-9_3047;34-9_2141;KKA02945.1;model.g2149.t1;KKA03540.1;model.g992.t1;34-9_0468;model.g3453.t1;34-9_1059;KKA02943.1;34-9_1916;KKA02076.1;model.g991.t1;KKA02944.1;34-9_2672;model.g3454.t1;34-9_3046;model.g3052.t1;

780 800 11 1.000 1 4 KKA01015.1;KKA02627.1;34-9_2118;model.g4565.t1;

782 802 26 1.000 1 5 34-9_3449;34-9_3560;model.g1776.t1;KKA02403.1;model.g4576.t1;

786 805 10 0.502 1 5 34-9_1551;34-9_2685;model.g3035.t1;model.g190.t1;KKA02800.1;

788 808 25 1.000 1 4 KKA01866.1;34-9_1896;model.g2281.t1;model.g4584.t1;

790 810 20 1.000 1 6 34-9_1076;34-9_3448;KKA03944.1;KKA02663.1;model.g2052.t1;model.g4579.t1;

792 812 31 1.000 1 5 34-9_2095;model.g162.t1;KKA02201.1;model.g4570.t1;34-9_1848;

800 820 18 1.000 1 5 KKA02758.1;model.g4639.t1;34-9_0239;34-9_1817;model.g4586.t1;

801 821 18 1.000 1 4 model.g3456.t1;KKA01758.1;34-9_1639;model.g4604.t1;

817 837 25 1.000 1 6 34-9_0351;34-9_1824;KKA02948.1;model.g4703.t1;model.g4647.t1;model.g984.t1;

820 840 6 0.502 1 18 34-9_2417;34-9_1355;KKA03047.1;model.g4608.t1;34-9_2494;model.g2608.t1;34-9_0619;model.g875.t1;KKA02705.1;model.g3211.t1;KKA03196.1;model.g627.t1;34-9_1598;model.g3256.t1;model.g666.t1;KKA02977.1;model.g275.t1;model.g721.t1;

826 846 15 1.000 1 8 model.g1313.t1;34-9_2994;KKA02922.1;KKA02965.1;34-9_3014;model.g4624.t1;model.g2795.t1;34-9_3088;

838 858 28 1.000 1 5 KKA02005.1;34-9_0637;34-9_1782;model.g2350.t1;model.g4694.t1;

844 864 9 0.667 1 6 model.g3263.t1;34-9_1793;KKA03002.1;KKA02017.1;34-9_2442;model.g4670.t1;

847 867 20 1.000 1 4 KKA02019.1;34-9_1795;KKA00994.1;model.g4672.t1;

851 871 24 1.000 1 5 34-9_1806;model.g415.t1;34-9_0268;model.g4684.t1;KKA02729.1;

852 871 20 1.000 1 6 model.g2481.t1;34-9_2383;KKA03294.1;model.g3177.t1;KKA02326.1;34-9_3489;

854 874 17 1.000 1 5 KKA02011.1;34-9_2992;34-9_1787;model.g2758.t1;model.g4698.t1;

858 878 24 1.000 1 7 KKA02749.1;KKA00993.1;model.g484.t1;KKA02020.1;34-9_1796;model.g4673.t1;34-9_0249;

859 879 23 1.000 1 4 model.g1751.t1;34-9_3580;KKA01028.1;KKA01017.1;

863 884 16 1.000 1 4 model.g3587.t1;34-9_0543;KKA01440.1;model.g4674.t1;

874 895 6 0.502 1 9 model.g4710.t1;KKA02785.1;model.g402.t1;34-9_1536;KKA02776.1;KKA01887.1;34-9_0345;34-9_0221;model.g2466.t1;

875 896 17 1.000 1 6 KKA01482.1;34-9_2441;model.g2734.t1;34-9_2896;model.g3262.t1;KKA03001.1;

878 899 33 1.000 1 6 34-9_2273;KKA01601.1;KKA03402.1;34-9_3336;model.g1917.t1;model.g4739.t1;

879 900 6 0.557 1 6 KKA01013.1;KKA01599.1;KKA02626.1;34-9_3334;model.g688.t1;model.g4737.t1;

887 908 7 0.667 1 4 KKA01597.1;34-9_3328;model.g2774.t1;model.g4730.t1;

895 916 11 1.000 1 6 model.g3661.t1;KKA01603.1;34-9_3650;34-9_3338;KKA01317.1;model.g4741.t1;

901 922 14 1.000 1 8 KKA00968.1;model.g2992.t1;KKA01012.1;KKA02419.1;KKA00966.1;34-9_0001;KKA00955.1;34-9_3062;

928 949 23 0.969 1 11 model.g2564.t1;34-9_3610;KKA02792.1;34-9_1545;model.g1882.t1;34-9_0852;model.g3535.t1;KKA02299.1;KKA03738.1;34-9_0074;KKA01553.1;

942 964 8 0.667 1 5 KKA02059.1;model.g716.t1;model.g3063.t1;34-9_2105;34-9_0084;

957 979 48 1.000 1 8 KKA02081.1;KKA02040.1;model.g3021.t1;model.g3022.t1;34-9_0473;34-9_0109;model.g3023.t1;34-9_3356;

980 1002 11 1.000 1 5 KKA02431.1;34-9_0175;model.g1233.t1;34-9_0174;KKA02430.1;

991 1013 22 1.000 1 5 model.g1260.t1;model.g786.t1;KKA03085.1;34-9_0188;34-9_2533;

996 1017 18 1.000 1 5 model.g1268.t1;34-9_3716;KKA01463.1;KKA01093.1;model.g883.t1;

1012 1034 16 1.000 1 12 KKA01123.1;34-9_0212;model.g2993.t1;34-9_0213;model.g2994.t1;34-9_2635;model.g2995.t1;model.g1290.t1;KKA01052.1;KKA01053.1;model.g2996.t1;model.g209.t1;

1035 1057 7 0.667 1 4 KKA02734.1;model.g396.t1;model.g1844.t1;34-9_0263;

1037 1060 6 0.427 1 8 model.g872.t1;KKA02732.1;KKA03697.1;model.g1841.t1;KKA03193.1;34-9_0265;34-9_0808;model.g2187.t1;

1041 1064 14 1.000 1 9 34-9_0271;34-9_3078;model.g2781.t1;34-9_0969;model.g2791.t1;model.g420.t1;KKA02726.1;KKA03846.1;model.g2096.t1;

1044 1067 6 0.502 1 6 model.g1864.t1;KKA00954.1;model.g21.t1;model.g1865.t1;34-9_0273;model.g3534.t1;

1049 1072 5 0.443 1 6 model.g3155.t1;KKA01397.1;34-9_2370;model.g3073.t1;KKA03305.1;34-9_0279;

1057 1080 31 1.000 1 10 KKA02690.1;34-9_1621;KKA02867.1;34-9_3704;model.g1583.t1;model.g200.t1;model.g552.t1;model.g1733.t1;34-9_0289;34-9_3404;

1073 1096 13 1.000 1 5 model.g3009.t1;34-9_3632;model.g3646.t1;34-9_0324;KKA01300.1;

1080 1103 27 1.000 1 5 KKA01890.1;34-9_1305;model.g3103.t1;model.g3105.t1;34-9_0341;

1084 1107 45 1.000 1 6 34-9_1823;KKA00958.1;model.g1944.t1;KKA02182.1;model.g22.t1;34-9_0352;

1096 1119 28 1.000 1 4 KKA00971.1;KKA01885.1;model.g2287.t1;34-9_1914;

1098 1121 14 0.890 1 12 34-9_3091;34-9_0370;KKA03770.1;34-9_3010;34-9_0888;KKA02919.1;34-9_3180;model.g50.t1;model.g1309.t1;model.g2454.t1;model.g1618.t1;KKA02873.1;

1122 1145 55 1.000 1 5 KKA02220.1;model.g3276.t1;model.g948.t1;34-9_2451;34-9_0407;

1127 1150 12 1.000 1 5 model.g268.t1;model.g3097.t1;34-9_0488;34-9_0412;KKA02092.1;

1142 1165 25 1.000 1 4 KKA02495.1;model.g2522.t1;model.g2513.t1;34-9_0432;

1148 1171 31 1.000 1 5 KKA02500.1;model.g143.t1;34-9_1687;KKA01727.1;34-9_0441;

1151 1174 29 1.000 1 5 KKA02502.1;model.g2339.t1;model.g919.t1;34-9_0646;34-9_0447;

1156 1179 22 1.000 1 5 model.g3045.t1;34-9_3368;model.g731.t1;34-9_0452;KKA02716.1;

1181 1204 9 0.502 1 5 model.g3077.t1;34-9_2867;model.g701.t1;34-9_0483;KKA01283.1;

1191 1214 30 1.000 1 5 KKA01366.1;model.g897.t1;model.g3083.t1;34-9_3729;34-9_0500;

1222 1245 36 1.000 1 4 KKA01436.1;model.g2105.t1;model.g2107.t1;34-9_0534;

1228 1251 12 0.820 1 18 34-9_3619;KKA02110.1;model.g1119.t1;34-9_0545;KKA02212.1;KKA01558.1;34-9_0555;model.g2556.t1;model.g189.t1;KKA02829.1;model.g442.t1;model.g3582.t1;34-9_1581;KKA01441.1;34-9_2721;KKA01446.1;34-9_1871;model.g316.t1;

1239 1262 24 1.000 1 4 KKA01449.1;KKA01686.1;model.g314.t1;34-9_0557;

1240 1263 92 1.000 1 4 KKA01450.1;KKA01687.1;model.g313.t1;34-9_0558;

1242 1265 50 1.000 1 5 KKA01689.1;34-9_1940;model.g311.t1;model.g1158.t1;34-9_0560;

1243 1266 12 1.000 1 5 34-9_0629;34-9_0958;model.g2147.t1;model.g2414.t1;KKA03836.1;

1245 1268 11 1.000 1 6 model.g2971.t1;KKA01692.1;34-9_2068;model.g298.t1;KKA02603.1;34-9_0565;

1247 1270 12 0.667 1 4 KKA01693.1;model.g296.t1;model.g297.t1;34-9_0566;

1268 1291 37 1.000 1 6 model.g3530.t1;KKA01716.1;KKA02859.1;model.g1400.t1;34-9_1614;34-9_0603;

1272 1295 15 1.000 1 4 KKA01718.1;model.g1406.t1;model.g2829.t1;34-9_0608;

1273 1295 9 0.502 1 4 KKA01719.1;34-9_0609;model.g2561.t1;34-9_3613;

1275 1298 16 1.000 1 5 KKA01720.1;34-9_2060;model.g1408.t1;model.g2974.t1;34-9_0611;

1303 1326 28 1.000 1 4 KKA01042.1;KKA03553.1;model.g2334.t1;34-9_0653;

1315 1338 12 0.855 1 21 34-9_0695;model.g798.t1;KKA02169.1;model.g198.t1;model.g3126.t1;KKA03918.1;34-9_1803;34-9_2328;34-9_1049;model.g657.t1;model.g2062.t1;34-9_2810;model.g2162.t1;model.g3127.t1;KKA03346.1;KKA03589.1;34-9_1261;model.g2161.t1;KKA01394.1;model.g2163.t1;model.g797.t1;

1316 1339 24 1.000 1 4 KKA03595.1;model.g695.t1;model.g2302.t1;34-9_0700;

1353 1376 8 0.780 1 6 34-9_1413;KKA03663.1;model.g1698.t1;model.g2379.t1;KKA01674.1;34-9_0771;

1378 1401 19 1.000 1 4 KKA03693.1;model.g1849.t1;model.g3638.t1;34-9_0804;

1394 1418 24 1.000 1 6 model.g2477.t1;KKA03714.1;34-9_3482;model.g2418.t1;KKA02320.1;34-9_0826;

1401 1425 16 0.776 1 6 model.g2620.t1;KKA03720.1;34-9_1365;model.g2426.t1;KKA01025.1;34-9_0833;

1407 1431 58 1.000 1 4 KKA00974.1;KKA03728.1;model.g2430.t1;34-9_0841;

1408 1432 8 0.835 1 7 KKA02861.1;KKA00975.1;model.g3532.t1;KKA03729.1;model.g2438.t1;34-9_0842;34-9_1616;

1427 1451 22 1.000 1 4 KKA03742.1;model.g1875.t1;model.g1874.t1;34-9_0860;

1431 1455 13 1.000 1 4 KKA03081.1;model.g3152.t1;model.g3154.t1;34-9_2529;

1450 1474 53 1.000 1 4 KKA03771.1;model.g2015.t1;model.g3511.t1;34-9_0889;

1467 1491 6 0.498 1 9 34-9_0909;34-9_2614;KKA01194.1;model.g3493.t1;KKA03947.1;model.g1969.t1;KKA03787.1;34-9_1079;model.g2048.t1;

1481 1506 34 1.000 1 6 34-9_2137;KKA03806.1;KKA03545.1;model.g2116.t1;model.g739.t1;34-9_0928;

1504 1529 39 1.000 1 13 model.g559.t1;model.g558.t1;model.g557.t1;34-9_0953;model.g1914.t1;model.g560.t1;model.g562.t1;KKA03831.1;KKA03830.1;model.g563.t1;model.g561.t1;34-9_0952;model.g564.t1;

1506 1531 15 1.000 1 5 KKA03832.1;model.g1699.t1;model.g2143.t1;34-9_1414;34-9_0954;

1518 1543 22 1.000 1 6 KKA02587.1;KKA03845.1;34-9_2044;model.g3111.t1;model.g1561.t1;34-9_0968;

1530 1555 12 1.000 1 6 model.g2199.t1;KKA03858.1;KKA03869.1;model.g2184.t1;34-9_0997;34-9_0985;

1549 1575 7 0.557 1 6 KKA03923.1;KKA03880.1;34-9_1054;model.g1029.t1;model.g2156.t1;34-9_1006;

1569 1595 9 0.667 1 4 KKA03915.1;model.g2170.t1;34-9_1044;34-9_1043;

1598 1624 15 1.000 1 8 34-9_2795;KKA03951.1;34-9_1286;model.g2054.t1;model.g388.t1;34-9_1083;KKA03263.1;model.g631.t1;

1611 1637 31 1.000 1 5 KKA03968.1;model.g586.t1;model.g2078.t1;KKA02544.1;34-9_1100;

1629 1656 13 1.000 1 5 model.g2227.t1;34-9_1307;model.g369.t1;34-9_1157;KKA01263.1;

1634 1661 37 1.000 1 5 KKA01792.1;model.g638.t1;model.g715.t1;34-9_1174;model.g3437.t1;

1637 1664 36 1.000 1 6 model.g1450.t1;KKA01790.1;KKA01824.1;model.g633.t1;34-9_3287;34-9_1177;

1642 1669 29 1.000 1 6 KKA03321.1;KKA01785.1;34-9_2354;model.g3556.t1;model.g3374.t1;34-9_1183;

1643 1670 39 1.000 1 5 KKA01784.1;model.g617.t1;34-9_1186;KKA01783.1;34-9_1185;

1668 1695 4 0.388 1 9 34-9_1247;KKA02311.1;model.g228.t1;KKA02308.1;KKA02310.1;model.g2026.t1;KKA01916.1;KKA02309.1;model.g395.t1;

1694 1721 20 0.749 1 5 KKA02891.1;34-9_3157;model.g126.t1;34-9_1281;model.g3134.t1;

1698 1725 25 1.000 1 6 model.g3335.t1;KKA00995.1;model.g2830.t1;KKA01243.1;model.g387.t1;34-9_1287;

1699 1726 16 1.000 1 4 KKA00996.1;KKA01244.1;model.g386.t1;34-9_1288;

1701 1728 45 1.000 1 4 KKA01250.1;model.g383.t1;model.g382.t1;34-9_1293;

1711 1738 11 0.776 1 6 KKA03078.1;KKA01260.1;34-9_2525;model.g3107.t1;model.g3200.t1;34-9_1303;

1717 1744 14 1.000 1 6 model.g416.t1;KKA01991.1;model.g1896.t1;KKA01993.1;34-9_1310;KKA01992.1;

1765 1792 27 1.000 1 5 KKA01022.1;KKA01669.1;model.g2793.t1;34-9_1368;34-9_1404;

1803 1831 44 1.000 1 6 KKA02610.1;KKA01683.1;34-9_2083;model.g2656.t1;model.g829.t1;34-9_1423;

1827 1855 19 0.502 1 4 KKA03175.1;KKA03176.1;model.g2672.t1;34-9_1462;

1828 1855 16 1.000 1 4 34-9_1569;model.g2671.t1;KKA02817.1;model.g447.t1;

1836 1864 15 1.000 1 8 model.g427.t1;model.g2691.t1;model.g93.t1;34-9_3129;34-9_3485;34-9_1470;KKA02904.1;KKA02322.1;

1848 1876 47 1.000 1 5 model.g869.t1;34-9_2291;model.g1900.t1;KKA03383.1;34-9_1487;

1865 1893 23 1.000 1 4 KKA03217.1;model.g1303.t1;model.g1304.t1;34-9_1523;

1891 1919 5 0.502 1 5 KKA02822.1;34-9_1709;model.g452.t1;model.g2927.t1;34-9_1574;

1896 1924 16 1.000 1 8 KKA01454.1;KKA02831.1;34-9_3696;model.g440.t1;model.g2703.t1;34-9_1583;model.g2451.t1;34-9_3530;

1899 1927 12 0.835 1 5 KKA02834.1;34-9_3247;model.g437.t1;model.g2524.t1;34-9_1586;

1934 1963 9 0.667 1 4 KKA01754.1;KKA01753.1;model.g3460.t1;34-9_1643;

1956 1986 8 0.702 1 7 model.g892.t1;KKA01737.1;model.g2749.t1;KKA01266.1;model.g2891.t1;34-9_1670;34-9_3724;

1976 2007 29 1.000 1 5 model.g502.t1;model.g2519.t1;KKA02517.1;34-9_1697;34-9_3251;

1994 2025 21 1.000 1 7 KKA01348.1;KKA02243.1;34-9_3767;model.g2872.t1;model.g3311.t1;34-9_1719;model.g2871.t1;

2054 2085 33 1.000 1 7 34-9_3381;model.g913.t1;model.g665.t1;KKA02453.1;34-9_1980;34-9_1851;KKA02706.1;

2062 2092 14 0.502 1 4 model.g2310.t1;KKA01869.1;34-9_1897;KKA01867.1;

2064 2094 29 1.000 1 4 model.g3202.t1;model.g3203.t1;KKA03041.1;34-9_2486;

2070 2101 22 1.000 1 5 KKA02216.1;34-9_3615;model.g943.t1;model.g2559.t1;34-9_1879;

2086 2117 19 1.000 1 7 KKA01554.1;KKA01878.1;34-9_3612;model.g2298.t1;model.g2297.t1;34-9_1907;model.g2563.t1;

2088 2119 8 0.502 1 11 KKA01617.1;34-9_1909;model.g271.t1;model.g164.t1;model.g163.t1;34-9_3236;34-9_3354;model.g2300.t1;KKA01880.1;34-9_3237;KKA02526.1;

2098 2129 67 1.000 1 4 KKA02487.1;model.g2316.t1;model.g543.t1;34-9_1923;

2102 2133 12 1.000 1 4 KKA02486.1;model.g2320.t1;model.g899.t1;34-9_1927;

2107 2138 6 0.502 1 4 model.g1166.t1;KKA01477.1;model.g2741.t1;34-9_1934;

2171 2202 79 1.000 1 4 KKA02583.1;model.g851.t1;model.g857.t1;34-9_2032;

2211 2242 27 1.000 1 6 34-9_2874;KKA02611.1;KKA01275.1;model.g2969.t1;model.g1666.t1;34-9_2087;

2247 2279 7 0.502 1 5 KKA03498.1;model.g367.t1;KKA03497.1;34-9_2184;34-9_2183;

2259 2291 11 0.776 1 6 model.g781.t1;KKA03433.1;34-9_2596;model.g748.t1;KKA01210.1;34-9_2244;

2268 2300 12 0.875 1 10 model.g1928.t1;34-9_3434;34-9_3217;KKA03382.1;model.g604.t1;34-9_3138;34-9_2292;KKA02672.1;model.g107.t1;model.g1494.t1;

2276 2308 15 1.000 1 6 34-9_2898;KKA03411.1;KKA01484.1;model.g745.t1;model.g2732.t1;34-9_2265;

2288 2320 22 1.000 1 4 34-9_2353;KKA03322.1;model.g1907.t1;model.g1908.t1;

2313 2347 34 1.000 1 4 KKA03360.1;model.g807.t1;model.g3148.t1;34-9_2315;

2421 2457 35 1.000 1 4 KKA03063.1;model.g3252.t1;model.g3253.t1;34-9_2508;

2435 2471 13 1.000 1 4 KKA01195.1;model.g3494.t1;model.g256.t1;34-9_2613;

2436 2473 16 1.000 1 6 KKA00951.1;KKA03077.1;KKA01837.1;model.g3229.t1;model.g682.t1;34-9_2524;

2458 2495 18 1.000 1 4 KKA03118.1;KKA03117.1;model.g3408.t1;34-9_2565;

2483 2520 7 0.667 1 7 34-9_3581;KKA01207.1;KKA01029.1;model.g3344.t1;KKA01018.1;34-9_2600;model.g1752.t1;

2524 2561 13 1.000 1 5 model.g3447.t1;KKA02113.1;model.g1123.t1;34-9_2667;34-9_2717;

2580 2617 17 1.000 1 4 KKA03239.1;model.g2219.t1;model.g928.t1;34-9_2760;

2601 2638 6 0.502 1 4 KKA03271.1;34-9_3616;model.g2558.t1;34-9_2818;

2669 2707 36 1.000 1 7 KKA01081.1;34-9_2921;KKA01082.1;34-9_2922;KKA01506.1;model.g1378.t1;model.g1377.t1;

2679 2717 33 1.000 1 4 KKA01070.1;model.g1363.t1;KKA01095.1;34-9_2932;

2683 2722 14 0.502 1 4 KKA01181.1;model.g1356.t1;model.g1355.t1;34-9_2937;

2716 2756 30 1.000 1 5 KKA01636.1;34-9_3695;model.g2705.t1;34-9_2993;model.g2704.t1;

2733 2773 6 0.502 1 4 KKA02957.1;model.g969.t1;34-9_3028;34-9_3027;

2737 2777 5 0.443 1 6 model.g1621.t1;KKA02954.1;34-9_3183;model.g974.t1;KKA02563.1;34-9_3032;

2768 2808 24 1.000 1 5 KKA02920.1;KKA01616.1;model.g2797.t1;model.g1427.t1;34-9_3090;

2778 2818 84 1.000 1 4 KKA01472.1;KKA02913.1;model.g2746.t1;34-9_3108;

2782 2822 7 0.667 1 5 KKA02910.1;model.g2250.t1;model.g71.t1;34-9_3345;34-9_3114;

2820 2860 9 0.667 1 4 KKA02560.1;KKA02559.1;model.g3557.t1;34-9_3187;

2832 2872 15 0.502 1 4 KKA02539.1;KKA02540.1;model.g598.t1;34-9_3213;

2863 2905 17 1.000 1 6 model.g2808.t1;KKA01843.1;34-9_3739;model.g2254.t1;KKA01138.1;34-9_3267;

2909 2952 16 1.000 1 5 KKA02724.1;KKA03056.1;model.g836.t1;model.g3242.t1;34-9_3358;

2937 2981 13 0.502 1 4 KKA02694.1;KKA02695.1;model.g673.t1;34-9_3400;

2938 2982 20 1.000 1 5 KKA02692.1;model.g668.t1;KKA02693.1;model.g669.t1;34-9_3401;

3008 3052 9 0.667 1 4 KKA02362.1;KKA02360.1;model.g2443.t1;34-9_3522;

3098 3144 21 1.000 1 4 KKA01010.1;KKA01452.1;model.g2707.t1;34-9_3693;

3099 3145 40 1.000 1 4 KKA01453.1;KKA01011.1;model.g2706.t1;34-9_3694;

**Single-copy**

1 0 13 1.000 1 3 KKA02400.1;34-9_3558;model.g3606.t1;

2 1 13 1.000 1 3 34-9_3637;KKA01304.1;model.g3640.t1;

3 2 48 1.000 1 3 KKA01318.1;34-9_3651;model.g3627.t1;

6 5 19 1.000 1 3 34-9_3630;KKA01298.1;model.g3648.t1;

10 9 76 1.000 1 3 34-9_2504;KKA03059.1;model.g3248.t1;

11 10 73 1.000 1 3 KKA02759.1;34-9_0238;model.g4587.t1;

13 12 57 1.000 1 3 34-9_1002;model.g1033.t1;KKA03876.1;

14 13 65 1.000 1 3 34-9_3563;model.g1773.t1;KKA02406.1;

19 16 25 1.000 1 3 model.g1234.t1;34-9_0173;KKA02432.1;

20 16 24 1.000 1 3 model.g3426.t1;KKA02988.1;34-9_2429;

21 16 51 1.000 1 3 KKA01102.1;model.g2587.t1;34-9_1625;

23 16 7 0.502 1 3 model.g2980.t1;34-9_2111;KKA02625.1;

24 16 35 1.000 1 3 model.g414.t1;KKA02730.1;34-9_0267;

27 16 23 1.000 1 3 model.g1269.t1;34-9_0194;KKA01092.1;

29 16 36 1.000 1 3 34-9_3524;KKA02364.1;model.g2440.t1;

31 16 43 1.000 1 3 model.g3049.t1;34-9_0466;KKA02074.1;

33 34 28 1.000 1 3 KKA01307.1;34-9_3640;model.g3665.t1;

34 35 37 1.000 1 3 KKA02391.1;34-9_3549;model.g3616.t1;

36 37 27 1.000 1 3 34-9_3546;KKA02387.1;model.g3620.t1;

37 38 31 1.000 1 3 34-9_3539;KKA02380.1;model.g3650.t1;

38 39 39 1.000 1 3 KKA01310.1;34-9_3643;model.g3662.t1;

41 42 36 1.000 1 3 KKA01301.1;34-9_3633;model.g3645.t1;

42 43 21 1.000 1 3 KKA01313.1;34-9_3645;model.g3628.t1;

44 45 40 1.000 1 3 KKA02386.1;34-9_3545;model.g3657.t1;

45 46 10 0.502 1 3 KKA00956.1;34-9_0013;model.g3659.t1;

50 51 44 1.000 1 3 34-9_1096;model.g2074.t1;KKA03965.1;

52 53 6 0.502 1 3 KKA01324.1;34-9_3656;model.g3633.t1;

55 55 29 1.000 1 3 34-9_1394;model.g1674.t1;KKA01663.1;

57 58 28 1.000 1 3 34-9_3547;KKA02388.1;model.g3619.t1;

58 59 20 1.000 1 3 34-9_3081;model.g2784.t1;KKA02925.1;

60 61 58 1.000 1 3 KKA01303.1;34-9_3636;model.g3643.t1;

62 64 6 0.502 1 3 KKA01316.1;34-9_3648;model.g3630.t1;

63 65 45 1.000 1 3 34-9_3629;KKA01297.1;model.g3649.t1;

64 66 56 1.000 1 3 34-9_3653;KKA01320.1;model.g3623.t1;

65 67 45 1.000 1 3 34-9_3542;KKA02383.1;model.g3651.t1;

66 67 26 1.000 1 3 KKA01855.1;model.g2266.t1;34-9_1885;

67 69 27 1.000 1 3 KKA02395.1;34-9_3553;model.g3611.t1;

68 70 51 1.000 1 3 KKA02396.1;34-9_3554;model.g3610.t1;

69 71 38 1.000 1 3 KKA02397.1;34-9_3555;model.g3609.t1;

72 74 18 1.000 1 3 34-9_0840;model.g2429.t1;KKA03727.1;

74 76 49 1.000 1 3 34-9_0692;KKA03587.1;model.g3710.t1;

75 77 54 1.000 1 3 34-9_0777;model.g2388.t1;KKA03668.1;

76 78 27 1.000 1 3 KKA03678.1;model.g1880.t1;34-9_0787;

77 79 23 1.000 1 3 34-9_0689;KKA03585.1;model.g3706.t1;

78 80 35 1.000 1 3 KKA03577.1;34-9_0679;model.g3699.t1;

79 81 38 1.000 1 3 KKA03560.1;34-9_0662;model.g3676.t1;

81 83 45 1.000 1 3 KKA03568.1;34-9_0669;model.g3686.t1;

84 87 22 1.000 1 3 KKA03574.1;34-9_0675;model.g3681.t1;

85 88 29 1.000 1 3 KKA03576.1;34-9_0678;model.g3685.t1;

86 89 18 1.000 1 3 34-9_0667;KKA03565.1;model.g3696.t1;

90 94 75 1.000 1 3 KKA03564.1;34-9_0666;model.g3697.t1;

91 95 22 1.000 1 3 KKA03571.1;34-9_0672;model.g3691.t1;

94 97 29 1.000 1 3 model.g4209.t1;KKA03531.1;34-9_2152;

95 99 25 1.000 1 3 34-9_2861;KKA01290.1;model.g1516.t1;

96 100 15 1.000 1 3 34-9_0664;KKA03562.1;model.g3678.t1;

98 102 33 1.000 1 3 34-9_0677;KKA03575.1;model.g3684.t1;

99 103 38 1.000 1 3 34-9_0636;KKA01574.1;model.g2351.t1;

100 104 17 1.000 1 3 KKA03578.1;34-9_0680;model.g3700.t1;

101 105 32 1.000 1 3 KKA03561.1;34-9_0663;model.g3677.t1;

113 112 23 1.000 1 3 model.g1078.t1;34-9_2657;KKA02153.1;

114 112 83 1.000 1 3 KKA03745.1;34-9_0863;model.g2399.t1;

115 112 72 1.000 1 3 model.g2253.t1;34-9_3268;KKA01842.1;

116 112 33 1.000 1 3 KKA01493.1;34-9_2908;model.g2720.t1;

118 112 26 1.000 1 3 model.g1611.t1;KKA02875.1;34-9_3176;

121 125 25 1.000 1 3 KKA03145.1;model.g1721.t1;34-9_3679;

123 126 37 1.000 1 3 34-9_2955;KKA01167.1;model.g1327.t1;

125 129 38 1.000 1 3 34-9_3513;KKA02348.1;model.g3731.t1;

127 131 15 1.000 1 3 KKA02349.1;34-9_3514;model.g3732.t1;

129 133 56 1.000 1 3 KKA01850.1;34-9_3260;model.g3718.t1;

130 134 53 1.000 1 3 KKA02353.1;34-9_3518;model.g3737.t1;

131 135 25 1.000 1 3 KKA02351.1;34-9_3516;model.g3733.t1;

132 136 33 1.000 1 3 34-9_1356;model.g2609.t1;KKA01945.1;

134 138 33 1.000 1 3 34-9_2437;KKA02997.1;model.g3744.t1;

135 139 78 1.000 1 3 KKA03000.1;34-9_2440;model.g3747.t1;

136 140 26 1.000 1 3 KKA02995.1;34-9_2435;model.g3742.t1;

139 141 27 1.000 1 3 KKA02914.1;34-9_3104;model.g34.t1;

142 145 18 1.000 1 3 34-9_1345;model.g349.t1;KKA01955.1;

144 148 84 1.000 1 3 KKA02996.1;34-9_2436;model.g3743.t1;

145 149 27 1.000 1 3 KKA02999.1;34-9_2439;model.g3746.t1;

150 154 39 1.000 1 3 KKA02802.1;34-9_1552;model.g3765.t1;

151 155 41 1.000 1 3 34-9_2840;model.g2950.t1;KKA01151.1;

153 157 21 1.000 1 3 KKA02819.1;34-9_1571;model.g3748.t1;

154 158 31 1.000 1 3 KKA02804.1;34-9_1554;model.g3763.t1;

155 159 20 1.000 1 3 34-9_1560;KKA02809.1;model.g3757.t1;

156 160 31 1.000 1 3 KKA02812.1;34-9_1563;model.g3754.t1;

157 161 30 1.000 1 3 34-9_1561;KKA02810.1;model.g3756.t1;

159 163 36 1.000 1 3 34-9_1562;KKA02811.1;model.g3755.t1;

160 164 85 1.000 1 3 KKA02806.1;34-9_1557;model.g3760.t1;

162 166 45 1.000 1 3 KKA03154.1;model.g3140.t1;34-9_3668;

163 167 87 1.000 1 3 KKA02813.1;34-9_1564;model.g3753.t1;

164 168 35 1.000 1 3 KKA02805.1;34-9_1556;model.g3761.t1;

165 169 51 1.000 1 3 KKA02807.1;34-9_1558;model.g3759.t1;

166 170 23 1.000 1 3 34-9_3073;model.g2775.t1;KKA02928.1;

167 171 32 1.000 1 3 KKA03622.1;34-9_0730;model.g3798.t1;

169 173 30 1.000 1 3 34-9_0696;KKA03590.1;model.g3787.t1;

170 174 5 0.502 1 3 34-9_0707;KKA03602.1;model.g3773.t1;

171 175 37 1.000 1 3 34-9_0706;KKA03601.1;model.g3774.t1;

172 176 43 1.000 1 3 KKA03629.1;34-9_0738;model.g3807.t1;

173 177 22 1.000 1 3 KKA03593.1;34-9_0698;model.g3783.t1;

178 182 39 1.000 1 3 KKA03617.1;34-9_0723;model.g3790.t1;

181 185 10 1.000 1 3 34-9_3759;KKA01340.1;model.g1629.t1;

183 187 25 1.000 1 3 34-9_0732;KKA03624.1;model.g3800.t1;

185 189 66 1.000 1 3 KKA03620.1;34-9_0728;model.g3795.t1;

186 190 11 1.000 1 3 KKA03625.1;34-9_0733;model.g3802.t1;

190 193 33 1.000 1 3 34-9_2574;model.g3415.t1;KKA03123.1;

191 195 50 1.000 1 3 34-9_0709;KKA03604.1;model.g3771.t1;

192 196 19 1.000 1 3 KKA03600.1;34-9_0705;model.g3775.t1;

193 197 29 1.000 1 3 KKA03616.1;34-9_0721;model.g3789.t1;

194 198 24 1.000 1 3 KKA03592.1;34-9_0697;model.g3786.t1;

196 200 14 1.000 1 3 34-9_0720;KKA03615.1;model.g3788.t1;

198 202 32 1.000 1 3 KKA03619.1;34-9_0727;model.g3794.t1;

199 203 40 1.000 1 3 KKA01982.1;model.g1887.t1;34-9_1320;

200 204 17 1.000 1 3 34-9_1912;model.g2288.t1;KKA01883.1;

202 206 22 1.000 1 3 KKA03621.1;34-9_0729;model.g3796.t1;

203 207 14 1.000 1 3 KKA02292.1;34-9_0064;model.g3926.t1;

205 209 27 1.000 1 3 34-9_1012;KKA03885.1;model.g3816.t1;

206 209 28 1.000 1 3 34-9_0540;model.g3602.t1;KKA01438.1;

209 213 35 1.000 1 3 KKA03882.1;34-9_1009;model.g3813.t1;

210 214 52 1.000 1 3 KKA03884.1;34-9_1011;model.g3815.t1;

211 215 34 1.000 1 3 KKA02751.1;34-9_0247;model.g3819.t1;

212 216 19 1.000 1 3 34-9_0246;KKA02752.1;model.g3820.t1;

214 218 53 1.000 1 3 34-9_0243;KKA02754.1;model.g3825.t1;

215 219 50 1.000 1 3 34-9_1632;KKA01112.1;model.g3817.t1;

216 221 37 1.000 1 3 34-9_0242;KKA02755.1;model.g3824.t1;

220 225 41 1.000 1 3 KKA03279.1;34-9_2830;model.g3830.t1;

223 228 93 1.000 1 3 KKA02553.1;34-9_3194;model.g3851.t1;

225 230 22 1.000 1 3 34-9_2829;KKA03278.1;model.g3831.t1;

229 234 26 1.000 1 3 34-9_3689;KKA01041.1;model.g3845.t1;

233 235 59 1.000 1 3 KKA02975.1;34-9_2415;model.g4121.t1;

235 235 63 1.000 1 3 model.g793.t1;KKA03349.1;34-9_2325;

236 241 19 1.000 1 3 KKA03159.1;34-9_3662;model.g3840.t1;

237 242 35 1.000 1 3 34-9_3193;KKA02554.1;model.g3848.t1;

238 243 51 1.000 1 3 model.g4472.t1;34-9_0101;KKA02047.1;

242 247 28 1.000 1 3 34-9_1026;KKA03899.1;model.g3857.t1;

243 248 19 1.000 1 3 34-9_0393;KKA01531.1;model.g456.t1;

245 250 32 1.000 1 3 KKA03892.1;34-9_1018;model.g3866.t1;

246 251 17 1.000 1 3 34-9_1024;KKA03898.1;model.g3859.t1;

247 252 35 1.000 1 3 KKA03896.1;34-9_1022;model.g3861.t1;

249 254 10 1.000 1 3 34-9_1023;KKA03897.1;model.g3860.t1;

252 257 22 1.000 1 3 34-9_1021;KKA03895.1;model.g3862.t1;

253 258 33 1.000 1 3 34-9_1014;KKA03887.1;model.g3870.t1;

254 259 25 1.000 1 3 KKA01899.1;34-9_1224;model.g3882.t1;

257 262 36 1.000 1 3 KKA02329.1;34-9_3492;model.g3878.t1;

260 265 20 1.000 1 3 KKA02328.1;34-9_3491;model.g3877.t1;

264 267 58 1.000 1 3 34-9_2846;model.g1535.t1;KKA01156.1;

265 267 7 0.502 1 3 KKA01928.1;model.g4716.t1;34-9_1268;

266 267 24 1.000 1 3 KKA02255.1;34-9_1739;model.g2845.t1;

267 274 42 1.000 1 3 34-9_0437;KKA02497.1;model.g3886.t1;

268 275 12 1.000 1 3 34-9_3232;KKA02528.1;model.g3897.t1;

272 279 59 1.000 1 3 KKA02191.1;34-9_1835;model.g3893.t1;

275 282 59 1.000 1 3 KKA02210.1;34-9_1868;model.g3899.t1;

278 285 17 1.000 1 3 34-9_2469;KKA03026.1;model.g4107.t1;

282 289 40 1.000 1 3 KKA03203.1;34-9_1501;model.g3910.t1;

283 290 27 1.000 1 3 KKA03204.1;34-9_1502;model.g3911.t1;

284 291 46 1.000 1 3 model.g4298.t1;34-9_2217;KKA03460.1;

287 294 11 1.000 1 3 34-9_1495;KKA03199.1;model.g3904.t1;

288 295 33 1.000 1 3 KKA03205.1;34-9_1504;model.g3913.t1;

289 295 43 1.000 1 3 KKA03373.1;model.g3353.t1;34-9_2300;

291 298 33 1.000 1 3 KKA03200.1;34-9_1497;model.g3906.t1;

292 299 21 1.000 1 3 KKA02277.1;34-9_0041;model.g3962.t1;

294 301 27 1.000 1 3 KKA02280.1;34-9_0049;model.g3946.t1;

295 302 28 1.000 1 3 34-9_0743;KKA03633.1;model.g3982.t1;

298 305 35 1.000 1 3 34-9_0053;KKA02286.1;model.g3938.t1;

303 310 36 1.000 1 3 KKA01125.1;34-9_2633;model.g3919.t1;

305 312 31 1.000 1 3 KKA01630.1;34-9_2983;model.g4056.t1;

308 314 31 1.000 1 3 model.g4272.t1;KKA03509.1;34-9_2172;

309 316 21 1.000 1 3 KKA03076.1;model.g3224.t1;34-9_2523;

318 322 28 1.000 1 3 34-9_0497;model.g3088.t1;KKA00982.1;

321 329 23 1.000 1 3 KKA02274.1;34-9_1766;model.g3958.t1;

322 330 22 1.000 1 3 34-9_0613;KKA01721.1;model.g1411.t1;

323 331 33 1.000 1 3 KKA02283.1;34-9_0051;model.g3941.t1;

324 333 22 1.000 1 3 34-9_0058;KKA02289.1;model.g3932.t1;

331 340 47 1.000 1 3 KKA02270.1;34-9_1762;model.g3966.t1;

332 341 37 1.000 1 3 KKA02263.1;34-9_1748;model.g3976.t1;

334 343 31 1.000 1 3 KKA02296.1;34-9_0069;model.g3920.t1;

337 346 80 1.000 1 3 KKA02295.1;34-9_0067;model.g3922.t1;

350 360 28 1.000 1 3 KKA01406.1;34-9_0161;model.g3998.t1;

352 362 43 1.000 1 3 KKA01422.1;34-9_0132;model.g4013.t1;

355 365 82 1.000 1 3 KKA01410.1;34-9_0156;model.g4003.t1;

359 369 62 1.000 1 3 34-9_3582;model.g1754.t1;KKA01535.1;

360 370 34 1.000 1 3 KKA01404.1;34-9_0163;model.g3996.t1;

362 372 68 1.000 1 3 KKA01416.1;34-9_0144;model.g4027.t1;

363 373 30 1.000 1 3 KKA01418.1;34-9_0140;model.g4023.t1;

365 375 10 1.000 1 3 34-9_3102;KKA02915.1;model.g39.t1;

368 378 43 1.000 1 3 KKA01421.1;34-9_0133;model.g4014.t1;

372 382 39 1.000 1 3 KKA01407.1;34-9_0159;model.g4000.t1;

373 383 6 0.502 1 3 KKA01409.1;34-9_0157;model.g4002.t1;

377 387 16 1.000 1 3 KKA01405.1;34-9_0162;model.g3999.t1;

380 390 17 1.000 1 3 KKA01403.1;34-9_0164;model.g3995.t1;

384 394 32 1.000 1 3 34-9_0128;KKA01425.1;model.g4007.t1;

387 397 30 1.000 1 3 34-9_2012;KKA02574.1;model.g4049.t1;

390 400 24 1.000 1 3 34-9_2001;KKA02564.1;model.g4035.t1;

391 401 15 1.000 1 3 KKA02573.1;34-9_2011;model.g4048.t1;

394 404 23 1.000 1 3 KKA02571.1;34-9_2008;model.g4045.t1;

395 405 35 1.000 1 3 KKA02569.1;34-9_2005;model.g4041.t1;

396 406 68 1.000 1 3 KKA01629.1;34-9_2982;model.g4055.t1;

397 407 72 1.000 1 3 KKA01628.1;34-9_2980;model.g4053.t1;

400 410 33 1.000 1 3 34-9_2865;model.g704.t1;KKA01285.1;

405 417 16 1.000 1 3 34-9_0070;KKA02297.1;model.g4052.t1;

406 418 67 1.000 1 3 KKA02565.1;34-9_2002;model.g4034.t1;

407 419 48 1.000 1 3 KKA01231.1;34-9_1143;model.g4083.t1;

409 421 30 1.000 1 3 34-9_1140;KKA01228.1;model.g4080.t1;

410 422 26 1.000 1 3 KKA01229.1;34-9_1141;model.g4081.t1;

412 424 56 1.000 1 3 34-9_1130;KKA01218.1;model.g4066.t1;

415 427 49 1.000 1 3 KKA01233.1;34-9_1145;model.g4086.t1;

418 430 27 1.000 1 3 34-9_0189;model.g1261.t1;KKA01101.1;

419 431 53 1.000 1 3 34-9_1135;KKA01223.1;model.g4074.t1;

420 432 26 1.000 1 3 KKA03206.1;34-9_1507;model.g4070.t1;

421 433 18 1.000 1 3 34-9_1126;KKA01213.1;model.g4060.t1;

422 434 12 0.502 1 3 34-9_1134;KKA01222.1;model.g4071.t1;

423 435 29 1.000 1 3 34-9_1137;KKA01225.1;model.g4077.t1;

424 436 27 1.000 1 3 34-9_1146;KKA01234.1;model.g4087.t1;

426 438 34 1.000 1 3 KKA01230.1;34-9_1142;model.g4082.t1;

428 440 21 1.000 1 3 34-9_1139;KKA01227.1;model.g4079.t1;

431 443 11 1.000 1 3 34-9_1147;KKA01235.1;model.g4088.t1;

432 444 75 1.000 1 3 KKA01224.1;34-9_1136;model.g4076.t1;

433 445 51 1.000 1 3 34-9_1129;KKA01217.1;model.g4065.t1;

434 446 77 1.000 1 3 KKA01237.1;34-9_1149;model.g4089.t1;

435 447 13 1.000 1 3 34-9_2553;KKA03106.1;model.g4168.t1;

437 449 33 1.000 1 3 KKA03110.1;34-9_2557;model.g4164.t1;

441 453 13 1.000 1 3 34-9_2408;KKA02970.1;model.g4128.t1;

442 454 74 1.000 1 3 KKA02971.1;34-9_2409;model.g4126.t1;

443 455 15 1.000 1 3 34-9_2474;KKA03031.1;model.g4112.t1;

445 457 24 1.000 1 3 34-9_2547;KKA03100.1;model.g4147.t1;

447 459 15 1.000 1 3 34-9_2541;KKA03094.1;model.g4139.t1;

449 461 41 1.000 1 3 KKA03029.1;34-9_2472;model.g4110.t1;

450 462 58 1.000 1 3 KKA03283.1;34-9_2394;model.g4156.t1;

451 463 30 1.000 1 3 KKA03108.1;34-9_2555;model.g4166.t1;

452 464 17 1.000 1 3 KKA03021.1;34-9_2462;model.g4097.t1;

453 465 43 1.000 1 3 34-9_2404;KKA01048.1;model.g4132.t1;

454 466 78 1.000 1 3 KKA03112.1;34-9_2560;model.g4162.t1;

455 467 11 1.000 1 3 KKA03020.1;34-9_2461;model.g4096.t1;

457 469 15 1.000 1 3 KKA03092.1;34-9_2539;model.g4151.t1;

459 470 44 1.000 1 3 model.g1097.t1;KKA02132.1;34-9_2690;

462 475 74 1.000 1 3 KKA03098.1;34-9_2545;model.g4149.t1;

463 476 19 1.000 1 3 KKA03286.1;34-9_2390;model.g4146.t1;

464 477 64 1.000 1 3 34-9_2412;KKA02973.1;model.g4118.t1;

465 478 53 1.000 1 3 KKA03284.1;34-9_2393;model.g4157.t1;

468 482 44 1.000 1 3 34-9_2401;KKA01045.1;model.g4136.t1;

471 485 53 1.000 1 3 KKA03027.1;34-9_2470;model.g4108.t1;

473 487 16 1.000 1 3 34-9_2476;KKA03033.1;model.g4114.t1;

474 488 75 1.000 1 3 34-9_2556;KKA03109.1;model.g4165.t1;

475 489 57 1.000 1 3 KKA03107.1;34-9_2554;model.g4167.t1;

476 490 16 1.000 1 3 KKA01046.1;34-9_2402;model.g4137.t1;

480 494 27 1.000 1 3 model.g4218.t1;KKA03466.1;34-9_2211;

482 496 31 1.000 1 3 34-9_2561;KKA03113.1;model.g4161.t1;

483 497 52 1.000 1 3 34-9_2473;KKA03030.1;model.g4111.t1;

485 498 33 1.000 1 3 KKA02045.1;model.g4474.t1;34-9_0103;

486 498 37 1.000 1 3 model.g1661.t1;KKA01333.1;34-9_3752;

488 498 44 1.000 1 3 34-9_3240;KKA02522.1;model.g2541.t1;

490 498 31 1.000 1 3 KKA03037.1;model.g3232.t1;34-9_2480;

492 507 56 1.000 1 3 34-9_2475;KKA03032.1;model.g4113.t1;

495 510 9 0.502 1 3 34-9_2552;KKA03105.1;model.g4170.t1;

496 511 24 1.000 1 3 34-9_2286;KKA03389.1;model.g4173.t1;

497 512 26 1.000 1 3 KKA03390.1;34-9_2285;model.g4174.t1;

498 513 28 1.000 1 3 KKA03391.1;34-9_2284;model.g4175.t1;

499 514 68 1.000 1 3 34-9_2631;KKA01127.1;model.g4178.t1;

502 517 39 1.000 1 3 34-9_2640;KKA01007.1;model.g4183.t1;

505 520 34 1.000 1 3 KKA01122.1;34-9_2636;model.g4190.t1;

506 521 12 1.000 1 3 KKA01119.1;34-9_2639;model.g4185.t1;

508 523 61 1.000 1 3 34-9_3764;model.g3305.t1;KKA01345.1;

510 524 37 1.000 1 3 KKA03747.1;34-9_0865;model.g2401.t1;

512 524 38 1.000 1 3 34-9_3526;model.g2447.t1;KKA02366.1;

519 535 57 1.000 1 3 KKA01120.1;34-9_2638;model.g4186.t1;

521 537 34 1.000 1 3 KKA03529.1;34-9_2154;model.g4204.t1;

522 538 17 1.000 1 3 KKA03549.1;34-9_2133;model.g4195.t1;

524 540 51 1.000 1 3 34-9_2227;KKA03450.1;model.g4329.t1;

525 541 40 1.000 1 3 34-9_2229;KKA03448.1;model.g4312.t1;

526 542 22 1.000 1 3 34-9_2149;KKA03533.1;model.g4328.t1;

527 543 67 1.000 1 3 KKA03473.1;34-9_2205;model.g4223.t1;

528 544 56 1.000 1 3 KKA01114.1;34-9_1634;model.g4198.t1;

529 545 32 1.000 1 3 KKA03457.1;34-9_2220;model.g4290.t1;

532 548 42 1.000 1 3 34-9_2163;KKA03521.1;model.g4315.t1;

533 549 18 1.000 1 3 34-9_2262;KKA03414.1;model.g4335.t1;

534 550 13 1.000 1 3 KKA03520.1;34-9_2164;model.g4318.t1;

535 551 22 1.000 1 3 34-9_2197;KKA03481.1;model.g4233.t1;

538 554 61 1.000 1 3 34-9_3490;KKA02327.1;model.g2480.t1;

543 559 27 1.000 1 3 KKA03524.1;34-9_2160;model.g4324.t1;

544 560 24 1.000 1 3 34-9_1889;model.g2270.t1;KKA01858.1;

545 561 29 1.000 1 3 34-9_2143;KKA03538.1;model.g4201.t1;

546 562 29 1.000 1 3 34-9_2225;KKA03452.1;model.g4331.t1;

548 564 30 1.000 1 3 34-9_2201;KKA03477.1;model.g4228.t1;

549 565 13 1.000 1 3 34-9_2343;model.g3390.t1;KKA03330.1;

551 567 22 1.000 1 3 34-9_2192;KKA03488.1;model.g4240.t1;

552 568 32 1.000 1 3 KKA03530.1;34-9_2153;model.g4205.t1;

553 569 12 1.000 1 3 34-9_2147;KKA03535.1;model.g4326.t1;

554 570 54 1.000 1 3 KKA03471.1;34-9_2207;model.g4213.t1;

558 574 44 1.000 1 3 KKA03395.1;model.g1911.t1;34-9_2279;

559 575 50 1.000 1 3 34-9_2221;KKA03456.1;model.g4291.t1;

561 577 16 1.000 1 3 34-9_2610;KKA01198.1;model.g4242.t1;

563 579 41 1.000 1 3 34-9_2132;KKA03550.1;model.g4197.t1;

564 580 28 1.000 1 3 KKA03511.1;34-9_2171;model.g4270.t1;

565 580 16 1.000 1 3 34-9_0005;model.g573.t1;KKA01039.1;

567 583 50 1.000 1 3 KKA03416.1;34-9_2260;model.g4333.t1;

569 585 22 1.000 1 3 KKA03463.1;34-9_2214;model.g4219.t1;

571 587 38 1.000 1 3 34-9_0753;KKA03644.1;model.g2358.t1;

573 588 24 1.000 1 3 34-9_1232;KKA01904.1;model.g1999.t1;

575 591 26 1.000 1 3 KKA03476.1;34-9_2202;model.g4227.t1;

576 592 50 1.000 1 3 KKA03464.1;34-9_2213;model.g4220.t1;

577 593 30 1.000 1 3 34-9_2189;KKA03493.1;model.g4246.t1;

580 596 26 1.000 1 3 34-9_2261;KKA03415.1;model.g4334.t1;

582 598 48 1.000 1 3 KKA03478.1;34-9_2200;model.g4229.t1;

583 598 89 1.000 1 3 KKA03809.1;model.g2119.t1;34-9_0931;

584 600 15 1.000 1 3 KKA03455.1;34-9_2222;model.g4292.t1;

585 601 40 1.000 1 3 34-9_2174;KKA03508.1;model.g4274.t1;

588 604 67 1.000 1 3 KKA03534.1;34-9_2148;model.g4325.t1;

589 605 75 1.000 1 3 KKA03480.1;34-9_2198;model.g4232.t1;

592 607 50 1.000 1 3 KKA01852.1;34-9_1883;model.g2261.t1;

594 610 44 1.000 1 3 KKA03537.1;34-9_2144;model.g4202.t1;

595 611 43 1.000 1 3 34-9_2203;KKA03475.1;model.g4226.t1;

596 612 18 1.000 1 3 KKA03513.1;34-9_2169;model.g4275.t1;

597 613 13 1.000 1 3 KKA03437.1;34-9_2240;model.g4289.t1;

598 614 33 1.000 1 3 KKA03539.1;34-9_2142;model.g4200.t1;

599 615 5 0.502 1 3 KKA03523.1;34-9_2161;model.g4322.t1;

600 616 13 1.000 1 3 KKA03528.1;34-9_2155;model.g4203.t1;

601 617 11 1.000 1 3 KKA03525.1;34-9_2158;model.g4307.t1;

605 621 11 1.000 1 3 KKA03445.1;34-9_2233;model.g4278.t1;

607 623 24 1.000 1 3 model.g4575.t1;34-9_3450;KKA02662.1;

608 624 30 1.000 1 3 34-9_2206;KKA03472.1;model.g4212.t1;

610 626 20 1.000 1 3 KKA03856.1;model.g2180.t1;34-9_0983;

611 627 17 1.000 1 3 KKA03527.1;34-9_2156;model.g4304.t1;

612 628 44 1.000 1 3 34-9_2186;KKA03496.1;model.g4249.t1;

613 629 59 1.000 1 3 KKA03439.1;34-9_2238;model.g4285.t1;

614 630 16 1.000 1 3 KKA02870.1;34-9_1623;model.g4296.t1;

615 631 48 1.000 1 3 KKA03532.1;34-9_2151;model.g4210.t1;

619 636 33 1.000 1 3 KKA03444.1;34-9_2234;model.g4279.t1;

620 637 15 1.000 1 3 34-9_2178;KKA03504.1;model.g4260.t1;

621 638 20 1.000 1 3 KKA03474.1;34-9_2204;model.g4224.t1;

622 639 12 1.000 1 3 KKA03483.1;34-9_2195;model.g4235.t1;

623 640 44 1.000 1 3 KKA03458.1;34-9_2219;model.g4301.t1;

624 641 63 1.000 1 3 KKA03494.1;34-9_2188;model.g4247.t1;

626 643 46 1.000 1 3 34-9_2741;KKA03223.1;model.g4400.t1;

630 648 21 1.000 1 3 KKA03264.1;34-9_2799;model.g4379.t1;

631 649 26 1.000 1 3 34-9_0228;KKA02769.1;model.g4593.t1;

640 659 40 1.000 1 3 KKA01798.1;34-9_1161;model.g4383.t1;

642 659 48 1.000 1 3 34-9_0237;KKA02760.1;model.g4588.t1;

644 663 40 1.000 1 3 34-9_3234;KKA02527.1;model.g4395.t1;

647 666 58 1.000 1 3 KKA03244.1;34-9_2766;model.g4436.t1;

648 667 18 1.000 1 3 KKA03255.1;34-9_2786;model.g4422.t1;

654 673 30 1.000 1 3 34-9_2822;KKA03274.1;model.g4417.t1;

655 674 84 1.000 1 3 KKA01764.1;34-9_1212;model.g4345.t1;

657 676 31 1.000 1 3 KKA01768.1;34-9_1203;model.g4358.t1;

664 683 20 1.000 1 3 34-9_1193;KKA01776.1;model.g4368.t1;

667 686 26 1.000 1 3 KKA03222.1;34-9_2739;model.g4398.t1;

675 695 32 1.000 1 3 34-9_1171;KKA01793.1;model.g4413.t1;

676 696 12 0.502 1 3 34-9_2742;KKA03224.1;model.g4401.t1;

677 697 14 1.000 1 3 KKA01775.1;34-9_1195;model.g4371.t1;

678 698 19 1.000 1 3 KKA03253.1;34-9_2782;model.g4428.t1;

679 699 19 1.000 1 3 34-9_2764;KKA03243.1;model.g4434.t1;

680 700 81 1.000 1 3 34-9_2785;KKA03254.1;model.g4423.t1;

681 701 77 1.000 1 3 KKA03245.1;34-9_2767;model.g4437.t1;

682 702 20 1.000 1 3 34-9_0223;KKA02774.1;model.g4351.t1;

683 703 23 1.000 1 3 KKA01765.1;34-9_1206;model.g4347.t1;

684 704 45 1.000 1 3 34-9_1198;KKA01772.1;model.g4350.t1;

685 705 15 1.000 1 3 34-9_2748;KKA03228.1;model.g4408.t1;

694 714 35 1.000 1 3 KKA03628.1;34-9_0737;model.g4421.t1;

695 715 13 1.000 1 3 34-9_2745;KKA03226.1;model.g4405.t1;

704 724 35 1.000 1 3 KKA01461.1;model.g1727.t1;34-9_3712;

705 725 33 1.000 1 3 34-9_3004;KKA01647.1;model.g1022.t1;

706 726 12 1.000 1 3 KKA03241.1;34-9_2763;model.g4450.t1;

707 727 39 1.000 1 3 KKA02043.1;34-9_0105;model.g4476.t1;

708 728 20 1.000 1 3 34-9_3464;KKA02303.1;model.g4462.t1;

709 729 13 1.000 1 3 KKA02305.1;34-9_3467;model.g4454.t1;

712 732 10 1.000 1 3 KKA02086.1;34-9_0479;model.g4460.t1;

713 733 42 1.000 1 3 34-9_0097;KKA02050.1;model.g4480.t1;

715 735 10 1.000 1 3 34-9_0104;KKA02044.1;model.g4475.t1;

717 737 29 1.000 1 3 34-9_3465;KKA02304.1;model.g4461.t1;

720 740 28 1.000 1 3 KKA01524.1;34-9_0380;model.g4464.t1;

722 742 43 1.000 1 3 KKA01522.1;34-9_0378;model.g4466.t1;

723 743 38 1.000 1 3 KKA02739.1;34-9_0258;model.g4470.t1;

724 744 16 1.000 1 3 34-9_0100;KKA02048.1;model.g4477.t1;

725 745 22 1.000 1 3 34-9_0257;KKA02740.1;model.g4471.t1;

728 748 8 0.502 1 3 34-9_3699;KKA01457.1;model.g4456.t1;

730 750 38 1.000 1 3 34-9_3231;model.g137.t1;KKA02529.1;

731 751 24 1.000 1 3 KKA01523.1;34-9_0379;model.g4465.t1;

732 752 28 1.000 1 3 KKA02546.1;34-9_3204;model.g4494.t1;

733 753 9 0.502 1 3 KKA02547.1;34-9_3202;model.g4489.t1;

739 759 18 1.000 1 3 KKA02550.1;34-9_3199;model.g4486.t1;

744 764 45 1.000 1 3 KKA03164.1;34-9_1441;model.g4504.t1;

745 765 33 1.000 1 3 34-9_1452;KKA03170.1;model.g4511.t1;

746 766 53 1.000 1 3 KKA03167.1;34-9_1445;model.g4508.t1;

748 768 51 1.000 1 3 34-9_1439;KKA03163.1;model.g4502.t1;

751 771 24 1.000 1 3 KKA03169.1;34-9_1450;model.g4509.t1;

754 774 32 1.000 1 3 KKA03165.1;34-9_1443;model.g4506.t1;

756 775 17 1.000 1 3 34-9_1933;KKA02483.1;model.g2326.t1;

758 778 23 1.000 1 3 KKA01709.1;34-9_0590;model.g4521.t1;

759 779 28 1.000 1 3 KKA01713.1;34-9_0594;model.g4517.t1;

761 781 37 1.000 1 3 KKA01706.1;34-9_0588;model.g4526.t1;

763 783 35 1.000 1 3 34-9_1481;KKA03188.1;model.g2681.t1;

765 785 17 1.000 1 3 KKA01711.1;34-9_0592;model.g4515.t1;

767 787 27 1.000 1 3 34-9_1229;KKA01903.1;model.g1996.t1;

768 788 75 1.000 1 3 KKA01700.1;34-9_0575;model.g4536.t1;

771 791 41 1.000 1 3 KKA03231.1;34-9_2751;model.g4548.t1;

773 793 28 1.000 1 3 KKA01794.1;34-9_1168;model.g4546.t1;

775 795 11 1.000 1 3 KKA03234.1;34-9_2753;model.g4550.t1;

776 796 13 1.000 1 3 34-9_1164;KKA01796.1;model.g4543.t1;

778 798 23 1.000 1 3 KKA01840.1;34-9_3270;model.g4560.t1;

779 799 43 1.000 1 3 KKA02660.1;34-9_3453;model.g4580.t1;

781 801 61 1.000 1 3 34-9_3451;KKA02661.1;model.g4578.t1;

784 802 24 1.000 1 3 model.g981.t1;34-9_3039;KKA02950.1;

787 807 18 1.000 1 3 KKA02585.1;34-9_2042;model.g4577.t1;

789 809 43 1.000 1 3 34-9_3342;KKA01609.1;model.g4556.t1;

794 814 23 1.000 1 3 KKA02617.1;34-9_2093;model.g4568.t1;

795 815 68 1.000 1 3 KKA02767.1;34-9_0230;model.g4595.t1;

796 816 14 1.000 1 3 KKA02772.1;34-9_0226;model.g4601.t1;

797 817 70 1.000 1 3 34-9_0236;KKA02761.1;model.g4589.t1;

798 818 17 1.000 1 3 KKA02895.1;model.g119.t1;34-9_3150;

799 819 36 1.000 1 3 34-9_0229;KKA02768.1;model.g4594.t1;

803 823 48 1.000 1 3 KKA02757.1;34-9_0240;model.g4585.t1;

804 824 54 1.000 1 3 KKA02771.1;34-9_0227;model.g4602.t1;

807 827 15 1.000 1 3 34-9_0234;KKA02763.1;model.g4592.t1;

812 832 34 1.000 1 3 KKA02313.1;34-9_3475;model.g4618.t1;

813 833 57 1.000 1 3 34-9_1593;KKA02843.1;model.g4616.t1;

814 834 16 1.000 1 3 KKA02846.1;34-9_1595;model.g4612.t1;

816 836 30 1.000 1 3 KKA02847.1;34-9_1596;model.g4611.t1;

821 840 31 1.000 1 3 model.g201.t1;KKA02865.1;34-9_1619;

823 843 24 1.000 1 3 KKA02737.1;34-9_0260;model.g4622.t1;

829 849 40 1.000 1 3 KKA01644.1;34-9_3000;model.g4631.t1;

831 851 48 1.000 1 3 KKA01639.1;34-9_2995;model.g4625.t1;

832 852 46 1.000 1 3 34-9_3057;KKA02938.1;model.g4635.t1;

833 853 26 1.000 1 3 KKA01645.1;34-9_3001;model.g4632.t1;

834 854 68 1.000 1 3 34-9_1776;KKA02001.1;model.g4688.t1;

839 859 50 1.000 1 3 KKA02009.1;34-9_1786;model.g4664.t1;

840 860 8 0.502 1 3 34-9_1777;KKA02002.1;model.g4689.t1;

841 861 27 1.000 1 3 KKA02165.1;34-9_1799;model.g4677.t1;

843 863 36 1.000 1 3 KKA02003.1;34-9_1778;model.g4690.t1;

845 865 30 1.000 1 3 34-9_1818;KKA02179.1;model.g4640.t1;

846 866 12 1.000 1 3 34-9_3703;KKA01458.1;model.g1734.t1;

848 868 52 1.000 1 3 34-9_1804;KKA02170.1;model.g4682.t1;

849 869 84 1.000 1 3 KKA02016.1;34-9_1792;model.g4669.t1;

853 873 55 1.000 1 3 KKA02171.1;34-9_1805;model.g4683.t1;

856 876 58 1.000 1 3 34-9_1794;KKA02018.1;model.g4671.t1;

857 877 62 1.000 1 3 34-9_1295;KKA01252.1;model.g380.t1;

860 881 7 0.502 1 3 34-9_1780;KKA02004.1;model.g4692.t1;

861 882 83 1.000 1 3 KKA02181.1;34-9_1820;model.g4644.t1;

862 883 48 1.000 1 3 KKA02013.1;34-9_1789;model.g4666.t1;

864 885 29 1.000 1 3 KKA02168.1;34-9_1802;model.g4681.t1;

866 887 7 0.502 1 3 34-9_1813;KKA02176.1;model.g4662.t1;

868 889 34 1.000 1 3 34-9_1788;KKA02012.1;model.g4687.t1;

869 890 79 1.000 1 3 KKA02174.1;34-9_1809;model.g4657.t1;

870 891 13 1.000 1 3 34-9_1785;KKA02008.1;model.g4665.t1;

876 897 32 1.000 1 3 34-9_0809;KKA03698.1;model.g1840.t1;

880 901 10 1.000 1 3 KKA01598.1;34-9_3331;model.g4724.t1;

883 904 21 1.000 1 3 34-9_3322;KKA01592.1;model.g4721.t1;

885 906 12 1.000 1 3 34-9_3321;KKA01591.1;model.g4720.t1;

889 910 80 1.000 1 3 KKA01600.1;34-9_3335;model.g4736.t1;

892 913 45 1.000 1 3 34-9_3337;KKA01602.1;model.g4740.t1;

894 915 6 0.502 1 3 KKA01606.1;34-9_3340;model.g4745.t1;

897 918 37 1.000 1 3 34-9_3326;KKA01595.1;model.g4727.t1;

905 926 36 1.000 1 3 KKA02649.1;model.g812.t1;34-9_0023;

908 929 54 1.000 1 3 KKA02613.1;model.g2955.t1;34-9_2089;

909 930 12 1.000 1 3 KKA02646.1;model.g2945.t1;34-9_0028;

910 931 29 1.000 1 3 KKA02645.1;model.g2944.t1;34-9_0029;

911 932 39 1.000 1 3 KKA02644.1;model.g2943.t1;34-9_0030;

913 934 82 1.000 1 3 KKA02643.1;model.g692.t1;34-9_0032;

914 935 69 1.000 1 3 34-9_2420;KKA02980.1;model.g3430.t1;

915 936 20 1.000 1 3 KKA02642.1;model.g1194.t1;34-9_0033;

922 943 26 1.000 1 3 KKA02637.1;model.g2988.t1;34-9_0040;

933 955 48 1.000 1 3 KKA02068.1;model.g3043.t1;34-9_0076;

936 958 56 1.000 1 3 KKA02065.1;model.g3058.t1;34-9_0079;

938 960 27 1.000 1 3 KKA02064.1;model.g3059.t1;34-9_0080;

940 962 16 0.502 1 3 KKA02060.1;model.g3061.t1;34-9_0082;

943 965 51 1.000 1 3 KKA02058.1;model.g3064.t1;34-9_0085;

945 967 23 1.000 1 3 KKA02056.1;model.g3067.t1;34-9_0087;

948 970 21 1.000 1 3 KKA02054.1;model.g492.t1;34-9_0091;

951 973 54 1.000 1 3 KKA02051.1;model.g429.t1;34-9_0095;

958 980 50 1.000 1 3 KKA02039.1;model.g3037.t1;34-9_0110;

959 981 30 1.000 1 3 KKA02038.1;model.g3038.t1;34-9_0111;

961 983 30 1.000 1 3 KKA02036.1;model.g3033.t1;34-9_0113;

962 984 36 1.000 1 3 34-9_2712;KKA02118.1;model.g1132.t1;

964 986 64 1.000 1 3 KKA02035.1;model.g3025.t1;34-9_0115;

965 986 33 1.000 1 3 KKA03584.1;model.g89.t1;34-9_0688;

968 990 34 1.000 1 3 KKA02031.1;model.g478.t1;34-9_0120;

974 996 29 1.000 1 3 KKA01415.1;model.g2820.t1;34-9_0149;

975 997 49 1.000 1 3 KKA01414.1;model.g2821.t1;34-9_0150;

976 998 25 1.000 1 3 KKA01413.1;model.g2822.t1;34-9_0151;

978 1000 29 1.000 1 3 KKA01412.1;model.g2824.t1;34-9_0153;

979 1001 17 1.000 1 3 KKA02433.1;model.g1235.t1;34-9_0171;

981 1003 86 1.000 1 3 KKA02429.1;model.g1231.t1;34-9_0176;

983 1005 13 1.000 1 3 KKA02427.1;model.g1228.t1;34-9_0179;

987 1009 37 1.000 1 3 34-9_3498;KKA02334.1;model.g1993.t1;

988 1010 41 1.000 1 3 KKA02422.1;model.g1222.t1;34-9_0185;

989 1011 37 1.000 1 3 KKA02421.1;model.g1221.t1;34-9_0186;

993 1015 36 1.000 1 3 KKA01099.1;model.g1262.t1;34-9_0191;

994 1016 8 0.502 1 3 KKA01096.1;model.g1265.t1;34-9_0192;

995 1017 31 1.000 1 3 KKA01094.1;model.g1267.t1;34-9_0193;

997 1019 39 1.000 1 3 KKA01090.1;model.g1272.t1;34-9_0196;

998 1020 36 1.000 1 3 KKA01088.1;model.g1274.t1;34-9_0197;

999 1021 35 1.000 1 3 KKA01087.1;model.g1275.t1;34-9_0198;

1000 1022 64 1.000 1 3 KKA01086.1;model.g1276.t1;34-9_0199;

1004 1026 57 1.000 1 3 KKA01060.1;model.g1280.t1;34-9_0203;

1008 1030 71 1.000 1 3 KKA01057.1;model.g1286.t1;34-9_0208;

1011 1033 58 1.000 1 3 KKA01054.1;model.g1289.t1;34-9_0211;

1014 1036 66 1.000 1 3 KKA02782.1;model.g398.t1;34-9_0215;

1015 1037 16 1.000 1 3 KKA02781.1;model.g399.t1;34-9_0216;

1017 1039 34 1.000 1 3 KKA02778.1;model.g2470.t1;34-9_0219;

1018 1040 70 1.000 1 3 KKA02777.1;model.g2468.t1;34-9_0220;

1024 1046 30 1.000 1 3 34-9_0810;KKA03699.1;model.g1839.t1;

1028 1050 51 1.000 1 3 KKA02747.1;model.g487.t1;34-9_0251;

1030 1052 51 1.000 1 3 KKA02742.1;model.g3029.t1;34-9_0255;

1031 1053 82 1.000 1 3 KKA02741.1;model.g3030.t1;34-9_0256;

1032 1054 47 1.000 1 3 KKA02736.1;model.g408.t1;34-9_0261;

1033 1054 30 1.000 1 3 KKA02683.1;model.g1472.t1;34-9_3414;

1034 1056 82 1.000 1 3 KKA02735.1;model.g407.t1;34-9_0262;

1036 1058 42 1.000 1 3 KKA02733.1;model.g409.t1;34-9_0264;

1038 1061 12 1.000 1 3 KKA02731.1;model.g412.t1;34-9_0266;

1039 1062 58 1.000 1 3 KKA02728.1;model.g417.t1;34-9_0269;

1040 1063 52 1.000 1 3 KKA02727.1;model.g419.t1;34-9_0270;

1043 1066 64 1.000 1 3 KKA02725.1;model.g421.t1;34-9_0272;

1045 1068 19 1.000 1 3 KKA01401.1;model.g423.t1;34-9_0275;

1046 1069 18 1.000 1 3 KKA01400.1;model.g424.t1;34-9_0276;

1047 1070 25 1.000 1 3 KKA01399.1;model.g3070.t1;34-9_0277;

1048 1071 42 1.000 1 3 KKA01398.1;model.g3071.t1;34-9_0278;

1078 1101 33 1.000 1 3 KKA01891.1;model.g3101.t1;34-9_0339;

1081 1104 12 1.000 1 3 KKA01889.1;model.g1946.t1;34-9_0342;

1082 1105 14 1.000 1 3 KKA01888.1;model.g1945.t1;34-9_0343;

1086 1109 14 1.000 1 3 model.g1939.t1;KKA03104.1;34-9_0356;

1088 1111 20 1.000 1 3 KKA01508.1;model.g1935.t1;34-9_0357;

1089 1112 43 1.000 1 3 KKA01509.1;model.g1934.t1;34-9_0358;

1090 1113 20 1.000 1 3 KKA01510.1;model.g1933.t1;34-9_0359;

1091 1114 78 1.000 1 3 KKA01511.1;model.g177.t1;34-9_0360;

1093 1116 14 1.000 1 3 KKA01512.1;model.g175.t1;34-9_0362;

1094 1117 74 1.000 1 3 KKA01513.1;model.g173.t1;34-9_0363;

1099 1122 16 1.000 1 3 KKA01516.1;model.g656.t1;34-9_0371;

1102 1125 67 1.000 1 3 KKA01525.1;model.g2011.t1;34-9_0383;

1103 1126 13 1.000 1 3 KKA01526.1;model.g1950.t1;34-9_0384;

1104 1127 32 1.000 1 3 KKA01527.1;model.g231.t1;34-9_0386;

1105 1128 59 1.000 1 3 KKA01528.1;model.g232.t1;34-9_0387;

1107 1130 56 1.000 1 3 34-9_0973;model.g2093.t1;KKA03850.1;

1110 1133 33 1.000 1 3 KKA01530.1;model.g454.t1;34-9_0391;

1111 1134 15 1.000 1 3 KKA01532.1;model.g460.t1;34-9_0395;

1113 1136 23 1.000 1 3 KKA01533.1;model.g465.t1;34-9_0398;

1115 1138 55 1.000 1 3 KKA02023.1;model.g467.t1;34-9_0400;

1116 1139 27 1.000 1 3 KKA02024.1;model.g468.t1;34-9_0401;

1117 1140 38 1.000 1 3 KKA02025.1;model.g469.t1;34-9_0402;

1118 1141 39 1.000 1 3 KKA02026.1;model.g470.t1;34-9_0403;

1119 1142 21 1.000 1 3 KKA02027.1;model.g471.t1;34-9_0404;

1123 1146 71 1.000 1 3 KKA02221.1;model.g949.t1;34-9_0408;

1125 1148 44 1.000 1 3 KKA02222.1;model.g951.t1;34-9_0410;

1126 1149 17 1.000 1 3 KKA02223.1;model.g952.t1;34-9_0411;

1128 1151 37 1.000 1 3 KKA02224.1;model.g269.t1;34-9_0413;

1130 1153 46 1.000 1 3 KKA02226.1;model.g260.t1;34-9_0415;

1131 1154 44 1.000 1 3 KKA02227.1;model.g264.t1;34-9_0416;

1133 1156 57 1.000 1 3 KKA02229.1;model.g2536.t1;34-9_0420;

1137 1160 11 1.000 1 3 KKA02492.1;model.g170.t1;34-9_0425;

1146 1169 29 1.000 1 3 KKA02499.1;model.g147.t1;34-9_0439;

1155 1178 58 1.000 1 3 KKA02505.1;model.g921.t1;34-9_0451;

1159 1182 89 1.000 1 3 KKA02508.1;model.g930.t1;34-9_0456;

1163 1186 6 0.502 1 3 KKA02509.1;model.g252.t1;34-9_0460;

1167 1190 30 1.000 1 3 KKA02072.1;model.g2961.t1;34-9_0464;

1170 1193 65 1.000 1 3 34-9_0874;KKA03756.1;model.g1387.t1;

1172 1195 19 1.000 1 3 KKA02078.1;model.g3054.t1;34-9_0470;

1175 1198 26 1.000 1 3 KKA02080.1;model.g3057.t1;34-9_0472;

1177 1200 25 1.000 1 3 KKA02083.1;model.g430.t1;34-9_0476;

1178 1201 17 1.000 1 3 KKA02084.1;model.g431.t1;34-9_0477;

1179 1202 34 1.000 1 3 KKA02087.1;model.g3074.t1;34-9_0481;

1180 1203 24 1.000 1 3 KKA02088.1;model.g3076.t1;34-9_0482;

1183 1206 56 1.000 1 3 KKA02093.1;model.g3098.t1;34-9_0489;

1184 1207 34 1.000 1 3 KKA02094.1;model.g3094.t1;34-9_0490;

1185 1208 38 1.000 1 3 KKA02095.1;model.g3093.t1;34-9_0491;

1186 1209 34 1.000 1 3 KKA02096.1;model.g3092.t1;34-9_0493;

1187 1210 23 1.000 1 3 KKA02097.1;model.g3090.t1;34-9_0494;

1188 1211 40 1.000 1 3 KKA00981.1;model.g3089.t1;34-9_0496;

1189 1212 37 1.000 1 3 KKA00984.1;model.g3085.t1;34-9_0498;

1190 1213 13 1.000 1 3 KKA00985.1;model.g3084.t1;34-9_0499;

1192 1215 39 1.000 1 3 KKA01367.1;model.g3082.t1;34-9_0501;

1193 1216 40 1.000 1 3 KKA01368.1;model.g3081.t1;34-9_0502;

1194 1217 17 1.000 1 3 KKA01369.1;model.g3080.t1;34-9_0503;

1195 1217 39 1.000 1 3 KKA01576.1;model.g2348.t1;34-9_0639;

1196 1219 76 1.000 1 3 KKA01370.1;model.g3504.t1;34-9_0504;

1197 1220 46 1.000 1 3 KKA01371.1;model.g3505.t1;34-9_0505;

1198 1221 10 1.000 1 3 KKA01372.1;model.g3508.t1;34-9_0507;

1200 1223 64 1.000 1 3 KKA01374.1;model.g3510.t1;34-9_0509;

1201 1224 21 1.000 1 3 KKA01375.1;model.g3512.t1;34-9_0510;

1202 1225 33 1.000 1 3 KKA01376.1;model.g3513.t1;34-9_0511;

1211 1234 33 1.000 1 3 KKA01386.1;model.g3574.t1;34-9_0520;

1214 1237 39 1.000 1 3 KKA01391.1;model.g2572.t1;34-9_0523;

1216 1239 17 1.000 1 3 KKA01393.1;model.g2569.t1;34-9_0525;

1217 1240 22 1.000 1 3 KKA01432.1;model.g3590.t1;34-9_0528;

1220 1243 55 1.000 1 3 KKA01434.1;model.g3596.t1;34-9_0532;

1221 1244 36 1.000 1 3 KKA01435.1;model.g3597.t1;34-9_0533;

1226 1249 29 1.000 1 3 KKA01439.1;model.g3588.t1;34-9_0542;

1230 1253 63 1.000 1 3 34-9_2990;KKA01635.1;model.g2756.t1;

1231 1254 29 1.000 1 3 KKA02926.1;model.g2782.t1;34-9_3079;

1234 1257 18 1.000 1 3 34-9_1578;KKA02825.1;model.g488.t1;

1235 1258 12 1.000 1 3 KKA01442.1;model.g321.t1;34-9_0551;

1241 1264 20 1.000 1 3 KKA01688.1;model.g312.t1;34-9_0559;

1244 1267 25 1.000 1 3 KKA01691.1;model.g300.t1;34-9_0564;

1246 1268 55 1.000 1 3 KKA03753.1;model.g2407.t1;34-9_0871;

1250 1273 42 1.000 1 3 KKA01697.1;model.g292.t1;34-9_0570;

1251 1274 24 1.000 1 3 KKA01698.1;model.g291.t1;34-9_0571;

1253 1276 18 1.000 1 3 KKA02791.1;model.g199.t1;34-9_1544;

1257 1280 24 1.000 1 3 KKA01702.1;model.g285.t1;34-9_0579;

1267 1290 51 1.000 1 3 KKA01715.1;model.g1399.t1;34-9_0602;

1270 1293 79 1.000 1 3 KKA01717.1;model.g1402.t1;34-9_0605;

1276 1299 12 1.000 1 3 34-9_2498;KKA03051.1;model.g3215.t1;

1278 1301 16 1.000 1 3 KKA01723.1;model.g272.t1;34-9_0615;

1282 1305 15 1.000 1 3 34-9_3486;model.g426.t1;KKA02323.1;

1285 1308 22 1.000 1 3 KKA01567.1;model.g1394.t1;34-9_0624;

1287 1310 36 1.000 1 3 KKA01569.1;model.g3192.t1;34-9_0626;

1289 1312 34 1.000 1 3 KKA01571.1;model.g2411.t1;34-9_0627;

1293 1316 36 1.000 1 3 KKA01572.1;model.g2409.t1;34-9_0633;

1301 1324 7 0.502 1 3 KKA01581.1;model.g2336.t1;34-9_0649;

1304 1327 60 1.000 1 3 KKA03555.1;model.g2331.t1;34-9_0655;

1328 1351 42 1.000 1 3 KKA03630.1;model.g2462.t1;34-9_0740;

1330 1353 32 1.000 1 3 KKA03634.1;model.g2543.t1;34-9_0744;

1332 1355 22 1.000 1 3 KKA03636.1;model.g2545.t1;34-9_0746;

1334 1357 22 1.000 1 3 KKA03638.1;model.g2548.t1;34-9_0748;

1335 1358 70 1.000 1 3 KKA03639.1;model.g2353.t1;34-9_0749;

1336 1359 20 1.000 1 3 KKA03640.1;model.g2354.t1;34-9_0750;

1338 1361 14 1.000 1 3 KKA03645.1;model.g2359.t1;34-9_0754;

1339 1362 28 1.000 1 3 KKA03647.1;model.g2362.t1;34-9_0755;

1342 1365 15 1.000 1 3 KKA03649.1;model.g2365.t1;34-9_0758;

1345 1368 23 1.000 1 3 KKA03652.1;model.g2368.t1;34-9_0761;

1346 1369 11 1.000 1 3 KKA03653.1;model.g2369.t1;34-9_0762;

1347 1370 27 1.000 1 3 KKA03654.1;model.g2370.t1;34-9_0763;

1348 1371 18 1.000 1 3 KKA03655.1;model.g2373.t1;34-9_0764;

1351 1374 34 1.000 1 3 KKA03659.1;model.g2375.t1;34-9_0767;

1352 1375 27 1.000 1 3 KKA03662.1;model.g2378.t1;34-9_0770;

1355 1378 16 1.000 1 3 KKA03667.1;model.g2384.t1;34-9_0775;

1357 1380 43 1.000 1 3 KKA03669.1;model.g2390.t1;34-9_0778;

1358 1381 11 1.000 1 3 KKA03670.1;model.g2396.t1;34-9_0779;

1363 1386 70 1.000 1 3 KKA03676.1;model.g1877.t1;34-9_0785;

1364 1387 13 1.000 1 3 KKA03677.1;model.g1879.t1;34-9_0786;

1369 1392 11 1.000 1 3 KKA03684.1;model.g1861.t1;34-9_0792;

1371 1394 87 1.000 1 3 KKA03686.1;model.g1859.t1;34-9_0794;

1372 1395 58 1.000 1 3 KKA03687.1;model.g1858.t1;34-9_0795;

1373 1396 29 1.000 1 3 KKA03688.1;model.g1857.t1;34-9_0796;

1375 1398 69 1.000 1 3 KKA03690.1;model.g1852.t1;34-9_0801;

1376 1399 30 1.000 1 3 KKA03691.1;model.g1851.t1;34-9_0802;

1377 1400 28 1.000 1 3 KKA03692.1;model.g1850.t1;34-9_0803;

1381 1404 16 1.000 1 3 KKA03696.1;model.g1843.t1;34-9_0807;

1382 1405 36 1.000 1 3 34-9_3130;KKA02903.1;model.g94.t1;

1383 1406 33 1.000 1 3 KKA03700.1;model.g1838.t1;34-9_0811;

1385 1408 30 1.000 1 3 KKA03702.1;model.g1836.t1;34-9_0813;

1386 1409 6 0.502 1 3 KKA03703.1;model.g2469.t1;34-9_0814;

1387 1410 47 1.000 1 3 KKA03704.1;model.g1835.t1;34-9_0815;

1388 1411 25 1.000 1 3 KKA03707.1;model.g1831.t1;34-9_0817;

1390 1413 17 1.000 1 3 KKA03710.1;model.g1830.t1;34-9_0819;

1391 1415 27 1.000 1 3 KKA03711.1;model.g1847.t1;34-9_0821;

1396 1420 21 1.000 1 3 KKA03717.1;model.g2422.t1;34-9_0829;

1398 1422 30 1.000 1 3 34-9_1898;KKA01870.1;model.g2309.t1;

1400 1424 18 1.000 1 3 KKA03719.1;model.g2425.t1;34-9_0832;

1402 1426 14 1.000 1 3 KKA03721.1;model.g2427.t1;34-9_0834;

1403 1427 15 1.000 1 3 KKA03722.1;model.g2428.t1;34-9_0835;

1405 1429 31 1.000 1 3 KKA03725.1;model.g2433.t1;34-9_0838;

1406 1430 72 1.000 1 3 KKA03726.1;model.g2431.t1;34-9_0839;

1409 1433 73 1.000 1 3 34-9_2660;KKA02151.1;model.g1080.t1;

1413 1437 69 1.000 1 3 KKA03732.1;model.g1819.t1;34-9_0846;

1415 1439 20 1.000 1 3 KKA03734.1;model.g1823.t1;34-9_0848;

1416 1440 24 1.000 1 3 KKA03735.1;model.g1822.t1;34-9_0849;

1418 1442 42 1.000 1 3 KKA03737.1;model.g1806.t1;34-9_0851;

1420 1444 51 1.000 1 3 KKA03739.1;model.g1868.t1;34-9_0854;

1422 1445 57 1.000 1 3 KKA02653.1;34-9_3459;model.g3539.t1;

1425 1449 13 1.000 1 3 KKA03741.1;model.g1872.t1;34-9_0858;

1429 1453 24 1.000 1 3 KKA03744.1;model.g1876.t1;34-9_0862;

1430 1454 36 1.000 1 3 KKA01962.1;model.g339.t1;34-9_1338;

1432 1456 60 1.000 1 3 KKA03746.1;model.g2400.t1;34-9_0864;

1435 1459 39 1.000 1 3 KKA03750.1;model.g40.t1;34-9_0868;

1436 1460 29 1.000 1 3 KKA03751.1;model.g2404.t1;34-9_0869;

1437 1461 25 1.000 1 3 KKA03752.1;model.g2406.t1;34-9_0870;

1438 1462 30 1.000 1 3 KKA03754.1;model.g2415.t1;34-9_0872;

1443 1467 18 1.000 1 3 KKA03760.1;model.g1382.t1;34-9_0878;

1446 1470 13 1.000 1 3 KKA03765.1;model.g2460.t1;34-9_0883;

1449 1473 42 1.000 1 3 KKA03769.1;model.g2455.t1;34-9_0887;

1452 1476 21 1.000 1 3 KKA03773.1;model.g2012.t1;34-9_0892;

1455 1479 32 1.000 1 3 KKA03775.1;model.g1956.t1;34-9_0895;

1456 1480 22 1.000 1 3 KKA03776.1;model.g1957.t1;34-9_0896;

1457 1481 69 1.000 1 3 KKA03777.1;model.g1958.t1;34-9_0897;

1458 1482 49 1.000 1 3 KKA03778.1;model.g1959.t1;34-9_0898;

1459 1483 47 1.000 1 3 KKA03779.1;model.g1960.t1;34-9_0899;

1463 1487 41 1.000 1 3 KKA03783.1;model.g1964.t1;34-9_0904;

1464 1488 37 1.000 1 3 KKA03784.1;model.g1965.t1;34-9_0905;

1466 1490 17 1.000 1 3 KKA03786.1;model.g1967.t1;34-9_0907;

1469 1493 83 1.000 1 3 KKA03789.1;model.g1971.t1;34-9_0911;

1470 1494 19 1.000 1 3 KKA03790.1;model.g1973.t1;34-9_0912;

1471 1495 62 1.000 1 3 KKA03791.1;model.g1974.t1;34-9_0913;

1475 1499 17 1.000 1 3 KKA03801.1;model.g2110.t1;34-9_0922;

1477 1501 17 1.000 1 3 KKA03803.1;model.g2112.t1;34-9_0924;

1478 1502 12 1.000 1 3 KKA03804.1;model.g2113.t1;34-9_0925;

1480 1505 17 1.000 1 3 KKA03805.1;model.g2115.t1;34-9_0927;

1482 1507 14 1.000 1 3 KKA03807.1;model.g2117.t1;34-9_0929;

1483 1508 36 1.000 1 3 KKA03808.1;model.g2118.t1;34-9_0930;

1484 1509 37 1.000 1 3 KKA03810.1;model.g2120.t1;34-9_0932;

1485 1510 23 1.000 1 3 KKA03812.1;model.g2122.t1;34-9_0934;

1487 1512 58 1.000 1 3 KKA03814.1;model.g2125.t1;34-9_0936;

1488 1513 12 1.000 1 3 KKA03815.1;model.g2126.t1;34-9_0937;

1491 1516 56 1.000 1 3 KKA03819.1;model.g2132.t1;34-9_0941;

1492 1517 35 1.000 1 3 KKA03820.1;model.g2133.t1;34-9_0942;

1493 1518 13 1.000 1 3 KKA03821.1;model.g2134.t1;34-9_0943;

1494 1519 50 1.000 1 3 KKA03822.1;model.g2135.t1;34-9_0944;

1495 1520 12 1.000 1 3 KKA03823.1;model.g2136.t1;34-9_0945;

1496 1521 30 1.000 1 3 KKA03824.1;model.g2137.t1;34-9_0946;

1497 1522 39 1.000 1 3 KKA03825.1;model.g2138.t1;34-9_0947;

1500 1525 11 1.000 1 3 KKA03828.1;model.g2141.t1;34-9_0950;

1501 1526 12 1.000 1 3 KKA03829.1;model.g2142.t1;34-9_0951;

1502 1527 23 1.000 1 3 34-9_2134;model.g741.t1;KKA03548.1;

1503 1528 14 1.000 1 3 34-9_1243;model.g2028.t1;KKA01913.1;

1505 1530 75 1.000 1 3 34-9_2617;model.g3490.t1;KKA01191.1;

1508 1533 30 1.000 1 3 KKA03835.1;model.g2146.t1;34-9_0957;

1509 1534 5 0.502 1 3 KKA03837.1;model.g2148.t1;34-9_0959;

1512 1537 63 1.000 1 3 KKA03839.1;model.g2106.t1;34-9_0962;

1514 1539 29 1.000 1 3 KKA03841.1;model.g2103.t1;34-9_0964;

1515 1540 32 1.000 1 3 KKA03842.1;model.g2102.t1;34-9_0965;

1516 1541 57 1.000 1 3 KKA03843.1;model.g2101.t1;34-9_0966;

1517 1542 19 1.000 1 3 KKA03844.1;model.g2099.t1;34-9_0967;

1520 1545 38 1.000 1 3 KKA03848.1;model.g2095.t1;34-9_0971;

1521 1546 22 1.000 1 3 KKA03849.1;model.g2094.t1;34-9_0972;

1526 1551 49 1.000 1 3 KKA03853.1;model.g2175.t1;34-9_0980;

1527 1552 19 1.000 1 3 KKA03854.1;model.g2176.t1;34-9_0981;

1528 1553 36 1.000 1 3 KKA03855.1;model.g2177.t1;34-9_0982;

1529 1554 76 1.000 1 3 KKA03857.1;model.g2183.t1;34-9_0984;

1533 1558 28 1.000 1 3 KKA03861.1;model.g2190.t1;34-9_0989;

1534 1559 78 1.000 1 3 KKA03862.1;model.g2191.t1;34-9_0990;

1535 1560 24 1.000 1 3 KKA03863.1;model.g2192.t1;34-9_0991;

1537 1560 35 1.000 1 3 KKA01865.1;34-9_1895;model.g2280.t1;

1538 1560 15 1.000 1 3 model.g1695.t1;34-9_1410;KKA01672.1;

1540 1565 11 1.000 1 3 KKA03866.1;model.g2195.t1;34-9_0994;

1541 1566 24 1.000 1 3 KKA03867.1;model.g2196.t1;34-9_0995;

1542 1567 34 1.000 1 3 KKA03868.1;model.g2197.t1;34-9_0996;

1544 1570 20 1.000 1 3 KKA03873.1;model.g2202.t1;34-9_1000;

1546 1572 45 1.000 1 3 KKA03877.1;model.g1032.t1;34-9_1003;

1547 1573 44 1.000 1 3 KKA03878.1;model.g1031.t1;34-9_1004;

1548 1574 15 1.000 1 3 KKA03879.1;model.g1030.t1;34-9_1005;

1550 1576 17 1.000 1 3 KKA03881.1;model.g2214.t1;34-9_1007;

1554 1580 85 1.000 1 3 KKA03902.1;model.g2213.t1;34-9_1029;

1558 1584 31 1.000 1 3 KKA03905.1;model.g2210.t1;34-9_1033;

1559 1585 18 1.000 1 3 KKA03906.1;model.g2209.t1;34-9_1034;

1560 1586 21 1.000 1 3 KKA03907.1;model.g2208.t1;34-9_1035;

1561 1587 77 1.000 1 3 KKA03908.1;model.g1040.t1;34-9_1036;

1564 1590 71 1.000 1 3 KKA03910.1;model.g1035.t1;34-9_1039;

1565 1591 46 1.000 1 3 KKA03911.1;model.g2205.t1;34-9_1040;

1567 1593 27 1.000 1 3 KKA03914.1;model.g2207.t1;34-9_1042;

1568 1593 16 1.000 1 3 34-9_1328;model.g326.t1;KKA01971.1;

1574 1600 75 1.000 1 3 KKA03919.1;model.g2160.t1;34-9_1050;

1577 1603 23 1.000 1 3 KKA03922.1;model.g2157.t1;34-9_1053;

1579 1605 11 1.000 1 3 KKA03925.1;model.g2153.t1;34-9_1056;

1580 1606 43 1.000 1 3 KKA03926.1;model.g2152.t1;34-9_1057;

1582 1608 31 1.000 1 3 KKA03930.1;model.g2008.t1;34-9_1061;

1583 1609 12 1.000 1 3 KKA03931.1;model.g2036.t1;34-9_1062;

1587 1613 49 1.000 1 3 KKA03937.1;model.g2037.t1;34-9_1069;

1588 1614 19 1.000 1 3 KKA03938.1;model.g2038.t1;34-9_1070;

1590 1616 75 1.000 1 3 KKA03940.1;model.g2040.t1;34-9_1072;

1591 1617 37 1.000 1 3 KKA03942.1;model.g2042.t1;34-9_1074;

1594 1620 58 1.000 1 3 KKA03946.1;model.g2049.t1;34-9_1078;

1595 1621 33 1.000 1 3 KKA03948.1;model.g2046.t1;34-9_1080;

1596 1622 22 1.000 1 3 KKA03949.1;model.g2045.t1;34-9_1081;

1597 1623 37 1.000 1 3 KKA03950.1;model.g2044.t1;34-9_1082;

1600 1626 34 1.000 1 3 KKA03954.1;model.g2058.t1;34-9_1086;

1601 1627 35 1.000 1 3 KKA03955.1;model.g2060.t1;34-9_1087;

1604 1630 72 1.000 1 3 KKA03957.1;model.g2066.t1;34-9_1090;

1605 1631 13 1.000 1 3 KKA03958.1;model.g2067.t1;34-9_1091;

1606 1632 24 1.000 1 3 KKA03959.1;model.g2068.t1;34-9_1092;

1607 1633 59 1.000 1 3 KKA03960.1;model.g2069.t1;34-9_1093;

1608 1634 15 1.000 1 3 KKA03961.1;model.g2070.t1;34-9_1094;

1609 1635 52 1.000 1 3 KKA03966.1;model.g2075.t1;34-9_1097;

1610 1636 22 1.000 1 3 KKA03967.1;model.g2076.t1;34-9_1098;

1613 1639 63 1.000 1 3 KKA03971.1;model.g2084.t1;34-9_1105;

1616 1642 33 1.000 1 3 KKA03974.1;model.g2089.t1;34-9_1108;

1617 1643 24 1.000 1 3 KKA03976.1;model.g2091.t1;34-9_1110;

1618 1644 19 1.000 1 3 KKA03977.1;model.g1043.t1;34-9_1111;

1619 1645 12 1.000 1 3 KKA03978.1;model.g1044.t1;34-9_1112;

1620 1646 48 1.000 1 3 KKA03980.1;model.g1046.t1;34-9_1114;

1621 1647 63 1.000 1 3 KKA03981.1;model.g1047.t1;34-9_1115;

1623 1650 75 1.000 1 3 KKA03985.1;model.g1051.t1;34-9_1118;

1625 1652 56 1.000 1 3 KKA03989.1;model.g1054.t1;34-9_1122;

1630 1657 35 1.000 1 3 KKA01800.1;model.g2225.t1;34-9_1158;

1633 1660 31 1.000 1 3 KKA01795.1;model.g634.t1;34-9_1165;

1639 1666 54 1.000 1 3 KKA01787.1;model.g3561.t1;34-9_1180;

1640 1667 42 1.000 1 3 KKA01786.1;model.g3559.t1;34-9_1181;

1646 1673 29 1.000 1 3 KKA01777.1;model.g615.t1;34-9_1191;

1647 1674 44 1.000 1 3 KKA01774.1;model.g616.t1;34-9_1196;

1656 1683 19 1.000 1 3 KKA01905.1;model.g2000.t1;34-9_1233;

1659 1686 20 1.000 1 3 KKA01906.1;model.g2004.t1;34-9_1236;

1661 1688 52 1.000 1 3 KKA01909.1;model.g2032.t1;34-9_1239;

1662 1689 14 1.000 1 3 KKA01910.1;model.g2031.t1;34-9_1240;

1664 1691 72 1.000 1 3 KKA01911.1;model.g2029.t1;34-9_1242;

1665 1692 12 1.000 1 3 KKA01914.1;model.g2022.t1;34-9_1244;

1667 1694 26 1.000 1 3 KKA01915.1;model.g2025.t1;34-9_1246;

1669 1696 74 1.000 1 3 KKA01917.1;model.g2021.t1;34-9_1248;

1671 1698 12 1.000 1 3 KKA01918.1;model.g2017.t1;34-9_1250;

1672 1699 82 1.000 1 3 KKA01919.1;model.g3118.t1;34-9_1251;

1673 1699 59 1.000 1 3 KKA02450.1;model.g1216.t1;34-9_1983;

1676 1703 64 1.000 1 3 KKA01920.1;model.g3115.t1;34-9_1254;

1678 1705 11 1.000 1 3 KKA01921.1;model.g3113.t1;34-9_1256;

1679 1706 10 1.000 1 3 KKA01922.1;model.g3112.t1;34-9_1257;

1680 1707 13 1.000 1 3 KKA01923.1;model.g3123.t1;34-9_1258;

1682 1709 19 1.000 1 3 KKA01925.1;model.g3125.t1;34-9_1260;

1683 1710 20 1.000 1 3 KKA01926.1;model.g3131.t1;34-9_1262;

1688 1715 31 1.000 1 3 KKA01931.1;model.g3142.t1;34-9_1272;

1691 1718 20 1.000 1 3 KKA01933.1;model.g3138.t1;34-9_1276;

1692 1719 20 1.000 1 3 KKA01934.1;model.g3137.t1;34-9_1277;

1693 1720 87 1.000 1 3 KKA01935.1;model.g3136.t1;34-9_1279;

1700 1727 32 1.000 1 3 KKA01249.1;model.g384.t1;34-9_1292;

1702 1729 38 1.000 1 3 KKA01251.1;model.g381.t1;34-9_1294;

1703 1729 30 1.000 1 3 KKA01828.1;model.g1456.t1;34-9_3282;

1706 1733 15 1.000 1 3 KKA01255.1;model.g374.t1;34-9_1298;

1707 1734 27 1.000 1 3 KKA01256.1;model.g373.t1;34-9_1299;

1708 1735 18 1.000 1 3 KKA01257.1;model.g372.t1;34-9_1300;

1709 1736 14 1.000 1 3 KKA01258.1;model.g3110.t1;34-9_1301;

1710 1737 33 1.000 1 3 KKA01259.1;model.g3109.t1;34-9_1302;

1712 1738 14 1.000 1 3 model.g3199.t1;34-9_2526;KKA03079.1;

1713 1740 30 1.000 1 3 KKA01261.1;model.g3106.t1;34-9_1304;

1715 1742 19 1.000 1 3 34-9_2877;KKA01271.1;model.g219.t1;

1716 1743 39 1.000 1 3 KKA01264.1;model.g3120.t1;34-9_1309;

1718 1745 23 1.000 1 3 KKA01990.1;model.g1898.t1;34-9_1311;

1719 1746 29 1.000 1 3 KKA01989.1;model.g1894.t1;34-9_1312;

1720 1747 14 1.000 1 3 KKA01988.1;model.g1893.t1;34-9_1313;

1721 1748 23 1.000 1 3 KKA01987.1;model.g1892.t1;34-9_1314;

1722 1749 15 1.000 1 3 KKA01986.1;model.g1891.t1;34-9_1315;

1725 1752 42 1.000 1 3 KKA01983.1;model.g1889.t1;34-9_1318;

1727 1754 68 1.000 1 3 KKA01981.1;model.g1886.t1;34-9_1321;

1728 1754 38 1.000 1 3 KKA01166.1;model.g1331.t1;34-9_2959;

1729 1756 25 1.000 1 3 KKA01980.1;model.g324.t1;34-9_1322;

1730 1757 26 1.000 1 3 KKA01977.1;model.g322.t1;34-9_1323;

1731 1758 34 1.000 1 3 KKA01975.1;model.g327.t1;34-9_1324;

1732 1759 48 1.000 1 3 KKA01974.1;model.g329.t1;34-9_1325;

1733 1760 23 1.000 1 3 KKA01972.1;model.g1884.t1;34-9_1327;

1734 1761 32 1.000 1 3 KKA01970.1;model.g332.t1;34-9_1329;

1735 1762 27 1.000 1 3 KKA01969.1;model.g331.t1;34-9_1330;

1736 1763 33 1.000 1 3 KKA01968.1;model.g333.t1;34-9_1331;

1739 1766 81 1.000 1 3 34-9_1537;model.g401.t1;KKA02786.1;

1740 1767 33 1.000 1 3 KKA01965.1;model.g337.t1;34-9_1335;

1741 1768 22 1.000 1 3 KKA01964.1;model.g342.t1;34-9_1336;

1742 1769 37 1.000 1 3 KKA01963.1;model.g341.t1;34-9_1337;

1743 1770 41 1.000 1 3 KKA01961.1;model.g338.t1;34-9_1339;

1745 1772 33 1.000 1 3 KKA01958.1;model.g346.t1;34-9_1342;

1746 1773 33 1.000 1 3 KKA01957.1;model.g347.t1;34-9_1343;

1747 1774 37 1.000 1 3 KKA01956.1;model.g348.t1;34-9_1344;

1748 1775 25 1.000 1 3 KKA01954.1;model.g2605.t1;34-9_1346;

1749 1776 61 1.000 1 3 KKA01953.1;model.g2604.t1;34-9_1347;

1751 1778 17 1.000 1 3 KKA01951.1;model.g1540.t1;34-9_1349;

1752 1779 13 1.000 1 3 KKA01950.1;model.g2602.t1;34-9_1350;

1753 1780 22 1.000 1 3 KKA01949.1;model.g2601.t1;34-9_1351;

1754 1781 53 1.000 1 3 KKA01947.1;model.g352.t1;34-9_1353;

1757 1784 17 1.000 1 3 KKA01943.1;model.g1715.t1;34-9_1359;

1758 1785 76 1.000 1 3 KKA01942.1;model.g2614.t1;34-9_1360;

1759 1786 19 1.000 1 3 KKA01941.1;model.g2615.t1;34-9_1361;

1761 1788 41 1.000 1 3 KKA01939.1;model.g2617.t1;34-9_1363;

1762 1789 79 1.000 1 3 KKA01938.1;model.g2618.t1;34-9_1364;

1763 1790 38 1.000 1 3 KKA01024.1;model.g2623.t1;34-9_1366;

1764 1791 65 1.000 1 3 KKA01023.1;model.g2641.t1;34-9_1367;

1766 1793 28 1.000 1 3 KKA01021.1;model.g2640.t1;34-9_1369;

1767 1794 18 1.000 1 3 KKA01020.1;model.g2632.t1;34-9_1370;

1768 1796 15 1.000 1 3 KKA01652.1;model.g2633.t1;34-9_1372;

1771 1799 27 1.000 1 3 KKA01653.1;model.g2629.t1;34-9_1375;

1775 1803 33 1.000 1 3 KKA01654.1;model.g2625.t1;34-9_1380;

1777 1805 56 1.000 1 3 KKA01656.1;model.g1645.t1;34-9_1382;

1785 1813 26 1.000 1 3 KKA01664.1;model.g1680.t1;34-9_1397;

1790 1818 32 1.000 1 3 KKA01666.1;model.g1687.t1;34-9_1402;

1791 1819 34 1.000 1 3 KKA01668.1;model.g1689.t1;34-9_1403;

1792 1820 58 1.000 1 3 KKA01670.1;model.g1690.t1;34-9_1405;

1794 1822 18 1.000 1 3 KKA01671.1;model.g1692.t1;34-9_1407;

1796 1824 7 0.502 1 3 KKA01673.1;model.g1697.t1;34-9_1412;

1797 1825 19 1.000 1 3 KKA01676.1;model.g1701.t1;34-9_1415;

1798 1826 76 1.000 1 3 KKA01677.1;model.g1703.t1;34-9_1416;

1799 1827 23 1.000 1 3 KKA01678.1;model.g1705.t1;34-9_1417;

1807 1835 46 1.000 1 3 KKA01033.1;model.g2650.t1;34-9_1427;

1808 1836 33 1.000 1 3 KKA01034.1;model.g2649.t1;34-9_1428;

1810 1838 14 1.000 1 3 KKA01035.1;model.g2647.t1;34-9_1430;

1813 1841 18 1.000 1 3 KKA01069.1;model.g2645.t1;34-9_1433;

1814 1842 38 1.000 1 3 KKA01068.1;model.g2646.t1;34-9_1434;

1819 1847 8 0.502 1 3 KKA03168.1;model.g2659.t1;34-9_1449;

1821 1849 18 1.000 1 3 34-9_2090;KKA02614.1;model.g1585.t1;

1824 1852 40 1.000 1 3 KKA03172.1;model.g2666.t1;34-9_1458;

1825 1853 20 1.000 1 3 KKA03174.1;model.g2669.t1;34-9_1460;

1831 1859 7 0.502 1 3 KKA03178.1;model.g2676.t1;34-9_1465;

1832 1860 10 1.000 1 3 KKA03179.1;model.g2687.t1;34-9_1467;

1833 1861 57 1.000 1 3 KKA03180.1;model.g2688.t1;34-9_1468;

1834 1862 57 1.000 1 3 KKA03181.1;model.g2690.t1;34-9_1469;

1840 1868 17 1.000 1 3 KKA03186.1;model.g2684.t1;34-9_1476;

1842 1870 22 1.000 1 3 KKA03187.1;model.g2682.t1;34-9_1478;

1849 1877 20 1.000 1 3 KKA03194.1;model.g873.t1;34-9_1488;

1856 1884 36 1.000 1 3 KKA03207.1;model.g2494.t1;34-9_1508;

1861 1889 26 1.000 1 3 KKA03213.1;model.g2505.t1;34-9_1518;

1862 1890 47 1.000 1 3 KKA03215.1;model.g2508.t1;34-9_1520;

1866 1894 17 1.000 1 3 KKA03218.1;model.g1302.t1;34-9_1524;

1867 1895 24 1.000 1 3 KKA03219.1;model.g1299.t1;34-9_1527;

1872 1900 36 1.000 1 3 34-9_2594;KKA01211.1;model.g3339.t1;

1873 1901 52 1.000 1 3 KKA02363.1;model.g2441.t1;34-9_3523;

1874 1902 22 1.000 1 3 KKA02787.1;model.g400.t1;34-9_1538;

1876 1904 44 1.000 1 3 KKA02788.1;model.g425.t1;34-9_1539;

1877 1905 79 1.000 1 3 KKA02789.1;model.g2484.t1;34-9_1540;

1880 1908 21 1.000 1 3 KKA02794.1;model.g195.t1;34-9_1546;

1881 1909 15 1.000 1 3 KKA02796.1;model.g3407.t1;34-9_1547;

1882 1910 27 1.000 1 3 KKA02797.1;model.g193.t1;34-9_1548;

1884 1912 33 1.000 1 3 KKA02799.1;model.g191.t1;34-9_1550;

1892 1920 70 1.000 1 3 KKA02823.1;model.g448.t1;34-9_1575;

1895 1923 87 1.000 1 3 KKA02830.1;model.g441.t1;34-9_1582;

1897 1925 19 1.000 1 3 KKA02832.1;model.g439.t1;34-9_1584;

1898 1926 74 1.000 1 3 KKA02833.1;model.g438.t1;34-9_1585;

1903 1931 28 1.000 1 3 KKA02840.1;model.g432.t1;34-9_1591;

1905 1934 25 1.000 1 3 KKA02848.1;model.g1574.t1;34-9_1597;

1906 1935 64 1.000 1 3 KKA02849.1;model.g2490.t1;34-9_1599;

1907 1936 12 1.000 1 3 KKA02850.1;model.g3515.t1;34-9_1600;

1909 1938 72 1.000 1 3 KKA02853.1;model.g3520.t1;34-9_1603;

1910 1939 20 1.000 1 3 KKA02855.1;model.g3518.t1;34-9_1605;

1911 1940 69 1.000 1 3 KKA02856.1;model.g3523.t1;34-9_1607;

1913 1942 26 1.000 1 3 KKA02857.1;model.g3522.t1;34-9_1608;

1915 1944 23 1.000 1 3 KKA02858.1;model.g3525.t1;34-9_1610;

1917 1946 22 1.000 1 3 KKA02860.1;model.g3531.t1;34-9_1615;

1919 1948 87 1.000 1 3 KKA02864.1;model.g204.t1;34-9_1618;

1920 1949 18 1.000 1 3 KKA02866.1;model.g202.t1;34-9_1620;

1921 1950 20 1.000 1 3 KKA02869.1;model.g2592.t1;34-9_1622;

1924 1953 43 1.000 1 3 KKA01105.1;model.g2588.t1;34-9_1627;

1925 1954 82 1.000 1 3 KKA01106.1;model.g2581.t1;34-9_1628;

1926 1955 64 1.000 1 3 KKA01107.1;model.g2584.t1;34-9_1629;

1927 1956 38 1.000 1 3 KKA01109.1;model.g2586.t1;34-9_1630;

1928 1957 17 1.000 1 3 KKA01110.1;model.g2580.t1;34-9_1631;

1929 1958 27 1.000 1 3 KKA01113.1;model.g1662.t1;34-9_1633;

1932 1961 37 1.000 1 3 KKA01756.1;model.g576.t1;34-9_1640;

1940 1969 21 1.000 1 3 KKA01749.1;model.g515.t1;34-9_1649;

1950 1980 31 1.000 1 3 KKA01742.1;model.g2917.t1;34-9_1662;

1951 1981 33 1.000 1 3 KKA01740.1;model.g2919.t1;34-9_1664;

1955 1985 29 1.000 1 3 KKA01738.1;model.g2890.t1;34-9_1669;

1958 1989 15 1.000 1 3 KKA01736.1;model.g2894.t1;34-9_1672;

1961 1992 29 1.000 1 3 KKA01734.1;model.g2899.t1;34-9_1676;

1962 1993 73 1.000 1 3 KKA01733.1;model.g2900.t1;34-9_1677;

1963 1994 30 1.000 1 3 KKA01731.1;model.g2904.t1;34-9_1680;

1969 2000 30 1.000 1 3 KKA01728.1;model.g498.t1;34-9_1686;

1972 2003 44 1.000 1 3 KKA01726.1;model.g501.t1;34-9_1690;

1974 2005 30 1.000 1 3 KKA02232.1;model.g2934.t1;34-9_1694;

1980 2011 85 1.000 1 3 KKA02233.1;model.g18.t1;34-9_1701;

1981 2012 54 1.000 1 3 KKA02235.1;model.g2930.t1;34-9_1703;

1982 2013 13 1.000 1 3 KKA02236.1;model.g749.t1;34-9_1704;

1984 2015 30 1.000 1 3 KKA02239.1;model.g2920.t1;34-9_1708;

1986 2017 51 1.000 1 3 34-9_2054;KKA02593.1;model.g834.t1;

1989 2020 16 1.000 1 3 KKA02240.1;model.g2882.t1;34-9_1714;

1993 2024 23 1.000 1 3 KKA02242.1;model.g2873.t1;34-9_1718;

2003 2034 24 1.000 1 3 KKA02249.1;model.g2853.t1;34-9_1731;

2005 2036 14 1.000 1 3 KKA02250.1;model.g2851.t1;34-9_1733;

2008 2039 33 1.000 1 3 KKA02253.1;model.g2847.t1;34-9_1737;

2009 2040 31 1.000 1 3 KKA02254.1;model.g2846.t1;34-9_1738;

2013 2044 48 1.000 1 3 KKA02258.1;model.g2840.t1;34-9_1742;

2016 2047 27 1.000 1 3 KKA02261.1;model.g2838.t1;34-9_1745;

2021 2052 22 1.000 1 3 KKA01995.1;model.g364.t1;34-9_1769;

2024 2055 35 1.000 1 3 KKA01999.1;model.g357.t1;34-9_1774;

2025 2056 23 1.000 1 3 KKA02000.1;model.g354.t1;34-9_1775;

2037 2068 18 1.000 1 3 KKA02188.1;model.g909.t1;34-9_1829;

2039 2070 26 1.000 1 3 KKA02190.1;model.g249.t1;34-9_1832;

2042 2073 38 1.000 1 3 KKA02193.1;model.g153.t1;34-9_1837;

2045 2076 21 1.000 1 3 KKA02196.1;model.g154.t1;34-9_1840;

2048 2079 59 1.000 1 3 KKA02197.1;model.g157.t1;34-9_1843;

2050 2081 22 1.000 1 3 KKA02199.1;model.g161.t1;34-9_1846;

2058 2089 49 1.000 1 3 KKA02204.1;model.g917.t1;34-9_1855;

2059 2090 25 1.000 1 3 KKA02205.1;model.g237.t1;34-9_1858;

2060 2091 23 1.000 1 3 KKA02206.1;model.g238.t1;34-9_1859;

2069 2100 75 1.000 1 3 KKA02214.1;model.g182.t1;34-9_1877;

2073 2104 17 1.000 1 3 KKA01854.1;model.g2264.t1;34-9_1884;

2074 2105 23 1.000 1 3 KKA01856.1;model.g2267.t1;34-9_1886;

2075 2106 24 1.000 1 3 KKA01857.1;model.g2268.t1;34-9_1887;

2078 2109 46 1.000 1 3 KKA01862.1;model.g2277.t1;34-9_1893;

2080 2111 45 1.000 1 3 KKA01871.1;model.g2308.t1;34-9_1899;

2081 2112 11 1.000 1 3 KKA01872.1;model.g2306.t1;34-9_1901;

2083 2114 11 1.000 1 3 KKA01874.1;model.g2303.t1;34-9_1903;

2085 2116 26 1.000 1 3 KKA01876.1;model.g2293.t1;34-9_1906;

2087 2118 35 1.000 1 3 KKA01879.1;model.g2299.t1;34-9_1908;

2089 2120 27 1.000 1 3 KKA01881.1;model.g2291.t1;34-9_1910;

2090 2121 72 1.000 1 3 KKA01882.1;model.g2290.t1;34-9_1911;

2092 2123 94 1.000 1 3 KKA02491.1;model.g2284.t1;34-9_1915;

2093 2124 83 1.000 1 3 KKA02490.1;model.g2283.t1;34-9_1917;

2094 2125 14 1.000 1 3 KKA02489.1;model.g2314.t1;34-9_1918;

2105 2136 29 1.000 1 3 KKA02484.1;model.g2324.t1;34-9_1931;

2108 2139 17 1.000 1 3 KKA02480.1;model.g1162.t1;34-9_1935;

2112 2143 29 1.000 1 3 KKA02477.1;model.g1159.t1;34-9_1941;

2115 2146 28 1.000 1 3 KKA02473.1;model.g1173.t1;34-9_1947;

2117 2148 42 1.000 1 3 KKA02472.1;model.g1176.t1;34-9_1950;

2118 2149 60 1.000 1 3 KKA02471.1;model.g1177.t1;34-9_1951;

2123 2154 39 1.000 1 3 KKA02468.1;model.g1188.t1;34-9_1956;

2125 2156 26 1.000 1 3 KKA02465.1;model.g1190.t1;34-9_1959;

2131 2162 32 1.000 1 3 KKA02460.1;model.g1209.t1;34-9_1968;

2132 2163 43 1.000 1 3 KKA02458.1;model.g483.t1;34-9_1970;

2136 2167 33 1.000 1 3 KKA02457.1;model.g1203.t1;34-9_1974;

2138 2169 23 1.000 1 3 KKA02456.1;model.g1201.t1;34-9_1976;

2141 2172 42 1.000 1 3 KKA02452.1;model.g1218.t1;34-9_1981;

2142 2173 17 1.000 1 3 KKA02451.1;model.g1217.t1;34-9_1982;

2144 2175 40 1.000 1 3 KKA02449.1;model.g1258.t1;34-9_1985;

2146 2177 36 1.000 1 3 KKA02446.1;model.g1252.t1;34-9_1988;

2149 2180 32 1.000 1 3 KKA02445.1;model.g1249.t1;34-9_1991;

2150 2181 35 1.000 1 3 KKA02444.1;model.g1248.t1;34-9_1992;

2151 2182 53 1.000 1 3 KKA02443.1;model.g1247.t1;34-9_1993;

2158 2189 19 1.000 1 3 KKA02577.1;model.g1014.t1;34-9_2019;

2160 2191 52 1.000 1 3 KKA02578.1;model.g1016.t1;34-9_2021;

2162 2193 31 1.000 1 3 34-9_2854;KKA01163.1;model.g1524.t1;

2167 2198 49 1.000 1 3 KKA02580.1;model.g956.t1;34-9_2027;

2170 2201 23 1.000 1 3 KKA02582.1;model.g854.t1;34-9_2031;

2178 2209 35 1.000 1 3 KKA02586.1;model.g1560.t1;34-9_2043;

2179 2210 70 1.000 1 3 KKA02588.1;model.g1565.t1;34-9_2046;

2183 2214 62 1.000 1 3 34-9_3071;model.g2770.t1;KKA02931.1;

2184 2215 40 1.000 1 3 KKA02591.1;model.g1549.t1;34-9_2050;

2188 2219 47 1.000 1 3 KKA02594.1;model.g2959.t1;34-9_2056;

2189 2220 57 1.000 1 3 KKA02595.1;model.g2958.t1;34-9_2057;

2190 2221 16 1.000 1 3 KKA02596.1;model.g2957.t1;34-9_2058;

2191 2222 35 1.000 1 3 KKA02598.1;model.g2973.t1;34-9_2059;

2192 2223 46 1.000 1 3 KKA02599.1;model.g2968.t1;34-9_2061;

2195 2226 84 1.000 1 3 KKA02600.1;model.g2964.t1;34-9_2064;

2197 2228 35 1.000 1 3 KKA02604.1;model.g2975.t1;34-9_2069;

2204 2235 19 1.000 1 3 KKA02607.1;model.g822.t1;34-9_2076;

2213 2244 29 1.000 1 3 34-9_2671;KKA02144.1;model.g3452.t1;

2214 2245 29 1.000 1 3 KKA02612.1;model.g2956.t1;34-9_2088;

2217 2248 29 1.000 1 3 KKA02619.1;model.g705.t1;34-9_2097;

2219 2250 87 1.000 1 3 KKA02621.1;model.g707.t1;34-9_2099;

2230 2261 41 1.000 1 3 KKA02628.1;model.g1415.t1;34-9_2119;

2235 2266 64 1.000 1 3 KKA02632.1;model.g2982.t1;34-9_2125;

2236 2267 64 1.000 1 3 KKA02633.1;model.g2983.t1;34-9_2126;

2238 2269 26 1.000 1 3 KKA02634.1;model.g2986.t1;34-9_2128;

2242 2273 16 1.000 1 3 KKA03544.1;model.g738.t1;34-9_2138;

2244 2275 37 1.000 1 3 KKA03542.1;model.g735.t1;34-9_2140;

2263 2295 40 1.000 1 3 KKA03428.1;model.g766.t1;34-9_2248;

2265 2297 43 1.000 1 3 KKA03425.1;model.g752.t1;34-9_2251;

2266 2298 46 1.000 1 3 KKA03424.1;model.g764.t1;34-9_2252;

2267 2299 13 1.000 1 3 KKA03423.1;model.g761.t1;34-9_2254;

2269 2301 23 1.000 1 3 KKA03422.1;model.g762.t1;34-9_2255;

2271 2303 26 1.000 1 3 KKA03420.1;model.g759.t1;34-9_2257;

2272 2304 19 1.000 1 3 KKA03419.1;model.g757.t1;34-9_2258;

2273 2305 25 1.000 1 3 KKA03418.1;model.g756.t1;34-9_2259;

2274 2306 31 1.000 1 3 KKA03413.1;model.g742.t1;34-9_2263;

2275 2307 46 1.000 1 3 KKA03412.1;model.g744.t1;34-9_2264;

2277 2309 15 1.000 1 3 KKA03410.1;model.g754.t1;34-9_2266;

2278 2310 36 1.000 1 3 KKA03407.1;model.g1925.t1;34-9_2269;

2279 2311 76 1.000 1 3 KKA03406.1;model.g1926.t1;34-9_2270;

2280 2312 15 1.000 1 3 KKA03405.1;model.g1920.t1;34-9_2271;

2283 2315 26 1.000 1 3 KKA03399.1;model.g1916.t1;34-9_2276;

2284 2316 28 1.000 1 3 KKA03397.1;model.g1915.t1;34-9_2277;

2285 2317 19 1.000 1 3 KKA03396.1;model.g1913.t1;34-9_2278;

2286 2318 29 1.000 1 3 KKA03394.1;model.g1910.t1;34-9_2280;

2287 2319 31 1.000 1 3 KKA03393.1;model.g1909.t1;34-9_2281;

2290 2322 21 1.000 1 3 KKA03388.1;model.g1902.t1;34-9_2287;

2291 2323 67 1.000 1 3 KKA03387.1;model.g774.t1;34-9_2288;

2292 2324 14 1.000 1 3 KKA03386.1;model.g1903.t1;34-9_2289;

2293 2325 12 1.000 1 3 KKA03385.1;model.g772.t1;34-9_2290;

2294 2326 32 1.000 1 3 KKA03381.1;model.g1927.t1;34-9_2293;

2295 2327 36 1.000 1 3 KKA03379.1;model.g769.t1;34-9_2295;

2296 2328 20 1.000 1 3 KKA03378.1;model.g770.t1;34-9_2296;

2299 2333 16 1.000 1 3 KKA03374.1;model.g3354.t1;34-9_2299;

2302 2336 14 1.000 1 3 KKA03371.1;model.g3349.t1;34-9_2304;

2303 2337 18 1.000 1 3 KKA03370.1;model.g3347.t1;34-9_2305;

2304 2338 79 1.000 1 3 KKA03369.1;model.g1931.t1;34-9_2306;

2305 2339 28 1.000 1 3 KKA03368.1;model.g1930.t1;34-9_2307;

2306 2340 11 1.000 1 3 KKA03367.1;model.g1929.t1;34-9_2308;

2307 2341 15 1.000 1 3 KKA03366.1;model.g3357.t1;34-9_2309;

2310 2344 54 1.000 1 3 KKA03363.1;model.g3196.t1;34-9_2312;

2311 2345 69 1.000 1 3 KKA03362.1;model.g3197.t1;34-9_2313;

2314 2348 23 1.000 1 3 KKA03359.1;model.g3149.t1;34-9_2316;

2316 2350 23 1.000 1 3 KKA03357.1;model.g804.t1;34-9_2318;

2317 2351 29 1.000 1 3 KKA03356.1;model.g3174.t1;34-9_2319;

2319 2353 37 1.000 1 3 KKA03354.1;model.g3150.t1;34-9_2321;

2320 2354 49 1.000 1 3 KKA03352.1;model.g788.t1;34-9_2322;

2321 2355 13 1.000 1 3 KKA03351.1;model.g790.t1;34-9_2323;

2322 2356 35 1.000 1 3 KKA03350.1;model.g792.t1;34-9_2324;

2323 2357 14 1.000 1 3 KKA03348.1;model.g794.t1;34-9_2326;

2324 2358 17 1.000 1 3 KKA03347.1;model.g795.t1;34-9_2327;

2325 2359 13 1.000 1 3 KKA03345.1;model.g799.t1;34-9_2329;

2327 2361 42 1.000 1 3 KKA03341.1;model.g3395.t1;34-9_2333;

2330 2364 13 1.000 1 3 KKA03338.1;model.g3381.t1;34-9_2336;

2332 2366 12 1.000 1 3 KKA03335.1;model.g3384.t1;34-9_2338;

2333 2367 15 1.000 1 3 KKA03334.1;model.g3385.t1;34-9_2339;

2335 2369 15 1.000 1 3 KKA03332.1;model.g3392.t1;34-9_2341;

2336 2370 45 1.000 1 3 KKA03331.1;model.g3391.t1;34-9_2342;

2339 2373 17 1.000 1 3 KKA03328.1;model.g3376.t1;34-9_2346;

2340 2374 32 1.000 1 3 KKA03327.1;model.g3377.t1;34-9_2347;

2345 2379 13 1.000 1 3 KKA03317.1;model.g3363.t1;34-9_2357;

2346 2380 10 1.000 1 3 KKA03316.1;model.g3362.t1;34-9_2358;

2347 2381 11 1.000 1 3 KKA03315.1;model.g3361.t1;34-9_2359;

2349 2383 65 1.000 1 3 KKA03313.1;model.g3358.t1;34-9_2361;

2352 2386 31 1.000 1 3 KKA03310.1;model.g3185.t1;34-9_2364;

2353 2387 83 1.000 1 3 KKA03309.1;model.g3187.t1;34-9_2365;

2354 2388 18 1.000 1 3 KKA03308.1;model.g3186.t1;34-9_2366;

2356 2390 23 1.000 1 3 KKA03306.1;model.g3156.t1;34-9_2369;

2361 2395 22 1.000 1 3 KKA03301.1;model.g3179.t1;34-9_2375;

2364 2398 39 1.000 1 3 KKA03299.1;model.g3161.t1;34-9_2378;

2366 2400 15 0.502 1 3 34-9_2687;KKA02135.1;model.g1094.t1;

2368 2402 54 1.000 1 3 KKA03295.1;model.g3182.t1;34-9_2382;

2377 2411 12 1.000 1 3 KKA02976.1;model.g2509.t1;34-9_2416;

2378 2412 43 1.000 1 3 KKA02979.1;model.g3419.t1;34-9_2419;

2381 2415 23 1.000 1 3 KKA02983.1;model.g3434.t1;34-9_2423;

2382 2416 19 1.000 1 3 KKA02984.1;model.g3433.t1;34-9_2424;

2384 2418 34 1.000 1 3 KKA02986.1;model.g3422.t1;34-9_2426;

2385 2419 62 1.000 1 3 KKA02987.1;model.g3255.t1;34-9_2428;

2386 2420 42 1.000 1 3 KKA02989.1;model.g3427.t1;34-9_2430;

2387 2421 55 1.000 1 3 KKA02990.1;model.g3428.t1;34-9_2431;

2388 2422 23 1.000 1 3 KKA02991.1;model.g3429.t1;34-9_2432;

2390 2424 22 1.000 1 3 KKA03003.1;model.g3264.t1;34-9_2443;

2392 2426 28 1.000 1 3 KKA03006.1;model.g3282.t1;34-9_2447;

2393 2427 11 1.000 1 3 KKA03007.1;model.g3273.t1;34-9_2448;

2394 2428 36 1.000 1 3 KKA03008.1;model.g3274.t1;34-9_2449;

2395 2429 15 1.000 1 3 KKA03009.1;model.g3275.t1;34-9_2450;

2396 2430 28 1.000 1 3 KKA03011.1;model.g3278.t1;34-9_2453;

2398 2433 18 1.000 1 3 KKA03014.1;model.g3272.t1;34-9_2456;

2400 2436 76 1.000 1 3 KKA03017.1;model.g3270.t1;34-9_2458;

2401 2437 78 1.000 1 3 KKA03018.1;model.g3269.t1;34-9_2459;

2405 2441 50 1.000 1 3 KKA03035.1;model.g3234.t1;34-9_2478;

2409 2445 31 1.000 1 3 KKA03042.1;model.g3204.t1;34-9_2487;

2411 2447 28 1.000 1 3 KKA03044.1;model.g3206.t1;34-9_2490;

2413 2449 80 1.000 1 3 KKA03045.1;model.g3209.t1;34-9_2492;

2415 2451 18 1.000 1 3 KKA03049.1;model.g3214.t1;34-9_2496;

2416 2452 41 1.000 1 3 KKA03052.1;model.g3217.t1;34-9_2499;

2417 2453 21 1.000 1 3 KKA03053.1;model.g3237.t1;34-9_2500;

2418 2454 24 1.000 1 3 KKA03058.1;model.g3247.t1;34-9_2503;

2420 2456 31 1.000 1 3 KKA03061.1;model.g3250.t1;34-9_2506;

2422 2458 50 1.000 1 3 KKA03064.1;model.g3236.t1;34-9_2509;

2424 2460 63 1.000 1 3 KKA03065.1;model.g3425.t1;34-9_2511;

2426 2462 23 1.000 1 3 KKA03067.1;model.g3219.t1;34-9_2513;

2428 2464 19 1.000 1 3 KKA03069.1;model.g3221.t1;34-9_2515;

2429 2465 28 1.000 1 3 KKA03070.1;model.g3222.t1;34-9_2516;

2432 2468 31 1.000 1 3 KKA03073.1;model.g3227.t1;34-9_2519;

2437 2473 16 1.000 1 3 34-9_3274;model.g679.t1;KKA01836.1;

2439 2476 69 1.000 1 3 KKA03080.1;model.g3151.t1;34-9_2528;

2441 2478 16 1.000 1 3 KKA03083.1;model.g2086.t1;34-9_2531;

2443 2480 22 1.000 1 3 KKA03086.1;model.g803.t1;34-9_2534;

2444 2481 62 1.000 1 3 KKA03087.1;model.g3172.t1;34-9_2535;

2446 2483 62 1.000 1 3 KKA03089.1;model.g3167.t1;34-9_2537;

2449 2486 90 1.000 1 3 KKA03095.1;model.g3164.t1;34-9_2542;

2452 2489 58 1.000 1 3 KKA03102.1;model.g3441.t1;34-9_2549;

2457 2494 39 1.000 1 3 KKA03116.1;model.g3439.t1;34-9_2564;

2460 2497 12 1.000 1 3 KKA03119.1;model.g3411.t1;34-9_2567;

2464 2501 13 1.000 1 3 KKA03122.1;model.g3418.t1;34-9_2572;

2466 2503 37 1.000 1 3 KKA03125.1;model.g3413.t1;34-9_2575;

2472 2509 33 1.000 1 3 KKA03132.1;model.g3397.t1;34-9_2582;

2475 2512 28 1.000 1 3 KKA03134.1;model.g802.t1;34-9_2585;

2482 2519 39 1.000 1 3 KKA01208.1;model.g3345.t1;34-9_2599;

2484 2521 14 1.000 1 3 KKA01205.1;model.g3341.t1;34-9_2602;

2485 2522 57 1.000 1 3 KKA01204.1;model.g3501.t1;34-9_2603;

2486 2523 23 1.000 1 3 KKA01203.1;model.g3502.t1;34-9_2604;

2488 2525 60 1.000 1 3 KKA01202.1;model.g778.t1;34-9_2606;

2489 2526 18 1.000 1 3 KKA01201.1;model.g779.t1;34-9_2607;

2490 2527 26 1.000 1 3 KKA01200.1;model.g3498.t1;34-9_2608;

2493 2530 75 1.000 1 3 KKA01196.1;model.g3495.t1;34-9_2612;

2494 2531 28 1.000 1 3 KKA01193.1;model.g3492.t1;34-9_2615;

2496 2533 19 1.000 1 3 KKA01190.1;model.g3474.t1;34-9_2618;

2497 2534 15 1.000 1 3 KKA01189.1;model.g3475.t1;34-9_2619;

2498 2535 57 1.000 1 3 KKA01188.1;model.g3479.t1;34-9_2620;

2499 2536 16 1.000 1 3 KKA01187.1;model.g3480.t1;34-9_2621;

2500 2537 33 1.000 1 3 KKA01186.1;model.g3486.t1;34-9_2622;

2503 2540 37 1.000 1 3 KKA01131.1;model.g3481.t1;34-9_2627;

2504 2541 41 1.000 1 3 KKA01130.1;model.g3478.t1;34-9_2628;

2507 2544 29 1.000 1 3 KKA01124.1;model.g68.t1;34-9_2634;

2510 2547 53 1.000 1 3 KKA02163.1;model.g1062.t1;34-9_2646;

2512 2549 24 1.000 1 3 KKA02161.1;model.g1065.t1;34-9_2648;

2513 2550 29 1.000 1 3 KKA02160.1;model.g1067.t1;34-9_2650;

2517 2554 23 1.000 1 3 KKA02157.1;model.g1072.t1;34-9_2655;

2526 2563 52 1.000 1 3 KKA02145.1;model.g3449.t1;34-9_2669;

2528 2565 50 1.000 1 3 KKA02143.1;model.g3455.t1;34-9_2673;

2529 2566 14 1.000 1 3 KKA02142.1;model.g1086.t1;34-9_2675;

2530 2567 29 1.000 1 3 KKA02141.1;model.g1088.t1;34-9_2677;

2532 2569 24 1.000 1 3 KKA02138.1;model.g1663.t1;34-9_2680;

2536 2573 34 1.000 1 3 KKA02136.1;model.g1095.t1;34-9_2686;

2538 2575 6 0.502 1 3 KKA02134.1;model.g1089.t1;34-9_2689;

2541 2578 24 1.000 1 3 KKA02129.1;model.g1155.t1;34-9_2695;

2548 2585 15 1.000 1 3 KKA02124.1;model.g1141.t1;34-9_2703;

2551 2588 5 0.502 1 3 KKA02122.1;model.g1138.t1;34-9_2706;

2553 2590 62 1.000 1 3 KKA02121.1;model.g1136.t1;34-9_2708;

2555 2592 18 1.000 1 3 KKA02119.1;model.g1135.t1;34-9_2710;

2558 2595 31 1.000 1 3 KKA02115.1;model.g1125.t1;34-9_2715;

2559 2596 52 1.000 1 3 KKA02114.1;model.g1124.t1;34-9_2716;

2561 2598 49 1.000 1 3 KKA02111.1;model.g1121.t1;34-9_2719;

2562 2599 51 1.000 1 3 KKA02109.1;model.g1118.t1;34-9_2722;

2564 2601 39 1.000 1 3 KKA02107.1;model.g1116.t1;34-9_2724;

2568 2605 68 1.000 1 3 KKA02099.1;model.g1106.t1;34-9_2731;

2569 2606 25 1.000 1 3 KKA01063.1;model.g1102.t1;34-9_2733;

2570 2607 46 1.000 1 3 KKA01064.1;model.g1706.t1;34-9_2734;

2571 2608 41 1.000 1 3 KKA01065.1;model.g1099.t1;34-9_2735;

2572 2609 81 1.000 1 3 KKA01066.1;model.g1098.t1;34-9_2736;

2577 2614 62 1.000 1 3 KKA03235.1;model.g2224.t1;34-9_2756;

2582 2619 7 0.502 1 3 KKA03240.1;model.g2217.t1;34-9_2762;

2587 2624 11 1.000 1 3 KKA03257.1;model.g628.t1;34-9_2790;

2588 2625 9 0.502 1 3 KKA03258.1;model.g624.t1;34-9_2791;

2590 2627 52 1.000 1 3 KKA03262.1;model.g632.t1;34-9_2794;

2592 2629 22 1.000 1 3 KKA03265.1;model.g2230.t1;34-9_2802;

2594 2631 30 1.000 1 3 KKA03266.1;model.g2235.t1;34-9_2806;

2600 2637 36 1.000 1 3 KKA03269.1;model.g3555.t1;34-9_2817;

2605 2642 15 1.000 1 3 KKA03276.1;model.g639.t1;34-9_2823;

2607 2644 82 1.000 1 3 KKA01803.1;model.g3567.t1;34-9_2834;

2608 2645 23 1.000 1 3 KKA01802.1;model.g3566.t1;34-9_2835;

2609 2646 20 1.000 1 3 KKA00989.1;model.g815.t1;34-9_2836;

2610 2647 19 1.000 1 3 KKA01148.1;model.g816.t1;34-9_2837;

2611 2648 17 1.000 1 3 KKA01149.1;model.g814.t1;34-9_2838;

2613 2650 18 1.000 1 3 KKA01152.1;model.g1541.t1;34-9_2841;

2618 2655 48 1.000 1 3 KKA01159.1;model.g1533.t1;34-9_2849;

2619 2656 52 1.000 1 3 KKA01160.1;model.g1532.t1;34-9_2850;

2622 2659 64 1.000 1 3 KKA01162.1;model.g1526.t1;34-9_2853;

2628 2665 37 1.000 1 3 KKA01291.1;model.g1518.t1;34-9_2860;

2629 2666 31 1.000 1 3 KKA01289.1;model.g1513.t1;34-9_2863;

2630 2667 24 1.000 1 3 KKA01288.1;model.g1514.t1;34-9_2864;

2631 2668 38 1.000 1 3 KKA01284.1;model.g702.t1;34-9_2866;

2632 2669 25 1.000 1 3 KKA01281.1;model.g700.t1;34-9_2868;

2634 2672 58 1.000 1 3 KKA01587.1;model.g3328.t1;34-9_3314;

2635 2673 10 1.000 1 3 KKA01279.1;model.g2991.t1;34-9_2870;

2636 2674 85 1.000 1 3 KKA01278.1;model.g858.t1;34-9_2871;

2638 2676 59 1.000 1 3 KKA01273.1;model.g954.t1;34-9_2875;

2639 2677 19 1.000 1 3 KKA01269.1;model.g218.t1;34-9_2880;

2640 2678 75 1.000 1 3 KKA01471.1;model.g2747.t1;34-9_2884;

2641 2679 21 1.000 1 3 KKA01474.1;model.g2744.t1;34-9_2887;

2642 2680 28 1.000 1 3 KKA01475.1;model.g2743.t1;34-9_2888;

2643 2681 34 1.000 1 3 KKA01476.1;model.g2742.t1;34-9_2889;

2647 2685 36 1.000 1 3 KKA01481.1;model.g2735.t1;34-9_2895;

2653 2691 21 1.000 1 3 KKA01488.1;model.g2725.t1;34-9_2904;

2654 2692 35 1.000 1 3 KKA01489.1;model.g2724.t1;34-9_2905;

2655 2693 14 1.000 1 3 KKA01490.1;model.g2723.t1;34-9_2906;

2657 2695 10 1.000 1 3 KKA01491.1;model.g2722.t1;34-9_2907;

2658 2696 42 1.000 1 3 KKA01495.1;model.g2718.t1;34-9_2910;

2659 2697 13 1.000 1 3 KKA01496.1;model.g2717.t1;34-9_2911;

2661 2699 44 1.000 1 3 KKA01498.1;model.g2715.t1;34-9_2913;

2662 2700 38 1.000 1 3 KKA01499.1;model.g1848.t1;34-9_2914;

2663 2701 20 1.000 1 3 KKA01500.1;model.g2713.t1;34-9_2915;

2666 2704 39 1.000 1 3 KKA01503.1;model.g1380.t1;34-9_2918;

2670 2708 43 1.000 1 3 34-9_3620;KKA01560.1;model.g1824.t1;

2671 2709 16 1.000 1 3 KKA01080.1;model.g1376.t1;34-9_2923;

2672 2710 45 1.000 1 3 KKA01079.1;model.g1374.t1;34-9_2924;

2676 2714 44 1.000 1 3 KKA01073.1;model.g1366.t1;34-9_2929;

2677 2715 23 1.000 1 3 KKA01072.1;model.g1365.t1;34-9_2930;

2681 2720 79 1.000 1 3 KKA01183.1;model.g1358.t1;34-9_2935;

2682 2721 22 1.000 1 3 KKA01182.1;model.g1357.t1;34-9_2936;

2685 2724 11 1.000 1 3 KKA01180.1;model.g1353.t1;34-9_2939;

2686 2725 11 1.000 1 3 KKA01179.1;model.g1351.t1;34-9_2940;

2690 2729 17 1.000 1 3 KKA01176.1;model.g1345.t1;34-9_2945;

2692 2732 15 1.000 1 3 KKA01174.1;model.g1342.t1;34-9_2949;

2693 2733 31 1.000 1 3 KKA01173.1;model.g1341.t1;34-9_2950;

2694 2734 25 1.000 1 3 KKA01172.1;model.g1340.t1;34-9_2951;

2696 2736 59 1.000 1 3 KKA01169.1;model.g1338.t1;34-9_2953;

2697 2737 40 1.000 1 3 KKA01168.1;model.g1337.t1;34-9_2954;

2699 2739 16 1.000 1 3 KKA01619.1;model.g1332.t1;34-9_2960;

2702 2742 25 1.000 1 3 KKA01620.1;model.g1335.t1;34-9_2964;

2704 2744 20 1.000 1 3 KKA01621.1;model.g1325.t1;34-9_2966;

2707 2747 30 1.000 1 3 KKA01623.1;model.g1317.t1;34-9_2972;

2708 2748 61 1.000 1 3 34-9_3149;model.g118.t1;KKA02896.1;

2709 2749 30 1.000 1 3 34-9_3652;KKA01319.1;model.g3624.t1;

2713 2753 5 0.502 1 3 KKA01633.1;model.g214.t1;34-9_2987;

2714 2754 24 1.000 1 3 KKA01634.1;model.g2754.t1;34-9_2988;

2719 2759 16 1.000 1 3 KKA01646.1;model.g1024.t1;34-9_3002;

2726 2766 34 1.000 1 3 KKA02966.1;model.g1312.t1;34-9_3013;

2738 2778 39 1.000 1 3 KKA02952.1;model.g978.t1;34-9_3036;

2739 2779 50 1.000 1 3 KKA02951.1;model.g980.t1;34-9_3038;

2741 2781 22 1.000 1 3 KKA02947.1;model.g982.t1;34-9_3041;

2748 2788 35 1.000 1 3 KKA02940.1;model.g994.t1;34-9_3053;

2751 2791 5 0.502 1 3 KKA02937.1;model.g2761.t1;34-9_3059;

2752 2792 36 1.000 1 3 KKA02936.1;model.g2765.t1;34-9_3061;

2753 2793 40 1.000 1 3 KKA02935.1;model.g2763.t1;34-9_3064;

2756 2796 21 1.000 1 3 KKA02933.1;model.g2769.t1;34-9_3067;

2760 2800 26 1.000 1 3 KKA02927.1;model.g2776.t1;34-9_3075;

2767 2807 15 1.000 1 3 KKA02921.1;model.g2796.t1;34-9_3089;

2772 2812 46 1.000 1 3 KKA02917.1;model.g45.t1;34-9_3095;

2784 2824 12 1.000 1 3 KKA02908.1;model.g77.t1;34-9_3117;

2789 2829 34 1.000 1 3 KKA02902.1;model.g475.t1;34-9_3131;

2791 2831 20 1.000 1 3 KKA02901.1;model.g97.t1;34-9_3133;

2796 2836 15 1.000 1 3 KKA02899.1;model.g109.t1;34-9_3140;

2804 2844 30 1.000 1 3 KKA02890.1;model.g127.t1;34-9_3158;

2807 2847 46 1.000 1 3 KKA02882.1;model.g1602.t1;34-9_3167;

2816 2856 28 1.000 1 3 KKA02872.1;model.g1619.t1;34-9_3181;

2817 2857 28 1.000 1 3 34-9_3573;KKA02415.1;model.g1762.t1;

2819 2859 16 1.000 1 3 KKA02561.1;model.g1627.t1;34-9_3186;

2821 2861 67 1.000 1 3 KKA02558.1;model.g3315.t1;34-9_3188;

2822 2862 70 1.000 1 3 KKA02557.1;model.g3319.t1;34-9_3190;

2829 2869 46 1.000 1 3 KKA02542.1;model.g593.t1;34-9_3210;

2835 2876 23 1.000 1 3 KKA02537.1;model.g605.t1;34-9_3218;

2838 2879 65 1.000 1 3 KKA02536.1;model.g609.t1;34-9_3222;

2843 2884 44 1.000 1 3 KKA02532.1;model.g133.t1;34-9_3228;

2844 2885 5 0.502 1 3 KKA02531.1;model.g179.t1;34-9_3229;

2845 2886 22 1.000 1 3 KKA02530.1;model.g180.t1;34-9_3230;

2846 2887 22 1.000 1 3 KKA02524.1;model.g2538.t1;34-9_3239;

2847 2889 13 1.000 1 3 KKA02521.1;model.g2532.t1;34-9_3241;

2848 2890 13 1.000 1 3 KKA02520.1;model.g2531.t1;34-9_3242;

2851 2893 8 0.502 1 3 model.g2527.t1;KKA02519.1;34-9_3245;

2854 2896 19 1.000 1 3 KKA02514.1;model.g247.t1;34-9_3253;

2856 2898 50 1.000 1 3 KKA02513.1;model.g243.t1;34-9_3256;

2858 2900 21 1.000 1 3 KKA01849.1;model.g675.t1;34-9_3261;

2860 2902 44 1.000 1 3 KKA01847.1;model.g676.t1;34-9_3263;

2861 2903 58 1.000 1 3 KKA01846.1;model.g677.t1;34-9_3264;

2866 2908 55 1.000 1 3 KKA01835.1;model.g678.t1;34-9_3276;

2869 2911 26 1.000 1 3 KKA01823.1;model.g1449.t1;34-9_3288;

2870 2913 37 1.000 1 3 KKA01822.1;model.g1448.t1;34-9_3289;

2871 2914 24 1.000 1 3 KKA01821.1;model.g1423.t1;34-9_3290;

2872 2915 49 1.000 1 3 KKA01820.1;model.g1422.t1;34-9_3291;

2875 2918 85 1.000 1 3 KKA01817.1;model.g1419.t1;34-9_3294;

2877 2920 21 1.000 1 3 KKA01815.1;model.g1430.t1;34-9_3296;

2880 2923 26 1.000 1 3 KKA01810.1;model.g1436.t1;34-9_3300;

2881 2924 13 1.000 1 3 KKA01809.1;model.g1435.t1;34-9_3301;

2883 2926 23 1.000 1 3 KKA01807.1;model.g1437.t1;34-9_3303;

2884 2927 29 1.000 1 3 KKA01806.1;model.g1438.t1;34-9_3304;

2892 2935 59 1.000 1 3 KKA01588.1;model.g3329.t1;34-9_3315;

2893 2936 17 1.000 1 3 KKA01589.1;model.g3330.t1;34-9_3316;

2904 2947 34 1.000 1 3 KKA01613.1;model.g3537.t1;34-9_3350;

2905 2948 61 1.000 1 3 KKA01614.1;model.g3536.t1;34-9_3351;

2907 2950 13 1.000 1 3 KKA01615.1;model.g1424.t1;34-9_3353;

2912 2955 38 1.000 1 3 KKA02720.1;model.g1589.t1;34-9_3361;

2916 2959 23 1.000 1 3 KKA02715.1;model.g732.t1;34-9_3370;

2920 2963 14 1.000 1 3 KKA02712.1;model.g724.t1;34-9_3375;

2921 2964 84 1.000 1 3 KKA02709.1;model.g660.t1;34-9_3377;

2926 2970 53 1.000 1 3 KKA02704.1;model.g1557.t1;34-9_3385;

2927 2971 44 1.000 1 3 KKA02702.1;model.g1512.t1;34-9_3388;

2928 2972 33 1.000 1 3 KKA02701.1;model.g1573.t1;34-9_3389;

2931 2975 23 1.000 1 3 KKA02698.1;model.g1464.t1;34-9_3393;

2934 2978 41 1.000 1 3 KKA02696.1;model.g670.t1;34-9_3396;

2942 2986 75 1.000 1 3 KKA02689.1;model.g1581.t1;34-9_3406;

2943 2987 16 1.000 1 3 KKA02687.1;model.g1579.t1;34-9_3407;

2946 2990 12 1.000 1 3 KKA02686.1;model.g1576.t1;34-9_3410;

2947 2991 33 1.000 1 3 KKA02685.1;model.g1577.t1;34-9_3411;

2950 2994 66 1.000 1 3 KKA02682.1;model.g1470.t1;34-9_3415;

2951 2995 11 1.000 1 3 KKA02681.1;model.g1468.t1;34-9_3416;

2953 2997 14 1.000 1 3 KKA02680.1;model.g1475.t1;34-9_3418;

2954 2998 72 1.000 1 3 KKA02679.1;model.g1476.t1;34-9_3419;

2956 3000 55 1.000 1 3 KKA02678.1;model.g1478.t1;34-9_3421;

2960 3004 22 1.000 1 3 KKA02676.1;model.g1484.t1;34-9_3425;

2974 3018 14 1.000 1 3 KKA02668.1;model.g1506.t1;34-9_3442;

2979 3023 13 1.000 1 3 KKA02656.1;model.g2243.t1;34-9_3456;

2980 3024 46 1.000 1 3 KKA02655.1;model.g2244.t1;34-9_3457;

2981 3025 21 1.000 1 3 KKA02654.1;model.g3538.t1;34-9_3458;

2983 3027 24 1.000 1 3 KKA02307.1;model.g230.t1;34-9_3470;

2986 3030 15 1.000 1 3 KKA02315.1;model.g3069.t1;34-9_3477;

2987 3031 54 1.000 1 3 KKA02316.1;model.g2473.t1;34-9_3478;

2988 3032 77 1.000 1 3 KKA02318.1;model.g2475.t1;34-9_3480;

2991 3035 36 1.000 1 3 KKA02324.1;model.g2483.t1;34-9_3487;

2992 3036 19 1.000 1 3 KKA02325.1;model.g2482.t1;34-9_3488;

2993 3037 16 1.000 1 3 KKA02330.1;model.g647.t1;34-9_3493;

2994 3038 48 1.000 1 3 KKA02331.1;model.g646.t1;34-9_3494;

2995 3039 53 1.000 1 3 KKA02333.1;model.g1994.t1;34-9_3497;

2996 3040 51 1.000 1 3 KKA02335.1;model.g1992.t1;34-9_3499;

2997 3041 31 1.000 1 3 KKA02336.1;model.g1989.t1;34-9_3500;

2998 3042 12 1.000 1 3 KKA02338.1;model.g1987.t1;34-9_3502;

3000 3044 53 1.000 1 3 KKA02339.1;model.g1985.t1;34-9_3504;

3001 3045 31 1.000 1 3 KKA02340.1;model.g1984.t1;34-9_3505;

3003 3047 22 1.000 1 3 KKA02343.1;model.g1980.t1;34-9_3508;

3004 3048 64 1.000 1 3 KKA02344.1;model.g1979.t1;34-9_3509;

3005 3049 56 1.000 1 3 KKA02347.1;model.g1976.t1;34-9_3512;

3009 3053 16 1.000 1 3 KKA02365.1;model.g2439.t1;34-9_3525;

3011 3055 38 1.000 1 3 KKA02370.1;model.g2450.t1;34-9_3529;

3014 3058 36 1.000 1 3 KKA02373.1;model.g1809.t1;34-9_3533;

3015 3059 10 1.000 1 3 KKA02376.1;model.g1812.t1;34-9_3536;

3016 3060 41 1.000 1 3 KKA02378.1;model.g1815.t1;34-9_3537;

3018 3062 24 1.000 1 3 KKA02379.1;model.g1816.t1;34-9_3538;

3019 3063 29 1.000 1 3 KKA02381.1;model.g1818.t1;34-9_3540;

3020 3064 35 1.000 1 3 KKA02382.1;model.g1817.t1;34-9_3541;

3021 3065 10 1.000 1 3 KKA02384.1;model.g3654.t1;34-9_3543;

3022 3066 29 1.000 1 3 KKA02385.1;model.g3655.t1;34-9_3544;

3025 3069 11 1.000 1 3 KKA02393.1;model.g3613.t1;34-9_3551;

3027 3071 31 1.000 1 3 KKA02404.1;model.g1775.t1;34-9_3561;

3028 3072 71 1.000 1 3 KKA02405.1;model.g1774.t1;34-9_3562;

3030 3074 22 1.000 1 3 KKA02407.1;model.g1771.t1;34-9_3565;

3031 3075 19 1.000 1 3 KKA02408.1;model.g1770.t1;34-9_3566;

3033 3077 22 1.000 1 3 KKA02410.1;model.g1768.t1;34-9_3568;

3035 3079 46 1.000 1 3 KKA02412.1;model.g1766.t1;34-9_3570;

3036 3080 21 1.000 1 3 KKA02413.1;model.g1765.t1;34-9_3571;

3037 3081 90 1.000 1 3 KKA02416.1;model.g1760.t1;34-9_3575;

3039 3083 39 1.000 1 3 KKA02417.1;model.g1759.t1;34-9_3576;

3040 3084 13 1.000 1 3 KKA02418.1;model.g1758.t1;34-9_3577;

3044 3088 54 1.000 1 3 KKA01536.1;model.g1756.t1;34-9_3584;

3046 3090 67 1.000 1 3 KKA01538.1;model.g1778.t1;34-9_3588;

3047 3091 47 1.000 1 3 KKA01539.1;model.g1779.t1;34-9_3589;

3049 3093 20 1.000 1 3 KKA01542.1;model.g1783.t1;34-9_3592;

3050 3094 44 1.000 1 3 KKA01543.1;model.g1785.t1;34-9_3594;

3051 3095 80 1.000 1 3 KKA01544.1;model.g1787.t1;34-9_3595;

3052 3096 17 1.000 1 3 KKA01546.1;model.g1790.t1;34-9_3598;

3055 3100 45 1.000 1 3 KKA01547.1;model.g1796.t1;34-9_3602;

3056 3101 20 1.000 1 3 KKA01548.1;model.g1798.t1;34-9_3603;

3057 3102 54 1.000 1 3 KKA01549.1;model.g1799.t1;34-9_3604;

3058 3103 25 1.000 1 3 KKA01550.1;model.g1800.t1;34-9_3605;

3061 3106 5 0.502 1 3 KKA01552.1;model.g1804.t1;34-9_3608;

3062 3107 69 1.000 1 3 KKA01555.1;model.g2560.t1;34-9_3614;

3064 3109 30 1.000 1 3 KKA01557.1;model.g645.t1;34-9_3618;

3068 3113 38 1.000 1 3 KKA01295.1;model.g2553.t1;34-9_3626;

3071 3116 44 1.000 1 3 KKA01306.1;model.g3666.t1;34-9_3639;

3072 3117 79 1.000 1 3 KKA01308.1;model.g3664.t1;34-9_3641;

3078 3123 37 1.000 1 3 KKA01325.1;model.g3634.t1;34-9_3657;

3079 3124 72 1.000 1 3 KKA01326.1;model.g3635.t1;34-9_3658;

3081 3126 27 1.000 1 3 KKA03155.1;model.g906.t1;34-9_3667;

3088 3133 18 1.000 1 3 KKA03146.1;model.g1722.t1;34-9_3678;

3090 3135 49 1.000 1 3 KKA03144.1;model.g1720.t1;34-9_3680;

3092 3137 20 1.000 1 3 KKA03143.1;model.g1735.t1;34-9_3682;

3093 3139 51 1.000 1 3 KKA03141.1;model.g1729.t1;34-9_3684;

3094 3140 64 1.000 1 3 KKA03140.1;model.g1741.t1;34-9_3685;

3102 3148 7 0.502 1 3 model.g1254.t1;KKA02447.1;34-9_3700;

3108 3154 20 1.000 1 3 KKA01462.1;model.g1738.t1;34-9_3715;

3111 3157 41 1.000 1 3 KKA01465.1;model.g1736.t1;34-9_3721;

3112 3158 66 1.000 1 3 KKA01466.1;model.g889.t1;34-9_3722;

3113 3159 35 1.000 1 3 KKA01467.1;model.g890.t1;34-9_3723;

3115 3161 47 1.000 1 3 KKA01468.1;model.g894.t1;34-9_3726;

3117 3163 65 1.000 1 3 KKA01469.1;model.g896.t1;34-9_3728;

3121 3167 18 1.000 1 3 KKA01137.1;model.g2805.t1;34-9_3738;

3122 3168 55 1.000 1 3 KKA01139.1;model.g2807.t1;34-9_3740;

3123 3169 15 1.000 1 3 KKA01140.1;model.g2806.t1;34-9_3741;

3124 3170 53 1.000 1 3 KKA01141.1;model.g2809.t1;34-9_3742;

3125 3171 59 1.000 1 3 KKA01142.1;model.g2810.t1;34-9_3743;

3127 3173 40 1.000 1 3 KKA01147.1;model.g2817.t1;34-9_3747;

3128 3174 10 1.000 1 3 KKA01330.1;model.g1658.t1;34-9_3749;

3129 3175 84 1.000 1 3 KKA01331.1;model.g1659.t1;34-9_3750;

3130 3176 29 1.000 1 3 KKA01332.1;model.g1660.t1;34-9_3751;

3131 3177 24 1.000 1 3 KKA01334.1;model.g1636.t1;34-9_3753;

3132 3178 22 1.000 1 3 KKA01336.1;model.g1631.t1;34-9_3755;

3134 3180 14 1.000 1 3 KKA01339.1;model.g3312.t1;34-9_3758;

3135 3181 68 1.000 1 3 KKA01341.1;model.g3302.t1;34-9_3760;

3136 3182 18 1.000 1 3 KKA01342.1;model.g3309.t1;34-9_3761;

3137 3183 25 1.000 1 3 KKA01343.1;model.g3306.t1;34-9_3762;

3140 3186 67 1.000 1 3 KKA01350.1;model.g3301.t1;34-9_3769;

3144 3190 10 1.000 1 3 KKA01354.1;model.g3296.t1;34-9_3774;

3145 3191 36 1.000 1 3 KKA01356.1;model.g3294.t1;34-9_3776;

3147 3193 6 0.502 1 3 KKA01357.1;model.g3288.t1;34-9_3778;

3148 3194 14 0.502 1 3 KKA01358.1;model.g3292.t1;34-9_3779;

3149 3195 40 1.000 1 3 KKA01359.1;model.g3291.t1;34-9_3780;

3151 3197 42 1.000 1 3 KKA01360.1;model.g3289.t1;34-9_3782;

3153 3199 60 1.000 1 3 KKA01362.1;model.g3285.t1;34-9_3784;
