# Supplementary material for: The Shared and Specific Genes and a Comparative Genomics Analysis within Three Hanseniaspora Strains
Source: Int J Genomics. 2019 Jun 2;2019:7910865. doi: 10.1155/2019/7910865 (PMC6589277; doi:10.1155/2019/7910865)
Supplement: Supplementary 6 — File 5: Lists of the specific genes of K. apiculata 34-9, H. uvarum DSM 2768, and H. vineae T02/19AF. [file 7910865.f6.docx]

**Supplementary file 5.** Lists of the specific genes of *K. apiculata* 34-9, *H.* *uvarum* DSM 2768 and *H.* *vineae* T02/19AF.

**Gene ID of 34-9_not-in-family**

34-9_0002

34-9_0004

34-9_0007

34-9_0008

34-9_0009

34-9_0010

34-9_0011

34-9_0015

34-9_0016

34-9_0017

34-9_0018

34-9_0054

34-9_0088

34-9_0130

34-9_0141

34-9_0146

34-9_0160

34-9_0172

34-9_0178

34-9_0183

34-9_0195

34-9_0207

34-9_0225

34-9_0284

34-9_0287

34-9_0292

34-9_0293

34-9_0296

34-9_0298

34-9_0305

34-9_0310

34-9_0311

34-9_0314

34-9_0316

34-9_0317

34-9_0318

34-9_0319

34-9_0322

34-9_0328

34-9_0329

34-9_0336

34-9_0346

34-9_0347

34-9_0350

34-9_0355

34-9_0366

34-9_0367

34-9_0369

34-9_0374

34-9_0375

34-9_0392

34-9_0396

34-9_0421

34-9_0434

34-9_0445

34-9_0454

34-9_0480

34-9_0492

34-9_0506

34-9_0527

34-9_0547

34-9_0548

34-9_0562

34-9_0563

34-9_0568

34-9_0578

34-9_0581

34-9_0585

34-9_0597

34-9_0606

34-9_0645

34-9_0676

34-9_0683

34-9_0690

34-9_0693

34-9_0739

34-9_0797

34-9_0820

34-9_0836

34-9_0921

34-9_0974

34-9_0987

34-9_1068

34-9_1099

34-9_1103

34-9_1162

34-9_1179

34-9_1194

34-9_1217

34-9_1220

34-9_1278

34-9_1280

34-9_1308

34-9_1357

34-9_1387

34-9_1408

34-9_1446

34-9_1459

34-9_1466

34-9_1474

34-9_1483

34-9_1491

34-9_1496

34-9_1503

34-9_1509

34-9_1510

34-9_1511

34-9_1512

34-9_1516

34-9_1525

34-9_1530

34-9_1531

34-9_1543

34-9_1606

34-9_1612

34-9_1637

34-9_1657

34-9_1673

34-9_1678

34-9_1691

34-9_1696

34-9_1723

34-9_1753

34-9_1755

34-9_1767

34-9_1781

34-9_1798

34-9_1833

34-9_1845

34-9_1856

34-9_1857

34-9_1862

34-9_1870

34-9_1875

34-9_1878

34-9_1900

34-9_1922

34-9_1929

34-9_1938

34-9_1942

34-9_1944

34-9_1945

34-9_1948

34-9_1962

34-9_1987

34-9_1996

34-9_1997

34-9_2016

34-9_2028

34-9_2033

34-9_2045

34-9_2051

34-9_2077

34-9_2080

34-9_2094

34-9_2101

34-9_2102

34-9_2113

34-9_2116

34-9_2130

34-9_2173

34-9_2231

34-9_2253

34-9_2268

34-9_2282

34-9_2396

34-9_2444

34-9_2455

34-9_2463

34-9_2483

34-9_2485

34-9_2558

34-9_2581

34-9_2591

34-9_2593

34-9_2595

34-9_2624

34-9_2653

34-9_2674

34-9_2676

34-9_2698

34-9_2732

34-9_2737

34-9_2740

34-9_2765

34-9_2773

34-9_2779

34-9_2789

34-9_2807

34-9_2815

34-9_2824

34-9_2826

34-9_2827

34-9_2828

34-9_2844

34-9_2862

34-9_2882

34-9_2883

34-9_2885

34-9_2925

34-9_2946

34-9_2948

34-9_2956

34-9_2961

34-9_2967

34-9_2971

34-9_2974

34-9_2979

34-9_2981

34-9_3009

34-9_3016

34-9_3017

34-9_3018

34-9_3042

34-9_3044

34-9_3055

34-9_3074

34-9_3097

34-9_3098

34-9_3121

34-9_3123

34-9_3128

34-9_3141

34-9_3146

34-9_3147

34-9_3165

34-9_3184

34-9_3189

34-9_3196

34-9_3209

34-9_3224

34-9_3248

34-9_3250

34-9_3254

34-9_3259

34-9_3272

34-9_3284

34-9_3285

34-9_3343

34-9_3365

34-9_3369

34-9_3378

34-9_3387

34-9_3437

34-9_3445

34-9_3461

34-9_3466

34-9_3574

34-9_3585

34-9_3593

34-9_3611

34-9_3621

34-9_3649

34-9_3663

34-9_3665

34-9_3671

34-9_3683

34-9_3687

34-9_3707

34-9_3708

34-9_3710

34-9_3713

34-9_3719

34-9_3730

34-9_3732

34-9_3735

34-9_3773

**apiculata_unique_orignal_name**

1930 1959 31 1.000 1 2 34-9_1636;34-9_2643;

2710 2750 56 1.000 1 3 34-9_2977;34-9_2978;34-9_2976;

2984 3028 35 1.000 1 3 34-9_3472;34-9_3473;34-9_3471;

**Gene ID of DSM not-in-family**

KKA03706.1

KKA03657.1

KKA03642.1

KKA03682.1

KKA03591.1

KKA03890.1

KKA03643.1

KKA03709.1

KKA03646.1

KKA03489.1

KKA03337.1

KKA03486.1

KKA03541.1

KKA03487.1

KKA03492.1

KKA03384.1

KKA03320.1

KKA03431.1

KKA03398.1

KKA03190.1

KKA03242.1

KKA03195.1

KKA03138.1

KKA03142.1

KKA03270.1

KKA03232.1

KKA03192.1

KKA03136.1

KKA03054.1

KKA03012.1

KKA02993.1

KKA02879.1

KKA02956.1

KKA02962.1

KKA02949.1

KKA02828.1

KKA02770.1

KKA02862.1

KKA02815.1

KKA02784.1

KKA02838.1

KKA02795.1

KKA02636.1

KKA02635.1

KKA02666.1

KKA02703.1

KKA02711.1

KKA02674.1

KKA02615.1

KKA02590.1

KKA02566.1

KKA02548.1

KKA02543.1

KKA02525.1

KKA02423.1

KKA02425.1

KKA02481.1

KKA02476.1

KKA02281.1

KKA02300.1

KKA02278.1

KKA02177.1

KKA02202.1

KKA02211.1

KKA02215.1

KKA02117.1

KKA02137.1

KKA02101.1

KKA02127.1

KKA02030.1

KKA02061.1

KKA02070.1

KKA02063.1

KKA02021.1

KKA01895.1

KKA01912.1

KKA01873.1

KKA01814.1

KKA01868.1

KKA01826.1

KKA01877.1

KKA01859.1

KKA01760.1

KKA01745.1

KKA01748.1

KKA01699.1

KKA01667.1

KKA01675.1

KKA01650.1

KKA01638.1

KKA01642.1

KKA01632.1

KKA01649.1

KKA01607.1

KKA01584.1

KKA01593.1

KKA01519.1

KKA01451.1

KKA01445.1

KKA01447.1

KKA01427.1

KKA01384.1

KKA01286.1

KKA01282.1

KKA01267.1

KKA01247.1

KKA01241.1

KKA01111.1

KKA01089.1

KKA01091.1

KKA01074.1

KKA01061.1

KKA01031.1

KKA00992.1

KKA00978.1

KKA00973.1

**DSM_unique_orignal_name**

503 518 100 1.000 1 2 KKA01002.1;KKA01006.1;

516 524 28 1.000 1 3 KKA00953.1;KKA03552.1;KKA00990.1;

3194 3240 100 1.000 1 2 KKA01685.1;KKA01448.1;

3203 3249 44 1.000 1 2 KKA01117.1;KKA00960.1;

3206 3252 68 1.000 1 2 KKA01118.1;KKA00961.1;

3211 3257 100 1.000 1 2 KKA01005.1;KKA01001.1;

**Gene ID of vineae_not-in-family**

model.g1.t1

model.g2.t1

model.g3.t1

model.g5.t1

model.g6.t1

model.g8.t1

model.g9.t1

model.g10.t1

model.g11.t1

model.g12.t1

model.g13.t1

model.g15.t1

model.g16.t1

model.g20.t1

model.g23.t1

model.g25.t1

model.g26.t1

model.g28.t1

model.g29.t1

model.g30.t1

model.g42.t1

model.g43.t1

model.g48.t1

model.g37.t1

model.g35.t1

model.g47.t1

model.g36.t1

model.g53.t1

model.g56.t1

model.g59.t1

model.g60.t1

model.g61.t1

model.g100.t1

model.g95.t1

model.g105.t1

model.g86.t1

model.g82.t1

model.g66.t1

model.g132.t1

model.g122.t1

model.g116.t1

model.g78.t1

model.g83.t1

model.g102.t1

model.g113.t1

model.g84.t1

model.g63.t1

model.g67.t1

model.g111.t1

model.g98.t1

model.g110.t1

model.g75.t1

model.g92.t1

model.g103.t1

model.g99.t1

model.g115.t1

model.g167.t1

model.g148.t1

model.g160.t1

model.g158.t1

model.g145.t1

model.g159.t1

model.g169.t1

model.g149.t1

model.g141.t1

model.g146.t1

model.g140.t1

model.g135.t1

model.g150.t1

model.g134.t1

model.g138.t1

model.g166.t1

model.g174.t1

model.g185.t1

model.g184.t1

model.g181.t1

model.g178.t1

model.g183.t1

model.g192.t1

model.g203.t1

model.g194.t1

model.g197.t1

model.g206.t1

model.g205.t1

model.g207.t1

model.g208.t1

model.g210.t1

model.g216.t1

model.g215.t1

model.g211.t1

model.g217.t1

model.g213.t1

model.g223.t1

model.g224.t1

model.g225.t1

model.g226.t1

model.g227.t1

model.g234.t1

model.g261.t1

model.g250.t1

model.g270.t1

model.g241.t1

model.g263.t1

model.g255.t1

model.g251.t1

model.g246.t1

model.g248.t1

model.g257.t1

model.g258.t1

model.g245.t1

model.g262.t1

model.g259.t1

model.g273.t1

model.g280.t1

model.g282.t1

model.g279.t1

model.g283.t1

model.g294.t1

model.g284.t1

model.g286.t1

model.g299.t1

model.g295.t1

model.g289.t1

model.g293.t1

model.g303.t1

model.g306.t1

model.g307.t1

model.g320.t1

model.g310.t1

model.g317.t1

model.g330.t1

model.g328.t1

model.g334.t1

model.g325.t1

model.g343.t1

model.g345.t1

model.g340.t1

model.g355.t1

model.g362.t1

model.g360.t1

model.g356.t1

model.g363.t1

model.g351.t1

model.g366.t1

model.g368.t1

model.g391.t1

model.g371.t1

model.g365.t1

model.g393.t1

model.g390.t1

model.g375.t1

model.g370.t1

model.g376.t1

model.g418.t1

model.g397.t1

model.g406.t1

model.g413.t1

model.g405.t1

model.g463.t1

model.g482.t1

model.g489.t1

model.g453.t1

model.g444.t1

model.g455.t1

model.g490.t1

model.g451.t1

model.g477.t1

model.g466.t1

model.g450.t1

model.g493.t1

model.g474.t1

model.g436.t1

model.g491.t1

model.g457.t1

model.g472.t1

model.g433.t1

model.g458.t1

model.g485.t1

model.g428.t1

model.g486.t1

model.g434.t1

model.g476.t1

model.g461.t1

model.g449.t1

model.g479.t1

model.g556.t1

model.g572.t1

model.g518.t1

model.g529.t1

model.g497.t1

model.g568.t1

model.g510.t1

model.g527.t1

model.g513.t1

model.g494.t1

model.g544.t1

model.g565.t1

model.g553.t1

model.g516.t1

model.g526.t1

model.g506.t1

model.g571.t1

model.g536.t1

model.g528.t1

model.g578.t1

model.g537.t1

model.g548.t1

model.g554.t1

model.g517.t1

model.g507.t1

model.g542.t1

model.g519.t1

model.g511.t1

model.g583.t1

model.g582.t1

model.g584.t1

model.g580.t1

model.g590.t1

model.g594.t1

model.g591.t1

model.g611.t1

model.g614.t1

model.g618.t1

model.g599.t1

model.g601.t1

model.g613.t1

model.g610.t1

model.g596.t1

model.g640.t1

model.g636.t1

model.g641.t1

model.g626.t1

model.g635.t1

model.g622.t1

model.g630.t1

model.g652.t1

model.g653.t1

model.g651.t1

model.g644.t1

model.g654.t1

model.g658.t1

model.g655.t1

model.g667.t1

model.g684.t1

model.g662.t1

model.g661.t1

model.g686.t1

model.g694.t1

model.g685.t1

model.g687.t1

model.g693.t1

model.g680.t1

model.g726.t1

model.g723.t1

model.g718.t1

model.g703.t1

model.g706.t1

model.g696.t1

model.g709.t1

model.g698.t1

model.g712.t1

model.g727.t1

model.g710.t1

model.g730.t1

model.g722.t1

model.g720.t1

model.g719.t1

model.g714.t1

model.g699.t1

model.g728.t1

model.g734.t1

model.g736.t1

model.g733.t1

model.g740.t1

model.g737.t1

model.g758.t1

model.g751.t1

model.g743.t1

model.g773.t1

model.g753.t1

model.g765.t1

model.g760.t1

model.g750.t1

model.g771.t1

model.g763.t1

model.g746.t1

model.g776.t1

model.g783.t1

model.g780.t1

model.g796.t1

model.g791.t1

model.g789.t1

model.g784.t1

model.g806.t1

model.g805.t1

model.g811.t1

model.g856.t1

model.g843.t1

model.g824.t1

model.g837.t1

model.g850.t1

model.g818.t1

model.g828.t1

model.g813.t1

model.g823.t1

model.g820.t1

model.g838.t1

model.g852.t1

model.g821.t1

model.g835.t1

model.g825.t1

model.g860.t1

model.g842.t1

model.g827.t1

model.g861.t1

model.g863.t1

model.g877.t1

model.g866.t1

model.g871.t1

model.g876.t1

model.g884.t1

model.g879.t1

model.g880.t1

model.g878.t1

model.g887.t1

model.g886.t1

model.g925.t1

model.g941.t1

model.g923.t1

model.g920.t1

model.g939.t1

model.g929.t1

model.g904.t1

model.g891.t1

model.g937.t1

model.g918.t1

model.g927.t1

model.g902.t1

model.g907.t1

model.g922.t1

model.g933.t1

model.g898.t1

model.g938.t1

model.g901.t1

model.g942.t1

model.g908.t1

model.g912.t1

model.g947.t1

model.g944.t1

model.g940.t1

model.g967.t1

model.g953.t1

model.g1011.t1

model.g959.t1

model.g964.t1

model.g970.t1

model.g1004.t1

model.g989.t1

model.g1009.t1

model.g999.t1

model.g983.t1

model.g1006.t1

model.g987.t1

model.g961.t1

model.g1007.t1

model.g1005.t1

model.g962.t1

model.g977.t1

model.g996.t1

model.g973.t1

model.g1021.t1

model.g1020.t1

model.g988.t1

model.g1039.t1

model.g1038.t1

model.g1037.t1

model.g1056.t1

model.g1053.t1

model.g1050.t1

model.g1049.t1

model.g1060.t1

model.g1064.t1

model.g1063.t1

model.g1074.t1

model.g1075.t1

model.g1070.t1

model.g1059.t1

model.g1068.t1

model.g1082.t1

model.g1101.t1

model.g1087.t1

model.g1091.t1

model.g1105.t1

model.g1092.t1

model.g1103.t1

model.g1085.t1

model.g1131.t1

model.g1142.t1

model.g1109.t1

model.g1117.t1

model.g1147.t1

model.g1114.t1

model.g1130.t1

model.g1149.t1

model.g1134.t1

model.g1108.t1

model.g1127.t1

model.g1157.t1

model.g1151.t1

model.g1144.t1

model.g1150.t1

model.g1126.t1

model.g1143.t1

model.g1113.t1

model.g1129.t1

model.g1140.t1

model.g1110.t1

model.g1122.t1

model.g1169.t1

model.g1170.t1

model.g1165.t1

model.g1161.t1

model.g1167.t1

model.g1164.t1

model.g1163.t1

model.g1191.t1

model.g1196.t1

model.g1171.t1

model.g1199.t1

model.g1186.t1

model.g1192.t1

model.g1193.t1

model.g1185.t1

model.g1174.t1

model.g1195.t1

model.g1172.t1

model.g1178.t1

model.g1181.t1

model.g1212.t1

model.g1220.t1

model.g1229.t1

model.g1224.t1

model.g1214.t1

model.g1230.t1

model.g1225.t1

model.g1226.t1

model.g1287.t1

model.g1245.t1

model.g1256.t1

model.g1244.t1

model.g1283.t1

model.g1285.t1

model.g1255.t1

model.g1243.t1

model.g1266.t1

model.g1271.t1

model.g1257.t1

model.g1236.t1

model.g1277.t1

model.g1259.t1

model.g1273.t1

model.g1270.t1

model.g1278.t1

model.g1246.t1

model.g1282.t1

model.g1281.t1

model.g1288.t1

model.g1253.t1

model.g1301.t1

model.g1295.t1

model.g1297.t1

model.g1305.t1

model.g1294.t1

model.g1308.t1

model.g1315.t1

model.g1319.t1

model.g1307.t1

model.g1318.t1

model.g1343.t1

model.g1375.t1

model.g1346.t1

model.g1373.t1

model.g1367.t1

model.g1352.t1

model.g1359.t1

model.g1336.t1

model.g1360.t1

model.g1334.t1

model.g1344.t1

model.g1320.t1

model.g1368.t1

model.g1350.t1

model.g1370.t1

model.g1379.t1

model.g1339.t1

model.g1328.t1

model.g1371.t1

model.g1361.t1

model.g1347.t1

model.g1362.t1

model.g1333.t1

model.g1364.t1

model.g1372.t1

model.g1383.t1

model.g1388.t1

model.g1403.t1

model.g1395.t1

model.g1398.t1

model.g1385.t1

model.g1405.t1

model.g1397.t1

model.g1384.t1

model.g1432.t1

model.g1439.t1

model.g1428.t1

model.g1441.t1

model.g1425.t1

model.g1420.t1

model.g1433.t1

model.g1443.t1

model.g1412.t1

model.g1418.t1

model.g1421.t1

model.g1417.t1

model.g1429.t1

model.g1416.t1

model.g1434.t1

model.g1426.t1

model.g1431.t1

model.g1463.t1

model.g1452.t1

model.g1495.t1

model.g1459.t1

model.g1447.t1

model.g1467.t1

model.g1453.t1

model.g1465.t1

model.g1486.t1

model.g1451.t1

model.g1461.t1

model.g1483.t1

model.g1498.t1

model.g1482.t1

model.g1466.t1

model.g1480.t1

model.g1491.t1

model.g1469.t1

model.g1474.t1

model.g1497.t1

model.g1490.t1

model.g1454.t1

model.g1558.t1

model.g1510.t1

model.g1528.t1

model.g1527.t1

model.g1552.t1

model.g1520.t1

model.g1542.t1

model.g1592.t1

model.g1509.t1

model.g1554.t1

model.g1545.t1

model.g1503.t1

model.g1587.t1

model.g1534.t1

model.g1575.t1

model.g1543.t1

model.g1563.t1

model.g1507.t1

model.g1538.t1

model.g1525.t1

model.g1562.t1

model.g1505.t1

model.g1531.t1

model.g1530.t1

model.g1564.t1

model.g1548.t1

model.g1537.t1

model.g1586.t1

model.g1588.t1

model.g1569.t1

model.g1594.t1

model.g1593.t1

model.g1501.t1

model.g1500.t1

model.g1603.t1

model.g1599.t1

model.g1600.t1

model.g1605.t1

model.g1601.t1

model.g1615.t1

model.g1612.t1

model.g1606.t1

model.g1622.t1

model.g1614.t1

model.g1628.t1

model.g1626.t1

model.g1630.t1

model.g1642.t1

model.g1625.t1

model.g1652.t1

model.g1648.t1

model.g1654.t1

model.g1649.t1

model.g1667.t1

model.g1665.t1

model.g1684.t1

model.g1700.t1

model.g1702.t1

model.g1678.t1

model.g1676.t1

model.g1710.t1

model.g1688.t1

model.g1711.t1

model.g1708.t1

model.g1683.t1

model.g1693.t1

model.g1686.t1

model.g1704.t1

model.g1714.t1

model.g1712.t1

model.g1726.t1

model.g1732.t1

model.g1725.t1

model.g1728.t1

model.g1713.t1

model.g1716.t1

model.g1744.t1

model.g1740.t1

model.g1748.t1

model.g1757.t1

model.g1767.t1

model.g1769.t1

model.g1761.t1

model.g1750.t1

model.g1746.t1

model.g1805.t1

model.g1802.t1

model.g1878.t1

model.g1791.t1

model.g1792.t1

model.g1784.t1

model.g1862.t1

model.g1821.t1

model.g1801.t1

model.g1797.t1

model.g1863.t1

model.g1837.t1

model.g1881.t1

model.g1845.t1

model.g1780.t1

model.g1820.t1

model.g1833.t1

model.g1777.t1

model.g1856.t1

model.g1842.t1

model.g1786.t1

model.g1846.t1

model.g1832.t1

model.g1827.t1

model.g1867.t1

model.g1860.t1

model.g1814.t1

model.g1829.t1

model.g1895.t1

model.g1897.t1

model.g1890.t1

model.g1904.t1

model.g1899.t1

model.g1901.t1

model.g1921.t1

model.g1912.t1

model.g1905.t1

model.g1919.t1

model.g1906.t1

model.g1938.t1

model.g1972.t1

model.g1955.t1

model.g1963.t1

model.g1948.t1

model.g1953.t1

model.g1949.t1

model.g1970.t1

model.g1952.t1

model.g1966.t1

model.g1990.t1

model.g1981.t1

model.g1983.t1

model.g1991.t1

model.g2006.t1

model.g2002.t1

model.g2007.t1

model.g2023.t1

model.g2050.t1

model.g2098.t1

model.g2035.t1

model.g2027.t1

model.g2064.t1

model.g2085.t1

model.g2088.t1

model.g2043.t1

model.g2100.t1

model.g2039.t1

model.g2087.t1

model.g2065.t1

model.g2104.t1

model.g2097.t1

model.g2077.t1

model.g2108.t1

model.g2059.t1

model.g2055.t1

model.g2033.t1

model.g2057.t1

model.g2063.t1

model.g2083.t1

model.g2109.t1

model.g2053.t1

model.g2081.t1

model.g2047.t1

model.g2123.t1

model.g2128.t1

model.g2130.t1

model.g2140.t1

model.g2129.t1

model.g2139.t1

model.g2127.t1

model.g2124.t1

model.g2144.t1

model.g2150.t1

model.g2151.t1

model.g2155.t1

model.g2169.t1

model.g2171.t1

model.g2178.t1

model.g2159.t1

model.g2167.t1

model.g2165.t1

model.g2179.t1

model.g2158.t1

model.g2185.t1

model.g2174.t1

model.g2182.t1

model.g2212.t1

model.g2188.t1

model.g2189.t1

model.g2186.t1

model.g2203.t1

model.g2206.t1

model.g2194.t1

model.g2204.t1

model.g2201.t1

model.g2198.t1

model.g2216.t1

model.g2232.t1

model.g2228.t1

model.g2223.t1

model.g2221.t1

model.g2236.t1

model.g2274.t1

model.g2278.t1

model.g2245.t1

model.g2258.t1

model.g2272.t1

model.g2262.t1

model.g2282.t1

model.g2256.t1

model.g2251.t1

model.g2238.t1

model.g2252.t1

model.g2239.t1

model.g2273.t1

model.g2255.t1

model.g2265.t1

model.g2294.t1

model.g2301.t1

model.g2322.t1

model.g2321.t1

model.g2295.t1

model.g2311.t1

model.g2289.t1

model.g2296.t1

model.g2313.t1

model.g2285.t1

model.g2304.t1

model.g2307.t1

model.g2328.t1

model.g2286.t1

model.g2332.t1

model.g2340.t1

model.g2341.t1

model.g2344.t1

model.g2342.t1

model.g2361.t1

model.g2349.t1

model.g2357.t1

model.g2363.t1

model.g2367.t1

model.g2366.t1

model.g2345.t1

model.g2360.t1

model.g2347.t1

model.g2371.t1

model.g2372.t1

model.g2436.t1

model.g2437.t1

model.g2374.t1

model.g2442.t1

model.g2445.t1

model.g2382.t1

model.g2417.t1

model.g2403.t1

model.g2386.t1

model.g2393.t1

model.g2385.t1

model.g2448.t1

model.g2412.t1

model.g2446.t1

model.g2402.t1

model.g2395.t1

model.g2424.t1

model.g2416.t1

model.g2387.t1

model.g2434.t1

model.g2420.t1

model.g2381.t1

model.g2458.t1

model.g2461.t1

model.g2459.t1

model.g2456.t1

model.g2452.t1

model.g2453.t1

model.g2472.t1

model.g2486.t1

model.g2476.t1

model.g2465.t1

model.g2485.t1

model.g2487.t1

model.g2478.t1

model.g2467.t1

model.g2488.t1

model.g2464.t1

model.g2491.t1

model.g2503.t1

model.g2504.t1

model.g2495.t1

model.g2496.t1

model.g2512.t1

model.g2502.t1

model.g2499.t1

model.g2506.t1

model.g2500.t1

model.g2520.t1

model.g2514.t1

model.g2526.t1

model.g2537.t1

model.g2518.t1

model.g2521.t1

model.g2516.t1

model.g2535.t1

model.g2515.t1

model.g2517.t1

model.g2550.t1

model.g2547.t1

model.g2546.t1

model.g2549.t1

model.g2544.t1

model.g2552.t1

model.g2562.t1

model.g2557.t1

model.g2555.t1

model.g2570.t1

model.g2567.t1

model.g2571.t1

model.g2589.t1

model.g2566.t1

model.g2590.t1

model.g2582.t1

model.g2574.t1

model.g2565.t1

model.g2573.t1

model.g2578.t1

model.g2579.t1

model.g2568.t1

model.g2597.t1

model.g2596.t1

model.g2599.t1

model.g2598.t1

model.g2612.t1

model.g2619.t1

model.g2606.t1

model.g2607.t1

model.g2603.t1

model.g2610.t1

model.g2611.t1

model.g2616.t1

model.g2613.t1

model.g2635.t1

model.g2631.t1

model.g2626.t1

model.g2637.t1

model.g2639.t1

model.g2644.t1

model.g2643.t1

model.g2651.t1

model.g2653.t1

model.g2638.t1

model.g2652.t1

model.g2642.t1

model.g2655.t1

model.g2702.t1

model.g2685.t1

model.g2678.t1

model.g2708.t1

model.g2674.t1

model.g2699.t1

model.g2658.t1

model.g2695.t1

model.g2663.t1

model.g2692.t1

model.g2668.t1

model.g2677.t1

model.g2675.t1

model.g2665.t1

model.g2693.t1

model.g2689.t1

model.g2679.t1

model.g2697.t1

model.g2694.t1

model.g2714.t1

model.g2716.t1

model.g2712.t1

model.g2710.t1

model.g2711.t1

model.g2727.t1

model.g2733.t1

model.g2731.t1

model.g2730.t1

model.g2739.t1

model.g2760.t1

model.g2768.t1

model.g2772.t1

model.g2787.t1

model.g2788.t1

model.g2780.t1

model.g2790.t1

model.g2789.t1

model.g2779.t1

model.g2802.t1

model.g2804.t1

model.g2801.t1

model.g2816.t1

model.g2800.t1

model.g2811.t1

model.g2831.t1

model.g2869.t1

model.g2861.t1

model.g2835.t1

model.g2879.t1

model.g2856.t1

model.g2849.t1

model.g2839.t1

model.g2834.t1

model.g2864.t1

model.g2880.t1

model.g2862.t1

model.g2888.t1

model.g2843.t1

model.g2865.t1

model.g2863.t1

model.g2844.t1

model.g2832.t1

model.g2866.t1

model.g2885.t1

model.g2854.t1

model.g2833.t1

model.g2842.t1

model.g2876.t1

model.g2926.t1

model.g2895.t1

model.g2906.t1

model.g2893.t1

model.g2937.t1

model.g2898.t1

model.g2924.t1

model.g2901.t1

model.g2931.t1

model.g2909.t1

model.g2910.t1

model.g2896.t1

model.g2902.t1

model.g2913.t1

model.g2935.t1

model.g2925.t1

model.g2922.t1

model.g2903.t1

model.g2908.t1

model.g2941.t1

model.g2978.t1

model.g2966.t1

model.g2951.t1

model.g2970.t1

model.g2984.t1

model.g2990.t1

model.g3002.t1

model.g3000.t1

model.g3003.t1

model.g2998.t1

model.g3005.t1

model.g3004.t1

model.g3007.t1

model.g3006.t1

model.g3010.t1

model.g3008.t1

model.g3012.t1

model.g3013.t1

model.g3019.t1

model.g3016.t1

model.g3014.t1

model.g3015.t1

model.g3040.t1

model.g3032.t1

model.g3026.t1

model.g3020.t1

model.g3034.t1

model.g3024.t1

model.g3031.t1

model.g3027.t1

model.g3053.t1

model.g3048.t1

model.g3044.t1

model.g3065.t1

model.g3050.t1

model.g3041.t1

model.g3066.t1

model.g3046.t1

model.g3047.t1

model.g3060.t1

model.g3056.t1

model.g3055.t1

model.g3068.t1

model.g3072.t1

model.g3075.t1

model.g3078.t1

model.g3086.t1

model.g3119.t1

model.g3108.t1

model.g3104.t1

model.g3121.t1

model.g3096.t1

model.g3122.t1

model.g3095.t1

model.g3124.t1

model.g3135.t1

model.g3133.t1

model.g3144.t1

model.g3146.t1

model.g3160.t1

model.g3153.t1

model.g3159.t1

model.g3158.t1

model.g3165.t1

model.g3183.t1

model.g3191.t1

model.g3188.t1

model.g3173.t1

model.g3163.t1

model.g3194.t1

model.g3176.t1

model.g3193.t1

model.g3166.t1

model.g3170.t1

model.g3189.t1

model.g3171.t1

model.g3195.t1

model.g3178.t1

model.g3169.t1

model.g3184.t1

model.g3175.t1

model.g3181.t1

model.g3212.t1

model.g3201.t1

model.g3223.t1

model.g3218.t1

model.g3220.t1

model.g3210.t1

model.g3207.t1

model.g3231.t1

model.g3238.t1

model.g3244.t1

model.g3226.t1

model.g3230.t1

model.g3246.t1

model.g3239.t1

model.g3240.t1

model.g3249.t1

model.g3228.t1

model.g3259.t1

model.g3258.t1

model.g3257.t1

model.g3261.t1

model.g3254.t1

model.g3265.t1

model.g3268.t1

model.g3266.t1

model.g3279.t1

model.g3281.t1

model.g3271.t1

model.g3280.t1

model.g3298.t1

model.g3297.t1

model.g3300.t1

model.g3299.t1

model.g3286.t1

model.g3307.t1

model.g3323.t1

model.g3318.t1

model.g3316.t1

model.g3314.t1

model.g3308.t1

model.g3322.t1

model.g3310.t1

model.g3324.t1

model.g3317.t1

model.g3331.t1

model.g3326.t1

model.g3355.t1

model.g3401.t1

model.g3432.t1

model.g3431.t1

model.g3435.t1

model.g3440.t1

model.g3337.t1

model.g3409.t1

model.g3399.t1

model.g3404.t1

model.g3405.t1

model.g3393.t1

model.g3400.t1

model.g3340.t1

model.g3338.t1

model.g3342.t1

model.g3356.t1

model.g3360.t1

model.g3367.t1

model.g3380.t1

model.g3370.t1

model.g3387.t1

model.g3369.t1

model.g3394.t1

model.g3382.t1

model.g3402.t1

model.g3389.t1

model.g3396.t1

model.g3375.t1

model.g3373.t1

model.g3359.t1

model.g3348.t1

model.g3406.t1

model.g3416.t1

model.g3383.t1

model.g3436.t1

model.g3371.t1

model.g3438.t1

model.g3378.t1

model.g3386.t1

model.g3366.t1

model.g3442.t1

model.g3450.t1

model.g3446.t1

model.g3448.t1

model.g3463.t1

model.g3461.t1

model.g3459.t1

model.g3468.t1

model.g3462.t1

model.g3457.t1

model.g3467.t1

model.g3464.t1

model.g3458.t1

model.g3491.t1

model.g3487.t1

model.g3473.t1

model.g3488.t1

model.g3500.t1

model.g3499.t1

model.g3485.t1

model.g3482.t1

model.g3484.t1

model.g3472.t1

model.g3497.t1

model.g3496.t1

model.g3476.t1

model.g3477.t1

model.g3509.t1

model.g3533.t1

model.g3528.t1

model.g3507.t1

model.g3529.t1

model.g3517.t1

model.g3524.t1

model.g3514.t1

model.g3543.t1

model.g3540.t1

model.g3541.t1

model.g3545.t1

model.g3542.t1

model.g3548.t1

model.g3550.t1

model.g3560.t1

model.g3553.t1

model.g3562.t1

model.g3563.t1

model.g3549.t1

model.g3552.t1

model.g3564.t1

model.g3551.t1

model.g3569.t1

model.g3575.t1

model.g3570.t1

model.g3573.t1

model.g3571.t1

model.g3603.t1

model.g3580.t1

model.g3594.t1

model.g3593.t1

model.g3576.t1

model.g3583.t1

model.g3577.t1

model.g3584.t1

model.g3579.t1

model.g3600.t1

model.g3637.t1

model.g3629.t1

model.g3621.t1

model.g3636.t1

model.g3642.t1

model.g3669.t1

model.g3639.t1

model.g3653.t1

model.g3622.t1

model.g3605.t1

model.g3626.t1

model.g3631.t1

model.g3658.t1

model.g3663.t1

model.g3607.t1

model.g3625.t1

model.g3618.t1

model.g3668.t1

model.g3641.t1

model.g3652.t1

model.g3680.t1

model.g3713.t1

model.g3674.t1

model.g3708.t1

model.g3704.t1

model.g3675.t1

model.g3689.t1

model.g3682.t1

model.g3687.t1

model.g3715.t1

model.g3694.t1

model.g3712.t1

model.g3711.t1

model.g3701.t1

model.g3679.t1

model.g3688.t1

model.g3690.t1

model.g3703.t1

model.g3707.t1

model.g3735.t1

model.g3722.t1

model.g3721.t1

model.g3719.t1

model.g3730.t1

model.g3724.t1

model.g3723.t1

model.g3729.t1

model.g3734.t1

model.g3741.t1

model.g3750.t1

model.g3764.t1

model.g3768.t1

model.g3767.t1

model.g3778.t1

model.g3769.t1

model.g3776.t1

model.g3777.t1

model.g3779.t1

model.g3812.t1

model.g3797.t1

model.g3784.t1

model.g3785.t1

model.g3770.t1

model.g3801.t1

model.g3793.t1

model.g3791.t1

model.g3799.t1

model.g3808.t1

model.g3814.t1

model.g3822.t1

model.g3843.t1

model.g3832.t1

model.g3827.t1

model.g3842.t1

model.g3836.t1

model.g3850.t1

model.g3834.t1

model.g3853.t1

model.g3833.t1

model.g3847.t1

model.g3837.t1

model.g3844.t1

model.g3849.t1

model.g3835.t1

model.g3838.t1

model.g3854.t1

model.g3864.t1

model.g3856.t1

model.g3867.t1

model.g3863.t1

model.g3872.t1

model.g3879.t1

model.g3889.t1

model.g3902.t1

model.g3895.t1

model.g3883.t1

model.g3884.t1

model.g3887.t1

model.g3891.t1

model.g3901.t1

model.g3890.t1

model.g3912.t1

model.g3903.t1

model.g3907.t1

model.g3905.t1

model.g3957.t1

model.g3965.t1

model.g3953.t1

model.g3916.t1

model.g3918.t1

model.g3960.t1

model.g3917.t1

model.g3951.t1

model.g3968.t1

model.g3959.t1

model.g3948.t1

model.g3983.t1

model.g3930.t1

model.g3943.t1

model.g3973.t1

model.g3985.t1

model.g3940.t1

model.g3945.t1

model.g3947.t1

model.g3944.t1

model.g3974.t1

model.g3937.t1

model.g3964.t1

model.g3954.t1

model.g3971.t1

model.g3942.t1

model.g4017.t1

model.g3997.t1

model.g3988.t1

model.g4021.t1

model.g4025.t1

model.g4024.t1

model.g4030.t1

model.g4011.t1

model.g4018.t1

model.g4009.t1

model.g4043.t1

model.g4036.t1

model.g4046.t1

model.g4058.t1

model.g4038.t1

model.g4032.t1

model.g4040.t1

model.g4054.t1

model.g4062.t1

model.g4075.t1

model.g4095.t1

model.g4072.t1

model.g4094.t1

model.g4078.t1

model.g4093.t1

model.g4073.t1

model.g4061.t1

model.g4091.t1

model.g4085.t1

model.g4084.t1

model.g4117.t1

model.g4169.t1

model.g4160.t1

model.g4171.t1

model.g4122.t1

model.g4154.t1

model.g4116.t1

model.g4144.t1

model.g4104.t1

model.g4098.t1

model.g4148.t1

model.g4140.t1

model.g4127.t1

model.g4106.t1

model.g4102.t1

model.g4163.t1

model.g4124.t1

model.g4159.t1

model.g4143.t1

model.g4123.t1

model.g4145.t1

model.g4142.t1

model.g4150.t1

model.g4138.t1

model.g4135.t1

model.g4115.t1

model.g4119.t1

model.g4172.t1

model.g4182.t1

model.g4181.t1

model.g4187.t1

model.g4176.t1

model.g4179.t1

model.g4188.t1

model.g4316.t1

model.g4302.t1

model.g4284.t1

model.g4287.t1

model.g4256.t1

model.g4327.t1

model.g4300.t1

model.g4207.t1

model.g4214.t1

model.g4313.t1

model.g4294.t1

model.g4309.t1

model.g4241.t1

model.g4277.t1

model.g4276.t1

model.g4208.t1

model.g4193.t1

model.g4268.t1

model.g4323.t1

model.g4269.t1

model.g4310.t1

model.g4199.t1

model.g4319.t1

model.g4206.t1

model.g4317.t1

model.g4259.t1

model.g4253.t1

model.g4244.t1

model.g4286.t1

model.g4196.t1

model.g4194.t1

model.g4273.t1

model.g4295.t1

model.g4237.t1

model.g4264.t1

model.g4293.t1

model.g4238.t1

model.g4281.t1

model.g4297.t1

model.g4243.t1

model.g4299.t1

model.g4399.t1

model.g4342.t1

model.g4376.t1

model.g4393.t1

model.g4370.t1

model.g4390.t1

model.g4352.t1

model.g4344.t1

model.g4403.t1

model.g4369.t1

model.g4410.t1

model.g4425.t1

model.g4440.t1

model.g4418.t1

model.g4349.t1

model.g4385.t1

model.g4404.t1

model.g4386.t1

model.g4357.t1

model.g4355.t1

model.g4348.t1

model.g4419.t1

model.g4354.t1

model.g4361.t1

model.g4407.t1

model.g4443.t1

model.g4340.t1

model.g4444.t1

model.g4426.t1

model.g4387.t1

model.g4431.t1

model.g4384.t1

model.g4409.t1

model.g4430.t1

model.g4389.t1

model.g4394.t1

model.g4438.t1

model.g4375.t1

model.g4391.t1

model.g4397.t1

model.g4356.t1

model.g4388.t1

model.g4435.t1

model.g4481.t1

model.g4469.t1

model.g4473.t1

model.g4453.t1

model.g4468.t1

model.g4455.t1

model.g4459.t1

model.g4458.t1

model.g4452.t1

model.g4484.t1

model.g4493.t1

model.g4487.t1

model.g4507.t1

model.g4500.t1

model.g4501.t1

model.g4524.t1

model.g4530.t1

model.g4519.t1

model.g4535.t1

model.g4512.t1

model.g4525.t1

model.g4533.t1

model.g4514.t1

model.g4522.t1

model.g4531.t1

model.g4529.t1

model.g4523.t1

model.g4516.t1

model.g4537.t1

model.g4527.t1

model.g4541.t1

model.g4553.t1

model.g4545.t1

model.g4540.t1

model.g4542.t1

model.g4539.t1

model.g4547.t1

model.g4549.t1

model.g4566.t1

model.g4561.t1

model.g4555.t1

model.g4582.t1

model.g4569.t1

model.g4554.t1

model.g4581.t1

model.g4559.t1

model.g4563.t1

model.g4558.t1

model.g4567.t1

model.g4574.t1

model.g4562.t1

model.g4571.t1

model.g4583.t1

model.g4557.t1

model.g4564.t1

model.g4600.t1

model.g4603.t1

model.g4605.t1

model.g4590.t1

model.g4591.t1

model.g4606.t1

model.g4597.t1

model.g4620.t1

model.g4615.t1

model.g4607.t1

model.g4621.t1

model.g4614.t1

model.g4610.t1

model.g4609.t1

model.g4613.t1

model.g4628.t1

model.g4623.t1

model.g4630.t1

model.g4634.t1

model.g4626.t1

model.g4629.t1

model.g4633.t1

model.g4660.t1

model.g4659.t1

model.g4675.t1

model.g4638.t1

model.g4693.t1

model.g4676.t1

model.g4679.t1

model.g4649.t1

model.g4641.t1

model.g4648.t1

model.g4685.t1

model.g4643.t1

model.g4680.t1

model.g4652.t1

model.g4678.t1

model.g4653.t1

model.g4700.t1

model.g4668.t1

model.g4663.t1

model.g4696.t1

model.g4699.t1

model.g4656.t1

model.g4686.t1

model.g4651.t1

model.g4702.t1

model.g4708.t1

model.g4705.t1

model.g4704.t1

model.g4701.t1

model.g4706.t1

model.g4709.t1

model.g4717.t1

model.g4733.t1

model.g4714.t1

model.g4731.t1

model.g4726.t1

model.g4713.t1

model.g4743.t1

model.g4722.t1

model.g4728.t1

model.g4723.t1

model.g4742.t1

model.g4732.t1

model.g4725.t1

**vineae_unique_orignal_name**

213 217 30 1.000 1 5 model.g521.t1;model.g524.t1;model.g523.t1;model.g522.t1;model.g3823.t1;

345 354 77 1.000 1 2 model.g3915.t1;model.g1057.t1;

389 399 45 1.000 1 2 model.g4031.t1;model.g1293.t1;

504 519 51 1.000 1 3 model.g4192.t1;model.g3287.t1;model.g4191.t1;

586 602 5 0.502 1 3 model.g1083.t1;model.g1381.t1;model.g4282.t1;

670 688 14 1.000 1 3 model.g17.t1;model.g302.t1;model.g2492.t1;

671 688 7 0.502 1 4 model.g4482.t1;model.g1634.t1;model.g3423.t1;model.g2709.t1;

673 693 15 1.000 1 2 model.g1633.t1;model.g1635.t1;

785 805 51 1.000 1 2 model.g4572.t1;model.g62.t1;

811 831 32 1.000 1 2 model.g4596.t1;model.g353.t1;

937 959 71 1.000 1 2 model.g379.t1;model.g378.t1;

1022 1043 22 1.000 1 3 model.g1923.t1;model.g19.t1;model.g1922.t1;

3186 3232 21 1.000 1 3 model.g1747.t1;model.g2827.t1;model.g3284.t1;

3212 3258 100 1.000 1 2 model.g7.t1;model.g1656.t1;

3213 3259 24 1.000 1 2 model.g27.t1;model.g2181.t1;

3214 3260 100 1.000 1 2 model.g51.t1;model.g2798.t1;

3215 3261 31 1.000 1 3 model.g2942.t1;model.g3336.t1;model.g58.t1;

3216 3262 37 1.000 1 4 model.g72.t1;model.g73.t1;model.g2766.t1;model.g74.t1;

3217 3263 22 1.000 1 2 model.g350.t1;model.g3372.t1;

3218 3264 23 1.000 1 2 model.g545.t1;model.g546.t1;

3219 3265 11 1.000 1 2 model.g595.t1;model.g3568.t1;

3220 3266 50 1.000 1 2 model.g775.t1;model.g3503.t1;

3221 3267 40 1.000 1 2 model.g1623.t1;model.g1624.t1;

3222 3268 17 0.502 1 3 model.g2818.t1;model.g3346.t1;model.g1932.t1;

3223 3269 34 1.000 1 2 model.g2621.t1;model.g2622.t1;
