# Supplementary material for: The Shared and Specific Genes and a Comparative Genomics Analysis within Three Hanseniaspora Strains
Source: Int J Genomics. 2019 Jun 2;2019:7910865. doi: 10.1155/2019/7910865 (PMC6589277; doi:10.1155/2019/7910865)
Supplement: Supplementary 7 — File 6: Blocks of the genome synteny analysis between K. apiculata, H. uvarum, and H. vineae. [file 7910865.f7.docx]

**Supplementary Table 6.** Blocks of the genome synteny analysis between *K. apiculata*, *H. uvarum*

and *H.vineae.*

**blocks_34-9_DSM**

Species1 Species2 Chr1 Chr2 BlkNum Start1 End1 Start2 End2 #Hits Genes1 %Genes1 Genes2 %Genes2 PearsonR

Hanseniaspora_uvarum_34_9 Hanseniaspora_uvarum_DSM_2768 1 001 1 310725 1284217 1698 974843 31 0 0 0 0 1

Hanseniaspora_uvarum_34_9 Hanseniaspora_uvarum_DSM_2768 2 002 1 11334 538242 8001 554375 26 0 0 0 0 -0.999852

Hanseniaspora_uvarum_34_9 Hanseniaspora_uvarum_DSM_2768 2 004 1 584224 967169 24588 407777 16 0 0 0 0 0.999998

Hanseniaspora_uvarum_34_9 Hanseniaspora_uvarum_DSM_2768 4 011 1 11546 210065 8997 231931 10 0 0 0 0 0.999276

Hanseniaspora_uvarum_34_9 Hanseniaspora_uvarum_DSM_2768 5 010 1 74835 254622 61469 254005 11 0 0 0 0 -0.999636

Hanseniaspora_uvarum_34_9 Hanseniaspora_uvarum_DSM_2768 6 014 1 18576 194493 17011 200335 8 0 0 0 0 -0.999986

Hanseniaspora_uvarum_34_9 Hanseniaspora_uvarum_DSM_2768 7 003 1 19006 69911 3471 63606 9 0 0 0 0 -0.997358

Hanseniaspora_uvarum_34_9 Hanseniaspora_uvarum_DSM_2768 9 023 1 264918 360335 3047 108564 8 0 0 0 0 0.999497

Hanseniaspora_uvarum_34_9 Hanseniaspora_uvarum_DSM_2768 9 005 1 382133 698864 24260 353504 18 0 0 0 0 -0.999978

Hanseniaspora_uvarum_34_9 Hanseniaspora_uvarum_DSM_2768 9 009 1 724889 903719 92613 306081 18 0 0 0 0 -0.999911

Hanseniaspora_uvarum_34_9 Hanseniaspora_uvarum_DSM_2768 13 012 1 137231 247398 20191 137202 8 0 0 0 0 0.999917

Hanseniaspora_uvarum_34_9 Hanseniaspora_uvarum_DSM_2768 14 006 1 12834 199316 145658 351019 11 0 0 0 0 0.999951

Hanseniaspora_uvarum_34_9 Hanseniaspora_uvarum_DSM_2768 15 008 1 1036 288587 1088 317245 14 0 0 0 0 0.999768

Hanseniaspora_uvarum_34_9 Hanseniaspora_uvarum_DSM_2768 16 019 1 21487 117116 21862 122991 7 0 0 0 0 -0.999059

Hanseniaspora_uvarum_34_9 Hanseniaspora_uvarum_DSM_2768 17 017 1 1986 137195 39193 182034 9 0 0 0 0 0.99985

Hanseniaspora_uvarum_34_9 Hanseniaspora_uvarum_DSM_2768 18 013 1 83552 220946 16097 173452 8 0 0 0 0 0.999796

Hanseniaspora_uvarum_34_9 Hanseniaspora_uvarum_DSM_2768 19 003 1 3172 232240 317784 563661 13 0 0 0 0 0.999904

Hanseniaspora_uvarum_34_9 Hanseniaspora_uvarum_DSM_2768 20 007 1 195716 406693 103196 334672 15 0 0 0 0 -0.999488

Hanseniaspora_uvarum_34_9 Hanseniaspora_uvarum_DSM_2768 22 003 1 20417 173838 114113 290846 8 0 0 0 0 0.999868

**blocks_34-9_vineae**

Species1 Species2 Chr1 Chr2 BlkNum Start1 End1 Start2 End2 #Hits Genes1 %Genes1 Genes2 %Genes2 PearsonR

Hanseniaspora_uvarum_34_9 Hanseniaspora_vineae_T02_19AF 1 152 1 622645 682344 117615 187335 9 0 0 0 0 -0.998331

Hanseniaspora_uvarum_34_9 Hanseniaspora_vineae_T02_19AF 1 166 1 1201083 1273484 35974 130107 16 0 0 0 0 0.979771

Hanseniaspora_uvarum_34_9 Hanseniaspora_vineae_T02_19AF 1 166 2 902389 1001627 136806 196414 9 0 0 0 0 -0.900826

Hanseniaspora_uvarum_34_9 Hanseniaspora_vineae_T02_19AF 1 185 1 574461 785866 5431 163765 15 0 0 0 0 0.860546

Hanseniaspora_uvarum_34_9 Hanseniaspora_vineae_T02_19AF 1 267 1 325123 466649 1045 114743 15 0 0 0 0 0.80574

Hanseniaspora_uvarum_34_9 Hanseniaspora_vineae_T02_19AF 2 256 1 429662 472775 42564 114098 8 0 0 0 0 -0.823157

Hanseniaspora_uvarum_34_9 Hanseniaspora_vineae_T02_19AF 2 287 1 519879 721515 3192 136106 20 0 0 0 0 -0.830203

Hanseniaspora_uvarum_34_9 Hanseniaspora_vineae_T02_19AF 2 291 1 38724 186246 35307 299695 26 0 0 0 0 -0.964929

Hanseniaspora_uvarum_34_9 Hanseniaspora_vineae_T02_19AF 4 152 1 245255 285389 2910 56418 10 0 0 0 0 0.996517

Hanseniaspora_uvarum_34_9 Hanseniaspora_vineae_T02_19AF 6 128 1 125850 209764 2547 112385 18 0 0 0 0 -0.999014

Hanseniaspora_uvarum_34_9 Hanseniaspora_vineae_T02_19AF 7 122 1 16369 157078 3723 38626 7 0 0 0 0 -0.853046

Hanseniaspora_uvarum_34_9 Hanseniaspora_vineae_T02_19AF 9 080 1 566110 668147 876 139317 17 0 0 0 0 0.997028

Hanseniaspora_uvarum_34_9 Hanseniaspora_vineae_T02_19AF 9 123 1 401098 467384 33809 129895 14 0 0 0 0 0.941079

Hanseniaspora_uvarum_34_9 Hanseniaspora_vineae_T02_19AF 9 137 1 190202 278133 5166 135693 16 0 0 0 0 -0.996631

Hanseniaspora_uvarum_34_9 Hanseniaspora_vineae_T02_19AF 9 141 1 9826 64544 28600 105578 11 0 0 0 0 -0.987846

Hanseniaspora_uvarum_34_9 Hanseniaspora_vineae_T02_19AF 13 218 1 75120 222477 12761 119950 14 0 0 0 0 -0.910151

Hanseniaspora_uvarum_34_9 Hanseniaspora_vineae_T02_19AF 13 282 1 209953 243828 78155 121874 7 0 0 0 0 -0.813561

Hanseniaspora_uvarum_34_9 Hanseniaspora_vineae_T02_19AF 14 103 1 98406 132385 30796 79694 9 0 0 0 0 -0.996788

Hanseniaspora_uvarum_34_9 Hanseniaspora_vineae_T02_19AF 15 118 1 78905 221451 12420 113549 12 0 0 0 0 -0.90198

Hanseniaspora_uvarum_34_9 Hanseniaspora_vineae_T02_19AF 18 303 1 20470 119376 2757 144782 19 0 0 0 0 -0.807588

Hanseniaspora_uvarum_34_9 Hanseniaspora_vineae_T02_19AF 19 293 1 84807 109192 213479 241569 8 0 0 0 0 -0.995716

Hanseniaspora_uvarum_34_9 Hanseniaspora_vineae_T02_19AF 20 140 1 310452 365670 56104 124393 10 0 0 0 0 0.993195

Hanseniaspora_uvarum_34_9 Hanseniaspora_vineae_T02_19AF 22 203 1 29898 88901 5574 67976 8 0 0 0 0 0.866453

Hanseniaspora_uvarum_34_9 Hanseniaspora_vineae_T02_19AF 25 104 1 5286 69758 75116 181973 11 0 0 0 0 -0.972058

**blocks_DSM_vineae**

Species1 Species2 Chr1 Chr2 BlkNum Start1 End1 Start2 End2 #Hits Genes1 %Genes1 Genes2 %Genes2 PearsonR

Hanseniaspora_uvarum_DSM_2768 Hanseniaspora_vineae_T02_19AF 001 152 1 313743 373416 117568 187335 9 0 0 0 0 -0.998328

Hanseniaspora_uvarum_DSM_2768 Hanseniaspora_vineae_T02_19AF 001 166 1 891405 963756 35974 130107 16 0 0 0 0 0.979713

Hanseniaspora_uvarum_DSM_2768 Hanseniaspora_vineae_T02_19AF 001 166 2 593230 692205 136811 196527 9 0 0 0 0 -0.899589

Hanseniaspora_uvarum_DSM_2768 Hanseniaspora_vineae_T02_19AF 001 185 1 265163 476879 5431 163765 15 0 0 0 0 0.860797

Hanseniaspora_uvarum_DSM_2768 Hanseniaspora_vineae_T02_19AF 001 267 1 16418 157420 1045 114743 14 0 0 0 0 0.838765

Hanseniaspora_uvarum_DSM_2768 Hanseniaspora_vineae_T02_19AF 010 133 1 30592 68680 4553 49894 8 0 0 0 0 0.974872

Hanseniaspora_uvarum_DSM_2768 Hanseniaspora_vineae_T02_19AF 010 177 1 218040 246827 69120 103180 7 0 0 0 0 -0.999587

Hanseniaspora_uvarum_DSM_2768 Hanseniaspora_vineae_T02_19AF 012 218 1 45291 111333 11684 94578 13 0 0 0 0 -0.955906

Hanseniaspora_uvarum_DSM_2768 Hanseniaspora_vineae_T02_19AF 012 282 1 99602 200303 16085 121874 13 0 0 0 0 -0.939015

Hanseniaspora_uvarum_DSM_2768 Hanseniaspora_vineae_T02_19AF 013 303 1 5928 56904 2774 111073 9 0 0 0 0 -0.893902

Hanseniaspora_uvarum_DSM_2768 Hanseniaspora_vineae_T02_19AF 014 128 1 393 87463 2547 112392 18 0 0 0 0 0.998829

Hanseniaspora_uvarum_DSM_2768 Hanseniaspora_vineae_T02_19AF 018 177 1 124437 158973 5003 57119 9 0 0 0 0 -0.903081

Hanseniaspora_uvarum_DSM_2768 Hanseniaspora_vineae_T02_19AF 002 256 1 73297 116398 42564 113945 8 0 0 0 0 0.822575

Hanseniaspora_uvarum_DSM_2768 Hanseniaspora_vineae_T02_19AF 002 291 1 373275 526058 35307 299695 26 0 0 0 0 0.965747

Hanseniaspora_uvarum_DSM_2768 Hanseniaspora_vineae_T02_19AF 026 152 1 25004 103569 2910 109478 13 0 0 0 0 0.997954

Hanseniaspora_uvarum_DSM_2768 Hanseniaspora_vineae_T02_19AF 003 203 1 123969 192200 5574 67976 8 0 0 0 0 0.872025

Hanseniaspora_uvarum_DSM_2768 Hanseniaspora_vineae_T02_19AF 003 293 1 402154 427433 213464 241556 8 0 0 0 0 -0.997245

Hanseniaspora_uvarum_DSM_2768 Hanseniaspora_vineae_T02_19AF 004 287 1 129319 341555 3192 168163 15 0 0 0 0 0.97722

Hanseniaspora_uvarum_DSM_2768 Hanseniaspora_vineae_T02_19AF 005 080 1 56056 161196 876 139443 17 0 0 0 0 -0.996988

Hanseniaspora_uvarum_DSM_2768 Hanseniaspora_vineae_T02_19AF 005 123 1 263408 331790 33801 129902 14 0 0 0 0 -0.942371

Hanseniaspora_uvarum_DSM_2768 Hanseniaspora_vineae_T02_19AF 006 103 1 47495 277396 30796 159354 12 0 0 0 0 -0.980197

Hanseniaspora_uvarum_DSM_2768 Hanseniaspora_vineae_T02_19AF 007 140 1 145644 203304 56104 124557 11 0 0 0 0 -0.993245

Hanseniaspora_uvarum_DSM_2768 Hanseniaspora_vineae_T02_19AF 008 118 1 84148 237481 12420 113585 14 0 0 0 0 -0.871783
